# A systematic molecular and pharmacologic evaluation of AKT inhibitors reveals new insight into their biological activity

**DOI:** 10.1038/s41416-020-0889-4

**Published:** 2020-05-22

**Authors:** Eleftherios Kostaras, Teresa Kaserer, Glorianne Lazaro, Sara Farrah Heuss, Aasia Hussain, Pedro Casado, Angela Hayes, Cihangir Yandim, Nicolaos Palaskas, Yi Yu, Brian Schwartz, Florence Raynaud, Yuen-Li Chung, Pedro R. Cutillas, Igor Vivanco

**Affiliations:** 1 Division of Cancer Therapeutics, The Institute of Cancer Research, 15 Cotswold Road, SM2 5NG London, UK; 2 Cancer Research UK Cancer Therapeutics Unit, Division of Cancer Therapeutics, The Institute of Cancer Research, London, SW7 3RP UK; 3 Department of Pharmacology and Toxicology, Institute of Pharmacy, Center for Molecular Biosciences, University of Innsbruck, Innsbruck, A-6020 Austria; 4 Centre for Haemato-Oncology, Barts Cancer Institute, Queen Mary University of London, Charterhouse Square, London, EC1M 6BQ UK; 5 Division of Hematology and Oncology, David Geffen School of Medicine at UCLA, Los Angeles, CA USA; 6 ArQule, Inc. (a wholly-owned subsidiary of Merck & Co., Inc., Kenilworth, NJ, USA), Burlington, MA 01803 USA; 7 Cancer Research UK Cancer Imaging Centre, Division of Radiotherapy and Imaging, The Institute of Cancer Research London and Royal Marsden Hospital, London, SW7 3RP UK; 8 Present Address: Department of Genetics and Bioengineering, Faculty of Engineering, Izmir University of Economics, 35330 Balçova, Izmir Turkey

**Keywords:** Cancer therapeutic resistance, Oncogenes, Cell signalling, Drug development

## Abstract

**Background:**

AKT, a critical effector of the phosphoinositide 3-kinase (PI3K) signalling cascade, is an intensely pursued therapeutic target in oncology. Two distinct classes of AKT inhibitors have been in clinical development, ATP-competitive and allosteric. Class-specific differences in drug activity are likely the result of differential structural and conformational requirements governing efficient target binding, which ultimately determine isoform-specific potency, selectivity profiles and activity against clinically relevant AKT mutant variants.

**Methods:**

We have carried out a systematic evaluation of clinical AKT inhibitors using in vitro pharmacology, molecular profiling and biochemical assays together with structural modelling to better understand the context of drug-specific and drug-class-specific cell-killing activity.

**Results:**

Our data demonstrate clear differences between ATP-competitive and allosteric AKT inhibitors, including differential effects on non-catalytic activity as measured by a novel functional readout. Surprisingly, we found that some mutations can cause drug resistance in an isoform-selective manner despite high structural conservation across AKT isoforms. Finally, we have derived drug-class-specific phosphoproteomic signatures and used them to identify effective drug combinations.

**Conclusions:**

These findings illustrate the utility of individual AKT inhibitors, both as drugs and as chemical probes, and the benefit of AKT inhibitor pharmacological diversity in providing a repertoire of context-specific therapeutic options.

## Background

The serine/threonine kinase AKT is a critical effector of the phosphoinositide 3-kinase (PI3K) signalling pathway and regulates a number of oncogenic functions, including cell survival.^1^ It is aberrantly activated in a wide range of human tumours, and consequently, it has been actively pursued as a therapeutic target.^1,2^ Although pharmacological inactivation of AKT has been shown to have broad anti-tumour activity in a number of PI3K-active pre-clinical models,^3^ the clinical activity of AKT inhibitors as single agents has been primarily limited to tumours with a rare AKT-activating hot-spot mutation. This is clearly illustrated by data from two independent basket studies of the AKT inhibitor capivasertib (also called AZD5363),^4,5^ which have shown significant response rates in patients whose tumours carried the AKT1 E17K mutation.

Two distinct classes of AKT inhibitors are currently in clinical development, ATP-competitive and allosteric. Interestingly, in contrast to capivasertib, an ATP-competitive inhibitor, the allosteric AKT inhibitor MK-2206, has failed to show single-agent activity in a number of clinical trials,^6^ while another allosteric inhibitor called miransertib (also called ARQ 092) has shown promising results in early phase studies, including in a patient with an E17K mutation.^7^ It has been previously shown that the half-maximal inhibitory concentration (IC_50_) for the allosteric tool compound AKT1/2 inhibitor VIII is approximately 5-fold higher in AKT1 E17K compared to the wild-type enzyme while having no effect on the IC_50_ for an ATP-competitive inhibitor.^8^ Similarly, it has been shown in isogenic cell line models that the IC_50_ for capivasertib was unaffected by the E17K mutation, while that of MK-2206 was right-shifted.^9^ These observations are consistent with the reported differences in clinical activity between capivasertib and MK-2206, but raise interesting questions about the mechanistic basis for the observed activity of other allosteric inhibitors such as miransertib.^7,10,11^ The reasons for these varying outcomes and for the disappointing clinical activity of various AKT inhibitors in other tumours with hyperactive (but wild-type) AKT are therefore not entirely clear. However, given that AKT inhibitors can differ not only in their pharmacological properties, potency against individual isoforms and selectivity profiles, but also in their structural requirements for efficient target binding as well as their mode of inhibition and effects on downstream signalling, it is likely that the biological activity of different AKT inhibitors will be significantly influenced by AKT genotype in addition to the overall genetic background of the target cell. For example, allosteric inhibitors bind to AKT through interactions with the PH-domain/kinase-domain interface,^12^ which is a key feature of the inactive kinase. ATP-competitive inhibitors, on the other hand, bind to the active conformation in which the PH domain has swung away from the kinase domain and expose the ATP-binding pocket. Therefore, mutations that disrupt drug binding are expected to target different regions of the molecule depending on which class of drugs is interrogated. Unfortunately, determinants of context-specific drug activity are ill-defined, a limitation that complicates the efficient implementation of AKT-targeting strategies and limits the therapeutic scope of AKT-targeting agents. To improve our understanding of genotype/drug activity relationships, we have carried out extensive in vitro characterisation of several clinical AKT inhibitors to determine their biochemical and biological activity in clinically relevant mutant variants of AKT, and used molecular docking and homology models to understand the structural basis of these relationships. To explore whether and how the mode of AKT inhibition could influence the extent of drug-induced cell killing, we carried out phosphoproteomic profiling experiments in AKT-dependent cells treated with allosteric or ATP-competitive AKT inhibitors. Indeed, we found that drug-class-specific phosphopeptide signatures can be identified, and that co-targeting kinases involved in the regulation of these peptides can effectively induce cell death and overcome acquired AKT inhibitor resistance. Additionally, we have identified acetate excretion as a functional readout of non-catalytic AKT function, and show that it can be perturbed by allosteric (but not ATP-competitive) AKT inhibitors. Our analysis provides insight into the context-specific activity of clinical AKT inhibitors and offers a new set of tools to probe and study AKT function.

## Methods

### Antibodies and western blot

Antibodies against Vinculin (13901), AKT1 (2938), AKT2 (3063), pAKT T308 (13038), pAKT S473 (4060), c-PARP (D214) (5625), pPRAS40 T246 (13175), PRAS40 (8858), pAKT1 S473 (9018), pAKT2 S474 (8599), panAKT (4685), pBAD S136 (4366), BAD (9239), pChk2 T68 (2197), Chk2 (6334), LC3B (3868), GAPDH (5174), pS6 S235/236 (4858) and S6 (2217) were purchased from Cell Signalling. Cell lysates were prepared using 1× lysis buffer (9803, Cell Signalling) supplemented with 1% sodium dodecyl sulfate (SDS) (Sigma, L3771). Following sonication, lysates were quantified using the DC assay (Bio-Rad, 5000111) and equal amount of protein lysates were loaded into Tris-Glycine-SDS gels (Bio-Rad, 567-1095). Proteins were transferred into nitrocellulose membrane (Bio-Rad, 1704271) using the Trans-Blot Turbo Transfer System (Bio-Rad, 1704150) and were detected using Enhanced Chemiluminescence (ECL, Amersham, RPN2106/Bio-Rad, 1705062). Signal was captured on film (Amersham Hyper films ECL, 28906836) and developed using the Xorgaph X4 imaging system. Western blots were performed at least twice and a representative experiment is shown.

### Cell lines and chemicals

EBC1 cells were obtained from the Japanese Collection of Research Bioresources Cell Bank. AU-565, HCC1419, H1694, HT29, ZR-75-1, Hs 746T, BT-474 and MDA-MB-361 cells were purchased from ATCC. EFM-192A cells were purchased from DSMZ. HGC27 cells were purchased from Cell Lines Service. All commercially procured cell lines were cultured in Dulbecco’s modified Eagle’s medium (DMEM) (Life Science) media supplemented with foetal bovine serum. In order to create MK-2206-resistant MDA-MB-361 cells, 125 × 10^6^ cells were seeded in a Hyper Flasks (Corning) and treated with 2 μM MK-2206 for 3 months with media and drug being refreshed every other week. Wild-type and AKT1^−/−^/AKT2^−/−^ (AKT1/2 DKO) HCT116 or DLD-1 cells (a gift from Bert Volgestein, John Hopkins University School of Medicine) were maintained in McCoy’s or RPMI media, respectively. All media were supplemented with 10% foetal bovine serum (FBS) (Life Science, 10270106), primocin (Invivogen, ant-pm-2) and normocin (Invivogen, ant-nr-2). Acetate Colorimetric Assay Kit was purchased from Sigma (MAK086). MK-2206 2HCl (Selleckchem, S1078), ARQ 751 (ArQule), miransertib (ArQule), GSK690693 (Cayman, 16891), ipatasertib (MedKoo, 205467), capivasertib (Selleckchem, S8019), AZD0156 (APExBIO, B7822), KU-57788 (APExBIO, A8315), GSK2334470 (MedChemExpress, HY-14981), OSU-T315 (MedChemExpress, HY-18676), GSK2578215A (Cayman, 14603), apilimod (MedChemExpress, HY-14644), rapamycin (Selleckchem, S1039) and torin1 (Selleckchem, S2827) were diluted in dimethyl sulfoxide (DMSO) according to the manufacturer’s instructions and used at the indicated concentrations.

### Stable cell lines and site-directed mutagenesis

Human pLPCX-AKT1 and pLPCX-AKT2 were created with standard cloning techniques. Mutant complementary DNAs (cDNAs) were generated by site-directed mutagenesis using the Quickchange Lightning Kit (Agilent, 210513-5)) and the primers (Sigma) are described in Table 1. Stable cell lines expressing specific mutants of AKT1 or AKT2 were created with virus supernatants prepared from HEK293T cells transfected with retroviral packaging vectors (*ψ*-ampho) and retroviral constructs overexpressing either AKT1, AKT2 or different mutants. Infected cells were selected using puromycin 2 μg/mL (Life Science A1113803).

**Table 1:** Mutagenesis primers.

	Forward	Reverse
AKT1 D323H	gccgtagtcattgtgctccagcacctcgg	ccgaggtgctggagcacaatgactacggc
AKT1 D292A	cttgcacagcccgaaggctgtgatcttaatgtgc	gcacattaagatcacagccttcgggctgtgcaag
AKT1 W80A	cgatgacagtggtcgcctgcaggcagcgga	tccgctgcctgcaggcgaccactgtcatcg
AKT1 Q79K	gacagtggtccacttcaggcagcggatga	tcatccgctgcctgaagtggaccactgtc
AKT1 M227Q	ccgttggcgtactcctggacaaagcagaggcg	cgcctctgctttgtccaggagtacgccaacgg
AKT2 W80A	ctcgatgactgtggtcgcctgcaggcagcgtatg	catacgctgcctgcaggcgaccacagtcatcgag

### CRISPR-CAS9 gene editing and siRNA transfections

MDA-MB-361 cells were first transduced with a doxycycline-inducible Cas9 vector. Briefly, virus supernatants were prepared from HEK293T cells transfected with second-generation packaging vectors (psPAX and pMD.2) and Edit-R-Inducible Lentiviral Cas9 Nuclease vector (Horizon Discovery, CAS11229). A stable line (MDA-MB-361-iCas9) was generated through blasticidin selection (4 μg/mL). Subsequently, MDA-MB-361-iCas9 cells were similarly transduced with lentiviral guide RNA constructs targeting AKT1 (Addgene, 75500) and AKT2 (Addgene, 77506), and stable expressing lines were subjected to puromycin selection (2 μg/mL). To induce simultaneous AKT1 and AKT2 deletion in these cells, doxycycline (2 μg/mL) was added for 7 days prior to downstream functional assays.

For RNA interference (RNAi) silencing, MDA-MB-361 cells were transfected with SMARTpool ON-Target plus siRNAs (small interfering RNAs) targeting AKT1 (L-003000-00-0005) and AKT2 (L-003001-00-0005) or non-targeting siRNAs (D-001810-10-05) (Dharmacon). In brief, 2.5 × 10^5^ MDA-MB-361 or 1.5 × 10^5^ EBC1 cells were seeded in 12-well plate and at the same time transfected with siRNAs (17.5 nM each) against AKT1 and AKT2 or non-targeting (CTR) siRNA in DMEM media containing 10% FBS. At 48 h after transfection, lysates were collected from each experimental condition for western blot analysis and media were changed to DMEM (5% FBS). Media were collected 48 h later for acetate measurement.

### Viability assays

Cell viability and cell death were assessed using the trypan blue exclusion method. Briefly 1–2 × 10^5^ cells were seeded in triplicates into 60 mm plates and allowed to attach overnight. The following day, cells were counted again to establish a baseline reading and treated with different drug concentrations in media containing 5% foetal bovine serum. Cells were harvested after 4 days of drug treatment and counted with a ViCell Cell Viability Analyzer (Beckman). In all figures “cell number (fold change)” refers to the fold change in the number of viable cells between the start of drug treatment and the end of the experiment. All growth assays have been performed a minimum of two times. Shown are results from one representative experiment. Statistical significance was performed using either one- or two-way analysis of variance (GraphPad Prism) and all statistics can be found in Supplementary Table 1. Symbols n.s., *, **, *** and **** represent values that are not significant or having a *P* value ≤ 0.05, 0.01, 0.001 or 0.0001, respectively.

For CellTiter-Glo (CTG) viability assays, 5 × 10^3^ MDA-MB-361 or 2.5 × 10^3^ EBC1 cells were seeded in 96 wells. Following treatments with different drug concentrations, CTG (Promega, G7570) was added and the plates were read in Spectramax I3 reader. The depicted “normalised survival” is the fold change in cell number from the start to end of treatments (4 days), and the curves were created using GraphPad Prism [nonlinear regression, log(inhibitor) vs. response − variable slope (four parameters)].

### Structural modelling

The crystal structure of inhibitor VIII in complex with AKT1 (PDB entry 3O96^12^) was prepared using the default setting of the protein preparation wizard in Maestro release 2017-2 (Schrödinger release 2017-2: Maestro, Schrödinger, LLC, New York, NY, 2018) and used for induced fit docking of MK-2206. The default parameters were applied, except that re-docking was performed with XP settings.

The miransertib-AKT1 crystal structure (PDB entry 5KCV^13^) was employed as a template to create an AKT2 homology model using MOE 2018.0101.^14^ Miransertib was included as environment during model generation and neither the intermediates nor the final model were refined.

Please refer to the Supplementary Information for references regarding the PDB entries and detailed methods and references regarding the MD simulation.

### Cell lysis and sample preparation for mass spectrometry

For each treatment condition, five independent biological replicates were performed. Cells were washed twice with cold phosphate-buffered saline supplemented with 1 mM Na_3_VO_4_ and 1 mM NaF, and lysed in 0.5 mL of urea buffer [8 M urea in 20 mM HEPES (pH 8.0), supplemented with 1 mM Na_3_VO_4_, 1 mM NaF, 1 mM Na_4_P_2_O_7_ and 1 mM β-glycerophosphate]. Cell lysates were further homogenised by sonication (three cycles of 10 s on and 10 s off) and insoluble material was removed by centrifugation. Protein was quantified by the BCI assay. For each replicate, 325 μg of protein was reduced, alkylated and digested with TLCK-trypsin (Thermo Fisher Scientific) as previously described.^15^ The resultant peptide solutions were desalted with C18-Oasis cartridges (Waters, Manchester, UK) as indicated by the manufacturer with slight modifications as previously described.^16^ Enrichment of phosphorylated peptides was performed with TiO_2_ as previously described.^15,16^

### Phosphopeptide detection, identification and quantification

Phosphopeptides were resuspended in 12 μL of reconstitution buffer (20 fmol/µL enolase in 3% acetonitrile, 0.1% trifluoroacetic acid) and 5.0 µL were loaded onto a liquid chromatography with tandem mass spectrometry (LC-MS/MS) system consisting of a Dionex UltiMate 3000 RSLC directly coupled to an Orbitrap Q-Exactive Plus mass spectrometer (Thermo Fisher Scientific) through an EasySpray system. LC-MS/MS was performed as previously described.^15^ Mascot Daemon 2.5.0 was used to automate peptide identification from MS data as indicated before.^15^ Label-free peptide quantification was performed using Pescal, an in-house developed software, that constructed extracted ion chromatograms (XICs) for all identified peptides across all samples (±7 p.p.m. mass and ±2 min retention time windows) and calculated the peak areas of the generated XICs.^15,17^ Normalised peak areas of phosphopeptides were used to calculate fold change and statistical significance between conditions. All raw data and statistical analysis are presented as Supplementary Spreadsheet.

### Kinase substrate enrichment analysis

Kinase activity was estimated from phosphoproteomics data using a kinase substrate enrichment analysis (KSEA) approach.^15,17^ Briefly, phosphorylated peptides were grouped into substrate groups associated to particular kinases as annotated in the PhosphoSite database. *Z*-scores for each kinase were calculated as (mS − mP) × *m*1/2/*d*, where mS is the log_2_ of the mean abundances for each kinase group, mP is the log_2_ of the mean abundances of the whole data set, *m* is the size of each substrate group and *d* is the standard deviation of the mean abundances of the entire data set. Excel software was used to transform *Z*-scores into *P* values. All raw data and statistical analysis are presented as Supplementary Spreadsheet.

### ^1^H-MRS of culture medium

Media from cultured cells (five biological replicates) were analysed by proton magnetic resonance spectroscopy (^1^H-MRS). Five hundred microlitres of culture media and 50 µL of deuterated water (D_2_O, Sigma Aldrich) were placed in a NMR tube, and 50 µL of 0.75% of TSP (sodium 3-(trimethylsilyl)-2,2,3,3-tetradeuteropropionate) in D_2_O (Sigma Aldrich) was added to the samples for chemical shift calibration and quantification.

^1^H-MRS was performed on a Bruker 500 MHz spectrometer (Bruker Biospin, Ettlingen, Germany). ^1^H-MRS: 7500 Hz spectral width, 32 K time domain points, relaxation delay 2.7, 64 scans and temperature 298 K. The water resonance was suppressed by a gated irradiation centred on the water frequency. Spectral processing was carried out using the Bruker Topspin-3 software package. Spectral assignments were based on literature values.^18^

### Colorimetric quantitation of acetate in cell culture media

Briefly, in a 6-well plate, cells were seeded at 1–2 × 10^5^ cells/well and treatments added as indicated. Cell culture media were collected and centrifuged at 13,000 × *g* for 10 min to remove insoluble cell debris. Acetate concentrations in the media were determined using the Acetate Colorimetric Assay Kit (Sigma, MAK086) according to the manufacturer’s instructions and all values were normalised using the cell number.

### DNA extraction and exome sequencing

Genomic DNA was extracted from parental and MK-2206-resistant MD-MB-361 cells using Qiagen DNeasy Blood & Tissue Kit (Cat#: 69504) as per the manufacturer’s instructions. Exome library preparation was done using Illumina Nextera DNA Exome Kit (Cat#: 20020616) as per the manufacturer’s instructions. Library underwent quality control on a 4200 Agilent Tapestation. Paired-end (2 × 150) sequencing was done using a HiSeq 4000 at 50× coverage. The results have been deposited at the ArrayExpress database at EMBL-EBI under accession number E-MTAB-8066 (https://www.ebi.ac.uk/arrayexpress/experiments/E-MTAB-8066).

### Detection of MK-2206 via LC-MS/MS

MDA-MB-361 cells were seeded at 3 × 10^6^ cells per T75 flask in triplicate per timepoint and per cell line. Following MK-2206 incubation (2 µM) or DMSO, culturing media were harvested, whereas cells were trypsinised, counted and lysed to a concentration of 1 million cells per 100 µL (9803, Cell Signalling). An aliquot of lysate or cell media were diluted with acetonitrile:water (1:1), spiked with DMSO and internal standard was added (three volumes per diluted lysate in acetonitrile). Calibration curves were prepared as per treated samples by spiking MK-2206 into lysates or media, respectively, of DMSO-treated cells. All samples were centrifuged and supernatant diluted 1 in 10 with water prior to analysis. Samples were analysed by LC-MS/MS using a Waters (Milford, MA, USA) Acquity I-class plus LC coupled to a Xevo TQ-XS triple quadrupole mass spectrometer with electrospray ionisation in positive ion mode. The LC gradient consisted of formic acid (0.1%) in water (A) and acetonitrile (B) from 10% to 100% B over 4 min on a Kinetex C18 5 cm × 2.1 mm, 1.7 µm UPLC column (Phenomenex, Macclesfield, UK) at 0.6 mL/min. MK-2206 was quantified using the multiple reaction monitoring transition 408.24 > 380.09 at collision energy of 24 eV.

## Results

### Allosteric AKT inhibitors consistently induce more cell death compared to ATP-competitive inhibitors

**Fig. 1:**
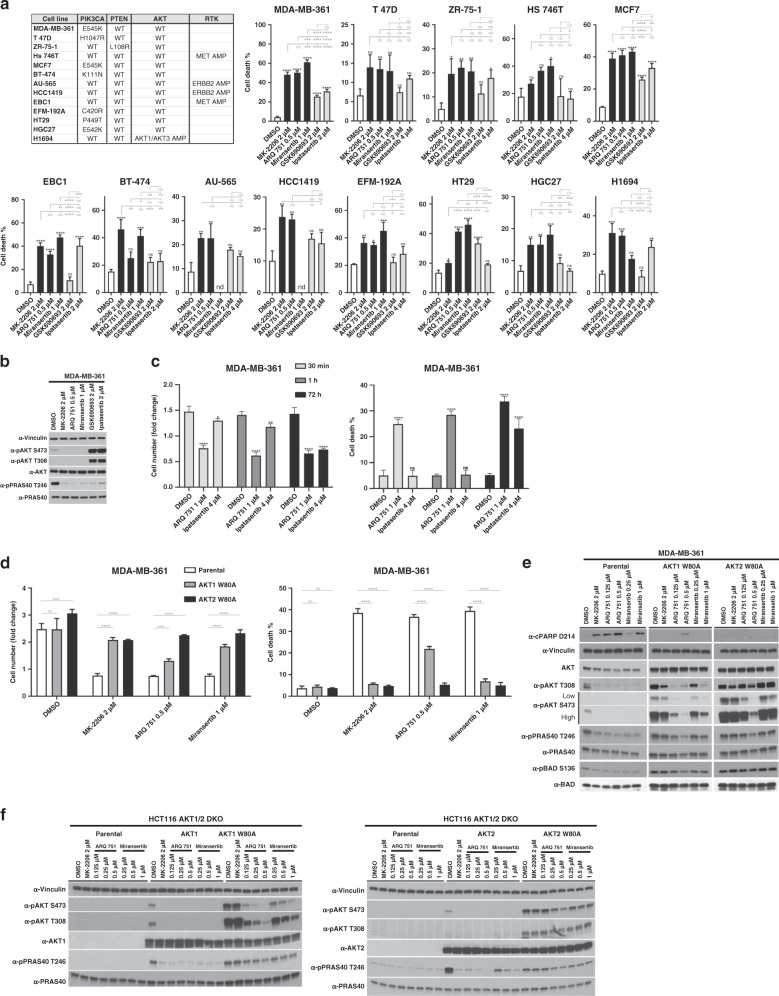
Characterisation of the cell death-inducing effects of various AKT inhibitors and of the W80A mutation on allosteric AKT inhibitor response. **a** Allosteric AKT inhibitors have higher cell death-inducing activity compared to ATP-competitive inhibitors. A panel of cancer cell lines with the various PI3K/AKT-activating lesions (shown in the table on the left) was treated with three allosteric AKT inhibitors (MK-2206, miransertib and ARQ 751) and two ATP-competitive AKT inhibitors (GSK690693 and ipatasertib) at the indicated doses. The fraction of dead cells following 4 days of drug treatment is shown (cell death %). **b** Allosteric and ATP-competitive inhibitors have comparable effects on AKT kinase inhibition. The effects of AKT inhibitors on AKT and AKT substrate phosphorylation was assessed in MDA-MB-361 cells by immunoblot using the indicated antibodies following 18 h of treatment. **c** The cell death-inducing effects of the allosteric AKT inhibitor ARQ 751 and the ATP-competitive inhibitor ipatasertib were compared in washout experiments. Cells were treated with the indicated doses of drug for either 30 min or 1 h. The drug was then removed and cells were extensively washed before allowing them to grow for an additional 72 h. As a control, cells were also continuously exposed to drug for the duration of the experiment. The fold change in cell numbers (left) and the fraction of dead cells following 3 days of drug treatment (right) is shown. **d** The W80A mutation confers resistance to some but not all allosteric inhibitors in an isoform-selective manner. AKT1 W80A or AKT2 W80A was ectopically expressed in MDA-MB-361 cells. Cells were treated with MK-2206, miransertib or ARQ 751 at the indicated doses. The fold change in cell numbers (left) and the fraction of dead cells following 4 days of drug treatment (right) is shown. **e** Cells were also treated for 18 h and lysed. Lysates were analysed by immunoblot with the indicated antibodies. **f** HCT116 AKT1/2 DKO cells were stably transduced with wild-type or W80A variants of either AKT1 or AKT2. Cells were then treated with the indicated drugs and lysed. Lysates were analysed by immunoblot with the indicated antibodies. n.s., not significant. *P ≤ 0.05, **P ≤ 0.01, ***P ≤ 0.001 and ****P ≤ 0.0001.

Although both allosteric and ATP-competitive AKT inhibitors have demonstrated strong single-agent growth inhibitory activity in human cancer cell lines and xenografts, some evidence suggests that only the latter can induce regressions in AKT1 E17K-driven tumours in vivo.^9^ To establish whether ATP-competitive and allosteric AKT inhibitors differ in their ability to induce cell death, we measured the cell-killing effects of treatment with two different ATP-competitive (ipatasertib and GSK690693) and three different allosteric inhibitors (MK-2206, miransertib and ARQ 751) of AKT in a panel of cell lines with genetic lesions known to activate the PI3K/AKT pathway. Our panel included cancer cell lines with activating PIK3CA mutations, inactivating PTEN mutations, AKT gene amplification or amplification of either ERBB2 or MET (both of which have been previously shown to be associated with AKT dependence^19,20^). Based on our previous studies (PMID: 25551293), we chose a dose of 2 μM MK-2206 as our benchmark, as this was sufficient to induce robust cell death in MDA-MB-361 cells. We then performed CTG assays in both MDA-MB-361 and EBC1 cells using a range of doses of GSK690693, ipatasertib, capivasertib, MK-2206, ARQ 751 and miransertib, and selected doses that had similar growth-suppressive effects compared to 2 μM MK-2206 (Supplementary Fig. 1A). We found that in all cell lines tested, allosteric inhibitors (MK-2206, miransertib or ARQ 751) caused more or equal cell death at lower concentrations compared to the two ATP-competitive inhibitors (ipatasertib and GSK690693), despite similar cytostatic activity (Fig. 1a and Supplementary Fig. 1B). Interestingly, we could not detect any significant differences in the extent of inhibition of AKT substrates phosphorylation (PRAS40 and BAD) between ATP-competitive and allosteric AKT inhibitors at concentrations where the latter induce more cell death (Fig. 1b and Supplementary Fig. 1C, D). We also compared the cell-killing effects of ipatasertib and ARQ 751 in washout experiments using four times higher concentrations of the former. We found that as little as 30 min of drug exposure were sufficient to induce significant cell death with ARQ 751, but not ipatasertib, which did not induce any measurable cell death during this time (Fig. 1c). To rule out the possibility that the higher cell-killing activity of allosteric inhibitors was due to off-target effects, we ectopically expressed AKT1 W80A or AKT2 W80A, which we and others have previously shown to confer resistance to MK-2206.^19,21^ As expected, expression of either mutant in drug-sensitive MDA-MB-361 cells completely rescued the growth inhibitory and cell death-inducing effects of MK-2206 (Fig. 1d). However, to our surprise, cells expressing AKT1 W80A and not AKT2 W80A remained significantly sensitive to ARQ 751 (Fig. 1d), suggesting that this mutation does not universally confer resistance to all allosteric inhibitors, and that its effects on drug binding can be isoform-specific. Western blot analysis confirmed that phosphorylation of AKT and AKT substrates, such as PRAS40 and BAD, were sensitive to ARQ 751 treatment and also partially sensitive to miransertib (Fig. 1e). However, this model did not allow us to determine whether the observed biochemical resistance was influenced by the presence of endogenous AKT isoforms. We therefore expressed each mutant in HCT116 cells where AKT1 and AKT2 (the only isoforms expressed in these cells) had been knocked out (hereafter referred to as HCT116 AKT1/2 DKO).^22^ Again, we found that AKT1 W80A was resistant to dephosphorylation induced by MK-2206 and to a lesser degree by miransertib, but remained sensitive to ARQ 751 (Fig. 1f). However, AKT2 W80A was significantly resistant to all three inhibitors at all concentrations tested (Fig. 1f). These data suggested that these clinically relevant allosteric AKT inhibitors differed in their individual requirements for target binding.

### Individual allosteric AKT inhibitors have common and distinct target binding requirements that influence the nature of resistance mutations

**Fig. 2:**
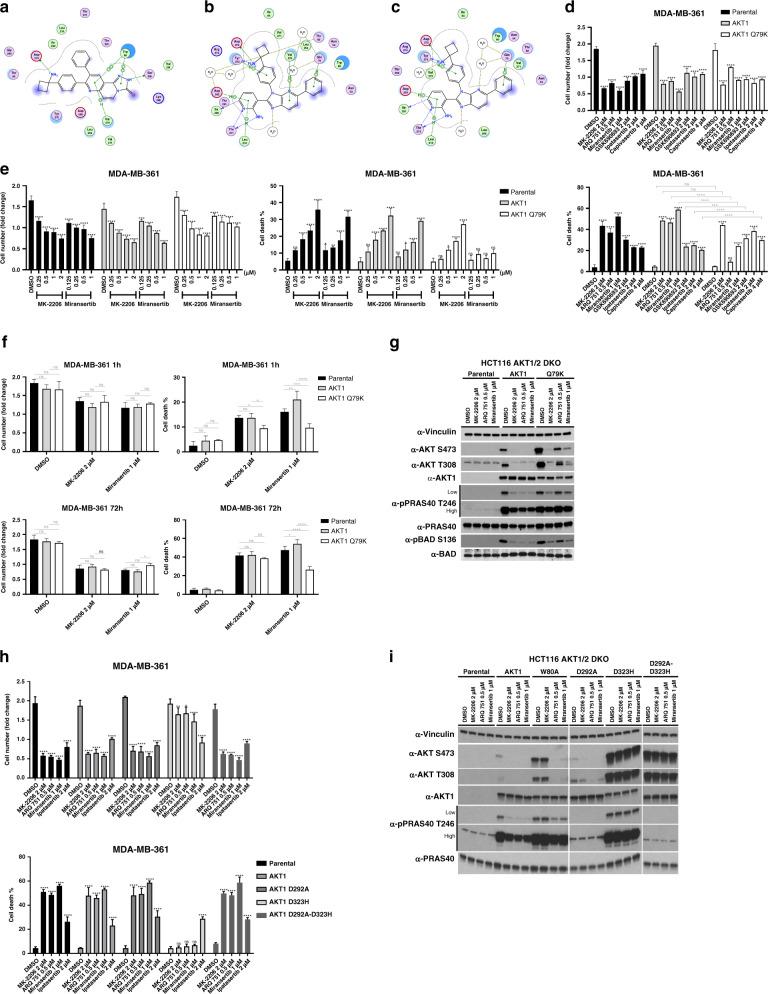
Mutations in both the kinase and PH domains of AKT can cause drug-specific allosteric AKT inhibitor resistance. **a** A docking model of MK-2206 bound to AKT1. **b** Crystal structure of AKT1 bound to miransertib. **c** A homology model of AKT2 bound to miransertib. **d**, **e** Effects of the Q79K mutation on the response to AKT inhibitors. **d** MDA-MB-361 cells were stably transduced with wild-type AKT1 or AKT1 Q79K. Cells were then treated with the indicated doses of MK-2206, ARQ 751, miransertib, GSK690693, ipatasertib or capivasertib. The fold change in cell numbers (top) and the fraction of dead cells (bottom) following 3 days of drug treatment is shown. **e** MDA-MB-361 cells expressing either AKT1 or the Q79K mutant were treated with increasing doses of MK-2206 or miransertib. The fold change in cell numbers (left) and the fraction of dead cells (right) following 3 days of drug treatment is shown. **f** The effects of the AKT1 Q79K mutation in response to MK-2206 and miransertib was evaluated in washout experiments. MDA-MB-361 cells stably expressing wild-type AKT1 or AKT1 Q79K were treated with the indicated doses of drug for either 1 h or continuously for 72 h. The fold change in cell numbers and the fraction of dead cells (bottom) at 72 h is shown. **g** HCT116 AKT1/2 DKO cells were stably transduced with wild-type AKT1 or AKT1 Q79K. Cells were subjected to western blot analysis, following o/n drug treatment, with the indicated antibodies. **h** A mutation that disrupts the kinase-domain/PH-domain interface confers resistance to all allosteric inhibitors. MDA-MB-361 cells were stably transduced with wild-type AKT1 or the mutant variants D323H, D292A (a kinase-deficient mutant), and the double mutant D323H-D292A. Cells were treated with allosteric AKT inhibitors as shown. The fold change in cell numbers and the fraction of dead cells following 4 days of drug treatment is shown (cell death %). **i** HCT116 AKT1/2 DKO cells were stably reconstituted with the indicated AKT variants and treated o/n with allosteric AKT inhibitors as shown. Cells were then lysed and analysed by immunoblot with the indicated antibodies. n.s., not significant. *P ≤ 0.05, **P ≤ 0.01, ***P ≤ 0.001 and ****P ≤ 0.0001.

We next wanted to explore how differences in target binding requirements of individual allosteric inhibitors could help predict drug-specific resistance mutations. Due to the absence of an experimental structure, we generated a docking model of MK-2206 bound to AKT1 and compared it to the miransertib-AKT1 crystal structure (PDB entry 5KCV^13^) to determine possible sites of interaction that were unique to each compound. Our analysis revealed that the side chain of Trp 80 in the PH domain of AKT1 π-stacks with MK-2206 (similar to what has been reported for AKT inhibitor VIII^12^) (Fig. 2a). This is consistent with the observed effects of the W80A mutation on AKT1 phosphorylation and cell survival in response to inhibitor treatment (Fig. 1d–f). In contrast, miransertib seems to engage less extensively with Trp 80 in AKT1 (Fig. 2b). However, ectopic expression of this mutant in MDA-MB-361 cells also confers a significant degree of resistance to miransertib- and ARQ 751-induced cell death (Fig. 1d, right panel) and causes a right-shift in the biochemical response to these drugs both in MDA-MB-361 cells and in our HCT116 AKT1/2 DKO reconstitution model (Fig. 1e, f). To investigate this in more detail, we have conducted 50 ns MD simulations of AKT1 in complex with MK-2206 and miransertib. The centre of imidazo(4,5-*b*)pyridine ring of miransertib is located further away from the W80 indole centroid compared to the centroid of the miransertib tricyclic ring system (Supplementary Fig. 2B). Whenever miransertib and W80 are in closer proximity, the ring systems partly overlap. This can be observed, for example, after 22 or 40 ns of simulation, but does not appear to be stable (Supplementary Fig. 2B, C). Full stacking of both bicycles onto each other requires pronounced changes to the miransertib-binding mode (Supplementary Fig. 2D). Consistent with the static input model, miransertib engages in less stacking interactions with W80 during the simulation. In contrast, W80 forms extensive stacking interactions with the MK-2206 tricyclic core throughout the whole course of the simulation and no changes in the MK-2206-binding mode were observed (Supplementary Fig. 2B, C).

Interestingly, we noticed that the effects of the W80A mutation on AKT and AKT substrate phosphorylation (PRAS40 and BAD) as well as on cell survival in response to miransertib and ARQ 751 were significantly less pronounced in AKT1 compared to AKT2. In fact, ARQ 751 was able to induce cell death in cells expressing AKT1 W80A, but not AKT2 W80A (Fig. 1d–f). To better understand the isoform-specific effects of the W80A mutation on inhibitor response, we created a homology model of miransertib bound to AKT2 (Fig. 2c) as the chemical structure of ARQ 751 is currently unavailable. Surprisingly, we found that Trp 80 interacts with miransertib in a similar manner compared to AKT1. Overall, the majority of residues in close proximity to miransertib are conserved, and the two non-conserved residues (Gln 59 and Ser 205 in AKT1 vs. Gln 59 and Thr 207 in AKT2) cannot explain the observed differences. This suggests that perhaps differences in the kinetics of the PH-domain and kinase-domain interactions in AKT1 vs. AKT2 could regulate the stability of the interface and therefore impact drug binding.

We had also included ATP-competitive inhibitors in our evaluation of W80A mutants as controls. Surprisingly, we have found that the W80A mutation in AKT2 (but not AKT1) also right-shifted the response to ipatasertib (Supplementary Fig. 2A). This was highly unexpected given that the active kinase domain, which primarily exists in the PH-out conformation, should be positioned far away from the PH domain where Trp 80 is. However, we noticed that compared to wild-type AKT2, AKT2 W80A expression led to a significantly lower level of substrate phosphorylation in vehicle-treated HCT116 AKT1/2 DKO cells (Fig. 1f), suggesting that this mutation interferes with the active protein conformation that is required for both catalytic turnover and inhibitor binding. Importantly, this is in contrast to AKT1, where substrate phosphorylation levels do not appear to be affected by the W80A mutation (Fig. 1f). This further highlights major differences in these highly conserved isoforms in response to protein mutation and treatment with a variety of compounds.

Compared to MK-2206, miransertib seems to engage in more extensive contacts with the kinase domain and a different set of contacts with the PH domain, including a hydrophobic interaction with Gln 79 (Fig. 2b, c), a site that has been reported to be mutated in a small number of human tumours (cbioportal.org). We therefore predicted that mutation of this site could selectively confer resistance to miransertib, but not MK-2206. We mutated Gln 79 to a Lys residue and expressed the mutant cDNA in MDA-MB-361 (Fig. 2d–f) and HCT116 AKT1/2 DKO cells (Fig. 2g). Consistent with our prediction, we found that expression of AKT1 Q79K confers biochemical and biological resistance to miransertib and ARQ 751, but not MK-2206 (Fig. 2d–g). This was evident in both dose titration (Fig. 2e) and washout (Fig. 2f) experiments.

Given that the binding pocket targeted by all existing allosteric inhibitors is in the PH-domain/kinase-domain interface, mutational disruption of the interface should lead to resistance to all allosteric inhibitors. Indeed, we see that mutation of Asp 323 to His in AKT1, which has been found to disrupt the interface,^23^ confers biological (Fig. 2h) and biochemical (Fig. 2i) resistance to miransertib, ARQ 751 and MK-2206. This protective effect is not observed when a mutant cDNA that contains both the D323H mutation and a kinase-inactivating mutation (D292A) is expressed (Fig. 2h–i).

### Mutation of the AKT gatekeeper residue “right-shifts” the response to an ATP-competitive inhibitor, but does not affect response to others

**Fig. 3:**
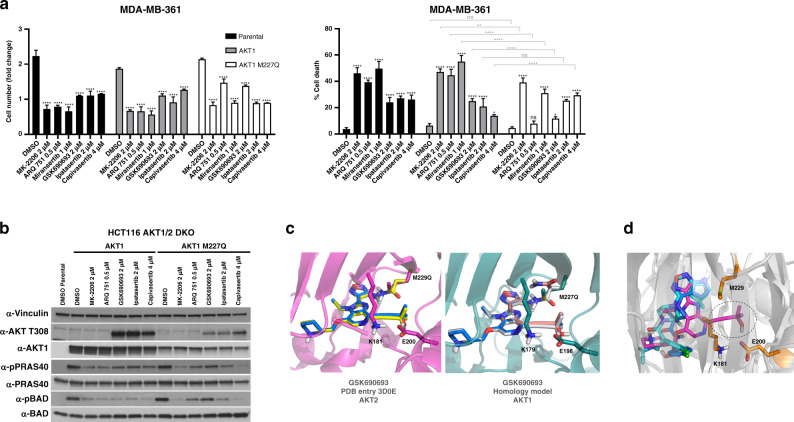
The M227Q gatekeeper mutation confers resistance to select ATP-competitive and allosteric AKT inhibitors. **a** MDA-MB-361 cells were stably transduced with wild-type AKT1 or AKT1 M227Q. Cells were then treated with the indicated doses of various AKT inhibitors as shown. The fold change in cell numbers (left) and the fraction of dead cells (right) following 4 days of drug treatment is shown. **b** HCT116 AKT1/2 DKO cells stably transduced with either wild-type AKT1 or AKT1 M227Q were treated with the indicated doses of various AKT inhibitors as shown. Cells were lysed following treatment and analysed by western blot with the indicated antibodies. **c** Crystal structure of AKT2 bound to GSK690693 (left) and a homology model of AKT1 bound to GSK690693 (right). The proteins in the wild-type models are depicted in magenta (left side, AKT2 crystal structure) and dark green (right side, AKT1 homology model), whereas GSK690693 is shown in blue. In the mutant models, the compound and mutant residue conformations are coloured yellow (left side) and rose and grey (right side, please note that two different M227Q side chain rotamers are included), respectively. **d** Alignment of the crystal structures of GSK690693 (magenta, PDB entry 3D0E, in complex with AKT2), ipatasertib (light blue, PDB entry 4EKL, in complex with AKT1) and capivasertib (dark blue, PDB entry 4GV1, in complex with AKT1). GSK690693 extends to an area between the gatekeeper M229 (orange sticks) and the K181–E200 salt bridge (orange sticks), which is not occupied by ipatasertib and capivasertib. We have highlighted the part of GSK690693 that extends into this area with dotted lines. The protein in all three complexes is depicted as grey cartoon. n.s., not significant. *P ≤ 0.05, **P ≤ 0.01, ***P ≤ 0.001 and ****P ≤ 0.0001.

The active site of protein kinases, including AKT, contains a residue that partially or fully blocks a hydrophobic region in the ATP-binding pocket. This residue is referred to as the gatekeeper, and mutations of this site are the mechanistic basis for resistance to a number of clinically relevant ATP-competitive inhibitors of oncogenic kinases. Given the encouraging data from phase 2 clinical trials of capivasertib in AKT mutant cancers, understanding the impact of mutations on the response to this class of drugs could be critical in predicting potential mechanisms of acquired resistance. We therefore mutated the gatekeeper residue Met 227 in AKT1, and assessed its impact on the response to various ATP-competitive and allosteric AKT inhibitors. We find that mutation of Met 227 to Gln (M227Q) significantly affected the biochemical and biological response (in the HCT116 AKT1/2 DKO and MDA-MB-361 cell line models, respectively) to GSK690693, while having no effect on ipatasertib (Fig. 3a, b). Interestingly, cells were more sensitive to capivasertib as shown by an increase in cell death and decreased phosphorylation of PRAS40 and BAD proteins. (Fig. 3a, b). The crystal structure of AKT2 bound to GSK690693 (PDB entry 3D0E^24^) shows that this compound extends to an area between the gatekeeper and the Lys181–Glu200 salt bridge that is otherwise unoccupied in the capivasertib and ipatasertib structures (Fig. 3d). This both restricts the available space and creates a hydrophobic environment around the gatekeeper. To assess the impact of the M227Q mutation on GSK690693 binding computationally, we employed the protein design program Osprey version 2.2 beta^25,26^ as described previously.^27^ This program estimates ligand affinity by calculating a so-called K* score, where higher values predict tighter binding. Indeed, the results suggested decreased binding of GSK690693 to the corresponding AKT2 M229Q mutant (K* scores of 2.97e^+59^ and 6.9e^+52^ for wild-type and M229Q, respectively), but not complete loss due to, for example, steric incompatibilities (Fig. 3c, left panel), consistent with the observed right-shift. AKT1 and AKT2 residues within the ATP-binding site are highly conserved. However, to ensure that our modelling scores are not due to an AKT2 effect, we repeated the analysis with a GSK690693-bound AKT1 homology model and obtained similar results (K* scores of 1.6e^+59^ and 5.9e^+52^ for wild-type and M227Q, respectively) (Fig. 3c, right panel).

Surprisingly, we found that this gatekeeper mutation also significantly lowers the sensitivity to the various allosteric inhibitors tested (MK-2206, ARQ 751 and miransertib) (Fig. 3a, b), suggesting that the stability or the conformational kinetics of the kinase-domain/PH-domain interface might be affected by the introduction of a polar group at the gatekeeper site that is in close proximity.

### Allosteric and ATP-competitive inhibitors of AKT have distinct effects on downstream signalling

**Fig. 4:**
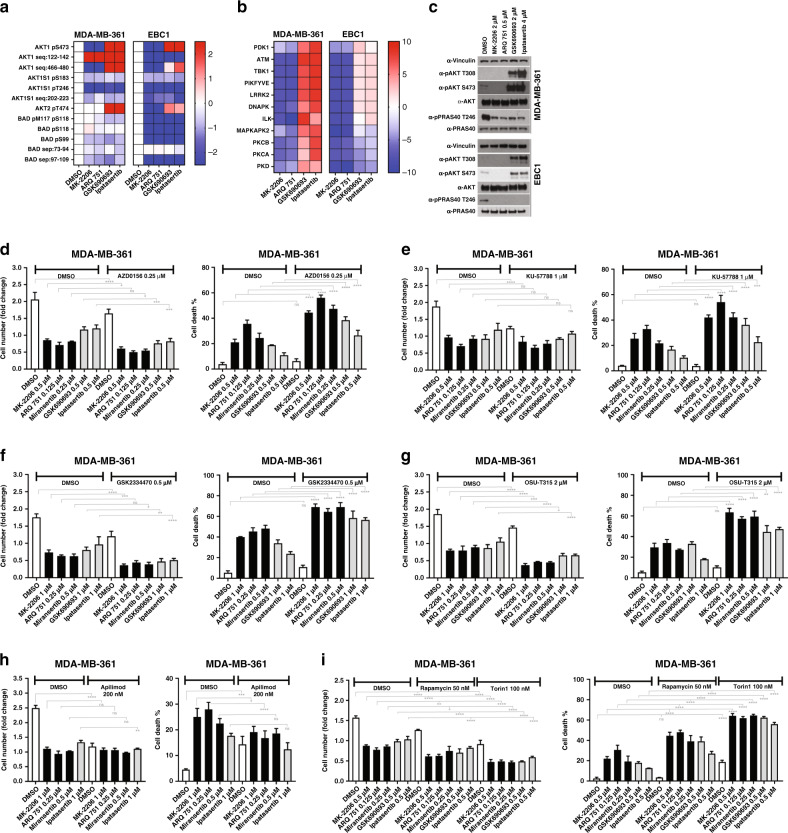
Phosphoproteomic signatures of AKT inhibitors uncover potential therapeutic co-targets. **a** MDA-MB-361 and EBC1 cells were treated with the indicated drugs for 6 h and lysed. Lysates were subjected to phosphopeptide enrichment, and analysed by quantitative LC-MS/MS. Shown are the quantification of mass spectra corresponding to known AKT substrates. **b** Phosphoproteomic data was analysed by kinase substrate enrichment analysis (KSEA). Shown are all kinases predicted to be associated with the observed phosphopeptide signatures, that were affected differently by each inhibitor class (i.e. ATP-competitive vs allosteric) but similarly by both inhibitors of the same class in at least one of the cell lines. **c** Lysates from **a**, **b** were also subjected to immunoblot to further document drug activity using the indicated antibodies. **d**–**g** MDA-MB-361 cells were treated with various AKT inhibitors either alone or in combination with the ATM inhibitor AZD0156 (**d**), the DNA-PK inhibitor KU-57788 (**e**), the PDK1 inhibitor GSK2334470 (**f**), or the ILK inhibitor OSU-T315 (**g**)**. h**, **i** MDA-MB-361 cells were also treated with various AKT inhibitors either alone or in combination with the PIKFYVE inhibitor apilimod (**h**), or the mTORC inhibitors rapamycin or torin1 (**i**) at the indicated doses. The fold change in cell number and fraction of dead cells following 4 days of drug treatment is shown (cell death %). n.s., not significant. *P ≤ 0.05, **P ≤ 0.01, ***P ≤ 0.001 and ****P ≤ 0.0001.

Given the differential pattern of cell-killing activity between allosteric and ATP-competitive inhibitors, we reasoned that each inhibitor class might be associated with distinct effects on downstream signalling. We therefore treated AKT-dependent MDA-MB-361 and EBC1 cells with two allosteric (MK-2206 and ARQ 751) and two ATP-competitive inhibitors (GSK690693 and ipatasertib) and profiled their effects on the phosphoproteome using MS (Fig. 4a, b). Drug concentrations were chosen based on similar potency in suppression of cell proliferation and AKT substrate phosphorylation in cells. We confirmed target engagement with all inhibitors tested in the phosphoproteome data as demonstrated by a decrease in the phosphorylation of known AKT substrates in samples that were treated with all AKT inhibitors, and an increase or a decrease in AKT phosphorylation in samples that were treated with ATP-competitive or allosteric AKT inhibitors, respectively (Fig. 4a). On western blots, we observed a decrease in the phosphorylation of the AKT substrate PRAS40 following treatment with all four inhibitors (Fig. 4c). Consistent with known mechanisms of action, we also detected either an increase or a decrease in the phosphorylation of AKT following treatment with ATP-competitive or allosteric inhibitors, respectively (Fig. 4c). We found identical results when we quantified our MS data, including the increase in AKT Ser473/Ser474 phosphorylation, which has previously been difficult to observe in MS experiments (Fig. 4a). To identify effector kinases whose function might be selectively altered by one class of AKT inhibitors, we carried out KSEA and asked whether treatment with both the allosteric inhibitors or both the ATP-competitive inhibitors were associated with a significant enrichment (positive or negative) of any kinase signatures. We identified seven potential kinases (PDK1, ATM, TBK1, PIKFYVE, LRRK2, DNA-PK, ILK) whose activity was differentially affected by active-site AKT inhibitors vs. allosteric inhibitors in both cell lines tested (Fig. 4b). We hypothesised that these kinases could represent signalling intermediates in survival pathways that were regulated by aspects of AKT function differentially targeted by each inhibitor class. To assess whether inhibition of any of these kinases impaired or enhanced the cell-killing activity of AKT inhibitors, we combined inhibitors of each kinase with either ATP-competitive or allosteric inhibitors of AKT. We excluded the use of TBK1 inhibitors because currently available compounds are not sufficiently selective.^28,29^ We chose to use lower concentrations of AKT inhibitors than we had used previously to both minimise the potentially additive off-target effects of the drug combinations, and to maximise the probability of seeing enhancing effects. First, we examined the effects of ATM and DNA-PK inhibitors because both of these kinases are regulators of the DNA damage response and AKT has previously been shown to promote DNA repair and checkpoint activation.^30^ We found that the ATM inhibitor AZD0156 (Fig. 4d) or the DNA-PK inhibitor KU-57788 (Fig. 4e) potentiated the cell death-inducing effect of AKT inhibitors in MDA-MB-361 cells (and to a lesser degree in EBC1 cells; Supplementary Fig. 3A), despite having no detectable effects as single agents. The biochemical activity of the various compounds was documented via western blot by assessing phosphorylation of relevant substrates and downstream effectors (Supplementary Fig. 3B). Although the magnitude of the effect was variable, we observed a similar pattern of cell killing when we tested these combinations in one additional PIK3CA-mutant breast cancer cell line, namely BT-474 (Supplementary Fig. 3C). Next, we looked at the effects of combining AKT inhibitors with inhibitors of either PDK1 or ILK. PDK1 phosphorylation of AKT is necessary for its activation and has been shown to be involved in negative feedback regulation of PI3K signalling.^31^ Therefore, it is possible that its activation could mitigate the effects of AKT inhibitors. ILK, on the other hand, has been shown to directly modulate AKT activity.^32^ Indeed, we found that the combination of the PDK1 inhibitor GSK2334470 (Fig. 4f) or the ILK inhibitor OSU-T315 (Fig. 4g) with AKT inhibitors of either class potentiated cell death in all cell lines tested (Fig. 4f, g and Supplementary Fig. 4A–C), including MK-2206-resistant cells. We also assessed the effects of the LRRK2 inhibitor GSK2578215A in combination with either MK-2206 or ipatasertib, and found that this compound did not alter the response to AKT inhibitors and did not have any single-agent activity in either MDA-MB-361 or BT-474 cells (Supplementary Fig. 4D, E). Finally, we examined the effects of combining AKT inhibitors with the PIKFYVE inhibitor apilimod and found that it significantly blunted the cell-killing effect of allosteric AKT inhibitors in all cell lines tested, but only modestly altered the effects of ATP-competitive inhibitors (Fig. 4h, Supplementary Figs. 3C, 5A). PIKFYVE is a PI5 kinase that regulates lysosomal function. Inhibition of PIKFYVE has been shown to block autophagy by impairing lysosomal maturation.^33^ Therefore, our data suggest that the cell-killing activity of AKT inhibitors might require functional autophagic flux. Consistently, we see induction of the autophagic marker LC3B in MDA-MB-361 cells treated with MK-2206, but not GSK690693 (Supplementary Fig. 5B). We therefore hypothesised that the pharmacological induction of autophagy would facilitate AKT inhibitor-induced cell killing. Given that mammalian target of rapamycin (mTOR) is a critical cellular inhibitor of autophagy, we decided to combine a variety of ATP-competitive or allosteric AKT inhibitors with inhibitors of mTOR. Indeed, we found that the combination of AKT inhibitors with either rapamycin or torin1 significantly potentiated the cell-killing activity of five different AKT inhibitors in MDA-MB-361 cells (Fig. 4i and Supplementary Fig. 6).

### Acquired resistance to MK-2206 can be driven by mechanisms that do not involve AKT mutation, but confer biochemical resistance to the drug

**Fig. 5:**
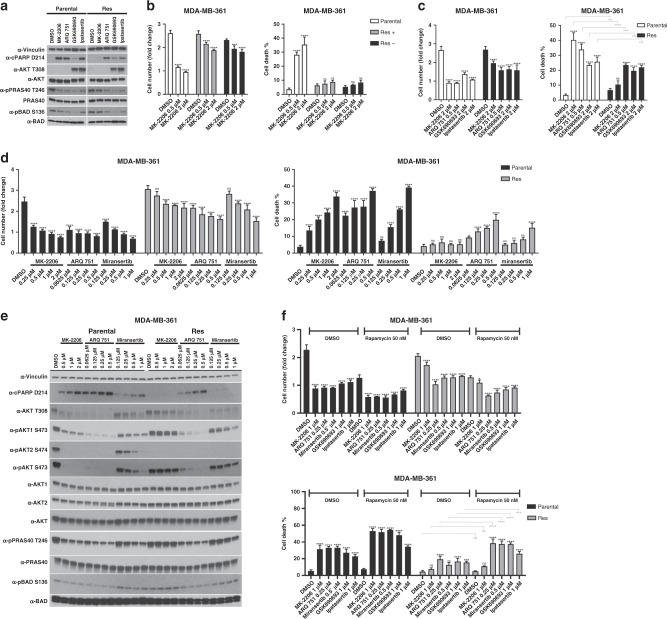
Acquired biological resistance to MK-2206 is associated with biochemical AKT1 resistance that is not mediated by AKT mutation. **a** MDA-MB-361 cells were chronically treated with 2 μM MK-2206 for 12 weeks. Surviving cells were removed from treatment and re-challenged with the indicated drugs for 18 h. Cells were lysed and lysates examined by immunoblot with the indicated antibodies. **b** Parental and MK-2206- resistant MDA-MB-361 cells cultured in MK-2206 (Res +) or not (Res −) were treated with the indicated doses of MK-2206. The fold change in cell numbers (left) and the fraction of dead cells (right) following 4 days of drug treatment is shown. **c** Parental and MK-2206-resistant MDA-MB-361 cells were treated with the indicated doses of ATP-competitive and allosteric AKT inhibitors. The fraction of dead cells following 4 days of drug treatment is shown (cell death %). **d** Parental and MK-2206-resistant MDA-MB-361 cells were treated with the indicated doses of various allosteric AKT inhibitors. The fold change in cell numbers (left) and the fraction of dead cells (right) following 4 days of drug treatment is shown. **e** Cells treated as in **d** were also lysed following 24 h of drug treatment, and lysates were examined by western blot using the indicated antibodies. **f** MDA-MB-361 cells were treated with various AKT inhibitors either alone or in combination 50 nM rapamycin. The fold change in cell numbers (top) and the fraction of dead cells (bottom) following 4 days of drug treatment is shown. n.s., not significant. *P ≤ 0.05, **P ≤ 0.01, ***P ≤ 0.001 and ****P ≤ 0.0001.

In order to model acquired resistance to an allosteric AKT inhibitor, we exposed MDA-MB-361 cells to a high dose of MK-2206. Drug treatment led to significant cell death with only a small fraction of cells surviving after 12 weeks of treatment. Surviving cells were expanded in the presence of drug for an additional 2 weeks. Western blot analysis showed that phosphorylation of AKT and two of its substrates (PRAS40, BAD) were unaffected by MK-2206 in the resistant subline (Fig. 5a, e). To assess whether the resistance phenotype was stable, we cultured the resistant population for 4 weeks in the absence of drug (Res −) and then re-challenge with MK-2206. Cells remained drug resistant following this 4-week drug holiday, suggesting that the phenotype was indeed stable (Fig. 5b).

In order to examine whether AKT remained a relevant therapeutic target in these cells, we evaluated the response of MK-2206-resistant cells to additional allosteric and ATP-competitive AKT inhibitors (Fig. 5c, d). We find that resistant cells remained sensitive to both ipatasertib and GSK690693. Interestingly, these cells also showed a blunted response to both miransertib and ARQ 751 (Fig. 5d).

We also assessed the effects of MK-2206, ARQ 751 and miransertib on the phosphorylation of total AKT, AKT1 or AKT2, as well as on phosphorylation of the AKT substrates PRAS40 and BAD, in both parental and MK-2206-resistant MDA-MB-361 cells (Fig. 5e). We found that compared to parental cells, drug-resistant cells showed no changes in phosphorylation of total AKT (on both T308 and S473) or AKT1 in response to MK-2206, and showed no evidence of AKT2 phosphorylation in the presence or absence of drug (Fig. 5e). Consistently, MK-2206 treatment had little to no effect on the phosphorylation of PRAS40 or BAD in MK-2206-resistant cells, compared to parental controls (Fig. 5e). We also found that phosphorylation of total AKT and AKT1 was significantly (but not completely) resistant to miransertib treatment, as was phosphorylation of both PRAS40 and BAD. Furthermore, we found that compared to parental cells, AKT phosphorylation was only slightly protected (most clearly seen on total pAKT S473) from ARQ 751 treatment in MK-2206-resistant cells and only at the lowest doses, and AKT substrate (PRAS40 and BAD) phosphorylation remained highly sensitive to this compound (Fig. 5e). Given these results and our functional mutational analysis, we hypothesised that the mechanism of resistance could involve the acquisition of an AKT1 mutation. We therefore carried out whole-exome sequencing in our parental and MK-2206-resistant cells. To our surprise, we could not detect any changes in the coding sequences of either AKT1 or AKT2 in MK-2206-resistant cells, suggesting that biochemical protection from drug-induced dephosphorylation does not occur as a consequence of AKT mutation. Next, we examined intracellular MK-2206 drug concentrations following 1 or 6 h of drug exposure and found that resistant cells showed lower drug accumulation, suggesting that drug efflux could contribute to the observed biochemical resistance (Supplementary Fig. 7).

Finally, given that rapamycin is a clinically approved drug, and that our data demonstrate enhanced cell death when combining rapamycin with AKT inhibitors, we wanted to know whether acquired resistance to MK-2206 could be overcome with this particular drug combination. Indeed, we found that although MK-2206-resistant cells remain largely resistant to the MK-2206/rapamycin combination, treatment with ARQ 751, miransertib, GSK690693 or ipatasertib in combination with rapamycin did induce significant cell death in drug-resistant cells (Fig. 5f).

### Acetate excretion is a functional readout of non-catalytic AKT function, and can be perturbed by allosteric AKT inhibitors

**Fig. 6:**
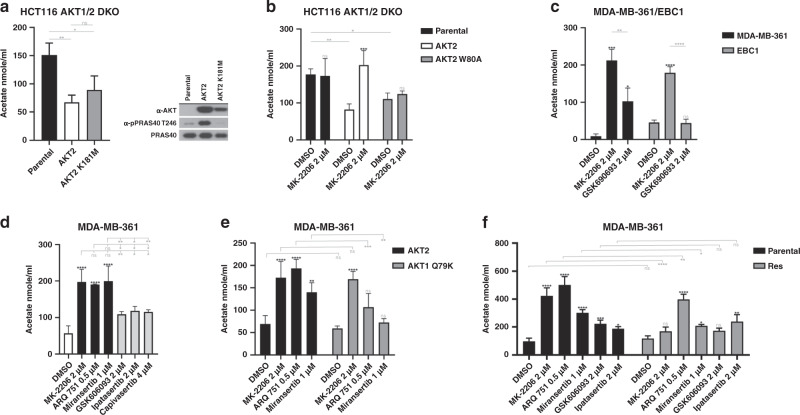
AKT regulates acetate excretion independent of kinase activity. **a** Conditioned media from HCT116 AKT1/2 DKO cells stably transduced with either wild-type (AKT2) or kinase-dead AKT2 (AKT2 K181M) was collected for acetate quantification. Acetate was quantified using a colorimetric assay (left). Expression of AKT2 was confirmed by western blot (right). **b** HCT116 AKT1/2 DKO cell stably transduced with wild-type (AKT2) or MK-2206-resistant AKT2 (AKT2 W80A) were cultured for 24 h in the presence or absence of MK-2206 as indicated. Culture media was collected following treatment and acetate levels were assessed with a colorimetric assay. **c** MDA-MB-361 and EBC1 cells were cultured for 24 h in the presence or absence of MK-2206 or GSK690693 as indicated. Culture media was collected following treatment and acetate levels were assessed with a colorimetric assay. **d** MDA-MB-361 cells were cultured for 24 h in the presence or absence of three allosteric (MK-2206, ARQ 751 and miransertib) or three ATP-competitive (GSK690693, ipatasertib and capivasertib) AKT inhibitors. Culture media was collected following treatment and acetate levels were assessed with a colourimetric assay. **e** MDA-MB-361 cells stably transduced with either wild-type AKT1 or AKT1 Q79K were cultured for 24 h in the presence or absence of MK-2206, ARQ 751, miransertib, GSK690693, or ipatasetrtib as indicated. Culture media was collected following treatment and acetate levels were assessed with a colourimetric assay. **f** Parental or MK-2206-resistant MDA-MB-361 were cultured for 24 h in the presence or absence of the indicated drugs. Culture media was collected and acetate levels were assessed with a colorimetric assay. n.s., not significant. *P ≤ 0.05, **P ≤ 0.01, ***P ≤ 0.001 and ****P ≤ 0.0001.

Our data suggest that allosteric AKT inhibitors can target aspects of AKT function that cannot be effectively inhibited by ATP-competitive compounds. Furthermore, we have previously shown that AKT can signal through non-catalytic functions,^19^ but biomarkers of kinase-independent function have not yet been identified. We hypothesised that allosteric AKT inhibitors could be better suppressors of non-catalytic function compared to ATP-competitive inhibitors, and set out to identify biomarkers of these kinase-independent functions. Given that AKT-dependent phosphorylation cannot explain certain aspects of AKT-dependent cellular metabolism,^34^ we decided to explore the potential involvement of non-catalytic AKT function in regulating metabolite concentrations. First, we used MRS to measure the concentration of 16 different metabolites in cell culture media from AKT-deficient and AKT-reconstituted cells. Of these, four metabolites (lactate, glucose, formate and acetate) showed a significant change following AKT reconstitution. Of these four, acetate had the greatest fold change and the highest statistical significance. Therefore, we decided to focus on acetate for our follow-up work. Our results showed that excretion of acetate was significantly decreased when AKT2 expression was reconstituted through retroviral transduction of AKT1/2-deficient HCT116 (DKO) cells (Supplementary Table 2). A similar decrease was observed using a commercially available acetate detection Colourimetric Assay Kit (Fig. 6a). We also asked whether this effect required AKT catalytic function, and found that the decrease in acetate excretion could be induced through expression of either wild-type kinase-competent AKT2 or a kinase-deficient AKT2 mutant (AKT2 K181M) in AKT1/2 DKO HCT116 cells. Unless, AKT2 K181M is able to retain some level of kinase activity that is below our level of detection, these data suggest that this function of AKT does not require catalytic activity (Fig. 6a). To confirm our results, we simultaneously knocked down AKT1 and AKT2 in MDA-MB-361 or EBC1 through either RNAi or CRISPR (clustered regularly interspaced short palindromic repeats), and we found a significant increase in acetate excretion following AKT knockdown (Supplementary Fig. 8A, B). We also examined AKT1/2 DKO HCT116 or DLD-1 cells and found that, compared to their AKT-proficient parental counterparts, acetate excretion was increased (Supplementary Fig. 8C). We then asked whether the observed decrease in acetate excretion could be reversed by AKT inhibitors. Indeed, we found that the decrease in acetate excretion could be reversed by treatment with MK-2206 in cells that expressed wild-type AKT2, but not the MK-2206-resistant AKT2 W80A mutant (Fig. 6b). We then assessed the effects of AKT inhibitors on acetate excretion in MDA-MB-361 and EBC1 cells (Fig. 6c, d). We found that three different allosteric AKT inhibitors were significantly more efficient at inducing acetate excretion compared to three different ATP-competitive inhibitors. In fact, no increase in acetate excretion could be detected in EBC1 cells treated with GSK690693 compared with MK-2206. We also found that ectopic expression of AKT1 Q79K in MDA-MB-361 cells significantly impaired miransertib- and ARQ 751-induced acetate excretion compared to cells ectopically expressing wild-type AKT1 (Fig. 6e), suggesting that the drug effect on acetate excretion was indeed due to inhibition of AKT. These data strongly suggest that acetate excretion can be regulated by a kinase-independent function of AKT that is highly sensitive to allosteric AKT inhibitors, but virtually insensitive to ATP-competitive inhibitors. Finally, we wanted to know whether acquired resistance to MK-2206 had an impact on drug-induced acetate excretion. Indeed, we found that in MK-2206-resistant MDA-MB-361 cells, MK-2206-induced acetate excretion was severely impaired (Fig. 6f). Furthermore, acetate excretion after treatment with ARQ 751 was only partially impaired, while the impairment was more pronounced following treatment with miransertib, mirroring the cell-killing effects of these compounds in MK-2206-resistant cells (Figs. 6f, 5d).

## Discussion

Recent encouraging data from biomarker-driven clinical trials of AKT inhibitors have re-boosted enthusiasm about the clinical utility of these compounds as single agents, and more generally, about AKT as a therapeutic target in oncology. Despite these emerging signs of clinical activity, the rarity of the genotype that defines the sensitive patient population remains at odds with the much broader pre-clinical therapeutic effects reported for many AKT-targeting agents that have subsequently failed in the clinic. This discrepancy is likely due to a number of different factors including the poor predictive value of some pre-clinical models, the complexity of the PI3K pathway and the impact that co-occurring mutations of several pathway components may have on response to inhibition of a single node, as well as the differences in the pharmacological requirements that define the anti-tumour activity of individual AKT inhibitors. Given the rich repertoire of AKT inhibitors in clinical development, we carried out a systematic study of the most advanced compounds to better understand and define the context of their anti-tumour activity.

Our work shows that in cell lines with active wild-type AKT, allosteric inhibitors are more potent inducers of cell death compared to ATP-competitive inhibitors, particularly in washout experiments wherein cancer cells are not continuously exposed to these drugs. This is an important pharmacological distinction because, in humans, exposure of tumours to peak biologically active drug concentrations is generally transient, suggesting that ATP-competitive inhibitors may not satisfy the pharmacokinetic requirements for meaningful target engagement. It also warrants more careful interrogation of intermittent dosing schedules using high-dose allosteric inhibitors in AKT-active tumours.

We have also found that mutations that confer resistance to one allosteric inhibitor do not always confer resistance to other allosteric inhibitors. In one case, we found that a clinically relevant activating AKT1 mutation,^35,36^ Q79K, confers resistance to miransertib, but not MK-2206, further highlighting the importance of understanding AKT genotype in order to select the appropriate targeting agent. Similarly, specific mutations can selectively confer resistance to some but not all ATP-competitive inhibitors. Of note, we found that the ability of some of these mutations to confer resistance depends on whether they target a specific AKT isoform. Given that the allosteric-binding site is held in place by inter-domain interactions between the PH and kinase domains of AKT, our data suggest that the nature of these interactions may be sufficiently different between individual AKT isoforms to modulate drug binding and possibly basal activity. It also suggests that the conformation kinetics of the interface might be critical in allowing drug access, and that mutations that alter this feature could also confer resistance or enhance sensitivity.

Consistent with the difference in cell death-inducing potency, we found, through phosphoproteomic profiling, that allosteric and ATP-competitive inhibitors can have distinct effects on signal transduction. This provides evidence that each compound class may differentially target different aspects of AKT function or have different effects on feedback regulation. Furthermore, we provide evidence that these phosphopeptide signatures can be used to identify kinase drug targets whose inhibition can effectively induce cell death when combined with AKT inhibitors, even in cells with acquired AKT inhibitor resistance. Interestingly, these potentiated effects were observed with both classes of AKT inhibitors, despite the fact that allosteric inhibitors suppressed, and ATP-competitive inhibitors increased the phosphorylation output of these kinases. These data suggest that allosteric inhibitors may partially block some of the downstream effector kinases of AKT that are important for AKT-dependent survival, while ATP-competitive inhibitors may cause their activation possibly through feedback relief. Importantly, we have also identified acetate excretion as a functional readout of AKT function that is independent of kinase activity and that is significantly more sensitive to allosteric AKT inhibitors. Given the role of non-catalytic AKT function in cancer cell survival,^19^ the incorporation of this type of readout in assay cascades for the discovery or optimisation of new AKT inhibitors could prove critical in improving the therapeutic potential of AKT-targeting agents.

Finally, we have identified a previously unknown mechanism of AKT inhibitor resistance, which involves biochemical protection of the primary drug target (AKT1 or AKT2 in this case) through a non-mutational event. This is an important development in our understanding of kinase inhibitor resistance mechanisms because it suggests that although the primary drug target remains relevant, its inactivation may require inhibition of another molecule, which could directly or indirectly impair drug binding. Our measurements of intracellular MK-2206 concentration in parental and resistant cells suggest that the observed biochemical resistance could be, at least partly, due to the activation of a drug efflux pump. However, the fact that the induced effect of combining MK-2206 with either GSK2334470 or OSU-T315 was partially retained (Supplementary Fig. 4C) suggests that some free MK-2206 can be accumulated in these cells.

Our work shows the importance of defining, in molecular detail, the mechanism of action of individual AKT inhibitors to understand their distinct biological effects. More broadly, it demonstrates the power of robust comparative pharmacology studies in determining the most favourable context for the use of specific therapeutic inhibitors with a shared primary target, and it highlights the benefits of pharmacological diversity.

## Supplementary information

Supplementary Information

## Data Availability

The whole-exome sequencing data described in this manuscript has been deposited in the publicly accessible ArrayExpress database (accession E-MTAB-8066). All other datasets can be found as part of this manuscript.
