# Supplementary material for: A systematic molecular and pharmacologic evaluation of AKT inhibitors reveals new insight into their biological activity
Source: Br J Cancer. 2020 May 22;123(4):542–55. doi: 10.1038/s41416-020-0889-4 (PMC7435276; doi:10.1038/s41416-020-0889-4)
Supplement: Supplementary file 1 — Supplementary Information [file 41416_2020_889_MOESM1_ESM.pdf]

Supplementary figure 1

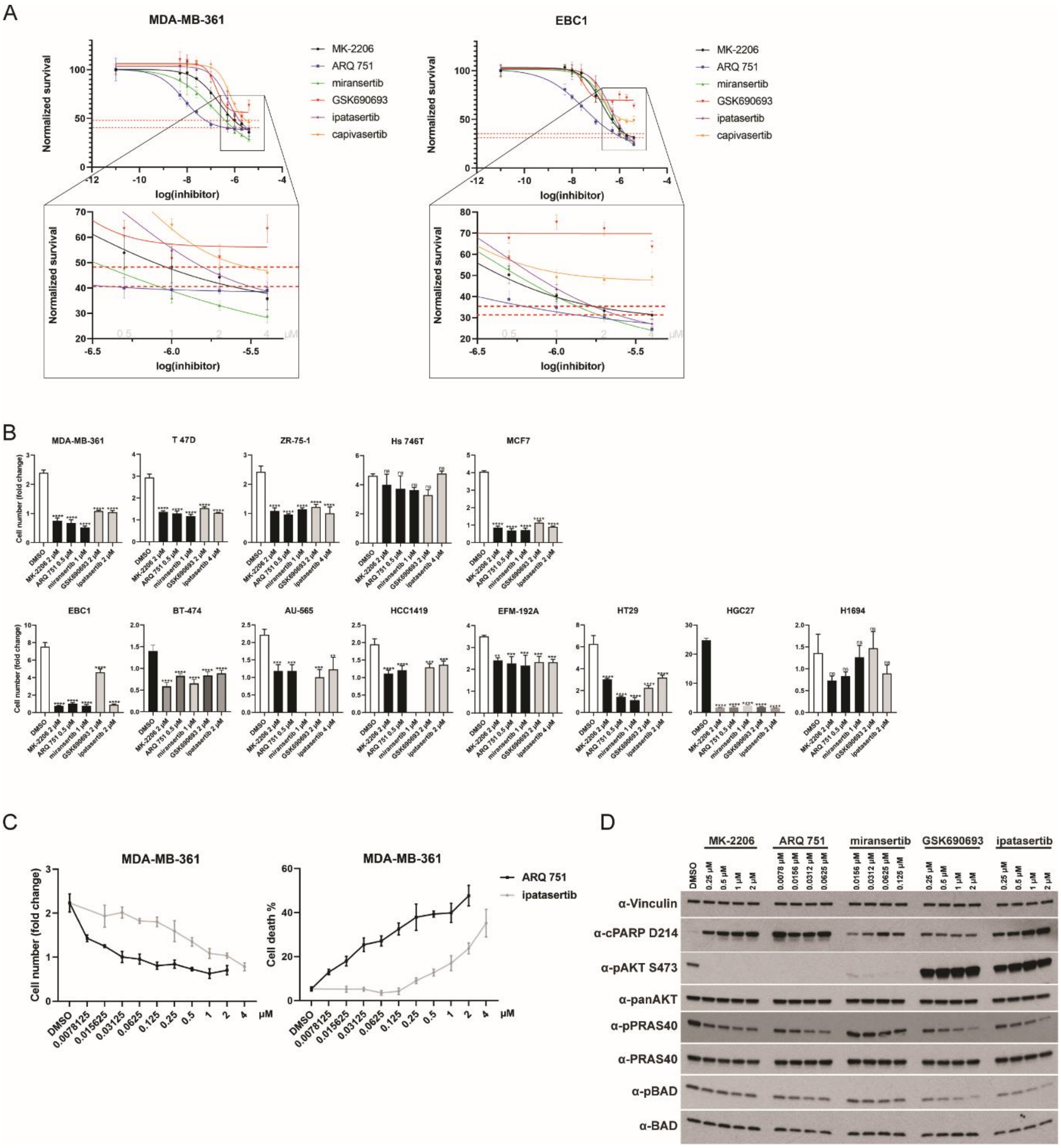

Sup. figure 1: A-B) Effect of AKT inhibitors in MDA-MB-361 cell growth. A) MDA-MB-361 or EBC1 cells were cultured in 96 well and the inhibition of cell growth (fold change in CTG luminescence) is shown after treatment with different concentrations of MK-2206, ARQ 751, miransertib, GSK690693, ipatasertib and capivasertib. The effect of 2 µM of MK-2206 was selected as a reference (dotted red line) in order to compare the response in the rest drug treatments (zoomed panel). **B)** Fold change in cell numbers of the cell lines shown in figure 1A. A baseline cell count was done prior to drug treatment. Cells were then counted following 4 days of drug treatment. Fold change is expressed as the ratio of the day 4 count and the baseline count. **C-D) Allosteric AKT inhibitor ARQ 751 induces more cell death compared to ipatasertib at doses that have similar effects on AKT substrate phosphorylation. C)** MDA-MB-361 cells were treated with increasing doses of either ARQ 751 or ipatasertib. The fold change in cell numbers (left) and the fraction of dead cells (right) following 4 days of drug treatment is shown. **D)** The biochemical effects of allosteric (MK-2206, ARQ 751, and miransertib) and ATP-competitive (GSK690693 and ipatasertib) AKT inhibitors was evaluated by western blot. MDA-MB-361 cells were treated o/n with the indicated doses of each compound and subjected to immunoblot with various antibodies as shown.

Supplementary figure 2

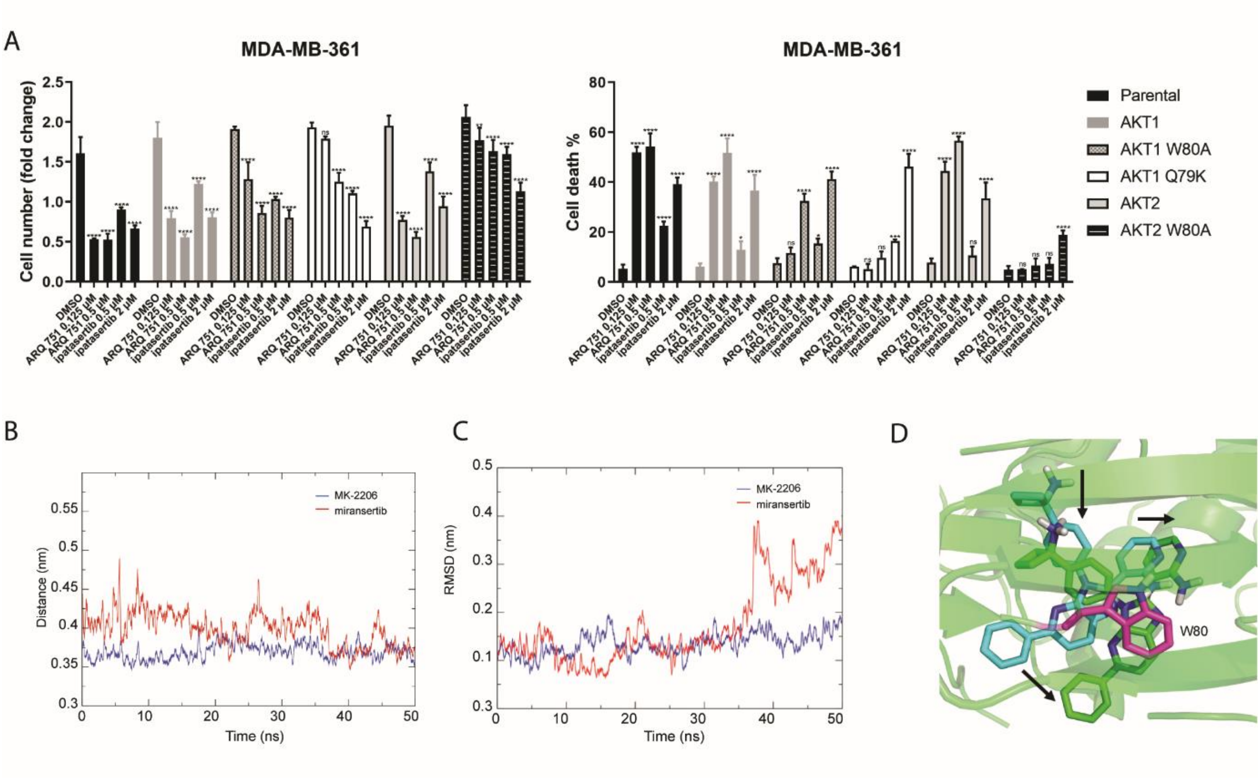

Sup. figure 2: **A) AKT mutations can confer resistance to allosteric inhibitors in a drug-specific and isoform-selective manner.** MBA-MB-361 ectopically expressing the indicated alleles of AKT1 or AKT2 were treated with two different doses of either ARQ 751 or ipatasertib. The fold change in cell numbers (left) and the fraction of dead cells (right) following 4 days of drug treatment is shown. **B) Distance between ligands and W80.** The distances (in nm) between the W80 indole centroid and MK-2206 (centroid of tricyclic core) or miransertib (centroid of imidazo(4,5-*b*)pyridine) throughout the MD simulation show that MK-2206 is generally in closer proximity to W80 than miransertib. Miransertib moves closer temporarily (e.g. after 22 ns or 40 ns), however, this conformation is not stable. **C-D) MK-2206 and miransertib binding modes. C)** The RMSDs of MK-2206 and miransertib throughout the simulation were calculated and reveal profound alterations of the miransertib binding mode after around 38 ns. **D)** The conformational changes (indicated by arrows) of miransertib after 50 ns (green) compared to the starting geometry (cyan) are required to fully stack the W80 indole (magenta) onto the imidazo(4,5-*b*)pyridine core.

Supplementary figure 3

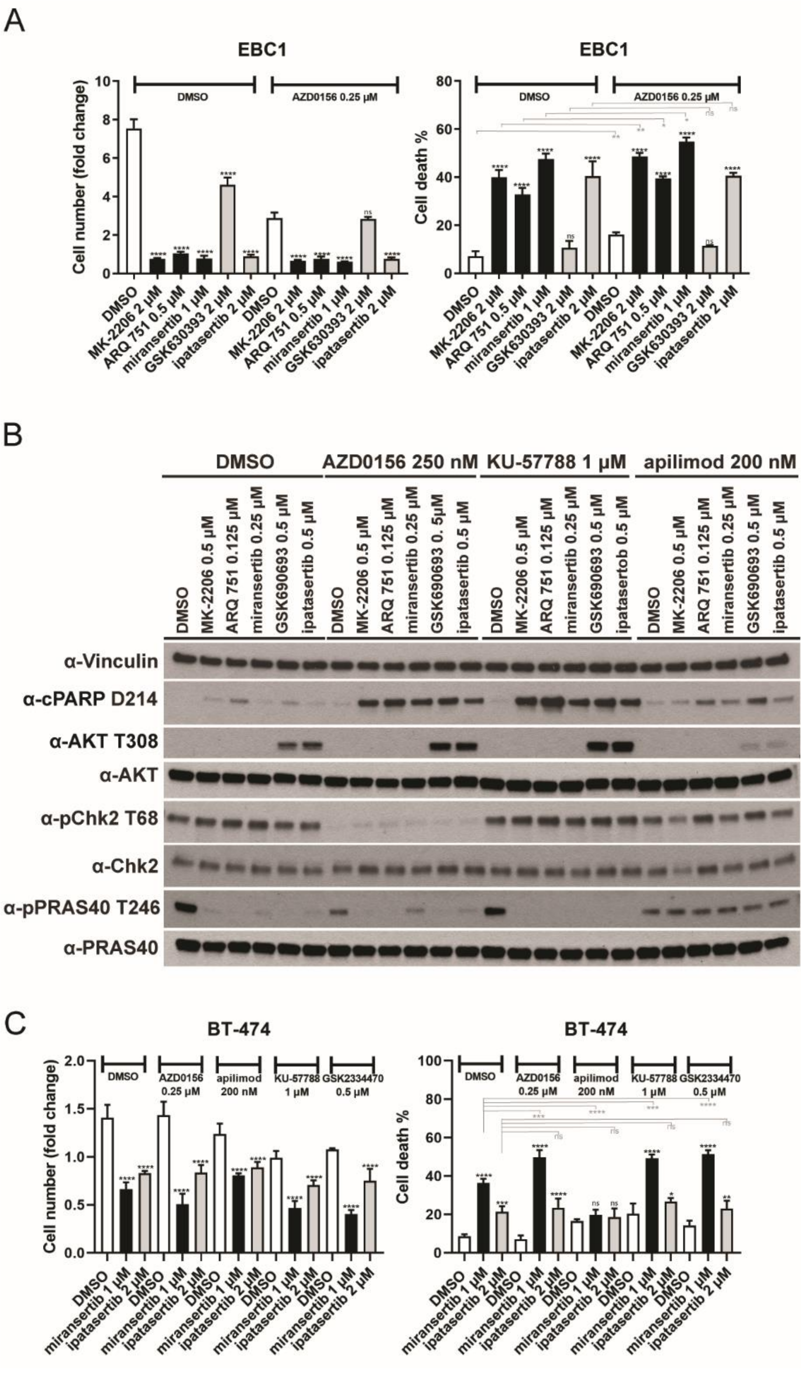

Sup. figure 3: **A) Effects of combining AKT and ATM inhibitors.** EBC1 cells were treated with various AKT inhibitors either alone or in combination with the ATM inhibitor AZD0156. The fold change in cell numbers (left) and the fraction of dead cells (right) following 4 days of drug treatment is shown. **B) Biochemical effects of AKT inhibitor treatment alone or in combination with ATM, DNA-PK, or PIKFYVE inhibitors.** MDA-MB-361 cells were treated with the indicated drugs. Cells were lysed following treatment and analysed by western blot with the indicated antibodies. **C) Effects of combining AKT inhibitors with inhibitors of ATM, PIKFYVE, DNA-PK, or PDK1.** BT-474 cells were treated with miransertib or ipatasertib either alone or in combination with the ATM inhibitor AZD0156, the PYKFYVE inhibitor apilimod, the DNA-PK inhibitor KU-57788, or the PDK1 inhibitor GSK2334470. The fold change in cell numbers (left) and the fraction of dead cells (right) following 4 days of drug treatment is shown.

Supplementary figure 4

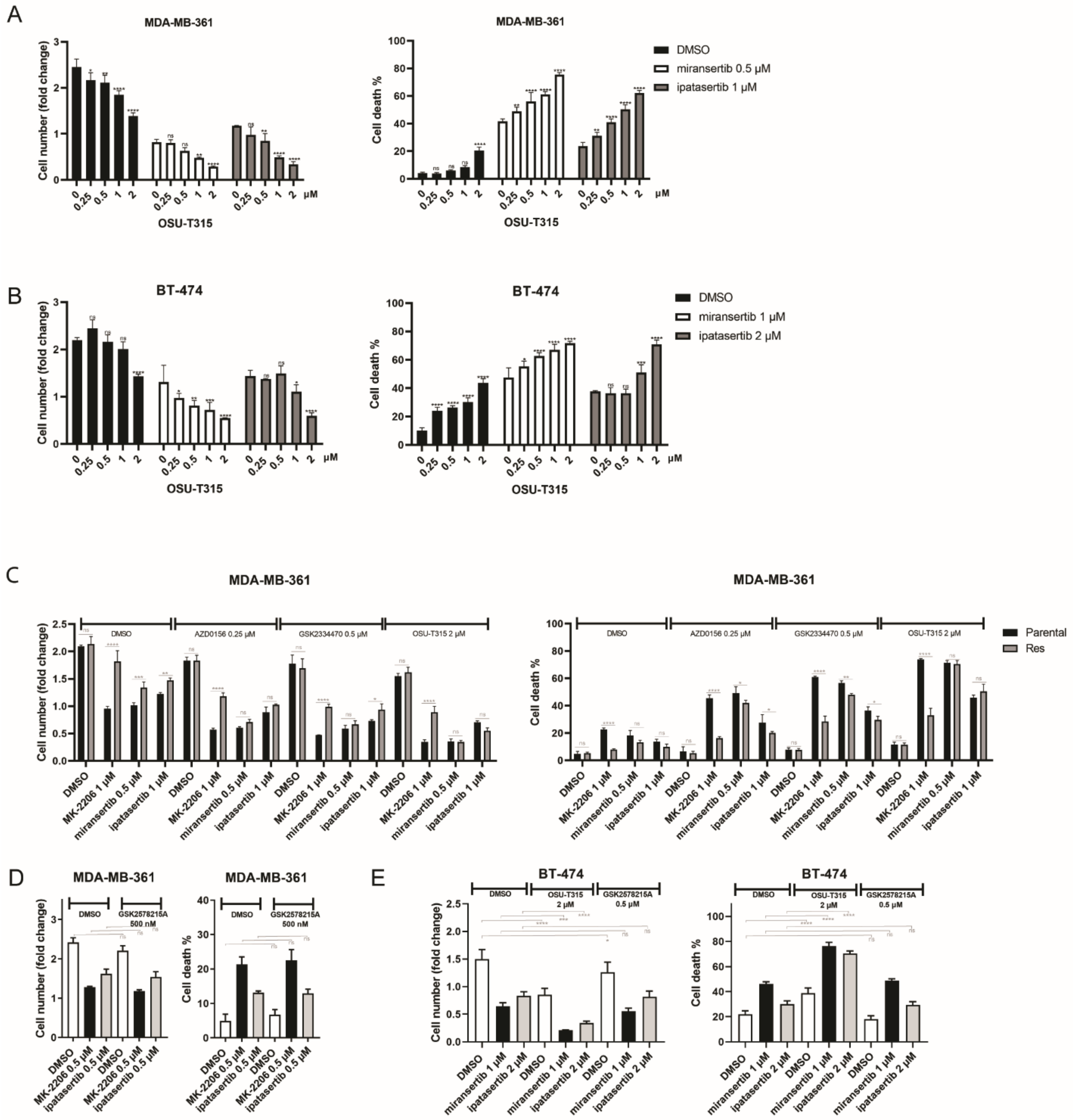

Sup. figure 4: **A, B) Effects of combining AKT and ILK inhibitors.** MDA-MB-361 (A) or BT-474 (B) cells were treated with miransertib or ipatasertib either alone or in combination with increasing doses of the ILK inhibitor OSU-T315. The fold change in cell numbers (left) and the fraction of dead cells (right) following 4 days of drug treatment is shown. **C) Effects of combining AKT with ATM, PDK and ILK inhibitors in parental and MK-22206-resistant cells.** Parental or MK-2206-resistant MDA-MB-361 cells were treated with MK-2206, miransertib or ipatasertib either alone or in combination with AZD0156, GSK2334470 or OSU-T315. The fold change in cell numbers (left) and the fraction of dead cells (right) following 4 days of drug treatment is shown. **D & E) Effects of combining AKT inhibitors with inhibitors of LRRK2 or ILK. D)** MDA-MB-361 cells were treated with MK-2206 or ipatasertib either alone or in combination with the LRRK2 inhibitor GSK2578215A. The fold change in cell numbers (left) and the fraction of dead cells (right) following 4 days of drug treatment is shown. **E)** BT-474 cells were treated with miransertib or ipatasertib either alone or incombnation with the ILK inhibitor OSU-T315 or the LRKK2 inhibitor GSK2578215A. The fold change in cell numbers (left) and the fraction of dead cells (right) following 4 days of drug treatment is shown.

Supplementary figure 5

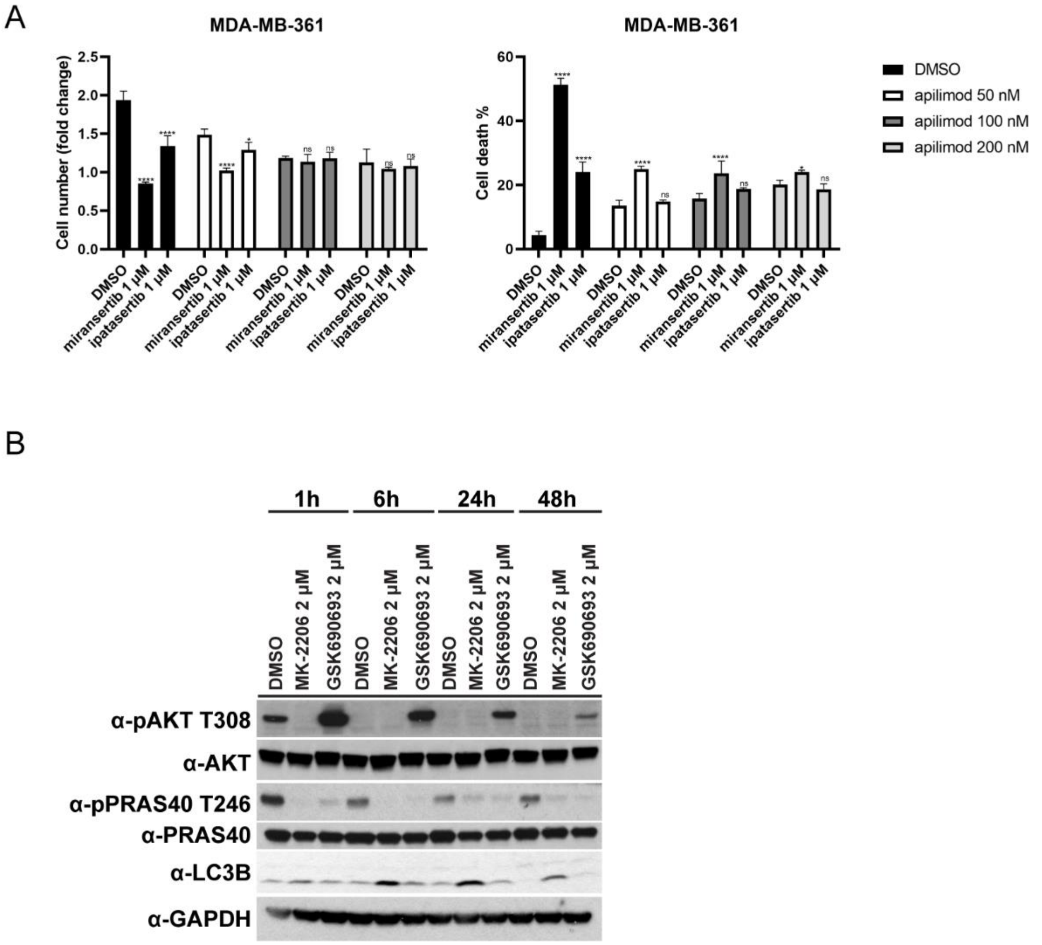

Sup. figure 5: **Effects of combining AKT and PIKFYVE inhibitors and evidence of autophagy activation following AKT inhibitor treatment. A)** MDA-MB-361 cells were treated with miransertib or ipatasertib either alone or in combination with increasing doses of the PIKFYVE inhibitor apilimod. The fold change in cell numbers (left) and the fraction of dead cells (right) following 4 days of drug treatment is shown. **B)** MDA-MB-361 cells were treated with the indicated doses of MK-2206 or GSK690693. Lysates were collected at the indicated time points and analysed by western blot. Note the increase in LC3B following treatment with MK-2206 but not GSK690693 despite equal suppression of PRAS40 phosphorylation.

Supplementary figure 6

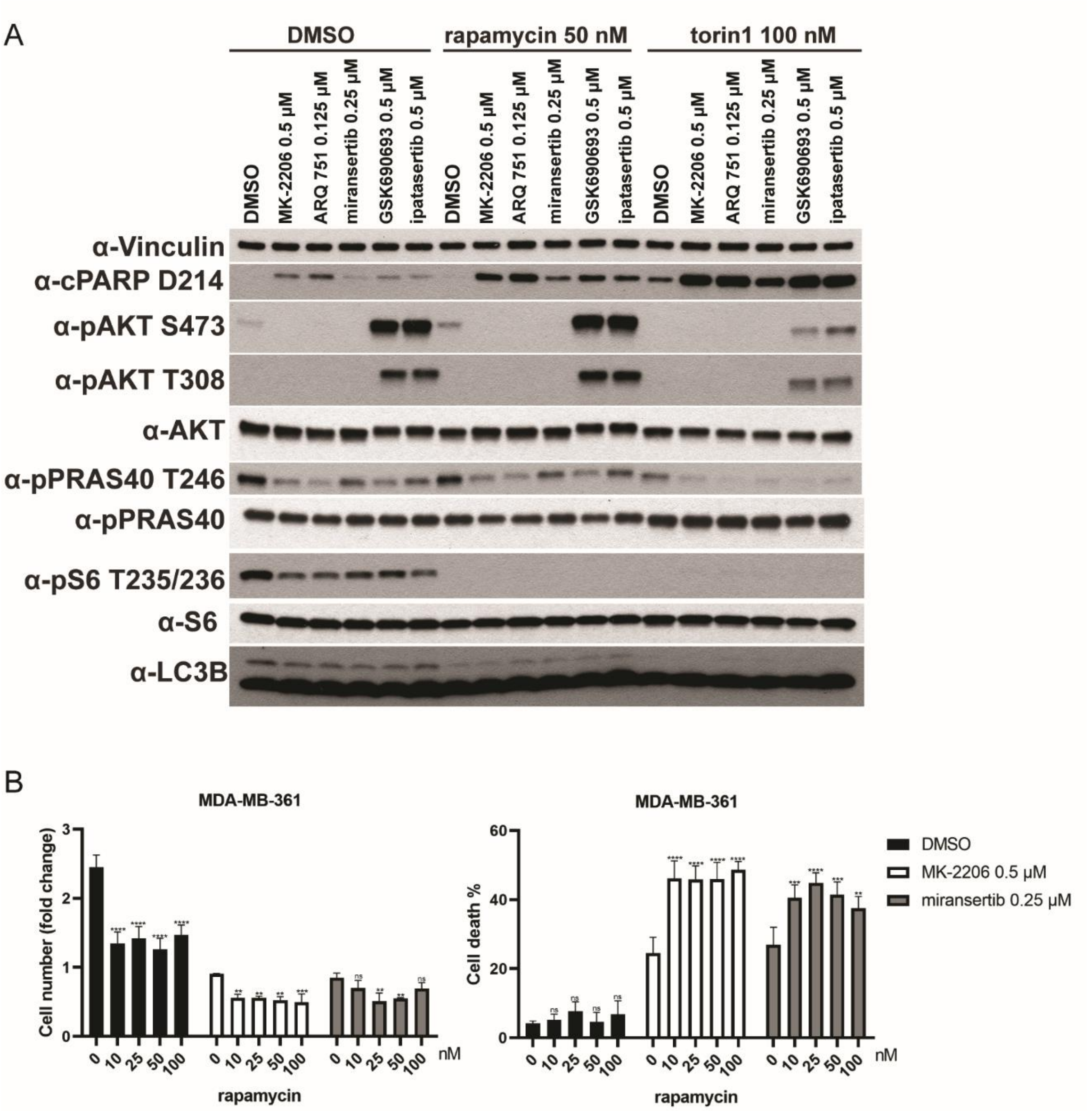

Sup. figure 6: Effects of combining AKT and mTOR inhibitors. A) MDA-MB-361 cells were treated with various AKT inhibitors either alone or in combination with either rapamycin or torin1. Cells were lysed following treatment and analysed by immunoblot with the indicated antibodies. **B)** MDA-MB-361 cells were treated with MK-2206 or miransertib either alone or in combination with increasing doses of rapamycin. The fold change in cell numbers (left) and the fraction of dead cells (right) following 4 days of drug treatment is shown.

Supplementary figure 7

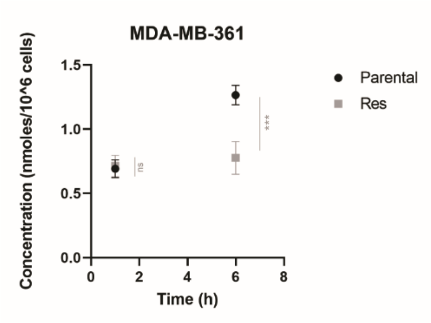

Sup. figure 7: MK-2206-resistant cells display lower intracellular drug concentrations compared to parental cells. MDA-MB-361 cells were treated with 2 µM MK-2206 for the indicated times. Following drug incubation, cells were lysed, and cell-associated MK-2206 levels were quantified via mass spectrometry.

Supplementary figure 8

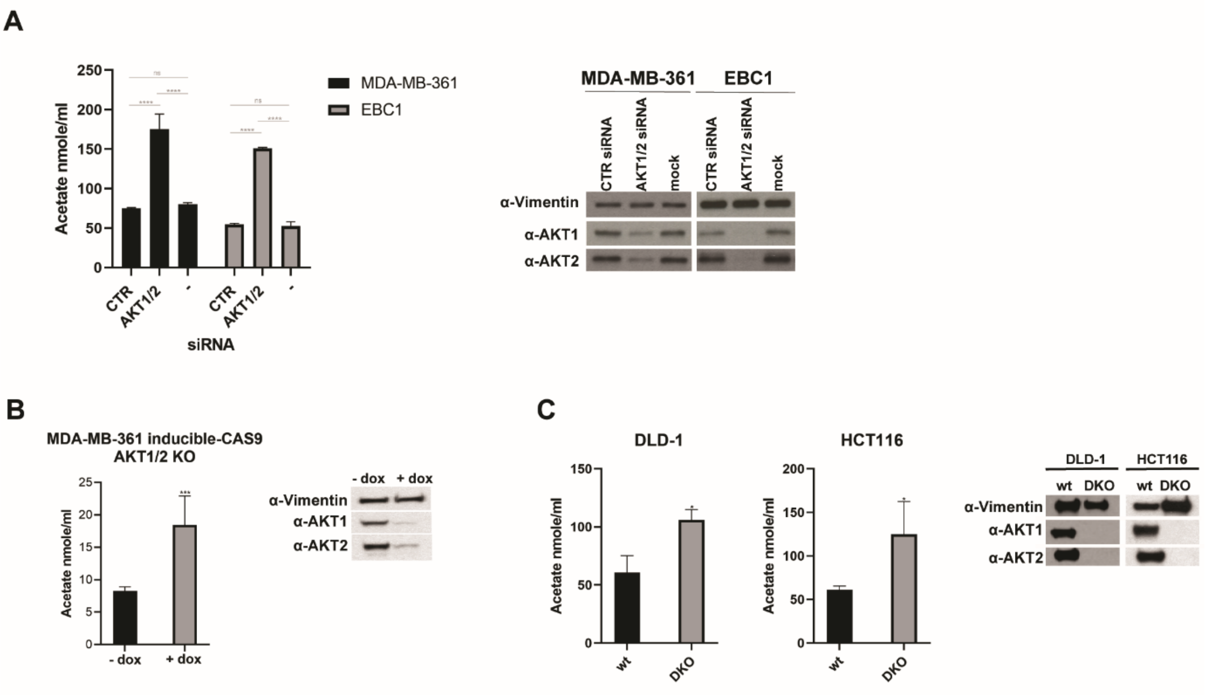

Sup. figure 8: AKT1 and AKT2 depletion causes acetate excretion in different cell lines. A) (left) Acetate secretion upon transfection of AKT1 and AKT2 siRNA, non-targeting (CTR) siRNA or no siRNA (mock) in MDA-MB-361 or EBC1 cells. (right) Western blot verifying the efficiency of the knock down 48 h after siRNA transfection. **B)** Acetate measurement after AKT1 and AKT2 depletion using CRISPR lentiviral vectors in tet-ON CAS9 MDA-MB-361. **C)** Acetate secretion and westerns from media or lysates, respectively, of wild type (wt) or AKT1/2 DKO DLD-1 and HCT116 cells.

Supplementary references for PDB entries (Figure 3D):

3D0E: Reference #24.

4EKL: K. Lin et al., An ATP-site on-off switch that restricts phosphatase accessibility of Akt. Sci. Signal. 5, ra37-ra37 (2012), **DOI:** 10.1126/scisignal.2002618

4GV1: M. Addie et al., Discovery of 4-Amino-N-[(1S)-1-(4-chlorophenyl)-3-hydroxypropyl]-1-(7H-pyrrolo[2,3-d]pyrimidin-4-yl)piperidine-4-carboxamide (AZD5363), an orally bioavailable, potent inhibitor of Akt kinases. J. Med. Chem. 56, 2059-2073 (2013), **DOI:** 10.1021/jm301762v

Methods and references for Supplementary figure 2B, C and D:

The input structures were prepared in MOE 2019.0102[1]. MK-2206 and miransertib were parameterized with Ambertools 19.0[2] and the resulting prmtop and inpcrd files were converted to Gromacs input files using acpype[3]. The protein, solvent and ions were parameterized using the Amber99SB[4] force field in GROMACS version 2019.2[5-12]. The protein was placed in a cubic box at least 1 nm apart from the edge. The box was then solvated with TIP3P[13] waters and neutralized with chloride ions. After minimization, the system was equilibrated with 100 ps runs at NVT and NPT conditions, respectively. This was followed by a 50 ns production run employing 2 fs time steps. Temperature and pressure were monitored with a modified Berendsen thermostat[14] and the Parrinello-Rahman[15] barostat and maintained at 300 K and 1 bar. The Particle-Mesh Ewald[16] and LINCS[17] and algorithms were applied to define long range electrostatics and bond parameters. A radius cut-off of 12 Å was applied to short range van der Waals and electrostatic terms. The inbuilt GROMAS tools were used for analysis of RMSDs and distances and figures were created using xmgrace[18] and PyMOL[19].

1) Molecular Operating Environment (MOE), 2019.0102; Chemical Computing Group ULC, 1010 Sherbooke St. West, Suite #910, Montreal, QC, Canada, H3A 2R7, 2019.
2) D.A. Case, I.Y. Ben-Shalom, S.R. Brozell, D.S. Cerutti, T.E. Cheatham, III, V.W.D. Cruzeiro, T.A. Darden,R.E. Duke, D. Ghoreishi, G. Giambasu, T. Giese, M.K. Gilson, H. Gohlke, A.W. Goetz, D. Greene,R Harris,N. Homeyer, Y. Huang, S. Izadi, A. Kovalenko, R. Krasny, T. Kurtzman, T.S. Lee, S. LeGrand, P. Li, C. Lin,J. Liu, T. Luchko, R. Luo, V. Man, D.J. Mermelstein, K.M. Merz, Y. Miao, G. Monard, C. Nguyen, H.Nguyen, A. Onufriev, F. Pan, R. Qi, D.R. Roe, A. Roitberg, C. Sagui, S. Schott-Verdugo, J. Shen, C.L.Simmerling, J. Smith, J. Swails, R.C. Walker, J. Wang, H. Wei, L. Wilson, R.M. Wolf, X. Wu, L. Xiao, Y.Xiong, D.M. York and P.A. Kollman (2019), AMBER 2019, University of California, San Francisco.
3) Sousa da Silva, A.W., Vranken, W.F. ACPYPE - AnteChamber PYthon Parser interfacE. *BMC Res Notes* **5,** 367 (2012) doi:10.1186/1756-0500-5-367
4) Hornak V, Abel R, Okur A, Strockbine B, Roitberg A, Simmerling C. Comparison of multiple Amber force fields and development of improved protein backbone parameters. *Proteins*. 2006;65(3):712–725. doi:10.1002/prot.21123
5) https://doi.org/10.5281/zenodo.2636382
6) M. J. Abraham, T. Murtola, R. Schulz, S. Páll, J. C. Smith, B. Hess, E.Lindahl. GROMACS: High performance molecular simulations through multi-level parallelism from laptops to supercomputers. SoftwareX 1 (2015) pp. 19-25
7) S. Páll, M. J. Abraham, C. Kutzner, B. Hess, E. Lindahl. Tackling Exascale Software Challenges in Molecular Dynamics Simulations with GROMACS. In S. Markidis & E. Laure (Eds.), Solving Software Challenges for Exascale 8759 (2015) pp. 3-27
8) S. Pronk, S. Páll, R. Schulz, P. Larsson, P. Bjelkmar, R. Apostolov, M. R. Shirts, J. C. Smith, P. M. Kasson, D. van der Spoel, B. Hess, and E. Lindahl. GROMACS 4.5: a high-throughput and highly parallel open source molecular simulation toolkit. Bioinformatics 29 (2013) pp. 845-54.
9) B. Hess and C. Kutzner and D. van der Spoel and E. Lindahl. GROMACS 4: Algorithms for highly efficient, load-balanced, and scalable molecular simulation. J. Chem. Theory Comput. 4 (2008) pp. 435-447.
10) D. van der Spoel, E. Lindahl, B. Hess, G. Groenhof, A. E. Mark and H. J. C. Berendsen. GROMACS: Fast, Flexible and Free. J. Comp. Chem. 26 (2005) pp. 1701-1719.
11) E. Lindahl and B. Hess and D. van der Spoel. GROMACS 3.0: A package for molecular simulation and trajectory analysis. J. Mol. Mod. 7 (2001) pp. 306-317.
12) H. J. C. Berendsen, D. van der Spoel and R. van Drunen. GROMACS: A message-passing parallel molecular dynamics implementation. Comp. Phys. Comm. 91 (1995) pp. 43-56.

13) Jorgensen, W. L., Chandrasekhar, J., Madura, J. D., Impey, R. W., and Klein, M. L. (1983) Comparison of simple potential functions for simulating liquid water, *The Journal of Chemical Physics 79*, 926-935.
14) Berendsen, H. J. C., Postma, J. P. M., van Gunsteren, W. F., DiNola, A., and Haak, J. R. (1984) Molecular dynamics with coupling to an external bath, *The Journal of Chemical Physics 81*, 3684-3690.
15) Parrinello, M., and Rahman, A. (1981) Polymorphic transitions in single crystals: A new molecular dynamics method, *Journal of Applied Physics 52*, 7182-7190
16) Darden, T., York, D., and Pedersen, L. (1993) Particle mesh Ewald: An N·log(N) method for Ewald sums in large systems, *The Journal of Chemical Physics 98*, 10089-10092
17) Hess, B., Bekker, H., Berendsen, H. J. C., and Fraaije, J. G. E. M. (1998) LINCS: A linear constraint solver for molecular simulations, *Journal of Computational Chemistry 18*, 1463-1472.
18) Grace version 5.1.25, Grace Team (2015-02-15), Grace User's Guide, retreived 2018-10-29.
19) PyMOL: The PyMOL Molecular Graphics System, Version 1.8.0.0 Schrödinger, LLC.

Supplementary table 1

Sidak's multiple comparisons test	Mean Diff.	95.00% CI of diff.	Significant?	Summary	Adjusted P Value		Mean 1	Mean 2	Mean Diff.	SE of diff.	n1	n2	t	DF
**Figure 1 A**														
MDA-MB-361														
**Cell number**														
DMSO vs. MK-2206 2 µM	1.652	1.456 to 1.848	Yes	****	<0.0001	B	2.398	0.7463	1.652	0.06445	3	3	25.63	12
DMSO vs. ARQ 751 0.5 µM	1.722	1.526 to 1.918	Yes	****	<0.0001	C	2.398	0.6757	1.722	0.06445	3	3	26.72	12
DMSO vs. miransertib 1 µM	1.874	1.678 to 2.070	Yes	****	<0.0001	D	2.398	0.5237	1.874	0.06445	3	3	29.08	12
DMSO vs. GSK690693 2 µM	1.314	1.118 to 1.510	Yes	****	<0.0001	E	2.398	1.084	1.314	0.06445	3	3	20.39	12
DMSO vs. ipatasertib 2 µM	1.35	1.154 to 1.546	Yes	****	<0.0001	F	2.398	1.048	1.35	0.06445	3	3	20.95	12
**Cell death**														
DMSO vs. MK-2206 2 µM	-44	-50.18 to -37.81	Yes	****	<0.0001	A-B	4.197	48.2	-44	1.701	3	3	25.87	12
DMSO vs. ARQ 751 0.5 µM	-46.03	-52.22 to -39.85	Yes	****	<0.0001	A-C	4.197	50.23	-46.03	1.701	3	3	27.07	12
DMSO vs. miransertib 1 µM	-56.83	-63.02 to -50.65	Yes	****	<0.0001	A-D	4.197	61.03	-56.83	1.701	3	3	33.42	12
DMSO vs. GSK690693 2 µM	-21.36	-27.54 to -15.18	Yes	****	<0.0001	A-E	4.197	25.56	-21.36	1.701	3	3	12.56	12
DMSO vs. ipatasertib 2 µM	-26.62	-32.81 to -20.44	Yes	****	<0.0001	A-F	4.197	30.82	-26.62	1.701	3	3	15.65	12
MK-2206 2 µM vs. ARQ 751 0.5 µM	-2.035	-8.219 to 4.149	No	ns	0.9878	B-C	48.2	50.23	-2.035	1.701	3	3	1.197	12
MK-2206 2 µM vs. miransertib 1 µM	-12.83	-19.02 to -6.650	Yes	***	0.0001	B-D	48.2	61.03	-12.83	1.701	3	3	7.546	12
MK-2206 2 µM vs. GSK690693 2 µM	22.64	16.45 to 28.82	Yes	****	<0.0001	B-E	48.2	25.56	22.64	1.701	3	3	13.31	12
MK-2206 2 µM vs. ipatasertib 2 µM	17.37	11.19 to 23.56	Yes	****	<0.0001	B-F	48.2	30.82	17.37	1.701	3	3	10.22	12
ARQ 751 0.5 µM vs. miransertib 1 µM	-10.8	-16.98 to -4.615	Yes	***	0.0005	C-D	50.23	61.03	-10.8	1.701	3	3	6.35	12
ARQ 751 0.5 µM vs. GSK690693 2 µM	24.67	18.49 to 30.86	Yes	****	<0.0001	C-E	50.23	25.56	24.67	1.701	3	3	14.51	12
ARQ 751 0.5 µM vs. ipatasertib 2 µM	19.41	13.23 to 25.59	Yes	****	<0.0001	C-F	50.23	30.82	19.41	1.701	3	3	11.41	12
miransertib 1 µM vs. GSK690693 2 µM	35.47	29.29 to 41.66	Yes	****	<0.0001	D-E	61.03	25.56	35.47	1.701	3	3	20.86	12
miransertib 1 µM vs. ipatasertib 2 µM	30.21	24.02 to 36.39	Yes	****	<0.0001	D-F	61.03	30.82	30.21	1.701	3	3	17.76	12
GSK690693 2 µM vs. ipatasertib 2 µM	-5.263	-11.45 to 0.9202	No	ns	0.1305	E-F	25.56	30.82	-5.263	1.701	3	3	3.095	12
T 47D														
**Cell number**														
DMSO vs. MK-2206 2 µM	1.574	1.343 to 1.805	Yes	****	<0.0001	B	2.941	1.367	1.574	0.07589	3	3	20.74	12
DMSO vs. ARQ 751 0.5 µM	1.649	1.419 to 1.880	Yes	****	<0.0001	C	2.941	1.291	1.649	0.07589	3	3	21.74	12
DMSO vs. miransertib 1 µM	1.768	1.537 to 1.999	Yes	****	<0.0001	D	2.941	1.173	1.768	0.07589	3	3	23.29	12
DMSO vs. GSK690693 2 µM	1.399	1.168 to 1.630	Yes	****	<0.0001	E	2.941	1.542	1.399	0.07589	3	3	18.43	12
DMSO vs. ipatasertib 4 µM	1.617	1.386 to 1.848	Yes	****	<0.0001	F	2.941	1.324	1.617	0.07589	3	3	21.31	12
**Cell death**														
DMSO vs. MK-2206 2 µM	-7.333	-14.92 to 0.2580	No	ns	0.0624	A-B	6.6	13.93	-7.333	2.088	3	3	3.512	12
DMSO vs. ARQ 751 0.5 µM	-6.867	-14.46 to 0.7246	No	ns	0.0928	A-C	6.6	13.47	-6.867	2.088	3	3	3.289	12
DMSO vs. miransertib 1 µM	-6.367	-13.96 to 1.225	No	ns	0.1412	A-D	6.6	12.97	-6.367	2.088	3	3	3.049	12
DMSO vs. GSK690693 2 µM	-0.8333	-8.425 to 6.758	No	ns	>0.9999	A-E	6.6	7.433	-0.8333	2.088	3	3	0.399	12
DMSO vs. ipatasertib 4 µM	-4.4	-11.99 to 3.191	No	ns	0.584	A-F	6.6	11	-4.4	2.088	3	3	2.107	12
MK-2206 2 µM vs. ARQ 751 0.5 µM	0.4667	-7.125 to 8.058	No	ns	>0.9999	B-C	13.93	13.47	0.4667	2.088	3	3	0.224	12
MK-2206 2 µM vs. miransertib 1 µM	0.9667	-6.625 to 8.558	No	ns	>0.9999	B-D	13.93	12.97	0.9667	2.088	3	3	0.463	12
MK-2206 2 µM vs. GSK690693 2 µM	6.5	-1.091 to 14.09	No	ns	0.1264	B-E	13.93	7.433	6.5	2.088	3	3	3.113	12
MK-2206 2 µM vs. ipatasertib 4 µM	2.933	-4.658 to 10.52	No	ns	0.9538	B-F	13.93	11	2.933	2.088	3	3	1.405	12
ARQ 751 0.5 µM vs. miransertib 1 µM	0.5	-7.091 to 8.091	No	ns	>0.9999	C-D	13.47	12.97	0.5	2.088	3	3	0.24	12
ARQ 751 0.5 µM vs. GSK690693 2 µM	6.033	-1.558 to 13.62	No	ns	0.1855	C-E	13.47	7.433	6.033	2.088	3	3	2.89	12
ARQ 751 0.5 µM vs. ipatasertib 4 µM	2.467	-5.125 to 10.06	No	ns	0.9891	C-F	13.47	11	2.467	2.088	3	3	1.181	12
miransertib 1 µM vs. GSK690693 2 µM	5.533	-2.058 to 13.12	No	ns	0.2746	D-E	12.97	7.433	5.533	2.088	3	3	2.65	12
miransertib 1 µM vs. ipatasertib 4 µM	1.967	-5.625 to 9.558	No	ns	0.9989	D-F	12.97	11	1.967	2.088	3	3	0.942	12
GSK690693 2 µM vs. ipatasertib 4 µM	-3.567	-11.16 to 4.025	No	ns	0.8353	E-F	7.433	11	-3.567	2.088	3	3	1.708	12
ZR75.1														
**Cell number**														
DMSO vs. MK-2206 2 µM	1.337	0.9976 to 1.677	Yes	****	<0.0001	B	2.423	1.086	1.337	0.1116	3	3	11.98	12
DMSO vs. ARQ 751 0.5 µM	1.47	1.130 to 1.809	Yes	****	<0.0001	C	2.423	0.9537	1.47	0.1116	3	3	13.17	12
DMSO vs. miransertib 1 µM	1.286	0.9462 to 1.626	Yes	****	<0.0001	D	2.423	1.137	1.286	0.1116	3	3	11.52	12
DMSO vs. GSK690693 2 µM	1.205	0.8650 to 1.545	Yes	****	<0.0001	E	2.423	1.219	1.205	0.1116	3	3	10.79	12
DMSO vs. ipatasertib 4 µM	1.423	1.083 to 1.762	Yes	****	<0.0001	F	2.423	1.001	1.423	0.1116	3	3	12.74	12
**Cell death**														
DMSO vs. MK-2206 2 µM	-14.53	-26.72 to -2.344	Yes	*	0.0145	A-B	5	19.53	-14.53	3.353	3	3	4.335	12
DMSO vs. ARQ 751 0.5 µM	-17.17	-29.36 to -4.977	Yes	**	0.0038	A-C	5	22.17	-17.17	3.353	3	3	5.12	12
DMSO vs. miransertib 1 µM	-15.6	-27.79 to -3.410	Yes	**	0.0083	A-D	5	20.6	-15.6	3.353	3	3	4.653	12
DMSO vs. GSK690693 2 µM	-6.433	-18.62 to 5.756	No	ns	0.7094	A-E	5	11.43	-6.433	3.353	3	3	1.919	12
DMSO vs. ipatasertib 4 µM	-12.9	-25.09 to -0.7103	Yes	*	0.0342	A-F	5	17.9	-12.9	3.353	3	3	3.848	12
MK-2206 2 µM vs. ARQ 751 0.5 µM	-2.633	-14.82 to 9.556	No	ns	0.9999	B-C	19.53	22.17	-2.633	3.353	3	3	0.786	12
MK-2206 2 µM vs. miransertib 1 µM	-1.067	-13.26 to 11.12	No	ns	>0.9999	B-D	19.53	20.6	-1.067	3.353	3	3	0.318	12
MK-2206 2 µM vs. GSK690693 2 µM	8.1	-4.090 to 20.29	No	ns	0.3913	B-E	19.53	11.43	8.1	3.353	3	3	2.416	12
MK-2206 2 µM vs. ipatasertib 4 µM	1.633	-10.56 to 13.82	No	ns	>0.9999	B-F	19.53	17.9	1.633	3.353	3	3	0.487	12
ARQ 751 0.5 µM vs. miransertib 1 µM	1.567	-10.62 to 13.76	No	ns	>0.9999	C-D	22.17	20.6	1.567	3.353	3	3	0.467	12
ARQ 751 0.5 µM vs. GSK690693 2 µM	10.73	-1.456 to 22.92	No	ns	0.1083	C-E	22.17	11.43	10.73	3.353	3	3	3.201	12
ARQ 751 0.5 µM vs. ipatasertib 4 µM	4.267	-7.923 to 16.46	No	ns	0.9791	C-F	22.17	17.9	4.267	3.353	3	3	1.273	12
miransertib 1 µM vs. GSK690693 2 µM	9.167	-3.023 to 21.36	No	ns	0.24	D-E	20.6	11.43	9.167	3.353	3	3	2.734	12
miransertib 1 µM vs. ipatasertib 4 µM	2.7	-9.490 to 14.89	No	ns	0.9998	D-F	20.6	17.9	2.7	3.353	3	3	0.805	12
GSK690693 2 µM vs. ipatasertib 4 µM	-6.467	-18.66 to 5.723	No	ns	0.703	E-F	11.43	17.9	-6.467	3.353	3	3	1.929	12
Hs 746T														
**Cell number**														
DMSO vs. MK-2206 2 µM	0.6134	-0.8756 to 2.102	No	ns	0.727	B	4.617	4.004	0.6134	0.4812	2	3	1.275	11
DMSO vs. ARQ 751 0.5 µM	0.8932	-0.5958 to 2.382	No	ns	0.3773	C	4.617	3.724	0.8932	0.4812	2	3	1.856	11
DMSO vs. miransertib 1 µM	1.001	-0.4882 to 2.490	No	ns	0.2728	D	4.617	3.616	1.001	0.4812	2	3	2.08	11
DMSO vs. GSK690693 2 µM	1.324	-0.1654 to 2.813	No	ns	0.0908	E	4.617	3.293	1.324	0.4812	2	3	2.751	11
DMSO vs. ipatasertib 4 µM	-0.1614	-1.650 to 1.328	No	ns	0.9989	F	4.617	4.778	-0.1614	0.4812	2	3	0.335	11
**Cell death**														
DMSO vs. MK-2206 2 µM	-9.433	-28.95 to 10.08	No	ns	0.8082	A-B	17.73	27.17	-9.433	5.367	3	3	1.758	12
DMSO vs. ARQ 751 0.5 µM	-18.93	-38.45 to 0.5791	No	ns	0.0606	A-C	17.73	36.67	-18.93	5.367	3	3	3.528	12
DMSO vs. miransertib 1 µM	-22.33	-41.85 to -2.821	Yes	*	0.0196	A-D	17.73	40.07	-22.33	5.367	3	3	4.162	12
DMSO vs. GSK690693 2 µM	-0.4333	-19.95 to 19.08	No	ns	>0.9999	A-E	17.73	18.17	-0.4333	5.367	3	3	0.081	12
DMSO vs. ipatasertib 4 µM	1.433	-18.08 to 20.95	No	ns	>0.9999	A-F	17.73	16.3	1.433	5.367	3	3	0.267	12
MK-2206 2 µM vs. ARQ 751 0.5 µM	-9.5	-29.01 to 10.01	No	ns	0.8011	B-C	27.17	36.67	-9.5	5.367	3	3	1.77	12
MK-2206 2 µM vs. miransertib 1 µM	-12.9	-32.41 to 6.612	No	ns	0.3982	B-D	27.17	40.07	-12.9	5.367	3	3	2.404	12
MK-2206 2 µM vs. GSK690693 2 µM	9	-10.51 to 28.51	No	ns	0.8514	B-E	27.17	18.17	9	5.367	3	3	1.677	12

MK-2206 2 µM vs. ipatasertib 4 µM	10.87	-8.646 to 30.38	No	ns	0.6393	B-F	27.17	16.3	10.87	5.367	3	3	2.025	12
ARQ 751 0.5 µM vs. miransertib 1 µM	-3.4	-22.91 to 16.11	No	ns	>0.9999	C-D	36.67	40.07	-3.4	5.367	3	3	0.634	12
ARQ 751 0.5 µM vs. GSK690693 2 µM	18.5	-1.012 to 38.01	No	ns	0.07	C-E	36.67	18.17	18.5	5.367	3	3	3.447	12
ARQ 751 0.5 µM vs. ipatasertib 4 µM	20.37	0.8542 to 39.88	Yes	*	0.0376	C-F	36.67	16.3	20.37	5.367	3	3	3.795	12
miransertib 1 µM vs. GSK690693 2 µM	21.9	2.388 to 41.41	Yes	*	0.0226	D-E	40.07	18.17	21.9	5.367	3	3	4.081	12
miransertib 1 µM vs. ipatasertib 4 µM	23.77	4.254 to 43.28	Yes	*	0.0123	D-F	40.07	16.3	23.77	5.367	3	3	4.429	12
GSK690693 2 µM vs. ipatasertib 4 µM	1.867	-17.65 to 21.38	No	ns	>0.9999	E-F	18.17	16.3	1.867	5.367	3	3	0.348	12
MCF7														
**Cell number**														
DMSO vs. MK-2206 2 µM	3.211	2.978 to 3.444	Yes	****	<0.0001	B	4.057	0.846	3.211	0.0766	3	3	41.92	12
DMSO vs. ARQ 751 0.5 µM	3.356	3.123 to 3.589	Yes	****	<0.0001	C	4.057	0.7013	3.356	0.0766	3	3	43.81	12
DMSO vs. miransertib 1 µM	3.336	3.103 to 3.570	Yes	****	<0.0001	D	4.057	0.7205	3.336	0.0766	3	3	43.56	12
DMSO vs. GSK690693 2 µM	2.918	2.685 to 3.151	Yes	****	<0.0001	E	4.057	1.139	2.918	0.0766	3	3	38.1	12
DMSO vs. ipatasertib 2 µM	3.159	2.926 to 3.392	Yes	****	<0.0001	F	4.057	0.8983	3.159	0.0766	3	3	41.24	12
**Cell death**														
DMSO vs. MK-2206 2 µM	-30.07	-37.18 to -22.95	Yes	****	<0.0001	A-B	8.867	38.93	-30.07	1.958	3	3	15.36	12
DMSO vs. ARQ 751 0.5 µM	-32.13	-39.25 to -25.02	Yes	****	<0.0001	A-C	8.867	41	-32.13	1.958	3	3	16.41	12
DMSO vs. miransertib 1 µM	-34.4	-41.52 to -27.28	Yes	****	<0.0001	A-D	8.867	43.27	-34.4	1.958	3	3	17.57	12
DMSO vs. GSK690693 2 µM	-17.03	-24.15 to -9.916	Yes	****	<0.0001	A-E	8.867	25.9	-17.03	1.958	3	3	8.701	12
DMSO vs. ipatasertib 2 µM	-24.33	-31.45 to -17.22	Yes	****	<0.0001	A-F	8.867	33.2	-24.33	1.958	3	3	12.43	12
MK-2206 2 µM vs. ARQ 751 0.5 µM	-2.067	-9.184 to 5.051	No	ns	0.9963	B-C	38.93	41	-2.067	1.958	3	3	1.056	12
MK-2206 2 µM vs. miransertib 1 µM	-4.333	-11.45 to 2.784	No	ns	0.5141	B-D	38.93	43.27	-4.333	1.958	3	3	2.214	12
MK-2206 2 µM vs. GSK690693 2 µM	13.03	5.916 to 20.15	Yes	***	0.0004	B-E	38.93	25.9	13.03	1.958	3	3	6.658	12
MK-2206 2 µM vs. ipatasertib 2 µM	5.733	-1.384 to 12.85	No	ns	0.1736	B-F	38.93	33.2	5.733	1.958	3	3	2.929	12
ARQ 751 0.5 µM vs. miransertib 1 µM	-2.267	-9.384 to 4.851	No	ns	0.991	C-D	41	43.27	-2.267	1.958	3	3	1.158	12
ARQ 751 0.5 µM vs. GSK690693 2 µM	15.1	7.982 to 22.22	Yes	****	<0.0001	C-E	41	25.9	15.1	1.958	3	3	7.714	12
ARQ 751 0.5 µM vs. ipatasertib 2 µM	7.8	0.6824 to 14.92	Yes	*	0.0268	C-F	41	33.2	7.8	1.958	3	3	3.984	12
miransertib 1 µM vs. GSK690693 2 µM	17.37	10.25 to 24.48	Yes	****	<0.0001	D-E	43.27	25.9	17.37	1.958	3	3	8.871	12
miransertib 1 µM vs. ipatasertib 2 µM	10.07	2.949 to 17.18	Yes	**	0.0037	D-F	43.27	33.2	10.07	1.958	3	3	5.142	12
GSK690693 2 µM vs. ipatasertib 2 µM	-7.3	-14.42 to -0.1824	Yes	*	0.0423	E-F	25.9	33.2	-7.3	1.958	3	3	3.729	12
EBC1														
**Cell number**														
DMSO vs. MK-2206 2 µM	6.775	6.133 to 7.417	Yes	****	<0.0001	B	7.55	0.7748	6.775	0.211	3	3	32.1	12
DMSO vs. ARQ 751 0.5 µM	6.502	5.860 to 7.144	Yes	****	<0.0001	C	7.55	1.048	6.502	0.211	3	3	30.81	12
DMSO vs. miransertib 1 µM	6.76	6.117 to 7.402	Yes	****	<0.0001	D	7.55	0.7901	6.76	0.211	3	3	32.03	12
DMSO vs. GSK690693 2 µM	2.932	2.290 to 3.575	Yes	****	<0.0001	E	7.55	4.617	2.932	0.211	3	3	13.89	12
DMSO vs. ipatasertib 2 µM	6.642	6.000 to 7.284	Yes	****	<0.0001	F	7.55	0.9078	6.642	0.211	3	3	31.47	12
**Cell death**														
DMSO vs. MK-2206 2 µM	-32.77	-43.03 to -22.50	Yes	****	<0.0001	A-B	7.233	40	-32.77	2.823	3	3	11.61	12
DMSO vs. ARQ 751 0.5 µM	-25.57	-35.83 to -15.30	Yes	****	<0.0001	A-C	7.233	32.8	-25.57	2.823	3	3	9.057	12
DMSO vs. miransertib 1 µM	-40.23	-50.50 to -29.97	Yes	****	<0.0001	A-D	7.233	47.47	-40.23	2.823	3	3	14.25	12
DMSO vs. GSK690693 2 µM	-3.467	-13.73 to 6.797	No	ns	0.9846	A-E	7.233	10.7	-3.467	2.823	3	3	1.228	12
DMSO vs. ipatasertib 2 µM	-33.27	-43.53 to -23.00	Yes	****	<0.0001	A-F	7.233	40.5	-33.27	2.823	3	3	11.78	12
MK-2206 2 µM vs. ARQ 751 0.5 µM	7.2	-3.064 to 17.46	No	ns	0.3206	B-C	40	32.8	7.2	2.823	3	3	2.551	12
MK-2206 2 µM vs. miransertib 1 µM	-7.467	-17.73 to 2.797	No	ns	0.2768	B-D	40	47.47	-7.467	2.823	3	3	2.645	12
MK-2206 2 µM vs. GSK690693 2 µM	29.3	19.04 to 39.56	Yes	****	<0.0001	B-E	40	10.7	29.3	2.823	3	3	10.38	12
MK-2206 2 µM vs. ipatasertib 2 µM	-0.5	-10.76 to 9.764	No	ns	>0.9999	B-F	40	40.5	-0.5	2.823	3	3	0.177	12
ARQ 751 0.5 µM vs. miransertib 1 µM	-14.67	-24.93 to -4.403	Yes	**	0.0033	C-D	32.8	47.47	-14.67	2.823	3	3	5.196	12
ARQ 751 0.5 µM vs. GSK690693 2 µM	22.1	11.84 to 32.36	Yes	****	<0.0001	C-E	32.8	10.7	22.1	2.823	3	3	7.829	12
ARQ 751 0.5 µM vs. ipatasertib 2 µM	-7.7	-17.96 to 2.564	No	ns	0.2425	C-F	32.8	40.5	-7.7	2.823	3	3	2.728	12
miransertib 1 µM vs. GSK690693 2 µM	36.77	26.50 to 47.03	Yes	****	<0.0001	D-E	47.47	10.7	36.77	2.823	3	3	13.02	12
miransertib 1 µM vs. ipatasertib 2 µM	6.967	-3.297 to 17.23	No	ns	0.3629	D-F	47.47	40.5	6.967	2.823	3	3	2.468	12
GSK690693 2 µM vs. ipatasertib 2 µM	-29.8	-40.06 to -19.54	Yes	****	<0.0001	E-F	10.7	40.5	-29.8	2.823	3	3	10.56	12
BT-474														
**Cell number**														
DMSO vs. MK-2206 2 µM	0.8098	0.5797 to 1.040	Yes	****	<0.0001	B	1.399	0.5892	0.8098	0.0756	3	3	10.71	12
DMSO vs. ARQ 751 0.5 µM	0.5704	0.3403 to 0.8005	Yes	****	<0.0001	C	1.399	0.8286	0.5704	0.0756	3	3	7.546	12
DMSO vs. miransertib 1 µM	0.7435	0.5135 to 0.9736	Yes	****	<0.0001	D	1.399	0.6554	0.7435	0.0756	3	3	9.836	12
DMSO vs. GSK690693 2 µM	0.5575	0.3275 to 0.7876	Yes	****	<0.0001	E	1.399	0.8415	0.5575	0.0756	3	3	7.375	12
DMSO vs. ipatasertib 2 µM	0.5115	0.2815 to 0.7416	Yes	****	<0.0001	F	1.399	0.8875	0.5115	0.0756	3	3	6.767	12
**Cell death**														
DMSO vs. MK-2206 2 µM	-30.63	-44.93 to -16.34	Yes	****	<0.0001	A-B	15.37	46	-30.63	3.931	3	3	7.793	12
DMSO vs. ARQ 751 0.5 µM	-9.7	-23.99 to 4.592	No	ns	0.363	A-C	15.37	25.07	-9.7	3.931	3	3	2.468	12
DMSO vs. miransertib 1 µM	-25.9	-40.19 to -11.61	Yes	***	0.0004	A-D	15.37	41.27	-25.9	3.931	3	3	6.589	12
DMSO vs. GSK690693 2 µM	-7	-21.29 to 7.292	No	ns	0.795	A-E	15.37	22.37	-7	3.931	3	3	1.781	12
DMSO vs. ipatasertib 2 µM	-7.567	-21.86 to 6.725	No	ns	0.7055	A-F	15.37	22.93	-7.567	3.931	3	3	1.925	12
MK-2206 2 µM vs. ARQ 751 0.5 µM	20.93	6.642 to 35.23	Yes	**	0.0027	B-C	46	25.07	20.93	3.931	3	3	5.326	12
MK-2206 2 µM vs. miransertib 1 µM	4.733	-9.558 to 19.03	No	ns	0.9871	B-D	46	41.27	4.733	3.931	3	3	1.204	12
MK-2206 2 µM vs. GSK690693 2 µM	23.63	9.342 to 37.93	Yes	***	0.0009	B-E	46	22.37	23.63	3.931	3	3	6.012	12
MK-2206 2 µM vs. ipatasertib 2 µM	23.07	8.775 to 37.36	Yes	**	0.0011	B-F	46	22.93	23.07	3.931	3	3	5.868	12
ARQ 751 0.5 µM vs. miransertib 1 µM	-16.2	-30.49 to -1.908	Yes	*	0.021	C-D	25.07	41.27	-16.2	3.931	3	3	4.121	12
ARQ 751 0.5 µM vs. GSK690693 2 µM	2.7	-11.59 to 16.99	No	ns	>0.9999	C-E	25.07	22.37	2.7	3.931	3	3	0.687	12
ARQ 751 0.5 µM vs. ipatasertib 2 µM	2.133	-12.16 to 16.43	No	ns	>0.9999	C-F	25.07	22.93	2.133	3.931	3	3	0.543	12
miransertib 1 µM vs. GSK690693 2 µM	18.9	4.608 to 33.19	Yes	**	0.0064	D-E	41.27	22.37	18.9	3.931	3	3	4.808	12
miransertib 1 µM vs. ipatasertib 2 µM	18.33	4.042 to 32.63	Yes	**	0.0082	D-F	41.27	22.93	18.33	3.931	3	3	4.664	12
GSK690693 2 µM vs. ipatasertib 2 µM	-0.5667	-14.86 to 13.73	No	ns	>0.9999	E-F	22.37	22.93	-0.5667	3.931	3	3	0.144	12
AU-565														
**Cell number**														
DMSO vs. MK-2206 2 µM	1.04	0.4771 to 1.603	Yes	***	0.0009	B	2.224	1.185	1.04	0.1859	3	3	5.593	10
DMSO vs. ARQ 751 0.5 µM	1.04	0.4771 to 1.603	Yes	***	0.0009	C	2.224	1.185	1.04	0.1859	3	3	5.593	10
DMSO vs. GSK690693 2 µM	1.222	0.6593 to 1.785	Yes	***	0.0003	E	2.224	1.002	1.222	0.1859	3	3	6.573	10
DMSO vs. ipatasertib 4 µM	0.9944	0.4316 to 1.557	Yes	**	0.0013	F	2.224	1.23	0.9944	0.1859	3	3	5.348	10
**Cell death**														
DMSO vs. MK-2206 2 µM	-13.93	-23.86 to -4.009	Yes	**	0.0053	A-B	8.767	22.7	-13.93	2.782	3	3	5.009	10
DMSO vs. ARQ 751 0.5 µM	-13.87	-23.79 to -3.942	Yes	**	0.0055	A-C	8.767	22.63	-13.87	2.782	3	3	4.985	10
DMSO vs. GSK690693 2 µM	-9.133	-19.06 to 0.7912	No	ns	0.0795	A-E	8.767	17.9	-9.133	2.782	3	3	3.283	10
DMSO vs. ipatasertib 4 µM	-6.5	-16.42 to 3.424	No	ns	0.346	A-F	8.767	15.27	-6.5	2.782	3	3	2.337	10

MK-2206 2 µM vs. ARQ 751 0.5 µM	0.06667	-9.858 to 9.991	No	ns	>0.9999	B-C	22.7	22.63	0.06667	2.782	3	3	0.024	10
MK-2206 2 µM vs. GSK690693 2 µM	4.8	-5.124 to 14.72	No	ns	0.7058	B-E	22.7	17.9	4.8	2.782	3	3	1.725	10
MK-2206 2 µM vs. ipatasertib 4 µM	7.433	-2.491 to 17.36	No	ns	0.2109	B-F	22.7	15.27	7.433	2.782	3	3	2.672	10
ARQ 751 0.5 µM vs. GSK690693 2 µM	4.733	-5.191 to 14.66	No	ns	0.7205	C-E	22.63	17.9	4.733	2.782	3	3	1.702	10
ARQ 751 0.5 µM vs. ipatasertib 4 µM	7.367	-2.558 to 17.29	No	ns	0.2188	C-F	22.63	15.27	7.367	2.782	3	3	2.648	10
GSK690693 2 µM vs. ipatasertib 4 µM	2.633	-7.291 to 12.56	No	ns	0.9895	E-F	17.9	15.27	2.633	2.782	3	3	0.947	10
HCC1419														
**Cell number**														
DMSO vs. MK-2206 2 µM	0.8337	0.5447 to 1.123	Yes	****	<0.0001	B	1.947	1.113	0.8337	0.09996	3	3	8.34	10
DMSO vs. ARQ 751 0.5 µM	0.739	0.4501 to 1.028	Yes	****	<0.0001	C	1.947	1.208	0.739	0.09996	3	3	7.393	10
DMSO vs. GSK690693 2 µM	0.6533	0.3644 to 0.9423	Yes	***	0.0003	E	1.947	1.294	0.6533	0.09996	3	3	6.536	10
DMSO vs. ipatasertib 2 µM	0.586	0.2971 to 0.8749	Yes	***	0.0006	F	1.947	1.361	0.586	0.09996	3	3	5.862	10
**Cell death**														
DMSO vs. MK-2206 2 µM	-13.69	-21.83 to -5.546	Yes	**	0.0013	A-B	10.05	23.74	-13.69	2.282	3	3	5.998	10
DMSO vs. ARQ 751 0.5 µM	-12.87	-21.01 to -4.730	Yes	**	0.0022	A-C	10.05	22.93	-12.87	2.282	3	3	5.64	10
DMSO vs. GSK690693 2 µM	-6.919	-15.06 to 1.222	No	ns	0.1194	A-E	10.05	16.97	-6.919	2.282	3	3	3.032	10
DMSO vs. ipatasertib 2 µM	-5.434	-13.58 to 2.707	No	ns	0.3249	A-F	10.05	15.49	-5.434	2.282	3	3	2.381	10
MK-2206 2 µM vs. ARQ 751 0.5 µM	0.816	-7.325 to 8.957	No	ns	>0.9999	B-C	23.74	22.93	0.816	2.282	3	3	0.358	10
MK-2206 2 µM vs. GSK690693 2 µM	6.768	-1.373 to 14.91	No	ns	0.1327	B-E	23.74	16.97	6.768	2.282	3	3	2.966	10
MK-2206 2 µM vs. ipatasertib 2 µM	8.253	0.1119 to 16.39	Yes	*	0.0462	B-F	23.74	15.49	8.253	2.282	3	3	3.617	10
ARQ 751 0.5 µM vs. GSK690693 2 µM	5.952	-2.189 to 14.09	No	ns	0.2325	C-E	22.93	16.97	5.952	2.282	3	3	2.608	10
ARQ 751 0.5 µM vs. ipatasertib 2 µM	7.437	-0.7041 to 15.58	No	ns	0.0826	C-F	22.93	15.49	7.437	2.282	3	3	3.259	10
GSK690693 2 µM vs. ipatasertib 2 µM	1.485	-6.656 to 9.626	No	ns	0.9995	E-F	16.97	15.49	1.485	2.282	3	3	0.651	10
EFM-192A														
**Cell number**														
DMSO vs. MK-2206 2 µM	1.099	0.4294 to 1.769	Yes	**	0.0016	B	3.504	2.404	1.099	0.2201	3	3	4.994	12
DMSO vs. ARQ 751 0.5 µM	1.235	0.5655 to 1.905	Yes	***	0.0006	C	3.504	2.268	1.235	0.2201	3	3	5.613	12
DMSO vs. miransertib 1 µM	1.34	0.6702 to 2.010	Yes	***	0.0003	D	3.504	2.163	1.34	0.2201	3	3	6.088	12
DMSO vs. GSK690693 2 µM	1.167	0.4975 to 1.837	Yes	***	0.0009	E	3.504	2.336	1.167	0.2201	3	3	5.304	12
DMSO vs. ipatasertib 4 µM	1.178	0.5079 to 1.848	Yes	***	0.0009	F	3.504	2.326	1.178	0.2201	3	3	5.351	12
**Cell death**														
DMSO vs. MK-2206 2 µM	-15.47	-27.62 to -3.315	Yes	**	0.0087	A-B	20.8	36.27	-15.47	3.342	3	3	4.628	12
DMSO vs. ARQ 751 0.5 µM	-13.87	-26.02 to -1.715	Yes	*	0.02	A-C	20.8	34.67	-13.87	3.342	3	3	4.149	12
DMSO vs. miransertib 1 µM	-24.33	-36.49 to -12.18	Yes	***	0.0001	A-D	20.8	45.13	-24.33	3.342	3	3	7.281	12
DMSO vs. GSK690693 2 µM	-1.367	-13.52 to 10.79	No	ns	>0.9999	A-E	20.8	22.17	-1.367	3.342	3	3	0.409	12
DMSO vs. ipatasertib 4 µM	-7.5	-19.65 to 4.652	No	ns	0.4946	A-F	20.8	28.3	-7.5	3.342	3	3	2.244	12
MK-2206 2 µM vs. ARQ 751 0.5 µM	1.6	-10.55 to 13.75	No	ns	>0.9999	B-C	36.27	34.67	1.6	3.342	3	3	0.479	12
MK-2206 2 µM vs. miransertib 1 µM	-8.867	-21.02 to 3.285	No	ns	0.2734	B-D	36.27	45.13	-8.867	3.342	3	3	2.653	12
MK-2206 2 µM vs. GSK690693 2 µM	14.1	1.948 to 26.25	Yes	*	0.0177	B-E	36.27	22.17	14.1	3.342	3	3	4.219	12
MK-2206 2 µM vs. ipatasertib 4 µM	7.967	-4.185 to 20.12	No	ns	0.4097	B-F	36.27	28.3	7.967	3.342	3	3	2.384	12
ARQ 751 0.5 µM vs. miransertib 1 µM	-10.47	-22.62 to 1.685	No	ns	0.1224	C-D	34.67	45.13	-10.47	3.342	3	3	3.132	12
ARQ 751 0.5 µM vs. GSK690693 2 µM	12.5	0.3481 to 24.65	Yes	*	0.0415	C-E	34.67	22.17	12.5	3.342	3	3	3.74	12
ARQ 751 0.5 µM vs. ipatasertib 4 µM	6.367	-5.785 to 18.52	No	ns	0.7185	C-F	34.67	28.3	6.367	3.342	3	3	1.905	12
miransertib 1 µM vs. GSK690693 2 µM	22.97	10.81 to 35.12	Yes	***	0.0003	D-E	45.13	22.17	22.97	3.342	3	3	6.872	12
miransertib 1 µM vs. ipatasertib 4 µM	16.83	4.681 to 28.99	Yes	**	0.0044	D-F	45.13	28.3	16.83	3.342	3	3	5.037	12
GSK690693 2 µM vs. ipatasertib 4 µM	-6.133	-18.29 to 6.019	No	ns	0.7625	E-F	22.17	28.3	-6.133	3.342	3	3	1.835	12
HT29														
**Cell number**														
DMSO vs. MK-2206 2 µM	3.253	2.383 to 4.122	Yes	****	<0.0001	B	6.273	3.02	3.253	0.2857	3	3	11.39	12
DMSO vs. ARQ 751 0.5 µM	4.853	3.983 to 5.722	Yes	****	<0.0001	C	6.273	1.42	4.853	0.2857	3	3	16.99	12
DMSO vs. miransertib 1 µM	5.153	4.284 to 6.023	Yes	****	<0.0001	D	6.273	1.12	5.153	0.2857	3	3	18.04	12
DMSO vs. GSK690693 2 µM	4.028	3.159 to 4.898	Yes	****	<0.0001	E	6.273	2.245	4.028	0.2857	3	3	14.1	12
DMSO vs. ipatasertib 2 µM	3.096	2.227 to 3.966	Yes	****	<0.0001	F	6.273	3.177	3.096	0.2857	3	3	10.84	12
**Cell death**														
DMSO vs. MK-2206 2 µM	-6.6	-13.05 to -0.1526	Yes	*	0.0429	A-B	13.5	20.1	-6.6	1.773	3	3	3.722	12
DMSO vs. ARQ 751 0.5 µM	-27.8	-34.25 to -21.35	Yes	****	<0.0001	A-C	13.5	41.3	-27.8	1.773	3	3	15.68	12
DMSO vs. miransertib 1 µM	-32.43	-38.88 to -25.99	Yes	****	<0.0001	A-D	13.5	45.93	-32.43	1.773	3	3	18.29	12
DMSO vs. GSK690693 2 µM	-19.83	-26.28 to -13.39	Yes	****	<0.0001	A-E	13.5	33.33	-19.83	1.773	3	3	11.18	12
DMSO vs. ipatasertib 2 µM	-5.567	-12.01 to 0.8807	No	ns	0.1208	A-F	13.5	19.07	-5.567	1.773	3	3	3.139	12
MK-2206 2 µM vs. ARQ 751 0.5 µM	-21.2	-27.65 to -14.75	Yes	****	<0.0001	B-C	20.1	41.3	-21.2	1.773	3	3	11.96	12
MK-2206 2 µM vs. miransertib 1 µM	-25.83	-32.28 to -19.39	Yes	****	<0.0001	B-D	20.1	45.93	-25.83	1.773	3	3	14.57	12
MK-2206 2 µM vs. GSK690693 2 µM	-13.23	-19.68 to -6.786	Yes	***	0.0001	B-E	20.1	33.33	-13.23	1.773	3	3	7.463	12
MK-2206 2 µM vs. ipatasertib 2 µM	1.033	-5.414 to 7.481	No	ns	>0.9999	B-F	20.1	19.07	1.033	1.773	3	3	0.583	12
ARQ 751 0.5 µM vs. miransertib 1 µM	-4.633	-11.08 to 1.814	No	ns	0.2912	C-D	41.3	45.93	-4.633	1.773	3	3	2.613	12
ARQ 751 0.5 µM vs. GSK690693 2 µM	7.967	1.519 to 14.41	Yes	*	0.011	C-E	41.3	33.33	7.967	1.773	3	3	4.493	12
ARQ 751 0.5 µM vs. ipatasertib 2 µM	22.23	15.79 to 28.68	Yes	****	<0.0001	C-F	41.3	19.07	22.23	1.773	3	3	12.54	12
miransertib 1 µM vs. GSK690693 2 µM	12.6	6.153 to 19.05	Yes	***	0.0002	D-E	45.93	33.33	12.6	1.773	3	3	7.106	12
miransertib 1 µM vs. ipatasertib 2 µM	26.87	20.42 to 33.31	Yes	****	<0.0001	D-F	45.93	19.07	26.87	1.773	3	3	15.15	12
GSK690693 2 µM vs. ipatasertib 2 µM	14.27	7.819 to 20.71	Yes	****	<0.0001	E-F	33.33	19.07	14.27	1.773	3	3	8.045	12
HGC27														
**Cell number**														
DMSO vs. MK-2206 2 µM	22.98	22.02 to 23.94	Yes	****	<0.0001	B	24.72	1.742	22.98	0.316	3	3	72.74	12
DMSO vs. ARQ 751 0.5 µM	22.95	21.99 to 23.91	Yes	****	<0.0001	C	24.72	1.77	22.95	0.316	3	3	72.65	12
DMSO vs. miransertib 1 µM	22.42	21.45 to 23.38	Yes	****	<0.0001	D	24.72	2.307	22.42	0.316	3	3	70.95	12
DMSO vs. GSK690693 2 µM	22.84	21.88 to 23.80	Yes	****	<0.0001	E	24.72	1.882	22.84	0.316	3	3	72.29	12
DMSO vs. ipatasertib 2 µM	23.22	22.26 to 24.18	Yes	****	<0.0001	F	24.72	1.504	23.22	0.316	3	3	73.49	12
**Cell death**														
DMSO vs. MK-2206 2 µM	-8.1	-14.13 to -2.069	Yes	**	0.0056	A-B	6.8	14.9	-8.1	1.659	3	3	4.883	12
DMSO vs. ARQ 751 0.5 µM	-8.167	-14.20 to -2.135	Yes	**	0.0053	A-C	6.8	14.97	-8.167	1.659	3	3	4.923	12
DMSO vs. miransertib 1 µM	-11.3	-17.33 to -5.269	Yes	***	0.0003	A-D	6.8	18.1	-11.3	1.659	3	3	6.812	12
DMSO vs. GSK690693 2 µM	-2.433	-8.465 to 3.598	No	ns	0.9368	A-E	6.8	9.233	-2.433	1.659	3	3	1.467	12
DMSO vs. ipatasertib 2 µM	-0.1	-6.131 to 5.931	No	ns	>0.9999	A-F	6.8	6.9	-0.1	1.659	3	3	0.06	12
MK-2206 2 µM vs. ARQ 751 0.5 µM	-0.06667	-6.098 to 5.965	No	ns	>0.9999	B-C	14.9	14.97	-0.06667	1.659	3	3	0.04	12
MK-2206 2 µM vs. miransertib 1 µM	-3.2	-9.231 to 2.831	No	ns	0.7028	B-D	14.9	18.1	-3.2	1.659	3	3	1.929	12
MK-2206 2 µM vs. GSK690693 2 µM	5.667	-0.3648 to 11.70	No	ns	0.0741	B-E	14.9	9.233	5.667	1.659	3	3	3.416	12
MK-2206 2 µM vs. ipatasertib 2 µM	8	1.969 to 14.03	Yes	**	0.0062	B-F	14.9	6.9	8	1.659	3	3	4.823	12
ARQ 751 0.5 µM vs. miransertib 1 µM	-3.133	-9.165 to 2.898	No	ns	0.7288	C-D	14.97	18.1	-3.133	1.659	3	3	1.889	12

ARQ 751 0.5 µM vs. GSK690693 2 µM	5.733	-0.2981 to 11.76	No	ns	0.0689	C-E	14.97	9.233	5.733	1.659	3	3	3.456	12
ARQ 751 0.5 µM vs. ipatasertib 2 µM	8.067	2.035 to 14.10	Yes	**	0.0058	C-F	14.97	6.9	8.067	1.659	3	3	4.863	12
miransertib 1 µM vs. GSK690693 2 µM	8.867	2.835 to 14.90	Yes	**	0.0026	D-E	18.1	9.233	8.867	1.659	3	3	5.345	12
miransertib 1 µM vs. ipatasertib 2 µM	11.2	5.169 to 17.23	Yes	***	0.0003	D-F	18.1	6.9	11.2	1.659	3	3	6.752	12
GSK690693 2 µM vs. ipatasertib 2 µM	2.333	-3.698 to 8.365	No	ns	0.9534	E-F	9.233	6.9	2.333	1.659	3	3	1.407	12
H1694														
**Cell number**														
DMSO vs. MK-2206 2 µM	0.6302	-0.05805 to 1.318	No	ns	0.0795	B								
DMSO vs. ARQ 751 0.5 µM	0.5269	-0.1613 to 1.215	No	ns	0.1763	C								
DMSO vs. miransertib 1 µM	0.09681	-0.5914 to 0.7850	No	ns	0.9964	D								
DMSO vs. GSK690693 2 µM	-0.1097	-0.7979 to 0.5786	No	ns	0.9936	E								
DMSO vs. ipatasertib 2 µM	0.4667	-0.2215 to 1.155	No	ns	0.2713	F								
**Cell death**														
DMSO vs. MK-2206 2 µM	-21.2	-32.11 to -10.29	Yes	***	0.0003	A-B	9.9	31.1	-21.2	2.937	2	3	7.219	11
DMSO vs. ARQ 751 0.5 µM	-19.8	-30.71 to -8.891	Yes	***	0.0005	A-C	9.9	29.7	-19.8	2.937	2	3	6.742	11
DMSO vs. miransertib 1 µM	-7.6	-18.51 to 3.309	No	ns	0.3184	A-D	9.9	17.5	-7.6	2.937	2	3	2.588	11
DMSO vs. GSK690693 2 µM	1.467	-9.442 to 12.38	No	ns	>0.9999	A-E	9.9	8.433	1.467	2.937	2	3	0.499	11
DMSO vs. ipatasertib 2 µM	-13.9	-24.81 to -2.991	Yes	**	0.0092	A-F	9.9	23.8	-13.9	2.937	2	3	4.733	11
MK-2206 2 µM vs. ARQ 751 0.5 µM	1.4	-8.357 to 11.16	No	ns	>0.9999	B-C	31.1	29.7	1.4	2.627	3	3	0.533	11
MK-2206 2 µM vs. miransertib 1 µM	13.6	3.843 to 23.36	Yes	**	0.0046	B-D	31.1	17.5	13.6	2.627	3	3	5.178	11
MK-2206 2 µM vs. GSK690693 2 µM	22.67	12.91 to 32.42	Yes	****	<0.0001	B-E	31.1	8.433	22.67	2.627	3	3	8.629	11
MK-2206 2 µM vs. ipatasertib 2 µM	7.3	-2.457 to 17.06	No	ns	0.2377	B-F	31.1	23.8	7.3	2.627	3	3	2.779	11
ARQ 751 0.5 µM vs. miransertib 1 µM	12.2	2.443 to 21.96	Yes	*	0.0106	C-D	29.7	17.5	12.2	2.627	3	3	4.645	11
ARQ 751 0.5 µM vs. GSK690693 2 µM	21.27	11.51 to 31.02	Yes	****	<0.0001	C-E	29.7	8.433	21.27	2.627	3	3	8.096	11
ARQ 751 0.5 µM vs. ipatasertib 2 µM	5.9	-3.857 to 15.66	No	ns	0.5081	C-F	29.7	23.8	5.9	2.627	3	3	2.246	11
miransertib 1 µM vs. GSK690693 2 µM	9.067	-0.6906 to 18.82	No	ns	0.0782	D-E	17.5	8.433	9.067	2.627	3	3	3.452	11
miransertib 1 µM vs. ipatasertib 2 µM	-6.3	-16.06 to 3.457	No	ns	0.417	D-F	17.5	23.8	-6.3	2.627	3	3	2.398	11
GSK690693 2 µM vs. ipatasertib 2 µM	-15.37	-25.12 to -5.609	Yes	**	0.0017	E-F	8.433	23.8	-15.37	2.627	3	3	5.85	11
**Figure 1C**														
**Cell number**														
30 min														
DMSO vs. ARQ 751 1 µM	0.7145	0.5686 to 0.8604	Yes	****	<0.0001		1.474	0.7598	0.7145	0.05983	3	3	11.94	18
DMSO vs. ipatasertib 4 µM	0.1727	0.02679 to 0.3186	Yes	*	0.0196		1.474	1.302	0.1727	0.05983	3	3	2.887	18
1 h														
DMSO vs. ARQ 751 1 µM	0.7937	0.6478 to 0.9396	Yes	****	<0.0001		1.412	0.6178	0.7937	0.05983	3	3	13.27	18
DMSO vs. ipatasertib 4 µM	0.2342	0.08830 to 0.3801	Yes	**	0.002		1.412	1.177	0.2342	0.05983	3	3	3.915	18
72 h														
DMSO vs. ARQ 751 1 µM	0.7772	0.6312 to 0.9231	Yes	****	<0.0001		1.435	0.658	0.7772	0.05983	3	3	12.99	18
DMSO vs. ipatasertib 4 µM	0.6991	0.5532 to 0.8450	Yes	****	<0.0001		1.435	0.7361	0.6991	0.05983	3	3	11.68	18
**Cell death**														
30 min														
DMSO vs. ARQ 751 1 µM	-19.93	-23.94 to -15.93	Yes	****	<0.0001									
DMSO vs. ipatasertib 4 µM	0.1333	-3.874 to 4.141	No	ns	0.9959		4.967	24.9	-19.93	1.643	3	3	12.13	18
							4.967	4.833	0.1333	1.643	3	3	0.081	18
1 h														
DMSO vs. ARQ 751 1 µM	-23.47	-27.47 to -19.46	Yes	****	<0.0001									
DMSO vs. ipatasertib 4 µM	-0.3333	-4.341 to 3.674	No	ns	0.9749		5	28.47	-23.47	1.643	3	3	14.28	18
							5	5.333	-0.3333	1.643	3	3	0.203	18
72 h														
DMSO vs. ARQ 751 1 µM	-28.5	-32.51 to -24.49	Yes	****	<0.0001									
DMSO vs. ipatasertib 4 µM	-18.07	-22.07 to -14.06	Yes	****	<0.0001		5.1	33.6	-28.5	1.643	3	3	17.34	18
							5.1	23.17	-18.07	1.643	3	3	10.99	18
**Figure 1D**														
**Cell number**														
DMSO														
Parental vs. AKT1 W80A	0.006667	-0.2865 to 0.2998	No	ns	0.9982		2.482	2.476	0.006667	0.1229	3	3	0.054	24
Parental vs. AKT2 W80A	-0.5797	-0.8728 to -0.2865	Yes	***	0.0002		2.482	3.062	-0.5797	0.1229	3	3	4.716	24
MK-2206 2 µM														
Parental vs. AKT1 W80A	-1.317	-1.610 to -1.024	Yes	****	<0.0001		0.769	2.086	-1.317	0.1229	3	3	10.72	24
Parental vs. AKT2 W80A	-1.307	-1.600 to -1.014	Yes	****	<0.0001		0.769	2.076	-1.307	0.1229	3	3	10.64	24
ARQ 751 0.5 µM														
Parental vs. AKT1 W80A	-0.5517	-0.8448 to -0.2585	Yes	***	0.0003		0.7527	1.304	-0.5517	0.1229	3	3	4.489	24
Parental vs. AKT2 W80A	-1.502	-1.795 to -1.209	Yes	****	<0.0001		0.7527	2.254	-1.502	0.1229	3	3	12.22	24
miransertib 1 µM														
Parental vs. AKT1 W80A	-1.082	-1.375 to -0.7885	Yes	****	<0.0001		0.763	1.845	-1.082	0.1229	3	3	8.801	24
Parental vs. AKT2 W80A	-1.564	-1.857 to -1.271	Yes	****	<0.0001		0.763	2.327	-1.564	0.1229	3	3	12.73	24
**Cell death**														
DMSO														
Parental vs. AKT1 W80A	-0.685	-2.889 to 1.519	No	ns	0.7146		3.708	4.393	-0.685	0.9242	3	3	0.741	24
Parental vs. AKT2 W80A	-0.047	-2.251 to 2.157	No	ns	0.9984		3.708	3.755	-0.047	0.9242	3	3	0.051	24
MK-2206 2 µM														
Parental vs. AKT1 W80A	32.86	30.65 to 35.06	Yes	****	<0.0001		38.54	5.687	32.86	0.9242	3	3	35.55	24
Parental vs. AKT2 W80A	33.77	31.56 to 35.97	Yes	****	<0.0001		38.54	4.776	33.77	0.9242	3	3	36.54	24
ARQ 751 0.5 µM														
Parental vs. AKT1 W80A	14.78	12.58 to 16.99	Yes	****	<0.0001		36.68	21.9	14.78	0.9242	3	3	16	24
Parental vs. AKT2 W80A	31.33	29.13 to 33.53	Yes	****	<0.0001		36.68	5.352	31.33	0.9242	3	3	33.9	24
miransertib 1 µM														
Parental vs. AKT1 W80A	32.55	30.35 to 34.76	Yes	****	<0.0001		39.45	6.896	32.55	0.9242	3	3	35.22	24
Parental vs. AKT2 W80A	34.48	32.28 to 36.69	Yes	****	<0.0001		39.45	4.962	34.48	0.9242	3	3	37.31	24

**Figure 2D**													
**Cell number**													
Parental													
DMSO vs. MK-2206 2 µM	1.182	0.9995 to 1.364	Yes	****	<0.0001	1.85	0.6687	1.182	0.06598	3	3	17.91	42
DMSO vs. ARQ 751 0.5 µM	1.025	0.8432 to 1.207	Yes	****	<0.0001	1.85	0.825	1.025	0.06598	3	3	15.54	42
DMSO vs. miransertib 1 µM	1.258	1.076 to 1.440	Yes	****	<0.0001	1.85	0.592	1.258	0.06598	3	3	19.07	42
DMSO vs. GSK690693 2 µM	0.9603	0.7782 to 1.142	Yes	****	<0.0001	1.85	0.89	0.9603	0.06598	3	3	14.56	42
DMSO vs. ipatasertib 2 µM	0.8227	0.6405 to 1.005	Yes	****	<0.0001	1.85	1.028	0.8227	0.06598	3	3	12.47	42
DMSO vs. capivasertib 4 µM	0.7463	0.5642 to 0.9285	Yes	****	<0.0001	1.85	1.104	0.7463	0.06598	3	3	11.31	42
AKT1													
DMSO vs. MK-2206 2 µM	1.146	0.9635 to 1.328	Yes	****	<0.0001	1.946	0.8007	1.146	0.06598	3	3	17.37	42
DMSO vs. ARQ 751 0.5 µM	1.062	0.8802 to 1.244	Yes	****	<0.0001	1.946	0.884	1.062	0.06598	3	3	16.1	42
DMSO vs. miransertib 1 µM	1.378	1.196 to 1.560	Yes	****	<0.0001	1.946	0.5683	1.378	0.06598	3	3	20.89	42
DMSO vs. GSK690693 2 µM	0.817	0.6349 to 0.9991	Yes	****	<0.0001	1.946	1.129	0.817	0.06598	3	3	12.38	42
DMSO vs. ipatasertib 2 µM	0.931	0.7489 to 1.113	Yes	****	<0.0001	1.946	1.015	0.931	0.06598	3	3	14.11	42
DMSO vs. capivasertib 4 µM	0.852	0.6699 to 1.034	Yes	****	<0.0001	1.946	1.094	0.852	0.06598	3	3	12.91	42
AKT1 Q79K													
DMSO vs. MK-2206 2 µM	1.037	0.8545 to 1.219	Yes	****	<0.0001	1.814	0.777	1.037	0.06598	3	3	15.71	42
DMSO vs. ARQ 751 0.5 µM	0.512	0.3299 to 0.6941	Yes	****	<0.0001	1.814	1.302	0.512	0.06598	3	3	7.76	42
DMSO vs. miransertib 1 µM	0.8973	0.7152 to 1.079	Yes	****	<0.0001	1.814	0.9163	0.8973	0.06598	3	3	13.6	42
DMSO vs. GSK690693 2 µM	0.8777	0.6955 to 1.060	Yes	****	<0.0001	1.814	0.936	0.8777	0.06598	3	3	13.3	42
DMSO vs. ipatasertib 2 µM	0.988	0.8059 to 1.170	Yes	****	<0.0001	1.814	0.8257	0.988	0.06598	3	3	14.98	42
DMSO vs. capivasertib 4 µM	0.8793	0.6972 to 1.061	Yes	****	<0.0001	1.814	0.9343	0.8793	0.06598	3	3	13.33	42
**Cell death**													
Parental													
DMSO vs. MK-2206 2 µM	-39.3	-44.24 to -34.36	Yes	****	<0.0001	4.035	43.34	-39.3	1.789	3	3	21.97	42
DMSO vs. ARQ 751 0.5 µM	-32.9	-37.84 to -27.97	Yes	****	<0.0001	4.035	36.94	-32.9	1.789	3	3	18.4	42
DMSO vs. miransertib 1 µM	-48.23	-53.17 to -43.29	Yes	****	<0.0001	4.035	52.27	-48.23	1.789	3	3	26.96	42
DMSO vs. GSK690693 2 µM	-26.19	-31.13 to -21.25	Yes	****	<0.0001	4.035	30.22	-26.19	1.789	3	3	14.64	42
DMSO vs. ipatasertib 2 µM	-18.98	-23.92 to -14.04	Yes	****	<0.0001	4.035	23.01	-18.98	1.789	3	3	10.61	42
DMSO vs. capivasertib 4 µM	-18.8	-23.74 to -13.87	Yes	****	<0.0001	4.035	22.84	-18.8	1.789	3	3	10.51	42
AKT1													
DMSO vs. MK-2206 2 µM	-43.59	-48.52 to -38.65	Yes	****	<0.0001	4.702	48.29	-43.59	1.789	3	3	24.37	42
DMSO vs. ARQ 751 0.5 µM	-41.73	-46.67 to -36.80	Yes	****	<0.0001	4.702	46.44	-41.73	1.789	3	3	23.33	42
DMSO vs. miransertib 1 µM	-54	-58.94 to -49.06	Yes	****	<0.0001	4.702	58.7	-54	1.789	3	3	30.19	42
DMSO vs. GSK690693 2 µM	-18.95	-23.89 to -14.01	Yes	****	<0.0001	4.702	23.65	-18.95	1.789	3	3	10.6	42
DMSO vs. ipatasertib 2 µM	-20.26	-25.19 to -15.32	Yes	****	<0.0001	4.702	24.96	-20.26	1.789	3	3	11.32	42
DMSO vs. capivasertib 4 µM	-15.85	-20.79 to -10.91	Yes	****	<0.0001	4.702	20.55	-15.85	1.789	3	3	8.862	42
AKT1 Q79K													
DMSO vs. MK-2206 2 µM	-39.18	-44.11 to -34.24	Yes	****	<0.0001	4.936	44.11	-39.18	1.789	3	3	21.9	42
DMSO vs. ARQ 751 0.5 µM	-4.385	-9.323 to 0.5528	No	ns	0.1058	4.936	9.322	-4.385	1.789	3	3	2.452	42
DMSO vs. miransertib 1 µM	-19.31	-24.25 to -14.37	Yes	****	<0.0001	4.936	24.25	-19.31	1.789	3	3	10.8	42
DMSO vs. GSK690693 2 µM	-26.61	-31.54 to -21.67	Yes	****	<0.0001	4.936	31.54	-26.61	1.789	3	3	14.88	42
DMSO vs. ipatasertib 2 µM	-33.56	-38.50 to -28.62	Yes	****	<0.0001	4.936	38.5	-33.56	1.789	3	3	18.76	42
DMSO vs. capivasertib 4 µM	-24.82	-29.76 to -19.88	Yes	****	<0.0001	4.936	29.76	-24.82	1.789	3	3	13.88	42
**Extra Cell death**													
DMSO													
Parental vs. AKT1	-0.6673	-5.115 to 3.781	No	ns	0.9759	4.035	4.702	-0.6673	1.789	3	3	0.373	42
Parental vs. AKT1 Q79K	-0.9013	-5.349 to 3.547	No	ns	0.9438	4.035	4.936	-0.9013	1.789	3	3	0.504	42
AKT1 vs. AKT1 Q79K	-0.234	-4.682 to 4.214	No	ns	0.9989	4.702	4.936	-0.234	1.789	3	3	0.131	42
MK-2206 2 µM													
Parental vs. AKT1	-4.951	-9.399 to -0.5034	Yes	*	0.0248	43.34	48.29	-4.951	1.789	3	3	2.768	42
Parental vs. AKT1 Q79K	-0.775	-5.223 to 3.673	No	ns	0.9631	43.34	44.11	-0.775	1.789	3	3	0.433	42
AKT1 vs. AKT1 Q79K	4.176	-0.2716 to 8.624	No	ns	0.0714	48.29	44.11	4.176	1.789	3	3	2.335	42
ARQ 751 0.5 µM													
Parental vs. AKT1	-9.498	-13.95 to -5.050	Yes	****	<0.0001	36.94	46.44	-9.498	1.789	3	3	5.31	42
Parental vs. AKT1 Q79K	27.62	23.17 to 32.07	Yes	****	<0.0001	36.94	9.322	27.62	1.789	3	3	15.44	42
AKT1 vs. AKT1 Q79K	37.12	32.67 to 41.56	Yes	****	<0.0001	46.44	9.322	37.12	1.789	3	3	20.75	42
miransertib 1 µM													
Parental vs. AKT1	-6.437	-10.89 to -1.989	Yes	**	0.0025	52.27	58.7	-6.437	1.789	3	3	3.599	42
Parental vs. AKT1 Q79K	28.02	23.57 to 32.47	Yes	****	<0.0001	52.27	24.25	28.02	1.789	3	3	15.66	42
AKT1 vs. AKT1 Q79K	34.46	30.01 to 38.90	Yes	****	<0.0001	58.7	24.25	34.46	1.789	3	3	19.26	42
GSK690693 2 µM													
Parental vs. AKT1	6.569	2.121 to 11.02	Yes	**	0.002	30.22	23.65	6.569	1.789	3	3	3.673	42
Parental vs. AKT1 Q79K	-1.32	-5.768 to 3.128	No	ns	0.8465	30.22	31.54	-1.32	1.789	3	3	0.738	42
AKT1 vs. AKT1 Q79K	-7.889	-12.34 to -3.441	Yes	***	0.0002	23.65	31.54	-7.889	1.789	3	3	4.411	42
ipatasertib 2 µM													
Parental vs. AKT1	-1.944	-6.392 to 2.504	No	ns	0.6319	23.01	24.96	-1.944	1.789	3	3	1.087	42
Parental vs. AKT1 Q79K	-15.49	-19.93 to -11.04	Yes	****	<0.0001	23.01	38.5	-15.49	1.789	3	3	8.658	42
AKT1 vs. AKT1 Q79K	-13.54	-17.99 to -9.094	Yes	****	<0.0001	24.96	38.5	-13.54	1.789	3	3	7.571	42
capivasertib 4 µM													
Parental vs. AKT1	2.285	-2.163 to 6.733	No	ns	0.5039	22.84	20.55	2.285	1.789	3	3	1.278	42
Parental vs. AKT1 Q79K	-6.918	-11.37 to -2.470	Yes	**	0.0011	22.84	29.76	-6.918	1.789	3	3	3.868	42
AKT1 vs. AKT1 Q79K	-9.203	-13.65 to -4.755	Yes	****	<0.0001	20.55	29.76	-9.203	1.789	3	3	5.145	42
**Figure 2E**													
**Cell number**													
Parental													

DMSO vs. 0.25	0.4893	0.3060 to 0.6727	Yes	****	<0.0001	1.65	1.161	0.4893	0.06461	3	3	7.574	54
DMSO vs. 0.5	0.738	0.5547 to 0.9213	Yes	****	<0.0001	1.65	0.9123	0.738	0.06461	3	3	11.42	54
DMSO vs. 1	0.7547	0.5713 to 0.9380	Yes	****	<0.0001	1.65	0.8957	0.7547	0.06461	3	3	11.68	54
DMSO vs. 2	0.9083	0.7250 to 1.092	Yes	****	<0.0001	1.65	0.742	0.9083	0.06461	3	3	14.06	54
DMSO vs. 0.125	0.542	0.3587 to 0.7253	Yes	****	<0.0001	1.65	1.108	0.542	0.06461	3	3	8.389	54
DMSO vs. 0.25	0.646	0.4627 to 0.8293	Yes	****	<0.0001	1.65	1.004	0.646	0.06461	3	3	9.999	54
DMSO vs. 0.5	0.684	0.5007 to 0.8673	Yes	****	<0.0001	1.65	0.9663	0.684	0.06461	3	3	10.59	54
DMSO vs. 1	0.898	0.7147 to 1.081	Yes	****	<0.0001	1.65	0.7523	0.898	0.06461	3	3	13.9	54
AKT1													
DMSO vs. 0.25	0.335	0.1517 to 0.5183	Yes	****	<0.0001	1.444	1.109	0.335	0.06461	3	3	5.185	54
DMSO vs. 0.5	0.5643	0.3810 to 0.7477	Yes	****	<0.0001	1.444	0.88	0.5643	0.06461	3	3	8.735	54
DMSO vs. 1	0.707	0.5237 to 0.8903	Yes	****	<0.0001	1.444	0.7373	0.707	0.06461	3	3	10.94	54
DMSO vs. 2	0.787	0.6037 to 0.9703	Yes	****	<0.0001	1.444	0.6573	0.787	0.06461	3	3	12.18	54
DMSO vs. 0.125	0.2857	0.1023 to 0.4690	Yes	***	0.0004	1.444	1.159	0.2857	0.06461	3	3	4.422	54
DMSO vs. 0.25	0.3943	0.2110 to 0.5777	Yes	****	<0.0001	1.444	1.05	0.3943	0.06461	3	3	6.104	54
DMSO vs. 0.5	0.571	0.3877 to 0.7543	Yes	****	<0.0001	1.444	0.8733	0.571	0.06461	3	3	8.838	54
DMSO vs. 1	0.804	0.6207 to 0.9873	Yes	****	<0.0001	1.444	0.6403	0.804	0.06461	3	3	12.44	54
AKT1 Q79K													
DMSO vs. 0.25	0.432	0.2487 to 0.6153	Yes	****	<0.0001	1.734	1.302	0.432	0.06461	3	3	6.687	54
DMSO vs. 0.5	0.7503	0.5670 to 0.9337	Yes	****	<0.0001	1.734	0.9837	0.7503	0.06461	3	3	11.61	54
DMSO vs. 1	0.896	0.7127 to 1.079	Yes	****	<0.0001	1.734	0.838	0.896	0.06461	3	3	13.87	54
DMSO vs. 2	0.9213	0.7380 to 1.105	Yes	****	<0.0001	1.734	0.8127	0.9213	0.06461	3	3	14.26	54
DMSO vs. 0.125	0.451	0.2677 to 0.6343	Yes	****	<0.0001	1.734	1.283	0.451	0.06461	3	3	6.981	54
DMSO vs. 0.25	0.59	0.4067 to 0.7733	Yes	****	<0.0001	1.734	1.144	0.59	0.06461	3	3	9.132	54
DMSO vs. 0.5	0.619	0.4357 to 0.8023	Yes	****	<0.0001	1.734	1.115	0.619	0.06461	3	3	9.581	54
DMSO vs. 1	0.706	0.5227 to 0.8893	Yes	****	<0.0001	1.734	1.028	0.706	0.06461	3	3	10.93	54
**Cell death**													
Parental													
DMSO vs. MK-2206 0.25 µM	-6.135	-12.27 to 0.004183	No	ns	0.0503	5.56	11.7	-6.135	2.164	3	3	2.836	54
DMSO vs. MK-2206 0.5 µM	-12.71	-18.85 to -6.570	Yes	****	<0.0001	5.56	18.27	-12.71	2.164	3	3	5.874	54
DMSO vs. MK-2206 1 µM	-17.79	-23.93 to -11.65	Yes	****	<0.0001	5.56	23.35	-17.79	2.164	3	3	8.223	54
DMSO vs. MK-2206 2 µM	-30.22	-36.36 to -24.08	Yes	****	<0.0001	5.56	35.78	-30.22	2.164	3	3	13.97	54
DMSO vs. miransertib 0.125 µM	-6.167	-12.31 to -0.02715	Yes	*	0.0484	5.56	11.73	-6.167	2.164	3	3	2.85	54
DMSO vs. miransertib 0.25 µM	-7.709	-13.85 to -1.569	Yes	**	0.0062	5.56	13.27	-7.709	2.164	3	3	3.563	54
DMSO vs. miransertib 0.5 µM	-12.03	-18.17 to -5.892	Yes	****	<0.0001	5.56	17.59	-12.03	2.164	3	3	5.561	54
DMSO vs. miransertib 1 µM	-26.06	-32.20 to -19.92	Yes	****	<0.0001	5.56	31.62	-26.06	2.164	3	3	12.04	54
AKT1													
DMSO vs. MK-2206 0.25 µM	-5.797	-11.94 to 0.3425	No	ns	0.0755	5.063	10.86	-5.797	2.164	3	3	2.679	54
DMSO vs. MK-2206 0.5 µM	-12.9	-19.04 to -6.765	Yes	****	<0.0001	5.063	17.97	-12.9	2.164	3	3	5.964	54
DMSO vs. MK-2206 1 µM	-18.27	-24.41 to -12.13	Yes	****	<0.0001	5.063	23.33	-18.27	2.164	3	3	8.444	54
DMSO vs. MK-2206 2 µM	-27.26	-33.40 to -21.12	Yes	****	<0.0001	5.063	32.33	-27.26	2.164	3	3	12.6	54
DMSO vs. miransertib 0.125 µM	-2.871	-9.011 to 3.269	No	ns	0.8149	5.063	7.934	-2.871	2.164	3	3	1.327	54
DMSO vs. miransertib 0.25 µM	-6.947	-13.09 to -0.8072	Yes	*	0.0177	5.063	12.01	-6.947	2.164	3	3	3.211	54
DMSO vs. miransertib 0.5 µM	-11.54	-17.68 to -5.397	Yes	****	<0.0001	5.063	16.6	-11.54	2.164	3	3	5.332	54
DMSO vs. miransertib 1 µM	-24.03	-30.17 to -17.89	Yes	****	<0.0001	5.063	29.09	-24.03	2.164	3	3	11.11	54
AKT1 Q79K													
DMSO vs. MK-2206 0.25 µM	-1.696	-7.836 to 4.444	No	ns	0.9898	4.948	6.644	-1.696	2.164	3	3	0.784	54
DMSO vs. MK-2206 0.5 µM	-7.032	-13.17 to -0.8922	Yes	*	0.0158	4.948	11.98	-7.032	2.164	3	3	3.25	54
DMSO vs. MK-2206 1 µM	-12.27	-18.40 to -6.125	Yes	****	<0.0001	4.948	17.21	-12.27	2.164	3	3	5.669	54
DMSO vs. MK-2206 2 µM	-22.2	-28.34 to -16.06	Yes	****	<0.0001	4.948	27.15	-22.2	2.164	3	3	10.26	54
DMSO vs. miransertib 0.125 µM	-1.081	-7.221 to 5.059	No	ns	0.9996	4.948	6.029	-1.081	2.164	3	3	0.5	54
DMSO vs. miransertib 0.25 µM	-4.465	-10.60 to 1.675	No	ns	0.3017	4.948	9.413	-4.465	2.164	3	3	2.063	54
DMSO vs. miransertib 0.5 µM	-2.004	-8.144 to 4.135	No	ns	0.9713	4.948	6.953	-2.004	2.164	3	3	0.926	54
DMSO vs. miransertib 1 µM	-5.014	-11.15 to 1.126	No	ns	0.1787	4.948	9.962	-5.014	2.164	3	3	2.317	54
**Figure 2F**													
**1 h**													
**Cell number**													
DMSO													
Parental vs. AKT1	0.15	-0.1239 to 0.4239	No	ns	0.4215	1.83	1.68	0.15	0.1041	3	3	1.441	18
Parental vs. AKT1 Q79K	0.17	-0.1039 to 0.4439	No	ns	0.3181	1.83	1.66	0.17	0.1041	3	3	1.633	18
AKT1 vs. AKT1 Q79K	0.02	-0.2539 to 0.2939	No	ns	0.9966	1.68	1.66	0.02	0.1041	3	3	0.192	18
MK-2206 2 µM													
Parental vs. AKT1	nsnsns	-0.1106 to 0.4372	No	ns	0.3507	1.35	1.187	0.1633	0.1041	3	3	1.569	18
Parental vs. AKT1 Q79K	0.02333	-0.2506 to 0.2972	No	ns	0.9947	1.35	1.327	0.02333	0.1041	3	3	0.224	18
AKT1 vs. AKT1 Q79K	-0.14	-0.4139 to 0.1339	No	ns	0.4791	1.187	1.327	-0.14	0.1041	3	3	1.345	18
miransertib 1 µM													
Parental vs. AKT1	-0.02333	-0.2972 to 0.2506	No	ns	0.9947	1.167	1.19	-0.02333	0.1041	3	3	0.224	18
Parental vs. AKT1 Q79K	-0.1133	-0.3872 to 0.1606	No	ns	0.6431	1.167	1.28	-0.1133	0.1041	3	3	1.089	18
AKT1 vs. AKT1 Q79K	-0.09	-0.3639 to 0.1839	No	ns	0.7826	1.19	1.28	-0.09	0.1041	3	3	0.865	18
**Cell death**													
DMSO													
Parental vs. AKT1	-2.02	-5.831 to 1.791	No	ns	0.4488	2.49	4.51	-2.02	1.448	3	3	1.395	18
Parental vs. AKT1 Q79K	-2.22	-6.031 to 1.591	No	ns	0.3699	2.49	4.71	-2.22	1.448	3	3	1.533	18
AKT1 vs. AKT1 Q79K	-0.2	-4.011 to 3.611	No	ns	0.9987	4.51	4.71	-0.2	1.448	3	3	0.138	18
MK-2206 2 µM													
Parental vs. AKT1	0	-3.811 to 3.811	No	ns	>0.9999	13.66	13.66	0	1.448	3	3	0	18
Parental vs. AKT1 Q79K	4.23	0.4195 to 8.041	Yes	*	0.0271	13.66	9.43	4.23	1.448	3	3	2.921	18
AKT1 vs. AKT1 Q79K	4.23	0.4195 to 8.041	Yes	*	0.0271	13.66	9.43	4.23	1.448	3	3	2.921	18
miransertib 1 µM													
Parental vs. AKT1	-4.92	-8.731 to -1.109	Yes	**	0.0096	16.11	21.03	-4.92	1.448	3	3	3.397	18
Parental vs. AKT1 Q79K	6.397	2.586 to 10.21	Yes	***	0.001	16.11	9.717	6.397	1.448	3	3	4.417	18

AKT1 vs. AKT1 Q79K	11.32	7.506 to 15.13	Yes	****	<0.0001	21.03	9.717	11.32	1.448	3	3	7.814	18
**72h**													
**Cell number**													
DMSO													
Parental vs. AKT1	0.06667	-0.1094 to 0.2428	No	ns	0.7024	1.833	1.767	0.06667	0.06693	3	3	0.996	18
Parental vs. AKT1 Q79K	0.1167	-0.05942 to 0.2928	No	ns	0.267	1.833	1.717	0.1167	0.06693	3	3	1.743	18
AKT1 vs. AKT1 Q79K	0.05	-0.1261 to 0.2261	No	ns	0.8466	1.767	1.717	0.05	0.06693	3	3	0.747	18
MK-2206 2 µM													
Parental vs. AKT1	-0.06667	-0.2428 to 0.1094	No	ns	0.7024	0.86	0.9267	-0.06667	0.06693	3	3	0.996	18
Parental vs. AKT1 Q79K	0.04	-0.1361 to 0.2161	No	ns	0.9134	0.86	0.82	0.04	0.06693	3	3	0.598	18
AKT1 vs. AKT1 Q79K	0.1067	-0.06942 to 0.2828	No	ns	0.3378	0.9267	0.82	0.1067	0.06693	3	3	1.594	18
miransertib 1 µM													
Parental vs. AKT1	0.04667	-0.1294 to 0.2228	No	ns	0.8709	0.81	0.7633	0.04667	0.06693	3	3	0.697	18
Parental vs. AKT1 Q79K	-0.16	-0.3361 to 0.01609	No	ns	0.0815	0.81	0.97	-0.16	0.06693	3	3	2.391	18
AKT1 vs. AKT1 Q79K	-0.2067	-0.3828 to -0.03058	Yes	*	0.0189	0.7633	0.97	-0.2067	0.06693	3	3	3.088	18
**Cell death**													
DMSO													
Parental vs. AKT1	-1.09	-7.304 to 5.124	No	ns	0.9571	4.83	5.92	-1.09	2.362	3	3	0.462	18
Parental vs. AKT1 Q79K	0.6967	-5.518 to 6.911	No	ns	0.9881	4.83	4.133	0.6967	2.362	3	3	0.295	18
AKT1 vs. AKT1 Q79K	1.787	-4.428 to 8.001	No	ns	0.8418	5.92	4.133	1.787	2.362	3	3	0.757	18
MK-2206 2 µM													
Parental vs. AKT1	-0.52	-6.734 to 5.694	No	ns	0.9949	41.72	42.24	-0.52	2.362	3	3	0.22	18
Parental vs. AKT1 Q79K	3.143	-3.071 to 9.358	No	ns	0.4877	41.72	38.58	3.143	2.362	3	3	1.331	18
AKT1 vs. AKT1 Q79K	3.663	-2.551 to 9.878	No	ns	0.3602	42.24	38.58	3.663	2.362	3	3	1.551	18
miransertib 1 µM													
Parental vs. AKT1	-6.633	-12.85 to -0.4190	Yes	*	0.0345	47.48	54.11	-6.633	2.362	3	3	2.809	18
Parental vs. AKT1 Q79K	21.1	14.89 to 27.32	Yes	****	<0.0001	47.48	26.38	21.1	2.362	3	3	8.935	18
AKT1 vs. AKT1 Q79K	27.74	21.52 to 33.95	Yes	****	<0.0001	54.11	26.38	27.74	2.362	3	3	11.74	18
**Figure 2H**													
**Cell number**													
Parental													
DMSO vs. MK-2206 2 µM	1.359	1.142 to 1.576	Yes	****	<0.0001	1.938	0.5797	1.359	0.08397	3	3	16.18	50
DMSO vs. ARQ 751 0.5 µM	1.382	1.165 to 1.599	Yes	****	<0.0001	1.938	0.556	1.382	0.08397	3	3	16.46	50
DMSO vs. miransertib 1 µM	1.465	1.248 to 1.682	Yes	****	<0.0001	1.938	0.4732	1.465	0.08397	3	3	17.45	50
DMSO vs. ipatasertib 2 µM	1.131	0.9139 to 1.348	Yes	****	<0.0001	1.938	0.8074	1.131	0.08397	3	3	13.47	50
AKT1													
DMSO vs. MK-2206 2 µM	1.245	1.028 to 1.462	Yes	****	<0.0001	1.873	0.6281	1.245	0.08397	3	3	14.82	50
DMSO vs. ARQ 751 0.5 µM	1.217	0.9998 to 1.434	Yes	****	<0.0001	1.873	0.656	1.217	0.08397	3	3	14.49	50
DMSO vs. miransertib 1 µM	1.299	1.082 to 1.516	Yes	****	<0.0001	1.873	0.574	1.299	0.08397	3	3	15.47	50
DMSO vs. ipatasertib 2 µM	0.8611	0.6442 to 1.078	Yes	****	<0.0001	1.873	1.012	0.8611	0.08397	3	3	10.26	50
AKT1 D292A													
DMSO vs. MK-2206 2 µM	1.391	1.174 to 1.608	Yes	****	<0.0001	2.099	0.7079	1.391	0.08397	3	3	16.57	50
DMSO vs. ARQ 751 0.5 µM	1.413	1.196 to 1.630	Yes	****	<0.0001	2.099	0.6864	1.413	0.08397	3	3	16.83	50
DMSO vs. miransertib 1 µM	1.531	1.314 to 1.748	Yes	****	<0.0001	2.099	0.5679	1.531	0.08397	3	3	18.24	50
DMSO vs. ipatasertib 2 µM	1.248	1.031 to 1.465	Yes	****	<0.0001	2.099	0.8515	1.248	0.08397	3	3	14.86	50
AKT1 D323H													
DMSO vs. MK-2206 2 µM	0.2753	0.05837 to 0.4923	Yes	**	0.0076	1.926	1.65	0.2753	0.08397	3	3	3.279	50
DMSO vs. ARQ 751 0.5 µM	0.2505	0.03353 to 0.4674	Yes	*	0.0175	1.926	1.675	0.2505	0.08397	3	3	2.983	50
DMSO vs. miransertib 1 µM	0.4596	0.2426 to 0.6765	Yes	****	<0.0001	1.926	1.466	0.4596	0.08397	3	3	5.473	50
DMSO vs. ipatasertib 2 µM	1.004	0.7870 to 1.221	Yes	****	<0.0001	1.926	0.9218	1.004	0.08397	3	3	11.96	50
AKT1 D292A-D323H													
DMSO vs. MK-2206 2 µM	1.155	0.9383 to 1.372	Yes	****	<0.0001	1.78	0.6251	1.155	0.08397	3	3	13.76	50
DMSO vs. ARQ 751 0.5 µM	1.18	0.9628 to 1.397	Yes	****	<0.0001	1.78	0.6007	1.18	0.08397	3	3	14.05	50
DMSO vs. miransertib 1 µM	1.308	1.091 to 1.525	Yes	****	<0.0001	1.78	0.4726	1.308	0.08397	3	3	15.58	50
DMSO vs. ipatasertib 2 µM	0.8854	0.6685 to 1.102	Yes	****	<0.0001								
**Cell death**													
Parental													
DMSO vs. MK-2206 2 µM	-46.6	-53.20 to -40.00	Yes	****	<0.0001	4.467	51.07	-46.6	2.553	3	3	18.25	50
DMSO vs. ARQ 751 0.5 µM	-44.07	-50.66 to -37.47	Yes	****	<0.0001	4.467	48.53	-44.07	2.553	3	3	17.26	50
DMSO vs. miransertib 1 µM	-51.67	-58.26 to -45.07	Yes	****	<0.0001	4.467	56.13	-51.67	2.553	3	3	20.24	50
DMSO vs. ipatasertib 2 µM	-21.87	-28.46 to -15.27	Yes	****	<0.0001	4.467	26.33	-21.87	2.553	3	3	8.566	50
AKT1													
DMSO vs. MK-2206 2 µM	-43.2	-49.80 to -36.60	Yes	****	<0.0001	4.633	47.83	-43.2	2.553	3	3	16.92	50
DMSO vs. ARQ 751 0.5 µM	-41.27	-47.86 to -34.67	Yes	****	<0.0001	4.633	45.9	-41.27	2.553	3	3	16.17	50
DMSO vs. miransertib 1 µM	-48.37	-54.96 to -41.77	Yes	****	<0.0001	4.633	53	-48.37	2.553	3	3	18.95	50
DMSO vs. ipatasertib 2 µM	-18.5	-25.10 to -11.90	Yes	****	<0.0001	4.633	23.13	-18.5	2.553	3	3	7.247	50
AKT1 D292A													
DMSO vs. MK-2206 2 µM	-43.67	-50.26 to -37.07	Yes	****	<0.0001	4.5	48.17	-43.67	2.553	3	3	17.11	50
DMSO vs. ARQ 751 0.5 µM	-44.87	-51.46 to -38.27	Yes	****	<0.0001	4.5	49.37	-44.87	2.553	3	3	17.58	50
DMSO vs. miransertib 1 µM	-54.17	-60.76 to -47.57	Yes	****	<0.0001	4.5	58.67	-54.17	2.553	3	3	21.22	50
DMSO vs. ipatasertib 2 µM	-25.97	-32.56 to -19.37	Yes	****	<0.0001	4.5	30.47	-25.97	2.553	3	3	10.17	50
AKT1 D323H													
DMSO vs. MK-2206 2 µM	-0.6333	-7.229 to 5.962	No	ns	0.9986	4.333	4.967	-0.6333	2.553	3	3	0.248	50
DMSO vs. ARQ 751 0.5 µM	-1.6	-8.196 to 4.996	No	ns	0.9527	4.333	5.933	-1.6	2.553	3	3	0.627	50
DMSO vs. miransertib 1 µM	-2.533	-9.129 to 4.062	No	ns	0.7934	4.333	6.867	-2.533	2.553	3	3	0.992	50
DMSO vs. ipatasertib 2 µM	-24.43	-31.03 to -17.84	Yes	****	<0.0001	4.333	28.77	-24.43	2.553	3	3	9.571	50
AKT1 D292A-D323H													

DMSO vs. MK-2206 2 µM	-41.63	-48.23 to -35.04	Yes	****	<0.0001	8.133	49.77	-41.63	2.553	3	3	16.31	50
DMSO vs. ARQ 751 0.5 µM	-39.97	-46.56 to -33.37	Yes	****	<0.0001	8.133	48.1	-39.97	2.553	3	3	15.66	50
DMSO vs. miransertib 1 µM	-50.57	-57.16 to -43.97	Yes	****	<0.0001	8.133	58.7	-50.57	2.553	3	3	19.81	50
DMSO vs. ipatasertib 2 µM	-20.07	-26.66 to -13.47	Yes	****	<0.0001	8.133	28.2	-20.07	2.553	3	3	7.861	50

Figure 3A

Cell Number

Parental													
DMSO vs. MK-2206 2 µM	1.509	1.318 to 1.699	Yes	****	<0.0001	2.235	0.7258	1.509	0.06888	3	3	21.9	42
DMSO vs. ARQ 751 0.5 µM	1.446	1.256 to 1.636	Yes	****	<0.0001	2.235	0.7888	1.446	0.06888	3	3	20.99	42
DMSO vs. miransertib 1 µM	1.575	1.385 to 1.765	Yes	****	<0.0001	2.235	0.6596	1.575	0.06888	3	3	22.86	42
DMSO vs. GSK690693 2 µM	1.136	0.9459 to 1.326	Yes	****	<0.0001	2.235	1.098	1.136	0.06888	3	3	16.49	42
DMSO vs. ipatasertib 2 µM	1.128	0.9376 to 1.318	Yes	****	<0.0001	2.235	1.107	1.128	0.06888	3	3	16.37	42
DMSO vs. capivasertib 4 µM	1.078	0.8879 to 1.268	Yes	****	<0.0001	2.235	1.156	1.078	0.06888	3	3	15.65	42
AKT1													
DMSO vs. MK-2206 2 µM	1.2	1.010 to 1.390	Yes	****	<0.0001	1.868	0.6676	1.2	0.06888	3	3	17.42	42
DMSO vs. ARQ 751 0.5 µM	1.208	1.018 to 1.398	Yes	****	<0.0001	1.868	0.6597	1.208	0.06888	3	3	17.54	42
DMSO vs. miransertib 1 µM	1.303	1.113 to 1.493	Yes	****	<0.0001	1.868	0.565	1.303	0.06888	3	3	18.91	42
DMSO vs. GSK690693 2 µM	0.7618	0.5716 to 0.9519	Yes	****	<0.0001	1.868	1.106	0.7618	0.06888	3	3	11.06	42
DMSO vs. ipatasertib 2 µM	0.951	0.7609 to 1.141	Yes	****	<0.0001	1.868	0.9168	0.951	0.06888	3	3	13.81	42
DMSO vs. capivasertib 4 µM	0.5962	0.4060 to 0.7863	Yes	****	<0.0001	1.868	1.272	0.5962	0.06888	3	3	8.655	42
AKT1 M227Q													
DMSO vs. MK-2206 2 µM	1.305	1.115 to 1.495	Yes	****	<0.0001	2.142	0.8371	1.305	0.06888	3	3	18.94	42
DMSO vs. ARQ 751 0.5 µM	0.6711	0.4809 to 0.8612	Yes	****	<0.0001	2.142	1.471	0.6711	0.06888	3	3	9.742	42
DMSO vs. miransertib 1 µM	1.243	1.053 to 1.433	Yes	****	<0.0001	2.142	0.899	1.243	0.06888	3	3	18.04	42
DMSO vs. GSK690693 2 µM	0.7618	0.5716 to 0.9519	Yes	****	<0.0001	2.142	1.38	0.7618	0.06888	3	3	11.06	42
DMSO vs. ipatasertib 2 µM	1.251	1.061 to 1.442	Yes	****	<0.0001	2.142	0.8904	1.251	0.06888	3	3	18.17	42
DMSO vs. capivasertib 4 µM	1.241	1.051 to 1.431	Yes	****	<0.0001	2.142	0.9005	1.241	0.06888	3	3	18.02	42

Cell death

Parental													
DMSO vs. MK-2206 2 µM	-42.4	-49.36 to -35.44	Yes	****	<0.0001	3.667	46.07	-42.4	2.519	3	3	16.83	42
DMSO vs. ARQ 751 0.5 µM	-35.63	-42.59 to -28.68	Yes	****	<0.0001	3.667	39.3	-35.63	2.519	3	3	14.14	42
DMSO vs. miransertib 1 µM	-46.03	-52.99 to -39.08	Yes	****	<0.0001	3.667	49.7	-46.03	2.519	3	3	18.27	42
DMSO vs. GSK690693 2 µM	-20.43	-27.39 to -13.48	Yes	****	<0.0001	3.667	24.1	-20.43	2.519	3	3	8.111	42
DMSO vs. ipatasertib 2 µM	-23.47	-30.42 to -16.51	Yes	****	<0.0001	3.667	27.13	-23.47	2.519	3	3	9.315	42
DMSO vs. capivasertib 4 µM	-22.53	-29.49 to -15.58	Yes	****	<0.0001	3.667	26.2	-22.53	2.519	3	3	8.944	42
AKT1													
DMSO vs. MK-2206 2 µM	-40.8	-47.76 to -33.84	Yes	****	<0.0001	6.267	47.07	-40.8	2.519	3	3	16.19	42
DMSO vs. ARQ 751 0.5 µM	-38.27	-45.22 to -31.31	Yes	****	<0.0001	6.267	44.53	-38.27	2.519	3	3	15.19	42
DMSO vs. miransertib 1 µM	-48.73	-55.69 to -41.78	Yes	****	<0.0001	6.267	55	-48.73	2.519	3	3	19.34	42
DMSO vs. GSK690693 2 µM	-18.67	-25.62 to -11.71	Yes	****	<0.0001	6.267	24.93	-18.67	2.519	3	3	7.409	42
DMSO vs. ipatasertib 2 µM	-14.57	-21.52 to -7.611	Yes	****	<0.0001	6.267	20.83	-14.57	2.519	3	3	5.782	42
DMSO vs. capivasertib 4 µM	-7.6	-14.56 to -0.6446	Yes	*	0.0257	6.267	13.87	-7.6	2.519	3	3	3.017	42
AKT1 M227Q													
DMSO vs. MK-2206 2 µM	-34.63	-41.59 to -27.68	Yes	****	<0.0001	4.5	39.13	-34.63	2.519	3	3	13.75	42
DMSO vs. ARQ 751 0.5 µM	-3.333	-10.29 to 3.622	No	ns	0.7237	4.5	7.833	-3.333	2.519	3	3	1.323	42
DMSO vs. miransertib 1 µM	-26.5	-33.46 to -19.54	Yes	****	<0.0001	4.5	31	-26.5	2.519	3	3	10.52	42
DMSO vs. GSK690693 2 µM	-7.167	-14.12 to -0.2112	Yes	*	0.0404	4.5	11.67	-7.167	2.519	3	3	2.845	42
DMSO vs. ipatasertib 2 µM	-20.73	-27.69 to -13.78	Yes	****	<0.0001	4.5	25.23	-20.73	2.519	3	3	8.23	42
DMSO vs. capivasertib 4 µM	-24.83	-31.79 to -17.88	Yes	****	<0.0001	4.5	29.33	-24.83	2.519	3	3	9.857	42

Extra Cell death

DMSO													
Parental vs. AKT1	-2.6	-8.865 to 3.665	No	ns	0.6686	3.667	6.267	-2.6	2.519	3	3	1.032	42
Parental vs. AKT1 M227Q	-0.8333	-7.098 to 5.432	No	ns	0.9829	3.667	4.5	-0.8333	2.519	3	3	0.331	42
AKT1 vs. AKT1 M227Q	1.767	-4.498 to 8.032	No	ns	0.865	6.267	4.5	1.767	2.519	3	3	0.701	42
MK-2206 2 µM													
Parental vs. AKT1	-1	-7.265 to 5.265	No	ns	0.9712	46.07	47.07	-1	2.519	3	3	0.397	42
Parental vs. AKT1 M227Q	6.933	0.6684 to 13.20	Yes	*	0.0259	46.07	39.13	6.933	2.519	3	3	2.752	42
AKT1 vs. AKT1 M227Q	7.933	1.668 to 14.20	Yes	**	0.009	47.07	39.13	7.933	2.519	3	3	3.149	42
ARQ 751 0.5 µM													
Parental vs. AKT1	-5.233	-11.50 to 1.032	No	ns	0.1261	39.3	44.53	-5.233	2.519	3	3	2.077	42
Parental vs. AKT1 M227Q	31.47	25.20 to 37.73	Yes	****	<0.0001	39.3	7.833	31.47	2.519	3	3	12.49	42
AKT1 vs. AKT1 M227Q	36.7	30.44 to 42.96	Yes	****	<0.0001	44.53	7.833	36.7	2.519	3	3	14.57	42
miransertib 1 µM													
Parental vs. AKT1	-5.3	-11.56 to 0.9649	No	ns	0.1192	49.7	55	-5.3	2.519	3	3	2.104	42
Parental vs. AKT1 M227Q	18.7	12.44 to 24.96	Yes	****	<0.0001	49.7	31	18.7	2.519	3	3	7.423	42
AKT1 vs. AKT1 M227Q	24	17.74 to 30.26	Yes	****	<0.0001	55	31	24	2.519	3	3	9.526	42
GSK690693 2 µM													
Parental vs. AKT1	-0.8333	-7.098 to 5.432	No	ns	0.9829	24.1	24.93	-0.8333	2.519	3	3	0.331	42
Parental vs. AKT1 M227Q	12.43	6.168 to 18.70	Yes	****	<0.0001	24.1	11.67	12.43	2.519	3	3	4.935	42
AKT1 vs. AKT1 M227Q	13.27	7.002 to 19.53	Yes	****	<0.0001	24.93	11.67	13.27	2.519	3	3	5.266	42
ipatasertib 2 µM													
Parental vs. AKT1	6.3	0.03507 to 12.56	Yes	*	0.0484	27.13	20.83	6.3	2.519	3	3	2.501	42
Parental vs. AKT1 M227Q	1.9	-4.365 to 8.165	No	ns	0.8381	27.13	25.23	1.9	2.519	3	3	0.754	42
AKT1 vs. AKT1 M227Q	-4.4	-10.66 to 1.865	No	ns	0.2415	20.83	25.23	-4.4	2.519	3	3	1.746	42
capivasertib 4 µM													
Parental vs. AKT1	12.33	6.068 to 18.60	Yes	****	<0.0001	26.2	13.87	12.33	2.519	3	3	4.895	42
Parental vs. AKT1 M227Q	-3.133	-9.398 to 3.132	No	ns	0.5264	26.2	29.33	-3.133	2.519	3	3	1.244	42
AKT1 vs. AKT1 M227Q	-15.47	-21.73 to -9.202	Yes	****	<0.0001	13.87	29.33	-15.47	2.519	3	3	6.139	42

Figure 4D

Cell number

DMSO - AZD0156													
DMSO	0.4162	0.1956 to 0.6367	Yes	****	<0.0001	2.064	1.648	0.4162	0.07695	3	3	5.409	24
MK-2206 0.5 µM	0.2477	0.02716 to 0.4682	Yes	*	0.0218	0.8558	0.6081	0.2477	0.07695	3	3	3.219	24
ARQ 751 0.125 µM	0.2102	-0.01028 to 0.4308	No	ns	0.0677	0.7147	0.5044	0.2102	0.07695	3	3	2.732	24
miransertib 0.25 µM	0.2707	0.05020 to 0.4913	Yes	*	0.0105	0.8184	0.5476	0.2707	0.07695	3	3	3.518	24
GSK690693 0.5 µM	0.4104	0.1899 to 0.6309	Yes	***	0.0001	1.173	0.7622	0.4104	0.07695	3	3	5.334	24
ipatasertib 0.5 µM	0.3859	0.1654 to 0.6065	Yes	***	0.0002	1.207	0.8213	0.3859	0.07695	3	3	5.016	24

Cell death

DMSO - AZD0156													
DMSO	-2.433	-8.412 to 3.545	No	ns	0.8288	3.833	6.267	-2.433	2.086	3	3	1.167	24
MK-2206 0.5 µM	-23.33	-29.31 to -17.36	Yes	****	<0.0001	21.03	44.37	-23.33	2.086	3	3	11.19	24
ARQ 751 0.125 µM	-20.5	-26.48 to -14.52	Yes	****	<0.0001	35.57	56.07	-20.5	2.086	3	3	9.828	24
miransertib 0.25 µM	-22.87	-28.84 to -16.89	Yes	****	<0.0001	24.47	47.33	-22.87	2.086	3	3	10.96	24
GSK690693 0.5 µM	-19.73	-25.71 to -13.76	Yes	****	<0.0001	18.73	38.47	-19.73	2.086	3	3	9.46	24
ipatasertib 0.5 µM	-15.6	-21.58 to -9.622	Yes	****	<0.0001	10.97	26.57	-15.6	2.086	3	3	7.479	24

Figure 4E

Cell number

DMSO - KU-57788													
DMSO	0.6471	0.3980 to 0.8961	Yes	****	<0.0001	1.882	1.235	0.6471	0.0869	3	3	7.446	24
MK-2206 0.5 µM	0.1176	-0.1314 to 0.3667	No	ns	0.7142	0.9608	0.8431	0.1176	0.0869	3	3	1.354	24
ARQ 751 0.125 µM	0.04314	-0.2059 to 0.2922	No	ns	0.9972	0.7059	0.6627	0.04314	0.0869	3	3	0.496	24
miransertib 0.25 µM	0.1373	-0.1118 to 0.3863	No	ns	0.5583	0.9216	0.7843	0.1373	0.0869	3	3	1.58	24
GSK690693 0.5 µM	3.333E-07	-0.2490 to 0.2490	No	ns	>0.9999	0.9216	0.9216	3.333E-07	0.0869	3	3	4E-06	24
ipatasertib 0.5 µM	0.1176	-0.1314 to 0.3667	No	ns	0.7142	1.196	1.078	0.1176	0.0869	3	3	1.354	24

Cell death

DMSO - KU-57788													
DMSO	0.2	-7.434 to 7.834	No	ns	>0.9999	4.033	3.833	0.2	2.664	3	3	0.075	24
MK-2206 0.5 µM	-16.7	-24.33 to -9.066	Yes	****	<0.0001	25.3	42	-16.7	2.664	3	3	6.27	24
ARQ 751 0.125 µM	-21.17	-28.80 to -13.53	Yes	****	<0.0001	32.97	54.13	-21.17	2.664	3	3	7.947	24
miransertib 0.25 µM	-20.43	-28.07 to -12.80	Yes	****	<0.0001	21.7	42.13	-20.43	2.664	3	3	7.671	24
GSK690693 0.5 µM	-19.57	-27.20 to -11.93	Yes	****	<0.0001	16.73	36.3	-19.57	2.664	3	3	7.346	24
ipatasertib 0.5 µM	-12.3	-19.93 to -4.666	Yes	***	0.0007	10.3	22.6	-12.3	2.664	3	3	4.618	24

Figure 4F

Cell number

DMSO - GSK2334470													
DMSO	0.552	0.3194 to 0.7846	Yes	****	<0.0001	1.757	1.205	0.552	0.08116	3	3	6.801	24
MK-2206 1 µM	0.3757	0.1430 to 0.6083	Yes	***	0.0006	0.734	0.3583	0.3757	0.08116	3	3	4.628	24
ARQ 751 0.25 µM	0.1917	-0.04095 to 0.4243	No	ns	0.1497	0.6293	0.4377	0.1917	0.08116	3	3	2.361	24
miransertib 0.5 µM	0.2397	0.007048 to 0.4723	Yes	*	0.0409	0.623	0.3833	0.2397	0.08116	3	3	2.953	24
GSK690693 1 µM	0.334	0.1014 to 0.5666	Yes	**	0.0024	0.805	0.471	0.334	0.08116	3	3	4.115	24
ipatasertib 1 µM	0.4613	0.2287 to 0.6940	Yes	****	<0.0001	0.9717	0.5103	0.4613	0.08116	3	3	5.684	24

Cell death

DMSO - GSK2334470													
DMSO	-5.371	-13.19 to 2.452	No	ns	0.3135	5.412	10.78	-5.371	2.73	3	3	1.968	24
MK-2206 1 µM	-29.33	-37.16 to -21.51	Yes	****	<0.0001	39.75	69.08	-29.33	2.73	3	3	10.75	24
ARQ 751 0.25 µM	-18.9	-26.72 to -11.08	Yes	****	<0.0001	45.49	64.39	-18.9	2.73	3	3	6.924	24
miransertib 0.5 µM	-20.96	-28.78 to -13.13	Yes	****	<0.0001	48.21	69.16	-20.96	2.73	3	3	7.677	24
GSK690693 1 µM	-24.59	-32.41 to -16.76	Yes	****	<0.0001	33.89	58.48	-24.59	2.73	3	3	9.007	24
ipatasertib 1 µM	-32.83	-40.66 to -25.01	Yes	****	<0.0001	23.74	56.58	-32.83	2.73	3	3	12.03	24

Figure 4G

Cell number

DMSO - OSU-T315													
DMSO	0.3897	0.2022 to 0.5771	Yes	****	<0.0001								
MK-2206 1 µM	0.4233	0.2359 to 0.6108	Yes	****	<0.0001	1.855	1.465	0.3897	0.06541	3	3	5.958	24
ARQ 751 0.25 µM	0.3337	0.1462 to 0.5211	Yes	***	0.0002	0.794	0.3707	0.4233	0.06541	3	3	6.472	24
miransertib 0.5 µM	0.4383	0.2509 to 0.6258	Yes	****	<0.0001	0.7963	0.4627	0.3337	0.06541	3	3	5.102	24
GSK690693 1 µM	0.2137	0.02621 to 0.4011	Yes	*	0.0194	0.8883	0.45	0.4383	0.06541	3	3	6.702	24
ipatasertib 1 µM	0.395	0.2075 to 0.5825	Yes	****	<0.0001	0.869	0.6553	0.2137	0.06541	3	3	3.267	24
						1.053	0.6577	0.395	0.06541	3	3	6.039	24

Cell death

DMSO - OSU-T315													
DMSO	-4.916	-12.50 to 2.667	No	ns	0.3755	5.322	10.24	-4.916	2.646	3	3	1.858	24
MK-2206 1 µM	-34.02	-41.60 to -26.43	Yes	****	<0.0001	29.56	63.58	-34.02	2.646	3	3	12.86	24
ARQ 751 0.25 µM	-23.62	-31.20 to -16.03	Yes	****	<0.0001	33.69	57.31	-23.62	2.646	3	3	8.926	24
miransertib 0.5 µM	-32.31	-39.89 to -24.72	Yes	****	<0.0001	27.12	59.42	-32.31	2.646	3	3	12.21	24
GSK690693 1 µM	-11.74	-19.33 to -4.161	Yes	**	0.001	32.84	44.59	-11.74	2.646	3	3	4.439	24
ipatasertib 1 µM	-29.19	-36.77 to -21.60	Yes	****	<0.0001	18.02	47.2	-29.19	2.646	3	3	11.03	24

Figure 4H

Cell number

DMSO - Apilimod													
DMSO	1.305	1.144 to 1.466	Yes	****	<0.0001	2.492	1.187	1.305	0.05685	3	3	22.95	20
MK-2206 1 µM	0.04069	-0.1205 to 0.2019	No	ns	0.9629	1.107	1.067	0.04069	0.05685	3	3	0.716	20
ARQ 751 0.25 µM	-0.1268	-0.2880 to 0.03443	No	ns	0.1732	0.9333	1.06	-0.1268	0.05685	3	3	2.231	20
miransertib 0.5 µM	0.0511	-0.1101 to 0.2123	No	ns	0.908	1.028	0.9769	0.0511	0.05685	3	3	0.899	20
ipatasertib 1 µM	0.2243	0.06304 to 0.3855	Yes	**	0.004	1.33	1.106	0.2243	0.05685	3	3	3.945	20

Cell death

DMSO - Apilimod													
DMSO	-9.833	-15.30 to -4.369	Yes	***	0.0003	4.567	14.4	-9.833	1.927	3	3	5.104	20
MK-2206 1 µM	5.867	0.4027 to 11.33	Yes	*	0.0315	25	19.13	5.867	1.927	3	3	3.045	20

ARQ 751 0.25 µM	11.17	5.703 to 16.63	Yes	****	<0.0001	27.93	16.77	11.17	1.927	3	3	5.796	20
miransertib 0.5 µM	3.867	-1.597 to 9.331	No	ns	0.26	22.47	18.6	3.867	1.927	3	3	2.007	20
ipatasertib 1 µM	5.2	-0.2639 to 10.66	No	ns	0.0671	17.63	12.43	5.2	1.927	3	3	2.699	20
**Figure 4I**													
**Cell number**													
DMSO													
DMSO vs. rapamycin	0.3047	0.1967 to 0.4126	Yes	****	<0.0001	1.571	1.266	0.3047	0.04626	3	3	6.586	36
DMSO vs. torin1	0.656	0.5481 to 0.7639	Yes	****	<0.0001	1.571	0.915	0.656	0.04626	3	3	14.18	36
MK-2206 0.5 µM													
DMSO vs. rapamycin	0.261	0.1531 to 0.3689	Yes	****	<0.0001	0.8753	0.6143	0.261	0.04626	3	3	5.642	36
DMSO vs. torin1	0.3987	0.2907 to 0.5066	Yes	****	<0.0001	0.8753	0.4767	0.3987	0.04626	3	3	8.618	36
ARQ 751 0.125 µM													
DMSO vs. rapamycin	0.151	0.04306 to 0.2589	Yes	**	0.0048	0.7747	0.6237	0.151	0.04626	3	3	3.264	36
DMSO vs. torin1	0.287	0.1791 to 0.3949	Yes	****	<0.0001	0.7747	0.4877	0.287	0.04626	3	3	6.204	36
miransertib 0.25 µM													
DMSO vs. rapamycin	0.1103	0.002389 to 0.2183	Yes	*	0.0444	0.8577	0.7473	0.1103	0.04626	3	3	2.385	36
DMSO vs. torin1	0.3897	0.2817 to 0.4976	Yes	****	<0.0001	0.8577	0.468	0.3897	0.04626	3	3	8.424	36
GSK690693 0.5 µM													
DMSO vs. rapamycin	0.2797	0.1717 to 0.3876	Yes	****	<0.0001	0.982	0.7023	0.2797	0.04626	3	3	6.046	36
DMSO vs. torin1	0.4943	0.3864 to 0.6023	Yes	****	<0.0001	0.982	0.4877	0.4943	0.04626	3	3	10.69	36
ipatasertib 0.5 µM													
DMSO vs. rapamycin	0.2107	0.1027 to 0.3186	Yes	***	0.0001	1.035	0.8243	0.2107	0.04626	3	3	4.554	36
DMSO vs. torin1	0.443	0.3351 to 0.5509	Yes	****	<0.0001	1.035	0.592	0.443	0.04626	3	3	9.577	36
**Cell death**													
DMSO													
DMSO vs. rapamycin	-0.9803	-6.334 to 4.373	No	ns	0.8922	2.644	3.625	-0.9803	2.294	3	3	0.427	36
DMSO vs. torin1	-16.14	-21.49 to -10.78	Yes	****	<0.0001	2.644	18.78	-16.14	2.294	3	3	7.033	36
MK-2206 0.5 µM													
DMSO vs. rapamycin	-22.41	-27.77 to -17.06	Yes	****	<0.0001	22.08	44.5	-22.41	2.294	3	3	9.77	36
DMSO vs. torin1	-42.13	-47.49 to -36.78	Yes	****	<0.0001	22.08	64.22	-42.13	2.294	3	3	18.37	36
ARQ 751 0.125 µM													
DMSO vs. rapamycin	-17.33	-22.68 to -11.97	Yes	****	<0.0001	30.66	47.98	-17.33	2.294	3	3	7.553	36
DMSO vs. torin1	-31.24	-36.59 to -25.89	Yes	****	<0.0001	30.66	61.9	-31.24	2.294	3	3	13.62	36
miransertib 0.25 µM													
DMSO vs. rapamycin	-19.91	-25.26 to -14.55	Yes	****	<0.0001	19.32	39.23	-19.91	2.294	3	3	8.677	36
DMSO vs. torin1	-45.12	-50.47 to -39.77	Yes	****	<0.0001	19.32	64.44	-45.12	2.294	3	3	19.67	36
GSK690693 0.5 µM													
DMSO vs. rapamycin	-21.33	-26.68 to -15.97	Yes	****	<0.0001	17.83	39.16	-21.33	2.294	3	3	9.296	36
DMSO vs. torin1	-44.56	-49.91 to -39.20	Yes	****	<0.0001	17.83	62.39	-44.56	2.294	3	3	19.42	36
ipatasertib 0.5 µM													
DMSO vs. rapamycin	-14.19	-19.54 to -8.837	Yes	****	<0.0001	12.79	26.98	-14.19	2.294	3	3	6.185	36
DMSO vs. torin1	-43.13	-48.48 to -37.78	Yes	****	<0.0001	12.79	55.92	-43.13	2.294	3	3	18.8	36
**Figure 5B**													
**Cell number**													
Parental													
DMSO vs. MK-2206 0.5 µM	1.446	1.265 to 1.627	Yes	****	<0.0001	2.61	1.164	1.446	0.07424	3	3	19.48	18
DMSO vs. MK-2206 2 µM	1.656	1.475 to 1.837	Yes	****	<0.0001	2.61	0.9543	1.656	0.07424	3	3	22.31	18
Res +													
DMSO vs. MK-2206 0.5 µM	0.4311	0.2500 to 0.6121	Yes	****	<0.0001	2.582	2.151	0.4311	0.07424	3	3	5.806	18
DMSO vs. MK-2206 2 µM	0.6974	0.5163 to 0.8784	Yes	****	<0.0001	2.582	1.885	0.6974	0.07424	3	3	9.393	18
Res -													
DMSO vs. MK-2206 0.5 µM	0.3758	0.1948 to 0.5569	Yes	***	0.0002	2.318	1.942	0.3758	0.07424	3	3	5.062	18
DMSO vs. MK-2206 2 µM	0.5042	0.3232 to 0.6853	Yes	****	<0.0001	2.318	1.813	0.5042	0.07424	3	3	6.792	18
**Cell death**													
Parental													
DMSO vs. MK-2206 0.5 µM	-24.63	-28.98 to -20.28	Yes	****	<0.0001	3.533	28.17	-24.63	1.784	3	3	13.81	18
DMSO vs. MK-2206 2 µM	-31.93	-36.28 to -27.58	Yes	****	<0.0001	3.533	35.47	-31.93	1.784	3	3	17.9	18
Res +													
DMSO vs. MK-2206 0.5 µM	-1.2	-5.551 to 3.151	No	ns	0.7597	6.333	7.533	-1.2	1.784	3	3	0.673	18
DMSO vs. MK-2206 2 µM	-2.533	-6.885 to 1.818	No	ns	0.3156	6.333	8.867	-2.533	1.784	3	3	1.42	18
Res -													
DMSO vs. MK-2206 0.5 µM	-1.467	-5.818 to 2.885	No	ns	0.6657	5.333	6.8	-1.467	1.784	3	3	0.822	18
DMSO vs. MK-2206 2 µM	-3	-7.351 to 1.351	No	ns	0.2078	5.333	8.333	-3	1.784	3	3	1.681	18
**Figure 5C**													
**Cell number**													
Parental													
DMSO vs. MK-2206 2 µM	1.746	1.409 to 2.084	Yes	****	<0.0001	2.653	0.907	1.746	0.1234	3	3	14.16	20
DMSO vs. ARQ 751 0.5 µM	1.75	1.413 to 2.088	Yes	****	<0.0001	2.653	0.903	1.75	0.1234	3	3	14.19	20
DMSO vs. GSK690693 2 µM	1.288	0.9505 to 1.625	Yes	****	<0.0001	2.653	1.365	1.288	0.1234	3	3	10.44	20
DMSO vs. ipatasertib 2 µM	1.575	1.238 to 1.912	Yes	****	<0.0001	2.653	1.078	1.575	0.1234	3	3	12.77	20
Res													

DMSO vs. MK-2206 2 μM	0.7187	0.3812 to 1.056	Yes	****	<0.0001	2.683	1.965	0.7187	0.1234	3	3	5.825	20
DMSO vs. ARQ 751 0.5 μM	1.104	0.7662 to 1.441	Yes	****	<0.0001	2.683	1.58	1.104	0.1234	3	3	8.946	20
DMSO vs. GSK690693 2 μM	1.048	0.7102 to 1.385	Yes	****	<0.0001	2.683	1.636	1.048	0.1234	3	3	8.492	20
DMSO vs. ipatasertib 2 μM	1.102	0.7645 to 1.439	Yes	****	<0.0001	2.683	1.581	1.102	0.1234	3	3	8.933	20
**Cell death**													
Parental													
DMSO vs. MK-2206 2 μM	-36.82	-42.40 to -31.24	Yes	****	<0.0001	3.44	40.26	-36.82	2.039	3	3	18.06	20
DMSO vs. ARQ 751 0.5 μM	-30.4	-35.98 to -24.82	Yes	****	<0.0001	3.44	33.84	-30.4	2.039	3	3	14.91	20
DMSO vs. GSK690693 2 μM	-20.05	-25.63 to -14.47	Yes	****	<0.0001	3.44	23.49	-20.05	2.039	3	3	9.833	20
DMSO vs. ipatasertib 2 μM	-22.23	-27.81 to -16.66	Yes	****	<0.0001	3.44	25.67	-22.23	2.039	3	3	10.9	20
Res													
DMSO vs. MK-2206 2 μM	-3.693	-9.271 to 1.885	No	ns	0.2996	6.743	10.44	-3.693	2.039	3	3	1.811	20
DMSO vs. ARQ 751 0.5 μM	-16.76	-22.34 to -11.18	Yes	****	<0.0001	6.743	23.5	-16.76	2.039	3	3	8.22	20
DMSO vs. GSK690693 2 μM	-12.95	-18.53 to -7.371	Yes	****	<0.0001	6.743	19.69	-12.95	2.039	3	3	6.351	20
DMSO vs. ipatasertib 2 μM	-15.24	-20.81 to -9.658	Yes	****	<0.0001	6.743	21.98	-15.24	2.039	3	3	7.473	20
**Extra cell death**													
Parental - Res													
DMSO	-3.303	-9.086 to 2.480	No	ns	0.475	3.44	6.743	-3.303	2.039	3	3	1.62	20
MK-2206 2 μM	29.83	24.04 to 35.61	Yes	****	<0.0001	40.26	10.44	29.83	2.039	3	3	14.63	20
ARQ 751 0.5 μM	10.34	4.553 to 16.12	Yes	***	0.0003	33.84	23.5	10.34	2.039	3	3	5.069	20
GSK690693 2 μM	3.797	-1.986 to 9.580	No	ns	0.3313	23.49	19.69	3.797	2.039	3	3	1.862	20
ipatasertib 2 μM	3.694	-2.088 to 9.477	No	ns	0.3588	25.67	21.98	3.694	2.039	3	3	1.812	20
**Figure 5D**													
**Cell number**													
Parental													
MK-2206													
DMSO vs. 0.25 μM	1.207	0.8824 to 1.532	Yes	****	<0.0001	2.466	1.259	1.207	0.1087	3	3	11.1	52
DMSO vs. 0.5 μM	1.393	1.068 to 1.718	Yes	****	<0.0001	2.466	1.074	1.393	0.1087	3	3	12.81	52
DMSO vs. 1 μm	1.555	1.230 to 1.880	Yes	****	<0.0001	2.466	0.9113	1.555	0.1087	3	3	14.3	52
DMSO vs. 2 μM	1.71	1.385 to 2.035	Yes	****	<0.0001	2.466	0.7562	1.71	0.1087	3	3	15.73	52
ARQ 751													
DMSO vs. 0.0625 μM	1.361	1.036 to 1.686	Yes	****	<0.0001	2.466	1.105	1.361	0.1087	3	3	12.52	52
DMSO vs. 0.125 μM	1.518	1.193 to 1.843	Yes	****	<0.0001	2.466	0.9489	1.518	0.1087	3	3	13.96	52
DMSO vs. 0.25 μM	1.524	1.199 to 1.849	Yes	****	<0.0001	2.466	0.9429	1.524	0.1087	3	3	14.01	52
DMSO vs. 0.5 μM	1.661	1.336 to 1.986	Yes	****	<0.0001	2.466	0.8059	1.661	0.1087	3	3	15.27	52
miransentib													
DMSO vs. 0.125 μM	0.9726	0.6476 to 1.298	Yes	****	<0.0001	2.466	1.494	0.9726	0.1087	3	3	8.945	52
DMSO vs. 0.25 μM	1.34	1.015 to 1.665	Yes	****	<0.0001	2.466	1.127	1.34	0.1087	3	3	12.32	52
DMSO vs. 0.5 μM	1.57	1.245 to 1.895	Yes	****	<0.0001	2.466	0.8962	1.57	0.1087	3	3	14.44	52
DMSO vs. 1 μM	1.781	1.456 to 2.106	Yes	****	<0.0001	2.466	0.6854	1.781	0.1087	3	3	16.38	52
Res													
MK-2206													
DMSO vs. 0.25 μM	0.3053	-0.01967 to 0.6303	No	ns	0.0808	3.052	2.747	0.3053	0.1087	3	3	2.808	52
DMSO vs. 0.5 μM	0.6996	0.3746 to 1.025	Yes	****	<0.0001	3.052	2.353	0.6996	0.1087	3	3	6.434	52
DMSO vs. 1 μm	0.7658	0.4408 to 1.091	Yes	****	<0.0001	3.052	2.287	0.7658	0.1087	3	3	7.043	52
DMSO vs. 2 μM	0.8886	0.5636 to 1.214	Yes	****	<0.0001	3.052	2.164	0.8886	0.1087	3	3	8.172	52
ARQ 751													
DMSO vs. 0.0625 μM	0.8934	0.5684 to 1.218	Yes	****	<0.0001	3.052	2.159	0.8934	0.1087	3	3	8.217	52
DMSO vs. 0.125 μM	1.202	0.8770 to 1.527	Yes	****	<0.0001	3.052	1.85	1.202	0.1087	3	3	11.06	52
DMSO vs. 0.25 μM	1.288	0.9627 to 1.613	Yes	****	<0.0001	3.052	1.765	1.288	0.1087	3	3	11.84	52
DMSO vs. 0.5 μM	1.433	1.108 to 1.758	Yes	****	<0.0001	3.052	1.619	1.433	0.1087	3	3	13.18	52
miransentib													
DMSO vs. 0.125 μM	0.2262	-0.09883 to 0.5512	No	ns	0.4059	3.052	2.826	0.2262	0.1087	3	3	2.08	52
DMSO vs. 0.25 μM	0.6818	0.3568 to 1.007	Yes	****	<0.0001	3.052	2.371	0.6818	0.1087	3	3	6.27	52
DMSO vs. 0.5 μM	0.9726	0.6476 to 1.298	Yes	****	<0.0001	3.052	2.08	0.9726	0.1087	3	3	8.945	52
DMSO vs. 1 μM	1.517	1.192 to 1.842	Yes	****	<0.0001	3.052	1.535	1.517	0.1087	3	3	13.95	52
**Cell death**													
Parental													
MK-2206													
DMSO vs. 0.25 μM	-9.8	-14.32 to -5.282	Yes	****	<0.0001	3.733	13.53	-9.8	1.512	3	3	6.483	52
DMSO vs. 0.5 μM	-16.23	-20.75 to -11.71	Yes	****	<0.0001	3.733	19.97	-16.23	1.512	3	3	10.74	52
DMSO vs. 1 μm	-20.5	-25.02 to -15.98	Yes	****	<0.0001	3.733	24.23	-20.5	1.512	3	3	13.56	52
DMSO vs. 2 μM	-30.07	-34.59 to -25.55	Yes	****	<0.0001	3.733	33.8	-30.07	1.512	3	3	19.89	52
ARQ 751													
DMSO vs. 0.0625 μM	-18.63	-23.15 to -14.11	Yes	****	<0.0001	3.733	22.37	-18.63	1.512	3	3	12.33	52
DMSO vs. 0.125 μM	-23.5	-28.02 to -18.98	Yes	****	<0.0001	3.733	27.23	-23.5	1.512	3	3	15.55	52
DMSO vs. 0.25 μM	-24	-28.52 to -19.48	Yes	****	<0.0001	3.733	27.73	-24	1.512	3	3	15.88	52
DMSO vs. 0.5 μM	-33.4	-37.92 to -28.88	Yes	****	<0.0001	3.733	37.13	-33.4	1.512	3	3	22.1	52
miransentib													
DMSO vs. 0.125 μM	-3.567	-8.085 to 0.9518	No	ns	0.2351	3.733	7.3	-3.567	1.512	3	3	2.359	52
DMSO vs. 0.25 μM	-11.77	-16.29 to -7.248	Yes	****	<0.0001	3.733	15.5	-11.77	1.512	3	3	7.784	52
DMSO vs. 0.5 μM	-22.37	-26.89 to -17.85	Yes	****	<0.0001	3.733	26.1	-22.37	1.512	3	3	14.8	52
DMSO vs. 1 μM	-35.43	-39.95 to -30.91	Yes	****	<0.0001	3.733	39.17	-35.43	1.512	3	3	23.44	52
Res													
MK-2206													
DMSO vs. 0.25 μM	-1.067	-5.585 to 3.452	No	ns	0.9996	4.067	5.133	-1.067	1.512	3	3	0.706	52
DMSO vs. 0.5 μM	-2.3	-6.818 to 2.218	No	ns	0.8225	4.067	6.367	-2.3	1.512	3	3	1.522	52
DMSO vs. 1 μm	-1.1	-5.618 to 3.418	No	ns	0.9995	4.067	5.167	-1.1	1.512	3	3	0.728	52
DMSO vs. 2 μM	-1.167	-5.685 to 3.352	No	ns	0.9991	4.067	5.233	-1.167	1.512	3	3	0.772	52
ARQ 751													
DMSO vs. 0.0625 μM	-5.167	-9.685 to -0.6482	Yes	*	0.0147	4.067	9.233	-5.167	1.512	3	3	3.418	52
DMSO vs. 0.125 μM	-8.833	-13.35 to -4.315	Yes	****	<0.0001	4.067	12.9	-8.833	1.512	3	3	5.844	52
DMSO vs. 0.25 μM	-10.97	-15.49 to -6.448	Yes	****	<0.0001	4.067	15.03	-10.97	1.512	3	3	7.255	52
DMSO vs. 0.5 μM	-15.77	-20.29 to -11.25	Yes	****	<0.0001	4.067	19.83	-15.77	1.512	3	3	10.43	52

miransentib														
DMSO vs. 0.125 µM	-0.9667	-5.485 to 3.552	No	ns	0.9999		4.067	5.033	-0.9667	1.512	3	3	0.64	52
DMSO vs. 0.25 µM	-1.933	-6.452 to 2.585	No	ns	0.9378		4.067	6	-1.933	1.512	3	3	1.279	52
DMSO vs. 0.5 µM	-4.067	-8.585 to 0.4518	No	ns	0.1091		4.067	8.133	-4.067	1.512	3	3	2.69	52
DMSO vs. 1 µM	-11.07	-15.59 to -6.548	Yes	****	<0.0001		4.067	15.13	-11.07	1.512	3	3	7.321	52
**Figure 5F**														
**Cell number**														
DMSO par														
DMSO vs. MK2206 1 µM	1.39	1.226 to 1.555	Yes	****	<0.0001		2.278	0.8873	1.39	0.06152	3	3	22.6	48
DMSO vs. ARQ751 0.25 µM	1.363	1.198 to 1.528	Yes	****	<0.0001		2.278	0.9147	1.363	0.06152	3	3	22.16	48
DMSO vs. miransertib 0.5 µM	1.368	1.204 to 1.533	Yes	****	<0.0001		2.278	0.9093	1.368	0.06152	3	3	22.24	48
DMSO vs. GSK690693 1 µM	1.212	1.047 to 1.377	Yes	****	<0.0001		2.278	1.066	1.212	0.06152	3	3	19.7	48
DMSO vs. ipatasertib 1 µM	1.161	0.9965 to 1.326	Yes	****	<0.0001		2.278	1.117	1.161	0.06152	3	3	18.87	48
rapam par														
DMSO vs. MK2206 1 µM	0.6913	0.5268 to 0.8558	Yes	****	<0.0001		1.273	0.5817	0.6913	0.06152	3	3	11.24	48
DMSO vs. ARQ751 0.25 µM	0.6707	0.5062 to 0.8352	Yes	****	<0.0001		1.273	0.6023	0.6707	0.06152	3	3	10.9	48
DMSO vs. miransertib 0.5 µM	0.7137	0.5492 to 0.8782	Yes	****	<0.0001		1.273	0.5593	0.7137	0.06152	3	3	11.6	48
DMSO vs. GSK690693 1 µM	0.6053	0.4408 to 0.7698	Yes	****	<0.0001		1.273	0.6677	0.6053	0.06152	3	3	9.84	48
DMSO vs. ipatasertib 1 µM	0.459	0.2945 to 0.6235	Yes	****	<0.0001		1.273	0.814	0.459	0.06152	3	3	7.462	48
DMSO res														
DMSO vs. MK2206 1 µM	0.3313	0.1668 to 0.4958	Yes	****	<0.0001		2.06	1.728	0.3313	0.06152	3	3	5.386	48
DMSO vs. ARQ751 0.25 µM	1.023	0.8588 to 1.188	Yes	****	<0.0001		2.06	1.036	1.023	0.06152	3	3	16.64	48
DMSO vs. miransertib 0.5 µM	0.7827	0.6182 to 0.9472	Yes	****	<0.0001		2.06	1.277	0.7827	0.06152	3	3	12.72	48
DMSO vs. GSK690693 1 µM	0.7737	0.6092 to 0.9382	Yes	****	<0.0001		2.06	1.286	0.7737	0.06152	3	3	12.58	48
DMSO vs. ipatasertib 1 µM	0.7237	0.5592 to 0.8882	Yes	****	<0.0001		2.06	1.336	0.7237	0.06152	3	3	11.76	48
rapam res														
DMSO vs. MK2206 1 µM	0.189	0.02449 to 0.3535	Yes	*	0.0173		1.286	1.097	0.189	0.06152	3	3	3.072	48
DMSO vs. ARQ751 0.25 µM	0.6543	0.4898 to 0.8188	Yes	****	<0.0001		1.286	0.632	0.6543	0.06152	3	3	10.64	48
DMSO vs. miransertib 0.5 µM	0.544	0.3795 to 0.7085	Yes	****	<0.0001		1.286	0.7423	0.544	0.06152	3	3	8.843	48
DMSO vs. GSK690693 1 µM	0.4343	0.2698 to 0.5988	Yes	****	<0.0001		1.286	0.852	0.4343	0.06152	3	3	7.061	48
DMSO vs. ipatasertib 1 µM	0.3733	0.2088 to 0.5378	Yes	****	<0.0001		1.286	0.913	0.3733	0.06152	3	3	6.069	48
**Cell death**														
DMSO par														
DMSO vs. MK2206 1 µM	-25.97	-32.03 to -19.90	Yes	****	<0.0001		5.341	31.31	-25.97	2.267	3	3	11.45	48
DMSO vs. ARQ751 0.25 µM	-27.68	-33.74 to -21.61	Yes	****	<0.0001		5.341	33.02	-27.68	2.267	3	3	12.21	48
DMSO vs. miransertib 0.5 µM	-27.59	-33.65 to -21.52	Yes	****	<0.0001		5.341	32.93	-27.59	2.267	3	3	12.17	48
DMSO vs. GSK690693 1 µM	-21.77	-27.84 to -15.71	Yes	****	<0.0001		5.341	27.11	-21.77	2.267	3	3	9.603	48
DMSO vs. ipatasertib 1 µM	-17.54	-23.60 to -11.47	Yes	****	<0.0001		5.341	22.88	-17.54	2.267	3	3	7.735	48
rapam par														
DMSO vs. MK2206 1 µM	-45.68	-51.75 to -39.62	Yes	****	<0.0001		7.472	53.16	-45.68	2.267	3	3	20.15	48
DMSO vs. ARQ751 0.25 µM	-44.32	-50.38 to -38.25	Yes	****	<0.0001		7.472	51.79	-44.32	2.267	3	3	19.55	48
DMSO vs. miransertib 0.5 µM	-47	-53.07 to -40.94	Yes	****	<0.0001		7.472	54.47	-47	2.267	3	3	20.73	48
DMSO vs. GSK690693 1 µM	-40.68	-46.74 to -34.61	Yes	****	<0.0001		7.472	48.15	-40.68	2.267	3	3	17.94	48
DMSO vs. ipatasertib 1 µM	-26.7	-32.76 to -20.63	Yes	****	<0.0001		7.472	34.17	-26.7	2.267	3	3	11.77	48
DMSO res														
DMSO vs. MK2206 1 µM	-2.839	-8.903 to 3.224	No	ns	0.7048		4.642	7.481	-2.839	2.267	3	3	1.252	48
DMSO vs. ARQ751 0.25 µM	-14.72	-20.78 to -8.653	Yes	****	<0.0001		4.642	19.36	-14.72	2.267	3	3	6.491	48
DMSO vs. miransertib 0.5 µM	-8.014	-14.08 to -1.951	Yes	**	0.0046		4.642	12.66	-8.014	2.267	3	3	3.535	48
DMSO vs. GSK690693 1 µM	-11.98	-18.04 to -5.912	Yes	****	<0.0001		4.642	16.62	-11.98	2.267	3	3	5.282	48
DMSO vs. ipatasertib 1 µM	-10.86	-16.92 to -4.793	Yes	****	<0.0001		4.642	15.5	-10.86	2.267	3	3	4.788	48
rapam res														
DMSO vs. MK2206 1 µM	-5.976	-12.04 to 0.08759	No	ns	0.0551		5.068	11.04	-5.976	2.267	3	3	2.636	48
DMSO vs. ARQ751 0.25 µM	-33.51	-39.57 to -27.45	Yes	****	<0.0001		5.068	38.58	-33.51	2.267	3	3	14.78	48
DMSO vs. miransertib 0.5 µM	-32.49	-38.55 to -26.42	Yes	****	<0.0001		5.068	37.55	-32.49	2.267	3	3	14.33	48
DMSO vs. GSK690693 1 µM	-32.35	-38.41 to -26.28	Yes	****	<0.0001		5.068	37.42	-32.35	2.267	3	3	14.27	48
DMSO vs. ipatasertib 1 µM	-20.88	-26.94 to -14.82	Yes	****	<0.0001		5.068	25.95	-20.88	2.267	3	3	9.208	48
**Extra cell death**														
DMSO res - rapam res														
DMSO	-0.4257	-6.986 to 6.135	No	ns	>0.9999		4.642	5.068	-0.4257	2.289	3	3	0.186	24
MK2206 1 µM	-3.562	-10.12 to 2.998	No	ns	0.5745		7.481	11.04	-3.562	2.289	3	3	1.556	24
ARQ751 0.25 µM	-19.22	-25.78 to -12.66	Yes	****	<0.0001		19.36	38.58	-19.22	2.289	3	3	8.396	24
miransertib 0.5 µM	-24.9	-31.46 to -18.34	Yes	****	<0.0001		12.66	37.55	-24.9	2.289	3	3	10.88	24
GSK690693 1 µM	-20.8	-27.36 to -14.24	Yes	****	<0.0001		16.62	37.42	-20.8	2.289	3	3	9.086	24
ipatasertib 1 µM	-10.45	-17.01 to -3.887	Yes	***	0.0008		15.5	25.95	-10.45	2.289	3	3	4.564	24
**Figure 6A**														
Parental vs. AKT2	83.66	29.47 to 137.8	Yes	**	0.007	A-B	150.9	67.27	83.66	16.55	3	3	5.054	6
Parental vs. AKT2 K181M	61.59	7.400 to 115.8	Yes	*	0.0292	A-C	150.9	89.34	61.59	16.55	3	3	3.721	6
AKT2 vs. AKT2 K181M	-22.07	-76.26 to 32.12	No	ns	0.545	B-C	67.27	89.34	-22.07	16.55	3	3	1.333	6
**Figure 6B**														
DMSO - MK-2206 2 µM														
Parental	4.196	-58.06 to 66.46	No	ns	0.997		177.3	173.1	4.196	22.47	3	3	0.187	12
AKT2	-121.1	-183.4 to -58.83	Yes	***	0.0005		81.83	202.9	-121.1	22.47	3	3	5.388	12
AKT2 W80A	-13.71	-75.97 to 48.54	No	ns	0.9107		110.5	124.2	-13.71	22.47	3	3	0.61	12
**Extra**														
DMSO														
Parental vs. AKT2	95.51	33.25 to 157.8	Yes	**	0.0034		177.3	81.83	95.51	22.47	3	3	4.25	12
Parental vs. AKT2 W80A	66.82	4.562 to 129.1	Yes	*	0.0345		177.3	110.5	66.82	22.47	3	3	2.973	12
AKT2 vs. AKT2 W80A	-28.69	-90.95 to 33.57	No	ns	0.5362		81.83	110.5	-28.69	22.47	3	3	1.277	12

MK-2206 2 µM														
Parental vs. AKT2	-29.78	-92.04 to 32.48	No	ns	0.5066		173.1	202.9	-29.78	22.47	3	3	1.325	12
Parental vs. AKT2 W80A	48.91	-13.35 to 111.2	No	ns	0.1432		173.1	124.2	48.91	22.47	3	3	2.176	12
AKT2 vs. AKT2 W80A	78.69	16.43 to 140.9	Yes	*	0.0131		202.9	124.2	78.69	22.47	3	3	3.501	12
**Figure 6C**														
MDA-MB-361 DMSO vs. MDA-MB-361 MK-2	-203.2	-275.8 to -130.6	Yes	***	0.0003	A-B	8.983	212.2	-203.2	22.17	3	3	9.167	6
MDA-MB-361 DMSO vs. MDA-MB-361 GSK	-93.35	-165.9 to -20.79	Yes	*	0.0167	A-C	8.983	102.3	-93.35	22.17	3	3	4.212	6
MDA-MB-361 MK-2206 vs. MDA-MB-361 GS	109.8	37.27 to 182.4	Yes	**	0.0077	B-C	212.2	102.3	109.8	22.17	3	3	4.955	6
EBC1 DMSO vs. EBC1 MK-2206	-133	-164.5 to -101.6	Yes	****	<0.0001	D-E	45.77	178.8	-133	9.594	3	3	13.87	6
EBC1 DMSO vs. EBC1 GSK690693	1.316	-30.09 to 32.72	No	ns	0.9989	D-F	45.77	44.45	1.316	9.594	3	3	0.137	6
EBC1 MK-2206 vs. EBC1 GSK690693	134.4	103.0 to 165.8	Yes	****	<0.0001	E-F	178.8	44.45	134.4	9.594	3	3	14.01	6
**Figure 6D**														
DMSO vs. MK-2206 2 µM	-140.5	-207.3 to -73.73	Yes	****	<0.0001	A-B	56.52	197	-140.5	18.5	3	3	7.594	14
DMSO vs. ARQ 751 0.5 µM	-133.9	-200.7 to -67.11	Yes	****	<0.0001	A-C	56.52	190.4	-133.9	18.5	3	3	7.236	14
DMSO vs. miransertib 1 µM	-143.2	-210.0 to -76.45	Yes	****	<0.0001	A-D	56.52	199.8	-143.2	18.5	3	3	7.741	14
DMSO vs. GSK606093 2 µM	-52.08	-118.9 to 14.70	No	ns	0.2209	A-E	56.52	108.6	-52.08	18.5	3	3	2.815	14
DMSO vs. ipatasertib 2 µM	-61.63	-128.4 to 5.147	No	ns	0.0854	A-F	56.52	118.2	-61.63	18.5	3	3	3.331	14
DMSO vs. capivasertib 4 µM	-58.62	-125.4 to 8.156	No	ns	0.1162	A-G	56.52	115.1	-58.62	18.5	3	3	3.168	14
MK-2206 2 µM vs. GSK606093 2 µM	88.43	21.65 to 155.2	Yes	**	0.0053	B-E	197	108.6	88.43	18.5	3	3	4.779	14
MK-2206 2 µM vs. ipatasertib 2 µM	78.87	12.09 to 145.7	Yes	*	0.0141	B-F	197	118.2	78.87	18.5	3	3	4.263	14
MK-2206 2 µM vs. capivasertib 4 µM	81.88	15.10 to 148.7	Yes	*	0.0103	B-G	197	115.1	81.88	18.5	3	3	4.426	14
ARQ 751 0.5 µM vs. GSK606093 2 µM	81.81	15.03 to 148.6	Yes	*	0.0104	C-E	190.4	108.6	81.81	18.5	3	3	4.422	14
ARQ 751 0.5 µM vs. ipatasertib 2 µM	72.26	5.476 to 139.0	Yes	*	0.0282	C-F	190.4	118.2	72.26	18.5	3	3	3.905	14
ARQ 751 0.5 µM vs. capivasertib 4 µM	75.27	8.485 to 142.0	Yes	*	0.0205	C-G	190.4	115.1	75.27	18.5	3	3	4.068	14
miransertib 1 µM vs. GSK606093 2 µM	91.15	24.37 to 157.9	Yes	**	0.004	D-E	199.8	108.6	91.15	18.5	3	3	4.927	14
miransertib 1 µM vs. ipatasertib 2 µM	81.6	14.82 to 148.4	Yes	*	0.0106	D-F	199.8	118.2	81.6	18.5	3	3	4.41	14
miransertib 1 µM vs. capivasertib 4 µM	84.61	17.83 to 151.4	Yes	**	0.0078	D-G	199.8	115.1	84.61	18.5	3	3	4.573	14
MK-2206 2 µM vs. ARQ 751 0.5 µM	6.618	-60.16 to 73.40	No	ns	>0.9999	B-C	197	190.4	6.618	18.5	3	3	0.358	14
MK-2206 2 µM vs. miransertib 1 µM	-2.728	-69.51 to 64.05	No	ns	>0.9999	B-D	197	199.8	-2.728	18.5	3	3	0.147	14
ARQ 751 0.5 µM vs. miransertib 1 µM	-9.346	-76.13 to 57.43	No	ns	>0.9999	C-D	190.4	199.8	-9.346	18.5	3	3	0.505	14
**Figure 6E**														
AKT1														
DMSO vs. MK-2206 2 µM	-103.3	-151.3 to -55.36	Yes	****	<0.0001		69.06	172.4	-103.3	18	3	3	5.741	16
DMSO vs. ARQ 751 0.5 µM	-124.5	-172.4 to -76.49	Yes	****	<0.0001		69.06	193.5	-124.5	18	3	3	6.915	16
DMSO vs. miransertib 1 µM	-71.03	-119.0 to -23.07	Yes	**	0.0035		69.06	140.1	-71.03	18	3	3	3.947	16
AKT1 Q79K														
DMSO vs. MK-2206 2 µM	-110	-157.9 to -62.03	Yes	****	<0.0001		59.3	169.3	-110	18	3	3	6.111	16
DMSO vs. ARQ 751 0.5 µM	-47.23	-95.19 to 0.7278	No	ns	0.0542		59.3	106.5	-47.23	18	3	3	2.624	16
DMSO vs. miransertib 1 µM	-13.17	-61.13 to 34.79	No	ns	0.8552		59.3	72.47	-13.17	18	3	3	0.732	16
**Extra**														
AKT1 - AKT1 Q79K														
DMSO	9.758	-40.70 to 60.22	No	ns	0.9731		69.06	59.3	9.758	18	3	3	0.542	16
MK-2206 2 µM	3.089	-47.37 to 53.55	No	ns	0.9997		172.4	169.3	3.089	18	3	3	0.172	16
ARQ 751 0.5 µM	86.98	36.52 to 137.4	Yes	***	0.0007		193.5	106.5	86.98	18	3	3	4.833	16
miransertib 1 µM	67.62	17.16 to 118.1	Yes	**	0.0069		140.1	72.47	67.62	18	3	3	3.757	16
**Figure 6F**														
Parental														
DMSO vs. MK-2206 2 µM	-325.7	-404.9 to -246.6	Yes	****	<0.0001		97.81	423.6	-325.7	28.4	3	3	11.47	24
DMSO vs. ARQ 751 0.5 µM	-402.3	-481.5 to -323.1	Yes	****	<0.0001		97.81	500.1	-402.3	28.4	3	3	14.17	24
DMSO vs. miransertib 1 µM	-203.1	-282.3 to -123.9	Yes	****	<0.0001		97.81	300.9	-203.1	28.4	3	3	7.15	24
DMSO vs. GSK690693 2 µM	-125	-204.2 to -45.82	Yes	***	0.001		97.81	222.8	-125	28.4	3	3	4.401	24
DMSO vs. ipatasertib 2 µM	-89.85	-169.0 to -10.66	Yes	*	0.0208		97.81	187.7	-89.85	28.4	3	3	3.163	24
Res														
DMSO vs. MK-2206 2 µM	-53.04	-132.2 to 26.16	No	ns	0.3196		116.9	169.9	-53.04	28.4	3	3	1.867	24
DMSO vs. ARQ 751 0.5 µM	-281.5	-360.7 to -202.3	Yes	****	<0.0001		116.9	398.4	-281.5	28.4	3	3	9.91	24
DMSO vs. miransertib 1 µM	-90.88	-170.1 to -11.69	Yes	*	0.0191		116.9	207.8	-90.88	28.4	3	3	3.2	24
DMSO vs. GSK690693 2 µM	-55.91	-135.1 to 23.28	No	ns	0.2687		116.9	172.8	-55.91	28.4	3	3	1.968	24
DMSO vs. ipatasertib 2 µM	-122	-201.2 to -42.86	Yes	**	0.0012		116.9	238.9	-122	28.4	3	3	4.297	24
**Extra**														
Parental - Res														
DMSO	-19.09	-100.5 to 62.31	No	ns	0.9858		97.81	116.9	-19.09	28.4	3	3	0.672	24
MK-2206 2 µM	253.6	172.2 to 335.0	Yes	****	<0.0001		423.6	169.9	253.6	28.4	3	3	8.929	24
ARQ 751 0.5 µM	101.8	20.36 to 183.2	Yes	**	0.009		500.1	398.4	101.8	28.4	3	3	3.583	24
miransertib 1 µM	93.1	11.70 to 174.5	Yes	*	0.0189		300.9	207.8	93.1	28.4	3	3	3.278	24
GSK690693 2 µM	50.01	-31.40 to 131.4	No	ns	0.436		222.8	172.8	50.01	28.4	3	3	1.761	24
ipatasertib 2 µM	-51.29	-132.7 to 30.11	No	ns	0.4074		187.7	238.9	-51.29	28.4	3	3	1.806	24
**Supplamentary figure 2**														
**Cell number**														
Parental														
DMSO vs. ARQ 751 0.125 µM	1.077	0.8576 to 1.296	Yes	****	<0.0001		1.607	0.5297	1.077	0.08545	3	3	12.6	60
DMSO vs. ARQ 751 0.5 µM	1.082	0.8623 to 1.301	Yes	****	<0.0001		1.607	0.525	1.082	0.08545	3	3	12.66	60
DMSO vs. ipatasertib 0.5 µM	0.706	0.4866 to 0.9254	Yes	****	<0.0001		1.607	0.9007	0.706	0.08545	3	3	8.262	60
DMSO vs. ipatasertib 2 µM	0.9437	0.7243 to 1.163	Yes	****	<0.0001		1.607	0.663	0.9437	0.08545	3	3	11.04	60
AKT1														
DMSO vs. ARQ 751 0.125 µM	1.006	0.7863 to 1.225	Yes	****	<0.0001		1.801	0.7957	1.006	0.08545	3	3	11.77	60
DMSO vs. ARQ 751 0.5 µM	1.244	1.024 to 1.463	Yes	****	<0.0001		1.801	0.5577	1.244	0.08545	3	3	14.55	60
DMSO vs. ipatasertib 0.5 µM	0.5773	0.3579 to 0.7967	Yes	****	<0.0001		1.801	1.224	0.5773	0.08545	3	3	6.757	60
DMSO vs. ipatasertib 2 µM	0.997	0.7776 to 1.216	Yes	****	<0.0001		1.801	0.8043	0.997	0.08545	3	3	11.67	60
AKT1 W80A														

DMSO vs. ARQ 751 0.125 µM	0.625	0.4056 to 0.8444	Yes	****	<0.0001	1.909	1.284	0.625	0.08545	3	3	7.314	60
DMSO vs. ARQ 751 0.5 µM	1.048	0.8283 to 1.267	Yes	****	<0.0001	1.909	0.8617	1.048	0.08545	3	3	12.26	60
DMSO vs. ipatasertib 0.5 µM	0.8753	0.6559 to 1.095	Yes	****	<0.0001	1.909	1.034	0.8753	0.08545	3	3	10.24	60
DMSO vs. ipatasertib 2 µM	1.107	0.8873 to 1.326	Yes	****	<0.0001	1.909	0.8027	1.107	0.08545	3	3	12.95	60
AKT1 Q79K													
DMSO vs. ARQ 751 0.125 µM	0.139	-0.08042 to 0.3584	No	ns	0.3698	1.93	1.791	0.139	0.08545	3	3	1.627	60
DMSO vs. ARQ 751 0.5 µM	0.677	0.4576 to 0.8964	Yes	****	<0.0001	1.93	1.253	0.677	0.08545	3	3	7.923	60
DMSO vs. ipatasertib 0.5 µM	0.822	0.6026 to 1.041	Yes	****	<0.0001	1.93	1.108	0.822	0.08545	3	3	9.62	60
DMSO vs. ipatasertib 2 µM	1.242	1.023 to 1.462	Yes	****	<0.0001	1.93	0.6877	1.242	0.08545	3	3	14.54	60
AKT2													
DMSO vs. ARQ 751 0.125 µM	1.178	0.9589 to 1.398	Yes	****	<0.0001	1.953	0.7747	1.178	0.08545	3	3	13.79	60
DMSO vs. ARQ 751 0.5 µM	1.392	1.173 to 1.612	Yes	****	<0.0001	1.953	0.5607	1.392	0.08545	3	3	16.29	60
DMSO vs. ipatasertib 0.5 µM	0.5727	0.3533 to 0.7921	Yes	****	<0.0001	1.953	1.38	0.5727	0.08545	3	3	6.702	60
DMSO vs. ipatasertib 2 µM	1.011	0.7916 to 1.230	Yes	****	<0.0001	1.953	0.942	1.011	0.08545	3	3	11.83	60
AKT2 W80A													
DMSO vs. ARQ 751 0.125 µM	0.292	0.07258 to 0.5114	Yes	**	0.0046	2.066	1.774	0.292	0.08545	3	3	3.417	60
DMSO vs. ARQ 751 0.5 µM	0.4283	0.2089 to 0.6477	Yes	****	<0.0001	2.066	1.637	0.4283	0.08545	3	3	5.013	60
DMSO vs. ipatasertib 0.5 µM	0.4673	0.2479 to 0.6867	Yes	****	<0.0001	2.066	1.598	0.4673	0.08545	3	3	5.469	60
DMSO vs. ipatasertib 2 µM	0.9347	0.7153 to 1.154	Yes	****	<0.0001	2.066	1.131	0.9347	0.08545	3	3	10.94	60
**Cell death**													
Parental													
DMSO vs. ARQ 751 0.125 µM	-46.61	-53.19 to -40.03	Yes	****	<0.0001	5.324	51.93	-46.61	2.562	3	3	18.2	60
DMSO vs. ARQ 751 0.5 µM	-49	-55.58 to -42.42	Yes	****	<0.0001	5.324	54.33	-49	2.562	3	3	19.13	60
DMSO vs. ipatasertib 0.5 µM	-17.2	-23.78 to -10.62	Yes	****	<0.0001	5.324	22.53	-17.2	2.562	3	3	6.715	60
DMSO vs. ipatasertib 2 µM	-33.87	-40.45 to -27.29	Yes	****	<0.0001	5.324	39.19	-33.87	2.562	3	3	13.22	60
AKT1													
DMSO vs. ARQ 751 0.125 µM	-34.05	-40.63 to -27.47	Yes	****	<0.0001	6.137	40.19	-34.05	2.562	3	3	13.29	60
DMSO vs. ARQ 751 0.5 µM	-45.55	-52.13 to -38.98	Yes	****	<0.0001	6.137	51.69	-45.55	2.562	3	3	17.78	60
DMSO vs. ipatasertib 0.5 µM	-6.738	-13.32 to -0.1605	Yes	*	0.0426	6.137	12.88	-6.738	2.562	3	3	2.631	60
DMSO vs. ipatasertib 2 µM	-30.48	-37.06 to -23.91	Yes	****	<0.0001	6.137	36.62	-30.48	2.562	3	3	11.9	60
****													
AKT1 W80A													
DMSO vs. ARQ 751 0.125 µM	-4.112	-10.69 to 2.466	No	ns	0.383	7.586	11.7	-4.112	2.562	3	3	1.605	60
DMSO vs. ARQ 751 0.5 µM	-24.85	-31.42 to -18.27	Yes	****	<0.0001	7.586	32.43	-24.85	2.562	3	3	9.699	60
DMSO vs. ipatasertib 0.5 µM	-7.809	-14.39 to -1.232	Yes	*	0.0136	7.586	15.4	-7.809	2.562	3	3	3.049	60
DMSO vs. ipatasertib 2 µM	-33.68	-40.26 to -27.11	Yes	****	<0.0001	7.586	41.27	-33.68	2.562	3	3	13.15	60
AKT1 Q79K													
DMSO vs. ARQ 751 0.125 µM	0.9997	-5.578 to 7.577	No	ns	0.9917	6.144	5.144	0.9997	2.562	3	3	0.39	60
DMSO vs. ARQ 751 0.5 µM	-3.531	-10.11 to 3.047	No	ns	0.5328	6.144	9.675	-3.531	2.562	3	3	1.378	60
DMSO vs. ipatasertib 0.5 µM	-10.27	-16.85 to -3.691	Yes	***	0.0007	6.144	16.41	-10.27	2.562	3	3	4.009	60
DMSO vs. ipatasertib 2 µM	-40.11	-46.69 to -33.53	Yes	****	<0.0001	6.144	46.26	-40.11	2.562	3	3	15.66	60
AKT2													
DMSO vs. ARQ 751 0.125 µM	-36.67	-43.25 to -30.09	Yes	****	<0.0001	7.774	44.44	-36.67	2.562	3	3	14.31	60
DMSO vs. ARQ 751 0.5 µM	-48.87	-55.45 to -42.29	Yes	****	<0.0001	7.774	56.64	-48.87	2.562	3	3	19.08	60
DMSO vs. ipatasertib 0.5 µM	-2.807	-9.385 to 3.771	No	ns	0.7276	7.774	10.58	-2.807	2.562	3	3	1.096	60
DMSO vs. ipatasertib 2 µM	-25.82	-32.40 to -19.24	Yes	****	<0.0001	7.774	33.6	-25.82	2.562	3	3	10.08	60
AKT2 W80A													
DMSO vs. ARQ 751 0.125 µM	-0.122	-6.700 to 6.456	No	ns	>0.9999	4.965	5.087	-0.122	2.562	3	3	0.048	60
DMSO vs. ARQ 751 0.5 µM	-1.638	-8.215 to 4.940	No	ns	0.9491	4.965	6.603	-1.638	2.562	3	3	0.639	60
DMSO vs. ipatasertib 0.5 µM	-2.268	-8.846 to 4.310	No	ns	0.8517	4.965	7.233	-2.268	2.562	3	3	0.885	60
DMSO vs. ipatasertib 2 µM	-13.86	-20.44 to -7.285	Yes	****	<0.0001	4.965	18.83	-13.86	2.562	3	3	5.412	60
**Supplementary figure 3A**													
**Cell number**													
DMSO													
DMSO vs. MK-2206 2 µM	6.775	6.304 to 7.245	Yes	****	<0.0001	7.55	0.7748	6.775	0.1687	3	3	40.15	24
DMSO vs. ARQ 751 0.5 µM	6.502	6.031 to 6.972	Yes	****	<0.0001	7.55	1.048	6.502	0.1687	3	3	38.53	24
DMSO vs. miransertib 1 µM	6.76	6.289 to 7.230	Yes	****	<0.0001	7.55	0.7901	6.76	0.1687	3	3	40.06	24
DMSO vs. GSK630393 2 µM	2.932	2.462 to 3.403	Yes	****	<0.0001	7.55	4.617	2.932	0.1687	3	3	17.38	24
DMSO vs. ipatasertib 2 µM	6.642	6.172 to 7.112	Yes	****	<0.0001	7.55	0.9078	6.642	0.1687	3	3	39.36	24
AZD0156													
DMSO vs. MK-2206 2 µM	2.23	1.760 to 2.700	Yes	****	<0.0001	2.89	0.6599	2.23	0.1687	3	3	13.22	24
DMSO vs. ARQ 751 0.5 µM	2.118	1.647 to 2.588	Yes	****	<0.0001	2.89	0.7723	2.118	0.1687	3	3	12.55	24
DMSO vs. miransertib 1 µM	2.266	1.795 to 2.736	Yes	****	<0.0001	2.89	0.6243	2.266	0.1687	3	3	13.43	24
DMSO vs. GSK630393 2 µM	0.06115	-0.4093 to 0.5316	No	ns	0.9983	2.89	2.829	0.06115	0.1687	3	3	0.362	24
DMSO vs. ipatasertib 2 µM	2.117	1.647 to 2.588	Yes	****	<0.0001	2.89	0.7725	2.117	0.1687	3	3	12.55	24
**Cell death**													
DMSO													
DMSO vs. MK-2206 2 µM	-32.77	-38.65 to -26.88	Yes	****	<0.0001	7.233	40	-32.77	2.111	3	3	15.52	24
DMSO vs. ARQ 751 0.5 µM	-25.57	-31.45 to -19.68	Yes	****	<0.0001	7.233	32.8	-25.57	2.111	3	3	12.11	24
DMSO vs. miransertib 1 µM	-40.23	-46.12 to -34.35	Yes	****	<0.0001	7.233	47.47	-40.23	2.111	3	3	19.06	24
DMSO vs. GSK630393 2 µM	-3.467	-9.353 to 2.420	No	ns	0.4529	7.233	10.7	-3.467	2.111	3	3	1.642	24
DMSO vs. ipatasertib 2 µM	-33.27	-39.15 to -27.38	Yes	****	<0.0001	7.233	40.5	-33.27	2.111	3	3	15.76	24
AZD0156													
DMSO vs. MK-2206 2 µM	-32.5	-38.39 to -26.61	Yes	****	<0.0001	16.1	48.6	-32.5	2.111	3	3	15.39	24
DMSO vs. ARQ 751 0.5 µM	-23.37	-29.25 to -17.48	Yes	****	<0.0001	16.1	39.47	-23.37	2.111	3	3	11.07	24
DMSO vs. miransertib 1 µM	-38.73	-44.62 to -32.85	Yes	****	<0.0001	16.1	54.83	-38.73	2.111	3	3	18.35	24
DMSO vs. GSK630393 2 µM	4.633	-1.253 to 10.52	No	ns	0.1766	16.1	11.47	4.633	2.111	3	3	2.195	24
DMSO vs. ipatasertib 2 µM	-24.53	-30.42 to -18.65	Yes	****	<0.0001	16.1	40.63	-24.53	2.111	3	3	11.62	24
**extra**													

DMSO - AZD0156													
DMSO	-8.867	-14.92 to -2.816	Yes	**	0.0019	7.233	16.1	-8.867	2.111	3	3	4.2	24
MK-2206 2 µM	-8.6	-14.65 to -2.549	Yes	**	0.0026	40	48.6	-8.6	2.111	3	3	4.073	24
ARQ 751 0.5 µM	-6.667	-12.72 to -0.6159	Yes	*	0.0253	32.8	39.47	-6.667	2.111	3	3	3.158	24
miransertib 1 µM	-7.367	-13.42 to -1.316	Yes	*	0.0113	47.47	54.83	-7.367	2.111	3	3	3.489	24
GSK630393 2 µM	-0.7667	-6.817 to 5.284	No	ns	0.9995	10.7	11.47	-0.7667	2.111	3	3	0.363	24
ipatasertib 2 µM	-0.1333	-6.184 to 5.917	No	ns	>0.9999	40.5	40.63	-0.1333	2.111	3	3	0.063	24
**Supplementary figure 3C**													
**Cell number**													
DMSO													
DMSO vs. miransertib 1 µM	0.7438	0.5819 to 0.9058	Yes	****	<0.0001	1.408	0.6642	0.7438	0.06881	3	3	10.81	30
DMSO vs. ipatasertib 2 µM	0.5796	0.4176 to 0.7416	Yes	****	<0.0001	1.408	0.8284	0.5796	0.06881	3	3	8.424	30
AZD0156													
DMSO vs. miransertib 1 µM	0.9289	0.7669 to 1.091	Yes	****	<0.0001	1.434	0.5054	0.9289	0.06881	3	3	13.5	30
DMSO vs. ipatasertib 2 µM	0.5976	0.4356 to 0.7595	Yes	****	<0.0001	1.434	0.8367	0.5976	0.06881	3	3	8.684	30
apilimod													
DMSO vs. miransertib 1 µM	0.4278	0.2658 to 0.5898	Yes	****	<0.0001	1.236	0.8077	0.4278	0.06881	3	3	6.217	30
DMSO vs. ipatasertib 2 µM	0.345	0.1830 to 0.5070	Yes	****	<0.0001	1.236	0.8905	0.345	0.06881	3	3	5.014	30
KU-57788													
DMSO vs. miransertib 1 µM	0.5245	0.3626 to 0.6865	Yes	****	<0.0001	0.9899	0.4654	0.5245	0.06881	3	3	7.623	30
DMSO vs. ipatasertib 2 µM	0.2829	0.1209 to 0.4449	Yes	***	0.0006	0.9899	0.707	0.2829	0.06881	3	3	4.112	30
GSK2234470													
DMSO vs. miransertib 1 µM	0.6751	0.5131 to 0.8371	Yes	****	<0.0001	1.077	0.4018	0.6751	0.06881	3	3	9.811	30
DMSO vs. ipatasertib 2 µM	0.3257	0.1637 to 0.4877	Yes	****	<0.0001	1.077	0.7512	0.3257	0.06881	3	3	4.733	30
**Cell death**													
DMSO													
DMSO vs. miransertib 1 µM	-27.87	-33.87 to -21.86	Yes	****	<0.0001	8.533	36.4	-27.87	2.55	3	3	10.93	30
DMSO vs. ipatasertib 2 µM	-12.83	-18.84 to -6.831	Yes	****	<0.0001	8.533	21.37	-12.83	2.55	3	3	5.033	30
AZD0156													
DMSO vs. miransertib 1 µM	-42.6	-48.60 to -36.60	Yes	****	<0.0001	7.1	49.7	-42.6	2.55	3	3	16.71	30
DMSO vs. ipatasertib 2 µM	-16.37	-22.37 to -10.36	Yes	****	<0.0001	7.1	23.47	-16.37	2.55	3	3	6.418	30
apilimod													
DMSO vs. miransertib 1 µM	-3.1	-9.103 to 2.903	No	ns	0.4126	16.57	19.67	-3.1	2.55	3	3	1.216	30
DMSO vs. ipatasertib 2 µM	-1.933	-7.936 to 4.069	No	ns	0.7022	16.57	18.5	-1.933	2.55	3	3	0.758	30
KU-57788													
DMSO vs. miransertib 1 µM	-28.8	-34.80 to -22.80	Yes	****	<0.0001	20.37	49.17	-28.8	2.55	3	3	11.29	30
DMSO vs. ipatasertib 2 µM	-6.433	-12.44 to -0.4307	Yes	*	0.0341	20.37	26.8	-6.433	2.55	3	3	2.523	30
GSK2234470													
DMSO vs. miransertib 1 µM	-37.13	-43.14 to -31.13	Yes	****	<0.0001	14.27	51.4	-37.13	2.55	3	3	14.56	30
DMSO vs. ipatasertib 2 µM	-8.867	-14.87 to -2.864	Yes	**	0.0031	14.27	23.13	-8.867	2.55	3	3	3.477	30
**Extra**													
DMSO - AZD0156													
DMSO	1.433	-5.400 to 8.267	No	ns	0.9216	8.533	7.1	1.433	2.467	3	3	0.581	12
miransertib 1 µM	-13.3	-20.13 to -6.467	Yes	***	0.0005	36.4	49.7	-13.3	2.467	3	3	5.392	12
ipatasertib 2 µM	-2.1	-8.933 to 4.733	No	ns	0.7959	21.37	23.47	-2.1	2.467	3	3	0.851	12
DMSO - apilimod													
DMSO	-8.033	-14.17 to -1.899	Yes	*	0.0104	8.533	16.57	-8.033	2.214	3	3	3.628	12
miransertib 1 µM	16.73	10.60 to 22.87	Yes	****	<0.0001	36.4	19.67	16.73	2.214	3	3	7.556	12
ipatasertib 2 µM	2.867	-3.268 to 9.001	No	ns	0.5251	21.37	18.5	2.867	2.214	3	3	1.295	12
DMSO - KU-57788													
DMSO	-11.83	-18.34 to -5.325	Yes	***	0.0009	8.533	20.37	-11.83	2.349	3	3	5.037	12
miransertib 1 µM	-12.77	-19.27 to -6.258	Yes	***	0.0005	36.4	49.17	-12.77	2.349	3	3	5.434	12
ipatasertib 2 µM	-5.433	-11.94 to 1.075	No	ns	0.1133	21.37	26.8	-5.433	2.349	3	3	2.313	12
DMSO - GSK2234470													
DMSO	-5.733	-11.64 to 0.1726	No	ns	0.0579	8.533	14.27	-5.733	2.132	3	3	2.689	12
miransertib 1 µM	-15	-20.91 to -9.094	Yes	****	<0.0001	36.4	51.4	-15	2.132	3	3	7.036	12
ipatasertib 2 µM	-1.767	-7.673 to 4.139	No	ns	0.8084	21.37	23.13	-1.767	2.132	3	3	0.829	12
**Supplementary figure 4A**													
**Cell number**													
DMSO													
0 vs. 0.25	0.286	0.05523 to 0.5169	Yes	*	0.0104	2.452	2.166	0.286	0.08712	3	3	3.283	30
0 vs. 0.5	0.3402	0.1094 to 0.5711	Yes	**	0.002	2.452	2.112	0.3402	0.08712	3	3	3.905	30
0 vs. 1	0.6052	0.3744 to 0.8360	Yes	****	<0.0001	2.452	1.847	0.6052	0.08712	3	3	6.947	30
0 vs. 2	1.066	0.8351 to 1.297	Yes	****	<0.0001	2.452	1.386	1.066	0.08712	3	3	12.23	30
miransertib 0.5 µM													
0 vs. 0.25	0.02108	-0.2097 to 0.2519	No	ns	0.9987	0.8221	0.801	0.02108	0.08712	3	3	0.242	30
0 vs. 0.5	0.1927	-0.03811 to 0.4235	No	ns	0.1319	0.8221	0.6294	0.1927	0.08712	3	3	2.212	30
0 vs. 1	0.3433	0.1124 to 0.5741	Yes	**	0.0018	0.8221	0.4788	0.3433	0.08712	3	3	3.94	30
0 vs. 2	0.539	0.3082 to 0.7698	Yes	****	<0.0001	0.8221	0.2831	0.539	0.08712	3	3	6.186	30
ipatasertib 1 µM													
0 vs. 0.25	0.199	-0.03181 to 0.4298	No	ns	0.1133	1.175	0.9762	0.199	0.08712	3	3	2.284	30
0 vs. 0.5	0.3287	0.09793 to 0.5596	Yes	**	0.0028	1.175	0.8465	0.3287	0.08712	3	3	3.773	30
0 vs. 1	0.6843	0.4535 to 0.9151	Yes	****	<0.0001	1.175	0.4909	0.6843	0.08712	3	3	7.855	30
0 vs. 2	0.8379	0.6071 to 1.069	Yes	****	<0.0001	1.175	0.3373	0.8379	0.08712	3	3	9.617	30

**Cell death**													
DMSO													
0 vs. 0.25	0.3667	-5.303 to 6.036	No	ns	0.9997	4.233	3.867	0.3667	2.14	3	3	0.171	30
0 vs. 0.5	-1.7	-7.369 to 3.969	No	ns	0.8968	4.233	5.933	-1.7	2.14	3	3	0.794	30
0 vs. 1	-4.3	-9.969 to 1.369	No	ns	0.1976	4.233	8.533	-4.3	2.14	3	3	2.009	30
0 vs. 2	-16.23	-21.90 to -10.56	Yes	****	<0.0001	4.233	20.47	-16.23	2.14	3	3	7.586	30
miransertib 0.5 µM													
0 vs. 0.25	-7.2	-12.87 to -1.531	Yes	**	0.0084	41.7	48.9	-7.2	2.14	3	3	3.365	30
0 vs. 0.5	-14.33	-20.00 to -8.664	Yes	****	<0.0001	41.7	56.03	-14.33	2.14	3	3	6.698	30
0 vs. 1	-19.6	-25.27 to -13.93	Yes	****	<0.0001	41.7	61.3	-19.6	2.14	3	3	9.16	30
0 vs. 2	-33.8	-39.47 to -28.13	Yes	****	<0.0001	41.7	75.5	-33.8	2.14	3	3	15.8	30
ipatasertib 1 µM													
0 vs. 0.25	-7.5	-13.17 to -1.831	Yes	**	0.0058	23.6	31.1	-7.5	2.14	3	3	3.505	30
0 vs. 0.5	-17.43	-23.10 to -11.76	Yes	****	<0.0001	23.6	41.03	-17.43	2.14	3	3	8.147	30
0 vs. 1	-26.77	-32.44 to -21.10	Yes	****	<0.0001	23.6	50.37	-26.77	2.14	3	3	12.51	30
0 vs. 2	-38.7	-44.37 to -33.03	Yes	****	<0.0001	23.6	62.3	-38.7	2.14	3	3	18.09	30
**Supplementary figure 4B**													
**Cell number**													
DMSO													
0 vs. 0.25	-0.2473	-0.5659 to 0.07128	No	ns	0.1804	2.2	2.447	-0.2473	0.1202	3	3	2.057	30
0 vs. 0.5	0.03754	-0.2810 to 0.3561	No	ns	0.9965	2.2	2.162	0.03754	0.1202	3	3	0.312	30
0 vs. 1	0.1899	-0.1287 to 0.5085	No	ns	0.4132	2.2	2.01	0.1899	0.1202	3	3	1.579	30
0 vs. 2	0.7662	0.4476 to 1.085	Yes	****	<0.0001	2.2	1.434	0.7662	0.1202	3	3	6.372	30
miransertib 1 µM													
0 vs. 0.25	0.3334	0.01484 to 0.6520	Yes	*	0.0373	1.308	0.9744	0.3334	0.1202	3	3	2.773	30
0 vs. 0.5	0.4968	0.1782 to 0.8154	Yes	**	0.0011	1.308	0.811	0.4968	0.1202	3	3	4.132	30
0 vs. 1	0.59	0.2714 to 0.9085	Yes	***	0.0001	1.308	0.7179	0.59	0.1202	3	3	4.906	30
0 vs. 2	0.758	0.4394 to 1.077	Yes	****	<0.0001	1.308	0.5499	0.758	0.1202	3	3	6.303	30
ipatasertib 2 µM													
0 vs. 0.25	0.05962	-0.2590 to 0.3782	No	ns	0.9799	1.438	1.378	0.05962	0.1202	3	3	0.496	30
0 vs. 0.5	-0.05299	-0.3716 to 0.2656	No	ns	0.987	1.438	1.491	-0.05299	0.1202	3	3	0.441	30
0 vs. 1	0.3334	0.01484 to 0.6520	Yes	*	0.0373	1.438	1.105	0.3334	0.1202	3	3	2.773	30
0 vs. 2	0.8441	0.5255 to 1.163	Yes	****	<0.0001	1.438	0.594	0.8441	0.1202	3	3	7.019	30
**Cell death**													
DMSO													
0 vs. 0.25	-13.97	-21.34 to -6.598	Yes	****	<0.0001								
0 vs. 0.5	-16.17	-23.54 to -8.798	Yes	****	<0.0001	10.2	24.17	-13.97	2.781	3	3	5.022	30
0 vs. 1	-19.9	-27.27 to -12.53	Yes	****	<0.0001	10.2	26.37	-16.17	2.781	3	3	5.813	30
0 vs. 2	-33.5	-40.87 to -26.13	Yes	****	<0.0001	10.2	30.1	-19.9	2.781	3	3	7.155	30
						10.2	43.7	-33.5	2.781	3	3	12.04	30
miransertib 1 µM													
0 vs. 0.25	-7.733	-15.10 to -0.3648	Yes	*	0.0366								
0 vs. 0.5	-15.17	-22.54 to -7.798	Yes	****	<0.0001	47.63	55.37	-7.733	2.781	3	3	2.781	30
0 vs. 1	-19.37	-26.74 to -12.00	Yes	****	<0.0001	47.63	62.8	-15.17	2.781	3	3	5.453	30
0 vs. 2	-24.1	-31.47 to -16.73	Yes	****	<0.0001	47.63	67	-19.37	2.781	3	3	6.963	30
						47.63	71.73	-24.1	2.781	3	3	8.665	30
ipatasertib 2 µM													
0 vs. 0.25	1.133	-6.235 to 8.502	No	ns	0.9903								
0 vs. 0.5	1.2	-6.169 to 8.569	No	ns	0.988	37.7	36.57	1.133	2.781	3	3	0.408	30
0 vs. 1	-13.43	-20.80 to -6.065	Yes	***	0.0002	37.7	36.5	1.2	2.781	3	3	0.432	30
0 vs. 2	-33.23	-40.60 to -25.86	Yes	****	<0.0001	37.7	51.13	-13.43	2.781	3	3	4.83	30
						37.7	70.93	-33.23	2.781	3	3	11.95	30
**Supplementary figure 4C**													
**Cell number**													
Parental - Res													
**DMSO**													
DMSO	-0.0414	-0.2453 to 0.1625	No	ns	>0.9999	2.097	2.138	-0.0414	0.06657	3	3	0.622	64
MK-2206 1 µM	-0.8611	-1.065 to -0.6572	Yes	****	<0.0001	0.9577	1.819	-0.8611	0.06657	3	3	12.94	64
miransertib 0.5 µM	-0.3246	-0.5285 to -0.1206	Yes	***	0.0001	1.014	1.339	-0.3246	0.06657	3	3	4.876	64
ipatasertib 1 µM	-0.2517	-0.4557 to -0.04778	Yes	**	0.0055	1.221	1.473	-0.2517	0.06657	3	3	3.781	64
**AZD0156**													
DMSO	0	-0.2039 to 0.2039	No	ns	>0.9999	1.832	1.832	0	0.06657	3	3	0	64
MK-2206 1 µM	-0.6111	-0.8150 to -0.4071	Yes	****	<0.0001	0.5718	1.183	-0.6111	0.06657	3	3	9.179	64
miransertib 0.5 µM	-0.101	-0.3050 to 0.1029	No	ns	0.9001	0.6083	0.7093	-0.101	0.06657	3	3	1.517	64
ipatasertib 1 µM	-0.1358	-0.3397 to 0.06814	No	ns	0.5253	0.8898	1.026	-0.1358	0.06657	3	3	2.04	64
**GSK2334470**													
DMSO	0.0828	-0.1211 to 0.2867	No	ns	0.9805	1.776	1.693	0.0828	0.06657	3	3	1.244	64
MK-2206 1 µM	-0.5138	-0.7178 to -0.3099	Yes	****	<0.0001	0.4737	0.9875	-0.5138	0.06657	3	3	7.718	64
miransertib 0.5 µM	-0.07949	-0.2834 to 0.1244	No	ns	0.9868	0.5884	0.6679	-0.07949	0.06657	3	3	1.194	64
ipatasertib 1 µM	-0.207	-0.4109 to -0.003068	Yes	*	0.0438	0.7325	0.9395	-0.207	0.06657	3	3	3.109	64
**OSU-T315**													
DMSO	-0.06955	-0.2735 to 0.1344	No	ns	0.9967	1.547	1.617	-0.06955	0.06657	3	3	1.045	64
MK-2206 1 µM	-0.5387	-0.7426 to -0.3347	Yes	****	<0.0001	0.3495	0.8881	-0.5387	0.06657	3	3	8.091	64
miransertib 0.5 µM	0.00828	-0.1957 to 0.2122	No	ns	>0.9999	0.3561	0.3478	0.00828	0.06657	3	3	0.124	64
ipatasertib 1 µM	0.1542	-0.04977 to 0.3581	No	ns	0.3197	0.7043	0.5502	0.1542	0.06657	3	3	2.316	64
**Cell death**													
Parental - Res													
DMSO													
**DMSO**	-0.4333	-6.858 to 5.991	No	ns	>0.9999	4.867	5.3	-0.4333	2.097	3	3	0.207	64
MK-2206 1 µM	14.63	8.209 to 21.06	Yes	****	<0.0001	22.63	8	14.63	2.097	3	3	6.978	64
miransertib 0.5 µM	5.033	-1.391 to 11.46	No	ns	0.2681	18.3	13.27	5.033	2.097	3	3	2.4	64
ipatasertib 1 µM	3.667	-2.758 to 10.09	No	ns	0.7594	13.67	10	3.667	2.097	3	3	1.748	64
**AZD0156**													

DMSO	1.333	-5.091 to 7.758	No	ns	>0.9999	6.667	5.333	1.333	2.097	3	3	0.636	64
MK-2206 1 µM	29.23	22.81 to 35.66	Yes	****	<0.0001	45.57	16.33	29.23	2.097	3	3	13.94	64
miransertib 0.5 µM	7.367	0.9422 to 13.79	Yes	*	0.013	49.4	42.03	7.367	2.097	3	3	3.513	64
ipatasertib 1 µM	7.433	1.009 to 13.86	Yes	*	0.0118	27.63	20.2	7.433	2.097	3	3	3.544	64
**GSK2334470**													
DMSO	0.1333	-6.291 to 6.558	No	ns	>0.9999	7.8	7.667	0.1333	2.097	3	3	0.064	64
MK-2206 1 µM	32.6	26.18 to 39.02	Yes	****	<0.0001	61.03	28.43	32.6	2.097	3	3	15.54	64
miransertib 0.5 µM	8.567	2.142 to 14.99	Yes	**	0.002	56.6	48.03	8.567	2.097	3	3	4.085	64
ipatasertib 1 µM	6.833	0.4089 to 13.26	Yes	*	0.0284	36.57	29.73	6.833	2.097	3	3	3.258	64
**OSU-T315**													
DMSO	0.1333	-6.291 to 6.558	No	ns	>0.9999	11.7	11.57	0.1333	2.097	3	3	0.064	64
MK-2206 1 µM	40.8	34.38 to 47.22	Yes	****	<0.0001	73.87	33.07	40.8	2.097	3	3	19.45	64
miransertib 0.5 µM	1.1	-5.324 to 7.524	No	ns	>0.9999	71.57	70.47	1.1	2.097	3	3	0.525	64
ipatasertib 1 µM	-4.567	-10.99 to 1.858	No	ns	0.4168	45.97	50.53	-4.567	2.097	3	3	2.178	64
**Supplementary figure 4D**													
**Cell number**													
DMSO - GSK2578215A													
DMSO	0.2087	-0.02865 to 0.4460	No	ns	0.0913	2.415	2.206	0.2087	0.08566	3	3	2.436	12
MK-2206 0.5 µM	0.1027	-0.1346 to 0.3400	No	ns	0.5845	1.282	1.18	0.1027	0.08566	3	3	1.199	12
ipatasertib 0.5 µM	0.0828	-0.1545 to 0.3201	No	ns	0.7289	1.62	1.537	0.0828	0.08566	3	3	0.967	12
**Cell death**													
DMSO - GSK2578215A													
DMSO	-1.767	-6.114 to 2.580	No	ns	0.6302	4.933	6.7	-1.767	1.569	3	3	1.126	12
MK-2206 0.5 µM	-1.133	-5.480 to 3.214	No	ns	0.8626	21.37	22.5	-1.133	1.569	3	3	0.722	12
ipatasertib 0.5 µM	0.2333	-4.114 to 4.580	No	ns	0.9984	13.13	12.9	0.2333	1.569	3	3	0.149	12
**Supplementary figure 4E**													
**Cell number**													
DMSO													
DMSO vs. OSU-T315	0.6485	0.4386 to 0.8584	Yes	****	<0.0001	1.501	0.853	0.6485	0.08606	3	3	7.535	18
DMSO vs. GSK2578215A	0.2371	0.02719 to 0.4470	Yes	*	0.0259	1.501	1.264	0.2371	0.08606	3	3	2.755	18
miransertib 1 µM													
DMSO vs. OSU-T315	0.4328	0.2229 to 0.6427	Yes	***	0.0002	0.642	0.2092	0.4328	0.08606	3	3	5.029	18
DMSO vs. GSK2578215A	0.0908	-0.1191 to 0.3007	No	ns	0.5174	0.642	0.5512	0.0908	0.08606	3	3	1.055	18
ipatasertib 2 µM													
DMSO vs. OSU-T315	0.4938	0.2839 to 0.7037	Yes	****	<0.0001	0.832	0.3382	0.4938	0.08606	3	3	5.738	18
DMSO vs. GSK2578215A	0.01918	-0.1907 to 0.2291	No	ns	0.9698	0.832	0.8129	0.01918	0.08606	3	3	0.223	18
**Cell death**													
DMSO													
DMSO vs. OSU-T315	-16.87	-22.25 to -11.49	Yes	****	<0.0001	21.87	38.73	-16.87	2.205	3	3	7.648	18
DMSO vs. GSK2578215A	3.8	-1.579 to 9.179	No	ns	0.1936	21.87	18.07	3.8	2.205	3	3	1.723	18
miransertib 1 µM													
DMSO vs. OSU-T315	-30.03	-35.41 to -24.65	Yes	****	<0.0001	46.2	76.23	-30.03	2.205	3	3	13.62	18
DMSO vs. GSK2578215A	-2.567	-7.945 to 2.812	No	ns	0.452	46.2	48.77	-2.567	2.205	3	3	1.164	18
ipatasertib 2 µM													
DMSO vs. OSU-T315	-40.37	-45.75 to -34.99	Yes	****	<0.0001	29.97	70.33	-40.37	2.205	3	3	18.3	18
DMSO vs. GSK2578215A	0.5	-4.879 to 5.879	No	ns	0.9687	29.97	29.47	0.5	2.205	3	3	0.227	18
**Supplemetary figure 5A**													
**Cell Number**													
DMSO													
DMSO vs. miransertib 1 µM	1.082	0.9013 to 1.262	Yes	****	<0.0001	1.934	0.852	1.082	0.07561	3	3	14.31	24
DMSO vs. ipatasertib 1 µM	0.595	0.4147 to 0.7753	Yes	****	<0.0001	1.934	1.339	0.595	0.07561	3	3	7.869	24
Apilimod 50 nM													
DMSO vs. miransertib 1 µM	0.4623	0.2820 to 0.6427	Yes	****	<0.0001	1.485	1.022	0.4623	0.07561	3	3	6.114	24
DMSO vs. ipatasertib 1 µM	0.196	0.01565 to 0.3763	Yes	*	0.0317	1.485	1.289	0.196	0.07561	3	3	2.592	24
Apilimod 100 nM													
DMSO vs. miransertib 1 µM	0.05133	-0.1290 to 0.2317	No	ns	0.7537	1.187	1.136	0.05133	0.07561	3	3	0.679	24
DMSO vs. ipatasertib 1 µM	0.005667	-0.1747 to 0.1860	No	ns	0.9965	1.187	1.182	0.005667	0.07561	3	3	0.075	24
Apilimod 200 nM													
DMSO vs. miransertib 1 µM	0.08367	-0.09668 to 0.2640	No	ns	0.4808	1.128	1.044	0.08367	0.07561	3	3	1.107	24
DMSO vs. ipatasertib 1 µM	0.04733	-0.1330 to 0.2277	No	ns	0.7858	1.128	1.08	0.04733	0.07561	3	3	0.626	24
**Cell death**													
DMSO													
DMSO vs. miransertib 1 µM	-46.92	-50.51 to -43.33	Yes	****	<0.0001	4.376	51.29	-46.92	1.505	3	3	31.18	24
DMSO vs. ipatasertib 1 µM	-19.66	-23.24 to -16.07	Yes	****	<0.0001	4.376	24.03	-19.66	1.505	3	3	13.06	24
Apilimod 50 nM													
DMSO vs. miransertib 1 µM	-11.47	-15.05 to -7.878	Yes	****	<0.0001	13.51	24.98	-11.47	1.505	3	3	7.621	24
DMSO vs. ipatasertib 1 µM	-1.293	-4.881 to 2.296	No	ns	0.6385	13.51	14.81	-1.293	1.505	3	3	0.859	24
Apilimod 100 nM													
DMSO vs. miransertib 1 µM	-7.956	-11.54 to -4.368	Yes	****	<0.0001	15.74	23.69	-7.956	1.505	3	3	5.288	24
DMSO vs. ipatasertib 1 µM	-3.05	-6.638 to 0.5385	No	ns	0.1049	15.74	18.79	-3.05	1.505	3	3	2.027	24
Apilimod 200 nM													
DMSO vs. miransertib 1 µM	-3.919	-7.508 to -0.3308	Yes	*	0.0308	20.17	24.09	-3.919	1.505	3	3	2.605	24
DMSO vs. ipatasertib 1 µM	1.475	-2.113 to 5.063	No	ns	0.56	20.17	18.7	1.475	1.505	3	3	0.98	24
**Supplementary figure 6B**													

**Cell number**														
DMSO														
0 vs. 10	1.105	0.8615 to 1.349	Yes	****	<0.0001		2.452	1.347	1.105	0.09191	3	3	12.02	30
0 vs. 25	1.03	0.7863 to 1.273	Yes	****	<0.0001		2.452	1.422	1.03	0.09191	3	3	11.2	30
0 vs. 50	1.192	0.9489 to 1.436	Yes	****	<0.0001		2.452	1.26	1.192	0.09191	3	3	12.97	30
0 vs. 100	0.9786	0.7351 to 1.222	Yes	****	<0.0001		2.452	1.473	0.9786	0.09191	3	3	10.65	30
MK-2206 0.5 µM														
0 vs. 10	0.3435	0.1000 to 0.5870	Yes	**	0.0031		0.9037	0.5601	0.3435	0.09191	3	3	3.738	30
0 vs. 25	0.3435	0.1000 to 0.5870	Yes	**	0.0031		0.9037	0.5601	0.3435	0.09191	3	3	3.738	30
0 vs. 50	0.3766	0.1331 to 0.6201	Yes	**	0.0012		0.9037	0.527	0.3766	0.09191	3	3	4.098	30
0 vs. 100	0.4068	0.1633 to 0.6503	Yes	***	0.0005		0.9037	0.4969	0.4068	0.09191	3	3	4.426	30
miransertib 0.25 µM														
0 vs. 10	0.1538	-0.08967 to 0.3973	No	ns	0.3571		0.8525	0.6986	0.1538	0.09191	3	3	1.674	30
0 vs. 25	0.3405	0.09702 to 0.5840	Yes	**	0.0034		0.8525	0.512	0.3405	0.09191	3	3	3.705	30
0 vs. 50	0.3044	0.06088 to 0.5479	Yes	**	0.0097		0.8525	0.5481	0.3044	0.09191	3	3	3.312	30
0 vs. 100	0.1568	-0.08665 to 0.4003	No	ns	0.3388		0.8525	0.6956	0.1568	0.09191	3	3	1.707	30
**Cell death**														
DMSO														
0 vs. 10	-1	-8.903 to 6.903	No	ns	0.9954		4.233	5.233	-1	2.983	3	3	0.335	30
0 vs. 25	-3.5	-11.40 to 4.403	No	ns	0.6835		4.233	7.733	-3.5	2.983	3	3	1.173	30
0 vs. 50	-0.3333	-8.237 to 7.570	No	ns	>0.9999		4.233	4.567	-0.3333	2.983	3	3	0.112	30
0 vs. 100	-2.533	-10.44 to 5.370	No	ns	0.8725		4.233	6.767	-2.533	2.983	3	3	0.849	30
MK-2206 0.5 µM														
0 vs. 10	-21.67	-29.57 to -13.76	Yes	****	<0.0001		24.47	46.13	-21.67	2.983	3	3	7.263	30
0 vs. 25	-21.43	-29.34 to -13.53	Yes	****	<0.0001		24.47	45.9	-21.43	2.983	3	3	7.185	30
0 vs. 50	-21.5	-29.40 to -13.60	Yes	****	<0.0001		24.47	45.97	-21.5	2.983	3	3	7.207	30
0 vs. 100	-24.13	-32.04 to -16.23	Yes	****	<0.0001		24.47	48.6	-24.13	2.983	3	3	8.09	30
miransertib 0.25 µM														
0 vs. 10	-13.67	-21.57 to -5.763	Yes	***	0.0003		26.87	40.53	-13.67	2.983	3	3	4.581	30
0 vs. 25	-17.93	-25.84 to -10.03	Yes	****	<0.0001		26.87	44.8	-17.93	2.983	3	3	6.012	30
0 vs. 50	-14.57	-22.47 to -6.663	Yes	***	0.0001		26.87	41.43	-14.57	2.983	3	3	4.883	30
0 vs. 100	-10.63	-18.54 to -2.730	Yes	**	0.005		26.87	37.5	-10.63	2.983	3	3	3.565	30
**Supplementary figure 7**														
Parental 1h vs. Parental 6h	-0.5734	-0.8126 to -0.3342	Yes	***	0.0002	A-B	0.6921	1.266	-0.5734	0.07492	3	3	7.654	8
Res 1h vs. Res 6h	-0.06427	-0.3035 to 0.1750	No	ns	0.8836	C-D	0.7128	0.7771	-0.06427	0.07492	3	3	0.858	8
Parental 1h vs. Res 1h	-0.02067	-0.2599 to 0.2186	No	ns	0.998	A-C	0.6921	0.7128	-0.02067	0.07492	3	3	0.276	8
Parental 6h vs. Res 6h	0.4885	0.2492 to 0.7277	Yes	***	0.0007	B-D	1.266	0.7771	0.4885	0.07492	3	3	6.52	8
**Supplementary figure 8**														
**MDA-MB-361**														
CTR siRNA vs. AKT1+2 siRNA	-100.1	-130.1 to -70.17	Yes	***	0.0001	A-B	75.11	175.2	-100.1	9.151	3	3	10.94	6
AKT1+2 siRNA vs. no sirNA	95.42	65.46 to 125.4	Yes	***	0.0001	B-C	175.2	79.82	95.42	9.151	3	3	10.43	6
CTR siRNA vs. no sirNA	-4.711	-34.67 to 25.25	No	ns	0.9473	A-C	75.11	79.82	-4.711	9.151	3	3	0.515	6
**EBC1**														
CTR siRNA vs. AKT1+2 siRNA	-96.62	-105.8 to -87.42	Yes	****	<0.0001	A-B	54.54	151.2	-96.62	2.812	3	3	34.36	6
CTR siRNA vs. no sirNA	2.074	-7.134 to 11.28	No	ns	0.8664	A-C	54.54	52.47	2.074	2.812	3	3	0.737	6
AKT1+2 siRNA vs. no sirNA	98.7	89.49 to 107.9	Yes	****	<0.0001	B-C	151.2	52.47	98.7	2.812	3	3	35.09	6

MDA-MB-361 Inducible-Cas9 AKT1/2 KO

Column B	+dox
vs.	vs.
Column A	-dox
Unpaired t test	
P value	0.0003
P value summary	***
Significantly different (P < 0.05)?	Yes
One- or two-tailed P value?	Two-tailed
t, df	t=5.495, df=10

DLD-1

Column B	AKT1/2 KO
vs.	vs.
Column A	Parental
Unpaired t test	
P value	0.0106
P value summary	*
Significantly different (P < 0.05)?	Yes
One- or two-tailed P value?	Two-tailed
t, df	t=4.533, df=4

HCT116

Column B	AKT 1/2 KO
vs.	vs.
Column A	Parental

Unpaired t test	
P value	0.0431
P value summary	*
Significantly different (P < 0.05)?	Yes
One- or two-tailed P value?	Two-tailed
t, df	t=2.923, df=4

Sup. table 1: Statistical analysis of all the experiments described in the manuscript.
(Green: 1-way Anova, Blue: 2-way Anova)

Supplementary table 2

µmol/million cell/hr

	sample		Leucine	Iso-leucin	Valine	Threonine	Lactate	Alanine	Acetate	Pyruvate	Glutamine	Choline	Glycine	Glucose	Histidine	Tyrosine	Phe	Formate
**HCT116 DKO**																		
1	DMSO1a		0.006019	0.005656	0.004574	-0.00233	-0.44867	-0.00766	-0.001166213	0.271949	0.490803	0.006926	0.002943	0.09761	0.000647	0.001537	0.0014	-0.00296
2	DMSO1b		0.003429	0.002832	0.002264	-0.00395	-0.49687	-0.00686	-0.001554792	0.255876	0.48467	0.006871	-0.00877	0.090325	0.000293	0.000989	0.000151	-0.00357
3	DMSO2a		0.00328	0.002	0.001952	-0.01887	-0.53603	-0.00713	-0.001442618	0.266401	0.452164	0.008263	-0.01094	0.110089	0.000436	0.001305	0.001534	-0.00364
4	DMSO2b		0.002861	0.001476	0.000957	-0.00547	-0.58424	-0.00709	-0.001444921	0.279555	0.374633	0.006145	-0.01226	0.107065	0.000922	0.000622	-0.00413	
5	DMSO3a		0.004836	0.0041	0.003413	-0.00304	-0.53577	-0.00757	-0.001461909	0.272987	0.40762	0.008073	-0.0085	0.10983	0.000765	0.001317	0.00127	-0.00358
6	DMSO3b		0.004386	0.003229	0.002936	-0.00369	-0.54411	-0.00727	-0.001271183	0.280819	0.414613	0.007021	-0.00875	0.118022	0.000552	0.000976	0.001116	-0.00354
		Mean	0.004135	0.003215	0.002683	-0.00623	-0.52428	-0.00726	**-0.001390273**	0.271264	0.437417	0.007217	-0.00771	0.10549	0.000603	0.001124	0.000224	-0.00346
		St Dev	0.001179	0.001509	0.001254	0.006285	0.046317	0.000307	0.000143046	0.009208	0.046152	0.000802	0.005431	0.009915	0.000226	0.000326	0.002188	0.000278
		SEM	0.000527	0.000675	0.000561	0.002811	0.020714	0.000137	6.39719E-05	0.004118	0.02064	0.000359	0.002429	0.004434	0.000101	0.000146	0.000979	0.000124
**HCT116 AKT2**																		
1	DMSO1a		0.003533	0.002891	0.002245	-0.00469	-0.43088	-0.00575	-0.000734102	0.240358	0.426546	0.008538	-0.00794	0.088089	0.000832	0.0013	0.001556	-0.00273
2	DMSO1b		0.003703	0.003424	0.002824	-0.00831	-0.42525	-0.00668	-0.000894274	0.234214	0.389387	0.007715	-0.01029	0.085541	0.000134	0.000951	0.000505	-0.00268
3	DMSO2a		0.005285	0.004845	0.004183	-0.00647	-0.46217	-0.00688	-0.00069781	0.247146	0.420771	0.008072	-0.00832	0.097214	0.000348	0.000895	0.001475	-0.00293
4	DMSO2b		0.004157	0.003479	0.003033	-0.00398	-0.50738	-0.0073	-0.000779155	0.248113	0.42851	0.009326	-0.00493	0.095986	-0.00012	0.000606	0.001013	-0.00315
5	DMSO3a		0.004802	0.004257	0.003499	-0.00183	-0.46404	-0.00552	-0.000443383	0.247891	0.41013	0.006673	-0.00624	0.091358	0.00069	0.000942	0.000909	-0.00269
6	DMSO3b		0.005259	0.005091	0.004214	-0.00253	-0.47427	-0.007	-0.000575983	0.246765	0.422707	0.007792	-0.00724	0.096823	0.00029	0.001471	0.001937	-0.00292
		Mean	0.004457	0.003998	0.003333	-0.00463	-0.46067	-0.00652	**-0.000687451**	0.244081	0.416342	0.00802	-0.0075	0.092502	0.000362	0.001028	0.001233	-0.00285
		St Dev	0.000769	0.000872	0.000782	0.002433	0.030073	0.00072	0.000158407	0.005632	0.01468	0.000887	0.001838	0.004945	0.000351	0.000309	0.000518	0.000183
		SEM	0.000344	0.00039	0.00035	0.001088	0.013449	0.000322	7.08417E-05	0.002519	0.006565	0.000397	0.000822	0.002211	0.000157	0.000138	0.000232	8.18E-05
p value HCT116 DKO vs AKT2			0.58824	0.297383	0.306509	0.575473	0.018109	0.042773	1.09575E-05	0.000106	0.311511	0.131172	0.927768	0.016629	0.189573	0.610642	0.297486	0.001832
			Leucine	Iso-leucine	Valine	Threonine	Lactate	Alanine	Acetate	Pyruvate	Glutamine	Choline	Glycine	Glucose	Histidine	Tyrosine	Phe	Formate

Sup. table 2: Acetate excretion is decreased upon AKT2 overexpression. Magnetic resonance spectroscopy (MRS) was used to measure the concentration of 16 different metabolites incell culture media from AKT1/2 DKO and AKT2-reconstituted HCT116 cells.

Supplementary Spreadsheet

	QE2_Igor_Exp2_PP_1A	QE2_Igor_Exp2_PP_1B	QE2_Igor_Exp2_PP_1C	QE2_Igor_Exp2_PP_1D	QE2_Igor_Exp2_PP_1E	QE2_Igor_Exp2_PP_2A	QE2_Igor_Exp2_PP_2B	QE2_Igor_Exp2_PP_2C	QE2_Igor_Exp2_PP_2D	QE2_Igor_Exp2_PP_2E	QE2_Igor_Exp2_PP_3A	QE2_Igor_Exp2_PP_3B	QE2_Igor_Exp2_PP_3C	QE2_Igor_Exp2_PP_3D	QE2_Igor_Exp2_PP_3E	QE2_Igor_Exp2_PP_4A	QE2_Igor_Exp2_PP_4B	QE2_Igor_Exp2_PP_4C	QE2_Igor_Exp2_PP_4D	QE2_Igor_Exp2_PP_4E	QE2_Igor_Exp2_PP_5A	QE2_Igor_Exp2_PP_5B	QE2_Igor_Exp2_PP_5C	QE2_Igor_Exp2_PP_5D	QE2_Igor_Exp2_PP_5E	QE2_Igor_Exp2_PP_6A	QE2_Igor_Exp2_PP_6B	QE2_Igor_Exp2_PP_6C	QE2_Igor_Exp2_PP_6D	QE2_Igor_Exp2_PP_6E	QE2_Igor_Exp2_PP_7A	QE2_Igor_Exp2_PP_7B	QE2_Igor_Exp2_PP_7C	QE2_Igor_Exp2_PP_7D	QE2_Igor_Exp2_PP_7E	QE2_Igor_Exp2_PP_8A	QE2_Igor_Exp2_PP_8B	QE2_Igor_Exp2_PP_8C	QE2_Igor_Exp2_PP_8D	QE2_Igor_Exp2_PP_8E	QE2_Igor_Exp2_PP_9A	QE2_Igor_Exp2_PP_9B	QE2_Igor_Exp2_PP_9C	QE2_Igor_Exp2_PP_9D	QE2_Igor_Exp2_PP_9E	QE2_Igor_Exp2_PP_10	QE2_Igor_Exp2_PP_10	QE2_Igor_Exp2_PP_10	QE2_Igor_Exp2_PP_10	QE2_Igor_Exp2_PP_10	db id	Protein name
NPM1 pS70	38324	37149	39656	13654	33854	1E+09	2E+07	34892	34104	26736	1E+09	9E+08	39754	40879	18798	1E+08	33072	34984	19162	28832	39759	50401	21442	19024	42741	6E+08	44396	34144	19500	29103	45838	31655	19257	24890	17227	4E+07	27262	25062	27887	36936	100327	64618	38440	41974	13188	134692	41467	25947	30272	25732	p01906	Nucleophosmin
G3BP1 seq: 79 - 106 no mod	38324	37149	39656	13654	33854	5E+08	25552	34892	34104	26736	4E+08	3E+08	39754	40879	18798	9E+07	33072	34984	19162	28832	39759	50401	21442	19024	42741	5E+08	44396	34144	19500	29103	7E+06	31655	703196	24890	17227	6E+07	27262	25062	27887	36936	100327	64618	38440	41974	13188	134692	41467	25947	30272	25732	p14574	Ras GTPase-activating protein-binding protein 1
C6orf132 seq: 963 - 1008 + Phospho (ST)	38324	37149	39656	13654	33854	2E+08	1E+08	34892	8E+07	26736	4E+08	2E+08	39754	2E+08	18798	3E+07	33072	34984	19162	28832	39759	50401	21442	1E+08	42741	1E+08	44396	34144	19500	29103	45838	31655	19257	24890	17227	44771	27262	25062	27887	36936	100327	64618	38440	41974	13188	134692	41467	25947	30272	25732	p05816	Uncharacterized protein C6orf132
LMAN1 seq: 161 - 192 no mod	38324	37149	39656	13654	33854	5E+08	3E+08	34892	34104	2E+08	1E+09	5E+08	39754	2E+08	2E+08	5E+08	33072	34984	1E+08	28832	3E+08	50401	21442	1E+08	42741	4E+08	44396	34144	19500	29103	45838	31655	19257	24890	17227	44771	27262	25062	27887	36936	100327	64618	38440	41974	13188	134692	41467	25947	30272	25732	p09320	Protein ERGIC-53
FKBP3 seq: 127 - 154 + Phospho (ST)	38324	37149	39656	13654	33854	4E+08	25552	34892	34104	26736	3E+08	53206	39754	40879	18798	40867	33072	34984	2E+08	28832	39759	50401	21442	2E+08	42741	3E+07	44396	34144	2E+07	29103	45838	31655	19257	24890	17227	44771	27262	25062	27887	36936	100327	64618	38440	41974	13188	134692	41467	25947	30272	25732	p13204	Peptidyl-prolyl cis-trans isomerase FKBP3
seq: 467 - 485 + Gln->pyro-Glu (N-term Q); Phospho (ST)	38324	37149	39656	13654	33854	45779	25552	34892	34104	26736	8E+07	53206	39754	40879	18798	3E+07	33072	34984	1E+07	1E+07	39759	50401	21442	19024	9E+06	68554	44396	34144	19500	1E+07	45838	31655	19257	24890	17227	44771	27262	25062	27887	36936	100327	64618	38440	2E+07	13188	134692	41467	25947	2E+07	25732	p08621	Cyclin-T2
TPD52L2 pS96	38324	37149	39656	13654	33854	8E+07	25552	34892	34104	26736	2E+07	53206	39754	40879	18798	2E+07	33072	34984	19162	28832	2E+07	50401	21442	19024	42741	9E+08	44396	34144	19500	29103	5E+08	31655	19257	24890	17227	3E+08	27262	25062	27887	36936	4E+08	64618	38440	41974	13188	134692	41467	25947	30272	25732	p03194	Tumor protein D54
TP53BP1 seq: 1017 - 1043 + Phospho (ST)	38324	37149	39656	13654	33854	45779	25552	4E+07	2E+07	1E+07	56234	53206	39754	40879	1E+07	40867	33072	34984	19162	9E+06	1E+07	50401	21442	2E+07	42741	6E+07	2E+06	6E+07	19500	29103	45838	31655	19257	24890	17227	3E+07	7E+07	25062	2E+07	36936	3E+07	64618	38440	41974	13188	3E+07	41467	25947	30272	4E+07	p09591	Tumor suppressor p53-binding protein 1
RABGAP1L pS490	38324	37149	39656	13654	33854	45779	8E+07	34892	6E+07	5E+07	56234	5E+07	39754	40879	18798	40867	33072	8E+07	7E+07	5E+07	39759	4E+07	5E+07	5E+07	42741	68554	3E+07	34144	19500	29103	45838	31655	19257	24890	17227	44771	27262	25062	27887	36936	100327	64618	38440	41974	13188	134692	41467	25947	30272	2E+07	p17180	Rab GTPase-activating protein 1-like
MSL1 seq: 148 - 198 + Phospho (ST)	38324	37149	39656	13654	33854	45779	25552	7E+07	34104	5E+07	56234	53206	39754	8E+07	8E+07	40867	33072	6E+07	19162	28832	9E+07	50401	21442	19024	42741	68554	4E+07	34144	19500	29103	45838	31655	19257	24890	17227	7E+07	7E+07	1E+08	27887	36936	100327	64618	38440	41974	13188	134692	41467	25947	30272	25732	p17299	Male-specific lethal 1 homolog
RAD9A seq: 253 - 280 + 2 Phospho (ST)	38324	37149	39656	13654	33854	3E+08	25552	34892	34104	26736	1E+08	2E+07	39754	40879	18798	2E+07	33072	34984	19162	28832	2E+07	50401	21442	19024	42741	2E+07	44396	34144	19500	29103	3E+06	31655	19257	24890	17227	8E+06	27262	25062	27887	36936	100327	64618	38440	41974	13188	134692	41467	25947	30272	25732	p18259	Cell cycle checkpoint control protein RAD9A
AKT1 seq: 122 - 142 + 2 Phospho (ST)	38324	37149	39656	13654	33854	7E+08	8E+07	34892	34104	26736	2E+08	53206	39754	40879	18798	2E+08	33072	34984	19162	2E+07	3E+08	50401	1E+08	19024	5E+07	68554	44396	34144	19500	1E+07	45838	31655	19257	24890	17227	44771	27262	25062	27887	36936	100327	64618	38440	41974	13188	134692	41467	25947	30272	25732	p05664	RAC-alpha serine/threonine-protein kinase
CDC6 pS54	38324	37149	39656	13654	33854	4E+07	1E+07	34892	34104	26736	56234	53206	39754	40879	18798	40867	33072	1E+07	19162	28832	39759	50401	21442	7E+06	42741	1E+09	4E+08	3E+08	5E+08	4E+08	5E+08	3E+08	3E+08	4E+08	4E+08	7E+08	5E+08	3E+08	4E+08	5E+08	7E+08	4E+08	3E+08	3E+08	3E+08	4E+08	3E+08	3E+08	3E+08	3E+08	p20206	Cell division control protein 6 homolog
MINK1 pS601	38324	37149	39656	13654	33854	45779	25552	34892	34104	26736	56234	53206	39754	40879	18798	40867	5E+07	34984	19162	4E+07	39759	50401	3E+07	2E+07	4E+07	68554	1E+08	1E+08	9E+07	9E+07	45838	1E+08	5E+07	1E+08	1E+08	44771	6E+07	7E+07	5E+07	9E+07	2E+08	2E+08	9E+07	1E+08	1E+08	1E+08	1E+08	1E+08	7E+07	8E+07	p04185	Misshapen-like kinase 1
KLHDC8A seq: 14 - 20 + Phospho (ST)	38324	37149	39656	13654	33854	2E+07	1E+07	34892	34104	26736	56234	53206	39754	40879	18798	40867	33072	1E+07	9E+06	28832	39759	50401	21442	7E+06	42741	1E+09	4E+08	3E+08	5E+08	4E+08	5E+08	3E+08	3E+08	4E+08	4E+08	7E+08	5E+08	3E+08	4E+08	5E+08	7E+08	4E+08	3E+08	3E+08	3E+08	4E+08	3E+08	3E+08	3E+08	3E+08	p12211	Kelch domain-containing protein 8A
CREBBP seq: 1876 - 1926 + 2 Phospho (ST)	38324	37149	39656	13654	33854	45779	25552	34892	34104	8E+07	56234	53206	7E+07	40879	18798	40867	5E+07	9E+07	6E+07	28832	39759	50401	1E+08	4E+07	8E+07	68554	44396	34144	19500	3E+07	45838	31655	19257	24890	17227	44771	27262	6E+07	27887	36936	100327	64618	38440	41974	13188	134692	41467	25947	30272	25732	p08952	CREB-binding protein
PHLDB1 seq: 329 - 344 + Phospho (ST)	38324	37149	39656	13654	33854	45779	25552	34892	4E+06	26736	4E+07	53206	39754	40879	18798	40867	33072	34984	6E+06	1E+07	39759	9E+06	1E+07	7E+06	42741	8E+07	7E+07	1E+08	4E+07	2E+07	3E+07	2E+07	2E+07	3E+07	2E+07	6E+07	6E+07	1E+08	27887	36936	7E+07	5E+07	1E+08	4E+07	13188	5E+07	3E+07	7E+07	30272	25732	p15694	Pleckstrin homology-like domain family B member 1
LMO7 seq: 1524 - 1556 + Phospho (ST)	38324	37149	39656	13654	33854	45779	25552	2E+07	34104	2E+07	56234	53206	39754	40879	18798	2E+07	33072	34984	19162	28832	39759	6E+06	21442	19024	42741	7E+08	4E+08	2E+08	5E+08	5E+08	3E+08	3E+08	9E+07	4E+08	4E+08	4E+08	3E+08	2E+08	3E+08	2E+08	5E+08	6E+08	3E+08	4E+08	4E+08	5E+08	4E+08	4E+08	6E+08	4E+08	p08108	LIM domain only protein 7
RFC2 seq: 92 - 111 no mod	38324	37149	39656	13654	33854	6E+07	25552	34892	34104	26736	8E+07	53206	39754	40879	18798	1E+07	33072	34984	19162	2E+06	2E+07	50401	21442	19024	42741	4E+07	44396	34144	19500	4E+06	3E+07	31655	19257	24890	4E+06	3E+07	27262	25062	27887	36936	100327	64618	38440	41974	1E+07	134692	41467	25947	30272	25732	p20236	Replication factor C subunit 2

GAPDH pM175	[illegible]	p01389	Glyceraldehyde-3-phosphate dehydrogenase
MAP4K4 seq: 638 - 662 + Phospho (ST)	[illegible]	p18231	Mitogen-activated protein kinase kinase kinase kinase 4
ABLIM3 pS585	[illegible]	p06262	Actin-binding LIM protein 3
ZC3HAV1 seq: 271 - 295 + Phospho (ST)	[illegible]	p06126	Zinc finger CCCH-type antiviral protein 1
PDIA3 seq: 148 - 173 no mod	[illegible]	p16670	Protein disulfide-isomerase A3
MAP1B seq: 1912 - 1928 + Phospho (ST)	[illegible]	p02044	Microtubule-associated protein 1B
PLEC seq: 2610 - 2642 no mod	[illegible]	p06040	Plectin
ATN1 seq: 73 - 88 + 2 Phospho (ST)	[illegible]	p06044	Atrophin-1
POLA2 pS141	[illegible]	p04592	DNA polymerase alpha subunit B
DHX9 seq: 366 - 385 no mod	[illegible]	p09675	ATP-dependent RNA helicase A
CHD4 pS428	[illegible]	p17737	Chromodomain-helicase-DNA-binding protein 4
ZYX pS267	[illegible]	p15482	Zyxin
SP1 seq: 20 - 39 no mod	[illegible]	p15038	Transcription factor Sp1
PAIP2 seq: 117 - 123 no mod	[illegible]	p17776	Polyadenylate-binding protein-interacting protein 2
LMO7 seq: 908 - 922 + Phospho (ST)	[illegible]	p10484	LIM domain only protein 7
YARS seq: 433 - 450 no mod	[illegible]	p15043	Tyrosine--tRNA ligase, cytoplasmic
GAB2 pS543	[illegible]	p09287	GRB2-associated-binding protein 2
IRF2BP2 seq: 389 - 442 + 2 Phospho (ST)	[illegible]	p13875	Interferon regulatory factor 2-binding protein 2
PNN seq: 374 - 397 + Oxidation (M); Phospho (ST)	[illegible]	p17167	Pinin
ACSS2 pS28 pS30	[illegible]	p19671	Acetyl-coenzyme A synthetase, cytoplasmic
BRWD1 pS696	[illegible]	p07131	Bromodomain and WD repeat-containing protein 1
KIF15 pS1141 pT1144	[illegible]	p08395	Kinesin-like protein KIF15
SCEL seq: 89 - 106 + Phospho (ST)	[illegible]	p00075	Sciellin
ANXA3 seq: 305 - 317 no mod	[illegible]	p06068	Annexin A3
RASAL2 pS954	[illegible]	p07997	Ras GTPase-activating protein nGAP
MAP7 seq: 240 - 261 + Phospho (ST)	[illegible]	p05347	Ensconsin
SEPT9 seq: 269 - 289 + Phospho (Y)	[illegible]	p09678	Septin-9
PITHD1 seq: 110 - 131 no mod	[illegible]	p09538	PITH domain-containing protein 1
USP36 pS871	[illegible]	p08133	Ubiquitin carboxyl-terminal hydrolase 36
SRRM2 pS2032	[illegible]	p03981	Serine/arginine repetitive matrix protein 2
ENTHD2 pS356	[illegible]	p14837	AP-4 complex accessory subunit tepsin
AHNAK seq: 711 - 721 + Phospho (Y)	[illegible]	p01316	Neuroblast differentiation-associated protein AHNAK
MAFK pS25	[illegible]	p17968	Transcription factor MafK
MDC1 seq: 960 - 1000 + Phospho (ST)	[illegible]	p15412	Mediator of DNA damage checkpoint protein 1
TPM3 seq: 191 - 206 no mod	[illegible]	p19019	Tropomyosin alpha-3 chain
UGGT1 seq: 1436 - 1466 no mod	[illegible]	p15362	UDP-glucose:glycoprotein glucosyltransferase 1
EPPK1 pT1543	[illegible]	p12488	Epiplakin
AKAP12 seq: 488 - 508 + Phospho (ST)	[illegible]	p01175	A-kinase anchor protein 12
INADL seq: 1207 - 1225 + Phospho (ST)	[illegible]	p20137	InaD-like protein
ETV6 pS213	[illegible]	p07102	Transcription factor ETV6
TAB3 pM32	[illegible]	p07983	TGF-beta-activated kinase 1 and MAP3K7-binding protein 3
MPHOSPH8 pS403	[illegible]	p15616	M-phase phosphoprotein 8
CDC26 pS42	[illegible]	p08470	Anaphase-promoting complex subunit CDC26
CNKSR1 seq: 564 - 582 + Phospho (ST)	[illegible]	p02184	Connector enhancer of kinase suppressor of ras 1
SHARPIN seq: 129 - 164 + Phospho (ST)	[illegible]	p04767	Sharpin
NCAPG pS973 pS975	[illegible]	p07537	Condensin complex subunit 3
PLEKHA5 pS596	[illegible]	p03309	Pleckstrin homology domain-containing family A member 5
HDGF seq: 12 - 19 no mod	[illegible]	p19057	Hepatoma-derived growth factor
BIN1 seq: 296 - 311 + Phospho (ST)	[illegible]	p04390	Myc box-dependent-interacting protein 1
FASN seq: 968 - 992 no mod	[illegible]	p11909	Fatty acid synthase
PLIN3 pS130	[illegible]	p17374	Perilipin-3
PHLDB1 seq: 532 - 560 + Phospho (Y)	[illegible]	p15289	Pleckstrin homology-like domain family B member 1
SLIT1 seq: 316 - 322 no mod	[illegible]	p11961	Slit homolog 1 protein
GLOD4 seq: 199 - 205 no mod	[illegible]	p11956	Glyoxalase domain-containing protein 4
ATRX seq: 590 - 616 + 2 Phospho (ST)	[illegible]	p10512	Transcriptional regulator ATRX
INCENP seq: 19 - 29 no mod	[illegible]	p11238	Inner centromere protein
EEPD1 pS173	[illegible]	p03370	Endonuclease/exonuclease/phosphatase family domain-containing protein 1
SAMD4B seq: 603 - 615 + Phospho (ST)	[illegible]	p14895	Protein Smaug homolog 2
HN1 pS131	[illegible]	p16170	Hematological and neurological expressed 1 protein
RPL27A seq: 66 - 87 no mod	[illegible]	p09213	60S ribosomal protein L27a
TJP2 pS1159	[illegible]	p15131	Tight junction protein ZO-2
EWSR1 seq: 487 - 494 no mod	[illegible]	p10121	RNA-binding protein EWS
HNRNPM seq: 544 - 550 no mod	[illegible]	p10092	Heterogeneous nuclear ribonucleoprotein M
GAPDH seq: 163 - 186 no mod	[illegible]	p01391	Glyceraldehyde-3-phosphate dehydrogenase
PTPN11 seq: 583 - 594 + Phospho (Y)	[illegible]	p00537	Tyrosine-protein phosphatase non-receptor type 11
NAP1L4 seq: 108 - 138 + Oxidation (M); Phospho (ST)	[illegible]	p17797	Nucleosome assembly protein 1-like 4
MYH9 seq: 1677 - 1694 no mod	[illegible]	p04908	Myosin-9
U2SURP pS37	[illegible]	p03927	U2 snRNP-associated SURP motif-containing protein
AHNAK2 pS593	[illegible]	p13853	Protein AHNAK2
HNRNPK seq: 423 - 433 no mod	[illegible]	p14439	Heterogeneous nuclear ribonucleoprotein K
NAA10 seq: 199 - 235 + 2 Phospho (ST)	[illegible]	p15788	N-alpha-acetyltransferase 10
SCRIB pS1541 pT1545 pS1547	[illegible]	p12281	Protein scribble homolog
KMT2D seq: 2289 - 2322 + Phospho (ST)	[illegible]	p13305	Histone-lysine N-methyltransferase 2D
SPAG1 pS347	[illegible]	p09830	Sperm-associated antigen 1
SVIL pS221	[illegible]	p08855	Supervillin
MAP1B seq: 1394 - 1423 + Phospho (ST)	[illegible]	p01161	Microtubule-associated protein 1B
CHORDC1 seq: 45 - 61 + Phospho (ST)	[illegible]	p06708	Cysteine and histidine-rich domain-containing protein 1
STAU2 pS455	[illegible]	p18486	Double-stranded RNA-binding protein Staufen homolog 2
PXN pS126	[illegible]	p06354	Paxillin
KRT18 seq: 265 - 301 no mod	[illegible]	p17832	Keratin, type I cytoskeletal 18
CRKL seq: 190 - 241 + Phospho (ST)	[illegible]	p14801	Crk-like protein
GPS1 pS474	[illegible]	p17675	COP9 signalosome complex subunit 1
RICTOR seq: 1217 - 1238 + Phospho (ST)	[illegible]	p04200	Rapamycin-insensitive companion of mTOR
GIT1 seq: 373 - 398 + Phospho (ST)	[illegible]	p04213	ARF GTPase-activating protein GIT1
RPRD2 pS374	[illegible]	p18118	Regulation of nuclear pre-mRNA domain-containing protein 2
NDRG3 seq: 346 - 367 + Phospho (ST)	[illegible]	p03222	Protein NDRG3
TJP2 pS413 pS415	[illegible]	p05826	Tight junction protein ZO-2
SH3KBP1 seq: 177 - 208 + Phospho (ST)	[illegible]	p17035	SH3 domain-containing kinase-binding protein 1
DOCK7 seq: 896 - 916 + Phospho (ST)	[illegible]	p05036	Dedicator of cytokinesis protein 7
TNRC6B seq: 1488 - 1523 + Phospho (ST)	[illegible]	p15860	Trinucleotide repeat-containing gene 6B protein
PTGFRN pS875	[illegible]	p07166	Prostaglandin F2 receptor negative regulator
RPL32 pM90	[illegible]	p17496	60S ribosomal protein L32
LASP1 seq: 209 - 246 no mod	[illegible]	p19142	LIM and SH3 domain protein 1
HDAC1 pS421 pS423	[illegible]	p14534	Histone deacetylase 1
CTNND1 seq: 320 - 344 + Phospho (Y)	[illegible]	p03166	Catenin delta-1
RNF19A seq: 627 - 643 + Phospho (ST)	[illegible]	p16505	E3 ubiquitin-protein ligase RNF19A
NFIX seq: 280 - 300 + Phospho (ST)	[illegible]	p05537	Nuclear factor 1 X-type
PSMD2 seq: 457 - 478 no mod	[illegible]	p09691	26S proteasome non-ATPase regulatory subunit 2
TBC1D4 seq: 749 - 768 + 2 Phospho (ST)	[illegible]	p02089	TBC1 domain family member 4
SPAG9 seq: 728 - 746 + 2 Phospho (ST)	[illegible]	p06234	C-Jun-amino-terminal kinase-interacting protein 4
PRPF4B pS20 pS23	[illegible]	p17270	Serine/threonine-protein kinase PRP4 homolog
SNRPGP15 seq: 26 - 32 no mod	[illegible]	p14559	Putative small nuclear ribonucleoprotein G-like protein 15
CYFIP2 seq: 754 - 760 no mod	[illegible]	p14558	Cytoplasmic FMR1-interacting protein 2
MDH2 seq: 258 - 269 + Oxidation (M)	[illegible]	p16397	Malate dehydrogenase, mitochondrial
SLBP seq: 192 - 241 + Phospho (ST)	[illegible]	p01797	Histone RNA hairpin-binding protein
DNTTIP2 seq: 78 - 105 + 2 Phospho (ST)	[illegible]	p15101	Deoxynucleotidyltransferase terminal-interacting protein 2
GORASP2 pT222	[illegible]	p13714	Golgi reassembly-stacking protein 2
KLF16 seq: 224 - 252 + Phospho (ST)	[illegible]	p03442	Krueppel-like factor 16
FKBP15 seq: 975 - 1007 + Phospho (ST)	[illegible]	p17224	FK506-binding protein 15
DGCR14 pS391 pS395	[illegible]	p15641	Protein DGCR14
GOLGA4 seq: 2146 - 2157 + Phospho (Y)	[illegible]	p08933	Golgin subfamily A member 4
PSAP seq: 405 - 414 no mod	[illegible]	p18828	Prosaposin
RNF113A pS84 pS85	[illegible]	p20967	RING finger protein 113A
ERF seq: 515 - 528 + Phospho (ST)	[illegible]	p16164	ETS domain-containing transcription factor ERF
IWS1 seq: 435 - 451 + 2 Phospho (ST)	[illegible]	p02727	Protein IWS1 homolog
PLS3 seq: 289 - 297 no mod	[illegible]	p13906	Plastin-3
NCAPH seq: 21 - 32 + Phospho (ST)	[illegible]	p15928	Condensin complex subunit 2
LMO7 seq: 1195 - 1222 + Phospho (ST)	[illegible]	p06719	LIM domain only protein 7
RRP36 pS73	[illegible]	p08263	Ribosomal RNA processing protein 36 homolog
HMGCS1 seq: 469 - 498 + Phospho (ST)	[illegible]	p06830	Hydroxymethylglutaryl-CoA synthase, cytoplasmic
MLLT6 seq: 361 - 397 + Phospho (ST)	[illegible]	p11938	Protein AF-17
seq: 189 - 213 + Gln->pyro-Glu (N-term Q); Phospho (ST)	[illegible]	p08000	Signal transducing adapter molecule 1
IRF2BPL seq: 655 - 670 + 3 Phospho (ST)	[illegible]	p07009	Interferon regulatory factor 2-binding protein-like
FH pM151	[illegible]	p11203	Fumarate hydratase, mitochondrial
GOLGA5 pS116	[illegible]	p13038	Golgin subfamily A member 5
HSPB1 pS65	[illegible]	p11127	Heat shock protein beta-1
PAK2 seq: 53 - 62 no mod	[illegible]	p14138	Serine/threonine-protein kinase PAK 2
GNG7 seq: 13 - 18 no mod	[illegible]	p10437	Guanine nucleotide-binding protein G(I)/G(S)/G(O) subunit gamma-7
KDM5A pS1423	[illegible]	p12561	Lysine-specific demethylase 5A
MEAF6 seq: 115 - 154 + Phospho (ST)	[illegible]	p17309	Chromatin modification-related protein MEAF6
DCAF6 pS336	[illegible]	p18831	DDB1- and CUL4-associated factor 6
PFN1 seq: 92 - 105 + Phospho (ST)	[illegible]	p03523	Profilin-1
DOCK7 seq: 855 - 881 + Phospho (ST)	[illegible]	p11101	Dedicator of cytokinesis protein 7
FUBP1 pT153	[illegible]	p06146	Far upstream element-binding protein 1
CLIC6 seq: 333 - 350 no mod	[illegible]	p13244	Chloride intracellular channel protein 6
ATXN1L seq: 61 - 84 + Phospho (ST)	[illegible]	p00827	Ataxin-1-like
TP53BP2 pS458	[illegible]	p00896	Apoptosis-stimulating of p53 protein 2
GIT1 seq: 373 - 398 + 2 Phospho (ST)	[illegible]	p04214	ARF GTPase-activating protein GIT1
C2orf44 seq: 498 - 526 + Phospho (ST)	[illegible]	p17603	WD repeat and coiled-coil-containing protein C2orf44
RPLP2 seq: 62 - 94 + Phospho (ST)	[illegible]	p12166	60S acidic ribosomal protein P2
TPR seq: 1831 - 1857 + Phospho (ST)	[illegible]	p02230	Nucleoprotein TPR
FLNA pS1734	[illegible]	p16758	Filamin-A
FRYL pT1959	[illegible]	p04745	Protein furry homolog-like
HIRIP3 seq: 17 - 30 + Phospho (ST)	[illegible]	p15336	HIRA-interacting protein 3
PPP1R18 pS224	[illegible]	p10709	Phostensin
ARFGAP1 pT189	[illegible]	p00027	ADP-ribosylation factor GTPase-activating protein 1
LAD1 pS177	[illegible]	p15005	Ladinin-1
CASKIN2 seq: 906 - 943 + Phospho (ST)	[illegible]	p02558	Caskin-2
RRM2 seq: 6 - 41 + Phospho (ST)	[illegible]	p01005	Ribonucleoside-diphosphate reductase subunit M2
TPR pS2155	[illegible]	p02967	Nucleoprotein TPR
MCL1 seq: 137 - 176 + Phospho (ST)	[illegible]	p06964	Induced myeloid leukemia cell differentiation protein Mcl-1
ABCC2 seq: 865 - 904 + Phospho (ST)	[illegible]	p14590	Canalicular multispecific organic anion transporter 1
ARL6IP4 seq: 230 - 247 + Phospho (ST)	[illegible]	p19459	ADP-ribosylation factor-like protein 6-interacting protein 4
SHARPIN pS165	[illegible]	p04630	Sharpin
PPP1R16A seq: 351 - 357 + Phospho (ST)	[illegible]	p06436	Protein phosphatase 1 regulatory subunit 16A
CEP170 seq: 481 - 517 + Phospho (ST)	[illegible]	p09245	Centrosomal protein of 170 kDa
SMARCC1 seq: 470 - 478 no mod	[illegible]	p20209	SWI/SNF complex subunit SMARCC1
AHNAK seq: 103 - 123 + Phospho (Y)	[illegible]	p16988	Neuroblast differentiation-associated protein AHNAK
ARHGEF5 seq: 671 - 720 + Phospho (ST)	[illegible]	p08754	Rho guanine nucleotide exchange factor 5
ARHGEF5 seq: 671 - 720 + Phospho (Y)	[illegible]	p08753	Rho guanine nucleotide exchange factor 5
FLNC pS2233	[illegible]	p17229	Filamin-C
CUX1 seq: 1214 - 1245 + Phospho (ST)	[illegible]	p14601	Homeobox protein cut-like 1
HNRNPC pS233	[illegible]	p09708	Heterogeneous nuclear ribonucleoproteins C1/C2
ZFP36L2 seq: 436 - 481 + Phospho (ST)	[illegible]	p07145	Zinc finger protein 36, C3H1 type-like 2
GDI2 seq: 380 - 390 no mod	[illegible]	p16378	Rab GDP dissociation inhibitor beta
PCM1 pS93	[illegible]	p00212	Pericentriolar material 1 protein
FAM129A seq: 566 - 599 + Phospho (ST)	[illegible]	p14712	Protein Niban
GOLGB1 pS38	[illegible]	p19971	Golgin subfamily B member 1
HNRNPK seq: 38 - 46 no mod	[illegible]	p09036	Heterogeneous nuclear ribonucleoprotein K
TTLL12 seq: 15 - 40 + Phospho (ST)	[illegible]	p03809	Tubulin--tyrosine ligase-like protein 12
G6PD seq: 387 - 393 no mod	[illegible]	p07035	Glucose-6-phosphate 1-dehydrogenase
SNX3 seq: 11 - 43 + Phospho (ST)	[illegible]	p11546	Sorting nexin-3
RBM27 seq: 581 - 593 + Gln->pyro-Glu (N-term Q)	[illegible]	p08633	RNA-binding protein 27
PA2G4 seq: 377 - 394 no mod	[illegible]	p03098	Proliferation-associated protein 2G4
SNX3 seq: 11 - 43 + Phospho (Y)	[illegible]	p11545	Sorting nexin-3
HN1 seq: 86 - 141 + Phospho (ST)	[illegible]	p09086	Hematological and neurological expressed 1 protein
RANBP2 pS1509	[illegible]	p12643	E3 SUMO-protein ligase RanBP2
STK4 pS320	[illegible]	p17004	Serine/threonine-protein kinase 4
TRIM47 pS588	[illegible]	p07386	Tripartite motif-containing protein 47
DVL3 pS192	[illegible]	p06760	Segment polarity protein dishevelled homolog DVL-3
KDM2A seq: 739 - 753 + Phospho (ST)	[illegible]	p03817	Lysine-specific demethylase 2A
CCDC97 pS257	[illegible]	p11303	Coiled-coil domain-containing protein 97
MAPK3 pT202	[illegible]	p14524	Mitogen-activated protein kinase 3
WASF1 seq: 271 - 315 + Phospho (ST)	[illegible]	p01118	Wiskott-Aldrich syndrome protein family member 1
FRMD8 pS446	[illegible]	p08303	FERM domain-containing protein 8

Peptide / site	Intensities	Accession	Protein
GPHN seq: 178 - 203 + 2 Phospho (ST)	[illegible]	p16984	Gephyrin
CHAMP1 pS139 pS184	[illegible]	p11675	Chromosome alignment-maintaining phosphoprotein 1
PALLD pS893	[illegible]	p14475	Palladin
SNX1 pS32 pS39 pT41	[illegible]	p11098	Sorting nexin-1
MKL2 seq: 53 - 75 + Phospho (ST)	[illegible]	p17277	MKL/myocardin-like protein 2
SON seq: 1663 - 1677 no mod	[illegible]	p18754	Protein SON
ANXA1 seq: 30 - 53 + Phospho (Y)	[illegible]	p15993	Annexin A1
PPL seq: 189 - 195 no mod	[illegible]	p17271	Periplakin
SNX1 pS32 pS39 pT41	[illegible]	p11097	Sorting nexin-1
ACBD3 seq: 279 - 306 + Gln->pyro-Glu (N-term Q)	[illegible]	p08332	Golgi resident protein GCP60
ALDOA pS360	[illegible]	p00047	Fructose-bisphosphate aldolase A
EIF4G1 pS1185 pS1187	[illegible]	p05844	Eukaryotic translation initiation factor 4 gamma 1
HNRNPA2B1 seq: 130 - 147 no mod	[illegible]	p18050	Heterogeneous nuclear ribonucleoproteins A2/B1
G6PD seq: 82 - 89 no mod	[illegible]	p12651	Glucose-6-phosphate 1-dehydrogenase
FAM129B pS641 pS646	[illegible]	p07681	Niban-like protein 1
TP53BP1 pS993	[illegible]	p10350	Tumor suppressor p53-binding protein 1
PTPN23 seq: 1505 - 1559 + 2 Phospho (ST)	[illegible]	p10776	Tyrosine-protein phosphatase non-receptor type 23
ARHGEF12 seq: 742 - 775 + Phospho (ST)	[illegible]	p13257	Rho guanine nucleotide exchange factor 12
NQO1 seq: 78 - 90 no mod	[illegible]	p17744	NAD(P)H dehydrogenase [quinone] 1
GTSE1 seq: 238 - 246 + Phospho (ST)	[illegible]	p13291	G2 and S phase-expressed protein 1
IQCE seq: 128 - 141 + Phospho (ST)	[illegible]	p06253	IQ domain-containing protein E
MARCKSL1 seq: 111 - 144 no mod	[illegible]	p17752	MARCKS-related protein
KHSRP seq: 88 - 109 no mod	[illegible]	p14269	Far upstream element-binding protein 2
HTATSF1 pS642	[illegible]	p01575	HIV Tat-specific factor 1
FLNB seq: 2105 - 2139 + Phospho (ST)	[illegible]	p19873	Filamin-B
IRS2 seq: 760 - 796 + Phospho (Y)	[illegible]	p11331	Insulin receptor substrate 2
IRS2 seq: 760 - 796 + Phospho (ST)	[illegible]	p11332	Insulin receptor substrate 2
UBE4B seq: 76 - 102 + Phospho (ST)	[illegible]	p04167	Ubiquitin conjugation factor E4 B
FLNA seq: 1072 - 1087 no mod	[illegible]	p20774	Filamin-A
RPLP2 pS102 pS105	[illegible]	p18897	60S acidic ribosomal protein P2
FASN seq: 2429 - 2436 no mod	[illegible]	p21169	Fatty acid synthase
TPT1 seq: 124 - 130 no mod	[illegible]	p14764	Translationally-controlled tumor protein
HIST1H1B seq: 38 - 49 no mod	[illegible]	p19376	Histone H1.5
GOT1 seq: 61 - 81 + Phospho (Y)	[illegible]	p14495	Aspartate aminotransferase, cytoplasmic
RBM34 pS14	[illegible]	p03295	RNA-binding protein 34
YWHAE seq: 107 - 118 no mod	[illegible]	p14746	14-3-3 protein epsilon
RPS11 seq: 119 - 136 no mod	[illegible]	p18093	40S ribosomal protein S11
RPS19 seq: 28 - 38 no mod	[illegible]	p11473	40S ribosomal protein S19
NAP1L4 seq: 37 - 44 no mod	[illegible]	p01283	Nucleosome assembly protein 1-like 4
HSP90B2P seq: 151 - 160 no mod	[illegible]	p11665	Putative endoplasmin-like protein
HSP90B1 seq: 429 - 434 no mod	[illegible]	p00250	Endoplasmin
PDIA3 seq: 275 - 280 no mod	[illegible]	p15236	Protein disulfide-isomerase A3
DSP seq: 2606 - 2630 + Phospho (ST)	[illegible]	p03713	Desmoplakin
RPL12 seq: 32 - 40 no mod	[illegible]	p14249	60S ribosomal protein L12
SH3BP1 seq: 608 - 615 + 2 Phospho (ST)	[illegible]	p07062	SH3 domain-binding protein 1
FLNB seq: 1136 - 1147 no mod	[illegible]	p00654	Filamin-B
FASN seq: 71 - 80 no mod	[illegible]	p08851	Fatty acid synthase
RPS6 pS235 pS236	[illegible]	p06753	40S ribosomal protein S6
FAM47A pS258 pS259	[illegible]	p06717	Protein FAM47A
GAPDH seq: 201 - 215 no mod	[illegible]	p16292	Glyceraldehyde-3-phosphate dehydrogenase
YWHAZ seq: 86 - 91 no mod	[illegible]	p14329	14-3-3 protein zeta/delta
ABCA13 seq: 3080 - 3085 no mod	[illegible]	p10569	ATP-binding cassette sub-family A member 13
ENO1 seq: 407 - 412 no mod	[illegible]	p00199	Alpha-enolase
PKM seq: 490 - 498 no mod	[illegible]	p01086	Pyruvate kinase PKM
SSH1 seq: 982 - 1023 + Phospho (ST)	[illegible]	p11693	Protein phosphatase Slingshot homolog 1
SF3B2 seq: 460 - 466 no mod	[illegible]	p16406	Splicing factor 3B subunit 2
TFG pS197	[illegible]	p08960	Protein TFG
RPS11 seq: 13 - 20 no mod	[illegible]	p08094	40S ribosomal protein S11
NCL seq: 117 - 124 no mod	[illegible]	p20123	Nucleolin
ENO1 seq: 254 - 262 no mod	[illegible]	p05734	Alpha-enolase
LMNA seq: 528 - 541 no mod	[illegible]	p03080	Prelamin-A/C
PKM seq: 462 - 467 no mod	[illegible]	p08853	Pyruvate kinase PKM
RPL4 seq: 354 - 364 no mod	[illegible]	p21210	60S ribosomal protein L4
NPM1 seq: 268 - 273 no mod	[illegible]	p16678	Nucleophosmin
PICK1 seq: 14 - 22 no mod	[illegible]	p11756	PRKCA-binding protein
TKTL2 seq: 400 - 405 no mod	[illegible]	p20781	Transketolase-like protein 2
AHNAK seq: 585 - 593 no mod	[illegible]	p15946	Neuroblast differentiation-associated protein AHNAK
SAFB seq: 286 - 294 no mod	[illegible]	p20892	Scaffold attachment factor B1
SFRP2 seq: 285 - 291 + 2 Phospho (ST)	[illegible]	p07242	Secreted frizzled-related protein 2
SFPQ seq: 280 - 286 no mod	[illegible]	p20046	Splicing factor, proline- and glutamine-rich
FSIP2 seq: 5315 - 5321 no mod	[illegible]	p12220	Fibrous sheath-interacting protein 2
FAM98C seq: 82 - 88 no mod	[illegible]	p08380	Protein FAM98C
RPS19 seq: 103 - 111 no mod	[illegible]	p01192	40S ribosomal protein S19
HSPE1 pM32	[illegible]	p16040	10 kDa heat shock protein, mitochondrial
TKT seq: 103 - 114 no mod	[illegible]	p13668	Transketolase
CTTN seq: 162 - 171 no mod	[illegible]	p00335	Src substrate cortactin
CFL1 seq: 115 - 121 no mod	[illegible]	p10058	Cofilin-1
LDHB seq: 120 - 127 no mod	[illegible]	p16679	L-lactate dehydrogenase B chain
FASN seq: 523 - 533 no mod	[illegible]	p06114	Fatty acid synthase
PSMD1 seq: 130 - 136 no mod	[illegible]	p11985	26S proteasome non-ATPase regulatory subunit 1
FLNA seq: 2599 - 2607 no mod	[illegible]	p02413	Filamin-A
DPP3 seq: 460 - 466 no mod	[illegible]	p11847	Dipeptidyl peptidase 3
NUTF2 seq: 56 - 63 no mod	[illegible]	p10659	Nuclear transport factor 2
RAD23B seq: 213 - 220 no mod	[illegible]	p19622	UV excision repair protein RAD23 homolog B
P4HB seq: 445 - 452 no mod	[illegible]	p16782	Protein disulfide-isomerase
RUVBL1 seq: 34 - 46 no mod	[illegible]	p08875	RuvB-like 1
PCNA seq: 249 - 254 no mod	[illegible]	p00008	Proliferating cell nuclear antigen
TRIP4 seq: 257 - 264 no mod	[illegible]	p05727	Activating signal cointegrator 1
RPL21 seq: 64 - 78 no mod	[illegible]	p00524	60S ribosomal protein L21
RPL7A seq: 90 - 97 no mod	[illegible]	p07815	60S ribosomal protein L7a
MEX3D seq: 525 - 531 no mod	[illegible]	p11960	RNA-binding protein MEX3D
EIF4B seq: 357 - 372 no mod	[illegible]	p21042	Eukaryotic translation initiation factor 4B
KRT7 seq: 65 - 86 no mod	[illegible]	p16941	Keratin, type II cytoskeletal 7
FLNA seq: 1938 - 1951 no mod	[illegible]	p00208	Filamin-A
RPL35 seq: 57 - 66 no mod	[illegible]	p01128	60S ribosomal protein L35
ST13 seq: 5 - 10 no mod	[illegible]	p12420	Hsc70-interacting protein
PLEC seq: 1351 - 1364 no mod	[illegible]	p17185	Plectin
MUCL1 seq: 79 - 87 no mod	[illegible]	p00465	Mucin-like protein 1
XPNPEP3 seq: 149 - 155 no mod	[illegible]	p20463	Probable Xaa-Pro aminopeptidase 3
UPF2 seq: 607 - 613 no mod	[illegible]	p20263	Regulator of nonsense transcripts 2
HSP90AA1 seq: 339 - 345 no mod	[illegible]	p20237	Heat shock protein HSP 90-alpha
SETX seq: 2020 - 2025 no mod	[illegible]	p14160	Probable helicase senataxin
GCSAM seq: 51 - 56 no mod	[illegible]	p14035	Germinal center-associated signaling and motility protein
DNAJA1 seq: 320 - 325 no mod	[illegible]	p11592	DnaJ homolog subfamily A member 1
ASB7 seq: 135 - 140 no mod	[illegible]	p11361	Ankyrin repeat and SOCS box protein 7
TUBA1B seq: 85 - 96 no mod	[illegible]	p08387	Tubulin alpha-1B chain
TUBA4B seq: 24 - 35 no mod	[illegible]	p08525	Putative tubulin-like protein alpha-4B
RPL30 seq: 10 - 17 no mod	[illegible]	p05248	60S ribosomal protein L30
RPLP2 pS101 pS104 pM108	[illegible]	p13299	60S acidic ribosomal protein P2
CEP85 seq: 476 - 481 no mod	[illegible]	p14327	Centrosomal protein of 85 kDa
AHNAK seq: 2707 - 2720 no mod	[illegible]	p13706	Neuroblast differentiation-associated protein AHNAK
USP5 seq: 407 - 415 no mod	[illegible]	p16958	Ubiquitin carboxyl-terminal hydrolase 5
VCP seq: 296 - 312 no mod	[illegible]	p09758	Transitional endoplasmic reticulum ATPase
SFN seq: 149 - 160 no mod	[illegible]	p06164	14-3-3 protein sigma
RPS4X seq: 192 - 198 no mod	[illegible]	p14221	40S ribosomal protein S4, X isoform
H3F3A seq: 42 - 50 no mod	[illegible]	p00140	Histone H3.3
CFL1 pM74	[illegible]	p10021	Cofilin-1
PDCD6IP seq: 351 - 357 no mod	[illegible]	p16429	Programmed cell death 6-interacting protein
PKM seq: 448 - 455 no mod	[illegible]	p19979	Pyruvate kinase PKM
RPS11 seq: 72 - 79 no mod	[illegible]	p13988	40S ribosomal protein S11
HSPH1 seq: 621 - 629 no mod	[illegible]	p10068	Heat shock protein 105 kDa
GOT1 seq: 87 - 97 no mod	[illegible]	p12240	Aspartate aminotransferase, cytoplasmic
PLEC seq: 4004 - 4018 no mod	[illegible]	p18917	Plectin
GAPDH seq: 300 - 306 no mod	[illegible]	p10555	Glyceraldehyde-3-phosphate dehydrogenase
PUF60 seq: 244 - 251 no mod	[illegible]	p03352	Poly(U)-binding-splicing factor PUF60
LMNB1 seq: 277 - 290 no mod	[illegible]	p10672	Lamin-B1
RPL36A seq: 70 - 78 no mod	[illegible]	p12006	60S ribosomal protein L36a
CCT5 seq: 345 - 352 no mod	[illegible]	p16483	T-complex protein 1 subunit epsilon
PKM seq: 126 - 136 no mod	[illegible]	p15272	Pyruvate kinase PKM
KRT19 pM291	[illegible]	p17021	Keratin, type I cytoskeletal 19
PGK1 seq: 142 - 151 no mod	[illegible]	p20818	Phosphoglycerate kinase 1
HSPA8 pM237	[illegible]	p09819	Heat shock cognate 71 kDa protein
PLEC seq: 4600 - 4609 no mod	[illegible]	p11439	Plectin
PDAP1 seq: 96 - 105 no mod	[illegible]	p00688	28 kDa heat- and acid-stable phosphoprotein
PRDX2 seq: 128 - 135 no mod	[illegible]	p15752	Peroxiredoxin-2
EEF1G seq: 401 - 414 no mod	[illegible]	p13014	Elongation factor 1-gamma
ANXA1 seq: 229 - 234 no mod	[illegible]	p03150	Annexin A1
SSFA2 pT1140	[illegible]	p05338	Sperm-specific antigen 2
PGK1 seq: 366 - 382 no mod	[illegible]	p16242	Phosphoglycerate kinase 1
HSPH1 seq: 354 - 360 no mod	[illegible]	p14502	Heat shock protein 105 kDa
RPL7A seq: 138 - 150 no mod	[illegible]	p20543	60S ribosomal protein L7a
SEPT11 seq: 176 - 184 no mod	[illegible]	p01072	Septin-11
SEPT14 seq: 187 - 195 no mod	[illegible]	p01071	Septin-14
FLNB seq: 837 - 847 no mod	[illegible]	p01286	Filamin-B
RPL7 seq: 10 - 20 no mod	[illegible]	p16962	60S ribosomal protein L7
EEF2 pS502	[illegible]	p16435	Elongation factor 2
HSPA5 seq: 377 - 386 no mod	[illegible]	p17803	78 kDa glucose-regulated protein
TKT seq: 344 - 353 no mod	[illegible]	p09061	Transketolase
CFL2 pM115	[illegible]	p10057	Cofilin-2
RPL13 seq: 75 - 82 no mod	[illegible]	p16093	60S ribosomal protein L13
PSMA1 seq: 197 - 208 no mod	[illegible]	p17057	Proteasome subunit alpha type-1
TKT pM604	[illegible]	p10143	Transketolase
NUDC seq: 257 - 267 no mod	[illegible]	p10345	Nuclear migration protein nudC
ALDOC seq: 209 - 215 no mod	[illegible]	p01282	Fructose-bisphosphate aldolase C
ATP13A1 seq: 685 - 691 no mod	[illegible]	p01279	Manganese-transporting ATPase 13A1
ADIRF seq: 57 - 71 no mod	[illegible]	p03071	Adipogenesis regulatory factor
HSPA8 seq: 602 - 609 no mod	[illegible]	p01714	Heat shock cognate 71 kDa protein
NONO seq: 6 - 11 no mod	[illegible]	p02859	Non-POU domain-containing octamer-binding protein
FAM186A seq: 107 - 112 no mod	[illegible]	p02487	Protein FAM186A
KRT18 seq: 176 - 186 + Gln->pyro-Glu (N-term Q)	[illegible]	p07818	Keratin, type I cytoskeletal 18
ERP44 seq: 390 - 396 no mod	[illegible]	p12209	Endoplasmic reticulum resident protein 44
COTL1 seq: 103 - 110 no mod	[illegible]	p16932	Coactosin-like protein
UBA1 seq: 90 - 97 no mod	[illegible]	p09549	Ubiquitin-like modifier-activating enzyme 1
MYH11 seq: 1814 - 1822 no mod	[illegible]	p03516	Myosin-11
ABCB6 seq: 764 - 771 no mod	[illegible]	p20438	ATP-binding cassette sub-family B member 6, mitochondrial
MYH9 seq: 1807 - 1815 no mod	[illegible]	p19595	Myosin-9
MYH10 seq: 1814 - 1822 no mod	[illegible]	p19368	Myosin-10
CALML5 seq: 106 - 124 no mod	[illegible]	p20100	Calmodulin-like protein 5
EIF2B1 seq: 103 - 108 + 2 Phospho (ST)	[illegible]	p07248	Translation initiation factor eIF-2B subunit alpha
ZC3H3 pS569 pS571	[illegible]	p07115	Zinc finger CCCH domain-containing protein 3
POLH pS379 pS380	[illegible]	p07086	DNA polymerase eta
ZNF75A seq: 257 - 262 + 2 Phospho (ST)	[illegible]	p06608	Zinc finger protein 75A
SRSF3 seq: 29 - 37 no mod	[illegible]	p20766	Serine/arginine-rich splicing factor 3
FLNA seq: 2576 - 2584 no mod	[illegible]	p03905	Filamin-A
CTSB seq: 80 - 87 no mod	[illegible]	p11140	Cathepsin B
CLIC1 seq: 21 - 29 no mod	[illegible]	p14253	Chloride intracellular channel protein 1
ENO1 seq: 82 - 92 no mod	[illegible]	p11144	Alpha-enolase
RPL12 seq: 84 - 90 no mod	[illegible]	p20240	60S ribosomal protein L12
EEF1A2 seq: 256 - 266 no mod	[illegible]	p14263	Elongation factor 1-alpha 2
HNRNPA2B1 pM193	[illegible]	p08730	Heterogeneous nuclear ribonucleoproteins A2/B1
PLEC seq: 1933 - 1942 no mod	[illegible]	p16506	Plectin
MYH9 seq: 1662 - 1669 no mod	[illegible]	p17839	Myosin-9
EEF1A1P5 seq: 42 - 51 no mod	[illegible]	p16794	Putative elongation factor 1-alpha-like 3
SRSF6 seq: 146 - 154 no mod	[illegible]	p02490	Serine/arginine-rich splicing factor 6
DDX5 seq: 57 - 67 no mod	[illegible]	p09616	Probable ATP-dependent RNA helicase DDX5
AHNAK seq: 2695 - 2703 no mod	[illegible]	p16648	Neuroblast differentiation-associated protein AHNAK
RPS26 seq: 52 - 66 no mod	[illegible]	p18728	40S ribosomal protein S26
TPI1 seq: 244 - 256 no mod	[illegible]	p14121	Triosephosphate isomerase
HMGB1P1 seq: 77 - 86 no mod	[illegible]	p01869	Putative high mobility group protein B1-like 1
KRT19 seq: 25 - 32 no mod	[illegible]	p16745	Keratin, type I cytoskeletal 19
HIST1H2AC seq: 83 - 89 no mod	[illegible]	p14737	Histone H2A type 1-C
ENO3 seq: 344 - 358 no mod	[illegible]	p01052	Beta-enolase
ENO1 seq: 344 - 358 no mod	[illegible]	p01050	Alpha-enolase
CBX3 seq: 45 - 50 no mod	[illegible]	p01581	Chromobox protein homolog 3
ST13 seq: 18 - 29 no mod	[illegible]	p08759	Hsc70-interacting protein
PSMD2 seq: 358 - 369 no mod	[illegible]	p16756	26S proteasome non-ATPase regulatory subunit 2
TPM4 seq: 191 - 197 no mod	[illegible]	p11295	Tropomyosin alpha-4 chain
TTN seq: 19134 - 19141 no mod	[illegible]	p10778	Titin
HSP90AA1 seq: 315 - 327 no mod	[illegible]	p04949	Heat shock protein HSP 90-alpha
CLIC1 seq: 209 - 216 no mod	[illegible]	p00234	Chloride intracellular channel protein 1
HIST1H1B seq: 68 - 78 no mod	[illegible]	p20337	Histone H1.5
ESPNL seq: 602 - 608 no mod	[illegible]	p05043	Espin-like protein
CCT6A seq: 371 - 377 no mod	[illegible]	p03226	T-complex protein 1 subunit zeta
NUCB2 seq: 114 - 119 no mod	[illegible]	p10880	Nucleobindin-2
NUCB1 seq: 111 - 116 no mod	[illegible]	p10879	Nucleobindin-1
TTN seq: 12999 - 13004 no mod	[illegible]	p07059	Titin
PSMA5 seq: 197 - 203 no mod	[illegible]	p03852	Proteasome subunit alpha type-5
VCP seq: 278 - 288 no mod	[illegible]	p12263	Transitional endoplasmic reticulum ATPase
RPL8 seq: 31 - 37 no mod	[illegible]	p19264	60S ribosomal protein L8
GAPDH seq: 201 - 215 no mod	[illegible]	p16293	Glyceraldehyde-3-phosphate dehydrogenase

SRSF4 seq: 106 - 114 no mod	[illegible]	p11539	Serine/arginine-rich splicing factor 4
G6PD seq: 220 - 227 no mod	[illegible]	p14309	Glucose-6-phosphate 1-dehydrogenase
HSPA8 seq: 113 - 126 no mod	[illegible]	p05798	Heat shock cognate 71 kDa protein
HIST1H2AH seq: 22 - 30 no mod	[illegible]	p20630	Histone H2A type 1-H
RPS8 seq: 185 - 193 no mod	[illegible]	p20917	40S ribosomal protein S8
ALDOA seq: 29 - 42 no mod	[illegible]	p15890	Fructose-bisphosphate aldolase A
HSPA1A seq: 172 - 187 no mod	[illegible]	p14154	Heat shock 70 kDa protein 1A
FLNB seq: 1331 - 1339 no mod	[illegible]	p00970	Filamin-B
HINT3 seq: 121 - 130 no mod	[illegible]	p09341	Histidine triad nucleotide-binding protein 3
NCL seq: 430 - 437 no mod	[illegible]	p15907	Nucleolin
RPL27A seq: 95 - 105 no mod	[illegible]	p02828	60S ribosomal protein L27a
ALDOA seq: 305 - 312 no mod	[illegible]	p20197	Fructose-bisphosphate aldolase A
ACTC1 seq: 85 - 95 no mod	[illegible]	p13509	Actin, alpha cardiac muscle 1
MYH9 seq: 1923 - 1932 no mod	[illegible]	p07400	Myosin-9
AHNAK2 pS1112	[illegible]	p12072	Protein AHNAK2
RPS19 seq: 134 - 143 no mod	[illegible]	p14512	40S ribosomal protein S19
RPL26L1 seq: 78 - 84 no mod	[illegible]	p00023	60S ribosomal protein L26-like 1
SH3BGRL3 seq: 6 - 15 no mod	[illegible]	p00521	SH3 domain-binding glutamic acid-rich-like protein 3
CORO1C seq: 382 - 391 no mod	[illegible]	p09790	Coronin-1C
PDIA4 seq: 110 - 119 no mod	[illegible]	p18691	Protein disulfide-isomerase A4
HNRNPCL4 seq: 18 - 29 no mod	[illegible]	p01548	Heterogeneous nuclear ribonucleoprotein C-like 4
CTTN seq: 209 - 218 no mod	[illegible]	p06175	Src substrate cortactin
ABCA13 seq: 272 - 277 no mod	[illegible]	p00911	ATP-binding cassette sub-family A member 13
PRKCSH seq: 168 - 177 no mod	[illegible]	p05267	Glucosidase 2 subunit beta
HSPA2 seq: 104 - 109 no mod	[illegible]	p00915	Heat shock-related 70 kDa protein 2
TKT seq: 531 - 542 no mod	[illegible]	p01255	Transketolase
CLTC seq: 1435 - 1441 no mod	[illegible]	p19218	Clathrin heavy chain 1
ACTG1 seq: 318 - 328 no mod	[illegible]	p17593	Actin, cytoplasmic 2
PRKCSH seq: 143 - 149 no mod	[illegible]	p14036	Glucosidase 2 subunit beta
USP5 seq: 134 - 144 no mod	[illegible]	p18816	Ubiquitin carboxyl-terminal hydrolase 5
PLEC seq: 4590 - 4599 no mod	[illegible]	p04862	Plectin
TPI1 seq: 198 - 212 no mod	[illegible]	p00610	Triosephosphate isomerase
THOC3 seq: 70 - 77 no mod	[illegible]	p12187	THO complex subunit 3
HSP90AB1 seq: 315 - 327 no mod	[illegible]	p04950	Heat shock protein HSP 90-beta
PGAM2 pM243	[illegible]	p20095	Phosphoglycerate mutase 2
PLEC seq: 4147 - 4158 no mod	[illegible]	p19166	Plectin
NPC2 seq: 117 - 123 no mod	[illegible]	p05939	Epididymal secretory protein E1
MIPEP seq: 305 - 312 no mod	[illegible]	p20193	Mitochondrial intermediate peptidase
FLNA seq: 2541 - 2559 no mod	[illegible]	p19409	Filamin-A
PGK1 seq: 193 - 199 no mod	[illegible]	p17438	Phosphoglycerate kinase 1
HUWE1 seq: 2372 - 2400 + Phospho (ST)	[illegible]	p05774	E3 ubiquitin-protein ligase HUWE1
CCT6B seq: 46 - 55 no mod	[illegible]	p09990	T-complex protein 1 subunit zeta-2
HSPH1 seq: 706 - 714 no mod	[illegible]	p16521	Heat shock protein 105 kDa
LDHB seq: 78 - 91 no mod	[illegible]	p13572	L-lactate dehydrogenase B chain
CCT2 seq: 224 - 230 no mod	[illegible]	p14219	T-complex protein 1 subunit beta
HIST1H1E seq: 35 - 46 no mod	[illegible]	p19612	Histone H1.4
RBBP4 seq: 60 - 65 no mod	[illegible]	p18856	Histone-binding protein RBBP4
HSPA8 seq: 113 - 126 no mod	[illegible]	p05799	Heat shock cognate 71 kDa protein
PABPC3 seq: 232 - 240 no mod	[illegible]	p16123	Polyadenylate-binding protein 3
TPI1 seq: 180 - 186 no mod	[illegible]	p00627	Triosephosphate isomerase
HNRNPU seq: 566 - 572 no mod	[illegible]	p16692	Heterogeneous nuclear ribonucleoprotein U
PRKCSH seq: 383 - 392 no mod	[illegible]	p05180	Glucosidase 2 subunit beta
PPIA seq: 20 - 31 no mod	[illegible]	p00866	Peptidyl-prolyl cis-trans isomerase A
LMNA seq: 79 - 89 no mod	[illegible]	p20969	Prelamin-A/C
EEF1A1P5 seq: 173 - 179 no mod	[illegible]	p16945	Putative elongation factor 1-alpha-like 3
NCL seq: 363 - 370 no mod	[illegible]	p20276	Nucleolin
YWHAG seq: 133 - 143 no mod	[illegible]	p00274	14-3-3 protein gamma
SKP1 seq: 114 - 121 no mod	[illegible]	p15711	S-phase kinase-associated protein 1
EEF1D seq: 220 - 232 no mod	[illegible]	p05484	Elongation factor 1-delta
ENO1 seq: 222 - 228 no mod	[illegible]	p17729	Alpha-enolase
TUBB4B pM73	[illegible]	p19220	Tubulin beta-4B chain
ATP5B seq: 110 - 121 no mod	[illegible]	p02733	ATP synthase subunit beta, mitochondrial
PSPC1 seq: 135 - 143 no mod	[illegible]	p20827	Paraspeckle component 1
RPSA seq: 121 - 128 no mod	[illegible]	p11249	40S ribosomal protein SA
EEF1D seq: 96 - 107 no mod	[illegible]	p14964	Elongation factor 1-delta
EVPL seq: 44 - 50 no mod	[illegible]	p17980	Envoplakin
LAP3 seq: 283 - 294 no mod	[illegible]	p15838	Cytosol aminopeptidase
KRT8 seq: 253 - 264 no mod	[illegible]	p05293	Keratin, type II cytoskeletal 8
NCL seq: 555 - 561 no mod	[illegible]	p11959	Nucleolin
RPS25 seq: 67 - 76 no mod	[illegible]	p11543	40S ribosomal protein S25
BUD13 pT159	[illegible]	p14851	BUD13 homolog
HSPD1 pM356	[illegible]	p18924	60 kDa heat shock protein, mitochondrial
RPL23 seq: 114 - 123 no mod	[illegible]	p15314	60S ribosomal protein L23
DLD seq: 496 - 505 no mod	[illegible]	p17933	Dihydrolipoyl dehydrogenase, mitochondrial
HNRNPH1 seq: 82 - 87 no mod	[illegible]	p00028	Heterogeneous nuclear ribonucleoprotein H
MARCKSL1 seq: 145 - 157 no mod	[illegible]	p21195	MARCKS-related protein
PFN1 seq: 128 - 136 no mod	[illegible]	p19002	Profilin-1
HSPA1A seq: 329 - 342 no mod	[illegible]	p19801	Heat shock 70 kDa protein 1A
CTSD seq: 314 - 331 no mod	[illegible]	p20476	Cathepsin D
CAPN14 seq: 484 - 489 no mod	[illegible]	p06032	Calpain-14
PRDX1 seq: 111 - 120 no mod	[illegible]	p02729	Peroxiredoxin-1
NME2 pM75	[illegible]	p01109	Nucleoside diphosphate kinase B
KRT38 seq: 395 - 405 no mod	[illegible]	p02227	Keratin, type I cuticular Ha8
MANF seq: 40 - 45 no mod	[illegible]	p16362	Mesencephalic astrocyte-derived neurotrophic factor
ADH5 seq: 160 - 168 no mod	[illegible]	p14402	Alcohol dehydrogenase class-3
YWHAE seq: 107 - 118 no mod	[illegible]	p14745	14-3-3 protein epsilon
PPIB seq: 46 - 51 no mod	[illegible]	p00535	Peptidyl-prolyl cis-trans isomerase B
P4HB seq: 453 - 461 no mod	[illegible]	p01962	Protein disulfide-isomerase
KIAA1614 seq: 582 - 588 no mod	[illegible]	p11340	Uncharacterized protein KIAA1614
HNRNPH3 seq: 68 - 76 no mod	[illegible]	p17635	Heterogeneous nuclear ribonucleoprotein H3
ACTB seq: 331 - 337 no mod	[illegible]	p14197	Actin, cytoplasmic 1
TOPORS seq: 89 - 118 + Phospho (ST)	[illegible]	p10940	E3 ubiquitin-protein ligase Topors
GAPDH pM130	[illegible]	p01376	Glyceraldehyde-3-phosphate dehydrogenase
CFL1 pM18	[illegible]	p01541	Cofilin-1
MTMR12 pS564	[illegible]	p08301	Myotubularin-related protein 12
PDCD6IP seq: 270 - 276 no mod	[illegible]	p16767	Programmed cell death 6-interacting protein
CSE1L seq: 825 - 832 no mod	[illegible]	p14166	Exportin-2
EPS15L1 pS49	[illegible]	p05721	Epidermal growth factor receptor substrate 15-like 1
ALYREF seq: 145 - 155 no mod	[illegible]	p05207	THO complex subunit 4
TUBB seq: 283 - 297 no mod	[illegible]	p20124	Tubulin beta chain
GSTO1 seq: 31 - 37 no mod	[illegible]	p16823	Glutathione S-transferase omega-1
IDH1 seq: 302 - 314 no mod	[illegible]	p01972	Isocitrate dehydrogenase [NADP] cytoplasmic
CCT6B seq: 296 - 307 no mod	[illegible]	p15905	T-complex protein 1 subunit zeta-2
ANXA11 seq: 462 - 470 no mod	[illegible]	p05961	Annexin A11
NUCB2 seq: 34 - 44 no mod	[illegible]	p00942	Nucleobindin-2
LINC00479 seq: 36 - 42 no mod	[illegible]	p11378	Putative uncharacterized protein encoded by LINC00479
VCP seq: 506 - 512 no mod	[illegible]	p16749	Transitional endoplasmic reticulum ATPase
PTBP1 seq: 445 - 471 no mod	[illegible]	p09634	Polypyrimidine tract-binding protein 1
DENR seq: 23 - 30 no mod	[illegible]	p12099	Density-regulated protein
MYDGF seq: 101 - 109 no mod	[illegible]	p03157	Myeloid-derived growth factor
CCT6A seq: 296 - 307 no mod	[illegible]	p15906	T-complex protein 1 subunit zeta
TMSB4X seq: 27 - 39 no mod	[illegible]	p09268	Thymosin beta-4
LMNB1 seq: 80 - 90 no mod	[illegible]	p20108	Lamin-B1
DECR1 seq: 235 - 244 no mod	[illegible]	p16546	2,4-dienoyl-CoA reductase, mitochondrial
PSMA7 seq: 175 - 189 no mod	[illegible]	p08906	Proteasome subunit alpha type-7
DAZAP1 seq: 79 - 88 no mod	[illegible]	p09571	DAZ-associated protein 1
PFN1 seq: 39 - 54 no mod	[illegible]	p02835	Profilin-1
TPT1 seq: 101 - 110 no mod	[illegible]	p15811	Translationally-controlled tumor protein
RPS12 seq: 85 - 93 no mod	[illegible]	p11794	40S ribosomal protein S12
PGK2 seq: 157 - 171 no mod	[illegible]	p11806	Phosphoglycerate kinase 2
PSMA4 seq: 9 - 17 no mod	[illegible]	p02048	Proteasome subunit alpha type-4
HDLBP seq: 483 - 494 no mod	[illegible]	p14358	Vigilin
EXPH5 pM1123 pS1124	[illegible]	p20079	Exophilin-5
CORO1B seq: 226 - 233 no mod	[illegible]	p20479	Coronin-1B
LASP1 seq: 11 - 17 no mod	[illegible]	p13515	LIM and SH3 domain protein 1
HNRNPF seq: 193 - 200 no mod	[illegible]	p03146	Heterogeneous nuclear ribonucleoprotein F
TRAPPC8 seq: 954 - 982 + Phospho (ST)	[illegible]	p06949	Trafficking protein particle complex subunit 8
RPL12 seq: 17 - 31 no mod	[illegible]	p19011	60S ribosomal protein L12
KRT19 seq: 371 - 381 no mod	[illegible]	p04104	Keratin, type I cytoskeletal 19
S100A11 seq: 4 - 12 no mod	[illegible]	p13653	Protein S100-A11
U2AF1 seq: 54 - 66 no mod	[illegible]	p09256	Splicing factor U2AF 35 kDa subunit
NME2 seq: 129 - 137 no mod	[illegible]	p06163	Nucleoside diphosphate kinase B
POTEE seq: 21 - 30 no mod	[illegible]	p20676	POTE ankyrin domain family member E
NPM1 seq: 251 - 257 no mod	[illegible]	p09941	Nucleophosmin
RPL13 seq: 93 - 101 no mod	[illegible]	p02711	60S ribosomal protein L13
UBA1 seq: 628 - 635 no mod	[illegible]	p05504	Ubiquitin-like modifier-activating enzyme 1
ANP32C seq: 76 - 86 no mod	[illegible]	p00861	Acidic leucine-rich nuclear phosphoprotein 32 family member C
STMN1 seq: 28 - 41 no mod	[illegible]	p05425	Stathmin
HNRNPM seq: 571 - 578 no mod	[illegible]	p10134	Heterogeneous nuclear ribonucleoprotein M
PPM1G seq: 210 - 220 no mod	[illegible]	p19095	Protein phosphatase 1G
PKM seq: 207 - 224 no mod	[illegible]	p13136	Pyruvate kinase PKM
MDH2 seq: 166 - 176 no mod	[illegible]	p14308	Malate dehydrogenase, mitochondrial
NME2P1 seq: 35 - 41 + Gln->pyro-Glu (N-term Q)	[illegible]	p08560	Putative nucleoside diphosphate kinase
G3BP2 seq: 278 - 290 no mod	[illegible]	p01644	Ras GTPase-activating protein-binding protein 2
YWHAB seq: 30 - 43 no mod	[illegible]	p19169	14-3-3 protein beta/alpha
ALB seq: 162 - 168 no mod	[illegible]	p00225	Serum albumin
SNRPD3 seq: 55 - 64 no mod	[illegible]	p01772	Small nuclear ribonucleoprotein Sm D3
ALB seq: 287 - 298 no mod	[illegible]	p00316	Serum albumin
PKM seq: 208 - 230 no mod	[illegible]	p15013	Pyruvate kinase PKM
RPS6 seq: 120 - 131 no mod	[illegible]	p18734	40S ribosomal protein S6
ATP5A1 seq: 262 - 270 no mod	[illegible]	p07068	ATP synthase subunit alpha, mitochondrial
GDI2 seq: 71 - 79 no mod	[illegible]	p18069	Rab GDP dissociation inhibitor beta
PGAM1 seq: 142 - 162 no mod	[illegible]	p00460	Phosphoglycerate mutase 1
ANXA2 seq: 314 - 324 no mod	[illegible]	p04919	Annexin A2
MYL6 seq: 95 - 107 no mod	[illegible]	p20256	Myosin light polypeptide 6
NCL seq: 72 - 79 no mod	[illegible]	p01738	Nucleolin
FUBP3 seq: 328 - 337 no mod	[illegible]	p18830	Far upstream element-binding protein 3
IMPDH2 seq: 182 - 195 no mod	[illegible]	p07491	Inosine-5'-monophosphate dehydrogenase 2
TUBB2A pM73	[illegible]	p20454	Tubulin beta-2A chain
ALB seq: 258 - 264 no mod	[illegible]	p10341	Serum albumin
TUBA4A seq: 340 - 352 no mod	[illegible]	p05488	Tubulin alpha-4A chain
HIST1H2BM seq: 59 - 73 no mod	[illegible]	p20093	Histone H2B type 1-M
PDIA3 seq: 95 - 104 no mod	[illegible]	p00334	Protein disulfide-isomerase A3
CNN2 seq: 135 - 145 no mod	[illegible]	p15662	Calponin-2
RPS3 seq: 228 - 243 no mod	[illegible]	p16035	40S ribosomal protein S3
GMPS seq: 425 - 435 no mod	[illegible]	p17486	GMP synthase [glutamine-hydrolyzing]
P4HB seq: 327 - 338 no mod	[illegible]	p00287	Protein disulfide-isomerase
EIF3G seq: 252 - 267 no mod	[illegible]	p17073	Eukaryotic translation initiation factor 3 subunit G
SNU13 seq: 10 - 20 no mod	[illegible]	p19118	NHP2-like protein 1
HNRNPA2B1 seq: 214 - 228 no mod	[illegible]	p16056	Heterogeneous nuclear ribonucleoproteins A2/B1
LSM14B seq: 154 - 172 + Oxidation (M); Phospho (ST)	[illegible]	p04527	Protein LSM14 homolog B
HNRNPF seq: 180 - 185 no mod	[illegible]	p00305	Heterogeneous nuclear ribonucleoprotein F
PDIA3 seq: 352 - 363 no mod	[illegible]	p16593	Protein disulfide-isomerase A3
SNRPN seq: 26 - 32 no mod	[illegible]	p14303	Small nuclear ribonucleoprotein-associated protein N
AHSG seq: 312 - 317 no mod	[illegible]	p20494	Alpha-2-HS-glycoprotein
MDH2 seq: 177 - 185 no mod	[illegible]	p20018	Malate dehydrogenase, mitochondrial
TUBB3 seq: 283 - 297 no mod	[illegible]	p20129	Tubulin beta-3 chain
SGTA seq: 138 - 150 no mod	[illegible]	p11724	Small glutamine-rich tetratricopeptide repeat-containing protein alpha
U2AF2 seq: 463 - 471 no mod	[illegible]	p00433	Splicing factor U2AF 65 kDa subunit
PLEC seq: 1428 - 1438 no mod	[illegible]	p19795	Plectin
LMNB1 seq: 146 - 157 no mod	[illegible]	p19001	Lamin-B1
ACTN3 seq: 96 - 103 no mod	[illegible]	p20307	Alpha-actinin-3
HSPA8 seq: 129 - 137 no mod	[illegible]	p17637	Heat shock cognate 71 kDa protein
KRT18 seq: 166 - 175 no mod	[illegible]	p01284	Keratin, type I cytoskeletal 18
EPS8 pS659	[illegible]	p08159	Epidermal growth factor receptor kinase substrate 8
PLEC seq: 2893 - 2903 no mod	[illegible]	p17656	Plectin
HIST1H2BH seq: 102 - 110 no mod	[illegible]	p11349	Histone H2B type 1-H
AHNAK seq: 2131 - 2141 no mod	[illegible]	p01318	Neuroblast differentiation-associated protein AHNAK
TKT seq: 233 - 241 no mod	[illegible]	p14671	Transketolase
B3GAT2 seq: 161 - 168 no mod	[illegible]	p09780	Galactosylgalactosylxylosylprotein 3-beta-glucuronosyltransferase 2
HNRNPA2B1 seq: 174 - 185 no mod	[illegible]	p00319	Heterogeneous nuclear ribonucleoproteins A2/B1
RPL22 seq: 53 - 65 no mod	[illegible]	p20609	60S ribosomal protein L22
HMGB1 seq: 115 - 127 no mod	[illegible]	p16161	High mobility group protein B1
RPL27 seq: 52 - 59 no mod	[illegible]	p12391	60S ribosomal protein L27
NIT2 seq: 158 - 168 no mod	[illegible]	p16873	Omega-amidase NIT2
ANXA5 seq: 30 - 45 no mod	[illegible]	p15721	Annexin A5
HIST1H2BN seq: 95 - 101 no mod	[illegible]	p17600	Histone H2B type 1-N
GNB2L1 seq: 258 - 264 no mod	[illegible]	p13512	Guanine nucleotide-binding protein subunit beta-2-like 1
ANXA2 seq: 11 - 28 no mod	[illegible]	p10767	Annexin A2
PGK1 seq: 107 - 123 no mod	[illegible]	p20960	Phosphoglycerate kinase 1
RPL7 seq: 10 - 19 no mod	[illegible]	p16963	60S ribosomal protein L7
RPL4 seq: 21 - 29 no mod	[illegible]	p08935	60S ribosomal protein L4
TUBA3E seq: 340 - 352 no mod	[illegible]	p02701	Tubulin alpha-3E chain
CFL1 seq: 153 - 166 no mod	[illegible]	p11771	Cofilin-1
F8 seq: 1012 - 1018 no mod	[illegible]	p00837	Coagulation factor VIII
KRT7 seq: 352 - 363 no mod	[illegible]	p20381	Keratin, type II cytoskeletal 7
CPNE3 seq: 476 - 484 no mod	[illegible]	p04561	Copine-3
CCT5 seq: 371 - 378 no mod	[illegible]	p09991	T-complex protein 1 subunit epsilon
CPA2 seq: 196 - 202 no mod	[illegible]	p13534	Carboxypeptidase A2
TUBA1A seq: 157 - 163 no mod	[illegible]	p10608	Tubulin alpha-1A chain

Peptide	Intensity values (approx. 60 columns)	Accession	Protein
MAP3K9 seq: 641 - 647 no mod	[illegible]	p04937	Mitogen-activated protein kinase kinase kinase 9
PLEKHJ1 seq: 7 - 13 no mod	[illegible]	p17421	Pleckstrin homology domain-containing family J member 1
TPT1 seq: 22 - 34 no mod	[illegible]	p17638	Translationally-controlled tumor protein
EIF5A seq: 40 - 47 no mod	[illegible]	p13558	Eukaryotic translation initiation factor 5A-1
CTTN seq: 337 - 346 no mod	[illegible]	p01929	Src substrate cortactin
NOS1AP seq: 208 - 235 + Phospho (ST)	[illegible]	p19454	Carboxyl-terminal PDZ ligand of neuronal nitric oxide synthase protein
EEF1A1 seq: 167 - 172 no mod	[illegible]	p00415	Elongation factor 1-alpha 1
PCNA seq: 211 - 217 no mod	[illegible]	p00251	Proliferating cell nuclear antigen
SMYD3 seq: 292 - 297 no mod	[illegible]	p13102	Histone-lysine N-methyltransferase SMYD3
EXOG seq: 457 - 462 no mod	[illegible]	p13007	Nuclease EXOG, mitochondrial
HSPE1 seq: 41 - 54 no mod	[illegible]	p01190	10 kDa heat shock protein, mitochondrial
APRT seq: 28 - 40 no mod	[illegible]	p18719	Adenine phosphoribosyltransferase
NCR3 seq: 77 - 84 no mod	[illegible]	p12212	Natural cytotoxicity triggering receptor 3
EIF5A seq: 56 - 67 no mod	[illegible]	p01418	Eukaryotic translation initiation factor 5A-1
AGR2 seq: 149 - 166 no mod	[illegible]	p10312	Anterior gradient protein 2 homolog
NME1 seq: 19 - 26 no mod	[illegible]	p15622	Nucleoside diphosphate kinase A
EEF2 seq: 513 - 519 no mod	[illegible]	p09282	Elongation factor 2
SF3B2 seq: 389 - 394 no mod	[illegible]	p14314	Splicing factor 3B subunit 2
ACTB pM192	[illegible]	p18523	Actin, cytoplasmic 1
SRRM2 seq: 1497 - 1508 + 3 Phospho (ST)	[illegible]	p04018	Serine/arginine repetitive matrix protein 2
KRT19 seq: 126 - 138 no mod	[illegible]	p18035	Keratin, type I cytoskeletal 19
GNB2L1 seq: 265 - 271 no mod	[illegible]	p14125	Guanine nucleotide-binding protein subunit beta-2-like 1
COTL1 seq: 111 - 117 no mod	[illegible]	p17778	Coactosin-like protein
GBA3 seq: 103 - 109 no mod	[illegible]	p14192	Cytosolic beta-glucosidase
ATP5B seq: 125 - 143 no mod	[illegible]	p01252	ATP synthase subunit beta, mitochondrial
FLNA seq: 2265 - 2280 no mod	[illegible]	p17972	Filamin-A
HSPA2 seq: 303 - 314 no mod	[illegible]	p19716	Heat shock-related 70 kDa protein 2
ITPR3 seq: 2611 - 2616 no mod	[illegible]	p13306	Inositol 1,4,5-trisphosphate receptor type 3
INVS seq: 595 - 600 no mod	[illegible]	p13287	Inversin
HSP90B1 seq: 88 - 95 no mod	[illegible]	p11588	Endoplasmin
FASN seq: 317 - 326 no mod	[illegible]	p08723	Fatty acid synthase
KRT8 seq: 317 - 325 no mod	[illegible]	p11050	Keratin, type II cytoskeletal 8
DES seq: 194 - 201 no mod	[illegible]	p11016	Desmin
RPS11 seq: 49 - 59 no mod	[illegible]	p17957	40S ribosomal protein S11
SRSF4 seq: 28 - 34 no mod	[illegible]	p14050	Serine/arginine-rich splicing factor 4
SRSF6 seq: 28 - 34 no mod	[illegible]	p11404	Serine/arginine-rich splicing factor 6
RPS7 seq: 42 - 49 no mod	[illegible]	p17440	40S ribosomal protein S7
RPL6 seq: 252 - 260 no mod	[illegible]	p19261	60S ribosomal protein L6
AHCY seq: 9 - 19 no mod	[illegible]	p01829	Adenosylhomocysteinase
ANP32B seq: 102 - 111 no mod	[illegible]	p18714	Acidic leucine-rich nuclear phosphoprotein 32 family member B
ANP32D seq: 102 - 111 no mod	[illegible]	p18526	Acidic leucine-rich nuclear phosphoprotein 32 family member D
ITSN2 seq: 953 - 960 no mod	[illegible]	p14147	Intersectin-2
SUCLG1 seq: 58 - 66 no mod	[illegible]	p14194	Succinyl-CoA ligase [ADP/GDP-forming] subunit alpha, mitochondrial
ATP5A1 seq: 254 - 261 no mod	[illegible]	p03406	ATP synthase subunit alpha, mitochondrial
SNRNP70 seq: 122 - 131 no mod	[illegible]	p17804	U1 small nuclear ribonucleoprotein 70 kDa
PEBP1 seq: 180 - 187 no mod	[illegible]	p10306	Phosphatidylethanolamine-binding protein 1
MSN seq: 28 - 35 no mod	[illegible]	p08390	Moesin
PSMD11 seq: 410 - 417 no mod	[illegible]	p00643	26S proteasome non-ATPase regulatory subunit 11
CDC37 seq: 84 - 92 no mod	[illegible]	p11056	Hsp90 co-chaperone Cdc37
RPS20 seq: 88 - 99 no mod	[illegible]	p11611	40S ribosomal protein S20
CALB2 seq: 152 - 161 no mod	[illegible]	p11004	Calretinin
MTHFD1 seq: 151 - 157 no mod	[illegible]	p16240	C-1-tetrahydrofolate synthase, cytoplasmic
HIST2H2BE seq: 36 - 44 no mod	[illegible]	p17084	Histone H2B type 2-E
ENO1 seq: 33 - 50 no mod	[illegible]	p20980	Alpha-enolase
RPL11 seq: 170 - 178 no mod	[illegible]	p00427	60S ribosomal protein L11
TBCA seq: 99 - 108 no mod	[illegible]	p10396	Tubulin-specific chaperone A
HIST3H2BB seq: 49 - 59 + Gln->pyro-Glu (N-term Q)	[illegible]	p07724	Histone H2B type 3-B
PGK1 seq: 354 - 361 no mod	[illegible]	p20222	Phosphoglycerate kinase 1
CAPNS2 seq: 159 - 165 no mod	[illegible]	p11792	Calpain small subunit 2
CALR seq: 99 - 111 no mod	[illegible]	p14840	Calreticulin
RANBP1 seq: 58 - 68 no mod	[illegible]	p16845	Ran-specific GTPase-activating protein
ATP5B seq: 427 - 432 no mod	[illegible]	p14005	ATP synthase subunit beta, mitochondrial
CTBP1 seq: 98 - 108 no mod	[illegible]	p14239	C-terminal-binding protein 1
LMNA seq: 42 - 48 no mod	[illegible]	p12136	Prelamin-A/C
KRT8 seq: 41 - 47 no mod	[illegible]	p01436	Keratin, type II cytoskeletal 8
PPIA seq: 20 - 31 no mod	[illegible]	p00865	Peptidyl-prolyl cis-trans isomerase A
OTUD7B seq: 98 - 111 + Phospho (ST)	[illegible]	p15850	OTU domain-containing protein 7B
NPM1 seq: 240 - 248 no mod	[illegible]	p15431	Nucleophosmin
ENO1 seq: 10 - 15 no mod	[illegible]	p17628	Alpha-enolase
SEPT6 seq: 280 - 286 no mod	[illegible]	p01062	Septin-6
HSP90B1 seq: 144 - 150 no mod	[illegible]	p17801	Endoplasmin
MYL12B seq: 151 - 160 no mod	[illegible]	p15587	Myosin regulatory light chain 12B
CLIC1 seq: 196 - 204 no mod	[illegible]	p16085	Chloride intracellular channel protein 1
ARHGDIB seq: 51 - 63 no mod	[illegible]	p02599	Rho GDP-dissociation inhibitor 2
MARCKSL1 pS22	[illegible]	p16191	MARCKS-related protein
ARHGDIA seq: 121 - 127 no mod	[illegible]	p17581	Rho GDP-dissociation inhibitor 1
RNH1 seq: 218 - 226 no mod	[illegible]	p18646	Ribonuclease inhibitor
ECM29 seq: 405 - 410 no mod	[illegible]	p11572	Proteasome-associated protein ECM29 homolog
DBI seq: 34 - 53 no mod	[illegible]	p08802	Acyl-CoA-binding protein
PPIA seq: 132 - 144 + Oxidation (M)	[illegible]	p01323	Peptidyl-prolyl cis-trans isomerase A
FASN seq: 1143 - 1151 no mod	[illegible]	p15619	Fatty acid synthase
PPIA seq: 77 - 91 no mod	[illegible]	p05438	Peptidyl-prolyl cis-trans isomerase A
RPS6KL1 seq: 276 - 282 no mod	[illegible]	p14500	Ribosomal protein S6 kinase-like 1
RPN1 seq: 525 - 536 no mod	[illegible]	p20131	Dolichyl-diphosphooligosaccharide--protein glycosyltransferase subunit 1
HSP90B1 seq: 725 - 733 no mod	[illegible]	p05615	Endoplasmin
ANXA5 seq: 90 - 97 no mod	[illegible]	p10310	Annexin A5
ATP5A1 seq: 74 - 83 no mod	[illegible]	p01176	ATP synthase subunit alpha, mitochondrial
TUBB seq: 47 - 58 no mod	[illegible]	p13630	Tubulin beta chain
FLNB seq: 1233 - 1241 no mod	[illegible]	p01556	Filamin-B
NONO seq: 143 - 153 no mod	[illegible]	p16880	Non-POU domain-containing octamer-binding protein
PHPT1 seq: 67 - 78 no mod	[illegible]	p08658	14 kDa phosphohistidine phosphatase
PROSC seq: 267 - 275 no mod	[illegible]	p19970	Proline synthase co-transcribed bacterial homolog protein
COQ6 seq: 65 - 73 no mod	[illegible]	p14024	Ubiquinone biosynthesis monooxygenase COQ6, mitochondrial
BOLA2 seq: 74 - 82 no mod	[illegible]	p02518	BolA-like protein 2
RUVBL1 seq: 23 - 33 no mod	[illegible]	p15738	RuvB-like 1
ALDOA seq: 154 - 173 no mod	[illegible]	p14276	Fructose-bisphosphate aldolase A
ARHGEF28 seq: 509 - 532 + Phospho (ST)	[illegible]	p02813	Rho guanine nucleotide exchange factor 28
RPL8 seq: 164 - 174 no mod	[illegible]	p19153	60S ribosomal protein L8
CEP135 pM295	[illegible]	p17442	Centrosomal protein of 135 kDa
HSPA5 seq: 563 - 573 no mod	[illegible]	p09676	78 kDa glucose-regulated protein
HIST2H2BF seq: 36 - 44 no mod	[illegible]	p17083	Histone H2B type 2-F
KRT8 seq: 214 - 225 no mod	[illegible]	p11983	Keratin, type II cytoskeletal 8
PKM seq: 401 - 422 no mod	[illegible]	p12215	Pyruvate kinase PKM
GNB2L1 seq: 89 - 99 no mod	[illegible]	p10328	Guanine nucleotide-binding protein subunit beta-2-like 1
PHACTR2 seq: 437 - 472 + 4 Phospho (ST); Phospho (Y)	[illegible]	p16899	Phosphatase and actin regulator 2
TUBA1A seq: 65 - 79 no mod	[illegible]	p19246	Tubulin alpha-1A chain
HSPA5 seq: 153 - 163 no mod	[illegible]	p10052	78 kDa glucose-regulated protein
PCBP1 seq: 315 - 325 no mod	[illegible]	p14492	Poly(rC)-binding protein 1
HSPE1 seq: 71 - 80 no mod	[illegible]	p01211	10 kDa heat shock protein, mitochondrial
CDV3 seq: 92 - 99 no mod	[illegible]	p17002	Protein CDV3 homolog
MIF seq: 79 - 87 no mod	[illegible]	p11463	Macrophage migration inhibitory factor
HMGB3 seq: 113 - 125 no mod	[illegible]	p03492	High mobility group protein B3
LDHB seq: 234 - 244 no mod	[illegible]	p09814	L-lactate dehydrogenase B chain
CTSD seq: 349 - 357 no mod	[illegible]	p10717	Cathepsin D
ALDH1B1 seq: 266 - 273 no mod	[illegible]	p00709	Aldehyde dehydrogenase X, mitochondrial
KRT18 seq: 51 - 81 + Phospho (ST)	[illegible]	p03461	Keratin, type I cytoskeletal 18
RPL38 seq: 10 - 16 no mod	[illegible]	p18874	60S ribosomal protein L38
PGK1 seq: 31 - 39 no mod	[illegible]	p09315	Phosphoglycerate kinase 1
P4HB seq: 82 - 97 no mod	[illegible]	p01706	Protein disulfide-isomerase
SF3B2 seq: 269 - 275 no mod	[illegible]	p13864	Splicing factor 3B subunit 2
KRT8 seq: 123 - 130 no mod	[illegible]	p00475	Keratin, type II cytoskeletal 8
HIST1H2AC seq: 101 - 119 no mod	[illegible]	p00718	Histone H2A type 1-C
HIST1H2AD seq: 101 - 119 no mod	[illegible]	p00717	Histone H2A type 1-D
PRDX6 seq: 57 - 63 no mod	[illegible]	p12218	Peroxiredoxin-6
KRT19 seq: 8 - 24 no mod	[illegible]	p07852	Keratin, type I cytoskeletal 19
TUBB4A seq: 283 - 297 no mod	[illegible]	p20128	Tubulin beta-4A chain
SFPQ seq: 272 - 279 no mod	[illegible]	p13750	Splicing factor, proline- and glutamine-rich
RHOC seq: 169 - 176 no mod	[illegible]	p16992	Rho-related GTP-binding protein RhoC
HSPD1 seq: 421 - 429 no mod	[illegible]	p00746	60 kDa heat shock protein, mitochondrial
LASP1 seq: 247 - 261 no mod	[illegible]	p02821	LIM and SH3 domain protein 1
NCL seq: 458 - 467 no mod	[illegible]	p05468	Nucleolin
PKM seq: 93 - 115 no mod	[illegible]	p03008	Pyruvate kinase PKM
MAPRE1 seq: 205 - 214 no mod	[illegible]	p10472	Microtubule-associated protein RP/EB family member 1
PRDX5 seq: 103 - 116 no mod	[illegible]	p02753	Peroxiredoxin-5, mitochondrial
SLC2A13 pS6	[illegible]	p13401	Proton myo-inositol cotransporter
HSP90AB2P seq: 258 - 267 no mod	[illegible]	p05437	Putative heat shock protein HSP 90-beta 2
HNRNPH2 seq: 30 - 35 no mod	[illegible]	p16779	Heterogeneous nuclear ribonucleoprotein H2
RBMX seq: 81 - 93 no mod	[illegible]	p01604	RNA-binding motif protein, X chromosome
NPC2 seq: 26 - 35 no mod	[illegible]	p18930	Epididymal secretory protein E1
HNRNPK seq: 397 - 405 no mod	[illegible]	p18650	Heterogeneous nuclear ribonucleoprotein K
CHRNA7 seq: 436 - 441 no mod	[illegible]	p14071	Neuronal acetylcholine receptor subunit alpha-7
CDKN2D seq: 2 - 7 no mod	[illegible]	p11422	Cyclin-dependent kinase 4 inhibitor D
TKT pM266	[illegible]	p09384	Transketolase
H2AFY seq: 190 - 196 no mod	[illegible]	p05238	Core histone macro-H2A.1
RPL29 seq: 135 - 149 no mod	[illegible]	p19840	60S ribosomal protein L29
TUBA1C seq: 65 - 79 no mod	[illegible]	p19245	Tubulin alpha-1C chain
HSPD1 seq: 397 - 405 no mod	[illegible]	p10829	60 kDa heat shock protein, mitochondrial
KRT19 seq: 398 - 406 no mod	[illegible]	p11942	Keratin, type I cytoskeletal 19
NAV1 seq: 1554 - 1564 no mod	[illegible]	p10391	Neuron navigator 1
ALDOA seq: 15 - 22 no mod	[illegible]	p17409	Fructose-bisphosphate aldolase A
HSPD1 seq: 406 - 418 no mod	[illegible]	p01476	60 kDa heat shock protein, mitochondrial
VBP1 seq: 105 - 114 no mod	[illegible]	p16609	Prefoldin subunit 3
LMNA seq: 12 - 25 no mod	[illegible]	p05790	Prelamin-A/C
POLD1 seq: 226 - 232 no mod	[illegible]	p11411	DNA polymerase delta catalytic subunit
HNRNPM seq: 73 - 83 no mod	[illegible]	p20765	Heterogeneous nuclear ribonucleoprotein M
TUBA3C seq: 353 - 370 no mod	[illegible]	p01470	Tubulin alpha-3C/D chain
GDI2 seq: 211 - 218 no mod	[illegible]	p10293	Rab GDP dissociation inhibitor beta
PABPC1 seq: 313 - 324 no mod	[illegible]	p17783	Polyadenylate-binding protein 1
PABPC1L seq: 96 - 104 no mod	[illegible]	p05620	Polyadenylate-binding protein 1-like
RPL18 seq: 120 - 132 no mod	[illegible]	p13975	60S ribosomal protein L18
CLTC seq: 1227 - 1245 no mod	[illegible]	p12174	Clathrin heavy chain 1
PRDX3 seq: 197 - 207 no mod	[illegible]	p14725	Thioredoxin-dependent peroxide reductase, mitochondrial
PHGDH seq: 385 - 394 no mod	[illegible]	p08871	D-3-phosphoglycerate dehydrogenase
FUBP1 seq: 309 - 321 no mod	[illegible]	p13821	Far upstream element-binding protein 1
EEF1D seq: 25 - 38 no mod	[illegible]	p16370	Elongation factor 1-delta
SNRPD2 seq: 103 - 111 no mod	[illegible]	p16198	Small nuclear ribonucleoprotein Sm D2
SRSF1 seq: 182 - 191 no mod	[illegible]	p05609	Serine/arginine-rich splicing factor 1
TKT seq: 284 - 302 no mod	[illegible]	p14086	Transketolase
HSPA9 seq: 635 - 646 no mod	[illegible]	p08874	Stress-70 protein, mitochondrial
PSMC2 seq: 24 - 34 no mod	[illegible]	p20311	26S protease regulatory subunit 7
EEF1A1P5 seq: 396 - 423 no mod	[illegible]	p05783	Putative elongation factor 1-alpha-like 3
HNRNPA1L2 seq: 147 - 161 no mod	[illegible]	p16143	Heterogeneous nuclear ribonucleoprotein A1-like 2
MTHFD1 seq: 314 - 324 no mod	[illegible]	p02300	C-1-tetrahydrofolate synthase, cytoplasmic
PARK7 seq: 49 - 63 no mod	[illegible]	p18080	Protein deglycase DJ-1
HNRNPM seq: 457 - 471 no mod	[illegible]	p10102	Heterogeneous nuclear ribonucleoprotein M
TGM2 seq: 664 - 674 no mod	[illegible]	p10334	Protein-glutamine gamma-glutamyltransferase 2
TUBB4B seq: 47 - 58 no mod	[illegible]	p13891	Tubulin beta-4B chain
HSPA5 seq: 198 - 214 no mod	[illegible]	p14156	78 kDa glucose-regulated protein
KIF5C seq: 731 - 736 no mod	[illegible]	p14189	Kinesin heavy chain isoform 5C
KRT18 seq: 364 - 369 no mod	[illegible]	p14187	Keratin, type I cytoskeletal 18
CCDC88A seq: 980 - 985 no mod	[illegible]	p11600	Girdin
OTOA seq: 106 - 111 no mod	[illegible]	p11430	Otoancorin
PGK1 seq: 200 - 216 no mod	[illegible]	p20270	Phosphoglycerate kinase 1
PPIA seq: 92 - 118 + Oxidation (M)	[illegible]	p14588	Peptidyl-prolyl cis-trans isomerase A
TKT seq: 303 - 310 no mod	[illegible]	p09947	Transketolase
FUBP1 seq: 163 - 170 no mod	[illegible]	p11445	Far upstream element-binding protein 1
EZR seq: 428 - 435 no mod	[illegible]	p14497	Ezrin
CROCC seq: 1247 - 1254 no mod	[illegible]	p12237	Rootletin
ATP5B seq: 202 - 212 no mod	[illegible]	p14256	ATP synthase subunit beta, mitochondrial
APOA1BP seq: 262 - 269 no mod	[illegible]	p16383	NAD(P)H-hydrate epimerase
PKM seq: 401 - 422 no mod	[illegible]	p12216	Pyruvate kinase PKM
EEF1D seq: 39 - 48 no mod	[illegible]	p08729	Elongation factor 1-delta
KRT7 seq: 123 - 130 no mod	[illegible]	p00468	Keratin, type II cytoskeletal 7
LASP1 seq: 60 - 73 + Gln->pyro-Glu (N-term Q)	[illegible]	p07919	LIM and SH3 domain protein 1
RCN1 seq: 115 - 122 no mod	[illegible]	p00303	Reticulocalbin-1
DSTN seq: 14 - 19 no mod	[illegible]	p14288	Destrin
TPI1 seq: 97 - 106 no mod	[illegible]	p14459	Triosephosphate isomerase
KHSRP seq: 191 - 203 no mod	[illegible]	p03273	Far upstream element-binding protein 2
GLO1 seq: 52 - 60 no mod	[illegible]	p01230	Lactoylglutathione lyase
KRT8 seq: 286 - 295 no mod	[illegible]	p00414	Keratin, type II cytoskeletal 8
KRT79 seq: 341 - 350 no mod	[illegible]	p00413	Keratin, type II cytoskeletal 79
TPI1 seq: 213 - 225 no mod	[illegible]	p03005	Triosephosphate isomerase
PDIA3 seq: 108 - 119 no mod	[illegible]	p18833	Protein disulfide-isomerase A3
CRABP2 seq: 13 - 21 no mod	[illegible]	p06012	Cellular retinoic acid-binding protein 2
PDIA3 seq: 449 - 460 no mod	[illegible]	p16105	Protein disulfide-isomerase A3
HNRNPK seq: 208 - 219 no mod	[illegible]	p14164	Heterogeneous nuclear ribonucleoprotein K
KRT84 seq: 339 - 346 no mod	[illegible]	p19722	Keratin, type II cuticular Hb4
RALY seq: 199 - 207 no mod	[illegible]	p04843	RNA-binding protein Raly
PGK1 pM29	[illegible]	p01692	Phosphoglycerate kinase 1
KRT18 seq: 254 - 261 no mod	[illegible]	p19726	Keratin, type I cytoskeletal 18

LDHB seq: 320 - 329 no mod	2E+08	2E+08	[illegible]	1E+08	p06364	L-lactate dehydrogenase B chain
PLEC seq: 2809 - 2821 no mod	3E+07	2E+07	[illegible]	3E+07	p12200	Plectin
KRT8 pM406	8E+08	8E+08	[illegible]	5E+09	p11926	Keratin, type II cytoskeletal 8
PSMC5 pM329	4E+06	4E+07	[illegible]	25732	p01370	26S protease regulatory subunit 8
TAGLN2 seq: 5 - 12 no mod	3E+07	7E+07	[illegible]	2E+08	p15529	Transgelin-2
HNRNPD seq: 130 - 136 no mod	5E+08	6E+08	[illegible]	9E+07	p12058	Heterogeneous nuclear ribonucleoprotein D0
GNB2 seq: 69 - 78 no mod	5E+07	1E+08	[illegible]	6E+07	p11251	Guanine nucleotide-binding protein G(I)/G(S)/G(T) subunit beta-2
PFN1 seq: 92 - 105 no mod	5E+08	8E+08	[illegible]	9E+08	p03524	Profilin-1
TRIM28 seq: 508 - 524 no mod	38324	2E+07	[illegible]	25732	p01536	Transcription intermediary factor 1-beta
SQSTM1 seq: 421 - 435 no mod	38324	5E+07	[illegible]	3E+07	p08925	Sequestosome-1
P4HB seq: 58 - 65 no mod	4E+08	5E+08	[illegible]	2E+08	p20322	Protein disulfide-isomerase
CALR seq: 279 - 286 no mod	1E+08	2E+08	[illegible]	8E+07	p08534	Calreticulin
SP100 seq: 449 - 473 + Phospho (ST)	7E+07	6E+07	[illegible]	4E+07	p07416	Nuclear autoantigen Sp-100
CLTA seq: 103 - 112 no mod	2E+08	2E+08	[illegible]	1E+07	p10937	Clathrin light chain A
KRT19 seq: 227 - 247 no mod	1E+08	2E+08	[illegible]	1E+08	p15368	Keratin, type I cytoskeletal 19
RBM33 pS765	1E+09	5E+09	[illegible]	5E+09	p01307	RNA-binding protein 33
RPL17 seq: 56 - 61 no mod	1E+07	3E+07	[illegible]	3E+06	p08785	60S ribosomal protein L17
PCMT1 seq: 136 - 144 no mod	1E+08	1E+08	[illegible]	7E+06	p00945	Protein-L-isoaspartate(D-aspartate) O-methyltransferase
HSPD1 seq: 61 - 72 no mod	1E+08	4E+08	[illegible]	1E+08	p01961	60 kDa heat shock protein, mitochondrial
SRSF1 seq: 155 - 164 no mod	2E+08	2E+08	[illegible]	6E+07	p18794	Serine/arginine-rich splicing factor 1
HNRNPD seq: 190 - 197 no mod	3E+06	6E+07	[illegible]	7E+06	p16124	Heterogeneous nuclear ribonucleoprotein D0
PGAM4 seq: 118 - 138 no mod	5E+07	5E+07	[illegible]	6E+07	p03171	Probable phosphoglycerate mutase 4
PDIA6 seq: 246 - 256 no mod	1E+08	9E+07	[illegible]	1E+07	p16149	Protein disulfide-isomerase A6
ULK1 seq: 636 - 648 + Phospho (ST)	6E+07	8E+07	[illegible]	25732	p02311	Serine/threonine-protein kinase ULK1
ARHGEF2 seq: 654 - 660 no mod	2E+08	4E+08	[illegible]	25732	p11320	Rho guanine nucleotide exchange factor 2
APRT seq: 58 - 67 no mod	1E+08	1E+08	[illegible]	4E+07	p14386	Adenine phosphoribosyltransferase
STIP1 seq: 14 - 32 no mod	38324	1E+07	[illegible]	1E+07	p20143	Stress-induced-phosphoprotein 1
CDK14 seq: 22 - 29 + Phospho (ST)	3E+07	3E+07	[illegible]	25732	p02557	Cyclin-dependent kinase 14
IDH2 seq: 341 - 353 no mod	2E+08	3E+08	[illegible]	25732	p02722	Isocitrate dehydrogenase [NADP], mitochondrial
CCT2 seq: 73 - 82 no mod	1E+08	1E+08	[illegible]	7E+07	p09562	T-complex protein 1 subunit beta
ACO2 seq: 371 - 378 no mod	5E+06	3E+07	[illegible]	25732	p17655	Aconitate hydratase, mitochondrial
ANXA4 seq: 260 - 270 no mod	2E+07	4E+07	[illegible]	25732	p15730	Annexin A4
PPIB pM176	2E+07	4E+07	[illegible]	1E+08	p01241	Peptidyl-prolyl cis-trans isomerase B
GDI2 seq: 391 - 402 no mod	5E+07	1E+08	[illegible]	9E+07	p18604	Rab GDP dissociation inhibitor beta
DNAJA1 seq: 207 - 214 no mod	2E+07	2E+08	[illegible]	3E+07	p14049	DnaJ homolog subfamily A member 1
ANXA2P2 seq: 29 - 47 no mod	38324	5E+07	[illegible]	4E+08	p19091	Putative annexin A2-like protein
P4HB seq: 301 - 308 no mod	2E+07	2E+08	[illegible]	3E+07	p14069	Protein disulfide-isomerase
PDIA3 seq: 483 - 496 no mod	5E+08	4E+08	[illegible]	5E+08	p17912	Protein disulfide-isomerase A3
KRT12 seq: 343 - 350 no mod	2E+08	2E+08	[illegible]	1E+08	p05944	Keratin, type I cytoskeletal 12
PPIB seq: 72 - 84 no mod	2E+08	3E+08	[illegible]	3E+08	p01974	Peptidyl-prolyl cis-trans isomerase B
DNAJA4 seq: 208 - 215 no mod	2E+07	2E+08	[illegible]	3E+07	p14179	DnaJ homolog subfamily A member 4
HNRNPA2B1 seq: 47 - 59 no mod	1E+08	2E+08	[illegible]	2E+07	p10577	Heterogeneous nuclear ribonucleoproteins A2/B1
RPSA seq: 64 - 80 no mod	1E+08	3E+08	[illegible]	2E+08	p20417	40S ribosomal protein SA
TMSB4X seq: 21 - 32 no mod	2E+08	1E+08	[illegible]	2E+08	p02880	Thymosin beta-4
GALNT15 seq: 260 - 267 no mod	4E+07	2E+08	[illegible]	25732	p07183	Polypeptide N-acetylgalactosaminyltransferase 15
FABP5 seq: 25 - 33 no mod	38324	9E+06	[illegible]	25732	p17481	Fatty acid-binding protein, epidermal
ERP29 seq: 209 - 223 no mod	6E+07	2E+08	[illegible]	25732	p14078	Endoplasmic reticulum resident protein 29
EEF1A1 pM276	38324	3E+08	[illegible]	3E+08	p01590	Elongation factor 1-alpha 1
KATNAL2 seq: 418 - 425 no mod	2E+08	4E+08	[illegible]	2E+08	p13973	Katanin p60 ATPase-containing subunit A-like 2
NAP1L1 seq: 56 - 72 no mod	38324	3E+06	[illegible]	4E+06	p12086	Nucleosome assembly protein 1-like 1
HSPE1 seq: 9 - 15 no mod	4E+07	2E+08	[illegible]	5E+07	p16597	10 kDa heat shock protein, mitochondrial
NUCKS1 seq: 176 - 184 no mod	9E+07	2E+08	[illegible]	3E+07	p19290	Nuclear ubiquitous casein and cyclin-dependent kinase substrate 1
C19orf68 seq: 535 - 542 no mod	6E+07	1E+08	[illegible]	2E+07	p15761	Uncharacterized protein C19orf68
CCT6A seq: 29 - 35 no mod	6E+07	1E+08	[illegible]	2E+07	p15665	T-complex protein 1 subunit zeta
RBM43 seq: 72 - 78 no mod	6E+07	1E+08	[illegible]	2E+07	p01818	RNA-binding protein 43
PTMS seq: 5 - 15 no mod	3E+08	3E+08	[illegible]	25732	p03374	Parathymosin
MYH9 seq: 1558 - 1566 no mod	3E+07	7E+07	[illegible]	7E+07	p11920	Myosin-9
MYH11 seq: 1565 - 1573 no mod	3E+07	7E+07	[illegible]	7E+07	p11919	Myosin-11
ALDOA seq: 343 - 364 no mod	1E+08	3E+08	[illegible]	1E+08	p00049	Fructose-bisphosphate aldolase A
ELAVL1 seq: 137 - 147 no mod	5E+07	1E+08	[illegible]	4E+07	p01125	ELAV-like protein 1
PURA seq: 145 - 150 no mod	2E+07	37149	[illegible]	25732	p06033	Transcriptional activator protein Pur-alpha
FGD3 seq: 6 - 31 + Phospho (ST)	3E+08	2E+08	[illegible]	1E+07	p15156	FYVE, RhoGEF and PH domain-containing protein 3
FLNB seq: 747 - 754 no mod	7E+07	1E+08	[illegible]	1E+07	p01555	Filamin-B
RPS2 seq: 239 - 246 no mod	8E+07	1E+08	[illegible]	5E+07	p19386	40S ribosomal protein S2
PKM seq: 368 - 376 no mod	6E+08	5E+08	[illegible]	2E+08	p16188	Pyruvate kinase PKM
SORL1 seq: 491 - 497 no mod	2E+08	4E+08	[illegible]	6E+07	p07058	Sortilin-related receptor
FKBP4 seq: 8 - 28 no mod	2E+08	2E+08	[illegible]	2E+08	p19391	Peptidyl-prolyl cis-trans isomerase FKBP4
PSMA5 seq: 54 - 66 no mod	7E+07	9E+07	[illegible]	3E+07	p13581	Proteasome subunit alpha type-5
CALM1 seq: 15 - 31 no mod	5E+08	7E+08	[illegible]	4E+08	p17973	Calmodulin
YWHAZ seq: 92 - 103 no mod	7E+07	2E+08	[illegible]	5E+08	p18769	14-3-3 protein zeta/delta
ANXA5 seq: 30 - 45 no mod	38324	7E+07	[illegible]	4E+07	p15722	Annexin A5
NCL seq: 371 - 377 no mod	2E+08	2E+08	[illegible]	1E+08	p01557	Nucleolin
GDI2 seq: 424 - 436 no mod	2E+07	2E+07	[illegible]	5E+06	p09857	Rab GDP dissociation inhibitor beta
FLNA seq: 1376 - 1381 no mod	4E+07	3E+07	[illegible]	3E+07	p16405	Filamin-A
LRBA seq: 964 - 997 + Phospho (ST)	1E+08	6E+07	[illegible]	5E+07	p18737	Lipopolysaccharide-responsive and beige-like anchor protein
IDH1 seq: 250 - 260 no mod	3E+07	7E+07	[illegible]	25732	p11617	Isocitrate dehydrogenase [NADP] cytoplasmic
KAZN pS387	1E+08	2E+08	[illegible]	4E+08	p10125	Kazrin
TKT seq: 335 - 343 no mod	3E+08	2E+08	[illegible]	3E+07	p14198	Transketolase
RPS20 seq: 9 - 19 no mod	4E+07	1E+08	[illegible]	5E+07	p02302	40S ribosomal protein S20
BANF1 seq: 9 - 32 no mod	1E+07	3E+07	[illegible]	1E+08	p18847	Barrier-to-autointegration factor
HSPD1 seq: 345 - 352 no mod	2E+08	4E+08	[illegible]	6E+07	p01496	60 kDa heat shock protein, mitochondrial
TKT seq: 396 - 401 no mod	7E+07	8E+07	[illegible]	7E+06	p20782	Transketolase
PEBP1 seq: 63 - 76 no mod	3E+08	5E+08	[illegible]	2E+08	p10285	Phosphatidylethanolamine-binding protein 1
ENO1 seq: 270 - 281 no mod	5E+08	9E+08	[illegible]	4E+09	p00299	Alpha-enolase
PPIA pM136	9E+07	8E+07	[illegible]	5E+07	p17713	Peptidyl-prolyl cis-trans isomerase A
LGALS3 seq: 145 - 151 no mod	5E+07	1E+08	[illegible]	5E+07	p14501	Galectin-3
RPS4Y2 seq: 246 - 252 no mod	5E+06	6E+07	[illegible]	3E+07	p10547	40S ribosomal protein S4, Y isoform 2
HSPA5 seq: 139 - 152 no mod	7E+07	2E+08	[illegible]	8E+07	p02877	78 kDa glucose-regulated protein
NUGGC seq: 348 - 355 no mod	2E+08	4E+08	[illegible]	6E+07	p18132	Nuclear GTPase SLIP-GC
GAPDH seq: 235 - 248 no mod	3E+08	7E+08	[illegible]	3E+08	p00977	Glyceraldehyde-3-phosphate dehydrogenase
TUBB seq: 63 - 77 no mod	2E+08	5E+08	[illegible]	4E+08	p20456	Tubulin beta chain
TBC1D8B seq: 358 - 364 no mod	2E+07	1E+08	[illegible]	1E+07	p03330	TBC1 domain family member 8B
CLTA seq: 236 - 242 no mod	2E+07	1E+08	[illegible]	1E+07	p03319	Clathrin light chain A
CNGB1 seq: 1017 - 1023 no mod	2E+07	1E+08	[illegible]	1E+07	p03318	Cyclic nucleotide-gated cation channel beta-1
AHNAK seq: 2326 - 2336 no mod	5E+06	7E+06	[illegible]	2E+06	p14114	Neuroblast differentiation-associated protein AHNAK
TPI1 seq: 187 - 193 no mod	3E+08	4E+08	[illegible]	4E+08	p01410	Triosephosphate isomerase
EEF1A1 seq: 85 - 96 no mod	3E+08	6E+08	[illegible]	2E+08	p00001	Elongation factor 1-alpha 1
HSPD1 seq: 474 - 481 no mod	3E+07	2E+08	[illegible]	2E+07	p13888	60 kDa heat shock protein, mitochondrial
CTSD seq: 404 - 411 no mod	2E+08	5E+08	[illegible]	2E+08	p01494	Cathepsin D
HIST1H2BL pM60 pM63	2E+08	8E+08	[illegible]	9E+08	p20092	Histone H2B type 1-L
HSPA5 seq: 61 - 74 no mod	3E+07	5E+07	[illegible]	1E+07	p13585	78 kDa glucose-regulated protein
HNRNPA2B1 seq: 191 - 200 no mod	2E+08	2E+08	[illegible]	25732	p08731	Heterogeneous nuclear ribonucleoproteins A2/B1
HSPA1A seq: 160 - 171 no mod	6E+07	2E+08	[illegible]	1E+08	p18979	Heat shock 70 kDa protein 1A
FBP2 seq: 245 - 255 no mod	4E+07	1E+08	[illegible]	25732	p00025	Fructose-1,6-bisphosphatase isozyme 2
DPP3 seq: 126 - 141 no mod	1E+08	6E+07	[illegible]	25732	p01373	Dipeptidyl peptidase 3
PLEC seq: 714 - 724 no mod	38324	5E+06	[illegible]	25732	p06113	Plectin
PLEC seq: 1499 - 1508 no mod	1E+07	2E+07	[illegible]	1E+07	p12269	Plectin
ANP32D seq: 100 - 110 no mod	6E+06	8E+06	[illegible]	2E+06	p14119	Acidic leucine-rich nuclear phosphoprotein 32 family member D
ANP32B seq: 100 - 110 no mod	6E+06	8E+06	[illegible]	2E+06	p11522	Acidic leucine-rich nuclear phosphoprotein 32 family member B
FKBP4 seq: 427 - 445 no mod	1E+08	1E+08	[illegible]	25732	p20867	Peptidyl-prolyl cis-trans isomerase FKBP4
TPD52L2 seq: 81 - 90 no mod	38324	1E+07	[illegible]	2E+07	p11733	Tumor protein D54
ACTA1 pM229	38324	1E+08	[illegible]	3E+07	p12110	Actin, alpha skeletal muscle
FLNB seq: 823 - 836 no mod	38324	7E+07	[illegible]	25732	p01236	Filamin-B
TKT seq: 610 - 617 no mod	3E+08	2E+08	[illegible]	1E+06	p18976	Transketolase
BLVRB seq: 146 - 170 no mod	38324	3E+07	[illegible]	3E+08	p00038	Flavin reductase (NADPH)
RPL24 seq: 81 - 93 no mod	2E+07	9E+07	[illegible]	5E+07	p20426	60S ribosomal protein L24
PGD seq: 120 - 136 no mod	5E+07	1E+08	[illegible]	25732	p15882	6-phosphogluconate dehydrogenase, decarboxylating
COPE seq: 24 - 40 no mod	38324	3E+07	[illegible]	25732	p09782	Coatomer subunit epsilon
ARHGAP39 pS406	8E+07	2E+08	[illegible]	25732	p08258	Rho GTPase-activating protein 39
DIABLO seq: 208 - 217 no mod	3E+08	5E+08	[illegible]	8E+07	p12278	Diablo homolog, mitochondrial
ATP5A1 seq: 150 - 161 no mod	3E+08	5E+08	[illegible]	8E+07	p00653	ATP synthase subunit alpha, mitochondrial
CCT7 seq: 441 - 447 no mod	2E+07	6E+07	[illegible]	25732	p20281	T-complex protein 1 subunit eta
LMNA seq: 299 - 311 no mod	6E+06	1E+07	[illegible]	2E+07	p14397	Prelamin-A/C
DDT seq: 88 - 95 no mod	1E+08	7E+07	[illegible]	25732	p17539	D-dopachrome decarboxylase
CA2 seq: 28 - 39 no mod	2E+07	2E+07	[illegible]	25732	p07860	Carbonic anhydrase 2
EEF1G seq: 18 - 30 no mod	1E+07	4E+07	[illegible]	2E+07	p20252	Elongation factor 1-gamma
STMN1 seq: 15 - 27 no mod	2E+07	1E+08	[illegible]	1E+08	p19611	Stathmin
LMNA seq: 320 - 329 no mod	2E+06	5E+06	[illegible]	9E+06	p10909	Prelamin-A/C
PPIA seq: 83 - 91 no mod	5E+08	5E+08	[illegible]	3E+08	p16803	Peptidyl-prolyl cis-trans isomerase A
PKM seq: 33 - 43 no mod	1E+09	2E+09	[illegible]	1E+09	p12081	Pyruvate kinase PKM
RPL23A seq: 116 - 123 no mod	3E+07	6E+07	[illegible]	6E+07	p10309	60S ribosomal protein L23a
KRT86 seq: 280 - 288 no mod	38324	37149	[illegible]	25732	p19727	Keratin, type II cuticular Hb6
P4HB seq: 339 - 345 no mod	4E+07	1E+06	[illegible]	25732	p13620	Protein disulfide-isomerase
ANXA5 seq: 277 - 285 no mod	2E+07	4E+07	[illegible]	3E+07	p06024	Annexin A5
PSMC3 seq: 47 - 53 no mod	3E+07	3E+07	[illegible]	1E+07	p11442	26S protease regulatory subunit 6A
HSPA2 seq: 419 - 426 no mod	3E+07	7E+07	[illegible]	1E+07	p06990	Heat shock-related 70 kDa protein 2
YWHAE seq: 62 - 69 no mod	1E+08	1E+08	[illegible]	1E+08	p14132	14-3-3 protein epsilon
FKBP4 seq: 288 - 294 no mod	4E+07	1E+08	[illegible]	25732	p08845	Peptidyl-prolyl cis-trans isomerase FKBP4
TUBA4A seq: 353 - 370 no mod	5E+08	1E+09	[illegible]	5E+08	p01471	Tubulin alpha-4A chain
HSPE1 seq: 57 - 66 no mod	2E+08	3E+08	[illegible]	7E+07	p16064	10 kDa heat shock protein, mitochondrial
RABGEF1 pS302	6E+08	5E+08	[illegible]	25732	p19896	Rab5 GDP/GTP exchange factor
PSMA6 seq: 96 - 102 no mod	2E+07	3E+07	[illegible]	25732	p00419	Proteasome subunit alpha type-6
PDIA6 seq: 314 - 322 no mod	38324	4E+07	[illegible]	1E+07	p09042	Protein disulfide-isomerase A6
PCLO seq: 3474 - 3482 no mod	3E+07	4E+07	[illegible]	25732	p01708	Protein piccolo
CCT6A seq: 80 - 104 no mod	38324	5E+07	[illegible]	8E+06	p01745	T-complex protein 1 subunit zeta
PCMT1 seq: 125 - 135 no mod	9E+07	9E+07	[illegible]	25732	p13351	Protein-L-isoaspartate(D-aspartate) O-methyltransferase
ARFIP1 seq: 249 - 258 no mod	2E+07	3E+08	[illegible]	2E+08	p02721	Arfaptin-1
RBBP4 seq: 342 - 349 no mod	2E+07	5E+07	[illegible]	25732	p11143	Histone-binding protein RBBP4
AHNAK seq: 5513 - 5524 no mod	38324	4E+07	[illegible]	7E+06	p11065	Neuroblast differentiation-associated protein AHNAK
ICAM5 seq: 65 - 73 no mod	7E+06	4E+07	[illegible]	2E+07	p16033	Intercellular adhesion molecule 5
PSME1 seq: 191 - 198 + Gln->pyro-Glu (N-term Q)	5E+07	5E+07	[illegible]	3E+06	p08129	Proteasome activator complex subunit 1
GRIP1 seq: 428 - 435 + Phospho (ST)	2E+08	2E+08	[illegible]	25732	p04920	Glutamate receptor-interacting protein 1
P4HB seq: 121 - 130 no mod	2E+08	3E+08	[illegible]	9E+07	p17996	Protein disulfide-isomerase
TLN1 seq: 1097 - 1122 no mod	38324	1E+07	[illegible]	25732	p19176	Talin-1
PDIA3 seq: 131 - 140 no mod	1E+07	5E+07	[illegible]	25732	p08858	Protein disulfide-isomerase A3
ALDOA seq: 61 - 69 no mod	2E+08	2E+08	[illegible]	1E+08	p08355	Fructose-bisphosphate aldolase A
RPL11 seq: 39 - 52 no mod	3E+07	1E+08	[illegible]	5E+07	p01238	60S ribosomal protein L11
CALM1 seq: 92 - 107 no mod	5E+08	6E+08	[illegible]	3E+08	p01571	Calmodulin
CSDE1 seq: 428 - 434 no mod	1E+07	2E+07	[illegible]	25732	p16615	Cold shock domain-containing protein E1
CRIP2 pS111	2E+08	3E+08	[illegible]	5E+07	p13399	Cysteine-rich protein 2
LMOD1 seq: 576 - 583 no mod	2E+08	2E+08	[illegible]	4E+07	p18373	Leiomodin-1
RAD23B seq: 205 - 212 no mod	2E+08	2E+08	[illegible]	4E+07	p17259	UV excision repair protein RAD23 homolog B
PRDX2 seq: 17 - 26 no mod	1E+08	2E+08	[illegible]	1E+08	p19410	Peroxiredoxin-2
BLVRB seq: 125 - 134 no mod	9E+08	7E+08	[illegible]	2E+07	p11029	Flavin reductase (NADPH)
ACTN4 seq: 194 - 204 no mod	38324	2E+07	[illegible]	4E+06	p18817	Alpha-actinin-4
PRKCSH seq: 197 - 206 no mod	9E+07	2E+08	[illegible]	7E+07	p10327	Glucosidase 2 subunit beta
ALDH9A1 seq: 31 - 38 no mod	3E+07	6E+07	[illegible]	25732	p20779	4-trimethylaminobutyraldehyde dehydrogenase
DDX17 seq: 469 - 480 no mod	1E+08	2E+08	[illegible]	3E+07	p19978	Probable ATP-dependent RNA helicase DDX17
B2M seq: 69 - 78 no mod	2E+07	37149	[illegible]	4E+07	p01631	Beta-2-microglobulin
VCP pM219	4E+07	6E+07	[illegible]	2E+07	p17352	Transitional endoplasmic reticulum ATPase
EEF1A2 seq: 431 - 439 + Gln->pyro-Glu (N-term Q)	5E+07	2E+08	[illegible]	25732	p07757	Elongation factor 1-alpha 2
HNRNPM seq: 222 - 232 no mod	2E+06	1E+07	[illegible]	25732	p16991	Heterogeneous nuclear ribonucleoprotein M
GLO1 seq: 45 - 51 no mod	2E+08	3E+08	[illegible]	2E+07	p05304	Lactoylglutathione lyase
TPI1 seq: 198 - 212 no mod	38324	3E+07	[illegible]	6E+06	p00609	Triosephosphate isomerase
FASN seq: 42 - 49 no mod	6E+07	2E+08	[illegible]	25732	p20621	Fatty acid synthase
ANXA6 seq: 213 - 220 no mod	4E+06	2E+07	[illegible]	25732	p10433	Annexin A6
PGAM2 seq: 163 - 176 no mod	1E+07	1E+08	[illegible]	2E+08	p20208	Phosphoglycerate mutase 2
KRT19 seq: 343 - 349 no mod	2E+08	1E+08	[illegible]	1E+08	p05945	Keratin, type I cytoskeletal 19
[illegible]T1 OS=Homo sapiens PE=1 SV=2 seq: 788 - 794 no mod	6E+07	9E+07	[illegible]	8E+07	p00901	Scavenger receptor cysteine-rich domain-containing protein SCART1
PITPNC1 seq: 270 - 292 + Phospho (ST)	1E+09	1E+09	[illegible]	25732	p06286	Cytoplasmic phosphatidylinositol transfer protein 1
RAB3IP seq: 288 - 308 + Phospho (ST)	2E+08	3E+08	[illegible]	3E+07	p03438	Rab-3A-interacting protein
RPLP0P6 seq: 17 - 26 no mod	6E+07	1E+08	[illegible]	9E+07	p14142	60S acidic ribosomal protein P0-like
SRSF7 seq: 45 - 65 no mod	2E+07	7E+07	[illegible]	7E+07	p09260	Serine/arginine-rich splicing factor 7
G6PD seq: 394 - 403 no mod	2E+08	2E+08	[illegible]	3E+07	p00938	Glucose-6-phosphate 1-dehydrogenase
TCEA1 seq: 124 - 132 no mod	2E+07	3E+07	[illegible]	25732	p18136	Transcription elongation factor A protein 1
TKT seq: 544 - 550 no mod	1E+08	2E+08	[illegible]	5E+07	p11583	Transketolase
TPD52L1 pS174	2E+08	4E+08	[illegible]	25732	p01477	Tumor protein D53
GAPDH seq: 220 - 227 no mod	3E+09	2E+09	[illegible]	2E+09	p01361	Glyceraldehyde-3-phosphate dehydrogenase
[illegible]: 3008 - 3031 + Gln->pyro-Glu (N-term Q); Phospho (ST)	1E+08	1E+08	[illegible]	25732	p08817	Golgin subfamily B member 1
TBC1D1 seq: 257 - 289 + Phospho (ST)	9E+06	2E+07	[illegible]	25732	p03900	TBC1 domain family member 1
MCM6 seq: 191 - 197 no mod	38324	5E+06	[illegible]	2E+07	p16607	DNA replication licensing factor MCM6
YWHAQ seq: 28 - 41 no mod	6E+07	6E+07	[illegible]	2E+07	p19170	14-3-3 protein theta
CLIC1 seq: 96 - 113 no mod	1E+07	8E+07	[illegible]	2E+08	p12306	Chloride intracellular channel protein 1
LDHA seq: 233 - 243 + Gln->pyro-Glu (N-term Q)	1E+07	2E+07	[illegible]	2E+07	p07687	L-lactate dehydrogenase A chain
PSAP seq: 233 - 242 no mod	1E+07	3E+07	[illegible]	25732	p11719	Prosaposin
AK2 seq: 86 - 93 no mod	1E+07	37149	[illegible]	3E+07	p09467	Adenylate kinase 2, mitochondrial
CCT8 seq: 441 - 450 no mod	4E+07	6E+07	[illegible]	2E+07	p16874	T-complex protein 1 subunit theta
SRRT seq: 860 - 865 no mod	3E+06	5E+07	[illegible]	1E+07	p20414	Serrate RNA effector molecule homolog
DNAJA1 seq: 326 - 344 no mod	2E+06	1E+06	[illegible]	25732	p01084	DnaJ homolog subfamily A member 1
PLEKHA5 pS933	9E+07	1E+08	[illegible]	25732	p15682	Pleckstrin homology domain-containing family A member 5
FKBP4 seq: 75 - 83 no mod	2E+07	5E+07	[illegible]	25732	p18689	Peptidyl-prolyl cis-trans isomerase FKBP4
ILF3 seq: 312 - 323 no mod	5E+07	9E+07	[illegible]	5E+06	p17882	Interleukin enhancer-binding factor 3
PDIA4 seq: 120 - 131 no mod	2E+08	3E+08	[illegible]	4E+07	p14446	Protein disulfide-isomerase A4
HNRNPUL2 seq: 213 - 218 no mod	3E+07	4E+07	[illegible]	1E+07	p19080	Heterogeneous nuclear ribonucleoprotein U-like protein 2
KRT7 seq: 137 - 149 no mod	2E+06	3E+07	[illegible]	2E+08	p11135	Keratin, type II cytoskeletal 7
RPLP1 seq: 34 - 49 no mod	8E+06	2E+08	[illegible]	2E+08	p21134	60S acidic ribosomal protein P1
PLEKHA5 pS382	3E+08	4E+08	[illegible]	25732	p13542	Pleckstrin homology domain-containing family A member 5
SRSF2 seq: 40 - 47 no mod	2E+08	3E+08	[illegible]	1E+08	p01506	Serine/arginine-rich splicing factor 2
TCOF1 pS233	8E+06	4E+06	[illegible]	3E+07	p19465	Treacle protein
DDX39A seq: 209 - 215 no mod	5E+07	3E+07	[illegible]	25732	p18094	ATP-dependent RNA helicase DDX39A

UBA6 pS36	1E+08 3E+08 8E+08 6E+08 6E+08 1E+09 5E+08 7E+08 4E+08 4E+08 1E+09 4E+08 6E+08 4E+08 4E+08 3E+08 2E+08 7E+08 5E+08 3E+08 3E+08 2E+08 4E+08 3E+08 [illegible]	p0a426	Ubiquitin-like modifier-activating enzyme 6
HNRNPA1L2 seq: 123 - 130 no mod	3E+08 2E+08 3E+08 6E+07 2E+08 6E+08 1E+08 2E+08 1E+08 1E+08 2E+09 2E+08 5E+08 1E+08 1E+08 3E+08 2E+08 2E+08 2E+08 8E+07 2E+08 1E+08 2E+08 1E+08 [illegible]	[illegible]	Heterogeneous nuclear ribonucleoprotein A1-like 2
DPYSL2 pT509	1E+08 2E+08 3E+08 3E+07 1E+08 5E+08 1E+08 3E+08 4E+07 8E+07 1E+09 2E+08 3E+08 5E+07 9E+07 3E+08 4E+07 1E+08 1E+08 4E+07 1E+08 1E+08 9E+07 9E+07 [illegible]	[illegible]	Dihydropyrimidinase-related protein 2
FAM21A pS909	9E+07 8E+07 1E+08 8E+07 8E+07 2E+08 2E+08 8E+07 6E+07 3E+07 3E+08 2E+08 2E+08 6E+07 3E+07 5E+07 8E+07 1E+08 6E+07 4E+07 1E+08 4E+07 8E+07 2E+07 [illegible]	[illegible]	WASH complex subunit FAM21A
WDR73 seq: 200 - 206 no mod	2E+08 2E+08 3E+08 5E+07 1E+08 45779 6E+07 3E+08 7E+07 2E+07 2E+09 8E+07 3E+08 7E+07 2E+07 1E+08 1E+08 7E+07 1E+08 1E+07 1E+08 7E+07 9E+07 9E+07 [illegible]	[illegible]	WD repeat-containing protein 73
LDHA seq: 233 - 243 no mod	7E+07 1E+08 1E+08 1E+07 5E+07 1E+08 5E+07 5E+07 2E+07 2E+07 7E+08 3E+07 1E+08 3E+07 2E+07 5E+07 4E+07 34984 7E+07 4E+06 1E+07 2E+07 2E+07 2E+07 [illegible]	[illegible]	L-lactate dehydrogenase A chain
UQCRC1 seq: 214 - 225 no mod	38324 5E+07 1E+08 2E+07 8E+07 1E+08 1E+07 7E+07 4E+07 3E+07 6E+08 2E+07 2E+08 3E+07 2E+07 40867 1E+07 1E+07 9E+07 1E+07 39759 2E+07 3E+07 3E+07 [illegible]	[illegible]	Cytochrome b-c1 complex subunit 1, mitochondrial
U2AF2 seq: 204 - 225 no mod	38324 4E+07 2E+07 3E+06 4E+06 1E+08 2E+07 8E+06 1E+07 4E+06 4E+08 4E+07 1E+07 1E+07 18798 5E+07 33072 34984 1E+07 28832 1E+07 50401 2E+06 19024 [illegible]	[illegible]	Splicing factor U2AF 65 kDa subunit
PSMC4 seq: 230 - 238 no mod	2E+07 4E+07 5E+07 6E+06 2E+07 45779 25552 4E+07 9E+06 4E+06 56234 8E+06 5E+07 9E+06 18798 40867 33072 34984 3E+07 28832 39759 9E+06 1E+07 19024 [illegible]	[illegible]	26S protease regulatory subunit 6B
CCT4 seq: 194 - 206 no mod	4E+07 8E+07 8E+07 13654 4E+07 2E+08 1E+07 6E+07 2E+07 26736 8E+08 2E+07 9E+07 40879 5E+06 4E+07 6E+06 8E+06 5E+07 28832 3E+07 7E+06 1E+07 2E+07 [illegible]	[illegible]	T-complex protein 1 subunit delta
ETFA seq: 322 - 331 no mod	5E+07 1E+08 1E+08 2E+07 5E+07 2E+08 4E+07 7E+07 3E+07 2E+07 1E+09 4E+07 2E+08 2E+07 18798 1E+08 3E+07 4E+07 8E+07 7E+06 5E+07 2E+07 4E+07 3E+07 [illegible]	[illegible]	Electron transfer flavoprotein subunit alpha, mitochondrial
KRT4 pM440	4E+07 5E+07 1E+08 2E+07 4E+07 6E+07 1E+07 2E+08 1E+07 3E+07 3E+08 3E+07 1E+08 40879 8E+06 3E+07 3E+07 3E+07 1E+08 2E+06 39759 3E+07 4E+07 4E+07 [illegible]	[illegible]	Keratin, type II cytoskeletal 4
COBL seq: 233 - 245 + Phospho (ST)	3E+07 5E+07 1E+08 2E+08 3E+08 3E+08 8E+07 6E+07 2E+08 5E+08 9E+07 6E+07 9E+07 4E+07 4E+08 1E+08 6E+07 1E+08 2E+08 3E+08 1E+08 1E+08 1E+08 7E+07 [illegible]	[illegible]	Protein cordon-bleu
CCT6A seq: 118 - 127 no mod	8E+07 8E+07 2E+08 3E+07 5E+07 1E+08 1E+07 1E+08 4E+07 3E+07 8E+08 3E+07 2E+08 3E+07 4E+07 9E+07 4E+07 4E+07 1E+08 7E+06 6E+07 2E+07 5E+07 3E+07 [illegible]	[illegible]	T-complex protein 1 subunit zeta
BLM seq: 410 - 418 no mod	8E+07 8E+07 2E+08 3E+07 5E+07 1E+08 1E+07 1E+08 4E+07 3E+07 8E+08 3E+07 2E+08 3E+07 4E+07 9E+07 4E+07 4E+07 1E+08 7E+06 6E+07 2E+07 5E+07 3E+07 [illegible]	[illegible]	Bloom syndrome protein
ENO1 seq: 163 - 179 no mod	38324 7E+06 6E+07 1E+06 4E+07 6E+06 1E+07 1E+07 1E+07 2E+06 56234 1E+07 4E+07 5E+06 4E+06 3E+06 1E+06 5E+06 2E+07 1E+06 3E+06 4E+06 5E+06 5E+06 [illegible]	[illegible]	Alpha-enolase
PHGDH seq: 237 - 247 no mod	6E+07 1E+08 2E+08 3E+07 1E+08 3E+08 4E+07 1E+08 5E+07 5E+07 1E+09 3E+07 2E+08 6E+07 3E+07 1E+08 3E+07 6E+07 2E+08 1E+07 4E+07 3E+07 5E+07 5E+07 [illegible]	[illegible]	D-3-phosphoglycerate dehydrogenase
FLNB seq: 496 - 502 no mod	7E+07 9E+07 2E+08 3E+07 7E+07 45779 3E+07 1E+08 4E+07 4E+07 56234 4E+07 2E+08 4E+07 18798 40867 4E+07 5E+07 1E+08 28832 4E+07 4E+07 5E+07 4E+07 [illegible]	[illegible]	Filamin-B
[illegible]	[illegible]	[illegible]	[illegible]

Peptide	Intensity values	Accession	Protein
PRKCSH seq: 159 - 166 no mod	[illegible]	p11604	Glucosidase 2 subunit beta
TPI1 seq: 71 - 90 no mod	[illegible]	p01036	Triosephosphate isomerase
HSP90B1 seq: 53 - 61 no mod	[illegible]	p15755	Endoplasmin
UBE2N seq: 86 - 92 no mod	[illegible]	p14456	Ubiquitin-conjugating enzyme E2 N
LRRC16A seq: 11 - 17 no mod	[illegible]	p17472	Leucine-rich repeat-containing protein 16A
TTC26 seq: 30 - 36 no mod	[illegible]	p11995	Intraflagellar transport protein 56
EPB41L4B seq: 564 - 570 no mod	[illegible]	p11964	Band 4.1-like protein 4B
TBCA seq: 71 - 80 no mod	[illegible]	p12011	Tubulin-specific chaperone A
HYOU1 seq: 439 - 451 no mod	[illegible]	p18937	Hypoxia up-regulated protein 1
ACTN4 seq: 883 - 899 no mod	[illegible]	p10247	Alpha-actinin-4
ANKRD26 seq: 925 - 931 no mod	[illegible]	p11974	Ankyrin repeat domain-containing protein 26
CEP83 seq: 313 - 319 no mod	[illegible]	p11972	Centrosomal protein of 83 kDa
HNRNPK seq: 306 - 316 no mod	[illegible]	p09425	Heterogeneous nuclear ribonucleoprotein K
LCP1 seq: 69 - 76 no mod	[illegible]	p13737	Plastin-2
TAPT1 seq: 552 - 558 no mod	[illegible]	p18586	Transmembrane anterior posterior transformation protein 1 homolog
GANAB seq: 158 - 164 no mod	[illegible]	p12071	Neutral alpha-glucosidase AB
MDH2 seq: 192 - 203 no mod	[illegible]	p01042	Malate dehydrogenase, mitochondrial
KRT18 seq: 371 - 381 no mod	[illegible]	p01311	Keratin, type I cytoskeletal 18
VIM seq: 160 - 168 no mod	[illegible]	p07747	Vimentin
RPL4 seq: 166 - 172 no mod	[illegible]	p17902	60S ribosomal protein L4
VCP seq: 218 - 225 no mod	[illegible]	p17353	Transitional endoplasmic reticulum ATPase
GRB2 seq: 208 - 215 no mod	[illegible]	p08905	Growth factor receptor-bound protein 2
CLTC seq: 1123 - 1130 no mod	[illegible]	p17959	Clathrin heavy chain 1
FLNB seq: 1320 - 1330 no mod	[illegible]	p01729	Filamin-B
RBM39 seq: 168 - 178 no mod	[illegible]	p18631	RNA-binding protein 39
ETFA seq: 86 - 101 no mod	[illegible]	p15706	Electron transfer flavoprotein subunit alpha, mitochondrial
AHNAK seq: 1141 - 1152 no mod	[illegible]	p16165	Neuroblast differentiation-associated protein AHNAK
NCL seq: 600 - 610 no mod	[illegible]	p13527	Nucleolin
CMBL seq: 16 - 23 no mod	[illegible]	p11914	Carboxymethylenebutenolidase homolog
TKT seq: 439 - 456 no mod	[illegible]	p03296	Transketolase
KRT7 seq: 254 - 265 no mod	[illegible]	p05298	Keratin, type II cytoskeletal 7
KRT19 pM252	[illegible]	p13994	Keratin, type I cytoskeletal 19
PSMD7 seq: 181 - 190 no mod	[illegible]	p18147	26S proteasome non-ATPase regulatory subunit 7
GIT1 seq: 531 - 555 + Phospho (ST)	[illegible]	p10964	ARF GTPase-activating protein GIT1
CAMKK2 pS100	[illegible]	p12918	Calcium/calmodulin-dependent protein kinase kinase 2
GLUD1 seq: 528 - 535 no mod	[illegible]	p00202	Glutamate dehydrogenase 1, mitochondrial
PDIA3 seq: 290 - 296 no mod	[illegible]	p16634	Protein disulfide-isomerase A3
CNDP2 seq: 150 - 159 no mod	[illegible]	p02805	Cytosolic non-specific dipeptidase
HSP90AA1 seq: 465 - 478 no mod	[illegible]	p00005	Heat shock protein HSP 90-alpha
RPS8 seq: 194 - 200 no mod	[illegible]	p17508	40S ribosomal protein S8
RUVBL1 seq: 47 - 57 no mod	[illegible]	p18000	RuvB-like 1
SRRM1 seq: 411 - 422 + Phospho (ST)	[illegible]	p02229	Serine/arginine repetitive matrix protein 1
HSPA2 seq: 352 - 360 no mod	[illegible]	p11319	Heat shock-related 70 kDa protein 2
PKM seq: 189 - 207 no mod	[illegible]	p16345	Pyruvate kinase PKM
PDS5B seq: 1403 - 1438 + Phospho (ST)	[illegible]	p17345	Sister chromatid cohesion protein PDS5 homolog B
RPS3A seq: 188 - 195 no mod	[illegible]	p11569	40S ribosomal protein S3a
MTMR3 seq: 613 - 630 + Phospho (ST)	[illegible]	p03174	Myotubularin-related protein 3
SARNP seq: 157 - 166 no mod	[illegible]	p16750	SAP domain-containing ribonucleoprotein
TMF1 seq: 935 - 978 + Phospho (ST)	[illegible]	p03703	TATA element modulatory factor
P4HB seq: 402 - 409 no mod	[illegible]	p08404	Protein disulfide-isomerase
PDIA6 seq: 61 - 66 no mod	[illegible]	p10524	Protein disulfide-isomerase A6
CLTC seq: 1011 - 1022 no mod	[illegible]	p13547	Clathrin heavy chain 1
STT3B pS18	[illegible]	p03848	Dolichyl-diphosphooligosaccharide--protein glycosyltransferase subunit STT3B
ANXA2P2 seq: 296 - 302 no mod	[illegible]	p05951	Putative annexin A2-like protein
SRSF2 seq: 18 - 24 no mod	[illegible]	p01669	Serine/arginine-rich splicing factor 2
PDIA4 seq: 132 - 141 no mod	[illegible]	p16804	Protein disulfide-isomerase A4
DLD seq: 289 - 300 no mod	[illegible]	p14389	Dihydrolipoyl dehydrogenase, mitochondrial
RPL10 seq: 190 - 198 no mod	[illegible]	p11570	60S ribosomal protein L10
HNRNPA2B1 pS29	[illegible]	p11885	Heterogeneous nuclear ribonucleoproteins A2/B1
ALDOA seq: 61 - 69 + Gln->pyro-Glu (N-term Q)	[illegible]	p08354	Fructose-bisphosphate aldolase A
ANXA2 seq: 234 - 245 no mod	[illegible]	p03128	Annexin A2
TUBB4B seq: 123 - 154 + Oxidation (M)	[illegible]	p17985	Tubulin beta-4B chain
TUBB8 seq: 123 - 154 + Oxidation (M)	[illegible]	p17983	Tubulin beta-8 chain
RPL22 seq: 70 - 80 no mod	[illegible]	p13573	60S ribosomal protein L22
PSMA6 seq: 31 - 43 no mod	[illegible]	p20451	Proteasome subunit alpha type-6
MESDC2 seq: 96 - 106 no mod	[illegible]	p14401	LDLR chaperone MESD
CCT2 seq: 205 - 223 no mod	[illegible]	p11769	T-complex protein 1 subunit beta
NANS seq: 321 - 334 no mod	[illegible]	p14920	Sialic acid synthase
LRRFIP1 seq: 720 - 749 + Phospho (ST)	[illegible]	p06057	Leucine-rich repeat flightless-interacting protein 1
FSCN1 seq: 202 - 217 no mod	[illegible]	p10449	Fascin
TNS1 seq: 1046 - 1062 + Phospho (ST)	[illegible]	p05861	Tensin-1
RPS3 seq: 19 - 27 no mod	[illegible]	p20821	40S ribosomal protein S3
SCEL pS68	[illegible]	p14706	Sciellin
FARP1 pS403	[illegible]	p05312	FERM, RhoGEF and pleckstrin domain-containing protein 1
PPIA seq: 77 - 91 no mod	[illegible]	p05439	Peptidyl-prolyl cis-trans isomerase A
PABPN1 pS150	[illegible]	p08241	Polyadenylate-binding protein 2
CDKL5 pS641	[illegible]	p20031	Cyclin-dependent kinase-like 5
ZC3H13 seq: 237 - 249 + Phospho (ST)	[illegible]	p02194	Zinc finger CCCH domain-containing protein 13
ATP2A3 seq: 820 - 825 + Phospho (ST)	[illegible]	p11082	Sarcoplasmic/endoplasmic reticulum calcium ATPase 3
RBM15B pS609	[illegible]	p02050	Putative RNA-binding protein 15B
EEF2 seq: 573 - 580 no mod	[illegible]	p06092	Elongation factor 2
EEF1A1P5 pM404 pM410	[illegible]	p05782	Putative elongation factor 1-alpha-like 3
PRRC2B pS480	[illegible]	p08011	Protein PRRC2B
GAPDH pM130 pM133	[illegible]	p01380	Glyceraldehyde-3-phosphate dehydrogenase
AHNAK seq: 1083 - 1091 no mod	[illegible]	p15557	Neuroblast differentiation-associated protein AHNAK
PPP1R13L pS526 pM527	[illegible]	p07333	RelA-associated inhibitor
+ Gln->pyro-Glu (N-term Q); Oxidation (M); Phospho (ST)	[illegible]	p08046	AN1-type zinc finger protein 5
WAC pS452	[illegible]	p03109	WW domain-containing adapter protein with coiled-coil
C14orf166 seq: 177 - 185 no mod	[illegible]	p17723	UPF0568 protein C14orf166
FLNB seq: 2017 - 2028 no mod	[illegible]	p02869	Filamin-B
KHDRBS1 seq: 37 - 43 + Gln->pyro-Glu (N-term Q)	[illegible]	p08115	KH domain-containing, RNA-binding, signal transduction-associated protein 1
CAMSAP3 seq: 681 - 701 + Phospho (ST)	[illegible]	p19168	Calmodulin-regulated spectrin-associated protein 3
TUBB seq: 155 - 162 no mod	[illegible]	p13774	Tubulin beta chain
SKP2 pS64	[illegible]	p02186	S-phase kinase-associated protein 2
CTAGE5 seq: 611 - 632 + Phospho (ST)	[illegible]	p05875	cTAGE family member 5
TRA2B seq: 191 - 197 no mod	[illegible]	p01689	Transformer-2 protein homolog beta
COMT seq: 152 - 159 no mod	[illegible]	p09835	Catechol O-methyltransferase
GIGYF2 seq: 1016 - 1028 + Gln->pyro-Glu (N-term Q)	[illegible]	p08028	PERQ amino acid-rich with GYF domain-containing protein 2
XRCC5 seq: 535 - 543 no mod	[illegible]	p02610	X-ray repair cross-complementing protein 5
QPRT seq: 150 - 161 no mod	[illegible]	p00362	Nicotinate-nucleotide pyrophosphorylase [carboxylating]
HNRNPC pM74	[illegible]	p10077	Heterogeneous nuclear ribonucleoproteins C1/C2
LSM7 seq: 10 - 17 no mod	[illegible]	p17158	U6 snRNA-associated Sm-like protein LSm7
CSRP1 seq: 179 - 193 no mod	[illegible]	p16130	Cysteine and glycine-rich protein 1
STRAP seq: 45 - 59 + Gln->pyro-Glu (N-term Q)	[illegible]	p08655	Serine-threonine kinase receptor-associated protein
PSAP seq: 430 - 438 no mod	[illegible]	p08740	Prosaposin
SRRM1 pS636 pS638	[illegible]	p06441	Serine/arginine repetitive matrix protein 1
LASP1 seq: 112 - 121 no mod	[illegible]	p12479	LIM and SH3 domain protein 1
EEF2 seq: 2 - 10 no mod	[illegible]	p01081	Elongation factor 2
STIP1 seq: 145 - 153 no mod	[illegible]	p02597	Stress-induced-phosphoprotein 1
HSPA1B seq: 237 - 246 no mod	[illegible]	p10387	Heat shock 70 kDa protein 1B
HNRNPK seq: 279 - 286 no mod	[illegible]	p18065	Heterogeneous nuclear ribonucleoprotein K
CTR9 pS970	[illegible]	p16177	RNA polymerase-associated protein CTR9 homolog
FTSJ3 pS584	[illegible]	p02908	pre-rRNA processing protein FTSJ3
TAF15 seq: 349 - 357 no mod	[illegible]	p21128	TATA-binding protein-associated factor 2N
MDH2 seq: 230 - 239 no mod	[illegible]	p13834	Malate dehydrogenase, mitochondrial
VIM seq: 29 - 36 no mod	[illegible]	p03108	Vimentin
HNRNPA2B1 seq: 130 - 137 no mod	[illegible]	p18051	Heterogeneous nuclear ribonucleoproteins A2/B1
SLAIN2 seq: 430 - 446 + Phospho (ST)	[illegible]	p03738	SLAIN motif-containing protein 2
PNPLA2 seq: 401 - 416 + Phospho (ST)	[illegible]	p06450	Patatin-like phospholipase domain-containing protein 2
TRAF4 pS426	[illegible]	p15249	TNF receptor-associated factor 4
TCP1 seq: 434 - 443 no mod	[illegible]	p17283	T-complex protein 1 subunit alpha
SHANK2 seq: 668 - 708 + Phospho (ST)	[illegible]	p03750	SH3 and multiple ankyrin repeat domains protein 2
CFL1 seq: 54 - 73 no mod	[illegible]	p17609	Cofilin-1
SRRM2 seq: 771 - 776 + Phospho (ST)	[illegible]	p04100	Serine/arginine repetitive matrix protein 2
U2AF2 seq: 277 - 286 no mod	[illegible]	p17450	Splicing factor U2AF 65 kDa subunit
KRT18 seq: 51 - 90 + Phospho (ST)	[illegible]	p03455	Keratin, type I cytoskeletal 18
PPIG seq: 372 - 379 + Phospho (ST)	[illegible]	p09916	Peptidyl-prolyl cis-trans isomerase G
HNRNPA3 seq: 168 - 182 no mod	[illegible]	p16142	Heterogeneous nuclear ribonucleoprotein A3
PPP2R1A seq: 261 - 272 no mod	[illegible]	p00211	Serine/threonine-protein phosphatase 2A 65 kDa regulatory subunit A alpha isoform
EFCAB12 seq: 168 - 173 + Phospho (ST)	[illegible]	p10688	EF-hand calcium-binding domain-containing protein 12
CTTN seq: 111 - 119 no mod	[illegible]	p14868	Src substrate cortactin
SIK3 pS920	[illegible]	p14634	Serine/threonine-protein kinase SIK3
TPD52L1 seq: 134 - 142 + Phospho (ST)	[illegible]	p14641	Tumor protein D53
RPL6 seq: 88 - 100 no mod	[illegible]	p01270	60S ribosomal protein L6
G3BP1 seq: 124 - 132 no mod	[illegible]	p16361	Ras GTPase-activating protein-binding protein 1
ALDH8A1 seq: 250 - 257 no mod	[illegible]	p10762	Aldehyde dehydrogenase family 8 member A1
ATP5A1 seq: 134 - 149 no mod	[illegible]	p02826	ATP synthase subunit alpha, mitochondrial
FKBP2 seq: 104 - 115 no mod	[illegible]	p10402	Peptidyl-prolyl cis-trans isomerase FKBP2
SNRPA1 seq: 144 - 149 no mod	[illegible]	p01262	U2 small nuclear ribonucleoprotein A'
SEPT2 seq: 4 - 29 + Phospho (Y)	[illegible]	p08034	Septin-2
ERP29 seq: 37 - 48 no mod	[illegible]	p16295	Endoplasmic reticulum resident protein 29
LRRTM4 seq: 193 - 198 + Phospho (ST)	[illegible]	p10866	Leucine-rich repeat transmembrane neuronal protein 4
CNDP2 seq: 403 - 413 no mod	[illegible]	p01970	Cytosolic non-specific dipeptidase
ASH2L pS623	[illegible]	p04472	Set1/Ash2 histone methyltransferase complex subunit ASH2
HDLBP seq: 551 - 557 no mod	[illegible]	p06105	Vigilin
PSMC4 seq: 274 - 283 no mod	[illegible]	p07453	26S protease regulatory subunit 6B
HOOK3 seq: 707 - 718 + Phospho (ST)	[illegible]	p03153	Protein Hook homolog 3
ENAH pM503 pS508	[illegible]	p02448	Protein enabled homolog
SLC9A3R1 seq: 211 - 220 + Gln->pyro-Glu (N-term Q)	[illegible]	p08568	Na(+)/H(+) exchange regulatory cofactor NHE-RF1
FLNB seq: 1268 - 1299 no mod	[illegible]	p20532	Filamin-B
TUFM seq: 136 - 162 no mod	[illegible]	p09372	Elongation factor Tu, mitochondrial
PSMC2 seq: 47 - 57 + Gln->pyro-Glu (N-term Q)	[illegible]	p07744	26S protease regulatory subunit 7
TCEB2 seq: 20 - 29 no mod	[illegible]	p17108	Transcription elongation factor B polypeptide 2
HSPA1L seq: 113 - 126 no mod	[illegible]	p20730	Heat shock 70 kDa protein 1-like
TPM4 seq: 154 - 161 no mod	[illegible]	p14774	Tropomyosin alpha-4 chain
SRSF3 seq: 44 - 57 no mod	[illegible]	p09261	Serine/arginine-rich splicing factor 3
GDI2 seq: 157 - 164 no mod	[illegible]	p02871	Rab GDP dissociation inhibitor beta
ALDH7A1 seq: 529 - 537 no mod	[illegible]	p18561	Alpha-aminoadipic semialdehyde dehydrogenase
PDIA4 seq: 273 - 288 no mod	[illegible]	p00365	Protein disulfide-isomerase A4
PDLIM5 seq: 305 - 324 + 2 Phospho (ST)	[illegible]	p13423	PDZ and LIM domain protein 5
HOOK3 seq: 707 - 718 + Phospho (Y)	[illegible]	p03151	Protein Hook homolog 3
FASN seq: 275 - 298 no mod	[illegible]	p04921	Fatty acid synthase
TUBB seq: 310 - 318 no mod	[illegible]	p00228	Tubulin beta chain
LDB1 pS302	[illegible]	p12860	LIM domain-binding protein 1
DDX3Y seq: 593 - 600 no mod	[illegible]	p16470	ATP-dependent RNA helicase DDX3Y
MYL6B seq: 84 - 94 no mod	[illegible]	p13967	Myosin light chain 6B
EPPK1 seq: 2421 - 2432 no mod	[illegible]	p15310	Epiplakin
TFAP2B pS223	[illegible]	p10756	Transcription factor AP-2-beta
LMNB2 seq: 117 - 123 no mod	[illegible]	p10992	Lamin-B2
LMNB1 seq: 103 - 109 no mod	[illegible]	p10991	Lamin-B1
RBMX seq: 23 - 30 no mod	[illegible]	p20289	RNA-binding motif protein, X chromosome
GSE1 seq: 849 - 863 no mod	[illegible]	p00099	Genetic suppressor element 1
HSP90B1 seq: 416 - 428 no mod	[illegible]	p01547	Endoplasmin
VCP seq: 466 - 487 no mod	[illegible]	p17028	Transitional endoplasmic reticulum ATPase
EDC4 seq: 696 - 726 + Phospho (ST)	[illegible]	p15508	Enhancer of mRNA-decapping protein 4
GLOD4 seq: 156 - 165 no mod	[illegible]	p00711	Glyoxalase domain-containing protein 4
ZMYND8 seq: 481 - 505 + Phospho (ST)	[illegible]	p02807	Protein kinase C-binding protein 1
THOP1 seq: 311 - 317 no mod	[illegible]	p19239	Thimet oligopeptidase
MDH2 seq: 216 - 229 no mod	[illegible]	p01690	Malate dehydrogenase, mitochondrial
ENO1 seq: 163 - 179 + Oxidation (M)	[illegible]	p12223	Alpha-enolase
RALGAPA1 seq: 752 - 765 + Phospho (ST)	[illegible]	p08420	Ral GTPase-activating protein subunit alpha-1
ATP5B seq: 311 - 324 no mod	[illegible]	p16415	ATP synthase subunit beta, mitochondrial
SUCLG2 seq: 339 - 347 no mod	[illegible]	p17921	Succinyl-CoA ligase [GDP-forming] subunit beta, mitochondrial
LUC7L3 pS431	[illegible]	p17177	Luc7-like protein 3
HOOK3 seq: 707 - 718 + Phospho (ST)	[illegible]	p03152	Protein Hook homolog 3
RPS13 seq: 10 - 19 no mod	[illegible]	p15639	40S ribosomal protein S13
CCAR2 seq: 888 - 893 no mod	[illegible]	p11311	Cell cycle and apoptosis regulator protein 2
CCDC116 seq: 200 - 205 no mod	[illegible]	p11308	Coiled-coil domain-containing protein 116
CNDP2 seq: 54 - 66 no mod	[illegible]	p08382	Cytosolic non-specific dipeptidase
NCOR1 seq: 153 - 175 + Phospho (ST)	[illegible]	p14849	Nuclear receptor corepressor 1
TUBB seq: 63 - 77 no mod	[illegible]	p20455	Tubulin beta chain
COX5A seq: 99 - 107 no mod	[illegible]	p11207	Cytochrome c oxidase subunit 5A, mitochondrial
SRPR seq: 402 - 407 + Oxidation (M)	[illegible]	p06472	Signal recognition particle receptor subunit alpha
ARSK seq: 170 - 175 + Oxidation (M)	[illegible]	p00885	Arylsulfatase K
ELAVL1 seq: 105 - 115 no mod	[illegible]	p18969	ELAV-like protein 1
EVL seq: 235 - 252 + Phospho (ST)	[illegible]	p00933	Ena/VASP-like protein
G6PD seq: 98 - 104 no mod	[illegible]	p12005	Glucose-6-phosphate 1-dehydrogenase
VCP seq: 324 - 336 no mod	[illegible]	p13529	Transitional endoplasmic reticulum ATPase
KIAA0100 seq: 908 - 914 no mod	[illegible]	p10977	Protein KIAA0100
SHROOM2 seq: 408 - 427 + Phospho (ST)	[illegible]	p11034	Protein Shroom2
PPFIBP2 seq: 509 - 526 + Phospho (ST)	[illegible]	p06498	Liprin-beta-2
FASN seq: 1712 - 1724 no mod	[illegible]	p16531	Fatty acid synthase
APRT seq: 15 - 27 no mod	[illegible]	p05870	Adenine phosphoribosyltransferase
IDH1 seq: 375 - 381 no mod	[illegible]	p18654	Isocitrate dehydrogenase [NADP] cytoplasmic
WNK1 seq: 1273 - 1311 + Oxidation (M); Phospho (ST)	[illegible]	p01535	Serine/threonine-protein kinase WNK1
TCOF1 pS233	[illegible]	p19466	Treacle protein
ADD1 pS586	[illegible]	p12674	Alpha-adducin
RPS6 pS236 pS240	[illegible]	p10654	40S ribosomal protein S6
FLNB seq: 1511 - 1519 no mod	[illegible]	p01193	Filamin-B
INTS4 seq: 780 - 786 + Oxidation (M)	[illegible]	p11450	Integrator complex subunit 4

SETX seq: 1980 - 1986 no mod	[illegible]	p11264	Probable helicase senataxin
KRT7 seq: 137 - 149 no mod	[illegible]	p11134	Keratin, type II cytoskeletal 7
DIP2B seq: 69 - 84 no mod	[illegible]	p09209	Disco-interacting protein 2 homolog B
ENO1 pM165 pM169	[illegible]	p12224	Alpha-enolase
GAPDH seq: 108 - 117 no mod	[illegible]	p20721	Glyceraldehyde-3-phosphate dehydrogenase
DCDC2 seq: 261 - 276 + Phospho (ST)	[illegible]	p03405	Doublecortin domain-containing protein 2
SH3D19 seq: 367 - 376 + Phospho (ST)	[illegible]	p03727	SH3 domain-containing protein 19
PRKRA pS18	[illegible]	p17867	Interferon-inducible double-stranded RNA-dependent protein kinase activator A
CANX seq: 78 - 87 no mod	[illegible]	p15085	Calnexin
EZR seq: 413 - 427 no mod	[illegible]	p04264	Ezrin
SFPQ seq: 350 - 358 no mod	[illegible]	p20828	Splicing factor, proline- and glutamine-rich
STIP1 seq: 506 - 513 no mod	[illegible]	p11580	Stress-induced-phosphoprotein 1
SERPINB8 seq: 69 - 79 no mod	[illegible]	p16100	Serpin B8
PGM3 seq: 493 - 505 no mod	[illegible]	p20734	Phosphoacetylglucosamine mutase
TPM2 seq: 169 - 178 no mod	[illegible]	p10411	Tropomyosin beta chain
PRDX5 seq: 58 - 83 no mod	[illegible]	p01517	Peroxiredoxin-5, mitochondrial
ANP32B seq: 76 - 86 no mod	[illegible]	p14310	Acidic leucine-rich nuclear phosphoprotein 32 family member B
P4HB seq: 223 - 230 no mod	[illegible]	p17336	Protein disulfide-isomerase
AARS seq: 752 - 762 no mod	[illegible]	p13564	Alanine--tRNA ligase, cytoplasmic
PPIG seq: 739 - 754 + Phospho (ST)	[illegible]	p13259	Peptidyl-prolyl cis-trans isomerase G
P3H2 seq: 377 - 383 no mod	[illegible]	p11930	Prolyl 3-hydroxylase 2
FSIP2 seq: 5157 - 5163 no mod	[illegible]	p10574	Fibrous sheath-interacting protein 2
PDIA3 seq: 83 - 94 no mod	[illegible]	p01701	Protein disulfide-isomerase A3
RERE seq: 142 - 170 + Phospho (ST)	[illegible]	p04329	Arginine-glutamic acid dipeptide repeats protein
P4HB seq: 231 - 247 no mod	[illegible]	p14709	Protein disulfide-isomerase
PDIA6 seq: 374 - 386 no mod	[illegible]	p15291	Protein disulfide-isomerase A6
HSP90B1 seq: 672 - 682 no mod	[illegible]	p18713	Endoplasmin
SRRM1 pS636 pS638	[illegible]	p06440	Serine/arginine repetitive matrix protein 1
SEC16A seq: 769 - 802 + Phospho (ST)	[illegible]	p20585	Protein transport protein Sec16A
RAB11FIP1 pS958	[illegible]	p04579	Rab11 family-interacting protein 1
HDAC7 seq: 89 - 95 no mod	[illegible]	p12268	Histone deacetylase 7
HNRNPH1 seq: 300 - 316 no mod	[illegible]	p19392	Heterogeneous nuclear ribonucleoprotein H
SRSF1 seq: 31 - 38 no mod	[illegible]	p18761	Serine/arginine-rich splicing factor 1
PHGDH seq: 462 - 469 no mod	[illegible]	p18560	D-3-phosphoglycerate dehydrogenase
AHNAK seq: 2579 - 2589 no mod	[illegible]	p13711	Neuroblast differentiation-associated protein AHNAK
CAMK2D pT286	[illegible]	p08705	Calcium/calmodulin-dependent protein kinase type II subunit delta
NOLC1 seq: 497 - 510 no mod	[illegible]	p01813	Nucleolar and coiled-body phosphoprotein 1
REPS2 pS260	[illegible]	p05808	RalBP1-associated Eps domain-containing protein 2
RASGRF2 seq: 650 - 655 + Phospho (Y)	[illegible]	p00440	Ras-specific guanine nucleotide-releasing factor 2
RASGRF2 seq: 650 - 655 + Phospho (ST)	[illegible]	p00441	Ras-specific guanine nucleotide-releasing factor 2
GAPVD1 seq: 735 - 754 + Phospho (ST)	[illegible]	p11009	GTPase-activating protein and VPS9 domain-containing protein 1
HSPH1 seq: 656 - 667 no mod	[illegible]	p16697	Heat shock protein 105 kDa
PA2G4 seq: 356 - 364 no mod	[illegible]	p20231	Proliferation-associated protein 2G4
HSPA5 seq: 139 - 152 no mod	[illegible]	p02876	78 kDa glucose-regulated protein
HSPA1A seq: 417 - 447 no mod	[illegible]	p09058	Heat shock 70 kDa protein 1A
ANXA2P2 seq: 38 - 47 no mod	[illegible]	p18972	Putative annexin A2-like protein
KRT8 seq: 24 - 32 no mod	[illegible]	p03114	Keratin, type II cytoskeletal 8
DDX5 seq: 228 - 236 no mod	[illegible]	p11615	Probable ATP-dependent RNA helicase DDX5
RPS6 seq: 233 - 243 + 2 Phospho (ST)	[illegible]	p07084	40S ribosomal protein S6
RPS6 seq: 234 - 249 + 2 Phospho (ST)	[illegible]	p10655	40S ribosomal protein S6
ATRX seq: 1988 - 2019 + 2 Phospho (ST)	[illegible]	p19308	Transcriptional regulator ATRX
CTDSPL2 pS9	[illegible]	p13381	CTD small phosphatase-like protein 2
UBA1 seq: 831 - 843 no mod	[illegible]	p19362	Ubiquitin-like modifier-activating enzyme 1
FLNB seq: 1231 - 1241 no mod	[illegible]	p14101	Filamin-B
STIP1 seq: 534 - 543 no mod	[illegible]	p11240	Stress-induced-phosphoprotein 1
CAMSAP3 seq: 337 - 357 + Phospho (ST)	[illegible]	p15111	Calmodulin-regulated spectrin-associated protein 3
CELSR3 pS3140	[illegible]	p16732	Cadherin EGF LAG seven-pass G-type receptor 3
ACTN4 seq: 522 - 535 + Gln->pyro-Glu (N-term Q)	[illegible]	p08399	Alpha-actinin-4
GPI seq: 195 - 211 no mod	[illegible]	p02632	Glucose-6-phosphate isomerase
AARS seq: 154 - 165 + Gln->pyro-Glu (N-term Q)	[illegible]	p08480	Alanine--tRNA ligase, cytoplasmic
PSMC3 seq: 335 - 344 no mod	[illegible]	p01681	26S protease regulatory subunit 6A
UNCX pS443	[illegible]	p04704	Homeobox protein unc-4 homolog
HSPD1 seq: 293 - 301 no mod	[illegible]	p01466	60 kDa heat shock protein, mitochondrial
TBC1D4 pT752 pS754	[illegible]	p02090	TBC1 domain family member 4
FRYL seq: 1061 - 1070 + Phospho (ST)	[illegible]	p06672	Protein furry homolog-like
FUBP3 seq: 300 - 308 no mod	[illegible]	p13077	Far upstream element-binding protein 3
PPIB seq: 146 - 158 no mod	[illegible]	p18162	Peptidyl-prolyl cis-trans isomerase B
TXNDC12 seq: 115 - 123 no mod	[illegible]	p14047	Thioredoxin domain-containing protein 12
HSP90B1 seq: 614 - 623 no mod	[illegible]	p17806	Endoplasmin
PKM seq: 174 - 186 no mod	[illegible]	p13482	Pyruvate kinase PKM
HADH seq: 193 - 202 no mod	[illegible]	p02868	Hydroxyacyl-coenzyme A dehydrogenase, mitochondrial
SRRM1 pS725 pT727	[illegible]	p06430	Serine/arginine repetitive matrix protein 1
HSP90AB1 seq: 281 - 289 no mod	[illegible]	p19987	Heat shock protein HSP 90-beta
HSPA4 seq: 222 - 234 no mod	[illegible]	p01271	Heat shock 70 kDa protein 4
MAPT pS411	[illegible]	p09195	Microtubule-associated protein tau
BAG4 pS179	[illegible]	p03891	BAG family molecular chaperone regulator 4
AGR2 seq: 128 - 138 no mod	[illegible]	p13961	Anterior gradient protein 2 homolog
PLEKHA6 pS777	[illegible]	p04351	Pleckstrin homology domain-containing family A member 6
CCT3 seq: 238 - 248 no mod	[illegible]	p13546	T-complex protein 1 subunit gamma
HSP90AB4P seq: 2 - 12 no mod	[illegible]	p05183	Putative heat shock protein HSP 90-beta 4
DDX5 seq: 242 - 254 no mod	[illegible]	p16224	Probable ATP-dependent RNA helicase DDX5
ACO2 seq: 145 - 160 no mod	[illegible]	p18739	Aconitate hydratase, mitochondrial
FASN seq: 2221 - 2233 no mod	[illegible]	p05141	Fatty acid synthase
ANXA2 seq: 274 - 279 no mod	[illegible]	p10307	Annexin A2
MAPRE1 seq: 215 - 220 no mod	[illegible]	p18846	Microtubule-associated protein RP/EB family member 1
CPNE7 seq: 501 - 509 no mod	[illegible]	p18699	Copine-7
PADI2 seq: 641 - 652 no mod	[illegible]	p16616	Protein-arginine deiminase type-2
AHNAK pS5746 pS5749 pS5752	[illegible]	p16214	Neuroblast differentiation-associated protein AHNAK
PKM seq: 343 - 376 no mod	[illegible]	p20830	Pyruvate kinase PKM
KIAA1671 pT1713	[illegible]	p20810	Uncharacterized protein KIAA1671
TMEM57 seq: 297 - 320 + Phospho (ST)	[illegible]	p07163	Macoilin
ETFA seq: 86 - 101 no mod	[illegible]	p15707	Electron transfer flavoprotein subunit alpha, mitochondrial
KRT19 seq: 212 - 223 no mod	[illegible]	p09871	Keratin, type I cytoskeletal 19
GSPT2 seq: 449 - 456 no mod	[illegible]	p08758	Eukaryotic peptide chain release factor GTP-binding subunit ERF3B
ABCA2 seq: 1057 - 1064 no mod	[illegible]	p02904	ATP-binding cassette sub-family A member 2
HNRNPF seq: 91 - 98 no mod	[illegible]	p02901	Heterogeneous nuclear ribonucleoprotein F
PKN1 seq: 772 - 794 + Phospho (ST)	[illegible]	p02077	Serine/threonine-protein kinase N1
UNKL pS15	[illegible]	p21207	Putative E3 ubiquitin-protein ligase UNKL
CDC42BPA seq: 1719 - 1732 + Phospho (ST)	[illegible]	p05046	Serine/threonine-protein kinase MRCK alpha
HSP90B1 seq: 475 - 486 no mod	[illegible]	p14529	Endoplasmin
PPP6R3 seq: 304 - 310 no mod	[illegible]	p03322	Serine/threonine-protein phosphatase 6 regulatory subunit 3
CD2BP2 seq: 188 - 198 + Phospho (ST)	[illegible]	p13178	CD2 antigen cytoplasmic tail-binding protein 2
POLR2I seq: 58 - 80 no mod	[illegible]	p13612	DNA-directed RNA polymerase II subunit RPB9
SOGA1 seq: 1301 - 1332 + 2 Phospho (ST)	[illegible]	p19891	Protein SOGA1
EIF2B1 seq: 103 - 108 + Phospho (ST)	[illegible]	p07247	Translation initiation factor eIF-2B subunit alpha
LLGL2 seq: 958 - 978 + 2 Phospho (ST)	[illegible]	p19702	Lethal(2) giant larvae protein homolog 2
C1orf174 pS189	[illegible]	p08224	UPF0688 protein C1orf174
PSAP seq: 439 - 449 no mod	[illegible]	p16237	Prosaposin
ZYX seq: 280 - 295 no mod	[illegible]	p16455	Zyxin
ACO2 seq: 145 - 160 no mod	[illegible]	p18738	Aconitate hydratase, mitochondrial
RPS3A seq: 66 - 82 no mod	[illegible]	p01560	40S ribosomal protein S3a
TBL1X seq: 35 - 47 + Phospho (ST)	[illegible]	p17749	F-box-like/WD repeat-containing protein TBL1X
FLNB seq: 2206 - 2219 no mod	[illegible]	p16184	Filamin-B
PSMB6 seq: 221 - 230 + Gln->pyro-Glu (N-term Q)	[illegible]	p07721	Proteasome subunit beta type-6
FASN seq: 1159 - 1171 no mod	[illegible]	p09812	Fatty acid synthase
GAPDH seq: 67 - 80 no mod	[illegible]	p10409	Glyceraldehyde-3-phosphate dehydrogenase
CLTC seq: 1610 - 1620 no mod	[illegible]	p01675	Clathrin heavy chain 1
SPTBN1 seq: 91 - 99 no mod	[illegible]	p11402	Spectrin beta chain, non-erythrocytic 1
RPS6 pS236 pS240	[illegible]	p10657	40S ribosomal protein S6
CD2BP2 pS195	[illegible]	p13177	CD2 antigen cytoplasmic tail-binding protein 2
LARP1 seq: 845 - 871 + Phospho (ST)	[illegible]	p03031	La-related protein 1
NELFE pS181	[illegible]	p05793	Negative elongation factor E
IRS1 seq: 328 - 353 + Phospho (ST)	[illegible]	p19508	Insulin receptor substrate 1
FLNB seq: 2059 - 2074 no mod	[illegible]	p00755	Filamin-B
NQO1 seq: 60 - 77 no mod	[illegible]	p11515	NAD(P)H dehydrogenase [quinone] 1
SYAP1 seq: 259 - 291 + Phospho (ST)	[illegible]	p02287	Synapse-associated protein 1
GRIP1 pT972	[illegible]	p04742	Glutamate receptor-interacting protein 1
PACSIN3 pS319	[illegible]	p15976	Protein kinase C and casein kinase substrate in neurons protein 3
NCL pS67	[illegible]	p00561	Nucleolin
HSPA8 seq: 362 - 384 no mod	[illegible]	p05506	Heat shock cognate 71 kDa protein
TCEA2 seq: 5 - 14 no mod	[illegible]	p17844	Transcription elongation factor A protein 2
SRSF1 seq: 143 - 154 no mod	[illegible]	p17971	Serine/arginine-rich splicing factor 1
RUVBL1 seq: 379 - 400 no mod	[illegible]	p19743	RuvB-like 1
MLPH seq: 145 - 185 + 2 Phospho (ST)	[illegible]	p10999	Melanophilin
PPP4R3A seq: 104 - 139 + Phospho (ST)	[illegible]	p18394	Serine/threonine-protein phosphatase 4 regulatory subunit 3A
RPL28 seq: 40 - 46 no mod	[illegible]	p00206	60S ribosomal protein L28
NONO seq: 127 - 135 no mod	[illegible]	p01623	Non-POU domain-containing octamer-binding protein
LTBP1 seq: 1406 - 1437 + Phospho (ST)	[illegible]	p16084	Latent-transforming growth factor beta-binding protein 1
TRIM3 pS7	[illegible]	p07488	Tripartite motif-containing protein 3
TLN1 seq: 2032 - 2043 no mod	[illegible]	p01120	Talin-1
TLN1 seq: 2090 - 2099 no mod	[illegible]	p20261	Talin-1
VCP seq: 454 - 465 no mod	[illegible]	p00510	Transitional endoplasmic reticulum ATPase
PDIA3 seq: 219 - 226 no mod	[illegible]	p01978	Protein disulfide-isomerase A3
PRDX5 seq: 149 - 159 no mod	[illegible]	p11470	Peroxiredoxin-5, mitochondrial
NOLC1 seq: 187 - 193 no mod	[illegible]	p13584	Nucleolar and coiled-body phosphoprotein 1
GSN seq: 742 - 748 no mod	[illegible]	p02385	Gelsolin
STRN seq: 222 - 235 + Phospho (ST)	[illegible]	p06014	Striatin
GSN seq: 361 - 368 no mod	[illegible]	p03053	Gelsolin
MAPRE2 seq: 2 - 30 no mod	[illegible]	p08893	Microtubule-associated protein RP/EB family member 2
HSPA1A seq: 349 - 357 no mod	[illegible]	p11318	Heat shock 70 kDa protein 1A
ARHGDIB seq: 125 - 131 no mod	[illegible]	p00016	Rho GDP-dissociation inhibitor 2
VDAC1 seq: 21 - 28 no mod	[illegible]	p14932	Voltage-dependent anion-selective channel protein 1
BUD13 pT135 pS139	[illegible]	p14779	BUD13 homolog
PIWIL2 pM92	[illegible]	p17358	Piwi-like protein 2
ATP5A1 seq: 208 - 214 no mod	[illegible]	p17471	ATP synthase subunit alpha, mitochondrial
ZC3HAV1L seq: 249 - 268 + Phospho (ST)	[illegible]	p10032	Zinc finger CCCH-type antiviral protein 1-like
KCTD3 pS711	[illegible]	p12565	BTB/POZ domain-containing protein KCTD3
GRB7 seq: 60 - 91 + Phospho (ST)	[illegible]	p07542	Growth factor receptor-bound protein 7
TCOF1 pS233	[illegible]	p19464	Treacle protein
WWC2 seq: 220 - 226 no mod	[illegible]	p20457	Protein WWC2
SHISA8 seq: 178 - 184 no mod	[illegible]	p20135	Putative protein shisa-8
SRRM1 pS626 pS628	[illegible]	p07563	Serine/arginine repetitive matrix protein 1
S100A14 seq: 21 - 27 no mod	[illegible]	p20481	Protein S100-A14
PDHA2 pS291 pS298	[illegible]	p00327	Pyruvate dehydrogenase E1 component subunit alpha, testis-specific form, mitochondrial
MATR3 seq: 103 - 120 no mod	[illegible]	p16232	Matrin-3
SRRM1 pS738 pS740 pS743	[illegible]	p13469	Serine/arginine repetitive matrix protein 1
TJP3 pS346	[illegible]	p02695	Tight junction protein ZO-3
TXNDC5 seq: 288 - 296 no mod	[illegible]	p16888	Thioredoxin domain-containing protein 5
TANK pS354	[illegible]	p21190	TRAF family member-associated NF-kappa-B activator
TPD52L2 seq: 120 - 127 no mod	[illegible]	p00687	Tumor protein D54
TANC2 pS1740	[illegible]	p03642	Protein TANC2
YTHDF3 seq: 361 - 397 no mod	[illegible]	p16324	YTH domain-containing family protein 3
MYH9 seq: 1278 - 1295 no mod	[illegible]	p10917	Myosin-9
ACAT1 seq: 41 - 49 no mod	[illegible]	p16935	Acetyl-CoA acetyltransferase, mitochondrial
ULK1 pS758	[illegible]	p20649	Serine/threonine-protein kinase ULK1
TWF1 pS143	[illegible]	p00258	Twinfilin-1
GOLGB1 seq: 1519 - 1532 no mod	[illegible]	p05322	Golgin subfamily B member 1
TPI1 seq: 57 - 70 no mod	[illegible]	p07890	Triosephosphate isomerase
XRCC6 seq: 36 - 46 no mod	[illegible]	p18262	X-ray repair cross-complementing protein 6
EEF1A1P5 seq: 85 - 96 no mod	[illegible]	p00002	Putative elongation factor 1-alpha-like 3
PSMA7 seq: 219 - 227 no mod	[illegible]	p14013	Proteasome subunit alpha type-7
ACAT2 seq: 201 - 210 no mod	[illegible]	p17582	Acetyl-CoA acetyltransferase, cytosolic
DOPEY2 pS1169	[illegible]	p06320	Protein dopey-2
ZNF777 seq: 6 - 21 + Phospho (ST)	[illegible]	p03803	Zinc finger protein 777
ZC3H14 seq: 43 - 61 no mod	[illegible]	p04280	Zinc finger CCCH domain-containing protein 14
HNRNPA2B1 pM53	[illegible]	p10578	Heterogeneous nuclear ribonucleoproteins A2/B1
HSPA9 seq: 349 - 360 no mod	[illegible]	p19817	Stress-70 protein, mitochondrial
CCT3 seq: 32 - 38 no mod	[illegible]	p02737	T-complex protein 1 subunit gamma
CDO1 seq: 11 - 17 no mod	[illegible]	p02638	Cysteine dioxygenase type 1
TUFM seq: 239 - 252 no mod	[illegible]	p11459	Elongation factor Tu, mitochondrial
ACO2 seq: 634 - 648 no mod	[illegible]	p09735	Aconitate hydratase, mitochondrial
SYNCRIP seq: 185 - 192 no mod	[illegible]	p20602	Heterogeneous nuclear ribonucleoprotein Q
EPS8L1 pT187	[illegible]	p04736	Epidermal growth factor receptor kinase substrate 8-like protein 1
FAM193A pS293	[illegible]	p11574	Protein FAM193A
PRDX3 seq: 171 - 184 no mod	[illegible]	p18042	Thioredoxin-dependent peroxide reductase, mitochondrial
ACTN4 pM379	[illegible]	p09815	Alpha-actinin-4
UFL1 seq: 539 - 552 no mod	[illegible]	p18554	E3 UFM1-protein ligase 1
CS seq: 35 - 43 no mod	[illegible]	p18743	Citrate synthase, mitochondrial
PLIN3 seq: 239 - 245 no mod	[illegible]	p11694	Perilipin-3
EWSR1 seq: 269 - 292 + Gln->pyro-Glu (N-term Q)	[illegible]	p08770	RNA-binding protein EWS
SCAF4 pS154	[illegible]	p14350	Splicing factor, arginine/serine-rich 15
FSCN1 seq: 111 - 118 no mod	[illegible]	p00385	Fascin
MYH9 seq: 1418 - 1433 no mod	[illegible]	p10954	Myosin-9
RPS6 seq: 234 - 249 + Phospho (ST)	[illegible]	p10653	40S ribosomal protein S6
ATP5B seq: 125 - 133 no mod	[illegible]	p01253	ATP synthase subunit beta, mitochondrial
DDX5 seq: 452 - 461 + Gln->pyro-Glu (N-term Q)	[illegible]	p07693	Probable ATP-dependent RNA helicase DDX5
TC2N pS168	[illegible]	p02486	Tandem C2 domains nuclear protein
EIF2S3 seq: 91 - 103 no mod	[illegible]	p12092	Eukaryotic translation initiation factor 2 subunit 3
PEX1 seq: 1179 - 1193 + Phospho (ST)	[illegible]	p03015	Peroxisome biogenesis factor 1
EWSR1 seq: 411 - 424 no mod	[illegible]	p16231	RNA-binding protein EWS

SRSF2 pS26	p02157	Serine/arginine-rich splicing factor 2
FLNB seq: 2476 - 2495 no mod	p10349	Filamin-B
HOMER3 pS256	p17726	Homer protein homolog 3
HYOU1 seq: 252 - 262 no mod	p17965	Hypoxia up-regulated protein 1
POLH pS380	p07085	DNA polymerase eta
PRKCD seq: 644 - 669 + Phospho (ST)	p10588	Protein kinase C delta type
CLIC1 seq: 166 - 183 no mod	p13242	Chloride intracellular channel protein 1
BOD1L1 pS800	p12896	Biorientation of chromosomes in cell division protein 1-like 1
CD81 seq: 125 - 144 + Gln->pyro-Glu (N-term Q)	p08666	CD81 antigen
AHNAK seq: 5356 - 5366 no mod	p11148	Neuroblast differentiation-associated protein AHNAK
RBM27 pS120	p12339	RNA-binding protein 27
ACIN1 pS729	p10965	Apoptotic chromatin condensation inducer in the nucleus
G6PD seq: 433 - 439 no mod	p11138	Glucose-6-phosphate 1-dehydrogenase
PDIA4 seq: 560 - 570 no mod	p08394	Protein disulfide-isomerase A4
ENO2 seq: 203 - 228 no mod	p18943	Gamma-enolase
EPPK1 pS2716	p07696	Epiplakin
NUCKS1 pS58	p09096	Nuclear ubiquitous casein and cyclin-dependent kinase substrate 1
SCP2 seq: 471 - 479 no mod	p17907	Non-specific lipid-transfer protein
YWHAG seq: 126 - 132 no mod	p10050	14-3-3 protein gamma
NFATC1 pS233	p15746	Nuclear factor of activated T-cells, cytoplasmic 1
HIST1H4A pS48	p07254	Histone H4
ZC3H13 pS1208	p10862	Zinc finger CCCH domain-containing protein 13
AMOT seq: 707 - 726 + Phospho (ST)	p18146	Angiomotin
EPPK1 pT34	p20104	Epiplakin
CCT5 seq: 503 - 513 + Gln->pyro-Glu (N-term Q)	p08056	T-complex protein 1 subunit epsilon
TLN1 seq: 862 - 869 no mod	p14098	Talin-1
CENPF seq: 588 - 596 + Phospho (ST)	p10770	Centromere protein F
UBE2I seq: 50 - 59 no mod	p15074	SUMO-conjugating enzyme UBC9
UBE2N seq: 131 - 141 no mod	p02492	Ubiquitin-conjugating enzyme E2 N
RPS6 pS240	p10656	40S ribosomal protein S6
EFS seq: 305 - 329 + 2 Phospho (ST)	p06917	Embryonal Fyn-associated substrate
HSPA4 seq: 391 - 405 no mod	p17785	Heat shock 70 kDa protein 4
EIF4EBP1 seq: 20 - 51 + Phospho (ST)	p06418	Eukaryotic translation initiation factor 4E-binding protein 1
ATP5A1 seq: 435 - 441 no mod	p11967	ATP synthase subunit alpha, mitochondrial
HNRNPK seq: 378 - 396 no mod	p15137	Heterogeneous nuclear ribonucleoprotein K
MAPT seq: 724 - 755 + Oxidation (M); Phospho (ST)	p14733	Microtubule-associated protein tau
LIMK2 seq: 287 - 300 + Phospho (ST)	p04793	LIM domain kinase 2
SRSF11 pS449	p12782	Serine/arginine-rich splicing factor 11
LMNB1 seq: 458 - 473 no mod	p09004	Lamin-B1
RAP1GAP seq: 604 - 630 + Phospho (ST)	p07515	Rap1 GTPase-activating protein 1
UBFD1 seq: 52 - 83 no mod	p15245	Ubiquitin domain-containing protein UBFD1
FLNB seq: 554 - 566 no mod	p19136	Filamin-B
HSP90AA1 pS263	p17172	Heat shock protein HSP 90-alpha
CCT5 seq: 484 - 496 no mod	p17357	T-complex protein 1 subunit epsilon
IDH1 seq: 225 - 233 no mod	p18755	Isocitrate dehydrogenase [NADP] cytoplasmic
VWA5B2 pS768	p04991	von Willebrand factor A domain-containing protein 5B2
HIRIP3 pS196	p17817	HIRA-interacting protein 3
SMARCC2 seq: 318 - 326 + Phospho (ST)	p13172	SWI/SNF complex subunit SMARCC2
ACTN1 seq: 715 - 726 no mod	p01427	Alpha-actinin-1
HNRNPM seq: 202 - 214 no mod	p11678	Heterogeneous nuclear ribonucleoprotein M
TUBB seq: 363 - 379 + Oxidation (M)	p10229	Tubulin beta chain
PPIAL4G seq: 50 - 55 no mod	p15309	Peptidyl-prolyl cis-trans isomerase A-like 4G
seq: 740 - 757 + Gln->pyro-Glu (N-term Q); Phospho (ST)	p08806	Protein SMG8
TPD52 seq: 188 - 224 no mod	p01482	Tumor protein D52
HECTD4 seq: 1105 - 1124 + Phospho (ST)	p04881	Probable E3 ubiquitin-protein ligase HECTD4
MEF2A seq: 95 - 114 + Phospho (ST)	p16245	Myocyte-specific enhancer factor 2A
FASN seq: 1695 - 1705 no mod	p01522	Fatty acid synthase
NUP98 seq: 604 - 633 + 2 Phospho (ST)	p09430	Nuclear pore complex protein Nup98-Nup96
PKP4 seq: 1164 - 1175 + Phospho (Y)	p03414	Plakophilin-4
YWHAG seq: 199 - 217 no mod	p03096	14-3-3 protein gamma
TRIP12 seq: 1419 - 1429 + Phospho (ST)	p19744	E3 ubiquitin-protein ligase TRIP12
EIF6 seq: 224 - 237 no mod	p11199	Eukaryotic translation initiation factor 6
PTCD3 seq: 271 - 286 no mod	p19130	Pentatricopeptide repeat domain-containing protein 3, mitochondrial
SIPA1L1 pS255	p15286	Signal-induced proliferation-associated 1-like protein 1
APEX1 seq: 64 - 73 no mod	p14452	DNA-(apurinic or apyrimidinic site) lyase
PPP4R3A seq: 104 - 139 + Phospho (ST)	p18393	Serine/threonine-protein phosphatase 4 regulatory subunit 3A
NPM1 seq: 81 - 101 + Oxidation (M)	p09867	Nucleophosmin
GAPDH seq: 264 - 271 no mod	p08827	Glyceraldehyde-3-phosphate dehydrogenase
TP53BP1 seq: 313 - 348 + Phospho (ST)	p01893	Tumor suppressor p53-binding protein 1
NPM1 seq: 222 - 229 no mod	p05413	Nucleophosmin
SAP30L seq: 91 - 120 + 2 Phospho (ST)	p12471	Histone deacetylase complex subunit SAP30L
TUFM seq: 316 - 327 no mod	p20872	Elongation factor Tu, mitochondrial
A seq: 11 - 51 + Gln->pyro-Glu (N-term Q); Phospho (ST)	p08201	Protein transport protein Sec16A
ATP5J seq: 100 - 108 no mod	p16788	ATP synthase-coupling factor 6, mitochondrial
TPI1 seq: 71 - 90 no mod	p01035	Triosephosphate isomerase
UBA1 pS46	p09597	Ubiquitin-like modifier-activating enzyme 1
UBA1 seq: 650 - 657 no mod	p18902	Ubiquitin-like modifier-activating enzyme 1
SRRM2 pS1987	p03959	Serine/arginine repetitive matrix protein 2
BLVRA seq: 254 - 261 no mod	p18369	Biliverdin reductase A
PAK4 pS474	p04931	Serine/threonine-protein kinase PAK 4
ALOX12 seq: 243 - 250 + Phospho (ST)	p06554	Arachidonate 12-lipoxygenase, 12S-type
PARD3B seq: 744 - 758 + Phospho (ST)	p03693	Partitioning defective 3 homolog B
HNRNPH3 seq: 206 - 222 no mod	p19395	Heterogeneous nuclear ribonucleoprotein H3
PSMC3 seq: 251 - 266 no mod	p12256	26S protease regulatory subunit 6A
DUSP16 seq: 506 - 530 + Phospho (ST)	p05643	Dual specificity protein phosphatase 16
FRYL seq: 2330 - 2349 + Phospho (ST)	p03437	Protein furry homolog-like
NPM1 seq: 81 - 101 no mod	p09868	Nucleophosmin
DDX20 seq: 672 - 693 + Phospho (ST)	p03160	Probable ATP-dependent RNA helicase DDX20
THRAP3 pS248	p17204	Thyroid hormone receptor-associated protein 3
SRSF9 pS204	p15353	Serine/arginine-rich splicing factor 9
ACTB seq: 197 - 206 + Phospho (ST)	p14915	Actin, cytoplasmic 1
MYH9 pS1943	p13219	Myosin-9
SIPA1L3 pS1685	p19190	Signal-induced proliferation-associated 1-like protein 3
SRSF9 pS204	p15352	Serine/arginine-rich splicing factor 9
ARHGEF26 pS22	p05497	Rho guanine nucleotide exchange factor 26
TNRC6B seq: 430 - 451 no mod	p15443	Trinucleotide repeat-containing gene 6B protein
SP4 pS757	p16046	Transcription factor Sp4
ARVCF pT642	p09656	Armadillo repeat protein deleted in velo-cardio-facial syndrome
SOS2 pS1315	p17403	Son of sevenless homolog 2
ULK1 seq: 401 - 417 + Phospho (ST)	p02320	Serine/threonine-protein kinase ULK1
GNL1 seq: 44 - 61 + 2 Phospho (ST)	p07484	Guanine nucleotide-binding protein-like 1
HBP1 seq: 378 - 393 + Phospho (ST)	p07578	HMG box-containing protein 1
PRKAR2A pS99	p06425	cAMP-dependent protein kinase type II-alpha regulatory subunit
SHROOM2 seq: 762 - 776 + Phospho (ST)	p03141	Protein Shroom2
CCDC85A pS255	p06247	Coiled-coil domain-containing protein 85A
PKM seq: 271 - 278 no mod	p14342	Pyruvate kinase PKM
FLNB seq: 787 - 810 no mod	p01179	Filamin-B
CAPNS1 pM239	p12095	Calpain small subunit 1
TCEAL3 pS65	p17892	Transcription elongation factor A protein-like 3
FUBP1 pS140	p13817	Far upstream element-binding protein 1
HSPB1 pS98	p00832	Heat shock protein beta-1
SHANK2 seq: 94 - 101 + Phospho (ST)	p04019	SH3 and multiple ankyrin repeat domains protein 2
STK11IP pS398	p07580	Serine/threonine-protein kinase 11-interacting protein
HSPA5 seq: 327 - 336 no mod	p16797	78 kDa glucose-regulated protein
PKMYT1 pS143	p04868	Membrane-associated tyrosine- and threonine-specific cdc2-inhibitory kinase
HBP1 seq: 134 - 144 + Phospho (ST)	p03764	HMG box-containing protein 1
CSTF2 seq: 181 - 189 no mod	p13562	Cleavage stimulation factor subunit 2
ABI1 seq: 321 - 334 + Phospho (ST)	p14702	Abl interactor 1
SRI seq: 77 - 84 no mod	p11216	Sorcin
NUCKS1 pS58 pS61	p05738	Nuclear ubiquitous casein and cyclin-dependent kinase substrate 1
RGPD3 pT2450 pS2454	p18261	RanBP2-like and GRIP domain-containing protein 3
MLLT4 pS1275	p04270	Afadin
q: 230 - 268 + Gln->pyro-Glu (N-term Q); 2 Phospho (ST)	p07904	Chromosome alignment-maintaining phosphoprotein 1
EIF4H seq: 192 - 198 no mod	p15240	Eukaryotic translation initiation factor 4H
UHRF1BP1L seq: 987 - 1000 + Phospho (ST)	p05694	UHRF1-binding protein 1-like
MYH9 pS1943	p13218	Myosin-9
RPL23 seq: 92 - 109 no mod	p18792	60S ribosomal protein L23
HNRNPM seq: 592 - 606 no mod	p10104	Heterogeneous nuclear ribonucleoprotein M
AHCTF1 pS528	p19038	Protein ELYS
OXSR1 pS339	p11646	Serine/threonine-protein kinase OSR1
C2CD2L pS662	p17967	C2 domain-containing protein 2-like
NAB2 pS6	p19895	NGFI-A-binding protein 2
KRT8 seq: 313 - 325 + Phospho (ST)	p09534	Keratin, type II cytoskeletal 8
PRPF40B pS832	p17416	Pre-mRNA-processing factor 40 homolog B
TUBB seq: 263 - 276 no mod	p11652	Tubulin beta chain
WAC seq: 61 - 77 + Phospho (ST)	p06692	WW domain-containing adapter protein with coiled-coil
WWTR1 seq: 87 - 114 + Phospho (ST)	p05556	WW domain-containing transcription regulator protein 1
CBX8 seq: 253 - 274 + Phospho (ST)	p06819	Chromobox protein homolog 8
TFIP11 seq: 9 - 39 no mod	p18832	Tuftelin-interacting protein 11
TPRN pS274	p01596	Taperin
TTLL12 seq: 10 - 40 + Phospho (ST)	p06978	Tubulin--tyrosine ligase-like protein 12
PRRC2C seq: 924 - 939 + Phospho (ST)	p03277	Protein PRRC2C
NFIL3 pS301	p15499	Nuclear factor interleukin-3-regulated protein
TNKS1BP1 seq: 701 - 726 + Phospho (ST)	p07403	182 kDa tankyrase-1-binding protein
TANC2 pS1827	p06160	Protein TANC2
GORASP2 pS451	p19865	Golgi reassembly-stacking protein 2
C1QBP seq: 129 - 154 no mod	p13574	Complement component 1 Q subcomponent-binding protein, mitochondrial
1 seq: 63 - 92 + Gln->pyro-Glu (N-term Q); Phospho (ST)	p08338	MAP7 domain-containing protein 1
STMN1 pS38	p17093	Stathmin
ZMYND8 seq: 397 - 408 + Phospho (ST)	p01310	Protein kinase C-binding protein 1
BCAR3 seq: 288 - 299 + Oxidation (M); Phospho (ST)	p07112	Breast cancer anti-estrogen resistance protein 3
TAF3 pS755	p14102	Transcription initiation factor TFIID subunit 3
CAST pS297	p18164	Calpastatin
PER2 pS977	p19540	Period circadian protein homolog 2
CAMSAP3 pS814	p13226	Calmodulin-regulated spectrin-associated protein 3
AHDC1 pS1064	p19443	AT-hook DNA-binding motif-containing protein 1
AMOTL2 pS540	p08863	Angiomotin-like protein 2
ADNP pS953 pS955	p11233	Activity-dependent neuroprotector homeobox protein
MKI67 pS2002	p11506	Antigen KI-67
ZMYM2 pS305	p09226	Zinc finger MYM-type protein 2
RBBP8NL pS145	p20406	RBBP8 N-terminal-like protein
DHX16 pS160	p07998	Putative pre-mRNA-splicing factor ATP-dependent RNA helicase DHX16
RGPD8 seq: 2441 - 2455 + Phospho (ST)	p03059	RANBP2-like and GRIP domain-containing protein 8
FADD pM170	p06139	FAS-associated death domain protein
PIK3C2A pS259	p00814	Phosphatidylinositol 4-phosphate 3-kinase C2 domain-containing subunit alpha
ZMYM2 pS305	p08130	Zinc finger MYM-type protein 2
KATNB1 seq: 390 - 398 + Phospho (ST)	p09154	Katanin p80 WD40 repeat-containing subunit B1
JUN pS63	p09167	Transcription factor AP-1
BCL2L12 pS273	p20949	Bcl-2-like protein 12
TNKS1BP1 pS178	p13263	182 kDa tankyrase-1-binding protein
seq: 736 - 770 + Gln->pyro-Glu (N-term Q); Phospho (ST)	p08692	Palladin
AKR1A1 seq: 276 - 287 no mod	p01573	Alcohol dehydrogenase [NADP(+)]
STMN1 pS38	p05424	Stathmin
EPN1 seq: 446 - 468 + Phospho (ST)	p15257	Epsin-1
STAU2 pS455	p18485	Double-stranded RNA-binding protein Staufen homolog 2
LARP1 seq: 822 - 839 + Phospho (ST)	p14605	La-related protein 1
BAIAP2L1 pT257	p02415	Brain-specific angiogenesis inhibitor 1-associated protein 2-like protein 1
PALLD seq: 736 - 770 + Phospho (ST)	p08690	Palladin
XRCC1 seq: 256 - 271 + Phospho (ST)	p12480	DNA repair protein XRCC1
PPP1R9A seq: 838 - 856 + Phospho (ST)	p06492	Neurabin-1
EPPK1 pS2716	p07697	Epiplakin
GOLGB1 pS17	p10792	Golgin subfamily B member 1
LMNA pS458	p09491	Prelamin-A/C
RNF31 pS466	p07151	E3 ubiquitin-protein ligase RNF31
PABPC4 seq: 84 - 89 no mod	p13933	Polyadenylate-binding protein 4
PABPC4L seq: 83 - 88 no mod	p11215	Polyadenylate-binding protein 4-like
PDLIM2 pS161	p05865	PDZ and LIM domain protein 2
GPKOW pS42	p12287	G patch domain and KOW motifs-containing protein
ARPC5L seq: 90 - 102 no mod	p03925	Actin-related protein 2/3 complex subunit 5-like protein
ZC3HAV1 seq: 374 - 385 + Phospho (ST)	p12459	Zinc finger CCCH-type antiviral protein 1
TPT1 seq: 39 - 85 no mod	p02912	Translationally-controlled tumor protein
LMNA seq: 12 - 25 + 2 Phospho (ST)	p05789	Prelamin-A/C
KIAA1217 seq: 1640 - 1654 + Phospho (ST)	p06817	Sickle tail protein homolog
RBM4B pS86	p11624	RNA-binding protein 4B
PPIL4 seq: 156 - 190 + Phospho (ST)	p13920	Peptidyl-prolyl cis-trans isomerase-like 4
FANCE seq: 238 - 260 + Phospho (ST)	p05246	Fanconi anemia group E protein
EXPH5 seq: 1070 - 1083 + Phospho (ST)	p16450	Exophilin-5
CRYBG3 pS636	p05596	Very large A-kinase anchor protein
PKM pS57	p03353	Pyruvate kinase PKM
SCRIB pS1448	p07868	Protein scribble homolog
SGOL1 seq: 457 - 463 + Phospho (ST)	p07116	Shugoshin-like 1
DNAJC21 pS370	p08050	DnaJ homolog subfamily C member 21
PLEKHA3 pS244	p01867	Pleckstrin homology domain-containing family A member 3
NOLC1 pS643	p01827	Nucleolar and coiled-body phosphoprotein 1
WDR62 pS1144	p20549	WD repeat-containing protein 62
STK17A pS28	p15648	Serine/threonine-protein kinase 17A
TXLNG seq: 94 - 115 + Phospho (ST)	p09388	Gamma-taxilin
RAF1 pS296 pS301	p05584	RAF proto-oncogene serine/threonine-protein kinase
MKL2 seq: 535 - 559 + Phospho (ST)	p09851	MKL/myocardin-like protein 2
IKBKG seq: 360 - 385 + Phospho (ST)	p14570	NF-kappa-B essential modulator

UBE2B seq: 140 - 152 no mod	9E+07	5E+08	5E+08	4E+08	4E+08	1E+09	8E+08	4E+08	4E+08	4E+08	1E+09	8E+08	5E+08	4E+08	4E+08	8E+08	3E+08	5E+08	5E+08	1E+08	7E+08	3E+08	3E+08	5E+08	3E+08	1E+09	3E+08	3E+08	5E+08	4E+08	5E+08	2E+08	2E+08	5E+08	4E+08	1E+09	3E+08	3E+08	3E+08	4E+08	1E+09	4E+08	3E+08	4E+08	4E+08	8E+08	3E+08	3E+08	4E+08	4E+08	p06448	Ubiquitin-conjugating enzyme E2 B
CASC3 pS265	4E+06	37149	39656	13654	3E+07	45779	25552	1E+07	34104	2E+06	8E+07	53206	39754	2E+06	1E+08	40867	33072	3E+06	19162	28832	5E+07	50401	8E+07	6E+06	7E+07	1E+08	8E+07	1E+08	1E+08	29103	2E+08	31655	5E+07	1E+08	1E+08	3E+08	27262	1E+08	2E+07	2E+08	2E+08	64618	1E+08	1E+08	1E+08	2E+08	2E+08	2E+08	8E+07	9E+07	p00342	Protein CASC3
ARFGEF2 pS218 pS227	1E+09	7E+08	1E+09	8E+08	5E+08	2E+09	1E+09	8E+08	7E+08	5E+08	3E+09	1E+09	2E+09	1E+09	9E+08	1E+09	6E+08	8E+08	7E+08	5E+08	1E+09	6E+08	8E+08	7E+08	6E+08	9E+08	7E+08	7E+08	6E+08	4E+08	9E+08	5E+08	4E+08	7E+08	5E+08	1E+09	7E+08	7E+08	6E+08	6E+08	1E+09	6E+08	7E+08	5E+08	4E+08	8E+08	7E+08	7E+08	5E+08	4E+08	p17506	Brefeldin A-inhibited guanine nucleotide-exchange protein 2
XRCC1 seq: 257 - 271 + Phospho (ST)	2E+08	2E+08	3E+08	2E+08	2E+08	4E+08	3E+08	2E+08	2E+08	2E+08	8E+08	4E+08	3E+08	4E+08	3E+08	3E+08	2E+08	2E+08	2E+08	4E+08	2E+08	2E+08	2E+08	5E+08	2E+08	2E+08	2E+08	3E+08	2E+08	1E+08	2E+08	7E+08	2E+08	4E+08	2E+08	2E+08	2E+08	2E+08	4E+08	2E+08	2E+08	2E+08	2E+08	3E+08	2E+08	1E+08	1E+08	1E+08	1E+08	2E+08	p02331	DNA repair protein XRCC1
TNRC6C seq: 554 - 578 + Phospho (ST)	3E+08	1E+08	1E+08	2E+08	2E+08	2E+09	5E+08	2E+08	2E+08	2E+08	3E+09	1E+09	3E+08	5E+08	3E+08	4E+08	7E+07	2E+08	3E+08	8E+07	6E+08	6E+07	4E+07	3E+08	1E+08	5E+08	1E+08	5E+07	8E+07	8E+07	45838	1E+08	19257	9E+07	3E+07	2E+08	8E+07	4E+07	27887	6E+07	4E+08	1E+08	38440	41974	4E+07	4E+08	1E+08	3E+07	6E+07	5E+07	p05745	Trinucleotide repeat-containing gene 6C protein
CIC seq: 559 - 586 + Phospho (ST)	2E+08	3E+08	2E+08	2E+08	2E+08	7E+08	4E+08	2E+08	2E+08	2E+08	6E+08	4E+08	2E+08	2E+08	2E+08	6E+08	3E+08	2E+08	2E+08	2E+08	6E+08	2E+08	2E+08	2E+08	5E+08	2E+08	2E+08	1E+08	2E+08	1E+08	2E+08	1E+08	1E+08	1E+08	4E+08	2E+08	2E+08	2E+08	2E+08	2E+08	1E+08	2E+08	2E+08	2E+08	3E+08	2E+08	2E+08	2E+08	2E+08	2E+08	p19819	Protein capicua homolog
TNFRSF10D seq: 331 - 359 + Phospho (ST)	38324	37149	39656	13654	33854	45779	25552	34892	34104	26736	56234	53206	39754	40879	18798	40867	33072	34984	19162	28832	39759	50401	21442	19024	42741	9E+07	44396	34144	9E+07	29103	45838	31655	19257	8E+07	17227	1E+08	27262	25062	7E+07	5E+07	6E+07	7E+07	5E+07	1E+08	13188	134692	41467	25947	5E+07	3E+07	p11252	Tumor necrosis factor receptor superfamily member 10D
FAM83H seq: 508 - 517 + 2 Phospho (ST)	2E+08	1E+08	1E+08	1E+08	1E+08	45779	1E+08	1E+08	1E+08	1E+08	56234	1E+08	2E+08	8E+07	1E+08	40867	2E+08	2E+08	2E+08	2E+08	39759	1E+08	2E+08	2E+08	68554	2E+07	4E+07	3E+07	3E+07	45838	4E+07	19257	3E+07	17227	44771	3E+07	3E+07	2E+07	3E+07	100327	64618	2E+07	4E+07	4E+07	134692	1E+08	6E+07	2E+07	25732		p12017	Protein FAM83H
SH3KBP1 pS410	7E+06	37149	39656	4E+06	3E+07	45779	25552	34892	9E+06	3E+06	3E+08	53206	3E+07	40879	5E+06	2E+07	33072	34984	2E+07	956234	6E+06	4E+07	1E+07	3E+07	1E+06	2E+09	2E+09	1E+09	1E+09	1E+09	1E+09	1E+09	2E+09	1E+09	1E+09	1E+09	9E+08	1E+09	1E+09	2E+09	1E+09	1E+09	1E+09	1E+09	1E+09	1E+09	1E+09	1E+09	1E+09	1E+09	p02465	SH3 domain-containing kinase-binding protein 1
PRKAR2A pS99	5E+08	9E+08	7E+08	7E+08	5E+08	3E+09	1E+09	6E+08	6E+08	6E+08	2E+09	9E+08	6E+08	5E+08	5E+08	1E+09	8E+08	9E+08	9E+08	8E+08	1E+09	9E+08	9E+08	7E+08	9E+08	1E+09	5E+08	7E+08	6E+08	7E+08	6E+08	2E+08	6E+08	7E+08	7E+08	4E+08	3E+08	3E+08	5E+08	1E+09	1E+09	4E+08	6E+08	6E+08	1E+09	7E+08	4E+08	6E+08	6E+08		p06424	cAMP-dependent protein kinase type II-alpha regulatory subunit
MYO9B seq: 712 - 729 + Phospho (ST)	7E+07	3E+07	5E+07	3E+07	3E+07	2E+08	2E+08	5E+07	5E+07	2E+07	3E+08	1E+08	1E+08	6E+07	5E+07	9E+07	5E+07	9E+07	8E+07	4E+07	1E+08	4E+07	8E+07	6E+07	7E+07	3E+07	6E+06	1E+07	9E+06	6E+06	1E+07	31655	19257	24890	17227	5E+07	27262	1E+07	4E+07	27887	4E+06	4E+07	1E+07	7E+06	41974	1E+07	2E+07	8E+06	30272	25732	p21140	Unconventional myosin-IXb
PACSIN3 pS383	4E+07	1E+08	7E+07	3E+07	1E+08	2E+08	2E+08	7E+07	1E+08	2E+08	1E+08	1E+08	6E+07	2E+08	1E+08	9E+07	1E+08	4E+07	1E+08	1E+08	1E+08	21442	2E+08	1E+08	68554	8E+07	6E+07	3E+08	3E+08	45838	9E+07	5E+07	2E+08	3E+08	7E+07	5E+07	4E+07	2E+08	1E+08	6E+07	7E+07	3E+07	2E+08	13188	1E+08	1E+08	9E+07	3E+08	25732		p20110	Protein kinase C and casein kinase substrate in neurons protein 3
GTF2I seq: 828 - 858 + Phospho (ST)	5E+07	7E+07	39656	6E+07	5E+07	45779	7E+07	34892	1E+08	1E+08	56234	3E+07	2E+07	5E+07	9E+07	40867	1E+08	8E+07	9E+07	1E+08	39759	4E+07	9E+07	9E+07	9E+07	5E+07	44396	8E+07	5E+07	4E+07	45838	31655	19257	24890	6E+07	5E+07	8E+07	7E+07	2E+07	36936	5E+07	3E+07	7E+07	41974	6E+07	134692	4E+07	8E+07	4E+07	4E+07	p13894	General transcription factor II-I
FNBP1L seq: 486 - 499 + Phospho (ST)	3E+08	2E+08	2E+08	7E+07	33854	3E+09	8E+08	2E+08	7E+06	26736	3E+09	1E+09	3E+08	2E+08	18798	4E+08	3E+07	2E+08	19162	28832	6E+08	2E+07	5E+07	19024	42741	2E+09	2E+07	4E+06	19500	29103	6E+08	6E+07	1E+07	24890	17227	2E+09	9E+07	3E+07	27887	36936	1E+09	1E+08	6E+07	41974	13188	1E+09	8E+06	1E+07	30272	25732	p07292	Formin-binding protein 1-like
IRF2BPL seq: 655 - 670 + 2 Phospho (ST)	4E+08	4E+08	5E+08	5E+08	3E+08	45779	6E+08	6E+08	4E+08	4E+08	1E+09	6E+08	7E+08	5E+08	6E+08	5E+08	3E+08	3E+08	3E+08	4E+08	7E+08	5E+08	5E+08	4E+08	1E+09	5E+08	3E+08	3E+08	2E+08	6E+08	4E+08	3E+08	4E+08	4E+08	8E+08	6E+08	3E+08	3E+08	4E+08	6E+08	3E+08	2E+08	3E+08	1E+08	8E+08	6E+08	3E+08	4E+08	3E+08		p07010	Interferon regulatory factor 2-binding protein-like
BBX seq: 838 - 855 + Phospho (ST)	2E+08	3E+08	3E+08	2E+08	2E+08	9E+08	5E+08	3E+08	2E+08	2E+08	8E+08	4E+08	2E+08	3E+08	2E+08	4E+08	3E+08	3E+08	3E+08	3E+08	5E+08	3E+08	3E+08	2E+08	4E+08	2E+09	4E+08	3E+08	3E+08	6E+08	4E+08	4E+08	3E+08	2E+08	4E+08	3E+08	1E+09	7E+08	3E+08	3E+08	5E+08	3E+08	2E+08	1E+09	5E+08	3E+08	4E+08	7E+08	5E+08	3E+08	p03099	HMG box transcription factor BBX
LUZP1 seq: 992 - 1005 + Phospho (ST)	8E+06	37149	39656	1E+07	33854	1E+07	25552	34892	5E+06	26736	2E+08	53206	39754	5E+07	2E+07	2E+07	33072	34984	3E+06	1E+07	2E+07	50401	3E+06	19024	1E+07	1E+09	3E+08	3E+08	4E+08	3E+08	2E+08	1E+08	1E+08	3E+08	3E+08	4E+08	2E+08	2E+08	2E+08	3E+08	4E+08	2E+08	2E+08	2E+08	2E+08	3E+08	2E+08	3E+08	3E+08		p06496	Leucine zipper protein 1
FGD3 pS56	2E+08	1E+08	2E+08	1E+08	2E+08	5E+08	2E+08	1E+08	1E+08	9E+07	8E+08	3E+08	4E+08	3E+08	2E+08	2E+08	8E+07	2E+08	1E+08	6E+07	2E+08	9E+07	2E+08	1E+08	68554	44396	34144	19500	29103	45838	31655	19257	24890	17227	44771	27262	25062	27887	36936	100327	64618	38440	41974	13188	134692	41467	25947	30272	25732		p20537	FYVE, RhoGEF and PH domain-containing protein 3
HSPBP1 pS354	1E+08	1E+08	2E+08	1E+08	1E+08	3E+08	1E+08	2E+08	1E+08	1E+08	6E+08	4E+08	3E+08	2E+08	3E+08	2E+08	8E+07	2E+08	2E+08	7E+07	2E+08	4E+07	2E+08	1E+08	2E+08	8E+07	2E+08	3E+08	1E+08	1E+08	31655	8E+07	2E+08	2E+08	2E+08	2E+08	2E+08	2E+08	2E+08	2E+08	1E+08	2E+08	2E+08	1E+08	1E+08	1E+08	3E+08	2E+08	2E+08		p11301	Hsp70-binding protein 1
PHKA2 pS1015	3E+08	4E+08	6E+08	6E+08	7E+08	2E+09	1E+09	7E+08	9E+08	8E+08	1E+09	9E+08	9E+08	7E+08	1E+09	6E+08	4E+08	8E+08	7E+08	6E+08	5E+08	3E+08	7E+08	9E+08	9E+08	4E+07	44396	5E+07	6E+07	7E+07	45838	31655	1E+07	8E+07	7E+07	44771	2E+07	25062	5E+07	7E+07	9E+07	64618	4E+07	6E+07	4E+07	134692	41467	25947	6E+07	5E+07	p07443	Phosphorylase b kinase regulatory subunit alpha, liver isoform
SREK1IP1 seq: 143 - 154 + Phospho (ST)	1E+07	4E+07	2E+07	4E+06	1E+07	9E+07	5E+07	2E+07	34104	26736	7E+07	4E+07	2E+07	40879	2E+07	2E+07	3E+07	2E+07	2E+07	28832	1E+07	50401	1E+07	19024	3E+07	5E+07	6E+06	4E+07	3E+07	1E+07	3E+07	3E+06	19257	3E+07	3E+07	4E+07	3E+07	2E+07	1E+07	1E+07	4E+07	3E+07	2E+07	2E+07	2E+07	4E+07	3E+07	5E+07	30272	2E+07	p14602	Protein SREK1IP1
TNKS1BP1 pS178	7E+07	1E+08	1E+08	1E+08	1E+08	4E+08	1E+08	1E+08	1E+08	8E+07	6E+08	2E+08	1E+08	1E+08	2E+08	1E+08	2E+08	8E+07	2E+08	8E+07	2E+08	2E+08	1E+08	2E+08	5E+08	2E+08	1E+08	3E+08	2E+08	2E+08	1E+08	5E+07	2E+08	2E+08	2E+08	4E+08	2E+08	2E+08	2E+08	2E+08	3E+08	2E+08	3E+08	2E+08	2E+08	3E+08	2E+08	2E+08	2E+08	2E+08	p16975	182 kDa tankyrase-1-binding protein
ARFGAP2 seq: 337 - 357 + Phospho (ST)	3E+07	7E+07	1E+08	2E+08	1E+08	1E+08	9E+07	2E+08	2E+08	3E+08	1E+08	5E+07	39754	2E+08	18798	9E+07	2E+07	2E+08	2E+08	2E+08	7E+07	5E+07	2E+08	2E+08	1E+08	4E+07	2E+08	19500	2E+08	4E+07	4E+07	2E+08	2E+08	5E+08	6E+07	5E+07	25062	27887	36936	9E+07	8E+07	4E+08	3E+08	13188	7E+07	5E+07	3E+08	30272	4E+08		p03737	ADP-ribosylation factor GTPase-activating protein 2
SUB1 pS118	38324	3E+07	8E+07	1E+08	5E+08	3E+09	5E+08	1E+08	2E+08	2E+07	1E+09	1E+09	2E+08	4E+08	5E+07	4E+08	33072	2E+08	4E+08	8E+07	39759	50401	5E+07	3E+08	3E+08	1E+08	44396	34144	6E+07	1E+08	45838	31655	19257	7E+07	2E+07	6E+07	27262	25062	27887	1E+08	5E+07	2E+07	7E+06	4E+07	3E+07	134692	41467	6E+07	2E+08	7E+07	p15847	Activated RNA polymerase II transcriptional coactivator p15
LRRFIP1 seq: 763 - 789 + Phospho (ST)	3E+08	2E+08	3E+08	3E+08	2E+08	1E+09	5E+08	3E+08	4E+08	2E+08	7E+08	4E+08	5E+08	4E+08	3E+08	3E+08	4E+08	5E+08	4E+08	3E+08	4E+08	4E+08	2E+08	3E+08	68554	44396	4E+07	4E+07	29103	7E+07	31655	19257	24890	6E+07	44771	27262	4E+07	27887	36936	1E+08	5E+07	38440	41974	13188	134692	41467	25947	30272	3E+07		p20301	Leucine-rich repeat flightless-interacting protein 1
SPEN seq: 1057 - 1087 + Phospho (ST)	4E+07	1E+08	2E+08	1E+08	2E+08	6E+08	5E+08	2E+08	2E+08	2E+08	5E+08	4E+08	2E+08	3E+08	2E+08	1E+08	2E+08	2E+08	2E+08	2E+08	1E+08	2E+08	2E+08	2E+08	5E+08	8E+07	7E+07	1E+08	1E+08	1E+08	6E+07	3E+07	1E+08	1E+08	1E+08	3E+08	1E+08	8E+07	1E+08	1E+08	1E+08	3E+08	1E+08	1E+08	2E+08	6E+07	8E+07	1E+08	1E+08		p11151	Msx2-interacting protein
WIZ seq: 1139 - 1161 + Phospho (ST)	2E+08	7E+08	6E+08	4E+08	5E+08	2E+09	1E+09	6E+08	5E+08	6E+08	2E+09	1E+09	7E+08	1E+09	8E+08	9E+08	6E+08	5E+08	5E+08	5E+08	8E+08	5E+08	5E+08	5E+08	2E+09	5E+08	3E+08	4E+08	5E+08	5E+08	4E+08	2E+08	3E+08	4E+08	3E+08	8E+08	5E+08	3E+08	3E+08	4E+08	9E+08	6E+08	3E+08	4E+08	6E+08	6E+08	4E+08	2E+08	4E+08	3E+08	p12945	Protein Wiz
SRRM2 pS351	2E+08	2E+08	3E+08	2E+08	1E+08	6E+08	3E+08	3E+08	3E+08	2E+08	5E+08	3E+08	3E+08	2E+08	2E+08	4E+08	3E+08	4E+08	2E+08	1E+08	4E+08	3E+08	3E+08	1E+08	2E+08	5E+08	3E+08	2E+08	2E+08	3E+08	2E+08	7E+07	1E+08	1E+08	5E+08	2E+08	2E+08	1E+08	3E+08	5E+08	4E+08	1E+08	3E+08	3E+08	3E+08	2E+08	2E+08	2E+08	2E+08		p06190	Serine/arginine repetitive matrix protein 2
RACGAP1 pS628	6E+07	8E+07	1E+08	8E+07	6E+07	4E+08	2E+08	1E+08	7E+07	6E+07	2E+08	1E+08	5E+07	5E+07	5E+07	2E+08	1E+08	1E+08	1E+08	9E+07	2E+08	6E+07	1E+08	7E+07	8E+07	2E+08	7E+07	1E+08	1E+08	5E+07	1E+08	6E+07	3E+07	6E+07	7E+07	2E+08	7E+07	9E+07	7E+07	6E+07	1E+08	7E+07	1E+08	9E+07	1E+08	1E+08	6E+07	1E+08	9E+07	8E+07	p08634	Rac GTPase-activating protein 1
C9orf142 pS148	2E+09	3E+09	2E+09	2E+09	2E+09	1E+10	3E+09	2E+09	2E+09	2E+09	1E+10	4E+09	3E+09	2E+09	2E+09	4E+09	3E+09	2E+09	3E+09	4E+09	3E+09	2E+09	2E+09	2E+09	1E+09	2E+09	1E+09	2E+09	6E+08	7E+08	2E+09	1E+09	2E+09	9E+08	8E+08	2E+09	3E+09	2E+09	1E+09	7E+08	1E+09	1E+09	2E+09	9E+08	7E+08	2E+09	1E+09				p12324	Protein PAXX
SH3GL1 pS288	9E+08	8E+08	8E+08	8E+08	7E+08	3E+09	1E+09	9E+08	9E+08	7E+08	4E+09	2E+09	1E+09	1E+09	1E+09	1E+09	8E+08	1E+09	7E+08	6E+08	2E+09	1E+09	1E+09	7E+08	8E+08	3E+09	2E+09	1E+09	1E+09	1E+09	1E+09	2E+09	9E+08	2E+09	1E+09	2E+09	1E+09	8E+08	1E+09	9E+08	2E+09	1E+09	1E+09	1E+09	2E+09	2E+09	2E+09	1E+09	1E+09		p14538	Endophilin-A2
DLGAP5 seq: 652 - 672 + Phospho (ST)	38324	37149	39656	13654	33854	45779	25552	34892	34104	26736	56234	53206	39754	40879	18798	40867	33072	34984	19162	28832	39759	50401	21442	19024	42741	1E+08	2E+07	2E+07	3E+07	2E+07	45838	3E+07	19257	1E+07	4E+07	5E+07	5E+07	2E+07	3E+07	7E+07	4E+07	38440	4E+07	6E+07	4E+07	41467	3E+07	30272	3E+07		p09674	Disks large-associated protein 5
C17orf59 pS168	4E+07	5E+07	39656	1E+08	33854	45779	1E+08	8E+07	1E+08	2E+08	1E+08	6E+07	7E+07	40879	9E+07	1E+08	8E+07	1E+08	1E+08	1E+08	7E+07	6E+07	1E+08	1E+08	8E+07	3E+07	5E+07	4E+07	4E+07	45838	2E+07	3E+07	4E+07	2E+07	27262	2E+07	2E+07	36936	6E+07	4E+07	4E+07	41974	3E+07	5E+07	41467	4E+07	2E+07	2E+07			p05301	Uncharacterized protein C17orf59
ASAP1 pS1027	8E+07	6E+07	8E+07	5E+07	3E+07	1E+08	8E+07	6E+07	5E+07	3E+07	4E+08	2E+08	1E+08	1E+08	8E+07	8E+07	5E+07	7E+07	4E+07	3E+07	1E+08	6E+07	6E+07	5E+07	4E+07	5E+08	4E+08	2E+08	3E+08	4E+08	3E+08	2E+08	3E+08	3E+08	5E+08	4E+08	3E+08	3E+08	3E+08	5E+08	4E+08	4E+08	2E+08	3E+08	2E+08	3E+08	4E+08	3E+08	2E+08	3E+08	p05587	Arf-GAP with SH3 domain, ANK repeat and PH domain-containing protein 1
TFRC seq: 40 - 53 no mod	3E+08	2E+08	3E+08	3E+08	2E+08	6E+08	5E+08	1E+08	2E+08	2E+08	1E+09	6E+08	6E+08	6E+08	4E+08	2E+08	2E+08	2E+08	1E+08	4E+08	2E+08	2E+08	2E+08	2E+08	6E+08	9E+07	1E+08	2E+08	1E+08	3E+08	8E+07	1E+08	2E+08	1E+08	5E+08	3E+08	1E+08	9E+07	1E+08	4E+08	2E+08	2E+08	1E+08	3E+08	2E+08	1E+08	1E+08	9E+07			p12145	Transferrin receptor protein 1
SMARCA5 seq: 98 - 119 + 2 Phospho (ST)	5E+07	2E+08	2E+08	2E+08	2E+08	4E+08	2E+08	2E+08	2E+08	2E+08	3E+08	3E+08	2E+08	2E+08	2E+08	2E+08	2E+08	2E+08	2E+08	9E+07	1E+08	2E+08	2E+08	1E+08	4E+08	1E+08	1E+08	1E+08	1E+08	1E+08	7E+07	1E+08	1E+08	1E+08	1E+08	2E+08	2E+08	1E+08	2E+08	2E+08	2E+08	2E+08	1E+08	2E+08	2E+08	1E+08	2E+08	1E+08	1E+08		p07809	SWI/SNF-related matrix-associated actin-dependent regulator of chromatin subfamily A member 5
MAP2K2 pS23	3E+08	3E+08	7E+08	8E+08	5E+08	2E+09	8E+08	6E+08	8E+08	9E+07	2E+10	3E+09	2E+09	4E+09	7E+07	8E+08	2E+08	7E+08	1E+09	28832	6E+08	3E+08	4E+08	1E+09	42741	5E+08	5E+07	6E+07	5E+08	29103	2E+08	5E+07	4E+06	2E+08	17227	5E+08	1E+08	2E+08	27887	36936	2E+08	1E+08	2E+08	3E+07	13188	2E+08	4E+07	3E+08	30272	25732	p07216	Dual specificity mitogen-activated protein kinase kinase 2
EIF2AK3 pS715	38324	7E+07	9E+07	3E+07	3E+07	2E+08	1E+08	8E+07	3E+07	6E+07	3E+08	2E+08	2E+08	2E+08	1E+08	2E+08	2E+08	5E+07	7E+07	5E+07	5E+07	1E+08	4E+07	7E+07	5E+07	6E+07	1E+08	1E+07	2E+07	19500	2E+07	5E+07	8E+06	8E+06	2E+07	17227	7E+07	4E+06	2E+07	7E+06	2E+07	6E+07	4E+07	4E+06	1E+07	5E+06	134692	6E+06	2E+07	2E+07	p17651	Eukaryotic translation initiation factor 2-alpha kinase 3
CGNL1 seq: 312 - 328 no mod	3E+07	3E+07	39656	13654	33854	3E+08	5E+07	34892	34104	26736	3E+08	5E+07	39754	40879	7E+07	5E+07	1E+07	34984	19162	28832	3E+07	1E+07	21442	19024	42741	2E+08	2E+07	34144	19500	29103	4E+07	1E+07	19257	24890	2E+07	1E+08	27262	25062	27887	3E+07	1E+08	9E+07	38440	41974	13188	9E+07	41467	25947	30272	2E+07	p16608	Cingulin-like protein 1
SIPA1L1 seq: 1148 - 1167 + Phospho (ST)	2E+07	37149	2E+07	2E+07	1E+07	6E+07	25552	2E+07	3E+07	2E+07	2E+08	53206	2E+07	2E+07	2E+08	33072	34984	3E+07	3E+07	9E+07	50401	4E+07	2E+07	4E+07	68554	44396	1E+07	19500	2E+07	45838	31655	7E+06	24890	17227	44771	1E+07	2E+07	27887	36936	6E+07	64618	2E+07	41974	13188	134692	7E+06	2E+07	30272	25732		p09116	Signal-induced proliferation-associated 1-like protein 1
LASP1 pS146	1E+10	1E+10	2E+10	2E+10	2E+10	8E+10	4E+10	3E+10	3E+10	2E+10	5E+10	2E+10	2E+10	1E+10	1E+10	1E+10	2E+10	1E+10	2E+10	1E+10	3E+10	2E+10	2E+10	1E+10	1E+10	3E+09	4E+09	6E+09	3E+09	8E+09	3E+09	3E+09	2E+09	6E+09	4E+09	1E+10	6E+09	5E+09	5E+09	6E+09	7E+09	3E+09	2E+09	3E+09	3E+09	7E+09	4E+09	3E+09	4E+09	4E+09	p10094	LIM and SH3 domain protein 1
WIZ pS1146	3E+08	7E+08	5E+08	4E+08	5E+08	45779	1E+09	5E+08	4E+08	6E+08	2E+09	1E+09	7E+08	1E+09	1E+09	7E+08	6E+08	4E+08	5E+08	5E+08	5E+08	6E+08	4E+08	6E+08	6E+08	2E+09	5E+08	3E+08	5E+08	5E+08	6E+08	5E+08	2E+08	4E+08	4E+08	8E+08	5E+08	3E+08	4E+08	4E+08	1E+09	6E+08	4E+08	5E+08	6E+08	6E+08	3E+08	2E+08	4E+08	4E+08	p12946	Protein Wiz
ASNA1 seq: 261 - 267 no mod	38324	1E+06	3E+06	13654	33854	2E+07	25552	2E+06	34104	26736	1E+08	53206	39754	40879	18798	6E+06	33072	34984	19162	28832	39759	50401	21442	19024	42741	68554	44396	34144	19500	29103	45838	31655	19257	24890	17227	7E+06	27262	25062	27887	36936	100327	64618	38440	41974	13188	134692	41467	25947	30272	25732	p11564	ATPase ASNA1
SURF6 seq: 288 - 294 no mod	38324	1E+06	3E+06	13654	33854	2E+07	25552	2E+06	34104	26736	1E+08	53206	39754	40879	18798	6E+06	33072	34984	19162	28832	39759	50401	21442	19024	42741	68554	44396	34144	19500	29103	45838	31655	19257	24890	17227	7E+06	27262	25062	27887	36936	100327	64618	38440	41974	13188	134692	41467	25947	30272	25732	p11314	Surfeit locus protein 6
TPR pT2116	38324	5E+07	39656	13654	33854	1E+08	1E+07	8E+06	34104	26736	2E+08	3E+07	39754	1E+07	5E+06	40867	1E+07	34984	1E+07	28832	39759	50401	21442	6E+06	42741	4E+08	6E+07	2E+08	2E+08	1E+08	2E+08	4E+07	6E+07	2E+08	2E+08	4E+08	1E+08	3E+08	2E+08	3E+08	5E+08	2E+08	2E+08	2E+08	2E+08	4E+08	2E+08	3E+08	2E+08	2E+08	p15664	Nucleoprotein TPR
RPL17 seq: 106 - 121 no mod	38324	5E+07	1E+08	9E+07	2E+08	2E+09	1E+08	8E+07	1E+08	1E+08	7E+08	1E+08	1E+08	2E+08	1E+08	2E+08	9E+07	1E+08	1E+07	9E+07	2E+08	5E+07	7E+07	3E+07	7E+07	2E+08	1E+08	5E+08	3E+07	9E+07	3E+08	3E+08	3E+08	8E+06	3E+06	4E+08	3E+08	3E+08	6E+07	2E+08	3E+08	2E+08	4E+08	4E+08	3E+08	2E+07	2E+08	4E+08	4E+08		p15766	60S ribosomal protein L17
AHNAK pS93	6E+08	8E+08	9E+08	1E+09	1E+09	2E+09	2E+09	9E+08	1E+09	1E+09	5E+09	2E+09	1E+09	2E+09	1E+09	1E+09	5E+08	1E+09	2E+09	1E+09	2E+09	6E+08	5E+08	2E+09	1E+09	9E+09	9E+08	1E+09	2E+09	3E+09	2E+09	1E+09	7E+08	3E+09	2E+09	7E+09	2E+09	2E+09	1E+09	2E+09	4E+09	2E+09	9E+08	2E+09	2E+09	3E+09	8E+08	1E+09	3E+09	3E+09	p13211	Neuroblast differentiation-associated protein AHNAK
SVIL pS221	3E+08	4E+08	5E+08	3E+08	2E+08	1E+09	7E+08	5E+08	3E+08	3E+08	1E+09	6E+08	6E+08	3E+08	3E+08	7E+08	3E+08	5E+08	4E+08	2E+08	7E+08	4E+08	4E+08	3E+08	3E+08	7E+08	5E+08	8E+08	4E+08	1E+09	6E+08	3E+08	5E+08	7E+08	2E+09	7E+08	6E+08	5E+08	5E+08	2E+09	8E+08	7E+08	6E+08	8E+08	1E+09	5E+08	6E+08	6E+08	5E+08		p08854	Supervillin
SRRM2 seq: 2275 - 2301 + Phospho (ST)	2E+08	2E+08	2E+08	2E+08	2E+08	8E+08	4E+08	2E+08	2E+08	2E+08	1E+09	5E+08	3E+08	3E+08	2E+08	5E+08	3E+08	3E+08	2E+08	4E+08	2E+08	2E+08	3E+08	2E+08	9E+08	4E+08	3E+08	3E+08	3E+08	2E+08	1E+08	3E+08	3E+08	7E+08	4E+08	2E+08	3E+08	3E+08	7E+08	4E+08	3E+08	3E+08	4E+08	5E+08	2E+08	2E+08	3E+08	3E+08			p03001	Serine/arginine repetitive matrix protein 2
FKBP15 seq: 1082 - 1124 + 3 Phospho (ST)	1E+08	1E+08	1E+08	13654	33854	45779	2E+08	1E+08	34104	7E+07	56234	3E+08	4E+08	40879	2E+08	40867	2E+08	1E+08	19162	28832	39759	2E+08	9E+07	19024	42741	68554	9E+08	9E+08	19500	29103	4E+08	1E+09	2E+08	6E+08	17227	7E+08	1E+09	7E+08	27887	36936	8E+08	9E+08	38440	5E+08	13188	9E+08	1E+09	25947	30272	1E+08	p17019	FK506-binding protein 15
PPP1R12C seq: 558 - 569 + Phospho (ST)	1E+07	1E+09	5E+09	5E+08	1E+08	2E+07	7E+07	4E+09	5E+09	4E+09	1E+07	5E+07	39754	6E+09	4E+09	4E+07	2E+09	5E+09	6E+09	2E+09	4E+07	4E+07	2E+09	3E+09	8E+07	5E+09	4E+09	4E+09	6E+09	4E+09	45838	6E+07	3E+09	5E+09	5E+07	2E+07	6E+09	4E+09	5E+09	2E+07	3E+07	6E+09	4E+09	1E+08	4E+09	5E+07	6E+07	3E+09	6E+07	2E+09	p06556	Protein phosphatase 1 regulatory subunit 12C
LARP4 pS583	2E+08	2E+08	3E+08	3E+08	3E+08	45779	5E+08	3E+08	4E+08	2E+08	4E+08	5E+08	5E+08	4E+08	4E+08	2E+08	2E+08	4E+08	3E+08	2E+08	39759	2E+08	3E+08	3E+08	1E+09	2E+08	3E+08	4E+08	3E+08	3E+08	3E+08	2E+08	4E+08	4E+08	7E+08	4E+08	3E+08	2E+08	3E+08	4E+08	8E+08	4E+08	3E+08	3E+08	4E+08	6E+08	2E+08	3E+08	3E+08		p18600	La-related protein 4
seq: 109 - 139 + Gln->pyro-Glu (N-term Q); Phospho (ST)	2E+08	2E+08	4E+08	3E+08	4E+08	3E+08	3E+08	3E+08	3E+08	3E+08	1E+09	6E+08	5E+08	7E+08	6E+08	6E+08	2E+08	3E+08	3E+08	2E+08	6E+08	2E+08	3E+08	3E+08	3E+08	5E+07	34144	1E+08	29103	5E+07	5E+07	1E+07	24890	8E+07	9E+07	8E+07	6E+07	7E+07	36936	1E+08	64618	7E+07	5E+07	1E+08	5E+07	41467	7E+07	1E+08	1E+08		p07964	Protein DDI1 homolog 2
RB1 seq: 359 - 376 + Phospho (ST)	1E+08	1E+08	1E+08	1E+08	9E+07	1E+09	3E+08	1E+08	6E+07	1E+09	3E+08	1E+08	5E+07	5E+07	4E+08	1E+08	1E+08	1E+08	7E+07	3E+08	7E+07	1E+08	9E+07	9E+07	2E+09	5E+08	6E+08	7E+08	5E+08	7E+08	3E+08	2E+08	6E+08	6E+08	2E+09	6E+08	4E+08	5E+08	6E+08	1E+09	5E+08	5E+08	4E+08	5E+08	9E+08	4E+08	4E+08	4E+08	4E+08		p12568	Retinoblastoma-associated protein
ADNP seq: 731 - 757 + Phospho (ST)	2E+07	5E+07	1E+08	7E+07	1E+08	4E+08	2E+08	8E+07	1E+08	1E+08	3E+08	2E+08	1E+08	1E+08	1E+08	9E+07	3E+07	1E+08	2E+08	1E+08	6E+07	4E+07	7E+07	2E+08	1E+08	1E+08	44396	3E+07	6E+07	2E+07	3E+07	4E+06	19257	4E+07	6E+07	5E+07	2E+07	3E+07	3E+07	2E+07	7E+07	5E+07	3E+07	3E+07	5E+07	4E+07	1E+07	4E+07	5E+07	2E+07	p13019	Activity-dependent neuroprotector homeobox protein
CPSF6 seq: 401 - 420 + Phospho (ST)	4E+07	3E+07	4E+07	2E+07	33854	2E+08	2E+08	8E+07	3E+07	2E+07	1E+07	2E+08	7E+07	5E+07	4E+07	4E+07	5E+07	4E+07	3E+07	4E+07	2E+07	2E+07	3E+07	4E+07	6E+07	4E+07	2E+07	4E+07	6E+07	29103	2E+07	31655	19257	4E+07	3E+07	8E+07	2E+07	2E+07	2E+07	36936	2E+07	1E+08	64618	3E+07	3E+07	2E+07	8E+07	4E+07	6E+07	3E+07	p17367	Cleavage and polyadenylation specificity factor subunit 6
TMPO pS66 pS67 pT74	1E+08	37149	2E+09	8E+08	1E+09	45779	1E+09	1E+09	1E+09	2E+09	56234	8E+08	1E+09	1E+09	2E+09	40867	6E+08	8E+08	1E+09	7E+08	39759	8E+08	1E+09	1E+09	2E+09	68554	3E+08	6E+08	7E+08	6E+08	45838	4E+08	6E+08	5E+08	9E+08	44771	5E+08	6E+08	4E+08	7E+08	100327	3E+08	4E+08	4E+08	134692	5E+08	9E+08	7E+08	6E+08		p15477	Lamina-associated polypeptide 2, isoform alpha
THRAP3 pT874	2E+08	2E+08	1E+08	1E+08	9E+08	3E+08	2E+08	2E+08	1E+08	5E+08	2E+08	2E+08	1E+08	2E+08	4E+08	2E+08	2E+08	2E+08	2E+08	3E+08	2E+08	3E+08	2E+08	1E+09	3E+08	4E+08	4E+08	5E+08	4E+08	4E+08	3E+08	3E+08	3E+08	5E+08	5E+08	7E+08	3E+08	3E+08	3E+08	5E+08	9E+08	4E+08	5E+08	3E+08	5E+08	6E+08	3E+08	4E+08	3E+08	4E+08	p09198	Thyroid hormone receptor-associated protein 3
PALLD pS641	3E+07	2E+08	3E+08	4E+08	6E+08	45779	3E+08	2E+08	4E+08	3E+08	56234	1E+09	7E+08	2E+09	1E+09	3E+08	8E+07	2E+08	4E+08	3E+08	3E+08	1E+08	3E+08	6E+08	6E+08	5E+09	1E+09	1E+09	2E+09	2E+09	2E+09	5E+08	4E+08	2E+09	2E+09	3E+09	1E+09	1E+09	2E+09	2E+09	3E+09	1E+09	1E+09	1E+09	2E+09	2E+09	6E+08	2E+09	2E+09	1E+09	p05100	Palladin
MYL12B pT134	2E+07	6E+07	1E+08	2E+07	2E+07	2E+08	1E+08	1E+08	34104	2E+07	2E+08	1E+08	1E+08	5E+07	7E+07	5E+07	2E+07	34984	2E+07	2E+07	4E+07	4E+07	4E+07	3E+07	42741	6E+08	7E+07	1E+08	3E+08	1E+08	1E+08	4E+07	7E+06	2E+08	2E+08	2E+08	6E+07	7E+07	1E+08	1E+08	2E+08	1E+08	9E+07	1E+08	2E+08	3E+07	1E+08	2E+08	2E+08		p17449	Myosin regulatory light chain 12B
PPIG pS356 pT358	7E+08	2E+08	4E+08	3E+08	2E+08	2E+08	7E+09	1E+09	2E+08	4E+08	8E+07	5E+09	2E+09	7E+08	5E+08	2E+07	1E+09	9E+07	5E+08	6E+08	4E+07	1E+09	5E+07	3E+07	3E+08	2E+07	2E+09	5E+07	2E+07	3E+08	2E+08	9E+08	1E+08	3E+06	1E+09	2E+08	2E+09	1E+08	3E+07	2E+07	1E+07	1E+09	2E+08	6E+07	2E+07	1E+06	2E+09	2E+07	2E+07	2E+07	p16501	Peptidyl-prolyl cis-trans isomerase G
SPTAN1 pS1217	1E+08	1E+08	2E+08	2E+08	2E+08	9E+08	3E+08	2E+08	1E+08	8E+07	2E+08	1E+08	4E+08	2E+08	3E+08	2E+08	1E+08	5E+08	1E+08	3E+08	1E+08	2E+08	5E+08	1E+08	1E+08	1E+08	8E+07	1E+08	9E+07	2E+08	1E+08	2E+08	1E+08	1E+08	9E+07	1E+08	2E+08	1E+08	1E+08	2E+08	2E+08	2E+08	1E+08	9E+07	2E+08	3E+08	1E+08	1E+08	1E+08		p05080	Spectrin alpha chain, non-erythrocytic 1
KIAA1191 seq: 168 - 185 + Phospho (ST)	1E+08	2E+08	5E+06	13654	8E+07	4E+08	3E+08	34892	34104	9E+07	7E+08	3E+08	1E+07	40879	1E+08	4E+08	1E+08	1E+07	9E+07	8E+07	5E+08	6E+07	21442	19024	2E+08	9E+08	4E+08	4E+08	3E+08	3E+08	5E+08	2E+08	2E+08	3E+08	8E+08	6E+08	5E+08	3E+08	3E+08	5E+08	4E+08	3E+08	2E+08	2E+08	3E+08	5E+08	2E+08	4E+08	2E+08	2E+08	p08144	Putative monooxygenase p33MONOX
HNRNPUL1 seq: 691 - 731 + Phospho (ST)	2E+09	3E+09	5E+09	4E+09	6E+09	8E+09	5E+09	4E+09	4E+09	5E+09	7E+09	4E+09	4E+09	4E+09	5E+09	5E+09	3E+09	5E+09	5E+09	5E+09	5E+09	2E+09	3E+09	5E+09	6E+09	1E+10	4E+09	5E+09	8E+09	9E+09	7E+09	3E+09	3E+09	9E+09	9E+09	8E+09	5E+09	5E+09	8E+09	1E+10	1E+10	5E+09	5E+09	9E+09	8E+09	7E+09	4E+09	6E+09	8E+09	8E+09	p19946	Heterogeneous nuclear ribonucleoprotein U-like protein 1
NUP214 pS657	2E+08	2E+08	3E+08	2E+08	2E+08	6E+08	3E+08	2E+08	2E+08	1E+08	7E+08	3E+08	2E+08	2E+08	3E+08	2E+08	3E+08	2E+08	2E+08	3E+08	1E+08	2E+08	2E+08	1E+08	1E+08	2E+08	2E+08	9E+07	2E+08	7E+07	6E+07	1E+08	1E+08	4E+08	2E+08	2E+08	2E+08	1E+08	3E+08	1E+08	1E+08	1E+08	2E+08	1E+08	1E+08	1E+08	1E+08	1E+08			p06280	Nuclear pore complex protein Nup214
BCKDHA pS337	3E+09	4E+09	2E+09	2E+09	2E+09	1E+10	8E+09	3E+09	5E+09	2E+09	6E+09	3E+09	3E+09	2E+09	6E+09	4E+09	4E+09	4E+09	4E+09	7E+09	3E+09	3E+09	3E+09	5E+09	2E+09	8E+08	6E+08	1E+09	1E+09	7E+08	5E+08	8E+08	8E+08	1E+09	7E+08	7E+08	7E+08	2E+08	1E+09	2E+09	1E+09	7E+08	6E+08	8E+08	1E+09	6E+08	5E+08	6E+08	5E+08		p14261	2-oxoisovalerate dehydrogenase subunit alpha, mitochondrial
SH3BGRL seq: 87 - 104 no mod	9E+08	2E+09	3E+09	3E+09	4E+09	2E+09	5E+09	3E+09	3E+09	2E+09	6E+09	3E+09	4E+09	3E+09	6E+08	2E+09	3E+09	4E+09	3E+09	3E+08	2E+09	3E+09	4E+09	4E+09	5E+09	2E+09	2E+09	19500	3E+09	2E+09	2E+09	7E+08	3E+09	2E+09	4E+09	3E+09	3E+09	3E+09	2E+09	3E+09	4E+09	3E+09	3E+09	3E+09	2E+09	3E+09	2E+09	2E+09	4E+09	4E+09	p17333	SH3 domain-binding glutamic acid-rich-like protein
PAPOLG seq: 645 - 656 + Phospho (ST)	2E+08	2E+08	2E+08	2E+08	1E+08	5E+08	3E+08	2E+08	2E+08	2E+08	5E+08	2E+08	2E+08	2E+08	2E+08	5E+08	1E+08	3E+08	2E+08	9E+07	5E+08	1E+08	2E+08	1E+08	3E+08	4E+08	2E+08	2E+08	2E+08	3E+08	1E+08	2E+08	2E+08	1E+08	3E+08	2E+08	2E+08	3E+08	8E+07	1E+08	4E+08	3E+08	2E+08	1E+08	2E+08	2E+08	2E+08	1E+08	2E+08		p06673	Poly(A) polymerase gamma
BCKDK seq: 31 - 44 + Phospho (ST)	4E+09	4E+09	5E+09	3E+09	4E+09	5E+09	6E+09	5E+09	5E+09	3E+09	5E+09	7E+09	6E+09	5E+09	4E+09	3E+09	4E+09	6E+09	5E+09	4E+09	5E+09	4E+09	4E+09	5E+09	4E+09	2E+09	1E+09	1E+09	2E+09	1E+09	2E+09	2E+09	8E+08	2E+09	2E+09	2E+09	2E+09	1E+09	1E+09	2E+09	2E+09	2E+09	1E+09	2E+09	2E+09	1E+09	2E+09	1E+09	2E+09	1E+09	p03470	[3-methyl-2-oxobutanoate dehydrogenase [lipoamide]] kinase, mitochondrial
AIFM1 pS268	6E+07	1E+08	1E+08	9E+07	1E+08	2E+08	2E+08	1E+08	9E+07	1E+08	1E+08	2E+08	1E+08	1E+08	2E+08	2E+08	1E+08	1E+08	1E+08	2E+08	1E+08	1E+08	9E+07	2E+08	8E+07	5E+07	5E+07	6E+07	45838	7E+07	5E+07	6E+07	8E+07	2E+08	27262	6E+07	4E+07	6E+07	1E+08	1E+08	5E+07	5E+07	6E+07	134692	8E+07	5E+07	6E+07	7E+07			p05074	Apoptosis-inducing factor 1, mitochondrial
GORASP2 seq: 421 - 452 + 2 Phospho (ST)	4E+08	4E+08	6E+08	8E+08	6E+08	45779	6E+08	5E+08	5E+08	2E+08	4E+08	1E+09	1E+09	6E+08	6E+08	7E+08	4E+08	7E+08	5E+08	3E+08	7E+08	6E+08	3E+08	1E+08	1E+08	2E+08	2E+08	1E+08	2E+08	8E+07	1E+08	1E+08	2E+08	2E+08	1E+08	2E+08	3E+08	2E+08	3E+08	2E+08	2E+08	2E+08	2E+08	5E+07	1E+08	1E+08					p19866	Golgi reassembly-stacking protein 2
TUBB seq: 78 - 103 no mod	2E+09	1E+09	3E+09	2E+09	4E+09	1E+10	5E+09	3E+09	2E+09	2E+09	2E+10	6E+09	5E+09	3E+09	6E+09	4E+09	6E+09	5E+09	2E+09	6E+09	1E+09	2E+09	3E+09	8E+09	6E+09	4E+09	1E+09	2E+09	4E+09	2E+09	2E+09	4E+09	2E+09	4E+09	2E+09	3E+09	6E+09	4E+09	3E+09	1E+09	3E+09	1E+09	2E+09	2E+09	2E+09	2E+09	2E+09	4E+09	5E+09	2E+09	p05711	Tubulin beta chain
TMEM151A pT66	2E+09	2E+09	3E+09	2E+09	2E+09	5E+09	3E+09	2E+09	2E+09	2E+09	5E+09	3E+09	2E+09	2E+09	2E+09	2E+09	3E+09	3E+09	3E+09	3E+09	2E+09	2E+09	2E+09	5E+09	2E+09	3E+09	3E+09	2E+09	2E+09	3E+09	1E+09	3E+09	3E+09	4E+09	3E+09	3E+09	2E+09	3E+09	3E+09	5E+09	4E+09	3E+09	3E+09	3E+09	4E+09	3E+09	2E+09	3E+09	3E+09		p12147	Transmembrane protein 151A
WDR77 seq: 180 - 191 no mod	3E+08	3E+08	2E+08	1E+08	2E+08	1E+09	4E+08	2E+08	2E+08	2E+08	1E+09	4E+08	2E+08	2E+08	2E+08	3E+08	2E+08	3E+08	2E+08	2E+08	4E+08	1E+08	3E+08	2E+08	3E+08	4E+08	1E+08	1E+08	1E+08	1E+08	2E+08	9E+07	8E+07	1E+08	2E+08	2E+08	1E+08	1E+08	1E+08	2E+08	1E+08	1E+08	1E+08	1E+08	2E+08	1E+08	1E+08	1E+08	1E+08		p18201	Methylosome protein 50
SORBS3 seq: 595 - 618 + Phospho (ST)	5E+07	1E+08	7E+07	9E+07	7E+07	45779	1E+08	6E+07	1E+08	3E+07	6E+08	2E+08	1E+08	2E+08	7E+07	2E+08	4E+07	7E+07	1E+08	3E+07	3E+08	6E+07	4E+07	8E+07	4E+07	9E+08	2E+08	2E+08	3E+08	3E+08	6E+08	2E+08	1E+08	3E+08	4E+08	8E+08	2E+08	3E+08	3E+08	4E+08	8E+08	3E+08	3E+08	4E+08	6E+08	2E+08	3E+08	2E+08	3E+08		p15442	Vinexin
TRIP10 pS296	7E+07	6E+07	1E+08	8E+07	7E+07	45779	1E+08	9E+07	2E+08	26736	56234	8E+07	1E+08	9E+07	18798	2E+08	9E+07	1E+08	1E+08	28832	2E+08	8E+07	2E+08	1E+08	42741	1E+09	4E+08	6E+08	7E+08	4E+08	9E+08	4E+08	2E+08	5E+08	5E+08	8E+08	4E+08	4E+08	4E+08	5E+08	9E+08	5E+08	7E+08	5E+08	5E+08	9E+08	4E+08	7E+08	4E+08	4E+08	p19925	Cdc42-interacting protein 4
RAN pS135	1E+08	1E+08	2E+08	1E+08	9E+07	45779	3E+08	2E+08	2E+08	1E+08	56234	2E+08	2E+08	2E+08	1E+08	40867	2E+08	2E+08	2E+08	1E+08	39759	2E+08	2E+08	1E+08	68554	2E+08	3E+08	1E+08	1E+08	2E+08	1E+08	1E+08	1E+08	2E+08	2E+08	2E+08	1E+08	2E+08	1E+08	2E+08	2E+08	1E+08	2E+08	2E+08	1E+08	1E+08	2E+08	1E+08	1E+08		p05444	GTP-binding nuclear protein Ran
SH2D3A seq: 178 - 208 + 2 Phospho (ST)	3E+08	6E+08	6E+08	4E+08	5E+08	3E+08	5E+08	4E+08	5E+08	3E+08	2E+09	1E+09	1E+09	9E+08	8E+08	7E+08	5E+08	6E+08	5E+08	4E+08	4E+08	6E+08	3E+08	6E+08	1E+09	3E+08	4E+08	1E+09	5E+08	4E+08	3E+08	2E+08	9E+08	1E+09	6E+08	3E+08	4E+08	6E+08	8E+08	6E+08	5E+08	5E+08	8E+08	8E+08	5E+08	2E+08	5E+08	1E+09	8E+08		p02117	SH2 domain-containing protein 3A
MYRIP pS280	8E+07	1E+08	8E+07	7E+07	7E+07	45779	1E+08	8E+07	1E+08	9E+07	3E+08	1E+08	7E+07	9E+07	9E+07	1E+08	1E+08	9E+07	1E+08	9E+07	1E+08	9E+07	8E+07	1E+08	68554	44396	34144	19500	29103	45838	31655	19257	24890	17227	44771	27262	25062	27887	36936	100327	64618	38440	41974	13188	134692	41467	25947	30272	25732		p01830	Rab effector MyRIP
LSR pS643 pS646	1E+08	2E+08	3E+08	8E+07	1E+08	6E+08	2E+08	2E+08	8E+07	1E+08	2E+09	1E+08	3E+08	8E+07	7E+07	3E+08	1E+08	2E+08	1E+08	1E+08	2E+08	1E+08	8E+07	1E+08	1E+08	4E+08	9E+07	1E+08	2E+08	8E+07	4E+08	7E+07	6E+07	1E+08	9E+07	7E+08	2E+08	2E+07	6E+07	6E+07	3E+08	1E+08	1E+08	1E+08	1E+08	7E+08	1E+08	2E+08	1E+08	7E+07	p12846	Lipolysis-stimulated lipoprotein receptor
C18orf63 pS293	4E+08	4E+08	5E+08	4E+08	5E+08	2E+09	7E+08	6E+08	5E+08	4E+08	2E+09	1E+09	9E+08	6E+08	7E+08	2E+09	4E+08	8E+08	5E+08	5E+08	2E+09	4E+08	5E+08	4E+08	5E+08	68554	44396	4E+06	2E+07	1E+07	2E+07	31655	19257	24890	1E+07	44771	6E+06	25062	7E+06	9E+06	100327	1E+07	5E+06	41974	1E+07	134692	8E+06	7E+06	30272	25732	p05886	Uncharacterized protein C18orf63
DUSP8 pS401	4E+08	4E+08	5E+08	4E+08	5E+08	2E+09	7E+08	6E+08	5E+08	4E+08	2E+09	1E+09	9E+08	6E+08	7E+08	2E+09	4E+08	8E+08	5E+08	5E+08	2E+09	4E+08	5E+08	4E+08	5E+08	68554	44396	4E+06	2E+07	1E+07	2E+07	31655	19257	24890	1E+07	44771	6E+06	25062	7E+06	9E+06	100327	1E+07	5E+06	41974	1E+07	134692	8E+06	7E+06	30272	25732	p05837	Dual specificity protein phosphatase 8
MKL2 pS543	5E+08	4E+08	6E+08	6E+08	6E+08	2E+09	8E+08	5E+08	6E+08	5E+08	3E+09	1E+09	1E+09	1E+09	9E+08	7E+08	7E+08	6E+08	7E+08	4E+08	8E+08	1E+09	5E+08	9E+08	7E+08	1E+09	8E+08	3E+08	7E+08	3E+08	6E+08	3E+08	4E+08	6E+08	7E+08	6E+08	3E+08	4E+08	6E+08	6E+08	5E+08	3E+08	4E+08	5E+08	5E+08	2E+08	4E+08	6E+08	4E+08		p09849	MKL/myocardin-like protein 2
PNPT1 seq: 46 - 54 no mod	38324	8E+07	6E+07	4E+07	4E+07	2E+08	9E+07	6E+07	5E+07	3E+07	2E+08	1E+08	6E+07	4E+07	4E+07	1E+08	4E+07	5E+07	5E+07	4E+07	1E+08	5E+07	5E+07	4E+07	1E+08	3E+07	1E+07	2E+07	2E+07	5E+07	1E+07	2E+07	2E+07	3E+07	8E+07	2E+07	2E+07	2E+07	6E+07	3E+07	2E+07	2E+07	3E+07	5E+07	41467	3E+07	2E+07	3E+07			p19268	Polyribonucleotide nucleotidyltransferase 1, mitochondrial
AHNAK seq: 5748 - 5767 + 2 Phospho (ST)	5E+07	3E+07	8E+07	1E+08	8E+07	6E+07	7E+07	6E+07	7E+07	9E+07	2E+08	1E+08	2E+08	3E+08	2E+08	40867	33072	6E+07	7E+07	7E+07	4E+07	2E+08	7E+07	1E+08	1E+08	8E+08	5E+08	3E+08	7E+08	5E+08	5E+08	1E+08	5E+08	6E+08	5E+08	5E+08	3E+08	5E+08	8E+08	5E+08	5E+08	2E+08	6E+08	6E+08	4E+08	1E+08	2E+08	6E+08	5E+08		p19581	Neuroblast differentiation-associated protein AHNAK
SGK223 seq: 694 - 705 + Phospho (ST)	3E+07	5E+07	6E+07	3E+07	5E+07	1E+08	9E+07	1E+07	2E+07	2E+08	8E+07	7E+07	40879	2E+07	1E+08	4E+07	1E+08	7E+07	5E+07	1E+08	4E+07	1E+08	4E+07	2E+07	6E+08	2E+08	1E+08	3E+08	2E+08	3E+08	1E+08	2E+08	2E+08	3E+08	1E+08	2E+08	2E+08	2E+08	3E+08	1E+08	3E+08	2E+08	2E+08	2E+08	1E+08	3E+08	2E+08	2E+08			p06267	Tyrosine-protein kinase SgK223
BAIAP2L1 pT257 pM263	1E+07	37149	39656	13654	33854	6E+07	25552	1E+07	2E+07	2E+07	56234	53206	39754	5E+07	6E+07	40867	33072	34984	19162	28832	3E+07	50401	2E+07	19024	3E+07	2E+09	1E+09	1E+09	1E+09	9E+08	1E+09	7E+08	6E+08	1E+09	8E+08	2E+09	1E+09	1E+09	1E+09	1E+09	1E+09	8E+08	6E+08	8E+08	6E+08	1E+09	7E+08	1E+09	7E+08	6E+08	p02417	Brain-specific angiogenesis inhibitor 1-associated protein 2-like protein 1
MAP1B seq: 1963 - 1976 + Phospho (ST)	38324	37149	39656	13654	33854	45779	25552	34892	34104	26736	56234	53206	39754	40879	18798	40867	33072	34984	19162	28832	39759	50401	21442	19024	42741	4E+08	2E+08	3E+08	4E+08	2E+08	3E+08	3E+08	2E+08	3E+08	3E+08	4E+08	3E+08	2E+08	3E+08	5E+08	2E+08	3E+08	3E+08	3E+08	3E+08	3E+08	3E+08	3E+08	2E+08		p02010	Microtubule-associated protein 1B
SNRPA1 seq: 178 - 191 + Phospho (ST)	5E+07	6E+07	5E+07	4E+07	3E+07	7E+08	8E+07	4E+07	5E+07	4E+07	6E+07	4E+07	2E+07	6E+07	3E+07	8E+08	6E+07	7E+07	7E+07	5E+07	1E+08	5E+07	3E+07	5E+07	5E+07	2E+08	1E+08	1E+08	8E+07	7E+07	1E+08	1E+08	1E+08	7E+07	2E+08	1E+08	9E+07	8E+07	9E+07	2E+08	8E+07	2E+08	9E+07	8E+07	2E+08	7E+07	1E+08	9E+07	8E+07		p05341	U2 small nuclear ribonucleoprotein A'
HMGA1 pS99 pS102 pS103	3E+08	3E+08	5E+08	7E+08	5E+08	45779	6E+08	2E+08	4E+08	8E+08	56234	6E+08	4E+08	5E+08	7E+08	40867	5E+08	3E+08	5E+08	8E+08	39759	4E+08	1E+08	3E+08	7E+08	4E+09	1E+10	1E+10	2E+10	3E+10	6E+09	2E+10	8E+09	2E+10	3E+10	5E+09	3E+10	2E+10	1E+10	1E+10	4E+10	4E+09	4E+10	1E+10	2E+10	3E+10	2E+10	1E+10	2E+10	3E+10	p13000	High mobility group protein HMG-I/HMG-Y
SHC3 pS79	4E+07	37149	39656	13654	33854	9E+07	25552	6E+06	2E+07	26736	4E+07	2E+07	3E+07	1E+07	18798	40867	2E+07	34984	19162	28832	4E+07	50401	21442	19024	42741	2E+08	7E+07	2E+08	8E+07	8E+07	2E+08	6E+07	6E+07	1E+08	1E+08	3E+08	27262	1E+08	7E+07	2E+08	4E+08	8E+07	1E+08	7E+07	1E+08	2E+08	1E+08	8E+07	1E+08		p10635	SHC-transforming protein 3
RTKN pS220	4E+07	37149	39656	13654	33854	9E+07	25552	6E+06	2E+07	26736	4E+07	2E+07	3E+07	1E+07	18798	40867	2E+07	34984	19162	28832	4E+07	50401	21442	19024	42741	2E+08	7E+07	2E+08	8E+07	8E+07	2E+08	6E+07	6E+07	1E+08	1E+08	3E+08	27262	1E+08	7E+07	2E+08	4E+08	8E+07	1E+08	7E+07	1E+08	2E+08	1E+08	8E+07	1E+08		p10634	Rhotekin
SGMS2 seq: 44 - 50 + Phospho (ST)	4E+07	37149	39656	13654	33854	9E+07	25552	6E+06	2E+07	26736	4E+07	2E+07	3E+07	1E+07	18798	40867	2E+07	34984	19162	28832	4E+07	50401	21442	19024	42741	2E+08	7E+07	2E+08	8E+07	8E+07	2E+08	6E+07	6E+07	1E+08	1E+08	3E+08	27262	1E+08	7E+07	2E+08	4E+08	8E+07	1E+08	7E+07	1E+08	2E+08	1E+08	8E+07	1E+08		p04994	Phosphatidylcholine:ceramide cholinephosphotransferase 2
RPS19 seq: 122 - 129 no mod	2E+07	3E+07	7E+07	5E+07	1E+08	2E+08	2E+07	34892	9E+07	6E+07	5E+08	4E+07	1E+08	5E+07	18798	2E+08	4E+07	3E+07	1E+08	1E+07	1E+08	5E+07	21442	4E+07	42741	4E+08	9E+07	2E+08	7E+06	3E+08	8E+08	1E+08	3E+07	24890	1E+08	8E+08	1E+08	2E+08	2E+08	9E+07	4E+08	1E+08	9E+07	2E+08	2E+08	1E+09	1E+08	2E+08	2E+08		p12888	40S ribosomal protein S19
PALLD pS641	3E+07	2E+08	3E+08	4E+08	5E+08	45779	3E+08	2E+08	4E+08	3E+08	2E+09	1E+09	1E+09	2E+09	1E+09	4E+08	1E+08	2E+08	4E+08	3E+08	4E+08	2E+08	3E+08	6E+08	6E+08	5E+09	1E+09	2E+09	2E+09	2E+09	5E+08	5E+08	2E+09	2E+09	4E+09	1E+09	1E+09	2E+09	2E+09	3E+09	2E+09	1E+09	2E+09	2E+09	2E+09	7E+08	2E+09	2E+09	2E+09		p05099	Palladin
PPP1R12A pS422	5E+08	5E+08	7E+08	4E+08	3E+08	45779	7E+08	5E+08	4E+08	3E+08	56234	2E+09	1E+09	8E+08	7E+08	7E+08	4E+08	5E+08	3E+08	3E+08	1E+09	4E+08	6E+08	4E+08	3E+08	1E+09	5E+08	5E+08	4E+08	4E+08	1E+09	5E+08	3E+08	5E+08	4E+08	8E+08	3E+08	4E+08	4E+08	1E+09	7E+08	4E+08	3E+08	4E+08	9E+08	6E+08	5E+08	4E+08	4E+08		p16526	Protein phosphatase 1 regulatory subunit 12A
EPPK1 pS2716	1E+10	1E+10	2E+10	1E+10	2E+10	5E+10	2E+10	2E+10	1E+10	1E+10	6E+10	4E+10	3E+10	2E+10	1E+10	1E+10	3E+10	2E+10	1E+10	2E+10	1E+10	2E+10	1E+10	1E+10	8E+08	1E+09	4E+08	6E+08	4E+08	1E+09	5E+08	9E+08	4E+08	7E+08	6E+08	7E+08	6E+08	4E+08	7E+08	5E+08	9E+08	6E+08	5E+08	6E+08	5E+08	7E+08	8E+08	4E+08	7E+08	6E+08	p07698	Epiplakin
C9orf142 pS148	1E+07	4E+07	4E+07	1E+07	2E+07	2E+08	4E+07	4E+07	2E+07	2E+07	3E+08	7E+07	5E+07	2E+07	3E+07	9E+07	6E+07	4E+07	2E+07	2E+07	9E+07	5E+07	4E+07	2E+07	2E+07	68554	1E+07	1E+07	5E+06	6E+06	1E+07	31655	6E+06	24890	2E+06	44771	1E+07	25062	5E+06	7E+06	2E+07	2E+07	5E+06	3E+06	13188	1E+07	1E+07	4E+06	30272	5E+06	p12323	Protein PAXX
TAGLN2 seq: 172 - 182 no mod	4E+07	1E+08	2E+08	1E+08	2E+08	6E+08	2E+08	1E+08	1E+08	2E+07	1E+09	3E+08	1E+08	1E+08	6E+07	7E+07	3E+07	8E+07	1E+08	6E+07	4E+07	5E+07	1E+08	6E+07	68554	1E+08	1E+08	3E+08	3E+08	6E+08	1E+08	2E+08	3E+08	2E+08	1E+09	2E+08	1E+08	3E+08	2E+08	8E+08	4E+08	2E+08	3E+08	2E+08	1E+09	1E+08	1E+08	3E+08	1E+08		p08971	Transgelin-2
FRS2 seq: 325 - 342 + Phospho (ST)	6E+07	1E+08	8E+07	5E+07	6E+07	45779	1E+08	1E+08	1E+08	1E+08	56234	1E+08	1E+08	1E+08	1E+08	3E+07	6E+07	1E+08	8E+07	6E+07	3E+07	7E+07	7E+07	6E+07	68554	44396	34144	19500	29103	45838	31655	19257	24890	17227	44771	27262	25062	27887	36936	100327	64618	38440	41974	13188	134692	41467	25947	30272	25732		p10486	Fibroblast growth factor receptor substrate 2
MAP7D1 seq: 63 - 92 + Phospho (ST)	8E+07	2E+08	2E+08	9E+07	9E+07	3E+08	3E+08	1E+08	9E+07	9E+07	3E+08	4E+08	2E+08	3E+08	2E+08	1E+08	2E+08	2E+08	7E+07	2E+08	1E+08	1E+08	1E+08	2E+08	7E+08	1E+08	1E+08	1E+08	7E+08	1E+08	6E+08	5E+08	8E+08	1E+08	1E+09	8E+08	8E+08	7E+08	8E+08	1E+09	8E+08	8E+08	7E+08	8E+08	1E+09	6E+08	8E+08	7E+08	8E+08		p08337	MAP7 domain-containing protein 1
COMT pM152	2E+08	2E+08	1E+08	2E+07	5E+07	4E+08	1E+08	1E+08	2E+07	3E+08	3E+09	5E+07	1E+08	40879	3E+07	40867	7E+07	34984	6E+07	2E+08	1E+08	7E+07	5E+07	3E+07	42741	7E+07	4E+06	3E+07	19500	5E+07	8E+07	31655	1E+08	3E+07	4E+06	9E+07	1E+09	2E+08	25062	4E+07	6E+07	4E+07	4E+07	9E+07	2E+07	13188	7E+07	3E+07	2E+07	30272	p09834	Catechol O-methyltransferase
TGFB1I1 seq: 170 - 201 + Phospho (ST)	38324	37149	39656	13654	33854	45779	25552	34892	34104	26736	56234	53206	39754	40879	18798	40867	33072	34984	19162	28832	39759	50401	21442	19024	42741	1E+09	3E+08	3E+08	5E+08	6E+08	3E+08	3E+08	5E+08	5E+08	9E+08	4E+08	4E+08	4E+08	1E+09	4E+08	4E+08	4E+08	5E+08	4E+08	8E+08	4E+08	3E+08	5E+08	4E+08		p00943	Transforming growth factor beta-1-induced transcript 1 protein
ABLIM3 pS282	38324	37149	39656	13654	33854	45779	25552	34892	34104	26736	56234	53206	39754	40879	18798	40867	33072	34984	19162	28832	39759	50401	21442	19024	42741	5E+08	3E+08	3E+08	2E+08	4E+08	2E+08	2E+08	3E+08	3E+08	5E+08	3E+08	2E+08	2E+08	3E+08	7E+08	4E+08	2E+08	3E+08	3E+08	4E+08	3E+08	3E+08	2E+08	2E+08		p02185	Actin-binding LIM protein 3
AHNAK seq: 5780 - 5812 + Phospho (ST)	1E+09	2E+09	2E+09	2E+09	3E+09	2E+09	1E+10	2E+09	3E+09	3E+09	2E+09	2E+09	2E+09	2E+09	2E+09	4E+09	3E+09	4E+09	3E+09	4E+09	3E+09	2E+09	2E+09	2E+09	3E+09	2E+09	1E+09	2E+09	2E+09	1E+09	1E+09	2E+09	2E+09	2E+09	1E+09	1E+09	1E+09	2E+09	2E+09	4E+09	2E+09	3E+09	2E+09	3E+09	2E+09	3E+09	1E+09	2E+09	3E+09	2E+09	p04805	Neuroblast differentiation-associated protein AHNAK
HNRNPA1 seq: 337 - 352 + Phospho (ST)	3E+08	3E+08	5E+08	6E+08	5E+08	5E+08	6E+08	5E+08	6E+08	5E+08	3E+08	5E+08	4E+08	6E+08	4E+08	6E+08	6E+08	6E+08	6E+08	6E+08	5E+08	8E+08	4E+08	5E+08	1E+09	1E+09	1E+09	2E+09	1E+09	1E+09	1E+09	1E+09	1E+09	1E+09	1E+09	1E+09	1E+09	1E+09	2E+09	1E+09	2E+09	1E+09	1E+09	1E+09	9E+08	2E+09	8E+08	1E+09	2E+09		p03886	Heterogeneous nuclear ribonucleoprotein A1
AHNAK pT4430	2E+08	1E+08	1E+08	13654	33854	7E+08	25552	1E+08	6E+07	5E+07	1E+09	53206	2E+08	3E+08	3E+08	33072	34984	19162	28832	4E+08	50401	7E+07	8E+07	42741	6E+08	44396	34144	19500	29103	2E+08	31655	19257	24890	17227	7E+08	1E+08	25062	27887	5E+07	4E+08	64618	38440	41974	13188	2E+08	4E+07	25947	30272	25732		p15440	Neuroblast differentiation-associated protein AHNAK
MRPS16 seq: 117 - 137 + Phospho (ST)	38324	6E+07	7E+07	7E+07	4E+07	45779	7E+07	5E+07	8E+07	8E+07	56234	8E+07	5E+07	9E+07	6E+07	8E+07	6E+07	6E+07	9E+07	8E+07	4E+07	7E+07	3E+07	5E+07	42741	2E+08	5E+07	7E+07	8E+07	6E+07	5E+07	6E+07	6E+07	6E+07	7E+07	1E+08	6E+07	4E+07	8E+07	1E+08	1E+08	6E+07	7E+07	1E+08	8E+07	5E+07	3E+07	7E+07	5E+07		p16972	28S ribosomal protein S16, mitochondrial
NEK9 seq: 853 - 871 + Phospho (ST)	3E+09	2E+09	2E+09	2E+09	2E+09	6E+09	3E+09	3E+09	3E+09	2E+09	8E+09	4E+09	4E+09	3E+09	2E+09	2E+09	3E+09	3E+09	2E+09	2E+09	3E+09	2E+09	4E+09	3E+09	3E+09	2E+09	3E+09	1E+09	2E+09	2E+09	3E+09	4E+09	4E+09	2E+09	2E+09	4E+09	2E+09	2E+09	3E+09	2E+09	3E+09	2E+09	3E+09	2E+09	2E+09	2E+09	2E+09				p01766	Serine/threonine-protein kinase Nek9
RELA seq: 134 - 149 no mod	2E+09	2E+09	2E+09	1E+09	2E+09	7E+09	3E+09	2E+09	2E+09	1E+09	8E+09	3E+09	3E+09	2E+09	2E+09	1E+09	2E+09	1E+09	1E+09	4E+09	2E+09	2E+09	1E+09	1E+09	6E+09	1E+09	1E+09	1E+09	1E+09	1E+09	9E+08	2E+09	2E+09	5E+09	1E+09	1E+09	2E+09	1E+09	5E+09	2E+09	1E+09	2E+09	1E+09	3E+09	1E+09	1E+09	1E+09	1E+09			p13782	Transcription factor p65
HDGF pS165	3E+09	3E+09	2E+09	3E+09	2E+09	9E+09	4E+09	3E+09	3E+09	2E+09	8E+09	4E+09	3E+09	2E+09	2E+09	4E+09	2E+09	3E+09	2E+09	4E+09	2E+09	3E+09	3E+09	2E+09	4E+09	1E+09	1E+09	1E+09	2E+09	9E+08	1E+09	2E+09	1E+09	1E+09	2E+09	1E+09	1E+09	1E+09	1E+09	2E+09	1E+09	1E+09	1E+09	2E+09	1E+09	1E+09	1E+09	1E+09			p20702	Hepatoma-derived growth factor
ZC3H13 pT354 pS356	6E+08	6E+08	8E+08	1E+09	1E+09	2E+09	7E+08	7E+08	1E+09	2E+09	8E+08	1E+09	8E+08	1E+09	2E+09	7E+08	1E+09	1E+09	1E+09	7E+08	6E+08	7E+08	2E+09	1E+09	2E+09	2E+09	2E+09	2E+09	2E+09	1E+09	4E+08	2E+09	3E+09	1E+09	1E+09	1E+09	2E+09	2E+09	9E+08	3E+09	8E+08	1E+09	3E+09	9E+08	1E+09	1E+09	2E+09				p12611	Zinc finger CCCH domain-containing protein 13
AHNAK pS2580	38324	1E+07	3E+07	13654	2E+07	8E+07	4E+07	2E+07	2E+07	1E+07	2E+07	3E+07	2E+07	2E+07	1E+07	1E+07	3E+06	2E+07	3E+07	2E+07	3E+07	1E+07	2E+07	2E+07	2E+07	44396	34144	1E+07	1E+07	45838	2E+06	19257	1E+07	5E+06	1E+07	27262	4E+06	8E+06	7E+06	9E+06	64618	7E+06	6E+06	1E+07	1E+07	3E+06	2E+07	1E+07	5E+06		p13710	Neuroblast differentiation-associated protein AHNAK
SHROOM1 seq: 186 - 200 + Phospho (ST)	2E+07	4E+07	2E+07	3E+07	3E+07	45779	5E+07	2E+07	4E+07	2E+07	1E+08	6E+07	5E+07	5E+07	40867	2E+07	3E+07	2E+07	2E+07	4E+07	2E+07	3E+07	4E+07	2E+07	1E+08	2E+08	2E+08	1E+08	1E+08	1E+08	8E+07	2E+08	2E+08	1E+08	1E+08	1E+08	1E+08	2E+08	2E+08	1E+08	1E+08	1E+08	1E+08	1E+08	1E+08	1E+08	1E+08	2E+08	1E+08		p06261	Protein Shroom1
CTNNB1 pS675	38324	4E+07	9E+07	4E+07	3E+08	45779	2E+08	9E+07	7E+07	8E+07	56234	5E+08	1E+08	1E+08	5E+07	3E+08	8E+06	1E+08	1E+08	5E+07	5E+07	2E+07	1E+08	1E+08	3E+08	8E+06	34144	5E+07	8E+07	6E+07	31655	6E+06	1E+08	1E+08	3E+07	2E+08	27262	2E+07	3E+07	1E+08	3E+08	8E+07	2E+07	7E+07	1E+08	6E+07	41467	6E+07	2E+08	1E+08	p07072	Catenin beta-1
RANBP2 pS2900	38324	3E+07	1E+08	1E+08	1E+08	2E+08	1E+08	1E+08	8E+07	2E+08	1E+08	2E+08	1E+08	1E+08	33072	1E+08	2E+08	4E+07	2E+08	3E+07	1E+08	2E+08	1E+08	1E+08	4E+08	8E+08	3E+07	19500	1E+08	2E+08	4E+06	19257	2E+08	2E+08	1E+08	2E+07	1E+08	27887	36936	2E+08	3E+08	2E+08	2E+08	1E+07	13188	2E+08	41467	5E+08	2E+08	1E+08	p01505	E3 SUMO-protein ligase RanBP2
MYO9B pS1405	4E+08	2E+08	5E+08	4E+08	3E+08	9E+08	2E+08	3E+08	4E+08	3E+08	2E+09	1E+09	1E+09	1E+09	7E+08	1E+08	1E+08	3E+08	3E+08	5E+08	3E+08	4E+08	3E+08	9E+07	44396	4E+07	4E+07	6E+07	5E+07	31655	19257	6E+07	5E+07	1E+08	2E+08	2E+07	5E+07	8E+07	6E+07	100327	64618	7E+07	6E+07	4E+07	134692	2E+07	6E+07	5E+07	4E+07		p13039	Unconventional myosin-IXb
TFPI2 seq: 182 - 201 no mod	2E+08	3E+08	2E+08	2E+08	3E+08	8E+08	5E+08	3E+08	2E+08	3E+08	1E+09	6E+08	3E+08	3E+08	3E+08	5E+08	3E+08	3E+08	2E+08	5E+08	2E+08	2E+08	3E+08	4E+08	6E+07	4E+07	1E+08	9E+07	2E+08	5E+07	3E+07	9E+07	1E+08	1E+08	7E+07	5E+07	1E+08	9E+07	3E+08	9E+07	4E+07	1E+08	8E+07	2E+08	7E+07	5E+07	5E+07	6E+07			p02991	Tissue factor pathway inhibitor 2
TOR1AIP1 seq: 153 - 175 + 2 Phospho (ST)	2E+08	4E+08	3E+08	3E+08	4E+08	45779	6E+08	4E+08	3E+08	4E+08	5E+08	6E+08	4E+08	4E+08	7E+08	4E+08	5E+08	5E+08	6E+08	7E+08	3E+08	4E+08	7E+08	2E+08	2E+08	3E+08	4E+08	6E+08	3E+08	2E+08	1E+08	4E+08	2E+08	2E+08	2E+08	2E+08	7E+08	4E+08	2E+08	3E+08	4E+08	3E+08	2E+08	3E+08	2E+08						p18275	Torsin-1A-interacting protein 1
HCFC1 pS666	9E+07	2E+08	2E+08	3E+08	4E+08	3E+08	2E+08	3E+08	2E+08	1E+09	7E+08	3E+08	7E+08	3E+08	1E+08	3E+08	3E+08	3E+08	2E+08	2E+08	3E+08	3E+08	3E+08	1E+08	5E+08	4E+08	3E+08	4E+08	2E+08	3E+08	4E+08	3E+08	4E+08	3E+08	3E+08	4E+08	3E+08	3E+08	4E+08	6E+08	3E+08	3E+08	3E+08	8E+07	3E+08						p02685	Host cell factor 1
KHSRP pM207	1E+07	37149	39656	13654	2E+07	25552	34892	34104	2E+07	7E+07	53206	2E+07	40879	2E+07	3E+07	33072	34984	9E+06	28832	39759	50401	21442	5E+06	42741	68554	6E+06	8E+06	8E+06	3E+06	2E+07	31655	6E+06	24890	17227	3E+07	27262	4E+06	2E+07	27887	1E+07	1E+07	64618	6E+06	2E+07	1E+07	134692	6E+06	1E+07	1E+07	2E+07	p09986	Far upstream element-binding protein 2
CDK12 pT692	1E+09	2E+09	7E+08	6E+08	1E+09	4E+09	2E+09	1E+09	9E+08	1E+09	4E+09	2E+09	6E+08	8E+08	1E+09	3E+09	2E+09	9E+08	8E+08	1E+09	3E+09	1E+09	1E+09	1E+09	2E+09	8E+08	6E+08	7E+08	7E+08	1E+09	7E+08	5E+08	6E+08	6E+08	2E+09	8E+08	6E+08	6E+08	7E+08	2E+09	8E+08	6E+08	7E+08	9E+08	2E+09	8E+08	6E+08	6E+08	6E+08		p20421	Cyclin-dependent kinase 12
FKBP4 seq: 391 - 399 no mod	4E+07	5E+07	3E+07	8E+06	3E+07	2E+08	5E+07	4E+07	2E+07	1E+07	2E+08	4E+07	9E+07	2E+07	4E+07	33072	3E+07	4E+07	28832	6E+07	3E+07	1E+07	7E+06	1E+07	68554	44396	34144	3E+06	29103	45838	31655	892081	24890	17227	44771	27262	25062	27887	36936	100327	64618	38440	41974	13188	134692	41467	25947	30272	25732		p02279	Peptidyl-prolyl cis-trans isomerase FKBP4
TRIM28 pS489	1E+07	5E+07	7E+07	1E+08	1E+08	3E+08	2E+08	7E+07	1E+08	7E+07	5E+08	2E+08	8E+07	2E+08	6E+07	1E+08	1E+07	1E+08	2E+08	5E+07	6E+07	2E+07	1E+08	2E+08	1E+08	44396	34144	6E+07	2E+07	2E+07	5E+06	19257	7E+07	2E+07	1E+08	7E+06	2E+07	2E+07	5E+07	3E+07	100327	5E+06	1E+07	2E+07	2E+07	4E+07	41467	6E+07	6E+07	4E+07	p00828	Transcription intermediary factor 1-beta
NUMA1 seq: 231 - 242 no mod	9E+06	4E+07	1E+08	6E+07	9E+07	3E+08	1E+08	9E+07	8E+07	6E+07	4E+08	2E+08	1E+08	5E+07	6E+07	9E+07	4E+07	1E+08	4E+07	7E+07	3E+07	8E+07	1E+08	7E+07	6E+07	4E+06	34144	19500	3E+07	1E+07	31655	19257	1E+07	3E+07	1E+07	4E+07	3E+06	9E+06	27887	1E+07	3E+07	1E+07	9E+06	3E+07	3E+07	134692	2E+07	6E+06	30272	2E+07	p18895	Nuclear mitotic apparatus protein 1
ATF2 seq: 78 - 98 + Phospho (ST)	38324	37149	39656	13654	1E+07	1E+08	25552	34892	34104	26736	2E+08	2E+08	1E+08	2E+07	40867	33072	34984	19162	28832	5E+06	50401	21442	19024	7E+06	68554	44396	34144	19500	29103	45838	31655	19257	24890	17227	44771	27262	25062	27887	36936	100327	64618	38440	41974	13188	134692	41467	25947	30272	25732		p09730	Cyclic AMP-dependent transcription factor ATF-2
SSFA2 seq: 737 - 749 + Phospho (ST)	2E+09	2E+09	2E+09	2E+09	2E+09	7E+09	3E+09	3E+09	3E+09	6E+09	3E+09	4E+09	3E+09	3E+09	4E+09	3E+09	3E+09	4E+09	3E+09	4E+09	3E+09	3E+09	3E+09	9E+09	4E+09	5E+09	5E+09	6E+09	6E+09	3E+09	4E+09	4E+09	5E+09	5E+09	4E+09	4E+09	6E+09	6E+09	4E+09	4E+09	5E+09	4E+09	5E+09	3E+09	4E+09	7E+09	4E+09				p04187	Sperm-specific antigen 2
PKM seq: 384 - 399 no mod	38324	2E+07	39656	13654	5E+07	4E+08	3E+07	34892	1E+07	26736	7E+08	1E+08	6E+07	3E+07	18798	2E+07	33072	1E+07	4E+07	28832	39759	50401	21442	2E+07	5E+06	4E+07	44396	34144	19500	4E+06	45838	31655	19257	7E+06	17227	5E+07	27262	25062	27887	36936	2E+07	64618	38440	41974	13188	3E+07	41467	25947	1E+07	25732	p17991	Pyruvate kinase PKM
SCYL3 pS707	1E+08	1E+08	2E+08	8E+07	7E+07	4E+08	2E+08	7E+07	6E+07	9E+08	3E+08	3E+08	2E+08	1E+08	8E+07	1E+08	8E+07	7E+07	2E+08	7E+07	1E+08	8E+07	9E+07	5E+07	3E+07	4E+07	4E+07	2E+07	4E+07	3E+07	1E+07	3E+07	3E+07	5E+07	4E+07	3E+07	2E+07	4E+07	8E+07	4E+07	3E+07	2E+07	5E+07	2E+07	3E+07	2E+07	2E+07				p13344	Protein-associating with the carboxyl-terminal domain of ezrin
ZBTB21 pS1003	5E+07	7E+07	7E+07	6E+07	6E+07	2E+08	7E+07	7E+07	8E+07	56234	2E+08	1E+08	1E+08	1E+08	40867	6E+07	6E+07	8E+07	5E+07	9E+07	7E+07	5E+07	9E+07	1E+08	6E+08	9E+07	7E+07	1E+08	1E+08	2E+08	1E+08	7E+07	2E+08	3E+08	1E+08	2E+08	1E+08	7E+07	1E+08	2E+08	4E+08	1E+08	8E+07	1E+08	1E+08	3E+08	9E+07	1E+08	9E+07	1E+08	p13800	Zinc finger and BTB domain-containing protein 21
CLCC1 pS438	3E+08	8E+08	8E+08	7E+08	5E+08	45779	1E+09	7E+08	6E+08	6E+08	56234	1E+09	1E+09	1E+09	8E+08	40867	6E+08	8E+08	6E+08	5E+08	1E+09	5E+08	7E+08	7E+08	5E+08	8E+08	1E+08	2E+08	2E+08	1E+08	5E+08	1E+08	3E+08	1E+08	2E+08	5E+08	2E+08	1E+08	2E+08	5E+08	2E+08	2E+08	2E+08	2E+08	4E+08	2E+08	2E+08	2E+08	1E+08		p16508	Chloride channel CLIC-like protein 1
CAMSAP2 seq: 1017 - 1028 + Phospho (ST)	2E+07	7E+07	5E+07	6E+07	1E+08	2E+08	8E+07	6E+07	6E+07	7E+07	4E+08	2E+08	1E+08	2E+08	1E+08	4E+07	5E+07	5E+07	7E+07	1E+08	5E+07	5E+07	5E+07	1E+08	5E+08	2E+08	2E+08	2E+08	2E+08	2E+08	1E+08	1E+08	2E+08	4E+08	2E+08	2E+08	2E+08	2E+08	3E+08	2E+08	2E+08	2E+08	2E+08	2E+08	1E+08	2E+08	2E+08	2E+08			p07418	Calmodulin-regulated spectrin-associated protein 2
ACTB seq: 257 - 284 no mod	8E+06	9E+07	1E+08	13654	2E+08	6E+08	1E+08	1E+08	9E+07	2E+07	2E+09	9E+08	2E+08	4E+08	2E+07	1E+08	33072	5E+07	3E+08	28832	39759	1E+07	1E+07	1E+08	3E+07	3E+08	44396	34144	19500	1E+08	1E+08	31655	19257	9E+07	17227	3E+08	27262	2E+08	6E+07	5E+07	100327	3E+07	38440	2E+08	2E+08	41467	9E+07	1E+08	8E+07		p19033	Actin, cytoplasmic 1
BCLAF1 seq: 256 - 271 + 2 Phospho (ST)	1E+09	4E+08	7E+08	4E+08	5E+08	3E+09	5E+08	8E+08	5E+08	2E+09	2E+09	1E+09	7E+08	6E+08	1E+09	4E+08	1E+09	8E+08	6E+08	1E+09	2E+08	4E+08	7E+08	5E+08	2E+09	4E+08	2E+08	6E+08	9E+08	9E+08	7E+08	2E+08	4E+08	8E+08	2E+09	4E+08	3E+08	5E+08	1E+09	9E+08	5E+08	2E+08	1E+09	1E+09	4E+08	2E+08	8E+08	8E+08			p09010	Bcl-2-associated transcription factor 1
KLC4 pS590	2E+09	3E+09	5E+09	2E+09	2E+09	5E+09	3E+09	5E+09	2E+09	1E+09	5E+09	3E+09	4E+09	2E+09	2E+09	4E+09	2E+09	5E+09	3E+09	2E+09	3E+09	3E+09	4E+09	2E+09	3E+09	1E+09	2E+09	1E+09	2E+09	1E+09	1E+09	1E+09	8E+08	2E+09	1E+09	2E+09	2E+09	1E+09	2E+09	2E+09	1E+09	2E+09	2E+09	2E+09	2E+09	1E+09	2E+09	1E+09	1E+09		p21037	Kinesin light chain 4
PHIP seq: 1554 - 1571 + Phospho (ST)	2E+08	4E+08	3E+08	2E+08	2E+08	9E+08	5E+08	4E+08	2E+08	3E+08	7E+08	4E+08	2E+08	2E+08	3E+08	8E+08	3E+08	4E+08	3E+08	3E+08	7E+08	2E+08	3E+08	3E+08	2E+08	5E+09	2E+09	2E+09	2E+09	1E+09	3E+09	2E+09	1E+09	2E+09	3E+09	3E+09	2E+09	2E+09	1E+09	2E+09	4E+09	3E+09	2E+09	2E+09	3E+09	3E+09	2E+09	1E+09	2E+09	2E+09	p20214	PH-interacting protein
MAP7 pS219	1E+08	1E+08	1E+08	1E+08	9E+07	3E+08	2E+08	1E+08	1E+08	1E+08	4E+08	2E+08	2E+08	1E+08	3E+08	2E+08	2E+08	2E+08	1E+08	3E+08	1E+08	1E+08	2E+08	2E+08	2E+08	44396	4E+07	5E+07	6E+07	8E+07	2E+07	4E+07	5E+07	5E+07	1E+08	3E+07	5E+07	4E+07	5E+07	1E+08	4E+07	6E+07	6E+07	6E+07	1E+08	3E+07	6E+07	4E+07	5E+07		p10973	Ensconsin
TRA2B seq: 198 - 217 + Phospho (ST)	6E+08	5E+08	9E+08	4E+08	33854	3E+09	1E+09	1E+09	5E+08	26736	3E+09	1E+09	9E+08	5E+08	18798	6E+08	5E+08	1E+09	5E+08	28832	6E+08	5E+08	4E+08	5E+08	42741	2E+09	4E+08	2E+08	4E+08	29103	1E+09	5E+08	2E+08	24890	1E+09	2E+09	4E+08	2E+08	27887	36936	1E+09	4E+08	4E+08	41974	13188	2E+09	2E+08	3E+08	30272	25732	p06927	Transformer-2 protein homolog beta
PHACTR4 seq: 342 - 364 + 2 Phospho (ST)	5E+09	4E+09	7E+09	6E+09	8E+09	1E+10	8E+09	7E+09	6E+09	6E+09	1E+10	6E+09	6E+09	7E+09	8E+09	5E+09	7E+09	7E+09	6E+09	1E+10	6E+09	5E+09	7E+09	7E+09	4E+09	3E+09	4E+09	3E+09	2E+09	4E+09	3E+09	2E+09	3E+09	3E+09	2E+09	3E+09	2E+09	2E+09	3E+09	3E+09	3E+09	4E+09	3E+09	3E+09	4E+09	2E+09	4E+09	4E+09			p04389	Phosphatase and actin regulator 4
HDAC5 pS265	1E+09	1E+09	2E+09	7E+08	1E+09	2E+09	2E+09	1E+09	9E+08	8E+08	2E+09	2E+09	2E+09	1E+09	2E+09	2E+09	2E+09	2E+09	9E+08	1E+09	2E+09	1E+09	1E+09	7E+08	2E+09	2E+08	5E+07	34144	9E+07	4E+07	2E+08	7E+07	4E+07	1E+08	17227	2E+08	7E+07	25062	9E+07	5E+07	3E+08	2E+07	38440	9E+07	1E+07	134692	9E+07	1E+07	1E+07	6E+07	p03804	Histone deacetylase 5
LRRC16A seq: 1067 - 1075 + Phospho (ST)	2E+07	3E+07	3E+07	4E+07	2E+07	7E+07	3E+07	5E+07	3E+07	3E+07	5E+07	2E+07	1E+07	2E+07	2E+07	4E+07	4E+07	4E+07	5E+07	4E+07	3E+07	4E+07	3E+07	5E+07	3E+07	1E+07	34144	2E+07	2E+07	2E+07	9E+06	19257	2E+07	2E+07	2E+07	1E+07	2E+07	1E+07	1E+07	3E+07	64618	3E+07	1E+07	2E+07	2E+07	2E+07	2E+07	2E+07	2E+07		p03898	Leucine-rich repeat-containing protein 16A
CRTC3 pS370	2E+09	2E+09	4E+09	3E+09	4E+09	2E+10	3E+09	3E+09	3E+09	2E+09	2E+10	6E+09	7E+09	6E+09	3E+09	6E+09	2E+09	4E+09	4E+09	2E+09	7E+09	2E+09	3E+09	3E+09	2E+09	9E+08	4E+08	3E+08	4E+08	3E+08	4E+08	3E+08	2E+08	4E+08	4E+08	5E+08	3E+08	3E+08	4E+08	5E+08	3E+08	3E+08	4E+08	4E+08	2E+08	3E+08	5E+08	3E+08			p11860	CREB-regulated transcription coactivator 3
PDIA3 seq: 148 - 173 no mod	2E+08	2E+08	3E+08	1E+08	9E+07	2E+08	2E+08	4E+08	2E+08	1E+08	5E+08	8E+07	2E+08	1E+08	1E+08	8E+07	3E+08	2E+08	4E+08	1E+08	3E+08	4E+08	2E+08	1E+08	1E+07	2E+08	2E+08	1E+08	8E+07	45838	2E+08	6E+07	1E+08	1E+08	3E+08	2E+08	1E+08	7E+07	2E+08	2E+08	2E+08	1E+08	9E+07	1E+08	3E+08	2E+08	7E+07	1E+08			p16671	Protein disulfide-isomerase A3
PCDH1 seq: 379 - 390 no mod	3E+07	4E+07	39656	13654	33854	2E+08	1E+08	2E+07	34104	9E+06	3E+08	8E+07	3E+07	9E+06	18798	4E+07	5E+07	3E+07	4E+06	4E+06	1E+08	2E+07	21442	1E+07	42741	6E+07	6E+06	4E+07	2E+07	29103	2E+07	31655	19257	24890	17227	44771	3E+07	3E+07	3E+07	36936	5E+07	4E+07	2E+07	13188	5E+07	3E+06	4E+07	30272	25732		p18488	Protocadherin-1
SPECC1 pS847	5E+07	8E+07	9E+07	8E+07	7E+07	2E+08	8E+07	8E+07	4E+07	3E+08	2E+08	1E+08	1E+08	9E+07	5E+07	7E+07	8E+07	6E+07	1E+08	1E+08	7E+07	7E+07	8E+07	5E+08	4E+08	4E+08	4E+08	4E+08	5E+08	4E+08	4E+08	4E+08	5E+08	7E+08	5E+08	4E+08	5E+08	8E+08	5E+08	4E+08	4E+08	5E+08	4E+08	4E+08	4E+08	4E+08	5E+08				p04543	Cytospin-B
ARHGAP35 seq: 1070 - 1098 + Phospho (Y)	38324	9E+07	5E+07	2E+07	4E+07	7E+07	1E+08	8E+07	5E+07	5E+07	2E+07	8E+07	5E+07	6E+07	5E+07	8E+07	1E+08	8E+07	7E+07	6E+07	1E+08	1E+08	1E+08	5E+07	9E+07	3E+08	5E+08	2E+08	2E+08	1E+08	3E+08	2E+08	2E+08	4E+08	3E+08	2E+08	1E+08	1E+08	3E+08	1E+08	2E+08	3E+08	2E+08	3E+08	2E+08	2E+08	2E+08				p03246	Rho GTPase-activating protein 35
ATXN1 pS775	2E+08	2E+08	2E+08	2E+08	2E+08	5E+08	2E+08	1E+08	2E+08	1E+08	4E+08	2E+08	2E+08	3E+08	1E+08	9E+07	2E+08	2E+08	1E+08	2E+08	1E+08	2E+08	9E+07	4E+08	1E+08	2E+08	3E+08	2E+08	3E+08	2E+08	2E+08	3E+08	3E+08	6E+08	4E+08	2E+08	3E+08	3E+08	5E+08	2E+08	2E+08	3E+08	2E+08	4E+08	2E+08	2E+08	3E+08	3E+08			p06410	Ataxin-1
MDC1 seq: 360 - 393 + Phospho (ST)	38324	5E+07	1E+08	8E+07	8E+07	45779	1E+08	7E+07	1E+08	8E+07	56234	1E+08	2E+08	2E+08	2E+08	40867	8E+07	1E+08	1E+08	4E+07	9E+07	7E+07	7E+07	1E+08	7E+07	1E+09	3E+08	3E+08	7E+08	4E+08	6E+08	3E+08	3E+08	3E+08	6E+08	7E+08	5E+08	3E+08	5E+08	5E+08	5E+08	3E+08	4E+08	5E+08	4E+08	3E+08	4E+08	3E+08	7E+08	3E+08	p15520	Mediator of DNA damage checkpoint protein 1
CHD1 seq: 89 - 98 + Phospho (ST)	7E+07	6E+07	8E+07	6E+07	6E+07	3E+08	8E+07	6E+07	7E+07	6E+07	1E+08	6E+07	8E+07	5E+07	5E+07	2E+08	8E+07	1E+08	9E+07	7E+07	1E+08	6E+07	9E+07	6E+07	6E+07	2E+08	1E+08	1E+08	2E+08	2E+08	1E+08	2E+08	2E+08	1E+08	2E+08	2E+08	2E+08	1E+08	1E+08	2E+08	3E+08	1E+08	1E+08	2E+08	1E+08	1E+08	2E+08	1E+08	1E+08		p03782	Chromodomain-helicase-DNA-binding protein 1
AAK1 seq: 10 - 37 + Phospho (ST)	9E+07	1E+07	1E+08	1E+08	3E+08	2E+08	6E+07	9E+07	3E+08	2E+08	6E+07	7E+07	6E+07	1E+08	7E+07	7E+07	1E+08	3E+08	2E+08	3E+07	1E+08	9E+07	4E+08	1E+08	4E+07	6E+07	3E+07	8E+07	2E+07	4E+07	5E+07	17227	7E+07	2E+07	4E+07	3E+07	1E+08	4E+07	4E+07	3E+07	13188	8E+07	2E+07	6E+07	3E+07	5E+07					p17286	AP2-associated protein kinase 1
PAK1 seq: 216 - 237 + Phospho (ST)	3E+09	2E+09	3E+09	3E+09	3E+09	45779	4E+09	3E+09	3E+09	3E+09	56234	5E+09	5E+09	5E+09	4E+09	40867	3E+09	4E+09	3E+09	2E+09	39759	3E+09	3E+09	4E+09	3E+09	4E+09	4E+09	5E+09	5E+09	3E+09	45838	4E+09	2E+09	4E+09	4E+09	5E+09	6E+09	4E+09	4E+09	4E+09	6E+09	6E+09	4E+09	4E+09	5E+09	5E+09	5E+09	5E+09	4E+09	4E+09	p18130	Serine/threonine-protein kinase PAK 1
ANXA6 seq: 35 - 50 no mod	38324	37149	3E+06	13654	3E+06	2E+07	25552	2E+06	34104	26736	3E+08	53206	39754	40879	18798	40867	33072	34984	1E+07	28832	39759	50401	21442	19024	42741	68554	44396	34144	19500	29103	45838	31655	19257	24890	17227	44771	27262	25062	27887	36936	100327	64618	38440	41974	13188	134692	41467	25947	30272	25732	p16115	Annexin A6
CAMSAP1 seq: 1396 - 1424 + Phospho (ST)	8E+07	1E+08	2E+08	7E+07	7E+07	2E+08	2E+08	1E+08	8E+07	2E+08	9E+07	9E+07	8E+07	9E+07	2E+08	1E+08	2E+08	1E+08	1E+08	2E+08	1E+08	1E+08	2E+08	3E+08	1E+08	9E+07	2E+08	1E+08	2E+08	2E+08	2E+08	8E+07	1E+08	2E+08	1E+08	9E+07	1E+08	9E+07	4E+08	2E+08	2E+08	2E+08	2E+08	1E+08	1E+08	1E+08					p02259	Calmodulin-regulated spectrin-associated protein 1
YY1 pS247	38324	2E+08	3E+08	2E+08	2E+08	4E+08	5E+08	3E+08	3E+08	2E+08	1E+08	3E+08	3E+08	4E+08	4E+08	3E+08	3E+08	1E+08	2E+08	4E+08	2E+08	3E+08	3E+08	7E+08	44396	1E+08	6E+08	29103	4E+08	8E+07	2E+08	2E+08	24890	2E+08	4E+08	2E+08	2E+08	8E+07	1E+08	3E+08	3E+08	4E+08	3E+08	4E+07	2E+08	1E+08	25947	3E+08	25732		p13338	Transcriptional repressor protein YY1
ATXN2 pS758	4E+08	6E+08	5E+08	4E+08	3E+08	1E+09	6E+08	5E+08	5E+08	3E+08	1E+09	7E+08	5E+08	5E+08	6E+08	6E+08	3E+08	4E+08	4E+08	3E+08	6E+08	3E+08	5E+08	4E+08	3E+08	2E+09	6E+08	5E+08	7E+08	5E+08	6E+08	6E+08	6E+08	7E+08	1E+09	8E+08	1E+09	7E+08	6E+08	1E+09	8E+08	1E+09	6E+08	7E+08	1E+09	7E+08	1E+09	7E+08	6E+08		p08177	Ataxin-2
SEC16A seq: 408 - 437 + Phospho (ST)	6E+08	6E+08	7E+08	6E+08	6E+08	2E+09	1E+09	6E+08	7E+08	7E+08	2E+09	1E+09	9E+08	1E+09	1E+09	2E+09	5E+08	7E+08	7E+08	6E+08	1E+09	5E+08	7E+08	8E+08	1E+09	4E+08	4E+08	5E+08	4E+08	3E+08	6E+08	4E+08	4E+08	8E+08	3E+08	3E+08	4E+08	4E+08	9E+08	4E+08	3E+08	5E+08	4E+08	6E+08	4E+08	4E+08	5E+08	4E+08			p15141	Protein transport protein Sec16A
NXF1 seq: 259 - 276 no mod	9E+06	6E+07	7E+07	6E+07	1E+08	7E+08	1E+08	7E+07	5E+07	3E+07	6E+08	2E+08	4E+07	8E+07	3E+07	2E+08	7E+07	1E+08	2E+07	8E+07	1E+07	3E+07	8E+07	3E+07	2E+08	7E+06	34144	5E+07	4E+07	3E+07	5E+06	19257	7E+07	4E+07	6E+07	1E+07	2E+07	3E+07	5E+07	6E+07	8E+07	2E+07	8E+07	8E+07	6E+07	7E+06	3E+07	8E+07	6E+07		p14184	Nuclear RNA export factor 1
CCDC86 pS80 pS91	8E+07	6E+07	8E+07	3E+07	5E+07	45779	7E+07	7E+07	7E+07	6E+07	56234	1E+08	2E+08	8E+07	1E+08	40867	1E+08	7E+07	6E+07	39759	9E+07	1E+08	5E+07	6E+07	68554	3E+08	5E+08	3E+08	2E+08	45838	3E+08	2E+08	4E+08	2E+08	2E+08	44771	3E+08	3E+08	1E+08	3E+08	100327	2E+08	4E+08	2E+08	2E+08	134692	4E+08	5E+08	2E+08	2E+08	p10953	Coiled-coil domain-containing protein 86
CEP170P1 seq: 230 - 251 + Phospho (ST)	1E+08	2E+08	2E+08	1E+08	2E+08	6E+08	4E+08	2E+08	2E+08	2E+08	6E+08	4E+08	2E+08	3E+08	2E+08	4E+08	3E+08	2E+08	2E+08	1E+08	3E+08	2E+08	2E+08	1E+08	9E+08	4E+08	3E+08	3E+08	4E+08	3E+08	3E+08	4E+08	7E+08	5E+08	4E+08	3E+08	4E+08	8E+08	5E+08	3E+08	4E+08	4E+08	6E+08	4E+08	4E+08	5E+08	3E+08				p03761	Cep170-like protein
ARHGEF12 seq: 318 - 345 + Phospho (ST)	2E+08	2E+08	2E+08	2E+08	2E+08	9E+08	2E+08	2E+08	2E+08	2E+09	4E+08	4E+08	4E+08	3E+08	3E+08	5E+08	2E+08	2E+08	1E+08	4E+08	2E+08	2E+08	3E+08	1E+09	2E+08	3E+08	4E+08	2E+08	4E+08	3E+08	2E+08	3E+08	4E+08	6E+08	4E+08	3E+08	3E+08	4E+08	6E+08	4E+08	3E+08	3E+08	3E+08	5E+08	3E+08	4E+08	3E+08	3E+08			p13496	Rho guanine nucleotide exchange factor 12
TNK2 seq: 827 - 839 + Phospho (ST)	1E+08	2E+08	2E+08	8E+07	1E+08	9E+08	2E+08	1E+08	7E+07	1E+08	1E+09	2E+08	2E+08	1E+08	2E+08	4E+08	1E+08	2E+08	1E+08	1E+08	4E+08	1E+08	1E+08	9E+07	1E+08	5E+08	2E+08	2E+08	1E+08	1E+08	3E+08	1E+08	9E+07	1E+08	1E+08	2E+08	2E+08	1E+08	5E+08	2E+08	1E+08	2E+08	1E+08	3E+08	1E+08	2E+08	9E+07	1E+08			p00437	Activated CDC42 kinase 1
NIPBL seq: 365 - 402 + Phospho (ST)	1E+08	1E+08	1E+08	1E+08	1E+08	2E+08	1E+08	1E+08	1E+08	3E+08	2E+08	2E+08	3E+08	2E+08	40867	4E+07	6E+07	1E+08	7E+07	2E+08	1E+08	21442	2E+08	1E+08	2E+08	44396	34144	2E+08	8E+07	1E+08	7E+07	4E+07	1E+08	1E+08	1E+08	1E+08	25062	1E+08	1E+08	64618	8E+07	41974	13188	2E+08	6E+07	1E+08	2E+08	25732			p00890	Nipped-B-like protein
MICALL2 pS153	4E+07	4E+07	6E+07	6E+07	4E+07	1E+08	5E+07	2E+07	6E+07	3E+07	4E+08	1E+08	8E+07	8E+07	9E+07	1E+08	2E+07	3E+07	4E+07	3E+07	1E+08	3E+07	3E+07	6E+07	3E+07	7E+07	5E+07	5E+07	5E+07	1E+08	6E+07	3E+07	6E+07	7E+07	7E+07	2E+08	7E+07	5E+07	7E+07	6E+07	3E+07	9E+07	7E+07	6E+07	9E+07	2E+07	7E+07	6E+07	6E+07	8E+07	p12749	MICAL-like protein 2
TRA2A seq: 200 - 232 + 2 Phospho (ST)	1E+08	5E+07	8E+07	7E+07	9E+07	3E+08	1E+08	1E+08	8E+07	9E+07	2E+08	9E+07	1E+08	9E+07	8E+07	2E+08	1E+08	1E+08	1E+08	9E+07	2E+08	1E+08	1E+08	2E+08	1E+08	2E+08	1E+08	2E+07	2E+08	1E+08	2E+08	7E+07	1E+08	2E+08	2E+08	2E+08	2E+08	5E+07	8E+07	1E+08	2E+08	2E+08	8E+07	2E+08	2E+08	2E+08	5E+07	9E+07	2E+08	2E+08	p20499	Transformer-2 protein homolog alpha
MAP4 pS787	3E+08	4E+08	7E+08	2E+08	3E+08	1E+09	4E+08	5E+08	3E+08	4E+08	5E+08	5E+08	6E+08	4E+08	5E+08	1E+09	4E+08	6E+08	4E+08	2E+08	1E+09	4E+08	5E+08	3E+08	6E+08	5E+09	9E+08	1E+09	1E+09	2E+09	9E+08	8E+08	8E+08	9E+08	3E+09	1E+09	1E+09	8E+08	2E+09	9E+08	9E+08	5E+08	1E+09	2E+09	2E+09	1E+09	1E+09	2E+09	1E+09		p19531	Microtubule-associated protein 4
TAS2R42 seq: 216 - 222 + Phospho (ST)	4E+07	37149	39656	13654	33854	9E+07	25552	6E+06	2E+07	26736	4E+07	2E+07	3E+07	1E+07	18798	40867	2E+07	34984	19162	28832	4E+07	50401	21442	19024	42741	2E+08	7E+07	2E+08	8E+07	8E+07	2E+08	6E+07	6E+07	1E+08	1E+08	3E+08	27262	1E+08	7E+07	2E+08	4E+08	8E+07	1E+08	7E+07	1E+08	2E+08	1E+08	8E+07	1E+08		p10661	Taste receptor type 2 member 42
SQSTM1 seq: 22 - 46 + Phospho (ST)	1E+09	9E+08	8E+08	8E+08	1E+09	3E+09	2E+09	1E+09	2E+09	1E+09	1E+09	7E+08	8E+08	8E+08	1E+09	1E+09	1E+09	1E+09	1E+09	1E+09	1E+09	1E+09	7E+08	1E+09	3E+09	1E+09	6E+08	2E+09	9E+08	1E+09	9E+08	4E+08	2E+09	2E+09	1E+09	2E+09	9E+08	5E+08	1E+09	2E+09	1E+09	5E+08	2E+09	2E+09	2E+09	9E+08	6E+08	1E+09	1E+09		p07435	Sequestosome-1
AHNAK seq: 4087 - 4111 + Phospho (ST)	9E+07	3E+08	2E+08	2E+08	3E+08	4E+08	3E+08	2E+08	2E+08	2E+08	7E+08	4E+08	2E+08	3E+08	2E+08	3E+08	9E+07	1E+08	2E+08	2E+08	4E+08	2E+08	1E+08	2E+08	2E+08	7E+08	7E+07	1E+08	2E+08	2E+08	8E+07	19257	1E+08	2E+08	1E+08	6E+08	2E+08	3E+08	2E+08	4E+08	2E+08	2E+08	7E+07	2E+08	2E+08	3E+08	8E+07	1E+08	2E+08	2E+08	p20914	Neuroblast differentiation-associated protein AHNAK
HIRIP3 pS125	1E+08	7E+07	2E+07	6E+07	4E+07	2E+08	2E+08	3E+07	4E+07	7E+08	2E+08	5E+07	6E+07	5E+07	3E+08	3E+07	4E+07	5E+07	5E+07	2E+08	6E+07	21442	3E+07	7E+07	4E+08	8E+07	34144	5E+07	1E+08	2E+08	1E+08	1E+08	8E+06	1E+08	1E+08	5E+08	5E+07	25062	7E+07	1E+08	2E+08	9E+06	9E+07	1E+08	3E+08	6E+07	9E+06	1E+08	1E+08		p09589	HIRA-interacting protein 3
HPS4 pS272	6E+07	3E+07	3E+07	1E+07	8E+06	1E+08	5E+07	3E+07	3E+07	7E+06	5E+08	2E+08	1E+08	2E+08	9E+07	2E+07	2E+07	1E+07	3E+07	6E+06	6E+07	3E+07	3E+07	4E+07	1E+08	8E+07	2E+07	2E+07	2E+07	8E+06	4E+07	8E+06	1E+07	2E+07	1E+07	7E+07	5E+07	2E+07	1E+07	9E+07	2E+07	3E+07	1E+07	13188	7E+07	2E+07	3E+07	9E+06	25732		p05314	Hermansky-Pudlak syndrome 4 protein
TMPO pT208	6E+09	7E+09	2E+09	2E+09	2E+09	1E+10	8E+09	2E+09	2E+09	2E+09	1E+10	1E+10	1E+09	2E+09	2E+09	1E+10	8E+09	9E+08	1E+09	8E+08	8E+09	7E+09	1E+09	1E+09	2E+09	4E+10	2E+10	8E+08	2E+09	1E+09	3E+10	2E+10	4E+08	1E+10	1E+09	4E+10	2E+10	1E+09	1E+09	7E+09	5E+10	3E+10	4E+08	8E+09	9E+08	4E+10	2E+10	9E+08	1E+09	8E+09	p20357	Lamina-associated polypeptide 2, isoforms beta/gamma
HNRNPH2 pS104	2E+10	1E+10	1E+10	9E+09	8E+09	4E+10	2E+10	1E+10	9E+09	8E+09	4E+10	2E+10	9E+09	9E+09	2E+10	1E+10	2E+10	1E+10	8E+09	2E+10	1E+10	1E+10	7E+09	2E+10	8E+09	1E+10	8E+09	4E+09	2E+10	7E+09	1E+10	8E+09	7E+09	2E+10	1E+10	1E+10	9E+09	8E+09	2E+10	1E+10	1E+10	7E+09	7E+09	1E+10	8E+09	7E+09	5E+09				p14584	Heterogeneous nuclear ribonucleoprotein H2
BCL9 pS917	4E+07	6E+07	5E+07	3E+07	4E+07	2E+08	9E+07	6E+07	5E+07	4E+07	2E+08	6E+07	4E+07	3E+07	5E+07	1E+08	6E+07	6E+07	3E+07	4E+07	1E+08	5E+07	6E+07	4E+07	6E+07	4E+07	4E+07	4E+07	3E+07	2E+07	3E+07	9E+06	4E+07	3E+07	6E+07	3E+07	3E+07	3E+07	4E+07	1E+08	5E+07	2E+07	4E+07	3E+07	5E+07	4E+07	3E+07	3E+07	2E+07		p04475	B-cell CLL/lymphoma 9 protein
TNK2 seq: 827 - 839 + Phospho (Y)	1E+08	2E+08	2E+08	8E+07	1E+08	9E+08	2E+08	1E+08	7E+07	1E+08	1E+09	2E+08	2E+08	1E+08	2E+08	4E+08	1E+08	2E+08	1E+08	1E+08	4E+08	1E+08	1E+08	9E+07	2E+08	5E+08	2E+08	2E+08	2E+08	1E+08	3E+08	1E+08	9E+07	1E+08	1E+08	2E+08	1E+08	1E+08	2E+08	5E+08	2E+08	1E+08	1E+08	1E+08	3E+08	1E+08	1E+08	9E+07	1E+08		p00436	Activated CDC42 kinase 1
EPS15 seq: 562 - 584 + Phospho (ST)	1E+08	3E+07	4E+07	5E+07	5E+07	45779	25552	34892	8E+07	26736	56234	6E+07	9E+07	6E+07	4E+07	40867	6E+07	7E+07	1E+08	28832	39759	3E+07	6E+07	6E+07	5E+07	68554	44396	7E+07	2E+08	5E+07	7E+07	5E+07	5E+07	2E+08	9E+07	1E+08	8E+07	9E+07	6E+07	36936	2E+08	7E+07	8E+07	8E+07	4E+07	1E+08	6E+07	1E+08	1E+08	1E+08	p03822	Epidermal growth factor receptor substrate 15
EIF2S2 pT111	3E+08	3E+08	5E+08	4E+08	5E+08	9E+08	6E+08	6E+08	5E+08	4E+08	7E+08	6E+08	7E+08	6E+08	4E+08	6E+08	5E+08	7E+08	5E+08	6E+08	3E+08	6E+08	6E+08	5E+08	1E+09	2E+09	1E+09	9E+08	8E+08	3E+08	8E+08	1E+09	1E+09	9E+08	8E+08	8E+08	1E+09	8E+08	7E+08	9E+08	8E+08	1E+09	9E+08	8E+08	1E+09	7E+08					p14336	Eukaryotic translation initiation factor 2 subunit 2
FGD6 seq: 550 - 560 + Phospho (ST)	1E+07	7E+07	1E+08	3E+07	4E+07	2E+08	1E+08	1E+08	7E+07	4E+07	2E+08	1E+08	1E+08	9E+07	3E+07	8E+07	6E+07	1E+08	1E+08	28832	9E+07	6E+07	1E+08	3E+07	3E+07	2E+08	6E+07	8E+07	7E+07	7E+07	2E+08	4E+07	3E+07	6E+07	8E+07	1E+08	7E+07	1E+08	7E+07	7E+07	8E+07	3E+07	7E+07	6E+07	8E+07	9E+07	5E+07	7E+07	7E+07	4E+07	p14984	FYVE, RhoGEF and PH domain-containing protein 6
TMPO pS424	1E+09	9E+08	1E+09	1E+09	1E+09	4E+09	2E+09	1E+09	1E+09	9E+08	2E+09	1E+09	8E+08	1E+09	9E+08	5E+09	1E+09	2E+09	2E+09	1E+09	3E+09	1E+09	1E+09	1E+09	1E+09	3E+09	8E+08	1E+09	1E+09	1E+09	6E+08	9E+08	1E+09	1E+09	2E+09	9E+08	1E+09	1E+09	2E+09	1E+09	2E+09	1E+09	1E+09	1E+09	8E+08	2E+09	1E+09	1E+09			p16518	Lamina-associated polypeptide 2, isoform alpha
SERBP1 pS234	1E+09	2E+09	2E+09	2E+09	2E+09	4E+09	3E+09	2E+09	2E+09	2E+09	6E+09	5E+09	3E+09	4E+09	3E+09	2E+09	2E+09	2E+09	2E+09	2E+09	3E+09	1E+09	1E+09	2E+09	2E+09	2E+10	5E+09	4E+09	7E+09	5E+09	7E+09	5E+09	3E+09	4E+09	6E+09	1E+10	6E+09	4E+09	6E+09	6E+09	1E+10	6E+09	5E+09	5E+09	6E+09	7E+09	3E+09	3E+09	5E+09	5E+09	p15970	Plasminogen activator inhibitor 1 RNA-binding protein
CCT8 seq: 391 - 400 no mod	5E+07	5E+07	4E+09	3E+09	3E+09	9E+07	7E+08	4E+09	4E+09	4E+09	5E+08	53206	3E+09	3E+09	3E+09	5E+07	2E+09	3E+09	3E+09	4E+09	5E+07	2E+09	3E+09	3E+09	4E+09	4E+07	1E+09	8E+08	1E+09	1E+09	1E+08	1E+09	9E+08	1E+09	1E+09	1E+08	2E+09	9E+08	1E+09	1E+09	100327	2E+09	9E+08	1E+09	2E+09	2E+08	2E+09	7E+08	1E+09	1E+09	p19265	T-complex protein 1 subunit theta
PBRM1 seq: 37 - 58 + Phospho (ST)	38324	37149	39656	13654	3E+07	3E+08	25552	34892	3E+07	26736	2E+08	2E+08	2E+07	1E+08	2E+07	6E+07	33072	34984	19162	28832	39759	50401	21442	4E+07	42741	7E+07	44396	34144	19500	29103	45838	31655	19257	3E+07	17227	7E+07	27262	25062	27887	36936	100327	64618	38440	41974	13188	134692	41467	1E+07	4E+07	25732	p07107	Protein polybromo-1
CLK4 seq: 323 - 343 + Phospho (ST)	6E+08	5E+08	4E+08	3E+08	2E+08	1E+09	6E+08	3E+08	3E+08	3E+08	1E+09	6E+08	5E+08	4E+08	4E+08	6E+08	6E+08	4E+08	3E+08	3E+08	6E+08	3E+08	5E+08	3E+08	2E+08	68554	2E+07	3E+07	5E+07	1E+07	45838	1E+07	19257	3E+07	5E+07	5E+07	4E+07	27262	25062	3E+07	2E+07	100327	1E+08	7E+07	2E+08	1E+08	5E+07	3E+07	25947	2E+07	25732	p00652	Dual specificity protein kinase CLK4
ACTG1 seq: 53 - 63 no mod	8E+08	7E+08	6E+08	4E+08	6E+08	2E+09	5E+08	4E+08	5E+08	4E+08	2E+09	7E+08	8E+08	4E+08	5E+08	6E+08	4E+08	5E+08	7E+08	4E+08	5E+08	3E+08	5E+08	4E+08	6E+08	9E+08	4E+08	5E+08	5E+08	9E+08	7E+08	4E+08	4E+08	7E+08	1E+09	7E+08	6E+08	4E+08	6E+08	2E+09	1E+09	5E+08	8E+08	7E+08	1E+09	8E+08	6E+08	3E+08	5E+08		p18196	Actin, cytoplasmic 2
NELFA seq: 220 - 238 + Phospho (ST)	3E+07	1E+08	1E+08	6E+07	8E+07	6E+08	2E+08	1E+08	7E+07	7E+08	5E+08	2E+08	1E+08	8E+07	1E+08	2E+08	2E+08	1E+08	9E+07	2E+08	7E+07	1E+08	6E+07	1E+08	8E+07	2E+07	2E+07	2E+07	45838	8E+06	19257	8E+07	8E+06	17227	6E+07	2E+07	4E+07	9E+06	2E+07	7E+07	3E+07	2E+07	9E+06	2E+07	134692	3E+07	3E+07	1E+07	25732		p08842	Negative elongation factor A
HIVEP1 pS2682	7E+07	2E+08	3E+08	2E+08	1E+08	5E+08	4E+08	3E+08	2E+08	1E+08	4E+08	4E+08	3E+08	3E+08	2E+08	3E+08	2E+08	3E+08	2E+08	1E+08	4E+08	2E+08	3E+08	2E+08	2E+08	6E+08	1E+08	2E+08	2E+08	1E+08	3E+08	1E+08	2E+08	8E+07	1E+08	4E+08	2E+08	3E+08	2E+08	8E+07	3E+08	2E+08	3E+08	2E+08	1E+08	2E+08	1E+08	2E+08	1E+08	1E+08	p20520	Zinc finger protein 40
SSB pS366	2E+10	1E+10	6E+09	8E+09	7E+09	2E+10	2E+10	4E+09	9E+09	7E+09	3E+10	1E+10	8E+09	1E+10	8E+09	9E+09	6E+09	8E+09	1E+10	8E+09	1E+10	5E+09	4E+09	1E+10	8E+09	1E+10	3E+09	2E+09	7E+09	6E+09	7E+09	4E+09	2E+09	6E+09	9E+09	1E+10	6E+09	2E+09	5E+09	8E+09	1E+10	1E+10	3E+09	5E+09	7E+09	1E+10	5E+09	3E+09	6E+09	7E+09	p16846	Lupus La protein
SHANK2 pS1282	5E+09	7E+08	2E+09	6E+09	5E+09	1E+10	4E+09	2E+09	7E+09	6E+09	9E+09	6E+09	4E+09	1E+10	8E+09	3E+09	5E+09	6E+09	1E+10	1E+10	2E+09	3E+09	4E+09	9E+09	1E+10	3E+09	2E+09	1E+09	6E+09	6E+09	4E+09	2E+09	2E+08	3E+09	7E+09	2E+09	2E+09	1E+09	2E+09	4E+09	7E+09	2E+09	2E+09	3E+09	6E+09	5E+09	3E+09	2E+09	6E+09	4E+09	p04691	SH3 and multiple ankyrin repeat domains protein 2
CANX pS583	5E+09	3E+10	2E+10	2E+10	2E+10	4E+10	3E+10	2E+10	1E+10	3E+10	2E+10	3E+10	2E+10	2E+10	2E+10	3E+10	2E+10	2E+10	2E+10	2E+10	3E+10	2E+10	2E+10	1E+10	2E+10	3E+10	8E+09	7E+09	9E+09	1E+10	1E+10	1E+10	7E+09	5E+09	7E+09	1E+10	1E+10	9E+09	1E+10	8E+09	1E+10	1E+10	7E+09	1E+10	1E+10	8E+09	1E+10	5E+09	9E+09	8E+09	p20856	Calnexin
FOSL2 pS200 pM209	8E+06	2E+07	39656	1E+07	33854	5E+07	2E+07	1E+07	3E+06	7E+06	3E+07	4E+07	1E+07	40879	1E+07	3E+07	9E+06	5E+06	19162	7E+06	3E+07	2E+07	3E+06	19024	42741	3E+08	2E+08	6E+07	6E+07	7E+07	1E+08	2E+08	1E+08	7E+07	6E+07	2E+08	1E+08	1E+08	1E+08	2E+08	1E+08	6E+07	6E+07	7E+07	1E+08	2E+08	1E+08	7E+07	1E+08		p06646	Fos-related antigen 2
SPTBN1 pT2187	8E+07	8E+07	4E+07	6E+07	5E+07	3E+08	1E+08	1E+08	9E+07	8E+07	5E+08	9E+07	5E+07	7E+07	6E+07	1E+08	1E+08	1E+08	1E+08	9E+07	1E+08	1E+08	1E+08	7E+07	1E+08	1E+08	9E+07	6E+07	1E+08	8E+07	45838	6E+07	6E+07	8E+07	7E+07	7E+07	8E+07	4E+07	5E+07	36936	8E+07	2E+08	7E+07	8E+07	8E+07	1E+08	5E+07	5E+07	5E+07	25732	p03078	Spectrin beta chain, non-erythrocytic 1
EPPK1 seq: 2714 - 2732 + Phospho (ST)	4E+09	6E+09	1E+10	6E+09	7E+09	4E+10	1E+10	1E+10	7E+09	5E+09	6E+10	2E+10	3E+10	1E+10	1E+10	9E+09	1E+10	7E+09	1E+10	6E+09	4E+09	1E+10	6E+09	9E+09	5E+09	6E+09	4E+08	4E+08	2E+08	1E+08	2E+08	2E+08	2E+08	3E+08	2E+08	2E+08	2E+08	2E+08	2E+08	2E+08	2E+08	1E+08	1E+08	1E+08	2E+08	1E+08	1E+08				p07695	Epiplakin
DLGAP4 pT915	1E+08	1E+08	1E+08	2E+08	2E+08	7E+08	4E+08	2E+08	2E+08	2E+08	7E+08	4E+08	2E+08	2E+08	2E+08	3E+08	1E+08	2E+08	3E+08	2E+08	4E+08	9E+07	1E+08	2E+08	2E+08	5E+08	5E+07	5E+07	8E+07	7E+07	1E+08	4E+07	2E+07	7E+07	8E+07	1E+08	7E+07	4E+07	5E+07	9E+07	2E+08	7E+07	3E+07	8E+07	8E+07	1E+08	4E+07	6E+07	6E+07	6E+07	p20021	Disks large-associated protein 4
EPPK1 seq: 2713 - 2732 + Phospho (ST)	1E+09	3E+09	6E+09	6E+09	7E+09	4E+10	8E+09	5E+09	5E+09	4E+09	6E+10	1E+10	1E+10	1E+10	7E+09	1E+10	2E+09	6E+09	6E+09	4E+09	6E+09	2E+09	3E+09	5E+09	5E+09	1E+08	1E+07	1E+07	6E+07	4E+07	1E+07	9E+06	1E+08	9E+07	2E+07	3E+07	4E+07	8E+07	1E+08	2E+07	2E+07	4E+07	7E+07	3E+07	134692	1E+07	4E+07	9E+07	3E+07		p06771	Epiplakin
TOM1L2 seq: 470 - 494 + Phospho (ST)	7E+07	9E+07	1E+08	1E+08	1E+08	2E+08	1E+08	1E+08	8E+07	26736	4E+08	2E+08	2E+08	4E+08	2E+08	2E+08	1E+08	1E+08	2E+08	6E+07	1E+08	8E+07	1E+08	1E+08	7E+07	3E+08	8E+07	2E+08	1E+08	7E+07	1E+08	8E+07	9E+07	1E+08	1E+08	2E+08	1E+08	1E+08	9E+07	9E+07	2E+08	1E+08	1E+08	1E+08	1E+08	1E+08	1E+08	1E+08	2E+08	1E+08	p21170	TOM1-like protein 2
BUD13 pS222 pS226	3E+08	3E+08	3E+08	2E+08	1E+08	7E+08	4E+08	2E+08	2E+08	2E+08	6E+08	4E+08	3E+08	2E+08	3E+08	5E+08	4E+08	3E+08	3E+08	4E+08	4E+08	2E+08	2E+08	4E+08	3E+08	3E+09	1E+09	2E+09	1E+09	2E+09	4E+09	2E+09	8E+08	1E+09	2E+09	3E+09	2E+09	1E+09	7E+08	2E+09	3E+09	2E+09	1E+09	1E+09	2E+09	3E+09	2E+09	1E+09	9E+08	1E+09	p14856	BUD13 homolog
SFXN1 seq: 203 - 214 no mod	1E+09	9E+08	1E+09	1E+09	8E+08	2E+09	1E+09	1E+09	9E+08	5E+09	3E+09	2E+09	2E+09	2E+09	2E+09	7E+08	1E+09	1E+09	7E+08	2E+09	8E+08	1E+09	1E+09	1E+09	9E+08	1E+09	8E+08	1E+09	2E+09	1E+09	1E+09	9E+08	2E+09	1E+09	1E+09	1E+09	1E+09	9E+08	9E+08	3E+09	1E+09	1E+09	9E+08	9E+08	2E+09	1E+09	9E+08	9E+08			p01469	Sideroflexin-1
LMNA seq: 516 - 527 no mod	2E+07	4E+07	7E+07	1E+07	4E+07	1E+08	2E+07	34892	34104	26736	5E+08	6E+07	39754	5E+07	4E+07	2E+07	3E+07	34984	3E+07	4E+06	6E+07	4E+07	1E+07	19024	42741	3E+08	1E+08	1E+08	1E+08	8E+07	3E+08	1E+08	1E+08	1E+08	2E+08	5E+08	2E+08	2E+08	2E+08	2E+08	5E+08	2E+08	2E+08	1E+08	2E+08	5E+08	8E+07	1E+08	1E+08	1E+08	p19782	Prelamin-A/C
MPDZ pS230	4E+07	5E+07	7E+07	6E+07	7E+07	2E+08	5E+07	4E+07	5E+07	6E+07	3E+08	1E+08	7E+07	1E+08	1E+08	3E+07	5E+07	6E+07	6E+07	1E+08	5E+07	4E+07	6E+07	1E+08	1E+09	9E+08	3E+08	4E+08	4E+08	6E+08	4E+08	3E+08	4E+08	6E+08	5E+08	1E+09	6E+08	4E+08	5E+08	5E+08	1E+09	5E+08	4E+08	5E+08	5E+08	8E+08	7E+08	4E+08	6E+08	4E+08	p15246	Multiple PDZ domain protein
CEP55 seq: 422 - 442 + Phospho (ST)	9E+07	1E+08	9E+07	1E+08	9E+07	4E+08	2E+08	1E+08	1E+08	7E+07	8E+08	3E+08	2E+08	2E+08	2E+08	40867	6E+07	1E+08	1E+08	7E+07	2E+08	9E+07	7E+07	1E+08	8E+07	2E+09	4E+08	3E+08	6E+08	4E+08	7E+08	4E+08	3E+08	5E+08	5E+08	1E+09	5E+08	3E+08	4E+08	5E+08	1E+09	5E+08	3E+08	5E+08	5E+08	8E+08	3E+08	4E+08	5E+08	4E+08	p01834	Centrosomal protein of 55 kDa
TRIM28 pS473 pM482	1E+08	5E+07	1E+08	1E+08	9E+07	45779	1E+07	1E+08	1E+08	1E+08	1E+08	53206	2E+08	3E+08	3E+08	40867	2E+07	3E+07	19162	6E+07	39759	50401	1E+08	1E+08	1E+08	6E+07	4E+07	1E+08	8E+07	4E+07	45838	1E+08	1E+08	2E+08	1E+08	2E+08	3E+08	4E+08	3E+08	2E+08	100327	2E+07	38440	2E+07	1E+07	1E+08	2E+08	2E+08	1E+08	9E+07	p05773	Transcription intermediary factor 1-beta
HNRNPM seq: 457 - 471 + Phospho (ST)	38324	6E+07	9E+07	1E+08	1E+08	3E+08	1E+08	9E+07	1E+08	26736	3E+08	1E+08	1E+08	1E+08	2E+08	1E+08	4E+07	1E+08	1E+08	2E+08	1E+08	1E+08	5E+07	1E+08	1E+08	2E+08	9E+07	2E+08	5E+07	6E+07	6E+07	4E+07	1E+08	3E+07	7E+07	8E+07	8E+07	2E+07	4E+07	5E+07	7E+07	5E+07	7E+07	5E+07	8E+07	4E+07	2E+07	7E+07	5E+07	7E+07	p10100	Heterogeneous nuclear ribonucleoprotein M
UCK1 seq: 251 - 267 + Phospho (ST)	3E+07	2E+08	3E+08	3E+08	3E+08	8E+08	4E+08	3E+08	3E+08	2E+08	9E+08	4E+08	3E+08	4E+08	3E+08	4E+08	1E+08	3E+08	3E+08	2E+08	4E+08	2E+08	3E+08	2E+08	3E+08	5E+07	1E+08	1E+08	6E+07	6E+07	1E+08	7E+07	1E+08	4E+07	2E+08	2E+08	2E+08	1E+08	3E+08	9E+07	1E+08	1E+08	7E+07	2E+08	8E+07	9E+07	1E+08	6E+07			p02845	Uridine-cytidine kinase 1
UBA1 seq: 471 - 485 no mod	38324	6E+06	39656	13654	33854	1E+08	25552	34892	34104	26736	4E+08	5E+06	39754	40879	18798	3E+06	33072	34984	19162	28832	39759	50401	21442	19024	42741	68554	44396	34144	19500	29103	45838	31655	19257	24890	17227	44771	27262	25062	27887	36936	100327	64618	38440	41974	13188	134692	41467	25947	30272	25732	p00384	Ubiquitin-like modifier-activating enzyme 1
EHMT1 seq: 433 - 442 + Phospho (ST)	4E+07	3E+07	3E+07	4E+07	3E+07	2E+08	5E+07	4E+07	4E+07	4E+07	1E+08	5E+07	3E+07	3E+07	3E+07	8E+07	6E+07	4E+07	5E+07	2E+07	1E+08	4E+07	4E+07	5E+07	4E+07	9E+07	4E+07	3E+07	4E+07	2E+07	6E+07	3E+07	19257	4E+07	3E+07	8E+07	4E+07	2E+07	2E+07	4E+07	9E+07	5E+07	3E+07	3E+07	4E+07	6E+07	6E+07	3E+07	2E+07	4E+07	p02947	Histone-lysine N-methyltransferase EHMT1
KIF16B seq: 662 - 668 + Phospho (ST)	2E+08	2E+08	2E+08	1E+08	9E+07	7E+06	4E+08	2E+08	1E+08	7E+07	5E+08	2E+08	2E+08	1E+08	4E+08	1E+08	2E+08	2E+08	1E+08	3E+08	2E+08	2E+08	2E+08	1E+08	3E+07	44396	34144	2E+07	8E+06	1E+07	31655	19257	24890	17227	44771	4E+07	3E+07	2E+07	36936	100327	5E+07	5E+06	2E+07	2E+07	134692	3E+07	3E+07	2E+07	25732		p05902	Kinesin-like protein KIF16B
STK11IP seq: 396 - 410 + Phospho (ST)	1E+08	1E+08	1E+08	6E+07	3E+08	2E+08	6E+07	8E+07	9E+07	4E+08	2E+08	1E+08	1E+08	8E+07	1E+08	1E+08	2E+08	1E+08	8E+07	9E+07	1E+08	2E+08	1E+08	1E+08	5E+07	2E+08	7E+07	1E+08	4E+07	6E+07	2E+08	7E+06	4E+07	1E+08	7E+07	9E+07	2E+08	7E+07	6E+07	1E+08	2E+08	3E+08	4E+08	1E+08	2E+08	2E+08	41467	6E+07	1E+08	6E+07	p07579	Serine/threonine-protein kinase 11-interacting protein
EPB41L5 seq: 413 - 445 + Phospho (ST)	2E+08	4E+08	3E+08	3E+08	4E+08	9E+08	4E+08	2E+08	2E+08	2E+08	1E+09	6E+08	4E+08	6E+08	4E+08	4E+08	2E+08	3E+08	2E+08	2E+08	4E+08	2E+08	2E+08	2E+08	3E+08	5E+07	9E+07	9E+07	1E+08	5E+07	8E+07	7E+07	7E+07	1E+08	9E+07	2E+08	7E+07	1E+08	1E+08	2E+08	5E+07	38440	1E+08	1E+08	2E+08	41467	1E+08	8E+07	8E+07		p06318	Band 4.1-like protein 5
CALR seq: 186 - 207 no mod	1E+09	2E+09	2E+09	2E+09	2E+09	5E+09	2E+09	2E+09	2E+09	1E+09	8E+09	3E+09	3E+09	3E+09	2E+09	2E+09	1E+09	2E+09	2E+09	2E+09	2E+09	1E+09	2E+09	3E+09	2E+09	4E+09	6E+08	6E+08	2E+09	9E+08	2E+09	7E+08	5E+08	2E+09	2E+09	3E+09	9E+08	7E+08	2E+09	1E+09	3E+09	1E+09	9E+08	3E+09	2E+09	3E+09	5E+08	1E+09	2E+09	1E+09	p14403	Calreticulin
NCL seq: 524 - 537 no mod	1E+07	9E+06	2E+07	4E+07	7E+07	1E+08	3E+07	5E+07	4E+07	4E+07	3E+08	1E+08	5E+07	5E+07	2E+07	7E+07	33072	3E+07	8E+07	2E+07	3E+07	8E+06	2E+07	6E+07	3E+07	1E+08	44396	34144	3E+07	4E+07	45838	7E+06	19257	6E+07	1E+07	1E+08	1E+07	2E+07	4E+07	3E+07	5E+07	3E+07	9E+06	3E+07	7E+07	8E+07	41467	3E+07	6E+07	1E+07	p14941	Nucleolin
SPAG9 seq: 223 - 241 + Phospho (ST)	1E+08	2E+08	4E+08	2E+08	2E+08	1E+08	3E+08	4E+08	2E+08	2E+08	8E+08	5E+08	7E+08	7E+08	5E+08	1E+08	8E+07	4E+08	3E+08	2E+08	2E+08	2E+08	1E+08	3E+08	3E+08	5E+08	1E+08	2E+08	4E+08	2E+08	45838	31655	2E+08	3E+08	17227	4E+08	5E+08	3E+08	3E+08	2E+08	100327	3E+08	2E+08	3E+08	3E+08	134692	4E+08	2E+08	2E+08	2E+08	p16062	C-Jun-amino-terminal kinase-interacting protein 4

ALDH7A1 seq: 63 - 79 no mod	3E+08	3E+08	[illegible]	5E+07	p16148	Alpha-aminoadipic semialdehyde dehydrogenase
GNL1 seq: 45 - 61 + 2 Phospho (ST)	1E+08	2E+08	[illegible]	6E+07	p17823	Guanine nucleotide-binding protein-like 1
GAPDH seq: 146 - 162 no mod	2E+09	3E+09	[illegible]	4E+09	p14136	Glyceraldehyde-3-phosphate dehydrogenase
TNKS1BP1 seq: 556 - 608 + Phospho (ST)	4E+08	4E+08	[illegible]	25732	p16225	182 kDa tankyrase-1-binding protein
KRT79 pS191	3E+07	9E+07	[illegible]	1E+08	p16839	Keratin, type II cytoskeletal 79
USP20 seq: 121 - 142 + Phospho (ST)	9E+07	1E+08	[illegible]	3E+07	p19215	Ubiquitin carboxyl-terminal hydrolase 20
CLSPN pS1289	4E+07	4E+07	[illegible]	1E+08	p09620	Claspin
STARD6 pS57	2E+08	2E+08	[illegible]	6E+07	p01634	StAR-related lipid transfer protein 6
PDLIM7 seq: 196 - 233 + Phospho (ST)	3E+08	1E+08	[illegible]	2E+09	p02930	PDZ and LIM domain protein 7
MICALL2 seq: 644 - 657 + Phospho (ST)	1E+08	2E+08	[illegible]	1E+08	p02342	MICAL-like protein 2
PRKD1 pS742	38324	37149	[illegible]	25732	p03217	Serine/threonine-protein kinase D1
HNRNPAB pS242	5E+07	2E+07	[illegible]	9E+07	p16929	Heterogeneous nuclear ribonucleoprotein A/B
STRN seq: 283 - 302 no mod	3E+07	3E+07	[illegible]	2E+08	p17942	Striatin
DDX3X pS594	1E+08	1E+08	[illegible]	7E+07	p03740	ATP-dependent RNA helicase DDX3X
FMOD seq: 308 - 324 no mod	8E+08	2E+09	[illegible]	25732	p13865	Fibromodulin
SKA3 pS155	5E+08	3E+08	[illegible]	6E+08	p04463	Spindle and kinetochore-associated protein 3
STMN1 pS38	6E+09	3E+09	[illegible]	6E+09	p17094	Stathmin
HSPA1B seq: 540 - 550 no mod	1E+08	2E+08	[illegible]	1E+08	p09772	Heat shock 70 kDa protein 1B
CUX1 seq: 1214 - 1245 + Phospho (ST)	3E+08	3E+08	[illegible]	8E+08	p14600	Homeobox protein cut-like 1
LSR pS493	3E+08	4E+08	[illegible]	4E+08	p19674	Lipolysis-stimulated lipoprotein receptor
ZNF654 seq: 359 - 371 + Phospho (ST)	1E+08	1E+08	[illegible]	1E+08	p07803	Zinc finger protein 654
SEC16A seq: 768 - 802 + Phospho (ST)	2E+08	3E+08	[illegible]	25732	p13439	Protein transport protein Sec16A
SAP130 pS855 pT856	2E+08	6E+07	[illegible]	2E+08	p12657	Histone deacetylase complex subunit SAP130
PPP1R12A pS299	1E+09	9E+08	[illegible]	4E+08	p18679	Protein phosphatase 1 regulatory subunit 12A
IRF2BP2 seq: 389 - 411 + Phospho (ST)	2E+09	2E+09	[illegible]	1E+09	p13876	Interferon regulatory factor 2-binding protein 2
FAM110B pS95	2E+08	2E+08	[illegible]	2E+06	p20255	Protein FAM110B
BCLAF1 pS648	6E+09	6E+09	[illegible]	9E+09	p08428	Bcl-2-associated transcription factor 1
SSH3 pS649	38324	37149	[illegible]	25732	p08803	Protein phosphatase Slingshot homolog 3
CAMSAP1 pS575	1E+07	5E+07	[illegible]	2E+08	p04465	Calmodulin-regulated spectrin-associated protein 1
NOS1AP pS371	1E+07	1E+08	[illegible]	2E+07	p10783	Carboxyl-terminal PDZ ligand of neuronal nitric oxide synthase protein
SNW1 pS224 pM230	2E+07	3E+07	[illegible]	1E+08	p15465	SNW domain-containing protein 1
SCRIB pS1475	4E+08	6E+08	[illegible]	2E+08	p10850	Protein scribble homolog
LMNA pS406 pS407	1E+07	37149	[illegible]	25732	p15355	Prelamin-A/C
AIDA pS144	1E+08	1E+08	[illegible]	8E+07	p14091	Axin interactor, dorsalization-associated protein
TJP1 pS968	1E+08	3E+08	[illegible]	2E+08	p11989	Tight junction protein ZO-1
ANKS1A pS677	2E+08	2E+08	[illegible]	25732	p13937	Ankyrin repeat and SAM domain-containing protein 1A
NUP153 pS334	1E+08	3E+08	[illegible]	4E+08	p07261	Nuclear pore complex protein Nup153
LSR pS530	1E+09	1E+09	[illegible]	8E+08	p04134	Lipolysis-stimulated lipoprotein receptor
TXNL1 seq: 182 - 192 no mod	2E+08	1E+08	[illegible]	1E+08	p16515	Thioredoxin-like protein 1
TP53BP1 seq: 1017 - 1031 + Phospho (ST)	9E+07	9E+07	[illegible]	25732	p09592	Tumor suppressor p53-binding protein 1
MVD pS96	9E+08	1E+09	[illegible]	1E+09	p07015	Diphosphomevalonate decarboxylase
ETV6 pS271	38324	2E+06	[illegible]	4E+07	p01355	Transcription factor ETV6
LAD1 pT19	38324	1E+08	[illegible]	1E+08	p07934	Ladinin-1
RABEP1 seq: 405 - 420 + Phospho (ST)	7E+07	5E+07	[illegible]	5E+07	p19748	Rab GTPase-binding effector protein 1
LMNB1 pS284	7E+07	6E+07	[illegible]	7E+07	p10670	Lamin-B1
NCL seq: 334 - 342 no mod	3E+08	4E+08	[illegible]	6E+08	p09707	Nucleolin
CEP170B seq: 844 - 865 + Phospho (ST)	38324	2E+08	[illegible]	5E+08	p09825	Centrosomal protein of 170 kDa protein B
[illegible]: 1000 - 1029 + Gln->pyro-Glu (N-term Q); Phospho (ST)	1E+08	2E+08	[illegible]	4E+08	p08001	Unconventional myosin-Ie
ATAD2 pS342	5E+08	6E+08	[illegible]	1E+08	p10683	ATPase family AAA domain-containing protein 2
SZRD1 pS107	1E+09	9E+08	[illegible]	2E+09	p14038	SUZ domain-containing protein 1
PML pS527	5E+07	37149	[illegible]	3E+08	p19181	Protein PML
CEP170 seq: 230 - 251 + Phospho (ST)	7E+07	1E+08	[illegible]	2E+08	p03760	Centrosomal protein of 170 kDa
NSFL1C pS114	2E+09	4E+09	[illegible]	4E+09	p13032	NSFL1 cofactor p47
PEX1 seq: 1207 - 1224 + Phospho (ST)	3E+08	2E+08	[illegible]	1E+08	p00129	Peroxisome biogenesis factor 1
EPCAM seq: 34 - 44 no mod	1E+09	6E+09	[illegible]	1E+09	p12140	Epithelial cell adhesion molecule
AIM1 seq: 515 - 544 + Phospho (ST)	1E+08	1E+08	[illegible]	2E+08	p09956	Absent in melanoma 1 protein
OR2G3 seq: 303 - 309 + Phospho (ST)	38324	7E+07	[illegible]	25732	p12956	Olfactory receptor 2G3
KMT2A seq: 2938 - 2948 + Phospho (ST)	38324	2E+07	[illegible]	6E+07	p04321	Histone-lysine N-methyltransferase 2A
CIAPIN1 seq: 86 - 108 no mod	38324	37149	[illegible]	4E+08	p13998	Anamorsin
ILF3 pS812	2E+08	1E+08	[illegible]	8E+07	p16544	Interleukin enhancer-binding factor 3
DLGAP5 seq: 652 - 672 + Phospho (ST)	4E+07	8E+07	[illegible]	1E+08	p09673	Disks large-associated protein 5
TNKS1BP1 pS836	3E+09	1E+09	[illegible]	7E+08	p04274	182 kDa tankyrase-1-binding protein
SHANK2 pS877	1E+09	3E+09	[illegible]	4E+09	p09917	SH3 and multiple ankyrin repeat domains protein 2
MAP4 pS280	1E+09	3E+09	[illegible]	4E+09	p18493	Microtubule-associated protein 4
ATOX1 pS47	38324	1E+07	[illegible]	25732	p01716	Copper transport protein ATOX1
SRRM2 seq: 2736 - 2745 + 2 Phospho (ST)	2E+09	2E+09	[illegible]	4E+09	p07458	Serine/arginine repetitive matrix protein 2
FAM83H pS903	7E+08	6E+08	[illegible]	3E+08	p15194	Protein FAM83H
KLC1 seq: 521 - 531 + Phospho (ST)	1E+09	1E+09	[illegible]	2E+08	p04118	Kinesin light chain 1
RBMX seq: 332 - 339 no mod	5E+06	37149	[illegible]	2E+07	p06096	RNA-binding motif protein, X chromosome
DHX16 pS103 pS106	1E+08	2E+08	[illegible]	3E+08	p11427	Putative pre-mRNA-splicing factor ATP-dependent RNA helicase DHX16
PTPN13 seq: 344 - 358 + Phospho (ST)	4E+07	8E+07	[illegible]	3E+06	p00125	Tyrosine-protein phosphatase non-receptor type 13
MCM10 seq: 69 - 99 + Phospho (ST)	1E+08	3E+08	[illegible]	2E+08	p18898	Protein MCM10 homolog
HSP90B1 seq: 76 - 84 no mod	7E+08	2E+09	[illegible]	5E+08	p16869	Endoplasmin
SRRM2 pS952 pS954	4E+08	4E+08	[illegible]	5E+08	p03269	Serine/arginine repetitive matrix protein 2
MSL3 seq: 398 - 420 + 2 Phospho (ST)	1E+08	1E+08	[illegible]	1E+08	p03660	Male-specific lethal 3 homolog
ARFGAP2 pS146	3E+07	3E+07	[illegible]	1E+08	p14781	ADP-ribosylation factor GTPase-activating protein 2
SRP9 seq: 42 - 52 no mod	38324	7E+07	[illegible]	4E+07	p00741	Signal recognition particle 9 kDa protein
TGFB1I1 pS137	38324	37149	[illegible]	2E+07	p12623	Transforming growth factor beta-1-induced transcript 1 protein
ZNF280D pS545	2E+07	2E+08	[illegible]	9E+07	p19441	Zinc finger protein 280D
NPTX2 pS228	7E+06	1E+07	[illegible]	5E+07	p00824	Neuronal pentraxin-2
PDAP1 seq: 76 - 88 no mod	5E+06	9E+06	[illegible]	4E+07	p15048	28 kDa heat- and acid-stable phosphoprotein
SHROOM3 seq: 1067 - 1079 + Phospho (ST)	3E+08	4E+08	[illegible]	2E+08	p20938	Protein Shroom3
CTNND1 pS268 pS269	38324	37149	[illegible]	1E+07	p01485	Catenin delta-1
AHNAK seq: 3405 - 3414 + Phospho (ST)	5E+08	9E+08	[illegible]	1E+09	p09966	Neuroblast differentiation-associated protein AHNAK
ATAD1 pS322	1E+08	2E+08	[illegible]	2E+08	p16884	ATPase family AAA domain-containing protein 1
USO1 pS942	2E+08	2E+08	[illegible]	4E+08	p18609	General vesicular transport factor p115
MTSS1 seq: 613 - 619 + Phospho (ST)	5E+09	4E+09	[illegible]	4E+08	p02301	Metastasis suppressor protein 1
RALGAPA2 seq: 371 - 385 + Phospho (ST)	38324	2E+08	[illegible]	7E+07	p07106	Ral GTPase-activating protein subunit alpha-2
MAP1B pS1396	7E+06	1E+07	[illegible]	5E+07	p01167	Microtubule-associated protein 1B
MYH10 seq: 1738 - 1758 no mod	8E+07	1E+08	[illegible]	2E+08	p14486	Myosin-10
LPIN1 pS162	6E+07	7E+07	[illegible]	2E+08	p04244	Phosphatidate phosphatase LPIN1
CTTN pT401 pS405 pT411	2E+09	8E+08	[illegible]	7E+09	p02242	Src substrate cortactin
TRIP10 seq: 294 - 311 + 2 Phospho (ST)	4E+08	2E+08	[illegible]	1E+09	p19926	Cdc42-interacting protein 4
SRRM1 pT572 pT574	1E+10	1E+10	[illegible]	7E+09	p06709	Serine/arginine repetitive matrix protein 1
EEA1 pS356	2E+07	1E+07	[illegible]	1E+08	p10837	Early endosome antigen 1
VCP seq: 544 - 560 no mod	38324	5E+06	[illegible]	25732	p15525	Transitional endoplasmic reticulum ATPase
TNRC18 seq: 1230 - 1258 + Phospho (ST)	2E+06	6E+06	[illegible]	25732	p01661	Trinucleotide repeat-containing gene 18 protein
ARHGEF6 seq: 648 - 661 + Phospho (ST)	2E+07	1E+07	[illegible]	3E+06	p12524	Rho guanine nucleotide exchange factor 6
SRA1 seq: 63 - 83 + Oxidation (M); Phospho (ST)	9E+07	1E+08	[illegible]	3E+08	p01029	Steroid receptor RNA activator 1
PPP1R1B seq: 41 - 56 + 2 Phospho (ST)	5E+07	7E+07	[illegible]	25732	p10816	Protein phosphatase 1 regulatory subunit 1B
L3MBTL2 seq: 60 - 78 + 2 Phospho (ST)	6E+07	3E+07	[illegible]	1E+08	p17970	Lethal(3)malignant brain tumor-like protein 2
MYO18A seq: 1065 - 1093 + 2 Phospho (ST)	6E+07	9E+07	[illegible]	25732	p06428	Unconventional myosin-XVIIIa
EIF3E pS399	2E+08	1E+08	[illegible]	2E+08	p11762	Eukaryotic translation initiation factor 3 subunit E
PKP2 pS151	1E+08	1E+08	[illegible]	9E+07	p07187	Plakophilin-2
MCCC2 seq: 362 - 382 no mod	38324	8E+07	[illegible]	3E+07	p14306	Methylcrotonoyl-CoA carboxylase beta chain, mitochondrial
PPP1R12A pS299	3E+08	2E+08	[illegible]	7E+07	p18678	Protein phosphatase 1 regulatory subunit 12A
ATRX seq: 1519 - 1536 + Phospho (ST)	4E+08	2E+08	[illegible]	5E+07	p16981	Transcriptional regulator ATRX
KIAA1671 pS1800	1E+08	2E+08	[illegible]	6E+07	p12622	Uncharacterized protein KIAA1671
RPS27 pS27	3E+07	3E+07	[illegible]	1E+08	p10366	40S ribosomal protein S27
RALGPS2 seq: 293 - 314 + Phospho (ST)	1E+08	6E+07	[illegible]	1E+08	p06370	Ras-specific guanine nucleotide-releasing factor RalGPS2
CTTN pT401 pS405 pT411	4E+08	2E+08	[illegible]	1E+09	p02241	Src substrate cortactin
SFRP2 seq: 286 - 291 + Phospho (ST)	1E+10	6E+09	[illegible]	7E+09	p13669	Secreted frizzled-related protein 2
HSPB1 seq: 172 - 188 + Phospho (ST)	8E+07	6E+07	[illegible]	25732	p12149	Heat shock protein beta-1
JUND pS255	2E+08	2E+08	[illegible]	2E+09	p12308	Transcription factor jun-D
G6PD seq: 105 - 120 no mod	7E+08	5E+08	[illegible]	1E+08	p09040	Glucose-6-phosphate 1-dehydrogenase
PRKAR2B pS114	1E+08	2E+08	[illegible]	25732	p07548	cAMP-dependent protein kinase type II-beta regulatory subunit
CBX3 seq: 172 - 183 + Phospho (ST)	2E+08	2E+08	[illegible]	5E+08	p10459	Chromobox protein homolog 3
KAT7 seq: 117 - 155 + 3 Phospho (ST)	1E+08	37149	[illegible]	25732	p09037	Histone acetyltransferase KAT7
SP100 pS362	38324	37149	[illegible]	3E+08	p06004	Nuclear autoantigen Sp-100
TBC1D10B seq: 648 - 666 + Phospho (ST)	1E+07	1E+08	[illegible]	5E+07	p06805	TBC1 domain family member 10B
CXorf67 pS397	6E+07	8E+07	[illegible]	25732	p19536	Uncharacterized protein CXorf67
PNPO pS241	3E+08	3E+08	[illegible]	2E+08	p15668	Pyridoxine-5'-phosphate oxidase
SET pS7	1E+09	1E+09	[illegible]	4E+09	p06790	Protein SET
RBM17 seq: 207 - 241 + Phospho (Y)	4E+09	8E+09	[illegible]	8E+09	p21123	Splicing factor 45
SAMD4B seq: 260 - 291 + Phospho (ST)	4E+08	3E+08	[illegible]	7E+08	p21159	Protein Smaug homolog 2
SRRM1 pS738 pS740	2E+08	1E+08	[illegible]	2E+08	p21025	Serine/arginine repetitive matrix protein 1
THOC2 pS1393	3E+07	6E+07	[illegible]	2E+07	p02298	THO complex subunit 2
SRRM2 pS778	2E+08	2E+08	[illegible]	6E+08	p05052	Serine/arginine repetitive matrix protein 2
ANLN seq: 95 - 127 + Phospho (ST)	7E+08	1E+09	[illegible]	2E+09	p06132	Actin-binding protein anillin
PTRF seq: 61 - 78 no mod	38324	37149	[illegible]	25732	p14174	Polymerase I and transcript release factor
RBMX seq: 10 - 22 no mod	1E+08	2E+08	[illegible]	1E+08	p11893	RNA-binding motif protein, X chromosome
PHLDB2 seq: 196 - 206 + Phospho (ST)	2E+08	2E+08	[illegible]	6E+08	p05797	Pleckstrin homology-like domain family B member 2
RPRD1B pS134	3E+09	4E+09	[illegible]	4E+09	p10729	Regulation of nuclear pre-mRNA domain-containing protein 1B
SNAP23 pS110	3E+08	3E+08	[illegible]	8E+07	p01989	Synaptosomal-associated protein 23
MAP1A pS526 pS527	2E+06	4E+07	[illegible]	25732	p17377	Microtubule-associated protein 1A
ACTG1 seq: 292 - 312 no mod	1E+08	2E+08	[illegible]	2E+08	p18505	Actin, cytoplasmic 2
EPS15L1 pS229	3E+09	4E+09	[illegible]	4E+09	p01966	Epidermal growth factor receptor substrate 15-like 1
PHIP seq: 1313 - 1323 + Phospho (ST)	4E+07	7E+07	[illegible]	2E+07	p19746	PH-interacting protein
ACTB pM305	6E+07	1E+08	[illegible]	1E+08	p18503	Actin, cytoplasmic 1
MTDH pS298	38324	1E+08	[illegible]	3E+08	p10643	Protein LYRIC
CAPZB seq: 95 - 108 no mod	38324	5E+07	[illegible]	9E+07	p12990	F-actin-capping protein subunit beta
THRAP3 pS248 pS253	3E+08	2E+08	[illegible]	2E+08	p17203	Thyroid hormone receptor-associated protein 3
SEC24C seq: 320 - 340 no mod	5E+08	1E+09	[illegible]	1E+09	p07191	Protein transport protein Sec24C
RAB1B seq: 141 - 156 no mod	1E+08	2E+08	[illegible]	1E+08	p17811	Ras-related protein Rab-1B
MAP4 seq: 825 - 832 + Phospho (ST)	4E+08	8E+08	[illegible]	4E+08	p04361	Microtubule-associated protein 4
KRT18 seq: 359 - 370 no mod	4E+07	1E+08	[illegible]	2E+07	p08836	Keratin, type I cytoskeletal 18
FAM21A pS284	2E+08	6E+08	[illegible]	6E+08	p04082	WASH complex subunit FAM21A
TICRR pS1078	38324	3E+07	[illegible]	25732	p05164	Treslin
SOWAHC seq: 207 - 217 + Phospho (ST)	1E+08	1E+08	[illegible]	4E+07	p18514	Ankyrin repeat domain-containing protein SOWAHC
SEC16A pS391	3E+08	2E+08	[illegible]	7E+07	p08530	Protein transport protein Sec16A
UTP14A seq: 556 - 573 + Phospho (ST)	38324	37149	[illegible]	5E+07	p17275	U3 small nucleolar RNA-associated protein 14 homolog A
FILIP1L pS791	8E+07	8E+07	[illegible]	1E+09	p07258	Filamin A-interacting protein 1-like
TFPI2 seq: 182 - 201 no mod	8E+07	2E+08	[illegible]	3E+07	p02992	Tissue factor pathway inhibitor 2
ZNF185 seq: 496 - 528 + Phospho (ST)	3E+08	2E+08	[illegible]	4E+08	p15984	Zinc finger protein 185
LMNA seq: 458 - 470 no mod	6E+07	3E+07	[illegible]	9E+07	p04853	Prelamin-A/C
LARP4B seq: 509 - 545 + 2 Phospho (ST)	2E+08	5E+08	[illegible]	5E+09	p16410	La-related protein 4B
SVIL pS221	2E+08	2E+08	[illegible]	7E+08	p08856	Supervillin
AKAP2 pS393	38324	4E+07	[illegible]	3E+09	p15391	A-kinase anchor protein 2
WBP11 pS237	6E+07	4E+07	[illegible]	2E+07	p18903	WW domain-binding protein 11
HNRNPK pS379	9E+08	7E+08	[illegible]	3E+08	p15136	Heterogeneous nuclear ribonucleoprotein K
CCT5 seq: 133 - 142 no mod	4E+08	3E+08	[illegible]	3E+07	p14526	T-complex protein 1 subunit epsilon
MAP7D1 seq: 111 - 129 + 2 Phospho (ST)	4E+07	1E+08	[illegible]	2E+08	p06582	MAP7 domain-containing protein 1
ZMYND8 pS547	3E+08	3E+08	[illegible]	4E+08	p17404	Protein kinase C-binding protein 1
NFKB1 pS907	2E+08	2E+08	[illegible]	3E+08	p02005	Nuclear factor NF-kappa-B p105 subunit
HMGB2 seq: 129 - 139 no mod	6E+09	4E+09	[illegible]	5E+09	p11796	High mobility group protein B2
CTTN pT401 pS405	1E+09	9E+08	[illegible]	4E+09	p20352	Src substrate cortactin
TPI1 seq: 213 - 225 no mod	2E+09	2E+09	[illegible]	1E+09	p03004	Triosephosphate isomerase
CTTN seq: 397 - 428 + Phospho (ST); Phospho (Y)	1E+09	9E+08	[illegible]	4E+09	p20345	Src substrate cortactin
CNOT2 pS165	4E+08	3E+08	[illegible]	5E+08	p02457	CCR4-NOT transcription complex subunit 2
KIF5B seq: 645 - 656 no mod	38324	6E+07	[illegible]	4E+07	p04957	Kinesin-1 heavy chain
INCENP seq: 127 - 154 + 2 Phospho (ST)	4E+07	1E+08	[illegible]	2E+08	p21214	Inner centromere protein
PHF3 pS1642	8E+07	1E+08	[illegible]	5E+07	p04467	PHD finger protein 3
PGRMC1 pS181	38324	2E+08	[illegible]	1E+08	p17763	Membrane-associated progesterone receptor component 1
SLC9A4 seq: 583 - 593 no mod	38324	6E+06	[illegible]	25732	p07101	Sodium/hydrogen exchanger 4
LMNA pS458 pM464	5E+07	6E+07	[illegible]	1E+08	p09495	Prelamin-A/C
PAWR seq: 32 - 100 no mod	38324	3E+08	[illegible]	3E+08	p20379	PRKC apoptosis WT1 regulator protein
GPRC5A seq: 299 - 333 + Phospho (ST)	38324	37149	[illegible]	3E+08	p19101	Retinoic acid-induced protein 3
MAPT seq: 543 - 557 + 2 Phospho (ST)	4E+09	3E+09	[illegible]	1E+09	p01726	Microtubule-associated protein tau
RAN seq: 143 - 152 no mod	3E+08	4E+08	[illegible]	3E+08	p09418	GTP-binding nuclear protein Ran
ETV3 pS159	2E+08	5E+08	[illegible]	2E+09	p16708	ETS translocation variant 3
SRRM2 seq: 1316 - 1334 + Phospho (ST)	5E+08	5E+08	[illegible]	9E+08	p17396	Serine/arginine repetitive matrix protein 2
SOX13 seq: 315 - 330 + Phospho (ST)	1E+08	4E+08	[illegible]	3E+07	p09877	Transcription factor SOX-13
AHNAK pS511	1E+09	2E+09	[illegible]	6E+09	p13695	Neuroblast differentiation-associated protein AHNAK
HCFC1 seq: 595 - 608 + Phospho (ST)	2E+08	2E+08	[illegible]	4E+08	p01749	Host cell factor 1
NUMA1 pS1862	5E+08	1E+09	[illegible]	1E+09	p11691	Nuclear mitotic apparatus protein 1
ANXA2 seq: 179 - 196 no mod	6E+07	1E+08	[illegible]	4E+08	p07645	Annexin A2
ATRX pS677	2E+08	2E+08	[illegible]	2E+08	p06831	Transcriptional regulator ATRX
LDHA seq: 306 - 315 no mod	2E+08	2E+08	[illegible]	8E+07	p00706	L-lactate dehydrogenase A chain
ARHGAP35 seq: 973 - 997 + Phospho (ST)	1E+09	6E+08	[illegible]	3E+07	p20565	Rho GTPase-activating protein 35
UQCRC1 seq: 379 - 390 no mod	8E+08	8E+08	[illegible]	8E+07	p12114	Cytochrome b-c1 complex subunit 1, mitochondrial
AHNAK pT4766	8E+08	1E+09	[illegible]	7E+08	p01680	Neuroblast differentiation-associated protein AHNAK
AIM1 seq: 515 - 544 + Phospho (ST)	5E+08	5E+08	[illegible]	1E+09	p09957	Absent in melanoma 1 protein
PCYT1A pS331	1E+09	1E+09	[illegible]	1E+09	p04382	Choline-phosphate cytidylyltransferase A
SNTB2 seq: 384 - 405 + Phospho (ST)	38324	8E+07	[illegible]	4E+08	p10418	Beta-2-syntrophin
SZRD1 pS107	2E+09	2E+09	[illegible]	5E+09	p14039	SUZ domain-containing protein 1
CENPF pS3094	1E+08	1E+08	[illegible]	8E+07	p02129	Centromere protein F
FOSL2 seq: 15 - 35 + Phospho (ST)	1E+08	1E+08	[illegible]	8E+08	p15173	Fos-related antigen 2
KRT19 seq: 265 - 274 no mod	3E+07	4E+07	[illegible]	2E+07	p13356	Keratin, type I cytoskeletal 19
TJP1 pS968	4E+07	37149	[illegible]	2E+08	p11990	Tight junction protein ZO-1
LASP1 pT68	5E+08	5E+08	[illegible]	5E+08	p07914	LIM and SH3 domain protein 1
DIDO1 pS1456	6E+08	1E+09	[illegible]	1E+09	p06996	Death-inducer obliterator 1
PDIA4 seq: 145 - 155 no mod	2E+08	2E+08	[illegible]	7E+06	p13171	Protein disulfide-isomerase A4
LMO7 pS867	4E+08	3E+08	[illegible]	3E+09	p00671	LIM domain only protein 7
MADD seq: 1236 - 1255 + Phospho (ST)	3E+08	2E+08	[illegible]	7E+07	p15086	MAP kinase-activating death domain protein
SAP130 pS465	1E+08	3E+08	[illegible]	2E+08	p19543	Histone deacetylase complex subunit SAP130
BOD1L1 seq: 1515 - 1534 + Phospho (ST)	2E+08	2E+08	[illegible]	2E+08	p02069	Biorientation of chromosomes in cell division protein 1-like 1
NDE1 pS306	8E+08	8E+08	[illegible]	9E+07	p06841	Nuclear distribution protein nudE homolog 1
HSPA8 seq: 424 - 447 no mod	38324	9E+07	[illegible]	1E+08	p07775	Heat shock cognate 71 kDa protein
FUBP1 seq: 322 - 331 no mod	1E+08	1E+08	[illegible]	3E+07	p14490	Far upstream element-binding protein 1
ETFA seq: 188 - 203 no mod	3E+07	8E+07	[illegible]	4E+07	p19470	Electron transfer flavoprotein subunit alpha, mitochondrial

WDR62 pS1123	3E+07 4E+07 4E+07 13654 4E+07 1E+08 4E+07 5E+07 3E+07 26736 3E+08 1E+08 9E+07 1E+08 7E+07 7E+07 2E+07 3E+07 2E+07 28832 8E+07 4E+07 2E+07 4E+07 5E+07 2E+08 1E+08 [illegible]	p19338	WD repeat-containing protein 62
ZBTB7A pS549	38324 2E+08 1E+08 1E+08 1E+08 8E+08 3E+08 1E+08 1E+08 2E+08 5E+08 3E+08 2E+08 1E+08 2E+08 4E+08 3E+08 2E+08 2E+08 2E+08 3E+08 1E+08 1E+08 8E+07 2E+08 2E+08 44396 [illegible]	p14835	Zinc finger and BTB domain-containing protein 7A
ARHGAP1 seq: 49 - 59 + Phospho (ST)	1E+09 8E+08 1E+09 6E+08 5E+08 6E+07 9E+08 8E+08 9E+08 6E+08 56234 2E+09 2E+09 1E+09 1E+09 40867 8E+08 1E+09 8E+08 6E+08 1E+09 7E+08 1E+09 7E+08 8E+08 6E+08 2E+08 [illegible]	p03669	Rho GTPase-activating protein 1
PRKD1 seq: 203 - 216 + 2 Phospho (ST)	2E+07 3E+07 2E+07 13654 1E+07 45779 5E+07 2E+07 2E+07 2E+07 56234 3E+07 2E+07 2E+07 2E+07 3E+07 2E+07 2E+07 19162 9E+06 3E+07 2E+07 2E+07 2E+07 42741 1E+08 3E+07 [illegible]	p07105	Serine/threonine-protein kinase D1
RBM33 pS205	7E+07 1E+08 1E+08 1E+08 7E+07 45779 2E+08 8E+07 9E+07 1E+08 56234 2E+08 2E+08 1E+08 1E+08 2E+08 2E+08 2E+08 1E+08 1E+08 1E+08 2E+08 2E+08 1E+08 1E+08 3E+08 2E+08 [illegible]	p18746	RNA-binding protein 33
DDX54 pS782	2E+08 2E+08 2E+08 2E+08 1E+08 2E+08 2E+08 1E+08 2E+08 2E+08 2E+08 2E+08 3E+08 2E+08 2E+08 3E+08 3E+08 3E+08 3E+08 2E+08 2E+08 3E+08 2E+08 1E+08 4E+08 9E+08 5E+08 [illegible]	p14443	ATP-dependent RNA helicase DDX54
SURF6 pS74	2E+07 2E+07 1E+07 1E+07 2E+07 1E+08 3E+07 2E+07 2E+07 3E+07 5E+07 3E+07 9E+06 1E+07 2E+07 8E+07 3E+07 3E+07 2E+07 4E+07 8E+07 3E+07 2E+07 2E+07 5E+07 2E+08 8E+07 [illegible]	p05229	Surfeit locus protein 6
RAD23B seq: 393 - 409 no mod	1E+09 3E+09 2E+09 2E+09 2E+09 5E+09 2E+09 2E+09 2E+09 2E+09 6E+09 2E+09 2E+09 2E+09 2E+09 2E+09 2E+09 2E+09 2E+09 2E+09 2E+09 2E+09 3E+09 2E+09 3E+09 2E+09 6E+08 [illegible]	p09672	UV excision repair protein RAD23 homolog B
MAP1B pS1265	3E+07 8E+07 1E+07 3E+07 9E+07 5E+08 1E+08 3E+07 4E+07 4E+07 4E+08 8E+07 7E+07 5E+07 6E+07 1E+08 3E+07 34984 4E+07 5E+07 2E+08 50401 3E+07 3E+07 8E+07 1E+09 3E+08 [illegible]	p04412	Microtubule-associated protein 1B
THRAP3 pT874	3E+07 3E+07 2E+07 4E+07 2E+07 2E+08 5E+07 2E+07 3E+07 4E+07 2E+08 4E+07 1E+07 3E+07 3E+07 1E+08 3E+07 6E+07 8E+07 4E+07 7E+07 3E+07 3E+07 5E+07 6E+07 4E+08 8E+07 [illegible]	p09199	Thyroid hormone receptor-associated protein 3
KIF23 seq: 910 - 932 + Phospho (ST)	1E+08 2E+08 2E+08 2E+08 2E+08 3E+08 3E+08 2E+08 2E+08 2E+08 3E+08 3E+08 3E+08 3E+08 3E+08 1E+08 1E+08 2E+08 1E+08 2E+08 2E+08 1E+08 2E+08 2E+08 3E+08 1E+09 3E+08 [illegible]	p06564	Kinesin-like protein KIF23
TEX2 seq: 746 - 760 + Phospho (ST)	9E+07 1E+08 2E+08 1E+08 1E+08 3E+08 3E+08 2E+08 2E+08 1E+08 4E+08 4E+08 4E+08 4E+08 3E+08 1E+08 9E+07 2E+08 1E+08 8E+07 3E+08 1E+08 2E+08 1E+08 1E+08 3E+07 1E+07 [illegible]	p03700	Testis-expressed sequence 2 protein
LMO7 seq: 1195 - 1222 + Phospho (ST)	38324 37149 39656 13654 33854 45779 25552 34892 34104 26736 56234 53206 39754 40879 18798 40867 33072 34984 19162 28832 39759 50401 21442 19024 42741 1E+09 7E+07 [illegible]	p06720	LIM domain only protein 7
PKM seq: 295 - 305 no mod	1E+09 1E+09 2E+09 7E+08 1E+09 3E+09 1E+09 1E+09 9E+08 6E+08 1E+10 9E+08 2E+09 8E+08 7E+08 2E+09 7E+08 7E+08 2E+09 4E+08 2E+09 7E+08 7E+08 8E+08 7E+08 2E+09 5E+08 [illegible]	p16216	Pyruvate kinase PKM
TACC3 pS71	3E+08 3E+08 2E+08 2E+08 2E+08 1E+09 4E+08 2E+08 4E+08 2E+08 2E+09 5E+08 3E+08 4E+08 4E+08 5E+08 3E+08 3E+08 4E+08 2E+08 5E+08 2E+08 3E+08 3E+08 3E+08 8E+08 3E+08 [illegible]	p13982	Transforming acidic coiled-coil-containing protein 3
SRSF11 pS207	7E+08 8E+08 1E+09 8E+08 7E+08 45779 1E+09 1E+09 1E+09 7E+08 56234 1E+09 1E+09 1E+09 1E+09 40867 1E+09 2E+09 1E+09 8E+08 39759 9E+08 1E+09 1E+09 1E+09 2E+09 5E+08 [illegible]	p11187	Serine/arginine-rich splicing factor 11
PRPF4B seq: 427 - 440 + 2 Phospho (ST)	3E+09 1E+09 3E+09 2E+09 1E+09 5E+09 3E+09 2E+09 2E+09 2E+09 4E+09 3E+09 2E+09 2E+09 1E+09 4E+09 3E+09 4E+09 3E+09 3E+09 3E+09 2E+09 2E+09 2E+09 1E+09 2E+09 1E+09 [illegible]	p05436	Serine/threonine-protein kinase PRP4 homolog
ACADSB seq: 409 - 426 no mod	38324 4E+07 5E+07 3E+07 33854 45779 7E+07 34892 3E+07 4E+07 56234 4E+07 5E+07 40879 5E+07 7E+07 6E+07 8E+07 6E+07 3E+07 7E+07 5E+07 21442 5E+07 7E+07 68554 44396 [illegible]	p14224	Short/branched chain specific acyl-CoA dehydrogenase, mitochondrial
seq: 333 - 348 + Gln->pyro-Glu (N-term Q); Phospho (ST)	38324 37149 3E+07 13654 33854 6E+07 25552 4E+07 34104 26736 56234 53206 3E+07 40879 18798 40867 3E+06 5E+07 9E+06 3E+07 39759 50401 7E+07 19024 42741 4E+07 1E+08 [illegible]	p08533	Ethanolamine-phosphate cytidylyltransferase
PI4KB seq: 275 - 291 + Phospho (ST)	2E+07 3E+07 5E+07 3E+07 2E+07 1E+08 7E+07 3E+07 5E+07 1E+07 3E+08 8E+07 9E+07 9E+07 4E+07 6E+07 2E+07 3E+07 19162 4E+06 9E+07 2E+07 3E+07 4E+07 3E+07 8E+08 3E+08 [illegible]	p05379	Phosphatidylinositol 4-kinase beta
ERCC5 pS384	3E+08 2E+08 2E+08 2E+08 2E+08 1E+09 3E+08 2E+08 2E+08 2E+08 6E+08 3E+08 3E+08 2E+08 2E+08 5E+08 3E+08 3E+08 2E+08 2E+08 4E+08 2E+08 3E+08 2E+08 2E+08 2E+08 7E+07 [illegible]	p09759	DNA repair protein complementing XP-G cells
RPS24 seq: 11 - 16 no mod	38324 37149 39656 13654 727438 45779 25552 34892 34104 338189 1E+08 53206 397536 40879 18798 40867 2E+06 34984 19162 593767 39759 3E+06 21442 19024 42741 2E+07 3E+06 [illegible]	p13239	40S ribosomal protein S24
CIC seq: 1359 - 1384 + 2 Phospho (ST)	2E+09 2E+09 4E+09 3E+09 3E+09 45779 4E+09 6E+09 5E+09 4E+09 56234 4E+09 5E+09 4E+09 5E+09 40867 5E+09 5E+09 4E+09 4E+09 39759 5E+09 4E+09 4E+09 4E+09 68554 4E+09 [illegible]	p16872	Protein capicua homolog
TNKS1BP1 seq: 1380 - 1397 + Phospho (ST)	2E+08 2E+08 7E+07 3E+08 2E+08 5E+08 4E+08 5E+07 2E+08 2E+08 2E+09 6E+08 2E+08 6E+08 2E+08 5E+08 3E+07 1E+08 3E+08 1E+08 4E+08 2E+08 9E+06 2E+08 2E+08 2E+09 3E+08 [illegible]	p14716	182 kDa tankyrase-1-binding protein
PXN seq: 148 - 193 + Phospho (ST)	38324 37149 6E+08 9E+08 1E+09 45779 1E+08 6E+08 9E+08 4E+08 56234 6E+08 9E+08 9E+08 1E+09 40867 33072 8E+08 1E+09 5E+08 39759 1E+08 7E+08 1E+09 1E+09 7E+08 1E+08 [illegible]	p11359	Paxillin
MTM1 seq: 582 - 603 + Phospho (ST)	[illegible]	p10828	Myotubularin
SH2D3A pS125	[illegible]	p05858	SH2 domain-containing protein 3A
GAB1 seq: 645 - 666 + Phospho (Y)	[illegible]	p03882	GRB2-associated-binding protein 1
NDE1 seq: 63 - 70 no mod	[illegible]	p18578	Nuclear distribution protein nudE homolog 1
HN1L seq: 68 - 81 + Phospho (ST)	[illegible]	p15281	Hematological and neurological expressed 1-like protein
FGFR4 pS573	[illegible]	p06889	Fibroblast growth factor receptor 4
SNX6 seq: 316 - 325 no mod	[illegible]	p04936	Sorting nexin-6
MPRIP seq: 991 - 1000 + Phospho (ST)	[illegible]	p05364	Myosin phosphatase Rho-interacting protein
PDLIM7 pS217	[illegible]	p02931	PDZ and LIM domain protein 7
SRRM1 pS389 pS391 pS393	[illegible]	p07093	Serine/arginine repetitive matrix protein 1
MRPS36 seq: 58 - 78 + Phospho (ST)	[illegible]	p15260	28S ribosomal protein S36, mitochondrial
TBC1D4 pS262	[illegible]	p15528	TBC1 domain family member 4
CAMSAP1 pS1080	[illegible]	p01334	Calmodulin-regulated spectrin-associated protein 1
TUBA1A seq: 327 - 336 no mod	[illegible]	p18104	Tubulin alpha-1A chain
SNRNP200 pS225	[illegible]	p17858	U5 small nuclear ribonucleoprotein 200 kDa helicase
NUP210 seq: 1836 - 1846 + Phospho (ST)	[illegible]	p18647	Nuclear pore membrane glycoprotein 210
SGOL1 pS507	[illegible]	p07399	Shugoshin-like 1
UBA1 pS810	[illegible]	p14200	Ubiquitin-like modifier-activating enzyme 1
MET seq: 988 - 1004 + Oxidation (M); Phospho (ST)	[illegible]	p03261	Hepatocyte growth factor receptor
ADGB pT1333	[illegible]	p03082	Androglobin
VASP seq: 36 - 48 + Phospho (Y)	[illegible]	p00947	Vasodilator-stimulated phosphoprotein
VIM seq: 171 - 184 no mod	[illegible]	p01586	Vimentin
EIF3J pT109	[illegible]	p01134	Eukaryotic translation initiation factor 3 subunit J
SOX5 seq: 97 - 118 + Phospho (ST)	[illegible]	p01098	Transcription factor SOX-5
SIN3A pT266	[illegible]	p00835	Paired amphipathic helix protein Sin3a
NAA10 pS186	[illegible]	p15566	N-alpha-acetyltransferase 10
SLK seq: 187 - 208 + Phospho (Y)	[illegible]	p07516	STE20-like serine/threonine-protein kinase
CTNNA2 seq: 652 - 670 + Phospho (ST)	[illegible]	p03955	Catenin alpha-2
MYH14 seq: 1755 - 1775 no mod	[illegible]	p11712	Myosin-14
TXN seq: 9 - 21 no mod	[illegible]	p03091	Thioredoxin
PPP1R12A pS422	[illegible]	p16527	Protein phosphatase 1 regulatory subunit 12A
NOC2L pS49 pS56	[illegible]	p18013	Nucleolar complex protein 2 homolog
LMO7 pS1026	[illegible]	p19356	LIM domain only protein 7
HSPA1L seq: 37 - 49 no mod	[illegible]	p02035	Heat shock 70 kDa protein 1-like
NUP35 seq: 295 - 310 + Phospho (ST)	[illegible]	p19444	Nucleoporin NUP53
ATRX seq: 702 - 718 + Phospho (ST)	[illegible]	p09088	Transcriptional regulator ATRX
PRDX1 seq: 28 - 35 no mod	[illegible]	p18722	Peroxiredoxin-1
HSPA1L seq: 501 - 507 no mod	[illegible]	p13609	Heat shock 70 kDa protein 1-like
RPS6KB1 seq: 444 - 459 + 2 Phospho (ST)	[illegible]	p02294	Ribosomal protein S6 kinase beta-1
FAM21A seq: 1169 - 1194 + Phospho (ST)	[illegible]	p04532	WASH complex subunit FAM21A
KRT8 pS24	[illegible]	p03112	Keratin, type II cytoskeletal 8
KLRG2 seq: 203 - 220 + Phospho (ST)	[illegible]	p19563	Killer cell lectin-like receptor subfamily G member 2
UBA1 seq: 658 - 671 + Gln->pyro-Glu (N-term Q)	[illegible]	p08152	Ubiquitin-like modifier-activating enzyme 1
BOD1L1 seq: 657 - 674 + Phospho (ST)	[illegible]	p06480	Biorientation of chromosomes in cell division protein 1-like 1
TRIM28 seq: 471 - 483 + Oxidation (M); Phospho (ST)	[illegible]	p04057	Transcription intermediary factor 1-beta
TIAM1 pS358	[illegible]	p04856	T-lymphoma invasion and metastasis-inducing protein 1
RAPH1 seq: 54 - 68 + Phospho (ST)	[illegible]	p04544	Ras-associated and pleckstrin homology domains-containing protein 1
TXNRD1 seq: 419 - 443 no mod	[illegible]	p00655	Thioredoxin reductase 1, cytoplasmic
RCHY1 pS257	[illegible]	p07250	RING finger and CHY zinc finger domain-containing protein 1
TSC1 seq: 385 - 420 + Phospho (ST)	[illegible]	p15081	Hamartin
PDLIM1 pS90	[illegible]	p00549	PDZ and LIM domain protein 1
MATR3 seq: 596 - 608 + Phospho (ST)	[illegible]	p03134	Matrin-3
PNN pS66	[illegible]	p16099	Pinin
MATR3 pS188	[illegible]	p07519	Matrin-3
KRT8 seq: 150 - 158 no mod	[illegible]	p08391	Keratin, type II cytoskeletal 8
MDP1 seq: 160 - 176 + Phospho (ST)	[illegible]	p19742	Magnesium-dependent phosphatase 1
AIM1 pS141	[illegible]	p06459	Absent in melanoma 1 protein
KDM2A pS558	[illegible]	p10513	Lysine-specific demethylase 2A
EPB41L1 seq: 537 - 548 + 2 Phospho (ST)	[illegible]	p07158	Band 4.1-like protein 1
KIAA1217 seq: 351 - 370 + Phospho (ST)	[illegible]	p13657	Sickle tail protein homolog
BCLAF1 pS268	[illegible]	p09007	Bcl-2-associated transcription factor 1
RRM2 seq: 6 - 41 + 2 Phospho (ST)	[illegible]	p01006	Ribonucleoside-diphosphate reductase subunit M2
SRRM2 pS2692 pS2694	[illegible]	p04963	Serine/arginine repetitive matrix protein 2
AHNAK pS1196	[illegible]	p13698	Neuroblast differentiation-associated protein AHNAK
NENF seq: 103 - 120 no mod	[illegible]	p09897	Neudesin
UROS seq: 66 - 79 no mod	[illegible]	p19259	Uroporphyrinogen-III synthase
SIPA1L3 seq: 170 - 188 + Phospho (ST)	[illegible]	p03720	Signal-induced proliferation-associated 1-like protein 3
ARHGAP21 seq: 456 - 469 + Phospho (ST)	[illegible]	p03256	Rho GTPase-activating protein 21
RPL18A pS71	[illegible]	p03901	60S ribosomal protein L18a
EPB41L1 pS510	[illegible]	p14682	Band 4.1-like protein 1
LARP4 pS583	[illegible]	p18813	La-related protein 4
ELF2 seq: 405 - 432 + Phospho (ST)	[illegible]	p13638	ETS-related transcription factor Elf-2
JUP pS665	[illegible]	p06423	Junction plakoglobin
THRAP3 pS248 pS253	[illegible]	p04724	Thyroid hormone receptor-associated protein 3
ARHGEF18 pS1124	[illegible]	p14632	Rho guanine nucleotide exchange factor 18
SHROOM3 seq: 1116 - 1150 + Phospho (ST); Phospho (Y)	[illegible]	p19769	Protein Shroom3
TACC3 pS317	[illegible]	p20068	Transforming acidic coiled-coil-containing protein 3
PRRC2C pS2105	[illegible]	p19797	Protein PRRC2C
U2AF2 seq: 175 - 195 no mod	[illegible]	p11774	Splicing factor U2AF 65 kDa subunit
PDIA3 seq: 235 - 252 no mod	[illegible]	p16674	Protein disulfide-isomerase A3
SNX3 pS72	[illegible]	p06400	Sorting nexin-3
STARD13 seq: 409 - 427 + Phospho (ST)	[illegible]	p20175	StAR-related lipid transfer protein 13
PRKD3 seq: 395 - 408 + Phospho (ST)	[illegible]	p02690	Serine/threonine-protein kinase D3
CLASP1 seq: 644 - 660 + 2 Phospho (ST)	[illegible]	p06783	CLIP-associating protein 1
TRPM5 seq: 1071 - 1079 + Phospho (ST)	[illegible]	p07521	Transient receptor potential cation channel subfamily M member 5
DCAF7 seq: 34 - 46 no mod	[illegible]	p12238	DDB1- and CUL4-associated factor 7
DIDO1 pS1456	[illegible]	p06997	Death-inducer obliterator 1
ITSN1 pS904	[illegible]	p06344	Intersectin-1
GIT1 pS362	[illegible]	p04990	ARF GTPase-activating protein GIT1
KRT19 seq: 150 - 158 no mod	[illegible]	p13545	Keratin, type I cytoskeletal 19
TRPS1 pS1081 pS1085	[illegible]	p14696	Zinc finger transcription factor Trps1
MAPT pS428 pS437	[illegible]	p14687	Microtubule-associated protein tau
TBC1D10B pS687	[illegible]	p19660	TBC1 domain family member 10B
GSE1 pS826 pS828	[illegible]	p17192	Genetic suppressor element 1
TKT pS295	[illegible]	p14085	Transketolase
ZC3H13 seq: 316 - 330 + 2 Phospho (ST)	[illegible]	p03448	Zinc finger CCCH domain-containing protein 13
NCAPH pT49	[illegible]	p13414	Condensin complex subunit 2
CFL1 seq: 46 - 73 no mod	[illegible]	p09551	Cofilin-1
STUB1 pS273	[illegible]	p04534	E3 ubiquitin-protein ligase CHIP
AP1G1 seq: 804 - 822 no mod	[illegible]	p15313	AP-1 complex subunit gamma-1
PRKD1 pS205	[illegible]	p07104	Serine/threonine-protein kinase D1
LRP5 seq: 45 - 52 no mod	[illegible]	p10447	Low-density lipoprotein receptor-related protein 5
MYO9B pS1405	[illegible]	p12796	Unconventional myosin-IXb
CABLES2 seq: 97 - 119 + Phospho (ST)	[illegible]	p02427	CDK5 and ABL1 enzyme substrate 2
FASN seq: 1172 - 1180 no mod	[illegible]	p18395	Fatty acid synthase
SIPA1L3 seq: 1542 - 1552 + Phospho (ST)	[illegible]	p02568	Signal-induced proliferation-associated 1-like protein 3
EIF4ENIF1 seq: 136 - 148 + Phospho (ST)	[illegible]	p05668	Eukaryotic translation initiation factor 4E transporter
DDX54 pS782	[illegible]	p14444	ATP-dependent RNA helicase DDX54
TJP3 pS164	[illegible]	p04644	Tight junction protein ZO-3
ARL6IP4 pS332	[illegible]	p06339	ADP-ribosylation factor-like protein 6-interacting protein 4
ACIN1 pS216	[illegible]	p12530	Apoptotic chromatin condensation inducer in the nucleus
FAM21A pS1075	[illegible]	p15502	WASH complex subunit FAM21A
PDIA3 seq: 63 - 73 no mod	[illegible]	p12217	Protein disulfide-isomerase A3
TMX1 pS270	[illegible]	p07959	Thioredoxin-related transmembrane protein 1
RPL34 seq: 44 - 52 no mod	[illegible]	p06369	60S ribosomal protein L34
EPB41L2 seq: 598 - 611 + Phospho (ST)	[illegible]	p04340	Band 4.1-like protein 2
MTHFD1 seq: 464 - 473 no mod	[illegible]	p14304	C-1-tetrahydrofolate synthase, cytoplasmic
PDIA3 seq: 352 - 362 no mod	[illegible]	p16595	Protein disulfide-isomerase A3
FAM53C seq: 250 - 263 + 2 Phospho (ST)	[illegible]	p03823	Protein FAM53C
DDX3X seq: 590 - 600 + Phospho (ST)	[illegible]	p03741	ATP-dependent RNA helicase DDX3X
DCLK1 seq: 346 - 360 + Phospho (ST)	[illegible]	p03748	Serine/threonine-protein kinase DCLK1
AKAP10 pS52	[illegible]	p19596	A-kinase anchor protein 10, mitochondrial
CDC20 pT70	[illegible]	p00918	Cell division cycle protein 20 homolog
STARD10 seq: 4 - 19 + Phospho (ST)	[illegible]	p12298	PCTP-like protein
BCL2L11 seq: 85 - 91 + Phospho (ST)	[illegible]	p06604	Bcl-2-like protein 11
CDK18 seq: 148 - 158 + Phospho (Y)	[illegible]	p11800	Cyclin-dependent kinase 18
GRB7 seq: 366 - 383 + Phospho (ST)	[illegible]	p06272	Growth factor receptor-bound protein 7
CCDC181 seq: 362 - 368 no mod	[illegible]	p14364	Coiled-coil domain-containing protein 181
ENO1 seq: 413 - 420 no mod	[illegible]	p14363	Alpha-enolase
TRIM34 seq: 538 - 544 no mod	[illegible]	p12002	Tripartite motif-containing protein 34
PRKRA seq: 8 - 25 + Phospho (ST)	[illegible]	p20869	Interferon-inducible double-stranded RNA-dependent protein kinase activator A
NAA10 seq: 199 - 235 + Phospho (ST)	[illegible]	p15787	N-alpha-acetyltransferase 10
PDCD4 pS457	[illegible]	p16379	Programmed cell death protein 4
LIG1 pS141	[illegible]	p02702	DNA ligase 1
ELMSAN1 pS461	[illegible]	p07559	ELM2 and SANT domain-containing protein 1
ZC3HC1 seq: 352 - 374 + Phospho (ST)	[illegible]	p02207	Nuclear-interacting partner of ALK
TMPO pS310	[illegible]	p03624	Lamina-associated polypeptide 2, isoform alpha
CDK16 pS153	[illegible]	p06437	Cyclin-dependent kinase 16
LRRFIP1 seq: 99 - 117 + Phospho (ST)	[illegible]	p09364	Leucine-rich repeat flightless-interacting protein 1
NACA seq: 2008 - 2042 + 2 Phospho (ST)	[illegible]	p00959	Nascent polypeptide-associated complex subunit alpha
COL21A1 seq: 247 - 252 + Phospho (ST)	[illegible]	p13808	Collagen alpha-1(XXI) chain
FAM83H pS974	[illegible]	p08350	Protein FAM83H
ATXN2L seq: 580 - 599 + Phospho (ST)	[illegible]	p17569	Ataxin-2-like protein
CTAGE5 seq: 515 - 532 + Phospho (Y)	[illegible]	p17644	cTAGE family member 5
NUFIP2 pS629	[illegible]	p18057	Nuclear fragile X mental retardation-interacting protein 2
CIC pS173	[illegible]	p06497	Protein capicua homolog
USP15 pS229	[illegible]	p04657	Ubiquitin carboxyl-terminal hydrolase 15
PCBP1 seq: 178 - 200 no mod	[illegible]	p01097	Poly(rC)-binding protein 1
USP10 pS370	[illegible]	p00095	Ubiquitin carboxyl-terminal hydrolase 10
SRRM1 pS429 pS431	[illegible]	p12546	Serine/arginine repetitive matrix protein 1
TMPO pS370	[illegible]	p20282	Lamina-associated polypeptide 2, isoform alpha
MISP seq: 263 - 289 + Phospho (ST)	[illegible]	p19204	Mitotic interactor and substrate of PLK1
DDX42 pS751	[illegible]	p20556	ATP-dependent RNA helicase DDX42
LDB1 seq: 300 - 320 + Phospho (ST)	[illegible]	p12859	LIM domain-binding protein 1
TACC3 seq: 313 - 332 + Phospho (ST)	[illegible]	p20067	Transforming acidic coiled-coil-containing protein 3
MICAL3 pS1310	[illegible]	p11688	Protein-methionine sulfoxide oxidase MICAL3
SUGT1 seq: 71 - 84 no mod	[illegible]	p05261	Protein SGT1 homolog
GLCCI1 seq: 169 - 187 + Phospho (ST)	[illegible]	p06484	Glucocorticoid-induced transcript 1 protein
PDIA3 seq: 434 - 448 no mod	[illegible]	p10223	Protein disulfide-isomerase A3
q: 852 - 870 + Gln->pyro-Glu (N-term Q); 2 Phospho (ST)	[illegible]	p08599	Serine/arginine repetitive matrix protein 2
SIPA1L1 seq: 1126 - 1143 + Phospho (ST)	[illegible]	p10708	Signal-induced proliferation-associated 1-like protein 1
HCFC1 seq: 2016 - 2024 + Phospho (ST)	[illegible]	p06899	Host cell factor 1
STARD10 pS284	[illegible]	p10123	PCTP-like protein
NUP50 pS221	[illegible]	p09164	Nuclear pore complex protein Nup50
PRR12 pT740 pS747	[illegible]	p12684	Proline-rich protein 12
EIF2S2 pT111	[illegible]	p14338	Eukaryotic translation initiation factor 2 subunit 2
RGS12 seq: 840 - 854 + Phospho (ST)	[illegible]	p20210	Regulator of G-protein signaling 12
IK seq: 39 - 44 + Phospho (ST)	[illegible]	p11345	Protein Red
PIGO pT601	[illegible]	p11259	GPI ethanolamine phosphate transferase 3
TES pS168	[illegible]	p08344	Testin
ATXN2L pS594	[illegible]	p17570	Ataxin-2-like protein
ENO3 seq: 344 - 358 no mod	[illegible]	p01053	Beta-enolase
ENO1 seq: 344 - 358 no mod	[illegible]	p01051	Alpha-enolase
SRCIN1 pS859	[illegible]	p07362	SRC kinase signaling inhibitor 1
CTAGE5 seq: 515 - 532 + Phospho (ST)	[illegible]	p17646	cTAGE family member 5
ARAP3 pS1444	[illegible]	p05282	Arf-GAP with Rho-GAP domain, ANK repeat and PH domain-containing protein 3
S100A6 seq: 48 - 55 no mod	[illegible]	p11027	Protein S100-A6
YWHAZ seq: 140 - 157 no mod	[illegible]	p15837	14-3-3 protein zeta/delta
LRRC59 seq: 41 - 50 no mod	[illegible]	p19369	Leucine-rich repeat-containing protein 59
RNF214 seq: 483 - 509 + Phospho (ST)	[illegible]	p05061	RING finger protein 214
TKT seq: 472 - 493 no mod	[illegible]	p02123	Transketolase
ZNF22 pS49	[illegible]	p05308	Zinc finger protein 22
KIF13B pS645	[illegible]	p07100	Kinesin-like protein KIF13B
S100A16 seq: 45 - 56 no mod	[illegible]	p17441	Protein S100-A16
CD2AP pS458	[illegible]	p03382	CD2-associated protein

KIAA1671 pS1488	p17217	Uncharacterized protein KIAA1671
HNRNPC seq: 51 - 61 no mod	p16144	Heterogeneous nuclear ribonucleoproteins C1/C2
DTNBP1 pS316 pS321	p18727	Dysbindin
HNRNPUL1 seq: 722 - 731 no mod	p09263	Heterogeneous nuclear ribonucleoprotein U-like protein 1
PLEKHA5 seq: 855 - 870 + Phospho (ST)	p04327	Pleckstrin homology domain-containing family A member 5
LARP4B seq: 509 - 545 + Phospho (ST)	p16409	La-related protein 4B
TNRC18 pS1540	p02757	Trinucleotide repeat-containing gene 18 protein
DSP pS22	p20808	Desmoplakin
CREBBP seq: 267 - 300 + Phospho (ST)	p10119	CREB-binding protein
SNW1 pS224	p15460	SNW domain-containing protein 1
PRR5 seq: 252 - 279 + Phospho (ST)	p05344	Proline-rich protein 5
MOCS2 pS20	p11109	Molybdopterin synthase catalytic subunit
SYBU pS555	p09264	Syntabulin
MVD pS96	p09092	Diphosphomevalonate decarboxylase
PCM1 pS65	p00700	Pericentriolar material 1 protein
NUP153 pS614	p17673	Nuclear pore complex protein Nup153
PSMD2 pS361	p16755	26S proteasome non-ATPase regulatory subunit 2
ARHGAP29 seq: 1178 - 1204 + Phospho (ST)	p15591	Rho GTPase-activating protein 29
MAP4 pS280	p18494	Microtubule-associated protein 4
FAM64A pS131	p15275	Protein FAM64A
CCT2 seq: 58 - 72 no mod	p18954	T-complex protein 1 subunit beta
RAD23B seq: 145 - 173 + Phospho (ST)	p08732	UV excision repair protein RAD23 homolog B
SARG seq: 409 - 439 + Phospho (ST)	p20318	Specifically androgen-regulated gene protein
C11orf84 seq: 144 - 170 + 2 Phospho (ST)	p20811	Uncharacterized protein C11orf84
SHANK2 pS877	p09918	SH3 and multiple ankyrin repeat domains protein 2
CABLES1 seq: 290 - 306 + Phospho (ST)	p03863	CDK5 and ABL1 enzyme substrate 1
SYTL1 pS392	p00995	Synaptotagmin-like protein 1
JUP pS182	p20220	Junction plakoglobin
SIPA1L1 seq: 205 - 227 + Phospho (ST)	p16908	Signal-induced proliferation-associated 1-like protein 1
MVD pS96	p09091	Diphosphomevalonate decarboxylase
FAM64A pS131	p15274	Protein FAM64A
ACTG1 seq: 197 - 206 no mod	p14916	Actin, cytoplasmic 2
LIN9 seq: 301 - 324 + Phospho (ST)	p11622	Protein lin-9 homolog
PHF20L1 pS584	p08576	PHD finger protein 20-like protein 1
SNRPA1 seq: 178 - 191 + Phospho (ST)	p05340	U2 small nuclear ribonucleoprotein A'
CBX5 pS92	p04845	Chromobox protein homolog 5
NEDD4L seq: 469 - 493 + Phospho (ST)	p19233	E3 ubiquitin-protein ligase NEDD4-like
RABL6 pS470 pS471	p15217	Rab-like protein 6
FAM195B seq: 21 - 38 + Phospho (ST)	p04485	Mapk-regulated corepressor-interacting protein 1
EPPK1 seq: 1279 - 1297 no mod	p11437	Epiplakin
KIAA1109 pS4124	p02815	Uncharacterized protein KIAA1109
LRRC16A seq: 1080 - 1096 + Phospho (ST)	p05998	Leucine-rich repeat-containing protein 16A
PEG10 seq: 23 - 36 no mod	p07930	Retrotransposon-derived protein PEG10
C2CD5 seq: 248 - 266 + Phospho (ST)	p10650	C2 domain-containing protein 5
PRRC2C pS2105	p19796	Protein PRRC2C
CLMN seq: 829 - 857 + Phospho (ST)	p17346	Calmin
LRRFIP1 seq: 99 - 117 + Phospho (ST)	p09365	Leucine-rich repeat flightless-interacting protein 1
CDCA3 seq: 199 - 216 + 2 Phospho (ST)	p04537	Cell division cycle-associated protein 3
RPS7 seq: 170 - 178 no mod	p01594	40S ribosomal protein S7
FAM117B seq: 218 - 235 + Phospho (ST)	p02099	Protein FAM117B
MYRIP pS295	p04221	Rab effector MyRIP
BCKDHA pS347	p03384	2-oxoisovalerate dehydrogenase subunit alpha, mitochondrial
SRRM2 pS1463	p14637	Serine/arginine repetitive matrix protein 2
NCOR1 seq: 2419 - 2440 + Phospho (ST)	p13511	Nuclear receptor corepressor 1
AKAP12 pS612	p17659	A-kinase anchor protein 12
PSPC1 pS409	p16305	Paraspeckle component 1
MAP4 pT521	p18083	Microtubule-associated protein 4
PRRC2B seq: 552 - 561 + Phospho (ST)	p16960	Protein PRRC2B
TMX1 pS247	p12446	Thioredoxin-related transmembrane protein 1
SIPA1L1 seq: 205 - 227 + Phospho (Y)	p16907	Signal-induced proliferation-associated 1-like protein 1
MAST4 pS1373	p06330	Microtubule-associated serine/threonine-protein kinase 4
FLNB seq: 503 - 522 + Phospho (ST)	p08476	Filamin-B
SRRM1 pS769	p12757	Serine/arginine repetitive matrix protein 1
HSPA4 seq: 361 - 374 no mod	p17391	Heat shock 70 kDa protein 4
FRMD5 pS351	p06142	FERM domain-containing protein 5
PFN1 pM86	p18254	Profilin-1
YAP1 seq: 350 - 375 + Phospho (ST)	p04241	Transcriptional coactivator YAP1
DLGAP4 pS665	p12905	Disks large-associated protein 4
CLIP2 seq: 346 - 360 + Phospho (ST)	p13070	CAP-Gly domain-containing linker protein 2
COBL pS347	p07619	Protein cordon-bleu
PTTG2 pS165	p11869	Securin-2
FAM21C pT331 pS333	p17206	WASH complex subunit FAM21C
VCP pS770	p16114	Transitional endoplasmic reticulum ATPase
KTN1 seq: 960 - 971 no mod	p02278	Kinectin
INF2 seq: 579 - 604 + Phospho (ST)	p17649	Inverted formin-2
MYH14 pS1969	p11998	Myosin-14
FAM117B seq: 217 - 235 + Phospho (ST)	p06483	Protein FAM117B
SRRM2 seq: 346 - 356 + 2 Phospho (ST)	p06192	Serine/arginine repetitive matrix protein 2
HCFC1 pS666	p04574	Host cell factor 1
KIAA1522 seq: 562 - 586 + Phospho (ST)	p04656	Uncharacterized protein KIAA1522
SSR4 seq: 121 - 139 no mod	p09348	Translocon-associated protein subunit delta
LIMA1 pS686	p05429	LIM domain and actin-binding protein 1
RPL5 seq: 210 - 221 no mod	p00253	60S ribosomal protein L5
DDX51 pS83	p01088	ATP-dependent RNA helicase DDX51
CTNNA2 seq: 652 - 670 + Phospho (ST)	p03956	Catenin alpha-2
KIAA1217 seq: 1785 - 1801 + Phospho (ST)	p12585	Sickle tail protein homolog
CHAMP1 seq: 270 - 279 + 2 Phospho (ST)	p06278	Chromosome alignment-maintaining phosphoprotein 1
TUBB seq: 242 - 251 no mod	p16536	Tubulin beta chain
PPIA seq: 20 - 28 no mod	p00867	Peptidyl-prolyl cis-trans isomerase A
EPS15L1 pS229	p01967	Epidermal growth factor receptor substrate 15-like 1
MET seq: 988 - 1004 + Phospho (ST)	p03259	Hepatocyte growth factor receptor
SULT2B1 seq: 134 - 139 + Phospho (ST)	p20775	Sulfotransferase family cytosolic 2B member 1
ECT2 pS842	p20747	Protein ECT2
CD58 seq: 73 - 78 + Phospho (ST)	p20744	Lymphocyte function-associated antigen 3
ERRFI1 seq: 249 - 263 + Phospho (ST)	p05581	ERBB receptor feedback inhibitor 1
KRT8 seq: 234 - 252 no mod	p17414	Keratin, type II cytoskeletal 8
CSTF3 pS691	p06898	Cleavage stimulation factor subunit 3
CAD seq: 1404 - 1411 + Phospho (ST)	p07087	CAD protein
KAT7 seq: 49 - 60 + Phospho (ST)	p10691	Histone acetyltransferase KAT7
SRRM2 seq: 346 - 356 + 2 Phospho (ST)	p06193	Serine/arginine repetitive matrix protein 2
LARGE pS159	p03321	Glycosyltransferase-like protein LARGE1
MKI67 pS1937	p12070	Antigen KI-67
WIPF2 seq: 257 - 279 + Phospho (ST)	p08116	WAS/WASL-interacting protein family member 2
NEDD4L seq: 472 - 493 + Phospho (ST)	p18168	E3 ubiquitin-protein ligase NEDD4-like
MT2A seq: 1 - 20 no mod	p10210	Metallothionein-2
SERPINB5 seq: 65 - 79 no mod	p18100	Serpin B5
RNF20 seq: 515 - 551 + Phospho (ST)	p05698	E3 ubiquitin-protein ligase BRE1A
LMO7 seq: 1423 - 1434 + Phospho (ST)	p03415	LIM domain only protein 7
DNPH1 seq: 37 - 47 + Phospho (ST)	p02507	2'-deoxynucleoside 5'-phosphate N-hydrolase 1
MAP3K3 seq: 350 - 363 + Phospho (ST)	p08950	Mitogen-activated protein kinase kinase kinase 3
FLNB seq: 2121 - 2139 + Phospho (ST)	p13883	Filamin-B
PKP3 pS123	p06180	Plakophilin-3
FAM129B seq: 686 - 705 + Phospho (ST)	p21077	Niban-like protein 1
CCDC86 pS91	p10952	Coiled-coil domain-containing protein 86
TCOF1 seq: 1094 - 1120 + Phospho (ST)	p02269	Treacle protein
DENND1A seq: 518 - 531 + Phospho (ST)	p06479	DENN domain-containing protein 1A
DDI2 pS121	p07965	Protein DDI1 homolog 2
SRSF11 pS207	p11186	Serine/arginine-rich splicing factor 11
GPN1 seq: 311 - 326 + Phospho (ST)	p18251	GPN-loop GTPase 1
PKP2 seq: 288 - 299 + Phospho (ST)	p03569	Plakophilin-2
FAF1 pS582	p13762	FAS-associated factor 1
;: 1235 - 1249 + Gln->pyro-Glu (N-term Q); Phospho (ST)	p08134	Neuron navigator 3
BRAF seq: 363 - 384 + Phospho (ST)	p03730	Serine/threonine-protein kinase B-raf
CNOT4 pS432	p17390	CCR4-NOT transcription complex subunit 4
PGM2L1 pS175	p19275	Glucose 1,6-bisphosphate synthase
FOXK1 pS213	p17860	Forkhead box protein K1
MATR3 pS598	p03130	Matrin-3
LMNB1 seq: 272 - 290 + Phospho (ST)	p11954	Lamin-B1
STAT3 pS727	p16695	Signal transducer and activator of transcription 3
TPI1 pS58	p07888	Triosephosphate isomerase
RAD23B seq: 145 - 173 + Phospho (ST)	p08733	UV excision repair protein RAD23 homolog B
TEAD3 seq: 144 - 160 + Phospho (ST)	p16452	Transcriptional enhancer factor TEF-5
PNN pS100	p08712	Pinin
ZC3H13 seq: 638 - 646 + Phospho (ST)	p18367	Zinc finger CCCH domain-containing protein 13
;: 3966 - 3977 + Gln->pyro-Glu (N-term Q); Phospho (ST)	p08437	Dystonin
AHNAK pT4766	p01679	Neuroblast differentiation-associated protein AHNAK
DOCK7 seq: 896 - 916 + Phospho (ST)	p05037	Dedicator of cytokinesis protein 7
TESK2 pS456	p05118	Dual specificity testis-specific protein kinase 2
NAPRT pS537	p11036	Nicotinate phosphoribosyltransferase
CDC42EP1 pS192	p06082	Cdc42 effector protein 1
LASP1 seq: 97 - 109 no mod	p16091	LIM and SH3 domain protein 1
MYH9 seq: 1731 - 1751 no mod	p14488	Myosin-9
RBM12 pS424	p04597	RNA-binding protein 12
RELT seq: 369 - 402 + Phospho (ST)	p02687	Tumor necrosis factor receptor superfamily member 19L
NONO seq: 384 - 398 no mod	p10087	Non-POU domain-containing octamer-binding protein
MYO9B pS1935	p11790	Unconventional myosin-IXb
CXXC1 pS224	p00383	CXXC-type zinc finger protein 1
AKAP12 pS286	p06351	A-kinase anchor protein 12
ZC3H13 seq: 316 - 330 + 2 Phospho (ST)	p03449	Zinc finger CCCH domain-containing protein 13
AHNAK pS5763	p19579	Neuroblast differentiation-associated protein AHNAK
KRT19 seq: 266 - 274 no mod	p18997	Keratin, type I cytoskeletal 19
CAV1 seq: 6 - 19 + Phospho (Y)	p00030	Caveolin-1
DBNL pS232	p00165	Drebrin-like protein
SQSTM1 seq: 268 - 281 no mod	p10509	Sequestosome-1
KRT8 seq: 24 - 32 + Phospho (Y)	p03111	Keratin, type II cytoskeletal 8
SRSF8 seq: 155 - 162 + Phospho (ST)	p00077	Serine/arginine-rich splicing factor 8
WIZ pS1012 pS1017	p04462	Protein Wiz
KRT17 seq: 224 - 232 no mod	p01265	Keratin, type I cytoskeletal 17
PPP4R4 seq: 773 - 784 + Phospho (ST)	p04201	Serine/threonine-protein phosphatase 4 regulatory subunit 4
CAMSAP3 seq: 1072 - 1088 + 2 Phospho (ST)	p19898	Calmodulin-regulated spectrin-associated protein 3
ZFYVE19 pS354	p11132	Abscission/NoCut checkpoint regulator
DCAF8 pS99	p01424	DDB1- and CUL4-associated factor 8
SURF6 pT229	p15577	Surfeit locus protein 6
ERBB2 pS1083	p04567	Receptor tyrosine-protein kinase erbB-2
KRT8 seq: 134 - 148 + Oxidation (M)	p04836	Keratin, type II cytoskeletal 8
RAD23A seq: 120 - 149 + Phospho (ST)	p17879	UV excision repair protein RAD23 homolog A
SH3BP5L pS343	p01981	SH3 domain-binding protein 5-like
XPC pS883 pS884	p06062	DNA repair protein complementing XP-C cells
TOM1 seq: 453 - 478 + Phospho (ST)	p21183	Target of Myb protein 1
HDGF seq: 198 - 235 + Phospho (ST)	p09053	Hepatoma-derived growth factor
ARMCX3 seq: 108 - 125 + Phospho (ST)	p07566	Armadillo repeat-containing X-linked protein 3
DDX42 pS751	p20557	ATP-dependent RNA helicase DDX42
MISP pS575	p07040	Mitotic interactor and substrate of PLK1
MPRIP pS977	p21001	Myosin phosphatase Rho-interacting protein
GPRC5A pS345	p20538	Retinoic acid-induced protein 3
KIF13B seq: 1791 - 1810 + Phospho (ST)	p06197	Kinesin-like protein KIF13B
AKAP11 pS448	p17868	A-kinase anchor protein 11
ENO1 seq: 16 - 28 no mod	p15574	Alpha-enolase
ENO2 seq: 16 - 28 no mod	p15573	Gamma-enolase
CUL3 seq: 355 - 364 no mod	p16606	Cullin-3
FDXR seq: 194 - 204 no mod	p14018	NADPH:adrenodoxin oxidoreductase, mitochondrial
COBL seq: 561 - 575 no mod	p09325	Protein cordon-bleu
LMO7 pS704	p04877	LIM domain only protein 7
CTNND1 pS864	p04165	Catenin delta-1
N4BP1 pS300	p08674	NEDD4-binding protein 1
TP53BP1 pS727	p13315	Tumor suppressor p53-binding protein 1
TNKS1BP1 pS435	p16484	182 kDa tankyrase-1-binding protein
LRRFIP1 seq: 720 - 749 + Phospho (ST)	p06056	Leucine-rich repeat flightless-interacting protein 1
GTF3C2 seq: 883 - 896 + Phospho (ST)	p20535	General transcription factor 3C polypeptide 2
USP6NL pS642	p15777	USP6 N-terminal-like protein
CRTC1 seq: 149 - 178 + Phospho (ST)	p02468	CREB-regulated transcription coactivator 1
SCRIB pS1378	p06433	Protein scribble homolog
DBF4 pS413	p11399	Protein DBF4 homolog A
CALB2 seq: 246 - 255 no mod	p03317	Calretinin
CD2AP seq: 505 - 517 + Phospho (ST)	p16562	CD2-associated protein
HSP90AA1 seq: 387 - 400 no mod	p14972	Heat shock protein HSP 90-alpha
FKBP3 seq: 127 - 154 + Phospho (ST)	p13205	Peptidyl-prolyl cis-trans isomerase FKBP3
HSPB1 pS82	p08279	Heat shock protein beta-1
RPIA pS106	p09312	Ribose-5-phosphate isomerase
NOMO3 seq: 1035 - 1052 no mod	p01398	Nodal modulator 3
CRKL seq: 194 - 241 + Phospho (Y)	p09128	Crk-like protein
RANBP2 seq: 2802 - 2823 + Phospho (ST)	p05459	E3 SUMO-protein ligase RanBP2
LIMD1 seq: 403 - 426 + Phospho (ST)	p17701	LIM domain-containing protein 1
RPS6KA1 pS380	p16096	Ribosomal protein S6 kinase alpha-1
TCOF1 seq: 571 - 586 + Phospho (ST)	p05220	Treacle protein
PPHLN1 seq: 192 - 202 + Phospho (ST)	p17211	Periphilin-1
RSF1 pT1305	p14330	Remodeling and spacing factor 1
TNKS1BP1 pS1385	p14718	182 kDa tankyrase-1-binding protein
LIN54 pT280	p01153	Protein lin-54 homolog
TJP3 seq: 163 - 181 + Phospho (ST)	p06651	Tight junction protein ZO-3

Peptide	Abundance values	Accession	Protein
YBX1 seq: 305 - 324 + Phospho (ST)	[illegible]	p21185	Nuclease-sensitive element-binding protein 1
RICTOR seq: 1282 - 1298 + Phospho (ST)	[illegible]	p04753	Rapamycin-insensitive companion of mTOR
PPIG pS356 pT358	[illegible]	p16502	Peptidyl-prolyl cis-trans isomerase G
CDK17 pS9	[illegible]	p07113	Cyclin-dependent kinase 17
LRIG1 pS1069	[illegible]	p10957	Leucine-rich repeats and immunoglobulin-like domains protein 1
FXR1 pS409	[illegible]	p07371	Fragile X mental retardation syndrome-related protein 1
NAP1L4 pS125	[illegible]	p17798	Nucleosome assembly protein 1-like 4
MATR3 seq: 596 - 609 + 2 Phospho (ST)	[illegible]	p03133	Matrin-3
TJP2 seq: 1108 - 1123 + Phospho (Y)	[illegible]	p14353	Tight junction protein ZO-2
EGFR pS991	[illegible]	p10080	Epidermal growth factor receptor
KIAA0753 seq: 507 - 513 + Phospho (ST)	[illegible]	p18172	Protein moonraker
ISCU pS29	[illegible]	p17412	Iron-sulfur cluster assembly enzyme ISCU, mitochondrial
HDGFRP2 pS652	[illegible]	p17707	Hepatoma-derived growth factor-related protein 2
RAPH1 pS610	[illegible]	p03160	Ras-associated and pleckstrin homology domains-containing protein 1
EIF3A pS882	[illegible]	p11811	Eukaryotic translation initiation factor 3 subunit A
PDCD6IP seq: 708 - 715 no mod	[illegible]	p18552	Programmed cell death 6-interacting protein
SON seq: 2009 - 2016 + 2 Phospho (ST)	[illegible]	p05953	Protein SON
BOLA2 seq: 15 - 30 no mod	[illegible]	p18635	BolA-like protein 2
BLVRB pM98	[illegible]	p09529	Flavin reductase (NADPH)
EXPH5 pS1124	[illegible]	p20077	Exophilin-5
AKAP2 pS152	[illegible]	p18973	A-kinase anchor protein 2
KIF5B seq: 287 - 297 no mod	[illegible]	p14006	Kinesin-1 heavy chain
KIF13B pS1797	[illegible]	p06198	Kinesin-like protein KIF13B
DLGAP4 seq: 663 - 679 + Phospho (ST)	[illegible]	p12904	Disks large-associated protein 4
IRF2BPL seq: 331 - 342 + Phospho (ST)	[illegible]	p06932	Interferon regulatory factor 2-binding protein-like
PIK3C2B pS155	[illegible]	p12912	Phosphatidylinositol 4-phosphate 3-kinase C2 domain-containing subunit beta
HUWE1 pS1907	[illegible]	p13271	E3 ubiquitin-protein ligase HUWE1
G6PD seq: 294 - 320 no mod	[illegible]	p13047	Glucose-6-phosphate 1-dehydrogenase
HSPD1 seq: 97 - 121 no mod	[illegible]	p10370	60 kDa heat shock protein, mitochondrial
HMG20A pS105	[illegible]	p04540	High mobility group protein 20A
SF3A1 pS329	[illegible]	p00409	Splicing factor 3A subunit 1
NKTR pS379	[illegible]	p19113	NK-tumor recognition protein
PHLDB2 seq: 172 - 195 + Phospho (ST)	[illegible]	p19602	Pleckstrin homology-like domain family B member 2
NDRG2 seq: 330 - 343 + 2 Phospho (ST)	[illegible]	p03027	Protein NDRG2
MLLT4 seq: 1229 - 1241 + Phospho (ST)	[illegible]	p14911	Afadin
COBL pS284	[illegible]	p04952	Protein cordon-bleu
CTNND1 pS349 pS352	[illegible]	p15253	Catenin delta-1
GTF3C2 seq: 883 - 896 + Phospho (ST)	[illegible]	p20536	General transcription factor 3C polypeptide 2
TRIM33 pS862	[illegible]	p07930	E3 ubiquitin-protein ligase TRIM33
TMPO pS184	[illegible]	p08215	Lamina-associated polypeptide 2, isoforms beta/gamma
TNKS1BP1 seq: 1023 - 1043 + 2 Phospho (ST)	[illegible]	p15285	182 kDa tankyrase-1-binding protein
LRRC16A seq: 1078 - 1096 + Phospho (ST)	[illegible]	p05371	Leucine-rich repeat-containing protein 16A
ACTC1 seq: 186 - 193 no mod	[illegible]	p18524	Actin, alpha cardiac muscle 1
LMNA seq: 598 - 624 + Phospho (ST)	[illegible]	p19649	Prelamin-A/C
KIAA1217 pS1044	[illegible]	p11779	Sickle tail protein homolog
WDFY4 pT634	[illegible]	p13960	WD repeat- and FYVE domain-containing protein 4
HMGXB4 pS197	[illegible]	p11575	HMG domain-containing protein 4
MORC2 pS743	[illegible]	p01389	MORC family CW-type zinc finger protein 2
MEF2BNB seq: 99 - 119 + Phospho (ST)	[illegible]	p18782	Protein MEF2BNB
NEURL4 seq: 500 - 508 + Phospho (ST)	[illegible]	p20168	Neuralized-like protein 4
RPLP0P6 pS304 pS307	[illegible]	p01646	60S acidic ribosomal protein P0-like
SCRIB seq: 1260 - 1269 + Phospho (Y)	[illegible]	p15663	Protein scribble homolog
NUFIP2 seq: 618 - 639 + Phospho (ST)	[illegible]	p18056	Nuclear fragile X mental retardation-interacting protein 2
ENO1 seq: 359 - 372 no mod	[illegible]	p12203	Alpha-enolase
AHNAK pS93	[illegible]	p16203	Neuroblast differentiation-associated protein AHNAK
FAM193A seq: 1120 - 1138 + Phospho (ST)	[illegible]	p04820	Protein FAM193A
NKTR pS463	[illegible]	p14034	NK-tumor recognition protein
HSP90AB1 seq: 73 - 82 no mod	[illegible]	p14422	Heat shock protein HSP 90-beta
LRBA seq: 1777 - 1799 + Phospho (ST)	[illegible]	p11067	Lipopolysaccharide-responsive and beige-like anchor protein
BAG6 seq: 943 - 969 + Phospho (ST)	[illegible]	p01514	Large proline-rich protein BAG6
TCOF1 pT914	[illegible]	p13220	Treacle protein
ADGRL2 pS1430	[illegible]	p15570	Adhesion G protein-coupled receptor L2
LPP pT333	[illegible]	p07473	Lipoma-preferred partner
ANLN seq: 95 - 127 + Phospho (ST)	[illegible]	p06131	Actin-binding protein anillin
RAB3GAP2 pS537	[illegible]	p12475	Rab3 GTPase-activating protein catalytic subunit
PKP3 pS314	[illegible]	p10665	Plakophilin-3
WIZ pS1127	[illegible]	p04436	Protein Wiz
NRCAM seq: 662 - 679 no mod	[illegible]	p03292	Neuronal cell adhesion molecule
MATR3 pS596 pS598	[illegible]	p20132	Matrin-3
PRRC2C pS2105	[illegible]	p11155	Protein PRRC2C
PHKB pS27	[illegible]	p06441	Phosphorylase b kinase regulatory subunit beta
PDLIM2 pS197	[illegible]	p20698	PDZ and LIM domain protein 2
SRRM2 seq: 2343 - 2356 + Phospho (ST)	[illegible]	p06237	Serine/arginine repetitive matrix protein 2
EPB41L3 pS962	[illegible]	p02382	Band 4.1-like protein 3
CHGB pS149	[illegible]	p20933	Secretogranin-1
LUC7L2 seq: 9 - 25 + Phospho (ST)	[illegible]	p20086	Putative RNA-binding protein Luc7-like 2
RPL18 seq: 79 - 91 no mod	[illegible]	p21994	60S ribosomal protein L18
PRICKLE1 pS683	[illegible]	p06095	Prickle-like protein 1
seq: 101 - 127 + Gln->pyro-Glu (N-term Q); Phospho (ST)	[illegible]	p07873	PC4 and SFRS1-interacting protein
XRN2 seq: 466 - 494 + 2 Phospho (ST)	[illegible]	p09930	5'-3' exoribonuclease 2
PSMD4 seq: 237 - 262 + Phospho (ST)	[illegible]	p01769	26S proteasome non-ATPase regulatory subunit 4
EPC1 seq: 344 - 367 + Phospho (ST)	[illegible]	p01194	Enhancer of polycomb homolog 1
SERPINB5 seq: 48 - 64 no mod	[illegible]	p16197	Serpin B5
GTF2F1 pS433	[illegible]	p10889	General transcription factor IIF subunit 1
HLA-A seq: 340 - 365 + 2 Phospho (ST)	[illegible]	p13192	HLA class I histocompatibility antigen, A-80 alpha chain
PRDX1 seq: 141 - 151 no mod	[illegible]	p08484	Peroxiredoxin-1
SHANK2 pS1234	[illegible]	p10326	SH3 and multiple ankyrin repeat domains protein 2
SRSF10 seq: 253 - 258 + Phospho (ST)	[illegible]	p00177	Serine/arginine-rich splicing factor 10
PUM2 pS182	[illegible]	p08815	Pumilio homolog 2
ARID1A seq: 377 - 389 + Phospho (ST)	[illegible]	p02346	AT-rich interactive domain-containing protein 1A
LIMA1 pS686	[illegible]	p17742	LIM domain and actin-binding protein 1
ADD1 pS358	[illegible]	p04610	Alpha-adducin
CEP57 seq: 53 - 67 + Phospho (ST)	[illegible]	p04422	Centrosomal protein of 57 kDa
ABL2 pS936	[illegible]	p00973	Abelson tyrosine-protein kinase 2
MEPCE seq: 208 - 222 + 2 Phospho (ST)	[illegible]	p02591	7SK snRNA methylphosphate capping enzyme
ATRX pS1244 pS1245	[illegible]	p14106	Transcriptional regulator ATRX
HIRIP3 pS196 pS199	[illegible]	p17818	HIRA-interacting protein 3
KRI1 pS136	[illegible]	p00036	Protein KRI1 homolog
KMT2D pS2274	[illegible]	p19632	Histone-lysine N-methyltransferase 2D
EIF4B pS497	[illegible]	p04168	Eukaryotic translation initiation factor 4B
ZFP36 pS186	[illegible]	p07892	Tristetraprolin
SFPQ seq: 299 - 315 no mod	[illegible]	p11851	Splicing factor, proline- and glutamine-rich
C6orf141 pS25	[illegible]	p05231	Uncharacterized protein C6orf141
SRRM2 pS1083	[illegible]	p07899	Serine/arginine repetitive matrix protein 2
GOLGA1 seq: 28 - 53 + Phospho (ST)	[illegible]	p02274	Golgin subfamily A member 1
DHRS2 seq: 190 - 197 no mod	[illegible]	p03079	Dehydrogenase/reductase SDR family member 2, mitochondrial
USP16 pS552	[illegible]	p09539	Ubiquitin carboxyl-terminal hydrolase 16
SRRM2 pS1124	[illegible]	p08757	Serine/arginine repetitive matrix protein 2
CTNND1 pS349	[illegible]	p15254	Catenin delta-1
AHNAK seq: 29 - 45 no mod	[illegible]	p18935	Neuroblast differentiation-associated protein AHNAK
TOX4 pS178	[illegible]	p10610	TOX high mobility group box family member 4
TNKS1BP1 seq: 976 - 1001 + Phospho (ST)	[illegible]	p05905	182 kDa tankyrase-1-binding protein
VAMP4 pS30	[illegible]	p09441	Vesicle-associated membrane protein 4
BRD4 seq: 457 - 478 + Phospho (ST)	[illegible]	p09971	Bromodomain-containing protein 4
CTNND1 pS349 pS352	[illegible]	p15252	Catenin delta-1
SRSF4 seq: 106 - 114 + Phospho (ST)	[illegible]	p11538	Serine/arginine-rich splicing factor 4
CORO7 pS21	[illegible]	p07459	Coronin-7
ZC3H4 pS1269	[illegible]	p02795	Zinc finger CCCH domain-containing protein 4
MYH9 seq: 1731 - 1751 no mod	[illegible]	p14489	Myosin-9
ADNP pS1071	[illegible]	p10713	Activity-dependent neuroprotector homeobox protein
PDCD5 seq: 114 - 125 + Oxidation (M); Phospho (ST)	[illegible]	p07200	Programmed cell death protein 5
MAP1S pS759	[illegible]	p19209	Microtubule-associated protein 1S
SAMD4B seq: 550 - 570 + Phospho (ST)	[illegible]	p14962	Protein Smaug homolog 2
C1orf106 pS246	[illegible]	p07025	Uncharacterized protein C1orf106
CDC42EP1 pS192	[illegible]	p06694	Cdc42 effector protein 1
CDC6 seq: 28 - 47 + Phospho (ST)	[illegible]	p09089	Cell division control protein 6 homolog
CXorf67 seq: 361 - 378 + Phospho (ST)	[illegible]	p05056	Uncharacterized protein CXorf67
HSPA5 seq: 475 - 492 no mod	[illegible]	p18462	78 kDa glucose-regulated protein
RPS2 seq: 228 - 238 no mod	[illegible]	p16235	40S ribosomal protein S2
ALS2 seq: 482 - 494 + Phospho (ST)	[illegible]	p20750	Alsin
MDC1 seq: 960 - 993 + Phospho (ST)	[illegible]	p13415	Mediator of DNA damage checkpoint protein 1
RPS6KA1 pS380	[illegible]	p16095	Ribosomal protein S6 kinase alpha-1
PHIP pS1783	[illegible]	p00388	PH-interacting protein
AHNAK pS5068	[illegible]	p09884	Neuroblast differentiation-associated protein AHNAK
TRIO seq: 234 - 243 no mod	[illegible]	p11994	Triple functional domain protein
SHROOM2 seq: 206 - 226 + Phospho (Y)	[illegible]	p07522	Protein Shroom2
NHSL1 pS1233	[illegible]	p09550	NHS-like protein 1
TMPO seq: 324 - 332 no mod	[illegible]	p18701	Lamina-associated polypeptide 2, isoform alpha
CDK13 seq: 1045 - 1069 + 2 Phospho (ST)	[illegible]	p13336	Cyclin-dependent kinase 13
WDR26 seq: 323 - 340 no mod	[illegible]	p12064	WD repeat-containing protein 26
SHROOM2 seq: 206 - 226 + Phospho (ST)	[illegible]	p07523	Protein Shroom2
FLNB seq: 2476 - 2495 + Phospho (ST)	[illegible]	p10346	Filamin-B
SRRM1 pS874	[illegible]	p13275	Serine/arginine repetitive matrix protein 1
CEP57 seq: 53 - 67 + Phospho (ST)	[illegible]	p04421	Centrosomal protein of 57 kDa
MEPCE seq: 208 - 222 + 2 Phospho (ST)	[illegible]	p02590	7SK snRNA methylphosphate capping enzyme
CASKIN1 pS1364	[illegible]	p13216	Caskin-1
KANK2 pS323	[illegible]	p08872	KN motif and ankyrin repeat domain-containing protein 2
PCDH18 seq: 441 - 449 + Phospho (ST)	[illegible]	p15076	Protocadherin-18
PURB pS304	[illegible]	p16047	Transcriptional activator protein Pur-beta
OSER1 pS82 pS84	[illegible]	p05351	Oxidative stress-responsive serine-rich protein 1
METAP2 seq: 157 - 170 no mod	[illegible]	p20503	Methionine aminopeptidase 2
MLLT4 seq: 1691 - 1710 + Phospho (ST)	[illegible]	p18054	Afadin
ANXA3 pM40	[illegible]	p10025	Annexin A3
RAF1 pS29	[illegible]	p18941	RAF proto-oncogene serine/threonine-protein kinase
MAST2 seq: 1362 - 1377 + Phospho (ST)	[illegible]	p05940	Microtubule-associated serine/threonine-protein kinase 2
ANXA3 seq: 249 - 257 no mod	[illegible]	p09552	Annexin A3
STAT3 pS727	[illegible]	p16696	Signal transducer and activator of transcription 3
NR2C2AP seq: 89 - 112 no mod	[illegible]	p11563	Nuclear receptor 2C2-associated protein
ANO8 pS669	[illegible]	p13757	Anoctamin-8
FAM84A seq: 57 - 70 + Phospho (ST)	[illegible]	p19956	Protein FAM84A
BCLAF1 seq: 225 - 255 + Phospho (ST)	[illegible]	p04588	Bcl-2-associated transcription factor 1
TRIM3 pS7	[illegible]	p07489	Tripartite motif-containing protein 3
SCRIB pS1378	[illegible]	p06634	Protein scribble homolog
RAPH1 seq: 1133 - 1156 + Phospho (ST)	[illegible]	p10505	Ras-associated and pleckstrin homology domains-containing protein 1
CD2AP seq: 505 - 517 + Phospho (ST)	[illegible]	p10661	CD2-associated protein
HOMER3 pS256	[illegible]	p17725	Homer protein homolog 3
PDHA2 pS291	[illegible]	p00324	Pyruvate dehydrogenase E1 component subunit alpha, testis-specific form, mitochondrial
DLG5 pS1115	[illegible]	p06288	Disks large homolog 5
INCENP seq: 127 - 154 + 2 Phospho (ST)	[illegible]	p11215	Inner centromere protein
TPRN seq: 356 - 383 + Phospho (ST)	[illegible]	p11065	Taperin
MYH10 seq: 297 - 308 no mod	[illegible]	p06099	Myosin-10
WDHD1 seq: 319 - 351 no mod	[illegible]	p01026	WD repeat and HMG-box DNA-binding protein 1
DYNC1LI1 pS516	[illegible]	p11704	Cytoplasmic dynein 1 light intermediate chain 1
ESYT1 seq: 1032 - 1040 + Phospho (ST)	[illegible]	p20544	Extended synaptotagmin-1
BCAR3 pS363	[illegible]	p11091	Breast cancer anti-estrogen resistance protein 3
TJP1 seq: 175 - 186 + 2 Phospho (ST)	[illegible]	p20382	Tight junction protein ZO-1
HIST1H2AB seq: 90 - 100 no mod	[illegible]	p00714	Histone H2A type 1-B/E
MAP7D1 pS254	[illegible]	p19012	MAP7 domain-containing protein 1
PHLDB2 pS934	[illegible]	p20529	Pleckstrin homology-like domain family B member 2
RBM17 seq: 222 - 241 + Phospho (ST)	[illegible]	p04242	Splicing factor 45
SPAST seq: 235 - 248 + Phospho (ST)	[illegible]	p07232	Spastin
XRN2 pS499 pS501	[illegible]	p13456	5'-3' exoribonuclease 2
WASL pS430	[illegible]	p12436	Neural Wiskott-Aldrich syndrome protein
NFIC pS339	[illegible]	p12505	Nuclear factor 1 C-type
PHLDB2 seq: 290 - 315 + Phospho (Y)	[illegible]	p19562	Pleckstrin homology-like domain family B member 2
BAIAP2 pT340	[illegible]	p10826	Brain-specific angiogenesis inhibitor 1-associated protein 2
PHKA1 seq: 972 - 996 + Phospho (ST)	[illegible]	p00288	Phosphorylase b kinase regulatory subunit alpha, skeletal muscle isoform
EIF4B seq: 445 - 472 + Phospho (ST)	[illegible]	p04169	Eukaryotic translation initiation factor 4B
SEC16A seq: 11 - 51 + 2 Phospho (ST)	[illegible]	p08205	Protein transport protein Sec16A
SMC3 seq: 707 - 726 no mod	[illegible]	p13907	Structural maintenance of chromosomes protein 3
SMC4 seq: 14 - 38 + Phospho (ST)	[illegible]	p07485	Structural maintenance of chromosomes protein 4
KLF4 seq: 315 - 328 + Phospho (ST)	[illegible]	p20334	Krueppel-like factor 4
EPS15L1 pS229	[illegible]	p01969	Epidermal growth factor receptor substrate 15-like 1
YLPM1 pS581	[illegible]	p01566	YLP motif-containing protein 1
SCAMP3 pS76	[illegible]	p12910	Secretory carrier-associated membrane protein 3
FLNB pS1505	[illegible]	p00462	Filamin-B
XRN2 pS499 pS501	[illegible]	p13455	5'-3' exoribonuclease 2
FAM83B pS664	[illegible]	p05867	Protein FAM83B
LEO1 pT629 pS630	[illegible]	p11884	RNA polymerase-associated protein LEO1
TMCC2 pS438	[illegible]	p19736	Transmembrane and coiled-coil domains protein 2
ECT2 seq: 866 - 877 + Phospho (ST)	[illegible]	p06365	Protein ECT2
PSMB7 seq: 63 - 76 no mod	[illegible]	p19307	Proteasome subunit beta type-7
PARK7 seq: 13 - 27 no mod	[illegible]	p16334	Protein deglycase DJ-1
POM121C pS289	[illegible]	p09936	Nuclear envelope pore membrane protein POM 121C
PLEC seq: 4610 - 4627 + Phospho (ST); Phospho (Y)	[illegible]	p14903	Plectin
TCOF1 pS503	[illegible]	p04448	Treacle protein
LGALSL seq: 14 - 35 + Phospho (ST)	[illegible]	p12093	Galectin-related protein
MICAL3 pS1310	[illegible]	p11689	Protein-methionine sulfoxide oxidase MICAL3
MDC1 pS495 pS498	[illegible]	p20515	Mediator of DNA damage checkpoint protein 1
LLGL2 pS961	[illegible]	p19698	Lethal(2) giant larvae protein homolog 2

HNRNPUL2 seq: 153 - 187 + Phospho (ST)	[illegible]	p17847	Heterogeneous nuclear ribonucleoprotein U-like protein 2
SCRIB pS1475	[illegible]	p00924	Protein scribble homolog
IDH2 seq: 90 - 106 no mod	[illegible]	p18362	Isocitrate dehydrogenase [NADP], mitochondrial
ERRFI1 seq: 118 - 148 + 2 Phospho (ST)	[illegible]	p10462	ERBB receptor feedback inhibitor 1
HNRNPA2B1 seq: 154 - 168 no mod	[illegible]	p16120	Heterogeneous nuclear ribonucleoproteins A2/B1
SEC16A pT1876	[illegible]	p16858	Protein transport protein Sec16A
FARP2 seq: 424 - 445 + Phospho (ST)	[illegible]	p18208	FERM, RhoGEF and pleckstrin domain-containing protein 2
CTTN seq: 415 - 428 + Phospho (ST)	[illegible]	p11070	Src substrate cortactin
ERRFI1 pS326	[illegible]	p00996	ERBB receptor feedback inhibitor 1
MAP1S pS759	[illegible]	p13366	Microtubule-associated protein 1S
RBM17 pS222	[illegible]	p04343	Splicing factor 45
TP53BP1 pS366	[illegible]	p04926	Tumor suppressor p53-binding protein 1
CAP2 pS309	[illegible]	p05551	Adenylyl cyclase-associated protein 2
FAM122B pS58	[illegible]	p14638	Protein FAM122B
CGN pS332	[illegible]	p12396	Cingulin
ULK1 seq: 615 - 626 + Phospho (ST)	[illegible]	p09269	Serine/threonine-protein kinase ULK1
RPLP2 seq: 62 - 94 no mod	[illegible]	p12169	60S acidic ribosomal protein P2
MACF1 seq: 3924 - 3936 + Phospho (ST)	[illegible]	p06814	Microtubule-actin cross-linking factor 1, isoforms 1/2/3/5
RPL27A seq: 66 - 77 no mod	[illegible]	p09214	60S ribosomal protein L27a
PPHLN1 seq: 192 - 216 + 2 Phospho (ST)	[illegible]	p17210	Periphilin-1
TJP2 seq: 168 - 177 + 2 Phospho (ST)	[illegible]	p03995	Tight junction protein ZO-2
P4HB seq: 469 - 502 no mod	[illegible]	p16619	Protein disulfide-isomerase
TNKS1BP1 seq: 976 - 1001 + Phospho (ST)	[illegible]	p05904	182 kDa tankyrase-1-binding protein
HNRNPC pS233	[illegible]	p08796	Heterogeneous nuclear ribonucleoproteins C1/C2
PRKAG2 pS122	[illegible]	p07049	5'-AMP-activated protein kinase subunit gamma-2
TMF1 pS344	[illegible]	p03283	TATA element modulatory factor
RSRC2 pS32	[illegible]	p17268	Arginine/serine-rich coiled-coil protein 2
NAA25 seq: 689 - 695 + Phospho (ST)	[illegible]	p13766	N-alpha-acetyltransferase 25, NatB auxiliary subunit
HNRNPK pS284	[illegible]	p07493	Heterogeneous nuclear ribonucleoprotein K
MAVS seq: 135 - 167 + Phospho (ST)	[illegible]	p17572	Mitochondrial antiviral-signaling protein
NDE1 seq: 304 - 315 + Phospho (ST)	[illegible]	p06840	Nuclear distribution protein nudE homolog 1
PHKB seq: 698 - 720 + Phospho (ST)	[illegible]	p06777	Phosphorylase b kinase regulatory subunit beta
FOSL2 pS200	[illegible]	p04517	Fos-related antigen 2
ZC3H4 pS1269 pS1275	[illegible]	p02794	Zinc finger CCCH domain-containing protein 4
BCLAF1 pS397	[illegible]	p08466	Bcl-2-associated transcription factor 1
KIAA1522 seq: 620 - 666 + Phospho (ST)	[illegible]	p04521	Uncharacterized protein KIAA1522
ABL2 seq: 629 - 640 + Phospho (ST)	[illegible]	p18666	Abelson tyrosine-protein kinase 2
SRRM2 pS1083	[illegible]	p07900	Serine/arginine repetitive matrix protein 2
TMPO seq: 174 - 195 + Phospho (ST)	[illegible]	p08213	Lamina-associated polypeptide 2, isoforms beta/gamma
ACIN1 pS710	[illegible]	p07091	Apoptotic chromatin condensation inducer in the nucleus
S100A10 seq: 38 - 54 no mod	[illegible]	p17792	Protein S100-A10
PHLDB2 seq: 482 - 505 + Phospho (ST)	[illegible]	p17507	Pleckstrin homology-like domain family B member 2
TNKS1BP1 pS1666	[illegible]	p09181	182 kDa tankyrase-1-binding protein
SRRM1 pS450	[illegible]	p07460	Serine/arginine repetitive matrix protein 1
SHARPIN pS165	[illegible]	p04629	Sharpin
UBXN7 seq: 278 - 295 + Phospho (ST)	[illegible]	p05984	UBX domain-containing protein 7
LRBA pS1498	[illegible]	p04320	Lipopolysaccharide-responsive and beige-like anchor protein
SRRM2 seq: 1689 - 1699 + 2 Phospho (ST)	[illegible]	p06576	Serine/arginine repetitive matrix protein 2
C2orf49 pS189 pS193	[illegible]	p12557	Ashwin
CETN2 pS20	[illegible]	p07043	Centrin-2
PSMD4 pS266	[illegible]	p09856	26S proteasome non-ATPase regulatory subunit 4
PDS5B seq: 1356 - 1373 + Phospho (ST)	[illegible]	p20803	Sister chromatid cohesion protein PDS5 homolog B
SSRP1 pM434 pS444	[illegible]	p17712	FACT complex subunit SSRP1
GPATCH8 seq: 976 - 984 + Phospho (ST)	[illegible]	p03416	G patch domain-containing protein 8
DHX38 seq: 523 - 529 + Phospho (ST)	[illegible]	p12579	Pre-mRNA-splicing factor ATP-dependent RNA helicase PRP16
SRRM1 pS549 pS551	[illegible]	p06726	Serine/arginine repetitive matrix protein 1
STAU1 pS390	[illegible]	p00730	Double-stranded RNA-binding protein Staufen homolog 1
SH3BGR seq: 83 - 94 + Gln->pyro-Glu (N-term Q)	[illegible]	p08066	SH3 domain-binding glutamic acid-rich protein
SH3BGRL seq: 20 - 31 + Gln->pyro-Glu (N-term Q)	[illegible]	p08073	SH3 domain-binding glutamic acid-rich-like protein
HNRNPDL seq: 163 - 170 no mod	[illegible]	p18522	Heterogeneous nuclear ribonucleoprotein D-like
ULK1 seq: 705 - 742 + Phospho (ST)	[illegible]	p21160	Serine/threonine-protein kinase ULK1
CAMKK2 pS495	[illegible]	p05909	Calcium/calmodulin-dependent protein kinase kinase 2
BCAR3 pS290	[illegible]	p07111	Breast cancer anti-estrogen resistance protein 3
RPRD2 pS1099	[illegible]	p07047	Regulation of nuclear pre-mRNA domain-containing protein 2
PSME1 pM215 pM217	[illegible]	p11217	Proteasome activator complex subunit 1
RALY seq: 275 - 306 + Phospho (ST)	[illegible]	p02231	RNA-binding protein Raly
PDCD5 pS119	[illegible]	p12421	Programmed cell death protein 5
CXorf67 pS383	[illegible]	p12266	Uncharacterized protein CXorf67
SGK223 pS745	[illegible]	p00789	Tyrosine-protein kinase SgK223
RBM34 pS28	[illegible]	p03294	RNA-binding protein 34
EPN1 seq: 412 - 434 + Phospho (ST)	[illegible]	p03077	Epsin-1
PPP1R13L seq: 100 - 115 + 2 Phospho (ST)	[illegible]	p05997	RelA-associated inhibitor
PWP1 pS50	[illegible]	p11017	Periodic tryptophan protein 1 homolog
HDAC2 pS422 pS424	[illegible]	p14536	Histone deacetylase 2
CHCHD3 pS50	[illegible]	p04228	MICOS complex subunit MIC19
EPPK1 seq: 2713 - 2732 + Phospho (ST)	[illegible]	p06770	Epiplakin
OXSR1 seq: 352 - 361 + Phospho (ST)	[illegible]	p21121	Serine/threonine-protein kinase OSR1
ENO3 seq: 33 - 50 no mod	[illegible]	p20979	Beta-enolase
GIT1 seq: 590 - 613 + Phospho (ST); Phospho (Y)	[illegible]	p14793	ARF GTPase-activating protein GIT1
NF1 seq: 2499 - 2517 + Phospho (ST)	[illegible]	p15298	Neurofibromin
TNRC6B seq: 1488 - 1523 + Phospho (ST)	[illegible]	p15861	Trinucleotide repeat-containing gene 6B protein
CTTN pT401 pS405	[illegible]	p02249	Src substrate cortactin
PSMA3 seq: 242 - 255 + Phospho (ST)	[illegible]	p17149	Proteasome subunit alpha type-3
SRRM1 pS450	[illegible]	p07461	Serine/arginine repetitive matrix protein 1
PLEC seq: 4610 - 4627 + 2 Phospho (ST)	[illegible]	p14905	Plectin
PHIP pS1783	[illegible]	p06543	PH-interacting protein
CFL1 seq: 46 - 73 no mod	[illegible]	p09552	Cofilin-1
NUP98 pS612	[illegible]	p09432	Nuclear pore complex protein Nup98-Nup96
SF3A1 pS329	[illegible]	p00410	Splicing factor 3A subunit 1
HSPH1 seq: 636 - 646 no mod	[illegible]	p09738	Heat shock protein 105 kDa
HNRNPD pS190	[illegible]	p14290	Heterogeneous nuclear ribonucleoprotein D0
PRPF40A seq: 948 - 957 no mod	[illegible]	p02600	Pre-mRNA-processing factor 40 homolog A
MPHOSPH10 pS242	[illegible]	p13352	U3 small nucleolar ribonucleoprotein protein MPP10
ABCF1 pS228	[illegible]	p13445	ATP-binding cassette sub-family F member 1
CAMSAP3 seq: 1067 - 1083 + 2 Phospho (ST)	[illegible]	p06824	Calmodulin-regulated spectrin-associated protein 3
CELSR3 seq: 3066 - 3077 + Phospho (ST)	[illegible]	p00071	Cadherin EGF LAG seven-pass G-type receptor 3
RBM15 seq: 257 - 281 + Phospho (ST); Phospho (Y)	[illegible]	p04033	Putative RNA-binding protein 15
RPS6KA1 seq: 359 - 377 + Phospho (ST)	[illegible]	p02383	Ribosomal protein S6 kinase alpha-1
SRRM2 seq: 950 - 962 + 2 Phospho (ST)	[illegible]	p03997	Serine/arginine repetitive matrix protein 2
SPAG9 seq: 728 - 742 + Phospho (ST)	[illegible]	p06235	C-Jun-amino-terminal kinase-interacting protein 4
TCOF1 pS1257	[illegible]	p08743	Treacle protein
CTNND1 pS288	[illegible]	p04894	Catenin delta-1
STK10 seq: 447 - 464 + Phospho (ST)	[illegible]	p19514	Serine/threonine-protein kinase 10
SCRIB seq: 1323 - 1355 + 2 Phospho (ST)	[illegible]	p20786	Protein scribble homolog
HMGXB4 seq: 99 - 114 + Phospho (ST)	[illegible]	p13031	HMG domain-containing protein 4
SAR1B seq: 28 - 38 no mod	[illegible]	p10432	GTP-binding protein SAR1b
SRRM1 pS874	[illegible]	p13274	Serine/arginine repetitive matrix protein 1
TRA2A pS86 pT88	[illegible]	p03115	Transformer-2 protein homolog alpha
ANXA2P2 seq: 120 - 135 no mod	[illegible]	p15728	Putative annexin A2-like protein
RALGPS2 seq: 314 - 331 + Phospho (ST)	[illegible]	p12521	Ras-specific guanine nucleotide-releasing factor RalGPS2
HSPA2 seq: 455 - 461 no mod	[illegible]	p18435	Heat shock-related 70 kDa protein 2
GPN1 pS338	[illegible]	p15089	GPN-loop GTPase 1
LMNB1 seq: 321 - 330 no mod	[illegible]	p13828	Lamin-B1
DLG5 pS1795	[illegible]	p05744	Disks large homolog 5
FAM129B seq: 686 - 705 + Phospho (ST)	[illegible]	p21078	Niban-like protein 1
VCP seq: 669 - 677 no mod	[illegible]	p18119	Transitional endoplasmic reticulum ATPase
DLST seq: 334 - 345 no mod	[illegible]	p08981	Dihydrolipoyllysine-residue succinyltransferase component of 2-oxoglutarate dehydrogenase complex, mitochondrial
CTTN seq: 397 - 428 + 2 Phospho (ST); Phospho (Y)	[illegible]	p20350	Src substrate cortactin
PAK2 pS197	[illegible]	p03337	Serine/threonine-protein kinase PAK 2
LRRFIP1 pS768	[illegible]	p20302	Leucine-rich repeat flightless-interacting protein 1
PALLD pS1104	[illegible]	p04426	Palladin
AIM1 seq: 515 - 532 + Phospho (ST)	[illegible]	p09961	Absent in melanoma 1 protein
HSP90AB1 seq: 96 - 107 no mod	[illegible]	p20908	Heat shock protein HSP 90-beta
RUVBL1 seq: 172 - 182 no mod	[illegible]	p12056	RuvB-like 1
INF2 seq: 413 - 452 + Phospho (ST)	[illegible]	p20272	Inverted formin-2
SPAG9 seq: 728 - 742 + 2 Phospho (ST)	[illegible]	p06237	C-Jun-amino-terminal kinase-interacting protein 4
MYH9 seq: 1302 - 1322 no mod	[illegible]	p18858	Myosin-9
HSPA5 seq: 307 - 324 no mod	[illegible]	p14352	78 kDa glucose-regulated protein
OSBP pS351	[illegible]	p16212	Oxysterol-binding protein 1
MAPK1 seq: 173 - 191 + Phospho (ST)	[illegible]	p01826	Mitogen-activated protein kinase 1
ZC3H14 pS409	[illegible]	p13684	Zinc finger CCCH domain-containing protein 14
ETV3 pS139	[illegible]	p03875	ETS translocation variant 3
HELZ pS1614	[illegible]	p04512	Probable helicase with zinc finger domain
FOXK2 pS369 pS373	[illegible]	p06305	Forkhead box protein K2
CD2AP seq: 164 - 209 + Phospho (ST)	[illegible]	p17495	CD2-associated protein
SF3B3 seq: 857 - 867 no mod	[illegible]	p20610	Splicing factor 3B subunit 3
LARP1 seq: 845 - 871 + Phospho (ST)	[illegible]	p03030	La-related protein 1
PDHA2 seq: 287 - 300 + Phospho (ST)	[illegible]	p00325	Pyruvate dehydrogenase E1 component subunit alpha, testis-specific form, mitochondrial
WAC seq: 523 - 550 + Phospho (ST)	[illegible]	p04396	WW domain-containing adapter protein with coiled-coil
RANBP2 seq: 3152 - 3177 no mod	[illegible]	p14587	E3 SUMO-protein ligase RanBP2
PPIA pM136 pM142	[illegible]	p17714	Peptidyl-prolyl cis-trans isomerase A
UBR5 pS2486	[illegible]	p03219	E3 ubiquitin-protein ligase UBR5
REPS1 seq: 473 - 502 + Phospho (ST)	[illegible]	p02800	RalBP1-associated Eps domain-containing protein 1
PITPNC1 seq: 299 - 312 + Phospho (ST)	[illegible]	p06292	Cytoplasmic phosphatidylinositol transfer protein 1
CDV3 pS107	[illegible]	p00971	Protein CDV3 homolog
CDK17 pS180	[illegible]	p07575	Cyclin-dependent kinase 17
GOT1 seq: 306 - 325 no mod	[illegible]	p13565	Aspartate aminotransferase, cytoplasmic
CAMKK2 pS511	[illegible]	p05073	Calcium/calmodulin-dependent protein kinase kinase 2
EPS15L1 pS229	[illegible]	p01968	Epidermal growth factor receptor substrate 15-like 1
AFF4 pS549	[illegible]	p08430	AF4/FMR2 family member 4
RNF31 pS466	[illegible]	p07150	E3 ubiquitin-protein ligase RNF31
WDR44 seq: 157 - 178 + Phospho (ST)	[illegible]	p10503	WD repeat-containing protein 44
USP36 pS713	[illegible]	p19377	Ubiquitin carboxyl-terminal hydrolase 36
CENPF pS3119	[illegible]	p12230	Centromere protein F
DKC1 pS494	[illegible]	p20642	H/ACA ribonucleoprotein complex subunit 4
STK3 seq: 382 - 403 + Phospho (ST)	[illegible]	p09739	Serine/threonine-protein kinase 3
FOXK1 seq: 416 - 430 + Oxidation (M); 2 Phospho (ST)	[illegible]	p06299	Forkhead box protein K1
STIP1 seq: 352 - 364 no mod	[illegible]	p12133	Stress-induced-phosphoprotein 1
EIF4G1 seq: 1140 - 1148 + Phospho (ST)	[illegible]	p00580	Eukaryotic translation initiation factor 4 gamma 1
SIPA1L3 pS1544	[illegible]	p02566	Signal-induced proliferation-associated 1-like protein 3
AKAP13 seq: 1904 - 1924 + Phospho (ST)	[illegible]	p03276	A-kinase anchor protein 13
SRSF7 seq: 44 - 57 no mod	[illegible]	p09262	Serine/arginine-rich splicing factor 7
DOCK5 seq: 1800 - 1823 + Phospho (ST)	[illegible]	p02539	Dedicator of cytokinesis protein 5
SNW1 pS224	[illegible]	p15464	SNW domain-containing protein 1
TNKS1BP1 pS712	[illegible]	p18155	182 kDa tankyrase-1-binding protein
ERF seq: 3 - 25 + Phospho (ST)	[illegible]	p02433	ETS domain-containing transcription factor ERF
CREB1 seq: 136 - 150 + Phospho (ST)	[illegible]	p13088	Cyclic AMP-responsive element-binding protein 1
EPS15 pS324	[illegible]	p09559	Epidermal growth factor receptor substrate 15
USP14 pS143 pM146	[illegible]	p00455	Ubiquitin carboxyl-terminal hydrolase 14
GLCCI1 seq: 103 - 123 + Phospho (ST)	[illegible]	p15434	Glucocorticoid-induced transcript 1 protein
CDK16 pS138	[illegible]	p10541	Cyclin-dependent kinase 16
KRT8 seq: 199 - 213 no mod	[illegible]	p18122	Keratin, type II cytoskeletal 8
MAPK1 seq: 173 - 191 + Phospho (Y)	[illegible]	p01824	Mitogen-activated protein kinase 1
SERPINB5 seq: 65 - 79 no mod	[illegible]	p18099	Serpin B5
ZDHHC5 seq: 375 - 392 + Phospho (ST)	[illegible]	p10689	Palmitoyltransferase ZDHHC5
PPFIA1 seq: 237 - 251 + 2 Phospho (ST)	[illegible]	p06625	Liprin-alpha-1
TNKS1BP1 pS1024	[illegible]	p15284	182 kDa tankyrase-1-binding protein
NAV2 pS1319	[illegible]	p00190	Neuron navigator 2
SSH3 pS9	[illegible]	p04504	Protein phosphatase Slingshot homolog 3
MPRIP seq: 1014 - 1025 + Phospho (ST)	[illegible]	p05345	Myosin phosphatase Rho-interacting protein
ZBTB22 pS202	[illegible]	p19633	Zinc finger and BTB domain-containing protein 22
CDK13 seq: 1045 - 1069 + 2 Phospho (ST)	[illegible]	p13337	Cyclin-dependent kinase 13
THRAP3 pS253	[illegible]	p04723	Thyroid hormone receptor-associated protein 3
HDAC1 pS421 pS423	[illegible]	p14533	Histone deacetylase 1
PARD3 seq: 798 - 820 + Phospho (ST)	[illegible]	p21122	Partitioning defective 3 homolog
PXN seq: 302 - 317 + Phospho (ST)	[illegible]	p03813	Paxillin
GIT1 seq: 590 - 613 + 2 Phospho (ST)	[illegible]	p14795	ARF GTPase-activating protein GIT1
PHACTR4 pS443	[illegible]	p20516	Phosphatase and actin regulator 4
YBX3 seq: 34 - 84 + Phospho (ST)	[illegible]	p04316	Y-box-binding protein 3
TNKS1BP1 pS601	[illegible]	p00397	182 kDa tankyrase-1-binding protein
CIC pS173	[illegible]	p02258	Protein capicua homolog
NUMB seq: 224 - 263 + Phospho (ST)	[illegible]	p13519	Protein numb homolog
PPP1R3D seq: 74 - 82 + 2 Phospho (ST)	[illegible]	p05103	Protein phosphatase 1 regulatory subunit 3D
MET seq: 988 - 1004 + Phospho (Y)	[illegible]	p03258	Hepatocyte growth factor receptor
FAM65A seq: 20 - 32 + Phospho (ST)	[illegible]	p04207	Protein FAM65A
PKM seq: 279 - 294 no mod	[illegible]	p07449	Pyruvate kinase PKM
NCAPG pS674	[illegible]	p02605	Condensin complex subunit 3
CDC42BPG pT1511 pS1535	[illegible]	p12699	Serine/threonine-protein kinase MRCK gamma
SRRM1 pT872 pS874	[illegible]	p13277	Serine/arginine repetitive matrix protein 1
PABPN1 pS95	[illegible]	p19957	Polyadenylate-binding protein 2
KLF3 seq: 90 - 106 + 2 Phospho (ST)	[illegible]	p07569	Krueppel-like factor 3
CTTN seq: 397 - 428 + 3 Phospho (ST)	[illegible]	p20348	Src substrate cortactin
FLNB pS2478	[illegible]	p10347	Filamin-B
KRT18 seq: 302 - 314 no mod	[illegible]	p01924	Keratin, type I cytoskeletal 18
NUP153 seq: 327 - 342 + 2 Phospho (ST)	[illegible]	p07262	Nuclear pore complex protein Nup153
FXR2 seq: 438 - 465 + Phospho (ST)	[illegible]	p02817	Fragile X mental retardation syndrome-related protein 2
PKM seq: 142 - 151 no mod	[illegible]	p13599	Pyruvate kinase PKM
SRRM2 pS2132	[illegible]	p04631	Serine/arginine repetitive matrix protein 2
SF3A1 pS329	[illegible]	p16765	Splicing factor 3A subunit 1
ROCK2 seq: 977 - 995 no mod	[illegible]	p02514	Rho-associated protein kinase 2

Peptide	Intensities	Accession	Protein
BRD3 seq: 261 - 271 + Phospho (ST)	[illegible]	p05970	Bromodomain-containing protein 3
MARCKSL1 pT148	[illegible]	p21194	MARCKS-related protein
FHOD1 pS498	[illegible]	p02344	FH1/FH2 domain-containing protein 1
TSC2 pS1132	[illegible]	p04872	Tuberin
TSC22D4 pT211	[illegible]	p20997	TSC22 domain family protein 4
TJP2 pS986	[illegible]	p21017	Tight junction protein ZO-2
TCOF1 seq: 302 - 322 + Phospho (ST)	[illegible]	p21051	Treacle protein
FAM21A pS1075	[illegible]	p15501	WASH complex subunit FAM21A
ILF3 pS382	[illegible]	p06904	Interleukin enhancer-binding factor 3
BAG3 seq: 366 - 388 + 2 Phospho (ST)	[illegible]	p01000	BAG family molecular chaperone regulator 3
CTNNB1 seq: 551 - 565 + Phospho (ST)	[illegible]	p02167	Catenin beta-1
ITIH6 pS175	[illegible]	p07135	Inter-alpha-trypsin inhibitor heavy chain H6
ARID3A pS119	[illegible]	p17705	AT-rich interactive domain-containing protein 3A
TRIM28 pS501	[illegible]	p12073	Transcription intermediary factor 1-beta
ERBB2 pT701	[illegible]	p11310	Receptor tyrosine-protein kinase erbB-2
LIMA1 pS15	[illegible]	p07671	LIM domain and actin-binding protein 1
DCP1A pS142	[illegible]	p18670	mRNA-decapping enzyme 1A
IQSEC2 seq: 727 - 744 + Phospho (ST)	[illegible]	p17041	IQ motif and SEC7 domain-containing protein 2
SHTN1 pS506 pM507	[illegible]	p04891	Shootin-1
CTTN pT401 pS405 pT411	[illegible]	p20349	Src substrate cortactin
SRRM2 seq: 2067 - 2076 + 3 Phospho (ST)	[illegible]	p04942	Serine/arginine repetitive matrix protein 2
NOMO1 seq: 1035 - 1052 no mod	[illegible]	p01397	Nodal modulator 1
SEC31A pS799	[illegible]	p19812	Protein transport protein Sec31A
RGS3 seq: 915 - 932 + Phospho (ST)	[illegible]	p06595	Regulator of G-protein signaling 3
MYL9 seq: 17 - 34 no mod	[illegible]	p19329	Myosin regulatory light polypeptide 9
RNF169 pS693	[illegible]	p13148	E3 ubiquitin-protein ligase RNF169
NCAPG pS674	[illegible]	p02606	Condensin complex subunit 3
FLNB pS2107	[illegible]	p19875	Filamin-B
MKL2 seq: 206 - 233 + Phospho (ST)	[illegible]	p00868	MKL/myocardin-like protein 2
YBX1 pM218	[illegible]	p06868	Nuclease-sensitive element-binding protein 1
CHCHD3 seq: 49 - 64 + Phospho (Y)	[illegible]	p00114	MICOS complex subunit MIC19
TMOD1 seq: 192 - 206 no mod	[illegible]	p09349	Tropomodulin-1
FILIP1L seq: 906 - 943 + Phospho (ST)	[illegible]	p00694	Filamin A-interacting protein 1-like
MCM3 pS672 pT674	[illegible]	p06052	DNA replication licensing factor MCM3
WDR44 pS403	[illegible]	p16883	WD repeat-containing protein 44
EIF5B pS113	[illegible]	p07924	Eukaryotic translation initiation factor 5B
EMSY seq: 206 - 218 + Phospho (ST)	[illegible]	p06515	Protein EMSY
PTK2 seq: 29 - 38 + Phospho (ST)	[illegible]	p04660	Focal adhesion kinase 1
CTNNB1 pS191	[illegible]	p04459	Catenin beta-1
NUCKS1 pS19	[illegible]	p00640	Nuclear ubiquitous casein and cyclin-dependent kinase substrate 1
RANBP2 seq: 2802 - 2822 + Phospho (ST)	[illegible]	p05460	E3 SUMO-protein ligase RanBP2
MEF2D pS251	[illegible]	p01362	Myocyte-specific enhancer factor 2D
NUCKS1 pS58 pS61	[illegible]	p09098	Nuclear ubiquitous casein and cyclin-dependent kinase substrate 1
TNKS1BP1 pS872	[illegible]	p18263	182 kDa tankyrase-1-binding protein
HNRNPH2 seq: 263 - 275 no mod	[illegible]	p18567	Heterogeneous nuclear ribonucleoprotein H2
ATG2B seq: 495 - 508 + Phospho (ST)	[illegible]	p03231	Autophagy-related protein 2 homolog B
ABLIM1 seq: 662 - 686 + Phospho (ST)	[illegible]	p09608	Actin-binding LIM protein 1
JUP pS182	[illegible]	p20221	Junction plakoglobin
GPN1 seq: 311 - 326 + Phospho (ST)	[illegible]	p18252	GPN-loop GTPase 1
SON pS1697	[illegible]	p18752	Protein SON
FOXK1 pS416 pS420 pM427	[illegible]	p06300	Forkhead box protein K1
TCEAL3 pS30	[illegible]	p09683	Transcription elongation factor A protein-like 3
WDR55 pS14	[illegible]	p02990	WD repeat-containing protein 55
PLEC seq: 2199 - 2217 no mod	[illegible]	p17292	Plectin
RASEF pS377	[illegible]	p05195	Ras and EF-hand domain-containing protein
RAI14 seq: 274 - 292 + 2 Phospho (ST)	[illegible]	p13413	Ankycorbin
SRRM1 pS389 pS391 pS393	[illegible]	p07092	Serine/arginine repetitive matrix protein 1
EIF5B pS113	[illegible]	p12649	Eukaryotic translation initiation factor 5B
SCRIB pS1486	[illegible]	p20169	Protein scribble homolog
HNRNPA2B1 seq: 326 - 350 no mod	[illegible]	p09380	Heterogeneous nuclear ribonucleoproteins A2/B1
CLTC pS1494	[illegible]	p02176	Clathrin heavy chain 1
NUP153 seq: 328 - 342 + Phospho (ST)	[illegible]	p13857	Nuclear pore complex protein Nup153
SAP30BP seq: 6 - 38 + Phospho (ST)	[illegible]	p08963	SAP30-binding protein
SAG seq: 19 - 24 + Phospho (ST)	[illegible]	p13067	S-arrestin
CCDC12 seq: 145 - 166 + Phospho (ST)	[illegible]	p15405	Coiled-coil domain-containing protein 12
MAP3K4 pS1274	[illegible]	p03201	Mitogen-activated protein kinase kinase kinase 4
TRPM3 seq: 63 - 67 + Phospho (ST)	[illegible]	p03189	Transient receptor potential cation channel subfamily M member 3
SLITRK3 seq: 245 - 249 + Phospho (ST)	[illegible]	p03187	SLIT and NTRK-like protein 3
UBR5 seq: 636 - 654 + Phospho (ST)	[illegible]	p03491	E3 ubiquitin-protein ligase UBR5
PPP1R2P3 pS121 pS122	[illegible]	p13825	Protein phosphatase inhibitor 2-like protein 3
GTF2F1 seq: 436 - 460 + 2 Phospho (ST)	[illegible]	p03481	General transcription factor IIF subunit 1
RAI14 pS286	[illegible]	p13412	Ankycorbin
ADNP pS953 pS955	[illegible]	p11232	Activity-dependent neuroprotector homeobox protein
NCOA2 pS565	[illegible]	p10106	Nuclear receptor coactivator 2
FASN seq: 968 - 992 + Phospho (ST)	[illegible]	p11908	Fatty acid synthase
TCOF1 pS1378	[illegible]	p11844	Treacle protein
BAIAP2L1 seq: 329 - 340 + Oxidation (M); Phospho (ST)	[illegible]	p03236	Brain-specific angiogenesis inhibitor 1-associated protein 2-like protein 1
CRTC3 seq: 386 - 407 + 2 Phospho (ST)	[illegible]	p06809	CREB-regulated transcription coactivator 3
LAD1 pS356	[illegible]	p13854	Ladinin-1
RBM17 pS155	[illegible]	p06959	Splicing factor 45
NPM1 pS70	[illegible]	p18889	Nucleophosmin
USP14 pS143	[illegible]	p19617	Ubiquitin carboxyl-terminal hydrolase 14
CTNND1 pS346	[illegible]	p15250	Catenin delta-1
NAV2 pS1319	[illegible]	p00191	Neuron navigator 2
NOL3 pT114	[illegible]	p03173	Nucleolar protein 3
ANXA1 seq: 263 - 269 no mod	[illegible]	p19039	Annexin A1
BIN1 seq: 36 - 53 no mod	[illegible]	p20920	Myc box-dependent-interacting protein 1
SLAIN2 pS391	[illegible]	p10749	SLAIN motif-containing protein 2
PPP1R2 pS121 pS122	[illegible]	p00144	Protein phosphatase inhibitor 2
TP53BP1 pS265	[illegible]	p06045	Tumor suppressor p53-binding protein 1
HNRNPK pS116	[illegible]	p13097	Heterogeneous nuclear ribonucleoprotein K
CDK11A pS56	[illegible]	p04488	Cyclin-dependent kinase 11A
KCNK12 pS5	[illegible]	p04442	Potassium channel subfamily K member 12
BCAS3 pS480	[illegible]	p08649	Breast carcinoma-amplified sequence 3
DTX3L pS202	[illegible]	p08064	E3 ubiquitin-protein ligase DTX3L
ACTB pM305	[illegible]	p13333	Actin, cytoplasmic 1
ATL1 pS10	[illegible]	p18345	Atlastin-1
CLIC1 seq: 38 - 49 no mod	[illegible]	p14981	Chloride intracellular channel protein 1
KIAA1671 seq: 1057 - 1086 + 2 Phospho (ST)	[illegible]	p13063	Uncharacterized protein KIAA1671
DIP2B seq: 254 - 272 + Phospho (ST)	[illegible]	p15237	Disco-interacting protein 2 homolog B
PGP seq: 187 - 203 no mod	[illegible]	p00244	Phosphoglycolate phosphatase
AHNAK seq: 5748 - 5765 + Phospho (ST)	[illegible]	p19583	Neuroblast differentiation-associated protein AHNAK
PRPF4B pS87 pS93	[illegible]	p17622	Serine/threonine-protein kinase PRP4 homolog
EPB41 seq: 707 - 715 + Phospho (ST)	[illegible]	p07075	Protein 4.1
CAPZB seq: 196 - 215 + Gln->pyro-Glu (N-term Q)	[illegible]	p08250	F-actin-capping protein subunit beta
DDX5 seq: 33 - 40 no mod	[illegible]	p13246	Probable ATP-dependent RNA helicase DDX5
ZMYND8 pS406	[illegible]	p11191	Protein kinase C-binding protein 1
NOP58 pS502 pS514	[illegible]	p14770	Nucleolar protein 58
NEDD4 seq: 724 - 744 + Phospho (ST)	[illegible]	p10546	E3 ubiquitin-protein ligase NEDD4
MAF1 seq: 46 - 71 + Phospho (ST)	[illegible]	p08695	Repressor of RNA polymerase III transcription MAF1 homolog
JUND seq: 236 - 268 + Phospho (ST)	[illegible]	p12307	Transcription factor jun-D
CUX1 seq: 1452 - 1469 + Phospho (ST)	[illegible]	p06847	Homeobox protein cut-like 1
CDC42EP3 pS89	[illegible]	p20022	Cdc42 effector protein 3
CACNA1C seq: 677 - 685 + Oxidation (M)	[illegible]	p16565	Voltage-dependent L-type calcium channel subunit alpha-1C
PLEKHA5 pS885	[illegible]	p06178	Pleckstrin homology domain-containing family A member 5
LCP1 pS5	[illegible]	p15144	Plastin-2
TJP1 seq: 1613 - 1637 + Phospho (ST)	[illegible]	p20440	Tight junction protein ZO-1
PHF2 pS899	[illegible]	p13166	Lysine-specific demethylase PHF2
MYH9 seq: 1302 - 1322 no mod	[illegible]	p18857	Myosin-9
SOS1 pS1134	[illegible]	p06210	Son of sevenless homolog 1
ENO1 seq: 240 - 253 no mod	[illegible]	p00616	Alpha-enolase
SRRM2 pS2702	[illegible]	p06856	Serine/arginine repetitive matrix protein 2
RBM15B pS109	[illegible]	p19619	Putative RNA-binding protein 15B
GLO1 seq: 142 - 148 no mod	[illegible]	p16799	Lactoylglutathione lyase
CAMSAP1 pS1080	[illegible]	p19863	Calmodulin-regulated spectrin-associated protein 1
TRIM16 seq: 51 - 73 + Phospho (ST)	[illegible]	p11707	Tripartite motif-containing protein 16
KRT8 pS24	[illegible]	p03113	Keratin, type II cytoskeletal 8
FAM129B seq: 619 - 654 + Phospho (ST)	[illegible]	p20376	Niban-like protein 1
EEF2 pT57	[illegible]	p20678	Elongation factor 2
ZC3HAV1 seq: 271 - 295 + Phospho (ST)	[illegible]	p06125	Zinc finger CCCH-type antiviral protein 1
EPPK1 seq: 1279 - 1297 no mod	[illegible]	p11438	Epiplakin
KIF21A seq: 851 - 875 + Phospho (ST)	[illegible]	p12903	Kinesin-like protein KIF21A
ANK3 seq: 512 - 541 + Phospho (Y)	[illegible]	p20910	Ankyrin-3
PDAP1 pS60 pS63	[illegible]	p05278	28 kDa heat- and acid-stable phosphoprotein
FOSL2 seq: 15 - 35 + Phospho (ST)	[illegible]	p15174	Fos-related antigen 2
AKAP13 seq: 1265 - 1290 + Phospho (ST)	[illegible]	p21154	A-kinase anchor protein 13
RPRD1B seq: 127 - 138 + Phospho (ST)	[illegible]	p10732	Regulation of nuclear pre-mRNA domain-containing protein 1B
PPP1R13L pS567	[illegible]	p14817	RelA-associated inhibitor
GAPDH pM133	[illegible]	p01379	Glyceraldehyde-3-phosphate dehydrogenase
EEF1D pT147 pS162	[illegible]	p12813	Elongation factor 1-delta
HNRNPA2B1 seq: 23 - 38 no mod	[illegible]	p11886	Heterogeneous nuclear ribonucleoproteins A2/B1
UBAP1 seq: 265 - 279 + Phospho (ST)	[illegible]	p05060	Ubiquitin-associated protein 1
REPS1 seq: 270 - 280 + Phospho (ST)	[illegible]	p06779	RalBP1-associated Eps domain-containing protein 1
EPS15 pS814	[illegible]	p11202	Epidermal growth factor receptor substrate 15
TANC2 seq: 1973 - 1982 + Phospho (ST)	[illegible]	p08619	Protein TANC2
NEDD4L seq: 472 - 493 + Phospho (ST)	[illegible]	p18167	E3 ubiquitin-protein ligase NEDD4-like
TRIM28 seq: 471 - 483 + Phospho (ST)	[illegible]	p04054	Transcription intermediary factor 1-beta
RNPC3 pS108	[illegible]	p17294	RNA-binding protein 40
ZMYND8 pS425	[illegible]	p07249	Protein kinase C-binding protein 1
HNRNPM pS575	[illegible]	p10133	Heterogeneous nuclear ribonucleoprotein M
HNRNPH1 pS63	[illegible]	p17689	Heterogeneous nuclear ribonucleoprotein H
RALY seq: 275 - 306 + Phospho (ST)	[illegible]	p02232	RNA-binding protein Raly
TBC1D10B pS132	[illegible]	p20340	TBC1 domain family member 10B
CHCHD3 seq: 49 - 64 + Phospho (ST)	[illegible]	p00115	MICOS complex subunit MIC19
PCYT1A seq: 310 - 317 + Phospho (ST)	[illegible]	p10008	Choline-phosphate cytidylyltransferase A
PCYT1B pS315	[illegible]	p10005	Choline-phosphate cytidylyltransferase B
SRGAP1 pS1005	[illegible]	p09106	SLIT-ROBO Rho GTPase-activating protein 1
CTR9 pS970	[illegible]	p16176	RNA polymerase-associated protein CTR9 homolog
MYH9 seq: 1755 - 1770 no mod	[illegible]	p20048	Myosin-9
UBXN1 seq: 179 - 208 + Phospho (ST)	[illegible]	p00370	UBX domain-containing protein 1
DENND4C seq: 1040 - 1059 + Phospho (ST)	[illegible]	p06603	DENN domain-containing protein 4C
TPX2 pT72	[illegible]	p20047	Targeting protein for Xklp2
TRIM28 seq: 471 - 483 + Phospho (ST)	[illegible]	p04055	Transcription intermediary factor 1-beta
CHD8 seq: 1962 - 1979 + Phospho (ST)	[illegible]	p09975	Chromodomain-helicase-DNA-binding protein 8
FHOD1 pS523	[illegible]	p17308	FH1/FH2 domain-containing protein 1
ZDHHC5 seq: 415 - 426 + Phospho (ST)	[illegible]	p05903	Palmitoyltransferase ZDHHC5
KDM2A pT550 pS558	[illegible]	p10514	Lysine-specific demethylase 2A
CBX3 seq: 91 - 103 + Phospho (ST)	[illegible]	p07210	Chromobox protein homolog 3
FAM53C pS234	[illegible]	p16463	Protein FAM53C
ERBB2 pS998	[illegible]	p16375	Receptor tyrosine-protein kinase erbB-2
MAP1S pS759	[illegible]	p19210	Microtubule-associated protein 1S
NUB1 seq: 59 - 64 no mod	[illegible]	p01403	NEDD8 ultimate buster 1
ERCC2 seq: 83 - 88 no mod	[illegible]	p01402	TFIIH basal transcription factor complex helicase XPD subunit
CHMP5 seq: 80 - 100 no mod	[illegible]	p18458	Charged multivesicular body protein 5
FLNB pS983	[illegible]	p12020	Filamin-B
ERRFI1 seq: 249 - 263 + Phospho (ST)	[illegible]	p05580	ERBB receptor feedback inhibitor 1
CAPZA2 seq: 20 - 37 no mod	[illegible]	p16681	F-actin-capping protein subunit alpha-2
SF3B1 seq: 431 - 451 + Phospho (ST)	[illegible]	p10580	Splicing factor 3B subunit 1
HGS seq: 211 - 221 no mod	[illegible]	p01719	Hepatocyte growth factor-regulated tyrosine kinase substrate
RERE pS656	[illegible]	p01767	Arginine-glutamic acid dipeptide repeats protein
NELFE seq: 49 - 66 + Phospho (ST)	[illegible]	p05063	Negative elongation factor E
SMG7 pS897	[illegible]	p04600	Protein SMG7
SCRIB pS1140	[illegible]	p18392	Protein scribble homolog
IQSEC2 pM378	[illegible]	p07268	IQ motif and SEC7 domain-containing protein 2
HSPA2 seq: 305 - 314 no mod	[illegible]	p16798	Heat shock-related 70 kDa protein 2
SEC16A seq: 408 - 437 + 2 Phospho (ST)	[illegible]	p15143	Protein transport protein Sec16A
SRRM1 pS450	[illegible]	p17135	Serine/arginine repetitive matrix protein 1
CTTN pS418	[illegible]	p11071	Src substrate cortactin
MAP1B seq: 1791 - 1810 + Phospho (ST)	[illegible]	p17113	Microtubule-associated protein 1B
FLNB pS827	[illegible]	p01235	Filamin-B
TJP2 seq: 168 - 177 + 2 Phospho (ST)	[illegible]	p03994	Tight junction protein ZO-2
CDCA3 pS199 pS209	[illegible]	p04538	Cell division cycle-associated protein 3
HNRNPA2B1 seq: 138 - 147 no mod	[illegible]	p14390	Heterogeneous nuclear ribonucleoproteins A2/B1
BAG3 seq: 366 - 388 + 2 Phospho (ST)	[illegible]	p01001	BAG family molecular chaperone regulator 3
ZZEF1 pS1518	[illegible]	p11069	Zinc finger ZZ-type and EF-hand domain-containing protein 1
NCOA3 seq: 562 - 590 + Phospho (ST)	[illegible]	p00813	Nuclear receptor coactivator 3
NCOA3 seq: 562 - 590 + Phospho (Y)	[illegible]	p00812	Nuclear receptor coactivator 3
ZNF609 seq: 411 - 431 + Phospho (ST)	[illegible]	p07320	Zinc finger protein 609
EIF5B pS113	[illegible]	p07922	Eukaryotic translation initiation factor 5B
BCAR1 pS428	[illegible]	p07147	Breast cancer anti-estrogen resistance protein 1
SRRM2 seq: 950 - 962 + 2 Phospho (ST)	[illegible]	p03998	Serine/arginine repetitive matrix protein 2
PROSC seq: 242 - 250 no mod	[illegible]	p14227	Proline synthase co-transcribed bacterial homolog protein
MYH9 seq: 1302 - 1322 no mod	[illegible]	p18859	Myosin-9
TOM1 pS355	[illegible]	p20626	Target of Myb protein 1
FAM47E pS265	[illegible]	p06548	Protein FAM47E
PLSCR3 seq: 232 - 238 + Phospho (ST)	[illegible]	p03341	Phospholipid scramblase 3
SAP30L seq: 92 - 120 + Phospho (ST)	[illegible]	p02193	Histone deacetylase complex subunit SAP30L
PAK4 pS181	[illegible]	p06910	Serine/threonine-protein kinase PAK 4
CRTC3 seq: 429 - 463 + Phospho (ST)	[illegible]	p04237	CREB-regulated transcription coactivator 3
AHNAK pS2397	[illegible]	p13700	Neuroblast differentiation-associated protein AHNAK

Peptide	Value 1	Value 2	Intermediate values	Last value	Accession	Protein
NDRG2 seq: 219 - 240 no mod	5E+06	2E+07	[illegible]	3E+07	p09547	Protein NDRG2
GPATCH8 seq: 1009 - 1018 + Phospho (ST)	7E+07	5E+07	[illegible]	1E+08	p03199	G patch domain-containing protein 8
ARHGDIA seq: 34 - 49 no mod	3E+07	2E+07	[illegible]	6E+07	p05489	Rho GDP-dissociation inhibitor 1
KDM3B pS798	3E+07	5E+07	[illegible]	6E+07	p07426	Lysine-specific demethylase 3B
PTPN12 seq: 565 - 592 + Phospho (ST)	2E+08	2E+08	[illegible]	25732	p01902	Tyrosine-protein phosphatase non-receptor type 12
CRTC3 seq: 429 - 463 + Phospho (Y)	2E+08	4E+08	[illegible]	4E+08	p04236	CREB-regulated transcription coactivator 3
YBX3 seq: 34 - 85 + Phospho (ST)	1E+09	7E+08	[illegible]	2E+09	p04315	Y-box-binding protein 3
FAM129B seq: 686 - 705 + 2 Phospho (ST)	5E+09	4E+09	[illegible]	4E+10	p21079	Niban-like protein 1
FOXO3 seq: 420 - 444 + Phospho (ST)	2E+07	1E+08	[illegible]	2E+08	p15278	Forkhead box protein O3
CALD1 seq: 782 - 793 + Phospho (ST)	6E+07	4E+07	[illegible]	5E+08	p07820	Caldesmon
SLTM pS289	1E+08	2E+08	[illegible]	1E+08	p18802	SAFB-like transcription modulator
NELFE seq: 113 - 125 + Phospho (ST)	1E+08	2E+08	[illegible]	3E+08	p05482	Negative elongation factor E
RALBP1 pS645	2E+09	2E+09	[illegible]	1E+09	p20645	RalA-binding protein 1
SIPA1 pS55	38324	37149	[illegible]	3E+08	p05697	Signal-induced proliferation-associated protein 1
RB1CC1 seq: 237 - 248 + Phospho (ST)	2E+08	2E+08	[illegible]	3E+08	p03542	RB1-inducible coiled-coil protein 1
EPS15 pS796	2E+08	1E+08	[illegible]	5E+07	p12043	Epidermal growth factor receptor substrate 15
MAPT pS579	4E+08	4E+08	[illegible]	25732	p14229	Microtubule-associated protein tau
HMGN1 seq: 5 - 18 + Phospho (ST)	7E+07	2E+08	[illegible]	3E+08	p12394	Non-histone chromosomal protein HMG-14
CCDC6 pS240 pS244	2E+08	7E+07	[illegible]	1E+08	p12049	Coiled-coil domain-containing protein 6
MICALL1 pM574 pS578	5E+07	4E+07	[illegible]	2E+08	p01600	MICAL-like protein 1
RPL17 seq: 4 - 13 no mod	5E+07	1E+08	[illegible]	1E+08	p00104	60S ribosomal protein L17
LIMA1 pS686 pS698	4E+08	3E+08	[illegible]	1E+09	p17740	LIM domain and actin-binding protein 1
SCRIB pT1342 pS1348	1E+08	2E+08	[illegible]	1E+09	p20787	Protein scribble homolog
KRT18 seq: 302 - 314 no mod	38324	4E+07	[illegible]	5E+07	p01923	Keratin, type I cytoskeletal 18
CTNND1 pS349	3E+08	3E+08	[illegible]	9E+08	p15251	Catenin delta-1
FAM49B seq: 168 - 186 no mod	3E+07	37149	[illegible]	7E+07	p13905	Protein FAM49B
TNRC6C seq: 1009 - 1040 + Phospho (ST)	6E+07	8E+07	[illegible]	25732	p17107	Trinucleotide repeat-containing gene 6C protein
TNKS1BP1 seq: 859 - 879 + Phospho (ST)	38324	37149	[illegible]	2E+07	p18995	182 kDa tankyrase-1-binding protein
[illegible]seq: 145 - 173 + Gln->pyro-Glu (N-term Q); Phospho (ST)	5E+09	3E+09	[illegible]	4E+09	p08734	UV excision repair protein RAD23 homolog B
CTSD pM219	38324	6E+07	[illegible]	7E+07	p13632	Cathepsin D
HNRNPA2B1 seq: 130 - 147 no mod	2E+08	7E+08	[illegible]	4E+08	p18049	Heterogeneous nuclear ribonucleoproteins A2/B1
AKAP13 pS1876	1E+07	1E+08	[illegible]	1E+08	p06173	A-kinase anchor protein 13
SH3BP2 pS416	9E+07	5E+07	[illegible]	7E+06	p04510	SH3 domain-binding protein 2
PPP1R2 pS121 pS122	2E+08	3E+08	[illegible]	6E+08	p13826	Protein phosphatase inhibitor 2
EDC3 pS161	1E+08	7E+07	[illegible]	9E+08	p14701	Enhancer of mRNA-decapping protein 3
PPIB seq: 205 - 216 no mod	6E+07	9E+07	[illegible]	7E+07	p14325	Peptidyl-prolyl cis-trans isomerase B
TNKS1BP1 pS601	1E+09	9E+08	[illegible]	8E+08	p00398	182 kDa tankyrase-1-binding protein
SCRIB seq: 1416 - 1443 + Phospho (ST)	7E+08	9E+08	[illegible]	1E+09	p12244	Protein scribble homolog
RPL19 pS13	38324	5E+06	[illegible]	2E+06	p12177	60S ribosomal protein L19
TOM1 pS160	38324	8E+06	[illegible]	2E+07	p13186	Target of Myb protein 1
MDC1 seq: 321 - 348 + Phospho (ST)	1E+07	2E+08	[illegible]	1E+09	p19781	Mediator of DNA damage checkpoint protein 1
SLC39A3 seq: 222 - 227 + Oxidation (M); Phospho (ST)	9E+07	5E+07	[illegible]	4E+07	p06309	Zinc transporter ZIP3
MOGAT3 seq: 77 - 81 + Phospho (ST)	9E+07	5E+07	[illegible]	4E+07	p05942	2-acylglycerol O-acyltransferase 3
CCDC158 seq: 958 - 963 + Phospho (ST)	9E+07	5E+07	[illegible]	4E+07	p04828	Coiled-coil domain-containing protein 158
BNIP2 seq: 112 - 128 + Phospho (ST)	38324	1E+07	[illegible]	1E+08	p13159	BCL2/adenovirus E1B 19 kDa protein-interacting protein 2
KIAA1217 seq: 351 - 370 + 2 Phospho (ST)	4E+08	4E+08	[illegible]	2E+08	p13658	Sickle tail protein homolog
MATR3 pS188	1E+08	2E+08	[illegible]	7E+08	p07520	Matrin-3
CDC42EP4 pS64	6E+07	6E+07	[illegible]	25732	p20689	Cdc42 effector protein 4
ZNF185 seq: 430 - 450 + Phospho (ST)	1E+08	2E+07	[illegible]	1E+08	p15988	Zinc finger protein 185
SRRM1 pS431	2E+08	3E+08	[illegible]	3E+08	p00757	Serine/arginine repetitive matrix protein 1
LMNA pS458	1E+08	7E+07	[illegible]	4E+08	p09494	Prelamin-A/C
IRF2BP1 pS436	4E+07	4E+07	[illegible]	25732	p08990	Interferon regulatory factor 2-binding protein 1
IRS2 pS1100	3E+08	4E+08	[illegible]	6E+08	p01752	Insulin receptor substrate 2
RB1CC1 seq: 249 - 260 + Phospho (ST)	1E+08	9E+07	[illegible]	1E+08	p02043	RB1-inducible coiled-coil protein 1
PDLIM2 pS197	7E+07	1E+08	[illegible]	2E+09	p20697	PDZ and LIM domain protein 2
TCF20 pS538	38324	37149	[illegible]	7E+07	p08314	Transcription factor 20
WEE1 pS139	2E+08	2E+08	[illegible]	1E+08	p04734	Wee1-like protein kinase
ATF2 pS112	1E+07	7E+07	[illegible]	1E+07	p12864	Cyclic AMP-dependent transcription factor ATF-2
REPS1 seq: 141 - 152 + Phospho (ST)	3E+08	4E+08	[illegible]	1E+09	p13147	RalBP1-associated Eps domain-containing protein 1
SRRM2 seq: 2067 - 2076 + 2 Phospho (ST)	8E+07	5E+07	[illegible]	2E+08	p04943	Serine/arginine repetitive matrix protein 2
FAM129B seq: 686 - 705 + 2 Phospho (ST)	8E+08	6E+08	[illegible]	7E+09	p21080	Niban-like protein 1
ZMYM2 seq: 830 - 846 + Phospho (ST)	38324	37149	[illegible]	7E+07	p09858	Zinc finger MYM-type protein 2
GPHN pS188 pS194	5E+08	5E+08	[illegible]	2E+09	p01321	Gephyrin
MET seq: 988 - 1004 + Phospho (Y)	38324	37149	[illegible]	1E+08	p03257	Hepatocyte growth factor receptor
STAT3 seq: 181 - 197 no mod	2E+08	1E+08	[illegible]	1E+08	p04254	Signal transducer and activator of transcription 3
IWS1 pS398 pS400	1E+07	2E+07	[illegible]	6E+07	p20983	Protein IWS1 homolog
GAB2 pS543	38324	3E+07	[illegible]	8E+08	p09286	GRB2-associated-binding protein 2
ENDOD1 seq: 257 - 278 no mod	7E+07	2E+08	[illegible]	1E+08	p11333	Endonuclease domain-containing 1 protein
ILF3 pT486 pS503	3E+08	2E+08	[illegible]	25732	p18214	Interleukin enhancer-binding factor 3
HDGF pS132 pS133	3E+09	3E+09	[illegible]	1E+09	p15599	Hepatoma-derived growth factor
STK3 pS316	3E+08	3E+08	[illegible]	2E+08	p17512	Serine/threonine-protein kinase 3
PPP6R1 seq: 729 - 763 + Phospho (ST)	2E+08	2E+08	[illegible]	6E+08	p00862	Serine/threonine-protein phosphatase 6 regulatory subunit 1
PLEKHA5 seq: 53 - 72 + Phospho (ST)	7E+07	4E+07	[illegible]	4E+07	p06776	Pleckstrin homology domain-containing family A member 5
ARMCX3 pS61	38324	2E+07	[illegible]	25732	p00209	Armadillo repeat-containing X-linked protein 3
RBMX2 seq: 178 - 190 + Phospho (ST)	2E+07	2E+07	[illegible]	2E+08	p16959	RNA-binding motif protein, X-linked 2
SGTA pS77 pT81	2E+08	2E+08	[illegible]	3E+08	p17356	Small glutamine-rich tetratricopeptide repeat-containing protein alpha
IRS2 pT520	4E+07	3E+07	[illegible]	25732	p04741	Insulin receptor substrate 2
NIPBL seq: 365 - 402 + Phospho (ST)	2E+08	2E+08	[illegible]	1E+09	p00889	Nipped-B-like protein
EEF1D pT147 pS162	1E+09	1E+09	[illegible]	4E+09	p12814	Elongation factor 1-delta
PJA1 pS265	1E+08	1E+08	[illegible]	2E+08	p09482	E3 ubiquitin-protein ligase Praja-1
AHNAK seq: 4096 - 4111 + Phospho (ST)	6E+07	5E+07	[illegible]	1E+07	p01684	Neuroblast differentiation-associated protein AHNAK
RASAL2 seq: 751 - 763 + Phospho (ST)	38324	37149	[illegible]	1E+08	p17042	Ras GTPase-activating protein nGAP
IWS1 pS438 pS440	1E+08	2E+08	[illegible]	5E+08	p02726	Protein IWS1 homolog
ARFGAP1 seq: 298 - 339 + Phospho (ST)	1E+07	4E+07	[illegible]	2E+08	p20833	ADP-ribosylation factor GTPase-activating protein 1
ACIN1 seq: 560 - 569 + Phospho (ST)	3E+07	4E+07	[illegible]	3E+07	p19598	Apoptotic chromatin condensation inducer in the nucleus
NSFL1C seq: 77 - 93 no mod	3E+07	37149	[illegible]	4E+07	p18599	NSFL1 cofactor p47
CFL1 seq: 54 - 73 no mod	8E+07	1E+08	[illegible]	1E+08	p17608	Cofilin-1
RBM12B pS839	9E+07	2E+07	[illegible]	25732	p04470	RNA-binding protein 12B
CDC27 pS364	5E+07	5E+07	[illegible]	1E+08	p16939	Cell division cycle protein 27 homolog
HNRNPU pS59	6E+08	5E+08	[illegible]	2E+08	p11059	Heterogeneous nuclear ribonucleoprotein U
CEP170B pS772	38324	3E+07	[illegible]	1E+08	p04469	Centrosomal protein of 170 kDa protein B
TCF20 seq: 556 - 570 + Phospho (ST)	5E+07	1E+08	[illegible]	3E+08	p20555	Transcription factor 20
HNRNPD seq: 68 - 85 + Phospho (ST)	8E+09	7E+09	[illegible]	9E+09	p14447	Heterogeneous nuclear ribonucleoprotein D0
FLNA seq: 2311 - 2333 no mod	3E+07	3E+07	[illegible]	2E+08	p16568	Filamin-A
STMN1 pS25	1E+09	1E+09	[illegible]	7E+09	p19606	Stathmin
MDH2 pM315	9E+07	2E+08	[illegible]	7E+07	p10064	Malate dehydrogenase, mitochondrial
TBX3 pS371 pS375	6E+07	3E+07	[illegible]	25732	p18645	T-box transcription factor TBX3
ERRFI1 seq: 118 - 134 + Phospho (ST)	38324	37149	[illegible]	3E+07	p10463	ERBB receptor feedback inhibitor 1
MFAP1 pS132 pS133	38324	37149	[illegible]	2E+08	p10176	Microfibrillar-associated protein 1
DDX20 seq: 694 - 720 + Phospho (ST)	3E+08	2E+08	[illegible]	5E+08	p13717	Probable ATP-dependent RNA helicase DDX20
SPRY4 pS125	9E+07	1E+08	[illegible]	1E+07	p11452	Protein sprouty homolog 4
HSP90B1 seq: 44 - 67 no mod	5E+07	2E+08	[illegible]	3E+08	p02973	Endoplasmin
AHNAK pM3417 pS3426	4E+07	1E+08	[illegible]	4E+08	p00819	Neuroblast differentiation-associated protein AHNAK
LCP1 seq: 4 - 15 + Oxidation (M); Phospho (ST)	38324	37149	[illegible]	1E+09	p15145	Plastin-2
ARHGEF35 pS450	4E+07	4E+07	[illegible]	9E+06	p20851	Rho guanine nucleotide exchange factor 35
FAM53C pS232 pS234	2E+08	2E+08	[illegible]	5E+08	p07421	Protein FAM53C
GAPDH seq: 87 - 107 no mod	2E+08	9E+08	[illegible]	4E+08	p00502	Glyceraldehyde-3-phosphate dehydrogenase
SVIL seq: 1221 - 1239 + Phospho (ST)	38324	1E+07	[illegible]	8E+07	p13189	Supervillin
NCOA2 pS565	1E+08	1E+08	[illegible]	1E+08	p10107	Nuclear receptor coactivator 2
ENO1 seq: 307 - 326 no mod	38324	1E+08	[illegible]	5E+08	p16433	Alpha-enolase
PTPN12 seq: 564 - 592 + Phospho (ST)	7E+07	2E+08	[illegible]	3E+07	p12457	Tyrosine-protein phosphatase non-receptor type 12
HNRNPUL2 seq: 153 - 187 + Phospho (ST)	4E+07	37149	[illegible]	1E+08	p17848	Heterogeneous nuclear ribonucleoprotein U-like protein 2
TJP2 pS1159	7E+07	5E+07	[illegible]	2E+08	p15134	Tight junction protein ZO-2
TUBB8 seq: 298 - 306 no mod	9E+07	2E+08	[illegible]	2E+08	p09370	Tubulin beta-8 chain
PPP1R12A seq: 504 - 517 + Phospho (ST)	8E+06	2E+07	[illegible]	5E+08	p07194	Protein phosphatase 1 regulatory subunit 12A
PRKAB1 pS108	5E+07	7E+07	[illegible]	4E+08	p05589	5'-AMP-activated protein kinase subunit beta-1
LGALS1 seq: 50 - 64 no mod	38324	37149	[illegible]	3E+07	p16575	Galectin-1
NUMA1 seq: 1763 - 1781 + 2 Phospho (ST)	6E+06	2E+07	[illegible]	1E+08	p11100	Nuclear mitotic apparatus protein 1
CARHSP1 pS30 pS32	7E+08	4E+08	[illegible]	3E+08	p17198	Calcium-regulated heat stable protein 1
LRRFIP1 seq: 99 - 118 + Phospho (ST)	4E+08	5E+08	[illegible]	4E+08	p09363	Leucine-rich repeat flightless-interacting protein 1
RALY pT286 pS288	1E+08	1E+08	[illegible]	3E+08	p02233	RNA-binding protein Raly
GAPDH seq: 85 - 107 no mod	2E+07	2E+08	[illegible]	2E+08	p14100	Glyceraldehyde-3-phosphate dehydrogenase
ILF3 pM374 pS382	3E+08	3E+08	[illegible]	7E+06	p06908	Interleukin enhancer-binding factor 3
ANXA1 seq: 59 - 72 no mod	38324	37149	[illegible]	6E+07	p15057	Annexin A1
ZYX pS344	1E+09	1E+09	[illegible]	3E+09	p04659	Zyxin
GAPDH seq: 87 - 107 no mod	2E+08	1E+09	[illegible]	4E+08	p00501	Glyceraldehyde-3-phosphate dehydrogenase
UTP18 seq: 139 - 159 no mod	2E+08	3E+08	[illegible]	2E+08	p12702	U3 small nucleolar RNA-associated protein 18 homolog
SRSF10 seq: 131 - 139 + Phospho (ST)	1E+08	8E+07	[illegible]	1E+08	p04061	Serine/arginine-rich splicing factor 10
ARHGAP1 pS51	2E+09	1E+09	[illegible]	5E+08	p03670	Rho GTPase-activating protein 1
CPSF6 seq: 407 - 420 no mod	1E+07	4E+07	[illegible]	2E+07	p02375	Cleavage and polyadenylation specificity factor subunit 6
CDKN1B pS10	2E+08	3E+08	[illegible]	1E+09	p00815	Cyclin-dependent kinase inhibitor 1B
TMPO seq: 151 - 173 + 2 Phospho (ST)	8E+07	2E+08	[illegible]	1E+08	p17285	Lamina-associated polypeptide 2, isoform alpha
KARS pM556	2E+08	3E+08	[illegible]	5E+08	p01787	Lysine--tRNA ligase
DPF2 pS142	6E+08	5E+08	[illegible]	1E+08	p01699	Zinc finger protein ubi-d4
ROCK1 pS1105	4E+08	3E+08	[illegible]	1E+08	p11447	Rho-associated protein kinase 1
GNL1 seq: 44 - 61 + Phospho (ST)	2E+08	7E+07	[illegible]	25732	p07483	Guanine nucleotide-binding protein-like 1
DPYSL2 pT509	3E+08	3E+08	[illegible]	3E+09	p15615	Dihydropyrimidinase-related protein 2
PLEKHA6 pS808	4E+08	4E+08	[illegible]	1E+08	p06177	Pleckstrin homology domain-containing family A member 6
VCL pS721	2E+08	1E+08	[illegible]	1E+07	p05172	Vinculin
CDC42BPG seq: 1508 - 1539 + 2 Phospho (ST)	9E+07	1E+08	[illegible]	2E+08	p12700	Serine/threonine-protein kinase MRCK gamma
ETFB seq: 60 - 76 no mod	2E+07	2E+07	[illegible]	6E+07	p16982	Electron transfer flavoprotein subunit beta
SCEL pS312	38324	37149	[illegible]	3E+08	p15858	Sciellin
PDIA3 seq: 367 - 395 no mod	3E+08	8E+08	[illegible]	3E+09	p06008	Protein disulfide-isomerase A3
RAB11A seq: 76 - 83 + Phospho (ST)	1E+07	2E+07	[illegible]	2E+07	p20420	Ras-related protein Rab-11A
ELF4 seq: 147 - 161 + Phospho (ST)	38324	37149	[illegible]	4E+07	p20696	ETS-related transcription factor Elf-4
KRT18 pS305	3E+06	6E+06	[illegible]	25732	p01920	Keratin, type I cytoskeletal 18
AKAP8 pS339	3E+07	5E+07	[illegible]	25732	p05778	A-kinase anchor protein 8
PPP1CA seq: 123 - 132 no mod	4E+08	3E+08	[illegible]	4E+09	p15586	Serine/threonine-protein phosphatase PP1-alpha catalytic subunit
USP15 seq: 950 - 981 + 2 Phospho (ST)	38324	37149	[illegible]	5E+07	p16269	Ubiquitin carboxyl-terminal hydrolase 15
TTK seq: 811 - 827 + Phospho (ST)	1E+07	4E+07	[illegible]	3E+07	p00019	Dual specificity protein kinase TTK
RSRC2 seq: 216 - 227 + 3 Phospho (ST)	2E+08	1E+08	[illegible]	4E+08	p04997	Arginine/serine-rich coiled-coil protein 2
IQSEC2 seq: 1128 - 1135 + Phospho (ST)	2E+08	2E+08	[illegible]	3E+06	p16289	IQ motif and SEC7 domain-containing protein 2
SHROOM2 pS1036	2E+09	2E+09	[illegible]	3E+07	p19761	Protein Shroom2
GAPDH seq: 119 - 139 no mod	2E+09	4E+09	[illegible]	8E+08	p01381	Glyceraldehyde-3-phosphate dehydrogenase
KRT8 pS274	8E+08	5E+08	[illegible]	5E+08	p04138	Keratin, type II cytoskeletal 8
AKAP1 pS151	1E+08	3E+08	[illegible]	4E+08	p05502	A-kinase anchor protein 1, mitochondrial
STMN1 pS25	2E+08	3E+08	[illegible]	2E+09	p07581	Stathmin
FAM117B seq: 218 - 235 + Phospho (ST)	5E+06	7E+07	[illegible]	6E+07	p02100	Protein FAM117B
PAK1 pS204	3E+08	3E+08	[illegible]	3E+08	p03333	Serine/threonine-protein kinase PAK 1
XBP1 pS47	4E+08	4E+08	[illegible]	25732	p16266	X-box-binding protein 1
MKI67 pS108	38324	6E+06	[illegible]	1E+07	p03546	Antigen KI-67
TK1 pS33	38324	6E+06	[illegible]	1E+07	p03543	Thymidine kinase, cytosolic
SHTN1 pS506	1E+09	1E+09	[illegible]	5E+08	p04890	Shootin-1
JUN seq: 222 - 252 + 2 Phospho (ST)	38324	37149	[illegible]	5E+07	p11039	Transcription factor AP-1
RALBP1 pS92 pS93	9E+07	37149	[illegible]	5E+07	p06532	RalA-binding protein 1
FLNA pS2152	1E+08	1E+08	[illegible]	4E+08	p19878	Filamin-A
HNRNPC pS260	3E+09	5E+09	[illegible]	4E+09	p10164	Heterogeneous nuclear ribonucleoproteins C1/C2
YTHDF1 seq: 335 - 370 no mod	5E+07	4E+07	[illegible]	25732	p09801	YTH domain-containing family protein 1
UCK1 seq: 251 - 266 + Phospho (ST)	2E+08	1E+08	[illegible]	6E+07	p02846	Uridine-cytidine kinase 1
SRSF10 seq: 131 - 139 + Phospho (ST)	8E+07	6E+07	[illegible]	2E+08	p04062	Serine/arginine-rich splicing factor 10
SNTB2 seq: 393 - 405 + Phospho (ST)	6E+07	2E+08	[illegible]	1E+08	p04417	Beta-2-syntrophin
PHKA1 seq: 972 - 996 + Phospho (ST)	4E+07	5E+07	[illegible]	1E+08	p03287	Phosphorylase b kinase regulatory subunit alpha, skeletal muscle isoform
TRIM28 pS473	3E+08	2E+08	[illegible]	3E+08	p05772	Transcription intermediary factor 1-beta
KRT8 seq: 274 - 285 + Oxidation (M); Phospho (ST)	1E+08	8E+07	[illegible]	7E+07	p04140	Keratin, type II cytoskeletal 8
TCF20 pS1322	38324	4E+07	[illegible]	25732	p18557	Transcription factor 20
SPTBN1 pS2102	6E+08	6E+08	[illegible]	1E+09	p06883	Spectrin beta chain, non-erythrocytic 1
ZRANB2 pS120	1E+08	5E+07	[illegible]	3E+08	p17324	Zinc finger Ran-binding domain-containing protein 2
HCFC1 seq: 1386 - 1415 + Phospho (ST)	38324	37149	[illegible]	4E+07	p01971	Host cell factor 1
SRRM1 pS450 pS452	2E+09	2E+09	[illegible]	1E+10	p07462	Serine/arginine repetitive matrix protein 1
PLEKHG3 seq: 959 - 979 + Phospho (ST)	4E+07	1E+07	[illegible]	2E+08	p18820	Pleckstrin homology domain-containing family G member 3
ANK3 pS4298	6E+08	5E+08	[illegible]	2E+08	p14214	Ankyrin-3
NECAP2 seq: 177 - 195 + Phospho (ST)	4E+08	6E+08	[illegible]	2E+09	p00897	Adaptin ear-binding coat-associated protein 2
HNRNPC pS253 pS260	2E+08	3E+08	[illegible]	5E+08	p10169	Heterogeneous nuclear ribonucleoproteins C1/C2
KRT18 pM58 pM74	38324	2E+08	[illegible]	25732	p16017	Keratin, type I cytoskeletal 18
MYH10 pS1956	3E+08	2E+08	[illegible]	2E+08	p08376	Myosin-10
FAM129B seq: 567 - 615 + 3 Phospho (ST)	38324	37149	[illegible]	4E+08	p18245	Niban-like protein 1
PPIP5K2 pS1006	2E+08	1E+08	[illegible]	5E+07	p05768	Inositol hexakisphosphate and diphosphoinositol-pentakisphosphate kinase 2
PEX19 pS147	1E+08	9E+07	[illegible]	5E+08	p09742	Peroxisomal biogenesis factor 19
GIGYF2 pS593	4E+07	7E+07	[illegible]	3E+08	p01016	PERQ amino acid-rich with GYF domain-containing protein 2
AHNAK pS5752	4E+08	6E+08	[illegible]	9E+08	p19584	Neuroblast differentiation-associated protein AHNAK
NCOR2 pS2065	1E+08	6E+07	[illegible]	3E+08	p05204	Nuclear receptor corepressor 2
EXOC8 pS19	3E+07	2E+07	[illegible]	25732	p08392	Exocyst complex component 8
PDIA4 seq: 211 - 218 no mod	38324	37149	[illegible]	25732	p13025	Protein disulfide-isomerase A4
CAPZA1 seq: 20 - 37 no mod	8E+07	3E+08	[illegible]	1E+08	p16667	F-actin-capping protein subunit alpha-1
THRAP3 pS672	5E+08	8E+08	[illegible]	441488	p09511	Thyroid hormone receptor-associated protein 3
TRAPPC12 seq: 182 - 194 + Phospho (ST)	6E+07	4E+07	[illegible]	3E+07	p04429	Trafficking protein particle complex subunit 12
ASAP1 seq: 733 - 750 + Phospho (ST)	3E+07	4E+07	[illegible]	2E+08	p04441	Arf-GAP with SH3 domain, ANK repeat and PH domain-containing protein 1
LIG1 pS141	3E+08	3E+08	[illegible]	5E+08	p02703	DNA ligase 1
PHC3 pS263	7E+07	2E+08	[illegible]	4E+07	p13674	Polyhomeotic-like protein 3
NPM1 pS70	7E+06	6E+07	[illegible]	3E+08	p18888	Nucleophosmin
NUP155 seq: 988 - 997 + Phospho (ST)	6E+08	2E+08	[illegible]	4E+08	p21069	Nuclear pore complex protein Nup155
ZMYM4 pS122	9E+06	2E+07	[illegible]	1E+07	p00690	Zinc finger MYM-type protein 4
NUCKS1 pS58	2E+08	1E+08	[illegible]	3E+08	p05736	Nuclear ubiquitous casein and cyclin-dependent kinase substrate 1
SPDL1 seq: 504 - 518 + Phospho (ST)	4E+07	1E+08	[illegible]	1E+07	p03275	Protein Spindly
ILF3 seq: 372 - 385 + Phospho (ST)	9E+08	9E+08	[illegible]	9E+08	p06906	Interleukin enhancer-binding factor 3
HN1 seq: 70 - 84 + Phospho (ST)	7E+07	8E+07	[illegible]	5E+07	p03897	Hematological and neurological expressed 1 protein
KAT6A seq: 999 - 1011 + Phospho (ST)	3E+08	4E+08	[illegible]	9E+07	p03657	Histone acetyltransferase KAT6A
RPRD2 pS1099	3E+07	5E+07	[illegible]	7E+07	p07048	Regulation of nuclear pre-mRNA domain-containing protein 2
NUP98 pS888	7E+07	5E+07	[illegible]	6E+07	p00361	Nuclear pore complex protein Nup98-Nup96
FGFR4 pS573	2E+08	3E+08	[illegible]	2E+08	p06890	Fibroblast growth factor receptor 4

HMGA1 seq: 89 - 107 + Phospho (ST)	[illegible]	p12998	High mobility group protein HMG-I/HMG-Y
SPTBN1 pS2358	[illegible]	p07425	Spectrin beta chain, non-erythrocytic 1
AKAP13 pS1876	[illegible]	p06174	A-kinase anchor protein 13
HNRNPC pS260	[illegible]	p10171	Heterogeneous nuclear ribonucleoproteins C1/C2
APOA1BP seq: 107 - 129 no mod	[illegible]	p04477	NAD(P)H-hydrate epimerase
FH seq: 132 - 160 no mod	[illegible]	p11205	Fumarate hydratase, mitochondrial
SP3 pS73	[illegible]	p14247	Transcription factor Sp3
CAAP1 seq: 89 - 104 + Phospho (ST)	[illegible]	p03550	Caspase activity and apoptosis inhibitor 1
KRT73 pS191	[illegible]	p16841	Keratin, type II cytoskeletal 73
SIPA1L1 seq: 1639 - 1658 + Phospho (ST)	[illegible]	p05198	Signal-induced proliferation-associated 1-like protein 1
FRYL seq: 1967 - 1975 + Phospho (ST)	[illegible]	p13923	Protein furry homolog-like
TCOF1 seq: 1225 - 1244 + Phospho (ST)	[illegible]	p12031	Treacle protein
CLDN12 seq: 228 - 244 + Phospho (ST)	[illegible]	p04101	Claudin-12
BUD13 seq: 220 - 230 + 2 Phospho (ST)	[illegible]	p14857	BUD13 homolog
GAPVD1 seq: 755 - 786 + 2 Phospho (ST)	[illegible]	p16949	GTPase-activating protein and VPS9 domain-containing protein 1
ZNF335 seq: 360 - 405 + Phospho (ST)	[illegible]	p11973	Zinc finger protein 335
MORC2 seq: 771 - 790 + 2 Phospho (ST)	[illegible]	p13320	MORC family CW-type zinc finger protein 2
E2F7 pS410	[illegible]	p14797	Transcription factor E2F7
SENP1 pS157	[illegible]	p05868	Sentrin-specific protease 1
AKAP13 pS1565	[illegible]	p07287	A-kinase anchor protein 13
ZC3H4 seq: 1265 - 1278 + 2 Phospho (ST)	[illegible]	p02796	Zinc finger CCCH domain-containing protein 4
SPTBN1 pS2102	[illegible]	p06884	Spectrin beta chain, non-erythrocytic 1
NUP50 pS221	[illegible]	p01848	Nuclear pore complex protein Nup50
MGA seq: 837 - 860 + Phospho (ST)	[illegible]	p11236	MAX gene-associated protein
PDAP1 seq: 56 - 74 + 2 Phospho (ST)	[illegible]	p12575	28 kDa heat- and acid-stable phosphoprotein
PHACTR4 seq: 173 - 187 + Phospho (ST)	[illegible]	p06909	Phosphatase and actin regulator 4
AHNAK pT2845	[illegible]	p13704	Neuroblast differentiation-associated protein AHNAK
ZC3H14 seq: 387 - 404 + Phospho (ST)	[illegible]	p02202	Zinc finger CCCH domain-containing protein 14
FAM117A pS207	[illegible]	p10719	Protein FAM117A
FAM21A seq: 996 - 1029 + Phospho (ST)	[illegible]	p05604	WASH complex subunit FAM21A
DBN1 pS339	[illegible]	p04433	Drebrin
RBM8A pS56	[illegible]	p09922	RNA-binding protein 8A
OR13H1 pS127	[illegible]	p00659	Olfactory receptor 13H1
CTNNA1 seq: 652 - 670 + 2 Phospho (ST)	[illegible]	p03958	Catenin alpha-1
LLGL2 seq: 958 - 978 + 2 Phospho (ST)	[illegible]	p19703	Lethal(2) giant larvae protein homolog 2
STAG2 seq: 1158 - 1181 + Phospho (ST)	[illegible]	p07300	Cohesin subunit SA-2
HNRNPK pS284	[illegible]	p18063	Heterogeneous nuclear ribonucleoprotein K
MACC1 pS115 pS116	[illegible]	p17629	Metastasis-associated in colon cancer protein 1
CARHSP1 pS30 pS32	[illegible]	p17195	Calcium-regulated heat stable protein 1
BAIAP2L1 seq: 239 - 266 + Oxidation (M); 3 Phospho (ST)	[illegible]	p13951	Brain-specific angiogenesis inhibitor 1-associated protein 2-like protein 1
CTTN seq: 397 - 414 + 2 Phospho (ST)	[illegible]	p20355	Src substrate cortactin
LRRFIP1 seq: 99 - 117 + Oxidation (M); Phospho (ST)	[illegible]	p09366	Leucine-rich repeat flightless-interacting protein 1
BAIAP2L1 seq: 248 - 266 + Oxidation (M); Phospho (ST)	[illegible]	p02416	Brain-specific angiogenesis inhibitor 1-associated protein 2-like protein 1
HNRNPUL2 pS161	[illegible]	p17850	Heterogeneous nuclear ribonucleoprotein U-like protein 2
FOXP4 seq: 442 - 462 + Phospho (ST)	[illegible]	p03936	Forkhead box protein P4
RGCC seq: 86 - 114 + 2 Phospho (ST)	[illegible]	p09161	Regulator of cell cycle RGCC
APRT pS66	[illegible]	p14385	Adenine phosphoribosyltransferase
ARAF pS186	[illegible]	p08573	Serine/threonine-protein kinase A-Raf
ARHGAP32 pS871	[illegible]	p10712	Rho GTPase-activating protein 32
SAMHD1 pS33	[illegible]	p02328	Deoxynucleoside triphosphate triphosphohydrolase SAMHD1
SEC16A pS1786	[illegible]	p11327	Protein transport protein Sec16A
USP42 pT983	[illegible]	p06713	Ubiquitin carboxyl-terminal hydrolase 42
CAMSAP1 pS1080	[illegible]	p19862	Calmodulin-regulated spectrin-associated protein 1
PABPC4 pM72	[illegible]	p20299	Polyadenylate-binding protein 4
ACTN4 seq: 126 - 140 no mod	[illegible]	p10352	Alpha-actinin-4
TAOK3 seq: 440 - 446 + Phospho (ST)	[illegible]	p06257	Serine/threonine-protein kinase TAO3
CASKIN1 pS826	[illegible]	p05110	Caskin-1
JMJD1C pS652	[illegible]	p13611	Probable JmjC domain-containing histone demethylation protein 2C
TJP3 pS319	[illegible]	p14886	Tight junction protein ZO-3
CDK16 pS138	[illegible]	p10542	Cyclin-dependent kinase 16
CGNL1 seq: 187 - 210 + 2 Phospho (ST)	[illegible]	p12701	Cingulin-like protein 1
NUCKS1 pS214	[illegible]	p17852	Nuclear ubiquitous casein and cyclin-dependent kinase substrate 1
GAPDH pT184	[illegible]	p01383	Glyceraldehyde-3-phosphate dehydrogenase
HSP90AB1 seq: 103 - 116 no mod	[illegible]	p17467	Heat shock protein HSP 90-beta
GLCCI1 seq: 26 - 59 + Phospho (ST)	[illegible]	p06379	Glucocorticoid-induced transcript 1 protein
CARHSP1 pS30 pS32	[illegible]	p17194	Calcium-regulated heat stable protein 1
RAD23A seq: 81 - 119 + Phospho (ST)	[illegible]	p20591	UV excision repair protein RAD23 homolog A
ARHGEF11 seq: 1452 - 1473 + Phospho (ST)	[illegible]	p05224	Rho guanine nucleotide exchange factor 11
PRKAR2A pT54	[illegible]	p20001	cAMP-dependent protein kinase type II-alpha regulatory subunit
IWS1 pS511 pS513	[illegible]	p17990	Protein IWS1 homolog
TACC1 seq: 262 - 283 + Phospho (ST)	[illegible]	p19430	Transforming acidic coiled-coil-containing protein 1
INADL pS645	[illegible]	p11910	InaD-like protein
ARHGEF16 pS21	[illegible]	p16706	Rho guanine nucleotide exchange factor 16
WTAP seq: 295 - 312 + Phospho (ST)	[illegible]	p17708	Pre-mRNA-splicing regulator WTAP
USP7 pS18	[illegible]	p20681	Ubiquitin carboxyl-terminal hydrolase 7
NBR1 seq: 656 - 670 + Phospho (ST)	[illegible]	p04953	Next to BRCA1 gene 1 protein
NELFE seq: 49 - 66 + Phospho (ST)	[illegible]	p05064	Negative elongation factor E
SRSF10 pS131 pS133	[illegible]	p04063	Serine/arginine-rich splicing factor 10
PNN pS381	[illegible]	p17166	Pinin
PPL pS1657	[illegible]	p05442	Periplakin
RAB3IP seq: 286 - 308 + Phospho (ST)	[illegible]	p09490	Rab-3A-interacting protein
TRAPPC10 seq: 705 - 728 + 2 Phospho (ST)	[illegible]	p06816	Trafficking protein particle complex subunit 10
HMMR pS20	[illegible]	p16571	Hyaluronan mediated motility receptor
ITPKB pS43	[illegible]	p19222	Inositol-trisphosphate 3-kinase B
ACBD3 seq: 10 - 49 + Phospho (ST)	[illegible]	p11917	Golgi resident protein GCP60
DUSP16 pS612	[illegible]	p03193	Dual specificity protein phosphatase 16
AGFG1 seq: 291 - 313 + Phospho (ST)	[illegible]	p03733	Arf-GAP domain and FG repeat-containing protein 1
MKI67 pS584	[illegible]	p19765	Antigen KI-67
ERLIN2 seq: 276 - 285 no mod	[illegible]	p10523	Erlin-2
DOCK7 pS888	[illegible]	p06172	Dedicator of cytokinesis protein 7
FLNB seq: 2105 - 2120 + Phospho (ST)	[illegible]	p19874	Filamin-B
RAI14 pS374	[illegible]	p17420	Ankycorbin
TP53BP1 pS294	[illegible]	p17278	Tumor suppressor p53-binding protein 1
NUP214 pS940 pM942	[illegible]	p01032	Nuclear pore complex protein Nup214
NCOA2 seq: 766 - 780 + Phospho (ST)	[illegible]	p11937	Nuclear receptor coactivator 2
PKM seq: 152 - 162 no mod	[illegible]	p19066	Pyruvate kinase PKM
BCLAF1 pS397	[illegible]	p08464	Bcl-2-associated transcription factor 1
MAP4K5 seq: 333 - 350 + Phospho (ST)	[illegible]	p03046	Mitogen-activated protein kinase kinase kinase kinase 5
INADL pT669	[illegible]	p06530	InaD-like protein
NEB seq: 4869 - 4874 no mod	[illegible]	p14873	Nebulin
LMNA seq: 379 - 386 no mod	[illegible]	p11421	Prelamin-A/C
RANBP2 seq: 1507 - 1522 + Phospho (ST)	[illegible]	p12642	E3 SUMO-protein ligase RanBP2
ATXN2L pS111	[illegible]	p15470	Ataxin-2-like protein
SLC4A1AP pS312	[illegible]	p12867	Kanadaptin
HNRNPUL2 seq: 161 - 187 + Phospho (ST)	[illegible]	p05775	Heterogeneous nuclear ribonucleoprotein U-like protein 2
CLASP2 pS525	[illegible]	p13861	CLIP-associating protein 2
SRRM1 pS450 pS452	[illegible]	p07463	Serine/arginine repetitive matrix protein 1
TMC8 seq: 520 - 526 + Phospho (ST)	[illegible]	p19477	Transmembrane channel-like protein 8
HNRNPUL2 seq: 175 - 198 + Phospho (ST)	[illegible]	p21174	Heterogeneous nuclear ribonucleoprotein U-like protein 2
TMOD3 seq: 195 - 209 no mod	[illegible]	p17347	Tropomodulin-3
SHROOM2 seq: 777 - 785 + Phospho (ST)	[illegible]	p14914	Protein Shroom2
ARHGAP5 seq: 1170 - 1185 + 2 Phospho (ST)	[illegible]	p06526	Rho GTPase-activating protein 5
MEF2D pS180	[illegible]	p11281	Myocyte-specific enhancer factor 2D
SEC16A pS1786	[illegible]	p11328	Protein transport protein Sec16A
UBR4 seq: 2884 - 2914 + Phospho (ST)	[illegible]	p02164	E3 ubiquitin-protein ligase UBR4
TPD52 pS171	[illegible]	p12004	Tumor protein D52
POF1B seq: 103 - 127 + Phospho (ST)	[illegible]	p13643	Protein POF1B
PPP1R13L pS187	[illegible]	p15223	RelA-associated inhibitor
LMNB1 seq: 300 - 312 no mod	[illegible]	p14335	Lamin-B1
VCP pS702	[illegible]	p12245	Transitional endoplasmic reticulum ATPase
ELF4 seq: 641 - 657 + Phospho (ST)	[illegible]	p04328	ETS-related transcription factor Elf-4
PPL pS1657	[illegible]	p05441	Periplakin
YEATS2 pS465	[illegible]	p13533	YEATS domain-containing protein 2
SRP72 seq: 618 - 645 + Phospho (ST)	[illegible]	p01887	Signal recognition particle subunit SRP72
TJP2 seq: 168 - 177 + Phospho (ST)	[illegible]	p03993	Tight junction protein ZO-2
HNRNPC pM251 pS260	[illegible]	p10166	Heterogeneous nuclear ribonucleoproteins C1/C2
KPNA3 pS60	[illegible]	p08954	Importin subunit alpha-4
MAP3K7 pS389	[illegible]	p07056	Mitogen-activated protein kinase kinase kinase 7
PDLIM2 seq: 132 - 153 + 2 Phospho (ST)	[illegible]	p20571	PDZ and LIM domain protein 2
TMEM57 seq: 644 - 664 + Phospho (ST)	[illegible]	p16400	Macoilin
RPS6KA1 seq: 729 - 735 + Phospho (ST)	[illegible]	p12941	Ribosomal protein S6 kinase alpha-1
ATXN2L pS111	[illegible]	p15469	Ataxin-2-like protein
CAST pS297	[illegible]	p18163	Calpastatin
PTMA seq: 91 - 104 no mod	[illegible]	p21178	Prothymosin alpha
CTBP2 seq: 387 - 434 + Phospho (ST)	[illegible]	p00181	C-terminal-binding protein 2
CDK16 pS119	[illegible]	p07123	Cyclin-dependent kinase 16
PDLIM2 pS134 pS137	[illegible]	p20572	PDZ and LIM domain protein 2
UBE2V2 seq: 86 - 101 no mod	[illegible]	p13910	Ubiquitin-conjugating enzyme E2 variant 2
CUX1 seq: 1137 - 1147 no mod	[illegible]	p16965	Homeobox protein cut-like 1
VCP seq: 639 - 651 no mod	[illegible]	p12054	Transitional endoplasmic reticulum ATPase
DNAJC19 seq: 62 - 75 + Phospho (ST)	[illegible]	p18018	Mitochondrial import inner membrane translocase subunit TIM14
DCP1A pS353 pS364	[illegible]	p07943	mRNA-decapping enzyme 1A
CHMP7 pS417	[illegible]	p13752	Charged multivesicular body protein 7
LSM14B seq: 85 - 118 + 2 Phospho (ST)	[illegible]	p19804	Protein LSM14 homolog B
ACIN1 pS365	[illegible]	p02011	Apoptotic chromatin condensation inducer in the nucleus
DUSP16 seq: 506 - 530 + Phospho (ST)	[illegible]	p05642	Dual specificity protein phosphatase 16
SAV1 pS94	[illegible]	p17142	Protein salvador homolog 1
HDLBP seq: 435 - 445 + Phospho (Y)	[illegible]	p10190	Vigilin
MYC pT58 pS62	[illegible]	p13253	Myc proto-oncogene protein
PPP1R12A pS299	[illegible]	p04554	Protein phosphatase 1 regulatory subunit 12A
TFAP2C pS434	[illegible]	p03156	Transcription factor AP-2 gamma
SAV1 seq: 89 - 101 + Phospho (ST)	[illegible]	p17143	Protein salvador homolog 1
HTATSF1 pS676	[illegible]	p11899	HIV Tat-specific factor 1
FAM21C seq: 284 - 296 + Phospho (ST)	[illegible]	p04081	WASH complex subunit FAM21C
PLEC pS125	[illegible]	p06510	Plectin
KIAA1522 pS562	[illegible]	p04655	Uncharacterized protein KIAA1522
HNRNPH2 seq: 151 - 167 no mod	[illegible]	p03525	Heterogeneous nuclear ribonucleoprotein H2
BCLAF1 pS496	[illegible]	p17044	Bcl-2-associated transcription factor 1
ENO1 seq: 307 - 326 no mod	[illegible]	p16432	Alpha-enolase
FAM21A pS284 pS288	[illegible]	p04084	WASH complex subunit FAM21A
AHNAK pM2388 pS2397	[illegible]	p13702	Neuroblast differentiation-associated protein AHNAK
ZNF592 pS348	[illegible]	p12580	Zinc finger protein 592
ALB seq: 438 - 452 no mod	[illegible]	p12412	Serum albumin
HNRNPC seq: 74 - 89 no mod	[illegible]	p10078	Heterogeneous nuclear ribonucleoproteins C1/C2
DENND4C pS1377	[illegible]	p07586	DENN domain-containing protein 4C
LRRC16A seq: 966 - 980 + Phospho (ST)	[illegible]	p06613	Leucine-rich repeat-containing protein 16A
ARHGEF11 pT1461 pT1462	[illegible]	p05225	Rho guanine nucleotide exchange factor 11
LSM14A seq: 191 - 214 + Phospho (ST)	[illegible]	p12554	Protein LSM14 homolog A
YBX1 pS174	[illegible]	p09682	Nuclease-sensitive element-binding protein 1
TPD52L2 pS21	[illegible]	p15701	Tumor protein D54
FAM129A seq: 566 - 599 + 2 Phospho (ST)	[illegible]	p14713	Protein Niban
EPN1 pS435	[illegible]	p03075	Epsin-1
PDIA3 seq: 367 - 379 no mod	[illegible]	p06009	Protein disulfide-isomerase A3
WRNIP1 pS65	[illegible]	p20401	ATPase WRNIP1
HSP90AB4P seq: 415 - 434 no mod	[illegible]	p16136	Putative heat shock protein HSP 90-beta 4
HSP90AB1 seq: 507 - 526 no mod	[illegible]	p16135	Heat shock protein HSP 90-beta
SON seq: 1663 - 1677 no mod	[illegible]	p18753	Protein SON
HSPA5 seq: 61 - 74 no mod	[illegible]	p13586	78 kDa glucose-regulated protein
SQSTM1 seq: 166 - 183 + Phospho (ST)	[illegible]	p12267	Sequestosome-1
C2CD5 seq: 657 - 675 + Phospho (ST)	[illegible]	p04208	C2 domain-containing protein 5
TRIM3 pS427	[illegible]	p20186	Tripartite motif-containing protein 3
PHACTR4 pS464	[illegible]	p05116	Phosphatase and actin regulator 4
PKP3 seq: 308 - 326 + Phospho (ST)	[illegible]	p02572	Plakophilin-3
HNRNPC pS260	[illegible]	p10165	Heterogeneous nuclear ribonucleoproteins C1/C2
MRE11A seq: 531 - 568 + Phospho (ST)	[illegible]	p04210	Double-strand break repair protein MRE11A
DNTTIP2 pS141 pS148	[illegible]	p17082	Deoxynucleotidyltransferase terminal-interacting protein 2
UQCRB seq: 50 - 59 no mod	[illegible]	p07162	Cytochrome b-c1 complex subunit 7
ZNRF2 pS145	[illegible]	p10414	E3 ubiquitin-protein ligase ZNRF2
ZFP91 seq: 80 - 100 + 2 Phospho (ST)	[illegible]	p06716	E3 ubiquitin-protein ligase ZFP91
STX7 seq: 124 - 138 + Phospho (ST)	[illegible]	p19476	Syntaxin-7
CRK seq: 190 - 241 + Phospho (Y)	[illegible]	p00141	Adapter molecule crk
ZKSCAN2 seq: 787 - 792 + Phospho (ST)	[illegible]	p04047	Zinc finger protein with KRAB and SCAN domains 2
NAA30 seq: 186 - 203 + Phospho (ST)	[illegible]	p11272	N-alpha-acetyltransferase 30
STK3 seq: 382 - 399 + Phospho (ST)	[illegible]	p09741	Serine/threonine-protein kinase 3
IRX5 pT237	[illegible]	p10997	Iroquois-class homeodomain protein IRX-5
PBDC1 pS197	[illegible]	p16341	Protein PBDC1
PXN pS126 pS130	[illegible]	p06356	Paxillin
GNL1 seq: 45 - 61 + Phospho (ST)	[illegible]	p17822	Guanine nucleotide-binding protein-like 1
AHNAK pS5099	[illegible]	p09808	Neuroblast differentiation-associated protein AHNAK
EIF3F seq: 255 - 278 + Phospho (ST)	[illegible]	p02988	Eukaryotic translation initiation factor 3 subunit F
MAVS pS222	[illegible]	p15418	Mitochondrial antiviral-signaling protein
SRSF7 pS163 pS165	[illegible]	p04049	Serine/arginine-rich splicing factor 7
UBE2O seq: 81 - 113 + 2 Phospho (ST)	[illegible]	p11594	(E3-independent) E2 ubiquitin-conjugating enzyme
ZC3H3 seq: 567 - 572 + Phospho (ST)	[illegible]	p07114	Zinc finger CCCH domain-containing protein 3
ZNF75D seq: 257 - 262 + Phospho (ST)	[illegible]	p06607	Zinc finger protein 75D
AHNAK pS5110	[illegible]	p16656	Neuroblast differentiation-associated protein AHNAK
PKP2 seq: 147 - 158 + 2 Phospho (ST)	[illegible]	p07188	Plakophilin-2
VPS35 pS7	[illegible]	p08882	Vacuolar protein sorting-associated protein 35
SPAG9 pS203	[illegible]	p17220	C-Jun-amino-terminal kinase-interacting protein 4
MAP3K7 pS389	[illegible]	p07055	Mitogen-activated protein kinase kinase kinase 7

ZFAND5 pS48	2E+08 2E+08 2E+08 1E+08 1E+08 8E+08 3E+08 2E+08 2E+08 1E+08 9E+08 3E+08 2E+08 2E+08 1E+08 4E+08 2E+08 3E+08 2E+08 2E+08 5E+08 2E+08 3E+08 2E+08 2E+08 9E+08 2E+08 [illegible]	3E+08	p09885	AN1-type zinc finger protein 5
TMF1 pS77	1E+08 8E+07 9E+07 1E+08 6E+07 6E+08 2E+08 1E+08 1E+08 6E+07 9E+08 4E+08 3E+08 3E+08 2E+08 3E+08 1E+08 1E+08 2E+08 1E+08 3E+08 1E+08 9E+07 2E+08 1E+08 7E+08 2E+08 [illegible]	2E+08	p04748	TATA element modulatory factor
FAM102B seq: 226 - 235 + Phospho (ST)	4E+07 3E+07 7E+07 3E+07 2E+07 1E+08 4E+07 5E+07 2E+07 2E+07 8E+07 4E+07 5E+07 2E+07 2E+07 4E+07 2E+07 6E+07 4E+07 2E+07 8E+07 2E+07 4E+07 2E+07 1E+07 68554 44396 [illegible]	25732	p03711	Protein FAM102B
UBAP1 pS146	1E+08 2E+08 2E+08 2E+08 1E+08 45779 4E+08 2E+08 2E+08 2E+08 56234 7E+08 4E+08 7E+08 5E+08 40867 3E+08 2E+08 2E+08 2E+08 39759 2E+08 2E+08 2E+08 2E+08 68554 9E+08 [illegible]	8E+08	p01158	Ubiquitin-associated protein 1
CTTNBP2NL seq: 568 - 573 + Phospho (ST)	38324 37149 39656 4E+06 33854 1E+07 25552 5E+06 2E+06 1E+06 56234 53206 39754 40879 6E+06 3E+06 33072 34984 5E+06 1E+06 4E+06 6E+06 1E+06 19024 42741 6E+07 44396 [illegible]	665997	p04341	CTTNBP2 N-terminal-like protein
LRRN4 seq: 328 - 333 + Phospho (ST)	1E+10 1E+10 1E+10 9E+09 6E+09 2E+09 4E+10 1E+10 9E+09 6E+09 4E+10 2E+10 8E+09 9E+09 7E+09 4E+10 2E+10 1E+10 1E+10 7E+09 3E+10 2E+10 1E+10 9E+09 7E+09 6E+09 1E+10 [illegible]	7E+09	p06476	Leucine-rich repeat neuronal protein 4
SF3B1 seq: 336 - 357 + Phospho (ST)	1E+08 7E+07 1E+08 8E+07 7E+07 3E+08 1E+08 1E+08 1E+08 6E+07 4E+08 1E+08 2E+08 1E+08 1E+08 2E+08 8E+07 2E+08 9E+07 9E+07 9E+07 4E+07 1E+08 9E+07 1E+08 1E+08 44396 [illegible]	8E+07	p03953	Splicing factor 3B subunit 1
FAM102B seq: 191 - 205 + Phospho (ST)	5E+07 4E+07 7E+07 5E+07 3E+07 2E+08 5E+07 4E+07 3E+07 3E+07 7E+07 3E+07 2E+07 3E+07 2E+07 7E+07 4E+07 6E+07 5E+07 5E+07 1E+08 3E+07 3E+07 4E+07 4E+07 68554 44396 [illegible]	25732	p06211	Protein FAM102B
MBP seq: 109 - 138 + Phospho (ST)	38324 37149 8E+06 13654 9E+06 45779 8E+06 6E+06 1E+07 26736 56234 53206 3E+07 2E+07 18798 40867 33072 2E+07 19162 28832 39759 4E+06 1E+07 19024 1E+07 2E+08 9E+07 [illegible]	2E+08	p18336	Myelin basic protein
INO80B pS127 pS130	5E+07 3E+07 6E+07 13654 4E+07 45779 9E+07 1E+08 6E+07 8E+07 56234 2E+07 9E+07 3E+07 8E+07 40867 7E+07 1E+08 7E+07 8E+07 39759 4E+07 1E+08 7E+07 6E+07 68554 1E+07 [illegible]	2E+07	p19135	INO80 complex subunit B
WDR44 seq: 542 - 551 no mod	38324 2E+07 1E+07 2E+07 2E+07 1E+08 5E+07 2E+07 1E+07 1E+07 2E+08 6E+07 2E+07 1E+07 2E+07 8E+07 1E+07 2E+07 2E+07 9E+06 7E+07 5E+06 9E+06 2E+07 2E+07 4E+07 44396 [illegible]	25732	p09783	WD repeat-containing protein 44
CLK1 pS140	2E+08 2E+08 3E+08 2E+08 2E+08 1E+09 3E+08 2E+08 2E+08 2E+08 9E+08 3E+08 3E+08 2E+08 3E+08 4E+08 4E+08 6E+08 4E+08 4E+08 4E+08 1E+08 2E+08 2E+08 2E+08 3E+08 5E+07 [illegible]	1E+08	p03372	Dual specificity protein kinase CLK1
HMGB3 seq: 127 - 139 no mod	38324 6E+08 39656 13654 33854 5E+08 25552 34892 34104 26736 5E+08 1E+09 6E+08 5E+08 5E+08 40867 33072 7E+08 19162 28832 39759 1E+09 5E+08 5E+08 6E+08 5E+08 4E+08 [illegible]	2E+08	p11797	High mobility group protein B3
AHNAK pS3426	3E+08 6E+08 7E+08 7E+08 6E+08 5E+09 1E+09 9E+08 9E+08 6E+08 3E+09 2E+09 1E+09 1E+09 9E+08 1E+09 6E+08 1E+09 1E+09 8E+08 1E+09 7E+08 7E+08 9E+08 8E+08 1E+10 4E+09 [illegible]	6E+09	p00817	Neuroblast differentiation-associated protein AHNAK
AAK1 pS637	6E+06 3E+07 5E+07 3E+07 1E+07 2E+08 6E+07 6E+07 5E+07 2E+07 7E+08 2E+08 2E+08 2E+08 7E+07 8E+07 2E+07 3E+07 5E+07 2E+07 8E+07 3E+07 4E+07 5E+07 4E+07 2E+09 4E+08 [illegible]	9E+08	p13992	AP2-associated protein kinase 1
PDIA3 pM338	5E+07 6E+07 2E+08 5E+07 5E+07 2E+08 5E+07 3E+08 3E+07 5E+07 4E+08 7E+07 2E+08 4E+07 1E+07 1E+08 5E+07 5E+07 2E+08 2E+06 5E+07 7E+07 7E+07 7E+07 3E+07 2E+08 8E+07 [illegible]	2E+08	p16387	Protein disulfide-isomerase A3
SHANK2 pS1330	4E+08 4E+08 5E+08 3E+08 3E+08 2E+09 7E+08 4E+08 4E+08 4E+08 2E+09 6E+08 4E+08 3E+08 5E+08 7E+08 4E+08 5E+08 5E+08 4E+08 8E+08 3E+08 7E+08 4E+08 4E+08 68554 2E+07 [illegible]	1E+07	p07613	SH3 and multiple ankyrin repeat domains protein 2
AHNAK pS4960	1E+08 1E+08 1E+08 9E+07 9E+07 6E+08 2E+08 1E+08 2E+08 8E+07 7E+08 3E+08 2E+08 4E+08 2E+08 4E+08 9E+07 2E+08 2E+08 1E+08 4E+08 1E+08 1E+08 2E+08 1E+08 3E+08 6E+07 [illegible]	1E+08	p01643	Neuroblast differentiation-associated protein AHNAK
STAU2 seq: 484 - 498 + 2 Phospho (ST)	7E+07 6E+07 9E+07 6E+07 8E+07 45779 1E+08 1E+08 1E+08 1E+08 56234 3E+08 3E+08 3E+08 3E+08 40867 1E+08 1E+08 1E+08 1E+08 39759 1E+08 1E+08 1E+08 2E+08 68554 3E+08 [illegible]	3E+08	p15163	Double-stranded RNA-binding protein Staufen homolog 2
AHNAK seq: 2930 - 2945 no mod	4E+07 2E+07 9E+07 13654 6E+07 3E+08 6E+07 4E+07 6E+07 3E+07 5E+08 4E+07 1E+08 4E+07 5E+07 2E+08 5E+07 3E+07 8E+07 3E+07 9E+07 1E+07 6E+07 1E+07 7E+07 68554 2E+07 [illegible]	8E+07	p01652	Neuroblast differentiation-associated protein AHNAK
HTT seq: 417 - 437 + Phospho (ST)	1E+08 2E+08 3E+08 3E+08 2E+08 5E+08 2E+08 3E+08 4E+08 2E+08 6E+08 4E+08 5E+08 4E+08 4E+08 2E+08 2E+08 5E+08 5E+08 3E+08 2E+08 2E+08 3E+08 3E+08 3E+08 2E+08 5E+07 [illegible]	2E+08	p05689	Huntingtin
MARS seq: 642 - 656 no mod	8E+06 1E+07 3E+07 2E+07 4E+07 2E+08 6E+07 3E+07 4E+07 2E+07 4E+08 1E+08 4E+07 5E+07 3E+07 4E+07 5E+06 3E+07 6E+07 2E+07 2E+08 8E+06 2E+07 5E+07 4E+07 7E+07 9E+06 [illegible]	5E+07	p09306	Methionine--tRNA ligase, cytoplasmic
SERBP1 seq: 53 - 68 no mod	5E+07 1E+08 5E+07 3E+08 6E+08 3E+09 2E+09 2E+07 4E+08 8E+08 3E+09 1E+09 4E+07 2E+08 6E+08 1E+08 3E+08 2E+08 5E+08 4E+08 2E+09 2E+08 4E+07 2E+08 42741 3E+09 5E+08 [illegible]	25732	p06374	Plasminogen activator inhibitor 1 RNA-binding protein
GAPDH pS83	2E+09 4E+09 6E+09 6E+09 8E+09 1E+10 1E+10 4E+09 8E+09 7E+09 3E+10 2E+10 8E+09 9E+09 1E+10 1E+10 1E+09 5E+09 8E+09 9E+09 4E+09 3E+09 2E+09 7E+09 1E+10 8E+09 2E+09 [illegible]	2E+10	p10404	Glyceraldehyde-3-phosphate dehydrogenase
AKAP2 seq: 141 - 160 + Phospho (ST)	38324 37149 3E+07 2E+07 4E+07 45779 25552 6E+07 4E+07 6E+07 56234 53206 7E+07 6E+07 6E+07 40867 33072 8E+07 6E+07 5E+07 39759 50401 5E+07 8E+07 7E+07 68554 3E+09 [illegible]	4E+09	p18974	A-kinase anchor protein 2
TUBB4B seq: 63 - 77 no mod	[illegible]	2E+08	p19221	Tubulin beta-4B chain
PDHA1 seq: 287 - 300 + 2 Phospho (ST)	[illegible]	25732	p00328	Pyruvate dehydrogenase E1 component subunit alpha, somatic form, mitochondrial
SCAF11 pS816	[illegible]	3E+08	p12625	Protein SCAF11
KRT19 pS35	[illegible]	2E+11	p19914	Keratin, type I cytoskeletal 19
LRRFIP1 seq: 762 - 789 + Phospho (ST)	[illegible]	1E+08	p13432	Leucine-rich repeat flightless-interacting protein 1
ARAF pS186	[illegible]	3E+08	p08574	Serine/threonine-protein kinase A-Raf
PGM3 pS64	[illegible]	6E+07	p03512	Phosphoacetylglucosamine mutase
TXNIP seq: 313 - 343 + Phospho (ST)	[illegible]	8E+06	p02113	Thioredoxin-interacting protein
MMACHC seq: 231 - 258 + Phospho (ST)	[illegible]	2E+08	p12236	Methylmalonic aciduria and homocystinuria type C protein
SVIL pS1225	[illegible]	6E+08	p15772	Supervillin
JUN pS100	[illegible]	7E+07	p12184	Transcription factor AP-1
SPECC1 pS134	[illegible]	2E+08	p03248	Cytospin-B
CCDC85A pS347	[illegible]	25732	p14897	Coiled-coil domain-containing protein 85A
FBLN1 seq: 147 - 179 no mod	[illegible]	25732	p04259	Fibulin-1
PXN pS126 pS130	[illegible]	1E+09	p06357	Paxillin
PROSC pS244	[illegible]	2E+07	p14226	Proline synthase co-transcribed bacterial homolog protein
EPS8L1 seq: 149 - 159 no mod	[illegible]	25732	p17883	Epidermal growth factor receptor kinase substrate 8-like protein 1
HCFC1 pS1205	[illegible]	5E+07	p04731	Host cell factor 1
EXPH5 pS1124	[illegible]	2E+07	p20078	Exophilin-5
FAM21A seq: 284 - 296 + 2 Phospho (ST)	[illegible]	8E+07	p04083	WASH complex subunit FAM21A
ATAD3A pS474	[illegible]	3E+07	p13747	ATPase family AAA domain-containing protein 3A
POLL seq: 359 - 364 + Phospho (ST)	[illegible]	3E+07	p05270	DNA polymerase lambda
DENND1A pS592	[illegible]	3E+07	p05269	DENN domain-containing protein 1A
AHNAK pS570	[illegible]	1E+09	p13692	Neuroblast differentiation-associated protein AHNAK
LSM14B seq: 85 - 118 + Phospho (ST)	[illegible]	3E+08	p19803	Protein LSM14 homolog B
CHRAC1 seq: 112 - 131 + Phospho (ST)	[illegible]	25732	p07487	Chromatin accessibility complex protein 1
HDGF pS132 pS133	[illegible]	1E+08	p15601	Hepatoma-derived growth factor
ITPKC seq: 24 - 30 no mod	[illegible]	2E+07	p10115	Inositol-trisphosphate 3-kinase C
CRTC3 seq: 429 - 463 + Phospho (ST)	[illegible]	6E+08	p04238	CREB-regulated transcription coactivator 3
FOXO3 seq: 420 - 444 + Phospho (ST)	[illegible]	9E+07	p15279	Forkhead box protein O3
AHNAK pS3426	[illegible]	2E+09	p00818	Neuroblast differentiation-associated protein AHNAK
MLLT1 pS292	[illegible]	9E+08	p06965	Protein ENL
STRN3 pS257	[illegible]	3E+08	p06615	Striatin-3
KRT8 pS274	[illegible]	4E+08	p04139	Keratin, type II cytoskeletal 8
PXN pS258	[illegible]	25732	p13754	Paxillin
MTMR3 pS613	[illegible]	4E+07	p03175	Myotubularin-related protein 3
AHNAK pS5110	[illegible]	1E+10	p16657	Neuroblast differentiation-associated protein AHNAK
PKP2 pS251	[illegible]	4E+07	p04901	Plakophilin-2
CTAGE5 seq: 588 - 604 + Phospho (ST)	[illegible]	5E+06	p19923	cTAGE family member 5
HNRNPD seq: 68 - 85 + Phospho (ST)	[illegible]	2E+08	p14448	Heterogeneous nuclear ribonucleoprotein D0
CLCC1 pS438	[illegible]	1E+09	p16509	Chloride channel CLIC-like protein 1
MCM3 pS672	[illegible]	1E+08	p06049	DNA replication licensing factor MCM3
TPD52L2 pS21 pM22	[illegible]	5E+08	p15702	Tumor protein D54
CHAF1A pS65	[illegible]	5E+08	p04700	Chromatin assembly factor 1 subunit A
CAST seq: 364 - 382 + Phospho (ST)	[illegible]	3E+08	p05991	Calpastatin
TNKS1BP1 seq: 1289 - 1302 + Phospho (ST)	[illegible]	7E+07	p09383	182 kDa tankyrase-1-binding protein
CCNK pS340	[illegible]	1E+08	p19145	Cyclin-K
NUP98 pS623	[illegible]	3E+08	p18296	Nuclear pore complex protein Nup98-Nup96
GPATCH2L pS31	[illegible]	1E+08	p11798	G patch domain-containing protein 2-like
LRRC16A pS1331	[illegible]	2E+08	p03196	Leucine-rich repeat-containing protein 16A
PLEKHA5 pS885	[illegible]	3E+08	p06179	Pleckstrin homology domain-containing family A member 5
TXNL4A pS132	[illegible]	1E+08	p15617	Thioredoxin-like protein 4A
DBNL seq: 24 - 41 no mod	[illegible]	2E+07	p04349	Drebrin-like protein
PPA1 seq: 212 - 221 no mod	[illegible]	3E+07	p18692	Inorganic pyrophosphatase
RNPS1 pS155 pS157	[illegible]	4E+09	p06728	RNA-binding protein with serine-rich domain 1
MTMR3 pS647	[illegible]	3E+07	p05260	Myotubularin-related protein 3
ARHGAP39 seq: 458 - 492 + Phospho (ST)	[illegible]	8E+07	p14620	Rho GTPase-activating protein 39
seq: 392 - 409 + Gln->pyro-Glu (N-term Q); Phospho (ST)	[illegible]	2E+07	p08465	Bcl-2-associated transcription factor 1
BUD13 pT159 pS163	[illegible]	3E+07	p14853	BUD13 homolog
EEF1D pS162	[illegible]	2E+08	p12815	Elongation factor 1-delta
KAT6A pS941	[illegible]	8E+07	p07144	Histone acetyltransferase KAT6A
RABL6 seq: 453 - 482 + 3 Phospho (ST)	[illegible]	7E+06	p15216	Rab-like protein 6
HIST3H3 seq: 25 - 37 + Phospho (ST)	[illegible]	2E+09	p01768	Histone H3.1t
MED15 seq: 595 - 613 + Phospho (ST)	[illegible]	2E+08	p11497	Mediator of RNA polymerase II transcription subunit 15
NOP2 pS58	[illegible]	4E+07	p11676	Probable 28S rRNA (cytosine(4447)-C(5))-methyltransferase
SYMPK pS494	[illegible]	3E+08	p07148	Symplekin
ANK3 seq: 1401 - 1411 + Phospho (ST)	[illegible]	2E+07	p13775	Ankyrin-3
SRSF7 pS231 pS233	[illegible]	8E+08	p06699	Serine/arginine-rich splicing factor 7
MYH10 pS1956	[illegible]	9E+07	p06813	Myosin-10
MAP2K2 seq: 6 - 39 + Phospho (ST)	[illegible]	25732	p12706	Dual specificity mitogen-activated protein kinase kinase 2
RIPK1 pS320	[illegible]	2E+07	p09925	Receptor-interacting serine/threonine-protein kinase 1
SARG seq: 558 - 566 + Phospho (ST)	[illegible]	2E+08	p07069	Specifically androgen-regulated gene protein
GRAMD4 seq: 16 - 42 + 2 Phospho (ST)	[illegible]	3E+07	p18918	GRAM domain-containing protein 4
TARDBP seq: 362 - 408 no mod	[illegible]	25732	p17306	TAR DNA-binding protein 43
HNRNPC seq: 285 - 306 + Phospho (ST)	[illegible]	9E+07	p18920	Heterogeneous nuclear ribonucleoproteins C1/C2
HMGA1 seq: 89 - 107 + Phospho (ST)	[illegible]	7E+07	p12999	High mobility group protein HMG-I/HMG-Y
TBC1D4 seq: 747 - 768 + 2 Phospho (ST)	[illegible]	4E+08	p12610	TBC1 domain family member 4
RAD23B seq: 290 - 308 no mod	[illegible]	1E+06	p08519	UV excision repair protein RAD23 homolog B
CDH2 seq: 431 - 456 no mod	[illegible]	2E+08	p16863	Cadherin-2
SCAF1 pS734 pS738	[illegible]	8E+07	p07456	Splicing factor, arginine/serine-rich 19
MPRIP pS619	[illegible]	8E+08	p10150	Myosin phosphatase Rho-interacting protein
MAP7 seq: 199 - 214 + 2 Phospho (ST)	[illegible]	3E+08	p10630	Ensconsin
NUP153 pS516	[illegible]	5E+08	p00944	Nuclear pore complex protein Nup153
HMGCR pS872	[illegible]	9E+07	p05407	3-hydroxy-3-methylglutaryl-coenzyme A reductase
CD109 seq: 859 - 872 no mod	[illegible]	6E+07	p03123	CD109 antigen
TRIM47 pS588	[illegible]	5E+08	p07385	Tripartite motif-containing protein 47
TCOF1 pS1350	[illegible]	8E+09	p12925	Treacle protein
USO1 seq: 678 - 698 no mod	[illegible]	25732	p19027	General vesicular transport factor p115
TCEAL3 pS65	[illegible]	25732	p07492	Transcription elongation factor A protein-like 3
TKT seq: 175 - 186 no mod	[illegible]	2E+07	p12065	Transketolase
CPSF7 seq: 202 - 214 + Phospho (ST)	[illegible]	2E+09	p19341	Cleavage and polyadenylation specificity factor subunit 7
TJP3 pS319	[illegible]	25732	p04692	Tight junction protein ZO-3
PHLDB1 seq: 518 - 531 + Phospho (ST)	[illegible]	1E+08	p04378	Pleckstrin homology-like domain family B member 1
MYOF pS1915	[illegible]	8E+07	p14583	Myoferlin
PSMA5 pS56	[illegible]	1E+09	p13579	Proteasome subunit alpha type-5
SRRM2 pS1975	[illegible]	3E+09	p03960	Serine/arginine repetitive matrix protein 2
AAK1 pS637	[illegible]	1E+09	p13993	AP2-associated protein kinase 1
RPS6KA4 pS745	[illegible]	4E+08	p07328	Ribosomal protein S6 kinase alpha-4
MTDH pS298	[illegible]	3E+08	p10644	Protein LYRIC
AHNAK pS135	[illegible]	4E+09	p11488	Neuroblast differentiation-associated protein AHNAK
GTF2F1 seq: 423 - 444 + 2 Phospho (ST)	[illegible]	6E+08	p10888	General transcription factor IIF subunit 1
LUC7L2 seq: 9 - 19 + Phospho (ST)	[illegible]	7E+07	p20088	Putative RNA-binding protein Luc7-like 2
CTTN seq: 399 - 414 + 2 Phospho (ST)	[illegible]	1E+08	p02248	Src substrate cortactin
SFN seq: 149 - 159 no mod	[illegible]	3E+07	p06165	14-3-3 protein sigma
CCT2 seq: 445 - 466 no mod	[illegible]	7E+07	p10011	T-complex protein 1 subunit beta
CCDC85C seq: 178 - 200 + Phospho (ST)	[illegible]	25732	p03871	Coiled-coil domain-containing protein 85C
DBNDD1 pS121	[illegible]	25732	p04354	Dysbindin domain-containing protein 1
U2AF2 seq: 432 - 444 no mod	[illegible]	3E+08	p14292	Splicing factor U2AF 65 kDa subunit
ZFAND5 pS48	[illegible]	4E+08	p09886	AN1-type zinc finger protein 5
PPP6R1 seq: 729 - 763 + Phospho (ST)	[illegible]	5E+08	p00863	Serine/threonine-protein phosphatase 6 regulatory subunit 1
TPI1 pS117	[illegible]	3E+08	p00697	Triosephosphate isomerase
CDK16 seq: 61 - 75 + Phospho (ST)	[illegible]	2E+07	p15487	Cyclin-dependent kinase 16
ENO1 seq: 286 - 306 no mod	[illegible]	1E+09	p18037	Alpha-enolase
NEDD4L seq: 89 - 98 no mod	[illegible]	2E+07	p11400	E3 ubiquitin-protein ligase NEDD4-like
DES seq: 378 - 386 no mod	[illegible]	4E+08	p16898	Desmin
ILF3 seq: 479 - 523 + Phospho (ST)	[illegible]	25732	p16178	Interleukin enhancer-binding factor 3
FOSL2 seq: 306 - 326 + Phospho (ST)	[illegible]	6E+08	p06570	Fos-related antigen 2
AHNAK pS5448	[illegible]	3E+09	p13757	Neuroblast differentiation-associated protein AHNAK
LRRC16A pS968	[illegible]	1E+08	p06614	Leucine-rich repeat-containing protein 16A
SLTM pS289	[illegible]	25732	p18801	SAFB-like transcription modulator
TNKS1BP1 pS221	[illegible]	3E+08	p14944	182 kDa tankyrase-1-binding protein
MAP4 pS507	[illegible]	2E+08	p18474	Microtubule-associated protein 4
STK10 seq: 447 - 464 + 2 Phospho (ST)	[illegible]	3E+08	p19516	Serine/threonine-protein kinase 10
PPP1R11 seq: 100 - 126 + Phospho (ST)	[illegible]	2E+08	p07545	Protein phosphatase 1 regulatory subunit 11
TXNDC17 seq: 41 - 54 no mod	[illegible]	1E+08	p03206	Thioredoxin domain-containing protein 17
FLNB pS983	[illegible]	7E+07	p12019	Filamin-B
PEX1 pS1211	[illegible]	2E+07	p04196	Peroxisome biogenesis factor 1
STUB1 seq: 129 - 140 no mod	[illegible]	8E+07	p11190	E3 ubiquitin-protein ligase CHIP
MLLT4 seq: 1229 - 1241 + Phospho (ST)	[illegible]	25732	p16912	Afadin
AIM1 seq: 515 - 532 + Phospho (ST)	[illegible]	8E+07	p09960	Absent in melanoma 1 protein
PTPN12 pS673	[illegible]	25732	p14715	Tyrosine-protein phosphatase non-receptor type 12
NOLC1 pS508	[illegible]	5E+08	p01811	Nucleolar and coiled-body phosphoprotein 1
RPS6KA4 seq: 338 - 354 + Phospho (ST)	[illegible]	3E+08	p11943	Ribosomal protein S6 kinase alpha-4
SPEN seq: 2450 - 2479 + Phospho (ST)	[illegible]	25732	p01417	Msx2-interacting protein
DCLK1 seq: 330 - 343 + Phospho (ST)	[illegible]	25732	p04379	Serine/threonine-protein kinase DCLK1
CDC25B seq: 373 - 390 + Phospho (ST)	[illegible]	1E+08	p05367	M-phase inducer phosphatase 2
SLC9A1 pS703	[illegible]	9E+07	p14241	Sodium/hydrogen exchanger 1
UBA1 seq: 11 - 43 + Phospho (ST)	[illegible]	9E+08	p06442	Ubiquitin-like modifier-activating enzyme 1
FAM21C pS498	[illegible]	2E+07	p19270	WASH complex subunit FAM21C
SYNJ1 seq: 1537 - 1573 + Phospho (ST)	[illegible]	25732	p06887	Synaptojanin-1
SNX1 pS188	[illegible]	1E+08	p07440	Sorting nexin-1
ATF7IP pS559	[illegible]	1E+08	p05373	Activating transcription factor 7-interacting protein 1
HSPB8 pT87	[illegible]	5E+07	p16724	Heat shock protein beta-8
AHNAK pS5448	[illegible]	3E+07	p13756	Neuroblast differentiation-associated protein AHNAK
AFF1 pS245	[illegible]	3E+08	p14939	AF4/FMR2 family member 1
APIP seq: 82 - 91 + Phospho (ST)	[illegible]	5E+08	p18724	Methylthioribulose-1-phosphate dehydratase
LYST seq: 2103 - 2120 + Phospho (ST)	[illegible]	25732	p04046	Lysosomal-trafficking regulator
EXOC1 seq: 467 - 486 + 2 Phospho (ST)	[illegible]	5E+08	p10548	Exocyst complex component 1
ROCK2 seq: 1121 - 1152 + 2 Phospho (ST)	[illegible]	2E+07	p04240	Rho-associated protein kinase 2
LMO7 seq: 949 - 974 + 2 Phospho (ST)	[illegible]	6E+07	p02363	LIM domain only protein 7
TACC2 pS2359	[illegible]	1E+08	p09938	Transforming acidic coiled-coil-containing protein 2
LRRC16A seq: 967 - 980 + Phospho (ST)	[illegible]	2E+08	p03899	Leucine-rich repeat-containing protein 16A
ATF2 pS112	[illegible]	25732	p12865	Cyclic AMP-dependent transcription factor ATF-2
EIF4G1 pS1231	[illegible]	1E+09	p18016	Eukaryotic translation initiation factor 4 gamma 1
NME2 pT79	[illegible]	1E+07	p01107	Nucleoside diphosphate kinase B
KIAA1671 pS1760	[illegible]	2E+08	p04671	Uncharacterized protein KIAA1671
MAPK3 seq: 190 - 208 + Phospho (Y)	[illegible]	4E+08	p14523	Mitogen-activated protein kinase 3
POLD3 pS307	[illegible]	1E+08	p01796	DNA polymerase delta subunit 3
PKM seq: 208 - 224 no mod	[illegible]	1E+08	p15014	Pyruvate kinase PKM
HTT pS432	[illegible]	1E+08	p05688	Huntingtin
GTF2F1 pS433	[illegible]	7E+07	p12029	General transcription factor IIF subunit 1
FOXK1 pS416 pS420	[illegible]	3E+09	p06302	Forkhead box protein K1
TXNRD1 seq: 219 - 234 no mod	[illegible]	7E+07	p11230	Thioredoxin reductase 1, cytoplasmic
MAP4K5 seq: 333 - 349 + Phospho (ST)	[illegible]	25732	p03047	Mitogen-activated protein kinase kinase kinase kinase 5
DOCK7 pS888	[illegible]	3E+08	p06171	Dedicator of cytokinesis protein 7
YWHAE seq: 215 - 224 no mod	[illegible]	2E+08	p18203	14-3-3 protein epsilon
AHNAK pS5099	[illegible]	2E+09	p09807	Neuroblast differentiation-associated protein AHNAK
CD2AP seq: 85 - 102 + Phospho (ST)	[illegible]	4E+08	p13637	CD2-associated protein
SCAF1 pS725	[illegible]	2E+08	p15439	Splicing factor, arginine/serine-rich 19
ADAM19 pT779	[illegible]	3E+07	p01364	Disintegrin and metalloproteinase domain-containing protein 19
LRBA seq: 1484 - 1501 + Phospho (ST)	[illegible]	25732	p06378	Lipopolysaccharide-responsive and beige-like anchor protein
CEP170B pS1135	[illegible]	8E+07	p11820	Centrosomal protein of 170 kDa protein B
GRIPAP1 pS655	[illegible]	1E+08	p05729	GRIP1-associated protein 1
RPS6KB1 pS447	[illegible]	25732	p02296	Ribosomal protein S6 kinase beta-1
RABL6 seq: 355 - 360 + Phospho (ST)	[illegible]	7E+09	p06671	Rab-like protein 6
H1FX pS31	[illegible]	9E+08	p20654	Histone H1x
DOCK7 seq: 577 - 586 + Phospho (ST)	[illegible]	9E+08	p08616	Dedicator of cytokinesis protein 7
PCNA seq: 118 - 138 no mod	[illegible]	4E+07	p11242	Proliferating cell nuclear antigen
SRRM2 pS1132	[illegible]	8E+07	p05716	Serine/arginine repetitive matrix protein 2
FAM126B pT306	[illegible]	25732	p01583	Protein FAM126B
VCL seq: 739 - 758 + Phospho (ST)	[illegible]	8E+07	p01789	Vinculin
TNKS1BP1 pS1620 pS1621	[illegible]	25732	p00987	182 kDa tankyrase-1-binding protein
ENO1 pM94 pM97	[illegible]	3E+08	p14410	Alpha-enolase
PIK3R2 seq: 257 - 286 + Phospho (ST)	[illegible]	8E+08	p19953	Phosphatidylinositol 3-kinase regulatory subunit beta
YWHAE seq: 197 - 215 no mod	[illegible]	2E+09	p21164	14-3-3 protein epsilon

Peptide	Intensity values	Accession	Protein
ADAM8 seq: 683 - 688 + Phospho (ST)	[illegible]	p04107	Disintegrin and metalloproteinase domain-containing protein 8
SARG pS560	[illegible]	p07070	Specifically androgen-regulated gene protein
CSPP1 pS920	[illegible]	p19760	Centrosome and spindle pole-associated protein 1
CD2AP seq: 85 - 102 + Phospho (Y)	[illegible]	p13636	CD2-associated protein
FOXK1 pS416 pS420	[illegible]	p06303	Forkhead box protein K1
HTATSF1 seq: 451 - 473 + Phospho (ST)	[illegible]	p17120	HIV Tat-specific factor 1
PSMD9 seq: 65 - 73 no mod	[illegible]	p06067	26S proteasome non-ATPase regulatory subunit 9
ATG4C seq: 449 - 458 + Phospho (ST)	[illegible]	p07415	Cysteine protease ATG4C
SIPA1L1 seq: 1560 - 1577 + 2 Phospho (ST)	[illegible]	p16712	Signal-induced proliferation-associated 1-like protein 1
ITPR3 pS916	[illegible]	p05485	Inositol 1,4,5-trisphosphate receptor type 3
DENND4A seq: 1587 - 1601 + Phospho (ST)	[illegible]	p05455	C-myc promoter-binding protein
SEC24D seq: 258 - 275 no mod	[illegible]	p13015	Protein transport protein Sec24D
CDCA3 seq: 199 - 216 no mod	[illegible]	p04539	Cell division cycle-associated protein 3
HNRNPUL2 pS185 pS188	[illegible]	p21175	Heterogeneous nuclear ribonucleoprotein U-like protein 2
CLIP2 pS348	[illegible]	p13071	CAP-Gly domain-containing linker protein 2
TPR pS2155	[illegible]	p02966	Nucleoprotein TPR
PPP1R7 pS24 pS27	[illegible]	p06468	Protein phosphatase 1 regulatory subunit 7
JUP pS665	[illegible]	p06422	Junction plakoglobin
PHRF1 seq: 1120 - 1131 + Phospho (ST)	[illegible]	p15350	PHD and RING finger domain-containing protein 1
NUCB1 pS369	[illegible]	p19771	Nucleobindin-1
LEO1 pT629 pS630	[illegible]	p12887	RNA polymerase-associated protein LEO1
ATXN2L seq: 678 - 688 + 2 Phospho (ST)	[illegible]	p03426	Ataxin-2-like protein
ZFP36 pS186	[illegible]	p07893	Tristetraprolin
PRPF4B pS328	[illegible]	p12780	Serine/threonine-protein kinase PRP4 homolog
NUP98 pS612	[illegible]	p09431	Nuclear pore complex protein Nup98-Nup96
TBR1 seq: 83 - 94 no mod	[illegible]	p10696	T-box brain protein 1
LAD1 seq: 345 - 360 + Phospho (ST)	[illegible]	p13855	Ladinin-1
MDC1 seq: 162 - 179 + 2 Phospho (ST)	[illegible]	p11368	Mediator of DNA damage checkpoint protein 1
SRRM2 seq: 1816 - 1827 + Phospho (ST)	[illegible]	p15967	Serine/arginine repetitive matrix protein 2
IK seq: 481 - 496 no mod	[illegible]	p00507	Protein Red
LIG1 seq: 41 - 54 + Phospho (ST)	[illegible]	p16926	DNA ligase 1
SEPT9 seq: 269 - 289 no mod	[illegible]	p09679	Septin-9
NUMA1 seq: 986 - 999 no mod	[illegible]	p21094	Nuclear mitotic apparatus protein 1
LAMC1 seq: 1358 - 1371 no mod	[illegible]	p18171	Laminin subunit gamma-1
RPS6KA4 seq: 681 - 699 + 2 Phospho (ST)	[illegible]	p03796	Ribosomal protein S6 kinase alpha-4
GIGYF1 seq: 636 - 644 + Phospho (ST)	[illegible]	p04966	PERQ amino acid-rich with GYF domain-containing protein 1
GAPDH pS83	[illegible]	p10405	Glyceraldehyde-3-phosphate dehydrogenase
EEF1B2 pS106	[illegible]	p18922	Elongation factor 1-beta
ERCC6L seq: 938 - 964 + Phospho (Y)	[illegible]	p12000	DNA excision repair protein ERCC-6-like
KIFAP3 pS60	[illegible]	p05139	Kinesin-associated protein 3
JUP pS665	[illegible]	p12609	Junction plakoglobin
CCT4 seq: 97 - 122 no mod	[illegible]	p19828	T-complex protein 1 subunit delta
EZR seq: 54 - 60 no mod	[illegible]	p16103	Ezrin
TPD52L2 seq: 16 - 34 + Phospho (ST)	[illegible]	p15700	Tumor protein D54
NAB2 seq: 157 - 169 + 2 Phospho (ST)	[illegible]	p05823	NGFI-A-binding protein 2
DUSP16 pS627	[illegible]	p06141	Dual specificity protein phosphatase 16
KIAA1671 seq: 1148 - 1167 + Phospho (ST)	[illegible]	p17183	Uncharacterized protein KIAA1671
EEF1B2 seq: 8 - 22 no mod	[illegible]	p04730	Elongation factor 1-beta
FAM114A2 seq: 132 - 167 + Phospho (ST)	[illegible]	p00042	Protein FAM114A2
RBM7 pS136	[illegible]	p05817	RNA-binding protein 7
CARHSP1 pS30 pS32	[illegible]	p17196	Calcium-regulated heat stable protein 1
KDM1A pS131 pS137	[illegible]	p17369	Lysine-specific histone demethylase 1A
CDC42EP4 pS77	[illegible]	p06664	Cdc42 effector protein 4
AKAP2 pS748	[illegible]	p07718	A-kinase anchor protein 2
PFDN4 seq: 8 - 22 no mod	[illegible]	p21203	Prefoldin subunit 4
ZC3H18 pS868	[illegible]	p11690	Zinc finger CCCH domain-containing protein 18
PARP4 seq: 1333 - 1345 + Phospho (ST)	[illegible]	p20513	Poly [ADP-ribose] polymerase 4
CBX1 pS89	[illegible]	p13462	Chromobox protein homolog 1
ARID5B pS1032	[illegible]	p19184	AT-rich interactive domain-containing protein 5B
AIM1 pS484	[illegible]	p18190	Absent in melanoma 1 protein
RBM7 pS204	[illegible]	p09978	RNA-binding protein 7
BCLAF1 pS648	[illegible]	p08427	Bcl-2-associated transcription factor 1
IRS2 seq: 1147 - 1170 + 2 Phospho (ST)	[illegible]	p14613	Insulin receptor substrate 2
EIF4G1 pS1231	[illegible]	p18015	Eukaryotic translation initiation factor 4 gamma 1
PRPF4B pS277	[illegible]	p04583	Serine/threonine-protein kinase PRP4 homolog
RRP1B seq: 713 - 741 + 2 Phospho (ST)	[illegible]	p01816	Ribosomal RNA processing protein 1 homolog B
C2CD5 seq: 816 - 844 + Phospho (ST)	[illegible]	p19453	C2 domain-containing protein 5
RAD23A seq: 81 - 119 + Phospho (ST)	[illegible]	p20592	UV excision repair protein RAD23 homolog A
CAMSAP2 seq: 595 - 607 + Phospho (ST)	[illegible]	p11167	Calmodulin-regulated spectrin-associated protein 2
CDC27 pS364	[illegible]	p16940	Cell division cycle protein 27 homolog
ARHGAP32 seq: 2014 - 2037 + Phospho (Y)	[illegible]	p07829	Rho GTPase-activating protein 32
EPPK1 seq: 1382 - 1401 no mod	[illegible]	p11743	Epiplakin
ADNP seq: 731 - 757 + Phospho (ST)	[illegible]	p13020	Activity-dependent neuroprotector homeobox protein
AHNAK pM5088 pS5099	[illegible]	p09809	Neuroblast differentiation-associated protein AHNAK
FASN pT2204	[illegible]	p20925	Fatty acid synthase
MTMR3 seq: 611 - 630 + Phospho (ST)	[illegible]	p02206	Myotubularin-related protein 3
PALLD pS893	[illegible]	p14473	Palladin
FOXK1 seq: 431 - 456 + 3 Phospho (ST)	[illegible]	p05747	Forkhead box protein K1
LARP7 pS261	[illegible]	p04088	La-related protein 7
PATL1 seq: 176 - 188 + Phospho (ST)	[illegible]	p06550	Protein PAT1 homolog 1
MACC1 pS515	[illegible]	p07108	Metastasis-associated in colon cancer protein 1
AHNAK pS135	[illegible]	p11489	Neuroblast differentiation-associated protein AHNAK
HNRNPM pM486 pM490	[illegible]	p10136	Heterogeneous nuclear ribonucleoprotein M
PCNA seq: 54 - 61 no mod	[illegible]	p06031	Proliferating cell nuclear antigen
TLK2 seq: 747 - 772 + Phospho (ST)	[illegible]	p12518	Serine/threonine-protein kinase tousled-like 2
ERCC6L seq: 938 - 964 + Phospho (ST)	[illegible]	p12001	DNA excision repair protein ERCC-6-like
SRRM1 pS549 pS551	[illegible]	p06727	Serine/arginine repetitive matrix protein 1
IRS2 pS1176	[illegible]	p14708	Insulin receptor substrate 2
AIM1 pS725	[illegible]	p09955	Absent in melanoma 1 protein
ADRBK1 pS670	[illegible]	p09502	Beta-adrenergic receptor kinase 1
PRPF38B seq: 526 - 537 + Phospho (ST)	[illegible]	p06634	Pre-mRNA-splicing factor 38B
ADIRF seq: 49 - 70 no mod	[illegible]	p02031	Adipogenesis regulatory factor
SHROOM1 pS166	[illegible]	p09895	Protein Shroom1
CARHSP1 pS30 pS32	[illegible]	p04393	Calcium-regulated heat stable protein 1
C1orf226 seq: 189 - 205 + Phospho (ST)	[illegible]	p01196	Uncharacterized protein C1orf226
EPN1 pS435	[illegible]	p04664	Epsin-1
SYNJ2 pS1124	[illegible]	p12608	Synaptojanin-2
AHNAK pS5110	[illegible]	p20870	Neuroblast differentiation-associated protein AHNAK
GAPDH pS83	[illegible]	p10403	Glyceraldehyde-3-phosphate dehydrogenase
ZC3H13 pS1208	[illegible]	p10861	Zinc finger CCCH domain-containing protein 13
FAM114A2 seq: 132 - 167 + Phospho (ST)	[illegible]	p00041	Protein FAM114A2
KAT7 seq: 117 - 155 + 2 Phospho (ST)	[illegible]	p09038	Histone acetyltransferase KAT7
EIF4EBP1 pT70	[illegible]	p09103	Eukaryotic translation initiation factor 4E-binding protein 1
EGFR pT693	[illegible]	p17384	Epidermal growth factor receptor
SLC4A2 seq: 169 - 185 + Phospho (ST)	[illegible]	p02137	Anion exchange protein 2
SHROOM3 pS439 pS443	[illegible]	p04677	Protein Shroom3
NT5E seq: 357 - 374 no mod	[illegible]	p17899	5'-nucleotidase
PPL pS887	[illegible]	p09194	Periplakin
UBA1 seq: 11 - 43 + 2 Phospho (ST)	[illegible]	p06444	Ubiquitin-like modifier-activating enzyme 1
GSDMD seq: 250 - 267 + Phospho (ST)	[illegible]	p03466	Gasdermin-D
HSD17B10 seq: 53 - 79 no mod	[illegible]	p12974	3-hydroxyacyl-CoA dehydrogenase type-2
PKLR seq: 436 - 442 + Gln->pyro-Glu (N-term Q)	[illegible]	p08388	Pyruvate kinase PKLR
RECQL4 pS89	[illegible]	p21000	ATP-dependent DNA helicase Q4
AKAP13 seq: 647 - 681 + Phospho (ST)	[illegible]	p15799	A-kinase anchor protein 13
SRSF11 pS483	[illegible]	p01080	Serine/arginine-rich splicing factor 11
NUCKS1 seq: 202 - 219 + 2 Phospho (ST)	[illegible]	p02326	Nuclear ubiquitous casein and cyclin-dependent kinase substrate 1
CASC5 seq: 1296 - 1306 no mod	[illegible]	p19731	Protein CASC5
GAPVD1 seq: 562 - 582 + 2 Phospho (ST)	[illegible]	p02571	GTPase-activating protein and VPS9 domain-containing protein 1
ZFAND5 seq: 47 - 68 + Oxidation (M); Phospho (ST)	[illegible]	p09887	AN1-type zinc finger protein 5
CASZ1 seq: 48 - 62 + Phospho (ST)	[illegible]	p20935	Zinc finger protein castor homolog 1
EPS15L1 seq: 103 - 124 + Phospho (ST)	[illegible]	p16709	Epidermal growth factor receptor substrate 15-like 1
TOM1 pS160 pT164	[illegible]	p13187	Target of Myb protein 1
MTDH pS84	[illegible]	p06735	Protein LYRIC
FAM129B pS641 pS646	[illegible]	p20378	Niban-like protein 1
PATL1 pS179	[illegible]	p06551	Protein PAT1 homolog 1
RPS6KA1 pT359 pS363	[illegible]	p02384	Ribosomal protein S6 kinase alpha-1
MCM3 seq: 668 - 688 + Phospho (ST)	[illegible]	p06051	DNA replication licensing factor MCM3
MPRIP seq: 878 - 897 + Phospho (ST)	[illegible]	p02595	Myosin phosphatase Rho-interacting protein
EIF4G1 pS1231	[illegible]	p18017	Eukaryotic translation initiation factor 4 gamma 1
FAM129B seq: 619 - 654 + 2 Phospho (ST)	[illegible]	p20377	Niban-like protein 1
CIRBP seq: 8 - 28 no mod	[illegible]	p11852	Cold-inducible RNA-binding protein
EP400NL seq: 141 - 167 + Phospho (ST)	[illegible]	p20715	EP400 N-terminal-like protein
HNRNPK pS116	[illegible]	p14144	Heterogeneous nuclear ribonucleoprotein K
WNK2 pS1862	[illegible]	p08820	Serine/threonine-protein kinase WNK2
EIF2S2 pS67	[illegible]	p13053	Eukaryotic translation initiation factor 2 subunit 2
PHF2 seq: 649 - 666 + Phospho (ST)	[illegible]	p20185	Lysine-specific demethylase PHF2
NPM1 seq: 46 - 73 + Phospho (ST)	[illegible]	p01908	Nucleophosmin
AHNAK seq: 960 - 970 + Phospho (Y)	[illegible]	p01317	Neuroblast differentiation-associated protein AHNAK
PPP1R3D pS46	[illegible]	p20946	Protein phosphatase 1 regulatory subunit 3D
SPAST seq: 266 - 279 + Phospho (ST)	[illegible]	p19870	Spastin
CAMSAP3 pS1074	[illegible]	p19899	Calmodulin-regulated spectrin-associated protein 3
SRRM1 seq: 313 - 318 + Phospho (ST)	[illegible]	p06744	Serine/arginine repetitive matrix protein 1
EIF4EBP1 seq: 64 - 99 + Phospho (ST)	[illegible]	p09099	Eukaryotic translation initiation factor 4E-binding protein 1
UBAP1 seq: 182 - 202 no mod	[illegible]	p09545	Ubiquitin-associated protein 1
PLEC seq: 4571 - 4576 no mod	[illegible]	p00229	Plectin
AHNAK pS5782	[illegible]	p04808	Neuroblast differentiation-associated protein AHNAK
CSNK1E seq: 402 - 416 + Phospho (ST)	[illegible]	p13886	Casein kinase I isoform epsilon
NPM1 seq: 55 - 80 + Oxidation (M); Phospho (ST)	[illegible]	p18891	Nucleophosmin
ABCF1 pS105 pT108 pS109	[illegible]	p12894	ATP-binding cassette sub-family F member 1
IWS1 pS511 pS513	[illegible]	p17989	Protein IWS1 homolog
UBE2O seq: 826 - 849 + Phospho (ST)	[illegible]	p09356	(E3-independent) E2 ubiquitin-conjugating enzyme
EIF4EBP1 pT70	[illegible]	p09104	Eukaryotic translation initiation factor 4E-binding protein 1
PAK4 pS181	[illegible]	p18669	Serine/threonine-protein kinase PAK 4
CNOT2 seq: 169 - 178 + Phospho (ST)	[illegible]	p03783	CCR4-NOT transcription complex subunit 2
NCOR2 seq: 493 - 514 no mod	[illegible]	p04230	Nuclear receptor corepressor 2
FGD1 pS135	[illegible]	p10868	FYVE, RhoGEF and PH domain-containing protein 1
seq: 554 - 576 + Gln->pyro-Glu (N-term Q); Phospho (ST)	[illegible]	p08648	Cleavage stimulation factor subunit 2 tau variant
LIG1 pS66	[illegible]	p01227	DNA ligase 1
SHROOM3 seq: 425 - 449 + Phospho (ST)	[illegible]	p04676	Protein Shroom3
CAPRIN1 seq: 14 - 55 + Phospho (ST)	[illegible]	p03890	Caprin-1
SNX2 pS277	[illegible]	p19219	Sorting nexin-2
TPPP seq: 30 - 57 + Phospho (ST)	[illegible]	p07128	Tubulin polymerization-promoting protein
TRIM47 pS588	[illegible]	p16039	Tripartite motif-containing protein 47
NMD3 pS468	[illegible]	p18309	60S ribosomal export protein NMD3
GSE1 seq: 1090 - 1120 + Phospho (ST)	[illegible]	p13175	Genetic suppressor element 1
HNRNPK seq: 104 - 139 + Phospho (ST)	[illegible]	p14145	Heterogeneous nuclear ribonucleoprotein K
KRT8 pM406 pM409	[illegible]	p11927	Keratin, type II cytoskeletal 8
TPM1 seq: 141 - 149 no mod	[illegible]	p10186	Tropomyosin alpha-1 chain
TPM3 seq: 142 - 150 no mod	[illegible]	p10183	Tropomyosin alpha-3 chain
ZC3H13 pS1409	[illegible]	p18271	Zinc finger CCCH domain-containing protein 13
CRTC2 pS433	[illegible]	p06457	CREB-regulated transcription coactivator 2
EIF6 seq: 165 - 188 no mod	[illegible]	p02175	Eukaryotic translation initiation factor 6
HIRIP3 pS142 pS143	[illegible]	p19257	HIRA-interacting protein 3
EXOC8 pS19	[illegible]	p08393	Exocyst complex component 8
CCDC86 pS113	[illegible]	p08137	Coiled-coil domain-containing protein 86
BOD1L1 seq: 2774 - 2794 + 2 Phospho (ST)	[illegible]	p12682	Biorientation of chromosomes in cell division protein 1-like 1
RALY pT151	[illegible]	p00664	RNA-binding protein Raly
WRAP53 seq: 484 - 506 + Phospho (ST)	[illegible]	p01537	Telomerase Cajal body protein 1
CDKL5 seq: 396 - 409 + Phospho (ST)	[illegible]	p18519	Cyclin-dependent kinase-like 5
TPP2 seq: 152 - 168 no mod	[illegible]	p12692	Tripeptidyl-peptidase 2
FLNA seq: 983 - 994 no mod	[illegible]	p01650	Filamin-A
DOCK5 pS1766	[illegible]	p05085	Dedicator of cytokinesis protein 5
PCYT1A seq: 148 - 162 no mod	[illegible]	p09751	Choline-phosphate cytidylyltransferase A
MAP1B pS1396 pS1400	[illegible]	p01164	Microtubule-associated protein 1B
1 seq: 80 - 89 + Gln->pyro-Glu (N-term Q); Phospho (ST)	[illegible]	p08281	Heat shock protein beta-1
ABLIM3 seq: 371 - 384 + Phospho (ST)	[illegible]	p19483	Actin-binding LIM protein 3
CEP170B pS969	[illegible]	p05703	Centrosomal protein of 170 kDa protein B
DBN1 seq: 355 - 390 + Phospho (ST)	[illegible]	p20310	Drebrin
FOXK2 seq: 421 - 441 + 2 Phospho (ST)	[illegible]	p16853	Forkhead box protein K2
TSC22D4 seq: 291 - 321 + Phospho (ST)	[illegible]	p16465	TSC22 domain family protein 4
CCDC12 seq: 145 - 166 + Phospho (ST)	[illegible]	p15406	Coiled-coil domain-containing protein 12
EIF4EBP2 seq: 21 - 51 + Phospho (ST)	[illegible]	p01988	Eukaryotic translation initiation factor 4E-binding protein 2
CDK17 pS165	[illegible]	p10989	Cyclin-dependent kinase 17
TBC1D5 pS522	[illegible]	p05979	TBC1 domain family member 5
H1FX seq: 24 - 35 + Phospho (ST)	[illegible]	p20653	Histone H1x
SGK223 pS492	[illegible]	p02676	Tyrosine-protein kinase SgK223
THRAP3 pS698	[illegible]	p14810	Thyroid hormone receptor-associated protein 3
NUMA1 pS2077	[illegible]	p20174	Nuclear mitotic apparatus protein 1
SHROOM2 seq: 921 - 937 + Phospho (ST)	[illegible]	p03775	Protein Shroom2
KPNA4 seq: 68 - 96 no mod	[illegible]	p00939	Importin subunit alpha-3
AKAP17A pS537	[illegible]	p00591	A-kinase anchor protein 17A
MAGED2 seq: 66 - 76 + Phospho (ST)	[illegible]	p19387	Melanoma-associated antigen D2
LASP1 seq: 60 - 73 + Oxidation (M); Phospho (ST)	[illegible]	p07915	LIM and SH3 domain protein 1
FTSJ3 seq: 329 - 376 + 2 Phospho (ST)	[illegible]	p20306	pre-rRNA processing protein FTSJ3
MPRIP pM614 pS619	[illegible]	p10151	Myosin phosphatase Rho-interacting protein
PLEC pS720	[illegible]	p06112	Plectin
TP53BP1 pS1114	[illegible]	p09829	Tumor suppressor p53-binding protein 1
CDC42EP4 pS174	[illegible]	p09615	Cdc42 effector protein 4
CCT6A seq: 146 - 153 no mod	[illegible]	p17059	T-complex protein 1 subunit zeta
SYNJ2 pS1124	[illegible]	p06275	Synaptojanin-2

EIF4EBP1 seq: 74 - 106 + Phospho (ST)	[illegible]	p18555	Eukaryotic translation initiation factor 4E-binding protein 1
CCT2 seq: 323 - 342 no mod	[illegible]	p12228	T-complex protein 1 subunit beta
NUP214 seq: 430 - 446 + 2 Phospho (ST)	[illegible]	p04605	Nuclear pore complex protein Nup214
GAPDH pM175 pT184	[illegible]	p01386	Glyceraldehyde-3-phosphate dehydrogenase
KRT8 pS330	[illegible]	p15378	Keratin, type II cytoskeletal 8
WWTR1 seq: 87 - 114 + 2 Phospho (ST)	[illegible]	p05558	WW domain-containing transcription regulator protein 1
LARP1 pS90	[illegible]	p17137	La-related protein 1
ACOT2 seq: 287 - 306 no mod	[illegible]	p18786	Acyl-coenzyme A thioesterase 2, mitochondrial
TCF20 pS574	[illegible]	p11214	Transcription factor 20
FLNA seq: 1273 - 1283 no mod	[illegible]	p17908	Filamin-A
MSL3 pS365	[illegible]	p10807	Male-specific lethal 3 homolog
EIF3G seq: 252 - 267 + Phospho (ST)	[illegible]	p17072	Eukaryotic translation initiation factor 3 subunit G
SON pS1697	[illegible]	p18186	Protein SON
MKI67 pS357	[illegible]	p02299	Antigen KI-67
RIN1 pS351	[illegible]	p04883	Ras and Rab interactor 1
MAPK1 pT185	[illegible]	p01825	Mitogen-activated protein kinase 1
KIAA1671 pS1555	[illegible]	p18187	Uncharacterized protein KIAA1671
SLC9A1 pS703	[illegible]	p14240	Sodium/hydrogen exchanger 1
SASH1 seq: 813 - 820 + Phospho (ST)	[illegible]	p05093	SAM and SH3 domain-containing protein 1
CAD seq: 1034 - 1048 no mod	[illegible]	p01225	CAD protein
SCAF1 pS734 pS738	[illegible]	p07457	Splicing factor, arginine/serine-rich 19
PEBP1 pT42	[illegible]	p01131	Phosphatidylethanolamine-binding protein 1
HNRNPCL1 seq: 194 - 203 no mod	[illegible]	p01662	Heterogeneous nuclear ribonucleoprotein C-like 1
GPN1 pS338	[illegible]	p15090	GPN-loop GTPase 1
WEE1 pT190	[illegible]	p11904	Wee1-like protein kinase
CTTN seq: 397 - 414 + 2 Phospho (ST)	[illegible]	p20356	Src substrate cortactin
ADNP seq: 392 - 419 + Phospho (ST)	[illegible]	p00103	Activity-dependent neuroprotector homeobox protein
EIF6 seq: 224 - 245 + Phospho (ST)	[illegible]	p11197	Eukaryotic translation initiation factor 6
IWS1 seq: 193 - 207 + 2 Phospho (ST)	[illegible]	p14683	Protein IWS1 homolog
ZBTB21 pS411	[illegible]	p05860	Zinc finger and BTB domain-containing protein 21
HDGF pS132 pS133	[illegible]	p15602	Hepatoma-derived growth factor
UBXN7 pS350	[illegible]	p04594	UBX domain-containing protein 7
ECHS1 seq: 93 - 101 no mod	[illegible]	p20788	Enoyl-CoA hydratase, mitochondrial
PTPN14 pS486	[illegible]	p09433	Tyrosine-protein phosphatase non-receptor type 14
DDX42 pS751 pS754	[illegible]	p20558	ATP-dependent RNA helicase DDX42
PDIA3 seq: 472 - 482 no mod	[illegible]	p17410	Protein disulfide-isomerase A3
HNRNPUL2 seq: 532 - 546 no mod	[illegible]	p09644	Heterogeneous nuclear ribonucleoprotein U-like protein 2
BPTF seq: 1292 - 1304 + Phospho (ST)	[illegible]	p01267	Nucleosome-remodeling factor subunit BPTF
ATOX1 seq: 40 - 57 + Phospho (ST)	[illegible]	p01715	Copper transport protein ATOX1
FAF1 pS320	[illegible]	p12560	FAS-associated factor 1
PML pS117	[illegible]	p07071	Protein PML
NPM1 seq: 55 - 73 no mod	[illegible]	p18894	Nucleophosmin
SCAF1 seq: 297 - 343 + Phospho (ST); Phospho (Y)	[illegible]	p13739	Splicing factor, arginine/serine-rich 19
FKBP15 seq: 1161 - 1173 + 2 Phospho (ST)	[illegible]	p03841	FK506-binding protein 15
seq: 852 - 869 + Gln->pyro-Glu (N-term Q); Phospho (ST)	[illegible]	p08601	Serine/arginine repetitive matrix protein 2
AHNAK pS570 pM571	[illegible]	p13693	Neuroblast differentiation-associated protein AHNAK
EPS8L2 pS570	[illegible]	p00014	Epidermal growth factor receptor kinase substrate 8-like protein 2
PATL1 seq: 177 - 188 + Phospho (ST)	[illegible]	p03443	Protein PAT1 homolog 1
ARID1B seq: 2125 - 2143 no mod	[illegible]	p10777	AT-rich interactive domain-containing protein 1B
HSP90AA1 seq: 621 - 631 no mod	[illegible]	p18419	Heat shock protein HSP 90-alpha
NFIC seq: 452 - 486 + Phospho (ST)	[illegible]	p12234	Nuclear factor 1 C-type
ENO2 seq: 33 - 50 + Phospho (Y)	[illegible]	p20975	Gamma-enolase
RPS8 seq: 99 - 110 no mod	[illegible]	p09729	40S ribosomal protein S8
FAM63A pS103	[illegible]	p20939	Protein FAM63A
PML pS403	[illegible]	p13388	Protein PML
FCHO2 pS394	[illegible]	p14623	F-BAR domain only protein 2
RRAGC seq: 85 - 98 + Phospho (ST)	[illegible]	p09884	Ras-related GTP-binding protein C
BNIP2 pS114	[illegible]	p13160	BCL2/adenovirus E1B 19 kDa protein-interacting protein 2
CASKIN1 pT1065	[illegible]	p02553	Caskin-1
ARHGAP29 seq: 552 - 565 + Phospho (ST)	[illegible]	p05277	Rho GTPase-activating protein 29
seq: 701 - 719 + Gln->pyro-Glu (N-term Q); Phospho (ST)	[illegible]	p08475	Trinucleotide repeat-containing gene 6C protein
PRPF4B seq: 255 - 264 + Phospho (ST)	[illegible]	p19676	Serine/threonine-protein kinase PRP4 homolog
PHF2 pS899	[illegible]	p07231	Lysine-specific demethylase PHF2
LMNA pS458	[illegible]	p04852	Prelamin-A/C
HSPB1 seq: 38 - 75 + Phospho (ST)	[illegible]	p11128	Heat shock protein beta-1
TPR pT2137	[illegible]	p01930	Nucleoprotein TPR
FKBP15 seq: 1161 - 1178 + 2 Phospho (ST)	[illegible]	p03840	FK506-binding protein 15
AFAP1L2 pT669	[illegible]	p06387	Actin filament-associated protein 1-like 2
EIF4G1 pS1185	[illegible]	p05843	Eukaryotic translation initiation factor 4 gamma 1
PDXDC1 pS757	[illegible]	p10663	Pyridoxal-dependent decarboxylase domain-containing protein 1
LGALS1 seq: 101 - 108 no mod	[illegible]	p11137	Galectin-1
PDCD4 seq: 87 - 96 + Phospho (ST)	[illegible]	p05717	Programmed cell death protein 4
EIF4G1 pS1092	[illegible]	p13600	Eukaryotic translation initiation factor 4 gamma 1
TRIOBP seq: 1783 - 1808 + Phospho (ST)	[illegible]	p14952	TRIO and F-actin-binding protein
NECAP2 seq: 177 - 195 + Phospho (ST)	[illegible]	p00898	Adaptin ear-binding coat-associated protein 2
PPP4R3A seq: 674 - 715 + Phospho (ST)	[illegible]	p02618	Serine/threonine-protein phosphatase 4 regulatory subunit 3A
SRRM1 seq: 651 - 659 + 2 Phospho (ST)	[illegible]	p06733	Serine/arginine repetitive matrix protein 1
BUD13 pS197 pS201	[illegible]	p14858	BUD13 homolog
SHOC2 pT81	[illegible]	p06896	Leucine-rich repeat protein SHOC-2
CXorf23 pS15 pS17	[illegible]	p14657	Uncharacterized protein CXorf23
MPZL1 seq: 258 - 267 + Phospho (Y)	[illegible]	p05967	Myelin protein zero-like protein 1
TDRD7 seq: 180 - 188 no mod	[illegible]	p14555	Tudor domain-containing protein 7
GAPDH pM175 pT184	[illegible]	p01387	Glyceraldehyde-3-phosphate dehydrogenase
TWF1 seq: 137 - 155 + Phospho (ST)	[illegible]	p00257	Twinfilin-1
PSMA5 seq: 54 - 66 + Oxidation (M); Phospho (ST)	[illegible]	p13580	Proteasome subunit alpha type-5
KMT2A seq: 3500 - 3524 + Phospho (ST)	[illegible]	p20159	Histone-lysine N-methyltransferase 2A
SRRM1 pS626 pS628	[illegible]	p07562	Serine/arginine repetitive matrix protein 1
NCBP1 seq: 20 - 37 + Phospho (ST)	[illegible]	p12472	Nuclear cap-binding protein subunit 1
MARCKS seq: 106 - 137 + Phospho (ST)	[illegible]	p17931	Myristoylated alanine-rich C-kinase substrate
YWHAG seq: 63 - 70 no mod	[illegible]	p01342	14-3-3 protein gamma
SFN seq: 61 - 68 no mod	[illegible]	p01149	14-3-3 protein sigma
TOM1 pS160	[illegible]	p15762	Target of Myb protein 1
NPM1 pS70	[illegible]	p01909	Nucleophosmin
NUCKS1 pS58	[illegible]	p09095	Nuclear ubiquitous casein and cyclin-dependent kinase substrate 1
TRA2B seq: 94 - 104 + 2 Phospho (ST)	[illegible]	p14643	Transformer-2 protein homolog beta
SLAIN2 pS315 pS323	[illegible]	p07308	SLAIN motif-containing protein 2
IRS2 seq: 1184 - 1205 + Phospho (ST)	[illegible]	p12545	Insulin receptor substrate 2
C1orf226 seq: 220 - 229 + 2 Phospho (ST)	[illegible]	p07551	Uncharacterized protein C1orf226
EIF5B pS164	[illegible]	p12361	Eukaryotic translation initiation factor 5B
KRT7 seq: 168 - 177 no mod	[illegible]	p04889	Keratin, type II cytoskeletal 7
EPB41L1 pS510	[illegible]	p16492	Band 4.1-like protein 1
SHROOM3 seq: 935 - 942 + Phospho (ST)	[illegible]	p01232	Protein Shroom3
EIF4G1 pS1092	[illegible]	p13601	Eukaryotic translation initiation factor 4 gamma 1
PPP1R12C pS407	[illegible]	p04305	Protein phosphatase 1 regulatory subunit 12C
MYH9 seq: 1677 - 1694 no mod	[illegible]	p04907	Myosin-9
AKAP8 pS328	[illegible]	p01663	A-kinase anchor protein 8
RBM14 seq: 658 - 667 no mod	[illegible]	p21064	RNA-binding protein 14
CIT seq: 363 - 369 no mod	[illegible]	p14388	Citron Rho-interacting kinase
MPHOSPH8 seq: 125 - 151 + 2 Phospho (ST)	[illegible]	p10751	M-phase phosphoprotein 8
HNRNPA3 seq: 37 - 52 no mod	[illegible]	p11890	Heterogeneous nuclear ribonucleoprotein A3
RBM12B seq: 638 - 644 + Phospho (ST)	[illegible]	p04348	RNA-binding protein 12B
ARAF pT181 pS186	[illegible]	p08575	Serine/threonine-protein kinase A-Raf
PRX seq: 962 - 968 + Phospho (ST)	[illegible]	p16862	Periaxin
RANBP2 pT1412	[illegible]	p16860	E3 SUMO-protein ligase RanBP2
NOC2L pS56	[illegible]	p04702	Nucleolar complex protein 2 homolog
IRF2BP1 pS384	[illegible]	p13387	Interferon regulatory factor 2-binding protein 1
GRB7 seq: 60 - 91 + Phospho (ST)	[illegible]	p07541	Growth factor receptor-bound protein 7
SF3B2 pS309	[illegible]	p03859	Splicing factor 3B subunit 2
PSMC6 seq: 198 - 213 + Phospho (Y)	[illegible]	p00572	26S protease regulatory subunit 10B
EEF2K seq: 434 - 457 + Phospho (ST)	[illegible]	p17165	Eukaryotic elongation factor 2 kinase
HNRNPC seq: 251 - 275 + Phospho (ST)	[illegible]	p10174	Heterogeneous nuclear ribonucleoproteins C1/C2
NAB2 pT434	[illegible]	p10522	NGFI-A-binding protein 2
ARHGEF2 seq: 682 - 706 + Phospho (ST)	[illegible]	p17314	Rho guanine nucleotide exchange factor 2
PML seq: 504 - 515 + Phospho (ST)	[illegible]	p03818	Protein PML
TCP11L1 seq: 50 - 59 + Phospho (ST)	[illegible]	p00932	T-complex protein 11-like protein 1
EIF4EBP1 seq: 74 - 99 + Phospho (ST)	[illegible]	p18556	Eukaryotic translation initiation factor 4E-binding protein 1
CCNK seq: 322 - 331 + Phospho (ST)	[illegible]	p12721	Cyclin-K
FOXK1 pT436 pS441	[illegible]	p05750	Forkhead box protein K1
EIF4G1 seq: 1206 - 1216 + Phospho (ST)	[illegible]	p13472	Eukaryotic translation initiation factor 4 gamma 1
TTC7A pS182	[illegible]	p11102	Tetratricopeptide repeat protein 7A
EPN1 pS454	[illegible]	p15258	Epsin-1
AHNAK2 seq: 585 - 596 + Phospho (ST)	[illegible]	p16650	Protein AHNAK2
TNKS1BP1 pS851	[illegible]	p12634	182 kDa tankyrase-1-binding protein
FLNA seq: 907 - 916 no mod	[illegible]	p12024	Filamin-A
SDCBP seq: 49 - 67 + Phospho (Y)	[illegible]	p10296	Syntenin-1
BUD13 seq: 233 - 242 + Phospho (ST)	[illegible]	p14783	BUD13 homolog
SORBS1 seq: 495 - 503 + Phospho (ST)	[illegible]	p06199	Sorbin and SH3 domain-containing protein 1
ANAPC1 pS688	[illegible]	p09657	Anaphase-promoting complex subunit 1
PABPN1 pS95	[illegible]	p19958	Polyadenylate-binding protein 2
SRSF4 pS431	[illegible]	p17692	Serine/arginine-rich splicing factor 4
BCLAF1 pS496	[illegible]	p13268	Bcl-2-associated transcription factor 1
MAPT seq: 529 - 538 + Phospho (ST)	[illegible]	p02330	Microtubule-associated protein tau
EIF6 seq: 224 - 237 + Phospho (ST)	[illegible]	p11198	Eukaryotic translation initiation factor 6
SERPINB5 seq: 216 - 224 no mod	[illegible]	p14183	Serpin B5
SOCS6 pS12	[illegible]	p05878	Suppressor of cytokine signaling 6
C1orf174 pS189	[illegible]	p08225	UPF0688 protein C1orf174
CHD5 seq: 1297 - 1306 no mod	[illegible]	p17846	Chromodomain-helicase-DNA-binding protein 5
HYOU1 seq: 199 - 217 no mod	[illegible]	p01187	Hypoxia up-regulated protein 1
MYH14 seq: 1755 - 1775 no mod	[illegible]	p11713	Myosin-14
KRT18 seq: 265 - 300 no mod	[illegible]	p17833	Keratin, type I cytoskeletal 18
PLEKHM3 pS132	[illegible]	p03316	Pleckstrin homology domain-containing family M member 3
NCL pT76	[illegible]	p01736	Nucleolin
WIPI2 seq: 375 - 406 + Phospho (ST)	[illegible]	p12083	WD repeat domain phosphoinositide-interacting protein 2
CSPP1 pS527	[illegible]	p05718	Centrosome and spindle pole-associated protein 1
SLK pS779	[illegible]	p18277	STE20-like serine/threonine-protein kinase
PYGB pS15	[illegible]	p12678	Glycogen phosphorylase, brain form
OSBP seq: 324 - 375 + Phospho (ST)	[illegible]	p16259	Oxysterol-binding protein 1
BUD13 seq: 298 - 303 + Phospho (ST)	[illegible]	p02154	BUD13 homolog
IRS2 seq: 1147 - 1170 + Phospho (ST)	[illegible]	p14612	Insulin receptor substrate 2
MLLT4 seq: 1691 - 1710 + Phospho (ST)	[illegible]	p18055	Afadin
PPP1R3D pS46	[illegible]	p20947	Protein phosphatase 1 regulatory subunit 3D
SRSF6 pS314 pS316	[illegible]	p19673	Serine/arginine-rich splicing factor 6
CCT2 seq: 432 - 441 no mod	[illegible]	p17906	T-complex protein 1 subunit beta
PHRF1 pS1124	[illegible]	p17896	PHD and RING finger domain-containing protein 1
EP400 pT3152	[illegible]	p11475	E1A-binding protein p400
TIGAR seq: 146 - 174 + Phospho (ST)	[illegible]	p17993	Fructose-2,6-bisphosphatase TIGAR
STIP1 pS481	[illegible]	p14855	Stress-induced-phosphoprotein 1
SBDS seq: 110 - 118 no mod	[illegible]	p18770	Ribosome maturation protein SBDS
MSL1 seq: 448 - 459 + Phospho (ST)	[illegible]	p17119	Male-specific lethal 1 homolog
SRSF4 seq: 277 - 287 no mod	[illegible]	p13803	Serine/arginine-rich splicing factor 4
USP10 seq: 74 - 98 + Phospho (ST)	[illegible]	p02310	Ubiquitin carboxyl-terminal hydrolase 10
CBX3 pS93 pS95	[illegible]	p07211	Chromobox protein homolog 3
RNF113A seq: 78 - 101 + Phospho (ST); Phospho (Y)	[illegible]	p20965	RING finger protein 113A
REPS1 pS562	[illegible]	p03695	RalBP1-associated Eps domain-containing protein 1
PAK4 pS104	[illegible]	p07501	Serine/threonine-protein kinase PAK 4
LMO7 seq: 112 - 130 + Phospho (ST)	[illegible]	p13897	LIM domain only protein 7
ZFAND5 seq: 47 - 68 + Oxidation (M); Phospho (ST)	[illegible]	p09888	AN1-type zinc finger protein 5
L2HGDH seq: 406 - 431 no mod	[illegible]	p19837	L-2-hydroxyglutarate dehydrogenase, mitochondrial
SOS1 pS1161	[illegible]	p06751	Son of sevenless homolog 1
SND1 seq: 250 - 255 no mod	[illegible]	p16777	Staphylococcal nuclease domain-containing protein 1
CANX pS554	[illegible]	p08447	Calnexin
MPST pS3	[illegible]	p19535	3-mercaptopyruvate sulfurtransferase
KIFAP3 seq: 58 - 72 + Phospho (ST)	[illegible]	p11487	Kinesin-associated protein 3
LDHA seq: 172 - 177 no mod	[illegible]	p00252	L-lactate dehydrogenase A chain
PRRC2A seq: 1686 - 1708 + Phospho (ST)	[illegible]	p06936	Protein PRRC2A
COTL1 seq: 119 - 126 no mod	[illegible]	p17513	Coactosin-like protein
MIA3 seq: 1658 - 1696 + Phospho (ST)	[illegible]	p07372	Melanoma inhibitory activity protein 3
EIF4EBP1 seq: 21 - 51 + Phospho (ST)	[illegible]	p00606	Eukaryotic translation initiation factor 4E-binding protein 1
DMXL2 pS1984	[illegible]	p01698	DmX-like protein 2
IRS2 seq: 1172 - 1183 + Phospho (ST)	[illegible]	p14707	Insulin receptor substrate 2
EIF4G1 seq: 1207 - 1216 + Phospho (ST)	[illegible]	p21036	Eukaryotic translation initiation factor 4 gamma 1
PTK2 pS910	[illegible]	p10959	Focal adhesion kinase 1
TRAF3IP3 seq: 108 - 113 + Phospho (ST)	[illegible]	p07241	TRAF3-interacting JNK-activating modulator
PRPF4B seq: 408 - 413 + Phospho (ST)	[illegible]	p07082	Serine/threonine-protein kinase PRP4 homolog
KIAA1462 pS231	[illegible]	p04045	Junctional protein associated with coronary artery disease
SRRM2 seq: 1589 - 1594 + Phospho (ST)	[illegible]	p04099	Serine/arginine repetitive matrix protein 2
MAP2K2 pS222	[illegible]	p12123	Dual specificity mitogen-activated protein kinase kinase 2
IRS2 pS915	[illegible]	p04649	Insulin receptor substrate 2
TRUB1 seq: 16 - 38 + Phospho (ST)	[illegible]	p02936	Probable tRNA pseudouridine synthase 1
FANCM seq: 1718 - 1723 + Phospho (ST)	[illegible]	p00887	Fanconi anemia group M protein
ZC3H13 pS873	[illegible]	p11277	Zinc finger CCCH domain-containing protein 13
RSF1 pS473	[illegible]	p17816	Remodeling and spacing factor 1
CD2AP pS458	[illegible]	p03383	CD2-associated protein
PPA1 seq: 156 - 176 no mod	[illegible]	p01408	Inorganic pyrophosphatase
IWS1 pS377	[illegible]	p10200	Protein IWS1 homolog
FAM117B seq: 135 - 173 + 2 Phospho (ST)	[illegible]	p15215	Protein FAM117B
LIMD1 seq: 255 - 293 + 2 Phospho (ST)	[illegible]	p03924	LIM domain-containing protein 1
OSMR seq: 286 - 291 no mod	[illegible]	p13021	Oncostatin-M-specific receptor subunit beta
NOLC1 pS538	[illegible]	p15213	Nucleolar and coiled-body phosphoprotein 1
EEF1A1P5 seq: 386 - 392 no mod	[illegible]	p13011	Putative elongation factor 1-alpha-like 3

Peptide	Intensity values	Accession	Protein
BCL9L pS21 pS25	38324 37149 39656 2E+08 33854 45779 2E+08 34892 2E+08 2E+08 56234 3E+08 39754 40879 3E+08 40867 3E+08 34984 3E+08 2E+08 39759 4E+08 21442 19024 3E+08 68554 44396 [illegible] 2E+08 30272 1E+08	p17928	B-cell CLL/lymphoma 9-like protein
ILF3 seq: 372 - 385 + Phospho (ST)	3E+06 7E+06 2E+06 6E+06 3E+06 1E+08 2E+07 2E+06 2E+07 8E+06 1E+08 4E+07 1E+07 1E+07 2E+07 3E+07 2E+07 2E+06 2E+07 6E+06 1E+07 3E+06 1E+07 8E+06 3E+07 8E+06 44396 [illegible] 2E+06 30272 25732	p06907	Interleukin enhancer-binding factor 3
HNRNPC pS233	2E+07 1E+07 39656 2E+07 33854 1E+08 1E+07 34892 2E+07 4E+07 5E+07 2E+07 39754 4E+06 1E+08 7E+07 2E+07 34984 3E+07 3E+07 9E+06 3E+07 2E+07 19024 2E+08 1E+08 4E+07 [illegible] 5E+07 7E+07 6E+07	p09710	Heterogeneous nuclear ribonucleoproteins C1/C2
PRRC2B seq: 1390 - 1413 + Phospho (ST)	2E+07 37149 39656 2E+07 33854 45779 25552 34892 5E+07 2E+08 3E+07 4E+07 1E+07 4E+07 2E+08 3E+08 8E+07 34984 19162 1E+08 4E+08 1E+08 1E+07 1E+08 2E+08 1E+09 1E+08 [illegible] 8E+07 5E+08 6E+08	p07481	Protein PRRC2B
STAT3 seq: 181 - 197 no mod	38324 37149 39656 1E+07 33854 8E+07 25552 34892 2E+07 26736 4E+08 3E+07 1E+07 2E+07 18798 9E+07 33072 34984 2E+07 8E+06 1E+08 50401 21442 2E+07 42741 8E+07 44396 [illegible] 25947 30272 25732	p04253	Signal transducer and activator of transcription 3
CANX seq: 552 - 570 + Phospho (ST)	38324 37149 39656 1E+07 8E+06 8E+07 25552 2E+07 2E+07 3E+07 56234 4E+07 5E+07 2E+07 4E+07 5E+07 9E+07 4E+07 2E+07 2E+07 2E+07 4E+07 7E+07 9E+06 3E+07 68554 5E+06 [illegible] 25947 30272 25732	p08446	Calnexin
TPR seq: 1657 - 1690 + Phospho (ST)	38324 37149 39656 13654 33854 45779 25552 34892 34104 26736 56234 53206 39754 40879 18798 4E+07 33072 34984 19162 3E+07 39759 50401 21442 4E+07 42741 4E+08 1E+08 [illegible] 1E+08 1E+08 25732	p15908	Nucleoprotein TPR
ANXA1 seq: 59 - 71 no mod	38324 37149 39656 13654 33854 3E+08 25552 34892 34104 26736 56234 53206 39754 40879 18798 40867 33072 34984 19162 28832 39759 50401 21442 19024 42741 3E+08 2E+07 [illegible] 5E+08 4E+08 5E+08	p15058	Annexin A1
EIF1B seq: 67 - 90 no mod	38324 37149 39656 13654 33854 45779 25552 34892 2E+07 26736 56234 53206 39754 40879 18798 40867 2E+07 34984 1E+07 28832 39759 50401 21442 19024 42741 68554 44396 [illegible] 25947 30272 25732	p16879	Eukaryotic translation initiation factor 1b
HNRNPC pS233	1E+07 6E+06 39656 1E+07 33854 7E+07 4E+06 34892 7E+06 3E+07 3E+07 8E+06 39754 40879 6E+07 4E+07 1E+07 34984 2E+07 2E+07 7E+06 2E+07 21442 19024 1E+08 7E+07 3E+07 [illegible] 3E+07 4E+07 4E+07	p09709	Heterogeneous nuclear ribonucleoproteins C1/C2
SHTN1 pS249	38324 3E+07 3E+07 1E+08 6E+07 1E+09 2E+08 1E+08 2E+08 5E+07 8E+08 3E+08 1E+08 2E+08 4E+07 2E+08 4E+07 2E+08 3E+08 3E+08 2E+08 5E+07 9E+07 2E+08 2E+08 68554 44396 [illegible] 25947 4E+07 3E+07	p06798	Shootin-1
PHLDB2 pS212	38324 37149 39656 13654 33854 45779 25552 34892 34104 26736 56234 53206 39754 40879 18798 40867 33072 2E+07 2E+07 28832 1E+07 50401 5E+06 19024 42741 3E+08 6E+07 [illegible] 1E+08 8E+07 1E+08	p12856	Pleckstrin homology-like domain family B member 2
SPEN pS1006	38324 37149 39656 13654 639890 3E+06 25552 34892 34104 26736 56234 53206 39754 40879 18798 1E+06 33072 34984 4E+06 3E+06 39759 50401 21442 19024 5E+06 2E+06 44396 [illegible] 25947 3E+06 1E+06	p12537	Msx2-interacting protein
NCL pT76	38324 37149 39656 13654 352755 45779 25552 34892 628547 476230 1E+07 53206 39754 40879 391038 40867 5E+06 34984 452790 3E+06 39759 50401 21442 889554 2E+07 1E+08 2E+07 [illegible] 6E+07 6E+07 3E+07	p12454	Nucleolin
COG1 seq: 443 - 463 + Phospho (ST)	2E+06 6E+06 1E+07 2E+07 6E+06 2E+08 2E+07 2E+07 4E+07 1E+07 8E+07 2E+07 8E+07 4E+07 4E+07 1E+08 6E+06 3E+07 2E+07 1E+07 3E+07 5E+06 3E+07 4E+07 3E+07 68554 44396 [illegible] 2E+07 6E+06 1E+07	p17448	Conserved oligomeric Golgi complex subunit 1
ABHD11 seq: 97 - 103 no mod	2E+07 37149 39656 13654 33854 1E+08 25552 34892 34104 26736 7E+08 8E+06 39754 40879 3E+06 4E+07 33072 34984 19162 28832 3E+07 7E+06 21442 2E+06 6E+06 5E+07 44396 [illegible] 25947 30272 25732	p01129	Alpha/beta hydrolase domain-containing protein 11
TGM2 seq: 241 - 262 + Oxidation (M)	38324 37149 39656 5E+06 33854 45779 25552 1E+08 34104 26736 7E+06 53206 39754 40879 18798 40867 2E+07 34984 19162 8E+07 39759 1E+08 4E+06 1E+07 5E+06 6E+07 44396 [illegible] 8E+07 1E+07 3E+07	p00505	Protein-glutamine gamma-glutamyltransferase 2
ANXA2 seq: 158 - 169 no mod	38324 37149 39656 13654 4E+06 4E+07 25552 34892 34104 26736 2E+08 53206 39754 4E+06 18798 6E+06 33072 34984 1E+07 28832 6E+06 50401 1E+07 4E+06 42741 4E+07 6E+06 [illegible] 3E+07 8E+06 4E+07	p18748	Annexin A2
KRT19 seq: 217 - 226 no mod	[illegible]	[illegible]	Keratin, type I cytoskeletal 19
[illegible] 107 - 124 + Gln->pyro-Glu (N-term Q); 2 Phospho (ST)	[illegible]	[illegible]	Transcriptional coactivator YAP1
CLK1 seq: 138 - 160 + Phospho (ST)	[illegible]	[illegible]	Dual specificity protein kinase CLK1
PPP1R13L seq: 262 - 271 + Phospho (ST)	[illegible]	[illegible]	RelA-associated inhibitor
SGTA seq: 301 - 313 no mod	[illegible]	[illegible]	Small glutamine-rich tetratricopeptide repeat-containing protein alpha
RGCC seq: 86 - 114 + Phospho (ST)	[illegible]	[illegible]	Regulator of cell cycle RGCC
VIM seq: 208 - 217 + Gln->pyro-Glu (N-term Q)	[illegible]	[illegible]	Vimentin
PHRF1 pS915	[illegible]	[illegible]	PHD and RING finger domain-containing protein 1
RAD23B seq: 145 - 173 + 2 Phospho (ST)	[illegible]	[illegible]	UV excision repair protein RAD23 homolog B
PDAP1 seq: 56 - 73 + 2 Phospho (ST)	[illegible]	[illegible]	28 kDa heat- and acid-stable phosphoprotein
TJP2 pS978	[illegible]	[illegible]	Tight junction protein ZO-2
PDHA1 seq: 227 - 235 + Phospho (ST)	[illegible]	[illegible]	Pyruvate dehydrogenase E1 component subunit alpha, somatic form, mitochondrial
FSCN1 seq: 69 - 82 no mod	[illegible]	[illegible]	Fascin
PA2G4 seq: 333 - 344 no mod	[illegible]	[illegible]	Proliferation-associated protein 2G4
GAPDHS pM303	[illegible]	[illegible]	Glyceraldehyde-3-phosphate dehydrogenase, testis-specific
KDM1A pT59	[illegible]	[illegible]	Lysine-specific histone demethylase 1A
MARCKSL1 seq: 111 - 144 + Phospho (ST)	[illegible]	[illegible]	MARCKS-related protein
MICALL1 pS640	[illegible]	[illegible]	MICAL-like protein 1
AFF4 pS549	[illegible]	[illegible]	AF4/FMR2 family member 4
IMUP seq: 75 - 82 + Gln->pyro-Glu (N-term Q)	[illegible]	[illegible]	Immortalization up-regulated protein
HLA-B seq: 60 - 68 no mod	[illegible]	[illegible]	HLA class I histocompatibility antigen, B-27 alpha chain
DIP2B seq: 258 - 272 + Phospho (ST)	[illegible]	[illegible]	Disco-interacting protein 2 homolog B
FOXK2 pS424 pS428	[illegible]	[illegible]	Forkhead box protein K2
ERBB2 seq: 1073 - 1096 + 2 Phospho (ST)	[illegible]	[illegible]	Receptor tyrosine-protein kinase erbB-2
TCF20 pS1259	[illegible]	[illegible]	Transcription factor 20
MAP4K4 pS868	[illegible]	[illegible]	Mitogen-activated protein kinase kinase kinase kinase 4
ZNF609 seq: 465 - 479 + Oxidation (M); Phospho (ST)	[illegible]	[illegible]	Zinc finger protein 609
ABI1 seq: 195 - 219 + Phospho (ST)	[illegible]	[illegible]	Abl interactor 1
KRT8 pS74	[illegible]	[illegible]	Keratin, type II cytoskeletal 8
KIAA1522 seq: 557 - 586 + 2 Phospho (ST)	[illegible]	[illegible]	Uncharacterized protein KIAA1522
PLEC pS4386 pS4389	[illegible]	[illegible]	Plectin
UBE2O seq: 1165 - 1213 + Phospho (ST)	[illegible]	[illegible]	(E3-independent) E2 ubiquitin-conjugating enzyme
MICALL1 seq: 536 - 570 + Phospho (ST)	[illegible]	[illegible]	MICAL-like protein 1
STRN3 seq: 240 - 275 + Phospho (ST)	[illegible]	[illegible]	Striatin-3
PFKFB2 pS466	[illegible]	[illegible]	6-phosphofructo-2-kinase/fructose-2,6-bisphosphatase 2
ANKRD20A4 pT54	[illegible]	[illegible]	Ankyrin repeat domain-containing protein 20A4
EIF3A pS584	[illegible]	[illegible]	Eukaryotic translation initiation factor 3 subunit A
ZDHHC5 seq: 294 - 322 + 2 Phospho (ST)	[illegible]	[illegible]	Palmitoyltransferase ZDHHC5
RSF1 seq: 1357 - 1378 + 2 Phospho (ST)	[illegible]	[illegible]	Remodeling and spacing factor 1
MLLT4 pS1799	[illegible]	[illegible]	Afadin
JPH1 pS162	[illegible]	[illegible]	Junctophilin-1
ACLY seq: 453 - 468 + Phospho (ST)	[illegible]	[illegible]	ATP-citrate synthase
PAN3 seq: 349 - 358 + Phospho (ST)	[illegible]	[illegible]	PAB-dependent poly(A)-specific ribonuclease subunit PAN3
ARHGAP32 seq: 1794 - 1810 + Phospho (ST)	[illegible]	[illegible]	Rho GTPase-activating protein 32
CCDC85C seq: 156 - 200 + Phospho (ST)	[illegible]	[illegible]	Coiled-coil domain-containing protein 85C
AHNAK seq: 2704 - 2720 + Phospho (ST)	[illegible]	[illegible]	Neuroblast differentiation-associated protein AHNAK
ACLY pS455	[illegible]	[illegible]	ATP-citrate synthase
MAP4K4 pS900	[illegible]	[illegible]	Mitogen-activated protein kinase kinase kinase kinase 4
CCDC120 seq: 358 - 368 + Phospho (ST)	[illegible]	[illegible]	Coiled-coil domain-containing protein 120
YWHAG seq: 78 - 83 no mod	[illegible]	[illegible]	14-3-3 protein gamma
AMOTL1 seq: 109 - 157 + Phospho (ST)	[illegible]	[illegible]	Angiomotin-like protein 1
MBD3 pS56 pM57	[illegible]	[illegible]	Methyl-CpG-binding domain protein 3
SYNRG seq: 849 - 875 + 2 Phospho (ST)	[illegible]	[illegible]	Synergin gamma
ENAH seq: 484 - 499 + Phospho (ST)	[illegible]	[illegible]	Protein enabled homolog
HDGFRP2 pS454 pM459	[illegible]	[illegible]	Hepatoma-derived growth factor-related protein 2
EAF1 seq: 157 - 175 + 2 Phospho (ST)	[illegible]	[illegible]	ELL-associated factor 1
SOX13 seq: 331 - 352 + Phospho (ST)	[illegible]	[illegible]	Transcription factor SOX-13
CEP170 seq: 1257 - 1264 + Phospho (ST)	[illegible]	[illegible]	Centrosomal protein of 170 kDa
PEA15 pS104	[illegible]	[illegible]	Astrocytic phosphoprotein PEA-15
HIST1H1D seq: 67 - 82 no mod	[illegible]	[illegible]	Histone H1.3
PHLDA2 seq: 117 - 150 + Phospho (ST)	[illegible]	[illegible]	Pleckstrin homology-like domain family A member 2
PLEC seq: 4361 - 4383 + Phospho (ST)	[illegible]	[illegible]	Plectin
PCBP1 seq: 244 - 268 + Oxidation (M); Phospho (ST)	[illegible]	[illegible]	Poly(rC)-binding protein 1
GRB10 seq: 426 - 443 + Phospho (ST)	[illegible]	[illegible]	Growth factor receptor-bound protein 10
MAP3K4 seq: 497 - 519 + Phospho (ST)	[illegible]	[illegible]	Mitogen-activated protein kinase kinase kinase 4
H1FX seq: 24 - 36 + Phospho (ST)	[illegible]	[illegible]	Histone H1x
R3HDM1 seq: 970 - 985 + Phospho (ST)	[illegible]	[illegible]	R3H domain-containing protein 1
LUZP1 seq: 406 - 419 no mod	[illegible]	[illegible]	Leucine zipper protein 1
TPR seq: 644 - 669 + Phospho (ST)	[illegible]	[illegible]	Nucleoprotein TPR
KLHDC4 seq: 400 - 432 + Phospho (ST)	[illegible]	[illegible]	Kelch domain-containing protein 4
RIOK3 pS512	[illegible]	[illegible]	Serine/threonine-protein kinase RIO3
ANK3 pS1459	[illegible]	[illegible]	Ankyrin-3
LRBA seq: 1666 - 1674 no mod	[illegible]	[illegible]	Lipopolysaccharide-responsive and beige-like anchor protein
TFEB seq: 325 - 347 + Phospho (ST)	[illegible]	[illegible]	Transcription factor EB
SARG pS502	[illegible]	[illegible]	Specifically androgen-regulated gene protein
TBC1D14 pM684 pS688	[illegible]	[illegible]	TBC1 domain family member 14
HIBADH seq: 285 - 297 no mod	[illegible]	[illegible]	3-hydroxyisobutyrate dehydrogenase, mitochondrial
TP53BP2 seq: 736 - 751 + Phospho (ST)	[illegible]	[illegible]	Apoptosis-stimulating of p53 protein 2
ACIN1 pS240 pS243	[illegible]	[illegible]	Apoptotic chromatin condensation inducer in the nucleus
GOLGA4 seq: 7 - 34 + Phospho (ST)	[illegible]	[illegible]	Golgin subfamily A member 4
PFKFB2 seq: 464 - 477 + Phospho (ST)	[illegible]	[illegible]	6-phosphofructo-2-kinase/fructose-2,6-bisphosphatase 2
UBXN6 seq: 85 - 109 + Phospho (ST)	[illegible]	[illegible]	UBX domain-containing protein 6
CDK17 seq: 14 - 44 no mod	[illegible]	[illegible]	Cyclin-dependent kinase 17
VCP seq: 714 - 732 no mod	[illegible]	[illegible]	Transitional endoplasmic reticulum ATPase
HSP90B1 seq: 672 - 683 no mod	[illegible]	[illegible]	Endoplasmin
NASP seq: 672 - 697 + Phospho (ST)	[illegible]	[illegible]	Nuclear autoantigenic sperm protein
SPTAN1 seq: 1024 - 1045 + Phospho (ST)	[illegible]	[illegible]	Spectrin alpha chain, non-erythrocytic 1
MAF1 seq: 46 - 71 + 2 Phospho (ST)	[illegible]	[illegible]	Repressor of RNA polymerase III transcription MAF1 homolog
BAIAP2L1 pS331 pM338 pM339	[illegible]	[illegible]	Brain-specific angiogenesis inhibitor 1-associated protein 2-like protein 1
NDRG1 seq: 362 - 388 + 2 Phospho (ST)	[illegible]	[illegible]	Protein NDRG1
STK10 pS438	[illegible]	[illegible]	Serine/threonine-protein kinase 10
PDS5B pS1358	[illegible]	[illegible]	Sister chromatid cohesion protein PDS5 homolog B
SOX11 seq: 126 - 158 + Phospho (ST)	[illegible]	[illegible]	Transcription factor SOX-11
TPR seq: 616 - 643 + Phospho (ST)	[illegible]	[illegible]	Nucleoprotein TPR
AFAP1 seq: 675 - 703 + Phospho (ST)	[illegible]	[illegible]	Actin filament-associated protein 1
MAST2 pS92	[illegible]	[illegible]	Microtubule-associated serine/threonine-protein kinase 2
PLEC seq: 4361 - 4383 + Oxidation (M); Phospho (ST)	[illegible]	[illegible]	Plectin
ARHGAP21 seq: 1739 - 1750 + Phospho (ST)	[illegible]	[illegible]	Rho GTPase-activating protein 21
PAK4 pS474 pM482	[illegible]	[illegible]	Serine/threonine-protein kinase PAK 4
WDR1 seq: 204 - 219 no mod	[illegible]	[illegible]	WD repeat-containing protein 1
SARG pS519	[illegible]	[illegible]	Specifically androgen-regulated gene protein
PCBP2 seq: 246 - 276 + Oxidation (M); Phospho (ST)	[illegible]	[illegible]	Poly(rC)-binding protein 2
MED13 pS395	[illegible]	[illegible]	Mediator of RNA polymerase II transcription subunit 13
RANBP3 seq: 122 - 138 + Phospho (ST)	[illegible]	[illegible]	Ran-binding protein 3
AKR1A1 seq: 146 - 153 no mod	[illegible]	[illegible]	Alcohol dehydrogenase [NADP(+)]
CAT seq: 432 - 444 no mod	[illegible]	[illegible]	Catalase
LARP1 seq: 766 - 778 + Phospho (ST); Phospho (Y)	[illegible]	[illegible]	La-related protein 1
LARP1 pS766 pS774	[illegible]	[illegible]	La-related protein 1
FOXQ1 pS248	[illegible]	[illegible]	Forkhead box protein Q1
CEP170B seq: 419 - 431 + Phospho (ST)	[illegible]	[illegible]	Centrosomal protein of 170 kDa protein B
USP18 pT87	[illegible]	[illegible]	Ubl carboxyl-terminal hydrolase 18
PABPN1 pS150	[illegible]	[illegible]	Polyadenylate-binding protein 2
DENND4C seq: 823 - 842 + Phospho (ST)	[illegible]	[illegible]	DENN domain-containing protein 4C
YAP1 seq: 103 - 124 + 2 Phospho (ST)	[illegible]	[illegible]	Transcriptional coactivator YAP1
SHROOM1 seq: 306 - 342 + Phospho (ST)	[illegible]	[illegible]	Protein Shroom1
PFKFB2 seq: 481 - 496 + 2 Phospho (ST)	[illegible]	[illegible]	6-phosphofructo-2-kinase/fructose-2,6-bisphosphatase 2
MISP seq: 263 - 289 + Phospho (ST)	[illegible]	[illegible]	Mitotic interactor and substrate of PLK1
CEP170B pS1179	[illegible]	[illegible]	Centrosomal protein of 170 kDa protein B
ACLY pS455	[illegible]	[illegible]	ATP-citrate synthase
PHLDB2 pS334	[illegible]	[illegible]	Pleckstrin homology-like domain family B member 2
EEF1D seq: 112 - 123 + Phospho (ST)	[illegible]	[illegible]	Elongation factor 1-delta
CLMN pS402	[illegible]	[illegible]	Calmin
PXN pS272	[illegible]	[illegible]	Paxillin
AFF4 pS836	[illegible]	[illegible]	AF4/FMR2 family member 4
ZNF609 pS447	[illegible]	[illegible]	Zinc finger protein 609
PLEC seq: 4384 - 4401 + Phospho (ST); Phospho (Y)	[illegible]	[illegible]	Plectin
[illegible] + Gln->pyro-Glu (N-term Q); Oxidation (M); Phospho (ST)	[illegible]	[illegible]	Poly(rC)-binding protein 1
HUWE1 seq: 718 - 761 + Phospho (ST)	[illegible]	[illegible]	E3 ubiquitin-protein ligase HUWE1
EEPD1 pS21	[illegible]	[illegible]	Endonuclease/exonuclease/phosphatase family domain-containing protein 1
MISP seq: 154 - 171 + Phospho (ST)	[illegible]	[illegible]	Mitotic interactor and substrate of PLK1
FAM134A pT279 pS281 pS283	[illegible]	[illegible]	Protein FAM134A
TMX1 pS270	[illegible]	[illegible]	Thioredoxin-related transmembrane protein 1
FASN seq: 1405 - 1423 no mod	[illegible]	[illegible]	Fatty acid synthase
ACIN1 seq: 236 - 257 + Phospho (ST)	[illegible]	[illegible]	Apoptotic chromatin condensation inducer in the nucleus
EIF4EBP1 seq: 64 - 99 + Oxidation (M); Phospho (ST)	[illegible]	[illegible]	Eukaryotic translation initiation factor 4E-binding protein 1
JOSD1 seq: 11 - 30 + Phospho (ST)	[illegible]	[illegible]	Josephin-1
CASC3 pS45	[illegible]	[illegible]	Protein CASC3
KMT2D pS2640	[illegible]	[illegible]	Histone-lysine N-methyltransferase 2D
FAM49B seq: 168 - 186 no mod	[illegible]	[illegible]	Protein FAM49B
PPFIA1 pT1159	[illegible]	[illegible]	Liprin-alpha-1
WDR20 seq: 491 - 500 + Phospho (ST)	[illegible]	[illegible]	WD repeat-containing protein 20
[illegible] Gln->pyro-Glu (N-term Q); 2 Oxidation (M); Phospho (ST)	[illegible]	[illegible]	Poly(rC)-binding protein 1
SORBS2 seq: 163 - 183 + Phospho (ST)	[illegible]	[illegible]	Sorbin and SH3 domain-containing protein 2
DTL seq: 483 - 498 + Phospho (ST)	[illegible]	[illegible]	Denticleless protein homolog
MYC pS62	[illegible]	[illegible]	Myc proto-oncogene protein
NPM1 pS254	[illegible]	[illegible]	Nucleophosmin
CLMN seq: 920 - 932 + Phospho (ST)	[illegible]	[illegible]	Calmin
RPLP0P6 pS304 pS307 pM311	[illegible]	[illegible]	60S acidic ribosomal protein P0-like
[illegible]IAIAP2L1 seq: 248 - 266 + Oxidation (M); 2 Phospho (ST)	[illegible]	[illegible]	Brain-specific angiogenesis inhibitor 1-associated protein 2-like protein 1
TNRC6A pS1890	[illegible]	[illegible]	Trinucleotide repeat-containing gene 6A protein
RAB11FIP5 seq: 393 - 411 + Phospho (ST)	[illegible]	[illegible]	Rab11 family-interacting protein 5
CCDC71L pT185	[illegible]	[illegible]	Coiled-coil domain-containing protein 71L
TPD52L1 pS149	[illegible]	[illegible]	Tumor protein D53
RPL13A seq: 188 - 197 no mod	[illegible]	[illegible]	60S ribosomal protein L13a
SFPQ pM683 pT687	[illegible]	[illegible]	Splicing factor, proline- and glutamine-rich
PKP3 pT86	[illegible]	[illegible]	Plakophilin-3
GAB1 pS253	[illegible]	[illegible]	GRB2-associated-binding protein 1
SRRM1 pT872 pS874	[illegible]	[illegible]	Serine/arginine repetitive matrix protein 1
REPS1 seq: 724 - 739 + Phospho (ST)	[illegible]	[illegible]	RalBP1-associated Eps domain-containing protein 1
TBC1D4 pS570	[illegible]	[illegible]	TBC1 domain family member 4
HP1BP3 seq: 150 - 160 + Oxidation (M); Phospho (ST)	[illegible]	[illegible]	Heterochromatin protein 1-binding protein 3
[illegible]IAIAP2L1 seq: 248 - 266 + Oxidation (M); Phospho (ST)	[illegible]	[illegible]	Brain-specific angiogenesis inhibitor 1-associated protein 2-like protein 1
SRRM1 seq: 444 - 458 + Phospho (ST)	[illegible]	[illegible]	Serine/arginine repetitive matrix protein 1
HNRNPF seq: 53 - 68 + Gln->pyro-Glu (N-term Q)	[illegible]	[illegible]	Heterogeneous nuclear ribonucleoprotein F
SYNRG pS1087	[illegible]	[illegible]	Synergin gamma
MIER2 seq: 129 - 162 + Phospho (ST)	[illegible]	[illegible]	Mesoderm induction early response protein 2
PLEC seq: 4382 - 4401 + 2 Phospho (ST)	[illegible]	[illegible]	Plectin
PROSER2 pS179	[illegible]	[illegible]	Proline and serine-rich protein 2
VCP seq: 600 - 615 no mod	[illegible]	[illegible]	Transitional endoplasmic reticulum ATPase
RTKN pS520	[illegible]	[illegible]	Rhotekin
PROSER2 pS43	[illegible]	[illegible]	Proline and serine-rich protein 2
NOS1AP pM369 pS371 pS374	[illegible]	[illegible]	Carboxyl-terminal PDZ ligand of neuronal nitric oxide synthase protein
PTPN14 pS593	[illegible]	[illegible]	Tyrosine-protein phosphatase non-receptor type 14
CTNND1 seq: 152 - 180 + Phospho (ST)	[illegible]	[illegible]	Catenin delta-1
PLEC seq: 4361 - 4383 + Phospho (ST)	[illegible]	[illegible]	Plectin
AMOTL1 pS793	[illegible]	[illegible]	Angiomotin-like protein 1
AKT1S1 seq: 202 - 223 + Phospho (ST)	[illegible]	[illegible]	Proline-rich AKT1 substrate 1
TST D1 seq: 85 - 93 no mod	[illegible]	[illegible]	Thiosulfate sulfurtransferase/rhodanese-like domain-containing protein 1
UBA1 seq: 227 - 239 no mod	[illegible]	[illegible]	Ubiquitin-like modifier-activating enzyme 1
SIPA1L2 seq: 1544 - 1556 + Phospho (ST)	[illegible]	[illegible]	Signal-induced proliferation-associated 1-like protein 2
UBA3 seq: 593 - 604 no mod	[illegible]	[illegible]	Ubiquitin-like modifier-activating enzyme 3
CALM1 seq: 92 - 107 + Phospho (Y)	[illegible]	[illegible]	Calmodulin
HNRNPM pM404	[illegible]	[illegible]	Heterogeneous nuclear ribonucleoprotein M
EFS pS298	[illegible]	[illegible]	Embryonal Fyn-associated substrate
[illegible]eq: 512 - 524 + Gln->pyro-Glu (N-term Q); Phospho (ST)	[illegible]	[illegible]	SPATS2-like protein
BCL7C seq: 110 - 142 + Phospho (ST)	[illegible]	[illegible]	B-cell CLL/lymphoma 7 protein family member C
FAM13A seq: 650 - 670 + Phospho (Y)	[illegible]	[illegible]	Protein FAM13A
PLEC pS4386	[illegible]	[illegible]	Plectin
SSH3 seq: 616 - 640 no mod	[illegible]	[illegible]	Protein phosphatase Slingshot homolog 3
ZNF326 seq: 117 - 125 no mod	[illegible]	[illegible]	DBIRD complex subunit ZNF326
HNRNPU seq: 602 - 609 no mod	[illegible]	[illegible]	Heterogeneous nuclear ribonucleoprotein U
PRRC2C pS1568	[illegible]	[illegible]	Protein PRRC2C

RPS17 pS113	38324	2E+07	[illegible]	1E+08	25732	p11453	40S ribosomal protein S17
ARHGAP29 seq: 349 - 362 + Phospho (ST)	38324	5E+06	[illegible]	6E+07	6E+07	p20855	Rho GTPase-activating protein 29
ALKBH5 seq: 359 - 369 + Phospho (ST)	2E+08	2E+08	[illegible]	1E+08	2E+08	p07353	RNA demethylase ALKBH5
GAB2 pT391	2E+07	4E+07	[illegible]	30272	6E+06	p06991	GRB2-associated-binding protein 2
TBC1D4 pS666	7E+08	8E+08	[illegible]	4E+08	4E+08	p19806	TBC1 domain family member 4
FAM83B pS466	1E+08	5E+07	[illegible]	9E+06	1E+06	p06681	Protein FAM83B
N4BP3 seq: 94 - 105 + Phospho (ST)	2E+08	2E+08	[illegible]	30272	25732	p20707	NEDD4-binding protein 3
LSM14B pS165	5E+08	4E+08	[illegible]	6E+07	25732	p04526	Protein LSM14 homolog B
SLC9A3R1 seq: 41 - 50 no mod	5E+08	7E+08	[illegible]	30272	25732	p10442	Na(+)/H(+) exchange regulatory cofactor NHE-RF1
SORBS2 pS302	6E+08	5E+08	[illegible]	30272	25732	p05804	Sorbin and SH3 domain-containing protein 2
KRT8 seq: 329 - 352 no mod	38324	3E+06	[illegible]	1E+08	1E+07	p19588	Keratin, type II cytoskeletal 8
SEPT9 seq: 30 - 45 + 2 Phospho (ST)	1E+08	1E+08	[illegible]	30272	9E+06	p05916	Septin-9
GSK3A pT19	1E+09	3E+09	[illegible]	3E+08	3E+08	p19669	Glycogen synthase kinase-3 alpha
DMAP1 seq: 401 - 449 + Phospho (ST)	38324	37149	[illegible]	1E+08	25732	p20544	DNA methyltransferase 1-associated protein 1
TSC2 seq: 979 - 988 + Phospho (ST)	1E+08	1E+08	[illegible]	6E+06	8E+06	p05449	Tuberin
STAU1 seq: 368 - 381 + Phospho (ST)	3E+06	2E+06	[illegible]	5E+08	1E+08	p06042	Double-stranded RNA-binding protein Staufen homolog 1
SHROOM2 pS413	1E+09	1E+09	[illegible]	30272	25732	p11035	Protein Shroom2
HSP90B1 seq: 745 - 794 no mod	38324	7E+07	[illegible]	30272	25732	p10753	Endoplasmin
MAP2K4 pS80	3E+08	3E+08	[illegible]	8E+06	25732	p10853	Dual specificity mitogen-activated protein kinase kinase 4
CLMN pS419	9E+07	2E+08	[illegible]	30272	25732	p04781	Calmin
GSE1 pT433	5E+07	6E+07	[illegible]	2E+07	2E+07	p15805	Genetic suppressor element 1
LMO7 pT1048	2E+07	1E+07	[illegible]	6E+08	5E+08	p13726	LIM domain only protein 7
ATXN1 pS88	2E+07	1E+07	[illegible]	1E+07	25732	p20147	Ataxin-1
PANK4 pS393	2E+08	1E+08	[illegible]	4E+07	25732	p16329	Pantothenate kinase 4
ARHGEF28 pS758	3E+07	1E+08	[illegible]	7E+07	1E+08	p03306	Rho guanine nucleotide exchange factor 28
HELZ pS1614 pS1620	2E+08	2E+08	[illegible]	30272	25732	p07700	Probable helicase with zinc finger domain
TDRD7 pS859	5E+07	5E+07	[illegible]	3E+08	1E+08	p18116	Tudor domain-containing protein 7
ZNF609 pS467	4E+08	4E+08	[illegible]	2E+08	1E+08	p02463	Zinc finger protein 609
CHMP3 seq: 184 - 216 + Oxidation (M); Phospho (ST)	3E+08	2E+08	[illegible]	2E+07	2E+07	p00744	Charged multivesicular body protein 3
FLNC pS2233	38324	37149	[illegible]	6E+08	7E+08	p11700	Filamin-C
IRF2BPL seq: 655 - 670 + Phospho (ST)	9E+07	8E+07	[illegible]	2E+07	2E+07	p07008	Interferon regulatory factor 2-binding protein-like
GFPT1 seq: 252 - 271 + Phospho (ST)	38324	37149	[illegible]	30272	6E+07	p15308	Glutamine--fructose-6-phosphate aminotransferase [isomerizing] 1
PXN seq: 126 - 147 + Phospho (ST)	2E+08	1E+08	[illegible]	2E+08	1E+08	p06353	Paxillin
WDR13 seq: 254 - 282 no mod	9E+06	1E+08	[illegible]	30272	1E+07	p17607	WD repeat-containing protein 13
HPS4 seq: 347 - 358 + Phospho (ST)	7E+07	8E+07	[illegible]	2E+07	25732	p16147	Hermansky-Pudlak syndrome 4 protein
RBMX2 seq: 312 - 321 + Phospho (ST)	5E+06	37149	[illegible]	30272	25732	p17121	RNA-binding motif protein, X-linked 2
ECH1 seq: 149 - 158 no mod	38324	6E+07	[illegible]	30272	25732	p00163	Delta(3,5)-Delta(2,4)-dienoyl-CoA isomerase, mitochondrial
FAM110C pS241	7E+07	6E+07	[illegible]	6E+06	25732	p17341	Protein FAM110C
LEO1 pS658	3E+07	6E+07	[illegible]	30272	25732	p00024	RNA polymerase-associated protein LEO1
URI1 seq: 371 - 386 + Phospho (ST)	3E+08	3E+08	[illegible]	4E+07	3E+07	p09060	Unconventional prefoldin RPB5 interactor 1
LIMA1 seq: 223 - 254 + Phospho (ST)	5E+08	1E+09	[illegible]	9E+08	9E+08	p13072	LIM domain and actin-binding protein 1
USP43 seq: 844 - 862 + Phospho (ST)	1E+07	2E+07	[illegible]	3E+07	3E+07	p03439	Ubiquitin carboxyl-terminal hydrolase 43
PRDX1 seq: 169 - 190 no mod	4E+08	4E+08	[illegible]	4E+07	4E+07	p14818	Peroxiredoxin-1
BAD seq: 97 - 109 + Phospho (ST)	2E+08	5E+08	[illegible]	30272	25732	p04074	Bcl2-associated agonist of cell death
RANBP3 seq: 124 - 138 + Phospho (ST)	2E+09	2E+09	[illegible]	1E+09	1E+09	p02098	Ran-binding protein 3
PHLDB2 pS212	38324	8E+06	[illegible]	2E+08	2E+08	p12855	Pleckstrin homology-like domain family B member 2
SLAIN2 seq: 239 - 271 + 2 Phospho (ST)	8E+07	37149	[illegible]	30272	25732	p03931	SLAIN motif-containing protein 2
FAM117A pS145	1E+09	9E+08	[illegible]	7E+07	9E+06	p06205	Protein FAM117A
GSK3A seq: 19 - 50 + Phospho (ST)	3E+09	3E+09	[illegible]	2E+08	3E+08	p02111	Glycogen synthase kinase-3 alpha
MLPH pS481 pS484	9E+07	1E+08	[illegible]	30272	7E+06	p01732	Melanophilin
seq: 46 - 71 + Gln->pyro-Glu (N-term Q); 2 Phospho (ST)	1E+09	9E+08	[illegible]	2E+08	2E+08	p08697	Repressor of RNA polymerase III transcription MAF1 homolog
UQCRC1 seq: 416 - 422 no mod	38324	2E+07	[illegible]	30272	25732	p05142	Cytochrome b-c1 complex subunit 1, mitochondrial
POLA2 pT127	38324	5E+06	[illegible]	2E+07	2E+07	p20427	DNA polymerase alpha subunit B
LIMA1 pS225	2E+08	6E+08	[illegible]	6E+08	4E+08	p13073	LIM domain and actin-binding protein 1
RTKN pS543	38324	37149	[illegible]	3E+08	4E+08	p03395	Rhotekin
FOXK1 seq: 236 - 259 + Phospho (ST)	38324	7E+07	[illegible]	1E+08	2E+08	p04859	Forkhead box protein K1
SIRT2 seq: 348 - 370 + Phospho (ST)	38324	37149	[illegible]	30272	2E+07	p17654	NAD-dependent protein deacetylase sirtuin-2
KIF4A seq: 799 - 806 + Phospho (ST)	9E+07	4E+08	[illegible]	2E+08	1E+08	p02839	Chromosome-associated kinesin KIF4A
TPD52 pS176	1E+08	8E+08	[illegible]	7E+07	4E+07	p09113	Tumor protein D52
UBA6 pS36	3E+07	2E+08	[illegible]	30272	25732	p09427	Ubiquitin-like modifier-activating enzyme 6
TUBB3 pM299 pM300	7E+08	6E+08	[illegible]	30272	25732	p09369	Tubulin beta-3 chain
ATN1 pS896	6E+07	9E+07	[illegible]	1E+07	25732	p02556	Atrophin-1
FAM83H pS647	7E+08	3E+08	[illegible]	30272	25732	p00972	Protein FAM83H
HID1 pT584	4E+08	4E+08	[illegible]	873747	25732	p06712	Protein HID1
PCBP2 seq: 246 - 276 + Phospho (ST)	2E+09	4E+09	[illegible]	6E+09	25732	p11640	Poly(rC)-binding protein 2
LMNB1 seq: 300 - 312 no mod	38324	4E+07	[illegible]	1E+07	25732	p14334	Lamin-B1
UBXN2B pS56	8E+07	6E+07	[illegible]	30272	25732	p19295	UBX domain-containing protein 2B
MAP4 seq: 937 - 953 + Phospho (ST)	7E+08	2E+09	[illegible]	2E+09	9E+08	p05330	Microtubule-associated protein 4
FKBP15 seq: 306 - 334 + Phospho (ST)	5E+07	5E+07	[illegible]	30272	2E+07	p18311	FK506-binding protein 15
KIAA1671 pS1757	38324	4E+07	[illegible]	2E+08	1E+08	p06941	Uncharacterized protein KIAA1671
RAB11FIP1 seq: 498 - 520 + Phospho (ST)	5E+07	2E+08	[illegible]	30272	9E+06	p08586	Rab11 family-interacting protein 1
SPTBN1 seq: 1070 - 1080 no mod	7E+06	3E+07	[illegible]	30272	25732	p18643	Spectrin beta chain, non-erythrocytic 1
CAMK2D seq: 313 - 328 + 2 Phospho (ST)	9E+07	2E+08	[illegible]	1E+08	25732	p09632	Calcium/calmodulin-dependent protein kinase type II subunit delta
ARL3 pS5	38324	977220	[illegible]	30272	25732	p15699	ADP-ribosylation factor-like protein 3
KCNIP4 pS150	38324	977220	[illegible]	30272	25732	p15645	Kv channel-interacting protein 4
TRIL seq: 255 - 261 + Phospho (ST)	38324	977220	[illegible]	30272	25732	p11742	TLR4 interactor with leucine rich repeats
AHNAK pS1123	1E+07	4E+07	[illegible]	2E+07	1E+07	p19999	Neuroblast differentiation-associated protein AHNAK
MCF2L seq: 959 - 978 + Phospho (ST)	2E+08	3E+08	[illegible]	5E+08	5E+08	p20271	Guanine nucleotide exchange factor DBS
RCC1 seq: 207 - 214 no mod	2E+07	37149	[illegible]	30272	25732	p01019	Regulator of chromosome condensation
MTMR2 pS58	1E+08	8E+07	[illegible]	1E+08	9E+07	p06209	Myotubularin-related protein 2
DLG5 seq: 1664 - 1678 + Phospho (ST)	2E+08	2E+08	[illegible]	2E+07	4E+06	p07109	Disks large homolog 5
NDRG1 seq: 364 - 388 + Phospho (ST)	4E+09	4E+09	[illegible]	5E+08	3E+08	p05550	Protein NDRG1
NCL pS563	9E+07	3E+08	[illegible]	9E+08	9E+08	p11958	Nucleolin
PACS1 seq: 754 - 790 + Phospho (ST)	1E+09	8E+08	[illegible]	4E+08	3E+08	p01463	Phosphofurin acidic cluster sorting protein 1
PARN pS557	1E+07	4E+07	[illegible]	30272	25732	p09304	Poly(A)-specific ribonuclease PARN
PAK1 seq: 422 - 438 + Phospho (ST)	7E+06	8E+06	[illegible]	30272	1E+07	p03497	Serine/threonine-protein kinase PAK 1
TBX3 seq: 431 - 447 + Phospho (ST)	4E+09	4E+09	[illegible]	30272	25732	p19552	T-box transcription factor TBX3
PCBP1 seq: 244 - 268 + Phospho (ST)	2E+09	3E+09	[illegible]	7E+08	7E+08	p08012	Poly(rC)-binding protein 1
NDRG1 seq: 364 - 388 + Phospho (ST)	1E+09	1E+09	[illegible]	3E+08	1E+08	p05549	Protein NDRG1
seq: 401 - 427 + Gln->pyro-Glu (N-term Q); Phospho (ST)	2E+08	9E+07	[illegible]	30272	25732	p07848	NGFI-A-binding protein 1
DBNL seq: 267 - 280 + Oxidation (M); 2 Phospho (ST)	6E+08	5E+08	[illegible]	30272	25732	p20072	Drebrin-like protein
CTTN pS261	7E+08	7E+08	[illegible]	1E+09	4E+08	p10979	Src substrate cortactin
RNF219 pS719	1E+08	2E+08	[illegible]	4E+07	7E+07	p13784	RING finger protein 219
CAMK2D seq: 319 - 344 + Phospho (ST)	2E+08	3E+08	[illegible]	9E+07	9E+07	p05154	Calcium/calmodulin-dependent protein kinase type II subunit delta
MDH2 seq: 280 - 297 no mod	38324	6E+07	[illegible]	6E+06	25732	p04260	Malate dehydrogenase, mitochondrial
EIF3B seq: 117 - 134 + Phospho (ST)	2E+08	3E+08	[illegible]	2E+07	2E+07	p06074	Eukaryotic translation initiation factor 3 subunit B
UBE4B seq: 76 - 102 + Phospho (ST)	7E+08	5E+08	[illegible]	30272	8E+07	p04166	Ubiquitin conjugation factor E4 B
DCLK1 seq: 162 - 177 + Phospho (ST)	3E+08	2E+08	[illegible]	2E+07	8E+06	p19174	Serine/threonine-protein kinase DCLK1
ULK1 pS477 pS479	4E+08	3E+08	[illegible]	8E+07	1E+08	p19530	Serine/threonine-protein kinase ULK1
TBC1D10B pS22	38324	7E+08	[illegible]	3E+08	3E+08	p14823	TBC1 domain family member 10B
TBC1D4 seq: 339 - 355 + Phospho (ST)	1E+09	1E+09	[illegible]	30272	25732	p14883	TBC1 domain family member 4
EEF1D seq: 25 - 38 no mod	1E+08	5E+07	[illegible]	6E+07	25732	p16369	Elongation factor 1-delta
BAIAP2L1 seq: 292 - 313 + Phospho (Y)	9E+06	2E+07	[illegible]	1E+08	2E+08	p19082	Brain-specific angiogenesis inhibitor 1-associated protein 2-like protein 1
CABLES1 seq: 413 - 426 + Phospho (ST)	1E+08	1E+08	[illegible]	30272	9E+07	p06993	CDK5 and ABL1 enzyme substrate 1
SYNRG seq: 908 - 928 + Phospho (ST)	1E+08	2E+08	[illegible]	5E+07	5E+07	p10711	Synergin gamma
MAPT seq: 703 - 723 + 3 Phospho (ST)	1E+09	9E+08	[illegible]	30272	25732	p02960	Microtubule-associated protein tau
seq: 449 - 465 + Gln->pyro-Glu (N-term Q); Phospho (ST)	38324	37149	[illegible]	2E+07	2E+07	p07986	MAP kinase-interacting serine/threonine-protein kinase 2
GPATCH8 seq: 1105 - 1122 + Phospho (ST)	6E+07	2E+08	[illegible]	7E+08	5E+08	p12711	G patch domain-containing protein 8
COBL seq: 862 - 874 + Phospho (ST)	2E+07	6E+07	[illegible]	30272	2E+06	p06482	Protein cordon-bleu
PARD3 seq: 713 - 731 + Phospho (ST)	9E+07	2E+08	[illegible]	8E+07	9E+07	p07253	Partitioning defective 3 homolog
FAM110A pS78	4E+07	4E+07	[illegible]	1E+08	1E+08	p08128	Protein FAM110A
CBY1 seq: 18 - 29 + Phospho (ST)	8E+07	6E+07	[illegible]	4E+07	4E+07	p06258	Protein chibby homolog 1
GSK3B seq: 7 - 27 + Phospho (ST)	2E+09	2E+09	[illegible]	7E+08	5E+08	p02021	Glycogen synthase kinase-3 beta
MISP pS394 pS397	4E+08	2E+08	[illegible]	4E+08	3E+08	p07627	Mitotic interactor and substrate of PLK1
CAMK2G pS311	5E+07	6E+07	[illegible]	8E+06	1E+07	p16310	Calcium/calmodulin-dependent protein kinase type II subunit gamma
CTDSPL2 seq: 77 - 88 + Phospho (ST)	2E+07	2E+07	[illegible]	9E+07	5E+07	p18763	CTD small phosphatase-like protein 2
RAB11FIP1 seq: 490 - 520 + 2 Phospho (ST)	38324	7E+07	[illegible]	30272	25732	p18192	Rab11 family-interacting protein 1
TBC1D5 pS539	3E+08	2E+08	[illegible]	2E+08	2E+08	p09532	TBC1 domain family member 5
PCBP1 seq: 244 - 268 + Phospho (ST)	2E+09	3E+09	[illegible]	8E+08	8E+08	p08013	Poly(rC)-binding protein 1
GRB10 pS104	9E+08	5E+08	[illegible]	1E+08	7E+07	p05483	Growth factor receptor-bound protein 10
FAM83B pS764	1E+07	5E+07	[illegible]	2E+07	1E+07	p16977	Protein FAM83B
TBC1D5 pS730	3E+08	2E+08	[illegible]	1E+07	5E+06	p15290	TBC1 domain family member 5
FUBP1 seq: 219 - 236 no mod	1E+07	5E+07	[illegible]	30272	25732	p09822	Far upstream element-binding protein 1
UNK pS411	1E+08	3E+08	[illegible]	1E+08	1E+08	p12747	RING finger protein unkempt homolog
EIF3A seq: 877 - 886 + Phospho (ST)	1E+08	2E+08	[illegible]	5E+07	7E+07	p07178	Eukaryotic translation initiation factor 3 subunit A
ANXA2 seq: 180 - 196 no mod	5E+06	37149	[illegible]	7E+07	7E+07	p20860	Annexin A2
HNRNPUL1 seq: 376 - 386 no mod	2E+07	4E+07	[illegible]	30272	25732	p09733	Heterogeneous nuclear ribonucleoprotein U-like protein 1
FOXK1 seq: 236 - 259 + 2 Phospho (ST)	5E+07	1E+08	[illegible]	6E+07	3E+07	p04861	Forkhead box protein K1
LARP4B pS498	9E+08	1E+09	[illegible]	5E+08	4E+08	p12840	La-related protein 4B
ZNF217 seq: 643 - 664 + Phospho (ST)	7E+07	2E+08	[illegible]	30272	3E+06	p02675	Zinc finger protein 217
PKP3 seq: 195 - 211 + Phospho (ST)	8E+09	6E+09	[illegible]	2E+09	2E+09	p00100	Plakophilin-3
REPS2 pS254	1E+09	2E+09	[illegible]	2E+07	8E+06	p14681	RalBP1-associated Eps domain-containing protein 2
SYNRG pS919	6E+07	1E+08	[illegible]	5E+07	4E+07	p10710	Synergin gamma
TJP3 seq: 358 - 375 + Phospho (ST)	2E+08	2E+08	[illegible]	2E+07	25732	p17105	Tight junction protein ZO-3
ITGB4 seq: 1481 - 1490 + Phospho (ST)	38324	2E+07	[illegible]	7E+07	5E+07	p01146	Integrin beta-4
BCLAF1 seq: 492 - 504 + Phospho (ST)	3E+07	3E+07	[illegible]	30272	25732	p13267	Bcl-2-associated transcription factor 1
GSG2 seq: 43 - 78 + Phospho (ST)	38324	37149	[illegible]	6E+07	5E+07	p16783	Serine/threonine-protein kinase haspin
LDB1 seq: 300 - 321 + Phospho (ST)	3E+08	3E+08	[illegible]	30272	25732	p12857	LIM domain-binding protein 1
BAP1 seq: 513 - 529 + Phospho (ST)	6E+08	8E+08	[illegible]	4E+08	6E+08	p06307	Ubiquitin carboxyl-terminal hydrolase BAP1
PNISR seq: 286 - 307 + Phospho (ST)	9E+07	1E+08	[illegible]	1E+07	2E+07	p05419	Arginine/serine-rich protein PNISR
PAWR seq: 226 - 240 + Phospho (ST)	1E+08	2E+08	[illegible]	2E+07	2E+07	p00284	PRKC apoptosis WT1 regulator protein
TNKS1BP1 pS494	5E+08	3E+08	[illegible]	30272	25732	p12041	182 kDa tankyrase-1-binding protein
SHANK2 pS725	8E+08	7E+08	[illegible]	5E+07	3E+07	p09127	SH3 and multiple ankyrin repeat domains protein 2
MYH11 seq: 664 - 669 + Phospho (ST)	2E+08	2E+08	[illegible]	2E+06	25732	p11219	Myosin-11
CEP170P1 seq: 230 - 251 + 2 Phospho (ST)	8E+07	4E+07	[illegible]	6E+07	6E+07	p03762	Cep170-like protein
CAD pS1859	2E+09	1E+09	[illegible]	4E+09	2E+06	p14205	CAD protein
ERBB2 pS1100	3E+08	5E+08	[illegible]	30272	25732	p15660	Receptor tyrosine-protein kinase erbB-2
DENND4B seq: 506 - 511 + Phospho (ST)	5E+07	1E+08	[illegible]	30272	25732	p12955	DENN domain-containing protein 4B
TBC1D4 pS666	7E+08	7E+08	[illegible]	3E+08	3E+08	p19807	TBC1 domain family member 4
BRD1 pS486	7E+08	7E+08	[illegible]	3E+08	3E+08	p10687	Bromodomain-containing protein 1
SET seq: 133 - 142 no mod	8E+07	2E+08	[illegible]	1E+08	1E+08	p01585	Protein SET
TBC1D1 seq: 625 - 636 + Phospho (ST)	7E+08	8E+08	[illegible]	2E+06	3E+06	p00320	TBC1 domain family member 1
LUZP1 pS1023	38324	37149	[illegible]	2E+08	1E+08	p01509	Leucine zipper protein 1
AXL seq: 866 - 882 + Phospho (Y)	38324	37149	[illegible]	30272	25732	p00021	Tyrosine-protein kinase receptor UFO
ECH1 seq: 186 - 196 no mod	5E+07	1E+08	[illegible]	30272	6E+07	p00434	Delta(3,5)-Delta(2,4)-dienoyl-CoA isomerase, mitochondrial
seq: 244 - 268 + Gln->pyro-Glu (N-term Q); Phospho (ST)	5E+09	7E+09	[illegible]	3E+09	3E+09	p08015	Poly(rC)-binding protein 1
SH3BP2 pS278	9E+07	1E+08	[illegible]	2E+07	25732	p07050	SH3 domain-binding protein 2
TRIM28 seq: 378 - 390 no mod	38324	8E+06	[illegible]	30272	25732	p10059	Transcription intermediary factor 1-beta
VIM pS55	1E+07	8E+07	[illegible]	6E+09	6E+09	p04925	Vimentin
BCL7C pS126	8E+08	7E+08	[illegible]	2E+08	1E+08	p15103	B-cell CLL/lymphoma 7 protein family member C
BAIAP2 pS492	1E+08	1E+08	[illegible]	9E+07	8E+07	p07969	Brain-specific angiogenesis inhibitor 1-associated protein 2
DHRS2 seq: 141 - 147 no mod	38324	4E+07	[illegible]	30272	25732	p13979	Dehydrogenase/reductase SDR family member 2, mitochondrial
ASS1 seq: 349 - 355 no mod	38324	4E+07	[illegible]	30272	25732	p00912	Argininosuccinate synthase
CACNG6 seq: 166 - 172 no mod	38324	7E+07	[illegible]	30272	25732	p16330	Voltage-dependent calcium channel gamma-6 subunit
GANAB seq: 858 - 863 no mod	38324	7E+07	[illegible]	30272	25732	p08742	Neutral alpha-glucosidase AB
BAIAP2L1 seq: 420 - 452 + Oxidation (M); Phospho (ST)	4E+06	37149	[illegible]	7E+08	5E+08	p05454	Brain-specific angiogenesis inhibitor 1-associated protein 2-like protein 1
ERBB2IP pS1179	4E+08	7E+08	[illegible]	1E+09	1E+09	p01453	Protein LAP2
RPS6KA3 seq: 365 - 383 + Phospho (ST)	1E+09	2E+09	[illegible]	30272	25732	p02382	Ribosomal protein S6 kinase alpha-3
BAIAP2L1 pT257 pS261	7E+07	1E+08	[illegible]	2E+09	2E+09	p13957	Brain-specific angiogenesis inhibitor 1-associated protein 2-like protein 1
PRKD1 seq: 247 - 261 + Phospho (ST)	1E+08	2E+08	[illegible]	3E+07	4E+07	p04765	Serine/threonine-protein kinase D1
A seq: 13 - 53 + Gln->pyro-Glu (N-term Q); Phospho (ST)	38324	37149	[illegible]	1E+08	2E+08	p07871	Alpha-taxilin
RAPGEF6 seq: 1068 - 1087 + Phospho (ST)	2E+08	1E+08	[illegible]	1E+08	9E+07	p05002	Rap guanine nucleotide exchange factor 6
HDGFRP2 pS454	3E+09	3E+09	[illegible]	5E+08	6E+08	p12619	Hepatoma-derived growth factor-related protein 2
UBE2O pM398 pS401	2E+09	1E+09	[illegible]	1E+08	1E+08	p13939	(E3-independent) E2 ubiquitin-conjugating enzyme
FAM13B pS760	7E+07	1E+08	[illegible]	3E+07	1E+07	p19594	Protein FAM13B
ARHGEF17 pS420	1E+08	2E+08	[illegible]	2E+08	1E+08	p04430	Rho guanine nucleotide exchange factor 17
URI1 seq: 370 - 386 + Phospho (ST)	2E+09	2E+09	[illegible]	8E+08	8E+08	p12832	Unconventional prefoldin RPB5 interactor 1
FAM53B pS118	7E+07	1E+08	[illegible]	30272	1E+07	p05059	Protein FAM53B
ERRFI1 pS302	3E+07	37149	[illegible]	3E+08	2E+08	p06411	ERBB receptor feedback inhibitor 1
HDGFRP2 pS664	1E+07	4E+07	[illegible]	2E+07	3E+07	p17252	Hepatoma-derived growth factor-related protein 2
HEY2 pS39	9E+07	9E+07	[illegible]	8E+08	7E+08	p11159	Hairy/enhancer-of-split related with YRPW motif protein 2
MAP7D1 pS313	38324	6E+07	[illegible]	4E+06	25732	p06166	MAP7 domain-containing protein 1
PIGK pS142	38324	2E+08	[illegible]	30272	25732	p13867	GPI-anchor transamidase
RBMX pS208	5E+07	2E+08	[illegible]	2E+08	1E+08	p18073	RNA-binding motif protein, X chromosome
SIPA1L1 seq: 205 - 227 + 2 Phospho (ST)	7E+08	5E+08	[illegible]	2E+08	5E+08	p16909	Signal-induced proliferation-associated 1-like protein 1
RUSC2 pS536	38324	1E+07	[illegible]	2E+08	2E+08	p10817	Iporin
KLC2 seq: 507 - 518 + Phospho (ST)	3E+07	5E+07	[illegible]	2E+06	2E+06	p03743	Kinesin light chain 2
EEF2K seq: 69 - 82 + Phospho (ST); Phospho (Y)	1E+09	1E+09	[illegible]	9E+07	1E+08	p00066	Eukaryotic elongation factor 2 kinase
HSPB8 pS24	2E+08	2E+08	[illegible]	2E+06	25732	p18407	Heat shock protein beta-8
TPD52L1 pS174	5E+07	6E+07	[illegible]	30272	25732	p01478	Tumor protein D53
FOXD2 pS96	8E+07	6E+07	[illegible]	30272	25732	p16360	Forkhead box protein D2
BAIAP2L1 seq: 239 - 266 + Oxidation (M); 4 Phospho (ST)	38324	37149	[illegible]	30272	9E+07	p13949	Brain-specific angiogenesis inhibitor 1-associated protein 2-like protein 1
RAB11FIP5 seq: 354 - 374 + Phospho (ST)	4E+07	2E+08	[illegible]	3E+07	6E+07	p14654	Rab11 family-interacting protein 5
PATL1 pT194	6E+08	5E+08	[illegible]	2E+08	2E+08	p19214	Protein PAT1 homolog 1
TPD52L2 seq: 128 - 139 no mod	2E+07	2E+07	[illegible]	4E+07	4E+07	p12478	Tumor protein D54
KIAA1671 pS1506	2E+07	2E+07	[illegible]	2E+07	3E+07	p05709	Uncharacterized protein KIAA1671
DDX3X seq: 82 - 88 no mod	4E+07	1E+08	[illegible]	6E+06	7E+06	p03911	ATP-dependent RNA helicase DDX3X
CRIP2 seq: 113 - 128 + Phospho (ST)	9E+08	8E+08	[illegible]	1E+07	6E+06	p19460	Cysteine-rich protein 2
ATXN2 seq: 695 - 738 + Oxidation (M); Phospho (ST)	1E+09	8E+08	[illegible]	2E+08	1E+08	p08185	Ataxin-2
EEF2K pS72 pS74	1E+09	1E+09	[illegible]	9E+07	1E+08	p00069	Eukaryotic elongation factor 2 kinase
PLCG1 pS1263	38324	37149	[illegible]	8E+07	6E+07	p00151	1-phosphatidylinositol 4,5-bisphosphate phosphodiesterase gamma-1
FLYWCH1 pS21	6E+07	2E+08	[illegible]	4E+07	25732	p20593	FLYWCH-type zinc finger-containing protein 1
PLEKHA6 pS472	2E+08	2E+08	[illegible]	2E+06	25732	p13487	Pleckstrin homology domain-containing family A member 6
MYO9B pS1331	5E+07	6E+07	[illegible]	30272	25732	p10458	Unconventional myosin-IXb
CAMK2D seq: 319 - 344 + 2 Phospho (ST)	1E+08	1E+08	[illegible]	2E+07	25732	p05155	Calcium/calmodulin-dependent protein kinase type II subunit delta
ERBB2 seq: 1054 - 1096 + Phospho (ST)	38324	6E+07	[illegible]	30272	25732	p05757	Receptor tyrosine-protein kinase erbB-2
ADNP seq: 1057 - 1096 + Oxidation (M); Phospho (ST)	9E+07	1E+08	[illegible]	30272	25732	p10725	Activity-dependent neuroprotector homeobox protein
KRT80 seq: 45 - 57 + Phospho (ST)	38324	7E+07	[illegible]	2E+08	2E+08	p04956	Keratin, type II cytoskeletal 80
ACO2 seq: 234 - 245 no mod	8E+07	1E+08	[illegible]	6E+07	8E+07	p10552	Aconitate hydratase, mitochondrial
HNRNPA2B1 seq: 47 - 60 no mod	8E+07	2E+08	[illegible]	7E+07	25732	p10576	Heterogeneous nuclear ribonucleoproteins A2/B1

TBCA seq: 52 - 61 no mod	[illegible]	p12698	Tubulin-specific chaperone A
PPP2R1A seq: 332 - 342 no mod	[illegible]	p17375	Serine/threonine-protein phosphatase 2A 65 kDa regulatory subunit A alpha isoform
ERBB2IP pS1179	[illegible]	p01452	Protein LAP2
NCL pS563	[illegible]	p11957	Nucleolin
SH3PXD2A pS644	[illegible]	p15177	SH3 and PX domain-containing protein 2A
VCP pM678	[illegible]	p09852	Transitional endoplasmic reticulum ATPase
DSG2 seq: 698 - 717 + Phospho (ST)	[illegible]	p15159	Desmoglein-2
[illegible] seq: 88 - 103 + Gln->pyro-Glu (N-term Q); Phospho (ST)	[illegible]	p08431	Neuron navigator 1
LIMA1 seq: 223 - 250 + Phospho (ST)	[illegible]	p13074	LIM domain and actin-binding protein 1
SSFA2 pM1038 pS1040	[illegible]	p09945	Sperm-specific antigen 2
ERBB2IP seq: 1156 - 1166 + Phospho (ST)	[illegible]	p02493	Protein LAP2
PKP3 pS238	[illegible]	p20662	Plakophilin-3
LMNA seq: 472 - 482 no mod	[illegible]	p08217	Prelamin-A/C
MARK3 pS469 pM471	[illegible]	p15910	MAP/microtubule affinity-regulating kinase 3
AKT1S1 pS183	[illegible]	p05092	Proline-rich AKT1 substrate 1
NAV1 seq: 646 - 665 + Phospho (ST)	[illegible]	p12465	Neuron navigator 1
DTL seq: 671 - 688 + Phospho (ST)	[illegible]	p13447	Denticleless protein homolog
TNS1 seq: 1263 - 1274 + Phospho (ST)	[illegible]	p01742	Tensin-1
SPECC1 pS218	[illegible]	p02592	Cytospin-B
PDCD4 pS457	[illegible]	p07410	Programmed cell death protein 4
USP10 seq: 74 - 98 + Phospho (ST)	[illegible]	p02309	Ubiquitin carboxyl-terminal hydrolase 10
SPIRE2 seq: 440 - 450 + Phospho (ST)	[illegible]	p05856	Protein spire homolog 2
DDX3X seq: 277 - 287 no mod	[illegible]	p17530	ATP-dependent RNA helicase DDX3X
BAIAP2L1 seq: 329 - 341 + Oxidation (M); Phospho (ST)	[illegible]	p03234	Brain-specific angiogenesis inhibitor 1-associated protein 2-like protein 1
RICTOR seq: 1133 - 1155 + Phospho (ST)	[illegible]	p02524	Rapamycin-insensitive companion of mTOR
SHC1 pS139	[illegible]	p14796	SHC-transforming protein 1
PADI2 seq: 384 - 395 no mod	[illegible]	p17461	Protein-arginine deiminase type-2
TEX2 pS266	[illegible]	p03067	Testis-expressed sequence 2 protein
RTKN seq: 518 - 531 + Phospho (ST)	[illegible]	p02841	Rhotekin
PFKL pS775	[illegible]	p02550	ATP-dependent 6-phosphofructokinase, liver type
SCRIB pM1292 pS1309	[illegible]	p10272	Protein scribble homolog
PHF8 seq: 1019 - 1033 + Phospho (ST)	[illegible]	p06834	Histone lysine demethylase PHF8
LMO7 pS1539	[illegible]	p08111	LIM domain only protein 7
FOXA1 pS307	[illegible]	p13328	Hepatocyte nuclear factor 3-alpha
OSBP pS351	[illegible]	p16211	Oxysterol-binding protein 1
SVIL seq: 960 - 981 + Phospho (ST)	[illegible]	p18285	Supervillin
PPHLN1 pS172	[illegible]	p03289	Periphilin-1
HSPB8 pS24	[illegible]	p07526	Heat shock protein beta-8
CASKIN2 seq: 798 - 831 + Phospho (ST)	[illegible]	p05571	Caskin-2
LMNB2 pS420	[illegible]	p19303	Lamin-B2
ADD3 pS42	[illegible]	p09361	Gamma-adducin
ATXN2 pS758	[illegible]	p08179	Ataxin-2
LARP1 seq: 1054 - 1083 + Phospho (ST)	[illegible]	p19028	La-related protein 1
PATL1 seq: 177 - 188 + 2 Phospho (ST)	[illegible]	p03444	Protein PAT1 homolog 1
NCBP3 pM410 pS415	[illegible]	p10061	Nuclear cap-binding protein subunit 3
PSMD6 seq: 2 - 18 no mod	[illegible]	p08890	26S proteasome non-ATPase regulatory subunit 6
GTF2I seq: 807 - 826 + Oxidation (M); Phospho (ST)	[illegible]	p12801	General transcription factor II-I
GPBP1 seq: 369 - 387 + Phospho (ST)	[illegible]	p03598	Vasculin
ZHX2 pS719	[illegible]	p13372	Zinc fingers and homeoboxes protein 2
PATL1 seq: 176 - 188 + 2 Phospho (ST)	[illegible]	p06552	Protein PAT1 homolog 1
NOP2 seq: 722 - 743 + Phospho (ST)	[illegible]	p15119	Probable 28S rRNA (cytosine(4447)-C(5))-methyltransferase
MLPH pS574	[illegible]	p03207	Melanophilin
NDRG3 seq: 327 - 345 + 2 Phospho (ST)	[illegible]	p03985	Protein NDRG3
PHLDB2 seq: 382 - 399 + Phospho (ST)	[illegible]	p09628	Pleckstrin homology-like domain family B member 2
STUB1 pS19	[illegible]	p11836	E3 ubiquitin-protein ligase CHIP
ANXA1 seq: 10 - 26 + Phospho (Y)	[illegible]	p08788	Annexin A1
UIMC1 seq: 167 - 179 no mod	[illegible]	p15270	BRCA1-A complex subunit RAP80
NAV3 pS1245	[illegible]	p00194	Neuron navigator 3
STIM1 pS512	[illegible]	p10566	Stromal interaction molecule 1
FOXK2 seq: 369 - 395 + Phospho (ST)	[illegible]	p06304	Forkhead box protein K2
PHF8 pS880	[illegible]	p09168	Histone lysine demethylase PHF8
LMNA seq: 400 - 417 + Phospho (ST)	[illegible]	p15354	Prelamin-A/C
SPEN pS1287	[illegible]	p11267	Msx2-interacting protein
SRRM2 pM2419 pS2426	[illegible]	p09845	Serine/arginine repetitive matrix protein 2
FRS2 seq: 325 - 343 no mod	[illegible]	p10485	Fibroblast growth factor receptor substrate 2
SRSF11 seq: 336 - 343 + Phospho (ST)	[illegible]	p04072	Serine/arginine-rich splicing factor 11
RBBP6 seq: 770 - 777 + Phospho (ST)	[illegible]	p04022	E3 ubiquitin-protein ligase RBBP6
PLEKHA6 pS777	[illegible]	p02647	Pleckstrin homology domain-containing family A member 6
GGCT seq: 89 - 101 no mod	[illegible]	p04842	Gamma-glutamylcyclotransferase
TUBA1C seq: 281 - 304 no mod	[illegible]	p19124	Tubulin alpha-1C chain
N4BP2 seq: 1208 - 1229 + Phospho (ST)	[illegible]	p19160	NEDD4-binding protein 2
SRRM2 seq: 889 - 900 + Phospho (ST)	[illegible]	p14652	Serine/arginine repetitive matrix protein 2
PI4K2A pS462	[illegible]	p03718	Phosphatidylinositol 4-kinase type 2-alpha
CSDE1 pS123	[illegible]	p04735	Cold shock domain-containing protein E1
TJP2 pS130	[illegible]	p00917	Tight junction protein ZO-2
RTCB seq: 284 - 291 no mod	[illegible]	p18684	tRNA-splicing ligase RtcB homolog
KRT18 seq: 383 - 407 + Phospho (ST)	[illegible]	p11432	Keratin, type I cytoskeletal 18
UBAP2L seq: 601 - 618 + Phospho (ST)	[illegible]	p06402	Ubiquitin-associated protein 2-like
ANXA2 seq: 221 - 227 no mod	[illegible]	p03304	Annexin A2
KRI1 pS628	[illegible]	p08341	Protein KRI1 homolog
EEF2K pS72 pS74	[illegible]	p00070	Eukaryotic elongation factor 2 kinase
PTPN12 seq: 564 - 592 + 2 Phospho (ST)	[illegible]	p12458	Tyrosine-protein phosphatase non-receptor type 12
PRPF40B pS764	[illegible]	p15206	Pre-mRNA-processing factor 40 homolog B
PARVB pT36	[illegible]	p11764	Beta-parvin
CDT1 pS31	[illegible]	p02327	DNA replication factor Cdt1
PRKCH pS675	[illegible]	p09623	Protein kinase C eta type
TPD52L1 pS149	[illegible]	p05920	Tumor protein D53
MKL2 pS543	[illegible]	p09848	MKL/myocardin-like protein 2
TFPT pS180	[illegible]	p06512	TCF3 fusion partner
SEPT9 pS22	[illegible]	p11812	Septin-9
SYNRG pS1075	[illegible]	p05045	Synergin gamma
PHLDA2 pS42	[illegible]	p10761	Pleckstrin homology-like domain family A member 2
PDCD4 seq: 70 - 86 + Phospho (ST)	[illegible]	p18283	Programmed cell death protein 4
MARK3 seq: 505 - 513 + Phospho (ST)	[illegible]	p06988	MAP/microtubule affinity-regulating kinase 3
C6orf106 pS215	[illegible]	p12442	Uncharacterized protein C6orf106
PABPC1 seq: 279 - 284 no mod	[illegible]	p13251	Polyadenylate-binding protein 1
BLVRB seq: 125 - 134 no mod	[illegible]	p11030	Flavin reductase (NADPH)
CBFA2T2 pS409	[illegible]	p08564	Protein CBFA2T2
HIST1H1B seq: 37 - 49 no mod	[illegible]	p13373	Histone H1.5
CORO1C seq: 392 - 398 no mod	[illegible]	p14777	Coronin-1C
EIF4G1 seq: 1184 - 1193 + Phospho (ST)	[illegible]	p06678	Eukaryotic translation initiation factor 4 gamma 1
CHAMP1 pS386	[illegible]	p04515	Chromosome alignment-maintaining phosphoprotein 1
CCDC43 seq: 129 - 162 + Phospho (ST)	[illegible]	p21097	Coiled-coil domain-containing protein 43
RAB11FIP1 seq: 433 - 444 + Phospho (ST)	[illegible]	p06605	Rab11 family-interacting protein 1
ZC3HC1 pS359 pS370	[illegible]	p03204	Nuclear-interacting partner of ALK
MAP7 pS209	[illegible]	p10628	Ensconsin
GREB1L seq: 982 - 987 no mod	[illegible]	p11737	GREB1-like protein
SLC9A3R1 seq: 35 - 40 no mod	[illegible]	p11708	Na(+)/H(+) exchange regulatory cofactor NHE-RF1
TCF20 pS1361	[illegible]	p13578	Transcription factor 20
CBY1 seq: 17 - 29 + Phospho (ST)	[illegible]	p12607	Protein chibby homolog 1
EIF4EBP1 seq: 64 - 99 + 2 Phospho (ST)	[illegible]	p09101	Eukaryotic translation initiation factor 4E-binding protein 1
ZBTB7A seq: 503 - 531 + Phospho (ST)	[illegible]	p00903	Zinc finger and BTB domain-containing protein 7A
CLSPN seq: 718 - 729 + Phospho (ST)	[illegible]	p04996	Claspin
PPFIBP1 pS579	[illegible]	p09123	Liprin-beta-1
HIST1H1B seq: 68 - 82 no mod	[illegible]	p20336	Histone H1.5
SDCBP seq: 186 - 191 + Phospho (ST)	[illegible]	p13593	Syntenin-1
PRR11 seq: 344 - 360 + Phospho (ST)	[illegible]	p04323	Proline-rich protein 11
EPS15L1 seq: 560 - 591 + Phospho (ST)	[illegible]	p05251	Epidermal growth factor receptor substrate 15-like 1
CCDC88C pS1601	[illegible]	p05990	Protein Daple
ANKIB1 seq: 877 - 895 + Phospho (ST)	[illegible]	p18872	Ankyrin repeat and IBR domain-containing protein 1
RAP1GAP2 pS564	[illegible]	p06677	Rap1 GTPase-activating protein 2
MTFR1 seq: 286 - 296 + Phospho (ST)	[illegible]	p00134	Mitochondrial fission regulator 1
PHKA2 seq: 698 - 705 no mod	[illegible]	p18742	Phosphorylase b kinase regulatory subunit alpha, liver isoform
CTTNBP2NL seq: 437 - 458 + Phospho (Y)	[illegible]	p11390	CTTNBP2 N-terminal-like protein
UBA1 pS816 pS820	[illegible]	p14201	Ubiquitin-like modifier-activating enzyme 1
DSP pS22	[illegible]	p10066	Desmoplakin
RPS6KB1 pS441	[illegible]	p07445	Ribosomal protein S6 kinase beta-1
NGDN seq: 200 - 207 + Phospho (ST)	[illegible]	p20149	Neuroguidin
ANXA7 seq: 390 - 397 no mod	[illegible]	p18575	Annexin A7
BCAR3 pS363	[illegible]	p11092	Breast cancer anti-estrogen resistance protein 3
ACOT1 seq: 307 - 313 no mod	[illegible]	p05899	Acyl-coenzyme A thioesterase 1
ACAD10 seq: 166 - 172 no mod	[illegible]	p05887	Acyl-CoA dehydrogenase family member 10
RBL2 seq: 1103 - 1116 + Phospho (ST)	[illegible]	p15877	Retinoblastoma-like protein 2
CGNL1 seq: 295 - 311 + Phospho (ST)	[illegible]	p06569	Cingulin-like protein 1
BCAR1 pS139	[illegible]	p19772	Breast cancer anti-estrogen resistance protein 1
CCDC120 seq: 278 - 289 + Phospho (ST)	[illegible]	p06999	Coiled-coil domain-containing protein 120
SMG9 pS451	[illegible]	p20603	Protein SMG9
RNPEP seq: 177 - 188 no mod	[illegible]	p20777	Aminopeptidase B
TSC2 seq: 1439 - 1451 + Phospho (ST)	[illegible]	p15385	Tuberin
SSH3 seq: 599 - 615 + Phospho (ST)	[illegible]	p04077	Protein phosphatase Slingshot homolog 3
CD81 seq: 125 - 144 + Gln->pyro-Glu (N-term Q)	[illegible]	p08665	CD81 antigen
HSP90B1 seq: 53 - 62 no mod	[illegible]	p15754	Endoplasmin
ANXA4 seq: 63 - 69 no mod	[illegible]	p18601	Annexin A4
ANXA5 seq: 64 - 70 no mod	[illegible]	p18590	Annexin A5
CAMK2D pS315 pS319	[illegible]	p09633	Calcium/calmodulin-dependent protein kinase type II subunit delta
CREM seq: 273 - 303 + Phospho (ST)	[illegible]	p19869	cAMP-responsive element modulator
DOCK1 seq: 1754 - 1774 + Phospho (ST)	[illegible]	p07414	Dedicator of cytokinesis protein 1
GREB1 pS1193	[illegible]	p14630	Protein GREB1
SARG pS519	[illegible]	p15293	Specifically androgen-regulated gene protein
ARHGEF16 pS107	[illegible]	p14668	Rho guanine nucleotide exchange factor 16
PXN seq: 298 - 317 + Oxidation (M); Phospho (ST)	[illegible]	p18827	Paxillin
MAP1B seq: 539 - 555 + Phospho (ST)	[illegible]	p20890	Microtubule-associated protein 1B
PRR12 seq: 254 - 261 + Phospho (ST)	[illegible]	p02110	Proline-rich protein 12
COBL pS324	[illegible]	p00826	Protein cordon-bleu
RGS3 seq: 915 - 932 + Phospho (ST)	[illegible]	p06596	Regulator of G-protein signaling 3
PTGES3 seq: 79 - 88 no mod	[illegible]	p13196	Prostaglandin E synthase 3
WNK2 pS1862	[illegible]	p08821	Serine/threonine-protein kinase WNK2
ITSN1 seq: 201 - 222 + Phospho (ST)	[illegible]	p19768	Intersectin-1
APBB2 pT34	[illegible]	p09125	Amyloid beta A4 precursor protein-binding family B member 2
INTS5 seq: 999 - 1005 no mod	[illegible]	p11751	Integrator complex subunit 5
PHIP seq: 1775 - 1804 + Phospho (ST)	[illegible]	p03088	PH-interacting protein
SEPT9 pS22	[illegible]	p11813	Septin-9
CDC42EP4 seq: 115 - 125 + Oxidation (M); Phospho (ST)	[illegible]	p09765	Cdc42 effector protein 4
TNS3 pS776	[illegible]	p12922	Tensin-3
USP43 seq: 1062 - 1074 + 2 Phospho (ST)	[illegible]	p18076	Ubiquitin carboxyl-terminal hydrolase 43
ACADSB seq: 285 - 294 no mod	[illegible]	p00451	Short/branched chain specific acyl-CoA dehydrogenase, mitochondrial
NFATC3 pS1063	[illegible]	p08841	Nuclear factor of activated T-cells, cytoplasmic 3
UBAP2 pS254	[illegible]	p16248	Ubiquitin-associated protein 2
ILF3 seq: 409 - 419 no mod	[illegible]	p11171	Interleukin enhancer-binding factor 3
MAP4 seq: 939 - 953 + Phospho (ST)	[illegible]	p01433	Microtubule-associated protein 4
AMBRA1 seq: 1191 - 1212 + Phospho (ST)	[illegible]	p03745	Activating molecule in BECN1-regulated autophagy protein 1
UNG seq: 6 - 17 + Phospho (ST)	[illegible]	p02506	Uracil-DNA glycosylase
TRIOBP pS1955	[illegible]	p08847	TRIO and F-actin-binding protein
MESDC2 seq: 150 - 156 no mod	[illegible]	p16664	LDLR chaperone MESD
STUB1 pS19	[illegible]	p11835	E3 ubiquitin-protein ligase CHIP
PIR seq: 85 - 97 no mod	[illegible]	p09979	Pirin
ARHGEF16 pS107	[illegible]	p14669	Rho guanine nucleotide exchange factor 16
SHROOM3 pS676	[illegible]	p07290	Protein Shroom3
KLC4 pS565	[illegible]	p03922	Kinesin light chain 4
BAIAP2L1 seq: 329 - 341 + Phospho (ST)	[illegible]	p03232	Brain-specific angiogenesis inhibitor 1-associated protein 2-like protein 1
TMPRSS13 seq: 54 - 73 + Phospho (ST)	[illegible]	p19560	Transmembrane protease serine 13
AMOTL2 seq: 595 - 621 + Phospho (ST)	[illegible]	p18148	Angiomotin-like protein 2
IFI16 pS153	[illegible]	p00870	Gamma-interferon-inducible protein 16
PGK1 seq: 172 - 184 + Oxidation (M)	[illegible]	p20509	Phosphoglycerate kinase 1
HN1 pM47 pT54	[illegible]	p10243	Hematological and neurological expressed 1 protein
MAP4 seq: 64 - 107 + Phospho (ST)	[illegible]	p13040	Microtubule-associated protein 4
ANXA2P2 seq: 11 - 28 + Phospho (ST)	[illegible]	p10766	Putative annexin A2-like protein
NONO seq: 191 - 202 no mod	[illegible]	p15834	Non-POU domain-containing octamer-binding protein
CCDC88C pS1887	[illegible]	p07427	Protein Daple
CABLES1 pS168	[illegible]	p16138	CDK5 and ABL1 enzyme substrate 1
PKP3 pS280	[illegible]	p15040	Plakophilin-3
RBSN pS215 pS217	[illegible]	p17148	Rabenosyn-5
EXPH5 seq: 1766 - 1778 + Phospho (ST)	[illegible]	p00782	Exophilin-5
NACA pS2029	[illegible]	p00955	Nascent polypeptide-associated complex subunit alpha, muscle-specific form
NUP153 seq: 1453 - 1465 + Phospho (ST)	[illegible]	p08977	Nuclear pore complex protein Nup153
PATL1 seq: 176 - 188 + 2 Phospho (ST)	[illegible]	p06553	Protein PAT1 homolog 1
ELK3 seq: 243 - 257 + Phospho (ST)	[illegible]	p04413	ETS domain-containing protein Elk-3
TWIST1 pS68	[illegible]	p06338	Twist-related protein 1
BCLAF1 pS496	[illegible]	p13266	Bcl-2-associated transcription factor 1
SRRM2 seq: 1457 - 1471 + Phospho (ST)	[illegible]	p14636	Serine/arginine repetitive matrix protein 2
BUD13 seq: 566 - 575 + Phospho (ST)	[illegible]	p00110	BUD13 homolog
KRT18 seq: 56 - 81 + Oxidation (M); Phospho (ST)	[illegible]	p16022	Keratin, type I cytoskeletal 18
ZNRF2 seq: 80 - 95 + Phospho (ST)	[illegible]	p04048	E3 ubiquitin-protein ligase ZNRF2
DPYSL2 seq: 521 - 532 + Phospho (ST)	[illegible]	p02161	Dihydropyrimidinase-related protein 2
RC3H1 seq: 457 - 488 + Phospho (ST)	[illegible]	p06911	Roquin-1
YAP1 seq: 103 - 124 + 2 Phospho (ST)	[illegible]	p05562	Transcriptional coactivator YAP1
EML4 pS144 pS146	[illegible]	p19528	Echinoderm microtubule-associated protein-like 4
PACSIN2 seq: 288 - 310 no mod	[illegible]	p20050	Protein kinase C and casein kinase substrate in neurons protein 2
EIF4EBP1 seq: 64 - 73 + 2 Phospho (ST)	[illegible]	p09105	Eukaryotic translation initiation factor 4E-binding protein 1
ZC3H18 pS534	[illegible]	p13043	Zinc finger CCCH domain-containing protein 18

ZC3H4 pS1275	2E+08	5E+08	[illegible]	p02790	Zinc finger CCCH domain-containing protein 4
TANC2 seq: 1738 - 1752 + 2 Phospho (ST)	9E+07	1E+08	[illegible]	p03643	Protein TANC2
KRT18 seq: 383 - 407 + Oxidation (M); Phospho (ST)	5E+08	3E+08	[illegible]	p11433	Keratin, type I cytoskeletal 18
MDH1 seq: 126 - 142 no mod	38324	4E+07	[illegible]	p01336	Malate dehydrogenase, cytoplasmic
CAMSAP3 seq: 358 - 378 + Phospho (ST)	3E+07	2E+08	[illegible]	p14697	Calmodulin-regulated spectrin-associated protein 3
DSP pM2819 pS2825	3E+08	3E+08	[illegible]	p15671	Desmoplakin
TJP2 seq: 315 - 322 no mod	1E+07	2E+07	[illegible]	p20059	Tight junction protein ZO-2
TTK pS436	3E+08	4E+08	[illegible]	p14578	Dual specificity protein kinase TTK
HIVEP1 seq: 1729 - 1745 + Phospho (ST)	2E+07	1E+08	[illegible]	p13635	Zinc finger protein 40
SRRM2 seq: 1041 - 1058 + Phospho (Y)	2E+09	9E+09	[illegible]	p03583	Serine/arginine repetitive matrix protein 2
FAM21A seq: 1125 - 1147 + Phospho (ST)	4E+07	1E+07	[illegible]	p16186	WASH complex subunit FAM21A
TGFB1I1 seq: 134 - 148 + 2 Phospho (ST)	8E+07	9E+07	[illegible]	p12624	Transforming growth factor beta-1-induced transcript 1 protein
TARDBP seq: 276 - 293 no mod	3E+08	3E+08	[illegible]	p16757	TAR DNA-binding protein 43
RPL24 pS86	4E+07	5E+07	[illegible]	p20424	60S ribosomal protein L24
MSL1 pS126	9E+08	9E+08	[illegible]	p08862	Male-specific lethal 1 homolog
KIF13B seq: 1422 - 1455 + Phospho (ST)	7E+07	2E+08	[illegible]	p15912	Kinesin-like protein KIF13B
RBM14 pS618	38324	1E+08	[illegible]	p07140	RNA-binding protein 14
TACC3 pS558	2E+08	6E+08	[illegible]	p12641	Transforming acidic coiled-coil-containing protein 3
C12orf45 seq: 173 - 183 + Phospho (ST)	3E+08	3E+08	[illegible]	p14321	Uncharacterized protein C12orf45
TOPORS pS194	1E+08	1E+08	[illegible]	p09743	E3 ubiquitin-protein ligase Topors
SMARCA5 seq: 35 - 83 + Phospho (ST)	38324	37149	[illegible]	p16000	SWI/SNF-related matrix-associated actin-dependent regulator of chromatin subfamily A member 5
ARHGEF17 pS914	2E+08	2E+08	[illegible]	p12290	Rho guanine nucleotide exchange factor 17
MAGED2 pS247	1E+09	2E+09	[illegible]	p20228	Melanoma-associated antigen D2
DENND4A pS731	3E+07	37149	[illegible]	p03659	C-myc promoter-binding protein
HMGCS2 seq: 222 - 228 no mod	2E+07	5E+07	[illegible]	p19973	Hydroxymethylglutaryl-CoA synthase, mitochondrial
TPRN pS241	2E+08	3E+08	[illegible]	p12254	Taperin
RTTN seq: 1915 - 1921 no mod	1E+09	8E+08	[illegible]	p13314	Rotatin
SVIL pS914	6E+07	6E+07	[illegible]	p16442	Supervillin
PLIN5 pS1160	1E+08	1E+08	[illegible]	p11698	Perilipin-5
TGFBR2 seq: 350 - 356 + Phospho (ST)	1E+08	1E+08	[illegible]	p11683	TGF-beta receptor type-2
ROBO4 seq: 120 - 126 + Phospho (ST)	1E+08	1E+08	[illegible]	p11672	Roundabout homolog 4
PDAP1 pS176	2E+08	2E+08	[illegible]	p09923	28 kDa heat- and acid-stable phosphoprotein
SRRM2 seq: 1537 - 1556 + Phospho (ST)	6E+08	4E+08	[illegible]	p04053	Serine/arginine repetitive matrix protein 2
TPD52L2 seq: 1 - 34 + 2 Phospho (ST)	38324	1E+07	[illegible]	p10201	Tumor protein D54
HN1 pS31	1E+09	2E+09	[illegible]	p01183	Hematological and neurological expressed 1 protein
TOP2B seq: 1397 - 1427 + 2 Phospho (ST)	38324	3E+08	[illegible]	p01331	DNA topoisomerase 2-beta
ARHGEF11 seq: 648 - 662 + Phospho (ST)	7E+07	7E+07	[illegible]	p11638	Rho guanine nucleotide exchange factor 11
ZNF652 seq: 194 - 208 + Phospho (ST)	2E+08	1E+09	[illegible]	p07652	Zinc finger protein 652
SEPT9 seq: 18 - 29 + Phospho (ST)	2E+09	4E+09	[illegible]	p07180	Septin-9
KLC4 seq: 587 - 606 + Phospho (ST)	2E+08	5E+08	[illegible]	p07657	Kinesin light chain 4
LIMA1 pS726	8E+07	6E+07	[illegible]	p04275	LIM domain and actin-binding protein 1
SRGAP1 pS932	7E+08	8E+08	[illegible]	p13112	SLIT-ROBO Rho GTPase-activating protein 1
PPHLN1 pS172	3E+08	3E+08	[illegible]	p03290	Periphilin-1
TMF1 seq: 338 - 366 + Phospho (ST)	38324	7E+07	[illegible]	p03282	TATA element modulatory factor
STK25 pT190	3E+07	3E+08	[illegible]	p06995	Serine/threonine-protein kinase 25
TMSB10 seq: 27 - 40 no mod	1E+08	1E+08	[illegible]	p09020	Thymosin beta-10
ITSN1 seq: 313 - 328 + Phospho (ST)	7E+08	7E+08	[illegible]	p05693	Intersectin-1
VRK3 pS82 pS83	1E+07	37149	[illegible]	p10760	Inactive serine/threonine-protein kinase VRK3
PLEKHA7 seq: 554 - 562 + Phospho (ST)	9E+07	3E+07	[illegible]	p04043	Pleckstrin homology domain-containing family A member 7
NDUFS1 seq: 484 - 499 no mod	38324	3E+07	[illegible]	p09712	NADH-ubiquinone oxidoreductase 75 kDa subunit, mitochondrial
PDS5B seq: 1152 - 1171 + Phospho (ST)	2E+08	1E+09	[illegible]	p10152	Sister chromatid cohesion protein PDS5 homolog B
LMO7 seq: 1491 - 1506 + Oxidation (M); Phospho (ST)	1E+08	1E+08	[illegible]	p06214	LIM domain only protein 7
CEP170 seq: 1158 - 1173 + 2 Phospho (ST)	2E+07	1E+07	[illegible]	p11697	Centrosomal protein of 170 kDa
DLGAP4 pS763	5E+08	7E+08	[illegible]	p09396	Disks large-associated protein 4
PRKRA seq: 6 - 25 + Phospho (ST)	6E+08	5E+08	[illegible]	p14665	Interferon-inducible double-stranded RNA-dependent protein kinase activator A
HSPA4 seq: 619 - 629 no mod	38324	1E+07	[illegible]	p09737	Heat shock 70 kDa protein 4
TMPO pS66 pS67 pT74	2E+08	4E+08	[illegible]	p09187	Lamina-associated polypeptide 2, isoforms beta/gamma
LSM14A seq: 192 - 215 + Phospho (ST)	2E+07	1E+08	[illegible]	p04330	Protein LSM14 homolog A
HIST1H1B seq: 68 - 82 no mod	2E+08	2E+09	[illegible]	p20335	Histone H1.5
NEDD4L seq: 365 - 394 + Phospho (ST)	2E+09	1E+09	[illegible]	p03575	E3 ubiquitin-protein ligase NEDD4-like
MAP1B pS2271	4E+08	5E+08	[illegible]	p05393	Microtubule-associated protein 1B
NUCB2 seq: 60 - 72 + Gln->pyro-Glu (N-term Q)	38324	3E+07	[illegible]	p07723	Nucleobindin-2
CLASP2 seq: 358 - 367 + Phospho (ST)	4E+06	3E+07	[illegible]	p19488	CLIP-associating protein 2
THRAP3 pS560	5E+07	5E+07	[illegible]	p03906	Thyroid hormone receptor-associated protein 3
MOB4 seq: 127 - 140 no mod	38324	2E+07	[illegible]	p14582	MOB-like protein phocein
GTF2F1 seq: 383 - 402 + Phospho (ST)	6E+08	7E+08	[illegible]	p15564	General transcription factor IIF subunit 1
SEPT10 pS451	7E+07	7E+07	[illegible]	p12835	Septin-10
GPHN pS188 pS194 pT198	1E+08	37149	[illegible]	p01320	Gephyrin
UQCRC1 seq: 433 - 442 no mod	5E+07	9E+07	[illegible]	p14516	Cytochrome b-c1 complex subunit 1, mitochondrial
RAD54L2 seq: 1193 - 1225 + Phospho (ST)	2E+08	1E+08	[illegible]	p07841	Helicase ARIP4
TCEB1 seq: 7 - 20 no mod	6E+07	3E+07	[illegible]	p01870	Transcription elongation factor B polypeptide 1
PDXDC1 pS718	2E+08	1E+08	[illegible]	p15307	Pyridoxal-dependent decarboxylase domain-containing protein 1
DOCK4 seq: 1803 - 1814 + Phospho (ST)	38324	8E+06	[illegible]	p14575	Dedicator of cytokinesis protein 4
PFIA1 seq: 1166 - 1178 + 2 Oxidation (M); Phospho (ST)	1E+09	1E+09	[illegible]	p00676	Liprin-alpha-1
1945 - 1976 + Gln->pyro-Glu (N-term Q); 2 Phospho (ST)	9E+08	7E+08	[illegible]	p08375	Myosin-10
CARD11 pS925	38324	37149	[illegible]	p14139	Caspase recruitment domain-containing protein 11
ARFGAP2 pS368	7E+08	6E+08	[illegible]	p00291	ADP-ribosylation factor GTPase-activating protein 2
PDIA3 seq: 297 - 304 no mod	5E+07	7E+07	[illegible]	p11192	Protein disulfide-isomerase A3
IQGAP1 seq: 812 - 817 + Phospho (ST)	1E+07	1E+07	[illegible]	p13785	Ras GTPase-activating-like protein IQGAP1
DOCK3 pS836	1E+07	1E+07	[illegible]	p10935	Dedicator of cytokinesis protein 3
UBA2 seq: 506 - 512 no mod	9E+08	3E+08	[illegible]	p10820	SUMO-activating enzyme subunit 2
HN1 pT54	2E+08	2E+08	[illegible]	p10241	Hematological and neurological expressed 1 protein
RNF219 pS719	1E+08	9E+07	[illegible]	p13076	RING finger protein 219
EIF4H pS14	1E+08	9E+07	[illegible]	p19099	Eukaryotic translation initiation factor 4H
MAP7D1 pS399	2E+09	5E+09	[illegible]	p07221	MAP7 domain-containing protein 1
FKBP15 seq: 1161 - 1178 + Phospho (ST)	4E+07	6E+07	[illegible]	p03838	FK506-binding protein 15
MTA1 seq: 446 - 456 + Oxidation (M); Phospho (ST)	2E+08	2E+08	[illegible]	p04831	Metastasis-associated protein MTA1
APC seq: 1436 - 1450 + Phospho (ST)	8E+06	4E+07	[illegible]	p05334	Adenomatous polyposis coli protein
CNBP pS47	2E+08	4E+08	[illegible]	p16102	Cellular nucleic acid-binding protein
HSPA5 seq: 82 - 97 no mod	2E+08	3E+08	[illegible]	p09222	78 kDa glucose-regulated protein
TSC2 pS1420	2E+08	5E+07	[illegible]	p04195	Tuberin
PALLD seq: 736 - 770 + 2 Phospho (ST)	1E+08	1E+08	[illegible]	p08694	Palladin
SRRM2 seq: 2419 - 2430 + Oxidation (M); Phospho (ST)	1E+09	1E+09	[illegible]	p09846	Serine/arginine repetitive matrix protein 2
CALML5 seq: 43 - 50 no mod	2E+08	2E+08	[illegible]	p09417	Calmodulin-like protein 5
IRS2 pS594	6E+07	5E+07	[illegible]	p07971	Insulin receptor substrate 2
CCDC125 seq: 502 - 511 + Phospho (ST)	7E+08	7E+08	[illegible]	p05574	Coiled-coil domain-containing protein 125
ARHGEF12 pS41	3E+08	3E+08	[illegible]	p14470	Rho guanine nucleotide exchange factor 12
HSPA1A seq: 518 - 526 no mod	4E+08	2E+08	[illegible]	p09818	Heat shock 70 kDa protein 1A
FKBP15 seq: 344 - 361 + Phospho (ST)	2E+08	3E+08	[illegible]	p04779	FK506-binding protein 15
KMT2B seq: 148 - 154 no mod	2E+07	5E+07	[illegible]	p19939	Histone-lysine N-methyltransferase 2B
PHF8 pS120	2E+08	2E+08	[illegible]	p02784	Histone lysine demethylase PHF8
ATXN2 pS784	9E+07	6E+07	[illegible]	p15844	Ataxin-2
CDKN1A pS130	3E+07	3E+07	[illegible]	p05769	Cyclin-dependent kinase inhibitor 1
CRIP2 seq: 109 - 128 + 2 Phospho (ST)	9E+07	1E+08	[illegible]	p15432	Cysteine-rich protein 2
+ Gln->pyro-Glu (N-term Q); Oxidation (M); Phospho (ST)	2E+08	3E+08	[illegible]	p07789	Heterochromatin protein 1-binding protein 3
IQSEC1 pS180	1E+08	2E+08	[illegible]	p09935	IQ motif and SEC7 domain-containing protein 1
AIP seq: 178 - 188 no mod	7E+07	2E+08	[illegible]	p19213	AH receptor-interacting protein
PPP1R37 pS591 pS597	8E+08	3E+08	[illegible]	p20816	Protein phosphatase 1 regulatory subunit 37
FOSL1 pS101	38324	3E+06	[illegible]	p06752	Fos-related antigen 1
HABP4 pS108	38324	7E+07	[illegible]	p05125	Intracellular hyaluronan-binding protein 4
AKAP13 seq: 2718 - 2731 + Phospho (ST)	3E+07	6E+07	[illegible]	p05685	A-kinase anchor protein 13
PLEC pS1435	2E+07	3E+07	[illegible]	p19793	Plectin
NUCKS1 pS181	1E+09	3E+09	[illegible]	p11526	Nuclear ubiquitous casein and cyclin-dependent kinase substrate 1
YAP1 seq: 162 - 181 + Phospho (ST)	5E+08	4E+08	[illegible]	p07847	Transcriptional coactivator YAP1
MYO6 pS1155	9E+08	8E+08	[illegible]	p03228	Unconventional myosin-VI
SAMD4B seq: 236 - 259 + Oxidation (M); Phospho (ST)	3E+08	3E+08	[illegible]	p04871	Protein Smaug homolog 2
TP53BP2 seq: 735 - 751 + Phospho (ST)	2E+08	2E+08	[illegible]	p06610	Apoptosis-stimulating of p53 protein 2
MAP3K11 seq: 514 - 529 + Phospho (ST)	5E+07	7E+07	[illegible]	p06987	Mitogen-activated protein kinase kinase kinase 11
PAGR1 pS237	3E+08	2E+08	[illegible]	p18612	PAXIP1-associated glutamate-rich protein 1
KIAA1522 seq: 255 - 294 + Phospho (ST)	2E+07	3E+08	[illegible]	p19954	Uncharacterized protein KIAA1522
DVL2 seq: 477 - 484 + Phospho (ST)	2E+08	6E+08	[illegible]	p12356	Segment polarity protein dishevelled homolog DVL-2
HDAC1 seq: 405 - 413 + Phospho (ST)	8E+07	1E+08	[illegible]	p13730	Histone deacetylase 1
IRF2BP1 pS66	3E+08	3E+08	[illegible]	p04625	Interferon regulatory factor 2-binding protein 1
ALKBH5 pS361	2E+08	3E+08	[illegible]	p07354	RNA demethylase ALKBH5
TERF2 pS365	3E+07	7E+07	[illegible]	p18516	Telomeric repeat-binding factor 2
ZNF687 pS1118	2E+07	7E+07	[illegible]	p03621	Zinc finger protein 687
YBX1 pS209	5E+08	4E+08	[illegible]	p06866	Nuclease-sensitive element-binding protein 1
TBC1D22B pS154	38324	7E+07	[illegible]	p08006	TBC1 domain family member 22B
EMSY seq: 206 - 218 + Phospho (ST)	2E+08	2E+08	[illegible]	p06516	Protein EMSY
TRIM3 pS437	2E+08	2E+08	[illegible]	p01296	Tripartite motif-containing protein 3
PHIP pS1315	1E+07	2E+07	[illegible]	p19747	PH-interacting protein
PTGES3 seq: 96 - 107 no mod	38324	37149	[illegible]	p11142	Prostaglandin E synthase 3
USP15 seq: 241 - 256 + Phospho (ST)	6E+07	4E+07	[illegible]	p13680	Ubiquitin carboxyl-terminal hydrolase 15
RAF1 seq: 283 - 309 + Phospho (ST)	5E+08	4E+08	[illegible]	p05583	RAF proto-oncogene serine/threonine-protein kinase
HES1 seq: 9 - 29 + Phospho (ST)	2E+08	1E+08	[illegible]	p09070	Transcription factor HES-1
PRR5 seq: 309 - 321 + Phospho (ST)	2E+08	2E+08	[illegible]	p03825	Proline-rich protein 5
RPL35 seq: 78 - 86 no mod	1E+06	5E+06	[illegible]	p00286	60S ribosomal protein L35
EXOSC1 pS21	9E+08	9E+08	[illegible]	p12118	Exosome complex component CSL4
ABI2 pM221 pS227	1E+09	1E+09	[illegible]	p09382	Abl interactor 2
CA2 seq: 159 - 167 no mod	2E+07	3E+07	[illegible]	p00641	Carbonic anhydrase 2
CRYBG3 seq: 344 - 376 + Phospho (ST)	38324	37149	[illegible]	p09179	Very large A-kinase anchor protein
SH3PXD2A seq: 402 - 411 + Phospho (ST)	6E+07	1E+08	[illegible]	p12199	SH3 and PX domain-containing protein 2A
AIM1 pS103	5E+09	4E+09	[illegible]	p05388	Absent in melanoma 1 protein
TUBB8 seq: 351 - 359 no mod	3E+08	6E+08	[illegible]	p03000	Tubulin beta-8 chain
PHACTR2 seq: 558 - 571 + Phospho (ST)	38324	37149	[illegible]	p12917	Phosphatase and actin regulator 2
SLC9A3R1 seq: 159 - 172 no mod	2E+08	5E+08	[illegible]	p13173	Na(+)/H(+) exchange regulatory cofactor NHE-RF1
MLF1 seq: 32 - 39 + Phospho (ST)	7E+06	37149	[illegible]	p05855	Myeloid leukemia factor 1
seq: 117 - 143 + Gln->pyro-Glu (N-term Q); Phospho (ST)	8E+06	7E+07	[illegible]	p08499	Mitochondrial fission regulator 1
SRRM2 seq: 889 - 900 + Phospho (ST)	3E+09	5E+09	[illegible]	p14651	Serine/arginine repetitive matrix protein 2
MTDH pS568	38324	2E+08	[illegible]	p05952	Protein LYRIC
STARD10 seq: 4 - 19 no mod	2E+08	4E+08	[illegible]	p12299	PCTP-like protein
FAM122B seq: 108 - 126 + 2 Phospho (ST)	2E+07	8E+07	[illegible]	p07275	Protein FAM122B
RPLP2 pS101	5E+09	4E+09	[illegible]	p13297	60S acidic ribosomal protein P2
PRKACA seq: 194 - 214 + Phospho (ST)	6E+08	7E+08	[illegible]	p15319	cAMP-dependent protein kinase catalytic subunit alpha
YTHDC1 seq: 117 - 132 + Phospho (ST)	2E+08	3E+08	[illegible]	p10640	YTH domain-containing protein 1
MCM2 seq: 18 - 30 + Phospho (ST)	1E+09	2E+09	[illegible]	p06755	DNA replication licensing factor MCM2
P4HB seq: 214 - 222 no mod	5E+08	7E+08	[illegible]	p09343	Protein disulfide-isomerase
ZNRF2 pS145 pS151	3E+07	2E+08	[illegible]	p10417	E3 ubiquitin-protein ligase ZNRF2
SLTM pM993 pS1002	1E+09	2E+09	[illegible]	p20725	SAFB-like transcription modulator
SMG1 seq: 3566 - 3583 + Phospho (ST)	3E+08	2E+08	[illegible]	p09479	Serine/threonine-protein kinase SMG1
SLAIN2 pS48	7E+07	7E+07	[illegible]	p05786	SLAIN motif-containing protein 2
MACF1 seq: 7328 - 7361 + Phospho (ST)	38324	37149	[illegible]	p07363	Microtubule-actin cross-linking factor 1, isoforms 1/2/3/5
MAP7D1 pS446	38324	4E+07	[illegible]	p07885	MAP7 domain-containing protein 1
DYNC1LI2 seq: 440 - 457 no mod	1E+07	1E+07	[illegible]	p12495	Cytoplasmic dynein 1 light intermediate chain 2
SRRM2 pM1396 pS1404 pT1413	9E+08	7E+08	[illegible]	p20612	Serine/arginine repetitive matrix protein 2
ATG2A pS403	5E+08	4E+08	[illegible]	p07149	Autophagy-related protein 2 homolog A
GTF2I pS823	6E+07	8E+08	[illegible]	p12800	General transcription factor II-I
seq: 134 - 165 + Gln->pyro-Glu (N-term Q); Phospho (ST)	1E+07	9E+07	[illegible]	p08477	Chromatin complexes subunit BAP18
FDXR seq: 80 - 90 no mod	2E+07	5E+07	[illegible]	p16727	NADPH:adrenodoxin oxidoreductase, mitochondrial
PDAP1 seq: 173 - 181 + Phospho (ST)	4E+07	6E+07	[illegible]	p07057	28 kDa heat- and acid-stable phosphoprotein
CRIP2 seq: 103 - 128 + 2 Phospho (ST)	1E+08	1E+08	[illegible]	p19613	Cysteine-rich protein 2
HSD17B10 seq: 54 - 69 no mod	1E+08	2E+08	[illegible]	p11728	3-hydroxyacyl-CoA dehydrogenase type-2
CTTNBP2NL seq: 437 - 458 + Phospho (ST)	4E+09	2E+09	[illegible]	p11391	CTTNBP2 N-terminal-like protein
ACTN4 seq: 498 - 512 no mod	1E+08	2E+08	[illegible]	p14457	Alpha-actinin-4
GTF2F1 pS433	2E+07	3E+07	[illegible]	p10890	General transcription factor IIF subunit 1
HIST1H1B pS18	4E+06	8E+07	[illegible]	p05965	Histone H1.5
SMG6 pT479	1E+08	1E+08	[illegible]	p02237	Telomerase-binding protein EST1A
SYTL4 pS217	1E+08	3E+08	[illegible]	p07512	Synaptotagmin-like protein 4
NRP2 seq: 229 - 239 no mod	6E+08	4E+08	[illegible]	p00432	Neuropilin-2
BICD2 pS582	2E+08	5E+08	[illegible]	p15327	Protein bicaudal D homolog 2
MSANTD3 seq: 96 - 108 + Phospho (ST)	38324	3E+07	[illegible]	p03268	Myb/SANT-like DNA-binding domain-containing protein 3
CCT6A seq: 130 - 138 no mod	3E+07	1E+08	[illegible]	p20194	T-complex protein 1 subunit zeta
RANBP2 pS1835	2E+08	2E+08	[illegible]	p11654	E3 SUMO-protein ligase RanBP2
MAP3K11 pS524	4E+08	4E+08	[illegible]	p08978	Mitogen-activated protein kinase kinase kinase 11
FKBP15 seq: 344 - 361 + Phospho (ST)	8E+08	9E+08	[illegible]	p04780	FK506-binding protein 15
NUCKS1 pS181	2E+10	4E+10	[illegible]	p11525	Nuclear ubiquitous casein and cyclin-dependent kinase substrate 1
DMXL1 seq: 1903 - 1915 + Phospho (ST)	3E+07	2E+07	[illegible]	p18927	DmX-like protein 1
DLGAP4 seq: 662 - 679 + Phospho (ST)	1E+07	1E+08	[illegible]	p07225	Disks large-associated protein 4
FAM83H seq: 1023 - 1041 + Phospho (ST)	7E+07	1E+08	[illegible]	p10675	Protein FAM83H
RAPH1 pS571	38324	2E+07	[illegible]	p04193	Ras-associated and pleckstrin homology domains-containing protein 1
MZT2A pS139	4E+08	8E+08	[illegible]	p08106	Mitotic-spindle organizing protein 2A
CAMSAP3 seq: 768 - 779 + Phospho (ST)	2E+08	2E+08	[illegible]	p06650	Calmodulin-regulated spectrin-associated protein 3
TBC1D4 pS341	3E+09	2E+09	[illegible]	p14884	TBC1 domain family member 4
BAIAP2L1 seq: 390 - 398 + Phospho (ST)	4E+07	1E+07	[illegible]	p14958	Brain-specific angiogenesis inhibitor 1-associated protein 2-like protein 1
CTNND1 pM158 pS169	2E+08	2E+08	[illegible]	p01928	Catenin delta-1
KHDRBS1 seq: 18 - 31 + Oxidation (M); Phospho (ST)	4E+09	3E+09	[illegible]	p05679	KH domain-containing, RNA-binding, signal transduction-associated protein 1
CD81 seq: 125 - 144 no mod	4E+07	1E+08	[illegible]	p08667	CD81 antigen
LASP1 pT104	3E+08	6E+08	[illegible]	p15825	LIM and SH3 domain protein 1
PLEC pS1435	8E+08	1E+09	[illegible]	p19792	Plectin
ZBTB7A seq: 503 - 531 + Phospho (ST)	3E+07	4E+08	[illegible]	p00902	Zinc finger and BTB domain-containing protein 7A
DDX10 seq: 2 - 18 + Phospho (ST)	4E+07	1E+08	[illegible]	p15796	Probable ATP-dependent RNA helicase DDX10
ZFX seq: 354 - 374 no mod	38324	2E+08	[illegible]	p02861	Zinc finger X-chromosomal protein
ZC2HC1A seq: 219 - 227 + Phospho (ST)	2E+08	3E+08	[illegible]	p10927	Zinc finger C2HC domain-containing protein 1A
REPS1 seq: 153 - 179 + 2 Phospho (ST)	1E+09	8E+08	[illegible]	p02199	RalBP1-associated Eps domain-containing protein 1
PDLIM2 pS129	9E+07	9E+07	[illegible]	p03564	PDZ and LIM domain protein 2
ERC1 seq: 7 - 23 + Phospho (ST)	2E+08	2E+08	[illegible]	p03343	ELKS/Rab6-interacting/CAST family member 1
BAP18 pS110	9E+07	2E+08	[illegible]	p01760	Chromatin complexes subunit BAP18
MISP seq: 375 - 391 + Phospho (ST)	6E+07	1E+08	[illegible]	p19445	Mitotic interactor and substrate of PLK1
RAPH1 seq: 569 - 583 + Phospho (ST)	38324	2E+07	[illegible]	p04192	Ras-associated and pleckstrin homology domains-containing protein 1
TNFRSF10A pS466	38324	37149	[illegible]	p16658	Tumor necrosis factor receptor superfamily member 10A

TCF12 seq: 75 - 90 + Phospho (ST)	3E+07	3E+07	3E+07 2E+07 1E+07 8E+07 7E+07 1E+07 1E+07 26736 6E+07 4E+07 1E+07 1E+07 9E+06 [illegible]	4E+07	2E+07	4E+07	p16088	Transcription factor 12
CDC42EP2 pS109	38324	37149	6E+06 13654 33854 1E+07 25552 6E+06 34104 26736 56234 53206 39754 40879 18798 [illegible]	1E+08	2E+08	2E+08	p09773	Cdc42 effector protein 2
TCF20 seq: 579 - 598 + Phospho (ST)	6E+07	4E+07	1E+08 1E+07 3E+07 1E+08 8E+07 5E+07 34104 1E+07 1E+08 2E+07 3E+07 3E+07 1E+07 [illegible]	7E+07	7E+07	4E+07	p17855	Transcription factor 20
PPP1R14B seq: 28 - 51 + Phospho (ST)	6E+07	2E+07	39656 13654 2E+07 45779 25552 4E+07 34104 26736 56234 53206 39754 9E+06 18798 [illegible]	3E+08	1E+08	1E+08	p00534	Protein phosphatase 1 regulatory subunit 14B
TSC22D4 pS165	8E+07	2E+08	8E+07 5E+07 8E+07 5E+07 1E+08 7E+07 3E+07 4E+07 3E+07 3E+07 3E+07 40879 3E+07 [illegible]	6E+08	7E+08	7E+08	p05806	TSC22 domain family protein 4
NAE1 seq: 271 - 293 no mod	1E+07	8E+06	1E+07 2E+07 4E+07 6E+08 7E+07 2E+07 4E+07 9E+06 2E+08 9E+07 2E+07 3E+07 3E+07 [illegible]	1E+07	30272	25732	p08957	NEDD8-activating enzyme E1 regulatory subunit
KHDRBS1 seq: 18 - 31 + Oxidation (M); Phospho (ST)	5E+09	5E+09	6E+09 3E+09 4E+09 4E+09 5E+09 5E+09 3E+09 2E+09 3E+09 4E+09 5E+09 4E+09 3E+09 [illegible]	4E+09	3E+09	2E+09	p05678	KH domain-containing, RNA-binding, signal transduction-associated protein 1
FAM122B seq: 108 - 122 + Phospho (ST)	9E+06	6E+07	3E+07 3E+07 3E+07 1E+08 4E+07 34892 1E+07 2E+07 3E+08 1E+08 6E+07 5E+07 9E+07 [illegible]	4E+07	4E+07	4E+07	p07277	Protein FAM122B
SEPT6 seq: 401 - 421 + Phospho (ST)	2E+08	2E+08	2E+08 2E+08 2E+08 4E+08 2E+08 2E+08 2E+08 1E+08 2E+08 1E+08 1E+08 1E+08 9E+07 [illegible]	5E+07	3E+07	3E+07	p03105	Septin-6
TANC2 pS1647	38324	1E+07	6E+07 3E+07 2E+07 45779 25552 4E+07 1E+07 3E+06 4E+07 2E+07 4E+07 40879 18798 [illegible]	25947	30272	25732	p00150	Protein TANC2
GAB1 seq: 385 - 395 + Phospho (ST)	8E+08	37149	39656 1E+07 4E+08 2E+09 8E+08 6E+06 2E+08 1E+07 2E+09 53206 4E+08 3E+08 4E+08 [illegible]	1E+08	9E+08	7E+07	p04746	GRB2-associated-binding protein 1
RALGAPA2 pS696	1E+08	2E+08	2E+08 2E+08 2E+08 3E+08 2E+08 2E+08 2E+08 1E+08 2E+08 2E+08 2E+08 2E+08 1E+08 [illegible]	1E+08	1E+08	1E+08	p05833	Ral GTPase-activating protein subunit alpha-2
SH2D3A seq: 123 - 140 + Phospho (ST)	38324	7E+06	2E+07 2E+07 1E+07 45779 25552 8E+06 34104 26736 3E+07 3E+07 39754 3E+07 2E+07 [illegible]	1E+08	3E+08	3E+08	p05857	SH2 domain-containing protein 3A
BRWD1 pS1475	2E+08	2E+08	4E+08 2E+08 2E+08 3E+08 4E+08 34892 2E+08 2E+08 1E+08 3E+08 2E+08 2E+08 3E+08 [illegible]	7E+07	5E+07	25732	p14151	Bromodomain and WD repeat-containing protein 1
HSPA4 seq: 306 - 316 no mod	38324	1E+07	39656 13654 1E+07 8E+07 25552 34892 34104 26736 4E+08 53206 4E+07 40879 18798 [illegible]	25947	30272	25732	p16631	Heat shock 70 kDa protein 4
DSP pS176	5E+08	2E+08	4E+08 4E+08 2E+08 6E+08 3E+08 3E+08 2E+08 1E+08 5E+08 2E+08 2E+08 1E+08 2E+08 [illegible]	25947	30272	25732	p16053	Desmoplakin
FAM122A seq: 136 - 150 + Phospho (ST)	8E+07	1E+08	1E+08 2E+08 1E+08 5E+08 25552 1E+08 7E+07 7E+07 6E+08 5E+08 2E+08 3E+08 2E+08 [illegible]	3E+08	2E+08	2E+08	p07281	Protein FAM122A
TPRN pS241	5E+08	7E+08	7E+08 7E+08 6E+08 1E+09 6E+08 6E+08 6E+08 4E+08 1E+09 8E+08 6E+08 6E+08 4E+08 [illegible]	1E+08	1E+08	2E+08	p12253	Taperin
PDS5B seq: 1152 - 1169 + Oxidation (M); Phospho (ST)	2E+08	4E+08	[illegible]	1E+07	1E+07	25732	p10155	Sister chromatid cohesion protein PDS5 homolog B
q: 756 - 781 + Gln->pyro-Glu (N-term Q); 2 Phospho (ST)	2E+08	2E+08	[illegible]	2E+08	2E+08	3E+08	p08176	Ataxin-2
EIF4G1 pS1194	2E+09	8E+08	[illegible]	9E+06	30272	25732	p04120	Eukaryotic translation initiation factor 4 gamma 1
AMOTL2 seq: 41 - 71 + Phospho (ST)	4E+08	5E+08	[illegible]	6E+08	5E+08	5E+08	p16079	Angiomotin-like protein 2
GAB2 pT391	4E+07	8E+07	[illegible]	2E+07	1E+08	1E+08	p06992	GRB2-associated-binding protein 2
DIP2B seq: 178 - 198 + Phospho (ST)	2E+08	8E+08	[illegible]	5E+07	4E+07	3E+07	p05486	Disco-interacting protein 2 homolog B
GAS2L1 pS316	6E+08	6E+08	[illegible]	2E+07	2E+07	2E+07	p07318	GAS2-like protein 1
TSC22D1 seq: 261 - 294 + Phospho (ST)	2E+08	4E+08	[illegible]	4E+07	1E+08	25732	p12898	TSC22 domain family protein 1
ANKIB1 seq: 877 - 895 + Phospho (ST)	2E+08	3E+08	[illegible]	2E+08	6E+07	9E+07	p18873	Ankyrin repeat and IBR domain-containing protein 1
eq: 584 - 602 + Gln->pyro-Glu (N-term Q); Phospho (ST)	1E+08	8E+07	[illegible]	4E+07	9E+07	5E+07	p07972	Insulin receptor substrate 2
PRRC2B seq: 1690 - 1710 + Phospho (ST)	2E+08	2E+08	[illegible]	3E+07	9E+07	5E+07	p03757	Protein PRRC2B
CDKL5 seq: 341 - 355 + Phospho (ST)	1E+08	2E+08	[illegible]	25947	30272	25732	p04788	Cyclin-dependent kinase-like 5
FAM83H pS914	3E+09	2E+09	[illegible]	4E+08	3E+08	4E+08	p15187	Protein FAM83H
TJP2 seq: 976 - 999 + 2 Phospho (ST)	3E+08	3E+08	[illegible]	4E+08	2E+09	1E+09	p21015	Tight junction protein ZO-2
WAPL pS77	1E+08	6E+07	[illegible]	1E+08	2E+08	2E+08	p12779	Wings apart-like protein homolog
IRS2 seq: 584 - 602 + Phospho (ST)	1E+08	2E+08	[illegible]	2E+08	1E+08	1E+08	p07970	Insulin receptor substrate 2
FAM122B pS115	2E+08	2E+08	[illegible]	2E+08	2E+08	1E+08	p07276	Protein FAM122B
HJURP pS185	7E+07	5E+07	[illegible]	25947	8E+07	2E+08	p00693	Holliday junction recognition protein
PPP1R12A seq: 694 - 709 + Phospho (ST)	7E+07	2E+08	[illegible]	6E+07	2E+08	1E+08	p06555	Protein phosphatase 1 regulatory subunit 12A
NCOR1 seq: 2392 - 2418 + Phospho (ST)	3E+08	4E+08	[illegible]	2E+08	5E+08	3E+08	p09996	Nuclear receptor corepressor 1
LMO7 seq: 930 - 948 + Phospho (ST)	1E+08	6E+07	[illegible]	25947	1E+08	6E+07	p03116	LIM domain only protein 7
LMNA seq: 297 - 311 + Phospho (ST)	4E+06	2E+07	[illegible]	7E+07	2E+08	1E+08	p13771	Prelamin-A/C
TBC1D15 pS70	4E+08	3E+08	[illegible]	7E+07	7E+07	8E+07	p13318	TBC1 domain family member 15
PROM2 seq: 86 - 95 no mod	1E+09	9E+08	[illegible]	5E+08	4E+08	25732	p20233	Prominin-2
HN1 pS31	1E+09	2E+09	[illegible]	3E+08	3E+08	3E+08	p01182	Hematological and neurological expressed 1 protein
RPL28 seq: 48 - 58 no mod	4E+07	7E+08	[illegible]	2E+08	1E+08	7E+07	p01963	60S ribosomal protein L28
VASP seq: 322 - 346 + Phospho (ST)	2E+08	1E+08	[illegible]	25947	30272	25732	p03619	Vasodilator-stimulated phosphoprotein
DPYSL2 seq: 512 - 525 + Phospho (ST)	2E+08	2E+08	[illegible]	5E+08	9E+08	9E+08	p01878	Dihydropyrimidinase-related protein 2
DTD1 pS197	38324	37149	[illegible]	2E+08	2E+08	2E+08	p06225	D-tyrosyl-tRNA(Tyr) deacylase 1
AIMP1 seq: 137 - 154 + Phospho (ST)	4E+07	5E+07	[illegible]	3E+08	7E+08	8E+06	p12653	Aminoacyl tRNA synthase complex-interacting multifunctional protein 1
OSBPL11 pS172 pS184	2E+08	8E+07	[illegible]	25947	3E+07	2E+08	p05841	Oxysterol-binding protein-related protein 11
AP3D1 pM5 pS9	4E+08	4E+08	[illegible]	25947	30272	25732	p09824	AP-3 complex subunit delta-1
RPS13 seq: 56 - 64 no mod	1E+08	2E+08	[illegible]	7E+06	30272	1E+07	p18276	40S ribosomal protein S13
TCEA1 seq: 20 - 29 no mod	3E+07	4E+07	[illegible]	25947	2E+07	2E+07	p09798	Transcription elongation factor A protein 1
MTDH pS308	2E+07	7E+08	[illegible]	2E+08	4E+08	3E+08	p10642	Protein LYRIC
PLCH1 pS1307	3E+08	3E+08	[illegible]	25947	2E+07	25732	p03102	1-phosphatidylinositol 4,5-bisphosphate phosphodiesterase eta-1
FKBP15 seq: 306 - 334 + Phospho (ST)	3E+09	2E+09	[illegible]	1E+09	8E+08	9E+08	p18310	FK506-binding protein 15
FLNA seq: 1360 - 1375 no mod	2E+08	2E+08	[illegible]	4E+08	1E+08	6E+06	p01420	Filamin-A
HPGD seq: 85 - 98 no mod	1E+07	1E+07	[illegible]	25947	30272	25732	p12079	15-hydroxyprostaglandin dehydrogenase [NAD(+)]
SRRM2 seq: 2302 - 2328 + Phospho (ST)	1E+08	1E+08	[illegible]	9E+07	30272	25732	p02438	Serine/arginine repetitive matrix protein 2
PRRC2B seq: 1420 - 1430 + Phospho (ST)	2E+08	2E+08	[illegible]	2E+08	2E+08	9E+07	p05811	Protein PRRC2B
ALDH2 seq: 347 - 355 no mod	4E+07	2E+08	[illegible]	25947	30272	25732	p00622	Aldehyde dehydrogenase, mitochondrial
USP15 seq: 950 - 981 + Phospho (ST)	1E+08	5E+07	[illegible]	9E+07	7E+07	25732	p16268	Ubiquitin carboxyl-terminal hydrolase 15
PLEKHG3 seq: 430 - 442 + Phospho (ST)	4E+08	3E+08	[illegible]	2E+08	2E+08	1E+08	p19133	Pleckstrin homology domain-containing family G member 3
FAM21A seq: 797 - 822 + 2 Phospho (ST)	3E+07	1E+08	[illegible]	8E+07	5E+07	7E+07	p02639	WASH complex subunit FAM21A
SUPT5H seq: 669 - 681 + Oxidation (M); Phospho (ST)	5E+08	6E+08	[illegible]	1E+08	5E+08	5E+08	p13656	Transcription elongation factor SPT5
RPL12 seq: 131 - 146 no mod	4E+08	3E+08	[illegible]	8E+07	7E+07	7E+07	p17614	60S ribosomal protein L12
EHD2 seq: 453 - 471 + Phospho (ST)	38324	37149	[illegible]	2E+08	30272	25732	p00429	EH domain-containing protein 2
PHLDB1 pS419	4E+08	4E+08	[illegible]	8E+08	5E+08	6E+08	p01130	Pleckstrin homology-like domain family B member 1
eq: 220 - 238 + Gln->pyro-Glu (N-term Q); Phospho (ST)	6E+07	4E+07	[illegible]	25947	1E+07	25732	p08843	Negative elongation factor A
RANBP9 seq: 173 - 186 no mod	9E+07	2E+08	[illegible]	25947	5E+07	2E+07	p18687	Ran-binding protein 9
DFFA pS110	38324	4E+08	[illegible]	5E+07	6E+07	8E+07	p00509	DNA fragmentation factor subunit alpha
TXLNA seq: 13 - 53 + Phospho (ST)	1E+08	1E+08	[illegible]	3E+08	2E+08	3E+08	p07870	Alpha-taxilin
HSPA8 seq: 127 - 137 no mod	3E+07	1E+08	[illegible]	8E+07	5E+07	6E+07	p10055	Heat shock cognate 71 kDa protein
NEK4 pS639	7E+07	9E+07	[illegible]	25947	30272	25732	p13392	Serine/threonine-protein kinase Nek4
STIM2 seq: 695 - 716 + Phospho (ST)	5E+07	3E+08	[illegible]	8E+07	1E+08	7E+07	p14579	Stromal interaction molecule 2
RTKN pS543	2E+08	2E+08	[illegible]	8E+08	7E+08	8E+08	p03394	Rhotekin
RPL12 pS38	1E+09	1E+09	[illegible]	2E+09	2E+09	2E+09	p14248	60S ribosomal protein L12
KRT19 pM259	7E+09	5E+09	[illegible]	3E+09	5E+09	2E+09	p04145	Keratin, type I cytoskeletal 19
DYNC1LI2 pS446	6E+07	7E+07	[illegible]	6E+07	5E+07	6E+07	p12494	Cytoplasmic dynein 1 light intermediate chain 2
FAM65A pS45	38324	7E+06	[illegible]	2E+08	8E+07	7E+07	p05866	Protein FAM65A
MAP7 seq: 199 - 214 + 2 Phospho (ST)	1E+08	2E+08	[illegible]	1E+08	9E+07	1E+08	p10629	Ensconsin
RPS17 seq: 24 - 33 no mod	2E+07	6E+07	[illegible]	9E+06	30272	25732	p11729	40S ribosomal protein S17
q: 475 - 497 + Gln->pyro-Glu (N-term Q); 2 Phospho (ST)	8E+08	6E+08	[illegible]	2E+07	4E+07	25732	p08650	Breast carcinoma-amplified sequence 3
RAB3IP seq: 161 - 170 + Phospho (ST)	2E+07	2E+07	[illegible]	25947	30272	25732	p10860	Rab-3A-interacting protein
SYNRG seq: 1073 - 1083 + Phospho (ST)	1E+08	1E+08	[illegible]	3E+08	3E+08	3E+08	p05044	Synergin gamma
DENND1A seq: 638 - 672 + Phospho (ST)	38324	3E+07	[illegible]	25947	30272	25732	p10322	DENN domain-containing protein 1A
LAD1 seq: 392 - 403 + Oxidation (M); Phospho (ST)	3E+08	3E+08	[illegible]	6E+08	6E+08	5E+08	p06256	Ladinin-1
PSMA7 seq: 152 - 165 no mod	9E+06	3E+07	[illegible]	25947	6E+06	25732	p10294	Proteasome subunit alpha type-7
R3HDM2 pS855	7E+08	9E+08	[illegible]	1E+08	2E+08	2E+08	p06221	R3H domain-containing protein 2
AHNAK pS5731 pS5739	2E+09	2E+09	[illegible]	1E+09	7E+08	7E+08	p15820	Neuroblast differentiation-associated protein AHNAK
PRRC2A pS1147	1E+08	2E+08	[illegible]	9E+07	2E+08	2E+08	p17670	Protein PRRC2A
EPB41 seq: 82 - 88 + Phospho (ST)	1E+07	3E+07	[illegible]	3E+07	7E+07	5E+07	p11855	Protein 4.1
FAM53B pS167	6E+08	6E+08	[illegible]	2E+08	2E+08	2E+08	p03710	Protein FAM53B
LARP1 seq: 66 - 113 + 2 Phospho (ST)	2E+08	37149	[illegible]	4E+08	30272	25732	p17671	La-related protein 1
CAD seq: 1855 - 1869 + Phospho (ST)	1E+09	8E+08	[illegible]	2E+09	1E+09	1E+09	p14206	CAD protein
ATXN2 seq: 876 - 896 + Phospho (ST)	2E+08	1E+08	[illegible]	2E+08	9E+07	1E+08	p07376	Ataxin-2
TRIOBP pS1955	4E+08	2E+08	[illegible]	3E+08	5E+08	5E+08	p08848	TRIO and F-actin-binding protein
NUPR1 pS58	8E+08	9E+08	[illegible]	3E+06	30272	25732	p18026	Nuclear protein 1
TKT seq: 319 - 327 no mod	1E+08	2E+08	[illegible]	3E+07	30272	3E+06	p13360	Transketolase
DCLK1 seq: 361 - 389 + Phospho (ST)	3E+08	2E+08	[illegible]	1E+07	30272	25732	p01712	Serine/threonine-protein kinase DCLK1
RPLP0 seq: 67 - 77 no mod	9E+07	8E+07	[illegible]	5E+07	1E+07	1E+07	p15930	60S acidic ribosomal protein P0
NAP1L1 pT62	1E+08	3E+08	[illegible]	7E+07	9E+07	9E+07	p12084	Nucleosome assembly protein 1-like 1
SRPK2 seq: 492 - 503 + Phospho (ST)	9E+07	1E+08	[illegible]	6E+07	6E+07	5E+07	p01918	SRSF protein kinase 2
FOXP1 pS83	1E+09	2E+09	[illegible]	25947	1E+07	8E+06	p08356	Forkhead box protein P1
HSPB1 seq: 113 - 123 no mod	1E+08	1E+08	[illegible]	25947	30272	25732	p02674	Heat shock protein beta-1
ATP6V1B1 seq: 455 - 465 no mod	2E+08	8E+07	[illegible]	25947	3E+07	1E+07	p09641	V-type proton ATPase subunit B, kidney isoform
GNS seq: 410 - 421 no mod	2E+08	8E+07	[illegible]	25947	3E+07	1E+07	p06065	N-acetylglucosamine-6-sulfatase
DPYSL2 pS542	1E+08	3E+08	[illegible]	2E+08	2E+08	1E+08	p09455	Dihydropyrimidinase-related protein 2
FAM83H pS914	3E+09	2E+09	[illegible]	4E+08	3E+08	4E+08	p07322	Protein FAM83H
FOXK1 pS416 pM427	1E+08	6E+08	[illegible]	8E+08	6E+08	5E+08	p06298	Forkhead box protein K1
ERBB2 pS1078 pS1083	38324	5E+07	[illegible]	25947	30272	25732	p04569	Receptor tyrosine-protein kinase erbB-2
CTTN seq: 397 - 428 + Phospho (ST)	2E+09	3E+09	[illegible]	1E+09	3E+09	2E+09	p20346	Src substrate cortactin
SLAIN2 pS391	3E+08	5E+08	[illegible]	25947	2E+07	25732	p10746	SLAIN motif-containing protein 2
TNKS1BP1 seq: 1154 - 1168 + Phospho (ST)	2E+07	2E+07	[illegible]	3E+07	5E+07	2E+07	p02060	182 kDa tankyrase-1-binding protein
SOWAHC seq: 207 - 218 + Oxidation (M); Phospho (ST)	4E+08	4E+08	[illegible]	2E+08	1E+08	2E+08	p18513	Ankyrin repeat domain-containing protein SOWAHC
TRIP11 seq: 404 - 419 no mod	1E+08	9E+07	[illegible]	5E+07	1E+08	4E+07	p10660	Thyroid receptor-interacting protein 11
GAPDH pM105	4E+08	5E+08	[illegible]	2E+08	1E+08	5E+07	p00499	Glyceraldehyde-3-phosphate dehydrogenase
ARHGEF10 seq: 377 - 386 + Phospho (ST)	6E+08	5E+08	[illegible]	3E+07	5E+07	7E+07	p02653	Rho guanine nucleotide exchange factor 10
PRDX6 seq: 175 - 182 no mod	7E+07	8E+07	[illegible]	4E+07	30272	25732	p01743	Peroxiredoxin-6
STARD10 seq: 164 - 179 + Phospho (ST)	7E+06	2E+07	[illegible]	8E+06	3E+07	2E+07	p19192	PCTP-like protein
YWHAQ seq: 140 - 158 no mod	8E+07	1E+08	[illegible]	5E+07	4E+07	7E+07	p07805	14-3-3 protein theta
TFRC seq: 7 - 22 + Phospho (ST)	2E+08	1E+08	[illegible]	4E+08	5E+08	5E+08	p06349	Transferrin receptor protein 1
MLPH seq: 241 - 269 + Phospho (ST)	5E+08	3E+08	[illegible]	4E+07	4E+07	2E+07	p20837	Melanophilin
NEK4 pS563	4E+08	4E+08	[illegible]	9E+07	8E+07	6E+07	p18621	Serine/threonine-protein kinase Nek4
LMO7 pS1586	2E+08	2E+08	[illegible]	6E+08	6E+08	6E+08	p02071	LIM domain only protein 7
C6orf132 pS736	5E+08	4E+08	[illegible]	1E+08	4E+07	3E+07	p16163	Uncharacterized protein C6orf132
ADIRF seq: 49 - 71 no mod	1E+09	1E+09	[illegible]	1E+08	30272	4E+07	p02030	Adipogenesis regulatory factor
HIST1H1C seq: 55 - 63 no mod	2E+07	2E+08	[illegible]	6E+07	5E+07	5E+07	p05618	Histone H1.2
ZNF646 seq: 1359 - 1365 no mod	2E+07	2E+08	[illegible]	6E+07	5E+07	5E+07	p04075	Zinc finger protein 646
SNAPIN pM123 pS133	8E+08	1E+09	[illegible]	5E+08	5E+08	3E+08	p20085	SNARE-associated protein Snapin
KRT18 seq: 383 - 407 + Phospho (ST)	8E+08	9E+08	[illegible]	9E+08	7E+08	6E+08	p11431	Keratin, type I cytoskeletal 18
TBC1D10B pS687	2E+08	6E+08	[illegible]	25947	9E+07	5E+07	p07597	TBC1 domain family member 10B
ZYX seq: 273 - 295 + Phospho (ST)	1E+07	5E+07	[illegible]	25947	1E+08	1E+08	p16416	Zyxin
ZZZ3 pS113	3E+07	3E+07	[illegible]	8E+07	8E+07	7E+07	p06775	ZZ-type zinc finger-containing protein 3
TCF20 pS871	4E+07	6E+07	[illegible]	3E+08	3E+08	3E+08	p03338	Transcription factor 20
DYNC1LI2 seq: 440 - 457 + Phospho (ST)	3E+08	3E+08	[illegible]	2E+08	2E+08	2E+08	p12493	Cytoplasmic dynein 1 light intermediate chain 2
PDS5B seq: 1172 - 1197 + Phospho (ST)	3E+07	3E+08	[illegible]	3E+07	2E+08	1E+08	p15346	Sister chromatid cohesion protein PDS5 homolog B
GSN seq: 374 - 390 + Gln->pyro-Glu (N-term Q)	1E+08	7E+07	[illegible]	25947	30272	25732	p07770	Gelsolin
RPA1 seq: 168 - 183 no mod	4E+07	2E+08	[illegible]	3E+08	1E+07	2E+07	p21139	Replication protein A 70 kDa DNA-binding subunit
EIF3J pS11 pS13	5E+08	3E+08	[illegible]	2E+09	2E+09	2E+09	p21212	Eukaryotic translation initiation factor 3 subunit J
FLNA pS2615	4E+06	37149	[illegible]	3E+07	4E+07	4E+07	p10287	Filamin-A
RAB11FIP1 seq: 183 - 211 + Phospho (ST)	2E+08	2E+08	[illegible]	4E+07	30272	25732	p18280	Rab11 family-interacting protein 1
MTMR1 pS39 pS43	4E+08	2E+08	[illegible]	3E+07	6E+07	4E+07	p21147	Myotubularin-related protein 1
FAM83H pS903	3E+07	6E+07	[illegible]	2E+07	2E+07	25732	p15191	Protein FAM83H
PHLDA2 seq: 117 - 150 + Phospho (ST)	4E+08	3E+08	[illegible]	1E+09	2E+09	1E+09	p03069	Pleckstrin homology-like domain family A member 2
DDX6 seq: 23 - 46 no mod	2E+08	3E+08	[illegible]	5E+07	5E+07	8E+07	p15419	Probable ATP-dependent RNA helicase DDX6
ARPP19 pS104	8E+08	1E+09	[illegible]	3E+08	4E+08	6E+08	p08336	cAMP-regulated phosphoprotein 19
MISP seq: 154 - 171 + Phospho (ST)	3E+09	3E+09	[illegible]	9E+08	1E+09	2E+09	p12525	Mitotic interactor and substrate of PLK1
CCDC92 seq: 209 - 223 + Phospho (ST)	38324	5E+07	[illegible]	3E+07	6E+07	3E+07	p05072	Coiled-coil domain-containing protein 92
ZNF516 seq: 928 - 938 + Phospho (ST)	1E+08	2E+08	[illegible]	25947	2E+07	4E+07	p06196	Zinc finger protein 516
TOP2B seq: 84 - 96 no mod	38324	7E+07	[illegible]	2E+07	2E+07	3E+07	p14316	DNA topoisomerase 2-beta
CDCA3 pS209	6E+06	3E+07	[illegible]	4E+07	3E+07	3E+07	p04535	Cell division cycle-associated protein 3
NPM1 pS125	9E+08	2E+09	[illegible]	1E+09	2E+10	6E+09	p11523	Nucleophosmin
LMNA pS301	3E+07	37149	[illegible]	1E+08	2E+08	9E+07	p14396	Prelamin-A/C
EPN3 pS264	1E+09	6E+08	[illegible]	25947	3E+07	1E+07	p03182	Epsin-3
PLEKHF2 pS16	1E+08	5E+08	[illegible]	25947	2E+08	25732	p07251	Pleckstrin homology domain-containing family F member 2
R3HDM2 pT860	1E+08	1E+08	[illegible]	8E+06	2E+07	2E+07	p06220	R3H domain-containing protein 2
PLEKHA7 seq: 554 - 562 + Phospho (ST)	4E+07	6E+07	[illegible]	25947	2E+07	4E+07	p04042	Pleckstrin homology domain-containing family A member 7
SIPA1L3 seq: 1418 - 1443 + Phospho (ST)	8E+07	1E+08	[illegible]	2E+08	3E+08	2E+08	p03055	Signal-induced proliferation-associated 1-like protein 3
FOXK1 pS416 pM427	7E+08	2E+09	[illegible]	1E+09	1E+09	1E+09	p06297	Forkhead box protein K1
ASAP1 seq: 837 - 852 + Phospho (ST)	38324	3E+07	[illegible]	3E+08	2E+08	2E+08	p02561	Arf-GAP with SH3 domain, ANK repeat and PH domain-containing protein 1
GPS1 seq: 462 - 486 + Phospho (ST)	2E+08	3E+08	[illegible]	5E+07	1E+08	1E+08	p09230	COP9 signalosome complex subunit 1
DNMT1 pS127	1E+08	1E+09	[illegible]	1E+08	7E+08	6E+08	p06466	DNA (cytosine-5)-methyltransferase 1
NDRG3 pS333 pS334	8E+07	6E+07	[illegible]	5E+07	4E+07	6E+07	p02747	Protein NDRG3
KLC2 seq: 580 - 605 + 2 Phospho (ST)	2E+08	2E+08	[illegible]	8E+07	6E+07	4E+07	p19489	Kinesin light chain 2
CCDC39 pT426	4E+07	6E+07	[illegible]	25947	2E+07	4E+07	p19224	Coiled-coil domain-containing protein 39
ZNF217 seq: 792 - 819 + Phospho (ST)	6E+07	3E+08	[illegible]	2E+07	4E+07	5E+07	p15803	Zinc finger protein 217
C2orf44 pS468	2E+07	1E+07	[illegible]	5E+08	4E+08	4E+08	p11602	WD repeat and coiled-coil-containing protein C2orf44
CCDC120 pS280	3E+08	3E+08	[illegible]	2E+07	3E+07	3E+07	p07000	Coiled-coil domain-containing protein 120
NEDD4L seq: 446 - 468 + Phospho (ST)	2E+07	9E+07	[illegible]	2E+08	3E+08	3E+08	p04984	E3 ubiquitin-protein ligase NEDD4-like
MTFR1L pS41	3E+07	6E+07	[illegible]	2E+07	5E+06	1E+07	p19623	Mitochondrial fission regulator 1-like
ARPC1B seq: 308 - 326 + Phospho (ST)	5E+07	3E+07	[illegible]	2E+07	2E+07	2E+07	p13380	Actin-related protein 2/3 complex subunit 1B
COG2 seq: 476 - 483 + Phospho (ST)	3E+08	3E+08	[illegible]	3E+08	1E+08	3E+08	p12770	Conserved oligomeric Golgi complex subunit 2
PPP1R13L pM391 pS395	5E+08	6E+08	[illegible]	1E+08	2E+08	2E+08	p20083	RelA-associated inhibitor
SH3RF1 pS127	9E+07	1E+08	[illegible]	1E+08	1E+08	1E+08	p00921	E3 ubiquitin-protein ligase SH3RF1
ENO1 seq: 81 - 92 no mod	4E+08	5E+08	[illegible]	4E+08	7E+08	4E+08	p12950	Alpha-enolase
CRABP2 seq: 32 - 45 no mod	3E+07	7E+07	[illegible]	25947	30272	25732	p14460	Cellular retinoic acid-binding protein 2
NDRG1 seq: 328 - 341 + Phospho (ST)	2E+09	2E+09	[illegible]	2E+09	2E+09	1E+09	p03033	Protein NDRG1
LASP1 pT104	3E+08	3E+08	[illegible]	9E+07	5E+07	4E+07	p16089	LIM and SH3 domain protein 1
NEDD4L pS448	3E+08	4E+08	[illegible]	4E+08	6E+08	6E+08	p04985	E3 ubiquitin-protein ligase NEDD4-like
NOC2L pS49	9E+07	2E+08	[illegible]	2E+08	2E+08	25732	p18011	Nucleolar complex protein 2 homolog
AKIRIN1 pS22	38324	4E+07	[illegible]	1E+08	1E+08	1E+08	p06901	Akirin-1
PLEKHA7 seq: 534 - 553 + Phospho (ST)	1E+08	1E+08	[illegible]	6E+07	8E+07	7E+07	p14784	Pleckstrin homology domain-containing family A member 7
TNKS1BP1 seq: 824 - 847 + Phospho (ST)	2E+08	4E+08	[illegible]	2E+08	7E+08	5E+08	p00623	182 kDa tankyrase-1-binding protein
NAV1 seq: 450 - 457 + Phospho (ST)	1E+09	2E+09	[illegible]	8E+07	8E+07	1E+08	p07006	Neuron navigator 1
ZNF428 pS99	6E+08	4E+08	[illegible]	2E+08	2E+08	2E+08	p04562	Zinc finger protein 428
PDIA3 pM434	2E+07	3E+07	[illegible]	2E+08	2E+08	2E+08	p10222	Protein disulfide-isomerase A3
TP53BP1 pS1430	1E+09	1E+09	[illegible]	5E+08	4E+08	5E+08	p17077	Tumor suppressor p53-binding protein 1
MEPCE pS152	2E+08	1E+08	[illegible]	4E+08	3E+08	3E+08	p07029	7SK snRNA methylphosphate capping enzyme
EFHD2 pS74	3E+09	4E+09	[illegible]	1E+10	8E+09	9E+09	p07647	EF-hand domain-containing protein D2
HSPB1 pS82	1E+11	1E+11	[illegible]	3E+07	2E+07	2E+07	p08280	Heat shock protein beta-1
ARHGEF10 seq: 371 - 376 + Phospho (ST)	4E+08	6E+08	[illegible]	9E+07	1E+08	2E+08	p15326	Rho guanine nucleotide exchange factor 10
FBRSL1 pT1010	2E+08	2E+08	[illegible]	8E+07	1E+08	1E+08	p02362	Fibrosin-1-like protein
CASKIN2 seq: 251 - 271 + Phospho (ST)	4E+07	6E+07	[illegible]	25947	30272	25732	p08994	Caskin-2
TUBA3C seq: 215 - 229 no mod	5E+07	3E+08	[illegible]	1E+08	3E+08	25732	p07031	Tubulin alpha-3C/D chain
PLEKHA7 pS612	8E+07	1E+08	[illegible]	3E+07	3E+07	4E+07	p03378	Pleckstrin homology domain-containing family A member 7
DBNL pM268 pS269	1E+10	1E+10	[illegible]	2E+09	2E+09	2E+09	p20071	Drebrin-like protein
PAGR1 pS237	6E+09	5E+09	[illegible]	2E+09	2E+09	2E+09	p18613	PAXIP1-associated glutamate-rich protein 1
EIF3A seq: 877 - 885 + Phospho (ST)	2E+09	2E+09	[illegible]	2E+09	1E+09	1E+09	p07179	Eukaryotic translation initiation factor 3 subunit A
TMSB10 seq: 27 - 40 no mod	8E+07	9E+07	[illegible]	2E+08	3E+08	2E+08	p09021	Thymosin beta-10
USP6NL pS680	5E+08	6E+08	[illegible]	4E+07	7E+07	1E+07	p06928	USP6 N-terminal-like protein
EPS8L1 seq: 549 - 572 + Phospho (ST)	3E+09	3E+09	[illegible]	9E+07	2E+08	2E+08	p05133	Epidermal growth factor receptor kinase substrate 8-like protein 1
PLCH1 pS970	2E+07	2E+07	[illegible]	25947	7E+06	1E+07	p05090	1-phosphatidylinositol 4,5-bisphosphate phosphodiesterase eta-1
FASN seq: 958 - 967 no mod	8E+07	8E+07	[illegible]	25947	4E+06	4E+06	p00523	Fatty acid synthase
CA2 seq: 28 - 39 + Gln->pyro-Glu (N-term Q)	8E+06	2E+07	[illegible]	1E+07	30272	25732	p07859	Carbonic anhydrase 2
LRBA seq: 1244 - 1271 + Phospho (ST)	1E+08	2E+08	[illegible]	8E+07	4E+07	25732	p12023	Lipopolysaccharide-responsive and beige-like anchor protein
USP36 seq: 609 - 631 + Phospho (ST)	2E+08	3E+08	[illegible]	1E+08	3E+08	2E+08	p15158	Ubiquitin carboxyl-terminal hydrolase 36
ATN1 seq: 73 - 88 + Phospho (ST)	2E+07	8E+06	[illegible]	3E+08	1E+08	1E+08	p06043	Atrophin-1
THRAP3 pS575	2E+07	1E+08	[illegible]	5E+07	1E+08	7E+07	p10197	Thyroid hormone receptor-associated protein 3
AFAP1 seq: 276 - 299 + Phospho (ST)	7E+07	3E+07	[illegible]	9E+07	30272	25732	p10669	Actin filament-associated protein 1
MKL2 seq: 157 - 205 + Phospho (ST)	2E+08	2E+08	[illegible]	25947	30272	25732	p17956	MKL/myocardin-like protein 2

SHANK2 seq: 719 - 741 + Phospho (ST)	[illegible]	p09126	SH3 and multiple ankyrin repeat domains protein 2
NUP210 seq: 1872 - 1887 + Phospho (ST)	[illegible]	p13382	Nuclear pore membrane glycoprotein 210
NDRG3 seq: 327 - 345 + Phospho (ST)	[illegible]	p03984	Protein NDRG3
HNRNPC seq: 188 - 198 no mod	[illegible]	p11509	Heterogeneous nuclear ribonucleoproteins C1/C2
SGK223 seq: 138 - 154 + Phospho (ST)	[illegible]	p14999	Tyrosine-protein kinase SgK223
MPRIP seq: 346 - 368 + Phospho (ST)	[illegible]	p18852	Myosin phosphatase Rho-interacting protein
SCRIB pS853	[illegible]	p16052	Protein scribble homolog
MYC seq: 2 - 10 + Phospho (ST)	[illegible]	p08888	Myc proto-oncogene protein
ANK3 pS1459	[illegible]	p07926	Ankyrin-3
YTHDF1 pS291	[illegible]	p19851	YTH domain-containing family protein 1
NOS1AP pM369 pS371 pS374	[illegible]	p10785	Carboxyl-terminal PDZ ligand of neuronal nitric oxide synthase protein
MAP7 pS209	[illegible]	p10627	Ensconsin
FAM83H pS914	[illegible]	p15188	Protein FAM83H
MISP pS394 pS400	[illegible]	p20158	Mitotic interactor and substrate of PLK1
VCP seq: 363 - 377 no mod	[illegible]	p16809	Transitional endoplasmic reticulum ATPase
CDC42EP4 pS118	[illegible]	p09761	Cdc42 effector protein 4
MISP seq: 345 - 353 + Phospho (Y)	[illegible]	p15330	Mitotic interactor and substrate of PLK1
NELFA seq: 374 - 428 + Phospho (ST)	[illegible]	p19961	Negative elongation factor A
ANXA3 seq: 139 - 153 no mod	[illegible]	p05233	Annexin A3
FAM83H pS914	[illegible]	p07323	Protein FAM83H
TAT pS448	[illegible]	p13831	Tyrosine aminotransferase
CTTN seq: 397 - 428 + Phospho (ST)	[illegible]	p20347	Src substrate cortactin
seq: 773 - 789 + Gln->pyro-Glu (N-term Q); Phospho (ST)	[illegible]	p08230	Probable 28S rRNA (cytosine(4447)-C(5))-methyltransferase
HSPH1 seq: 715 - 722 no mod	[illegible]	p10146	Heat shock protein 105 kDa
RPS21 seq: 52 - 60 no mod	[illegible]	p01872	40S ribosomal protein S21
MSH4 seq: 895 - 900 + Phospho (ST)	[illegible]	p10364	MutS protein homolog 4
PRPF6 seq: 756 - 761 + Phospho (ST)	[illegible]	p14244	Pre-mRNA-processing factor 6
ENAH pS531	[illegible]	p03172	Protein enabled homolog
NONO seq: 399 - 434 + Oxidation (M); Phospho (ST)	[illegible]	p16287	Non-POU domain-containing octamer-binding protein
RPL14 pS139	[illegible]	p21095	60S ribosomal protein L14
PCNA pM139	[illegible]	p09950	Proliferating cell nuclear antigen
SMTN seq: 30 - 35 + Phospho (ST)	[illegible]	p13769	Smoothelin
LEO1 seq: 543 - 548 + Phospho (ST)	[illegible]	p10911	RNA polymerase-associated protein LEO1
TMEM132A pS1495	[illegible]	p10910	Transmembrane protein 132A
NF1 seq: 1137 - 1142 + Phospho (ST)	[illegible]	p07195	Neurofibromin
PTPN23 seq: 1113 - 1152 + Phospho (ST)	[illegible]	p16357	Tyrosine-protein phosphatase non-receptor type 23
PRR36 pS110	[illegible]	p09001	Proline-rich protein 36
C6orf132 pS558	[illegible]	p05223	Uncharacterized protein C6orf132
KIAA1522 seq: 403 - 420 + Phospho (ST)	[illegible]	p15210	Uncharacterized protein KIAA1522
TUBA1B seq: 157 - 164 no mod	[illegible]	p10607	Tubulin alpha-1B chain
PPP1R13L seq: 577 - 608 + Oxidation (M); Phospho (ST)	[illegible]	p20600	RelA-associated inhibitor
CAP1 seq: 367 - 376 no mod	[illegible]	p13895	Adenylyl cyclase-associated protein 1
PDCD6IP seq: 708 - 745 + Phospho (ST)	[illegible]	p18551	Programmed cell death 6-interacting protein
GPRIN1 pS615	[illegible]	p01446	G protein-regulated inducer of neurite outgrowth 1
SARG seq: 451 - 475 + Phospho (ST)	[illegible]	p20037	Specifically androgen-regulated gene protein
ARID4B pS675	[illegible]	p07132	AT-rich interactive domain-containing protein 4B
THRAP3 pM573 pS575	[illegible]	p10198	Thyroid hormone receptor-associated protein 3
SH3RF2 pS649	[illegible]	p16514	Putative E3 ubiquitin-protein ligase SH3RF2
TTN seq: 20222 - 20229 no mod	[illegible]	p14405	Titin
ANKLE2 pS914	[illegible]	p09050	Ankyrin repeat and LEM domain-containing protein 2
SRRM2 seq: 1539 - 1556 + Phospho (ST)	[illegible]	p05653	Serine/arginine repetitive matrix protein 2
AMOTL2 pM178 pS183	[illegible]	p19919	Angiomotin-like protein 2
SEC13 seq: 217 - 239 no mod	[illegible]	p18129	Protein SEC13 homolog
ANKHD1 seq: 1675 - 1682 + Phospho (ST)	[illegible]	p11179	Ankyrin repeat and KH domain-containing protein 1
1 seq: 32 - 43 + Gln->pyro-Glu (N-term Q); Phospho (ST)	[illegible]	p07783	KH domain-containing, RNA-binding, signal transduction-associated protein 1
DCLK1 pS172	[illegible]	p19175	Serine/threonine-protein kinase DCLK1
PLEKHG3 pS1037	[illegible]	p04541	Pleckstrin homology domain-containing family G member 3
ADD3 seq: 664 - 684 + 2 Phospho (ST)	[illegible]	p04690	Gamma-adducin
RPS6KA4 pS745	[illegible]	p07327	Ribosomal protein S6 kinase alpha-4
PFN2 seq: 92 - 105 no mod	[illegible]	p04251	Profilin-2
TRAFD1 pS491	[illegible]	p10721	TRAF-type zinc finger domain-containing protein 1
PRIMPOL pS255	[illegible]	p11706	DNA-directed primase/polymerase protein
FLNB pS2465	[illegible]	p00372	Filamin-B
BAIAP2L1 pS281	[illegible]	p18059	Brain-specific angiogenesis inhibitor 1-associated protein 2-like protein 1
DBNL pT291	[illegible]	p08265	Drebrin-like protein
TNKS1BP1 seq: 1708 - 1723 + Phospho (ST)	[illegible]	p03884	182 kDa tankyrase-1-binding protein
EIF4ENIF1 seq: 453 - 473 no mod	[illegible]	p09056	Eukaryotic translation initiation factor 4E transporter
TPI1 seq: 232 - 243 no mod	[illegible]	p04739	Triosephosphate isomerase
PKP3 pS123	[illegible]	p06181	Plakophilin-3
TFRC seq: 7 - 22 + Phospho (Y)	[illegible]	p06348	Transferrin receptor protein 1
RGS3 seq: 940 - 952 + Phospho (ST)	[illegible]	p06525	Regulator of G-protein signaling 3
WNK1 seq: 1996 - 2021 + 2 Phospho (ST)	[illegible]	p13290	Serine/threonine-protein kinase WNK1
PAN3 seq: 349 - 358 + Phospho (ST)	[illegible]	p13589	PAB-dependent poly(A)-specific ribonuclease subunit PAN3
ZC3H4 pS1275	[illegible]	p02789	Zinc finger CCCH domain-containing protein 4
JMJD1C seq: 638 - 648 + Phospho (ST)	[illegible]	p03777	Probable JmjC domain-containing histone demethylation protein 2C
ATXN2 pM615 pS624	[illegible]	p09876	Ataxin-2
in ENSP00000471857 OS=Homo sapiens PE=2 SV=1 pS36	[illegible]	p13848	Uncharacterized protein ENSP00000471857
SPECC1L seq: 954 - 991 + Phospho (ST)	[illegible]	p18729	Cytospin-A
MACF1 seq: 7269 - 7299 + 2 Phospho (ST)	[illegible]	p11492	Microtubule-actin cross-linking factor 1, isoforms 1/2/3/5
R3HDM2 pS349	[illegible]	p19503	R3H domain-containing protein 2
PEBP1 seq: 48 - 62 no mod	[illegible]	p09185	Phosphatidylethanolamine-binding protein 1
AHNAK seq: 5780 - 5814 + Phospho (ST)	[illegible]	p04802	Neuroblast differentiation-associated protein AHNAK
EIF3A pS1188	[illegible]	p19719	Eukaryotic translation initiation factor 3 subunit A
AHNAK pS5031	[illegible]	p14208	Neuroblast differentiation-associated protein AHNAK
HDGF pS165	[illegible]	p07634	Hepatoma-derived growth factor
C12orf45 seq: 9 - 27 + Phospho (ST)	[illegible]	p19534	Uncharacterized protein C12orf45
C2CD5 pS852	[illegible]	p20066	C2 domain-containing protein 5
SGK223 pS653	[illegible]	p14365	Tyrosine-protein kinase SgK223
ARPP19 pS104	[illegible]	p12729	cAMP-regulated phosphoprotein 19
ATXN2 seq: 876 - 893 + Phospho (ST)	[illegible]	p07377	Ataxin-2
CCDC88C pS1806	[illegible]	p20746	Protein Daple
EVL pS369	[illegible]	p10049	Ena/VASP-like protein
TEX2 seq: 220 - 241 + Phospho (ST)	[illegible]	p04977	Testis-expressed sequence 2 protein
NIFK seq: 217 - 244 + Phospho (ST)	[illegible]	p00853	MKI67 FHA domain-interacting nucleolar phosphoprotein
FASN seq: 1935 - 1953 no mod	[illegible]	p08581	Fatty acid synthase
HIST1H1B pS18	[illegible]	p05964	Histone H1.5
HEBP2 seq: 141 - 150 no mod	[illegible]	p05932	Heme-binding protein 2
TBC1D5 seq: 550 - 562 + Phospho (ST)	[illegible]	p17780	TBC1 domain family member 5
TMSB4X seq: 20 - 32 no mod	[illegible]	p12502	Thymosin beta-4
GAPVD1 seq: 755 - 786 + Phospho (ST)	[illegible]	p16947	GTPase-activating protein and VPS9 domain-containing protein 1
RANBP10 pS365	[illegible]	p04277	Ran-binding protein 10
SGK223 seq: 138 - 154 + Phospho (ST)	[illegible]	p15000	Tyrosine-protein kinase SgK223
MAP1B seq: 1433 - 1461 + 2 Phospho (ST)	[illegible]	p05735	Microtubule-associated protein 1B
FRMD4B seq: 768 - 785 + Phospho (ST)	[illegible]	p12667	FERM domain-containing protein 4B
HSPD1 seq: 345 - 359 no mod	[illegible]	p01495	60 kDa heat shock protein, mitochondrial
CALR seq: 56 - 73 no mod	[illegible]	p16366	Calreticulin
ATXN2L seq: 554 - 570 + Phospho (ST)	[illegible]	p10957	Ataxin-2-like protein
C1orf226 seq: 256 - 272 + Phospho (ST)	[illegible]	p02103	Uncharacterized protein C1orf226
KIAA1671 seq: 390 - 406 + Phospho (ST)	[illegible]	p20397	Uncharacterized protein KIAA1671
SLC38A1 seq: 51 - 60 + Phospho (ST)	[illegible]	p06659	Sodium-coupled neutral amino acid transporter 1
SKA3 seq: 152 - 165 + Phospho (ST)	[illegible]	p04439	Spindle and kinetochore-associated protein 3
MAGI3 seq: 1469 - 1479 + Phospho (ST)	[illegible]	p16644	Membrane-associated guanylate kinase, WW and PDZ domain-containing protein 3
FILIP1L pS1035	[illegible]	p14900	Filamin A-interacting protein 1-like
GSDMD seq: 250 - 267 + Oxidation (M); Phospho (ST)	[illegible]	p03468	Gasdermin-D
DGCR14 seq: 378 - 388 + Phospho (ST)	[illegible]	p00733	Protein DGCR14
EPN3 seq: 524 - 553 + Phospho (ST)	[illegible]	p09273	Epsin-3
MTSS1L pS456	[illegible]	p15643	MTSS1-like protein
CCDC102A pS28	[illegible]	p15247	Coiled-coil domain-containing protein 102A
TAT pS448	[illegible]	p13832	Tyrosine aminotransferase
MRGBP seq: 182 - 201 + Phospho (ST)	[illegible]	p00736	MRG/MORF4L-binding protein
DLGAP4 pS763	[illegible]	p09398	Disks large-associated protein 4
MAP1B seq: 539 - 555 + Phospho (ST)	[illegible]	p20889	Microtubule-associated protein 1B
ABCF1 seq: 7 - 30 + Phospho (ST)	[illegible]	p08041	ATP-binding cassette sub-family F member 1
SRSF4 seq: 446 - 459 + Phospho (ST)	[illegible]	p04786	Serine/arginine-rich splicing factor 4
NOP2 seq: 773 - 789 + Phospho (ST)	[illegible]	p08229	Probable 28S rRNA (cytosine(4447)-C(5))-methyltransferase
CDC42EP4 pS118	[illegible]	p09762	Cdc42 effector protein 4
AHCTF1 seq: 1209 - 1225 + 2 Phospho (ST)	[illegible]	p03484	Protein ELYS
SRRT seq: 527 - 559 + Phospho (ST)	[illegible]	p11593	Serrate RNA effector molecule homolog
CAP1 seq: 114 - 119 no mod	[illegible]	p16979	Adenylyl cyclase-associated protein 1
GAB2 seq: 140 - 154 + Phospho (ST)	[illegible]	p03827	GRB2-associated-binding protein 2
BTF3 pS30	[illegible]	p19714	Transcription factor BTF3
SYNRG pS935	[illegible]	p13265	Synergin gamma
FOXK1 pS416	[illegible]	p06295	Forkhead box protein K1
C11orf86 seq: 34 - 39 + Phospho (ST)	[illegible]	p07629	Uncharacterized protein C11orf86
SFMBT2 seq: 73 - 78 + Phospho (ST)	[illegible]	p07539	Scm-like with four MBT domains protein 2
BCL9L pS750 pM753	[illegible]	p15696	B-cell CLL/lymphoma 9-like protein
ANKS1A seq: 622 - 644 + Phospho (ST)	[illegible]	p05050	Ankyrin repeat and SAM domain-containing protein 1A
UBAP1 seq: 283 - 294 + Phospho (ST)	[illegible]	p12175	Ubiquitin-associated protein 1
GIGYF2 pS1284	[illegible]	p20902	PERQ amino acid-rich with GYF domain-containing protein 2
PSMA7 seq: 205 - 212 no mod	[illegible]	p09568	Proteasome subunit alpha type-7
LARP4B seq: 711 - 724 + Phospho (ST)	[illegible]	p06977	La-related protein 4B
HDGF pS165	[illegible]	p07635	Hepatoma-derived growth factor
C9orf40 pS69	[illegible]	p13115	Uncharacterized protein C9orf40
MAP4 pS1073	[illegible]	p01448	Microtubule-associated protein 4
FBP1 seq: 315 - 330 no mod	[illegible]	p19861	Fructose-1,6-bisphosphatase 1
CCDC86 pS58	[illegible]	p20635	Coiled-coil domain-containing protein 86
MTMR1 seq: 31 - 59 + 2 Phospho (ST)	[illegible]	p21146	Myotubularin-related protein 1
CDC42EP4 seq: 18 - 35 + Phospho (ST)	[illegible]	p04142	Cdc42 effector protein 4
KCTD15 seq: 35 - 53 + Phospho (ST)	[illegible]	p04301	BTB/POZ domain-containing protein KCTD15
UBR3 seq: 1046 - 1064 + Phospho (ST)	[illegible]	p16520	E3 ubiquitin-protein ligase UBR3
MAP7D1 seq: 111 - 133 + Phospho (ST)	[illegible]	p06578	MAP7 domain-containing protein 1
HUWE1 seq: 2885 - 2903 + Phospho (ST)	[illegible]	p20552	E3 ubiquitin-protein ligase HUWE1
ARFGAP2 seq: 314 - 336 + Phospho (ST)	[illegible]	p03570	ADP-ribosylation factor GTPase-activating protein 2
MLLT4 seq: 1168 - 1186 + 2 Phospho (ST)	[illegible]	p20915	Afadin
AKAP11 seq: 419 - 438 + 2 Phospho (ST)	[illegible]	p12799	A-kinase anchor protein 11
TANC2 seq: 1720 - 1732 + Phospho (ST)	[illegible]	p04216	Protein TANC2
WDR20 seq: 432 - 446 + Phospho (ST)	[illegible]	p04783	WD repeat-containing protein 20
NAP1L1 pT62	[illegible]	p12085	Nucleosome assembly protein 1-like 1
CUL4B seq: 134 - 158 + Phospho (ST)	[illegible]	p10275	Cullin-4B
WWTR1 seq: 87 - 114 + 2 Phospho (ST)	[illegible]	p05559	WW domain-containing transcription regulator protein 1
MLPH seq: 241 - 269 + Phospho (ST)	[illegible]	p20836	Melanophilin
ERBB2 seq: 1054 - 1096 + Phospho (ST)	[illegible]	p05758	Receptor tyrosine-protein kinase erbB-2
seq: 488 - 508 + Gln->pyro-Glu (N-term Q); Phospho (ST)	[illegible]	p07968	Brain-specific angiogenesis inhibitor 1-associated protein 2
SHANK2 seq: 718 - 741 + 2 Phospho (ST)	[illegible]	p07018	SH3 and multiple ankyrin repeat domains protein 2
TCP1 seq: 539 - 556 no mod	[illegible]	p18912	T-complex protein 1 subunit alpha
GIGYF2 seq: 176 - 192 + Phospho (ST)	[illegible]	p18114	PERQ amino acid-rich with GYF domain-containing protein 2
USP39 pS82	[illegible]	p17016	U4/U6.U5 tri-snRNP-associated protein 2
MISP seq: 391 - 406 + 2 Phospho (ST)	[illegible]	p07626	Mitotic interactor and substrate of PLK1
AHNAK seq: 4993 - 5017 + Phospho (ST)	[illegible]	p04419	Neuroblast differentiation-associated protein AHNAK
S=Homo sapiens PE=2 SV=1 seq: 43 - 52 + Phospho (ST)	[illegible]	p08859	Uncharacterized protein ENSP00000471857
RBMX pS88	[illegible]	p20464	RNA-binding motif protein, X chromosome
RPS17 pS113 pM126	[illegible]	p11455	40S ribosomal protein S17
MEPCE pS60 pS69	[illegible]	p19075	7SK snRNA methylphosphate capping enzyme
NUDT5 seq: 176 - 181 no mod	[illegible]	p09705	ADP-sugar pyrophosphatase
SLX4IP seq: 390 - 400 + Phospho (ST)	[illegible]	p10616	Protein SLX4IP
UPF1 seq: 1117 - 1129 + Phospho (ST)	[illegible]	p19116	Regulator of nonsense transcripts 1
GAPDH seq: 119 - 145 no mod	[illegible]	p01378	Glyceraldehyde-3-phosphate dehydrogenase
NEDD4L seq: 340 - 362 + Phospho (ST)	[illegible]	p06130	E3 ubiquitin-protein ligase NEDD4-like
DLGAP4 pS973	[illegible]	p08193	Disks large-associated protein 4
EVPL seq: 69 - 85 no mod	[illegible]	p11163	Envoplakin
EFHD2 seq: 63 - 78 + Phospho (ST)	[illegible]	p20906	EF-hand domain-containing protein D2
EFHD2 pS74	[illegible]	p07649	EF-hand domain-containing protein D2
DYNC1LI2 seq: 440 - 458 + Phospho (ST)	[illegible]	p12492	Cytoplasmic dynein 1 light intermediate chain 2
BAIAP2L1 pS331	[illegible]	p03233	Brain-specific angiogenesis inhibitor 1-associated protein 2-like protein 1
PHF2 pS705	[illegible]	p18540	Lysine-specific demethylase PHF2
MYOF seq: 1562 - 1579 no mod	[illegible]	p15758	Myoferlin
FAM120A pS1023	[illegible]	p09246	Constitutive coactivator of PPAR-gamma-like protein 1
CAMSAP2 pS1313	[illegible]	p05968	Calmodulin-regulated spectrin-associated protein 2
EFHD2 pS74	[illegible]	p20905	EF-hand domain-containing protein D2
EFHD2 pS74	[illegible]	p07648	EF-hand domain-containing protein D2
MAPT seq: 671 - 686 no mod	[illegible]	p14238	Microtubule-associated protein tau
PRRC2B seq: 1420 - 1430 + Phospho (ST)	[illegible]	p05810	Protein PRRC2B
ARAF seq: 248 - 268 + 2 Phospho (ST)	[illegible]	p15975	Serine/threonine-protein kinase A-Raf
PRKCG seq: 497 - 517 + Phospho (ST)	[illegible]	p02874	Protein kinase C gamma type
FBP1 seq: 301 - 314 no mod	[illegible]	p17903	Fructose-1,6-bisphosphatase 1
LMO7 seq: 1006 - 1033 + Oxidation (M); Phospho (ST)	[illegible]	p19357	LIM domain only protein 7
AHNAK pS3360	[illegible]	p16564	Neuroblast differentiation-associated protein AHNAK
RBL1 pS749 pS762	[illegible]	p01294	Retinoblastoma-like protein 1
TNKS1BP1 pS1158	[illegible]	p02061	182 kDa tankyrase-1-binding protein
ATRX pS729	[illegible]	p05386	Transcriptional regulator ATRX
BARD1 pS330	[illegible]	p10648	BRCA1-associated RING domain protein 1
BAIAP2L1 pS281	[illegible]	p18060	Brain-specific angiogenesis inhibitor 1-associated protein 2-like protein 1
MINK1 pS674	[illegible]	p02104	Misshapen-like kinase 1
MTMR1 seq: 31 - 59 + Phospho (ST)	[illegible]	p21144	Myotubularin-related protein 1
SAMHD1 pT592	[illegible]	p09621	Deoxynucleoside triphosphate triphosphohydrolase SAMHD1
PDAP1 pM174 pS178	[illegible]	p09924	28 kDa heat- and acid-stable phosphoprotein
ARHGAP21 seq: 474 - 491 + Phospho (ST)	[illegible]	p06239	Rho GTPase-activating protein 21
OTUD5 seq: 506 - 520 + Phospho (ST)	[illegible]	p19318	OTU domain-containing protein 5
MGA pS1457	[illegible]	p15679	MAX gene-associated protein
AHNAK pS5731 pS5739	[illegible]	p15941	Neuroblast differentiation-associated protein AHNAK

FAM122A pS143	[illegible]	p07280	Protein FAM122A
UBAP2L seq: 460 - 484 + Oxidation (M); Phospho (ST)	[illegible]	p03475	Ubiquitin-associated protein 2-like
UBQLN4 seq: 88 - 136 + Phospho (ST)	[illegible]	p19824	Ubiquilin-4
OSBPL7 pS272	[illegible]	p11649	Oxysterol-binding protein-related protein 7
PTPN12 pS435	[illegible]	p09415	Tyrosine-protein phosphatase non-receptor type 12
CDV3 seq: 181 - 203 no mod	[illegible]	p12482	Protein CDV3 homolog
MAPRE3 seq: 151 - 167 + Phospho (ST)	[illegible]	p12968	Microtubule-associated protein RP/EB family member 3
ANKRD17 seq: 1634 - 1650 + Phospho (ST)	[illegible]	p12598	Ankyrin repeat domain-containing protein 17
MPDZ pS354	[illegible]	p03065	Multiple PDZ domain protein
AMOTL1 pS793	[illegible]	p03301	Angiomotin-like protein 1
HIST1H1B pS18	[illegible]	p05962	Histone H1.5
MED19 seq: 223 - 244 + Phospho (ST)	[illegible]	p09196	Mediator of RNA polymerase II transcription subunit 19
MAP4 pS1073	[illegible]	p01447	Microtubule-associated protein 4
PACS1 pS519	[illegible]	p08311	Phosphofurin acidic cluster sorting protein 1
ANKRD28 seq: 1009 - 1019 + Phospho (ST)	[illegible]	p01913	Serine/threonine-protein phosphatase 6 regulatory ankyrin repeat subunit A
ZNF592 pS1089	[illegible]	p01301	Zinc finger protein 592
TBC1D5 pS554	[illegible]	p17781	TBC1 domain family member 5
PTOV1 pS53	[illegible]	p19686	Prostate tumor-overexpressed gene 1 protein
PARK7 seq: 183 - 189 no mod	[illegible]	p19963	Protein deglycase DJ-1
SEPT9 pT49	[illegible]	p06449	Septin-9
HNRNPM pS528 pM529	[illegible]	p10111	Heterogeneous nuclear ribonucleoprotein M
ATAT1 pS315	[illegible]	p11371	Alpha-tubulin N-acetyltransferase 1
WDR20 seq: 432 - 446 + Phospho (ST)	[illegible]	p04782	WD repeat-containing protein 20
MEPCE pS152	[illegible]	p07028	7SK snRNA methylphosphate capping enzyme
MYO18A seq: 145 - 170 + Phospho (ST)	[illegible]	p04132	Unconventional myosin-XVIIIa
TNKS1BP1 seq: 1398 - 1442 + Phospho (Y)	[illegible]	p17482	182 kDa tankyrase-1-binding protein
MARK3 seq: 574 - 597 + Phospho (ST)	[illegible]	p03002	MAP/microtubule affinity-regulating kinase 3
BANF2 pM1	[illegible]	p10212	Barrier-to-autointegration factor-like protein
MED24 pS873	[illegible]	p11273	Mediator of RNA polymerase II transcription subunit 24
TNKS1BP1 pT1282	[illegible]	p15037	182 kDa tankyrase-1-binding protein
C7orf43 seq: 515 - 525 + Phospho (ST)	[illegible]	p04198	Uncharacterized protein C7orf43
ATXN2 pS758	[illegible]	p08178	Ataxin-2
TLE3 pS217	[illegible]	p14664	Transducin-like enhancer protein 3
PXN pS106	[illegible]	p02082	Paxillin
CASKIN2 pS858	[illegible]	p04205	Caskin-2
ARHGEF16 pS174	[illegible]	p15740	Rho guanine nucleotide exchange factor 16
VCP seq: 488 - 505 no mod	[illegible]	p17418	Transitional endoplasmic reticulum ATPase
NUFIP2 seq: 570 - 581 + Phospho (ST)	[illegible]	p06486	Nuclear fragile X mental retardation-interacting protein 2
CTNND1 seq: 600 - 622 no mod	[illegible]	p00168	Catenin delta-1
PUM2 pS587	[illegible]	p07464	Pumilio homolog 2
SAPCD2 pS276	[illegible]	p19664	Suppressor APC domain-containing protein 2
SEC22B pS137	[illegible]	p09462	Vesicle-trafficking protein SEC22b
SF3B2 seq: 771 - 791 + Oxidation (M); Phospho (ST)	[illegible]	p14368	Splicing factor 3B subunit 2
KLC2 seq: 506 - 518 + Phospho (ST)	[illegible]	p06577	Kinesin light chain 2
RACGAP1 seq: 598 - 608 + 2 Phospho (ST)	[illegible]	p00888	Rac GTPase-activating protein 1
MAST2 pS148	[illegible]	p09212	Microtubule-associated serine/threonine-protein kinase 2
EDF1 seq: 99 - 113 no mod	[illegible]	p13927	Endothelial differentiation-related factor 1
SCAF1 seq: 297 - 343 + Phospho (ST)	[illegible]	p13740	Splicing factor, arginine/serine-rich 19
XRN2 seq: 447 - 458 no mod	[illegible]	p09122	5'-3' exoribonuclease 2
PFDN4 pS125	[illegible]	p16733	Prefoldin subunit 4
CCDC86 seq: 30 - 53 + Phospho (ST)	[illegible]	p07622	Coiled-coil domain-containing protein 86
EDC3 pS131	[illegible]	p04276	Enhancer of mRNA-decapping protein 3
SSBP3 pT360	[illegible]	p09118	Single-stranded DNA-binding protein 3
TNRC18 pS611	[illegible]	p04678	Trinucleotide repeat-containing gene 18 protein
BRIP1 pS206	[illegible]	p13896	Fanconi anemia group J protein
HN1L pS97	[illegible]	p02192	Hematological and neurological expressed 1-like protein
DOCK7 seq: 894 - 916 + 2 Phospho (ST)	[illegible]	p04044	Dedicator of cytokinesis protein 7
ARFGAP1 pT135	[illegible]	p16921	ADP-ribosylation factor GTPase-activating protein 1
ANLN pS323	[illegible]	p02387	Actin-binding protein anillin
GAPDH seq: 324 - 335 no mod	[illegible]	p00648	Glyceraldehyde-3-phosphate dehydrogenase
PRKAB2 seq: 180 - 197 + Phospho (ST)	[illegible]	p18529	5'-AMP-activated protein kinase subunit beta-2
MARK2 seq: 464 - 482 + Phospho (ST)	[illegible]	p13028	Serine/threonine-protein kinase MARK2
PARN pS557	[illegible]	p09303	Poly(A)-specific ribonuclease PARN
ERC1 seq: 7 - 23 + Phospho (ST)	[illegible]	p03344	ELKS/Rab6-interacting/CAST family member 1
FAM122A seq: 60 - 68 + Phospho (ST)	[illegible]	p07004	Protein FAM122A
FAM122B seq: 48 - 56 + Phospho (ST)	[illegible]	p07001	Protein FAM122B
EXPH5 seq: 1766 - 1778 + Phospho (ST)	[illegible]	p00783	Exophilin-5
PARD6A pS278	[illegible]	p10553	Partitioning defective 6 homolog alpha
LUZP1 pS905	[illegible]	p05586	Leucine zipper protein 1
POTEE seq: 64 - 70 no mod	[illegible]	p07383	POTE ankyrin domain family member E
ZFP36L1 pS54	[illegible]	p07288	Zinc finger protein 36, C3H1 type-like 1
RB1 seq: 335 - 358 + Phospho (ST)	[illegible]	p11884	Retinoblastoma-associated protein
[illegible]F1 seq: 7 - 29 + Gln->pyro-Glu (N-term Q); Phospho (ST)	[illegible]	p08043	ATP-binding cassette sub-family F member 1
SYTL1 seq: 235 - 267 + 2 Phospho (ST)	[illegible]	p09998	Synaptotagmin-like protein 1
PARD3 pS827	[illegible]	p17753	Partitioning defective 3 homolog
RPS6KB1 pS441	[illegible]	p16690	Ribosomal protein S6 kinase beta-1
CCDC81 seq: 644 - 649 + Phospho (ST)	[illegible]	p16599	Coiled-coil domain-containing protein 81
HIVEP1 pS1180	[illegible]	p01394	Zinc finger protein 40
WDR1 seq: 66 - 81 no mod	[illegible]	p00446	WD repeat-containing protein 1
PLEKHG3 seq: 757 - 774 + Phospho (ST)	[illegible]	p16548	Pleckstrin homology domain-containing family G member 3
EIF4E seq: 163 - 173 no mod	[illegible]	p14504	Eukaryotic translation initiation factor 4E
OTUD5 pS64	[illegible]	p19538	OTU domain-containing protein 5
SPTAN1 pS1190	[illegible]	p03016	Spectrin alpha chain, non-erythrocytic 1
NCOR2 seq: 2265 - 2282 + Oxidation (M); Phospho (ST)	[illegible]	p10084	Nuclear receptor corepressor 2
RBM15B pS265 pS267	[illegible]	p07956	Putative RNA-binding protein 15B
CCT3 seq: 79 - 85 no mod	[illegible]	p04892	T-complex protein 1 subunit gamma
C7orf43 pS517	[illegible]	p04199	Uncharacterized protein C7orf43
SEPT9 pS30	[illegible]	p05913	Septin-9
ZC3H13 pS207	[illegible]	p05401	Zinc finger CCCH domain-containing protein 13
CASKIN2 seq: 841 - 851 + Phospho (ST)	[illegible]	p02135	Caskin-2
KRT8 seq: 342 - 352 no mod	[illegible]	p16157	Keratin, type II cytoskeletal 8
PCBP1 seq: 178 - 200 + 2 Oxidation (M); Phospho (ST)	[illegible]	p01096	Poly(rC)-binding protein 1
GRB7 pS66 pS76	[illegible]	p07543	Growth factor receptor-bound protein 7
CDCA3 seq: 199 - 216 + Phospho (ST)	[illegible]	p04536	Cell division cycle-associated protein 3
PLEKHM2 seq: 439 - 466 + Phospho (ST)	[illegible]	p02787	Pleckstrin homology domain-containing family M member 2
STMN3 seq: 121 - 130 no mod	[illegible]	p20283	Stathmin-3
MYDGF seq: 131 - 145 no mod	[illegible]	p17176	Myeloid-derived growth factor
EEF1D pS133 pM135	[illegible]	p19418	Elongation factor 1-delta
ABCF1 seq: 103 - 121 + Phospho (ST)	[illegible]	p12892	ATP-binding cassette sub-family F member 1
MTMR1 seq: 651 - 665 + Phospho (ST)	[illegible]	p15164	Myotubularin-related protein 1
SON seq: 1576 - 1610 + Phospho (ST)	[illegible]	p01952	Protein SON
LGALS3BP seq: 311 - 323 no mod	[illegible]	p06100	Galectin-3-binding protein
SHROOM4 pS1019	[illegible]	p20431	Protein Shroom4
PPP1R13L seq: 70 - 86 + Phospho (ST)	[illegible]	p00064	RelA-associated inhibitor
MUC5B seq: 1022 - 1040 no mod	[illegible]	p01717	Mucin-5B
PROSER2 seq: 41 - 52 + Phospho (ST)	[illegible]	p04058	Proline and serine-rich protein 2
KAT7 seq: 49 - 75 + Phospho (ST)	[illegible]	p10690	Histone acetyltransferase KAT7
TRIM28 seq: 592 - 629 + 2 Phospho (ST)	[illegible]	p12183	Transcription intermediary factor 1-beta
YTHDF1 seq: 335 - 370 + Phospho (ST)	[illegible]	p09800	YTH domain-containing family protein 1
HSPA9 seq: 542 - 563 no mod	[illegible]	p17269	Stress-70 protein, mitochondrial
LASP1 pT104	[illegible]	p16090	LIM and SH3 domain protein 1
MAP4 pS928	[illegible]	p12154	Microtubule-associated protein 4
AKAP2 seq: 461 - 481 + Phospho (ST)	[illegible]	p03311	A-kinase anchor protein 2
MAP7D1 seq: 111 - 133 + Phospho (ST)	[illegible]	p06579	MAP7 domain-containing protein 1
AKAP12 seq: 582 - 604 + Phospho (ST)	[illegible]	p15784	A-kinase anchor protein 12
MAP7D1 seq: 111 - 133 + 2 Phospho (ST)	[illegible]	p06580	MAP7 domain-containing protein 1
ACTG2 pM49	[illegible]	p14676	Actin, gamma-enteric smooth muscle
CAST seq: 213 - 249 + Phospho (ST)	[illegible]	p17734	Calpastatin
RAB11FIP1 seq: 433 - 443 + Phospho (ST)	[illegible]	p06606	Rab11 family-interacting protein 1
ARHGEF16 pS174	[illegible]	p15741	Rho guanine nucleotide exchange factor 16
EPS15 pT777	[illegible]	p14223	Epidermal growth factor receptor substrate 15
GTF2I seq: 556 - 567 + Phospho (ST)	[illegible]	p02444	General transcription factor II-I
ARHGEF17 pS142	[illegible]	p06862	Rho guanine nucleotide exchange factor 17
COL17A1 pS61	[illegible]	p07883	Collagen alpha-1(XVII) chain
TANK seq: 126 - 135 + Phospho (ST)	[illegible]	p05212	TRAF family member-associated NF-kappa-B activator
CGN seq: 129 - 146 + 2 Phospho (ST)	[illegible]	p05565	Cingulin
CABLES1 seq: 290 - 306 + Phospho (ST)	[illegible]	p03862	CDK5 and ABL1 enzyme substrate 1
DBNL seq: 265 - 280 + Phospho (ST)	[illegible]	p17253	Drebrin-like protein
DUSP3 seq: 159 - 176 no mod	[illegible]	p17627	Dual specificity protein phosphatase 3
DYNC1LI1 pS421	[illegible]	p03249	Cytoplasmic dynein 1 light intermediate chain 1
ACTC1 seq: 42 - 52 no mod	[illegible]	p14679	Actin, alpha cardiac muscle 1
TAF15 pM92	[illegible]	p07824	TATA-binding protein-associated factor 2N
EFHD2 pS74	[illegible]	p20907	EF-hand domain-containing protein D2
KIAA1671 seq: 770 - 795 + Phospho (ST)	[illegible]	p18331	Uncharacterized protein KIAA1671
SQSTM1 pS272	[illegible]	p04095	Sequestosome-1
SEPT9 pS85	[illegible]	p14571	Septin-9
KNOP1 seq: 119 - 136 + Phospho (ST)	[illegible]	p04570	Lysine-rich nucleolar protein 1
CDK13 pT871	[illegible]	p10288	Cyclin-dependent kinase 13
NDRG3 seq: 329 - 345 + 2 Phospho (ST)	[illegible]	p02746	Protein NDRG3
ZBTB7A seq: 505 - 531 + Phospho (ST)	[illegible]	p16076	Zinc finger and BTB domain-containing protein 7A
GRIP1 pS845 pS847	[illegible]	p07980	Glutamate receptor-interacting protein 1
[illegible]AM122B seq: 127 - 143 + Oxidation (M); 2 Phospho (ST)	[illegible]	p10138	Protein FAM122B
WDR59 seq: 599 - 606 + Phospho (ST)	[illegible]	p10292	WD repeat-containing protein 59
PCBP2 seq: 177 - 199 + Phospho (ST)	[illegible]	p14979	Poly(rC)-binding protein 2
IRS1 seq: 525 - 538 + Phospho (ST)	[illegible]	p02751	Insulin receptor substrate 1
CCDC64B pS36	[illegible]	p07514	Bicaudal D-related protein 2
IRF2BP1 seq: 472 - 507 + Phospho (ST)	[illegible]	p09612	Interferon regulatory factor 2-binding protein 1
FAM21A pS352	[illegible]	p10575	WASH complex subunit FAM21A
ULK1 pS450	[illegible]	p09420	Serine/threonine-protein kinase ULK1
NUP107 pS37	[illegible]	p01209	Nuclear pore complex protein Nup107
C2CD2L pM401 pS411	[illegible]	p20258	C2 domain-containing protein 2-like
SHROOM3 pS677	[illegible]	p14607	Protein Shroom3
DTL seq: 415 - 435 + Phospho (ST)	[illegible]	p17128	Denticleless protein homolog
MLLT4 seq: 1229 - 1237 + Phospho (Y)	[illegible]	p16913	Afadin
WRNIP1 seq: 138 - 155 + Phospho (ST)	[illegible]	p15280	ATPase WRNIP1
SCRIB pT475	[illegible]	p07544	Protein scribble homolog
SLC9A3R1 seq: 351 - 358 no mod	[illegible]	p12851	Na(+)/H(+) exchange regulatory cofactor NHE-RF1
MARK2 seq: 595 - 603 + Phospho (ST)	[illegible]	p03536	Serine/threonine-protein kinase MARK2
RPS17 seq: 34 - 45 no mod	[illegible]	p01721	40S ribosomal protein S17
LSM14B seq: 154 - 172 no mod	[illegible]	p04528	Protein LSM14 homolog B
SMAP seq: 76 - 91 no mod	[illegible]	p13932	Small acidic protein
ILF3 seq: 6 - 12 no mod	[illegible]	p14289	Interleukin enhancer-binding factor 3
PANK2 seq: 167 - 184 + Phospho (ST)	[illegible]	p19510	Pantothenate kinase 2, mitochondrial
PANK2 seq: 166 - 183 + Phospho (ST)	[illegible]	p07558	Pantothenate kinase 2, mitochondrial
PXN seq: 76 - 93 + Phospho (Y)	[illegible]	p16685	Paxillin
CGNL1 seq: 296 - 311 + Phospho (ST)	[illegible]	p03622	Cingulin-like protein 1
EEF2K pS74	[illegible]	p00068	Eukaryotic elongation factor 2 kinase
MAP3K7 pS439	[illegible]	p06741	Mitogen-activated protein kinase kinase kinase 7
SEPT9 pS30	[illegible]	p05914	Septin-9
FNBP1 seq: 495 - 515 + Phospho (ST)	[illegible]	p06799	Formin-binding protein 1
RBL2 seq: 444 - 449 no mod	[illegible]	p11939	Retinoblastoma-like protein 2
SHROOM2 seq: 122 - 146 + Phospho (ST)	[illegible]	p16485	Protein Shroom2
TOR4A seq: 44 - 65 + Phospho (ST)	[illegible]	p11304	Torsin-4A
SEPT9 pS30	[illegible]	p05915	Septin-9
RBBP8NL pS645	[illegible]	p12890	RBBP8 N-terminal-like protein
LMNA pS458 pM464	[illegible]	p09492	Prelamin-A/C
NCBP3 pM410 pS415	[illegible]	p10063	Nuclear cap-binding protein subunit 3
CTU2 seq: 428 - 443 + Phospho (ST)	[illegible]	p02358	Cytoplasmic tRNA 2-thiolation protein 2
KLF16 seq: 89 - 114 + Phospho (ST)	[illegible]	p15998	Krueppel-like factor 16
SRP72 seq: 618 - 645 + 3 Phospho (ST)	[illegible]	p01888	Signal recognition particle subunit SRP72
MYO18A seq: 726 - 745 + Phospho (ST)	[illegible]	p03454	Unconventional myosin-XVIIIa
FAM83H pS1003	[illegible]	p07125	Protein FAM83H
ALDOA seq: 29 - 43 no mod	[illegible]	p15887	Fructose-bisphosphate aldolase A
ARHGEF28 pS1538	[illegible]	p07957	Rho guanine nucleotide exchange factor 28
HIST1H1B seq: 2 - 22 + 2 Phospho (ST)	[illegible]	p05963	Histone H1.5
RBMX pS352 pM353	[illegible]	p15675	RNA-binding motif protein, X chromosome
PLEC seq: 3221 - 3231 no mod	[illegible]	p20622	Plectin
KDM3B seq: 725 - 763 + Phospho (ST)	[illegible]	p03652	Lysine-specific demethylase 3B
CGN pS131 pS134	[illegible]	p05566	Cingulin
CHTF18 seq: 59 - 79 + Phospho (ST)	[illegible]	p16233	Chromosome transmission fidelity protein 18 homolog
MORC2 seq: 722 - 736 + Phospho (ST)	[illegible]	p12503	MORC family CW-type zinc finger protein 2
RBM15 seq: 763 - 783 + Phospho (ST)	[illegible]	p11483	Putative RNA-binding protein 15
KIF1A pS1548	[illegible]	p12942	Kinesin-like protein KIF1A
CCDC86 pS58	[illegible]	p20636	Coiled-coil domain-containing protein 86
KIAA1522 pS858 pS862	[illegible]	p20181	Uncharacterized protein KIAA1522
TCF3 seq: 372 - 392 + Phospho (ST)	[illegible]	p20717	Transcription factor E2-alpha
CASKIN2 seq: 705 - 730 + Phospho (ST)	[illegible]	p04110	Caskin-2
HSPB1 seq: 141 - 171 no mod	[illegible]	p12328	Heat shock protein beta-1
PTPN23 seq: 1112 - 1145 + Phospho (ST)	[illegible]	p07408	Tyrosine-protein phosphatase non-receptor type 23
KIAA1522 pS545	[illegible]	p15778	Uncharacterized protein KIAA1522
RBM14 pS618	[illegible]	p07141	RNA-binding protein 14
RBMX seq: 318 - 331 + Phospho (ST)	[illegible]	p18785	RNA-binding motif protein, X chromosome
SLAIN2 pS63	[illegible]	p20709	SLAIN motif-containing protein 2
ZNF638 pS128	[illegible]	p07831	Zinc finger protein 638
GYS1 seq: 708 - 737 + Phospho (ST)	[illegible]	p06998	Glycogen [starch] synthase, muscle
THYN1 pS14	[illegible]	p12250	Thymocyte nuclear protein 1
ITSN2 seq: 559 - 564 no mod	[illegible]	p08353	Intersectin-2
PPFIA1 pS1172 pM1175	[illegible]	p00675	Liprin-alpha-1
PHF6 seq: 133 - 157 + Phospho (ST)	[illegible]	p12513	PHD finger protein 6
PKN1 seq: 772 - 794 + Phospho (ST)	[illegible]	p02076	Serine/threonine-protein kinase N1

SH3PXD2A seq: 513 - 521 + Phospho (ST)	[illegible]	p06481	SH3 and PX domain-containing protein 2A
MTSS1L seq: 566 - 575 + Phospho (ST)	[illegible]	p06500	MTSS1-like protein
DSG2 seq: 262 - 275 no mod	[illegible]	p14073	Desmoglein-2
BRAF pT401	[illegible]	p18382	Serine/threonine-protein kinase B-raf
LASP1 pM64 pT68	[illegible]	p07918	LIM and SH3 domain protein 1
TXN seq: 86 - 96 no mod	[illegible]	p01497	Thioredoxin
HUWE1 seq: 2885 - 2903 + Phospho (ST)	[illegible]	p20551	E3 ubiquitin-protein ligase HUWE1
MAGI3 seq: 722 - 735 + Phospho (ST)	[illegible]	p05669	Membrane-associated guanylate kinase, WW and PDZ domain-containing protein 3
APC pS780	[illegible]	p20293	Adenomatous polyposis coli protein
HUWE1 pS3757	[illegible]	p11684	E3 ubiquitin-protein ligase HUWE1
TMSB4X seq: 20 - 32 + Phospho (ST)	[illegible]	p12500	Thymosin beta-4
SEPT9 pS30	[illegible]	p05912	Septin-9
MYO9B seq: 1352 - 1362 + Phospho (ST)	[illegible]	p06494	Unconventional myosin-IXb
SMG9 seq: 22 - 37 + Phospho (ST)	[illegible]	p17312	Protein SMG9
ENAH seq: 462 - 483 + Phospho (ST)	[illegible]	p15153	Protein enabled homolog
SF3A1 seq: 116 - 131 no mod	[illegible]	p01100	Splicing factor 3A subunit 1
FAM83H pS914	[illegible]	p07321	Protein FAM83H
LUZP1 seq: 903 - 916 + Phospho (ST)	[illegible]	p05585	Leucine zipper protein 1
ABCF1 pT108 pS109	[illegible]	p10599	ATP-binding cassette sub-family F member 1
AMPD2 pS168	[illegible]	p08497	AMP deaminase 2
NAV1 pS90	[illegible]	p08432	Neuron navigator 1
KIAA0930 pS304	[illegible]	p09311	Uncharacterized protein KIAA0930
ERBB2IP seq: 915 - 941 + Phospho (Y)	[illegible]	p06200	Protein LAP2
ZNF318 seq: 234 - 248 + Phospho (ST)	[illegible]	p06063	Zinc finger protein 318
SLCO4A1 pS40	[illegible]	p19547	Solute carrier organic anion transporter family member 4A1
EIF4EBP1 seq: 21 - 51 + 2 Phospho (ST)	[illegible]	p00607	Eukaryotic translation initiation factor 4E-binding protein 1
LMO7 seq: 1491 - 1506 + Phospho (ST)	[illegible]	p06213	LIM domain only protein 7
UBA1 seq: 785 - 802 no mod	[illegible]	p20990	Ubiquitin-like modifier-activating enzyme 1
FLII seq: 12 - 21 no mod	[illegible]	p15051	Protein flightless-1 homolog
CTTNBP2NL seq: 555 - 573 + Phospho (ST)	[illegible]	p00777	CTTNBP2 N-terminal-like protein
AHCTF1 seq: 1209 - 1225 + Phospho (ST)	[illegible]	p03483	Protein ELYS
STK25 pT184	[illegible]	p12291	Serine/threonine-protein kinase 25
PPIB seq: 191 - 204 no mod	[illegible]	p18674	Peptidyl-prolyl cis-trans isomerase B
AMBP seq: 159 - 166 no mod	[illegible]	p17058	Protein AMBP
PKP3 seq: 174 - 182 + Phospho (Y)	[illegible]	p20877	Plakophilin-3
BTBD9 seq: 590 - 605 + Phospho (ST)	[illegible]	p19920	BTB/POZ domain-containing protein 9
RB1CC1 pS644	[illegible]	p19437	RB1-inducible coiled-coil protein 1
CD3EAP seq: 268 - 288 + Phospho (ST)	[illegible]	p01954	DNA-directed RNA polymerase I subunit RPA34
GOLGA4 seq: 39 - 64 + Phospho (ST)	[illegible]	p02108	Golgin subfamily A member 4
CASKIN2 seq: 251 - 271 + Phospho (ST)	[illegible]	p08993	Caskin-2
AKAP13 pT1149	[illegible]	p19171	A-kinase anchor protein 13
PPP2R5D pS573	[illegible]	p07215	Serine/threonine-protein phosphatase 2A 56 kDa regulatory subunit delta isoform
LARP1 pT526	[illegible]	p19159	La-related protein 1
CCDC9 pS80	[illegible]	p04602	Coiled-coil domain-containing protein 9
ARL3 seq: 80 - 98 no mod	[illegible]	p08924	ADP-ribosylation factor-like protein 3
RPS25 seq: 42 - 52 no mod	[illegible]	p18677	40S ribosomal protein S25
NME1 seq: 57 - 66 no mod	[illegible]	p18342	Nucleoside diphosphate kinase A
SVIL pS968	[illegible]	p00343	Supervillin
TJP2 seq: 452 - 478 + Phospho (ST)	[illegible]	p10126	Tight junction protein ZO-2
CTPS1 seq: 571 - 591 + Phospho (ST)	[illegible]	p05649	CTP synthase 1
C12orf45 seq: 9 - 27 + Phospho (ST)	[illegible]	p19533	Uncharacterized protein C12orf45
HNRNPA3 seq: 114 - 126 no mod	[illegible]	p17863	Heterogeneous nuclear ribonucleoprotein A3
RBM33 pS765	[illegible]	p01306	RNA-binding protein 33
SF3B1 seq: 431 - 451 + 2 Phospho (ST)	[illegible]	p10581	Splicing factor 3B subunit 1
seq: 465 - 476 + Gln->pyro-Glu (N-term Q); Phospho (ST)	[illegible]	p08707	Actin-binding protein anillin
LUZP1 seq: 434 - 449 + Phospho (ST)	[illegible]	p19599	Leucine zipper protein 1
AKAP11 pT1100	[illegible]	p18344	A-kinase anchor protein 11
GLCCI1 seq: 221 - 236 + Phospho (ST)	[illegible]	p06206	Glucocorticoid-induced transcript 1 protein
SRRM2 seq: 2419 - 2430 + Phospho (ST)	[illegible]	p09843	Serine/arginine repetitive matrix protein 2
PLEC seq: 2432 - 2442 no mod	[illegible]	p08408	Plectin
TNKS1BP1 pS762	[illegible]	p18550	182 kDa tankyrase-1-binding protein
EEF1D pS133 pM135	[illegible]	p19419	Elongation factor 1-delta
RBBP8NL pS645	[illegible]	p07224	RBBP8 N-terminal-like protein
SYNRG pS752	[illegible]	p08305	Synergin gamma
EIF3A pS1336	[illegible]	p06455	Eukaryotic translation initiation factor 3 subunit A
FAM122B pS50	[illegible]	p07002	Protein FAM122B
APC seq: 1436 - 1449 + Phospho (ST)	[illegible]	p05335	Adenomatous polyposis coli protein
UBA1 seq: 1033 - 1054 no mod	[illegible]	p20116	Ubiquitin-like modifier-activating enzyme 1
ACIN1 pS1004	[illegible]	p03056	Apoptotic chromatin condensation inducer in the nucleus
RSU1 seq: 98 - 103 + Phospho (ST)	[illegible]	p11153	Ras suppressor protein 1
CENPT seq: 40 - 49 + Phospho (ST)	[illegible]	p07631	Centromere protein T
MTA1 pS449	[illegible]	p04829	Metastasis-associated protein MTA1
TAGLN3 seq: 183 - 196 + Phospho (Y)	[illegible]	p16261	Transgelin-3
MPDZ pS354	[illegible]	p03066	Multiple PDZ domain protein
CAMSAP1 pS629	[illegible]	p06860	Calmodulin-regulated spectrin-associated protein 1
NCOR1 pS1472	[illegible]	p19786	Nuclear receptor corepressor 1
RPS10 pS157	[illegible]	p13457	40S ribosomal protein S10
SRRM2 pS1404 pT1413	[illegible]	p20613	Serine/arginine repetitive matrix protein 2
MAP7D1 seq: 273 - 283 + Phospho (ST)	[illegible]	p03947	MAP7 domain-containing protein 1
CNN3 seq: 54 - 64 no mod	[illegible]	p18822	Calponin-3
HNRNPA1 seq: 207 - 218 no mod	[illegible]	p16059	Heterogeneous nuclear ribonucleoprotein A1
AHDC1 pS1549	[illegible]	p15544	AT-hook DNA-binding motif-containing protein 1
TNKS1BP1 pS429	[illegible]	p07437	182 kDa tankyrase-1-binding protein
PANK2 seq: 165 - 183 + Phospho (ST)	[illegible]	p06767	Pantothenate kinase 2, mitochondrial
GRIPAP1 seq: 688 - 714 + Phospho (ST)	[illegible]	p04980	GRIP1-associated protein 1
PDXDC1 seq: 713 - 734 + Phospho (ST)	[illegible]	p15304	Pyridoxal-dependent decarboxylase domain-containing protein 1
TRA2A pT202	[illegible]	p20496	Transformer-2 protein homolog alpha
SEC14L1 pT234	[illegible]	p17719	SEC14-like protein 1
ATXN2 pS784	[illegible]	p15845	Ataxin-2
PANK2 seq: 166 - 184 + Phospho (ST)	[illegible]	p07557	Pantothenate kinase 2, mitochondrial
CTPS1 seq: 571 - 591 + Phospho (ST)	[illegible]	p05650	CTP synthase 1
TICRR seq: 915 - 927 + Phospho (ST)	[illegible]	p09464	Treslin
HNRNPDL seq: 198 - 209 no mod	[illegible]	p18999	Heterogeneous nuclear ribonucleoprotein D-like
BAIAP2 pT360	[illegible]	p09043	Brain-specific angiogenesis inhibitor 1-associated protein 2
KIAA1522 seq: 856 - 876 + Phospho (ST)	[illegible]	p20180	Uncharacterized protein KIAA1522
TCF20 seq: 1003 - 1015 + Phospho (ST)	[illegible]	p15321	Transcription factor 20
CEP170B pS1199	[illegible]	p03363	Centrosomal protein of 170 kDa protein B
NCOR2 pS1259	[illegible]	p14172	Nuclear receptor corepressor 2
CAST seq: 213 - 249 + Phospho (ST)	[illegible]	p17735	Calpastatin
C1orf198 seq: 173 - 184 + Phospho (ST)	[illegible]	p03697	Uncharacterized protein C1orf198
CCDC86 seq: 30 - 53 + Phospho (ST)	[illegible]	p07623	Coiled-coil domain-containing protein 86
SLTM seq: 549 - 564 + Oxidation (M); Phospho (ST)	[illegible]	p13663	SAFB-like transcription modulator
MAGI3 seq: 1469 - 1479 + Phospho (ST)	[illegible]	p16643	Membrane-associated guanylate kinase, WW and PDZ domain-containing protein 3
ANLN seq: 639 - 650 + Phospho (ST)	[illegible]	p06111	Actin-binding protein anillin
GNB4 seq: 79 - 89 no mod	[illegible]	p11585	Guanine nucleotide-binding protein subunit beta-4
SP110 pS380	[illegible]	p06421	Sp110 nuclear body protein
HCFC1 seq: 595 - 608 + Oxidation (M); Phospho (ST)	[illegible]	p01750	Host cell factor 1
ITSN1 seq: 976 - 992 + Phospho (ST)	[illegible]	p03450	Intersectin-1
EIF4B pS207	[illegible]	p19694	Eukaryotic translation initiation factor 4B
AP2B1 seq: 889 - 901 no mod	[illegible]	p08980	AP-2 complex subunit beta
SLAIN2 pS391	[illegible]	p10747	SLAIN motif-containing protein 2
TPD52L1 pS174	[illegible]	p01479	Tumor protein D53
FOXK1 pS416	[illegible]	p06296	Forkhead box protein K1
RANBP2 seq: 1563 - 1585 + Phospho (ST)	[illegible]	p19007	E3 SUMO-protein ligase RanBP2
PTPN12 pS449	[illegible]	p12413	Tyrosine-protein phosphatase non-receptor type 12
MZT2B pS139	[illegible]	p08107	Mitotic-spindle organizing protein 2B
RBBP8NL seq: 377 - 403 + Phospho (ST)	[illegible]	p15379	RBBP8 N-terminal-like protein
CAPRIN1 seq: 668 - 675 no mod	[illegible]	p18783	Caprin-1
PLEKHA6 pS448	[illegible]	p07117	Pleckstrin homology domain-containing family A member 6
ERF seq: 529 - 546 + Phospho (ST)	[illegible]	p06429	ETS domain-containing transcription factor ERF
FAM117A pS29	[illegible]	p16236	Protein FAM117A
PHF2 pS882	[illegible]	p18298	Lysine-specific demethylase PHF2
NCOA5 seq: 377 - 388 + Phospho (ST)	[illegible]	p03599	Nuclear receptor coactivator 5
LAD1 seq: 283 - 306 + Phospho (ST)	[illegible]	p07547	Ladinin-1
BCAS1 pS552	[illegible]	p08008	Breast carcinoma-amplified sequence 1
SRRM2 pM1168 pS1179	[illegible]	p10261	Serine/arginine repetitive matrix protein 2
AHNAK seq: 1006 - 1024 + Phospho (ST)	[illegible]	p16458	Neuroblast differentiation-associated protein AHNAK
SRRM2 seq: 2419 - 2430 + Phospho (ST)	[illegible]	p09844	Serine/arginine repetitive matrix protein 2
HNRNPU pS192	[illegible]	p03889	Heterogeneous nuclear ribonucleoprotein U
MTMR1 seq: 651 - 665 + Phospho (ST)	[illegible]	p15165	Myotubularin-related protein 1
LMO7 seq: 1491 - 1506 + Phospho (ST)	[illegible]	p06212	LIM domain only protein 7
WDR7 seq: 1152 - 1173 + Phospho (ST)	[illegible]	p03644	WD repeat-containing protein 7
HDAC6 pS22	[illegible]	p08197	Histone deacetylase 6
PALM pS116	[illegible]	p17349	Paralemmin-1
SAP130 seq: 433 - 444 + Phospho (ST)	[illegible]	p00585	Histone deacetylase complex subunit SAP130
PPP1R9B seq: 98 - 112 + Phospho (ST)	[illegible]	p19491	Neurabin-2
EMSY seq: 207 - 218 + Phospho (ST)	[illegible]	p02452	Protein EMSY
PKP3 seq: 82 - 94 + Phospho (Y)	[illegible]	p15363	Plakophilin-3
PRRC2C seq: 1542 - 1551 + Phospho (ST)	[illegible]	p05812	Protein PRRC2C
FAM83H seq: 890 - 911 + 2 Phospho (ST)	[illegible]	p07324	Protein FAM83H
DPF2 pT176	[illegible]	p14055	Zinc finger protein ubi-d4
WWC3 seq: 456 - 477 + Phospho (ST)	[illegible]	p03834	Protein WWC3
MRGBP seq: 182 - 200 + Phospho (ST)	[illegible]	p00738	MRG/MORF4L-binding protein
LARP1B pS340	[illegible]	p14232	La-related protein 1B
CASKIN2 seq: 944 - 961 + Phospho (ST)	[illegible]	p15176	Caskin-2
ABI1 seq: 200 - 219 + Phospho (ST)	[illegible]	p02612	Abl interactor 1
CCDC9 seq: 372 - 392 + Phospho (ST)	[illegible]	p17773	Coiled-coil domain-containing protein 9
GAB2 seq: 140 - 154 + Phospho (ST)	[illegible]	p03826	GRB2-associated-binding protein 2
SFSWAP pS909	[illegible]	p14986	Splicing factor, suppressor of white-apricot homolog
TBC1D1 seq: 594 - 612 + Phospho (ST)	[illegible]	p20016	TBC1 domain family member 1
KIAA0226 seq: 246 - 256 + Phospho (ST)	[illegible]	p03465	Run domain Beclin-1 interacting and cysteine-rich containing protein
KIAA1217 pS169	[illegible]	p02234	Sickle tail protein homolog
ORC2 seq: 217 - 230 + Phospho (ST)	[illegible]	p00578	Origin recognition complex subunit 2
EIF3D seq: 282 - 310 no mod	[illegible]	p18424	Eukaryotic translation initiation factor 3 subunit D
CEP170 pS1165	[illegible]	p11695	Centrosomal protein of 170 kDa
ZP2 seq: 453 - 460 + Phospho (ST)	[illegible]	p13671	Zona pellucida sperm-binding protein 2
HNRNPU pM53 pS59	[illegible]	p11060	Heterogeneous nuclear ribonucleoprotein U
YTHDC2 pS1279	[illegible]	p15259	Probable ATP-dependent RNA helicase YTHDC2
HOOK1 seq: 63 - 72 no mod	[illegible]	p14118	Protein Hook homolog 1
GPATCH8 seq: 730 - 746 + Phospho (ST)	[illegible]	p15496	G patch domain-containing protein 8
LMBRD2 seq: 683 - 695 + Phospho (ST)	[illegible]	p00235	LMBR1 domain-containing protein 2
ENO1 pS263	[illegible]	p05731	Alpha-enolase
CNKSR1 pS314	[illegible]	p04414	Connector enhancer of kinase suppressor of ras 1
SRRM2 seq: 433 - 451 + Phospho (ST)	[illegible]	p14876	Serine/arginine repetitive matrix protein 2
MIA3 pS1906	[illegible]	p18581	Melanoma inhibitory activity protein 3
KIAA1143 seq: 141 - 154 + Phospho (ST)	[illegible]	p09079	Uncharacterized protein KIAA1143
RPS19 seq: 8 - 16 no mod	[illegible]	p18103	40S ribosomal protein S19
ARFGAP2 pS432	[illegible]	p20433	ADP-ribosylation factor GTPase-activating protein 2
TAGLN2 seq: 50 - 57 no mod	[illegible]	p17344	Transgelin-2
ARID1B seq: 1554 - 1566 + Oxidation (M); Phospho (ST)	[illegible]	p09883	AT-rich interactive domain-containing protein 1B
FRMD6 pS544	[illegible]	p02078	FERM domain-containing protein 6
TFAP4 seq: 116 - 127 + Phospho (ST)	[illegible]	p16676	Transcription factor AP-4
RALGAPA1 seq: 795 - 809 + Phospho (ST)	[illegible]	p03612	Ral GTPase-activating protein subunit alpha-1
NCF2 seq: 233 - 238 no mod	[illegible]	p02404	Neutrophil cytosol factor 2
KIAA1671 pS128	[illegible]	p02096	Uncharacterized protein KIAA1671
PUM1 pS709	[illegible]	p07503	Pumilio homolog 1
ARHGAP21 seq: 440 - 453 + Phospho (ST)	[illegible]	p03453	Rho GTPase-activating protein 21
REPS1 pS709	[illegible]	p11490	RalBP1-associated Eps domain-containing protein 1
CBX5 pS92	[illegible]	p07209	Chromobox protein homolog 5
CSNK1E pS389	[illegible]	p16283	Casein kinase I isoform epsilon
DAB2 pS401	[illegible]	p03800	Disabled homolog 2
EEF1D seq: 60 - 83 no mod	[illegible]	p05319	Elongation factor 1-delta
MLF2 pS238	[illegible]	p12246	Myeloid leukemia factor 2
ADRBK1 pS670	[illegible]	p04297	Beta-adrenergic receptor kinase 1
DTL pT429	[illegible]	p17129	Denticleless protein homolog
TCEA1 seq: 270 - 279 no mod	[illegible]	p09722	Transcription elongation factor A protein 1
GIGYF2 pS139	[illegible]	p05933	PERQ amino acid-rich with GYF domain-containing protein 2
GRWD1 seq: 342 - 350 + Phospho (ST)	[illegible]	p05660	Glutamate-rich WD repeat-containing protein 1
DENND1A pS523	[illegible]	p02064	DENN domain-containing protein 1A
FOXA1 pS307	[illegible]	p13329	Hepatocyte nuclear factor 3-alpha
MRGBP seq: 182 - 200 + Phospho (ST)	[illegible]	p00739	MRG/MORF4L-binding protein
MDC1 pS168	[illegible]	p11367	Mediator of DNA damage checkpoint protein 1
TLN1 seq: 1432 - 1469 no mod	[illegible]	p20312	Talin-1
C17orf59 seq: 194 - 209 + Phospho (ST)	[illegible]	p07546	Uncharacterized protein C17orf59
COBL seq: 960 - 972 + Phospho (ST)	[illegible]	p12899	Protein cordon-bleu
ZZZ3 pS82	[illegible]	p17087	ZZ-type zinc finger-containing protein 3
NCOA3 seq: 701 - 730 + Phospho (ST)	[illegible]	p13623	Nuclear receptor coactivator 3
MGRN1 pS515	[illegible]	p21043	E3 ubiquitin-protein ligase MGRN1
HN1 pT54	[illegible]	p10242	Hematological and neurological expressed 1 protein
RPSA seq: 42 - 52 no mod	[illegible]	p12602	40S ribosomal protein SA
TFAP4 seq: 115 - 126 + Phospho (ST)	[illegible]	p07444	Transcription factor AP-4
LMNB1 pS391	[illegible]	p11501	Lamin-B1
ABCF1 seq: 103 - 121 + Phospho (ST)	[illegible]	p12893	ATP-binding cassette sub-family F member 1
SFPQ pM38	[illegible]	p04494	Splicing factor, proline- and glutamine-rich
KLC2 pS610 pM612	[illegible]	p02535	Kinesin light chain 2
ID2B pS5	[illegible]	p20745	Putative DNA-binding protein inhibitor ID-2B
EIF2A seq: 145 - 164 no mod	[illegible]	p08958	Eukaryotic translation initiation factor 2A
CTPS1 seq: 565 - 591 + 2 Phospho (ST)	[illegible]	p18140	CTP synthase 1
RAB11FIP1 seq: 498 - 520 + Phospho (ST)	[illegible]	p08585	Rab11 family-interacting protein 1
BCL3 seq: 164 - 172 no mod	[illegible]	p17526	B-cell lymphoma 3 protein

U2AF2 pS79	5E+09	3E+09	[illegible]	25732	p16304	Splicing factor U2AF 65 kDa subunit
PARD3 seq: 479 - 491 + Phospho (Y)	38324	37149	[illegible]	9E+07	p18087	Partitioning defective 3 homolog
AHNAK seq: 4993 - 5017 + Phospho (ST)	1E+08	3E+08	[illegible]	9E+07	p04418	Neuroblast differentiation-associated protein AHNAK
BAIAP2 pS366 pM367	1E+09	1E+09	[illegible]	1E+09	p03684	Brain-specific angiogenesis inhibitor 1-associated protein 2
CHMP3 pM197 pS200	2E+09	1E+09	[illegible]	4E+08	p00745	Charged multivesicular body protein 3
IRS2 pS577	5E+08	4E+08	[illegible]	3E+08	p01864	Insulin receptor substrate 2
PLAU seq: 369 - 387 no mod	38324	6E+07	[illegible]	4E+07	p02949	Urokinase-type plasminogen activator
HSPA1A seq: 57 - 72 no mod	4E+07	1E+08	[illegible]	5E+07	p09207	Heat shock 70 kDa protein 1A
RAB11FIP1 seq: 434 - 443 + Phospho (ST)	6E+07	2E+08	[illegible]	25732	p03850	Rab11 family-interacting protein 1
ARFGAP1 seq: 163 - 182 no mod	2E+07	3E+07	[illegible]	1E+07	p20780	ADP-ribosylation factor GTPase-activating protein 1
KHDRBS1 seq: 57 - 96 + Phospho (ST)	6E+09	6E+09	[illegible]	3E+09	p19556	KH domain-containing, RNA-binding, signal transduction-associated protein 1
SH3GL1 seq: 137 - 149 no mod	6E+07	1E+08	[illegible]	3E+08	p08222	Endophilin-A2
DTL seq: 483 - 498 + 2 Phospho (ST)	3E+08	2E+08	[illegible]	6E+08	p07310	Denticleless protein homolog
KDM1B pS247	7E+08	7E+08	[illegible]	6E+08	p21196	Lysine-specific histone demethylase 1B
CLTB seq: 135 - 142 no mod	5E+08	4E+08	[illegible]	3E+08	p18630	Clathrin light chain B
NAV1 seq: 90 - 102 + Phospho (ST)	4E+08	4E+08	[illegible]	6E+07	p04948	Neuron navigator 1
YAP1 seq: 107 - 124 + Phospho (ST)	2E+09	2E+09	[illegible]	1E+09	p08807	Transcriptional coactivator YAP1
U2AF2 pS79	1E+10	1E+10	[illegible]	25732	p16303	Splicing factor U2AF 65 kDa subunit
NAA30 pS89	38324	4E+07	[illegible]	9E+07	p19029	N-alpha-acetyltransferase 30
CCDC9 pS80	4E+09	4E+09	[illegible]	1E+09	p04603	Coiled-coil domain-containing protein 9
ZBTB7A seq: 503 - 530 + Phospho (ST)	3E+08	2E+09	[illegible]	8E+08	p00904	Zinc finger and BTB domain-containing protein 7A
RAPH1 pS894	9E+08	9E+08	[illegible]	2E+08	p08549	Ras-associated and pleckstrin homology domains-containing protein 1
C15orf52 seq: 198 - 214 + Phospho (ST)	1E+08	2E+08	[illegible]	6E+09	p00683	Uncharacterized protein C15orf52
PPP1R35 seq: 35 - 56 + 2 Phospho (ST)	2E+08	1E+08	[illegible]	1E+09	p19858	Protein phosphatase 1 regulatory subunit 35
PLEC seq: 3620 - 3628 no mod	2E+08	37149	[illegible]	25732	p00974	Plectin
CTPS1 seq: 565 - 591 + Phospho (ST); Phospho (Y)	1E+08	6E+07	[illegible]	5E+08	p18137	CTP synthase 1
AHNAK pS4522	6E+07	1E+08	[illegible]	1E+08	p04464	Neuroblast differentiation-associated protein AHNAK
TJP2 seq: 1048 - 1076 + Phospho (ST)	1E+08	8E+07	[illegible]	1E+09	p05516	Tight junction protein ZO-2
TBC1D1 seq: 208 - 229 + Phospho (ST)	3E+08	2E+08	[illegible]	25732	p15297	TBC1 domain family member 1
SMTN seq: 364 - 384 + Phospho (ST)	4E+07	37149	[illegible]	25732	p11395	Smoothelin
GPRIN1 pS615	5E+08	8E+08	[illegible]	2E+08	p01445	G protein-regulated inducer of neurite outgrowth 1
AHNAK pS5731	3E+09	4E+09	[illegible]	3E+09	p15819	Neuroblast differentiation-associated protein AHNAK
ANKRD36 seq: 378 - 383 + Phospho (ST)	5E+07	1E+08	[illegible]	25732	p13096	Ankyrin repeat domain-containing protein 36A
LRRC30 seq: 106 - 111 + Phospho (ST)	5E+07	1E+08	[illegible]	25732	p11486	Leucine-rich repeat-containing protein 30
SASH1 seq: 405 - 416 + Phospho (ST)	38324	37149	[illegible]	7E+07	p02984	SAM and SH3 domain-containing protein 1
USP24 pS2047	4E+08	37149	[illegible]	3E+08	p00876	Ubiquitin carboxyl-terminal hydrolase 24
PPP1R13L pT308	4E+08	6E+08	[illegible]	5E+08	p17122	RelA-associated inhibitor
PACS1 pS519	5E+07	3E+07	[illegible]	25732	p08309	Phosphofurin acidic cluster sorting protein 1
POLD2 seq: 10 - 26 + Phospho (ST)	9E+07	1E+08	[illegible]	8E+07	p20500	DNA polymerase delta subunit 2
USP1 seq: 305 - 320 + Phospho (ST)	3E+07	8E+07	[illegible]	1E+08	p13370	Ubiquitin carboxyl-terminal hydrolase 1
LARP1 pS143	4E+09	2E+09	[illegible]	5E+09	p09766	La-related protein 1
RAPH1 pS894	9E+08	8E+08	[illegible]	2E+08	p08550	Ras-associated and pleckstrin homology domains-containing protein 1
EIF4EBP1 seq: 20 - 51 + 2 Phospho (ST)	5E+10	4E+10	[illegible]	2E+10	p06419	Eukaryotic translation initiation factor 4E-binding protein 1
SLTM pS553 pM557	5E+08	8E+08	[illegible]	5E+08	p04640	SAFB-like transcription modulator
PLEKHN1 pS611	1E+08	1E+08	[illegible]	3E+08	p17680	Pleckstrin homology domain-containing family N member 1
SOX13 pS310	9E+08	1E+09	[illegible]	1E+08	p19995	Transcription factor SOX-13
TJP1 seq: 1577 - 1601 + Phospho (ST)	1E+08	3E+08	[illegible]	2E+09	p05575	Tight junction protein ZO-1
CTNND2 seq: 472 - 488 + Phospho (ST)	3E+08	4E+08	[illegible]	5E+07	p02779	Catenin delta-2
ZFAND3 pS129	4E+08	4E+08	[illegible]	6E+07	p06152	AN1-type zinc finger protein 3
PEA15 pS116	3E+09	4E+09	[illegible]	1E+09	p00294	Astrocytic phosphoprotein PEA-15
AHNAK seq: 2707 - 2720 + Phospho (ST)	4E+07	2E+08	[illegible]	2E+08	p13705	Neuroblast differentiation-associated protein AHNAK
TJP2 seq: 452 - 478 + Phospho (ST)	2E+08	3E+08	[illegible]	8E+08	p10127	Tight junction protein ZO-2
DENND4A seq: 1508 - 1526 + Phospho (ST)	2E+08	2E+08	[illegible]	8E+07	p03774	C-myc promoter-binding protein
PEAK1 seq: 208 - 224 + Phospho (ST)	7E+07	9E+07	[illegible]	2E+07	p14565	Pseudopodium-enriched atypical kinase 1
EEA1 seq: 488 - 503 no mod	8E+07	8E+07	[illegible]	2E+07	p08057	Early endosome antigen 1
PHLDB1 pS563	4E+08	3E+08	[illegible]	6E+08	p12906	Pleckstrin homology-like domain family B member 1
ACD pT422 pS435	2E+07	3E+07	[illegible]	1E+08	p02314	Adrenocortical dysplasia protein homolog
KDM1A pS166	6E+08	3E+08	[illegible]	2E+08	p11093	Lysine-specific histone demethylase 1A
[illegible]seq: 414 - 427 + Gln->pyro-Glu (N-term Q); Phospho (ST)	8E+07	1E+08	[illegible]	1E+08	p08296	Protein PRRC2B
PBK seq: 55 - 64 + Phospho (ST)	4E+07	7E+07	[illegible]	6E+07	p15646	Lymphokine-activated killer T-cell-originated protein kinase
FLNB seq: 153 - 169 no mod	3E+07	8E+08	[illegible]	2E+08	p13845	Filamin-B
RBM15B pS265 pS267	1E+08	2E+08	[illegible]	7E+07	p05007	Putative RNA-binding protein 15B
DMXL2 seq: 1877 - 1882 no mod	1E+09	1E+09	[illegible]	6E+08	p13911	DmX-like protein 2
GIT2 seq: 357 - 374 + Phospho (ST)	4E+07	7E+07	[illegible]	25732	p08058	ARF GTPase-activating protein GIT2
SPTBN1 seq: 2326 - 2345 + Phospho (ST)	3E+08	4E+08	[illegible]	2E+08	p19740	Spectrin beta chain, non-erythrocytic 1
NUFIP2 pS572	6E+08	9E+08	[illegible]	1E+09	p06487	Nuclear fragile X mental retardation-interacting protein 2
MTMR3 pS975	1E+08	2E+08	[illegible]	25732	p08322	Myotubularin-related protein 3
FAM122A pS62	2E+09	2E+09	[illegible]	4E+09	p07005	Protein FAM122A
SAMHD1 pT592	38324	37149	[illegible]	6E+08	p09622	Deoxynucleoside triphosphate triphosphohydrolase SAMHD1
FAM117A pS178	7E+08	5E+08	[illegible]	5E+07	p17233	Protein FAM117A
STX17 seq: 286 - 300 + Phospho (ST)	2E+08	9E+07	[illegible]	1E+08	p10490	Syntaxin-17
PPM1H seq: 214 - 228 + Phospho (ST)	2E+09	2E+09	[illegible]	1E+08	p15945	Protein phosphatase 1H
TFAP4 seq: 116 - 127 + Phospho (ST)	2E+08	2E+08	[illegible]	2E+08	p16675	Transcription factor AP-4
TPX2 pS486	2E+07	9E+07	[illegible]	3E+08	p01197	Targeting protein for Xklp2
PRPF31 pS439 pT440	2E+08	2E+08	[illegible]	2E+08	p07817	U4/U6 small nuclear ribonucleoprotein Prp31
EIF5B pS214	5E+08	1E+09	[illegible]	2E+09	p09506	Eukaryotic translation initiation factor 5B
ANKRD34A pS461	3E+08	3E+08	[illegible]	25732	p19912	Ankyrin repeat domain-containing protein 34A
NSFL1C pS114	38324	2E+08	[illegible]	25732	p13033	NSFL1 cofactor p47
CTNND1 pS252	5E+09	9E+09	[illegible]	2E+10	p19885	Catenin delta-1
FAM83H seq: 924 - 933 + Phospho (ST)	4E+09	3E+09	[illegible]	6E+08	p03759	Protein FAM83H
POLM pS12	2E+08	1E+08	[illegible]	4E+07	p01438	DNA-directed DNA/RNA polymerase mu
SUCO pS1081	2E+08	3E+08	[illegible]	2E+08	p05366	SUN domain-containing ossification factor
ARID1A seq: 1599 - 1612 + Oxidation (M); Phospho (ST)	6E+08	5E+08	[illegible]	2E+08	p02139	AT-rich interactive domain-containing protein 1A
TANC1 pS63 pS67	1E+09	1E+09	[illegible]	3E+08	p14990	Protein TANC1
KIF13B seq: 1790 - 1810 + 2 Phospho (ST)	2E+08	5E+08	[illegible]	2E+08	p06698	Kinesin-like protein KIF13B
AHNAK seq: 5726 - 5744 + 2 Phospho (ST)	1E+09	1E+09	[illegible]	5E+08	p15940	Neuroblast differentiation-associated protein AHNAK
FRMD8 pS439	7E+07	7E+07	[illegible]	2E+07	p02020	FERM domain-containing protein 8
CCNE2 seq: 12 - 31 + Phospho (ST)	2E+08	2E+08	[illegible]	1E+08	p08035	G1/S-specific cyclin-E2
INTS12 seq: 120 - 136 + Phospho (ST)	2E+07	2E+07	[illegible]	1E+07	p11970	Integrator complex subunit 12
KRT18 seq: 7 - 14 + Phospho (ST)	2E+10	2E+10	[illegible]	1E+10	p03531	Keratin, type I cytoskeletal 18
EPN3 seq: 436 - 464 + Phospho (ST)	6E+07	1E+08	[illegible]	25732	p17727	Epsin-3
ZFPM2 pS1031	5E+07	1E+08	[illegible]	25732	p18253	Zinc finger protein ZFPM2
TNRC18 pS1127	38324	37149	[illegible]	25732	p12231	Trinucleotide repeat-containing gene 18 protein
SPATS2L seq: 437 - 445 no mod	2E+07	9E+07	[illegible]	1E+08	p16550	SPATS2-like protein
ABCF1 seq: 104 - 121 + 2 Phospho (ST)	1E+09	2E+09	[illegible]	1E+09	p10598	ATP-binding cassette sub-family F member 1
ASPSCR1 seq: 272 - 283 + Phospho (ST)	3E+08	4E+08	[illegible]	9E+07	p04992	Tether containing UBX domain for GLUT4
OSBPL11 pS181	3E+07	8E+06	[illegible]	7E+07	p05839	Oxysterol-binding protein-related protein 11
CNOT2 pM89 pT93	2E+08	1E+08	[illegible]	4E+07	p15603	CCR4-NOT transcription complex subunit 2
SUPT5H seq: 669 - 681 + Phospho (ST)	1E+08	2E+08	[illegible]	8E+07	p13655	Transcription elongation factor SPT5
ABCF1 pS109	4E+08	6E+08	[illegible]	2E+08	p10597	ATP-binding cassette sub-family F member 1
NEDD4L seq: 338 - 362 + Phospho (ST)	3E+08	6E+08	[illegible]	5E+08	p10869	E3 ubiquitin-protein ligase NEDD4-like
TSC2 pS939	7E+07	1E+08	[illegible]	5E+07	p03452	Tuberin
BAZ1B pS349	5E+07	7E+08	[illegible]	3E+08	p05054	Tyrosine-protein kinase BAZ1B
CLASP1 seq: 555 - 570 + Phospho (ST)	38324	37149	[illegible]	7E+07	p03628	CLIP-associating protein 1
[illegible]seq: 285 - 294 + Gln->pyro-Glu (N-term Q); Phospho (ST)	8E+07	6E+07	[illegible]	4E+07	p07933	Pre-mRNA-splicing regulator WTAP
MICALL2 pS294	1E+08	1E+08	[illegible]	3E+08	p19693	MICAL-like protein 2
LGALS1 seq: 65 - 74 no mod	8E+07	8E+07	[illegible]	4E+08	p18829	Galectin-1
VPS13C pS3641	1E+09	1E+09	[illegible]	25732	p00329	Vacuolar protein sorting-associated protein 13C
DDX46 pS804 pM820	7E+08	1E+09	[illegible]	7E+08	p21100	Probable ATP-dependent RNA helicase DDX46
SRRM2 seq: 2098 - 2113 + Phospho (ST)	9E+08	1E+09	[illegible]	5E+08	p09580	Serine/arginine repetitive matrix protein 2
TANC2 pS400	1E+08	1E+08	[illegible]	733229	p08551	Protein TANC2
CDK13 pS525	8E+08	8E+08	[illegible]	2E+08	p14354	Cyclin-dependent kinase 13
MAST2 pS66	3E+08	3E+08	[illegible]	25732	p17293	Microtubule-associated serine/threonine-protein kinase 2
KIAA1522 seq: 160 - 172 + Phospho (ST)	2E+08	4E+08	[illegible]	5E+08	p06549	Uncharacterized protein KIAA1522
PPIG pS687	2E+09	3E+09	[illegible]	7E+06	p13464	Peptidyl-prolyl cis-trans isomerase G
CASKIN2 seq: 251 - 271 + Phospho (Y)	2E+08	3E+08	[illegible]	1E+08	p08992	Caskin-2
DBNL pS232	8E+08	7E+08	[illegible]	8E+08	p00164	Drebrin-like protein
CTNND1 seq: 859 - 875 + Phospho (ST)	2E+08	2E+08	[illegible]	8E+08	p04164	Catenin delta-1
MARCKS pS163 pS167	38324	5E+06	[illegible]	6E+07	p05895	Myristoylated alanine-rich C-kinase substrate
AHNAK pS5731	7E+08	8E+08	[illegible]	5E+08	p15939	Neuroblast differentiation-associated protein AHNAK
WWC3 seq: 456 - 477 + Phospho (ST)	5E+08	7E+08	[illegible]	25732	p03835	Protein WWC3
FLYWCH2 seq: 16 - 34 + Phospho (ST)	2E+08	1E+08	[illegible]	8E+07	p19522	FLYWCH family member 2
SEC14L1 seq: 215 - 246 + 2 Phospho (ST)	2E+08	1E+08	[illegible]	25732	p17720	SEC14-like protein 1
ANKLE2 pS662	3E+09	6E+09	[illegible]	2E+09	p09293	Ankyrin repeat and LEM domain-containing protein 2
PTGES3 seq: 26 - 33 no mod	6E+07	4E+07	[illegible]	1E+07	p18101	Prostaglandin E synthase 3
UBAP2L pS609	6E+09	7E+09	[illegible]	9E+09	p06401	Ubiquitin-associated protein 2-like
CTTN seq: 397 - 414 + Phospho (ST)	2E+09	2E+09	[illegible]	1E+09	p20353	Src substrate cortactin
LUZP1 seq: 943 - 967 + Phospho (ST)	9E+07	9E+07	[illegible]	2E+08	p08979	Leucine zipper protein 1
ANXA2 pT19	5E+08	5E+08	[illegible]	1E+09	p10765	Annexin A2
SSFA2 seq: 590 - 608 + Phospho (ST)	1E+09	7E+08	[illegible]	1E+08	p12583	Sperm-specific antigen 2
EIF4B pS359	8E+07	1E+08	[illegible]	5E+07	p21040	Eukaryotic translation initiation factor 4B
TNKS1BP1 pS1652	1E+09	2E+09	[illegible]	2E+09	p01065	182 kDa tankyrase-1-binding protein
ASPSCR1 pS275	1E+09	1E+09	[illegible]	8E+08	p04993	Tether containing UBX domain for GLUT4
FBRSL1 seq: 1032 - 1045 + Phospho (ST)	6E+08	6E+08	[illegible]	4E+08	p18034	Fibrosin-1-like protein
PLEKHG3 pS614	3E+08	3E+08	[illegible]	2E+08	p16377	Pleckstrin homology domain-containing family G member 3
TRIM36 seq: 83 - 95 + Phospho (ST)	2E+08	3E+08	[illegible]	25732	p10886	E3 ubiquitin-protein ligase TRIM36
NSMCE2 pS116	5E+07	4E+07	[illegible]	4E+07	p16636	E3 SUMO-protein ligase NSE2
CCDC85C pS246	3E+09	2E+09	[illegible]	6E+08	p05306	Coiled-coil domain-containing protein 85C
ARHGAP29 seq: 1142 - 1153 + Phospho (Y)	6E+07	5E+07	[illegible]	2E+08	p06617	Rho GTPase-activating protein 29
KLHDC4 seq: 400 - 432 + 2 Phospho (ST)	1E+08	1E+08	[illegible]	2E+08	p07716	Kelch domain-containing protein 4
ARHGAP29 seq: 1142 - 1153 + Phospho (ST)	6E+07	5E+07	[illegible]	2E+08	p06618	Rho GTPase-activating protein 29
BCL7B seq: 105 - 130 + Phospho (ST)	2E+08	2E+08	[illegible]	5E+08	p01655	B-cell CLL/lymphoma 7 protein family member B
MAP7D3 seq: 183 - 198 + Phospho (ST)	38324	1E+07	[illegible]	3E+08	p06218	MAP7 domain-containing protein 3
BAG3 seq: 171 - 193 + Phospho (ST)	2E+09	1E+09	[illegible]	4E+08	p04177	BAG family molecular chaperone regulator 3
RSF1 pS397	1E+08	2E+08	[illegible]	2E+08	p10832	Remodeling and spacing factor 1
ABI2 pS227	1E+09	9E+08	[illegible]	3E+08	p09381	Abl interactor 2
NPM1 pS125	2E+08	2E+09	[illegible]	1E+10	p19051	Nucleophosmin
ARFGAP1 seq: 360 - 374 + Phospho (ST)	4E+07	4E+07	[illegible]	9E+06	p03929	ADP-ribosylation factor GTPase-activating protein 1
PDLIM2 pS266	8E+07	7E+07	[illegible]	2E+08	p15949	PDZ and LIM domain protein 2
ALDOA seq: 2 - 13 + Phospho (Y)	38324	37149	[illegible]	25732	p08878	Fructose-bisphosphate aldolase A
SSFA2 pS330	3E+08	4E+08	[illegible]	5E+07	p14012	Sperm-specific antigen 2
SRSF1 seq: 192 - 204 + Phospho (ST)	9E+09	2E+10	[illegible]	9E+09	p01287	Serine/arginine-rich splicing factor 1
RPS8 seq: 129 - 139 no mod	2E+08	8E+07	[illegible]	5E+07	p10529	40S ribosomal protein S8
EHMT1 seq: 433 - 442 + Phospho (ST)	4E+08	3E+08	[illegible]	1E+08	p02948	Histone-lysine N-methyltransferase EHMT1
HSPA1B seq: 77 - 88 no mod	5E+07	1E+08	[illegible]	1E+07	p13247	Heat shock 70 kDa protein 1B
PRRC2B seq: 216 - 233 + Phospho (ST)	1E+09	1E+09	[illegible]	7E+08	p14771	Protein PRRC2B
HID1 seq: 591 - 614 + Oxidation (M); Phospho (ST)	4E+09	3E+09	[illegible]	25732	p02783	Protein HID1
ETFA seq: 76 - 85 no mod	9E+07	3E+08	[illegible]	1E+07	p01127	Electron transfer flavoprotein subunit alpha, mitochondrial
C2CD5 pS852	4E+08	3E+08	[illegible]	2E+08	p20065	C2 domain-containing protein 5
TANC2 pS1545	1E+09	1E+09	[illegible]	1E+08	p19541	Protein TANC2
DNTTIP2 seq: 322 - 338 + Phospho (ST)	38324	37149	[illegible]	4E+07	p09416	Deoxynucleotidyltransferase terminal-interacting protein 2
NEDD4L seq: 446 - 462 + 2 Phospho (ST)	2E+08	1E+08	[illegible]	2E+08	p04989	E3 ubiquitin-protein ligase NEDD4-like
IRF2BPL pS659	1E+09	9E+08	[illegible]	5E+08	p09068	Interferon regulatory factor 2-binding protein-like
ZC3H4 pS1275	5E+08	4E+08	[illegible]	2E+08	p02793	Zinc finger CCCH domain-containing protein 4
CDC42EP2 pS109	1E+07	8E+07	[illegible]	1E+09	p09774	Cdc42 effector protein 2
TBC1D4 pS591	4E+08	3E+08	[illegible]	4E+08	p11677	TBC1 domain family member 4
LRWD1 pS212	9E+08	7E+08	[illegible]	25732	p20028	Leucine-rich repeat and WD repeat-containing protein 1
ASAP2 seq: 820 - 835 + Phospho (ST)	1E+09	1E+09	[illegible]	3E+08	p03724	Arf-GAP with SH3 domain, ANK repeat and PH domain-containing protein 2
MLPH pS510	2E+08	2E+08	[illegible]	25732	p04841	Melanophilin
CTTNBP2 seq: 272 - 283 + Phospho (ST)	4E+07	4E+07	[illegible]	25732	p15299	Cortactin-binding protein 2
LPIN2 seq: 243 - 257 + Phospho (ST)	2E+08	3E+08	[illegible]	1E+08	p06089	Phosphatidate phosphatase LPIN2
FKBP15 seq: 975 - 1008 + Phospho (ST)	38324	1E+08	[illegible]	25732	p17223	FK506-binding protein 15
LASP1 seq: 131 - 144 no mod	2E+08	2E+08	[illegible]	2E+07	p10099	LIM and SH3 domain protein 1
FKBP4 seq: 190 - 206 no mod	2E+08	4E+08	[illegible]	1E+08	p16795	Peptidyl-prolyl cis-trans isomerase FKBP4
CTNND1 pS847	3E+08	3E+08	[illegible]	7E+07	p06109	Catenin delta-1
STMN1 pS38	38324	2E+07	[illegible]	1E+08	p17092	Stathmin
MARK2 pS456	3E+09	3E+09	[illegible]	1E+09	p01026	Serine/threonine-protein kinase MARK2
DNAJB1 seq: 250 - 259 no mod	5E+07	5E+07	[illegible]	25732	p18798	DnaJ homolog subfamily B member 1
DNAJC9 pS109	5E+07	7E+07	[illegible]	3E+08	p13069	DnaJ homolog subfamily C member 9
SCRIB seq: 1292 - 1318 + Phospho (ST)	1E+09	2E+09	[illegible]	8E+08	p10271	Protein scribble homolog
EIF4B seq: 418 - 435 + Phospho (ST)	1E+07	1E+07	[illegible]	6E+07	p03986	Eukaryotic translation initiation factor 4B
PPP1R13L pS395	1E+09	1E+09	[illegible]	8E+08	p20082	RelA-associated inhibitor
ZNF687 seq: 422 - 435 + Phospho (ST)	8E+07	2E+08	[illegible]	6E+07	p13362	Zinc finger protein 687
LAD1 seq: 120 - 136 + Phospho (ST)	1E+08	3E+08	[illegible]	4E+08	p09142	Ladinin-1
VCL seq: 282 - 300 + Phospho (ST)	1E+08	7E+07	[illegible]	2E+07	p14954	Vinculin
LUZP1 pS745	2E+08	3E+08	[illegible]	9E+08	p04229	Leucine zipper protein 1
FAM83E pS319 pS324	2E+08	1E+08	[illegible]	25732	p06715	Protein FAM83E
CEP170B pS829	5E+08	5E+08	[illegible]	9E+08	p12778	Centrosomal protein of 170 kDa protein B
CAST seq: 212 - 250 + Phospho (ST)	9E+07	1E+08	[illegible]	1E+09	p13293	Calpastatin
LASP1 seq: 74 - 85 no mod	6E+08	9E+08	[illegible]	1E+08	p11493	LIM and SH3 domain protein 1
HSD17B10 seq: 53 - 69 no mod	5E+08	7E+08	[illegible]	25732	p12975	3-hydroxyacyl-CoA dehydrogenase type-2
ZNF318 pS1896	3E+07	2E+08	[illegible]	6E+07	p13759	Zinc finger protein 318
YNC1H1 seq: 4366 - 4378 + Oxidation (M); Phospho (ST)	6E+08	3E+08	[illegible]	4E+07	p02941	Cytoplasmic dynein 1 heavy chain 1
BIN1 seq: 283 - 311 + Phospho (ST)	38324	37149	[illegible]	3E+08	p19776	Myc box-dependent-interacting protein 1
PRRC2B seq: 216 - 233 + Phospho (ST)	3E+08	4E+08	[illegible]	2E+08	p14772	Protein PRRC2B
TNRC18 pS263	3E+07	1E+08	[illegible]	25732	p12272	Trinucleotide repeat-containing gene 18 protein
SNX2 pS185	38324	3E+07	[illegible]	7E+06	p07441	Sorting nexin-2
ADD2 seq: 722 - 729 + Phospho (ST)	2E+08	4E+08	[illegible]	1E+08	p16496	Beta-adducin
LUZP1 pS995	5E+07	7E+07	[illegible]	3E+08	p02254	Leucine zipper protein 1
NFAT5 seq: 558 - 573 + Phospho (ST)	38324	37149	[illegible]	2E+08	p17596	Nuclear factor of activated T-cells 5
ASPH seq: 458 - 474 no mod	38324	7E+07	[illegible]	3E+07	p09706	Aspartyl/asparaginyl beta-hydroxylase
ANKLE2 seq: 509 - 521 + Phospho (ST)	4E+07	1E+08	[illegible]	1E+08	p00371	Ankyrin repeat and LEM domain-containing protein 2
TBX3 pS409	8E+08	9E+08	[illegible]	25732	p13639	T-box transcription factor TBX3
SVIL pS968	6E+08	3E+08	[illegible]	2E+08	p00344	Supervillin
PRKCD seq: 505 - 524 + Phospho (ST)	4E+08	4E+08	[illegible]	3E+07	p19451	Protein kinase C delta type
SCGB1D2 seq: 75 - 83 no mod	7E+07	4E+08	[illegible]	25732	p05186	Secretoglobin family 1D member 2
IDH1 seq: 271 - 301 no mod	3E+06	9E+07	[illegible]	7E+07	p08926	Isocitrate dehydrogenase [NADP] cytoplasmic
AHNAK seq: 1465 - 1481 + Phospho (Y)	38324	37149	[illegible]	1E+09	p10051	Neuroblast differentiation-associated protein AHNAK
STXBP5 seq: 897 - 921 + Phospho (ST)	1E+08	7E+07	[illegible]	25732	p06823	Syntaxin-binding protein 5
PCF11 pS777	9E+07	1E+08	[illegible]	6E+07	p10074	Pre-mRNA cleavage complex 2 protein Pcf11

Peptide	Values	Accession	Protein
DGCR8 pS95	5E+08 5E+08 5E+08 5E+08 4E+08 45779 7E+08 5E+08 5E+08 4E+08 9E+08 5E+08 4E+08 3E+08 3E+08 7E+08 6E+08 6E+08 4E+08 5E+08 9E+08 6E+08 4E+08 4E+08 4E+08 1E+09 1E+09 [illegible]	p11383	Microprocessor complex subunit DGCR8
CD3EAP seq: 163 - 175 + Phospho (ST)	2E+07 4E+07 3E+07 4E+07 2E+07 45779 4E+07 4E+07 2E+07 3E+07 4E+07 2E+07 2E+07 2E+07 1E+07 4E+06 3E+07 4E+07 3E+07 5E+07 7E+07 2E+07 2E+07 3E+07 4E+07 2E+08 9E+07 [illegible]	p06321	DNA-directed RNA polymerase I subunit RPA34
ABLIM2 seq: 474 - 497 + Phospho (ST)	2E+08 2E+08 2E+08 2E+08 2E+08 4E+08 3E+08 1E+08 1E+08 1E+08 7E+08 2E+08 2E+08 2E+08 2E+08 2E+08 1E+08 1E+08 2E+08 7E+07 2E+08 1E+08 1E+08 1E+08 1E+08 68554 44396 [illegible]	p02456	Actin-binding LIM protein 2
EIF4EBP2 seq: 21 - 51 + Phospho (ST); Phospho (Y)	2E+09 2E+09 3E+09 2E+09 3E+09 45779 3E+09 2E+09 2E+09 2E+09 56234 3E+09 3E+09 4E+09 3E+09 40867 2E+09 3E+09 3E+09 2E+09 4E+09 3E+09 3E+09 3E+09 3E+09 9E+09 4E+09 [illegible]	p01987	Eukaryotic translation initiation factor 4E-binding protein 2
SERPINH1 seq: 309 - 319 no mod	2E+07 4E+07 9E+07 7E+06 3E+07 45779 1E+07 4E+07 2E+07 9E+06 1E+07 9E+06 8E+07 2E+07 5E+06 40867 9E+06 2E+07 4E+07 2E+06 39759 2E+07 2E+07 2E+07 8E+06 68554 44396 [illegible]	p14971	Serpin H1
NOLC1 pT607 pT610	3E+08 3E+08 3E+08 2E+08 2E+08 45779 3E+08 2E+08 2E+08 2E+08 56234 2E+08 1E+08 2E+08 2E+08 40867 4E+08 4E+08 3E+08 3E+08 2E+07 3E+08 3E+08 2E+08 2E+08 68554 9E+08 [illegible]	p10926	Nucleolar and coiled-body phosphoprotein 1
TRIP12 pS312	4E+08 1E+09 1E+09 7E+08 8E+08 1E+09 2E+09 9E+08 6E+08 9E+08 8E+08 1E+09 7E+08 8E+08 8E+08 9E+08 1E+09 1E+09 8E+08 7E+08 1E+09 1E+09 9E+08 6E+08 6E+08 1E+09 9E+08 [illegible]	p05971	E3 ubiquitin-protein ligase TRIP12
KNOP1 pS42	1E+09 1E+09 1E+09 9E+08 8E+08 1E+09 1E+09 1E+09 9E+08 9E+08 1E+09 9E+08 7E+08 7E+08 8E+08 1E+09 1E+09 9E+08 9E+08 9E+08 1E+09 2E+09 9E+08 9E+08 8E+08 4E+08 5E+08 [illegible]	p00058	Lysine-rich nucleolar protein 1
LIMA1 pS617	2E+08 2E+08 4E+08 4E+08 4E+08 2E+09 5E+08 3E+08 4E+08 2E+08 1E+09 4E+08 3E+08 3E+08 2E+08 3E+08 2E+08 5E+08 3E+08 2E+08 4E+08 1E+08 2E+08 2E+08 2E+08 1E+08 2E+07 [illegible]	p13130	LIM domain and actin-binding protein 1
ZFP36L1 pS334	4E+08 9E+08 1E+09 8E+08 8E+08 1E+09 1E+09 1E+09 7E+08 8E+08 1E+09 1E+09 1E+09 1E+09 7E+08 5E+08 5E+08 8E+08 6E+08 5E+08 5E+08 1E+09 9E+08 8E+08 6E+08 7E+08 5E+08 [illegible]	p11118	Zinc finger protein 36, C3H1 type-like 1
HJURP seq: 116 - 143 + Phospho (ST)	1E+07 37149 1E+07 7E+06 1E+07 45779 25552 2E+07 34104 8E+06 56234 53206 2E+07 40879 18798 40867 2E+06 1E+07 1E+07 28832 39759 9E+06 2E+07 7E+06 42741 9E+07 1E+08 [illegible]	p05767	Holliday junction recognition protein
ZFP91 seq: 81 - 108 + 2 Phospho (ST)	1E+08 3E+08 4E+08 3E+08 2E+08 5E+08 5E+08 4E+08 3E+08 3E+08 5E+08 3E+08 4E+08 4E+08 3E+08 3E+08 2E+08 4E+08 2E+08 2E+08 4E+08 3E+08 2E+08 3E+08 2E+08 1E+09 8E+08 [illegible]	p06587	E3 ubiquitin-protein ligase ZFP91
MAP4K4 pS639	3E+08 3E+08 3E+08 2E+08 2E+08 5E+08 3E+08 2E+08 2E+08 1E+08 4E+08 3E+08 2E+08 1E+08 2E+08 4E+08 2E+08 2E+08 2E+08 1E+08 3E+08 2E+08 2E+08 1E+08 2E+08 7E+08 4E+08 [illegible]	p07502	Mitogen-activated protein kinase kinase kinase kinase 4
AHNAK seq: 1727 - 1738 no mod	2E+08 2E+08 4E+08 9E+07 2E+08 6E+08 1E+08 2E+08 1E+08 8E+07 2E+09 1E+08 4E+08 9E+07 9E+07 1E+08 7E+07 1E+08 2E+08 3E+07 1E+08 7E+07 1E+08 1E+08 9E+07 3E+08 1E+08 [illegible]	p20835	Neuroblast differentiation-associated protein AHNAK
EEF2 seq: 581 - 594 no mod	1E+08 7E+07 1E+08 13654 8E+07 4E+08 4E+07 6E+07 3E+07 3E+07 1E+09 53206 2E+08 40879 3E+07 1E+08 3E+07 34984 9E+07 28832 1E+08 2E+07 21442 4E+07 42741 4E+08 2E+07 [illegible]	p17029	Elongation factor 2
MORC2 seq: 851 - 893 + Phospho (ST)	2E+08 3E+08 3E+08 2E+08 3E+08 1E+09 7E+08 2E+08 3E+08 3E+08 7E+08 7E+08 3E+08 4E+08 4E+08 4E+08 1E+08 3E+08 3E+08 2E+08 5E+08 50401 2E+08 4E+08 3E+08 7E+08 44396 [illegible]	p11227	MORC family CW-type zinc finger protein 2
ABL1 seq: 264 - 271 + Phospho (ST)	1E+08 1E+08 2E+08 2E+08 1E+08 4E+08 2E+08 2E+08 1E+08 7E+07 3E+08 2E+08 2E+08 9E+07 8E+07 2E+08 1E+08 3E+08 2E+08 9E+07 2E+08 7E+07 1E+08 7E+07 6E+07 3E+08 2E+08 [illegible]	p00101	Tyrosine-protein kinase ABL1
UBA1 seq: 351 - 368 no mod	2E+08 3E+08 2E+08 2E+08 3E+08 7E+08 3E+08 3E+08 2E+08 2E+08 1E+09 3E+08 2E+08 2E+08 2E+08 3E+08 2E+08 2E+08 2E+08 2E+08 2E+08 2E+08 2E+08 2E+08 2E+08 4E+07 44396 [illegible]	p09688	Ubiquitin-like modifier-activating enzyme 1
TRIP10 pS482	9E+08 1E+09 1E+09 8E+08 1E+09 2E+09 1E+09 1E+09 8E+08 6E+08 1E+09 1E+09 8E+08 7E+08 8E+08 9E+08 1E+09 1E+09 6E+08 6E+08 8E+08 1E+09 1E+09 7E+08 9E+08 3E+09 2E+09 [illegible]	p01173	Cdc42-interacting protein 4
HSPA8 seq: 4 - 25 no mod	9E+06 2E+08 5E+08 13654 3E+08 7E+08 2E+08 3E+08 3E+08 26736 1E+09 3E+08 4E+08 2E+07 1E+07 2E+08 9E+07 34984 3E+08 28832 5E+07 50401 3E+08 2E+07 7E+07 2E+08 5E+07 [illegible]	p15530	Heat shock cognate 71 kDa protein
PDXDC1 seq: 713 - 734 + 2 Phospho (ST)	4E+08 4E+08 4E+08 5E+08 3E+08 1E+09 5E+08 3E+08 4E+08 2E+08 2E+08 4E+08 4E+08 5E+08 3E+08 7E+08 3E+08 5E+08 5E+08 3E+08 7E+08 3E+08 3E+08 4E+08 3E+08 4E+08 1E+08 [illegible]	p15306	Pyridoxal-dependent decarboxylase domain-containing protein 1
LMNA seq: 387 - 397 + 2 Phospho (ST)	4E+09 3E+09 4E+09 3E+09 2E+09 9E+09 4E+09 3E+09 2E+09 2E+09 6E+09 3E+09 3E+09 2E+09 2E+09 4E+09 3E+09 4E+09 2E+09 2E+09 6E+09 3E+09 3E+09 2E+09 2E+09 4E+10 2E+10 [illegible]	p10883	Prelamin-A/C
ABCF1 pS109	2E+09 4E+09 4E+09 3E+09 3E+09 8E+09 5E+09 3E+09 3E+09 3E+09 5E+09 4E+09 4E+09 4E+09 3E+09 5E+09 3E+09 4E+09 3E+09 2E+09 5E+09 2E+09 3E+09 3E+09 2E+09 8E+09 2E+09 [illegible]	p10596	ATP-binding cassette sub-family F member 1
RPS6KA6 pS227	3E+08 2E+08 3E+08 3E+08 3E+08 2E+08 5E+08 2E+08 2E+08 2E+08 8E+08 4E+08 3E+08 4E+08 3E+08 4E+08 2E+08 3E+08 3E+08 2E+08 3E+08 2E+08 2E+08 3E+08 3E+08 2E+09 9E+08 [illegible]	p19107	Ribosomal protein S6 kinase alpha-6
PRR12 pS44	2E+08 6E+08 4E+08 2E+08 2E+08 6E+08 8E+08 4E+08 2E+08 2E+08 4E+08 4E+08 3E+08 2E+08 2E+08 6E+08 3E+08 4E+08 3E+08 2E+08 5E+08 4E+08 4E+08 2E+08 2E+08 4E+08 2E+08 [illegible]	p05605	Proline-rich protein 12
NOLC1 pS397	6E+08 7E+08 7E+08 8E+08 6E+08 6E+08 1E+09 6E+08 7E+08 5E+08 6E+08 7E+08 4E+08 6E+08 3E+08 5E+08 8E+08 8E+08 1E+09 6E+08 5E+08 7E+08 5E+08 7E+08 5E+08 2E+09 2E+09 [illegible]	p09076	Nucleolar and coiled-body phosphoprotein 1
FOXK1 seq: 431 - 456 + 2 Phospho (ST)	3E+08 4E+08 7E+08 6E+08 6E+08 1E+09 7E+08 5E+08 5E+08 5E+08 8E+08 5E+08 6E+08 5E+08 6E+08 6E+08 4E+08 5E+08 6E+08 5E+08 7E+08 6E+08 4E+08 5E+08 5E+08 2E+09 8E+08 [illegible]	p05748	Forkhead box protein K1
FLYWCH2 seq: 16 - 34 + Phospho (ST)	2E+09 2E+09 2E+09 1E+09 2E+09 6E+09 2E+09 2E+09 2E+09 1E+09 4E+09 2E+09 1E+09 1E+09 1E+09 3E+09 2E+09 2E+09 2E+09 1E+09 4E+09 2E+09 2E+09 2E+09 1E+09 4E+09 2E+09 [illegible]	p19521	FLYWCH family member 2
CAMSAP1 pS416	[illegible]	p15128	Calmodulin-regulated spectrin-associated protein 1
HSPA8 seq: 417 - 447 no mod	[illegible]	p08997	Heat shock cognate 71 kDa protein
ACTN4 seq: 366 - 378 no mod	[illegible]	p10722	Alpha-actinin-4
RPGR pS961	[illegible]	p06454	X-linked retinitis pigmentosa GTPase regulator
TXLNA seq: 18 - 53 + Phospho (ST)	[illegible]	p03810	Alpha-taxilin
SH3BP1 pS262	[illegible]	p17339	SH3 domain-binding protein 1
CORO7 pS465	[illegible]	p05078	Coronin-7
RANBP3 seq: 337 - 378 + Phospho (ST)	[illegible]	p11193	Ran-binding protein 3
HDGFRP2 pS664	[illegible]	p19715	Hepatoma-derived growth factor-related protein 2
SNTB1 pS87	[illegible]	p16327	Beta-1-syntrophin
CCDC120 pS331	[illegible]	p07674	Coiled-coil domain-containing protein 120
TRIP12 seq: 309 - 321 + Phospho (ST)	[illegible]	p06685	E3 ubiquitin-protein ligase TRIP12
KIAA1522 seq: 921 - 938 + Phospho (ST)	[illegible]	p12935	Uncharacterized protein KIAA1522
PEA15 pS116	[illegible]	p18750	Astrocytic phosphoprotein PEA-15
SFSWAP pS909	[illegible]	p04108	Splicing factor, suppressor of white-apricot homolog
MID1 pS96	[illegible]	p19439	E3 ubiquitin-protein ligase Midline-1
FAM21C pS728	[illegible]	p17030	WASH complex subunit FAM21C
RBMX pS88	[illegible]	p01603	RNA-binding motif protein, X chromosome
RAB11FIP1 seq: 229 - 245 + Phospho (ST)	[illegible]	p02371	Rab11 family-interacting protein 1
ELMSAN1 seq: 698 - 722 + Phospho (ST)	[illegible]	p02470	ELM2 and SANT domain-containing protein 1
NCOR2 seq: 937 - 952 + Phospho (ST)	[illegible]	p11279	Nuclear receptor corepressor 2
MLLT4 seq: 1197 - 1228 + Phospho (ST)	[illegible]	p13576	Afadin
HIST1H4A seq: 69 - 78 no mod	[illegible]	p18938	Histone H4
JUP pT54	[illegible]	p07662	Junction plakoglobin
CTNNB1 seq: 550 - 565 + Oxidation (M); Phospho (ST)	[illegible]	p06491	Catenin beta-1
CDK13 pT871	[illegible]	p10289	Cyclin-dependent kinase 13
DLGAP4 pS744	[illegible]	p05124	Disks large-associated protein 4
KAT6A pS1113	[illegible]	p12578	Histone acetyltransferase KAT6A
SRRM2 seq: 1854 - 1863 + Phospho (ST)	[illegible]	p03962	Serine/arginine repetitive matrix protein 2
EPB41L2 seq: 87 - 94 + Phospho (ST)	[illegible]	p03117	Band 4.1-like protein 2
ARHGAP35 pS1150	[illegible]	p12399	Rho GTPase-activating protein 35
SKIL seq: 504 - 519 + Phospho (ST)	[illegible]	p01473	Ski-like protein
ZNF609 pS1055	[illegible]	p19911	Zinc finger protein 609
HIST2H2AC seq: 90 - 100 no mod	[illegible]	p09716	Histone H2A type 2-C
CCDC86 seq: 31 - 53 + Phospho (ST)	[illegible]	p20117	Coiled-coil domain-containing protein 86
MLPH pS337	[illegible]	p19507	Melanophilin
EPN1 pT470	[illegible]	p12484	Epsin-1
UFD1L pS247	[illegible]	p15044	Ubiquitin fusion degradation protein 1 homolog
PDLIM2 pS266	[illegible]	p15950	PDZ and LIM domain protein 2
SIK3 pT411	[illegible]	p07285	Serine/threonine-protein kinase SIK3
NUMA1 pS1969	[illegible]	p07573	Nuclear mitotic apparatus protein 1
PDZD2 pS568	[illegible]	p04969	PDZ domain-containing protein 2
ZNF768 seq: 158 - 169 + Phospho (ST)	[illegible]	p02257	Zinc finger protein 768
LATS1 pS613	[illegible]	p08486	Serine/threonine-protein kinase LATS1
INPP5F seq: 905 - 931 + Phospho (ST)	[illegible]	p04183	Phosphatidylinositide phosphatase SAC2
FOXK1 pS445	[illegible]	p17676	Forkhead box protein K1
SET seq: 165 - 177 no mod	[illegible]	p17799	Protein SET
TJP1 seq: 923 - 955 + 2 Phospho (ST)	[illegible]	p20866	Tight junction protein ZO-1
CASKIN2 pS403	[illegible]	p01465	Caskin-2
HNRNPM pM607 pS618	[illegible]	p10117	Heterogeneous nuclear ribonucleoprotein M
BAIAP2 pT340	[illegible]	p10824	Brain-specific angiogenesis inhibitor 1-associated protein 2
UNG seq: 50 - 73 + 2 Phospho (ST)	[illegible]	p13418	Uracil-DNA glycosylase
TBX3 pS680	[illegible]	p01844	T-box transcription factor TBX3
ACIN1 pT414	[illegible]	p19568	Apoptotic chromatin condensation inducer in the nucleus
LIMK1 seq: 296 - 315 + 2 Phospho (ST)	[illegible]	p06137	LIM domain kinase 1
SPECC1L seq: 830 - 842 + Phospho (ST)	[illegible]	p06565	Cytospin-A
ANKRD50 pS1352	[illegible]	p04233	Ankyrin repeat domain-containing protein 50
KIAA1522 seq: 161 - 172 + Phospho (ST)	[illegible]	p03404	Uncharacterized protein KIAA1522
TPI1 pS58	[illegible]	p12644	Triosephosphate isomerase
SYNRG seq: 930 - 946 + Oxidation (M); Phospho (ST)	[illegible]	p17039	Synergin gamma
EFS pS319	[illegible]	p06918	Embryonal Fyn-associated substrate
ZCCHC8 pT479 pT485	[illegible]	p15080	Zinc finger CCHC domain-containing protein 8
ESYT2 seq: 688 - 719 + Phospho (ST)	[illegible]	p05590	Extended synaptotagmin-2
FOXO3 seq: 272 - 290 + Phospho (ST)	[illegible]	p21090	Forkhead box protein O3
CALR seq: 56 - 73 no mod	[illegible]	p16365	Calreticulin
PPP1R12A pS903	[illegible]	p00396	Protein phosphatase 1 regulatory subunit 12A
ANK3 pS1459	[illegible]	p06803	Ankyrin-3
TBC1D4 pS588	[illegible]	p15345	TBC1 domain family member 4
CTPS1 seq: 565 - 591 + Phospho (ST); Phospho (Y)	[illegible]	p18138	CTP synthase 1
SFSWAP pS909	[illegible]	p14985	Splicing factor, suppressor of white-apricot homolog
HSPA5 seq: 82 - 96 no mod	[illegible]	p09223	78 kDa glucose-regulated protein
RALGAPA1 pT754	[illegible]	p08421	Ral GTPase-activating protein subunit alpha-1
ATRX seq: 590 - 616 + Phospho (ST)	[illegible]	p10510	Transcriptional regulator ATRX
BAD seq: 73 - 94 + Phospho (ST)	[illegible]	p14599	Bcl2-associated agonist of cell death
HNRNPL pS52	[illegible]	p00009	Heterogeneous nuclear ribonucleoprotein L
HIST1H2AJ seq: 90 - 100 no mod	[illegible]	p09715	Histone H2A type 1-J
SAMD1 pT107	[illegible]	p07402	Atherin
PKN2 pS360	[illegible]	p19300	Serine/threonine-protein kinase N2
UBE2O pS401	[illegible]	p13938	(E3-independent) E2 ubiquitin-conjugating enzyme
RHPN2 seq: 547 - 566 + Phospho (ST)	[illegible]	p08449	Rhophilin-2
CCDC88C seq: 1578 - 1598 + Phospho (ST)	[illegible]	p09000	Protein Daple
RAB11FIP1 seq: 181 - 211 + Phospho (ST)	[illegible]	p09521	Rab11 family-interacting protein 1
OGFR pS484	[illegible]	p01697	Opioid growth factor receptor
ZC3H13 pS242	[illegible]	p12474	Zinc finger CCCH domain-containing protein 13
STX17 seq: 286 - 300 + Phospho (ST)	[illegible]	p10489	Syntaxin-17
RC3H2 seq: 980 - 996 + Phospho (ST)	[illegible]	p13120	Roquin-2
TCF3 pS379	[illegible]	p20716	Transcription factor E2-alpha
HMGXB4 seq: 115 - 129 + Phospho (ST)	[illegible]	p20418	HMG domain-containing protein 4
HOMEZ pT451	[illegible]	p20793	Homeobox and leucine zipper protein Homez
ARAF pS257	[illegible]	p15209	Serine/threonine-protein kinase A-Raf
MYH9 seq: 1182 - 1191 no mod	[illegible]	p02755	Myosin-9
NAP1L1 seq: 37 - 55 no mod	[illegible]	p08264	Nucleosome assembly protein 1-like 1
MCM4 pS120	[illegible]	p07975	DNA replication licensing factor MCM4
TPT1 pS46 pM74	[illegible]	p02911	Translationally-controlled tumor protein
CRIP2 seq: 113 - 128 + Phospho (ST)	[illegible]	p19461	Cysteine-rich protein 2
SRRM2 seq: 2098 - 2113 + Phospho (ST)	[illegible]	p09579	Serine/arginine repetitive matrix protein 2
FAM122B pS50 pM54	[illegible]	p07003	Protein FAM122B
TNS3 seq: 774 - 792 + Phospho (ST)	[illegible]	p12921	Tensin-3
HSPA5 seq: 541 - 554 no mod	[illegible]	p09821	78 kDa glucose-regulated protein
MTFR1 pS119	[illegible]	p08500	Mitochondrial fission regulator 1
NFIA pS287	[illegible]	p11480	Nuclear factor 1 A-type
MCM4 pS120	[illegible]	p07974	DNA replication licensing factor MCM4
DOCK7 pS929	[illegible]	p04751	Dedicator of cytokinesis protein 7
EIF5B pS107 pS113	[illegible]	p12650	Eukaryotic translation initiation factor 5B
MAP7 pS335	[illegible]	p05117	Ensconsin
KRT8 pS253	[illegible]	p05289	Keratin, type II cytoskeletal 8
FIP1L1 pS492	[illegible]	p17247	Pre-mRNA 3'-end-processing factor FIP1
MCMBP pS154	[illegible]	p00791	Mini-chromosome maintenance complex-binding protein
FAM83H seq: 935 - 947 + Phospho (ST)	[illegible]	p15242	Protein FAM83H
PGK1 seq: 172 - 184 no mod	[illegible]	p20510	Phosphoglycerate kinase 1
SETDB1 pS1066	[illegible]	p08922	Histone-lysine N-methyltransferase SETDB1
PRRC2A pS456	[illegible]	p12640	Protein PRRC2A
TJP2 seq: 975 - 997 + 2 Phospho (ST)	[illegible]	p07654	Tight junction protein ZO-2
CAMSAP3 seq: 429 - 444 + Phospho (ST)	[illegible]	p03254	Calmodulin-regulated spectrin-associated protein 3
RBMXL1 seq: 245 - 258 + Phospho (ST)	[illegible]	p18046	RNA binding motif protein, X-linked-like-1
AAK1 pT389	[illegible]	p20589	AP2-associated protein kinase 1
BAIAP2L1 pT257	[illegible]	p02414	Brain-specific angiogenesis inhibitor 1-associated protein 2-like protein 1
RAB11FIP1 pS234	[illegible]	p02372	Rab11 family-interacting protein 1
CORO7 seq: 904 - 925 + Phospho (ST)	[illegible]	p11725	Coronin-7
ATRX seq: 590 - 616 + Phospho (ST)	[illegible]	p10511	Transcriptional regulator ATRX
BAG3 seq: 194 - 203 + Phospho (ST)	[illegible]	p03853	BAG family molecular chaperone regulator 3
SPAG1 pS423	[illegible]	p07599	Sperm-associated antigen 1
MKI67 pS2223 pT2231	[illegible]	p14530	Antigen KI-67
JUN pS243	[illegible]	p11038	Transcription factor AP-1
BCKDHA seq: 333 - 346 + Phospho (ST)	[illegible]	p14262	2-oxoisovalerate dehydrogenase subunit alpha, mitochondrial
PGM2L1 pM171 pS175	[illegible]	p19277	Glucose 1,6-bisphosphate synthase
CASKIN1 pS987	[illegible]	p07590	Caskin-1
DHX29 pS200	[illegible]	p16510	ATP-dependent RNA helicase DHX29
HIST1H4A seq: 47 - 56 no mod	[illegible]	p13734	Histone H4
ATF7IP seq: 664 - 690 + Phospho (ST)	[illegible]	p14846	Activating transcription factor 7-interacting protein 1
WDR44 pS96	[illegible]	p15827	WD repeat-containing protein 44
SLTM pS1014	[illegible]	p13540	SAFB-like transcription modulator
EEF1G seq: 46 - 51 no mod	[illegible]	p02406	Elongation factor 1-gamma
HNRNPH3 pM290	[illegible]	p18808	Heterogeneous nuclear ribonucleoprotein H3
C10orf12 seq: 288 - 297 + Phospho (ST)	[illegible]	p14107	Uncharacterized protein C10orf12
PKP4 pS221	[illegible]	p19758	Plakophilin-4
DAP seq: 38 - 65 + Phospho (ST)	[illegible]	p17837	Death-associated protein 1
CEP170 pS1205	[illegible]	p13776	Centrosomal protein of 170 kDa
RRBP1 pS615	[illegible]	p09033	Ribosome-binding protein 1
DLG1 pM564 pS573	[illegible]	p17274	Disks large homolog 1
CD2BP2 seq: 188 - 201 + Phospho (ST)	[illegible]	p13176	CD2 antigen cytoplasmic tail-binding protein 2
VCL pS290	[illegible]	p14953	Vinculin
TJP2 seq: 975 - 997 + 2 Phospho (ST)	[illegible]	p07655	Tight junction protein ZO-2
RB1 pS855	[illegible]	p13898	Retinoblastoma-associated protein
ATXN2 seq: 695 - 738 + Phospho (ST)	[illegible]	p08183	Ataxin-2
FOXK2 pS398	[illegible]	p17681	Forkhead box protein K2
EFS pS331	[illegible]	p13161	Embryonal Fyn-associated substrate
TLN1 seq: 405 - 427 + Phospho (ST)	[illegible]	p05435	Talin-1
CTNND1 pS171	[illegible]	p01927	Catenin delta-1
NCOR2 pS1487	[illegible]	p05184	Nuclear receptor corepressor 2
CD2BP2 seq: 189 - 201 + Phospho (ST)	[illegible]	p15510	CD2 antigen cytoplasmic tail-binding protein 2
CDR2 seq: 309 - 325 + Phospho (ST)	[illegible]	p03717	Cerebellar degeneration-related protein 2
BMP2K seq: 10 - 42 + Phospho (ST)	[illegible]	p06027	BMP-2-inducible protein kinase
TMPO pS424	[illegible]	p16517	Lamina-associated polypeptide 2, isoform alpha
HIST1H1B pT138	[illegible]	p20398	Histone H1.5
MAP2K6 seq: 18 - 36 + Phospho (ST)	[illegible]	p17978	Dual specificity mitogen-activated protein kinase kinase 6
RRM2 pT33	[illegible]	p15642	Ribonucleoside-diphosphate reductase subunit M2
PHB seq: 178 - 186 no mod	[illegible]	p17782	Prohibitin
STMN1 pS38	[illegible]	p05422	Stathmin
KMT2B pS351	[illegible]	p01450	Histone-lysine N-methyltransferase 2B
ZBTB21 seq: 429 - 445 + Phospho (ST)	[illegible]	p14104	Zinc finger and BTB domain-containing protein 21
NDRG3 seq: 329 - 345 + Phospho (ST)	[illegible]	p02745	Protein NDRG3
SQSTM1 pS366	[illegible]	p17006	Sequestosome-1
RPS17 seq: 108 - 129 + Oxidation (M); Phospho (ST)	[illegible]	p11454	40S ribosomal protein S17
ZFHX3 seq: 1576 - 1597 + Phospho (ST)	[illegible]	p20195	Zinc finger homeobox protein 3
ING3 seq: 250 - 256 no mod	[illegible]	p19432	Inhibitor of growth protein 3
LARP7 seq: 296 - 309 + Phospho (ST)	[illegible]	p12613	La-related protein 7
ANKLE2 pS914	[illegible]	p09049	Ankyrin repeat and LEM domain-containing protein 2
WIZ pS1134	[illegible]	p04435	Protein Wiz
MINK1 seq: 714 - 738 + Phospho (ST)	[illegible]	p15082	Misshapen-like kinase 1
FNBP1L pS295	[illegible]	p02698	Formin-binding protein 1-like
RAI14 pS358	[illegible]	p01759	Ankycorbin
MICALL1 pS295	[illegible]	p11010	MICAL-like protein 1
KRT7 seq: 318 - 329 no mod	[illegible]	p11051	Keratin, type II cytoskeletal 7
DENND1A seq: 518 - 531 + Phospho (ST)	[illegible]	p06478	DENN domain-containing protein 1A
ZBTB21 seq: 429 - 445 + Phospho (ST)	[illegible]	p14103	Zinc finger and BTB domain-containing protein 21
UBR5 pS1549	[illegible]	p07246	E3 ubiquitin-protein ligase UBR5
TFPT pS180	[illegible]	p06513	TCF3 fusion partner
PDLIM2 seq: 132 - 153 + Phospho (ST)	[illegible]	p20569	PDZ and LIM domain protein 2
TBC1D15 seq: 68 - 81 + Phospho (ST)	[illegible]	p13317	TBC1 domain family member 15
TFPT pS252	[illegible]	p11322	TCF3 fusion partner
PLEKHA5 pS933 pS937	[illegible]	p15683	Pleckstrin homology domain-containing family A member 5

GAB2 seq: 403 - 414 + Phospho (ST)	3E+08	2E+08	5E+08 3E+08 3E+08 9E+08 3E+08 4E+08 3E+08 2E+08 7E+08 5E+08 3E+08 3E+08 2E+08 4E+08 3E+08 4E+08 4E+08 3E+08 4E+08 3E+08 3E+08 2E+08 3E+08 2E+08 1E+08 [illegible]	2E+08	p19509	GRB2-associated-binding protein 2
HSD17B10 seq: 148 - 172 no mod	1E+07	2E+08	2E+08 6E+07 9E+07 2E+08 2E+08 1E+08 8E+07 9E+07 6E+08 3E+08 1E+08 1E+08 2E+07 2E+08 1E+07 1E+08 1E+08 2E+07 1E+08 5E+07 2E+07 7E+07 5E+07 68554 44396 [illegible]	25732	p15031	3-hydroxyacyl-CoA dehydrogenase type-2
STARD10 seq: 20 - 31 no mod	2E+08	1E+08	2E+08 1E+08 1E+08 4E+08 1E+08 1E+08 1E+08 8E+07 9E+08 1E+08 2E+08 1E+08 9E+07 2E+08 1E+08 1E+08 2E+08 5E+07 1E+08 6E+07 1E+08 1E+08 8E+07 68554 44396 [illegible]	25732	p17089	PCTP-like protein
TANC2 pS1722	2E+08	2E+08	3E+08 3E+08 3E+08 3E+08 3E+08 3E+08 2E+08 8E+07 3E+08 4E+08 2E+08 2E+08 1E+08 3E+08 2E+08 3E+08 2E+08 2E+08 3E+08 2E+08 1E+08 1E+08 9E+07 68554 44396 [illegible]	25732	p04217	Protein TANC2
FGD1 pT711 pS715	2E+08	1E+08	3E+08 2E+08 2E+08 3E+08 3E+08 2E+08 2E+08 2E+08 4E+08 3E+08 2E+08 2E+08 2E+08 2E+08 2E+08 2E+08 2E+08 1E+08 2E+08 2E+08 2E+08 2E+08 2E+08 2E+08 1E+08 [illegible]	7E+07	p11342	FYVE, RhoGEF and PH domain-containing protein 1
DSP seq: 46 - 64 + Phospho (ST)	38324	37149	39656 13654 33854 45779 25552 34892 34104 26736 56234 53206 39754 40879 18798 40867 33072 34984 19162 28832 39759 50401 21442 19024 42741 2E+07 44396 [illegible]	25732	p15028	Desmoplakin
CASKIN2 seq: 875 - 896 + Phospho (ST)	3E+07	6E+07	6E+07 5E+07 1E+08 3E+08 6E+07 6E+07 5E+07 4E+07 5E+08 6E+07 7E+07 3E+07 3E+07 9E+07 2E+07 5E+07 6E+07 3E+07 8E+07 4E+07 3E+07 4E+07 4E+07 2E+09 5E+08 [illegible]	5E+08	p07076	Caskin-2
SEC16A seq: 769 - 802 + Phospho (ST)	4E+08	7E+08	8E+08 6E+08 8E+08 5E+08 8E+08 6E+08 7E+08 5E+08 3E+09 2E+09 1E+09 1E+09 9E+08 8E+08 7E+08 8E+08 8E+08 4E+08 7E+08 5E+08 7E+08 7E+08 7E+08 6E+08 2E+08 [illegible]	2E+08	p20586	Protein transport protein Sec16A
MAP1B seq: 1496 - 1514 + Phospho (ST)	38324	3E+07	5E+07 2E+07 3E+07 2E+08 5E+07 34892 3E+07 26736 56234 53206 1E+07 40879 18798 8E+07 33072 4E+07 19162 28832 8E+07 2E+07 4E+07 19024 5E+07 2E+09 44396 [illegible]	25732	p12979	Microtubule-associated protein 1B
FASN seq: 1105 - 1116 no mod	3E+08	3E+08	3E+08 1E+08 2E+08 45779 2E+08 3E+08 2E+08 1E+08 3E+07 2E+08 3E+08 1E+08 7E+07 6E+06 8E+07 2E+08 2E+08 6E+07 39759 9E+07 2E+08 1E+08 1E+08 3E+07 44396 [illegible]	7E+06	p06807	Fatty acid synthase
GPATCH8 pS1107	4E+08	3E+08	4E+08 4E+08 4E+08 7E+08 3E+08 3E+08 3E+08 3E+08 7E+08 4E+08 4E+08 4E+08 4E+08 1E+08 1E+08 2E+08 2E+08 1E+08 3E+08 2E+08 3E+08 3E+08 3E+08 1E+09 4E+08 [illegible]	9E+08	p12712	G patch domain-containing protein 8
KMT2D pS3130	3E+08	6E+08	6E+08 5E+08 5E+08 1E+09 1E+09 6E+08 5E+08 4E+08 1E+09 9E+08 4E+08 5E+08 5E+08 7E+08 6E+08 6E+08 5E+08 4E+08 1E+09 5E+08 4E+08 5E+08 5E+08 8E+08 3E+08 [illegible]	2E+08	p01638	Histone-lysine N-methyltransferase 2D
FAM122A seq: 261 - 287 + Phospho (ST)	2E+09	2E+09	1E+09 1E+09 1E+09 1E+08 1E+09 2E+09 1E+09 1E+09 1E+08 9E+08 1E+09 1E+09 1E+09 1E+09 2E+09 1E+09 1E+09 1E+09 2E+09 2E+09 2E+09 1E+09 1E+09 2E+09 2E+09 [illegible]	1E+09	p00761	Protein FAM122A
GOLGA4 pS71	4E+09	4E+09	4E+09 3E+09 3E+09 4E+09 5E+09 3E+09 3E+09 3E+09 4E+09 4E+09 3E+09 2E+09 2E+09 3E+09 4E+09 4E+09 3E+09 3E+09 3E+09 3E+09 3E+09 2E+09 3E+09 6E+09 4E+09 [illegible]	3E+09	p00982	Golgin subfamily A member 4
BAG3 seq: 194 - 203 + Phospho (ST)	1E+09	1E+09	1E+09 6E+08 7E+08 2E+09 2E+09 1E+09 8E+08 8E+08 2E+09 8E+08 6E+08 5E+08 5E+08 1E+09 6E+08 1E+09 1E+09 6E+08 1E+09 8E+08 1E+09 7E+08 7E+08 3E+08 2E+08 [illegible]	2E+08	p03854	BAG family molecular chaperone regulator 3
RAB11FIP5 seq: 202 - 227 + Phospho (ST)	2E+07	9E+07	7E+07 2E+08 1E+08 3E+08 1E+08 1E+08 2E+08 1E+08 2E+08 1E+08 9E+07 2E+08 9E+07 1E+08 6E+07 1E+08 2E+08 2E+08 8E+07 1E+08 1E+08 2E+08 1E+08 2E+08 2E+07 [illegible]	5E+07	p12355	Rab11 family-interacting protein 5
ZYX pS267	4E+08	7E+08	6E+08 6E+08 6E+08 1E+09 7E+08 5E+08 5E+08 4E+08 2E+09 1E+09 7E+08 7E+08 7E+08 7E+08 4E+08 5E+08 4E+08 5E+08 7E+08 4E+08 4E+08 5E+08 6E+08 4E+09 2E+09 [illegible]	1E+09	p15481	Zyxin
TNKS1BP1 pS983	38324	37149	39656 4E+07 33854 2E+08 25552 34892 5E+07 26736 3E+08 8E+07 6E+07 1E+08 18798 40867 33072 34984 1E+08 28832 39759 50401 21442 19024 42741 2E+08 44396 [illegible]	5E+07	p05907	182 kDa tankyrase-1-binding protein
TUBB pM330	2E+09	2E+09	2E+09 2E+09 2E+09 4E+09 2E+09 2E+09 2E+09 1E+09 4E+09 2E+09 1E+09 2E+09 1E+09 2E+09 1E+09 2E+09 1E+09 2E+09 3E+09 1E+09 1E+09 1E+09 1E+09 3E+09 1E+09 [illegible]	1E+09	p17013	Tubulin beta chain
KRT8 seq: 303 - 312 no mod	2E+09	2E+09	3E+09 1E+09 2E+09 45779 2E+09 1E+09 1E+09 9E+08 3E+09 3E+09 3E+09 1E+09 1E+09 1E+09 2E+09 2E+09 2E+09 9E+08 1E+09 2E+09 2E+09 1E+09 1E+09 2E+09 2E+09 [illegible]	1E+09	p02642	Keratin, type II cytoskeletal 8
BAIAP2L1 pT257 pS261	3E+08	1E+08	4E+08 1E+08 2E+08 45779 2E+08 2E+08 1E+08 1E+08 56234 3E+08 3E+08 2E+08 2E+08 40867 2E+08 3E+08 2E+08 1E+08 39759 1E+08 3E+08 1E+08 2E+08 2E+08 2E+09 [illegible]	1E+09	p02420	Brain-specific angiogenesis inhibitor 1-associated protein 2-like protein 1
RASAL2 seq: 733 - 747 + Phospho (ST)	4E+06	37149	2E+08 7E+07 1E+08 5E+08 25552 2E+08 1E+08 7E+07 56234 1E+08 39754 40879 5E+07 40867 33072 2E+08 1E+08 4E+07 39759 50401 2E+08 19024 8E+07 7E+07 3E+07 [illegible]	4E+07	p10549	Ras GTPase-activating protein nGAP
NEDD4L seq: 469 - 493 + 2 Phospho (ST)	3E+07	3E+07	39656 7E+07 4E+07 45779 5E+07 2E+07 5E+07 3E+07 56234 2E+07 39754 3E+07 2E+07 40867 2E+07 4E+07 6E+07 3E+07 5E+07 3E+07 21442 4E+07 2E+07 2E+08 8E+07 [illegible]	1E+08	p19235	E3 ubiquitin-protein ligase NEDD4-like
PLCG1 seq: 1262 - 1274 + Phospho (ST)	9E+07	3E+07	[illegible]	2E+07	p13676	1-phosphatidylinositol 4,5-bisphosphate phosphodiesterase gamma-1
DUT seq: 85 - 103 + Phospho (ST)	5E+07	1E+08	[illegible]	6E+07	p20650	Deoxyuridine 5'-triphosphate nucleotidohydrolase, mitochondrial
FASN seq: 60 - 70 no mod	1E+08	2E+08	[illegible]	9E+06	p16819	Fatty acid synthase
STMN1 pS38	3E+09	3E+09	[illegible]	5E+09	p17091	Stathmin
RFC1 seq: 503 - 514 + Phospho (ST)	5E+06	1E+08	[illegible]	1E+08	p11934	Replication factor C subunit 1
LAD1 seq: 266 - 282 + Phospho (ST)	1E+09	9E+08	[illegible]	2E+09	p20317	Ladinin-1
LMNB1 pT20	2E+09	2E+09	[illegible]	2E+09	p20656	Lamin-B1
q: 736 - 770 + Gln->pyro-Glu (N-term Q); 2 Phospho (ST)	1E+08	6E+07	[illegible]	7E+07	p08693	Palladin
ITGB3BP seq: 28 - 54 + Phospho (ST)	1E+08	1E+08	[illegible]	6E+07	p03327	Centromere protein R
MED13 seq: 393 - 409 + Phospho (ST)	8E+07	5E+07	[illegible]	5E+07	p12337	Mediator of RNA polymerase II transcription subunit 13
CCDC88C seq: 1578 - 1598 + Phospho (ST)	4E+08	4E+08	[illegible]	4E+08	p08999	Protein Daple
CALR seq: 99 - 111 no mod	3E+08	3E+08	[illegible]	6E+07	p14841	Calreticulin
RSL1D1 pS392	3E+08	6E+08	[illegible]	8E+08	p12567	Ribosomal L1 domain-containing protein 1
DLGAP4 pS763	2E+09	2E+09	[illegible]	1E+08	p09397	Disks large-associated protein 4
ARHGAP29 pS1029	2E+08	2E+08	[illegible]	3E+09	p11365	Rho GTPase-activating protein 29
DIS3 seq: 586 - 596 no mod	3E+08	4E+08	[illegible]	2E+08	p19572	Exosome complex exonuclease RRP44
RANGAP1 seq: 413 - 445 + Phospho (ST)	3E+07	1E+08	[illegible]	7E+08	p13092	Ran GTPase-activating protein 1
ERCC6 pS158	7E+07	2E+08	[illegible]	7E+07	p14180	DNA excision repair protein ERCC-6
LASP1 pM131 pS146	9E+09	4E+09	[illegible]	2E+09	p10096	LIM and SH3 domain protein 1
CSDE1 seq: 278 - 286 no mod	9E+07	1E+08	[illegible]	2E+07	p09220	Cold shock domain-containing protein E1
IRF2BP2 pS71	38324	37149	[illegible]	2E+09	p20534	Interferon regulatory factor 2-binding protein 2
NDRG3 seq: 329 - 345 + Phospho (ST)	2E+09	2E+09	[illegible]	1E+09	p02744	Protein NDRG3
ATRIP pS224	4E+08	5E+08	[illegible]	5E+08	p12302	ATR-interacting protein
TOP2A pM1207 pS1213	1E+08	3E+08	[illegible]	1E+08	p02274	DNA topoisomerase 2-alpha
FAM195A seq: 75 - 99 + Phospho (ST)	6E+08	7E+08	[illegible]	1E+08	p07609	Protein FAM195A
TNKS1BP1 pS429	3E+09	3E+09	[illegible]	4E+08	p07436	182 kDa tankyrase-1-binding protein
LMNA seq: 387 - 397 + 2 Phospho (ST)	1E+10	8E+09	[illegible]	3E+10	p10884	Prelamin-A/C
SRRM2 seq: 1690 - 1699 + Phospho (ST)	1E+08	1E+08	[illegible]	9E+07	p03734	Serine/arginine repetitive matrix protein 2
EGLN1 pS125	8E+08	1E+09	[illegible]	2E+08	p20385	Egl nine homolog 1
TJP2 pS325	2E+08	2E+08	[illegible]	2E+08	p11686	Tight junction protein ZO-2
SEPT7 seq: 314 - 326 no mod	2E+09	3E+09	[illegible]	3E+09	p12296	Septin-7
BIN1 pS298	38324	3E+07	[illegible]	2E+09	p15581	Myc box-dependent-interacting protein 1
DIDO1 seq: 1011 - 1023 + Phospho (ST)	4E+08	7E+08	[illegible]	1E+08	p05521	Death-inducer obliterator 1
TCOF1 seq: 302 - 322 + Phospho (ST)	2E+08	3E+08	[illegible]	5E+08	p21052	Treacle protein
TUBB4B pM299	8E+08	1E+09	[illegible]	4E+08	p09368	Tubulin beta-4B chain
DOCK6 seq: 878 - 901 + 2 Phospho (ST)	8E+07	3E+07	[illegible]	25732	p05368	Dedicator of cytokinesis protein 6
KIAA0101 pS72	2E+07	1E+08	[illegible]	2E+08	p15899	PCNA-associated factor
KIF23 pM859 pS867	2E+08	2E+08	[illegible]	1E+08	p00219	Kinesin-like protein KIF23
RFFL seq: 238 - 256 + Phospho (ST)	6E+07	2E+08	[illegible]	4E+08	p07576	E3 ubiquitin-protein ligase rififylin
ARVCF seq: 913 - 932 + Phospho (ST)	3E+08	4E+08	[illegible]	25732	p16260	Armadillo repeat protein deleted in velo-cardio-facial syndrome
MAP7 seq: 198 - 212 + Phospho (ST)	2E+08	4E+08	[illegible]	3E+08	p07081	Ensconsin
ABI1 pS183 pM186	4E+09	3E+09	[illegible]	2E+09	p02477	Abl interactor 1
MBP seq: 38 - 69 + Phospho (ST)	4E+08	2E+08	[illegible]	1E+08	p02024	Myelin basic protein
NUMA1 pT2000	6E+08	1E+09	[illegible]	5E+08	p00829	Nuclear mitotic apparatus protein 1
RALGAPA1 pS721	1E+08	1E+08	[illegible]	25732	p14943	Ral GTPase-activating protein subunit alpha-1
LRRN3 pT317	5E+08	5E+08	[illegible]	5E+08	p13109	Leucine-rich repeat neuronal protein 3
ZAK pS633	2E+08	2E+08	[illegible]	2E+08	p00152	Mitogen-activated protein kinase kinase kinase MLT
SCYL3 pS707	1E+08	9E+07	[illegible]	1E+07	p13345	Protein-associating with the carboxyl-terminal domain of ezrin
CSNK1D pS382	9E+08	1E+09	[illegible]	4E+08	p16273	Casein kinase I isoform delta
ERBB2 seq: 1054 - 1072 + Phospho (ST)	2E+08	2E+08	[illegible]	25732	p05760	Receptor tyrosine-protein kinase erbB-2
PPL pS14	4E+08	1E+09	[illegible]	2E+09	p15790	Periplakin
CHD3 pS324	2E+08	37149	[illegible]	25732	p15951	Chromodomain-helicase-DNA-binding protein 3
ABI1 pS231	5E+08	5E+08	[illegible]	5E+08	p03029	Abl interactor 1
LMNB2 pS37	7E+08	7E+08	[illegible]	6E+08	p20659	Lamin-B2
LATS1 seq: 462 - 472 + Phospho (ST)	4E+08	4E+08	[illegible]	2E+08	p04809	Serine/threonine-protein kinase LATS1
OCIAD1 seq: 121 - 131 + Phospho (ST)	1E+08	2E+08	[illegible]	4E+07	p06593	OCIA domain-containing protein 1
STIP1 seq: 64 - 73 no mod	1E+08	2E+08	[illegible]	7E+07	p01975	Stress-induced-phosphoprotein 1
LIMD2 pS29	7E+08	7E+08	[illegible]	3E+08	p05369	LIM domain-containing protein 2
SNAPIN pS133	8E+08	1E+09	[illegible]	1E+09	p20084	SNARE-associated protein Snapin
PPL pS14	3E+08	8E+08	[illegible]	2E+09	p15791	Periplakin
TGS1 pS89	7E+07	5E+07	[illegible]	1E+08	p15896	Trimethylguanosine synthase
TCOF1 pS906	2E+08	2E+08	[illegible]	7E+08	p21108	Treacle protein
PALM pS116 pS124	3E+08	3E+08	[illegible]	25732	p17350	Paralemmin-1
CDK1 pT14	3E+08	2E+08	[illegible]	3E+08	p14279	Cyclin-dependent kinase 1
CDK18 pT152	3E+08	2E+08	[illegible]	3E+08	p11802	Cyclin-dependent kinase 18
MTMR4 seq: 627 - 641 + Phospho (ST)	6E+07	4E+07	[illegible]	25732	p02121	Myotubularin-related protein 4
KHDRBS1 seq: 57 - 96 + Phospho (ST)	1E+10	9E+09	[illegible]	5E+09	p19557	KH domain-containing, RNA-binding, signal transduction-associated protein 1
TSC2 seq: 1130 - 1138 + Oxidation (M); Phospho (ST)	6E+08	4E+08	[illegible]	25732	p04873	Tuberin
eq: 107 - 124 + Gln->pyro-Glu (N-term Q); Phospho (ST)	4E+09	3E+09	[illegible]	6E+09	p08810	Transcriptional coactivator YAP1
PCM1 seq: 577 - 587 no mod	4E+07	6E+07	[illegible]	1E+08	p01936	Pericentriolar material 1 protein
PKM seq: 207 - 230 no mod	38324	1E+08	[illegible]	9E+07	p13134	Pyruvate kinase PKM
PHLDB2 seq: 925 - 948 + Oxidation (M); Phospho (ST)	38324	37149	[illegible]	2E+08	p20530	Pleckstrin homology-like domain family B member 2
BMP2K pS1107	6E+08	7E+08	[illegible]	6E+08	p08180	BMP-2-inducible protein kinase
+ Gln->pyro-Glu (N-term Q); Oxidation (M); Phospho (ST)	1E+09	1E+09	[illegible]	2E+08	p08243	Polyadenylate-binding protein 2
TANC2 pS1744	6E+08	9E+08	[illegible]	3E+07	p03641	Protein TANC2
XRN1 pS1645	2E+08	2E+08	[illegible]	1E+08	p17923	5'-3' exoribonuclease 1
SPAG1 pS423	2E+08	7E+08	[illegible]	3E+08	p07600	Sperm-associated antigen 1
EPPK1 pM26 pT34	3E+08	3E+08	[illegible]	1E+08	p20105	Epiplakin
EIF4G1 seq: 4 - 48 + Phospho (ST)	2E+08	3E+08	[illegible]	3E+08	p19945	Eukaryotic translation initiation factor 4 gamma 1
TNK1 seq: 502 - 524 + 2 Phospho (ST)	1E+09	1E+09	[illegible]	1E+09	p05243	Non-receptor tyrosine-protein kinase TNK1
SUGP1 seq: 475 - 501 + Phospho (ST)	38324	5E+07	[illegible]	4E+08	p19198	SURP and G-patch domain-containing protein 1
MDC1 pS168	3E+07	5E+08	[illegible]	4E+09	p11366	Mediator of DNA damage checkpoint protein 1
MARCKS pS691	2E+09	4E+09	[illegible]	2E+09	p07433	Myristoylated alanine-rich C-kinase substrate
OSBPL11 pS181	6E+08	5E+08	[illegible]	1E+09	p05840	Oxysterol-binding protein-related protein 11
PPP1R13B seq: 708 - 724 + Phospho (ST)	8E+07	1E+08	[illegible]	25732	p06612	Apoptosis-stimulating of p53 protein 1
TPI1 seq: 56 - 70 no mod	38324	2E+08	[illegible]	2E+08	p12648	Triosephosphate isomerase
SYAP1 seq: 259 - 291 + Phospho (ST)	4E+08	3E+08	[illegible]	1E+09	p02288	Synapse-associated protein 1
TJAP1 seq: 543 - 557 + Phospho (ST)	1E+09	8E+08	[illegible]	2E+09	p13321	Tight junction-associated protein 1
OCIAD1 seq: 121 - 131 + Phospho (ST)	6E+09	1E+10	[illegible]	9E+08	p06592	OCIA domain-containing protein 1
RAB11FIP1 pS156	3E+08	8E+08	[illegible]	5E+08	p09326	Rab11 family-interacting protein 1
TTC28 pS2224	4E+08	7E+08	[illegible]	25732	p04234	Tetratricopeptide repeat protein 28
AMPD2 pS168	2E+09	2E+09	[illegible]	6E+08	p08498	AMP deaminase 2
CCDC6 seq: 349 - 387 + 3 Phospho (ST)	5E+08	5E+08	[illegible]	5E+08	p01892	Coiled-coil domain-containing protein 6
ABCF1 pT108 pS109	1E+09	2E+09	[illegible]	2E+09	p12895	ATP-binding cassette sub-family F member 1
FAM117B pS136 pT149 pS151	8E+08	5E+08	[illegible]	5E+08	p15214	Protein FAM117B
PLEKHA5 pT438	1E+08	2E+08	[illegible]	4E+08	p15029	Pleckstrin homology domain-containing family A member 5
EIF4B pS207	1E+09	1E+09	[illegible]	2E+07	p19695	Eukaryotic translation initiation factor 4B
CCNL1 pS352	2E+07	9E+07	[illegible]	4E+07	p16978	Cyclin-L1
CSPP1 pS901	3E+07	5E+07	[illegible]	1E+08	p10938	Centrosome and spindle pole-associated protein 1
SLAIN2 pS147	2E+08	3E+08	[illegible]	6E+07	p03266	SLAIN motif-containing protein 2
UBR5 pS1549	9E+08	7E+08	[illegible]	5E+08	p07245	E3 ubiquitin-protein ligase UBR5
G6PD seq: 371 - 386 no mod	1E+08	3E+08	[illegible]	25732	p11003	Glucose-6-phosphate 1-dehydrogenase
UBL7 pM220 pS230	1E+09	2E+09	[illegible]	7E+08	p18483	Ubiquitin-like protein 7
FNIP1 seq: 230 - 246 + Phospho (ST)	1E+08	1E+08	[illegible]	2E+07	p06266	Folliculin-interacting protein 1
ETFB pT194	5E+06	37149	[illegible]	2E+08	p00439	Electron transfer flavoprotein subunit beta
DGCR8 pS95	1E+08	2E+08	[illegible]	2E+08	p11384	Microprocessor complex subunit DGCR8
ATRIP pS224	3E+08	4E+08	[illegible]	3E+08	p12303	ATR-interacting protein
TUBA1B pS340	8E+08	1E+09	[illegible]	4E+08	p05487	Tubulin alpha-1B chain
MPLKIP seq: 93 - 117 + Phospho (ST)	4E+08	4E+08	[illegible]	1E+09	p04727	M-phase-specific PLK1-interacting protein
CTNND1 seq: 265 - 274 + Phospho (ST)	9E+09	8E+09	[illegible]	2E+10	p01484	Catenin delta-1
53BP1 seq: 1085 - 1106 + Oxidation (M); 2 Phospho (ST)	8E+08	5E+08	[illegible]	3E+08	p07854	Tumor suppressor p53-binding protein 1
CCT2 seq: 237 - 250 no mod	2E+07	5E+07	[illegible]	3E+07	p14037	T-complex protein 1 subunit beta
PHLDB2 pS73	2E+08	3E+08	[illegible]	4E+08	p06642	Pleckstrin homology-like domain family B member 2
LMNA seq: 145 - 156 no mod	2E+07	2E+07	[illegible]	7E+07	p18014	Prelamin-A/C
PRKCB seq: 497 - 517 + Phospho (ST)	5E+07	6E+07	[illegible]	2E+08	p02873	Protein kinase C beta type
SEC16A seq: 1178 - 1188 + Phospho (ST)	4E+08	5E+08	[illegible]	2E+08	p05188	Protein transport protein Sec16A
CIC pS496	4E+08	8E+08	[illegible]	2E+08	p12431	Protein capicua homolog
PGK1 seq: 323 - 330 no mod	6E+09	7E+09	[illegible]	2E+10	p12357	Phosphoglycerate kinase 1
VDAC1 seq: 237 - 256 no mod	38324	2E+08	[illegible]	2E+07	p01059	Voltage-dependent anion-selective channel protein 1
MAP7 seq: 198 - 212 + Phospho (ST)	7E+08	1E+09	[illegible]	8E+08	p07080	Ensconsin
GBF1 seq: 345 - 381 + 2 Phospho (ST)	1E+08	7E+07	[illegible]	25732	p04218	Golgi-specific brefeldin A-resistance guanine nucleotide exchange factor 1
LARP1B pS363	6E+08	7E+08	[illegible]	5E+07	p15635	La-related protein 1B
RPS26 seq: 43 - 51 no mod	1E+08	2E+08	[illegible]	1E+08	p09528	40S ribosomal protein S26
MAP4K5 seq: 431 - 454 + Phospho (ST)	7E+08	4E+08	[illegible]	5E+07	p06780	Mitogen-activated protein kinase kinase kinase kinase 5
DDA1 pS33	2E+08	3E+08	[illegible]	2E+08	p06832	DET1- and DDB1-associated protein 1
BICD2 seq: 598 - 621 + Phospho (ST)	38324	37149	[illegible]	4E+07	p20918	Protein bicaudal D homolog 2
MAPT seq: 543 - 557 + 2 Phospho (ST)	5E+08	4E+08	[illegible]	25732	p01727	Microtubule-associated protein tau
FAM83E pS351	2E+09	2E+09	[illegible]	25732	p00807	Protein FAM83E
KMT2B pS821	7E+07	2E+08	[illegible]	6E+07	p01836	Histone-lysine N-methyltransferase 2B
RPAP1 seq: 55 - 76 + Phospho (ST)	5E+07	2E+08	[illegible]	3E+08	p18078	RNA polymerase II-associated protein 1
ZC3H13 seq: 236 - 249 + Phospho (ST)	3E+08	4E+08	[illegible]	1E+08	p12473	Zinc finger CCCH domain-containing protein 13
PKN2 seq: 814 - 836 + Phospho (ST)	3E+08	3E+08	[illegible]	1E+08	p02074	Serine/threonine-protein kinase N2
ALDOA seq: 44 - 57 no mod	2E+09	1E+09	[illegible]	7E+08	p10932	Fructose-bisphosphate aldolase A
CHTOP pT33	1E+08	1E+08	[illegible]	3E+08	p09496	Chromatin target of PRMT1 protein
KIAA1522 pS327	6E+07	2E+08	[illegible]	7E+07	p19016	Uncharacterized protein KIAA1522
RGL2 pS13	3E+08	5E+08	[illegible]	4E+08	p11362	Ral guanine nucleotide dissociation stimulator-like 2
CENPC pT130	8E+07	2E+08	[illegible]	6E+07	p14089	Centromere protein C
BABAM1 seq: 38 - 71 + Phospho (ST)	9E+07	9E+07	[illegible]	7E+07	p19244	BRISC and BRCA1-A complex member 1
C2CD2L pS411	6E+07	7E+08	[illegible]	8E+07	p20257	C2 domain-containing protein 2-like
RBM14 pT206	6E+10	5E+10	[illegible]	3E+10	p08093	RNA-binding protein 14
SHANK2 seq: 668 - 708 + Phospho (ST)	38324	2E+08	[illegible]	25732	p03751	SH3 and multiple ankyrin repeat domains protein 2
SAC3D1 pS402	3E+07	3E+07	[illegible]	1E+08	p02617	SAC3 domain-containing protein 1
LAMTOR1 seq: 21 - 31 + Phospho (ST)	1E+08	1E+08	[illegible]	1E+08	p11364	Ragulator complex protein LAMTOR1
GOLGA4 seq: 9 - 34 + Phospho (ST)	5E+08	5E+08	[illegible]	5E+08	p13746	Golgin subfamily A member 4
DNTTIP2 pS381	2E+07	4E+07	[illegible]	2E+08	p04582	Deoxynucleotidyltransferase terminal-interacting protein 2
SPEN pS1278	3E+08	2E+08	[illegible]	2E+07	p14798	Msx2-interacting protein
ZNF787 pT145	8E+07	3E+07	[illegible]	9E+07	p14202	Zinc finger protein 787
SUCO seq: 1224 - 1235 + Phospho (ST)	2E+07	2E+08	[illegible]	1E+08	p05682	SUN domain-containing ossification factor
BAG3 pT285	1E+09	2E+09	[illegible]	2E+08	p03588	BAG family molecular chaperone regulator 3
SLTM seq: 549 - 564 + 2 Phospho (ST)	9E+08	1E+09	[illegible]	7E+08	p13664	SAFB-like transcription modulator
RPS3A pM229	4E+07	5E+07	[illegible]	6E+07	p11237	40S ribosomal protein S3a
KLRG2 seq: 193 - 220 + Phospho (ST)	4E+07	9E+07	[illegible]	25732	p02888	Killer cell lectin-like receptor subfamily G member 2
KRT7 seq: 2 - 13 + 2 Phospho (ST)	4E+08	4E+08	[illegible]	1E+09	p05522	Keratin, type II cytoskeletal 7
TCOF1 seq: 302 - 322 + 2 Phospho (ST)	1E+08	1E+08	[illegible]	6E+08	p21053	Treacle protein
TNS2 seq: 828 - 842 + Phospho (ST)	7E+07	8E+07	[illegible]	7E+07	p20577	Tensin-2
WDR44 seq: 86 - 101 + Phospho (ST)	3E+08	2E+08	[illegible]	1E+08	p15826	WD repeat-containing protein 44
CLASP2 pS596	3E+07	4E+07	[illegible]	4E+08	p01200	CLIP-associating protein 2
CTDP1 seq: 952 - 961 no mod	2E+08	1E+08	[illegible]	3E+07	p20292	RNA polymerase II subunit A C-terminal domain phosphatase
EMSY pS1136	7E+07	6E+07	[illegible]	8E+07	p10491	Protein EMSY
HNRNPU seq: 576 - 590 no mod	2E+08	3E+08	[illegible]	7E+07	p09643	Heterogeneous nuclear ribonucleoprotein U
CHM seq: 227 - 239 + Phospho (Y)	2E+08	3E+08	[illegible]	7E+07	p16559	Rab proteins geranylgeranyltransferase component A 1
HBS1L seq: 215 - 237 + Phospho (ST)	2E+08	1E+08	[illegible]	2E+08	p06308	HBS1-like protein
BCL11B seq: 752 - 767 + Phospho (ST)	6E+07	5E+08	[illegible]	25732	p16440	B-cell lymphoma/leukemia 11B
ATXN2L pS559	5E+09	5E+09	[illegible]	6E+09	p10958	Ataxin-2-like protein
ACTA2 seq: 53 - 64 no mod	1E+09	1E+09	[illegible]	9E+08	p18194	Actin, aortic smooth muscle
EIF4H pS21	3E+08	4E+08	[illegible]	5E+08	p19097	Eukaryotic translation initiation factor 4H
SART1 pS448	6E+08	5E+08	[illegible]	3E+08	p06445	U4/U6.U5 tri-snRNP-associated protein 1
RGL2 seq: 617 - 650 + Phospho (ST)	1E+08	1E+08	[illegible]	7E+07	p06276	Ral guanine nucleotide dissociation stimulator-like 2
CDC25B seq: 321 - 334 + Phospho (ST)	3E+07	8E+07	[illegible]	2E+08	p04410	M-phase inducer phosphatase 2
LASP1 seq: 76 - 85 no mod	6E+08	5E+08	[illegible]	1E+08	p08022	LIM and SH3 domain protein 1
C1orf106 seq: 386 - 397 + Phospho (ST)	2E+07	8E+06	[illegible]	1E+08	p02198	Uncharacterized protein C1orf106
TRIM36 pS279	1E+08	2E+08	[illegible]	25732	p04245	E3 ubiquitin-protein ligase TRIM36
MYRIP seq: 293 - 315 + 2 Phospho (ST)	2E+08	2E+08	[illegible]	2E+08	p04220	Rab effector MyRIP
MYH9 seq: 1846 - 1856 no mod	4E+07	5E+07	[illegible]	1E+07	p18108	Myosin-9
PYGO2 pT302	2E+09	1E+09	[illegible]	2E+09	p16045	Pygopus homolog 2
AMFR seq: 380 - 389 no mod	6E+06	6E+07	[illegible]	5E+07	p09896	E3 ubiquitin-protein ligase AMFR
NCOR2 seq: 981 - 1020 + Phospho (ST)	2E+09	1E+09	[illegible]	5E+08	p01423	Nuclear receptor corepressor 2
LPIN1 pS449	38324	2E+07	[illegible]	5E+07	p06306	Phosphatidate phosphatase LPIN1
C2CD3 seq: 2127 - 2135 + Phospho (ST)	2E+07	2E+07	[illegible]	5E+07	p03909	C2 domain-containing protein 3
COBLL1 seq: 851 - 879 + Phospho (ST)	4E+07	1E+08	[illegible]	25732	p17079	Cordon-bleu protein-like 1
RAD51AP1 pS120	3E+08	2E+08	[illegible]	2E+08	p17423	RAD51-associated protein 1
RPL23A pS43	38324	37149	[illegible]	6E+07	p02134	60S ribosomal protein L23a
LYSMD2 seq: 31 - 46 + Phospho (ST)	7E+07	1E+08	[illegible]	9E+08	p05695	LysM and putative peptidoglycan-binding domain-containing protein 2
AHNAK pS4386	38324	1E+07	[illegible]	7E+06	p13709	Neuroblast differentiation-associated protein AHNAK
PIKFYVE pS307	5E+08	3E+08	[illegible]	2E+08	p06263	1-phosphatidylinositol 3-phosphate 5-kinase
FIP1L1 pS259	1E+09	9E+08	[illegible]	8E+08	p20843	Pre-mRNA 3'-end-processing factor FIP1
SHROOM3 seq: 970 - 983 + Phospho (ST)	2E+08	2E+08	[illegible]	1E+08	p04693	Protein Shroom3

RAPGEF6 pS1094	[illegible]	p06609	Rap guanine nucleotide exchange factor 6
PSMB3 seq: 18 - 27 no mod	[illegible]	p09721	Proteasome subunit beta type-3
SIK3 seq: 336 - 344 + Oxidation (M)	[illegible]	p11830	Serine/threonine-protein kinase SIK3
CNTNAP3 seq: 146 - 153 no mod	[illegible]	p10962	Contactin-associated protein-like 3
ZC3H13 pT364	[illegible]	p02516	Zinc finger CCCH domain-containing protein 13
GAB1 seq: 273 - 301 + Phospho (ST)	[illegible]	p00792	GRB2-associated-binding protein 1
SEPT9 pS22	[illegible]	p07182	Septin-9
IRF2BP2 pS457 pS460	[illegible]	p09133	Interferon regulatory factor 2-binding protein 2
AMBP seq: 63 - 85 no mod	[illegible]	p09838	Protein AMBP
CAMSAP3 seq: 812 - 823 + Phospho (ST)	[illegible]	p13225	Calmodulin-regulated spectrin-associated protein 3
SMC4 pS28	[illegible]	p06761	Structural maintenance of chromosomes protein 4
ZC3HAV1 pS257	[illegible]	p18350	Zinc finger CCCH-type antiviral protein 1
MAP7 pS202	[illegible]	p10631	Ensconsin
TANK pS129	[illegible]	p05213	TRAF family member-associated NF-kappa-B activator
GPATCH2 seq: 127 - 137 + Phospho (ST)	[illegible]	p06843	G patch domain-containing protein 2
SRRM2 pS2407 pT2409	[illegible]	p19675	Serine/arginine repetitive matrix protein 2
KDM3B seq: 725 - 763 + Phospho (ST)	[illegible]	p03651	Lysine-specific demethylase 3B
TUBA4A seq: 265 - 280 no mod	[illegible]	p14215	Tubulin alpha-4A chain
ZNF638 seq: 1396 - 1403 + Phospho (ST)	[illegible]	p19236	Zinc finger protein 638
MLPH pS510	[illegible]	p07208	Melanophilin
HSPD1 seq: 134 - 141 no mod	[illegible]	p16285	60 kDa heat shock protein, mitochondrial
CSF3R seq: 227 - 233 no mod	[illegible]	p11947	Granulocyte colony-stimulating factor receptor
BRF1 pS553	[illegible]	p15638	Transcription factor IIIB 90 kDa subunit
INPP5F pS907	[illegible]	p04184	Phosphatidylinositide phosphatase SAC2
PSMB7 pM67	[illegible]	p19396	Proteasome subunit beta type-7
SGK223 seq: 790 - 818 + Phospho (ST)	[illegible]	p11913	Tyrosine-protein kinase SgK223
L1RE1 seq: 25 - 49 + Phospho (ST)	[illegible]	p03672	LINE-1 retrotransposable element ORF1 protein
'1 seq: 16 - 31 + Gln->pyro-Glu (N-term Q); Phospho (ST)	[illegible]	p07660	28 kDa heat- and acid-stable phosphoprotein
MAPRE1 seq: 223 - 241 no mod	[illegible]	p09567	Microtubule-associated protein RP/EB family member 1
NCOR2 seq: 2048 - 2076 + 2 Phospho (ST)	[illegible]	p05206	Nuclear receptor corepressor 2
SH3PXD2A seq: 817 - 836 + Phospho (ST)	[illegible]	p06575	SH3 and PX domain-containing protein 2A
PEBP1 seq: 162 - 179 no mod	[illegible]	p19864	Phosphatidylethanolamine-binding protein 1
PPFIA4 pS1139	[illegible]	p07606	Liprin-alpha-4
FRMD5 seq: 344 - 360 + Phospho (ST)	[illegible]	p20611	FERM domain-containing protein 5
KIAA1522 pS971 pS979	[illegible]	p12715	Uncharacterized protein KIAA1522
HMGA1 pT53	[illegible]	p17300	High mobility group protein HMG-I/HMG-Y
TBC1D9B pS435	[illegible]	p13374	TBC1 domain family member 9B
MDH1 seq: 171 - 179 no mod	[illegible]	p11660	Malate dehydrogenase, cytoplasmic
DIP2B pS203	[illegible]	p15072	Disco-interacting protein 2 homolog B
KIAA1522 pS215	[illegible]	p00822	Uncharacterized protein KIAA1522
PRDM2 seq: 737 - 751 + Phospho (ST)	[illegible]	p02095	PR domain zinc finger protein 2
HN1L seq: 113 - 124 no mod	[illegible]	p18774	Hematological and neurological expressed 1-like protein
SMTNL2 pS344	[illegible]	p04203	Smoothelin-like protein 2
TCF20 seq: 1367 - 1389 + Phospho (ST)	[illegible]	p06223	Transcription factor 20
MARK3 pS469	[illegible]	p15911	MAP/microtubule affinity-regulating kinase 3
SUPT5H seq: 669 - 681 + Phospho (ST)	[illegible]	p13654	Transcription elongation factor SPT5
PAK2 pS132	[illegible]	p16372	Serine/threonine-protein kinase PAK 2
KRT8 seq: 317 - 328 no mod	[illegible]	p11049	Keratin, type II cytoskeletal 8
ARHGEF10 seq: 1279 - 1300 + Phospho (ST)	[illegible]	p06097	Rho guanine nucleotide exchange factor 10
MTCL1 pS263	[illegible]	p16282	Microtubule cross-linking factor 1
MISP pS575	[illegible]	p09770	Mitotic interactor and substrate of PLK1
AKAP12 seq: 280 - 298 + Phospho (ST)	[illegible]	p06350	A-kinase anchor protein 12
DUSP16 pS612	[illegible]	p03192	Dual specificity protein phosphatase 16
BAP18 seq: 103 - 133 + Phospho (ST)	[illegible]	p12456	Chromatin complexes subunit BAP18
NCOR2 pS67	[illegible]	p06748	Nuclear receptor corepressor 2
RPRD2 pS909	[illegible]	p10633	Regulation of nuclear pre-mRNA domain-containing protein 2
ZC3HAV1 seq: 271 - 282 + Phospho (ST)	[illegible]	p06127	Zinc finger CCCH-type antiviral protein 1
KHDRBS1 seq: 18 - 31 + Phospho (ST)	[illegible]	p05676	KH domain-containing, RNA-binding, signal transduction-associated protein 1
SRRM2 seq: 1539 - 1556 + Phospho (ST)	[illegible]	p05654	Serine/arginine repetitive matrix protein 2
CDCA5 pS75	[illegible]	p13569	Sororin
PXN pS106	[illegible]	p02083	Paxillin
PEAK1 seq: 819 - 841 + Phospho (ST)	[illegible]	p05235	Pseudopodium-enriched atypical kinase 1
MLPH seq: 420 - 440 no mod	[illegible]	p03342	Melanophilin
ANK1 seq: 9 - 18 + Phospho (ST)	[illegible]	p17997	Ankyrin-1
HSP90B1 seq: 323 - 329 no mod	[illegible]	p02870	Endoplasmin
CTNND2 seq: 397 - 411 + Phospho (Y)	[illegible]	p19428	Catenin delta-2
LMNB1 pS375	[illegible]	p12241	Lamin-B1
GSPT1 seq: 368 - 391 no mod	[illegible]	p15904	Eukaryotic peptide chain release factor GTP-binding subunit ERF3A
ABLIM3 seq: 370 - 384 + Phospho (ST)	[illegible]	p07552	Actin-binding LIM protein 3
ZC3HC1 seq: 354 - 374 + Phospho (ST)	[illegible]	p03203	Nuclear-interacting partner of ALK
SRSF11 pS456	[illegible]	p02670	Serine/arginine-rich splicing factor 11
ORC6 pT195	[illegible]	p14242	Origin recognition complex subunit 6
TBC1D9B pS432 pS435	[illegible]	p13375	TBC1 domain family member 9B
S100A11 seq: 53 - 62 no mod	[illegible]	p09228	Protein S100-A11
KIF16B pS1172	[illegible]	p05234	Kinesin-like protein KIF16B
INCENP seq: 281 - 297 + Phospho (ST)	[illegible]	p01275	Inner centromere protein
TRA2A pT202	[illegible]	p20495	Transformer-2 protein homolog alpha
FILIP1L pT955	[illegible]	p13610	Filamin A-interacting protein 1-like
USP6NL pS396	[illegible]	p03339	USP6 N-terminal-like protein
ARFGEF3 pS2061	[illegible]	p15410	Brefeldin A-inhibited guanine nucleotide-exchange protein 3
PAK1 seq: 142 - 162 + Phospho (ST)	[illegible]	p00213	Serine/threonine-protein kinase PAK 1
CHEK1 pS280	[illegible]	p00680	Serine/threonine-protein kinase Chk1
ARHGAP21 seq: 316 - 336 + Phospho (ST)	[illegible]	p02007	Rho GTPase-activating protein 21
GRIPAP1 seq: 688 - 714 + Phospho (ST)	[illegible]	p04981	GRIP1-associated protein 1
CHAMP1 pS405	[illegible]	p03063	Chromosome alignment-maintaining phosphoprotein 1
DENND4C seq: 947 - 954 + Phospho (ST)	[illegible]	p03831	DENN domain-containing protein 4C
HEXB seq: 204 - 211 no mod	[illegible]	p15878	Beta-hexosaminidase subunit beta
HEXA seq: 171 - 178 no mod	[illegible]	p15709	Beta-hexosaminidase subunit alpha
DDB2 seq: 24 - 35 + Phospho (ST)	[illegible]	p04031	DNA damage-binding protein 2
SIK3 pS568	[illegible]	p07438	Serine/threonine-protein kinase SIK3
CENPV seq: 73 - 115 + Phospho (ST)	[illegible]	p19820	Centromere protein V
AIF1L seq: 130 - 144 + Phospho (ST)	[illegible]	p20058	Allograft inflammatory factor 1-like
PLEKHG3 pS76	[illegible]	p15488	Pleckstrin homology domain-containing family G member 3
FUBP3 pS569	[illegible]	p07993	Far upstream element-binding protein 3
SLTM pS553 pM557	[illegible]	p04641	SAFB-like transcription modulator
EEF2K pS74	[illegible]	p00067	Eukaryotic elongation factor 2 kinase
DYNC1LI2 seq: 398 - 413 + Phospho (ST)	[illegible]	p16003	Cytoplasmic dynein 1 light intermediate chain 2
UBAP2L seq: 601 - 618 + 2 Phospho (ST)	[illegible]	p06404	Ubiquitin-associated protein 2-like
HSPB1 seq: 189 - 198 no mod	[illegible]	p19791	Heat shock protein beta-1
KIF12 seq: 216 - 228 + Phospho (ST)	[illegible]	p07030	Kinesin-like protein KIF12
TJP3 pS591	[illegible]	p04130	Tight junction protein ZO-3
PAN2 seq: 612 - 618 no mod	[illegible]	p11561	PAB-dependent poly(A)-specific ribonuclease subunit PAN2
DDX5 seq: 471 - 478 no mod	[illegible]	p11306	Probable ATP-dependent RNA helicase DDX5
ANP32B seq: 87 - 99 no mod	[illegible]	p11103	Acidic leucine-rich nuclear phosphoprotein 32 family member B
KHDRBS1 seq: 18 - 31 + Phospho (ST)	[illegible]	p05677	KH domain-containing, RNA-binding, signal transduction-associated protein 1
EPN3 seq: 436 - 464 + Phospho (ST)	[illegible]	p17728	Epsin-3
PCDH1 pS949	[illegible]	p16556	Protocadherin-1
ARFGEF3 pS2079	[illegible]	p14648	Brefeldin A-inhibited guanine nucleotide-exchange protein 3
MLLT4 pS1201	[illegible]	p13577	Afadin
WIZ pS1012 pS1017	[illegible]	p09278	Protein Wiz
YAP1 seq: 125 - 161 + Phospho (ST)	[illegible]	p20503	Transcriptional coactivator YAP1
AHNAK pS5830	[illegible]	p16728	Neuroblast differentiation-associated protein AHNAK
TSC22D4 seq: 181 - 208 + Phospho (ST)	[illegible]	p20829	TSC22 domain family protein 4
DYNC1LI1 pS421	[illegible]	p03250	Cytoplasmic dynein 1 light intermediate chain 1
seq: 107 - 124 + Gln->pyro-Glu (N-term Q); Phospho (ST)	[illegible]	p08809	Transcriptional coactivator YAP1
MAPT pS673	[illegible]	p14237	Microtubule-associated protein tau
SRP14 seq: 43 - 55 no mod	[illegible]	p13142	Signal recognition particle 14 kDa protein
CLIP1 seq: 281 - 290 + Phospho (ST)	[illegible]	p14271	CAP-Gly domain-containing linker protein 1
PCNA seq: 150 - 164 no mod	[illegible]	p18539	Proliferating cell nuclear antigen
COIL seq: 270 - 287 + Phospho (ST)	[illegible]	p09085	Coilin
APC seq: 1031 - 1048 + Phospho (ST)	[illegible]	p00126	Adenomatous polyposis coli protein
SRGAP1 seq: 998 - 1018 + 2 Phospho (ST)	[illegible]	p09107	SLIT-ROBO Rho GTPase-activating protein 1
SPECC1L seq: 830 - 842 + Phospho (ST)	[illegible]	p06566	Cytospin-A
YEATS2 pS575	[illegible]	p00923	YEATS domain-containing protein 2
MAP7 seq: 240 - 261 + Phospho (ST)	[illegible]	p05346	Ensconsin
FAM117B seq: 8 - 31 + Phospho (ST)	[illegible]	p09593	Protein FAM117B
DLGAP4 seq: 597 - 623 + Phospho (ST)	[illegible]	p05943	Disks large-associated protein 4
RBM15B pS562	[illegible]	p07022	Putative RNA-binding protein 15B
BCAR3 pS370	[illegible]	p02799	Breast cancer anti-estrogen resistance protein 3
MAP4 pM278 pS280	[illegible]	p18495	Microtubule-associated protein 4
PDIA3 seq: 423 - 433 no mod	[illegible]	p10774	Protein disulfide-isomerase A3
ARHGAP29 seq: 925 - 942 + Phospho (ST)	[illegible]	p05240	Rho GTPase-activating protein 29
TXNDC5 seq: 119 - 134 no mod	[illegible]	p01702	Thioredoxin domain-containing protein 5
SLAIN2 pS391	[illegible]	p10748	SLAIN motif-containing protein 2
PNISR pS211	[illegible]	p07952	Arginine/serine-rich protein PNISR
seq: 34 - 56 + Gln->pyro-Glu (N-term Q); 2 Phospho (ST)	[illegible]	p08102	Protein PML
ASXL2 seq: 712 - 737 + Phospho (ST)	[illegible]	p15959	Putative Polycomb group protein ASXL2
HCFC1 pS1205	[illegible]	p17313	Host cell factor 1
MLLT4 seq: 214 - 223 + Phospho (ST)	[illegible]	p02692	Afadin
VGLL4 pS149	[illegible]	p09151	Transcription cofactor vestigial-like protein 4
INCENP pS306 pS311	[illegible]	p02946	Inner centromere protein
SLC25A43 seq: 327 - 332 + Phospho (ST)	[illegible]	p13249	Solute carrier family 25 member 43
SRRM1 pT846	[illegible]	p19283	Serine/arginine repetitive matrix protein 1
CDCA5 pS75	[illegible]	p13568	Sororin
BCLAF1 pS285	[illegible]	p00086	Bcl-2-associated transcription factor 1
RABEP1 pT408 pS410	[illegible]	p07604	Rab GTPase-binding effector protein 1
SF3B1 pT211	[illegible]	p06412	Splicing factor 3B subunit 1
PPP1R14C seq: 28 - 65 + Phospho (ST)	[illegible]	p16081	Protein phosphatase 1 regulatory subunit 14C
ACO2 seq: 507 - 521 no mod	[illegible]	p16553	Aconitate hydratase, mitochondrial
ARFGEF3 seq: 2095 - 2107 + Phospho (ST)	[illegible]	p05646	Brefeldin A-inhibited guanine nucleotide-exchange protein 3
PRKAB1 seq: 173 - 199 + Phospho (ST)	[illegible]	p19020	5'-AMP-activated protein kinase subunit beta-1
EIF4B seq: 280 - 287 + Phospho (ST)	[illegible]	p20768	Eukaryotic translation initiation factor 4B
HAPLN4 pS111	[illegible]	p20767	Hyaluronan and proteoglycan link protein 4
FAM177A1 pS70 pT71	[illegible]	p01382	Protein FAM177A1
RABEP1 seq: 404 - 420 + 2 Phospho (ST)	[illegible]	p07603	Rab GTPase-binding effector protein 1
SAMD4B seq: 236 - 259 + Phospho (ST)	[illegible]	p04869	Protein Smaug homolog 2
UBAP2 pS473	[illegible]	p10903	Ubiquitin-associated protein 2
CTNND1 pS230	[illegible]	p14549	Catenin delta-1
SORBS2 seq: 163 - 183 + Phospho (ST)	[illegible]	p18951	Sorbin and SH3 domain-containing protein 2
DCAF7 seq: 107 - 115 no mod	[illegible]	p12007	DDB1- and CUL4-associated factor 7
MAP7D1 pS460	[illegible]	p10835	MAP7 domain-containing protein 1
RALGAPA2 seq: 818 - 837 + Phospho (ST)	[illegible]	p06571	Ral GTPase-activating protein subunit alpha-2
GLCCI1 seq: 221 - 231 + Phospho (ST)	[illegible]	p06208	Glucocorticoid-induced transcript 1 protein
SHROOM2 seq: 644 - 662 + Phospho (ST)	[illegible]	p04001	Protein Shroom2
GLCCI1 seq: 221 - 236 + Phospho (ST)	[illegible]	p06207	Glucocorticoid-induced transcript 1 protein
BRD2 pS298	[illegible]	p13459	Bromodomain-containing protein 2
C1orf198 seq: 173 - 183 + Phospho (ST)	[illegible]	p03699	Uncharacterized protein C1orf198
FAM46C seq: 129 - 135 + Phospho (ST)	[illegible]	p13679	Protein FAM46C
GPALPP1 pS105	[illegible]	p08776	GPALPP motifs-containing protein 1
AMPD2 pS100	[illegible]	p05119	AMP deaminase 2
PSAP seq: 337 - 344 no mod	[illegible]	p17615	Prosaposin
ICE1 pS925	[illegible]	p05465	Little elongation complex subunit 1
AHNAK seq: 3357 - 3372 + Phospho (ST)	[illegible]	p16563	Neuroblast differentiation-associated protein AHNAK
MAP7D1 seq: 93 - 104 + Phospho (ST)	[illegible]	p04113	MAP7 domain-containing protein 1
SOD1 pS35	[illegible]	p00553	Superoxide dismutase [Cu-Zn]
SOX5 seq: 18 - 36 + Phospho (ST)	[illegible]	p06967	Transcription factor SOX-5
RBM15B seq: 265 - 278 + 2 Phospho (ST)	[illegible]	p05006	Putative RNA-binding protein 15B
NSFL1C pS140	[illegible]	p04650	NSFL1 cofactor p47
KIF21A seq: 1260 - 1281 + 2 Phospho (ST)	[illegible]	p14650	Kinesin-like protein KIF21A
PSMD1 seq: 270 - 302 + Phospho (ST)	[illegible]	p01964	26S proteasome non-ATPase regulatory subunit 1
seq: 750 - 783 + Gln->pyro-Glu (N-term Q); Phospho (ST)	[illegible]	p08304	Synergin gamma
KIF4A seq: 594 - 599 no mod	[illegible]	p10809	Chromosome-associated kinesin KIF4A
PAPOLA seq: 19 - 32 + Phospho (ST)	[illegible]	p14543	Poly(A) polymerase alpha
ARHGEF10 seq: 1279 - 1300 + 2 Phospho (ST)	[illegible]	p06098	Rho guanine nucleotide exchange factor 10
HSP90AA1 seq: 500 - 510 no mod	[illegible]	p18361	Heat shock protein HSP 90-alpha
SHROOM3 seq: 213 - 241 + Phospho (ST)	[illegible]	p04697	Protein Shroom3
KRT18 pM74	[illegible]	p16013	Keratin, type I cytoskeletal 18
NAA15 seq: 757 - 765 + Phospho (ST)	[illegible]	p07027	N-alpha-acetyltransferase 15, NatA auxiliary subunit
AHNAK pS2387	[illegible]	p13703	Neuroblast differentiation-associated protein AHNAK
CLASP1 seq: 793 - 809 + Phospho (ST)	[illegible]	p01144	CLIP-associating protein 1
TRIP11 seq: 1881 - 1895 + Oxidation (M); Phospho (ST)	[illegible]	p10603	Thyroid receptor-interacting protein 11
NONO seq: 435 - 456 no mod	[illegible]	p16742	Non-POU domain-containing octamer-binding protein
GON4L pS346	[illegible]	p14969	GON-4-like protein
SRRM2 seq: 1527 - 1536 + Phospho (ST)	[illegible]	p01982	Serine/arginine repetitive matrix protein 2
SORBS2 pS259	[illegible]	p07213	Sorbin and SH3 domain-containing protein 2
IDH1 seq: 261 - 270 no mod	[illegible]	p06028	Isocitrate dehydrogenase [NADP] cytoplasmic
ZC3H11A pS132	[illegible]	p10594	Zinc finger CCCH domain-containing protein 11A
ACTG1 seq: 53 - 64 no mod	[illegible]	p18195	Actin, cytoplasmic 2
GTF2I seq: 556 - 567 + Phospho (ST)	[illegible]	p02443	General transcription factor II-I
CHTF8 seq: 68 - 91 + Phospho (ST)	[illegible]	p09250	Chromosome transmission fidelity protein 8 homolog isoform 2
CLASRP pS285	[illegible]	p13068	CLK4-associating serine/arginine rich protein
SLTM seq: 549 - 564 + 2 Phospho (ST)	[illegible]	p13665	SAFB-like transcription modulator
EP400 pS1547	[illegible]	p14673	E1A-binding protein p400
AMOTL2 seq: 174 - 189 + Phospho (ST)	[illegible]	p19917	Angiomotin-like protein 2
LATS1 pS613	[illegible]	p08485	Serine/threonine-protein kinase LATS1
MAP1B seq: 1912 - 1928 + Phospho (ST)	[illegible]	p02045	Microtubule-associated protein 1B
NFX1 seq: 47 - 58 + Phospho (ST)	[illegible]	p08909	Transcriptional repressor NF-X1
VIM pS73	[illegible]	p10857	Vimentin
BCAR3 pS338	[illegible]	p20525	Breast cancer anti-estrogen resistance protein 3
AHNAK seq: 3403 - 3414 + Phospho (ST)	[illegible]	p16646	Neuroblast differentiation-associated protein AHNAK

IGF2BP2 seq: 152 - 168 + Phospho (ST)	4E+07	2E+07	[illegible]	3E+08	p13627	Insulin-like growth factor 2 mRNA-binding protein 2
FAM53C pS247	9E+07	8E+07	[illegible]	8E+07	p16596	Protein FAM53C
UBL4A pS90	7E+08	1E+09	[illegible]	2E+08	p12286	Ubiquitin-like protein 4A
PDIA3 seq: 235 - 258 no mod	38324	2E+07	[illegible]	1E+08	p16673	Protein disulfide-isomerase A3
ITGB4 seq: 1447 - 1460 + 2 Phospho (ST)	4E+07	9E+07	[illegible]	5E+08	p10218	Integrin beta-4
GIT1 seq: 559 - 582 + Phospho (ST)	3E+08	3E+08	[illegible]	8E+07	p14993	ARF GTPase-activating protein GIT1
KHDRBS1 seq: 18 - 36 + Phospho (ST)	8E+07	1E+08	[illegible]	2E+08	p05675	KH domain-containing, RNA-binding, signal transduction-associated protein 1
TPM2 seq: 92 - 105 no mod	7E+07	1E+08	[illegible]	2E+08	p13806	Tropomyosin beta chain
ZC3H18 pS532 pS534	1E+08	1E+08	[illegible]	2E+08	p11662	Zinc finger CCCH domain-containing protein 18
NUP35 seq: 93 - 111 + Phospho (ST)	3E+07	5E+07	[illegible]	5E+07	p05440	Nucleoporin NUP53
ULK1 seq: 476 - 492 + Phospho (ST)	1E+09	1E+09	[illegible]	1E+09	p19529	Serine/threonine-protein kinase ULK1
CSTF2T pS563	1E+08	7E+07	[illegible]	25732	p08646	Cleavage stimulation factor subunit 2 tau variant
FAM83H pS1025	5E+08	5E+08	[illegible]	7E+08	p10677	Protein FAM83H
RPS19 seq: 30 - 38 no mod	3E+06	2E+07	[illegible]	25732	p01018	40S ribosomal protein S19
MKL2 seq: 535 - 561 + Oxidation (M); Phospho (ST)	1E+08	1E+08	[illegible]	25732	p09850	MKL/myocardin-like protein 2
ENO1 seq: 184 - 193 no mod	8E+08	1E+09	[illegible]	1E+09	p14282	Alpha-enolase
FAM122A seq: 35 - 59 + 2 Phospho (ST)	4E+07	3E+07	[illegible]	2E+08	p04817	Protein FAM122A
TANC2 seq: 1564 - 1583 + Phospho (ST)	5E+08	6E+08	[illegible]	1E+08	p00153	Protein TANC2
INCENP seq: 281 - 297 + Phospho (ST)	8E+08	8E+08	[illegible]	6E+08	p01276	Inner centromere protein
TPI1 seq: 187 - 197 no mod	6E+07	2E+08	[illegible]	8E+07	p01409	Triosephosphate isomerase
KRT8 seq: 19 - 32 + Phospho (ST)	2E+07	5E+07	[illegible]	3E+07	p20743	Keratin, type II cytoskeletal 8
NFATC2IP pS204	4E+08	4E+08	[illegible]	3E+08	p02914	NFATC2-interacting protein
AMOTL2 pS183	2E+08	3E+08	[illegible]	2E+09	p19918	Angiomotin-like protein 2
MLPH pS314	4E+08	5E+08	[illegible]	1E+08	p09667	Melanophilin
KRT8 seq: 226 - 233 no mod	5E+07	4E+07	[illegible]	8E+07	p08255	Keratin, type II cytoskeletal 8
FOXA1 pS307	2E+08	3E+08	[illegible]	25732	p13330	Hepatocyte nuclear factor 3-alpha
KRT8 seq: 353 - 362 no mod	5E+07	1E+08	[illegible]	5E+07	p10813	Keratin, type II cytoskeletal 8
BAIAP2 seq: 488 - 508 + Phospho (ST)	1E+08	2E+08	[illegible]	3E+08	p07967	Brain-specific angiogenesis inhibitor 1-associated protein 2
YWHAG seq: 29 - 42 no mod	3E+08	2E+08	[illegible]	4E+07	p08936	14-3-3 protein gamma
SRRM1 pS402	2E+09	2E+09	[illegible]	6E+08	p14658	Serine/arginine repetitive matrix protein 1
HIST1H4A seq: 69 - 78 no mod	1E+08	3E+08	[illegible]	5E+07	p18939	Histone H4
SPAG9 pT586	1E+08	2E+08	[illegible]	2E+08	p00210	C-Jun-amino-terminal kinase-interacting protein 4
IFT81 seq: 208 - 213 no mod	3E+10	4E+10	[illegible]	2E+10	p14476	Intraflagellar transport protein 81 homolog
DVL3 seq: 106 - 143 + Phospho (ST)	7E+08	8E+08	[illegible]	6E+08	p02818	Segment polarity protein dishevelled homolog DVL-3
SLAIN2 pS413	2E+09	2E+09	[illegible]	3E+08	p05112	SLAIN motif-containing protein 2
ATAD2 seq: 335 - 347 + 2 Phospho (ST)	3E+08	2E+08	[illegible]	1E+08	p10681	ATPase family AAA domain-containing protein 2
ARFGEF2 seq: 612 - 641 + Phospho (ST)	3E+08	2E+08	[illegible]	25732	p07534	Brefeldin A-inhibited guanine nucleotide-exchange protein 2
MAP4 pS624	38324	1E+08	[illegible]	9E+08	p15853	Microtubule-associated protein 4
CD3EAP seq: 117 - 148 + 2 Phospho (ST)	1E+08	2E+08	[illegible]	5E+08	p14063	DNA-directed RNA polymerase I subunit RPA34
ULK1 pS556	2E+06	3E+07	[illegible]	4E+07	p11635	Serine/threonine-protein kinase ULK1
TFF3 seq: 38 - 50 no mod	1E+08	2E+08	[illegible]	25732	p18314	Trefoil factor 3
MAP4 pS624	3E+07	2E+08	[illegible]	5E+08	p15855	Microtubule-associated protein 4
FOXA1 seq: 419 - 447 + Phospho (ST)	3E+08	5E+08	[illegible]	25732	p19129	Hepatocyte nuclear factor 3-alpha
MAP1B seq: 1394 - 1423 + Phospho (ST)	38324	37149	[illegible]	2E+08	p01162	Microtubule-associated protein 1B
MAP1S pM753 pS759	3E+08	4E+08	[illegible]	4E+07	p19211	Microtubule-associated protein 1S
CTNNB1 pS552	3E+08	4E+08	[illegible]	3E+08	p06490	Catenin beta-1
ZAK pS637	7E+08	5E+08	[illegible]	4E+08	p03655	Mitogen-activated protein kinase kinase kinase MLT
TJP2 pT1027	1E+08	6E+07	[illegible]	3E+08	p04822	Tight junction protein ZO-2
NOLC1 pT607 pT610	6E+07	6E+07	[illegible]	2E+08	p14110	Nucleolar and coiled-body phosphoprotein 1
HNRNPA2B1 seq: 154 - 173 no mod	38324	3E+07	[illegible]	25732	p16119	Heterogeneous nuclear ribonucleoproteins A2/B1
TANC1 seq: 1656 - 1674 + Phospho (ST)	8E+07	7E+07	[illegible]	1E+07	p14700	Protein TANC1
[illegible]seq: 331 - 351 + Gln->pyro-Glu (N-term Q); Phospho (ST)	1E+08	2E+08	[illegible]	25732	p08157	MKL/myocardin-like protein 1
CDCA3 seq: 60 - 75 + Phospho (ST)	6E+08	6E+08	[illegible]	1E+08	p14887	Cell division cycle-associated protein 3
NCL pT121	2E+09	2E+09	[illegible]	3E+09	p20120	Nucleolin
SLC4A2 seq: 169 - 185 + Phospho (ST)	2E+08	2E+08	[illegible]	2E+08	p02136	Anion exchange protein 2
HNRNPC pS233	2E+08	4E+08	[illegible]	8E+08	p08795	Heterogeneous nuclear ribonucleoproteins C1/C2
DDX39A seq: 48 - 57 no mod	38324	5E+07	[illegible]	3E+07	p18877	ATP-dependent RNA helicase DDX39A
GAB2 seq: 443 - 467 + Phospho (ST)	7E+07	8E+07	[illegible]	25732	p06072	GRB2-associated-binding protein 2
EEF1D seq: 124 - 136 no mod	1E+08	2E+08	[illegible]	2E+08	p19421	Elongation factor 1-delta
KIAA1211L pS92	4E+08	7E+08	[illegible]	2E+08	p20177	Uncharacterized protein KIAA1211-like
AHNAK pT490	5E+08	9E+08	[illegible]	2E+09	p01291	Neuroblast differentiation-associated protein AHNAK
NFATC2IP seq: 231 - 263 + Phospho (ST)	4E+08	7E+08	[illegible]	2E+08	p06148	NFATC2-interacting protein
MAPT seq: 575 - 584 + Phospho (ST)	7E+07	9E+07	[illegible]	25732	p05408	Microtubule-associated protein tau
PGRMC1 pS57	6E+09	2E+10	[illegible]	5E+09	p13537	Membrane-associated progesterone receptor component 1
GOLGA2 pS981	6E+07	1E+08	[illegible]	4E+07	p17222	Golgin subfamily A member 2
LIMA1 seq: 614 - 629 + Phospho (ST)	6E+08	7E+08	[illegible]	2E+08	p13129	LIM domain and actin-binding protein 1
NOLC1 pT607 pT610	6E+07	5E+07	[illegible]	2E+08	p10924	Nucleolar and coiled-body phosphoprotein 1
RBMX seq: 81 - 94 + Phospho (ST)	5E+07	6E+07	[illegible]	7E+07	p01602	RNA-binding motif protein, X chromosome
CLSPN seq: 948 - 966 + Phospho (ST)	3E+07	3E+07	[illegible]	7E+07	p16413	Claspin
PKM seq: 462 - 467 + Gln->pyro-Glu (N-term Q)	3E+08	4E+08	[illegible]	3E+08	p08852	Pyruvate kinase PKM
FAM83B seq: 800 - 818 + Phospho (ST)	2E+08	2E+08	[illegible]	4E+07	p12115	Protein FAM83B
PRRC2C seq: 773 - 791 + Phospho (ST)	4E+07	5E+07	[illegible]	1E+08	p07524	Protein PRRC2C
PDS5A seq: 1204 - 1217 + Phospho (ST)	4E+07	6E+07	[illegible]	2E+07	p14130	Sister chromatid cohesion protein PDS5 homolog A
SKI pS432	3E+08	4E+08	[illegible]	4E+06	p00573	Ski oncogene
ADD1 pS586	4E+08	5E+08	[illegible]	25732	p08462	Alpha-adducin
PRC1 pT481	3E+08	4E+08	[illegible]	8E+07	p07382	Protein regulator of cytokinesis 1
CCT8 pS317	5E+07	4E+07	[illegible]	7E+06	p11160	T-complex protein 1 subunit theta
ZBTB7A seq: 505 - 530 + Phospho (ST)	1E+09	2E+09	[illegible]	1E+09	p16077	Zinc finger and BTB domain-containing protein 7A
PRRC2A seq: 454 - 466 + Phospho (ST)	4E+07	8E+07	[illegible]	1E+07	p12639	Protein PRRC2A
HMGN2 pS29	8E+08	2E+09	[illegible]	3E+09	p10842	Non-histone chromosomal protein HMG-17
BAP18 seq: 103 - 133 + Phospho (ST)	5E+08	1E+09	[illegible]	3E+08	p12455	Chromatin complexes subunit BAP18
FLNB seq: 1220 - 1230 no mod	2E+08	1E+08	[illegible]	2E+07	p01285	Filamin-B
NEDD4L pS446 pS464	1E+08	1E+08	[illegible]	2E+08	p04986	E3 ubiquitin-protein ligase NEDD4-like
MAPT pS579	8E+08	1E+09	[illegible]	2E+08	p05409	Microtubule-associated protein tau
CLASP1 seq: 555 - 570 + Phospho (ST)	38324	37149	[illegible]	3E+08	p03627	CLIP-associating protein 1
SHROOM2 pS847	3E+08	3E+08	[illegible]	25732	p02014	Protein Shroom2
ANKS1A seq: 622 - 644 + Oxidation (M); Phospho (ST)	3E+08	1E+08	[illegible]	1E+08	p05051	Ankyrin repeat and SAM domain-containing protein 1A
PCM1 seq: 382 - 399 + Phospho (ST)	3E+07	37149	[illegible]	4E+08	p12743	Pericentriolar material 1 protein
KTN1 pS75	38324	5E+08	[illegible]	4E+08	p17604	Kinectin
AHDC1 pS1549	1E+08	1E+08	[illegible]	9E+07	p15545	AT-hook DNA-binding motif-containing protein 1
AP3D1 seq: 673 - 691 + Phospho (ST)	4E+08	4E+08	[illegible]	9E+07	p12686	AP-3 complex subunit delta-1
MAP3K2 pS153	2E+08	5E+08	[illegible]	1E+08	p07134	Mitogen-activated protein kinase kinase kinase 2
NOLC1 pS397	4E+08	3E+08	[illegible]	4E+08	p04705	Nucleolar and coiled-body phosphoprotein 1
CLASP1 pS797	1E+08	1E+08	[illegible]	7E+08	p01145	CLIP-associating protein 1
CEP170 pS1198	2E+07	3E+07	[illegible]	7E+07	p10376	Centrosomal protein of 170 kDa
HNRNPU seq: 627 - 635 no mod	1E+08	2E+08	[illegible]	25732	p18564	Heterogeneous nuclear ribonucleoprotein U
PPP1R12A pS871	2E+08	2E+08	[illegible]	5E+08	p03517	Protein phosphatase 1 regulatory subunit 12A
IRF2BP1 pS125	3E+08	4E+08	[illegible]	5E+07	p11111	Interferon regulatory factor 2-binding protein 1
SAMD4B pS628	4E+07	3E+07	[illegible]	1E+08	p08207	Protein Smaug homolog 2
PANK2 seq: 185 - 198 + Phospho (ST)	3E+08	2E+08	[illegible]	8E+08	p18347	Pantothenate kinase 2, mitochondrial
ARPP21 pS383	9E+08	1E+09	[illegible]	25732	p03038	cAMP-regulated phosphoprotein 21
ST13P4 seq: 119 - 132 no mod	6E+08	4E+08	[illegible]	6E+07	p01846	Putative protein FAM10A4
DNAJC17 pS112	9E+07	9E+07	[illegible]	5E+07	p17208	DnaJ homolog subfamily C member 17
NONO seq: 399 - 434 + Phospho (ST)	1E+09	1E+09	[illegible]	1E+09	p16286	Non-POU domain-containing octamer-binding protein
TRIM28 seq: 471 - 483 + Oxidation (M); Phospho (ST)	8E+08	8E+08	[illegible]	4E+08	p04056	Transcription intermediary factor 1-beta
ARHGAP29 seq: 947 - 955 + Phospho (ST)	38324	37149	[illegible]	7E+07	p19334	Rho GTPase-activating protein 29
BAIAP2 pT360	9E+07	1E+08	[illegible]	2E+08	p09044	Brain-specific angiogenesis inhibitor 1-associated protein 2
NACA2 seq: 143 - 179 + Phospho (ST)	5E+08	7E+08	[illegible]	1E+09	p16493	Nascent polypeptide-associated complex subunit alpha-2
NUP153 seq: 622 - 657 + Phospho (ST)	38324	9E+07	[illegible]	1E+08	p14391	Nuclear pore complex protein Nup153
ZNRF1 pS123	7E+07	2E+08	[illegible]	5E+07	p19593	E3 ubiquitin-protein ligase ZNRF1
EMSY pS1213	5E+08	3E+08	[illegible]	2E+08	p17181	Protein EMSY
FAM122B seq: 221 - 247 + Phospho (ST)	1E+08	1E+08	[illegible]	4E+08	p15311	Protein FAM122B
DYNC1LI1 pS398	4E+08	3E+08	[illegible]	1E+08	p08113	Cytoplasmic dynein 1 light intermediate chain 1
TAGLN2 seq: 89 - 102 no mod	38324	4E+07	[illegible]	7E+07	p08249	Transgelin-2
PA2G4 seq: 312 - 320 no mod	8E+08	1E+09	[illegible]	7E+07	p17759	Proliferation-associated protein 2G4
NEDD4L seq: 469 - 493 + 2 Phospho (ST)	3E+08	2E+08	[illegible]	6E+08	p19234	E3 ubiquitin-protein ligase NEDD4-like
SYMPK seq: 1243 - 1271 + Phospho (ST)	2E+08	2E+08	[illegible]	1E+08	p04450	Symplekin
AMOT seq: 304 - 330 + Phospho (ST)	2E+08	1E+08	[illegible]	25732	p09093	Angiomotin
DLG5 pT1011	7E+07	2E+08	[illegible]	6E+07	p20601	Disks large homolog 5
ZC3H4 seq: 895 - 916 + Phospho (ST)	1E+09	2E+09	[illegible]	1E+09	p20202	Zinc finger CCCH domain-containing protein 4
MISP seq: 4 - 17 + Phospho (Y)	38324	37149	[illegible]	8E+07	p00681	Mitotic interactor and substrate of PLK1
MBP pT229	2E+08	37149	[illegible]	4E+08	p09527	Myelin basic protein
EIF4ENIF1 pS577	5E+08	5E+08	[illegible]	2E+08	p21056	Eukaryotic translation initiation factor 4E transporter
MYO18A seq: 88 - 114 + Phospho (ST)	1E+08	1E+08	[illegible]	1E+08	p15147	Unconventional myosin-XVIIIa
ZNF768 pS125	2E+08	9E+08	[illegible]	3E+08	p00404	Zinc finger protein 768
HSPA8 seq: 57 - 71 no mod	8E+08	1E+09	[illegible]	7E+08	p09206	Heat shock cognate 71 kDa protein
HECTD1 pS1533	6E+07	1E+08	[illegible]	3E+08	p10593	E3 ubiquitin-protein ligase HECTD1
MYO18A pS74	3E+08	2E+08	[illegible]	1E+08	p01761	Unconventional myosin-XVIIIa
LASP1 seq: 60 - 73 no mod	9E+06	3E+07	[illegible]	25732	p07920	LIM and SH3 domain protein 1
GPRASP2 seq: 510 - 529 + Phospho (ST)	1E+08	2E+08	[illegible]	3E+08	p03445	G-protein coupled receptor-associated sorting protein 2
LRBA pS1767	6E+08	6E+08	[illegible]	2E+08	p06167	Lipopolysaccharide-responsive and beige-like anchor protein
MSL1 seq: 400 - 412 + Phospho (ST)	2E+08	4E+08	[illegible]	9E+08	p10615	Male-specific lethal 1 homolog
CTNND1 seq: 916 - 930 + Phospho (ST)	2E+08	1E+08	[illegible]	2E+08	p02624	Catenin delta-1
TBC1D4 seq: 93 - 122 + Phospho (ST)	6E+08	5E+08	[illegible]	5E+08	p19003	TBC1 domain family member 4
STIM2 pS523	3E+08	1E+09	[illegible]	2E+08	p05443	Stromal interaction molecule 2
PPIAL4D seq: 119 - 125 no mod	9E+08	8E+08	[illegible]	3E+08	p02878	Peptidyl-prolyl cis-trans isomerase A-like 4D
CEP170B pS492	4E+08	5E+08	[illegible]	5E+08	p02423	Centrosomal protein of 170 kDa protein B
PEBP1 seq: 94 - 113 no mod	7E+08	8E+08	[illegible]	4E+08	p15594	Phosphatidylethanolamine-binding protein 1
STMN2 seq: 120 - 129 no mod	1E+10	2E+10	[illegible]	3E+10	p20284	Stathmin-2
CHAF1B seq: 408 - 423 + Phospho (ST)	3E+09	2E+09	[illegible]	2E+09	p15170	Chromatin assembly factor 1 subunit B
CCNK pS340	6E+08	5E+08	[illegible]	5E+08	p07538	Cyclin-K
LYSMD2 pS24	1E+08	1E+08	[illegible]	6E+08	p19938	LysM and putative peptidoglycan-binding domain-containing protein 2
CAST pS243	1E+08	2E+08	[illegible]	2E+09	p17733	Calpastatin
ITGB4 pS1069	5E+06	5E+07	[illegible]	1E+09	p11416	Integrin beta-4
ABLIM3 pS576	6E+06	5E+06	[illegible]	3E+07	p05543	Actin-binding LIM protein 3
NOP56 seq: 511 - 534 + Phospho (ST)	3E+08	8E+08	[illegible]	1E+09	p05845	Nucleolar protein 56
GIT2 seq: 413 - 434 + Phospho (ST)	9E+06	4E+07	[illegible]	4E+07	p08433	ARF GTPase-activating protein GIT2
CDV3 pS8	4E+08	5E+08	[illegible]	5E+08	p05287	Protein CDV3 homolog
BCLAF1 pS268	1E+10	7E+09	[illegible]	25732	p09008	Bcl-2-associated transcription factor 1
KRT19 pS234	6E+08	3E+08	[illegible]	2E+08	p15366	Keratin, type I cytoskeletal 19
SRRM2 pT2599	2E+08	3E+08	[illegible]	6E+08	p17691	Serine/arginine repetitive matrix protein 2
NCOR1 pS2184	2E+09	3E+09	[illegible]	2E+09	p04617	Nuclear receptor corepressor 1
PDZD2 pS920	6E+08	4E+08	[illegible]	2E+08	p20030	PDZ domain-containing protein 2
PALLD seq: 736 - 770 + Phospho (ST)	3E+08	4E+08	[illegible]	6E+08	p08691	Palladin
ZCCHC8 pS658	1E+08	1E+08	[illegible]	4E+08	p14203	Zinc finger CCHC domain-containing protein 8
EP400 seq: 742 - 784 + Phospho (ST)	38324	3E+08	[illegible]	25732	p02391	E1A-binding protein p400
MAP1B seq: 1809 - 1833 + Phospho (ST)	9E+07	3E+07	[illegible]	2E+08	p17546	Microtubule-associated protein 1B
PKM pM291	4E+06	2E+08	[illegible]	1E+08	p07448	Pyruvate kinase PKM
MRE11A pS688 pS689	4E+08	3E+08	[illegible]	9E+08	p15052	Double-strand break repair protein MRE11A
PPP1R13B seq: 708 - 724 + Phospho (ST)	4E+08	5E+08	[illegible]	7E+07	p06611	Apoptosis-stimulating of p53 protein 1
SRSF9 seq: 210 - 221 + Phospho (Y)	6E+08	7E+08	[illegible]	1E+09	p15224	Serine/arginine-rich splicing factor 9
MAST2 pS1240	2E+08	2E+08	[illegible]	6E+06	p08822	Microtubule-associated serine/threonine-protein kinase 2
ARHGAP21 seq: 474 - 491 + Phospho (ST)	1E+08	2E+08	[illegible]	1E+09	p06238	Rho GTPase-activating protein 21
MKI67 pS308	38324	37149	[illegible]	2E+08	p05746	Antigen KI-67
RBSN pS226	1E+08	7E+07	[illegible]	1E+07	p17147	Rabenosyn-5
HMG20A pS105	4E+08	4E+08	[illegible]	5E+08	p18238	High mobility group protein 20A
SRRM1 seq: 836 - 869 + Phospho (ST)	1E+08	7E+07	[illegible]	8E+07	p19282	Serine/arginine repetitive matrix protein 1
SRRM2 seq: 346 - 356 + Phospho (ST)	6E+08	6E+08	[illegible]	5E+08	p06191	Serine/arginine repetitive matrix protein 2
MARCKS pT150	5E+08	8E+08	[illegible]	2E+08	p20861	Myristoylated alanine-rich C-kinase substrate
ERCC6L pS820	1E+08	8E+07	[illegible]	3E+08	p16111	DNA excision repair protein ERCC-6-like
ZNF318 seq: 1035 - 1049 + Phospho (ST)	6E+07	37149	[illegible]	25732	p02650	Zinc finger protein 318
BTF3 seq: 178 - 206 no mod	9E+08	8E+08	[illegible]	1E+09	p19972	Transcription factor BTF3
CLASP1 pS646	2E+08	2E+08	[illegible]	4E+08	p06782	CLIP-associating protein 1
C10orf12 seq: 288 - 297 + Phospho (ST)	38324	3E+08	[illegible]	3E+08	p14108	Uncharacterized protein C10orf12
ZSWIM8 pS53	1E+08	7E+07	[illegible]	2E+07	p12652	Zinc finger SWIM domain-containing protein 8
AMOTL1 pS828	1E+08	1E+08	[illegible]	25732	p15266	Angiomotin-like protein 1
NCAPH pS81	2E+08	2E+08	[illegible]	2E+08	p01567	Condensin complex subunit 2
TRPS1 pS1081	1E+08	2E+08	[illegible]	25732	p14695	Zinc finger transcription factor Trps1
ESYT1 seq: 818 - 833 + Phospho (Y)	4E+06	37149	[illegible]	2E+07	p14729	Extended synaptotagmin-1
ARHGAP21 seq: 1666 - 1687 + Phospho (ST)	2E+08	2E+08	[illegible]	5E+08	p07026	Rho GTPase-activating protein 21
SCRIB seq: 1536 - 1574 + Phospho (ST)	1E+08	2E+08	[illegible]	4E+08	p12279	Protein scribble homolog
ANXA5 seq: 311 - 320 no mod	3E+08	3E+08	[illegible]	2E+08	p20235	Annexin A5
C2CD5 seq: 657 - 675 + 2 Phospho (ST)	2E+08	1E+08	[illegible]	4E+07	p04209	C2 domain-containing protein 5
VIM seq: 14 - 28 + Phospho (ST)	38324	37149	[illegible]	4E+08	p10144	Vimentin
THRAP3 pS253	4E+08	4E+08	[illegible]	9E+07	p04545	Thyroid hormone receptor-associated protein 3
KRT75 pS334	4E+07	1E+08	[illegible]	1E+08	p19724	Keratin, type II cytoskeletal 75
GIGYF2 seq: 362 - 396 + Phospho (ST)	4E+09	2E+09	[illegible]	3E+09	p01430	PERQ amino acid-rich with GYF domain-containing protein 2
NEDD4L seq: 340 - 362 + Phospho (ST)	4E+09	3E+09	[illegible]	3E+09	p06129	E3 ubiquitin-protein ligase NEDD4-like
DBP pS86	4E+08	4E+08	[illegible]	25732	p13473	D site-binding protein
TMPO pS351	6E+08	4E+08	[illegible]	4E+08	p05597	Lamina-associated polypeptide 2, isoform alpha
PPP1R9B pS94	6E+07	9E+07	[illegible]	25732	p14995	Neurabin-2
BAP18 pS96	8E+08	1E+09	[illegible]	1E+09	p00543	Chromatin complexes subunit BAP18
PLEC pS4406	5E+08	6E+08	[illegible]	2E+09	p02280	Plectin
NUMA1 pT2106	1E+08	1E+08	[illegible]	2E+08	p14462	Nuclear mitotic apparatus protein 1
CBX8 seq: 104 - 119 + Phospho (ST)	3E+08	3E+08	[illegible]	1E+08	p13843	Chromobox protein homolog 8
AMOTL2 seq: 667 - 690 + Phospho (ST)	4E+07	3E+07	[illegible]	7E+08	p03299	Angiomotin-like protein 2
NHSL1 seq: 849 - 874 + Phospho (ST)	1E+07	3E+07	[illegible]	7E+07	p01344	NHS-like protein 1
MAP7 seq: 181 - 189 + Phospho (ST)	1E+09	1E+09	[illegible]	8E+08	p03242	Ensconsin
YAP1 pS131 pS138	2E+08	2E+08	[illegible]	2E+09	p20507	Transcriptional coactivator YAP1
LMNB1 seq: 388 - 397 + 2 Phospho (ST)	6E+09	4E+09	[illegible]	5E+09	p11503	Lamin-B1
FAM83H seq: 934 - 947 + Phospho (ST)	8E+08	2E+09	[illegible]	6E+08	p07334	Protein FAM83H
ZC3H13 seq: 204 - 213 + 2 Phospho (ST)	6E+09	4E+09	[illegible]	5E+09	p05403	Zinc finger CCCH domain-containing protein 13
KMT2A seq: 2094 - 2108 + Phospho (ST)	5E+07	6E+07	[illegible]	3E+07	p02734	Histone-lysine N-methyltransferase 2A
WRNIP1 pS75	2E+08	2E+08	[illegible]	2E+08	p07139	ATPase WRNIP1
MASTL pS370	1E+08	1E+08	[illegible]	2E+08	p18625	Serine/threonine-protein kinase greatwall
KRT17 seq: 171 - 180 no mod	3E+08	4E+08	[illegible]	2E+08	p02662	Keratin, type I cytoskeletal 17
KRT7 seq: 287 - 296 no mod	6E+08	5E+08	[illegible]	7E+08	p16789	Keratin, type II cytoskeletal 7
BCLAF1 pS422	2E+08	2E+08	[illegible]	2E+07	p03324	Bcl-2-associated transcription factor 1
SAP30BP pS113	4E+08	3E+08	[illegible]	3E+08	p09362	SAP30-binding protein
CTNND1 pS230	7E+08	6E+08	[illegible]	1E+09	p14548	Catenin delta-1

PSMD13 seq: 133 - 154 no mod	5E+07 1E+08 3E+08 3E+08 3E+08 6E+08 2E+08 2E+08 2E+08 2E+08 3E+08 7E+07 8E+07 7E+07 7E+07 4E+08 2E+08 3E+08 2E+08 2E+08 4E+08 2E+08 2E+08 3E+08 2E+08 3E+08 2E+08 2E+08 2E+08 2E+08 3E+08 2E+08 5E+07 3E+08 2E+08 2E+08 1E+08 1E+08 1E+08 1E+08 3E+08 4E+08 3E+08 3E+08 3E+08 2E+08 3E+08 2E+08 3E+08 2E+08	p17062	26S proteasome non-ATPase regulatory subunit 13
TCN1 seq: 381 - 399 no mod	3E+07 2E+08 8E+07 1E+08 2E+08 2E+08 4E+08 9E+07 7E+07 2E+08 3E+08 4E+08 7E+07 1E+08 2E+08 2E+08 7E+07 7E+07 2E+08 1E+08 1E+08 5E+07 9E+06 2E+08 2E+08 68554 44396 34144 19500 29103 45838 31655 19257 24890 17227 8E+06 27262 25062 27887 36936 100327 64618 38440 41974 13188 134692 41467 25947 30272 25732	p03197	Transcobalamin-1
PKP2 pS329	4E+08 6E+08 7E+08 4E+08 4E+08 2E+09 8E+08 7E+08 5E+08 4E+08 6E+08 3E+08 3E+08 2E+08 2E+08 1E+09 6E+08 9E+08 8E+08 6E+08 9E+08 4E+08 6E+08 5E+08 6E+08 2E+09 7E+08 6E+08 7E+08 7E+08 9E+08 7E+08 4E+08 7E+08 8E+08 9E+08 6E+08 4E+08 4E+08 5E+08 1E+09 9E+08 7E+08 7E+08 8E+08 1E+09 9E+08 9E+08 7E+08 8E+08	p20528	Plakophilin-2
CAST seq: 213 - 250 + Phospho (ST)	1E+09 1E+09 2E+09 2E+09 2E+09 3E+09 2E+09 1E+09 1E+09 1E+09 5E+09 3E+09 2E+09 3E+09 2E+09 2E+09 1E+09 2E+09 2E+09 1E+09 2E+09 1E+09 1E+09 2E+09 1E+09 3E+10 1E+10 1E+10 2E+10 1E+10 1E+10 7E+09 7E+09 1E+10 2E+10 2E+10 1E+10 1E+10 1E+10 1E+10 2E+10 1E+10 1E+10 1E+10 1E+10 2E+10 7E+09 1E+10 2E+10 1E+10	p17732	Calpastatin
CTNND2 seq: 472 - 488 + Phospho (ST)	5E+07 8E+07 9E+07 3E+08 2E+08 3E+08 2E+08 1E+08 4E+08 3E+08 1E+08 6E+07 5E+07 2E+08 1E+08 3E+08 5E+07 2E+08 3E+08 2E+08 2E+08 4E+07 9E+07 3E+08 2E+08 68554 44396 34144 19500 29103 45838 31655 19257 24890 17227 44771 27262 25062 27887 36936 100327 64618 38440 41974 13188 134692 41467 25947 30272 25732	p02780	Catenin delta-2
ERBB2 seq: 1049 - 1072 + 2 Phospho (ST)	9E+07 4E+08 3E+08 2E+08 3E+08 6E+08 4E+08 2E+08 2E+08 2E+08 1E+08 7E+07 1E+08 5E+07 1E+08 4E+08 2E+08 3E+08 2E+08 2E+08 3E+08 1E+08 2E+08 2E+08 2E+08 68554 1E+07 2E+07 6E+07 3E+07 45838 31655 2E+07 8E+07 4E+07 44771 9E+07 3E+07 4E+07 5E+07 100327 64618 2E+07 7E+07 6E+07 134692 2E+08 25947 30272 1E+07	p03614	Receptor tyrosine-protein kinase erbB-2
DTL seq: 484 - 498 + Phospho (ST)	3E+07 3E+07 2E+07 3E+07 3E+07 1E+08 5E+07 1E+07 2E+07 1E+07 7E+07 3E+07 3E+07 9E+06 7E+06 8E+07 5E+07 5E+07 3E+07 2E+07 9E+07 4E+07 4E+07 19024 3E+07 1E+08 6E+07 5E+07 3E+07 5E+07 3E+07 3E+07 6E+06 6E+07 5E+07 5E+07 5E+07 4E+07 3E+07 4E+07 1E+08 7E+07 7E+07 4E+07 7E+07 1E+08 4E+07 5E+07 4E+07 4E+07	p15140	Denticleless protein homolog
RASAL2 seq: 89 - 107 + Phospho (ST)	7E+07 5E+07 5E+07 4E+07 5E+07 8E+07 9E+07 5E+07 4E+07 5E+07 4E+07 5E+07 3E+07 2E+07 4E+07 40867 7E+07 1E+08 6E+07 6E+07 2E+07 6E+07 21442 7E+07 4E+07 1E+08 2E+08 3E+08 4E+08 2E+08 2E+08 1E+08 2E+08 3E+08 3E+08 3E+08 2E+08 3E+08 2E+08 3E+08 3E+08 3E+08 3E+08 3E+08 3E+08 2E+08 3E+08 3E+08 3E+08 3E+08	p05607	Ras GTPase-activating protein nGAP
DDI2 seq: 109 - 139 + Phospho (ST)	7E+08 1E+09 1E+09 1E+09 1E+09 3E+09 1E+09 1E+09 1E+09 8E+08 5E+09 3E+09 2E+09 2E+09 1E+09 2E+09 7E+08 1E+09 1E+09 7E+08 2E+09 9E+08 8E+08 9E+08 9E+08 1E+09 3E+08 3E+08 6E+08 3E+08 5E+08 3E+08 2E+08 3E+08 4E+08 7E+08 4E+08 4E+08 4E+08 5E+08 6E+08 4E+08 3E+08 4E+08 4E+08 6E+08 2E+08 4E+08 3E+08 5E+08	p07962	Protein DDI1 homolog 2
HIRIP3 seq: 527 - 546 + Phospho (ST)	2E+09 9E+08 2E+09 2E+09 2E+09 6E+07 3E+09 1E+09 2E+09 2E+09 9E+09 4E+09 2E+09 3E+09 2E+09 8E+08 5E+08 2E+09 2E+09 2E+09 1E+09 6E+08 8E+08 2E+09 2E+09 1E+10 1E+09 2E+09 5E+09 4E+09 3E+09 2E+09 1E+09 4E+09 5E+09 8E+09 3E+09 2E+09 3E+09 5E+09 8E+09 3E+09 3E+09 3E+09 4E+09 7E+09 1E+09 2E+09 4E+09 4E+09	p02623	HIRA-interacting protein 3
CAST seq: 133 - 150 + Phospho (ST)	2E+08 2E+08 3E+08 2E+08 2E+08 6E+08 5E+08 2E+08 2E+08 2E+08 4E+08 2E+08 1E+08 1E+08 1E+08 3E+08 2E+08 2E+08 2E+08 2E+08 4E+08 3E+08 2E+08 2E+08 2E+08 3E+09 1E+09 1E+09 1E+09 7E+08 2E+09 1E+09 1E+09 1E+09 1E+09 2E+09 1E+09 1E+09 8E+08 1E+09 2E+09 1E+09 1E+09 1E+09 9E+08 2E+09 1E+09 1E+09 1E+09 8E+08	p04944	Calpastatin
CAST pS243	1E+09 1E+09 2E+09 2E+09 2E+09 4E+09 2E+09 1E+09 1E+09 1E+09 5E+09 3E+09 2E+09 3E+09 2E+09 2E+09 1E+09 2E+09 2E+09 1E+09 2E+09 1E+09 1E+09 2E+09 1E+09 3E+10 1E+10 1E+10 2E+10 1E+10 1E+10 7E+09 7E+09 1E+10 2E+10 2E+10 1E+10 1E+10 1E+10 1E+10 2E+10 1E+10 1E+10 1E+10 1E+10 2E+10 7E+09 1E+10 2E+10 1E+10	p13294	Calpastatin
KRT8 seq: 402 - 414 no mod	7E+08 9E+08 1E+09 6E+08 5E+08 1E+09 6E+08 6E+08 8E+08 4E+08 6E+08 8E+08 2E+09 5E+08 5E+08 3E+08 5E+08 6E+08 7E+08 3E+08 5E+08 5E+08 5E+08 6E+08 4E+08 2E+08 9E+08 6E+08 7E+08 5E+08 4E+09 6E+08 1E+09 6E+08 6E+08 4E+09 7E+08 4E+08 7E+08 8E+08 2E+09 6E+08 8E+08 5E+08 6E+08 5E+09 4E+08 7E+08 7E+08 6E+08	p11928	Keratin, type II cytoskeletal 8
UBAP2L seq: 601 - 618 + 2 Phospho (ST)	2E+09 2E+09 3E+09 3E+09 3E+09 4E+09 4E+09 3E+09 3E+09 2E+09 4E+09 2E+09 2E+09 2E+09 2E+09 4E+09 3E+09 4E+09 3E+09 3E+09 4E+09 4E+09 2E+09 3E+09 2E+09 1E+10 5E+09 4E+09 6E+09 6E+09 9E+09 6E+09 4E+09 5E+09 7E+09 6E+09 6E+09 4E+09 5E+09 4E+09 9E+09 6E+09 5E+09 5E+09 6E+09 6E+09 5E+09 4E+09 5E+09 6E+09	p06403	Ubiquitin-associated protein 2-like
PTPN12 pS449	6E+08 4E+08 8E+08 3E+08 3E+08 1E+09 8E+08 6E+08 3E+08 2E+08 1E+09 8E+08 8E+08 2E+08 3E+08 4E+08 4E+08 1E+09 3E+08 3E+08 5E+08 2E+08 5E+08 2E+08 3E+08 4E+07 3E+07 34144 5E+07 2E+07 5E+07 4E+07 4E+07 24890 3E+07 44771 3E+07 4E+07 27887 36936 2E+07 3E+07 3E+07 3E+07 3E+07 134692 8E+07 4E+07 2E+07 3E+07	p05931	Tyrosine-protein phosphatase non-receptor type 12
BAZ2A seq: 1173 - 1187 + Phospho (ST)	2E+07 5E+07 6E+07 4E+07 5E+07 2E+08 7E+07 7E+07 6E+07 5E+07 3E+07 4E+07 2E+07 3E+07 3E+07 9E+07 6E+07 5E+07 7E+07 7E+07 1E+08 2E+07 6E+07 5E+07 5E+07 9E+07 44396 3E+07 4E+07 4E+07 3E+07 31655 3E+07 9E+06 4E+07 5E+07 1E+07 2E+07 1E+07 2E+07 6E+07 3E+07 4E+07 2E+07 5E+07 134692 1E+07 3E+07 3E+07 4E+07	p10184	Bromodomain adjacent to zinc finger domain protein 2A
DDX20 seq: 548 - 565 + Phospho (ST)	3E+07 2E+07 4E+07 8E+06 7E+06 4E+07 3E+07 2E+07 1E+07 4E+06 1E+08 8E+07 7E+07 7E+07 2E+07 1E+07 1E+07 4E+07 9E+06 28832 1E+07 1E+07 2E+07 2E+07 1E+07 1E+07 3E+07 3E+07 2E+07 3E+06 2E+07 2E+07 2E+07 3E+07 2E+07 3E+07 5E+07 2E+07 9E+06 1E+07 4E+07 2E+07 2E+07 2E+07 2E+07 134692 3E+07 3E+07 1E+07 1E+07	p09046	Probable ATP-dependent RNA helicase DDX20
NACAP1 seq: 127 - 141 no mod	4E+07 4E+07 7E+07 2E+07 6E+07 2E+08 2E+07 5E+07 3E+07 1E+07 3E+08 2E+07 7E+07 5E+07 3E+07 5E+07 2E+07 3E+07 4E+07 1E+07 39759 2E+07 4E+07 2E+07 1E+07 2E+08 4E+07 2E+07 3E+07 6E+07 3E+08 3E+07 8E+07 4E+07 17227 3E+08 7E+07 4E+07 5E+07 2E+07 1E+08 4E+07 3E+07 4E+07 3E+07 3E+08 3E+07 5E+07 4E+07 3E+07	p14380	Putative nascent polypeptide-associated complex subunit alpha-like protein
MDC1 pS1086	38324 9E+07 [illegible]	p08772	Mediator of DNA damage checkpoint protein 1
NQO1 seq: 263 - 271 no mod	2E+08 2E+08 [illegible]	p05495	NAD(P)H dehydrogenase [quinone] 1
TLE3 pT328	1E+09 1E+09 [illegible]	p09718	Transducin-like enhancer protein 3
SMAP2 pS240	8E+08 8E+08 [illegible]	p12372	Stromal membrane-associated protein 2
MDC1 pS780	2E+08 5E+08 [illegible]	p17168	Mediator of DNA damage checkpoint protein 1
CTNNB1 seq: 550 - 565 + Phospho (ST)	2E+09 2E+09 [illegible]	p06489	Catenin beta-1
SP100 pS166	38324 7E+06 [illegible]	p05723	Nuclear autoantigen Sp-100
AHNAK seq: 5447 - 5460 no mod	2E+08 2E+08 [illegible]	p13758	Neuroblast differentiation-associated protein AHNAK
STIM1 seq: 515 - 530 + Phospho (ST)	7E+07 2E+08 [illegible]	p18520	Stromal interaction molecule 1
C2orf44 pS468	38324 37149 [illegible]	p11603	WD repeat and coiled-coil-containing protein C2orf44
EML4 seq: 657 - 665 no mod	3E+06 5E+07 [illegible]	p00503	Echinoderm microtubule-associated protein-like 4
CRTC3 seq: 366 - 384 + Phospho (ST)	6E+09 6E+09 [illegible]	p11861	CREB-regulated transcription coactivator 3
PPP1R13L seq: 352 - 362 + Oxidation (M); Phospho (ST)	9E+08 8E+08 [illegible]	p13879	RelA-associated inhibitor
CAMSAP3 pS1074	5E+08 9E+08 [illegible]	p19897	Calmodulin-regulated spectrin-associated protein 3
TJP2 pT925	2E+09 2E+09 [illegible]	p11556	Tight junction protein ZO-2
PHLDB1 pS470	38324 1E+07 [illegible]	p07628	Pleckstrin homology-like domain family B member 1
HMGA1 pS36 pT53	8E+08 8E+08 [illegible]	p12663	High mobility group protein HMG-I/HMG-Y
UBA1 seq: 831 - 843 + Phospho (ST)	1E+08 37149 [illegible]	p19361	Ubiquitin-like modifier-activating enzyme 1
NUP133 seq: 35 - 54 + 2 Phospho (ST)	1E+08 1E+08 [illegible]	p13184	Nuclear pore complex protein Nup133
MDC1 seq: 960 - 1000 + Phospho (ST)	38324 37149 [illegible]	p15413	Mediator of DNA damage checkpoint protein 1
OSTF1 pS213	38324 37149 [illegible]	p02545	Osteoclast-stimulating factor 1
CAST pS243	1E+08 2E+08 [illegible]	p13295	Calpastatin
MED26 pS535	1E+07 1E+08 [illegible]	p08371	Mediator of RNA polymerase II transcription subunit 26
MAP7 pS219	2E+07 9E+07 [illegible]	p07155	Ensconsin
CPNE6 seq: 333 - 342 + Gln->pyro-Glu (N-term Q)	3E+08 4E+08 [illegible]	p08122	Copine-6
RNF39 seq: 240 - 248 + Phospho (ST)	2E+08 5E+08 [illegible]	p07169	RING finger protein 39
MAST3 seq: 745 - 753 + Phospho (ST)	2E+08 5E+08 [illegible]	p07152	Microtubule-associated serine/threonine-protein kinase 3
DHX16 pS160	6E+07 1E+08 [illegible]	p07999	Putative pre-mRNA-splicing factor ATP-dependent RNA helicase DHX16
RPS3A seq: 86 - 94 no mod	2E+08 2E+08 [illegible]	p11548	40S ribosomal protein S3a
PRDX4 seq: 129 - 136 no mod	2E+09 5E+09 [illegible]	p15753	Peroxiredoxin-4
TMPRSS13 seq: 539 - 546 no mod	2E+08 5E+08 [illegible]	p09505	Transmembrane protease serine 13
AHNAK seq: 2707 - 2717 + Phospho (ST)	8E+07 2E+08 [illegible]	p13707	Neuroblast differentiation-associated protein AHNAK
KDM3A seq: 951 - 960 no mod	9E+07 3E+08 [illegible]	p11462	Lysine-specific demethylase 3A
VCP seq: 678 - 693 no mod	2E+09 1E+09 [illegible]	p09853	Transitional endoplasmic reticulum ATPase
RBMXL1 pS208	1E+10 1E+10 [illegible]	p18074	RNA binding motif protein, X-linked-like-1
SEPT9 pS22	3E+08 3E+08 [illegible]	p11814	Septin-9
AHNAK pS4908	1E+08 2E+08 [illegible]	p09948	Neuroblast differentiation-associated protein AHNAK
PSME1 seq: 199 - 210 + Gln->pyro-Glu (N-term Q)	3E+07 6E+07 [illegible]	p08260	Proteasome activator complex subunit 1
ZC3H14 pS343	4E+08 5E+08 [illegible]	p06858	Zinc finger CCCH domain-containing protein 14
EPS8 pS815	2E+08 2E+08 [illegible]	p13760	Epidermal growth factor receptor kinase substrate 8
CCDC43 pT139	5E+08 6E+08 [illegible]	p21098	Coiled-coil domain-containing protein 43
CEP131 seq: 87 - 109 + Oxidation (M); Phospho (ST)	4E+06 3E+07 [illegible]	p05667	Centrosomal protein of 131 kDa
NOLC1 pT607 pT610	6E+07 4E+07 [illegible]	p10923	Nucleolar and coiled-body phosphoprotein 1
ITSN2 pS210	5E+08 6E+08 [illegible]	p19766	Intersectin-2
DHX9 seq: 77 - 120 + Oxidation (M); Phospho (ST)	2E+09 2E+09 [illegible]	p06038	ATP-dependent RNA helicase A
SRRM1 pS616	2E+10 1E+10 [illegible]	p03017	Serine/arginine repetitive matrix protein 1
PKP4 seq: 325 - 340 + Phospho (ST)	1E+09 2E+09 [illegible]	p01753	Plakophilin-4
NCOR2 seq: 1023 - 1049 + Phospho (ST)	7E+08 1E+09 [illegible]	p04041	Nuclear receptor corepressor 2
SGK223 seq: 790 - 819 + Phospho (ST)	1E+08 2E+08 [illegible]	p11911	Tyrosine-protein kinase SgK223
KHSRP pS181	2E+09 2E+09 [illegible]	p00948	Far upstream element-binding protein 2
RSL1D1 seq: 462 - 471 + 2 Phospho (ST)	3E+08 2E+08 [illegible]	p16772	Ribosomal L1 domain-containing protein 1
VDAC2 seq: 178 - 185 no mod	1E+07 1E+08 [illegible]	p09344	Voltage-dependent anion-selective channel protein 2
CTTNBP2NL seq: 555 - 573 + 2 Phospho (ST)	1E+08 1E+08 [illegible]	p00779	CTTNBP2 N-terminal-like protein
NSUN2 seq: 451 - 470 + Phospho (ST)	8E+07 4E+07 [illegible]	p17102	tRNA (cytosine(34)-C(5))-methyltransferase
ZC3H18 seq: 790 - 814 + Phospho (ST)	4E+09 4E+09 [illegible]	p03746	Zinc finger CCCH domain-containing protein 18
NFATC2IP seq: 231 - 263 + Phospho (ST)	2E+08 2E+08 [illegible]	p06147	NFATC2-interacting protein
RRM2 pS20	3E+09 3E+09 [illegible]	p01007	Ribonucleoside-diphosphate reductase subunit M2
YWHAZ seq: 28 - 41 no mod	2E+08 2E+08 [illegible]	p03227	14-3-3 protein zeta/delta
AHNAK pT4564	2E+08 4E+08 [illegible]	p01474	Neuroblast differentiation-associated protein AHNAK
NCOR2 seq: 140 - 166 + 2 Phospho (ST)	1E+09 1E+09 [illegible]	p04954	Nuclear receptor corepressor 2
CHTF18 pS871	8E+08 7E+08 [illegible]	p01607	Chromosome transmission fidelity protein 18 homolog
SRRM2 pS780	6E+08 5E+08 [illegible]	p06663	Serine/arginine repetitive matrix protein 2
CRTC2 seq: 454 - 476 + Phospho (ST)	1E+08 2E+08 [illegible]	p08670	CREB-regulated transcription coactivator 2
ANLN seq: 180 - 198 + 2 Phospho (ST)	2E+08 1E+08 [illegible]	p21029	Actin-binding protein anillin
TPI1 seq: 232 - 243 no mod	1E+09 2E+09 [illegible]	p04740	Triosephosphate isomerase
ZNF318 pS2101	3E+08 1E+09 [illegible]	p13849	Zinc finger protein 318
AGAP1 seq: 516 - 526 + Phospho (ST)	1E+09 7E+08 [illegible]	p12057	Arf-GAP with GTPase, ANK repeat and PH domain-containing protein 1
CD2AP pS224	38324 2E+07 [illegible]	p15898	CD2-associated protein
NUMA1 pT2106	3E+08 4E+08 [illegible]	p14461	Nuclear mitotic apparatus protein 1
GAPDH seq: 146 - 162 no mod	5E+08 9E+08 [illegible]	p14135	Glyceraldehyde-3-phosphate dehydrogenase
PSMD1 pM351	4E+08 6E+08 [illegible]	p09321	26S proteasome non-ATPase regulatory subunit 1
CSTF2 seq: 500 - 537 + Oxidation (M); Phospho (ST)	5E+08 3E+08 [illegible]	p07710	Cleavage stimulation factor subunit 2
ABL1 pS718	2E+08 2E+08 [illegible]	p06138	Tyrosine-protein kinase ABL1
YAP1 seq: 125 - 161 + 3 Phospho (ST)	38324 37149 [illegible]	p20506	Transcriptional coactivator YAP1
ENO1 seq: 163 - 179 no mod	1E+08 2E+08 [illegible]	p12227	Alpha-enolase
RPS14 seq: 129 - 141 no mod	38324 6E+07 [illegible]	p14369	40S ribosomal protein S14
ZNF276 seq: 586 - 614 + Phospho (ST)	38324 1E+08 [illegible]	p20207	Zinc finger protein 276
NPM1 pS254	38324 37149 [illegible]	p09940	Nucleophosmin
RPL28 seq: 23 - 33 no mod	2E+08 3E+08 [illegible]	p07755	60S ribosomal protein L28
MARCKS pS167	3E+07 5E+07 [illegible]	p05892	Myristoylated alanine-rich C-kinase substrate
SIPA1L3 seq: 389 - 408 + Phospho (ST)	2E+08 1E+08 [illegible]	p20518	Signal-induced proliferation-associated 1-like protein 3
USP9X pS1600	3E+08 2E+08 [illegible]	p09609	Probable ubiquitin carboxyl-terminal hydrolase FAF-X
CD2AP pS224	6E+06 3E+07 [illegible]	p15897	CD2-associated protein
CDK12 pT893	4E+08 3E+08 [illegible]	p10297	Cyclin-dependent kinase 12
SRRM2 pS2692 pS2694	4E+07 2E+07 [illegible]	p04961	Serine/arginine repetitive matrix protein 2
ANXA6 seq: 588 - 598 no mod	4E+07 1E+08 [illegible]	p18986	Annexin A6
CDC42EP4 pS118	3E+09 3E+09 [illegible]	p09764	Cdc42 effector protein 4
LMNB1 seq: 43 - 49 no mod	6E+07 1E+08 [illegible]	p12137	Lamin-B1
TRIP10 pS296	1E+09 1E+09 [illegible]	p19924	Cdc42-interacting protein 4
VASP pS239	3E+08 4E+08 [illegible]	p12398	Vasodilator-stimulated phosphoprotein
PTPN12 seq: 565 - 592 + 2 Phospho (ST)	3E+08 2E+08 [illegible]	p01903	Tyrosine-protein phosphatase non-receptor type 12
MARCKS pS170	2E+09 4E+09 [illegible]	p10796	Myristoylated alanine-rich C-kinase substrate
SRRM1 seq: 603 - 612 + Phospho (ST); Phospho (Y)	7E+09 5E+09 [illegible]	p06388	Serine/arginine repetitive matrix protein 1
CDK16 pS12	2E+08 3E+08 [illegible]	p08291	Cyclin-dependent kinase 16
CASS4 seq: 147 - 152 + Phospho (ST)	7E+08 5E+08 [illegible]	p02554	Cas scaffolding protein family member 4
STRN seq: 363 - 384 + Phospho (ST)	4E+08 2E+08 [illegible]	p18121	Striatin
CCDC120 pS302	6E+07 1E+08 [illegible]	p03307	Coiled-coil domain-containing protein 120
ADD2 pS726	7E+08 5E+08 [illegible]	p02336	Beta-adducin
ZFPM1 pS909	3E+09 2E+09 [illegible]	p02434	Zinc finger protein ZFPM1
FAM21A seq: 613 - 640 + Phospho (ST)	1E+09 2E+09 [illegible]	p19654	WASH complex subunit FAM21A
TMPO seq: 18 - 33 no mod	1E+08 1E+08 [illegible]	p06013	Lamina-associated polypeptide 2, isoform alpha
CRTC3 pS329	2E+09 1E+09 [illegible]	p04821	CREB-regulated transcription coactivator 3
IQSEC1 seq: 84 - 92 + Phospho (ST)	5E+08 6E+08 [illegible]	p10995	IQ motif and SEC7 domain-containing protein 1
PCBP2 seq: 71 - 101 no mod	38324 37149 [illegible]	p20783	Poly(rC)-binding protein 2
PTRF seq: 182 - 217 + Phospho (ST)	38324 37149 [illegible]	p17764	Polymerase I and transcript release factor
NEK3 seq: 444 - 495 + Phospho (ST)	38324 37149 [illegible]	p15489	Serine/threonine-protein kinase Nek3
UNK seq: 158 - 164 no mod	38324 37149 [illegible]	p04295	RING finger protein unkempt homolog
FLNA seq: 1319 - 1357 + Phospho (ST)	38324 37149 [illegible]	p01580	Filamin-A
ZC3H4 seq: 157 - 179 + Phospho (Y)	2E+09 2E+09 [illegible]	p16890	Zinc finger CCCH domain-containing protein 4
CLIP1 seq: 145 - 177 + Phospho (ST)	1E+08 1E+08 [illegible]	p19320	CAP-Gly domain-containing linker protein 1
AGFG1 seq: 162 - 186 + 2 Phospho (ST)	1E+10 1E+10 [illegible]	p05161	Arf-GAP domain and FG repeat-containing protein 1
SERBP1 seq: 53 - 68 no mod	3E+08 2E+08 [illegible]	p06375	Plasminogen activator inhibitor 1 RNA-binding protein
SQSTM1 pS233	2E+07 37149 [illegible]	p20690	Sequestosome-1
APPL1 seq: 390 - 407 + Phospho (ST)	8E+08 9E+08 [illegible]	p01048	DCC-interacting protein 13-alpha
FASN pT2204	7E+08 5E+08 [illegible]	p20926	Fatty acid synthase
ERBB2IP seq: 913 - 941 + 2 Phospho (ST)	7E+07 2E+08 [illegible]	p05385	Protein LAP2
HMGA1 seq: 31 - 55 + 2 Phospho (ST)	1E+09 1E+09 [illegible]	p12664	High mobility group protein HMG-I/HMG-Y
SCRIB pS1306 pS1309	1E+09 1E+09 [illegible]	p10274	Protein scribble homolog
MCM3AP seq: 556 - 566 + Phospho (ST)	38324 6E+07 [illegible]	p03763	Germinal-center associated nuclear protein
PPP1R13L pS74	3E+08 3E+08 [illegible]	p00065	RelA-associated inhibitor
EIF3I seq: 214 - 224 no mod	7E+07 8E+07 [illegible]	p11905	Eukaryotic translation initiation factor 3 subunit I
SNRPA1 seq: 178 - 192 + Phospho (ST)	8E+07 9E+07 [illegible]	p05339	U2 small nuclear ribonucleoprotein A'
FZD6 pS620	1E+08 2E+08 [illegible]	p10907	Frizzled-6
NCOA1 seq: 693 - 712 + Phospho (ST)	9E+07 1E+08 [illegible]	p11313	Nuclear receptor coactivator 1
BCL11B pT417	38324 6E+08 [illegible]	p04673	B-cell lymphoma/leukemia 11B
LASP1 pS146	1E+10 9E+09 [illegible]	p10093	LIM and SH3 domain protein 1
WDR59 seq: 810 - 827 + Phospho (ST)	1E+08 8E+07 [illegible]	p17987	WD repeat-containing protein 59
LAMTOR2 seq: 59 - 70 no mod	3E+08 1E+08 [illegible]	p09603	Ragulator complex protein LAMTOR2
AHCY seq: 175 - 186 no mod	3E+08 6E+08 [illegible]	p01033	Adenosylhomocysteinase
DBNL seq: 149 - 164 no mod	8E+06 2E+08 [illegible]	p16522	Drebrin-like protein
SLC4A1AP seq: 130 - 150 + Phospho (ST)	3E+08 3E+08 [illegible]	p05086	Kanadaptin
SRRM1 pS713	8E+08 7E+08 [illegible]	p07861	Serine/arginine repetitive matrix protein 1
SCRIB pS1561 pS1566	1E+09 1E+09 [illegible]	p12283	Protein scribble homolog
LAMTOR1 pS27	3E+08 3E+08 [illegible]	p12959	Ragulator complex protein LAMTOR1
PLIN3 seq: 85 - 98 no mod	6E+07 6E+07 [illegible]	p11945	Perilipin-3
CFL1 seq: 74 - 81 no mod	3E+08 4E+08 [illegible]	p10022	Cofilin-1
RIC8A pS436 pT441	38324 37149 [illegible]	p15690	Synembryn-A
MEAF6 seq: 115 - 154 + 2 Phospho (ST)	9E+08 5E+08 [illegible]	p17310	Chromatin modification-related protein MEAF6
MAPT seq: 527 - 541 + 2 Phospho (ST)	1E+09 8E+08 [illegible]	p03968	Microtubule-associated protein tau
RPS20 seq: 5 - 19 no mod	2E+07 3E+07 [illegible]	p18179	40S ribosomal protein S20
GAPVD1 pS902	3E+08 3E+08 [illegible]	p03937	GTPase-activating protein and VPS9 domain-containing protein 1
GTSE1 seq: 164 - 179 + Phospho (ST)	1E+08 1E+08 [illegible]	p11466	G2 and S phase-expressed protein 1
OSBPL10 pS201	2E+08 3E+08 [illegible]	p04951	Oxysterol-binding protein-related protein 10
RANBP2 pS955	3E+08 3E+08 [illegible]	p16790	E3 SUMO-protein ligase RanBP2
KIAA1143 seq: 109 - 118 + Phospho (ST)	3E+08 4E+08 [illegible]	p00111	Uncharacterized protein KIAA1143
ARFGAP2 pS240	2E+08 1E+08 [illegible]	p00767	ADP-ribosylation factor GTPase-activating protein 2
AKIRIN2 pS57	3E+07 9E+07 [illegible]	p19076	Akirin-2
ARFGAP1 seq: 360 - 374 + Phospho (ST)	4E+08 4E+08 [illegible]	p03930	ADP-ribosylation factor GTPase-activating protein 1
LMNA seq: 124 - 133 no mod	7E+07 7E+07 [illegible]	p17770	Prelamin-A/C
TAB2 seq: 522 - 541 + Phospho (ST)	2E+08 3E+08 [illegible]	p12916	TGF-beta-activated kinase 1 and MAP3K7-binding protein 2
ARAF seq: 256 - 268 + Phospho (ST)	9E+08 6E+08 [illegible]	p15207	Serine/threonine-protein kinase A-Raf
CSNK1E seq: 356 - 365 + Phospho (ST)	8E+08 9E+08 [illegible]	p13801	Casein kinase I isoform epsilon
PCYT1A seq: 310 - 317 + Oxidation (M); Phospho (ST)	4E+09 4E+09 [illegible]	p10009	Choline-phosphate cytidylyltransferase A
PCYT1B pM310 pS315	4E+09 4E+09 [illegible]	p10006	Choline-phosphate cytidylyltransferase B
NCL pT121	7E+07 5E+07 [illegible]	p20122	Nucleolin
CCNK pS340	2E+08 3E+08 [illegible]	p19143	Cyclin-K
PSMG2 seq: 130 - 135 no mod	6E+07 7E+07 [illegible]	p09703	Proteasome assembly chaperone 2
LARP7 seq: 254 - 274 + Phospho (ST)	2E+08 1E+08 [illegible]	p04085	La-related protein 7
PDLIM2 seq: 120 - 131 + 2 Phospho (ST)	1E+08 4E+07 [illegible]	p03565	PDZ and LIM domain protein 2
DDX3X seq: 82 - 93 + Phospho (ST)	1E+08 1E+08 [illegible]	p03910	ATP-dependent RNA helicase DDX3X
NUMA1 seq: 1822 - 1839 + Phospho (ST)	1E+08 8E+07 [illegible]	p13013	Nuclear mitotic apparatus protein 1
RAD23B seq: 145 - 173 + Gln->pyro-Glu (N-term Q)	2E+08 8E+07 [illegible]	p08736	UV excision repair protein RAD23 homolog B
NUCKS1 pS58 pS61	5E+08 1E+08 [illegible]	p05737	Nuclear ubiquitous casein and cyclin-dependent kinase substrate 1
LMO7 seq: 749 - 758 + Phospho (ST)	4E+07 5E+07 [illegible]	p07202	LIM domain only protein 7
PPIF seq: 197 - 207 no mod	1E+07 5E+07 [illegible]	p13057	Peptidyl-prolyl cis-trans isomerase F, mitochondrial
PGM3 pM60 pS64	3E+09 2E+09 [illegible]	p03513	Phosphoacetylglucosamine mutase
BCAR3 seq: 315 - 329 + Phospho (ST)	4E+08 3E+08 [illegible]	p05657	Breast cancer anti-estrogen resistance protein 3
USP47 pS1353	7E+07 5E+07 [illegible]	p00660	Ubiquitin carboxyl-terminal hydrolase 47
NEDD4L seq: 472 - 493 + 2 Phospho (ST)	2E+08 1E+08 [illegible]	p18169	E3 ubiquitin-protein ligase NEDD4-like
EIF4G1 seq: 1072 - 1090 + Phospho (ST)	2E+09 2E+09 [illegible]	p13602	Eukaryotic translation initiation factor 4 gamma 1
HIST3H3 seq: 74 - 80 no mod	1E+08 4E+08 [illegible]	p17634	Histone H3.1t
SHROOM3 pS1221	2E+08 2E+08 [illegible]	p04882	Protein Shroom3
ARHGAP29 seq: 552 - 565 + Phospho (ST)	5E+07 3E+07 [illegible]	p05276	Rho GTPase-activating protein 29
CFL1 seq: 82 - 92 no mod	1E+09 1E+09 [illegible]	p00447	Cofilin-1
LRRFIP1 seq: 99 - 117 + Oxidation (M); Phospho (ST)	8E+08 7E+08 [illegible]	p09367	Leucine-rich repeat flightless-interacting protein 1
RABEP1 pT408 pS410	2E+08 2E+08 [illegible]	p19750	Rab GTPase-binding effector protein 1
ERP29 seq: 244 - 253 no mod	1E+07 9E+07 [illegible]	p05137	Endoplasmic reticulum resident protein 29
DNAJB1 seq: 223 - 240 no mod	2E+06 2E+07 [illegible]	p17766	DnaJ homolog subfamily B member 1
MDH2 seq: 270 - 279 no mod	1E+09 1E+09 [illegible]	p17657	Malate dehydrogenase, mitochondrial
PLEC seq: 2361 - 2371 no mod	2E+08 1E+08 [illegible]	p04150	Plectin
DYNLRB2 seq: 59 - 70 no mod	7E+07 9E+07 [illegible]	p18762	Dynein light chain roadblock-type 2
BCAR3 seq: 335 - 347 + Phospho (ST)	3E+08 2E+08 [illegible]	p20524	Breast cancer anti-estrogen resistance protein 3
KRT6C seq: 447 - 455 no mod	9E+08 1E+09 [illegible]	p16897	Keratin, type II cytoskeletal 6C
ITSN2 seq: 953 - 961 no mod	9E+07 7E+07 [illegible]	p14146	Intersectin-2
HSPA5 pM196	8E+07 2E+08 [illegible]	p18980	78 kDa glucose-regulated protein
CFL2 seq: 14 - 19 no mod	2E+08 4E+08 [illegible]	p01542	Cofilin-2
NFIC seq: 327 - 345 + 2 Phospho (ST)	2E+08 9E+07 [illegible]	p12506	Nuclear factor 1 C-type
TPX2 pS486	4E+08 3E+08 [illegible]	p04732	Targeting protein for Xklp2
KIAA1671 pS767	4E+08 3E+08 [illegible]	p03186	Uncharacterized protein KIAA1671
HSPA5 seq: 465 - 474 no mod	6E+08 1E+09 [illegible]	p00541	78 kDa glucose-regulated protein
DENND4C seq: 1087 - 1095 + Phospho (ST)	3E+08 4E+08 [illegible]	p03451	DENN domain-containing protein 4C
ACTA1 seq: 71 - 86 no mod	9E+08 2E+09 [illegible]	p00184	Actin, alpha skeletal muscle

YWHAE seq: 143 - 153 no mod	[illegible]	p18024	14-3-3 protein epsilon
GAPDH seq: 261 - 271 no mod	[illegible]	p00612	Glyceraldehyde-3-phosphate dehydrogenase
DAXX seq: 686 - 705 + 2 Phospho (ST)	[illegible]	p01660	Death domain-associated protein 6
FKBP1A seq: 2 - 14 no mod	[illegible]	p14998	Peptidyl-prolyl cis-trans isomerase FKBP1A
ENO1 seq: 336 - 343 no mod	[illegible]	p06145	Alpha-enolase
NCL seq: 325 - 333 no mod	[illegible]	p02812	Nucleolin
PPP1R14C pS25	[illegible]	p01559	Protein phosphatase 1 regulatory subunit 14C
AATK pS111	[illegible]	p03293	Serine/threonine-protein kinase LMTK1
ACTB pM305	[illegible]	p18504	Actin, cytoplasmic 1
TP53BP1 pS1426 pS1430	[illegible]	p17078	Tumor suppressor p53-binding protein 1
VCPIP1 seq: 996 - 1005 + Phospho (ST)	[illegible]	p17112	Deubiquitinating protein VCIP135
PPFIA1 pS666	[illegible]	p01449	Liprin-alpha-1
SMARCC1 pS328 pS330	[illegible]	p07227	SWI/SNF complex subunit SMARCC1
HNRNPC seq: 289 - 306 + Phospho (ST)	[illegible]	p17988	Heterogeneous nuclear ribonucleoproteins C1/C2
IMPDH2 seq: 292 - 311 no mod	[illegible]	p18655	Inosine-5'-monophosphate dehydrogenase 2
HSPA8 pM61	[illegible]	p09205	Heat shock cognate 71 kDa protein
RPL31 seq: 15 - 23 no mod	[illegible]	p06324	60S ribosomal protein L31
PSMA5 seq: 11 - 20 no mod	[illegible]	p15012	Proteasome subunit alpha type-5
TPI1 seq: 138 - 150 no mod	[illegible]	p14569	Triosephosphate isomerase
YAP1 pS131 pS138 pT141	[illegible]	p20505	Transcriptional coactivator YAP1
TXN seq: 86 - 94 no mod	[illegible]	p01498	Thioredoxin
PRDX1 seq: 159 - 168 no mod	[illegible]	p10373	Peroxiredoxin-1
KRT19 seq: 318 - 330 no mod	[illegible]	p21102	Keratin, type I cytoskeletal 19
MAST4 seq: 1398 - 1411 + Phospho (ST)	[illegible]	p04394	Microtubule-associated serine/threonine-protein kinase 4
TSC2 seq: 1385 - 1415 + 2 Phospho (ST)	[illegible]	p03632	Tuberin
SRSF11 seq: 440 - 451 + Phospho (ST)	[illegible]	p17835	Serine/arginine-rich splicing factor 11
ARAF pS157	[illegible]	p08063	Serine/threonine-protein kinase A-Raf
SYNRG seq: 1096 - 1111 + Phospho (ST)	[illegible]	p18330	Synergin gamma
EIF4EBP1 seq: 64 - 99 + 2 Phospho (ST)	[illegible]	p09102	Eukaryotic translation initiation factor 4E-binding protein 1
PRKD1 seq: 217 - 234 + Phospho (ST)	[illegible]	p02122	Serine/threonine-protein kinase D1
PCBP2 seq: 246 - 276 + Phospho (ST)	[illegible]	p11641	Poly(rC)-binding protein 2
[illegible]seq: 244 - 268 + Gln->pyro-Glu (N-term Q); Phospho (ST)	[illegible]	p08016	Poly(rC)-binding protein 1
MLLT4 pS1799	[illegible]	p11858	Afadin
CEP170B seq: 1175 - 1192 + Phospho (ST)	[illegible]	p12635	Centrosomal protein of 170 kDa protein B
ARHGEF12 seq: 1286 - 1304 + Phospho (ST)	[illegible]	p03014	Rho guanine nucleotide exchange factor 12
SYNRG pT1100	[illegible]	p04744	Synergin gamma
FKBP15 seq: 342 - 361 + Phospho (ST)	[illegible]	p02651	FK506-binding protein 15
ITPKC seq: 74 - 110 + Phospho (ST)	[illegible]	p20637	Inositol-trisphosphate 3-kinase C
SDSL seq: 291 - 317 no mod	[illegible]	p11054	Serine dehydratase-like
SSFA2 seq: 108 - 141 + Phospho (ST)	[illegible]	p09611	Sperm-specific antigen 2
CXorf67 pS338	[illegible]	p03423	Uncharacterized protein CXorf67
TSC22D2 seq: 550 - 595 + Phospho (ST)	[illegible]	p03707	TSC22 domain family protein 2
MAPT seq: 414 - 440 + Phospho (ST)	[illegible]	p14684	Microtubule-associated protein tau
UCKL1 seq: 61 - 73 + Phospho (ST)	[illegible]	p02004	Uridine-cytidine kinase-like 1
PHACTR4 seq: 269 - 298 + Phospho (ST)	[illegible]	p09135	Phosphatase and actin regulator 4
METTL1 pS27	[illegible]	p20514	tRNA (guanine-N(7)-)-methyltransferase
CTTNBP2NL seq: 464 - 498 + Phospho (ST)	[illegible]	p14759	CTTNBP2 N-terminal-like protein
NPM1 pS125	[illegible]	p19050	Nucleophosmin
BAD pS99	[illegible]	p06291	Bcl2-associated agonist of cell death
BAD pS99	[illegible]	p06290	Bcl2-associated agonist of cell death
LIMA1 seq: 666 - 703 + Phospho (ST)	[illegible]	p05426	LIM domain and actin-binding protein 1
SZT2 seq: 2948 - 2964 + Phospho (ST)	[illegible]	p20099	Protein SZT2
FGD1 seq: 46 - 75 + Phospho (ST)	[illegible]	p07346	FYVE, RhoGEF and PH domain-containing protein 1
TDRD3 seq: 487 - 503 + Phospho (ST)	[illegible]	p04810	Tudor domain-containing protein 3
LSM14B seq: 154 - 173 + Phospho (ST)	[illegible]	p04524	Protein LSM14 homolog B
NADK pS64	[illegible]	p05197	NAD kinase
RPS6KB1 seq: 444 - 459 + 2 Phospho (ST)	[illegible]	p02295	Ribosomal protein S6 kinase beta-1
MAP1B pS1396 pS1400	[illegible]	p01166	Microtubule-associated protein 1B
CASZ1 seq: 735 - 771 + Phospho (ST)	[illegible]	p09078	Zinc finger protein castor homolog 1
RAB3IP seq: 133 - 160 + Phospho (ST)	[illegible]	p17793	Rab-3A-interacting protein
MARCKS pS170	[illegible]	p10798	Myristoylated alanine-rich C-kinase substrate
CDKL5 seq: 341 - 355 + Phospho (ST)	[illegible]	p04789	Cyclin-dependent kinase-like 5
PDXDC1 seq: 732 - 769 + Phospho (ST)	[illegible]	p01598	Pyridoxal-dependent decarboxylase domain-containing protein 1
GRB10 seq: 426 - 443 + Phospho (ST)	[illegible]	p03281	Growth factor receptor-bound protein 10
LRRC59 seq: 293 - 307 no mod	[illegible]	p07299	Leucine-rich repeat-containing protein 59
FGD1 seq: 114 - 132 + Phospho (ST)	[illegible]	p05047	FYVE, RhoGEF and PH domain-containing protein 1
SEC14L1 pT234	[illegible]	p17718	SEC14-like protein 1
ADD1 pS586	[illegible]	p08461	Alpha-adducin
RUFY3 seq: 295 - 305 no mod	[illegible]	p10570	Protein RUFY3
MAP1B seq: 1496 - 1518 + Phospho (ST)	[illegible]	p12978	Microtubule-associated protein 1B
TNRC6A seq: 88 - 131 no mod	[illegible]	p19422	Trinucleotide repeat-containing gene 6A protein
RPN2 seq: 341 - 357 no mod	[illegible]	p16445	Dolichyl-diphosphooligosaccharide--protein glycosyltransferase subunit 2
FOXO3 pS253	[illegible]	p19188	Forkhead box protein O3
CRYZ seq: 62 - 88 no mod	[illegible]	p12775	Quinone oxidoreductase
PHB2 seq: 271 - 296 + Phospho (ST)	[illegible]	p13495	Prohibitin-2
ARFGAP3 seq: 365 - 380 + Phospho (ST)	[illegible]	p03709	ADP-ribosylation factor GTPase-activating protein 3
MLPH pT313 pS314	[illegible]	p09669	Melanophilin
FAM91A1 seq: 340 - 376 + Phospho (ST)	[illegible]	p04306	Protein FAM91A1
SH3BP2 pS427	[illegible]	p05854	SH3 domain-binding protein 2
BCAS3 pS869	[illegible]	p12294	Breast carcinoma-amplified sequence 3
TEX2 pS295	[illegible]	p07137	Testis-expressed sequence 2 protein
RAPH1 pS845	[illegible]	p08021	Ras-associated and pleckstrin homology domains-containing protein 1
ARFGAP2 seq: 360 - 372 + Phospho (ST)	[illegible]	p18431	ADP-ribosylation factor GTPase-activating protein 2
MAPT seq: 701 - 723 + 3 Phospho (ST)	[illegible]	p20358	Microtubule-associated protein tau
ZNF609 seq: 465 - 487 + Oxidation (M); Phospho (ST)	[illegible]	p02462	Zinc finger protein 609
PPP1R14C seq: 28 - 65 + Phospho (ST)	[illegible]	p16080	Protein phosphatase 1 regulatory subunit 14C
NPM1 seq: 46 - 80 + Oxidation (M); Phospho (ST)	[illegible]	p01907	Nucleophosmin
CHERP pS904	[illegible]	p03140	Calcium homeostasis endoplasmic reticulum protein
CDC42EP4 pS118	[illegible]	p09763	Cdc42 effector protein 4
GSK3B seq: 7 - 27 + Phospho (ST)	[illegible]	p02022	Glycogen synthase kinase-3 beta
TNRC6C pS714	[illegible]	p18219	Trinucleotide repeat-containing gene 6C protein
DYNC1I2 seq: 431 - 461 no mod	[illegible]	p19267	Cytoplasmic dynein 1 intermediate chain 2
PFKFB2 seq: 464 - 477 + Phospho (ST)	[illegible]	p07024	6-phosphofructo-2-kinase/fructose-2,6-bisphosphatase 2
PREX1 pS1001	[illegible]	p12731	Phosphatidylinositol 3,4,5-trisphosphate-dependent Rac exchanger 1 protein
LARP1 seq: 766 - 778 + 2 Phospho (ST)	[illegible]	p05097	La-related protein 1
RPLP0 seq: 302 - 317 + Oxidation (M); 2 Phospho (ST)	[illegible]	p17819	60S acidic ribosomal protein P0
MLLT4 pS1799	[illegible]	p11857	Afadin
TMEM238 seq: 157 - 176 + Phospho (ST)	[illegible]	p00899	Transmembrane protein 238
PRKCD seq: 642 - 669 + Phospho (ST)	[illegible]	p19704	Protein kinase C delta type
DHX9 seq: 121 - 141 no mod	[illegible]	p20819	ATP-dependent RNA helicase A
BAD pM117 pS118	[illegible]	p07053	Bcl2-associated agonist of cell death
RALGAPA2 pS486	[illegible]	p01866	Ral GTPase-activating protein subunit alpha-2
KIAA1211L pS609	[illegible]	p06061	Uncharacterized protein KIAA1211-like
FAM160A2 pS859	[illegible]	p06859	FTS and Hook-interacting protein
CRMP1 pS542	[illegible]	p09453	Dihydropyrimidinase-related protein 1
SLAIN2 seq: 312 - 341 + Phospho (ST)	[illegible]	p15098	SLAIN motif-containing protein 2
CD3EAP seq: 335 - 384 + Phospho (ST)	[illegible]	p15387	DNA-directed RNA polymerase I subunit RPA34
ELF2 seq: 358 - 386 + Phospho (ST)	[illegible]	p00594	ETS-related transcription factor Elf-2
PPP1R13B seq: 327 - 342 + Phospho (ST)	[illegible]	p01077	Apoptosis-stimulating of p53 protein 1
XPO6 pS3	[illegible]	p19636	Exportin-6
KCTD15 seq: 30 - 53 + 2 Phospho (ST)	[illegible]	p10738	BTB/POZ domain-containing protein KCTD15
PDLIM1 seq: 39 - 69 no mod	[illegible]	p21111	PDZ and LIM domain protein 1
FKBP15 pS346	[illegible]	p02652	FK506-binding protein 15
MAP2K2 pS23	[illegible]	p07217	Dual specificity mitogen-activated protein kinase kinase 2
SIPA1L3 seq: 366 - 388 + Phospho (ST)	[illegible]	p06989	Signal-induced proliferation-associated 1-like protein 3
COPRS seq: 70 - 105 + Phospho (ST)	[illegible]	p16162	Coordinator of PRMT5 and differentiation stimulator
CAMSAP1 seq: 860 - 888 + Phospho (ST)	[illegible]	p17266	Calmodulin-regulated spectrin-associated protein 1
ATP5J seq: 55 - 73 no mod	[illegible]	p07769	ATP synthase-coupling factor 6, mitochondrial
ADGRL1 seq: 1159 - 1178 + Phospho (ST)	[illegible]	p12636	Adhesion G protein-coupled receptor L1
FOXO3 pS253	[illegible]	p19187	Forkhead box protein O3
AKT1S1 pT246	[illegible]	p11152	Proline-rich AKT1 substrate 1
MKL1 seq: 444 - 460 + Phospho (ST)	[illegible]	p16730	MKL/myocardin-like protein 1
SLC25A46 seq: 32 - 53 + Phospho (ST)	[illegible]	p05809	Solute carrier family 25 member 46
AKT1 seq: 466 - 480 + 2 Phospho (ST)	[illegible]	p06931	RAC-alpha serine/threonine-protein kinase
TBC1D22B pS154	[illegible]	p08005	TBC1 domain family member 22B
ARHGEF12 seq: 1284 - 1304 + Phospho (ST)	[illegible]	p00128	Rho guanine nucleotide exchange factor 12
EAF2 pS22	[illegible]	p11795	ELL-associated factor 2
SYNRG seq: 1098 - 1111 + Phospho (ST)	[illegible]	p04743	Synergin gamma
PDS5B seq: 1174 - 1197 + Phospho (ST)	[illegible]	p12033	Sister chromatid cohesion protein PDS5 homolog B
FGD1 pS82	[illegible]	p01198	FYVE, RhoGEF and PH domain-containing protein 1
UBXN6 seq: 86 - 109 + Phospho (ST)	[illegible]	p17422	UBX domain-containing protein 6
ACBD3 seq: 279 - 306 no mod	[illegible]	p08333	Golgi resident protein GCP60
PPFIA3 pT714	[illegible]	p16167	Liprin-alpha-3
ANK3 pS1459	[illegible]	p06801	Ankyrin-3
SPAG9 pT595	[illegible]	p03595	C-Jun-amino-terminal kinase-interacting protein 4
HSPB1 pS15	[illegible]	p15427	Heat shock protein beta-1
FOXK1 seq: 236 - 259 + Phospho (ST)	[illegible]	p04860	Forkhead box protein K1
ID2 seq: 12 - 24 + Phospho (ST)	[illegible]	p12836	DNA-binding protein inhibitor ID-2
CEP170 seq: 463 - 477 + Phospho (ST)	[illegible]	p03881	Centrosomal protein of 170 kDa
DDI2 seq: 109 - 139 + Phospho (ST)	[illegible]	p07963	Protein DDI1 homolog 2
SGK223 seq: 504 - 526 + Phospho (ST)	[illegible]	p15417	Tyrosine-protein kinase SgK223
HELZ seq: 1874 - 1901 + Phospho (ST)	[illegible]	p08555	Probable helicase with zinc finger domain
KANK1 pS325	[illegible]	p03147	KN motif and ankyrin repeat domain-containing protein 1
ZNF185 seq: 62 - 80 + Phospho (ST)	[illegible]	p19332	Zinc finger protein 185
HBS1L seq: 46 - 80 + Phospho (ST)	[illegible]	p18673	HBS1-like protein
MDC1 seq: 1153 - 1188 + Phospho (ST)	[illegible]	p03573	Mediator of DNA damage checkpoint protein 1
GAB2 seq: 208 - 216 + Phospho (ST)	[illegible]	p06265	GRB2-associated-binding protein 2
IRS1 seq: 1029 - 1074 + Phospho (ST)	[illegible]	p19354	Insulin receptor substrate 1
FASN seq: 1828 - 1841 no mod	[illegible]	p19008	Fatty acid synthase
ZNF704 seq: 294 - 321 + Phospho (ST)	[illegible]	p06281	Zinc finger protein 704
FAM83E pS319 pS324	[illegible]	p03398	Protein FAM83E
CDC42EP4 seq: 18 - 35 + Oxidation (M); Phospho (ST)	[illegible]	p04144	Cdc42 effector protein 4
DBN1 seq: 328 - 354 + Phospho (ST)	[illegible]	p10250	Drebrin
KIAA1671 seq: 1596 - 1625 + Phospho (ST)	[illegible]	p03422	Uncharacterized protein KIAA1671
GDI2 seq: 69 - 79 no mod	[illegible]	p15351	Rab GDP dissociation inhibitor beta
MTHFD2 seq: 120 - 142 no mod	[illegible]	p11178	Bifunctional methylenetetrahydrofolate dehydrogenase/cyclohydrolase, mitochondrial
TPPP pS160	[illegible]	p20430	Tubulin polymerization-promoting protein
C16orf72 pS167	[illegible]	p10461	UPF0472 protein C16orf72
LDHD seq: 1 - 6 no mod	[illegible]	p10245	Probable D-lactate dehydrogenase, mitochondrial
FAM83E pS319 pS324	[illegible]	p06714	Protein FAM83E
PDS5B seq: 1152 - 1171 + Phospho (ST)	[illegible]	p10153	Sister chromatid cohesion protein PDS5 homolog B
TBC1D4 seq: 640 - 651 + Phospho (ST)	[illegible]	p20502	TBC1 domain family member 4
SHROOM2 seq: 646 - 662 + Phospho (ST)	[illegible]	p03407	Protein Shroom2
PDCD6IP seq: 121 - 151 no mod	[illegible]	p06119	Programmed cell death 6-interacting protein
CHD4 seq: 354 - 390 + Phospho (Y)	[illegible]	p13202	Chromodomain-helicase-DNA-binding protein 4
SYTL2 pS562	[illegible]	p03310	Synaptotagmin-like protein 2
H1F0 seq: 28 - 40 no mod	[illegible]	p00124	Histone H1.0
CGNL1 seq: 386 - 409 + Phospho (ST)	[illegible]	p03999	Cingulin-like protein 1
EIF4EBP2 pS65 pT70	[illegible]	p07016	Eukaryotic translation initiation factor 4E-binding protein 2
PHF23 seq: 145 - 179 + Phospho (ST)	[illegible]	p01756	PHD finger protein 23
PPP1R13L seq: 524 - 541 + Oxidation (M); Phospho (ST)	[illegible]	p07331	RelA-associated inhibitor
RNF19A seq: 282 - 303 + Phospho (ST)	[illegible]	p03702	E3 ubiquitin-protein ligase RNF19A
SH3PXD2A pS1038	[illegible]	p21020	SH3 and PX domain-containing protein 2A
SMARCAD1 seq: 118 - 140 + 2 Phospho (ST)	[illegible]	p18144	SWI/SNF-related matrix-associated actin-dependent regulator of chromatin subfamily A containing DEAD/H box 1
TMPO seq: 61 - 86 + Phospho (ST)	[illegible]	p15476	Lamina-associated polypeptide 2, isoform alpha
TBC1D4 seq: 640 - 652 + Phospho (ST)	[illegible]	p20501	TBC1 domain family member 4
TPR seq: 395 - 417 no mod	[illegible]	p10567	Nucleoprotein TPR
APBB2 pS160	[illegible]	p05888	Amyloid beta A4 precursor protein-binding family B member 2
ZNF574 seq: 296 - 329 + Phospho (ST)	[illegible]	p09301	Zinc finger protein 574
ADD1 seq: 447 - 467 + Phospho (ST)	[illegible]	p00492	Alpha-adducin
SNX1 seq: 14 - 55 + Phospho (ST)	[illegible]	p11095	Sorting nexin-1
CREBZF seq: 8 - 37 + 2 Phospho (ST)	[illegible]	p11472	CREB/ATF bZIP transcription factor
ARHGEF11 pS1155	[illegible]	p14666	Rho guanine nucleotide exchange factor 11
EPN3 seq: 492 - 515 + 2 Phospho (ST)	[illegible]	p20943	Epsin-3
LSM14B pS165	[illegible]	p04525	Protein LSM14 homolog B
FZD6 seq: 610 - 636 + Phospho (ST)	[illegible]	p10906	Frizzled-6
GLO1 pM36	[illegible]	p18875	Lactoylglutathione lyase
PAICS seq: 132 - 149 no mod	[illegible]	p18923	Multifunctional protein ADE2
HSPB8 pS57	[illegible]	p10673	Heat shock protein beta-8
TLN1 pS2040	[illegible]	p01119	Talin-1
AKT1 pS473	[illegible]	p06929	RAC-alpha serine/threonine-protein kinase
LIMD1 pS233	[illegible]	p08297	LIM domain-containing protein 1
ZNF609 seq: 592 - 629 + 2 Phospho (ST)	[illegible]	p12924	Zinc finger protein 609
NDRG1 seq: 362 - 388 + 2 Phospho (ST)	[illegible]	p04052	Protein NDRG1
DSG2 seq: 855 - 892 + Phospho (ST)	[illegible]	p08454	Desmoglein-2
MLPH seq: 145 - 185 + 3 Phospho (ST)	[illegible]	p10998	Melanophilin
LATS1 pT17	[illegible]	p02858	Serine/threonine-protein kinase LATS1
HID1 seq: 668 - 689 + Phospho (ST)	[illegible]	p06835	Protein HID1
PKP4 seq: 219 - 234 + Phospho (Y)	[illegible]	p19756	Plakophilin-4
ZNF185 pS64	[illegible]	p19333	Zinc finger protein 185
BLVRB pS82	[illegible]	p09700	Flavin reductase (NADPH)
ZNF652 pS197	[illegible]	p21010	Zinc finger protein 652
PKP4 seq: 219 - 234 + Phospho (ST)	[illegible]	p19757	Plakophilin-4
VCL pS346	[illegible]	p15398	Vinculin
SHROOM2 seq: 845 - 860 + Phospho (ST)	[illegible]	p02013	Protein Shroom2
CSRP1 seq: 70 - 91 + Phospho (ST)	[illegible]	p14930	Cysteine and glycine-rich protein 1
MTSS1L pS300	[illegible]	p16060	MTSS1-like protein
[illegible]seq: 230 - 268 + Gln->pyro-Glu (N-term Q); Phospho (ST)	[illegible]	p07902	Chromosome alignment-maintaining phosphoprotein 1
BAIAP2L2 seq: 293 - 312 + 3 Phospho (ST)	[illegible]	p05106	Brain-specific angiogenesis inhibitor 1-associated protein 2-like protein 2
PDZD2 pS1270	[illegible]	p02068	PDZ domain-containing protein 2

TBX3 pS707	[illegible]	p03593	T-box transcription factor TBX3
GSK3B pS9 pM26	[illegible]	p02023	Glycogen synthase kinase-3 beta
PFKFB2 pS483	[illegible]	p08907	6-phosphofructo-2-kinase/fructose-2,6-bisphosphatase 2
MAPT pS673	[illegible]	p14236	Microtubule-associated protein tau
DOPEY2 pS681	[illegible]	p12831	Protein dopey-2
ARHGEF6 pS488	[illegible]	p09902	Rho guanine nucleotide exchange factor 6
ATG2A seq: 1260 - 1281 + Phospho (ST)	[illegible]	p10808	Autophagy-related protein 2 homolog A
FRS2 seq: 323 - 342 + Phospho (ST)	[illegible]	p15337	Fibroblast growth factor receptor substrate 2
IQSEC1 seq: 84 - 92 + Phospho (ST)	[illegible]	p10994	IQ motif and SEC7 domain-containing protein 1
EIF4ENIF1 pS374	[illegible]	p12260	Eukaryotic translation initiation factor 4E transporter
RAP1GAP seq: 497 - 513 + Phospho (ST)	[illegible]	p06628	Rap1 GTPase-activating protein 1
ACLY seq: 453 - 468 + Phospho (ST)	[illegible]	p03036	ATP-citrate synthase
CCDC85C seq: 178 - 200 + Phospho (ST)	[illegible]	p03870	Coiled-coil domain-containing protein 85C
GIT1 seq: 559 - 582 + Phospho (ST)	[illegible]	p14994	ARF GTPase-activating protein GIT1
C1orf198 seq: 173 - 192 + Phospho (ST)	[illegible]	p03696	Uncharacterized protein C1orf198
HSPB1 seq: 97 - 112 no mod	[illegible]	p00833	Heat shock protein beta-1
KRT18 seq: 51 - 90 + 2 Oxidation (M); Phospho (ST)	[illegible]	p03460	Keratin, type I cytoskeletal 18
HNRNPR seq: 587 - 633 no mod	[illegible]	p07793	Heterogeneous nuclear ribonucleoprotein R
LDB1 seq: 300 - 320 + Oxidation (M); Phospho (ST)	[illegible]	p12862	LIM domain-binding protein 1
EPPK1 seq: 2366 - 2374 no mod	[illegible]	p14936	Epiplakin
ADD1 pS586	[illegible]	p08460	Alpha-adducin
HSPB1 seq: 28 - 37 no mod	[illegible]	p11907	Heat shock protein beta-1
LIMA1 seq: 614 - 629 + Oxidation (M); Phospho (ST)	[illegible]	p13131	LIM domain and actin-binding protein 1
APRT seq: 123 - 145 no mod	[illegible]	p00557	Adenine phosphoribosyltransferase
NDUFV2 seq: 200 - 209 no mod	[illegible]	p18760	NADH dehydrogenase [ubiquinone] flavoprotein 2, mitochondrial
CDH2 pS135	[illegible]	p10757	Cadherin-2
BAD seq: 97 - 109 + Phospho (ST)	[illegible]	p04073	Bcl2-associated agonist of cell death
RPLP0 seq: 150 - 162 no mod	[illegible]	p15091	60S acidic ribosomal protein P0
AP3D1 pS9	[illegible]	p09823	AP-3 complex subunit delta-1
RNF20 pS138	[illegible]	p13430	E3 ubiquitin-protein ligase BRE1A
PUF60 seq: 277 - 298 no mod	[illegible]	p19751	Poly(U)-binding-splicing factor PUF60
MIER2 seq: 167 - 192 + Phospho (ST)	[illegible]	p16637	Mesoderm induction early response protein 2
AMOT pS823	[illegible]	p15248	Angiomotin
MTFR1 pS119	[illegible]	p08502	Mitochondrial fission regulator 1
CCDC85C seq: 156 - 200 + Phospho (ST)	[illegible]	p21114	Coiled-coil domain-containing protein 85C
PAICS seq: 287 - 298 no mod	[illegible]	p20954	Multifunctional protein ADE2
RPL8 seq: 94 - 123 no mod	[illegible]	p19787	60S ribosomal protein L8
MYH14 pS1969	[illegible]	p07728	Myosin-14
LASP1 pS146	[illegible]	p10095	LIM and SH3 domain protein 1
ADCY6 seq: 572 - 583 + Phospho (ST)	[illegible]	p02235	Adenylate cyclase type 6
GRIA1 pT2517	[illegible]	p02072	Glutamate receptor 1
SSH3 seq: 599 - 615 + Phospho (ST)	[illegible]	p04076	Protein phosphatase Slingshot homolog 3
ZHX1 seq: 524 - 558 + Phospho (ST)	[illegible]	p04818	Zinc fingers and homeoboxes protein 1
PREX1 pS1182	[illegible]	p18200	Phosphatidylinositol 3,4,5-trisphosphate-dependent Rac exchanger 1 protein
MECP2 seq: 378 - 417 + Phospho (ST)	[illegible]	p19852	Methyl-CpG-binding protein 2
MYO18A pS987	[illegible]	p20584	Unconventional myosin-XVIIIa
CAP2 seq: 294 - 314 + Phospho (ST)	[illegible]	p19808	Adenylyl cyclase-associated protein 2
RBM14 seq: 627 - 636 + Phospho (ST)	[illegible]	p04339	RNA-binding protein 14
DARS2 seq: 389 - 408 no mod	[illegible]	p09662	Aspartate--tRNA ligase, mitochondrial
TRPS1 pS178	[illegible]	p19400	Zinc finger transcription factor Trps1
BCL2L14 pS152	[illegible]	p01345	Apoptosis facilitator Bcl-2-like protein 14
FAM63A pS441	[illegible]	p01174	Protein FAM63A
VEZT pS696	[illegible]	p10226	Vezatin
JUN seq: 55 - 70 + Phospho (ST)	[illegible]	p20396	Transcription factor AP-1
HIVEP1 pS1753	[illegible]	p10001	Zinc finger protein 40
VCP seq: 767 - 806 no mod	[illegible]	p16113	Transitional endoplasmic reticulum ATPase
PER2 pS627	[illegible]	p13369	Period circadian protein homolog 2
ARHGAP35 pS975	[illegible]	p20564	Rho GTPase-activating protein 35
CUL4A pS10	[illegible]	p13162	Cullin-4A
TRIM16 seq: 54 - 85 + Phospho (ST)	[illegible]	p17068	Tripartite motif-containing protein 16
VPS13D pS1042	[illegible]	p19755	Vacuolar protein sorting-associated protein 13D
PLEKHA1 seq: 330 - 342 + Phospho (ST)	[illegible]	p04775	Pleckstrin homology domain-containing family A member 1
DLGAP4 pS973	[illegible]	p08192	Disks large-associated protein 4
NAV1 seq: 464 - 482 + Phospho (ST)	[illegible]	p05320	Neuron navigator 1
SAP30L seq: 91 - 120 + 2 Phospho (ST)	[illegible]	p12470	Histone deacetylase complex subunit SAP30L
NAB2 pS479	[illegible]	p01454	NGFI-A-binding protein 2
COBLL1 pS382	[illegible]	p20575	Cordon-bleu protein-like 1
NPDC1 pS229	[illegible]	p19981	Neural proliferation differentiation and control protein 1
EIF4ENIF1 seq: 210 - 241 + Phospho (ST)	[illegible]	p07037	Eukaryotic translation initiation factor 4E transporter
MTMR4 seq: 608 - 626 + Phospho (ST)	[illegible]	p02215	Myotubularin-related protein 4
ARHGEF17 seq: 498 - 535 + Phospho (ST)	[illegible]	p15239	Rho guanine nucleotide exchange factor 17
GOLGB1 seq: 405 - 430 no mod	[illegible]	p11317	Golgin subfamily B member 1
SHANK2 pS1330	[illegible]	p07614	SH3 and multiple ankyrin repeat domains protein 2
TANC2 pS1545 pM1551	[illegible]	p19542	Protein TANC2
WWC3 pS927	[illegible]	p06090	Protein WWC3
USP5 seq: 361 - 379 no mod	[illegible]	p14295	Ubiquitin carboxyl-terminal hydrolase 5
NENF seq: 70 - 79 no mod	[illegible]	p14970	Neudesin
ASH1L seq: 1733 - 1751 + Phospho (ST)	[illegible]	p05538	Histone-lysine N-methyltransferase ASH1L
MYO9B pS1331	[illegible]	p10457	Unconventional myosin-IXb
PPP1R1A pS67	[illegible]	p03508	Protein phosphatase 1 regulatory subunit 1A
USP14 seq: 401 - 429 no mod	[illegible]	p00406	Ubiquitin carboxyl-terminal hydrolase 14
BAD pS118	[illegible]	p07051	Bcl2-associated agonist of cell death
PLEKHA6 seq: 1008 - 1038 + 3 Phospho (ST)	[illegible]	p09995	Pleckstrin homology domain-containing family A member 6
TAF15 seq: 222 - 255 no mod	[illegible]	p02981	TATA-binding protein-associated factor 2N
CASKIN1 pS1257	[illegible]	p12414	Caskin-1
UBE4B pS105	[illegible]	p04180	Ubiquitin conjugation factor E4 B
FNBP1L seq: 486 - 515 + 2 Phospho (ST)	[illegible]	p07291	Formin-binding protein 1-like
LATS1 seq: 244 - 252 + Phospho (ST)	[illegible]	p03223	Serine/threonine-protein kinase LATS1
SERBP1 seq: 328 - 345 + Phospho (ST)	[illegible]	p05372	Plasminogen activator inhibitor 1 RNA-binding protein
CHMP5 pS86	[illegible]	p18457	Charged multivesicular body protein 5
SNX29 pS268	[illegible]	p12382	Sorting nexin-29
GRB7 seq: 366 - 383 + Oxidation (M); Phospho (ST)	[illegible]	p06274	Growth factor receptor-bound protein 7
IQGAP1 seq: 283 - 302 no mod	[illegible]	p17246	Ras GTPase-activating-like protein IQGAP1
LSM14B seq: 196 - 221 no mod	[illegible]	p03050	Protein LSM14 homolog B
ITPR3 pS1832	[illegible]	p01764	Inositol 1,4,5-trisphosphate receptor type 3
PDS5B seq: 1396 - 1438 + Phospho (ST)	[illegible]	p05389	Sister chromatid cohesion protein PDS5 homolog B
DLG1 pS687	[illegible]	p14554	Disks large homolog 1
[illegible]eq: 331 - 351 + Gln->pyro-Glu (N-term Q); Phospho (ST)	[illegible]	p08156	MKL/myocardin-like protein 1
PHKA2 seq: 1039 - 1063 + Phospho (ST)	[illegible]	p03580	Phosphorylase b kinase regulatory subunit alpha, liver isoform
GSK3A seq: 19 - 50 + Phospho (ST)	[illegible]	p02112	Glycogen synthase kinase-3 alpha
RPL23 seq: 92 - 109 no mod	[illegible]	p18793	60S ribosomal protein L23
MAPT seq: 527 - 538 + Phospho (ST)	[illegible]	p03969	Microtubule-associated protein tau
SDHA seq: 601 - 615 no mod	[illegible]	p14432	Succinate dehydrogenase [ubiquinone] flavoprotein subunit, mitochondrial
RACGAP1 seq: 405 - 411 + Phospho (ST)	[illegible]	p01935	Rac GTPase-activating protein 1
NOP56 seq: 561 - 578 + 2 Phospho (ST)	[illegible]	p13230	Nucleolar protein 56
UBE2O pS515	[illegible]	p05501	(E3-independent) E2 ubiquitin-conjugating enzyme
TTN seq: 25718 - 25724 + Phospho (ST)	[illegible]	p05503	Titin
FH seq: 234 - 256 no mod	[illegible]	p02742	Fumarate hydratase, mitochondrial
E2F8 seq: 65 - 75 + Oxidation (M); Phospho (ST)	[illegible]	p10028	Transcription factor E2F8
TLK2 seq: 168 - 199 + Phospho (ST)	[illegible]	p16319	Serine/threonine-protein kinase tousled-like 2
ZSWIM8 seq: 1138 - 1161 + Phospho (ST)	[illegible]	p14591	Zinc finger SWIM domain-containing protein 8
IQSEC1 seq: 84 - 93 + Phospho (ST)	[illegible]	p10993	IQ motif and SEC7 domain-containing protein 1
SQSTM1 seq: 345 - 378 no mod	[illegible]	p17009	Sequestosome-1
TNS2 pS1003	[illegible]	p04307	Tensin-2
OXR1 seq: 330 - 359 + Phospho (ST)	[illegible]	p03547	Oxidation resistance protein 1
SHTN1 pS494	[illegible]	p12389	Shootin-1
FAM102B pS228	[illegible]	p03712	Protein FAM102B
OTUD5 seq: 63 - 80 + Phospho (ST)	[illegible]	p19539	OTU domain-containing protein 5
PPP1R3D seq: 23 - 39 + Phospho (ST)	[illegible]	p05069	Protein phosphatase 1 regulatory subunit 3D
TANC1 seq: 1675 - 1693 + Phospho (ST)	[illegible]	p09872	Protein TANC1
HTT seq: 386 - 416 + Phospho (ST)	[illegible]	p02357	Huntingtin
ARHGEF12 seq: 742 - 775 + Phospho (ST)	[illegible]	p13258	Rho guanine nucleotide exchange factor 12
TMPO seq: 216 - 239 + Phospho (ST)	[illegible]	p06471	Lamina-associated polypeptide 2, isoforms beta/gamma
CDC42EP4 pS174	[illegible]	p07033	Cdc42 effector protein 4
HDGFRP2 pS490	[illegible]	p16861	Hepatoma-derived growth factor-related protein 2
TJP3 seq: 344 - 357 + Phospho (ST)	[illegible]	p02694	Tight junction protein ZO-3
FOXP4 seq: 75 - 93 + Phospho (ST)	[illegible]	p08678	Forkhead box protein P4
STK3 pS369	[illegible]	p19301	Serine/threonine-protein kinase 3
TRIM28 seq: 747 - 767 + Phospho (ST)	[illegible]	p11011	Transcription intermediary factor 1-beta
FAM83H seq: 583 - 619 + Phospho (ST)	[illegible]	p12161	Protein FAM83H
AAK1 seq: 728 - 760 + Phospho (ST)	[illegible]	p11772	AP2-associated protein kinase 1
TPM3 seq: 92 - 101 no mod	[illegible]	p13807	Tropomyosin alpha-3 chain
SLC19A1 seq: 576 - 591 no mod	[illegible]	p11738	Folate transporter 1
RBM10 pS736 pS738	[illegible]	p15630	RNA-binding protein 10
RPRD2 pT723	[illegible]	p15422	Regulation of nuclear pre-mRNA domain-containing protein 2
FASN seq: 612 - 631 no mod	[illegible]	p17962	Fatty acid synthase
C2CD4C pS178	[illegible]	p17152	C2 calcium-dependent domain-containing protein 4C
IQSEC1 pS512	[illegible]	p09045	IQ motif and SEC7 domain-containing protein 1
PLEC seq: 1338 - 1344 no mod	[illegible]	p17501	Plectin
FER1L4 seq: 477 - 483 no mod	[illegible]	p11988	Fer-1-like protein 4
KIAA1467 seq: 52 - 93 + Phospho (ST)	[illegible]	p04563	Uncharacterized protein KIAA1467
GLO1 seq: 61 - 67 no mod	[illegible]	p19064	Lactoylglutathione lyase
HSPA5 seq: 620 - 633 no mod	[illegible]	p13049	78 kDa glucose-regulated protein
SMTN pS304	[illegible]	p04967	Smoothelin
KIF22 seq: 449 - 465 + Phospho (ST)	[illegible]	p11467	Kinesin-like protein KIF22
FUS seq: 275 - 306 no mod	[illegible]	p14862	RNA-binding protein FUS
TMX1 pS247	[illegible]	p12445	Thioredoxin-related transmembrane protein 1
THUMPD1 seq: 305 - 346 no mod	[illegible]	p09314	THUMP domain-containing protein 1
ZNF318 pS136	[illegible]	p04634	Zinc finger protein 318
FASN seq: 2234 - 2263 + Phospho (ST)	[illegible]	p11155	Fatty acid synthase
UGGT1 seq: 1178 - 1201 no mod	[illegible]	p13489	UDP-glucose:glycoprotein glucosyltransferase 1
CDC42EP1 seq: 99 - 117 + Phospho (ST)	[illegible]	p10238	Cdc42 effector protein 1
AKAP13 seq: 1936 - 1969 + 2 Phospho (ST)	[illegible]	p13718	A-kinase anchor protein 13
CNOT2 seq: 54 - 87 + Phospho (ST)	[illegible]	p06019	CCR4-NOT transcription complex subunit 2
TNKS1BP1 pS228	[illegible]	p14945	182 kDa tankyrase-1-binding protein
NAB2 seq: 157 - 183 + 2 Phospho (ST)	[illegible]	p05822	NGFI-A-binding protein 2
RBM23 seq: 137 - 146 + 2 Phospho (ST)	[illegible]	p00367	Probable RNA-binding protein 23
ARFGEF3 seq: 2078 - 2088 + Phospho (ST)	[illegible]	p14647	Brefeldin A-inhibited guanine nucleotide-exchange protein 3
MIS18BP1 pS110	[illegible]	p20009	Mis18-binding protein 1
ETHE1 pS19	[illegible]	p15972	Persulfide dioxygenase ETHE1, mitochondrial
GTF2I seq: 681 - 702 no mod	[illegible]	p01021	General transcription factor II-I
KIF21A seq: 409 - 436 no mod	[illegible]	p14190	Kinesin-like protein KIF21A
SLAIN2 pS377	[illegible]	p00796	SLAIN motif-containing protein 2
GOLPH3 seq: 192 - 210 + Gln->pyro-Glu (N-term Q)	[illegible]	p08220	Golgi phosphoprotein 3
RPS6KC1 pS644	[illegible]	p05874	Ribosomal protein S6 kinase delta-1
KIF5B seq: 356 - 367 no mod	[illegible]	p09023	Kinesin-1 heavy chain
SEPT9 seq: 187 - 222 no mod	[illegible]	p06470	Septin-9
DDX20 pS500	[illegible]	p09291	Probable ATP-dependent RNA helicase DDX20
ALDOC seq: 112 - 134 no mod	[illegible]	p14967	Fructose-bisphosphate aldolase C
MAP7D1 seq: 809 - 829 + Phospho (ST)	[illegible]	p04998	MAP7 domain-containing protein 1
TSC22D4 seq: 291 - 321 + Phospho (ST)	[illegible]	p16466	TSC22 domain family protein 4
FAM134C seq: 305 - 328 + 2 Phospho (ST)	[illegible]	p15369	Protein FAM134C
KIAA1671 seq: 1057 - 1086 + Phospho (ST)	[illegible]	p13062	Uncharacterized protein KIAA1671
ZNF318 seq: 1609 - 1648 + Phospho (ST)	[illegible]	p03928	Zinc finger protein 318
RXRA seq: 5 - 25 + Phospho (ST)	[illegible]	p14832	Retinoic acid receptor RXR-alpha
CFL2 seq: 96 - 112 no mod	[illegible]	p13313	Cofilin-2
RAD21 seq: 372 - 384 no mod	[illegible]	p11863	Double-strand-break repair protein rad21 homolog
ANO8 seq: 627 - 647 + Phospho (ST)	[illegible]	p06749	Anoctamin-8
RFX1 seq: 763 - 780 no mod	[illegible]	p19225	MHC class II regulatory factor RFX1
HSPB1 pS15	[illegible]	p15426	Heat shock protein beta-1
INPP5F pS1069	[illegible]	p19185	Phosphatidylinositide phosphatase SAC2
CRTC1 seq: 149 - 178 + Oxidation (M); Phospho (ST)	[illegible]	p02469	CREB-regulated transcription coactivator 1
TLN1 seq: 1626 - 1638 no mod	[illegible]	p18401	Talin-1
AHSA1 seq: 225 - 246 no mod	[illegible]	p01523	Activator of 90 kDa heat shock protein ATPase homolog 1
GAPDH seq: 163 - 186 no mod	[illegible]	p01390	Glyceraldehyde-3-phosphate dehydrogenase
PPP4R3A seq: 674 - 704 + Phospho (ST)	[illegible]	p02619	Serine/threonine-protein phosphatase 4 regulatory subunit 3A
CEP170B pS969 pS972	[illegible]	p05704	Centrosomal protein of 170 kDa protein B
AHSA1 seq: 17 - 30 no mod	[illegible]	p20933	Activator of 90 kDa heat shock protein ATPase homolog 1
FLNC pS2233	[illegible]	p17228	Filamin-C
FASN seq: 712 - 728 no mod	[illegible]	p00490	Fatty acid synthase
TNKS1BP1 seq: 1543 - 1572 + 2 Phospho (ST)	[illegible]	p06851	182 kDa tankyrase-1-binding protein
SAP130 pS855 pT856	[illegible]	p12658	Histone deacetylase complex subunit SAP130
RIF1 pS2260	[illegible]	p16107	Telomere-associated protein RIF1
UCKL1 seq: 61 - 73 + Phospho (ST)	[illegible]	p02003	Uridine-cytidine kinase-like 1
FOXO3 seq: 30 - 72 + Phospho (ST)	[illegible]	p06123	Forkhead box protein O3
SSH1 pS576	[illegible]	p13005	Protein phosphatase Slingshot homolog 1
HN1 seq: 47 - 64 no mod	[illegible]	p10244	Hematological and neurological expressed 1 protein
TCF3 seq: 335 - 371 + 2 Phospho (ST)	[illegible]	p20316	Transcription factor E2-alpha
VIM seq: 295 - 310 no mod	[illegible]	p16877	Vimentin
EIF4B seq: 118 - 125 no mod	[illegible]	p15689	Eukaryotic translation initiation factor 4B
SRRM2 seq: 1129 - 1158 + Phospho (ST)	[illegible]	p05715	Serine/arginine repetitive matrix protein 2
CDT1 pS318	[illegible]	p20758	DNA replication factor Cdt1
CLNS1A seq: 213 - 237 no mod	[illegible]	p18058	Methylosome subunit pICln
DPYSL2 seq: 521 - 532 + Phospho (ST)	[illegible]	p02162	Dihydropyrimidinase-related protein 2
PDXDC1 seq: 8 - 18 no mod	[illegible]	p14522	Pyridoxal-dependent decarboxylase domain-containing protein 1
NUP107 pS37	[illegible]	p01210	Nuclear pore complex protein Nup107
MCM2 seq: 34 - 79 + Oxidation (M); 2 Phospho (ST)	[illegible]	p06535	DNA replication licensing factor MCM2
ERBB2 seq: 1049 - 1096 + 2 Phospho (ST)	[illegible]	p03613	Receptor tyrosine-protein kinase erbB-2
LAMTOR1 seq: 92 - 103 + Phospho (ST)	[illegible]	p12142	Ragulator complex protein LAMTOR1
PSMC6 seq: 147 - 161 no mod	[illegible]	p16980	26S protease regulatory subunit 10B

CEP170B pS951 pS954	38324 4E+07 39656 13654 33854 45779 25552 34892 34104 26736 56234 53206 39754 40879 18798 40867 2E+07 34984 19162 28832 39759 50401 21442 19024 42741 6E+08 2E+08 [illegible]	p11285	Centrosomal protein of 170 kDa protein B
SYAP1 seq: 255 - 291 + Phospho (ST)	38324 37149 39656 2E+08 9E+07 45779 25552 34892 34104 26736 56234 53206 39754 8E+07 18798 40867 33072 34984 19162 4E+07 39759 50401 21442 19024 42741 68554 44396 [illegible]	p04243	Synapse-associated protein 1
[illegible]: 1524 - 1556 + Gln->pyro-Glu (N-term Q); Phospho (ST)	38324 37149 2E+07 2E+07 33854 45779 7E+06 34892 34104 26736 56234 53206 39754 40879 18798 40867 33072 34984 19162 28832 39759 1E+06 21442 19024 42741 3E+08 3E+08 [illegible]	p08110	LIM domain only protein 7
FOSL1 pS265	38324 37149 39656 2E+07 2E+07 45779 25552 34892 34104 26736 5E+07 53206 39754 2E+07 18798 40867 33072 34984 19162 28832 39759 50401 21442 19024 42741 2E+09 2E+09 [illegible]	p12529	Fos-related antigen 1
VCP pM508	38324 6E+08 7E+08 13654 3E+06 45779 25552 34892 34104 757930 3E+09 53206 1E+07 1E+07 1E+06 40867 33072 34984 19162 28832 39759 1E+07 21442 19024 42741 68554 638039 [illegible]	p16748	Transitional endoplasmic reticulum ATPase
NFIC seq: 452 - 486 + Phospho (ST)	38324 37149 7E+07 13654 33854 45779 25552 34892 34104 26736 56234 53206 39754 40879 18798 40867 33072 34984 19162 28832 39759 50401 21442 19024 42741 68554 3E+07 [illegible]	p12233	Nuclear factor 1 C-type
PTPN14 pS314	6E+07 37149 39656 13654 1E+08 3E+07 25552 34892 34104 26736 56234 53206 39754 1E+07 18798 40867 33072 34984 19162 28832 39759 50401 21442 19024 5E+06 3E+08 7E+07 [illegible]	p14451	Tyrosine-protein phosphatase non-receptor type 14
SUZ12 pS546	38324 37149 39656 1E+07 2E+07 45779 25552 34892 2E+07 26736 56234 53206 39754 40879 18798 40867 33072 34984 19162 1E+07 39759 50401 21442 19024 42741 68554 44396 [illegible]	p19567	Polycomb protein SUZ12
PHLDB2 pS334	2E+08 2E+08 39656 13654 33854 45779 3E+08 3E+08 34104 26736 56234 53206 39754 40879 18798 2E+08 33072 34984 19162 28832 39759 50401 21442 19024 42741 7E+08 2E+08 [illegible]	p02196	Pleckstrin homology-like domain family B member 2
CMIP pS377	2E+07 1E+08 39656 13654 5E+07 8E+07 25552 34892 34104 26736 2E+08 5E+07 39754 40879 18798 40867 33072 34984 19162 28832 39759 50401 21442 19024 1E+07 3E+08 1E+08 [illegible]	p11385	C-Maf-inducing protein
YBX1 pS209	38324 37149 39656 5E+07 33854 45779 25552 34892 34104 26736 2E+08 8E+07 5E+07 40879 5E+07 40867 33072 34984 19162 28832 39759 50401 1E+07 19024 42741 3E+08 8E+07 [illegible]	p06867	Nuclease-sensitive element-binding protein 1
FOXO3 pS253 pM254	7E+07 2E+08 2E+08 1E+08 1E+08 2E+07 8E+06 34892 34104 26736 4E+07 8E+06 39754 1E+06 3E+06 5E+07 3E+06 34984 19162 6E+06 2E+07 50401 21442 19024 1E+07 9E+08 5E+08 [illegible]	p19189	Forkhead box protein O3
TNS3 pM1438 pS1441	1E+08 37149 4E+07 13654 33854 45779 25552 2E+06 34104 26736 3E+07 9E+06 39754 1E+06 2E+06 40867 33072 34984 19162 28832 39759 50401 2E+07 19024 42741 2E+09 8E+08 [illegible]	p01114	Tensin-3
EPB41 seq: 652 - 662 + Phospho (Y)	7E+06 37149 39656 13654 33854 45779 25552 34892 34104 26736 56234 53206 39754 40879 18798 40867 33072 34984 19162 28832 39759 50401 21442 19024 42741 6E+08 2E+08 [illegible]	p17230	Protein 4.1
BAIAP2L1 seq: 239 - 266 + Oxidation (M); 4 Phospho (ST)	38324 37149 39656 6E+06 4E+06 45779 25552 34892 34104 26736 56234 53206 39754 40879 18798 40867 33072 34984 19162 28832 39759 50401 21442 19024 42741 68554 2E+08 [illegible]	p13950	Brain-specific angiogenesis inhibitor 1-associated protein 2-like protein 1
CDC42BPB seq: 1635 - 1656 + Phospho (Y)	2E+07 37149 39656 13654 33854 45779 25552 34892 34104 26736 56234 3E+07 39754 40879 18798 40867 33072 34984 19162 28832 39759 50401 21442 19024 42741 2E+08 2E+08 [illegible]	p09499	Serine/threonine-protein kinase MRCK beta
CAP1 seq: 356 - 364 no mod	2E+06 9E+06 2E+07 13654 4E+06 2E+07 25552 34892 4E+06 26736 2E+08 53206 39754 40879 18798 40867 33072 4E+06 19162 28832 39759 50401 21442 19024 42741 1E+07 44396 [illegible]	p19004	Adenylyl cyclase-associated protein 1
PAWR seq: 226 - 240 + Phospho (ST)	2E+07 5E+07 4E+07 8E+07 6E+07 4E+07 3E+07 4E+07 3E+07 26736 5E+07 4E+07 2E+07 4E+07 18798 3E+07 6E+06 5E+07 5E+07 2E+07 39759 50401 21442 19024 42741 68554 44396 [illegible]	p00285	PRKC apoptosis WT1 regulator protein
CHFR seq: 161 - 200 + Phospho (ST)	8E+07 37149 3E+07 3E+07 33854 45779 25552 34892 34104 26736 56234 53206 39754 7E+07 3E+07 40867 33072 34984 19162 28832 39759 3E+07 21442 5E+07 2E+07 2E+08 7E+07 [illegible]	p00994	E3 ubiquitin-protein ligase CHFR
UBE2M seq: 170 - 180 no mod	38324 37149 4E+07 2E+07 33854 45779 25552 34892 34104 26736 1E+08 53206 4E+07 40879 18798 3E+07 33072 34984 19162 28832 39759 50401 21442 19024 42741 1E+07 44396 [illegible]	p15935	NEDD8-conjugating enzyme Ubc12
[illegible] ENSP00000383309 OS=Homo sapiens PE=5 SV=3 pS109	38324 37149 3E+06 1E+08 33854 45779 25552 6E+07 34104 26736 56234 53206 39754 40879 18798 40867 33072 34984 19162 28832 5E+06 4E+07 5E+06 19024 42741 68554 44396 [illegible]	p05109	Putative uncharacterized protein ENSP00000383309
SNX1 pS32 pS39	2E+08 37149 39656 13654 1E+08 45779 25552 1E+08 34104 26736 56234 53206 39754 40879 18798 40867 33072 2E+08 19162 28832 39759 50401 21442 19024 42741 68554 44396 [illegible]	p11099	Sorting nexin-1
LGALS1 seq: 113 - 128 no mod	38324 2E+07 3E+07 13654 33854 45779 4E+07 34892 34104 26736 56234 53206 39754 40879 18798 40867 33072 34984 19162 28832 39759 50401 21442 19024 42741 3E+08 2E+07 [illegible]	p11183	Galectin-1
MTDH seq: 492 - 504 + Phospho (ST)	38324 37149 4E+07 2E+07 33854 45779 25552 34892 34104 26736 5E+07 1E+07 3E+07 4E+07 18798 40867 33072 5E+06 19162 28832 39759 50401 5E+06 1E+07 42741 2E+08 5E+07 [illegible]	p02688	Protein LYRIC
CTSD seq: 314 - 331 no mod	38324 5E+06 3E+07 13654 33854 45779 25552 1E+07 34104 26736 1E+08 53206 2E+07 40879 18798 40867 33072 34984 19162 28832 39759 50401 21442 19024 42741 6E+07 44396 [illegible]	p20475	Cathepsin D
RELA seq: 134 - 158 no mod	38324 37149 1E+07 1E+08 8E+07 45779 25552 34892 34104 26736 56234 53206 39754 6E+07 18798 40867 33072 34984 3E+07 28832 39759 50401 21442 7E+07 42741 4E+08 44396 [illegible]	p13781	Transcription factor p65
MYL12B pT18 pS19	3E+07 37149 39656 13654 1E+08 45779 25552 34892 7E+07 26736 56234 3E+07 39754 2E+08 2E+08 40867 33072 34984 19162 28832 39759 50401 21442 19024 42741 68554 1E+08 [illegible]	p19328	Myosin regulatory light chain 12B
FAM83H seq: 1023 - 1041 + Phospho (ST)	38324 37149 39656 3E+07 3E+07 45779 25552 34892 1E+07 26736 56234 53206 39754 40879 18798 40867 33072 34984 19162 28832 39759 50401 1E+07 2E+07 2E+07 2E+08 6E+07 [illegible]	p10676	Protein FAM83H
STOML2 seq: 115 - 135 no mod	38324 4E+06 1E+07 13654 6E+06 45779 25552 34892 34104 26736 6E+07 53206 39754 40879 18798 40867 33072 34984 19162 28832 39759 50401 21442 19024 42741 68554 44396 [illegible]	p19431	Stomatin-like protein 2, mitochondrial
PABPN1 seq: 228 - 238 no mod	38324 1E+07 [illegible]	p02172	Polyadenylate-binding protein 2
FSCN1 seq: 91 - 100 no mod	38324 2E+07 [illegible]	p16611	Fascin
PSMB1 seq: 147 - 160 no mod	3E+07 4E+07 [illegible]	p16249	Proteasome subunit beta type-1
ANXA11 seq: 431 - 439 no mod	3E+08 2E+08 [illegible]	p09013	Annexin A11
CLSPN pS839	38324 1E+07 [illegible]	p10811	Claspin
HNRNPLL seq: 402 - 408 no mod	38324 1E+08 [illegible]	p11150	Heterogeneous nuclear ribonucleoprotein L-like
BCAR3 pS375	38324 4E+06 [illegible]	p02798	Breast cancer anti-estrogen resistance protein 3
SGK223 pS907	3E+07 3E+07 [illegible]	p16073	Tyrosine-protein kinase SgK223
SCEL seq: 521 - 532 no mod	38324 1E+07 [illegible]	p09313	Sciellin
TCF20 pS1259	38324 37149 [illegible]	p05896	Transcription factor 20
LAMC2 seq: 957 - 965 no mod	38324 37149 [illegible]	p17808	Laminin subunit gamma-2
AHNAK2 pS1007	38324 37149 [illegible]	p16645	Protein AHNAK2
TRIM28 seq: 391 - 400 no mod	38324 3E+06 [illegible]	p16507	Transcription intermediary factor 1-beta
GSN seq: 598 - 615 no mod	1E+07 3E+07 [illegible]	p02341	Gelsolin
AGPS seq: 644 - 655 no mod	4E+07 6E+07 [illegible]	p16889	Alkyldihydroxyacetonephosphate synthase, peroxisomal
AHNAK seq: 204 - 225 + 2 Phospho (ST)	38324 37149 [illegible]	p01959	Neuroblast differentiation-associated protein AHNAK
CMBL seq: 109 - 115 no mod	6E+06 1E+07 [illegible]	p17599	Carboxymethylenebutenolidase homolog
SCEL pS289 pM296	7E+06 37149 [illegible]	p05245	Sciellin
PHF14 seq: 768 - 799 + 2 Phospho (ST)	38324 37149 [illegible]	p09039	PHD finger protein 14
PDCD2L seq: 114 - 154 + Phospho (ST)	38324 37149 [illegible]	p08632	Programmed cell death protein 2-like
CCT3 seq: 371 - 377 no mod	38324 1E+07 [illegible]	p20937	T-complex protein 1 subunit gamma
RPS20 seq: 35 - 41 no mod	38324 37149 [illegible]	p01724	40S ribosomal protein S20
PPP1R12A seq: 852 - 889 + 2 Phospho (ST)	6E+07 37149 [illegible]	p03518	Protein phosphatase 1 regulatory subunit 12A
DSP seq: 2606 - 2630 + Phospho (ST)	38324 37149 [illegible]	p03714	Desmoplakin
PPP1R13L seq: 186 - 235 + 2 Phospho (ST)	38324 37149 [illegible]	p15222	RelA-associated inhibitor
PDXDC1 seq: 605 - 614 no mod	38324 37149 [illegible]	p11415	Pyridoxal-dependent decarboxylase domain-containing protein 1
CEP131 seq: 87 - 110 + 2 Phospho (ST)	38324 37149 [illegible]	p05665	Centrosomal protein of 131 kDa
CCT8 seq: 172 - 181 no mod	4E+07 4E+07 [illegible]	p07670	T-complex protein 1 subunit theta
PPP1R13L seq: 186 - 235 + Phospho (ST); Phospho (Y)	38324 37149 [illegible]	p15220	RelA-associated inhibitor
RPL7A seq: 26 - 34 no mod	2E+07 5E+07 [illegible]	p12371	60S ribosomal protein L7a
CTTN pS261	2E+07 2E+07 [illegible]	p10980	Src substrate cortactin
FAM13A seq: 650 - 670 + Phospho (ST)	3E+07 37149 [illegible]	p03692	Protein FAM13A
VIM seq: 295 - 304 no mod	7E+07 1E+08 [illegible]	p16878	Vimentin
TLR7 seq: 116 - 121 no mod	38324 2E+08 [illegible]	p10990	Toll-like receptor 7
EIF4H seq: 211 - 216 no mod	38324 2E+08 [illegible]	p10978	Eukaryotic translation initiation factor 4H
C15orf52 seq: 198 - 214 + Phospho (ST)	38324 7E+07 [illegible]	p00684	Uncharacterized protein C15orf52
ALDH7A1 seq: 101 - 109 no mod	38324 1E+07 [illegible]	p13514	Alpha-aminoadipic semialdehyde dehydrogenase
UHRF1BP1 seq: 1101 - 1120 + 2 Phospho (ST)	7E+07 3E+07 [illegible]	p01894	UHRF1-binding protein 1
LRPPRC seq: 188 - 197 no mod	38324 2E+07 [illegible]	p10189	Leucine-rich PPR motif-containing protein, mitochondrial
MPP5 seq: 76 - 86 + Phospho (ST)	38324 37149 [illegible]	p12691	MAGUK p55 subfamily member 5
SND1 seq: 777 - 787 no mod	3E+06 1E+07 [illegible]	p11669	Staphylococcal nuclease domain-containing protein 1
BOLA1 pS81	38324 37149 [illegible]	p16796	BolA-like protein 1
LMO7 seq: 1491 - 1506 + Oxidation (M); Phospho (ST)	38324 37149 [illegible]	p06215	LIM domain only protein 7
PLEC seq: 2172 - 2182 + Gln->pyro-Glu (N-term Q)	1E+07 8E+06 [illegible]	p08331	Plectin
FUS seq: 275 - 306 no mod	38324 37149 [illegible]	p14863	RNA-binding protein FUS
LMNA seq: 209 - 216 no mod	7E+07 4E+07 [illegible]	p09526	Prelamin-A/C
EXPH5 seq: 1849 - 1867 + Phospho (ST)	3E+07 4E+07 [illegible]	p16499	Exophilin-5
SPAST pS268 pM274	38324 37149 [illegible]	p19872	Spastin
ADPRHL1 seq: 33 - 39 no mod	38324 37149 [illegible]	p13833	[Protein ADP-ribosylarginine] hydrolase-like protein 1
TCF20 pS1259	38324 1E+07 [illegible]	p05897	Transcription factor 20
HSPA1B seq: 77 - 88 no mod	3E+06 6E+06 [illegible]	p13248	Heat shock 70 kDa protein 1B
LAMTOR1 pS98	5E+06 2E+07 [illegible]	p12141	Ragulator complex protein LAMTOR1
TUFM seq: 406 - 411 no mod	38324 37149 [illegible]	p16557	Elongation factor Tu, mitochondrial
PHLDB2 seq: 511 - 536 + 2 Phospho (ST)	38324 37149 [illegible]	p13324	Pleckstrin homology-like domain family B member 2
FLNA pS968	1E+08 37149 [illegible]	p04667	Filamin-A
ABHD11 seq: 246 - 252 no mod	4E+06 3E+07 [illegible]	p14095	Alpha/beta hydrolase domain-containing protein 11
UCHL5 seq: 74 - 81 no mod	2E+06 6E+06 [illegible]	p12028	Ubiquitin carboxyl-terminal hydrolase isozyme L5
UBA1 seq: 527 - 537 no mod	3E+07 6E+07 [illegible]	p11491	Ubiquitin-like modifier-activating enzyme 1
PAK1 seq: 204 - 237 + Phospho (ST)	38324 37149 [illegible]	p03331	Serine/threonine-protein kinase PAK 1
PREX1 seq: 434 - 440 + Phospho (ST)	8E+06 4E+06 [illegible]	p12897	Phosphatidylinositol 3,4,5-trisphosphate-dependent Rac exchanger 1 protein
LMNA seq: 585 - 624 + Phospho (ST)	38324 37149 [illegible]	p01953	Prelamin-A/C
LMO7 seq: 1524 - 1556 + Phospho (ST)	5E+06 37149 [illegible]	p08109	LIM domain only protein 7
TRA2A seq: 180 - 188 no mod	1E+07 5E+06 [illegible]	p20053	Transformer-2 protein homolog alpha
AHNAK seq: 168 - 181 + Phospho (ST)	2E+07 37149 [illegible]	p17772	Neuroblast differentiation-associated protein AHNAK
SRGAP1 seq: 832 - 847 + Phospho (ST)	2E+07 2E+07 [illegible]	p18484	SLIT-ROBO Rho GTPase-activating protein 1
RPL13 seq: 124 - 129 no mod	1E+07 6E+07 [illegible]	p11579	60S ribosomal protein L13
GATSL3 seq: 30 - 35 no mod	1E+07 6E+07 [illegible]	p11398	GATS-like protein 3
PHLDB2 pT404	3E+07 7E+07 [illegible]	p08825	Pleckstrin homology-like domain family B member 2
CAPZA1 seq: 179 - 193 no mod	38324 4E+06 [illegible]	p16420	F-actin-capping protein subunit alpha-1
LMO7 pS895	2E+08 3E+08 [illegible]	p00877	LIM domain only protein 7
NCL seq: 578 - 597 no mod	2E+08 3E+08 [illegible]	p15649	Nucleolin
ARHGEF12 pS22	38324 7E+07 [illegible]	p14789	Rho guanine nucleotide exchange factor 12
MYH9 seq: 1924 - 1932 no mod	2E+06 9E+06 [illegible]	p16213	Myosin-9
CDR2 seq: 388 - 423 + Phospho (ST)	5E+07 37149 [illegible]	p18521	Cerebellar degeneration-related protein 2
TANC1 pS63	38324 2E+06 [illegible]	p14989	Protein TANC1
MYH14 pS1969	38324 37149 [illegible]	p07727	Myosin-14
DDX17 pS571	1E+08 7E+07 [illegible]	p02000	Probable ATP-dependent RNA helicase DDX17
NASP seq: 644 - 650 no mod	9E+06 2E+07 [illegible]	p17454	Nuclear autoantigenic sperm protein
ALOX15B seq: 8 - 21 no mod	5E+07 2E+07 [illegible]	p00764	Arachidonate 15-lipoxygenase B
SETD1A seq: 1167 - 1190 + Phospho (ST)	2E+08 3E+08 [illegible]	p01912	Histone-lysine N-methyltransferase SETD1A
SRRM1 pS549 pS551	2E+08 37149 [illegible]	p04388	Serine/arginine repetitive matrix protein 1
ALDH2 seq: 325 - 338 no mod	38324 4E+07 [illegible]	p02832	Aldehyde dehydrogenase, mitochondrial
TAF3 seq: 197 - 213 + Phospho (ST)	38324 37149 [illegible]	p11271	Transcription initiation factor TFIID subunit 3
G6PD seq: 199 - 205 no mod	3E+07 2E+07 [illegible]	p14423	Glucose-6-phosphate 1-dehydrogenase
DPP3 seq: 183 - 199 no mod	2E+07 2E+07 [illegible]	p12202	Dipeptidyl peptidase 3
GMFB seq: 111 - 119 no mod	2E+07 2E+07 [illegible]	p10365	Glia maturation factor beta
ANXA1 seq: 167 - 177 no mod	38324 37149 [illegible]	p18706	Annexin A1
EZR seq: 247 - 254 no mod	1E+08 9E+07 [illegible]	p09535	Ezrin
ANXA1 seq: 214 - 228 no mod	38324 37149 [illegible]	p13145	Annexin A1
PDCD6 seq: 161 - 166 no mod	6E+07 2E+07 [illegible]	p10573	Programmed cell death protein 6
SLC9A3R1 seq: 59 - 78 no mod	38324 9E+07 [illegible]	p10435	Na(+)/H(+) exchange regulatory cofactor NHE-RF1
TXLNA seq: 78 - 112 no mod	7E+07 8E+07 [illegible]	p08398	Alpha-taxilin
PHLDB2 seq: 290 - 315 + Phospho (ST)	3E+06 6E+06 [illegible]	p18563	Pleckstrin homology-like domain family B member 2
TJP2 pS130	1E+07 1E+07 [illegible]	p00916	Tight junction protein ZO-2
ENO1 seq: 282 - 306 no mod	38324 37149 [illegible]	p05900	Alpha-enolase
ETFB seq: 99 - 106 no mod	6E+06 6E+07 [illegible]	p11716	Electron transfer flavoprotein subunit beta
TNS3 pS1149	38324 37149 [illegible]	p19638	Tensin-3
MAGED2 seq: 77 - 100 no mod	3E+07 1E+07 [illegible]	p17930	Melanoma-associated antigen D2
TSTD1 seq: 94 - 101 no mod	2E+07 2E+07 [illegible]	p05203	Thiosulfate sulfurtransferase/rhodanese-like domain-containing protein 1
PLEC seq: 3506 - 3513 no mod	5E+06 5E+06 [illegible]	p00836	Plectin
KHSRP seq: 285 - 291 no mod	9E+06 3E+07 [illegible]	p14173	Far upstream element-binding protein 2
TBC1D5 seq: 535 - 549 + 2 Phospho (ST)	2E+07 37149 [illegible]	p09533	TBC1 domain family member 5
FKBP4 seq: 410 - 417 no mod	38324 6E+06 [illegible]	p10316	Peptidyl-prolyl cis-trans isomerase FKBP4
EHBP1L1 pS1257	38324 1E+07 [illegible]	p06833	EH domain-binding protein 1-like protein 1
TPD52L1 seq: 110 - 123 no mod	38324 5E+07 [illegible]	p19427	Tumor protein D53
CIAPIN1 seq: 42 - 48 no mod	1E+07 1E+07 [illegible]	p00760	Anamorsin
[illegible]: 1428 - 1476 + Gln->pyro-Glu (N-term Q); Phospho (ST)	1E+08 37149 [illegible]	p08060	Protein TANC1
BCLAF1 seq: 181 - 204 + Phospho (ST)	2E+07 37149 [illegible]	p05349	Bcl-2-associated transcription factor 1
TUBB2A seq: 253 - 262 no mod	38324 1E+07 [illegible]	p12139	Tubulin beta-2A chain
SP100 pS362	38324 37149 [illegible]	p06003	Nuclear autoantigen Sp-100
BCKDHA seq: 118 - 124 no mod	38324 1E+07 [illegible]	p13968	2-oxoisovalerate dehydrogenase subunit alpha, mitochondrial
RBM10 seq: 147 - 157 no mod	1E+07 2E+07 [illegible]	p15389	RNA-binding protein 10
ALDOC seq: 112 - 134 no mod	9E+07 1E+08 [illegible]	p14968	Fructose-bisphosphate aldolase C
VCP seq: 390 - 424 no mod	38324 7E+06 [illegible]	p12292	Transitional endoplasmic reticulum ATPase
HDGFRP3 seq: 11 - 18 no mod	9E+06 2E+07 [illegible]	p20701	Hepatoma-derived growth factor-related protein 3
RPL27 seq: 10 - 17 no mod	38324 3E+07 [illegible]	p00600	60S ribosomal protein L27
GOLGA2 seq: 271 - 280 no mod	5E+06 37149 [illegible]	p20183	Golgin subfamily A member 2
PTPN23 seq: 1113 - 1152 + Phospho (ST)	38324 37149 [illegible]	p16358	Tyrosine-protein phosphatase non-receptor type 23
NFIC pS339	38324 37149 [illegible]	p04672	Nuclear factor 1 C-type
PTK2 seq: 386 - 413 + Phospho (Y)	5E+07 37149 [illegible]	p02756	Focal adhesion kinase 1
ARHGAP21 seq: 609 - 619 + Phospho (ST)	9E+07 2E+08 [illegible]	p15843	Rho GTPase-activating protein 21
HNRNPM pM551	1E+08 1E+08 [illegible]	p10232	Heterogeneous nuclear ribonucleoprotein M
SFPQ seq: 366 - 376 no mod	38324 37149 [illegible]	p16831	Splicing factor, proline- and glutamine-rich
RPS19 seq: 44 - 56 no mod	38324 37149 [illegible]	p17537	40S ribosomal protein S19
SF1 seq: 261 - 271 no mod	8E+06 2E+07 [illegible]	p13997	Splicing factor 1
MAP1B seq: 1640 - 1664 + Oxidation (M); Phospho (ST)	38324 37149 [illegible]	p05999	Microtubule-associated protein 1B
MGA seq: 941 - 966 no mod	38324 37149 [illegible]	p09084	MAX gene-associated protein
MAP1B pS1501	2E+07 1E+07 [illegible]	p11808	Microtubule-associated protein 1B
MAP3K4 seq: 497 - 519 + Phospho (ST)	5E+07 3E+07 [illegible]	p11932	Mitogen-activated protein kinase kinase kinase 4
HSPA2 seq: 104 - 113 no mod	1E+08 2E+08 [illegible]	p00914	Heat shock-related 70 kDa protein 2
GGA2 seq: 90 - 96 no mod	38324 2E+07 [illegible]	p16601	ADP-ribosylation factor-binding protein GGA2
CALB2 seq: 40 - 50 no mod	38324 37149 [illegible]	p17503	Calretinin
EEF1A2 seq: 396 - 423 no mod	38324 37149 [illegible]	p05780	Elongation factor 1-alpha 2
BCAR3 seq: 178 - 193 + Phospho (ST)	3E+07 3E+07 [illegible]	p18250	Breast cancer anti-estrogen resistance protein 3
[illegible] seq: 654 - 676 + Gln->pyro-Glu (N-term Q); Phospho (ST)	38324 2E+07 [illegible]	p08473	C2 domain-containing protein 2-like
ENOX1 seq: 445 - 451 no mod	6E+06 1E+07 [illegible]	p09666	Ecto-NOX disulfide-thiol exchanger 1
CHPF seq: 597 - 603 no mod	1E+07 4E+07 [illegible]	p11241	Chondroitin sulfate synthase 2
CCT5 seq: 90 - 96 no mod	1E+07 4E+07 [illegible]	p11218	T-complex protein 1 subunit epsilon
ARPIN seq: 111 - 117 no mod	1E+07 2E+07 [illegible]	p10530	Arpin
IDH1 seq: 322 - 338 no mod	38324 5E+06 [illegible]	p15401	Isocitrate dehydrogenase [NADP] cytoplasmic
CCT4 seq: 90 - 96 no mod	1E+07 4E+07 [illegible]	p09992	T-complex protein 1 subunit delta
BCLAF1 seq: 225 - 255 + 2 Phospho (ST)	38324 37149 [illegible]	p04590	Bcl-2-associated transcription factor 1
CHMP5 seq: 38 - 44 no mod	2E+07 37149 [illegible]	p12098	Charged multivesicular body protein 5
CTPS1 seq: 565 - 591 + 3 Phospho (ST)	38324 37149 [illegible]	p18139	CTP synthase 1
PTPN14 pS809	2E+07 37149 [illegible]	p02954	Tyrosine-protein phosphatase non-receptor type 14
ASS1 seq: 158 - 165 no mod	5E+08 4E+08 [illegible]	p09704	Argininosuccinate synthase
FASN seq: 1044 - 1051 no mod	2E+07 4E+07 [illegible]	p14802	Fatty acid synthase
FGD6 seq: 414 - 448 + Phospho (ST)	38324 1E+07 [illegible]	p10253	FYVE, RhoGEF and PH domain-containing protein 6
CAMSAP1 seq: 1041 - 1061 + Phospho (ST)	38324 37149 [illegible]	p13675	Calmodulin-regulated spectrin-associated protein 1
STRAP seq: 60 - 69 no mod	7E+06 2E+07 [illegible]	p16250	Serine-threonine kinase receptor-associated protein
MSI2 seq: 63 - 77 no mod	38324 37149 [illegible]	p16122	RNA-binding protein Musashi homolog 2
RGS14 pS478	38324 37149 [illegible]	p19370	Regulator of G-protein signaling 14
BLVRB pM150	38324 37149 [illegible]	p00037	Flavin reductase (NADPH)
CFL1 seq: 35 - 45 no mod	4E+06 4E+07 [illegible]	p19228	Cofilin-1
DCAF6 seq: 648 - 668 + 2 Phospho (ST)	2E+08 5E+07 [illegible]	p18306	DDB1- and CUL4-associated factor 6
RPL23 seq: 16 - 35 no mod	38324 3E+07 [illegible]	p13716	60S ribosomal protein L23
FEN1 seq: 255 - 261 no mod	38324 7E+06 [illegible]	p05532	Flap endonuclease 1
VPS33A seq: 510 - 516 no mod	38324 7E+06 [illegible]	p05531	Vacuolar protein sorting-associated protein 33A
SRP72 seq: 302 - 310 no mod	1E+07 2E+07 [illegible]	p08335	Signal recognition particle subunit SRP72
HNRNPR seq: 587 - 633 + Gln->pyro-Glu (N-term Q)	38324 37149 [illegible]	p07792	Heterogeneous nuclear ribonucleoprotein R
BUB3 seq: 140 - 149 no mod	38324 2E+07 [illegible]	p00518	Mitotic checkpoint protein BUB3
UBE2L3 seq: 74 - 82 no mod	2E+08 2E+08 [illegible]	p13498	Ubiquitin-conjugating enzyme E2 L3
MCM2 seq: 83 - 115 + Phospho (ST)	38324 5E+06 [illegible]	p20446	DNA replication licensing factor MCM2
FLNA seq: 1360 - 1375 no mod	4E+06 3E+06 [illegible]	p01421	Filamin-A
ENO1 seq: 65 - 71 no mod	38324 37149 [illegible]	p19255	Alpha-enolase
AHNAK pS5077	618004 37149 [illegible]	p01649	Neuroblast differentiation-associated protein AHNAK
NFIC seq: 487 - 494 + Phospho (ST)	6E+06 5E+06 [illegible]	p05803	Nuclear factor 1 C-type
RPL34 seq: 11 - 19 no mod	2E+08 4E+08 [illegible]	p10591	60S ribosomal protein L34
NASP seq: 94 - 100 no mod	8E+06 2E+07 [illegible]	p05165	Nuclear autoantigenic sperm protein
CLTC seq: 1023 - 1034 no mod	38324 1E+07 [illegible]	p09421	Clathrin heavy chain 1
PLEC seq: 1596 - 1603 no mod	3E+07 37149 [illegible]	p10984	Plectin
CAPZA2 seq: 122 - 129 no mod	1E+07 37149 [illegible]	p02067	F-actin-capping protein subunit alpha-2
ACADSB seq: 160 - 170 no mod	38324 3E+07 [illegible]	p19284	Short/branched chain specific acyl-CoA dehydrogenase, mitochondrial
CEP170 pS1112	38324 3E+07 [illegible]	p11805	Centrosomal protein of 170 kDa
SLC9A3R1 seq: 211 - 220 no mod	7E+07 9E+07 [illegible]	p08569	Na(+)/H(+) exchange regulatory cofactor NHE-RF1
DAPP1 seq: 274 - 280 + Phospho (ST)	6E+06 37149 [illegible]	p04060	Dual adapter for phosphotyrosine and 3-phosphotyrosine and 3-phosphoinositide
USP5 seq: 361 - 379 no mod	2E+07 2E+07 [illegible]	p14296	Ubiquitin carboxyl-terminal hydrolase 5
NCL seq: 117 - 125 no mod	3E+07 7E+07 [illegible]	p20121	Nucleolin
AHNAK seq: 5822 - 5831 no mod	7E+06 4E+07 [illegible]	p16729	Neuroblast differentiation-associated protein AHNAK
DDX1 seq: 320 - 330 no mod	2E+06 9E+06 [illegible]	p17459	ATP-dependent RNA helicase DDX1
UBE3D seq: 10 - 15 no mod	2E+07 37149 [illegible]	p01544	E3 ubiquitin-protein ligase E3D

Peptide	Values	Accession	Protein
BCLAF1 seq: 347 - 352 no mod	[illegible]	p01543	Bcl-2-associated transcription factor 1
GORASP2 seq: 429 - 452 + 2 Phospho (ST)	[illegible]	p01513	Golgi reassembly-stacking protein 2
CORO1C seq: 20 - 28 no mod	[illegible]	p09699	Coronin-1C
PWP1 pS50	[illegible]	p11018	Periodic tryptophan protein 1 homolog
SF3B1 seq: 51 - 80 no mod	[illegible]	p16866	Splicing factor 3B subunit 1
ARHGDIB seq: 64 - 71 no mod	[illegible]	p19960	Rho GDP-dissociation inhibitor 2
CCDC86 pS91	[illegible]	p10950	Coiled-coil domain-containing protein 86
IDH1 seq: 382 - 388 no mod	[illegible]	p15676	Isocitrate dehydrogenase [NADP] cytoplasmic
RPS9 seq: 84 - 91 no mod	[illegible]	p14220	40S ribosomal protein S9
PLEC seq: 4402 - 4428 + Phospho (ST)	[illegible]	p02281	Plectin
SAP18 seq: 68 - 74 no mod	[illegible]	p17389	Histone deacetylase complex subunit SAP18
SPAST seq: 235 - 248 + Phospho (ST)	[illegible]	p07233	Spastin
FLNB seq: 734 - 744 no mod	[illegible]	p01074	Filamin-B
RPS18 seq: 67 - 75 no mod	[illegible]	p01339	40S ribosomal protein S18
EFTUD2 seq: 722 - 730 no mod	[illegible]	p00420	116 kDa U5 small nuclear ribonucleoprotein component
TBCA seq: 62 - 69 no mod	[illegible]	p09988	Tubulin-specific chaperone A
SF3A1 seq: 211 - 217 no mod	[illegible]	p10436	Splicing factor 3A subunit 1
PPP1R13L pS183 pS187	[illegible]	p20563	RelA-associated inhibitor
DBI seq: 54 - 63 no mod	[illegible]	p20343	Acyl-CoA-binding protein
HMGB1 seq: 155 - 163 no mod	[illegible]	p00411	High mobility group protein B1
CHAMP1 seq: 648 - 667 + 2 Phospho (ST)	[illegible]	p15403	Chromosome alignment-maintaining phosphoprotein 1
CTBP2 seq: 156 - 163 no mod	[illegible]	p00922	C-terminal-binding protein 2
HSPA8 seq: 78 - 88 no mod	[illegible]	p16818	Heat shock cognate 71 kDa protein
SDCCAG8 pS100	[illegible]	p12854	Serologically defined colon cancer antigen 8
SNRNP70 seq: 174 - 180 no mod	[illegible]	p01124	U1 small nuclear ribonucleoprotein 70 kDa
PCK2 seq: 32 - 45 no mod	[illegible]	p01178	Phosphoenolpyruvate carboxykinase [GTP], mitochondrial
SOX13 seq: 331 - 352 + Phospho (ST)	[illegible]	p18545	Transcription factor SOX-13
HS1BP3 pS194	[illegible]	p16181	HCLS1-binding protein 3
NACA seq: 1977 - 1990 no mod	[illegible]	p04712	Nascent polypeptide-associated complex subunit alpha, muscle-specific form
UGDH seq: 68 - 80 no mod	[illegible]	p09466	UDP-glucose 6-dehydrogenase
SUCLG1 seq: 82 - 90 no mod	[illegible]	p10429	Succinyl-CoA ligase [ADP/GDP-forming] subunit alpha, mitochondrial
BCLAF1 seq: 551 - 567 no mod	[illegible]	p10248	Bcl-2-associated transcription factor 1
PLIN3 seq: 332 - 356 no mod	[illegible]	p18771	Perilipin-3
KCNH5 seq: 934 - 940 + Gln->pyro-Glu (N-term Q)	[illegible]	p07754	Potassium voltage-gated channel subfamily H member 5
seq: 554 - 576 + Gln->pyro-Glu (N-term Q); Phospho (ST)	[illegible]	p08647	Cleavage stimulation factor subunit 2 tau variant
PSMD10 seq: 207 - 213 no mod	[illegible]	p02376	26S proteasome non-ATPase regulatory subunit 10
VASP seq: 11 - 22 no mod	[illegible]	p19294	Vasodilator-stimulated phosphoprotein
PGD seq: 88 - 107 no mod	[illegible]	p10381	6-phosphogluconate dehydrogenase, decarboxylating
STIP1 seq: 306 - 315 no mod	[illegible]	p14250	Stress-induced-phosphoprotein 1
P4HB seq: 70 - 78 no mod	[illegible]	p11533	Protein disulfide-isomerase
HAS1 seq: 194 - 201 no mod	[illegible]	p12144	Hyaluronan synthase 1
CCT2 seq: 182 - 189 no mod	[illegible]	p12143	T-complex protein 1 subunit beta
CLIP1 pS1364	[illegible]	p01615	CAP-Gly domain-containing linker protein 1
HSPA9 seq: 349 - 360 no mod	[illegible]	p19816	Stress-70 protein, mitochondrial
FAM65A seq: 345 - 359 + 2 Phospho (ST)	[illegible]	p16439	Protein FAM65A
RPS10P5 seq: 151 - 164 no mod	[illegible]	p20873	Putative 40S ribosomal protein S10-like
MOCS1 seq: 117 - 123 no mod	[illegible]	p17739	Molybdenum cofactor biosynthesis protein 1
RPS15 seq: 45 - 51 no mod	[illegible]	p11165	40S ribosomal protein S15
LRPAP1 seq: 114 - 121 no mod	[illegible]	p09429	Alpha-2-macroglobulin receptor-associated protein
BCL9L seq: 114 - 125 + Phospho (ST)	[illegible]	p18321	B-cell CLL/lymphoma 9-like protein
TCEB2 seq: 30 - 36 no mod	[illegible]	p13560	Transcription elongation factor B polypeptide 2
DUT seq: 217 - 224 no mod	[illegible]	p14483	Deoxyuridine 5'-triphosphate nucleotidohydrolase, mitochondrial
RPS6 seq: 233 - 243 + 3 Phospho (ST)	[illegible]	p07083	40S ribosomal protein S6
COPB2 seq: 319 - 328 no mod	[illegible]	p14649	Coatomer subunit beta'
PSME3 seq: 219 - 225 no mod	[illegible]	p11587	Proteasome activator complex subunit 3
TXN seq: 73 - 81 no mod	[illegible]	p19036	Thioredoxin
KRT83 seq: 200 - 209 no mod	[illegible]	p12275	Keratin, type II cuticular Hb3
BRD4 seq: 463 - 471 no mod	[illegible]	p12274	Bromodomain-containing protein 4
PLEKHG3 seq: 1079 - 1094 + Phospho (ST)	[illegible]	p05553	Pleckstrin homology domain-containing family G member 3
VIM seq: 322 - 342 no mod	[illegible]	p07701	Vimentin
PCSK1N seq: 202 - 216 no mod	[illegible]	p14094	ProSAAS
HSP90AA1 seq: 694 - 727 no mod	[illegible]	p11750	Heat shock protein HSP 90-alpha
DBN1 seq: 105 - 132 no mod	[illegible]	p01822	Drebrin
UBA1 seq: 289 - 296 no mod	[illegible]	p16037	Ubiquitin-like modifier-activating enzyme 1
TUBB3 seq: 283 - 297 + Oxidation (M)	[illegible]	p20127	Tubulin beta-3 chain
TUBB2B seq: 283 - 297 no mod	[illegible]	p20126	Tubulin beta-2B chain
USP5 seq: 517 - 524 no mod	[illegible]	p10266	Ubiquitin carboxyl-terminal hydrolase 5
PHLDB2 seq: 561 - 573 + Phospho (ST)	[illegible]	p19506	Pleckstrin homology-like domain family B member 2
FLNA seq: 1815 - 1831 no mod	[illegible]	p01770	Filamin-A
EZR seq: 578 - 586 no mod	[illegible]	p07979	Ezrin
RPS2 seq: 201 - 211 no mod	[illegible]	p15094	40S ribosomal protein S2
FAM114A1 seq: 277 - 285 no mod	[illegible]	p12198	Protein NOXP20
LUC7L2 seq: 54 - 61 no mod	[illegible]	p10213	Putative RNA-binding protein Luc7-like 2
LTA4H seq: 65 - 73 no mod	[illegible]	p00614	Leukotriene A-4 hydrolase
XRCC6 seq: 116 - 123 no mod	[illegible]	p14060	X-ray repair cross-complementing protein 6
SMC1A seq: 6 - 13 no mod	[illegible]	p11605	Structural maintenance of chromosomes protein 1A
VCL seq: 803 - 815 no mod	[illegible]	p19279	Vinculin
PKP3 seq: 308 - 326 + Phospho (ST)	[illegible]	p02573	Plakophilin-3
EIF5A2 pM43	[illegible]	p13557	Eukaryotic translation initiation factor 5A-2
RPS5 seq: 137 - 146 no mod	[illegible]	p08797	40S ribosomal protein S5
EIF4H seq: 11 - 19 no mod	[illegible]	p19100	Eukaryotic translation initiation factor 4H
NAPG seq: 6 - 15 no mod	[illegible]	p13930	Gamma-soluble NSF attachment protein
SPAG9 pT217	[illegible]	p17219	C-Jun-amino-terminal kinase-interacting protein 4
VCP seq: 600 - 614 no mod	[illegible]	p01366	Transitional endoplasmic reticulum ATPase
SH2B1 seq: 83 - 112 + Phospho (ST)	[illegible]	p19601	SH2B adapter protein 1
PLEC seq: 3270 - 3285 no mod	[illegible]	p11460	Plectin
FLNA seq: 64 - 76 no mod	[illegible]	p14493	Filamin-A
PPA1 seq: 10 - 18 no mod	[illegible]	p21076	Inorganic pyrophosphatase
SRI seq: 168 - 174 no mod	[illegible]	p20136	Sorcin
PTBP1 seq: 472 - 482 no mod	[illegible]	p01233	Polypyrimidine tract-binding protein 1
PCNA seq: 241 - 248 no mod	[illegible]	p14525	Proliferating cell nuclear antigen
ARHGAP29 seq: 532 - 551 + Phospho (ST)	[illegible]	p03178	Rho GTPase-activating protein 29
PRDX1 seq: 141 - 151 no mod	[illegible]	p08483	Peroxiredoxin-1
PDCD6IP seq: 607 - 614 no mod	[illegible]	p00531	Programmed cell death 6-interacting protein
OLA1 seq: 25 - 35 no mod	[illegible]	p14257	Obg-like ATPase 1
seq: 257 - 279 + Gln->pyro-Glu (N-term Q); Phospho (ST)	[illegible]	p08118	WAS/WASL-interacting protein family member 2
USP11 seq: 98 - 107 no mod	[illegible]	p20680	Ubiquitin carboxyl-terminal hydrolase 11
SDCBP seq: 204 - 214 no mod	[illegible]	p18204	Syntenin-1
PDIA3 seq: 483 - 496 no mod	[illegible]	p17913	Protein disulfide-isomerase A3
PSMA6 seq: 12 - 21 no mod	[illegible]	p14763	Proteasome subunit alpha type-6
RPS2 seq: 247 - 257 no mod	[illegible]	p01860	40S ribosomal protein S2
PRDX5 seq: 181 - 191 no mod	[illegible]	p16457	Peroxiredoxin-5, mitochondrial
DLD seq: 110 - 117 no mod	[illegible]	p15903	Dihydrolipoyl dehydrogenase, mitochondrial
MARCKSL1 seq: 100 - 106 no mod	[illegible]	p10791	MARCKS-related protein
PDYN seq: 98 - 104 no mod	[illegible]	p10782	Proenkephalin-B
RPS13 seq: 115 - 121 no mod	[illegible]	p11578	40S ribosomal protein S13
ATG4B pS383	[illegible]	p16786	Cysteine protease ATG4B
PEBP1 seq: 8 - 39 no mod	[illegible]	p00476	Phosphatidylethanolamine-binding protein 1
PLEC seq: 3447 - 3462 no mod	[illegible]	p19859	Plectin
SLC32A1 seq: 299 - 304 no mod	[illegible]	p16364	Vesicular inhibitory amino acid transporter
PURB seq: 73 - 78 no mod	[illegible]	p16363	Transcriptional activator protein Pur-beta
H2AFZ seq: 93 - 102 no mod	[illegible]	p16227	Histone H2A.Z
VCL seq: 571 - 581 no mod	[illegible]	p20314	Vinculin
PRDX4 seq: 213 - 223 no mod	[illegible]	p08492	Peroxiredoxin-4
HSPB8 seq: 79 - 86 no mod	[illegible]	p16725	Heat shock protein beta-8
EML6 seq: 1713 - 1719 no mod	[illegible]	p13513	Echinoderm microtubule-associated protein-like 6
EML5 seq: 1720 - 1726 no mod	[illegible]	p10329	Echinoderm microtubule-associated protein-like 5
HTATIP2 seq: 99 - 106 no mod	[illegible]	p20727	Oxidoreductase HTATIP2
MB seq: 18 - 32 no mod	[illegible]	p01648	Myoglobin
TXNRD1 seq: 400 - 416 no mod	[illegible]	p08668	Thioredoxin reductase 1, cytoplasmic
KHSRP seq: 332 - 340 no mod	[illegible]	p14595	Far upstream element-binding protein 2
NCL seq: 72 - 80 no mod	[illegible]	p01737	Nucleolin
CCT3 seq: 390 - 399 no mod	[illegible]	p09423	T-complex protein 1 subunit gamma
EEF2 seq: 768 - 785 no mod	[illegible]	p15922	Elongation factor 2
TALDO1 seq: 246 - 258 no mod	[illegible]	p20329	Transaldolase
AHNAK seq: 4916 - 4926 no mod	[illegible]	p00752	Neuroblast differentiation-associated protein AHNAK
PHGDH seq: 365 - 380 no mod	[illegible]	p09778	D-3-phosphoglycerate dehydrogenase
NCL seq: 487 - 508 no mod	[illegible]	p02510	Nucleolin
GGCT seq: 172 - 181 no mod	[illegible]	p00872	Gamma-glutamylcyclotransferase
MYH9 seq: 712 - 718 no mod	[illegible]	p00626	Myosin-9
UBE2I seq: 62 - 74 no mod	[illegible]	p10031	SUMO-conjugating enzyme UBC9
TCOF1 seq: 843 - 852 no mod	[illegible]	p15531	Treacle protein
HNRNPAB seq: 71 - 83 no mod	[illegible]	p10141	Heterogeneous nuclear ribonucleoprotein A/B
ZFYVE9 seq: 161 - 190 no mod	[illegible]	p02576	Zinc finger FYVE domain-containing protein 9
CTSD seq: 185 - 194 no mod	[illegible]	p08135	Cathepsin D
DNAJC8 seq: 119 - 127 no mod	[illegible]	p20298	DnaJ homolog subfamily C member 8
RPL12 seq: 41 - 48 no mod	[illegible]	p12430	60S ribosomal protein L12
PLIN3 seq: 99 - 116 no mod	[illegible]	p15769	Perilipin-3
TPD52 seq: 82 - 100 no mod	[illegible]	p17540	Tumor protein D52
NDUFA5 seq: 31 - 40 no mod	[illegible]	p14074	NADH dehydrogenase [ubiquinone] 1 alpha subcomplex subunit 5
API5 seq: 2 - 9 no mod	[illegible]	p08881	Apoptosis inhibitor 5
RPL13 seq: 146 - 158 no mod	[illegible]	p12153	60S ribosomal protein L13
FLNB seq: 37 - 49 no mod	[illegible]	p14251	Filamin-B
HADHB seq: 349 - 361 no mod	[illegible]	p18372	Trifunctional enzyme subunit beta, mitochondrial
CORO1B seq: 234 - 241 no mod	[illegible]	p01524	Coronin-1B
FLNB seq: 811 - 818 no mod	[illegible]	p00017	Filamin-B
CCT7 seq: 78 - 84 no mod	[illegible]	p02513	T-complex protein 1 subunit eta
EEF2 seq: 429 - 439 no mod	[illegible]	p13959	Elongation factor 2
LMNB2 seq: 417 - 430 no mod	[illegible]	p19304	Lamin-B2
CTSD seq: 288 - 309 no mod	[illegible]	p17771	Cathepsin D
HSPA5 seq: 465 - 474 no mod	[illegible]	p00542	78 kDa glucose-regulated protein
PLEC seq: 2833 - 2841 no mod	[illegible]	p03872	Plectin
TKT seq: 543 - 550 no mod	[illegible]	p12967	Transketolase
KRT74 seq: 189 - 197 no mod	[illegible]	p16840	Keratin, type II cytoskeletal 74
CTSD seq: 404 - 412 no mod	[illegible]	p01493	Cathepsin D
SHB seq: 293 - 319 + Phospho (ST)	[illegible]	p15862	SH2 domain-containing adapter protein B
ILF3 seq: 333 - 342 no mod	[illegible]	p01231	Interleukin enhancer-binding factor 3
CCT6A seq: 142 - 153 no mod	[illegible]	p17368	T-complex protein 1 subunit zeta
DNAH9 seq: 1148 - 1154 no mod	[illegible]	p07804	Dynein heavy chain 9, axonemal
VBP1 seq: 83 - 89 no mod	[illegible]	p07801	Prefoldin subunit 3
PPP1CA seq: 16 - 26 no mod	[illegible]	p11401	Serine/threonine-protein phosphatase PP1-alpha catalytic subunit
PALLD seq: 875 - 883 no mod	[illegible]	p14976	Palladin
HNRNPA0 seq: 177 - 189 no mod	[illegible]	p17881	Heterogeneous nuclear ribonucleoprotein A0
TUBB seq: 163 - 174 no mod	[illegible]	p13947	Tubulin beta chain
RPS11 seq: 49 - 59 no mod	[illegible]	p17958	40S ribosomal protein S11
G6PD seq: 488 - 498 no mod	[illegible]	p15424	Glucose-6-phosphate 1-dehydrogenase
NONO seq: 457 - 468 no mod	[illegible]	p21075	Non-POU domain-containing octamer-binding protein
HNRNPUL2 seq: 481 - 494 no mod	[illegible]	p00197	Heterogeneous nuclear ribonucleoprotein U-like protein 2
HNRNPM seq: 486 - 496 no mod	[illegible]	p10137	Heterogeneous nuclear ribonucleoprotein M
CSDE1 seq: 759 - 769 + Phospho (ST)	[illegible]	p09531	Cold shock domain-containing protein E1
TRIM28 seq: 408 - 427 no mod	[illegible]	p13571	Transcription intermediary factor 1-beta
SF3B3 seq: 604 - 615 no mod	[illegible]	p16635	Splicing factor 3B subunit 3
GSR seq: 369 - 379 no mod	[illegible]	p15832	Glutathione reductase, mitochondrial
RPL5 seq: 222 - 228 no mod	[illegible]	p08669	60S ribosomal protein L5
FLNB seq: 481 - 492 no mod	[illegible]	p21137	Filamin-B
RPS9 seq: 31 - 38 no mod	[illegible]	p11599	40S ribosomal protein S9
RPS3 seq: 188 - 197 no mod	[illegible]	p13958	40S ribosomal protein S3
TAF15 seq: 222 - 255 + Phospho (ST)	[illegible]	p02980	TATA-binding protein-associated factor 2N
DDAH1 seq: 137 - 144 no mod	[illegible]	p17802	N(G),N(G)-dimethylarginine dimethylaminohydrolase 1
STIP1 pM535	[illegible]	p11239	Stress-induced-phosphoprotein 1
ATP5B seq: 265 - 279 no mod	[illegible]	p01791	ATP synthase subunit beta, mitochondrial
NUMA1 seq: 1271 - 1282 no mod	[illegible]	p11321	Nuclear mitotic apparatus protein 1
KRT8 seq: 199 - 213 no mod	[illegible]	p18123	Keratin, type II cytoskeletal 8
GALNS seq: 2 - 9 no mod	[illegible]	p20973	N-acetylgalactosamine-6-sulfatase
FAM167B seq: 34 - 39 no mod	[illegible]	p10975	Protein FAM167B
TACC2 seq: 2697 - 2703 no mod	[illegible]	p08554	Transforming acidic coiled-coil-containing protein 2
IRGQ seq: 533 - 544 no mod	[illegible]	p20259	Immunity-related GTPase family Q protein
COPB1 seq: 328 - 337 no mod	[illegible]	p01147	Coatomer subunit beta
ILF3 seq: 578 - 587 no mod	[illegible]	p19131	Interleukin enhancer-binding factor 3
IDH1 seq: 21 - 27 no mod	[illegible]	p14123	Isocitrate dehydrogenase [NADP] cytoplasmic
USP5 seq: 463 - 468 no mod	[illegible]	p16579	Ubiquitin carboxyl-terminal hydrolase 5
PGD seq: 155 - 163 no mod	[illegible]	p02715	6-phosphogluconate dehydrogenase, decarboxylating
SIPA1L3 seq: 1156 - 1171 + Phospho (ST)	[illegible]	p08103	Signal-induced proliferation-associated 1-like protein 3
LIMA1 seq: 501 - 511 no mod	[illegible]	p01429	LIM domain and actin-binding protein 1
HNRNPM seq: 551 - 557 no mod	[illegible]	p10233	Heterogeneous nuclear ribonucleoprotein M
YWHAG seq: 199 - 217 no mod	[illegible]	p03097	14-3-3 protein gamma
RPL8 seq: 175 - 181 no mod	[illegible]	p14409	60S ribosomal protein L8
DLST seq: 431 - 437 no mod	[illegible]	p17901	Dihydrolipoyllysine-residue succinyltransferase component of 2-oxoglutarate dehydrogenase complex, mitochondrial
MSN seq: 372 - 379 no mod	[illegible]	p20277	Moesin
EZR seq: 372 - 379 no mod	[illegible]	p20190	Ezrin
PHLDB2 seq: 172 - 195 + Phospho (ST)	[illegible]	p19603	Pleckstrin homology-like domain family B member 2
USP5 seq: 239 - 247 no mod	[illegible]	p17063	Ubiquitin carboxyl-terminal hydrolase 5
CCT2 seq: 157 - 170 no mod	[illegible]	p08763	T-complex protein 1 subunit beta
AHCY seq: 336 - 343 no mod	[illegible]	p14159	Adenosylhomocysteinase
CPNE1 seq: 158 - 166 no mod	[illegible]	p06093	Copine-1
PSMB3 seq: 100 - 115 no mod	[illegible]	p16743	Proteasome subunit beta type-3
SRSF1 seq: 123 - 138 no mod	[illegible]	p00562	Serine/arginine-rich splicing factor 1
CTNNA1 seq: 671 - 681 no mod	[illegible]	p20453	Catenin alpha-1
SULT1A4 seq: 207 - 213 no mod	[illegible]	p14068	Sulfotransferase 1A4
COX5B seq: 122 - 129 no mod	[illegible]	p10379	Cytochrome c oxidase subunit 5B, mitochondrial

Peptide	Intensity values	Accession	Protein
CCT5 seq: 28 - 35 no mod	5E+06 3E+07 3E+07 13654 33854 2E+08 25552 2E+07 34104 26736 6E+08 53206 3E+07 40879 18798 2E+07 3E+06 5E+06 3E+07 28832 39759 50401 4E+06 19024 42741 68554 44396 [illegible]	p11234	T-complex protein 1 subunit epsilon
C21orf33 seq: 142 - 151 no mod	1E+07 4E+07 3E+07 4E+06 33854 45779 25552 2E+07 34104 26736 2E+08 53206 5E+07 40879 18798 2E+07 1E+07 6E+06 2E+07 2E+06 1E+07 50401 21442 19024 5E+06 68554 44396 [illegible]	p09399	ES1 protein homolog, mitochondrial
ATIC seq: 398 - 406 no mod	1E+07 2E+07 5E+07 5E+06 2E+07 45779 4E+06 2E+07 34104 6E+06 56234 7E+06 3E+07 4E+06 5E+06 40867 5E+06 5E+06 19162 28832 39759 50401 21442 19024 6E+06 68554 44396 [illegible]	p05237	Bifunctional purine biosynthesis protein PURH
MANF seq: 124 - 133 no mod	38324 1E+07 2E+07 13654 33854 2E+08 25552 1E+07 34104 26736 2E+08 53206 2E+07 40879 18798 40867 33072 34984 1E+07 28832 39759 50401 21442 19024 42741 68554 44396 [illegible]	p08536	Mesencephalic astrocyte-derived neurotrophic factor
AHNAK seq: 3415 - 3425 no mod	38324 2E+07 8E+06 13654 33854 3E+08 25552 34892 34104 26736 5E+08 53206 8E+06 40879 18798 40867 33072 34984 19162 28832 39759 3E+06 21442 19024 42741 68554 44396 [illegible]	p00821	Neuroblast differentiation-associated protein AHNAK
GPI seq: 67 - 73 no mod	9E+06 37149 39656 13654 33854 9E+07 25552 34892 34104 26736 6E+08 53206 39754 40879 18798 40867 33072 34984 19162 28832 39759 50401 21442 19024 42741 68554 44396 [illegible]	p09993	Glucose-6-phosphate isomerase
TCP1 seq: 516 - 526 no mod	2E+08 1E+08 7E+07 6E+06 4E+07 1E+08 1E+08 4E+07 1E+07 26736 4E+08 1E+08 1E+08 8E+06 18798 40867 5E+07 7E+06 4E+07 28832 39759 50401 1E+07 2E+07 42741 68554 44396 [illegible]	p16832	T-complex protein 1 subunit alpha
UBE2K seq: 56 - 61 no mod	2E+07 3E+07 4E+07 6E+06 2E+07 45779 8E+06 3E+07 1E+07 26736 56234 53206 5E+07 8E+06 5E+06 3E+06 33072 6E+06 4E+07 28832 39759 3E+06 8E+06 19024 5E+06 68554 44396 [illegible]	p00159	Ubiquitin-conjugating enzyme E2 K
PAFAH1B1 seq: 47 - 53 no mod	2E+07 3E+07 4E+07 6E+06 2E+07 45779 8E+06 3E+07 1E+07 26736 56234 53206 5E+07 8E+06 5E+06 3E+06 33072 6E+06 4E+07 28832 39759 3E+06 8E+06 19024 5E+06 68554 44396 [illegible]	p00452	Platelet-activating factor acetylhydrolase IB subunit alpha
ANKRD18B seq: 355 - 360 no mod	2E+07 3E+07 4E+07 6E+06 2E+07 45779 8E+06 3E+07 1E+07 26736 56234 53206 5E+07 8E+06 5E+06 3E+06 33072 6E+06 4E+07 28832 39759 3E+06 8E+06 19024 5E+06 68554 44396 [illegible]	p00301	Ankyrin repeat domain-containing protein 18B
OFD1 seq: 353 - 358 no mod	2E+07 3E+07 4E+07 6E+06 2E+07 45779 8E+06 3E+07 1E+07 26736 56234 53206 5E+07 8E+06 5E+06 3E+06 33072 6E+06 4E+07 28832 39759 3E+06 8E+06 19024 5E+06 68554 44396 [illegible]	p00158	Oral-facial-digital syndrome 1 protein
EEF1A1P5 pM404	6E+07 8E+07 2E+08 13654 33854 9E+07 3E+07 1E+08 6E+07 26736 6E+08 53206 2E+08 4E+07 18798 1E+08 33072 34984 2E+08 28832 39759 2E+07 21442 1E+08 42741 68554 44396 [illegible]	p05781	Putative elongation factor 1-alpha-like 3
MAGED1 pS166	2E+08 2E+08 4E+08 4E+08 7E+08 7E+08 3E+08 3E+08 3E+08 3E+08 6E+08 3E+08 3E+08 4E+08 5E+08 40867 9E+07 3E+08 2E+08 3E+08 2E+08 1E+08 2E+08 19024 2E+08 68554 44396 [illegible]	p01459	Melanoma-associated antigen D1
HSPA4 seq: 698 - 704 no mod	5E+07 7E+07 7E+07 13654 4E+07 2E+08 25552 5E+07 2E+07 26736 8E+08 3E+07 1E+08 2E+07 18798 7E+07 2E+07 34984 6E+07 28832 39759 1E+07 21442 19024 2E+07 68554 44396 [illegible]	p11900	Heat shock 70 kDa protein 4
ADIRF pM43	3E+08 3E+08 5E+08 7E+07 3E+08 5E+08 4E+07 1E+08 2E+08 3E+08 3E+09 5E+07 4E+08 1E+08 4E+08 3E+08 1E+07 5E+07 2E+08 3E+08 2E+08 3E+07 5E+07 5E+07 5E+08 1E+07 44396 [illegible]	p20096	Adipogenesis regulatory factor
RBL2 seq: 399 - 407 + Phospho (ST)	38324 3E+07 39656 13654 33854 45779 25552 34892 34104 26736 56234 53206 4E+07 40879 7E+06 40867 33072 34984 19162 6E+06 39759 50401 21442 19024 1E+07 68554 44396 [illegible]	p13642	Retinoblastoma-like protein 2
MADD seq: 856 - 871 + Phospho (ST)	2E+08 3E+08 7E+07 1E+08 2E+08 3E+08 5E+08 9E+07 1E+08 2E+08 1E+08 53206 39754 5E+07 2E+08 8E+07 33072 8E+07 3E+08 2E+08 2E+08 4E+07 6E+07 9E+07 2E+08 68554 44396 [illegible]	p09577	MAP kinase-activating death domain protein
AKR1A1 seq: 128 - 141 no mod	5E+07 4E+07 4E+07 1E+07 3E+07 9E+07 2E+07 4E+07 3E+07 6E+06 4E+08 3E+07 6E+07 2E+07 3E+06 2E+07 3E+07 2E+07 5E+07 2E+06 3E+07 2E+07 1E+07 2E+07 3E+06 68554 44396 [illegible]	p09794	Alcohol dehydrogenase [NADP(+)]
DNAJB1 seq: 332 - 340 no mod	38324 2E+07 4E+07 13654 1E+07 6E+07 2E+06 2E+07 4E+06 3E+06 1E+08 53206 3E+07 40879 18798 1E+07 33072 34984 19162 28832 4E+06 6E+06 4E+06 7E+06 42741 68554 44396 [illegible]	p01949	DnaJ homolog subfamily B member 1
CRIP2 seq: 187 - 205 no mod	1E+08 2E+08 3E+08 7E+07 2E+08 6E+08 1E+08 1E+08 1E+08 1E+08 2E+09 1E+08 3E+08 1E+08 1E+08 2E+08 6E+07 8E+07 2E+08 6E+07 1E+08 8E+07 8E+07 9E+07 1E+08 68554 44396 [illegible]	p15010	Cysteine-rich protein 2
PDHB seq: 309 - 324 no mod	38324 4E+06 2E+07 13654 7E+06 2E+07 25552 34892 34104 26736 2E+08 53206 3E+07 40879 18798 7E+06 33072 34984 19162 28832 39759 50401 21442 19024 42741 68554 44396 [illegible]	p13962	Pyruvate dehydrogenase E1 component subunit beta, mitochondrial
UBE2N seq: 54 - 68 no mod	38324 4E+06 2E+07 13654 7E+06 2E+07 25552 34892 34104 26736 2E+08 53206 3E+07 40879 18798 7E+06 33072 34984 19162 28832 39759 50401 21442 19024 42741 68554 44396 [illegible]	p11963	Ubiquitin-conjugating enzyme E2 N
PLEC seq: 1187 - 1201 no mod	38324 1E+07 39656 13654 33854 2E+07 25552 34892 34104 26736 4E+08 53206 2E+07 40879 18798 40867 33072 34984 19162 28832 39759 50401 21442 19024 42741 68554 44396 [illegible]	p01278	Plectin
COX7A2 seq: 34 - 46 no mod	38324 4E+07 9E+07 1E+07 4E+07 45779 2E+07 6E+07 3E+07 2E+07 1E+08 3E+07 1E+08 4E+07 2E+07 2E+07 4E+06 3E+07 7E+07 2E+07 3E+07 8E+06 2E+07 5E+07 2E+07 68554 44396 [illegible]	p11870	Cytochrome c oxidase subunit 7A2, mitochondrial
IQCB1 seq: 419 - 425 no mod	3E+07 6E+07 1E+08 13654 33854 45779 25552 4E+07 34104 26736 1E+07 3E+06 3E+07 40879 18798 40867 9E+06 34984 19162 28832 2E+07 50401 21442 19024 42741 68554 44396 [illegible]	p20974	IQ calmodulin-binding motif-containing protein 1
IDH1 seq: 302 - 314 no mod	[illegible]	p01973	Isocitrate dehydrogenase [NADP] cytoplasmic
EIF4H seq: 113 - 119 no mod	[illegible]	p01685	Eukaryotic translation initiation factor 4H
PGLS seq: 247 - 254 no mod	[illegible]	p11255	6-phosphogluconolactonase
AHNAK seq: 2184 - 2194 no mod	[illegible]	p13697	Neuroblast differentiation-associated protein AHNAK
DDB1 seq: 714 - 722 no mod	[illegible]	p01934	DNA damage-binding protein 1
PSMA6 seq: 72 - 88 no mod	[illegible]	p13619	Proteasome subunit alpha type-6
HSPA5 pM148	[illegible]	p02875	78 kDa glucose-regulated protein
AGR2 pM129	[illegible]	p13960	Anterior gradient protein 2 homolog
KIF12 seq: 88 - 94 no mod	[illegible]	p11799	Kinesin-like protein KIF12
SAFB2 seq: 389 - 395 no mod	[illegible]	p18744	Scaffold attachment factor B2
PAK2 seq: 238 - 246 no mod	[illegible]	p02677	Serine/threonine-protein kinase PAK 2
HSPD1 seq: 494 - 516 no mod	[illegible]	p13942	60 kDa heat shock protein, mitochondrial
GNA13 seq: 60 - 70 no mod	[illegible]	p14021	Guanine nucleotide-binding protein subunit alpha-13
GNAI3 seq: 36 - 46 no mod	[illegible]	p11351	Guanine nucleotide-binding protein G(k) subunit alpha
SRP72 seq: 618 - 645 + 2 Phospho (ST)	[illegible]	p01891	Signal recognition particle subunit SRP72
SEC31A seq: 106 - 120 no mod	[illegible]	p14199	Protein transport protein Sec31A
TLN1 seq: 1321 - 1332 no mod	[illegible]	p20148	Talin-1
PPA2 seq: 177 - 187 no mod	[illegible]	p13995	Inorganic pyrophosphatase 2, mitochondrial
NSMCE2 seq: 115 - 125 no mod	[illegible]	p09169	E3 SUMO-protein ligase NSE2
FLNA seq: 128 - 135 no mod	[illegible]	p20415	Filamin-A
EIF4B seq: 71 - 78 no mod	[illegible]	p05515	Eukaryotic translation initiation factor 4B
LMNB1 pM281	[illegible]	p10671	Lamin-B1
ATAD5 seq: 600 - 610 + Phospho (ST)	[illegible]	p13647	ATPase family AAA domain-containing protein 5
HNRNPUL2 seq: 522 - 528 no mod	[illegible]	p10368	Heterogeneous nuclear ribonucleoprotein U-like protein 2
G6PD seq: 96 - 104 no mod	[illegible]	p11505	Glucose-6-phosphate 1-dehydrogenase
BUB3 seq: 159 - 165 no mod	[illegible]	p01117	Mitotic checkpoint protein BUB3
PLEKHH1 seq: 89 - 94 no mod	[illegible]	p00166	Pleckstrin homology domain-containing family H member 1
LSM14B seq: 317 - 327 no mod	[illegible]	p05910	Protein LSM14 homolog B
UNCX seq: 329 - 344 + Phospho (ST)	[illegible]	p19548	Homeobox protein unc-4 homolog
RPLP2 seq: 42 - 49 no mod	[illegible]	p01350	60S acidic ribosomal protein P2
KRT19 seq: 44 - 51 no mod	[illegible]	p14983	Keratin, type I cytoskeletal 19
ARID1A seq: 1753 - 1793 + Phospho (ST)	[illegible]	p00784	AT-rich interactive domain-containing protein 1A
ACTN4 seq: 282 - 291 no mod	[illegible]	p12190	Alpha-actinin-4
PDIA3 seq: 306 - 329 no mod	[illegible]	p02843	Protein disulfide-isomerase A3
STAT3 pM185	[illegible]	p04252	Signal transducer and activator of transcription 3
SEC16A seq: 11 - 51 + Phospho (ST)	[illegible]	p08200	Protein transport protein Sec16A
LDHA seq: 91 - 99 no mod	[illegible]	p10412	L-lactate dehydrogenase A chain
CLIP1 pS510	[illegible]	p01739	CAP-Gly domain-containing linker protein 1
CCT3 seq: 492 - 502 no mod	[illegible]	p17487	T-complex protein 1 subunit gamma
SLAIN2 seq: 433 - 446 + Phospho (ST)	[illegible]	p06080	SLAIN motif-containing protein 2
GRHPR seq: 67 - 76 no mod	[illegible]	p14084	Glyoxylate reductase/hydroxypyruvate reductase
LSM3 seq: 89 - 100 no mod	[illegible]	p16221	U6 snRNA-associated Sm-like protein LSm3
SRSF5 seq: 119 - 125 no mod	[illegible]	p00756	Serine/arginine-rich splicing factor 5
HDAC2 seq: 366 - 371 no mod	[illegible]	p11895	Histone deacetylase 2
TALDO1 seq: 270 - 277 no mod	[illegible]	p10375	Transaldolase
NMRAL1 seq: 96 - 102 no mod	[illegible]	p11471	NmrA-like family domain-containing protein 1
HSP90B1 pM622	[illegible]	p17805	Endoplasmin
USO1 seq: 765 - 782 no mod	[illegible]	p03744	General vesicular transport factor p115
RPL6 seq: 193 - 200 no mod	[illegible]	p16395	60S ribosomal protein L6
AGR2 seq: 47 - 64 no mod	[illegible]	p14957	Anterior gradient protein 2 homolog
CRMP1 seq: 44 - 56 no mod	[illegible]	p08523	Dihydropyrimidinase-related protein 1
PBRM1 pS353	[illegible]	p10846	Protein polybromo-1
CCT5 seq: 353 - 368 no mod	[illegible]	p11793	T-complex protein 1 subunit epsilon
ZFAND2B seq: 144 - 151 no mod	[illegible]	p20644	AN1-type zinc finger protein 2B
ERP29 seq: 113 - 122 no mod	[illegible]	p17085	Endoplasmic reticulum resident protein 29
DDX46 pS804	[illegible]	p21099	Probable ATP-dependent RNA helicase DDX46
PSMD11 seq: 326 - 337 no mod	[illegible]	p10308	26S proteasome non-ATPase regulatory subunit 11
RPL8 seq: 129 - 144 no mod	[illegible]	p19615	60S ribosomal protein L8
LRRC59 seq: 117 - 126 no mod	[illegible]	p18429	Leucine-rich repeat-containing protein 59
ARHGEF28 seq: 509 - 532 + Phospho (ST)	[illegible]	p02814	Rho guanine nucleotide exchange factor 28
S100A13 seq: 73 - 78 no mod	[illegible]	p16566	Protein S100-A13
FASN seq: 1583 - 1591 no mod	[illegible]	p10718	Fatty acid synthase
CAMSAP3 seq: 337 - 357 + 2 Phospho (ST)	[illegible]	p15112	Calmodulin-regulated spectrin-associated protein 3
RBM19 seq: 361 - 366 no mod	[illegible]	p00304	Probable RNA-binding protein 19
ADRM1 seq: 22 - 27 no mod	[illegible]	p00227	Proteasomal ubiquitin receptor ADRM1
MATR3 seq: 781 - 798 no mod	[illegible]	p14246	Matrin-3
UBA1 seq: 679 - 693 no mod	[illegible]	p12252	Ubiquitin-like modifier-activating enzyme 1
LIG1 seq: 63 - 79 + 2 Phospho (ST)	[illegible]	p01228	DNA ligase 1
BAIAP2L1 seq: 193 - 199 no mod	[illegible]	p16829	Brain-specific angiogenesis inhibitor 1-associated protein 2-like protein 1
ETFA seq: 47 - 59 no mod	[illegible]	p11784	Electron transfer flavoprotein subunit alpha, mitochondrial
NDRG2 pT330 pS332	[illegible]	p03989	Protein NDRG2
LUZP1 seq: 956 - 967 + Phospho (ST)	[illegible]	p03600	Leucine zipper protein 1
STRBP seq: 240 - 249 no mod	[illegible]	p14951	Spermatid perinuclear RNA-binding protein
ILF3 seq: 248 - 257 no mod	[illegible]	p14950	Interleukin enhancer-binding factor 3
PDCL seq: 287 - 301 + Phospho (ST)	[illegible]	p09172	Phosducin-like protein
ME1 seq: 526 - 536 no mod	[illegible]	p03003	NADP-dependent malic enzyme
TP53BP1 pM262 pS265	[illegible]	p06046	Tumor suppressor p53-binding protein 1
EEF2 seq: 43 - 50 no mod	[illegible]	p20646	Elongation factor 2
HERC1 seq: 658 - 666 no mod	[illegible]	p11608	Probable E3 ubiquitin-protein ligase HERC1
IHRF1BP1L seq: 933 - 950 + Oxidation (M); Phospho (ST)	[illegible]	p04864	UHRF1-binding protein 1-like
ARCN1 seq: 486 - 496 no mod	[illegible]	p00686	Coatomer subunit delta
CRYBG3 seq: 1275 - 1286 + Phospho (ST)	[illegible]	p17555	Very large A-kinase anchor protein
SUCLG2 seq: 393 - 403 no mod	[illegible]	p11979	Succinyl-CoA ligase [GDP-forming] subunit beta, mitochondrial
SIN3A seq: 1105 - 1116 + Phospho (ST)	[illegible]	p00216	Paired amphipathic helix protein Sin3a
YTHDF3 seq: 361 - 397 + Phospho (ST)	[illegible]	p16322	YTH domain-containing family protein 3
PSMA1 seq: 108 - 115 no mod	[illegible]	p10351	Proteasome subunit alpha type-1
FUS seq: 335 - 348 no mod	[illegible]	p16183	RNA-binding protein FUS
USO1 seq: 850 - 858 no mod	[illegible]	p10840	General vesicular transport factor p115
PSMA7 seq: 104 - 117 no mod	[illegible]	p10471	Proteasome subunit alpha type-7
CTNNA1 seq: 247 - 287 + Gln->pyro-Glu (N-term Q)	[illegible]	p08328	Catenin alpha-1
CASKIN2 seq: 1123 - 1147 + Phospho (ST)	[illegible]	p11715	Caskin-2
FAM195A seq: 75 - 99 + 2 Phospho (ST)	[illegible]	p07610	Protein FAM195A
RPL9 seq: 174 - 184 no mod	[illegible]	p13243	60S ribosomal protein L9
YWHAB seq: 196 - 214 no mod	[illegible]	p03094	14-3-3 protein beta/alpha
RBBP6 seq: 1255 - 1280 + Phospho (ST)	[illegible]	p00725	E3 ubiquitin-protein ligase RBBP6
EEF1A1P5 seq: 147 - 154 + Gln->pyro-Glu (N-term Q)	[illegible]	p08366	Putative elongation factor 1-alpha-like 3
HSPA1A seq: 300 - 311 no mod	[illegible]	p19718	Heat shock 70 kDa protein 1A
DNAJC3 seq: 439 - 446 no mod	[illegible]	p16693	DnaJ homolog subfamily C member 3
RPL24 seq: 28 - 35 no mod	[illegible]	p01531	60S ribosomal protein L24
LRBA seq: 1506 - 1514 no mod	[illegible]	p11316	Lipopolysaccharide-responsive and beige-like anchor protein
FLNA seq: 286 - 299 no mod	[illegible]	p19126	Filamin-A
KRT18 seq: 51 - 90 + Oxidation (M); Phospho (ST)	[illegible]	p03459	Keratin, type I cytoskeletal 18
TPI1 seq: 257 - 285 no mod	[illegible]	p17532	Triosephosphate isomerase
FKBP3 seq: 174 - 184 no mod	[illegible]	p14961	Peptidyl-prolyl cis-trans isomerase FKBP3
EIF2S3 seq: 290 - 303 no mod	[illegible]	p01457	Eukaryotic translation initiation factor 2 subunit 3
PRDX3 seq: 185 - 196 no mod	[illegible]	p15751	Thioredoxin-dependent peroxide reductase, mitochondrial
CDV3 seq: 28 - 76 no mod	[illegible]	p21061	Protein CDV3 homolog
HEBP2 pS181	[illegible]	p00511	Heme-binding protein 2
SARNP seq: 181 - 199 no mod	[illegible]	p16752	SAP domain-containing ribonucleoprotein
CDR2L pT154	[illegible]	p02708	Cerebellar degeneration-related protein 2-like
TXNDC17 seq: 90 - 98 no mod	[illegible]	p00748	Thioredoxin domain-containing protein 17
GEMIN8 seq: 106 - 144 + 2 Phospho (ST)	[illegible]	p13837	Gem-associated protein 8
PKLR seq: 436 - 442 no mod	[illegible]	p08389	Pyruvate kinase PKLR
CRIP2 pS195	[illegible]	p15009	Cysteine-rich protein 2
CMPK1 seq: 89 - 96 no mod	[illegible]	p16614	UMP-CMP kinase
ATP5O seq: 27 - 40 no mod	[illegible]	p10361	ATP synthase subunit O, mitochondrial
IGFBP5 seq: 251 - 272 + Phospho (ST)	[illegible]	p11124	Insulin-like growth factor-binding protein 5
ETFB seq: 206 - 221 no mod	[illegible]	p14322	Electron transfer flavoprotein subunit beta
IBTK seq: 990 - 1012 + Phospho (ST)	[illegible]	p06073	Inhibitor of Bruton tyrosine kinase
PDHB seq: 220 - 227 no mod	[illegible]	p18878	Pyruvate dehydrogenase E1 component subunit beta, mitochondrial
TPD52 seq: 150 - 163 no mod	[illegible]	p19666	Tumor protein D52
TOMM20 seq: 126 - 132 no mod	[illegible]	p11064	Mitochondrial import receptor subunit TOM20 homolog
AFF3 pS878 pS881	[illegible]	p10002	AF4/FMR2 family member 3
HNRNPL seq: 47 - 56 no mod	[illegible]	p00011	Heterogeneous nuclear ribonucleoprotein L
RAPH1 pS845	[illegible]	p08019	Ras-associated and pleckstrin homology domains-containing protein 1
GOT2 seq: 326 - 337 no mod	[illegible]	p14541	Aspartate aminotransferase, mitochondrial
FASN seq: 1117 - 1131 no mod	[illegible]	p16828	Fatty acid synthase
SHANK2 pS1001 pM1002	[illegible]	p02682	SH3 and multiple ankyrin repeat domains protein 2
FLOT1 seq: 378 - 392 + Phospho (ST)	[illegible]	p13596	Flotillin-1
DGKD pS44	[illegible]	p12392	Diacylglycerol kinase delta
PDHB seq: 337 - 347 no mod	[illegible]	p14072	Pyruvate dehydrogenase E1 component subunit beta, mitochondrial
HNRNPM seq: 323 - 345 no mod	[illegible]	p15894	Heterogeneous nuclear ribonucleoprotein M
PSMB1 seq: 229 - 239 no mod	[illegible]	p17736	Proteasome subunit beta type-1
ECH1 seq: 104 - 112 no mod	[illegible]	p19151	Delta(3,5)-Delta(2,4)-dienoyl-CoA isomerase, mitochondrial
G6PD seq: 206 - 215 no mod	[illegible]	p17351	Glucose-6-phosphate 1-dehydrogenase
ANXA3 seq: 105 - 120 no mod	[illegible]	p16315	Annexin A3
HLA-B seq: 46 - 59 no mod	[illegible]	p16699	HLA class I histocompatibility antigen, B-57 alpha chain
FLNA seq: 44 - 51 no mod	[illegible]	p13787	Filamin-A
ATP5B pM478	[illegible]	p16582	ATP synthase subunit beta, mitochondrial
DDT seq: 2 - 14 no mod	[illegible]	p08901	D-dopachrome decarboxylase
MIF seq: 2 - 12 no mod	[illegible]	p08887	Macrophage migration inhibitory factor
TPRN seq: 413 - 437 + Phospho (ST)	[illegible]	p00483	Taperin
HSPD1 seq: 430 - 446 no mod	[illegible]	p20988	60 kDa heat shock protein, mitochondrial
HSPA1B seq: 302 - 311 no mod	[illegible]	p16800	Heat shock 70 kDa protein 1B
PDIA4 seq: 500 - 506 no mod	[illegible]	p17775	Protein disulfide-isomerase A4
SCRIB pS1378	[illegible]	p06435	Protein scribble homolog
TPM3 seq: 170 - 179 no mod	[illegible]	p10413	Tropomyosin alpha-3 chain
AHNAK seq: 2215 - 2221 no mod	[illegible]	p01024	Neuroblast differentiation-associated protein AHNAK
TMPO seq: 127 - 139 no mod	[illegible]	p00336	Lamina-associated polypeptide 2, isoforms beta/gamma
MYL6 pM73	[illegible]	p01263	Myosin light polypeptide 6
LIMA1 pM674 pS686	[illegible]	p17743	LIM domain and actin-binding protein 1
SPAG9 pT226	[illegible]	p16063	C-Jun-amino-terminal kinase-interacting protein 4
FASN seq: 1508 - 1515 no mod	[illegible]	p18842	Fatty acid synthase
ARID2 seq: 1381 - 1408 + Phospho (ST)	[illegible]	p02166	AT-rich interactive domain-containing protein 2
TUBB4A pM363	[illegible]	p10279	Tubulin beta-4A chain
TUBB6 seq: 363 - 379 no mod	[illegible]	p10236	Tubulin beta-6 chain
TUBB4B seq: 363 - 379 no mod	[illegible]	p09914	Tubulin beta-4B chain
FASN seq: 1740 - 1752 no mod	[illegible]	p15050	Fatty acid synthase
HSPA9 seq: 647 - 653 no mod	[illegible]	p11897	Stress-70 protein, mitochondrial
IMPDH2 seq: 357 - 375 no mod	[illegible]	p16723	Inosine-5'-monophosphate dehydrogenase 2
ITSN1 seq: 891 - 925 + 2 Phospho (ST)	[illegible]	p06343	Intersectin-1
EIF4G1 pS1077	[illegible]	p13603	Eukaryotic translation initiation factor 4 gamma 1
CCT6A seq: 450 - 465 no mod	[illegible]	p01273	T-complex protein 1 subunit zeta
LMNB1 seq: 183 - 191 no mod	[illegible]	p08410	Lamin-B1
CCT3 seq: 439 - 449 no mod	[illegible]	p19271	T-complex protein 1 subunit gamma
AGR2 seq: 40 - 46 no mod	[illegible]	p11084	Anterior gradient protein 2 homolog
BAP18 pS96	[illegible]	p12366	Chromatin complexes subunit BAP18
TAF15 seq: 75 - 101 no mod	[illegible]	p07827	TATA-binding protein-associated factor 2N
PHGDH seq: 22 - 33 no mod	[illegible]	p14007	D-3-phosphoglycerate dehydrogenase
PRDX6 seq: 2 - 22 no mod	[illegible]	p08899	Peroxiredoxin-6
RPL15 seq: 97 - 105 no mod	[illegible]	p05077	60S ribosomal protein L15
PGD seq: 77 - 87 no mod	[illegible]	p20594	6-phosphogluconate dehydrogenase, decarboxylating
RPL38 seq: 58 - 67 no mod	[illegible]	p07884	60S ribosomal protein L38
CLTC seq: 1102 - 1113 no mod	[illegible]	p19035	Clathrin heavy chain 1
HSPA9 seq: 378 - 391 no mod	[illegible]	p06107	Stress-70 protein, mitochondrial
PDIA4 seq: 350 - 362 no mod	[illegible]	p00790	Protein disulfide-isomerase A4
TGS1 seq: 511 - 517 no mod	[illegible]	p13986	Trimethylguanosine synthase
ACTR1A seq: 314 - 320 no mod	[illegible]	p11293	Alpha-centractin
HNRNPUL2 seq: 594 - 601 no mod	[illegible]	p20054	Heterogeneous nuclear ribonucleoprotein U-like protein 2
PCBP1 seq: 102 - 115 no mod	[illegible]	p10332	Poly(rC)-binding protein 1
CXorf67 pS259	[illegible]	p19372	Uncharacterized protein CXorf67
SSB seq: 270 - 276 no mod	[illegible]	p17738	Lupus La protein
MMP3 seq: 304 - 310 no mod	[illegible]	p16159	Stromelysin-1
FLNB seq: 1546 - 1564 no mod	[illegible]	p18985	Filamin-B
GSN seq: 585 - 597 no mod	[illegible]	p20718	Gelsolin

HSPA5 pM332	[illegible]	p20403	78 kDa glucose-regulated protein
MAPRE1 seq: 205 - 212 no mod	[illegible]	p10473	Microtubule-associated protein RP/EB family member 1
GAPDH seq: 67 - 80 no mod	[illegible]	p10408	Glyceraldehyde-3-phosphate dehydrogenase
HSD17B10 seq: 185 - 192 no mod	[illegible]	p18648	3-hydroxyacyl-CoA dehydrogenase type-2
RPL23 seq: 52 - 66 no mod	[illegible]	p11141	60S ribosomal protein L23
HSP90AA1 seq: 633 - 647 no mod	[illegible]	p14751	Heat shock protein HSP 90-alpha
RTCB seq: 4 - 14 no mod	[illegible]	p03155	tRNA-splicing ligase RtcB homolog
ATRX pS1527	[illegible]	p10902	Transcriptional regulator ATRX
MUC5B seq: 109 - 119 no mod	[illegible]	p20968	Mucin-5B
PLEC seq: 2319 - 2329 no mod	[illegible]	p19732	Plectin
TBC1D10A seq: 334 - 340 + Phospho (ST)	[illegible]	p10867	TBC1 domain family member 10A
CAAP1 pS203	[illegible]	p14067	Caspase activity and apoptosis inhibitor 1
PGM3 seq: 190 - 196 no mod	[illegible]	p20735	Phosphoacetylglucosamine mutase
FLNB seq: 2579 - 2586 no mod	[illegible]	p16187	Filamin-B
SNRNP70 seq: 17 - 27 no mod	[illegible]	p18404	U1 small nuclear ribonucleoprotein 70 kDa
UHRF1BP1 seq: 1427 - 1432 no mod	[illegible]	p11312	UHRF1-binding protein 1
PSMC3 seq: 130 - 144 no mod	[illegible]	p07756	26S protease regulatory subunit 6A
C14orf142 seq: 60 - 85 no mod	[illegible]	p01851	Uncharacterized protein C14orf142
LPXN seq: 36 - 86 + 2 Phospho (ST)	[illegible]	p13271	Leupaxin
PDAP1 seq: 57 - 73 + Phospho (ST)	[illegible]	p05280	28 kDa heat- and acid-stable phosphoprotein
PRDX2 seq: 151 - 157 no mod	[illegible]	p03387	Peroxiredoxin-2
SRP9 seq: 17 - 24 no mod	[illegible]	p10299	Signal recognition particle 9 kDa protein
SRSF6 pS316	[illegible]	p03264	Serine/arginine-rich splicing factor 6
LMNA seq: 420 - 427 + Phospho (ST)	[illegible]	p12991	Prelamin-A/C
MIA3 seq: 1878 - 1904 + Phospho (ST)	[illegible]	p18582	Melanoma inhibitory activity protein 3
COX5A seq: 88 - 94 no mod	[illegible]	p14193	Cytochrome c oxidase subunit 5A, mitochondrial
PRDX1 seq: 69 - 92 no mod	[illegible]	p11211	Peroxiredoxin-1
LAMC1 seq: 1358 - 1371 no mod	[illegible]	p18170	Laminin subunit gamma-1
PCBP2 pS173	[illegible]	p08539	Poly(rC)-binding protein 2
HNRNPAB seq: 172 - 190 no mod	[illegible]	p16917	Heterogeneous nuclear ribonucleoprotein A/B
TP53BP1 pS552	[illegible]	p14440	Tumor suppressor p53-binding protein 1
NME2P1 seq: 35 - 41 no mod	[illegible]	p08561	Putative nucleoside diphosphate kinase
CCDC6 pS323	[illegible]	p08319	Coiled-coil domain-containing protein 6
AGR2 seq: 47 - 64 no mod	[illegible]	p14956	Anterior gradient protein 2 homolog
FAM129B seq: 621 - 654 + 2 Phospho (ST)	[illegible]	p07683	Niban-like protein 1
PRRC2A seq: 1105 - 1128 + Phospho (ST)	[illegible]	p15296	Protein PRRC2A
SPTBN1 pS2102 pS2138	[illegible]	p06882	Spectrin beta chain, non-erythrocytic 1
FSCN1 seq: 33 - 41 no mod	[illegible]	p01090	Fascin
WBP11 seq: 310 - 316 no mod	[illegible]	p16876	WW domain-binding protein 11
RPS5 seq: 168 - 182 no mod	[illegible]	p02736	40S ribosomal protein S5
HERC2 seq: 3548 - 3554 no mod	[illegible]	p00564	E3 ubiquitin-protein ligase HERC2
HADHA seq: 423 - 440 no mod	[illegible]	p18274	Trifunctional enzyme subunit alpha, mitochondrial
ENO1 pM165 pM169	[illegible]	p12225	Alpha-enolase
DCP1B pS147	[illegible]	p20527	mRNA-decapping enzyme 1B
ERBB2 pS1054	[illegible]	p05761	Receptor tyrosine-protein kinase erbB-2
TKT seq: 187 - 204 no mod	[illegible]	p11710	Transketolase
PSMA3 seq: 101 - 110 no mod	[illegible]	p04846	Proteasome subunit alpha type-3
PPL seq: 772 - 780 no mod	[illegible]	p09443	Periplakin
SIPA1L2 seq: 160 - 190 + Phospho (ST)	[illegible]	p04811	Signal-induced proliferation-associated 1-like protein 2
PLEC seq: 2795 - 2808 no mod	[illegible]	p11022	Plectin
HNRNPM seq: 323 - 345 no mod	[illegible]	p15895	Heterogeneous nuclear ribonucleoprotein M
PARD3 pS728	[illegible]	p13731	Partitioning defective 3 homolog
HNRNPH3 seq: 206 - 222 no mod	[illegible]	p19394	Heterogeneous nuclear ribonucleoprotein H3
NCK2 seq: 88 - 108 + Phospho (ST)	[illegible]	p18952	Cytoplasmic protein NCK2
MYO6 seq: 1142 - 1167 no mod	[illegible]	p03229	Unconventional myosin-VI
AKAP2 seq: 55 - 105 + Phospho (ST)	[illegible]	p00271	A-kinase anchor protein 2
AHNAK seq: 4989 - 5017 + Phospho (ST)	[illegible]	p20911	Neuroblast differentiation-associated protein AHNAK
PSMA6 seq: 105 - 116 no mod	[illegible]	p00331	Proteasome subunit alpha type-6
ERBB2 pS1174	[illegible]	p02543	Receptor tyrosine-protein kinase erbB-2
HEBP1 seq: 153 - 162 no mod	[illegible]	p21101	Heme-binding protein 1
STIP1 seq: 514 - 530 no mod	[illegible]	p18400	Stress-induced-phosphoprotein 1
ENO1 seq: 72 - 80 no mod	[illegible]	p02732	Alpha-enolase
LARP1 pS774	[illegible]	p05095	La-related protein 1
ANKFY1 seq: 732 - 746 no mod	[illegible]	p20486	Rabankyrin-5
TPD52 seq: 111 - 120 no mod	[illegible]	p11757	Tumor protein D52
RPL4 seq: 284 - 291 no mod	[illegible]	p10067	60S ribosomal protein L4
FLNB seq: 1355 - 1372 no mod	[illegible]	p16320	Filamin-B
LDHA seq: 6 - 14 no mod	[illegible]	p18375	L-lactate dehydrogenase A chain
FAM114A1 pS196	[illegible]	p14212	Protein NOXP20
PHF8 seq: 690 - 717 + 2 Phospho (ST)	[illegible]	p00906	Histone lysine demethylase PHF8
NSFL1C seq: 114 - 124 no mod	[illegible]	p04523	NSFL1 cofactor p47
ITPKC seq: 74 - 110 + Phospho (ST)	[illegible]	p20638	Inositol-trisphosphate 3-kinase C
TNKS1BP1 pS1666	[illegible]	p06359	182 kDa tankyrase-1-binding protein
AHNAK seq: 2537 - 2545 no mod	[illegible]	p20885	Neuroblast differentiation-associated protein AHNAK
ALDOA seq: 112 - 134 no mod	[illegible]	p14966	Fructose-bisphosphate aldolase A
SMG8 seq: 740 - 757 + Phospho (ST)	[illegible]	p08805	Protein SMG8
HNRNPM seq: 372 - 381 no mod	[illegible]	p13928	Heterogeneous nuclear ribonucleoprotein M
TP53BP1 pS727	[illegible]	p17897	Tumor suppressor p53-binding protein 1
PCBP1 seq: 145 - 160 no mod	[illegible]	p20422	Poly(rC)-binding protein 1
ISY1 seq: 200 - 238 + Phospho (ST)	[illegible]	p16158	Pre-mRNA-splicing factor ISY1 homolog
FAM83E pS443	[illegible]	p00233	Protein FAM83E
SND1 seq: 770 - 776 no mod	[illegible]	p16967	Staphylococcal nuclease domain-containing protein 1
NCOA3 seq: 648 - 681 + Phospho (ST)	[illegible]	p17106	Nuclear receptor coactivator 3
STIP1 seq: 161 - 169 no mod	[illegible]	p09500	Stress-induced-phosphoprotein 1
PSMC1 seq: 250 - 258 no mod	[illegible]	p00620	26S protease regulatory subunit 4
CNDP2 seq: 454 - 461 no mod	[illegible]	p00196	Cytosolic non-specific dipeptidase
EEF1A1 seq: 220 - 247 no mod	[illegible]	p18807	Elongation factor 1-alpha 1
CDC42EP3 seq: 105 - 131 + Phospho (ST)	[illegible]	p09775	Cdc42 effector protein 3
PLEKHG3 pS423	[illegible]	p00145	Pleckstrin homology domain-containing family G member 3
MUC5B seq: 2758 - 2768 + Phospho (ST)	[illegible]	p05010	Mucin-5B
BCLAF1 seq: 225 - 255 + Phospho (ST)	[illegible]	p04589	Bcl-2-associated transcription factor 1
SNIP1 seq: 366 - 396 + Phospho (ST)	[illegible]	p16887	Smad nuclear-interacting protein 1
NOS1AP seq: 255 - 268 + Phospho (ST)	[illegible]	p00851	Carboxyl-terminal PDZ ligand of neuronal nitric oxide synthase protein
ABI1 pS323	[illegible]	p14703	Abl interactor 1
ENO1 seq: 82 - 89 no mod	[illegible]	p11145	Alpha-enolase
HNRNPC pS260	[illegible]	p10175	Heterogeneous nuclear ribonucleoproteins C1/C2
GPI seq: 212 - 226 no mod	[illegible]	p02837	Glucose-6-phosphate isomerase
HNRNPF seq: 300 - 316 no mod	[illegible]	p19393	Heterogeneous nuclear ribonucleoprotein F
PKM seq: 44 - 56 no mod	[illegible]	p09026	Pyruvate kinase PKM
API5 pS464	[illegible]	p19637	Apoptosis inhibitor 5
NAV1 pS377	[illegible]	p11955	Neuron navigator 1
ERRFI1 seq: 117 - 148 + 2 Phospho (ST)	[illegible]	p12881	ERBB receptor feedback inhibitor 1
GEMIN8 seq: 112 - 144 + 2 Phospho (ST)	[illegible]	p17872	Gem-associated protein 8
EEA1 seq: 1276 - 1292 no mod	[illegible]	p16160	Early endosome antigen 1
AHNAK seq: 2569 - 2575 no mod	[illegible]	p09968	Neuroblast differentiation-associated protein AHNAK
PA2G4 seq: 23 - 30 no mod	[illegible]	p10122	Proliferation-associated protein 2G4
LUZP2 seq: 156 - 162 no mod	[illegible]	p13078	Leucine zipper protein 2
MTHFD2 seq: 44 - 50 no mod	[illegible]	p13024	Bifunctional methylenetetrahydrofolate dehydrogenase/cyclohydrolase, mitochondrial
THOC7 seq: 104 - 110 no mod	[illegible]	p12681	THO complex subunit 7 homolog
VCP seq: 211 - 217 no mod	[illegible]	p12672	Transitional endoplasmic reticulum ATPase
DMGDH seq: 758 - 764 no mod	[illegible]	p08846	Dimethylglycine dehydrogenase, mitochondrial
MIF pM3	[illegible]	p08886	Macrophage migration inhibitory factor
ALDOA seq: 109 - 134 no mod	[illegible]	p01676	Fructose-bisphosphate aldolase A
q: 145 - 173 + Gln->pyro-Glu (N-term Q); 2 Phospho (ST)	[illegible]	p08735	UV excision repair protein RAD23 homolog B
GAPDH seq: 67 - 84 no mod	[illegible]	p10407	Glyceraldehyde-3-phosphate dehydrogenase
BCL7C seq: 110 - 142 + 3 Phospho (ST)	[illegible]	p15104	B-cell CLL/lymphoma 7 protein family member C
NPM1 seq: 81 - 101 no mod	[illegible]	p09869	Nucleophosmin
UBA1 seq: 11 - 43 + Phospho (ST)	[illegible]	p06443	Ubiquitin-like modifier-activating enzyme 1
CAST seq: 364 - 378 + Phospho (ST)	[illegible]	p05992	Calpastatin
HSPA4 seq: 799 - 840 no mod	[illegible]	p09786	Heat shock 70 kDa protein 4
EEF1B2 pS95 pS106	[illegible]	p00356	Elongation factor 1-beta
WDR1 seq: 197 - 203 no mod	[illegible]	p16456	WD repeat-containing protein 1
PRPF38B pS529	[illegible]	p04194	Pre-mRNA-splicing factor 38B
RLBP1 seq: 92 - 98 no mod	[illegible]	p18287	Retinaldehyde-binding protein 1
TJP2 seq: 604 - 610 no mod	[illegible]	p16201	Tight junction protein ZO-2
ATRX pS1348 pS1352	[illegible]	p14757	Transcriptional regulator ATRX
CDH1 pS793	[illegible]	p09694	Cadherin-1
PPP1R13L seq: 186 - 235 + Phospho (Y)	[illegible]	p15219	RelA-associated inhibitor
HNRNPH3 seq: 85 - 90 no mod	[illegible]	p00309	Heterogeneous nuclear ribonucleoprotein H3
ESRP1 seq: 398 - 403 no mod	[illegible]	p00307	Epithelial splicing regulatory protein 1
HDLBP seq: 315 - 324 no mod	[illegible]	p15568	Vigilin
eq: 971 - 987 + Gln->pyro-Glu (N-term Q); Phospho (ST)	[illegible]	p07942	Insulin receptor substrate 2
TBC1D4 pS754	[illegible]	p02088	TBC1 domain family member 4
HNRNPU seq: 525 - 536 no mod	[illegible]	p00205	Heterogeneous nuclear ribonucleoprotein U
IRS2 seq: 363 - 396 + 2 Phospho (ST)	[illegible]	p03049	Insulin receptor substrate 2
IRS2 seq: 812 - 832 + Phospho (Y)	[illegible]	p19841	Insulin receptor substrate 2
KRT18 pM58 pM74 pM84	[illegible]	p16016	Keratin, type I cytoskeletal 18
IRS2 seq: 363 - 396 + Phospho (ST)	[illegible]	p03048	Insulin receptor substrate 2
TXN seq: 9 - 36 no mod	[illegible]	p03090	Thioredoxin
SNX1 seq: 14 - 55 + Phospho (ST)	[illegible]	p11096	Sorting nexin-1
HNRNPA1 seq: 123 - 140 no mod	[illegible]	p18047	Heterogeneous nuclear ribonucleoprotein A1
NACC1 seq: 336 - 354 no mod	[illegible]	p00895	Nucleus accumbens-associated protein 1
BAG6 seq: 987 - 1030 no mod	[illegible]	p15473	Large proline-rich protein BAG6
ERCC6L seq: 1157 - 1192 + Phospho (ST)	[illegible]	p03568	DNA excision repair protein ERCC-6-like
EIF3I seq: 115 - 141 no mod	[illegible]	p08247	Eukaryotic translation initiation factor 3 subunit I
KRT19 seq: 327 - 342 no mod	[illegible]	p02579	Keratin, type I cytoskeletal 19
LRRFIP1 seq: 91 - 117 + Oxidation (M); Phospho (ST)	[illegible]	p18854	Leucine-rich repeat flightless-interacting protein 1
SERBP1 seq: 217 - 236 + Phospho (ST)	[illegible]	p15971	Plasminogen activator inhibitor 1 RNA-binding protein
GSN seq: 200 - 234 no mod	[illegible]	p19388	Gelsolin
MAP4 pS624	[illegible]	p15854	Microtubule-associated protein 4
BCLAF1 pS268	[illegible]	p09009	Bcl-2-associated transcription factor 1
RPL19 seq: 21 - 38 no mod	[illegible]	p12368	60S ribosomal protein L19
PRRC2B seq: 1231 - 1248 + Phospho (ST)	[illegible]	p04606	Protein PRRC2B
GAPDH pT184	[illegible]	p01384	Glyceraldehyde-3-phosphate dehydrogenase
LCP1 seq: 473 - 488 no mod	[illegible]	p16461	Plastin-2
AKR1A1 seq: 154 - 167 no mod	[illegible]	p15620	Alcohol dehydrogenase [NADP(+)]
ERRFI1 seq: 248 - 263 + Phospho (ST)	[illegible]	p06674	ERBB receptor feedback inhibitor 1
CDC42EP1 pS101 pS113	[illegible]	p10240	Cdc42 effector protein 1
CRAMP1L seq: 1178 - 1208 + Phospho (ST)	[illegible]	p10010	Protein cramped-like
ACSL1 seq: 408 - 414 no mod	[illegible]	p09298	Long-chain-fatty-acid--CoA ligase 1
PURB seq: 268 - 274 no mod	[illegible]	p16759	Transcriptional activator protein Pur-beta
HNRNPK seq: 103 - 139 + Phospho (ST)	[illegible]	p13098	Heterogeneous nuclear ribonucleoprotein K
TJP1 seq: 112 - 140 + Phospho (ST)	[illegible]	p00950	Tight junction protein ZO-1
ENO1 seq: 286 - 306 no mod	[illegible]	p18038	Alpha-enolase
ERRFI1 seq: 118 - 134 + Phospho (ST)	[illegible]	p10464	ERBB receptor feedback inhibitor 1
RPL28 seq: 12 - 19 no mod	[illegible]	p09723	60S ribosomal protein L28
PDLIM1 pS90	[illegible]	p00550	PDZ and LIM domain protein 1
ERRFI1 pS251	[illegible]	p05582	ERBB receptor feedback inhibitor 1
CCDC12 seq: 143 - 166 + Phospho (ST)	[illegible]	p11508	Coiled-coil domain-containing protein 12
UFL1 seq: 536 - 552 no mod	[illegible]	p02706	E3 UFM1-protein ligase 1
PPP1R15A pS143	[illegible]	p19965	Protein phosphatase 1 regulatory subunit 15A
EPPK1 seq: 2533 - 2547 no mod	[illegible]	p19203	Epiplakin
AHNAK pT490	[illegible]	p01292	Neuroblast differentiation-associated protein AHNAK
UBA1 seq: 486 - 510 no mod	[illegible]	p09660	Ubiquitin-like modifier-activating enzyme 1
PGM1 seq: 165 - 172 no mod	[illegible]	p01673	Phosphoglucomutase-1
GSDMD seq: 250 - 267 + Oxidation (M); Phospho (ST)	[illegible]	p03469	Gasdermin-D
LAMP2 seq: 145 - 152 no mod	[illegible]	p13880	Lysosome-associated membrane glycoprotein 2
EPHA2 seq: 891 - 907 + Phospho (ST)	[illegible]	p00839	Ephrin type-A receptor 2
PHACTR4 pS443	[illegible]	p20517	Phosphatase and actin regulator 4
PSME1 seq: 167 - 176 no mod	[illegible]	p11986	Proteasome activator complex subunit 1
AHNAK seq: 5748 - 5767 + Phospho (ST)	[illegible]	p19580	Neuroblast differentiation-associated protein AHNAK
NAV2 seq: 1277 - 1292 + Phospho (ST)	[illegible]	p15840	Neuron navigator 2
NCOA5 seq: 336 - 353 no mod	[illegible]	p16042	Nuclear receptor coactivator 5
FAM117A seq: 420 - 437 + Phospho (ST)	[illegible]	p13331	Protein FAM117A
DNAJB6 pS277	[illegible]	p14894	DnaJ homolog subfamily B member 6
CALML5 seq: 106 - 124 no mod	[illegible]	p20101	Calmodulin-like protein 5
CHERP seq: 477 - 510 no mod	[illegible]	p18959	Calcium homeostasis endoplasmic reticulum protein
GSTO1 seq: 49 - 57 no mod	[illegible]	p14838	Glutathione S-transferase omega-1
FKBP1A seq: 59 - 72 no mod	[illegible]	p14959	Peptidyl-prolyl cis-trans isomerase FKBP1A
ACTB seq: 214 - 238 no mod	[illegible]	p17562	Actin, cytoplasmic 1
PKP2 pS251	[illegible]	p04900	Plakophilin-2
KMT2B seq: 2197 - 2203 no mod	[illegible]	p11709	Histone-lysine N-methyltransferase 2B
AKT2 pS474	[illegible]	p02754	RAC-beta serine/threonine-protein kinase
PPP1R12A pS299	[illegible]	p18680	Protein phosphatase 1 regulatory subunit 12A
NUP93 seq: 418 - 434 no mod	[illegible]	p11166	Nuclear pore complex protein Nup93
CALM1 seq: 92 - 107 no mod	[illegible]	p01572	Calmodulin
ZNF185 seq: 151 - 175 + 2 Phospho (ST)	[illegible]	p06568	Zinc finger protein 185
ERRFI1 pS461	[illegible]	p14723	ERBB receptor feedback inhibitor 1
NAV2 pS1215	[illegible]	p05107	Neuron navigator 2
JUN pS243	[illegible]	p11037	Transcription factor AP-1
MKL2 seq: 829 - 867 + Phospho (ST)	[illegible]	p09756	MKL/myocardin-like protein 2
HSP90AA1 seq: 47 - 60 no mod	[illegible]	p17466	Heat shock protein HSP 90-alpha
SRI seq: 107 - 116 + Gln->pyro-Glu (N-term Q)	[illegible]	p08570	Sorcin
TNKS1BP1 seq: 976 - 1000 + 2 Phospho (ST)	[illegible]	p05908	182 kDa tankyrase-1-binding protein
PALLD pS893	[illegible]	p14474	Palladin
XRCC5 seq: 655 - 660 no mod	[illegible]	p16554	X-ray repair cross-complementing protein 5
RIPK1 seq: 333 - 377 + Phospho (ST)	[illegible]	p04814	Receptor-interacting serine/threonine-protein kinase 1
AHNAK seq: 1013 - 1024 no mod	[illegible]	p16166	Neuroblast differentiation-associated protein AHNAK
PABPN1 seq: 26 - 78 + Phospho (ST)	[illegible]	p14721	Polyadenylate-binding protein 2
AHNAK seq: 5834 - 5858 + Phospho (ST)	[illegible]	p15920	Neuroblast differentiation-associated protein AHNAK

Peptide	Col 1	Col 2	Intermediate intensity columns	Last col	Accession	Protein
KANSL1 seq: 267 - 283 + Phospho (ST)	38324	37149	[illegible]	1E+07	p12562	KAT8 regulatory NSL complex subunit 1
AMOTL2 pS166	6E+06	37149	[illegible]	1E+08	p11307	Angiomotin-like protein 2
AKAP12 seq: 582 - 604 + Phospho (ST)	38324	37149	[illegible]	4E+08	p15783	A-kinase anchor protein 12
HNRNPK seq: 434 - 456 no mod	38324	37149	[illegible]	25732	p14128	Heterogeneous nuclear ribonucleoprotein K
DESI1 pS25	38324	37149	[illegible]	1E+08	p07098	Desumoylating isopeptidase 1
CYR61 seq: 195 - 203 no mod	38324	37149	[illegible]	3E+07	p09347	Protein CYR61
ACTN2 seq: 148 - 156 no mod	38324	4E+06	[illegible]	25732	p17724	Alpha-actinin-2
JUN seq: 222 - 252 + 2 Phospho (ST)	38324	37149	[illegible]	1E+08	p11040	Transcription factor AP-1
CXorf67 pS105	38324	37149	[illegible]	25732	p04596	Uncharacterized protein CXorf67
RANBP2 seq: 2478 - 2506 + Phospho (ST)	38324	37149	[illegible]	2E+08	p17189	E3 SUMO-protein ligase RanBP2
LMO7 seq: 857 - 881 + Phospho (ST)	38324	37149	[illegible]	25732	p00672	LIM domain only protein 7
MAP1B pS1427	38324	37149	[illegible]	2E+08	p16328	Microtubule-associated protein 1B
PPP1CC seq: 112 - 122 no mod	38324	37149	[illegible]	25732	p14099	Serine/threonine-protein phosphatase PP1-gamma catalytic subunit
CCDC58 seq: 111 - 125 no mod	38324	37149	[illegible]	8E+07	p00489	Coiled-coil domain-containing protein 58
EPPK1 pS2716	38324	37149	[illegible]	25732	p06772	Epiplakin
TJP1 pS175 pS178 pS179	38324	37149	[illegible]	2E+08	p03391	Tight junction protein ZO-1
RAPGEF6 seq: 379 - 385 no mod	2E+06	37149	[illegible]	25732	p14014	Rap guanine nucleotide exchange factor 6
DDIT4 seq: 1 - 7 no mod	38324	37149	[illegible]	25732	p09946	DNA damage-inducible transcript 4 protein
ZFP36 seq: 83 - 103 + Phospho (ST)	38324	2E+07	[illegible]	2E+08	p11722	Tristetraprolin
EEF1E1 seq: 29 - 53 no mod	38324	37149	[illegible]	25732	p08511	Eukaryotic translation elongation factor 1 epsilon-1
MAP1B seq: 1394 - 1419 + Phospho (ST)	38324	37149	[illegible]	2E+08	p01165	Microtubule-associated protein 1B
FEZF1 seq: 88 - 96 + Phospho (ST)	38324	37149	[illegible]	25732	p20542	Fez family zinc finger protein 1
EI24 seq: 60 - 66 + Phospho (ST)	38324	37149	[illegible]	25732	p12685	Etoposide-induced protein 2.4 homolog
MAP3K6 seq: 912 - 919 + Phospho (ST)	38324	37149	[illegible]	25732	p04017	Mitogen-activated protein kinase kinase kinase 6
HUWE1 seq: 647 - 668 + Phospho (ST)	38324	37149	[illegible]	25732	p06621	E3 ubiquitin-protein ligase HUWE1
FLNA pS968	38324	1E+07	[illegible]	5E+08	p04668	Filamin-A
EIF4A3 seq: 340 - 358 no mod	38324	37149	[illegible]	25732	p15765	Eukaryotic initiation factor 4A-III
SRP68 seq: 312 - 335 no mod	38324	37149	[illegible]	25732	p14300	Signal recognition particle subunit SRP68
PDIA3 seq: 153 - 173 no mod	38324	4E+06	[illegible]	1E+07	p18955	Protein disulfide-isomerase A3
GOLGB1 seq: 272 - 288 no mod	38324	37149	[illegible]	25732	p19728	Golgin subfamily B member 1
ATAD2B seq: 11 - 42 + Phospho (ST)	38324	37149	[illegible]	6E+07	p11392	ATPase family AAA domain-containing protein 2B
STK10 seq: 941 - 955 + Phospho (ST)	38324	37149	[illegible]	3E+08	p10821	Serine/threonine-protein kinase 10
LMO7 seq: 704 - 717 + Phospho (ST)	38324	37149	[illegible]	6E+07	p04876	LIM domain only protein 7
BAIAP2L1 pS261	38324	37149	[illegible]	4E+08	p13948	Brain-specific angiogenesis inhibitor 1-associated protein 2-like protein 1
MARCKSL1 seq: 44 - 77 + Phospho (ST)	38324	37149	[illegible]	25732	p16169	MARCKS-related protein
TJP1 pS617	38324	4E+06	[illegible]	8E+08	p12550	Tight junction protein ZO-1
DAZAP1 seq: 136 - 150 no mod	38324	37149	[illegible]	25732	p16717	DAZ-associated protein 1
FRMD6 pS525	38324	37149	[illegible]	5E+07	p15372	FERM domain-containing protein 6
CLTC seq: 1131 - 1155 no mod	38324	37149	[illegible]	6E+07	p20928	Clathrin heavy chain 1
CNDP2 seq: 54 - 66 + Gln->pyro-Glu (N-term Q)	38324	37149	[illegible]	6E+06	p08381	Cytosolic non-specific dipeptidase
AKAP13 seq: 772 - 809 + Phospho (ST)	38324	37149	[illegible]	25732	p17376	A-kinase anchor protein 13
SNRPG seq: 64 - 76 no mod	38324	37149	[illegible]	25732	p15569	Small nuclear ribonucleoprotein G
FEN1 seq: 246 - 252 no mod	2E+06	37149	[illegible]	25732	p19263	Flap endonuclease 1
FGD1 seq: 453 - 459 no mod	2E+06	37149	[illegible]	25732	p14281	FYVE, RhoGEF and PH domain-containing protein 1
FGD3 seq: 233 - 239 no mod	2E+06	37149	[illegible]	25732	p11819	FYVE, RhoGEF and PH domain-containing protein 3
VRK2 seq: 124 - 130 no mod	2E+06	37149	[illegible]	25732	p11761	Serine/threonine-protein kinase VRK2
MYL9 seq: 13 - 34 + Phospho (ST)	38324	37149	[illegible]	25732	p06872	Myosin regulatory light polypeptide 9
DBNL seq: 177 - 182 no mod	2E+06	37149	[illegible]	25732	p18288	Drebrin-like protein
HNRNPM seq: 518 - 524 no mod	38324	3E+06	[illegible]	2E+07	p10105	Heterogeneous nuclear ribonucleoprotein M
PLEC seq: 3095 - 3106 no mod	38324	37149	[illegible]	25732	p14165	Plectin
SF3A1 seq: 448 - 467 + Phospho (ST)	38324	37149	[illegible]	1E+08	p17549	Splicing factor 3A subunit 1
HMHA1 pS23	38324	37149	[illegible]	1E+08	p20562	Minor histocompatibility protein HA-1
CEP250 pS2229	38324	37149	[illegible]	25732	p11634	Centrosome-associated protein CEP250
SMS seq: 297 - 304 no mod	38324	37149	[illegible]	25732	p11584	Spermine synthase
ERBB2 seq: 1054 - 1096 + 2 Phospho (ST)	38324	1E+07	[illegible]	25732	p05759	Receptor tyrosine-protein kinase erbB-2
RASSF7 pS232	38324	37149	[illegible]	1E+08	p20291	Ras association domain-containing protein 7
PTK2 pS910	4E+07	37149	[illegible]	5E+08	p10960	Focal adhesion kinase 1
METTL16 pS329	38324	37149	[illegible]	2E+06	p17401	Methyltransferase-like protein 16
SAFB seq: 217 - 252 + Phospho (ST)	38324	5E+07	[illegible]	25732	p14083	Scaffold attachment factor B1
LMO7 seq: 975 - 1005 + 2 Phospho (ST)	38324	37149	[illegible]	1E+09	p17026	LIM domain only protein 7
PLEKHA6 seq: 470 - 475 + Phospho (Y)	38324	37149	[illegible]	1E+06	p13486	Pleckstrin homology domain-containing family A member 6
RASAL2 seq: 629 - 648 + Phospho (ST)	38324	37149	[illegible]	3E+08	p16428	Ras GTPase-activating protein nGAP
EPN3 pT495	38324	37149	[illegible]	25732	p20942	Epsin-3
ASAP1 seq: 765 - 796 + Phospho (ST)	38324	37149	[illegible]	8E+08	p10592	Arf-GAP with SH3 domain, ANK repeat and PH domain-containing protein 1
YAP1 pS131 pS138	38324	37149	[illegible]	4E+08	p20508	Transcriptional coactivator YAP1
NFKB1 seq: 897 - 912 + Phospho (ST)	38324	37149	[illegible]	2E+07	p02006	Nuclear factor NF-kappa-B p105 subunit
HIRIP3 pS330 pS332 pS333	1E+07	7E+06	[illegible]	25732	p07136	HIRA-interacting protein 3
WNK1 pS1978	38324	37149	[illegible]	3E+08	p13292	Serine/threonine-protein kinase WNK1
ELL2 seq: 499 - 531 + Phospho (ST)	38324	37149	[illegible]	2E+08	p11174	RNA polymerase II elongation factor ELL2
CCT4 seq: 97 - 122 no mod	38324	37149	[illegible]	25732	p19829	T-complex protein 1 subunit delta
NUMA1 seq: 1574 - 1588 no mod	38324	37149	[illegible]	25732	p19202	Nuclear mitotic apparatus protein 1
AP2B1 seq: 345 - 359 no mod	38324	37149	[illegible]	25732	p12178	AP-2 complex subunit beta
CDC42EP1 pS101	38324	37149	[illegible]	3E+08	p10239	Cdc42 effector protein 1
PHB seq: 220 - 239 no mod	38324	37149	[illegible]	25732	p21171	Prohibitin
AAK1 seq: 634 - 651 + Phospho (ST)	38324	37149	[illegible]	3E+08	p07267	AP2-associated protein kinase 1
CELSR1 seq: 2649 - 2655 no mod	38324	37149	[illegible]	25732	p15833	Cadherin EGF LAG seven-pass G-type receptor 1
KRT18 seq: 270 - 300 no mod	38324	37149	[illegible]	25732	p00013	Keratin, type I cytoskeletal 18
CTDSPL2 pS28	38324	37149	[illegible]	1E+08	p12335	CTD small phosphatase-like protein 2
SLC4A1AP pS312	38324	37149	[illegible]	1E+07	p12868	Kanadaptin
PARP1 seq: 453 - 467 no mod	38324	37149	[illegible]	25732	p00577	Poly [ADP-ribose] polymerase 1
CTNND1 seq: 899 - 915 + Phospho (Y)	38324	37149	[illegible]	1E+08	p05283	Catenin delta-1
NHSL1 seq: 288 - 319 + Phospho (ST)	38324	8E+06	[illegible]	6E+07	p15399	NHS-like protein 1
PSMC4 seq: 239 - 245 no mod	3E+06	37149	[illegible]	25732	p00265	26S protease regulatory subunit 6B
TJP2 pS913 pS916	38324	37149	[illegible]	1E+08	p09865	Tight junction protein ZO-2
IDH2 seq: 262 - 272 no mod	38324	37149	[illegible]	25732	p16655	Isocitrate dehydrogenase [NADP], mitochondrial
MYH9 seq: 1299 - 1322 no mod	38324	37149	[illegible]	25732	p10545	Myosin-9
TGFB1I1 pS68	38324	37149	[illegible]	3E+06	p04572	Transforming growth factor beta-1-induced transcript 1 protein
SCAF1 pS498 pS500	38324	37149	[illegible]	25732	p04404	Splicing factor, arginine/serine-rich 19
GOLGB1 seq: 2553 - 2563 + Gln->pyro-Glu (N-term Q)	38324	37149	[illegible]	25732	p08361	Golgin subfamily B member 1
ZNF593 seq: 116 - 134 + Phospho (ST)	38324	37149	[illegible]	3E+08	p07193	Zinc finger protein 593
TCOF1 pS503	38324	37149	[illegible]	2E+08	p04449	Treacle protein
PTOV1 seq: 62 - 87 + Phospho (ST)	38324	37149	[illegible]	1E+07	p01558	Prostate tumor-overexpressed gene 1 protein
MISP seq: 567 - 587 no mod	38324	37149	[illegible]	7E+07	p09771	Mitotic interactor and substrate of PLK1
KIF5B seq: 356 - 367 no mod	38324	37149	[illegible]	25732	p09022	Kinesin-1 heavy chain
NUMB seq: 224 - 263 + 2 Phospho (ST)	38324	37149	[illegible]	25732	p13521	Protein numb homolog
TCF3 pS359	38324	37149	[illegible]	25732	p20315	Transcription factor E2-alpha
AIP seq: 107 - 119 no mod	38324	37149	[illegible]	25732	p09574	AH receptor-interacting protein
LMO7 seq: 803 - 830 + Phospho (ST)	38324	37149	[illegible]	3E+08	p09803	LIM domain only protein 7
NUMB seq: 218 - 263 + 2 Phospho (ST)	38324	37149	[illegible]	3E+08	p13442	Protein numb homolog
SPAG9 seq: 327 - 361 + 2 Phospho (ST)	38324	37149	[illegible]	25732	p08938	C-Jun-amino-terminal kinase-interacting protein 4
FLNA seq: 1020 - 1032 no mod	38324	37149	[illegible]	25732	p01606	Filamin-A
RBM10 pS89	38324	37149	[illegible]	2E+08	p14663	RNA-binding protein 10
EHBP1L1 seq: 308 - 326 + Phospho (ST)	38324	37149	[illegible]	5E+08	p13168	EH domain-binding protein 1-like protein 1
MARCKSL1 seq: 111 - 144 + 2 Phospho (ST)	38324	37149	[illegible]	25732	p17751	MARCKS-related protein
PALLD pS893	38324	37149	[illegible]	2E+08	p14472	Palladin
APC seq: 2455 - 2476 + 2 Phospho (ST)	38324	37149	[illegible]	5E+07	p13006	Adenomatous polyposis coli protein
ARHGEF7 pS694	38324	37149	[illegible]	1E+08	p12742	Rho guanine nucleotide exchange factor 7
HNRNPK seq: 378 - 396 no mod	7E+07	2E+08	[illegible]	2E+08	p15138	Heterogeneous nuclear ribonucleoprotein K
USP43 pS1065	38324	37149	[illegible]	9E+07	p18077	Ubiquitin carboxyl-terminal hydrolase 43
MLLT4 pS1083	2E+07	3E+07	[illegible]	25732	p02080	Afadin
PPFIBP1 seq: 538 - 565 + Phospho (ST)	38324	37149	[illegible]	9E+08	p03054	Liprin-beta-1
DCP1A seq: 171 - 208 + Phospho (ST)	38324	37149	[illegible]	25732	p09221	mRNA-decapping enzyme 1A
ERRFI1 seq: 117 - 134 + Phospho (ST)	38324	37149	[illegible]	2E+08	p12882	ERBB receptor feedback inhibitor 1
TOMM34 seq: 110 - 127 no mod	38324	37149	[illegible]	2E+08	p01945	Mitochondrial import receptor subunit TOM34
DHX9 seq: 392 - 410 no mod	38324	37149	[illegible]	25732	p13250	ATP-dependent RNA helicase A
SLAIN2 seq: 85 - 119 + Phospho (ST)	38324	37149	[illegible]	25732	p06485	SLAIN motif-containing protein 2
TOM1 seq: 453 - 478 + Phospho (ST)	38324	37149	[illegible]	25732	p21184	Target of Myb protein 1
YWHAB seq: 196 - 214 no mod	38324	37149	[illegible]	4E+07	p03095	14-3-3 protein beta/alpha
GOLGA3 seq: 201 - 214 + Phospho (Y)	38324	37149	[illegible]	8E+07	p19447	Golgin subfamily A member 3
FSCN1 seq: 23 - 32 no mod	38324	37149	[illegible]	3E+07	p00230	Fascin
RAD23B seq: 145 - 173 + 2 Phospho (ST)	38324	37149	[illegible]	3E+07	p08738	UV excision repair protein RAD23 homolog B
NOSIP seq: 22 - 37 + Phospho (ST)	38324	37149	[illegible]	5E+07	p18193	Nitric oxide synthase-interacting protein
PGM3 seq: 75 - 104 no mod	38324	37149	[illegible]	25732	p10446	Phosphoacetylglucosamine mutase
EIF4G1 seq: 1185 - 1193 + Phospho (ST)	38324	37149	[illegible]	25732	p05842	Eukaryotic translation initiation factor 4 gamma 1
FAM129B pS641 pS646	38324	37149	[illegible]	5E+08	p07684	Niban-like protein 1
MAVS seq: 135 - 167 + 2 Phospho (ST)	38324	37149	[illegible]	25732	p17574	Mitochondrial antiviral-signaling protein
CRTC2 seq: 429 - 445 + Phospho (ST)	38324	37149	[illegible]	3E+07	p06458	CREB-regulated transcription coactivator 2
KRT7 seq: 87 - 96 no mod	38324	37149	[illegible]	1E+07	p00894	Keratin, type II cytoskeletal 7
SZRD1 pS105 pS107	38324	37149	[illegible]	4E+07	p14040	SUZ domain-containing protein 1
CALB2 seq: 162 - 170 no mod	38324	37149	[illegible]	5E+06	p10147	Calretinin
G6PD seq: 408 - 427 no mod	38324	37149	[illegible]	25732	p12794	Glucose-6-phosphate 1-dehydrogenase
DBR1 pS514	38324	37149	[illegible]	6E+06	p07146	Lariat debranching enzyme
LAMA3 seq: 2669 - 2678 no mod	38324	37149	[illegible]	3E+08	p15041	Laminin subunit alpha-3
PROSER2 seq: 283 - 315 + Phospho (ST)	38324	37149	[illegible]	25732	p19730	Proline and serine-rich protein 2
UBA1 seq: 636 - 649 no mod	38324	37149	[illegible]	25732	p09637	Ubiquitin-like modifier-activating enzyme 1
FLNA seq: 725 - 738 no mod	38324	37149	[illegible]	3E+07	p18463	Filamin-A
HDGF pS132 pS133	38324	37149	[illegible]	25732	p15600	Hepatoma-derived growth factor
PSMA2 seq: 40 - 51 no mod	38324	37149	[illegible]	25732	p21085	Proteasome subunit alpha type-2
FOSL2 pS200	38324	4E+06	[illegible]	2E+08	p06645	Fos-related antigen 2
KIF16B seq: 1045 - 1075 + Phospho (ST)	38324	37149	[illegible]	25732	p17267	Kinesin-like protein KIF16B
TUBB4B seq: 78 - 103 no mod	38324	37149	[illegible]	25732	p05712	Tubulin beta-4B chain
DDX5 seq: 178 - 192 no mod	38324	37149	[illegible]	25732	p17536	Probable ATP-dependent RNA helicase DDX5
STIP1 seq: 14 - 32 no mod	38324	37149	[illegible]	25732	p20142	Stress-induced-phosphoprotein 1
PDLIM7 seq: 259 - 278 no mod	38324	37149	[illegible]	7E+07	p02173	PDZ and LIM domain protein 7
AMBP seq: 167 - 202 no mod	38324	37149	[illegible]	25732	p00656	Protein AMBP
MAP1B seq: 1963 - 1979 + Phospho (ST)	38324	7E+06	[illegible]	3E+08	p02009	Microtubule-associated protein 1B
TOM1 seq: 457 - 478 + Phospho (ST)	38324	37149	[illegible]	25732	p11104	Target of Myb protein 1
PTBP1 seq: 65 - 84 no mod	38324	37149	[illegible]	25732	p12948	Polypyrimidine tract-binding protein 1
SIPA1L3 seq: 22 - 76 + Phospho (ST)	38324	37149	[illegible]	25732	p01508	Signal-induced proliferation-associated 1-like protein 3
CIRBP seq: 8 - 28 no mod	38324	37149	[illegible]	25732	p11853	Cold-inducible RNA-binding protein
PRCC pS212	410721	5E+06	[illegible]	25732	p12741	Proline-rich protein PRCC
DLGAP5 seq: 796 - 820 + Phospho (ST)	38324	37149	[illegible]	1E+08	p04940	Disks large-associated protein 5
THRAP3 seq: 566 - 572 no mod	38324	37149	[illegible]	4E+06	p17922	Thyroid hormone receptor-associated protein 3
ARID1B seq: 1165 - 1217 + Phospho (ST)	38324	37149	[illegible]	25732	p10961	AT-rich interactive domain-containing protein 1B
FARSA seq: 43 - 56 no mod	38324	37149	[illegible]	25732	p05091	Phenylalanine--tRNA ligase alpha subunit
EPN1 seq: 446 - 499 + 2 Phospho (ST)	38324	37149	[illegible]	25732	p15256	Epsin-1
NAV2 pS1245	38324	37149	[illegible]	2E+07	p06331	Neuron navigator 2
STK10 pS417	38324	37149	[illegible]	2E+08	p04080	Serine/threonine-protein kinase 10
RUSC2 pS536	38324	37149	[illegible]	1E+08	p10818	Iporin
STMN1 pS16	6E+06	37149	[illegible]	8E+08	p07582	Stathmin
NFRKB pS298	38324	37149	[illegible]	25732	p13158	Nuclear factor related to kappa-B-binding protein
BAIAP2L1 seq: 239 - 266 + 2 Phospho (ST)	38324	37149	[illegible]	3E+08	p13956	Brain-specific angiogenesis inhibitor 1-associated protein 2-like protein 1
GSDMD seq: 250 - 267 + Phospho (ST)	38324	37149	[illegible]	1E+09	p03467	Gasdermin-D
ACTN4 seq: 675 - 691 no mod	38324	37149	[illegible]	2E+07	p13729	Alpha-actinin-4
SSB seq: 61 - 74 no mod	38324	37149	[illegible]	25732	p10483	Lupus La protein
SF3B2 seq: 149 - 163 no mod	38324	37149	[illegible]	25732	p12197	Splicing factor 3B subunit 2
KRT8 seq: 226 - 252 no mod	38324	37149	[illegible]	25732	p08254	Keratin, type II cytoskeletal 8
FLNA pS2152	38324	37149	[illegible]	1E+08	p19879	Filamin-A
PHLDB2 pS387	38324	37149	[illegible]	1E+08	p09629	Pleckstrin homology-like domain family B member 2
CORO1C seq: 224 - 239 no mod	38324	37149	[illegible]	1E+07	p20478	Coronin-1C
TJP1 seq: 1613 - 1637 + Phospho (ST)	38324	37149	[illegible]	6E+07	p20439	Tight junction protein ZO-1
GART seq: 5 - 13 no mod	38324	37149	[illegible]	4E+06	p01222	Trifunctional purine biosynthetic protein adenosine-3
RPS26P11 seq: 16 - 22 no mod	38324	6E+06	[illegible]	1E+08	p15921	Putative 40S ribosomal protein S26-like 1
TACO1 seq: 232 - 243 no mod	38324	37149	[illegible]	25732	p16698	Translational activator of cytochrome c oxidase 1
CLTC seq: 1074 - 1094 no mod	38324	37149	[illegible]	25732	p13255	Clathrin heavy chain 1
CDC42EP3 seq: 140 - 152 + Phospho (ST)	38324	37149	[illegible]	1E+08	p11083	Cdc42 effector protein 3
VASP seq: 362 - 375 no mod	38324	37149	[illegible]	25732	p01330	Vasodilator-stimulated phosphoprotein
NOC2L pS672 pS673	38324	37149	[illegible]	4E+08	p18620	Nucleolar complex protein 2 homolog
SRRM1 pS638	38324	7E+06	[illegible]	25732	p00849	Serine/arginine repetitive matrix protein 1
WASH6P pS327	38324	37149	[illegible]	1E+07	p08778	WAS protein family homolog 6
ANXA1 seq: 30 - 53 no mod	38324	37149	[illegible]	9E+08	p15995	Annexin A1
PTMA seq: 91 - 104 no mod	38324	37149	[illegible]	25732	p21179	Prothymosin alpha
PKM seq: 401 - 422 + Phospho (ST)	38324	37149	[illegible]	9E+07	p12213	Pyruvate kinase PKM
HNRNPH1 seq: 263 - 275 no mod	38324	37149	[illegible]	25732	p18568	Heterogeneous nuclear ribonucleoprotein H
RBM15B seq: 606 - 614 + Phospho (ST)	38324	37149	[illegible]	7E+07	p02049	Putative RNA-binding protein 15B
TDRD3 seq: 425 - 439 no mod	38324	37149	[illegible]	25732	p17332	Tudor domain-containing protein 3
ACTN4 seq: 653 - 668 no mod	38324	37149	[illegible]	25732	p08699	Alpha-actinin-4
ITGB1BP1 seq: 49 - 64 no mod	38324	37149	[illegible]	25732	p03885	Integrin beta-1-binding protein 1
CALU seq: 288 - 311 no mod	38324	37149	[illegible]	25732	p17838	Calumenin
SLK pS779	38324	37149	[illegible]	1E+08	p02672	STE20-like serine/threonine-protein kinase
LAP3 seq: 295 - 303 no mod	38324	37149	[illegible]	25732	p19653	Cytosol aminopeptidase
MVP seq: 862 - 893 + Phospho (ST)	38324	37149	[illegible]	1E+09	p01763	Major vault protein
ANXA3 seq: 280 - 288 no mod	38324	37149	[illegible]	5E+07	p06023	Annexin A3
ANXA1 seq: 114 - 124 no mod	38324	37149	[illegible]	5E+08	p02425	Annexin A1
GOLGA3 seq: 51 - 105 + Phospho (ST)	38324	37149	[illegible]	25732	p19452	Golgin subfamily A member 3
FLNA seq: 2585 - 2598 no mod	38324	37149	[illegible]	25732	p20608	Filamin-A
ARHGAP32 seq: 1555 - 1566 + Phospho (Y)	38324	37149	[illegible]	1E+08	p01579	Rho GTPase-activating protein 32
HSP90B1 seq: 244 - 265 no mod	38324	37149	[illegible]	25732	p15068	Endoplasmin
HSPA4 seq: 574 - 591 no mod	38324	37149	[illegible]	25732	p02070	Heat shock 70 kDa protein 4
YAP1 seq: 205 - 252 + Phospho (ST)	38324	37149	[illegible]	25732	p20081	Transcriptional coactivator YAP1
MAP1B pS1265	38324	37149	[illegible]	5E+08	p04411	Microtubule-associated protein 1B
EIF6 seq: 140 - 164 no mod	38324	37149	[illegible]	25732	p07759	Eukaryotic translation initiation factor 6
NCL seq: 142 - 176 + 2 Phospho (ST)	38324	37149	[illegible]	25732	p13311	Nucleolin
HSD17B4 seq: 33 - 46 no mod	38324	37149	[illegible]	25732	p16288	Peroxisomal multifunctional enzyme type 2
SNX29 seq: 439 - 471 + Phospho (ST)	38324	37149	[illegible]	25732	p00060	Sorting nexin-29
WDR70 pS638	38324	37149	[illegible]	8E+07	p02504	WD repeat-containing protein 70
USP10 seq: 208 - 237 + 2 Phospho (ST)	38324	37149	[illegible]	2E+08	p02987	Ubiquitin carboxyl-terminal hydrolase 10
C2CD3 seq: 315 - 326 no mod	38324	37149	[illegible]	25732	p18580	C2 domain-containing protein 3
GSTM3 seq: 178 - 186 no mod	38324	37149	[illegible]	25732	p19043	Glutathione S-transferase Mu 3
APRT seq: 146 - 163 no mod	38324	37149	[illegible]	25732	p11048	Adenine phosphoribosyltransferase

ARHGAP32 seq: 2014 - 2037 + Phospho (Y)	38324	37149	39656	13654	33854	3E+07	25552	34892	2E+07	4E+06	56234	53206	4E+07	1E+07	2E+07	2E+07	33072	34984	19162	28832	39759	50401	21442	19024	42741	68554	2E+08	1E+08	3E+08	3E+08	3E+08	2E+08	9E+07	2E+08	2E+08	6E+08	3E+08	2E+08	3E+08	36936	5E+08	2E+08	2E+08	2E+08	2E+08	3E+08	1E+08	2E+08	3E+08	3E+08	p07828	Rho GTPase-activating protein 32
CIT pS480	38324	37149	39656	13654	33854	2E+08	1E+07	34892	2E+07	26736	2E+08	53206	39754	40879	18798	40867	33072	34984	6E+06	28832	39759	50401	21442	19024	42741	9E+06	44396	34144	19500	29103	45838	31655	19257	24890	17227	44771	27262	25062	27887	36936	100327	64618	38440	41974	13188	134692	41467	25947	30272	25732	p06447	Citron Rho-interacting kinase
EEF1A2 seq: 267 - 290 no mod	38324	37149	39656	13654	33854	7E+07	25552	34892	34104	26736	2E+08	8E+06	39754	40879	18798	4E+07	33072	34984	19162	28832	39759	50401	21442	19024	42741	68554	44396	34144	19500	29103	45838	31655	19257	24890	17227	7E+06	27262	25062	27887	36936	100327	64618	38440	41974	13188	134692	41467	25947	30272	25732	p01593	Elongation factor 1-alpha 2
ATXN2L pS111	38324	37149	39656	13654	33854	45779	25552	34892	2E+07	26736	2E+08	53206	39754	2E+07	2E+07	40867	33072	34984	19162	28832	39759	50401	21442	3E+07	42741	4E+08	9E+07	34144	1E+08	29103	6E+07	5E+07	19257	1E+08	1E+08	7E+07	7E+07	1E+07	1E+08	2E+08	1E+08	9E+07	2E+07	1E+08	1E+08	2E+08	41467	25947	2E+08	1E+08	p15371	Ataxin-2-like protein
PSMA5 seq: 21 - 32 no mod	38324	37149	39656	13654	33854	4E+07	25552	34892	34104	4E+06	2E+08	53206	39754	40879	2E+06	9E+06	33072	34984	19162	28832	39759	50401	21442	19024	42741	1E+07	44396	34144	19500	29103	7E+06	31655	19257	24890	17227	3E+07	27262	25062	27887	36936	9E+06	64618	38440	41974	13188	3E+07	41467	25947	30272	25732	p11868	Proteasome subunit alpha type-5
NPM1 seq: 55 - 80 + Oxidation (M); Phospho (ST)	38324	37149	39656	13654	33854	2E+08	25552	8E+06	34104	26736	1E+08	3E+07	39754	40879	18798	1E+07	33072	34984	19162	28832	39759	50401	21442	19024	42741	1E+08	44396	34144	2E+07	2E+07	5E+07	31655	19257	4E+07	17227	1E+08	27262	25062	2E+07	1E+07	7E+07	2E+07	38440	2E+07	2E+07	4E+07	41467	3E+07	7E+07	2E+07	p18890	Nucleophosmin
AP1G1 seq: 365 - 379 no mod	38324	37149	39656	13654	33854	45779	25552	34892	34104	7E+06	6E+08	1E+08	39754	40879	4E+06	3E+07	33072	34984	19162	28832	39759	50401	21442	19024	42741	68554	44396	34144	19500	29103	45838	31655	19257	24890	17227	44771	27262	25062	27887	36936	100327	64618	38440	41974	13188	134692	41467	25947	30272	25732	p20094	AP-1 complex subunit gamma-1
HPRT1 seq: 116 - 128 no mod	38324	37149	39656	13654	33854	1E+07	25552	1E+07	34104	26736	8E+07	53206	39754	40879	18798	2E+07	33072	34984	19162	28832	39759	50401	21442	19024	42741	5E+07	2E+07	3E+07	1E+07	29103	1E+08	1E+07	3E+07	2E+07	2E+07	2E+08	5E+07	3E+07	3E+07	3E+07	5E+07	2E+07	5E+07	3E+07	3E+07	2E+08	3E+07	5E+07	3E+07	3E+07	p01396	Hypoxanthine-guanine phosphoribosyltransferase
IRF2BP2 seq: 13 - 23 + Phospho (ST)	3E+06	37149	39656	13654	33854	1E+08	4E+07	1E+07	2E+07	1E+07	9E+07	2E+07	39754	2E+07	18798	3E+07	1E+07	34984	7E+06	28832	6E+07	9E+06	1E+07	2E+07	5E+06	68554	44396	34144	19500	29103	45838	2E+06	19257	24890	17227	44771	27262	25062	27887	36936	100327	64618	38440	41974	13188	5E+06	41467	25947	3E+06	25732	p06804	Interferon regulatory factor 2-binding protein 2
LAD1 pS38	38324	37149	39656	13654	33854	45779	25552	34892	34104	26736	4E+07	1E+07	1E+07	1E+07	7E+06	8E+06	5E+06	34984	4E+06	28832	39759	4E+06	21442	19024	7E+06	1E+08	5E+07	6E+07	6E+07	7E+07	1E+08	1E+08	7E+07	8E+07	1E+08	7E+07	2E+08	8E+07	7E+07	1E+08	100327	1E+08	6E+07	8E+07	1E+08	1E+08	2E+08	1E+08	1E+08	1E+08	p09400	Ladinin-1
MATR3 seq: 121 - 146 no mod	38324	37149	39656	13654	33854	3E+08	25552	34892	34104	26736	9E+07	7E+06	39754	40879	18798	3E+07	33072	34984	19162	28832	39759	50401	21442	19024	42741	68554	44396	34144	19500	29103	45838	31655	19257	24890	17227	44771	27262	25062	27887	36936	100327	64618	38440	41974	13188	134692	41467	25947	30272	25732	p18640	Matrin-3
FKBP15 seq: 1082 - 1124 + Phospho (ST)	38324	37149	39656	13654	33854	1E+08	25552	34892	34104	4E+07	56234	5E+07	39754	40879	18798	40867	33072	34984	19162	28832	39759	50401	4E+07	19024	42741	2E+08	6E+07	6E+07	1E+08	3E+07	7E+07	7E+07	19257	8E+07	1E+08	9E+07	7E+07	5E+07	5E+07	7E+07	1E+08	1E+08	5E+07	6E+07	13188	1E+08	6E+07	1E+08	1E+08	25732	p17018	FK506-binding protein 15
FLNB seq: 2105 - 2120 no mod	38324	37149	39656	13654	33854	2E+08	25552	34892	34104	26736	3E+08	53206	39754	40879	18798	40867	33072	34984	19162	28832	39759	50401	21442	19024	42741	68554	44396	34144	19500	29103	45838	31655	19257	24890	17227	44771	27262	25062	27887	36936	100327	64618	38440	41974	13188	3E+07	41467	25947	30272	25732	p19876	Filamin-B
PXN seq: 148 - 193 + 2 Phospho (ST)	38324	37149	39656	13654	33854	45779	25552	34892	4E+07	26736	56234	53206	4E+07	40879	3E+07	40867	33072	34984	4E+07	28832	39759	50401	21442	19024	42741	68554	2E+07	1E+08	2E+08	2E+08	45838	31655	19257	24890	3E+08	1E+07	27262	4E+08	2E+08	3E+08	3E+07	1E+08	4E+08	41974	2E+08	2E+08	41467	4E+08	2E+08	25732	p11360	Paxillin
ETFB seq: 165 - 174 no mod	38324	37149	39656	13654	33854	1E+08	25552	34892	34104	26736	5E+08	53206	1E+08	40879	18798	40867	33072	34984	19162	28832	39759	50401	21442	19024	42741	68554	44396	34144	19500	29103	1E+07	31655	19257	24890	17227	44771	4E+06	25062	27887	36936	100327	64618	38440	41974	13188	134692	41467	25947	30272	25732	p17631	Electron transfer flavoprotein subunit beta
ERRFI1 seq: 117 - 148 + 2 Phospho (ST)	38324	37149	39656	13654	33854	2E+08	25552	34892	34104	26736	1E+08	53206	39754	40879	18798	40867	33072	34984	19162	28832	39759	50401	21442	19024	5E+06	4E+08	1E+08	34144	4E+08	3E+08	3E+08	1E+08	19257	8E+08	5E+08	5E+08	8E+07	25062	5E+08	6E+08	6E+08	4E+08	38440	6E+08	6E+08	7E+08	1E+08	25947	1E+09	5E+08	p12880	ERBB receptor feedback inhibitor 1
GAPVD1 seq: 583 - 631 + 2 Phospho (ST)	38324	37149	39656	13654	33854	1E+08	25552	34892	8E+07	26736	56234	53206	39754	40879	18798	40867	33072	34984	19162	28832	39759	50401	21442	8E+07	42741	68554	44396	34144	3E+07	29103	45838	31655	19257	8E+07	17227	44771	27262	25062	27887	1E+08	100327	64618	38440	41974	5E+07	134692	41467	25947	1E+08	7E+07	p04752	GTPase-activating protein and VPS9 domain-containing protein 1
HNRNPD seq: 198 - 218 no mod	38324	37149	39656	13654	33854	45779	7E+06	34892	34104	26736	4E+08	4E+07	39754	40879	18798	40867	33072	34984	19162	28832	39759	50401	21442	3E+06	42741	68554	44396	34144	19500	29103	45838	31655	19257	24890	17227	44771	27262	25062	27887	36936	100327	64618	38440	41974	2E+08	134692	41467	25947	30272	25732	p13773	Heterogeneous nuclear ribonucleoprotein D0
POLE3 seq: 122 - 147 no mod	38324	37149	39656	13654	33854	2E+08	3E+07	34892	34104	26736	2E+08	4E+07	39754	40879	18798	40867	2E+07	34984	19162	28832	39759	50401	21442	19024	42741	3E+08	44396	4E+07	19500	29103	1E+08	31655	19257	24890	17227	2E+08	5E+07	3E+07	27887	36936	3E+08	1E+08	3E+07	41974	13188	3E+08	8E+07	25947	30272	25732	p04133	DNA polymerase epsilon subunit 3
MYH14 seq: 217 - 232 no mod	38324	37149	39656	13654	33854	1E+08	2E+07	34892	34104	26736	2E+08	4E+07	39754	40879	18798	40867	33072	34984	19162	28832	39759	50401	21442	19024	42741	68554	44396	34144	19500	29103	45838	31655	19257	24890	17227	44771	27262	25062	27887	36936	100327	64618	38440	41974	13188	6E+07	41467	25947	30272	25732	p08352	Myosin-14
KRT18 seq: 265 - 300 no mod	38324	37149	39656	13654	33854	8E+07	25552	34892	34104	26736	2E+08	1E+08	39754	40879	18798	7E+07	33072	34984	19162	28832	39759	50401	21442	19024	42741	68554	44396	34144	19500	29103	45838	31655	19257	24890	17227	44771	27262	25062	27887	36936	100327	64618	38440	41974	13188	134692	41467	25947	30272	25732	p17834	Keratin, type I cytoskeletal 18
RPS5 seq: 168 - 182 no mod	38324	37149	39656	13654	33854	2E+08	3E+06	34892	34104	26736	6E+07	5E+06	39754	40879	18798	2E+07	33072	34984	19162	28832	39759	50401	21442	19024	42741	2E+07	44396	34144	19500	29103	1E+07	31655	19257	4E+06	17227	2E+07	27262	25062	1E+07	1E+06	1E+07	64618	38440	41974	3E+06	2E+07	41467	1E+06	2E+06	25732	p02735	40S ribosomal protein S5
HIVEP1 pS1180	38324	37149	39656	13654	33854	45779	2E+08	9E+06	34104	26736	56234	53206	8E+07	40879	18798	40867	4E+07	34984	19162	28832	39759	50401	21442	19024	42741	68554	44396	34144	19500	29103	45838	31655	19257	24890	17227	44771	27262	25062	27887	36936	100327	64618	38440	41974	13188	134692	41467	25947	30272	25732	p01395	Zinc finger protein 40
ATP5B seq: 325 - 345 no mod	38324	37149	39656	13654	33854	7E+07	25552	34892	34104	26736	6E+08	53206	2E+08	40879	18798	40867	33072	34984	19162	28832	39759	50401	21442	19024	42741	68554	44396	34144	19500	29103	45838	31655	19257	24890	17227	44771	27262	25062	27887	36936	100327	64618	38440	41974	13188	5E+07	41467	25947	30272	25732	p13860	ATP synthase subunit beta, mitochondrial
ASF1A seq: 149 - 184 + Phospho (ST)	38324	37149	39656	13654	33854	2E+08	6E+07	34892	34104	26736	3E+08	1E+08	39754	40879	18798	40867	33072	34984	19162	28832	39759	50401	21442	19024	42741	68554	44396	34144	19500	29103	45838	31655	19257	24890	17227	3E+07	27262	25062	27887	36936	100327	64618	38440	41974	13188	134692	41467	25947	30272	25732	p16707	Histone chaperone ASF1A
DIP2B seq: 254 - 272 + Phospho (ST)	38324	37149	39656	13654	1E+07	8E+07	4E+07	3E+07	34104	26736	9E+07	3E+07	3E+07	5E+07	18798	6E+07	33072	4E+07	3E+07	3E+07	7E+07	50401	2E+07	5E+07	42741	68554	44396	34144	19500	29103	45838	31655	19257	24890	17227	3E+07	27262	25062	8E+06	36936	100327	64618	38440	41974	13188	2E+07	41467	25947	30272	25732	p15238	Disco-interacting protein 2 homolog B
GCN1L1 seq: 1407 - 1414 no mod	38324	37149	39656	13654	33854	3E+07	25552	34892	34104	26736	3E+08	53206	39754	40879	18798	40867	33072	34984	19162	28832	1E+07	50401	21442	19024	42741	68554	44396	34144	19500	29103	1E+07	31655	19257	24890	17227	2E+06	27262	25062	27887	36936	100327	64618	38440	41974	13188	134692	41467	25947	30272	25732	p15742	Translational activator GCN1
CLTB seq: 123 - 129 no mod	38324	37149	39656	13654	33854	45779	25552	34892	34104	1E+06	8E+07	53206	1E+07	5E+06	18798	40867	33072	34984	8E+06	28832	39759	50401	21442	19024	3E+06	68554	44396	34144	19500	1E+06	2E+07	31655	19257	24890	1E+06	4E+07	4E+06	25062	27887	36936	100327	64618	38440	41974	2E+06	8E+07	41467	25947	30272	25732	p00732	Clathrin light chain B
CACTIN seq: 468 - 492 + Phospho (ST)	38324	37149	39656	13654	33854	2E+08	25552	34892	34104	26736	2E+08	5E+07	39754	40879	18798	40867	33072	6E+07	19162	28832	39759	50401	21442	19024	42741	6E+08	2E+08	2E+08	4E+08	2E+08	3E+08	2E+08	6E+07	4E+08	4E+08	5E+08	3E+08	2E+08	3E+08	3E+08	4E+08	2E+08	3E+08	3E+08	3E+08	4E+08	1E+08	3E+08	4E+08	3E+08	p11495	Cactin
RPS19 seq: 85 - 94 no mod	38324	37149	39656	13654	33854	45779	25552	34892	34104	26736	2E+08	53206	2E+06	40879	4E+06	3E+06	6E+06	34984	19162	28832	5E+06	4E+06	21442	19024	42741	2E+07	44396	34144	19500	29103	3E+07	1E+06	19257	24890	2E+06	3E+07	27262	25062	27887	2E+06	2E+07	4E+06	38440	2E+06	5E+06	7E+07	3E+06	25947	4E+06	25732	p09587	40S ribosomal protein S19
NONO pM384	38324	37149	39656	13654	33854	4E+08	2E+07	34892	34104	26736	4E+08	3E+07	39754	40879	18798	1E+07	33072	34984	19162	28832	39759	50401	21442	19024	42741	1E+08	44396	34144	19500	29103	4E+07	31655	19257	24890	17227	7E+07	27262	6E+07	27887	36936	9E+07	64618	1E+08	41974	13188	9E+07	41467	25947	30272	25732	p10086	Non-POU domain-containing octamer-binding protein
CDV3 seq: 118 - 136 no mod	38324	37149	39656	13654	33854	1E+08	25552	34892	34104	26736	7E+07	53206	39754	40879	18798	40867	33072	34984	19162	28832	39759	50401	9E+06	19024	42741	68554	44396	34144	19500	29103	45838	31655	19257	24890	17227	44771	27262	25062	27887	36936	2E+07	64618	38440	41974	13188	1E+07	41467	25947	30272	25732	p08760	Protein CDV3 homolog
AUH seq: 270 - 276 no mod	38324	753634	39656	13654	891987	1E+08	25552	3E+06	34104	5E+07	1E+09	53206	39754	40879	3E+07	691132	33072	34984	19162	28832	7E+07	50401	2E+06	19024	8E+07	6E+07	443957	388937	19500	29103	3E+08	31655	19257	24890	2E+07	3E+08	27262	25062	27887	36936	5E+07	64618	38440	41974	890951	4E+08	41467	259471	30272	25732	p13433	Methylglutaconyl-CoA hydratase, mitochondrial
OSBP seq: 238 - 254 + Phospho (ST)	38324	37149	39656	13654	33854	8E+08	25552	34892	34104	26736	9E+08	2E+08	1E+08	40879	18798	40867	33072	34984	19162	28832	39759	50401	21442	19024	2E+07	2E+08	3E+07	5E+07	8E+07	29103	1E+08	31655	5E+06	9E+07	9E+07	1E+08	4E+07	3E+07	6E+07	2E+07	3E+08	64618	7E+07	41974	13188	1E+08	2E+07	7E+07	30272	25732	p05066	Oxysterol-binding protein 1
SCRIB pS939	38324	37149	39656	13654	33854	2E+08	25552	34892	34104	26736	3E+08	2E+07	39754	40879	18798	5E+07	33072	34984	19162	28832	39759	50401	21442	19024	42741	6E+06	44396	34144	3E+07	1E+07	6E+06	31655	19257	4E+07	1E+07	3E+07	27262	25062	5E+06	2E+07	100327	1E+07	4E+06	3E+07	2E+07	2E+07	41467	1E+07	3E+07	25732	p14866	Protein scribble homolog
PPP1R10 pS591	38324	37149	39656	13654	33854	2E+08	25552	34892	34104	26736	2E+08	2E+07	39754	9E+06	18798	40867	33072	34984	19162	28832	39759	50401	21442	6E+06	42741	68554	44396	34144	19500	29103	45838	31655	19257	4E+06	17227	44771	27262	25062	27887	36936	100327	64618	38440	41974	13188	134692	41467	25947	30272	25732	p15457	Serine/threonine-protein phosphatase 1 regulatory subunit 10
UBA1 seq: 486 - 510 no mod	38324	37149	39656	13654	33854	2E+08	25552	34892	34104	26736	2E+08	2E+08	39754	3E+07	18798	4E+07	33072	34984	19162	28832	39759	50401	21442	19024	42741	68554	44396	34144	19500	7E+06	45838	31655	19257	1E+07	17227	44771	9E+06	3E+07	2E+07	36936	100327	64618	3E+07	41974	2E+07	134692	41467	25947	30272	25732	p09659	Ubiquitin-like modifier-activating enzyme 1
HSD17B4 seq: 85 - 92 no mod	38324	37149	39656	13654	33854	2E+07	4E+07	1E+07	34104	26736	3E+08	6E+06	39754	40879	18798	40867	33072	34984	19162	28832	39759	2E+06	21442	19024	2E+06	8E+06	44396	34144	19500	29103	7E+07	31655	19257	24890	17227	2E+07	2E+06	25062	27887	36936	100327	64618	38440	41974	13188	134692	41467	25947	30272	25732	p03081	Peroxisomal multifunctional enzyme type 2
PPP1R13L seq: 186 - 235 + Phospho (ST)	38324	37149	39656	13654	33854	45779	3E+07	34892	34104	26736	1E+08	1E+08	39754	40879	18798	7E+07	33072	34984	19162	28832	39759	50401	21442	19024	42741	9E+07	44396	34144	19500	29103	45838	31655	19257	3E+07	17227	1E+08	27262	25062	27887	36936	8E+07	64618	38440	8E+07	13188	6E+07	41467	25947	9E+07	25732	p15221	RelA-associated inhibitor
ACTG1 seq: 291 - 312 no mod	38324	37149	39656	13654	33854	45779	25552	34892	34104	26736	5E+08	53206	8E+07	40879	18798	40867	33072	34984	19162	28832	39759	50401	21442	19024	42741	68554	44396	34144	19500	29103	45838	31655	19257	24890	17227	2E+08	27262	25062	27887	36936	100327	64618	38440	41974	13188	1E+08	41467	25947	30272	25732	p13335	Actin, cytoplasmic 2
PARD3 seq: 142 - 168 + Phospho (ST)	38324	37149	39656	13654	33854	45779	25552	34892	1E+08	26736	56234	53206	39754	40879	18798	40867	3E+07	34984	19162	28832	39759	50401	21442	19024	42741	9E+07	44396	34144	19500	29103	1E+07	2E+08	19257	24890	17227	7E+07	27262	25062	3E+08	4E+08	3E+08	4E+08	38440	41974	2E+08	134692	41467	25947	1E+08	25732	p06622	Partitioning defective 3 homolog
MDH2 seq: 27 - 45 no mod	38324	37149	39656	13654	33854	4E+08	25552	34892	34104	26736	1E+08	8E+06	39754	40879	18798	40867	33072	34984	19162	28832	1E+07	50401	21442	19024	42741	68554	44396	34144	19500	29103	1E+07	31655	19257	24890	17227	44771	27262	25062	27887	36936	100327	64618	38440	41974	13188	134692	41467	25947	30272	25732	p01734	Malate dehydrogenase, mitochondrial
EIF4G1 seq: 525 - 535 no mod	38324	37149	39656	13654	33854	8E+06	25552	3E+06	34104	26736	7E+07	53206	39754	40879	18798	40867	33072	34984	19162	28832	39759	50401	21442	19024	42741	68554	44396	34144	19500	29103	5E+06	31655	19257	24890	17227	1E+07	27262	25062	27887	36936	3E+06	64618	2E+06	41974	13188	1E+07	41467	5E+06	30272	25732	p17905	Eukaryotic translation initiation factor 4 gamma 1
TPM4 seq: 105 - 113 no mod	38324	37149	39656	13654	33854	45779	3E+07	8E+06	34104	26736	9E+08	53206	5E+07	1E+07	6E+06	40867	33072	34984	19162	28832	39759	50401	21442	6E+06	7E+06	68554	44396	34144	19500	2E+07	45838	31655	1E+07	24890	17227	3E+07	27262	1E+07	27887	36936	100327	64618	2E+07	41974	13188	2E+08	41467	2E+07	5E+06	6E+06	p10185	Tropomyosin alpha-4 chain
TPM2 seq: 141 - 149 no mod	38324	37149	39656	13654	33854	45779	3E+07	8E+06	34104	26736	9E+08	53206	5E+07	1E+07	6E+06	40867	33072	34984	19162	28832	39759	50401	21442	6E+06	7E+06	68554	44396	34144	19500	2E+07	45838	31655	1E+07	24890	17227	3E+07	27262	1E+07	27887	36936	100327	64618	2E+07	41974	13188	2E+08	41467	2E+07	5E+06	6E+06	p10182	Tropomyosin beta chain
TCOF1 pS446	38324	37149	39656	13654	33854	4E+07	25552	34892	34104	6E+06	997174	53206	39754	40879	8E+06	40867	33072	34984	19162	2E+06	39759	50401	21442	19024	42741	5E+07	44396	34144	2E+06	29103	2E+08	1E+07	19257	7E+06	8E+07	7E+08	27262	2E+07	3E+06	8E+07	1E+09	4E+07	38440	1E+08	2E+07	7E+08	41467	4E+07	7E+06	25732	p21055	Treacle protein
SF3A1 seq: 431 - 437 no mod	38324	37149	39656	13654	33854	45779	25552	34892	34104	26736	2E+08	53206	6E+06	40879	18798	40867	33072	34984	5E+06	28832	39759	50401	21442	19024	42741	68554	44396	34144	19500	29103	45838	31655	19257	24890	17227	7E+07	27262	25062	27887	36936	100327	64618	38440	41974	13188	9E+07	41467	25947	30272	25732	p14255	Splicing factor 3A subunit 1
BUB3 seq: 150 - 157 no mod	38324	37149	39656	13654	33854	5E+07	25552	34892	34104	26736	5E+08	53206	39754	40879	18798	2E+06	33072	34984	19162	28832	6E+06	3E+06	21442	19024	42741	68554	44396	34144	19500	29103	4E+07	31655	19257	24890	17227	44771	27262	25062	27887	36936	100327	64618	38440	41974	13188	134692	41467	25947	30272	25732	p11537	Mitotic checkpoint protein BUB3
FAM182A seq: 114 - 119 no mod	38324	37149	39656	13654	33854	5E+07	25552	34892	34104	26736	5E+08	53206	39754	40879	18798	2E+06	33072	34984	19162	28832	6E+06	3E+06	21442	19024	42741	68554	44396	34144	19500	29103	4E+07	31655	19257	24890	17227	44771	27262	25062	27887	36936	100327	64618	38440	41974	13188	134692	41467	25947	30272	25732	p11248	Protein FAM182A
NONO seq: 257 - 270 no mod	38324	37149	39656	13654	33854	5E+07	25552	34892	34104	26736	5E+08	53206	5E+07	40879	18798	40867	33072	34984	19162	28832	39759	50401	21442	19024	42741	68554	44396	5E+06	19500	29103	5E+07	31655	3E+06	24890	17227	1E+08	27262	25062	27887	1E+07	100327	64618	1E+07	41974	1E+07	1E+08	41467	25947	30272	1E+07	p16855	Non-POU domain-containing octamer-binding protein
HNRNPA1 seq: 15 - 31 no mod	38324	37149	39656	13654	33854	4E+08	25552	34892	34104	26736	2E+08	1E+07	39754	40879	18798	3E+07	33072	34984	19162	28832	39759	50401	21442	19024	42741	1E+07	44396	34144	19500	8E+06	1E+07	31655	19257	8E+06	17227	4E+07	27262	3E+06	27887	8E+06	6E+06	64618	38440	1E+07	2E+07	5E+07	41467	25947	3E+07	7E+06	p12986	Heterogeneous nuclear ribonucleoprotein A1
EEF2 seq: 121 - 144 no mod	38324	37149	39656	13654	33854	2E+08	7E+06	34892	34104	26736	2E+08	6E+07	39754	1E+07	18798	6E+07	33072	34984	19162	28832	39759	50401	21442	19024	42741	68554	44396	34144	19500	2E+06	45838	31655	19257	24890	17227	2E+07	27262	25062	27887	36936	1E+07	64618	38440	41974	13188	134692	4E+06	25947	30272	25732	p00740	Elongation factor 2
CAPZB seq: 238 - 244 no mod	38324	37149	39656	13654	33854	2E+07	25552	34892	8E+06	7E+06	4E+08	53206	39754	7E+06	18798	3E+07	33072	4E+06	1E+07	28832	39759	50401	21442	19024	42741	68554	44396	34144	19500	29103	45838	31655	19257	3E+06	4E+06	44771	27262	25062	27887	5E+06	100327	64618	38440	41974	13188	134692	41467	25947	30272	25732	p18700	F-actin-capping protein subunit beta
HNRNPH1 seq: 36 - 44 no mod	38324	37149	39656	13654	33854	5E+06	25552	34892	34104	26736	6E+08	53206	39754	40879	18798	40867	33072	34984	19162	28832	5E+07	50401	21442	2E+06	920418	68554	44396	34144	19500	29103	45838	31655	19257	3E+06	17227	9E+06	27262	25062	27887	36936	100327	64618	38440	3E+06	2E+06	7E+07	1E+06	25947	30272	25732	p13804	Heterogeneous nuclear ribonucleoprotein H
SPAG9 pT217	38324	37149	39656	13654	33854	2E+08	25552	34892	34104	26736	7E+08	53206	39754	40879	18798	40867	8E+06	34984	19162	28832	39759	50401	21442	19024	42741	68554	44396	34144	19500	29103	45838	31655	19257	24890	17227	9E+06	27262	25062	27887	36936	100327	64618	38440	2E+07	13188	134692	41467	25947	30272	25732	p17221	C-Jun-amino-terminal kinase-interacting protein 4
SRRM2 pS510	38324	37149	39656	13654	33854	6E+07	25552	34892	34104	2E+07	2E+07	6E+06	39754	40879	6E+07	40867	33072	34984	19162	28832	2E+07	50401	21442	3E+06	42741	1E+07	44396	34144	19500	29103	45838	31655	19257	4E+07	17227	1E+08	27262	2E+07	27887	5E+07	2E+08	64618	38440	3E+07	3E+07	134692	41467	25947	30272	25732	p04447	Serine/arginine repetitive matrix protein 2
FAM171A2 pS810	38324	37149	39656	13654	33854	6E+07	25552	34892	34104	2E+07	2E+07	6E+06	39754	40879	6E+07	40867	33072	34984	19162	28832	2E+07	50401	21442	3E+06	42741	1E+07	44396	34144	19500	29103	45838	31655	19257	4E+07	17227	1E+08	27262	2E+07	27887	5E+07	2E+08	64618	38440	3E+07	3E+07	134692	41467	25947	30272	25732	p04294	Protein FAM171A2
PLA2G4B pM1	38324	37149	39656	13654	33854	2E+07	25552	34892	34104	26736	4E+07	53206	39754	40879	18798	3E+07	33072	34984	19162	28832	39759	50401	21442	19024	42741	68554	44396	34144	19500	29103	2E+07	31655	3E+06	24890	17227	3E+07	3E+06	25062	27887	36936	5E+06	64618	38440	41974	13188	134692	41467	25947	30272	25732	p10231	Cytosolic phospholipase A2 beta
DPYSL3 seq: 362 - 368 no mod	38324	37149	39656	13654	33854	2E+07	25552	34892	34104	26736	4E+07	53206	39754	40879	18798	3E+07	33072	34984	19162	28832	39759	50401	21442	19024	42741	68554	44396	34144	19500	29103	2E+07	31655	3E+06	24890	17227	3E+07	3E+06	25062	27887	36936	5E+06	64618	38440	41974	13188	134692	41467	25947	30272	25732	p09870	Dihydropyrimidinase-related protein 3
NDUFS4 seq: 85 - 95 no mod	38324	37149	39656	13654	33854	45779	25552	3E+06	34104	26736	56234	53206	39754	40879	6E+07	8E+06	33072	34984	19162	28832	39759	50401	21442	19024	42741	5E+06	44396	34144	19500	29103	9E+06	31655	394762	8E+06	1E+07	1E+08	27262	25062	27887	6E+07	2E+08	9E+06	38440	1E+07	4E+07	5E+07	41467	25947	8E+06	2E+06	p09330	NADH dehydrogenase [ubiquinone] iron-sulfur protein 4, mitochondrial
PLS3 seq: 600 - 613 no mod	38324	37149	39656	13654	33854	2E+07	25552	5E+06	34104	26736	9E+07	53206	2E+07	40879	18798	40867	33072	34984	6E+06	28832	39759	50401	21442	5E+06	42741	68554	44396	1E+07	6E+06	29103	6E+07	3E+06	1E+07	6E+06	5E+06	9E+07	2E+07	1E+07	8E+06	8E+06	6E+06	64618	3E+07	6E+06	1E+07	1E+08	6E+06	2E+07	1E+07	1E+07	p00548	Plastin-3
GOT1 seq: 115 - 122 no mod	38324	37149	39656	13654	33854	3E+07	25552	34892	34104	26736	2E+08	53206	39754	40879	18798	3E+06	1E+06	34984	19162	28832	39759	50401	21442	19024	42741	68554	44396	34144	19500	29103	2E+07	31655	19257	24890	17227	7E+07	27262	25062	27887	36936	100327	64618	38440	41974	13188	134692	41467	25947	30272	25732	p14284	Aspartate aminotransferase, cytoplasmic
HBP1 seq: 134 - 144 + Phospho (ST)	38324	4E+07	39656	13654	33854	45779	3E+08	3E+08	34104	26736	2E+08	53206	1E+08	2E+08	18798	2E+08	33072	3E+08	19162	2E+08	3E+08	2E+08	21442	3E+08	42741	68554	5E+07	34144	19500	29103	3E+08	2E+08	2E+08	4E+08	3E+08	3E+08	3E+08	2E+08	2E+08	36936	5E+08	4E+08	3E+08	41974	2E+08	4E+08	3E+08	3E+08	30272	25732	p03765	HMG box-containing protein 1
SRSF9 seq: 145 - 154 no mod	38324	37149	39656	13654	33854	2E+07	25552	8E+06	5E+06	26736	2E+08	53206	2E+07	40879	18798	40867	33072	34984	9E+06	28832	39759	50401	21442	19024	42741	68554	44396	34144	4E+06	2E+06	5E+07	31655	2E+07	24890	3E+06	9E+07	1E+07	3E+06	27887	4E+06	100327	64618	2E+07	5E+06	6E+06	1E+08	41467	7E+06	30272	25732	p18791	Serine/arginine-rich splicing factor 9
PPIA seq: 132 - 144 no mod	38324	37149	39656	13654	33854	45779	25552	34892	34104	26736	4E+08	53206	39754	40879	18798	40867	33072	34984	19162	28832	1E+07	50401	7E+07	19024	42741	68554	44396	34144	19500	29103	45838	31655	19257	24890	17227	4E+07	27262	25062	27887	6E+06	100327	64618	38440	41974	13188	9E+07	41467	25947	5E+06	1E+07	p01325	Peptidyl-prolyl cis-trans isomerase A
HSPA9 seq: 579 - 595 no mod	38324	37149	39656	13654	33854	2E+09	25552	34892	34104	26736	1E+09	2E+07	9E+07	40879	18798	40867	33072	34984	19162	28832	39759	50401	21442	19024	42741	68554	44396	34144	19500	29103	45838	31655	19257	24890	17227	44771	27262	25062	27887	36936	100327	64618	38440	41974	13188	134692	5E+08	25947	30272	25732	p01642	Stress-70 protein, mitochondrial
ROCK2 seq: 977 - 995 no mod	38324	37149	39656	13654	33854	7E+08	25552	34892	3E+07	26736	1E+09	53206	39754	40879	18798	40867	33072	34984	19162	28832	39759	50401	21442	19024	42741	68554	44396	34144	19500	29103	45838	31655	2E+07	24890	17227	44771	27262	25062	27887	36936	100327	64618	38440	41974	13188	134692	41467	25947	30272	9E+06	p02515	Rho-associated protein kinase 2
AIM1 seq: 19 - 35 + Phospho (ST)	38324	37149	39656	13654	33854	45779	25552	4E+07	34104	26736	2E+08	4E+07	39754	40879	18798	40867	33072	34984	19162	28832	39759	9E+06	4E+07	19024	42741	3E+07	44396	34144	19500	29103	8E+07	31655	1E+07	24890	2E+07	7E+07	27262	2E+07	27887	2E+07	9E+07	2E+07	1E+07	41974	7E+06	6E+07	8E+07	3E+07	30272	5E+06	p03552	Absent in melanoma 1 protein
RPN1 seq: 593 - 599 no mod	38324	37149	39656	13654	33854	5E+07	25552	34892	34104	26736	2E+08	53206	39754	40879	18798	40867	33072	34984	3E+06	28832	3E+07	50401	21442	19024	3E+07	68554	44396	34144	19500	2E+06	9E+07	31655	19257	24890	17227	1E+08	27262	25062	27887	36936	100327	64618	38440	41974	13188	8E+07	41467	25947	30272	25732	p06818	Dolichyl-diphosphooligosaccharide--protein glycosyltransferase subunit 1
ALDOC seq: 174 - 200 no mod	38324	37149	39656	13654	33854	45779	25552	34892	34104	26736	7E+08	53206	39754	40879	18798	40867	33072	4E+07	19162	28832	39759	50401	21442	19024	42741	68554	44396	34144	19500	29103	45838	31655	19257	24890	17227	1E+08	27262	25062	27887	36936	100327	64618	38440	41974	13188	1E+08	41467	9E+07	30272	25732	p00443	Fructose-bisphosphate aldolase C
MTHFD1 seq: 355 - 362 no mod	38324	37149	39656	13654	33854	7E+07	25552	7E+06	34104	26736	7E+08	53206	39754	40879	18798	40867	33072	34984	19162	28832	39759	50401	21442	19024	42741	68554	44396	34144	19500	29103	2E+07	31655	19257	24890	17227	6E+07	27262	25062	27887	36936	100327	64618	38440	41974	13188	8E+07	41467	25947	30272	25732	p01208	C-1-tetrahydrofolate synthase, cytoplasmic
MATR3 seq: 133 - 146 no mod	38324	37149	39656	13654	33854	3E+08	25552	34892	34104	26736	2E+08	1E+07	39754	40879	18798	5E+07	33072	34984	19162	28832	39759	50401	21442	19024	42741	68554	44396	34144	19500	29103	45838	31655	19257	24890	17227	5E+07	27262	25062	27887	36936	100327	64618	38440	41974	13188	3E+07	41467	25947	30272	25732	p13591	Matrin-3
AKT1 pS473	38324	37149	39656	13654	33854	45779	25552	34892	34104	26736	1E+09	4E+08	39754	40879	9E+07	8E+08	33072	34984	19162	3E+08	39759	50401	21442	19024	42741	68554	44396	34144	19500	29103	45838	31655	19257	24890	17227	44771	27262	25062	27887	5E+07	100327	64618	38440	41974	4E+07	134692	41467	25947	30272	25732	p06930	RAC-alpha serine/threonine-protein kinase
TUBB seq: 363 - 379 no mod	38324	37149	39656	13654	33854	1E+08	25552	34892	34104	26736	4E+08	1E+08	39754	40879	18798	8E+07	33072	34984	19162	28832	39759	50401	21442	19024	42741	68554	44396	34144	19500	29103	45838	31655	19257	24890	17227	4E+07	27262	25062	27887	36936	100327	64618	38440	41974	13188	3E+07	41467	25947	30272	25732	p10230	Tubulin beta chain
MYH9 seq: 1052 - 1075 no mod	38324	37149	39656	13654	33854	3E+08	7E+06	34892	34104	26736	7E+08	53206	39754	40879	18798	3E+07	33072	4E+06	19162	28832	39759	50401	21442	19024	42741	68554	44396	34144	19500	29103	45838	31655	19257	24890	17227	2E+07	27262	25062	27887	3E+07	8E+06	64618	38440	4E+06	13188	134692	41467	25947	30272	3E+06	p13004	Myosin-9
TMPO seq: 379 - 410 no mod	38324	37149	39656	13654	33854	2E+08	25552	34892	34104	26736	1E+08	53206	2E+07	40879	18798	3E+07	33072	34984	19162	28832	39759	50401	21442	19024	42741	68554	44396	34144	19500	29103	2E+07	31655	19257	24890	17227	44771	27262	25062	27887	36936	100327	64618	38440	5E+07	13188	6E+07	41467	25947	30272	25732	p18028	Lamina-associated polypeptide 2, isoform alpha

	MDA-MK2206_vs_MDA-DMS	MDA-GSK690693_vs_MDA-D	MDA-GDC0068_vs_MDA-DM	MDA-ARQ751_vs_MDA-DMS	EBC-MK2206_vs_EBC-DMSO.	EBC-GSK690693_vs_EBC-DM	EBC-GDC0068_vs_EBC-DMSC	EBC-ARQ751_vs_EBC-DMSO.	q	Sum	include	Cluster
PDK1	***	****	****	****	****	***		****	7	0.1286		4
ATM	****	****	****	****	****	*	**	****	4	-1.32		4
TBK1	****	****	****	****	****	*	**	****	3	-1.487		4
PIKFYVE	****	****	****	****	****	*	**	****	3	-1.487		4
LRRK2	****	****	****	****	****	*	**	****	3	-1.487		4
DNAPK	****	****	****	****	****	*	**	****	4	-1.635		4
ILK	****	****	****	****	****		**	****	4	-2.27		4
MAPKAPK2	****	****	****	****	****		***	****	8	-2.333		4
PKCB	****	****	****	****	****	****	***	****	13	-2.573		4
PKCA	****	***	****	****	****	****	***	****	11	-2.888		4
PKD1	****	****	****	****	****	****	****	****	12	-3.561		4

	MDA-MK2206_vs_MDA-DMS	MDA-GSK690693_vs_MDA-D	MDA-GDC0068_vs_MDA-DM	MDA-ARQ751_vs_MDA-DMS	EBC-MK2206_vs_EBC-DMSO.	EBC-GSK690693_vs_EBC-DM	EBC-GDC0068_vs_EBC-DMSC	EBC-ARQ751_vs_EBC-DMSO.
PDK1	0.00149	2.9E-09	3.3E-16	1.4E-05	3E-06	0.00598	0.3013	1.1E-05
ATM	7.2E-14	0	0	1.3E-14	2.2E-16	0.06193	0.01936	4.2E-15
TBK1	7.2E-14	1.1E-12	0	1.3E-14	2.2E-16	0.06193	0.01936	4.2E-15
PIKFYVE	7.2E-14	1.1E-12	0	1.3E-14	2.2E-16	0.06193	0.01936	4.2E-15
LRRK2	7.2E-14	1.1E-12	0	1.3E-14	2.2E-16	0.06193	0.01936	4.2E-15
DNAPK	7.2E-14	1.3E-07	4.2E-14	1.1E-09	2.2E-16	0.06193	0.01936	4.2E-15
ILK	7.2E-14	0	1.1E-05	0	2.2E-16	0.44087	0.01936	4.2E-15
MAPKAPK2	0.00017	3.6E-09	1.4E-12	3.4E-08	0	0.11254	0.00338	0
PKCB	1.6E-10	6E-05	4.4E-14	2E-08	7.2E-14	0.00012	0.00471	5.3E-11
PKCA	1.6E-10	0.00265	9.1E-14	2E-08	5.2E-13	1.8E-06	0.00125	1.1E-11
PKD1	3.9E-07	2.1E-06	4.7E-05	3.1E-05	0	2.5E-07	1.6E-05	0

db ID		MDA-MK2206_vs_MDA-DMSO.fold	MDA-GSK690693_vs_MDA-DMSO.fold	MDA-GDC0068_vs_MDA-DMSO.fold	MDA-ARQ751_vs_MDA-DMSO.fold		EBC-MK2206_vs_EBC-DMSO.fold	EBC-GSK690693_vs_EBC-DMSO.fold	EBC-GDC0068_vs_EBC-DMSO.fold	EBC-ARQ751_vs_EBC-DMSO.fold	db id	Protein name
AKT1 pS473		***	***	***	***		***	***	***	***	p06929	RAC-alpha serine/threonine-protein kinase
AKT1 pS473			**	*							p06930	RAC-alpha serine/threonine-protein kinase
AKT1 seq: 122 - 142 + 2 Phospho (ST)		*		*	**						p05664	RAC-alpha serine/threonine-protein kinase
AKT1 seq: 466 - 480 + 2 Phospho (ST)		***	***	***	***		***		**	***	p06931	RAC-alpha serine/threonine-protein kinase
AKT1S1 pS183		**	*	**	**		**	*	**	*	p05092	Proline-rich AKT1 substrate 1
AKT1S1 pT246		***	***	***	***		***	***	***	***	p11152	Proline-rich AKT1 substrate 1
AKT1S1 seq: 202 - 223 + Phospho (ST)								*	**	*	p03942	Proline-rich AKT1 substrate 1
AKT2 pS474			***	***			*			*	p02754	RAC-beta serine/threonine-protein kinase
BAD pM117 pS118			*	*							p07053	Bcl2-associated agonist of cell death
BAD pS118							*	***		*	p07051	Bcl2-associated agonist of cell death
BAD pS99		**	***	**	**		***	***	***	***	p06291	Bcl2-associated agonist of cell death
BAD pS99		***		**	**		**	**	**	**	p06290	Bcl2-associated agonist of cell death
BAD seq: 73 - 94 + Phospho (ST)				**	**						p14599	Bcl2-associated agonist of cell death
BAD seq: 97 - 109 + Phospho (ST)		*	**							**	p04074	Bcl2-associated agonist of cell death
BAD seq: 97 - 109 + Phospho (ST)		**	**	**	**		***	***	***	***	p04073	Bcl2-associated agonist of cell death

db ID	MDA-MK22	MDA-GSK6	MDA-GDC0	MDA-ARQ7	EBC-MK220	EBC-GSK69	EBC-GDC00	EBC-ARQ751_vs_EBC-DMSO.fold			Cluster number		pSite	MCTA	NCI	hSignalling
ZNF593 sec	9.534864	7.041679	0	0	-0.00576	-0.38643	-0.11301	-0.07057	p07193	Zinc finger p	8	2.000096				
ZNF185 sec	2.161645	3.823305	3.222207	5.016948	-1.79519	-0.12969	-1.4568	-0.61337	p06568	Zinc finger p	8	1.278632				
ZFP36 seq:	0.729979	6.51653	0.818416	2.51661	0.477057	0.628067	0.404769	0.371133	p11722	Tristetrapro	8	1.55782			ErbB1 downstream signaling;	
YWHAB seq	7.333868	9.835568	7.68765	0	-6.44661	1.467007	-5.9002	3.413799	p03095	14-3-3 prot	8	2.173885			p38 signalir	GO:0035329; P:hippo signaling; GO:0000186; P:activation of MAPKK activity; GO:0000165; P:MAPK cascade; GO:0007265; P:Ras protein signal transduction; GO:0008286; P:insuli [illegible]
YAP1 seq: 2	0	11.03629	0	8.545753	0.984106	1.095918	0.868486	0.317159	p20081	Transcriptic	8	2.855964			ErbB4 signa	GO:0014065; P:phosphatidylinositol 3-kinase signaling; GO:0035329; P:hippo signaling; GO:0090263; P:positive regulation of canonical Wnt signaling pathway;
YAP1 pS131	8.343082	6.820774	0	0	0.248028	-1.06737	0.040949	0.358752	p20508	Transcriptic	8	1.843027		PKCinh1 (G	ErbB4 signa	GO:0014065; P:phosphatidylinositol 3-kinase signaling; GO:0035329; P:hippo signaling; GO:0090263; P:positive regulation of canonical Wnt signaling pathway;
XRCC5 seq:	3.205795	6.262651	1.46684	0	0	0	0	0	p16554	X-ray repair	8	1.366911			DNA-PK pathway in nonhomologous end joining; BARD1 signaling events; Coregulation of Androgen receptor activity; Regulation of Telomerase;	
WNK1 pS19	3.491028	3.595614	2.202237	2.521796	0.154864	0.38984	0.839093	1.739285	p13292	Serine/threo	8	1.86672				GO:0035556; P:intracellular signal transduction;
WDR70 pS6	7.78654	8.71294	0	8.126799	-0.31476	-0.80958	-0.10667	-0.1526	p02504	WD repeat-	8	2.905333				
WASH6P pS	0	6.305932	6.854099	6.691325	0.524128	0.66106	0.538744	-0.11873	p08778	WAS protei	8	2.68207				
VRK2 seq: 1	3.960849	5.562832	1.683158	3.283569	-1.31229	2.907681	-5.26701	3.225928	p11761	Serine/threo	8	1.755589				GO:0043408; P:regulation of MAPK cascade;
VASP seq: 3	7.166155	7.856218	6.253785	0	0	0	0	0	p01330	Vasodilator	8	2.65952			E-cadherin :	GO:0050852; P:T cell receptor signaling pathway;
USP43 pS10	3.539757	3.953659	3.878471	3.817873	0.671003	-0.15418	0.506267	0.417097	p18077	Ubiquitin ca	8	2.078743				
USP10 seq:	9.369278	6.913613	0	5.951238	0.377906	0.313695	0.133576	0.184335	p02987	Ubiquitin ca	8	2.905455				GO:0030330; P:DNA damage response, signal transduction by p53 class mediator;
UFL1 seq: 5	0	8.094342	0	0	0	0	0	0	p02706	E3 UFM1-p	8	1.011793				
UBA1 seq: 6	8.512734	9.99787	0	0	0	0	0	0	p09637	Ubiquitin-li	8	2.313825				
UBA1 seq: 4	0	8.670423	0	0	0	0	0	0	p09660	Ubiquitin-li	8	1.083803				
UBA1 seq: 4	8.670704	10.85106	7.618811	0	0.677628	3.226536	2.744386	-5.23617	p09659	Ubiquitin-li	8	3.569119				
TXN seq: 9	6.868188	9.811877	0	0	-5.26285	-5.20851	-4.71644	-4.67559	p03090	Thioredoxin	8	-0.39792			p38 MAPK	GO:0035872; P:nucleotide-binding domain, leucine rich repeat containing receptor signaling pathway; GO:0007267; P:cell-cell signaling; GO:0007165; P:signal transduction;
TUBB4B sec	8.106632	10.73784	0	0	0	0	0	0	p05712	Tubulin bet	8	2.355559				
TUBB seq: 3	8.459688	10.77052	8.654239	0	0	6.959285	0	6.657543	p10230	Tubulin bet	8	5.187659				
TPM4 seq:	7.6762	11.37104	0	6.732098	-0.14627	0.983946	0.061364	3.159242	p10185	Tropomyos	8	3.729702				
TPM2 seq:	7.6762	11.37104	0	6.732098	-0.14627	0.983946	0.061364	3.159242	p10182	Tropomyos	8	3.729702				
TOMM34 s	8.502128	8.273201	0	0	-0.02235	0.013479	0.209614	0.032125	p01945	Mitochond	8	2.126025				
TOM1 seq:	0	11.18859	0	8.059997	0	0	0	0	p11104	Target of M	8	2.406074			C-MYB transcription factor network;	
TOM1 seq:	0	11.2563	6.059663	0	0	0	0	0	p21184	Target of M	8	2.164495			C-MYB transcription factor network;	
TNKS1BP1	0	9.853114	0	0	0.227313	0.430169	-0.15529	0.338899	p05908	182 kDa tar	8	1.336776				
TMPO seq:	8.787673	8.840037	7.341874	0	6.477414	0	8.107424	7.756973	p18028	Lamina-asso	8	5.913924				
TJP2 pS913	0	6.28162	0	5.371422	0.956768	0.851199	1.067981	1.173874	p09865	Tight juncti	8	1.962858			Validated ta	GO:0035329; P:hippo signaling;
TJP1 seq: 1	8.548748	6.341225	4.903284	0	0.220007	0.276602	0.384713	0.261058	p20439	Tight juncti	8	2.616955			Nephrin/Ne	GO:0035329; P:hippo signaling;
TJP1 seq: 1	0	7.243454	0	0	-0.03544	0.002405	0.08433	-0.07763	p00950	Tight juncti	8	0.90214			Nephrin/Ne	GO:0035329; P:hippo signaling;
TJP1 pS617	3.746463	3.461393	3.516593	3.557345	-0.03385	-0.14081	-0.07011	-0.31441	p12550	Tight juncti	8	1.715326			Nephrin/Ne	GO:0035329; P:hippo signaling;
TJP1 pS175	5.892796	5.046817	0	0	0.428463	-0.07936	0.375652	0.645289	p03391	Tight juncti	8	1.538707			Nephrin/Ne	GO:0035329; P:hippo signaling;
THRAP3 sec	7.704741	6.714358	0	7.235496	-1.20365	0.208861	-0.60773	-0.54694	p17922	Thyroid hor	8	2.438141				GO:0030518; P:intracellular steroid hormone receptor signaling pathway; GO:0030521; P:androgen receptor signaling pathway;
TGFB1I1 pS	0	6.761276	7.01133	0	0.078133	0.338175	0.483769	1.2256	p04572	Transformir	8	1.987285			Coregulatio	GO:0030511; P:positive regulation of transforming growth factor beta receptor signaling pathway; GO:0030521; P:androgen receptor signaling pathway; GO:0030512; P:negative regu [illegible]
TDRD3 seq:	3.743075	2.982711	4.205216	4.493171	6.2654	0	0	0	p17332	Tudor doma	8	2.711197				
TCOF1 pS5	3.856467	3.694572	4.206437	3.580754	-0.09523	0.089186	0.4815	0.263356	p04449	Treacle prot	8	2.00963				
TCOF1 pS4	7.303199	5.832724	0	0	3.463899	4.262762	5.069487	4.101182	p21055	Treacle prot	8	3.754157				
TCF3 pS35	0	9.778561	0	6.358619	0	0	0	0	p20315	Transcriptic	8	2.017147	MLK2; ERK2;		p38 signaling mediated by MAPKAP kinases; Notch-mediated HES/HEY network; Regulation of nuclear SMAD2/3 signaling;	
TACO1 seq:	0	9.443961	5.682697	5.882334	0	0	0	0	p16698	Translation	8	2.626124				
SZRD1 pS1	0	9.651748	7.093267	0	0.530015	0.929997	-0.59501	0.708964	p14040	SUZ domain	8	2.289873				
STMN1 pS1	4.507099	5.187865	4.166781	3.501624	0.306802	0.906365	0.624834	0.780975	p07582	Stathmin	8	2.497793	PKACA; PAK	Up with IGF	Signaling me	GO:0035556; P:intracellular signal transduction; GO:0007165; P:signal transduction;
STK10 seq:	0	6.193827	5.700155	0	0.342047	0.313393	0.50218	0.195868	p10821	Serine/threo	8	1.655934				GO:0023014; P:signal transduction by phosphorylation;
STK10 pS41	5.071649	3.867459	5.450046	5.310187	-0.053	-0.31139	-0.05348	0.422682	p04080	Serine/threo	8	2.46302				GO:0023014; P:signal transduction by phosphorylation;
STIP1 seq: 1	2.939491	3.691949	2.341283	1.800269	1.256865	0.874914	2.676573	3.328639	p20142	Stress-indu	8	2.363748				
SSB seq: 61	4.396441	4.822624	3.554014	-4.39758	0	6.203068	0	5.915278	p10483	Lupus La pr	8	2.56173			Regulation	GO:0007265; P:Ras protein signal transduction; GO:0007218; P:neuropeptide signaling pathway; GO:0048011; P:neurotrophin TRK receptor signaling pathway; GO:0007165; P:signa [illegible]
SRSF9 seq:	7.404857	9.056274	5.777233	0	3.478725	3.325245	2.399523	3.484258	p18791	Serine/argin	8	4.365764				
SRRM2 pS5	8.56724	8.993953	0	6.766697	2.776777	4.883629	5.011278	-4.77806	p04447	Serine/argin	8	4.027689				
SRRM1 pS6	5.449338	4.42902	4.784362	4.941076	0.491866	0.263733	1.544456	-0.48804	p00849	Serine/argin	8	2.676977				
SRP68 seq:	6.270124	6.673386	0	0	0	0	0	0	p14300	Signal recog	8	1.617939				
SRI seq: 10	0	7.871499	0	0	0.772533	0.790867	0.647793	0.603249	p08570	Sorcin	8	1.335743				GO:0007165; P:signal transduction;
SPAG9 seq:	8.019277	8.778059	0	0	-6.39161	1.096895	1.166927	3.776038	p08938	C-Jun-amin	8	2.055698			Arf6 trafficking events;	
SPAG9 pT2	8.855668	10.68012	5.825319	0	0	0	6.691714	0	p17221	C-Jun-amin	8	4.006603		Up with EGI	Arf6 trafficking events;	
SNX29 seq:	8.510583	7.584093	0	7.144711	0	0	0	0	p00060	Sorting nexi	8	2.904923				
SNX1 seq: 1	10.85104	10.83035	0	0	-8.81525	-8.7609	-8.26884	3.217083	p11096	Sorting nexi	8	-0.11832			PAR1-mediated thrombin signaling events; Posttranslational regulation of adherens junction stability and dissassembly;	
SNRPG seq	5.244589	8.772119	0	0	0	0	0	0	p15569	Small nucle	8	1.752088				
SMS seq: 2	5.913148	8.37952	0	0	0	0	0	0	p11584	Spermine sy	8	1.786584				
SLK pS779	5.850532	9.229581	0	6.90663	0.084343	-0.22002	0.127598	-0.14538	p02672	STE20-like s	8	2.72916			Caspase cas	GO:0023014; P:signal transduction by phosphorylation;
SLC4A1AP	6.505055	5.911729	0	0	0.511634	0.609348	1.132979	0.76361	p12868	Kanadaptin	8	1.929294				
SLAIN2 seq	7.924788	9.317705	0	0	0	0	0	0	p06485	SLAIN motif	8	2.155312				
SIPA1L3 sec	0	10.80003	0	8.47928	0	0	0	0	p01508	Signal-indu	8	2.409914				GO:0051056; P:regulation of small GTPase mediated signal transduction;
SF3B2 seq:	9.158602	8.846867	0	0	0.860811	0.655014	0.074506	0.939178	p12197	Splicing fact	8	2.566872				
SF3A1 seq:	6.552687	8.682502	0	0	-0.47333	-0.31001	-0.12011	-0.0768	p17549	Splicing fact	8	1.781867		CAMKinh2 (KN-62); AKTinh2 (MK-2206);		
SF3A1 seq:	0	9.233983	4.986709	0	0	7.731554	0	8.329126	p14255	Splicing fact	8	3.785172				
SERBP1 seq	0	8.522751	0	0	-1.73687	-0.30459	-0.73329	-1.90737	p15971	Plasminoge	8	0.480079		Up with IGF - down with PKCinh2 (Bisindolylmaleimide-1); Up with EGF - down with PKCinh2 (Bisindolylmaleimide-1); PKCinh2 (Bisindolylmaleimide-1);		
SCRIB pS93	8.945271	9.625218	7.812147	0	0.344397	0.23582	0.827925	0.580173	p14866	Protein scri	8	3.546369				
SCAF1 pS4	12.94869	0	0	0	0.534026	-0.14046	1.945852	0.614499	p04404	Splicing fact	8	1.987826				
SAFB seq: 2	3.954209	3.593542	3.893476	3.565479	-0.6545	-0.08072	0.239116	-0.02259	p14083	Scaffold att	8	1.811002			Validated n	GO:0030520; P:intracellular estrogen receptor signaling pathway;
RUSC2 pS5	5.966742	9.428713	0	5.66881	-0.5394	0.055502	-0.28361	-0.41977	p10818	Iporin	8	2.484623				
RPS5 seq: 1	8.867987	7.368644	6.827639	0	0.555191	1.652972	0.129714	0.7835	p02735	40S ribosor	8	3.273206				
RPS26P11	3.444012	4.718109	3.340002	3.907241	1.929836	0.714906	0.475652	2.455987	p15921	Putative 40	8	2.623218				
RPS19 seq:	0	9.059769	6.007944	5.784904	1.27241	0.888703	1.399292	2.481396	p09587	40S ribosor	8	3.361802				
RPN1 seq: 5	7.03983	8.635984	0	8.540362	4.620293	4.275549	0	4.16807	p06818	Dolichyl-dip	8	4.660011				
RPL28 seq:	0	9.915662	0	0	1.741067	1.657243	-7.2714	1.791898	p09723	60S ribosor	8	0.979309				
RPL19 seq:	7.486592	8.566505	0	0	-1.03207	-0.3681	-0.5698	-8.88624	p12368	60S ribosor	8	0.649612				
ROCK2 seq	11.0524	11.36136	0	0	7.652889	0	0	5.815542	p02515	Rho-associa	8	4.485274			PAR4-medi	GO:0035556; P:intracellular signal transduction;
RIPK1 seq:	0	11.28105	0	0	0	0	0	0	p04814	Receptor-in	8	1.410131			TRAIL signal	GO:0033209; P:tumor necrosis factor-mediated signaling pathway; GO:0043124; P:negative regulation of I-kappaB kinase/NF-kappaB signaling; GO:0035666; P:TRIF-dependent toll-li [illegible]
RBM15B se	3.4882	5.913484	2.859721	4.790853	0.016101	1.114343	1.691962	1.724168	p02049	Putative RN	8	2.699854				
RBM10 pS8	6.3352	6.057335	3.131441	2.318103	-0.33525	-0.23851	-0.52541	-0.28765	p14663	RNA-bindin	8	2.056906				
RASSF7 pS2	6.445422	8.222069	0	0	-0.40636	0.053394	0.092231	0.015831	p20291	Ras associa	8	1.802823				GO:0007165; P:signal transduction;
RASAL2 seq	0	4.984198	0	9.731534	-0.18664	0.052717	0.103551	-0.05777	p16428	Ras GTPase	8	1.828449				GO:0046580; P:negative regulation of Ras protein signal transduction; GO:0007165; P:signal transduction;
RAPGEF6 se	1.589279	5.261485	0	0	0	0	0	5.530548	p14014	Rap guanine	8	1.547664				GO:0007265; P:Ras protein signal transduction; GO:0051056; P:regulation of small GTPase mediated signal transduction;
RANBP2 se	0	10.82588	0	0	0.182974	0.363075	0.455403	0.281745	p17189	E3 SUMO-p	8	1.513634			Sumoylatio	GO:0006469; P:negative regulation of protein kinase activity; GO:0019221; P:cytokine-mediated signaling pathway;
RAD23B se	2.816081	4.175887	3.797609	4.143493	0.833324	-0.2085	1.023947	1.027503	p08738	UV excision	8	2.201168				
PXN seq: 1	7.794744	8.969634	7.849097	0	-1.35369	0.902531	0.423092	0.452664	p11360	Paxillin	8	3.129759	JNK1;		a4b7 Integr	GO:0007229; P:integrin-mediated signaling pathway; GO:0000187; P:activation of MAPK activity; GO:0007173; P:epidermal growth factor receptor signaling pathway; GO:0007165; [illegible]
PURB seq:	0	7.031662	0	0	0	0	0	0	p16759	Transcriptic	8	0.878958				
PTOV1 seq:	4.473898	5.092952	2.305184	3.027984	-0.71005	0.818352	0.590113	0.489021	p01558	Prostate tu	8	2.010931				
PTMA seq:	5.576062	6.981312	0	0	0.539864	2.63855	3.415205	2.40207	p21179	Prothymosi	8	2.694133			Validated targets of C-MYC transcriptional activation;	
PTK2 pS91	2.515123	3.851331	2.892838	3.054737	0.446295	0.539516	0.551094	0.597558	p10960	Focal adhes	8	1.806061	CDK5;		LPA recepto	GO:0043552; P:positive regulation of phosphatidylinositol 3-kinase activity; GO:0048013; P:ephrin receptor signaling pathway; GO:0045860; P:positive regulation of protein kinase a [illegible]
PTBP1 seq:	8.259395	10.08172	7.599955	0	-5.10662	-0.64537	-1.27336	0.361631	p12948	Polypyrimid	8	2.409669				
PSME1 seq	0	9.200636	0	0	0	0	0	0	p11986	Proteasome	8	1.150079				
PSMC4 seq	3.067267	5.388495	2.902399	-4.43949	2.26008	2.92054	1.044466	2.479064	p00265	26S proteas	8	1.952853				
PSMA5 seq	7.209911	8.889834	5.487005	0	-0.17408	1.308949	-0.32402	1.459041	p11868	Proteasome	8	2.982079				
PSMA2 seq	0	5.662925	3.298307	2.149525	0	0	0	7.526021	p21085	Proteasome	8	2.329597				
PRRC2B sec	0	7.059362	0	0	-0.95916	-0.16138	0.15145	-0.55473	p04606	Protein PRF	8	0.691942				
PROSER2 se	10.58305	9.993248	0	0	-1.25936	0.324239	0.382889	-1.52638	p19730	Proline and	8	2.31221		PKCinh2 (Bisindolylmaleimide-1);		
PRCC pS21	3.657126	4.322066	3.901003	5.105103	0.100745	0.371265	1.242083	0.646607	p12741	Proline-rich	8	2.41825				
PPP1R15A	0	8.166138	0	0	-0.01927	0.389867	-0.24186	-0.16654	p19965	Protein pho	8	1.016042			BMP recept	GO:0030512; P:negative regulation of transforming growth factor beta receptor signaling pathway; GO:0007179; P:transforming growth factor beta receptor signaling pathway;
PPP1R13L	7.0562	9.908918	8.401011	0	-0.69932	0.583367	1.708849	1.708646	p15221	RelA-associ	8	3.583459			p53 pathway;	
PPP1R12A	0	9.99563	0	0	0	0	0	0	p18680	Protein pho	8	1.249454			Integrin-link	GO:0007165; P:signal transduction;
PPP1R10 p	9.086238	9.446729	0	5.369704	4.588515	0	0	0	p15457	Serine/threo	8	3.561398		PI3Kinh2 (PI-103); p70S6Kinh2 (DG2); p70S6Kinh1 (PF-4708671);		
PPP1CC sec	0	6.782782	5.381869	0	0	0	0	0	p14099	Serine/threo	8	1.520581			Insulin-mec	GO:0030512; P:negative regulation of transforming growth factor beta receptor signaling pathway; GO:0007179; P:transforming growth factor beta receptor signaling pathway;
PPIA seq: 1	0	9.810028	0	9.188404	0	7.500302	0	8.901932	p01325	Peptidyl-pro	8	4.425083				
PPFIBP1 se	10.39197	7.854617	0	0	-0.14727	-0.7911	-0.25083	-0.40475	p03054	Liprin-beta-	8	2.081578				
POLE3 seq:	9.574996	9.525518	6.999917	0	-1.47194	0.085436	0.678482	0.435801	p04133	DNA polym	8	3.228526				
PLS3 seq: 6	6.554027	8.564869	5.129632	5.052222	2.19159	1.466357	1.759944	2.918793	p00548	Plastin-3	8	4.204679				
PLEKHA6 se	6.591299	5.685621	6.07116	0	-0.90435	-0.73185	-0.7903	-1.37742	p13486	Pleckstrin h	8	1.81802				
PLEC seq: 3	4.474764	7.629402	4.1096	-4.7065	0.912534	1.409806	-0.2064	0.562847	p14165	Plectin	8	1.773258				
PLA2G4B p	5.280213	6.717648	7.357835	0	7.105555	6.786203	0	0	p10231	Cytosolic pl	8	4.155932				GO:0019722; P:calcium-mediated signaling; GO:0006987; P:activation of signaling protein activity involved in unfolded protein response;
PKP2 pS25	4.399627	4.026654	3.331432	0.782724	-0.73932	-1.28757	-0.88004	-0.01135	p04900	Plakophilin-	8	1.20277				
PKM seq: 4	9.836814	10.97123	0	0	0.282734	0.166564	0.083299	0.228252	p12213	Pyruvate kir	8	2.696112			HIF-1-alpha transcription factor network;	
PHLDB2 pS	6.093159	6.569002	7.548612	0	0.18528	0.366393	-0.2642	0.369272	p09629	Pleckstrin h	8	2.60844				
PHB seq: 2	0	8.138218	7.076524	0	0	0	0	0	p21171	Prohibitin	8	1.901843				GO:0060766; P:negative regulation of androgen receptor signaling pathway; GO:0007165; P:signal transduction;
PHACTR4 p	3.89797	2.976425	3.91244	3.815295	-0.64449	-2.46129	-1.8658	-0.49513	p20517	Phosphatas	8	1.141928				GO:0007266; P:Rho protein signal transduction;
PGM3 seq:	9.399219	8.437902	0	0	0	0	0	0	p10446	Phosphoac	8	2.22964				
PGM1 seq:	0	6.342849	0	0	1.786616	2.089901	-4.75918	3.239954	p01673	Phosphoglu	8	1.087517			HIF-1-alpha transcription factor network;	
PDLIM7 sec	0	9.048583	5.845516	0	0.937312	0.948519	1.12609	1.186599	p02173	PDZ and LIN	8	2.386577			Signaling events regulated by Ret tyrosine kinase;	
PDLIM1 pS	0	8.186318	0	0	0.102359	-0.31262	0.021483	-0.09079	p00550	PDZ and LIN	8	0.988344				
PDIA3 seq:	1.613941	4.731211	3.243867	0.113793	0.359237	0.453142	1.012493	1.535111	p18955	Protein disu	8	1.632849				GO:0007165; P:signal transduction;
PARP1 seq:	0	8.070305	0	0	0	0	7.526152	0	p00577	Poly [ADP-r	8	1.949557			Integrin-link	GO:0007179; P:transforming growth factor beta receptor signaling pathway;
PARD3 seq	9.414016	0	7.667988	0	2.461054	4.250711	4.215925	1.554622	p06622	Partitioning	8	3.695539	MARK2;			GO:0007205; P:protein kinase C-activating G-protein coupled receptor signaling pathway; GO:0007179; P:transforming growth factor beta receptor signaling pathway;
PALLD pS8	0	11.55413	0	0	-0.10135	-0.24771	-0.50007	0.07229	p14474	Palladin	8	1.34716				
PALLD pS8	0	10.08937	0	6.762777	0.020967	0.181096	-0.7169	0.150768	p14472	Palladin	8	2.06101				
PABPN1 se	0	8.72997	0	0	0.777948	0.558174	0.220466	1.137302	p14721	Polyadenyla	8	1.427982				
OSBP seq: 2	10.91915	12.0519	0	6.764312	-0.02313	-0.62839	-0.39129	-0.50507	p05066	Oxysterol-b	8	3.523436				
NUP93 seq	6.083224	7.665662	0	0	-0.08944	-5.40651	1.540653	0.288879	p11166	Nuclear por	8	1.260309				GO:0019221; P:cytokine-mediated signaling pathway;
NUMB seq:	0	10.42955	0	0	1.102989	1.510854	1.086059	1.988523	p13521	Protein nur	8	2.014746			Notch signa	GO:0045746; P:negative regulation of Notch signaling pathway; GO:0007219; P:Notch signaling pathway;
NUMB seq:	0	11.97264	0	0	0.494584	1.232829	1.341057	1.318411	p13442	Protein nur	8	2.04494			Notch signa	GO:0045746; P:negative regulation of Notch signaling pathway; GO:0007219; P:Notch signaling pathway;
NUMA1 sec	3.242447	2.996071	3.631131	3.462338	0.051759	1.307144	0.530907	-0.27616	p19202	Nuclear mit	8	1.868204			Caspase cascade in apoptosis;	
NPM1 seq:	9.412192	9.049478	5.980551	0	-0.4404	-0.23234	-0.17549	0.369876	p18890	Nucleopho	8	2.995484	CDK2; CDK	Up with IGF	BARD1 sign	GO:0007165; P:signal transduction;
NOSIP seq:	0	5.771182	0	6.75085	1.534977	2.633002	-0.72369	1.744865	p18193	Nitric oxide	8	2.213899				
NONO seq:	7.094658	10.63951	0	0	2.867563	3.678431	2.286618	3.807275	p16855	Non-POU d	8	3.796757			Circadian rhythm pathway;	
NONO pM3	10.0723	10.2773	5.825016	0	-1.16888	0.904935	1.645586	-0.29729	p10086	Non-POU d	8	3.407372			Circadian rhythm pathway;	
NOC2L pS6	0	10.29998	0	8.407601	0.302125	0.35117	1.297746	0.688782	p18620	Nucleolar co	8	2.668426		AKTinh1 (Al	Validated transcriptional targets of TAp63 isoforms;	
NHSL1 seq:	3.100269	3.078258	3.576618	2.83535	0.181489	0.372165	1.088304	1.37764	p15399	NHS-like pro	8	1.951262				
NFRKB pS2	8.92307	6.177025	4.996628	0	0	0	0	0	p13158	Nuclear fact	8	2.512091				
NFKB1 seq:	4.454969	5.896534	2.941232	1.622994	-0.77072	0.349458	0.464425	-0.13081	p02006	Nuclear fact	8	1.853511			Alternative	GO:0090263; P:positive regulation of canonical Wnt signaling pathway; GO:0034166; P:toll-like receptor 10 signaling pathway; GO:0034146; P:toll-like receptor 5 signaling pathway; [illegible]
NDUFS4 se	0	8.618175	5.321157	0	3.618204	5.825061	6.165312	4.078357	p09330	NADH dehy	8	4.203283				GO:0019933; P:cAMP-mediated signaling;
NCOA5 seq	8.127445	9.798619	7.367057	0	-2.54599	-3.32448	-4.48102	-5.63886	p16042	Nuclear rec	8	1.162846				
NCL seq: 14	0	9.200799	10.23421	0	0.7486	0.77773	1.009877	1.21551	p13311	Nucleolin	8	2.89834			Aurora B signaling; Urokinase-type plasminogen activator (uPA) and uPAR-mediated signaling; Regulation of Telomerase; Validated targets of C-MYC transcriptional activation;	
NAV2 seq:	0	7.765338	0	0	0.572173	0.354462	0.428754	0.148866	p15840	Neuron nav	8	1.158699				
NAV2 pS12	0	7.360546	5.897392	5.125452	0.351358	0.904434	-0.28471	0.340475	p06331	Neuron nav	8	2.461869				
NAV2 pS12	2.615476	4.729672	1.714218	1.59621	0.005319	-0.01065	-0.22193	0.047103	p05107	Neuron nav	8	1.309428				
NACC1 seq	4.778818	4.339039	3.695849	-0.68485	-6.13859	-1.60955	-5.59218	2.00965	p00895	Nucleus acc	8	0.099774				
MYL9 seq:	8.006719	8.847917	6.963912	0	-2.93705	-2.36119	-1.95189	-2.496	p06872	Myosin regu	8	1.759052	smMLCK; R	Up with IGF	Integrin-linked kinase signaling;	
MYH9 seq:	0	8.079333	7.77672	0	0	0	0	0	p10545	Myosin-9	8	1.982007				GO:0038032; P:termination of G-protein coupled receptor signaling pathway; GO:0007229; P:integrin-mediated signaling pathway;
MYH9 seq:	9.437524	10.63537	7.567179	0	0	8.142232	5.980357	0	p13004	Myosin-9	8	5.220333				GO:0038032; P:termination of G-protein coupled receptor signaling pathway; GO:0007229; P:integrin-mediated signaling pathway;
MYH14 seq	8.587929	9.674337	0	0	0	0	0	7.702662	p08352	Myosin-14	8	3.245616				
MVP seq: 8	9.814663	11.09065	0	0	0.243496	0.333354	0.244489	0.356528	p01763	Major vault	8	2.760397				
MTHFD1 se	7.909394	10.58858	0	0	6.111869	7.579926	0	8.163657	p01208	C-1-tetrahy	8	5.044178				
MLLT4 pS1	4.83525	5.049129	4.851777	5.603974	-0.46504	-0.49345	-1.18051	-1.56565	p02080	Afadin	8	2.079436			SHP2 signal	GO:0007267; P:cell-cell signaling; GO:0007165; P:signal transduction;
MKL2 seq:	0	10.64302	0	0	0	0	0	0	p09756	MKL/myoca	8	1.330377				
MISP seq: 5	6.975201	5.286858	0	5.728854	-0.46616	-0.70166	-0.38027	-0.34781	p09771	Mitotic inte	8	2.011877				
METTL16 p	5.986908	6.163308	0	7.152035	-2.33294	0.131392	-0.28036	-2.34506	p17401	Methyltrans	8	1.80941				
MDH2 seq:	9.808425	8.139881	0	5.98391	5.655212	0	0	0	p01734	Malate dehy	8	3.698429				
MAVS seq:	0	11.50916	0	0	0	0	0	6.573529	p17574	Mitochond	8	2.260336				GO:0043123; P:positive regulation of I-kappaB kinase/NF-kappaB signaling; GO:0007165; P:signal transduction;
MATR3 seq	9.311458	9.166797	7.931448	0	0	7.187798	0	6.96631	p13591	Matrin-3	8	5.070476				
MATR3 seq	9.570207	8.010635	6.953735	0	0	0	0	0	p18640	Matrin-3	8	3.066822				
MARCKSL1	0	7.767605	0	0	0.230318	1.162104	3.179396	1.380349	p16169	MARCKS-re	8	1.714972			Signaling events mediated by the Hedgehog family; Reelin signaling pathway; Lissencephaly gene (LIS1) in neuronal migration and development;	
MARCKSL1	0	9.465285	7.003553	0	0	0	0	0	p17751	MARCKS-re	8	2.058605			Signaling events mediated by the Hedgehog family; Reelin signaling pathway; Lissencephaly gene (LIS1) in neuronal migration and development;	
MAP4 pS62	0	7.810468	0	0	-1.11454	-0.74639	-0.6121	-0.57087	p15854	Microtubul	8	0.595821		ERKinh1 (ERK inhbitor Calbiochem); AKTinh1 (Akt Inhibitor VIII);		
MAP3K6 se	3.94345	4.08165	1.669292	0	0	2.029555	0.971391	0	p04017	Mitogen-ac	8	1.586917			p38 MAPK	GO:0000186; P:activation of MAPKK activity; GO:0007165; P:signal transduction;
MAP1B seq	5.358686	4.776577	4.726871	5.822038	-0.0895	-0.37385	-0.39362	-0.61937	p02009	Microtubul	8	2.400979			Reelin signaling pathway; Lissencephaly gene (LIS1) in neuronal migration and development; Netrin-mediated signaling events;	
MAP1B seq	0	6.550002	5.351895	0	-0.52781	0.430132	0.378521	0.451385	p01165	Microtubul	8	1.579266			Reelin signaling pathway; Lissencephaly gene (LIS1) in neuronal migration and development; Netrin-mediated signaling events;	
MAP1B pS1	0	5.267745	6.334442	0	0.138082	0.220543	0.212792	-0.0286	p16328	Microtubul	8	1.518125			Reelin signaling pathway; Lissencephaly gene (LIS1) in neuronal migration and development; Netrin-mediated signaling events;	
MAP1B pS1	8.006811	8.372048	0	7.686509	-0.31678	-0.19834	-0.17212	-0.37974	p04411	Microtubul	8	2.874799			Reelin signaling pathway; Lissencephaly gene (LIS1) in neuronal migration and development; Netrin-mediated signaling events;	
LRRFIP1 se	0	6.925394	9.526533	0	-6.649	0.194924	-0.31515	-6.06174	p18854	Leucine-rich	8	0.45262		PKCinh2 (Bisindolylmaleimide-1);		
LMO7 seq:	1.879669	4.574309	2.596767	3.40087	0.477877	0.390108	0.529261	0.652056	p17026	LIM domain	8	1.812615				
LMO7 seq:	0	8.20789	0	0	0.767645	1.149443	1.117302	0.900976	p00672	LIM domain	8	1.517907				
LMO7 seq:	5.593362	9.664597	0	0	0.346418	0.100478	0.111084	0.486253	p09803	LIM domain	8	2.037774				
LMO7 seq:	5.960504	5.090526	0	0	0.6557	-0.00547	0.722609	0.949786	p04876	LIM domain	8	1.671707				
LCP1 seq: 4	0	7.348156	0	0	-0.46213	-0.3391	0.059206	-0.14881	p16461	Plastin-2	8	0.807166				
LAP3 seq: 2	7.661761	7.000567	7.292225	0	0	0	0	0	p19653	Cytosol ami	8	2.744319				
LAMP2 seq	0	8.885337	0	0	0	0	0	0	p13880	Lysosome-a	8	1.110667				
LAMA3 seq	0	6.443585	4.964121	6.466806	0.146252	-0.00386	0.119267	0.278529	p15041	Laminin sub	8	2.301837			Alpha6 beta4 integrin-ligand interactions; Syndecan-4-mediated signaling events; Syndecan-2-mediated signaling events; Validated transcriptional targets of AP1 family members Fra1 and Fra2; a6 [illegible]	
LAD1 pS38	0	8.250437	7.036814	6.479586	0.795106	0.521977	0.48388	0.896087	p09400	Ladinin-1	8	3.057986				
KRT8 seq: 2	9.743358	11.04473	0	0	0	0	0	0	p08254	Keratin, typ	8	2.598511			Signaling me	GO:0033209; P:tumor necrosis factor-mediated signaling pathway;
KRT7 seq: 8	3.255689	5.162072	1.868978	0	2.703981	1.713133	1.177508	2.374247	p00894	Keratin, typ	8	2.281951				
KRT19 seq:	8.468118	9.542032	5.185406	0	-3.85201	-2.35231	-6.88797	-6.84711	p02579	Keratin, typ	8	0.407019			Signaling mediated by p38-alpha and p38-beta;	
KRT18 seq:	9.472229	10.45677	0	0	-6.40935	0.666784	0.932132	0.262161	p00013	Keratin, typ	8	1.922591			Caspase cas	GO:0033209; P:tumor necrosis factor-mediated signaling pathway;
KRT18 seq:	7.708505	10.06759	8.351917	0	0	0	0	0	p17834	Keratin, typ	8	3.266002			Caspase cas	GO:0033209; P:tumor necrosis factor-mediated signaling pathway;
KMT2B seq	0	9.721426	0	0	0	0	0	0	p11709	Histone-lys	8	1.215178				
KIF5B seq:	8.210024	7.895743	0	0	0	0	0	0	p09022	Kinesin-1 h	8	2.013221			N-cadherin signaling events;	
KIF16B seq	9.540864	9.2364	0	0	0	0	0	0	p17267	Kinesin-like	8	2.347158				GO:0008543; P:fibroblast growth factor receptor signaling pathway; GO:0007173; P:epidermal growth factor receptor signaling pathway;
KANSL1 sec	4.189088	3.834376	2.80666	1.413267	0.358849	-0.6388	-0.56496	0.109565	p12562	KAT8 regula	8	1.438505				
JUN seq: 22	0	10.84394	0	0	0.255043	0.082755	0.374868	0.436754	p11040	Transcriptic	8	1.499169	GSK3B; CK2	Up with IGF	JNK signalir	GO:0060395; P:SMAD protein signal transduction; GO:0051403; P:stress-activated MAPK cascade; GO:0034166; P:toll-like receptor 10 signaling pathway; GO:0034146; P:toll-like re [illegible]
JUN pS243	7.059013	9.802768	0	0	-3.28036	-0.56947	-1.00487	-1.39132	p11037	Transcriptic	8	1.32697	GSK3B; CK2	Up with IGF	JNK signalir	GO:0060395; P:SMAD protein signal transduction; GO:0051403; P:stress-activated MAPK cascade; GO:0034166; P:toll-like receptor 10 signaling pathway; GO:0034146; P:toll-like re [illegible]
ITGB1BP1 s	6.93548	7.063704	5.938405	0	0.404903	0.609293	0.762349	0.04844	p03885	Integrin bet	8	2.720322				GO:0032148; P:activation of protein kinase B activity; GO:0044344; P:cellular response to fibroblast growth factor stimulus; GO:0035924; P:cellular response to vascular endothelial [illegible]
IRF2BP2 se	5.225171	4.678054	4.001338	4.933507	0	0	0	5.50657	p06804	Interferon r	8	3.04308				
IDH2 seq: 2	7.506484	8.208727	0	0	0	0	0	0	p16655	Isocitrate de	8	1.964401				
HUWE1 sec	3.946938	3.316297	2.546785	2.911201	0	0	0	0	p06621	E3 ubiquitin	8	1.590153		CAMKinh2	p53 pathway; Validated targets of C-MYC transcriptional activation;	
HSPA9 seq:	12.10888	12.01843	0	0	0	0	0	11.62234	p01642	Stress-70 p	8	4.468707				
HSPA4 seq:	8.602875	9.123779	0	5.107103	0	0	0	0	p02070	Heat shock	8	2.85422			Validated targets of C-MYC transcriptional activation;	
HSP90B1 s	6.269105	8.71039	7.827041	0	0	0	0	0	p15068	Endoplasmi	8	2.850817			IL6-mediate	GO:0006987; P:activation of signaling protein activity involved in unfolded protein response; GO:0002224; P:toll-like receptor signaling pathway;
HSP90AA1	0	10.65854	0	0	0	0	0	0	p17466	Heat shock	8	1.332318			ErbB recept	GO:0038096; P:Fc-gamma receptor signaling pathway involved in phagocytosis; GO:0007165; P:signal transduction;
HSD17B4 s	8.576266	9.512359	0	5.093613	3.604562	1.848813	0	0	p03081	Peroxisoma	8	3.579452				
HSD17B4 s	7.278922	9.051513	0	6.896309	0	0	0	0	p16288	Peroxisoma	8	2.903343				
HPRT1 seq:	6.971142	7.622276	6.872672	0	0.653731	1.05259	0.477215	0.600492	p01396	Hypoxanthi	8	3.031265				
HNRNPM s	2.574826	4.911365	2.98661	2.00984	0.444356	1.349539	-1.65222	1.538063	p10105	Heterogene	8	1.770298				
HNRNPK se	10.90712	10.04312	7.014074	0	-6.04199	-5.98765	-5.49559	1.311522	p14128	Heterogene	8	1.468826				GO:0007165; P:signal transduction;
HNRNPK se	3.866375	3.8736	-0.37324	1.905626	1.895328	1.608561	1.515516	2.264757	p15138	Heterogene	8	2.069565				GO:0007165; P:signal transduction;
HNRNPK se	3.893787	4.146347	2.507716	2.309406	-2.16079	-0.81893	-2.52556	-0.25236	p13098	Heterogene	8	0.887452		Up with IGF - down with		GO:0007165; P:signal transduction;
HNRNPH1	0	10.37198	0	8.02041	0	0	5.147077	7.98384	p13804	Heterogene	8	3.940414				
HNRNPH1	0	8.772599	0	0	0	5.538448	0	7.273618	p18568	Heterogene	8	2.698083				
HNRNPD se	4.949893	10.18786	0	0	0	0	10.2364	0	p13773	Heterogene	8	3.171769				
HNRNPA1 s	10.04346	9.121159	7.110789	0	0.17792	1.037379	1.074606	2.06438	p12986	Heterogene	8	3.828712			Coregulation of Androgen receptor activity;	
HNRNPA1 s	0	7.406361	6.693136	0	-4.00252	-0.97625	-1.74026	-6.60279	p18047	Heterogene	8	0.09721			Coregulation of Androgen receptor activity;	
HMHA1 pS	0	6.050619	7.653976	0	0.040897	0.300239	0.106527	0.126081	p20562	Minor histo	8	1.784792				GO:0051056; P:regulation of small GTPase mediated signal transduction; GO:0007264; P:small GTPase mediated signal transduction;
HIVEP1 pS1	9.557445	8.725345	7.986563	0	0	0	0	0	p01395	Zinc finger p	8	3.283669				
HIRIP3 pS3	3.699738	3.379526	3.711214	2.295707	0.247217	0.605146	0.418048	0.49432	p07136	HIRA-intera	8	1.856364				
HDGF pS13	3.238154	3.403451	2.837799	2.360821	2.141002	1.855702	0.522124	2.187289	p15600	Hepatoma-	8	2.318293				GO:0006987; P:activation of signaling protein activity involved in unfolded protein response; GO:0007165; P:signal transduction;
HBP1 seq:	3.945	3.612465	4.546032	4.65722	4.75287	4.224995	4.541838	4.432892	p03765	HMG box-c	8	4.339164			C-MYC path	GO:0016055; P:Wnt signaling pathway;
GSTO1 seq:	0	10.52716	0	0	-6.28097	2.870532	0.058455	2.347813	p14838	Glutathione	8	1.190374				
GSTM3 seq	6.65193	8.981134	7.695301	0	0	0	0	0	p19043	Glutathione	8	2.916046				
GSN seq: 2	2.589612	5.562078	1.211801	-1.07585	-5.99975	0.970716	0.49099	0.543298	p19388	Gelsolin	8	0.536613			Osteoponti	GO:0048015; P:phosphatidylinositol-mediated signaling;
GSDMD sec	6.060108	9.289897	0	5.9489	-0.04122	-0.46955	-0.41242	-0.20804	p03467	Gasdermin-	8	2.520959				
GSDMD sec	6.222119	5.113006	0	0	0.193318	-1.10251	-1.00879	-0.60168	p03469	Gasdermin-	8	1.101934				
GOT1 seq:	6.393244	8.55463	4.97713	0	6.351176	7.7006	0	0	p14284	Aspartate a	8	4.247098				
GOLGB1 se	7.229192	5.851097	0	0	0	0	0	0	p19728	Golgin subf	8	1.635036				
GOLGB1 se	3.882106	4.842951	3.406679	3.853544	0	0	0	0	p08361	Golgin subf	8	1.99816				
GOLGA3 se	0	9.300981	0	6.366505	6.696757	0	0	0	p19452	Golgin subf	8	2.79553				
GOLGA3 se	0	6.396709	5.85829	6.022086	-0.49228	0.412293	-0.38953	-0.24017	p19447	Golgin subf	8	2.195924				
GCN1L1 se	6.163885	9.267047	0	5.954049	5.414213	0	0	0	p15742	Translation	8	3.349899				
GART seq: 5	0	8.575495	0	6.599451	2.490496	0	1.179801	2.116401	p01222	Trifunction	8	2.620205				
GAPVD1 se	9.899695	0	0	8.99902	1.292627	1.721958	0.547895	2.874026	p04752	GTPase-acti	8	3.166903				GO:0051056; P:regulation of small GTPase mediated signal transduction; GO:0007165; P:signal transduction;
GAPDH pT1	3.157219	4.12611	1.383456	0.436169	-1.65299	-0.61227	-0.89861	0.068286	p01384	Glyceraldeh	8	0.750921		PKCinh2 (B	Validated targets of C-MYC transcriptional activation;	
G6PD seq:	8.877557	9.462921	0	0	0	0	0	0	p12794	Glucose-6-p	8	2.29256				
FSCN1 seq:	0	7.132375	0	0	2.904156	2.762514	1.757636	3.026007	p00230	Fascin	8	2.197836				
FRMD6 pS5	2.586726	4.00009	3.174557	3.363744	0.440358	0.453649	-0.23161	0.012331	p15372	FERM doma	8	1.72498				
FOSL2 pS2	4.887948	5.398445	3.959206	4.425811	-0.06551	0.249262	-0.17	0.090637	p06645	Fos-related	8	2.346975			Validated transcriptional targets of deltaNp63 isoforms; AP-1 transcription factor network;	
FLNB seq: 2	8.753929	9.471085	0	0	0	0	0	6.667629	p19876	Filamin-B	8	3.11158				GO:0019221; P:cytokine-mediated signaling pathway; GO:0007165; P:signal transduction;
FLNA seq: 7	0	7.186203	0	0	2.677421	2.300079	2.643797	3.726482	p18463	Filamin-A	8	2.316748			Noncanoni	GO:0043123; P:positive regulation of I-kappaB kinase/NF-kappaB signaling;
FLNA seq: 2	8.866495	9.29829	0	0	3.454205	2.794047	-5.57196	3.688039	p20608	Filamin-A	8	2.81614			Noncanoni	GO:0043123; P:positive regulation of I-kappaB kinase/NF-kappaB signaling;
FLNA seq: 1	0	7.65504	0	0	2.850966	2.552287	0.938006	2.45197	p01606	Filamin-A	8	2.056034			Noncanoni	GO:0043123; P:positive regulation of I-kappaB kinase/NF-kappaB signaling;
FLNA pS96	2.570013	4.721381	1.993396	2.078875	0.388541	0.260812	0.34922	0.377904	p04668	Filamin-A	8	1.592518			Noncanoni	GO:0043123; P:positive regulation of I-kappaB kinase/NF-kappaB signaling;
FLNA pS21	9.795553	10.10542	0	0	0.165552	0.230967	0.197275	0.296805	p19879	Filamin-A	8	2.598946	PKACA; PAK	AKTinh1 (Al	Noncanoni	GO:0043123; P:positive regulation of I-kappaB kinase/NF-kappaB signaling;
FKBP1A sec	0	7.195077	0	0	0.70418	0.581321	0.202012	0.85764	p14959	Peptidyl-pro	8	1.192529			ALK1 pathw	GO:0007179; P:transforming growth factor beta receptor signaling pathway; GO:0043123; P:positive regulation of I-kappaB kinase/NF-kappaB signaling;
FKBP15 sec	9.342515	7.897313	0	8.138854	0.042904	-0.33669	-0.4252	0.224171	p17018	FK506-bind	8	3.110483				
FGD3 seq:	3.960849	5.562832	1.683158	3.283569	-1.31229	2.907681	-5.26701	3.225928	p11819	FYVE, RhoG	8	1.755589				GO:0051056; P:regulation of small GTPase mediated signal transduction; GO:0048011; P:neurotrophin TRK receptor signaling pathway; GO:0007264; P:small GTPase mediated signal [illegible]
FGD1 seq:	3.960849	5.562832	1.683158	3.283569	-1.31229	2.907681	-5.26701	3.225928	p14281	FYVE, RhoG	8	1.755589			Regulation	GO:0051056; P:regulation of small GTPase mediated signal transduction; GO:0048011; P:neurotrophin TRK receptor signaling pathway; GO:0007264; P:small GTPase mediated signal [illegible]
FEZF1 seq:	3.94345	4.08165	1.669292	0	0	2.029555	0.971391	0	p20542	Fez family z	8	1.586917				
FEN1 seq: 2	3.960849	5.562832	1.683158	3.283569	-1.31229	2.907681	-5.26701	3.225928	p19263	Flap endon	8	1.755589				GO:0048015; P:phosphatidylinositol-mediated signaling;
FARSA seq:	7.196652	6.102776	0	0	0	0	6.279527	0	p05091	Phenylalani	8	2.447369				
FAM182A s	6.907345	10.13308	0	5.84992	7.447925	0	0	0	p11248	Protein FAN	8	3.792284				
FAM171A2	8.572365	8.992061	0	6.766697	2.776777	4.883629	5.011278	-4.77806	p04294	Protein FAN	8	4.028093				
FAM129B p	7.433627	8.538403	0	0	0.65473	0.809619	0.44906	0.054125	p07684	Niban-like p	8	2.242446		EGFRinh1 (PD-168393); CAMKinh2 (KN-62);		
FAM117A s	4.533971	4.954608	4.184797	3.771431	-0.38601	-0.52632	-6.89545	-0.29418	p13331	Protein FAN	8	1.167855				
ETFB seq: 1	8.205075	11.02439	0	0	5.966354	0	0	0	p17631	Electron tra	8	3.149477				
ERRFI1 seq:	3.039417	7.20099	0	0	-0.8829	-0.65443	-0.82124	-0.99634	p06674	ERBB recept	8	0.860686				
ERRFI1 seq:	0	6.736292	0	0	0.222142	0.144466	0.005935	0.536888	p10464	ERBB recept	8	0.955715				
ERRFI1 seq:	8.933866	8.218578	0	5.007128	0.634747	0.615214	0.880702	1.002658	p12880	ERBB recept	8	3.161612				
ERRFI1 seq:	0	8.88268	7.001442	0	0.261846	0.252235	0.355845	0.209937	p12882	ERBB recept	8	2.120498				
ERRFI1 pS4	0	10.04756	0	0	0.175243	0.122329	-0.01964	0.01808	p14723	ERBB recept	8	1.292946				
ERRFI1 pS2	0	8.264758	0	0	-0.04632	-0.12885	-0.12872	-0.0485	p05582	ERBB recept	8	0.989045				
ERCC6L seq	6.785251	5.515033	0	0	-2.81213	-0.99346	-2.5643	-4.64146	p03568	DNA excisic	8	0.161117			PLK1 signaling events;	
ERBB2 seq:	4.338099	5.296997	1.775953	2.995866	0	0	0	0	p05759	Receptor ty	8	1.800864		ERKinh1 (El	ErbB recept	GO:0070372; P:regulation of ERK1 and ERK2 cascade; GO:0014065; P:phosphatidylinositol 3-kinase signaling; GO:0023014; P:signal transduction by phosphorylation; GO:0007169 [illegible]
EPPK1 seq:	0	8.141298	0	0	0	0	0	0	p19203	Epiplakin	8	1.017662				
EPPK1 pS2	0	12.25556	0	0	0	0	0	0	p06772	Epiplakin	8	1.531945				
EPN3 pT49	5.428096	3.731278	1.9875	3.531395	0	0	0	0	p20942	Epsin-3	8	1.834783				
EPN1 seq: 4	10.02911	9.604025	0	0	0	0	0	0	p15256	Epsin-1	8	2.454141			Internalizati	GO:0042059; P:negative regulation of epidermal growth factor receptor signaling pathway; GO:0007219; P:Notch signaling pathway; GO:0007173; P:epidermal growth factor recepto [illegible]
EPHA2 seq:	0	8.438774	0	0	0.341889	0.405346	-0.32538	0.23031	p00839	Ephrin type	8	1.136368			EphrinA-EP	GO:0070848; P:response to growth factor; GO:0070372; P:regulation of ERK1 and ERK2 cascade; GO:0051898; P:negative regulation of protein kinase B signaling; GO:0048013; P:ep [illegible]
ENO1 seq:	10.27312	10.0983	0	0	-8.9333	-8.87895	1.09655	3.856143	p18038	Alpha-enola	8	0.938983			Notch signaling pathway; HIF-1-alpha transcription factor network; Validated targets of C-MYC transcriptional activation;	
ELL2 seq: 4	0	7.92185	6.632437	0	0.212606	-0.06373	0.178783	0.060226	p11174	RNA polyme	8	1.867771				
EIF6 seq: 14	0	8.754854	0	0	0	7.644614	0	6.659598	p07759	Eukaryotic t	8	2.882383				
EIF4G1 seq	5.466678	7.452023	0	0	0	5.384827	4.790611	6.584398	p17905	Eukaryotic t	8	3.709817			Validated ta	GO:0008286; P:insulin receptor signaling pathway; GO:0019221; P:cytokine-mediated signaling pathway;
EIF4G1 seq	2.738081	3.875287	4.585986	4.385072	1.124062	0.249722	1.515677	-0.63549	p05842	Eukaryotic t	8	2.229799		PAKinh (PF	Validated ta	GO:0008286; P:insulin receptor signaling pathway; GO:0019221; P:cytokine-mediated signaling pathway;
EIF4A3 seq:	8.147358	8.954831	6.129806	0	-2.35417	-2.93259	-2.80948	-2.32515	p15765	Eukaryotic i	8	1.601327				GO:0019221; P:cytokine-mediated signaling pathway;
EIF3I seq: 1	8.090438	9.142501	5.617261	0	-8.38594	-1.86409	-3.21313	-7.79868	p08247	Eukaryotic t	8	0.198545				
EI24 seq: 6	3.94345	4.08165	1.669292	0	0	2.029555	0.971391	0	p12685	Etoposide-i	8	1.586917				
EHBP1L1 se	7.181256	6.898565	0	6.846866	-0.91996	-1.09575	-1.36768	-1.08584	p13168	EH domain-	8	2.057182		Up with IGF - down with PI3Kinh2 (PI-103); Up with EGF - down with PI3Kinh2 (PI-103); Up with IGF - down with PI3Kinh1 (GDC-0941); Up with EGF - down with PI3Kinh1 (GDC-0941); PI3Kinh2 (PI-103); P [illegible]		
EEF2 seq: 1	8.817952	9.905151	8.219155	0	0	2.675766	1.610383	0	p00740	Elongation f	8	3.903551			mTOR signaling pathway;	
EEF1E1 seq	11.18209	12.08625	6.448558	0	-2.80834	-7.77161	-7.27955	0.719572	p08511	Eukaryotic t	8	1.572121				GO:0043517; P:positive regulation of DNA damage response, signal transduction by p53 class mediator;
EEF1A2 seq	7.508233	8.810147	7.490298	0	0	0	0	0	p01593	Elongation f	8	2.976085				
DPYSL3 seq	5.280213	6.717648	7.357835	0	7.105555	6.786203	0	0	p09870	Dihydropyr	8	4.155932				
DNAJB6 pS	5.628967	5.411951	0.062485	0.748679	-1.36046	-0.19881	-0.05783	-0.87923	p14894	DnaJ homo	8	1.16947				
DLGAP5 se	5.532497	7.390336	0	7.302892	-1.00901	-0.25124	0.40843	0.107384	p04940	Disks large-	8	2.435161		AKTinh1 (Al	Aurora A sig	GO:0007267; P:cell-cell signaling;
DIP2B seq:	3.022767	3.404348	3.605975	3.464618	0	7.342045	0	5.853733	p15238	Disco-intera	8	3.336686				
DHX9 seq:	8.062176	8.980605	0	0	0	0	0	0	p13250	ATP-depend	8	2.130348				
DESI1 pS25	0	8.286275	0	6.381178	-0.97775	-0.31679	-0.66645	-0.90178	p07098	Desumoyla	8	1.475586				
DDX5 seq:	0	10.13016	0	8.73242	0	0	0	0	p17536	Probable AT	8	2.357822			Direct p53	GO:0043517; P:positive regulation of DNA damage response, signal transduction by p53 class mediator;
DDIT4 seq:	6.593995	9.388329	0	8.660304	-2.50702	-2.7494	-6.39807	-0.55983	p09946	DNA damag	8	1.553539			mTOR signa	GO:0032007; P:negative regulation of TOR signaling; GO:0048011; P:neurotrophin TRK receptor signaling pathway;
DCP1A seq	8.297407	10.22474	0	0	-0.33057	-0.09344	-1.11533	-0.29499	p09221	mRNA-deca	8	2.085976		AKTinh2 (M	Regulation of nuclear SMAD2/3 signaling;	
DBR1 pS51	4.274019	4.46518	3.585498	2.493822	0.824782	1.294323	0.334074	1.142062	p07146	Lariat debra	8	2.30172				
DBNL seq:	2.5155	4.675472	1.710172	2.305786	0	2.415941	0.517163	0	p18288	Drebrin-like	8	1.767504			JNK signalir	GO:0016601; P:Rac protein signal transduction;
DAZAP1 sec	7.427156	6.326618	0	0	0	0	0	0	p16717	DAZ-associa	8	1.719222				
CYR61 seq:	0	10.33787	0	0	0.146812	1.475365	-0.11125	0.019501	p09347	Protein CYR	8	1.483537			Beta5 beta6	GO:0070372; P:regulation of ERK1 and ERK2 cascade; GO:0030513; P:positive regulation of BMP signaling pathway; GO:0045860; P:positive regulation of protein kinase activity;
CXorf67 pS	3.783291	3.392215	2.814405	2.083414	0	0	0	0	p04596	Uncharacte	8	1.509166				
CTNND1 se	4.951913	5.52027	0	5.763365	-0.0707	-0.43043	-0.06352	-0.061	p05283	Catenin delt	8	1.951237			E-cadherin :	GO:0090090; P:negative regulation of canonical Wnt signaling pathway; GO:0016055; P:Wnt signaling pathway;
CTDSPL2 pS	0	9.824603	0	6.995405	-0.44969	-0.05325	-0.7168	-0.17048	p12335	CTD small p	8	1.928723		Up with IGF - down with Akt-mTOR-p70S6K-PI3K; Up with EGF - down with Akt-mTOR-p70S6K-PI3K; Up with IGF - down with PKCinh2 (Bisindolylmaleimide-1); Up with EGF - down with PKCinh2 (Bisindolylm [illegible]		
CRTC2 seq:	7.697878	9.244757	0	0	-0.33144	0.522976	0.628337	0.46689	p06458	CREB-regula	8	2.278675	CDK2;		LKB1 signaling events;	
CRAMP1L s	2.086221	4.150948	3.938507	3.925142	-0.28206	-0.38541	-3.88911	-2.59833	p10010	Protein cran	8	0.86824				
CORO1C se	7.173347	8.17125	0	0	-0.58476	2.329104	1.218008	2.575192	p20478	Coronin-1C	8	2.610267				GO:0007165; P:signal transduction;
CNDP2 seq	0	6.648841	0	0	1.76684	2.403575	0.788806	2.378024	p08381	Cytosolic no	8	1.748261				
CLTC seq: 1	2.88556	4.312205	2.674466	2.545838	0.019144	0.853261	0.009469	0.585478	p20928	Clathrin hea	8	1.735678			FAS (CD95) signaling pathway; Arf6 trafficking events;	
CLTC seq: 1	10.47256	10.5826	0	0	0	0	0	0	p13255	Clathrin hea	8	2.631895			FAS (CD95) signaling pathway; Arf6 trafficking events;	
CLTB seq: 1	0	8.360856	5.6141	0	3.680846	4.254452	0	4.901913	p00732	Clathrin ligh	8	3.351521			Arf1 pathway;	
CIT pS480	9.468501	9.110925	5.191858	0	0	0	0	0	p06447	Citron Rho-	8	2.971411				GO:0035556; P:intracellular signal transduction;
CIRBP seq:	8.275498	11.05705	0	0	0	0	0	0	p11853	Cold-induci	8	2.416568				
CHERP seq:	0	9.415831	0	0	0	0	0	0	p18959	Calcium hor	8	1.176979				
CEP250 pS	7.817327	7.247821	0	0	0.113329	-0.08553	-0.69487	-0.11044	p11634	Centrosome	8	1.785954				
CELSR1 seq	0	8.452558	0	0	6.91539	0	0	0	p15833	Cadherin EG	8	1.920993				GO:0007266; P:Rho protein signal transduction; GO:0007218; P:neuropeptide signaling pathway; GO:0007186; P:G-protein coupled receptor signaling pathway;
CDV3 seq:	8.366685	7.277551	0	5.950132	0	0	5.757035	0	p08760	Protein CDV	8	3.418925				
CDC42EP3	0	7.569861	6.568083	6.179921	-0.00744	0.508252	-0.13774	0.403104	p11083	Cdc42 effec	8	2.635505				GO:0007165; P:signal transduction;
CDC42EP1	0	8.522401	0	0	-0.56233	0.156453	-0.71557	-0.51375	p10240	Cdc42 effec	8	0.8609				GO:0007266; P:Rho protein signal transduction;
CDC42EP1	6.909775	8.968926	0	0	-0.12774	-0.03772	-0.42695	-0.10463	p10239	Cdc42 effec	8	1.897707				GO:0007266; P:Rho protein signal transduction;
CCT4 seq: 9	6.585094	8.360345	0	0	0	0	0	0	p19829	T-complex p	8	1.86818				
CCDC58 se	3.449417	3.285692	3.459114	2.464772	-0.25078	-0.09195	0.022603	-0.10302	p00489	Coiled-coil	8	1.52948				
CCDC12 se	8.949807	0	9.943784	0	-1.4311	-3.12856	-3.08154	-3.16228	p11508	Coiled-coil	8	1.011264				
CAPZB seq:	7.640209	9.860479	8.257083	0	5.672306	0	0	0	p18700	F-actin-cap	8	3.92876				
CALU seq: 2	9.394344	10.90919	6.073164	0	-0.37431	-0.27898	0.807289	-4.70087	p17838	Calumenin	8	2.728728				
CALML5 se	0	9.372422	0	0	0	0	0	0	p20101	Calmodulin	8	1.171553				GO:0007165; P:signal transduction;
CALM1 seq	0	10.22479	0	0	0	0	0	0	p01572	Calmodulin	8	1.278099			Regulation	GO:0022400; P:regulation of rhodopsin mediated signaling pathway; GO:0016056; P:rhodopsin mediated signaling pathway; GO:0043647; P:inositol phosphate metabolic process; [illegible]
CALB2 seq:	0	6.179091	0	5.304891	1.893049	0.919396	1.138056	2.904379	p10147	Calretinin	8	2.292358				
CACTIN seq	9.133508	9.673382	8.30286	0	-0.17413	-0.05391	-0.02093	0.028348	p11495	Cactin	8	3.36114				GO:0034122; P:negative regulation of toll-like receptor signaling pathway;
C2CD3 seq	8.585282	6.938726	0	6.380453	0	0	1.409534	0	p18580	C2 domain-	8	2.91425				GO:0008589; P:regulation of smoothened signaling pathway;
BUB3 seq:	6.907345	10.13308	0	5.84992	7.447925	0	0	0	p11537	Mitotic che	8	3.792284			p73 transcription factor network;	
BCLAF1 pS2	4.590868	4.549888	1.515788	1.009663	-5.31663	-0.52206	-0.42653	-0.21478	p09009	Bcl-2-assoc	8	0.648276				
BAIAP2L1 s	7.677934	8.35015	0	7.202666	-0.71525	-0.68087	-0.7653	-0.91111	p13956	Brain-specif	8	2.519777				GO:0007165; P:signal transduction;
BAIAP2L1 p	0	9.179419	7.703677	0	-1.10822	-0.16706	-1.15173	-0.84725	p13948	Brain-specif	8	1.701105				GO:0007165; P:signal transduction;
BAG6 seq:	6.588434	9.390051	0	0	-0.2348	0.420789	-7.55504	-7.51419	p15473	Large prolin	8	0.136905				
AUH seq: 2	6.01845	7.642137	0	6.246504	2.738219	2.255813	-0.14025	2.900922	p13433	Methylgluta	8	3.457725				
ATXN2L pS	7.057561	9.633956	0	7.488667	-0.14058	0.016375	-0.27733	0.063541	p15371	Ataxin-2-lik	8	2.980274				
ATP5B seq:	7.375711	11.55654	0	0	0	0	0	7.434619	p13860	ATP synthas	8	3.295859				
ATAD2B sec	9.104065	0	5.523603	0	-0.13251	-0.61667	-0.26751	-0.39146	p11392	ATPase fam	8	1.65244				
ASF1A seq:	9.733996	10.36915	0	0	0	6.347998	0	0	p16707	Histone cha	8	3.306393				
ASAP1 seq:	0	8.044462	0	5.31939	0.091286	0.725416	0.305707	0.19637	p10592	Arf-GAP wit	8	1.835329			Arf1 pathway; Signaling events mediated by focal adhesion kinase;	
ARID1B seq	0	9.705851	0	9.855432	0	0	0	0	p10961	AT-rich inte	8	2.44516				
ARHGEF7 p	3.571074	4.373823	3.090444	3.620585	0.564835	0.656708	0.113691	0.533096	p12742	Rho guanin	8	2.065532			Regulation	GO:0042059; P:negative regulation of epidermal growth factor receptor signaling pathway; GO:0051056; P:regulation of small GTPase mediated signal transduction; GO:0007173; P:e [illegible]
ARHGAP32	8.106229	9.161481	6.673743	0	-0.24326	-0.06146	-0.03091	-0.01019	p07828	Rho GTPase	8	2.949454			Neurotroph	GO:0051056; P:regulation of small GTPase mediated signal transduction; GO:0007264; P:small GTPase mediated signal transduction; GO:0007165; P:signal transduction;
ARHGAP32	9.138891	8.701502	0	5.557216	-0.18633	-0.21946	-0.0223	-0.29436	p01579	Rho GTPase	8	2.834395			Neurotroph	GO:0051056; P:regulation of small GTPase mediated signal transduction; GO:0007264; P:small GTPase mediated signal transduction; GO:0007165; P:signal transduction;
APRT seq: 1	6.647053	8.872292	7.963837	0	0	0	0	0	p11048	Adenine ph	8	2.935398				
APC seq: 24	8.17443	9.689805	0	0	-0.23332	-0.57561	-0.25706	-0.2844	p13006	Adenomato	8	2.064231			Degradatio	GO:0043409; P:negative regulation of MAPK cascade; GO:0060070; P:canonical Wnt signaling pathway; GO:0090090; P:negative regulation of canonical Wnt signaling pathway;
AP2B1 seq:	7.968656	7.008091	0	0	0	0	0	0	p12178	AP-2 compl	8	1.872093				GO:0042059; P:negative regulation of epidermal growth factor receptor signaling pathway; GO:0007173; P:epidermal growth factor receptor signaling pathway; GO:0048011; P:neuro [illegible]
AP1G1 seq	5.747817	11.1518	7.169616	0	0	0	0	0	p20094	AP-1 compl	8	3.008654				
ANXA3 seq	6.214495	7.170457	4.838787	0	0.990656	1.025778	0.986481	0.906007	p06023	Annexin A3	8	2.766582				
ANXA1 seq	9.764262	0	0	8.303919	1.205628	0.903785	0.685509	0.615225	p15995	Annexin A1	8	2.684791				GO:0007166; P:cell surface receptor signaling pathway; GO:0007165; P:signal transduction;
ANXA1 seq	7.773898	5.524343	0	6.175397	0.987587	0.678983	0.347493	0.678909	p02425	Annexin A1	8	2.770826				GO:0007166; P:cell surface receptor signaling pathway; GO:0007165; P:signal transduction;
AMOTL2 pS	2.729653	6.676876	-0.24953	1.74987	-0.28124	1.269312	-0.34842	0.066528	p11307	Angiomotin	8	1.451632				GO:0035329; P:hippo signaling; GO:0016055; P:Wnt signaling pathway;
AMBP seq:	9.356524	9.792656	0	0	0	0	0	0	p00656	Protein AM	8	2.393648				
ALDOC seq	0	10.7346	7.8972	0	0	8.648072	0	10.39306	p00443	Fructose-bi	8	4.709117				
AKT2 pS47	0	11.73614	11.63877	0	-8.19917	1.328588	0.941383	-7.61191	p02754	RAC-beta se	8	1.229225			E-cadherin :	GO:0008286; P:insulin receptor signaling pathway; GO:0007165; P:signal transduction;
AKT1 pS47	0	12.64237	12.71191	0	0	8.053652	7.853327	0	p06930	RAC-alpha s	8	5.157658	TBK1; PKD1; PKCB; PKC		E-cadherin :	GO:0048009; P:insulin-like growth factor receptor signaling pathway; GO:0071364; P:cellular response to epidermal growth factor stimulus; GO:0043491; P:protein kinase B signaling [illegible]
AKR1A1 sec	0	6.472275	0	0	0	0	0	0	p15620	Alcohol deh	8	0.809034				
AKAP13 sec	3.062709	3.525466	2.554351	2.7823	0.184506	-0.09049	1.450373	0.518801	p17376	A-kinase an	8	1.748502		AKTinh1 (Al	PAR1-medi	GO:0045859; P:regulation of protein kinase activity; GO:0051056; P:regulation of small GTPase mediated signal transduction; GO:0048011; P:neurotrophin TRK receptor signaling pa [illegible]
AKAP12 sec	5.116347	5.165828	0	0	0.346806	0.458019	0.066407	0.538142	p15783	A-kinase an	8	1.461444				GO:0007186; P:G-protein coupled receptor signaling pathway;
AIP seq: 10	6.787699	9.103925	0	0	0.004054	0.306114	-0.85585	0.832656	p09574	AH receptor	8	2.022325				GO:0007165; P:signal transduction;
AIM1 seq: 1	7.85839	9.338792	0	8.319251	2.684248	2.615149	2.765381	3.573071	p03552	Absent in m	8	4.644285			Neurotrophic factor-mediated Trk receptor signaling;	
AHNAK seq	8.240588	8.098804	0	0	-2.1378	-1.54346	-1.57873	0.388431	p15920	Neuroblast	8	1.433479		Up with IGF - down with ROCKinh1 (H-1152); Up with IGF - down with PKCinh2 (Bisindolylmaleimide-1); Up with IGF - down with PKCinh1 (Go-6976); Up with IGF - down with p70S6Kinh1 (PF-4708671); R [illegible]		
AHNAK seq	0	9.680337	0	0	-0.01506	0.313691	-0.28485	-0.4902	p19580	Neuroblast	8	1.15049	CDK1;			
AHNAK seq	5.649756	8.646154	0	8.457358	-0.05442	-7.73564	-2.53361	-1.01911	p16166	Neuroblast	8	1.426312				
AHNAK pT4	0.614991	5.258351	1.477749	2.572519	-0.7508	-0.05426	-0.26741	-0.25615	p01292	Neuroblast	8	1.074375				
ACTN4 seq:	0	7.599075	6.230569	0	0	0	0	6.617205	p13729	Alpha-actin	8	2.555856			PDGFR-beta signaling pathway;	
ACTN4 seq:	0	9.879449	0	0	6.078679	0	5.768209	0	p08699	Alpha-actin	8	2.715792			PDGFR-beta signaling pathway;	
ACTN2 seq:	0.077225	5.335767	2.86096	2.272899	1.831402	2.002125	-1.0793	-1.33436	p17724	Alpha-actin	8	1.49584				
ACTG1 seq:	0	10.9073	0	0	0	9.273044	0	9.011884	p13335	Actin, cytop	8	3.649029				GO:0038096; P:Fc-gamma receptor signaling pathway involved in phagocytosis;
ACTB seq: 2	3.437456	4.585341	1.790442	-0.67185	-0.53927	0.687406	0.483425	-0.20309	p17562	Actin, cytop	8	1.196233				GO:0038096; P:Fc-gamma receptor signaling pathway involved in phagocytosis;
ACSL1 seq:	6.593995	8.227115	0	8.660304	-2.62641	-6.89013	-6.39807	-0.60814	p09298	Long-chain-	8	0.869833				
AAK1 seq: 6	9.863659	5.922764	0	0	-0.22647	-0.3451	-0.07256	0.211236	p07267	AP2-associa	8	1.919191				GO:0045747; P:positive regulation of Notch signaling pathway;
ZFAND2B s	0.291776	3.314346	-1.63169	-7.60455	9.158978	5.290807	5.519198	7.100163	p20644	AN1-type zi	7	2.679877				
YWHAG sec	0.169671	2.188376	-7.90765	-7.73206	6.411795	6.561591	8.122704	6.212497	p03097	14-3-3 prot	7	1.753365			p38 signalir	GO:0009966; P:regulation of signal transduction; GO:0006469; P:negative regulation of protein kinase activity;
YWHAB seq	0.503442	1.226299	-0.69772	-0.47451	5.065529	5.884238	6.057103	5.94331	p03094	14-3-3 prot	7	2.938461			p38 signalir	GO:0035329; P:hippo signaling; GO:0000186; P:activation of MAPKK activity; GO:0000165; P:MAPK cascade; GO:0007265; P:Ras protein signal transduction; GO:0008286; P:insuli [illegible]
YTHDF3 sec	-0.77521	0.39136	-0.17428	-0.87809	7.37159	0	7.54873	9.713268	p16322	YTH domain	7	2.899671				
WDR1 seq:	0.073099	0.346018	1.390414	0.757879	10.70295	11.22351	11.87263	10.36049	p16456	WD repeat-	7	5.840874				
WBP11 seq	0	7.71631	0	0	5.664205	6.247035	5.864808	6.966209	p16876	WW domain	7	4.057321				
VCP seq: 21	4.685514	7.395276	0	3.772811	7.20995	6.47493	4.830907	8.021014	p12672	Transitional	7	5.2988				
VBP1 seq: 8	-1.61553	0.629917	-7.60552	-7.42994	7.987417	7.573095	0	8.402824	p07801	Prefoldin su	7	0.992783				
USP5 seq: 4	0.643281	-8.14717	-0.04744	-1.97743	4.538348	5.200307	6.672247	6.577869	p16579	Ubiquitin ca	7	1.682502				
USP5 seq: 2	-1.51888	0.559419	-2.91409	-4.5165	8.43873	7.290504	0	7.176358	p17063	Ubiquitin ca	7	1.814443				
USO1 seq: 8	-1.09261	-1.06083	-1.89626	-1.1332	8.494245	7.542383	4.589172	7.8786	p10840	General ves	7	2.915189			Arf1 pathway;	
USO1 seq:	-1.00474	-0.48863	-1.80528	-1.66911	7.175836	5.682067	7.500155	5.877773	p03744	General ves	7	2.65851			Arf1 pathway;	
UNCX seq:	-0.40666	-0.90144	-0.1662	0.016613	0	6.75305	6.935435	7.613419	p19548	Homeobox	7	2.480528				
UHRF1BP1	0.203775	-0.18299	-0.22163	0.824666	6.644388	5.670127	4.283587	5.614203	p04864	UHRF1-bin	7	2.854516				
UHRF1BP1	-1.47358	-1.46709	-1.84313	-1.38464	10.25855	10.06252	6.86128	9.8084	p11312	UHRF1-bin	7	3.852789				
UBE2N seq	-1.91702	1.911399	-2.43383	-7.5157	7.037428	7.394598	5.504691	8.048911	p11963	Ubiquitin-c	7	2.25381			IL1-mediate	GO:0070423; P:nucleotide-binding oligomerization domain containing signaling pathway; GO:0035872; P:nucleotide-binding domain, leucine rich repeat containing receptor signalin [illegible]
UBE2K seq:	-1.27548	-0.94943	-1.33476	-2.5749	8.068662	6.933495	0	7.520405	p00159	Ubiquitin-c	7	2.048499				
UBA1 seq: 6	0.203244	1.038889	-0.18298	-0.54685	6.82834	7.268408	0	7.414133	p12252	Ubiquitin-li	7	2.752898				
UBA1 seq:	0.527633	1.278352	0.429753	0.664643	10.9796	11.0965	9.111615	11.19831	p06443	Ubiquitin-li	7	5.660801				
TXNDC17 s	-1.31944	-1.83614	-2.68036	-1.6073	8.930638	6.712498	8.084828	9.05198	p00748	Thioredoxin	7	3.167088				GO:0033209; P:tumor necrosis factor-mediated signaling pathway;
TUBB6 seq:	0.574287	1.553974	-1.0487	-0.8078	6.396271	7.221762	7.85528	6.645746	p10236	Tubulin bet	7	3.548853				
TUBB4B sec	0.574287	1.553974	-1.0487	-0.8078	6.396271	7.221762	7.85528	6.645746	p09914	Tubulin bet	7	3.548853				
TUBB4A pM	0.574287	1.553974	-1.0487	-0.8078	6.396271	7.221762	7.85528	6.645746	p10279	Tubulin bet	7	3.548853				
TUBB seq: 1	-2.34948	-2.76714	-3.72337	-3.23103	11.33146	0	0	9.581014	p13947	Tubulin bet	7	1.10518				
TRIM28 sec	-1.14442	2.398046	-9.16908	-3.1029	6.875494	6.772965	0	8.089857	p13571	Transcriptic	7	1.339996			ATM pathway; p53 pathway; E2F transcription factor network; C-MYB transcription factor network;	
TPRN seq: 4	-0.19801	-0.07303	-0.15673	0.009429	8.684355	0	8.846231	10.21361	p00483	Taperin	7	3.415732				GO:0019221; P:cytokine-mediated signaling pathway;
TPM3 seq:	1.072497	2.417827	-0.98544	-2.43811	7.230525	6.680284	6.142801	7.840443	p10413	Tropomyos	7	3.495104				
TPI1 seq: 2	3.454751	1.898856	-7.42177	1.299207	0	9.539322	8.239903	7.181094	p17532	Triosephos	7	3.02392				
TPD52 seq:	-0.33628	0.850733	-1.52332	-1.61317	9.072348	5.950837	5.765368	7.719188	p19666	Tumor prot	7	3.235713				
TPD52 seq:	0.24977	1.390649	0.885296	-1.08581	9.041065	8.716057	7.316939	9.100953	p11757	Tumor prot	7	4.451864				
TP53BP1 p	0.327206	0.772813	0.057406	0.544504	8.231464	8.908455	9.274741	9.772479	p17897	Tumor supp	7	4.736134		AKTinh2 (M	ATM pathway;	
TP53BP1 p	0.774114	1.012365	0.807022	0.328114	0	8.421597	10.28249	10.211	p14440	Tumor supp	7	3.979588			ATM pathway;	
TP53BP1 p	-0.31508	-0.4556	0.582357	0.38494	0	6.912461	9.142141	6.330477	p06046	Tumor supp	7	2.822713			ATM pathway;	
TOMM20 s	-1.06314	-1.24363	-1.95766	-0.98914	10.02339	7.06314	7.721429	6.378663	p11064	Mitochond	7	3.241632				
TNKS1BP1	0	0	0	0	10.01363	8.439817	9.289908	9.358786	p06359	182 kDa tar	7	4.637767				
TMPO seq:	-0.47255	0.440277	-2.23886	-1.4205	10.15513	7.124205	7.0221	7.561443	p00336	Lamina-asso	7	3.521405				
TLN1 seq: 1	-0.92713	0.672349	-2.62426	-1.5602	8.172276	7.382999	0	7.966306	p20148	Talin-1	7	2.385292			Nectin adhe	GO:0006987; P:activation of signaling protein activity involved in unfolded protein response;
TKT seq: 54	-10.5783	0.690012	-2.19863	-2.8953	5.817685	5.769474	3.485334	6.021643	p12967	Transketola	7	0.763986				
TKT seq: 18	-0.658	-0.1443	-0.68932	-0.78319	8.735974	8.959575	8.546252	9.201535	p11710	Transketola	7	4.146067				
TJP2 seq: 6	0	7.783254	5.0313	0	9.283868	8.959692	8.073902	8.557385	p16201	Tight juncti	7	5.961175			Validated ta	GO:0035329; P:hippo signaling;
THOC7 seq	4.685514	7.395276	0	3.772811	7.20995	6.47493	4.830907	8.021014	p12681	THO compl	7	5.2988				
TGS1 seq: 5	-1.00042	1.549514	-2.45242	-2.13345	9.959914	8.479956	5.640819	9.434788	p13986	Trimethylgu	7	3.684837				
TCP1 seq: 5	-1.29	-0.07285	-2.10595	-3.56374	6.095659	8.227837	0	9.043949	p16832	T-complex p	7	2.041863				
TBC1D4 pS	5.00736	4.105651	5.589513	5.023389	9.436352	9.654701	10.28618	9.581302	p02088	TBC1 doma	7	7.335556			Insulin-mediated glucose transport; Class I PI3K signaling events mediated by Akt;	
TBC1D10A	-0.6849	-0.7249	-0.69739	-0.6982	6.706648	10.02371	6.713725	9.822369	p10867	TBC1 doma	7	3.807633				
TALDO1 sec	-2.46727	-2.16183	-3.80989	-2.83159	8.765702	8.150624	8.443753	6.72637	p10375	Transaldola	7	2.601984				
TAF15 seq:	0.563318	0.496279	-0.2832	-0.2944	7.269326	6.978515	7.264844	7.189804	p07827	TATA-bindir	7	3.648061				
TAF15 seq:	-11.4386	-1.07059	-0.15359	-0.34123	8.854934	0	7.953008	8.546525	p02980	TATA-bindir	7	1.543803		AKTinh1 (Akt Inhibitor VIII);		
TACC2 seq:	-2.63038	-2.75672	-3.66308	-10.4843	9.895325	5.842831	8.962965	7.812573	p08554	Transformir	7	1.6224				
SULT1A4 se	0.430065	2.43009	-0.57433	-7.15086	0	7.633557	4.792264	8.058228	p14068	Sulfotransfe	7	1.952377				
SUCLG2 sec	-0.07636	0.331397	-0.27231	-0.19317	5.278053	5.570477	5.52941	6.923202	p11979	Succinyl-Co	7	2.886338				
STRBP seq:	-0.37981	1.230992	-1.6291	-1.39614	6.512041	6.061803	5.657187	6.315502	p14951	Spermatid p	7	2.79656				
STIP1 seq: 5	2.812393	5.438927	0.822208	-6.02246	6.204195	8.833719	7.411879	9.377238	p18400	Stress-indu	7	4.359762				
STIP1 seq: 1	0	5.541375	1.878272	3.721236	4.620276	7.932501	6.966808	7.962632	p09500	Stress-indu	7	4.827887				
STIP1 pM5	-6.7337	1.113408	-2.4085	-6.41932	7.113287	5.426553	5.82657	8.543425	p11239	Stress-indu	7	1.557715				
STAT3 pM1	-1.44677	-0.87562	-1.36321	-1.82102	5.949118	6.485709	7.056564	6.030191	p04252	Signal trans	7	2.501871			IL27-media	GO:0045747; P:positive regulation of Notch signaling pathway; GO:0060397; P:JAK-STAT cascade involved in growth hormone signaling pathway; GO:0007259; P:JAK-STAT cascade; [illegible]
SSB seq: 27	-1.16916	0.711081	-0.93221	-1.5734	8.61109	8.266186	7.238963	8.489458	p17738	Lupus La pr	7	3.70525			Regulation	GO:0007265; P:Ras protein signal transduction; GO:0007218; P:neuropeptide signaling pathway; GO:0048011; P:neurotrophin TRK receptor signaling pathway; GO:0007165; P:signa [illegible]
SRSF6 pS31	-0.592	1.448912	0.285383	0.302374	6.468722	11.85129	11.47503	0	p03264	Serine/argin	7	3.904963				
SRSF5 seq:	-1.18093	1.408428	-1.31575	-2.52668	8.074084	8.15288	0	8.183644	p00756	Serine/argin	7	2.59946				
SRSF1 seq:	-2.0447	2.486587	-3.6106	-8.95736	8.590059	8.967913	0	9.808922	p00562	Serine/argin	7	1.905102				
SRP9 seq: 1	-0.64711	1.420138	-1.72453	-1.66166	9.286219	8.378494	7.204773	8.92494	p10299	Signal recog	7	3.897658			IL3-mediated signaling events;	
SRP72 seq:	0.107785	0.131114	0.262709	0.010963	9.173549	0	0	9.192027	p01891	Signal recog	7	2.359768				
SPTBN1 pS	0	0	0	0	11.45099	9.88655	11.01683	p06882	Spectrin be	7	4.044296				TGF-beta receptor signaling;	
SPAG9 pT2	-0.17288	1.84558	-0.43364	0.383945	5.272801	7.636875	6.488993	7.311481	p16063	C-Jun-amin	7	3.541645			Arf6 trafficking events;	
SNRNP70 s	-0.57247	0.980794	-0.36137	-1.05786	8.420689	8.548333	6.461694	8.364537	p18404	U1 small nu	7	3.848043				
SNIP1 seq:	0	0	0	0	9.335623	10.60138	10.11097	9.687527	p16887	Smad nucle	7	4.966938			Regulation	GO:0007249; P:I-kappaB kinase/NF-kappaB signaling;
SND1 seq:	0.606572	1.277747	0.375382	0.466308	8.394124	8.870841	9.024823	9.199935	p16967	Staphyloco	7	4.776966			C-MYB transcription factor network;	
SMG8 seq:	0.432315	1.072552	0.168853	0.28277	8.655443	9.903332	8.65559	8.656665	p08805	Protein SMG	7	4.72844				GO:0045859; P:regulation of protein kinase activity;
SLAIN2 seq	0.359421	-1.08185	0.306036	0.260474	6.976049	6.374949	0	7.522852	p06080	SLAIN motif	7	2.589742				
SIPA1L3 sec	-1.06744	-12.0824	-12.0941	-2.83727	0	12.99101	14.76942	13.88705	p08103	Signal-indu	7	1.69578				GO:0051056; P:regulation of small GTPase mediated signal transduction;
SIPA1L2 sec	-0.25607	-0.27147	-0.08067	-0.3602	8.784005	8.972183	9.023885	7.623351	p04811	Signal-indu	7	4.179377				GO:0051056; P:regulation of small GTPase mediated signal transduction;
SIN3A seq:	0.452476	0.482516	0.402656	0.262088	7.595393	7.284699	0	6.680325	p00216	Paired ampl	7	2.895019			Hedgehog signaling events mediated by Gli proteins; Signaling events mediated by HDAC Class I; Regulation of Telomerase; Regulation of nuclear SMAD2/3 signaling; C-MYB transcription factor ne [illegible]	
SHB seq: 2	1.0602	-8.412	1.262473	-8.24816	7.769962	6.689922	0	6.557481	p15862	SH2 domain	7	0.834984		AKTinh1 (Al	PDGFR-alph	GO:0009967; P:positive regulation of signal transduction; GO:0007179; P:transforming growth factor beta receptor signaling pathway; GO:0007267; P:cell-cell signaling; GO:000718 [illegible]
SHANK2 pS	0.157892	-0.22709	0.005877	-0.86107	7.628362	7.64024	6.572663	5.403074	p02682	SH3 and mu	7	3.289992				
SF3B3 seq:	-0.6485	1.755233	-7.84107	-4.33463	6.885308	7.117797	0	7.906347	p16635	Splicing fact	7	1.35506				
SEC31A seq	-0.10492	-0.08682	-0.35785	-0.42485	0	5.930371	7.175944	6.770778	p14199	Protein tran	7	2.362832				GO:0006987; P:activation of signaling protein activity involved in unfolded protein response;
SEC16A seq	0.075637	1.231364	0.02467	0.185116	0	10.7632	0	8.075593	p08200	Protein tran	7	2.544447				
SCRIB pS13	0.941782	0.22001	0.465323	0.555506	6.500746	6.343031	6.858317	5.81569	p06435	Protein scri	7	3.462551				
SARNP seq:	-1.45496	1.224922	-1.98556	-8.61917	9.531795	8.961518	7.905077	9.300127	p16752	SAP domain	7	3.107969				
SAFB2 seq:	-1.2491	-3.79969	-9.56927	-2.84078	9.417269	9.311719	7.268026	10.11891	p18744	Scaffold att	7	2.332135				
S100A13 se	0.726615	2.929437	-0.79082	-6.41195	6.843461	6.856172	4.465747	7.191192	p15566	Protein S10	7	2.726231				GO:0043123; P:positive regulation of I-kappaB kinase/NF-kappaB signaling;
RTCB seq: 4	-1.66457	0.886083	-2.75039	-1.29761	8.329562	8.696176	8.403571	9.522131	p03155	tRNA-splicin	7	3.765619				
RPS9 seq: 3	-12.7173	0.824743	-7.1569	-1.60582	14.73955	8.273388	0	8.848373	p11599	40S ribosor	7	1.400757				
RPS5 seq: 1	4.028738	3.098089	0.735965	-5.74813	7.521424	8.227028	7.434771	7.403426	p02736	40S ribosor	7	4.087664				
RPS3 seq: 1	-0.24927	2.606179	-5.89564	-5.72006	6.12658	7.428448	0	7.876342	p13958	40S ribosor	7	1.521573			Validated tr	GO:0008543; P:fibroblast growth factor receptor signaling pathway; GO:0007165; P:signal transduction;
RPS11 seq:	-7.22986	0.83289	-7.09106	-6.91548	7.91068	7.463405	6.117702	8.070983	p17958	40S ribosor	7	1.144908				
RPLP2 seq:	-1.33266	1.256915	-2.23491	-1.52743	5.658439	5.78915	5.405504	6.854292	p01350	60S acidic r	7	2.483662				
RPL9 seq: 1	0.739765	3.13182	-6.21355	-2.25934	6.469474	7.582067	5.990741	8.039806	p13243	60S ribosor	7	2.935097				GO:0007264; P:small GTPase mediated signal transduction;
RPL8 seq: 1	-1.36284	-0.98774	-1.63029	-2.54077	5.162965	5.23657	4.636501	5.602286	p14409	60S ribosor	7	1.764585				
RPL8 seq: 1	-4.7389	4.618162	-4.60011	-4.42452	8.351482	6.99332	6.822476	8.593654	p19615	60S ribosor	7	2.701946				
RPL6 seq: 1	-2.34089	-1.83324	-3.15236	-3.38151	8.737136	8.06052	7.364461	7.841084	p16395	60S ribosor	7	2.661899				GO:0016055; P:Wnt signaling pathway; GO:0007264; P:small GTPase mediated signal transduction;
RPL5 seq: 2	-8.57149	2.55725	-8.43269	-8.25711	10.86528	8.23553	5.530343	9.078714	p08669	60S ribosor	7	1.375728			p53 pathway;	
RPL4 seq: 2	0.053808	-1.1806	-0.6386	-0.40367	9.909868	9.767355	7.579332	10.66345	p10067	60S ribosor	7	4.468868				
RPL38 seq:	-1.87134	1.894004	-2.27404	-2.84454	10.60385	8.564699	5.686011	9.578161	p07884	60S ribosor	7	3.667101				
RPL24 seq:	0.316655	2.529937	-6.67623	-1.92919	8.738152	6.088407	5.981728	8.642443	p01531	60S ribosor	7	2.961488				
RPL23 seq:	1.52635	3.3931	-0.15261	-1.62319	8.643936	9.058499	0	9.224341	p11141	60S ribosor	7	3.758803			p53 pathway;	
RPL15 seq:	-0.19313	0.638618	-2.53996	-2.41589	8.239603	9.002202	7.19106	9.3676	p05077	60S ribosor	7	3.661263				GO:0007264; P:small GTPase mediated signal transduction;
RLBP1 seq:	0	7.783254	5.0313	0	9.283868	8.959692	8.073902	8.557385	p18287	Retinaldehy	7	5.961175				
RBM19 seq	-1.8001	-0.99955	-3.03352	-4.07564	7.427106	8.618513	6.482872	9.252622	p00304	Probable RI	7	2.734039				
RBL2 seq: 3	-7.55672	0.950156	-1.91299	-1.039	8.36666	9.37112	9.261616	0	p13642	Retinoblast	7	2.180105	CDK6; CDK4;		FoxO family	GO:0007165; P:signal transduction;
RBBP6 seq:	-0.17079	-0.21674	-0.31932	0.105195	0	8.297411	7.260228	8.579386	p00725	E3 ubiquitin	7	2.94192		CAMKinh1 (KN-93); AKTinh2 (MK-2206); AKTinh1 (Akt Inhibitor VIII);		
RAPH1 pS8	-0.1946	-0.59428	-0.24555	-0.64083	7.066214	5.786645	6.53496	8.511759	p08019	Ras-associa	7	3.278041				GO:0007165; P:signal transduction;
RAD23B se	0	7.468294	0	0	9.701463	6.458078	9.605943	9.901018	p08735	UV excision	7	5.39185				
PSMD11 se	0.561946	2.279799	-0.59841	-0.94306	6.469257	7.273452	0	6.50626	p10308	26S proteas	7	2.693656				
PSMC3 seq	0.096315	0.291217	-0.59667	-2.99908	7.514708	8.359197	8.679808	9.486844	p07756	26S proteas	7	3.854043				
PSMC1 seq	-0.51226	1.788719	-0.16787	-0.84824	10.43996	10.02926	7.811947	10.13989	p00620	26S proteas	7	4.835175				
PSMB3 seq	-3.59605	1.194064	-1.39057	-2.27276	6.447788	6.830186	0	7.99801	p16743	Proteasome	7	1.901334				GO:0035556; P:intracellular signal transduction;
PSMB1 seq	-0.56095	0.941857	-0.98871	-1.7547	7.008656	7.968494	6.379974	7.71774	p17736	Proteasome	7	3.339045				GO:0035556; P:intracellular signal transduction;
PSMA7 seq	-0.43918	1.008729	-0.2302	-1.70713	9.566692	0	6.827303	8.397064	p10471	Proteasome	7	2.927908				
PSMA6 seq	-0.14084	0.468803	-1.28479	-1.05779	5.58895	4.896771	4.905999	5.005611	p13619	Proteasome	7	2.297839				
PSMA6 seq	-1.2455	-0.3585	-0.01298	-0.26628	9.426923	9.406647	7.913813	9.75262	p00331	Proteasome	7	4.327093				
PSMA3 seq	-0.439	-0.4606	-0.90641	-1.23456	9.698747	9.63127	8.264117	8.820296	p04846	Proteasome	7	4.171731			Aurora B signaling;	
PSMA1 seq	1.588284	3.962716	-0.59823	-0.39612	7.440516	0	4.360407	6.928709	p10351	Proteasome	7	2.910785				
PRRC2A sec	3.426523	2.430042	3.624102	3.396762	5.480948	6.643981	7.290353	0	p15296	Protein PRF	7	4.036589				
PRPF38B p	0	0	9.657902	9.189244	5.616965	6.436627	9.891468	6.8619	p04194	Pre-mRNA-	7	5.956763				
PRDX6 seq	-0.15069	0.773977	-0.89389	-1.45061	7.457589	8.600916	5.275014	9.601785	p08899	Peroxiredox	7	3.651762				
PRDX3 seq	-0.76946	-0.83825	-1.32257	-1.09263	7.372414	7.176466	6.303672	7.838998	p15751	Thioredoxin	7	3.08358			Validated targets of C-MYC transcriptional activation;	
PRDX2 seq	1.166499	0.620055	1.497746	-6.31481	5.144485	10.19444	8.419137	10.35625	p03387	Peroxiredox	7	3.885476				
PRDX1 seq	3.139767	3.469381	0.928609	-0.7557	6.398667	6.701917	4.751933	6.947667	p11211	Peroxiredox	7	3.94778			Coregulation of Androgen receptor activity;	
PPP1R13L	6.327029	8.430405	8.293663	0	0	9.797338	8.070194	8.829612	p15219	RelA-associ	7	6.21853			p53 pathway;	
PPP1CA sec	-1.94553	1.018494	-8.88669	-2.81278	6.467526	6.727958	0	7.477414	p11401	Serine/threo	7	1.0058			ALK1 signal	GO:0030512; P:negative regulation of transforming growth factor beta receptor signaling pathway; GO:0007179; P:transforming growth factor beta receptor signaling pathway;
PPL seq: 77	-0.40247	1.710496	-1.426	0.596356	8.43823	8.497711	7.589183	8.393893	p09443	Periplakin	7	4.174674				
PPA2 seq: 1	-1.03307	0.123127	-1.20118	-1.55672	6.157777	5.498178	4.997037	6.150351	p13995	Inorganic py	7	2.391937				
PLEKHH1 s	-0.57901	0.948106	-0.62114	-2.5749	8.071504	6.933495	0	7.520405	p00166	Pleckstrin h	7	2.462308				
PLEKHG3 p	0	5.562238	0	0	6.631161	9.779398	9.266504	8.272101	p00145	Pleckstrin h	7	4.938925				
PLEC seq: 2	-0.95493	-8.36744	-1.0598	-8.2036	8.00411	6.910479	0	9.73757	p03872	Plectin	7	0.758298				
PLEC seq: 2	1.407069	2.726511	1.153207	-7.08179	10.7121	7.762743	8.647671	8.449062	p11022	Plectin	7	4.222072				
PLEC seq: 2	-1.15385	2.332334	-0.4177	-8.4164	9.501545	9.817762	8.846561	9.79078	p19732	Plectin	7	3.787628				
PLEC seq: 1	-0.51045	3.748729	-6.27353	-6.09794	9.455073	8.580065	0	9.172819	p01278	Plectin	7	2.259346				
PKM seq: 4	-0.57769	0.458825	-0.98581	-1.59808	11.43944	11.17418	9.089783	11.97139	p09026	Pyruvate kir	7	5.121505			HIF-1-alpha transcription factor network;	
PKLR seq: 4	-0.72953	-0.28143	-1.15451	-0.2302	6.600547	7.692608	5.214542	8.287129	p08389	Pyruvate kir	7	3.174894			FOXA2 and FOXA3 transcription factor networks;	
PHLDB2 se	0.461069	-6.56674	-6.57848	-6.4029	8.917431	6.409705	9.473683	8.79443	p19603	Pleckstrin h	7	1.813525				
PHGDH sec	-0.23104	0.808167	-0.37275	-1.05295	7.957816	7.590227	5.792326	8.721238	p14007	D-3-phospl	7	3.651629				
PHF8 seq: 6	0.292157	0.274487	0.493048	0.086062	8.864079	8.631443	8.976035	9.231328	p00906	Histone lysi	7	4.60608				
PGM3 seq:	-0.27126	1.663613	-0.41134	-0.3614	8.623616	7.113713	6.900362	7.488113	p20735	Phosphoac	7	3.843177				
PGLS seq: 2	-1.52354	1.131146	-1.91082	-3.07528	7.286242	5.244689	4.28879	6.842462	p11255	6-phospho	7	2.285461				
PGD seq: 7	-0.82675	0.745979	0.041571	-1.17877	8.993351	7.196929	6.999627	7.34851	p20594	6-phospho	7	3.665055				
PGD seq: 1	-7.75782	1.856301	0.200796	-2.27878	7.91103	6.439668	0	7.161934	p02715	6-phospho	7	1.691642				
PDIA4 seq:	-0.44128	0.788523	-0.90374	-1.3573	8.19445	7.619497	5.702178	8.061937	p17775	Protein disu	7	3.458033				
PDIA4 seq:	-0.73332	0.736868	-0.78447	-1.34832	9.120197	8.223457	5.531333	8.654497	p00790	Protein disu	7	3.675031				
PDIA3 seq:	-0.56505	1.964381	1.017733	-0.49063	3.831102	4.331484	4.178714	5.689084	p02843	Protein disu	7	2.494603				GO:0007165; P:signal transduction;
PDHB seq:	-0.20204	0.62209	-0.4613	-1.0901	7.586451	7.223891	5.83776	6.974518	p14072	Pyruvate de	7	3.311409				
PDHB seq:	-1.91702	1.911399	-2.43383	-7.5157	7.037428	7.394598	5.504691	8.048911	p13962	Pyruvate de	7	2.25381				
PDHB seq:	-0.68546	0.355989	-0.27161	-0.92439	7.783744	7.207117	6.031687	6.371905	p18878	Pyruvate de	7	3.233623				
PDCL seq: 2	0.729562	0.524885	0.742375	0.62468	0	6.556188	7.060555	6.218288	p09172	Phosducin-	7	2.807067	CK2A1;			GO:0008277; P:regulation of G-protein coupled receptor protein signaling pathway; GO:0035556; P:intracellular signal transduction; GO:0007165; P:signal transduction;
PDAP1 seq	0	0	0	0	6.630668	8.237597	7.971215	8.164392	p05280	28 kDa heat	7	3.875484				GO:0007165; P:signal transduction;
PCBP2 pS1	-1.19193	0.879337	0.070347	2.719728	7.931077	10.40621	10.9532	0	p08539	Poly(rC)-bir	7	3.970995				
PCBP1 seq:	-0.31117	0.981632	-0.40527	-1.18982	10.31097	9.642481	8.613292	10.33064	p20422	Poly(rC)-bir	7	4.746594				
PCBP1 seq:	-0.61943	0.960697	-2.63436	-0.72654	10.49649	5.5739	7.781036	8.699785	p10332	Poly(rC)-bir	7	3.691447				
PBRM1 pS3	-0.04931	0.227128	-0.43133	-0.4523	0	10.87239	11.17834	0	p10846	Protein poly	7	2.668115			p38 signalir	GO:0043122; P:regulation of I-kappaB kinase/NF-kappaB signaling; GO:0035924; P:cellular response to vascular endothelial growth factor stimulus; GO:0006469; P:negative regulatio [illegible]

PARD3 pS7	0	0	0	0	11.47429	11.40532	11.15969	0	p13731	Partitioning	7	4.254913		ROCKinh1 (H-1152);		GO:0007205; P:protein kinase C-activating G-protein coupled receptor signaling pathway; GO:0007179; P:transforming growth fact [illegible]
PALLD seq:	-2.33414	1.635821	-2.76337	-7.2814	6.56321	6.311804	0	6.438562	p14976	Palladin	7	1.071311				
PAK2 seq: 2	-2.04305	-0.12006	-8.15378	-2.6276	9.401924	8.891221	5.18165	8.306453	p02677	Serine/threo	7	2.354596			C-MYC path	GO:0023014; P:signal transduction by phosphorylation; GO:0006469; P:negative regulation of protein kinase activity; GO:0050852 [illegible]
PAFAH1B1	-1.27548	-0.94943	-1.33476	-2.5749	8.071504	6.933495	0	7.520405	p00452	Platelet-acti	7	2.048854			Reelin signaling pathway; Lissencephaly gene (LIS1) in neuronal migration and development;	
PA2G4 seq:	0	8.496171	6.63494	0	6.931018	6.400454	6.921964	6.984728	p10122	Proliferation	7	5.296159			Coregulation of Androgen receptor activity;	
OFD1 seq: 3	-1.27548	-0.94943	-1.33476	-2.5749	8.071504	6.933495	0	7.520405	p00158	Oral-facial-d	7	2.048854				
NUMA1 seq	-4.41049	1.449557	-0.99382	-7.67177	8.805452	7.698386	0	7.822033	p11321	Nuclear mit	7	1.587418			Caspase cascade in apoptosis;	
NSMCE2 se	0	0	0	0	0	5.287933	7.890223	5.996087	p09169	E3 SUMO-p	7	2.39678				
NSFL1C seq	6.581848	6.208403	-0.74032	-2.01341	5.557755	5.481056	8.416936	7.407112	p04523	NSFL1 cofac	7	4.612422				
NPM1 seq:	3.377608	2.146508	0.856302	-0.70207	8.351149	10.5885	8.546149	11.26158	p09869	Nucleophos	7	5.553216			BARD1 sign	GO:0007165; P:signal transduction;
NOS1AP se	0.147318	0.59115	0.295437	0.566911	9.801473	10.17641	9.215708	9.210173	p00851	Carboxyl-te	7	5.000572				
NONO seq:	-5.49121	3.127984	-5.35242	-5.17683	7.044236	7.903315	0	8.004211	p21075	Non-POU d	7	1.25741			Circadian rhythm pathway;	
NMRAL1 se	-1.47358	-1.46709	-1.84313	-1.38464	10.25855	10.10787	6.802802	0	p11471	NmrA-like fa	7	2.625097				
NME2P1 se	0.587627	2.813139	0.105791	-0.61665	7.908909	7.508414	5.47572	8.124197	p08561	Putative nu	7	3.988393				
NDRG2 pT3	0.221032	0.05561	0.185585	-0.1149	7.198246	7.752367	6.928509	0	p03989	Protein NDI	7	2.778306	SGK1; p90RSK; p70S6K;		Validated ta	GO:0070373; P:negative regulation of ERK1 and ERK2 cascade; GO:0016055; P:Wnt signaling pathway;
NCOA3 seq	0.593237	1.218772	-0.10826	1.027408	8.92697	9.203208	9.796408	7.827706	p17106	Nuclear rec	7	4.810681			Validated nu	GO:0030521; P:androgen receptor signaling pathway;
NCK2 seq: 8	-0.11289	-0.00987	-0.07479	0.273836	8.344723	7.868164	9.244958	8.596344	p18952	Cytoplasmic	7	4.266309			Reelin signa	GO:0007173; P:epidermal growth factor receptor signaling pathway; GO:0007165; P:signal transduction;
NAV1 pS37	0.125559	0.006193	0.341528	0.21385	10.30344	9.232351	10.76229	10.06057	p11955	Neuron nav	7	5.130722				
MYO6 seq:	0.125738	-0.09144	-0.11992	0.139598	8.626344	8.959384	8.710088	7.865907	p03229	Unconventi	7	4.276963			Stabilization	GO:0030330; P:DNA damage response, signal transduction by p53 class mediator;
MYL6 pM7	1.408024	0.636008	-0.18491	-2.491	9.314203	6.160403	6.553923	6.849657	p01263	Myosin ligh	7	3.530789				
MUC5B seq	-0.07974	-0.2022	0.068556	0.011532	9.232082	10.68338	9.659332	10.16975	p05010	Mucin-5B	7	4.942837				
MUC5B seq	0.620682	1.406693	0.09582	-0.19352	5.670285	8.218931	7.004685	7.405735	p20968	Mucin-5B	7	3.778664				
MTHFD2 se	4.685514	7.395276	0	3.772811	7.20995	6.47493	4.830907	8.021014	p13024	Bifunctiona	7	5.2988				
MSN seq: 3	-1.44412	-1.15869	-0.9533	-1.51346	5.171036	4.850183	4.126746	5.184465	p20277	Moesin	7	1.782858			Plasma membrane estrogen receptor signaling; RhoA signaling pathway;	
MMP3 seq:	-1.16916	0.711081	-0.93221	-1.5734	8.61109	8.266186	7.238963	8.489458	p16159	Stromelysin	7	3.70525			Urokinase-type plasminogen activator (uPA) and uPAR-mediated signaling; Posttranslational regulation of adherens junction stability and dissas [illegible]	
MIF seq: 2 -	-1.47332	0.251216	-2.01908	-1.45207	9.419155	6.124532	8.010403	8.417655	p08887	Macrophag	7	3.40981				GO:0043518; P:negative regulation of DNA damage response, signal transduction by p53 class mediator; GO:0030330; P:DNA dama [illegible]
MIF pM3	0.021028	0.590016	-0.5868	-0.5775	9.715776	9.760407	10.75399	12.85249	p08886	Macrophag	7	5.316176				GO:0043518; P:negative regulation of DNA damage response, signal transduction by p53 class mediator; GO:0030330; P:DNA dama [illegible]
MIA3 seq: 1	-0.09484	-0.29008	-0.08228	-0.63644	7.037283	8.344869	8.534584	8.639834	p18582	Melanoma i	7	3.931616				
ME1 seq: 5	-0.14686	0.840539	-0.74863	-1.00316	6.972164	5.67494	4.648906	6.224836	p03003	NADP-depe	7	2.807841				
MATR3 seq	1.831594	0.67814	-0.91506	-1.89824	6.553859	0	7.52458	8.219258	p14246	Matrin-3	7	2.749267				
MAPRE1 se	-0.59926	3.431295	-0.2581	-1.67755	6.980877	7.537691	6.070637	8.274568	p10473	Microtubul	7	3.72002			N-cadherin signaling events;	
MANF seq:	1.061737	1.218942	-1.81676	-7.8112	8.041764	7.719269	0	7.815729	p08536	Mesenceph	7	2.028686				
MAGED1 p	-0.43817	-0.26777	-0.79015	-1.13874	7.993478	0	0	11.33221	p01459	Melanoma-	7	2.086356			Neurotroph	GO:0048011; P:neurotrophin TRK receptor signaling pathway;
MADD seq:	0.08101	-1.47133	-0.20882	-0.90557	0	9.766324	0	10.22247	p09577	MAP kinase	7	2.18551			TNF recepto	GO:0000187; P:activation of MAPK activity; GO:0007166; P:cell surface receptor signaling pathway;
LUZP2 seq:	4.685514	7.395276	0	3.772811	7.20995	6.47493	4.830907	8.021014	p13078	Leucine zipp	7	5.2988				
LUZP1 seq:	-0.82724	-6.79039	-6.80213	-0.29232	8.516146	9.399875	9.245618	9.883216	p03600	Leucine zipp	7	2.791596				
LSM3 seq: 8	-2.359	0.910695	-1.7896	-8.49807	9.095465	8.356222	6.196379	8.882207	p16221	U6 snRNA-a	7	2.599288				
LSM14B se	0.070157	1.405845	-0.46999	-0.72172	6.126928	6.927014	0	6.415088	p05910	Protein LSM	7	2.469165				
LRRC59 seq	-0.44004	0.453645	-1.13395	-1.34296	8.198895	5.57404	4.621725	5.693564	p18429	Leucine-rich	7	2.703115				
LRBA seq: 1	0.866659	0.231178	0.082928	0.077786	0	7.23878	8.158669	7.091379	p11316	Lipopolysac	7	2.968423				
LPXN seq: 3	0	0	0	0	0	8.09063	11.18096	11.72051	p13271	Leupaxin	7	3.874012				GO:0007165; P:signal transduction;
LMNB2 seq	-0.99183	-8.4797	-8.49144	-3.46994	9.005917	7.57977	0	7.927741	p19304	Lamin-B2	7	0.385065			Caspase cascade in apoptosis;	
LMNB1 seq	-0.92284	1.841063	-3.3293	-1.70364	8.939024	8.191213	7.344356	8.605517	p08410	Lamin-B1	7	3.620675			Caspase cascade in apoptosis;	
LMNB1 pM	1.200202	0.929538	0.573596	-1.51902	0	6.387825	4.619946	7.163882	p10671	Lamin-B1	7	2.419496			Caspase cascade in apoptosis;	
LMNA seq:	0	0	0	0	6.152379	10.09819	7.666973	7.499638	p12991	Prelamin-A/	7	3.927147			Caspase cas	GO:0006987; P:activation of signaling protein activity involved in unfolded protein response;
LIMA1 seq:	-6.97535	2.367188	0.105544	-1.27084	6.96457	0	4.701504	7.829746	p01429	LIM domain	7	1.715295			Stabilization and expansion of the E-cadherin adherens junction;	
LIMA1 pM6	-0.89014	-1.76942	-1.42105	-1.37729	6.790335	8.697593	8.569741	9.727838	p17743	LIM domain	7	3.540951			Stabilization and expansion of the E-cadherin adherens junction;	
LIG1 seq: 6	-1.13898	-0.49274	1.057472	1.565979	0	6.121569	6.374391	8.5478	p01228	DNA ligase	7	2.754437	CK2A1; CDK2; CDK1;			
LDHA seq: 9	0	0	0	0	6.218455	0	6.716789	7.560121	p10412	L-lactate de	7	2.561921			HIF-1-alpha transcription factor network; Validated targets of C-MYC transcriptional activation;	
LDHA seq: 6	-0.18399	1.953629	-0.64057	1.332356	8.077188	8.778686	7.688923	8.867802	p18375	L-lactate de	7	4.484253			HIF-1-alpha transcription factor network; Validated targets of C-MYC transcriptional activation;	
LARP1 pS7	0.274983	-0.00564	0.648038	0.316601	12.41212	9.662045	0	11.98557	p05095	La-related p	7	4.411715	mTOR; CDK	Akt-mTOR-PI3K; PI3Kinh		GO:0031929; P:TOR signaling;
LAMC1 seq	0.998069	2.173531	0.249212	-0.04051	6.215216	7.234044	7.444254	7.487005	p18170	Laminin sub	7	3.970103			Alpha6 beta4 integrin-ligand interactions; Beta3 integrin cell surface interactions; a6b1 and a6b4 Integrin signaling; Beta1 integrin cell surface int [illegible]	
KRT8 seq: 1	-1.62471	2.5393	-1.65209	-7.1235	6.353219	0	7.280372	7.012081	p18123	Keratin, typ	7	1.598083			Signaling me	GO:0033209; P:tumor necrosis factor-mediated signaling pathway;
KRT74 seq:	-7.51399	3.408765	-0.86309	-7.19961	3.815376	5.691486	2.967793	5.945288	p16840	Keratin, typ	7	0.781503				
KRT19 seq:	0.098802	0.735648	0.502016	-0.43861	4.68445	4.504834	4.741421	5.10107	p14983	Keratin, typ	7	2.491204			Signaling mediated by p38-alpha and p38-beta;	
KRT18 seq:	-1.25687	-0.10995	-0.22041	-2.13575	6.597193	10.90079	0	10.08007	p03459	Keratin, typ	7	2.981884		Up with IGF	Caspase cas	GO:0033209; P:tumor necrosis factor-mediated signaling pathway;
KRT18 pM5	10.93726	10.66033	9.350709	0	8.056622	10.97824	11.06603	10.73693	p16016	Keratin, typ	7	8.973265			Caspase cas	GO:0033209; P:tumor necrosis factor-mediated signaling pathway;
KIF12 seq: 8	-1.47358	-1.46709	-1.84313	-1.38464	10.25855	7.617575	6.802802	0	p11799	Kinesin-like	7	2.31381				

[illegible]

FLNB seq: 7	-3.89311	1.630915	-4.30372	-7.65997	0	0	0	6.194654	p01074	Filamin-B	6	-1.0039			GO:0019221; P:cytokine-mediated signaling pathway; GO:0007165; P:signal transduction;
FLNB seq: 3	2.360876	3.201514	-8.03129	-2.11267	0	0	0	7.288955	p14251	Filamin-B	6	0.338423			GO:0019221; P:cytokine-mediated signaling pathway; GO:0007165; P:signal transduction;
FLNA seq: 6	-5.49429	4.283584	-5.35549	-5.1799	2.087301	2.15407	1.164863	2.497733	p14493	Filamin-A	6	-0.48027		Noncanoni	GO:0043123; P:positive regulation of I-kappaB kinase/NF-kappaB signaling;
FLNA seq: 1	-0.54365	1.339501	-2.26493	-8.6102	1.620146	1.247533	0.760874	1.597006	p01770	Filamin-A	6	-0.60672		Noncanoni	GO:0043123; P:positive regulation of I-kappaB kinase/NF-kappaB signaling;
FLNA seq: 1	-1.52238	1.54248	-4.18574	-6.33881	0.252949	0.563378	-0.20281	0.684788	p01421	Filamin-A	6	-1.15077		Noncanoni	GO:0043123; P:positive regulation of I-kappaB kinase/NF-kappaB signaling;
FLNA pS968	-2.75387	-1.88082	-13.0126	-0.5585	0.195414	0.541111	1.040589	0.688956	p04667	Filamin-A	6	-1.96747		Noncanoni	GO:0043123; P:positive regulation of I-kappaB kinase/NF-kappaB signaling;
FKBP4 seq:	-2.7806	2.517055	-7.0628	-6.88722	-0.92387	-0.63379	1.775482	1.810614	p10316	Peptidyl-pro	6	-1.52314		Coregulatio	GO:0030521; P:androgen receptor signaling pathway;
FGD6 seq: 4	0.199682	-1.3466	0.333089	-8.09683	0.037711	-0.55636	-0.47379	-0.1513	p10253	FYVE, RhoG	6	-1.2568			
FEN1 seq: 2	-2.09133	0.650463	-5.56629	-5.3907	3.046382	0.782315	-4.5955	3.606624	p05532	Flap endonu	6	-1.19475			GO:0048015; P:phosphatidylinositol-mediated signaling;
FASN seq: 1	-2.07114	-1.66608	-2.51134	-3.94626	0	0	0	0	p14802	Fatty acid sy	6	-1.27435		Validated transcriptional targets of deltaNp63 isoforms; p73 transcription factor network;	
FAM83H se	-2.78048	-9.0084	-9.02014	-0.32169	-0.40267	-0.56717	-0.59391	-0.20998	p10676	Protein FAM	6	-2.86305			
FAM65A se	1.403926	-0.18059	-10.0385	1.486373	0.722229	0.421968	0.202621	-0.28697	p16439	Protein FAM	6	-0.78361			
FAM13A se	-10.3434	-0.12188	-2.68972	-1.1533	-0.11815	-1.74271	-1.48398	-0.35052	p03692	Protein FAM	6	-2.25047			GO:0051056; P:regulation of small GTPase mediated signal transduction; GO:0007264; P:small GTPase mediated signal transduction;
FAM114A1	-1.14625	-0.11881	-1.17202	-9.12951	2.636658	2.243925	0.412898	1.512673	p12198	Protein NO	6	-0.59505			
EZR seq: 57	-0.42139	0.744447	-2.86346	-4.46187	2.964839	2.42245	-5.96238	2.752546	p07979	Ezrin	6	-0.6031		Syndecan-2-mediated signaling events; FAS (CD95) signaling pathway; RhoA signaling pathway; LKB1 signaling events;	
EZR seq: 24	-10.7998	-0.90769	-1.69329	-2.61111	0.645651	1.109874	1.021565	0.295109	p09535	Ezrin	6	-1.61746		Syndecan-2-mediated signaling events; FAS (CD95) signaling pathway; RhoA signaling pathway; LKB1 signaling events;	
EXPH5 seq:	-0.6483	-2.10915	-9.72194	-2.82865	-0.59279	-0.44932	0.162784	-0.07922	p16499	Exophilin-5	6	-2.03332			
ETFB seq: 9	-4.15351	1.754927	-8.84316	-1.29137	0	0	0	0	p11716	Electron tra	6	-1.56664			
ERBB2 seq:	-3.98568	-11.5802	-0.91835	-11.4164	0	0	0	0	p03613	Receptor ty	6	-3.48758	ERKinh1 (EI	ErbB recept	GO:0070372; P:regulation of ERK1 and ERK2 cascade; GO:0014065; P:phosphatidylinositol 3-kinase signaling; GO:0023014; P:signal transduction by phosphorylation; GO:0007169; P:transmembrane receptor protein tyrosine kinase signaling pathway; GO:0048015; P:phosphatidylinositol-mediate [illegible]
EPB41 seq:	-5.86722	-5.71669	-5.72843	-5.55284	-0.50688	-0.45612	-0.08763	-0.45163	p17230	Protein 4.1	6	-3.04593		Syndecan-2	GO:0043408; P:regulation of MAPK cascade; GO:0060337; P:type I interferon signaling pathway; GO:0060333; P:interferon-gamma-mediated signaling pathway; GO:0008543; P:fibroblast growth factor receptor signaling pathway; GO:0019221; P:cytokine-mediated signaling pathway; GO:0007264; [illegible]
ENOX1 seq	-7.16099	-0.42084	-0.62852	-6.84661	1.807952	1.015932	-0.01457	1.656648	p09666	Ecto-NOX d	6	-1.32387			
ENO1 seq: 6	-7.59838	-4.57868	-7.45959	-4.71629	2.862548	4.165798	3.762185	4.434066	p19255	Alpha-enola	6	-1.14104		Notch signaling pathway; HIF-1-alpha transcription factor network; Validated targets of C-MYC transcriptional activation;	
ENO1 seq: 2	1.569186	2.679642	-1.12669	-9.62198	-3.54954	-1.21313	-0.81238	-0.49893	p05900	Alpha-enola	6	-1.57173		Notch signaling pathway; HIF-1-alpha transcription factor network; Validated targets of C-MYC transcriptional activation;	
EML6 seq: 1	-1.34697	0.962893	-3.41132	-4.62193	2.174035	1.827358	0.825763	1.800904	p13513	Echinoderm	6	-0.22366			
EML5 seq: 1	-1.34697	0.962893	-3.41132	-4.62193	2.174035	1.827358	0.825763	1.800904	p10329	Echinoderm	6	-0.22366			
EIF5A2 pM4	-1.74381	0.278487	-2.32163	-4.1291	1.426555	1.238266	0.14361	0.897217	p13557	Eukaryotic t	6	-0.5263			
EIF4H seq: 2	-10.183	1.298767	-2.69088	-2.89919	2.202353	-7.20501	-0.33996	1.861036	p10978	Eukaryotic t	6	-2.24448			
EIF4H seq: 1	-4.0703	-4.97697	-2.21705	-3.89183	3.906917	1.488878	2.914208	2.708263	p19100	Eukaryotic t	6	-0.51724			
EIF4B seq: 1	-8.86651	-8.71598	-8.72772	-4.71014	2.470666	-6.68355	1.171223	0.494581	p15689	Eukaryotic t	6	-4.19593		mTOR signa	GO:0008286; P:insulin receptor signaling pathway;
EHBP1L1 p	-7.94985	-1.2758	-1.57166	-1.56869	0.296555	-0.26289	-0.07566	0.261293	p06833	EH domain-	6	-1.51834			
EFTUD2 seq	-4.8716	2.513154	-0.98921	-4.55722	0	0	0	0	p00420	116 kDa U5	6	-0.98811			
EEF2 seq: 7	-0.17756	2.030981	-1.51227	-9.19657	1.91565	2.272961	1.21165	2.876854	p15922	Elongation f	6	-0.07229		mTOR signaling pathway;	
EEF2 seq: 4	-1.88225	2.203037	-7.25189	-7.0763	6.469232	5.567824	0	7.152725	p13959	Elongation f	6	0.647796		mTOR signaling pathway;	
EEF1A2 seq	-0.47998	1.565111	-0.57648	-9.9143	-6.52004	0.540721	1.328157	3.212922	p05780	Elongation f	6	-1.35549			
DUT seq: 2	-2.04542	-1.27175	-3.00189	-2.54076	1.186385	0.85142	0.600196	0.312986	p14483	Deoxyuridin	6	-0.73861			
DSP seq: 26	-0.09099	-9.63319	-9.64493	-9.46934	4.381568	2.147539	1.134267	2.244097	p03714	Desmoplaki	6	-2.36637		Posttranslational regulation of adherens junction stability and dissassembly;	
DPYSL2 seq	-6.60354	-6.453	-6.46475	-3.34262	-2.17306	-1.3355	-1.85341	-1.12423	p02162	Dihydropyr	6	-3.66876	DYRK2; CDK5;		GO:0007165; P:signal transduction;
DPP3 seq: 1	-8.50959	2.126918	1.520619	-8.19521	0	0	0	0	p12202	Dipeptidyl p	6	-1.63216			
DNAJC8 sec	-5.7271	2.497319	-1.91416	-5.41272	3.653991	3.182955	1.573941	2.899465	p20298	DnaJ homo	6	0.094211			
DLD seq: 1	-7.00161	1.795459	-6.86281	-2.0679	3.598599	3.249749	1.597186	2.891789	p15903	Dihydrolipo	6	-0.34994			
DDX17 pS5	-1.54819	-0.31826	-6.82561	-2.04556	-0.40993	0.591254	-2.82621	-0.40999	p20000	Probable AT	6	-1.72406		Validated nuclear estrogen receptor alpha network;	
DDX1 seq: 1	1.041224	-2.00383	-1.5165	-6.11908	0	0	6.532033	5.749703	p17459	ATP-depend	6	-1.07477			
DCAF6 seq:	-2.43638	-10.7579	-2.4911	-6.23629	0	0	0	0	p18306	DDB1- and	6	-1.205			
DBN1 seq:	0.114249	3.130957	-1.68445	-6.85701	0	0	0	0	p01822	Drebrin	6	-0.66203			
DBI seq: 54	-1.55627	2.499168	-8.05561	-1.34411	0	0	0	1.076631	p20343	Acyl-CoA-bi	6	-0.92252			
DAPP1 seq	-0.97055	1.775415	-7.02685	-2.44607	-0.03741	-0.62783	0.365788	0.285925	p04060	Dual adapte	6	-1.0852		Class I PI3K	GO:0007165; P:signal transduction;
CTTN pS26	-0.35303	-0.10328	-2.05924	-10.2947	-0.56245	-0.89187	-2.22661	-1.54544	p10980	Src substrat	6	-2.25458		Syndecan-3-mediated signaling events; N-cadherin signaling events; E-cadherin signaling in the nascent adherens junction; FGF signaling pathway; PDGFR-beta signaling pathway;	
CTSD seq: 3	-1.89887	1.053398	-7.96209	-7.7865	-0.75082	0.454959	-7.05797	0.769315	p20475	Cathepsin D	6	-2.89732		LKB1 signaling events; Ceramide signaling pathway; Validated nuclear estrogen receptor alpha network; Direct p53 effectors;	
CTSD seq: 1	-1.14422	1.453713	-2.71948	-9.13355	4.845436	2.750151	0	4.303716	p08135	Cathepsin D	6	0.044471		LKB1 signaling events; Ceramide signaling pathway; Validated nuclear estrogen receptor alpha network; Direct p53 effectors;	
CTPS1 seq:	-0.22235	-2.43407	0.079261	-8.75729	0.110079	0.158965	0.310875	0.393409	p18139	CTP syntha	6	-1.29514			
CTBP2 seq:	0.697349	2.285536	-7.85039	-0.12171	1.669957	2.391783	-5.05062	-1.30203	p00922	C-terminal-	6	-0.91002			
CSTF2T seq	-12.6996	-0.88718	-0.45383	-0.47559	7.905954	0	0	0	p08647	Cleavage sti	6	-0.82628			
CORO1C se	-5.97741	1.635839	-5.83862	-0.58125	0.467735	0.41472	0.712151	0.641966	p09699	Coronin-1C	6	-1.06561			GO:0007165; P:signal transduction;
CORO1B se	0.25067	-0.00392	-11.2356	0.606189	7.900626	0	0	6.320096	p01524	Coronin-1B	6	0.47976			
COPB2 seq	-2.25148	0.004639	-2.66316	-2.77744	1.00366	0.515209	-0.20838	0.485852	p14649	Coatomer s	6	-0.73639			
CMIP pS37	-2.69231	-0.50326	-10.3839	-3.78704	-1.965	-1.03654	-2.52177	-1.75488	p11385	C-Maf-indu	6	-3.08059			
CMBL seq:	-7.36094	2.045487	-7.22214	-7.04655	0	0	0	0	p17599	Carboxyme	6	-2.44802			
CLTC seq: 1	1.681288	1.868461	-6.32758	-6.15199	0	0	0	0	p09421	Clathrin hea	6	-1.11623		FAS (CD95) signaling pathway; Arf6 trafficking events;	
CLSPN pS8	-0.21654	-6.5353	-6.54704	-6.37145	-0.26418	-1.01743	-0.38972	-0.17949	p10811	Claspin	6	-2.69014		ATR signalir	GO:0032147; P:activation of protein kinase activity;
CLNS1A sec	-6.6971	-6.54656	-6.5583	-6.38271	-0.8928	-2.5547	-0.41227	0.354691	p18058	Methylosor	6	-3.71122			
CLIP1 pS13	0.186504	2.607155	-7.81763	-7.64204	1.440982	1.098161	1.897006	1.843412	p01615	CAP-Gly do	6	-0.79831	PLK1; CK2A1;	Lissencephaly gene (LIS1) in neuronal migration and development; mTOR signaling pathway;	
CIAPIN1 se	-2.93136	0.905361	-1.33541	-8.16271	2.304171	-5.94341	0.710258	2.432988	p00760	Anamorsin	6	-1.50251			
CHPF seq: 5	-8.97849	1.510774	-2.07012	-2.96852	1.51415	0.549172	-1.57002	1.424176	p11241	Chondroitir	6	-1.32361			
CHMP5 sec	-1.87997	0.208989	-8.80144	-8.62585	2.514466	2.686415	0.164907	3.366139	p12098	Charged mu	6	-1.29579			
CHFR seq: 1	-10.2296	-0.47806	-10.0908	-0.19017	-1.14893	-0.3675	-0.71321	-0.45697	p00994	E3 ubiquitir	6	-2.9594			
CHAMP1 se	-1.95997	-9.2377	-9.24944	-0.07352	0	0	6.699825	6.523673	p15403	Chromosor	6	-0.91214			
CFL1 seq: 3	-0.30881	-3.30269	-1.8243	-9.40809	0.604495	2.108076	0.759724	1.543667	p19228	Cofilin-1	6	-1.22849		RhoA signal	GO:0007266; P:Rho protein signal transduction; GO:0038096; P:Fc-gamma receptor signaling pathway involved in phagocytosis;
CEP170B p	-8.02312	-7.87259	-1.04854	-7.70874	-0.31705	-1.05376	-0.37697	-0.39234	p11285	Centrosoma	6	-3.34914	Up with IGF - down with ERKinh1 (ERK inhbitor Calbiochem); Up with IGF - down with EGFRinh1 (PD-168393); Up with IGF - down with CAMKinh2 (KN-62); Up with IGF - down with CAMKinh1 (KN-93); Up with IGF - down with AKTinh2 (MK-2206); Up with IGF - down with AKTinh1 (Akt Inhibitor VIII); Akt-CAMK2; ERKinh1 [illegible]		
CEP170 pS	-0.27494	0.777468	-0.06679	-9.00129	-0.16677	-0.06654	0.025418	-0.01491	p11805	Centrosoma	6	-1.09854			
CEP131 sec	-1.13008	-2.26435	-7.4989	-7.32331	-0.3373	0.004844	0.101956	-0.16264	p05665	Centrosoma	6	-2.32622			
CDT1 pS31	-2.30134	-9.19493	-9.20668	-9.03109	-0.62726	0.16257	-0.71275	-0.51932	p20758	DNA replica	6	-3.92885			
CDR2 seq: 3	-8.65585	2.402433	-8.51705	0.962348	-0.25766	0.335739	-0.14578	-0.2275	p18521	Cerebellar d	6	-1.76291			
CDC42BPB	-6.98257	0.445971	-6.84377	-6.66819	-1.32607	-1.23125	-0.49569	-0.97966	p09499	Serine/threo	6	-3.01015			GO:0035556; P:intracellular signal transduction; GO:0007165; P:signal transduction;
CCT8 seq: 1	-9.65285	-0.02074	-9.51406	-1.64364	-0.24814	0.852286	0.681329	0.964317	p07670	T-complex p	6	-2.32269			
CCT7 seq: 7	-1.49891	4.954056	-4.93153	-4.75594	3.812192	3.12626	0	3.901871	p02513	T-complex p	6	0.576			
CCT5 seq: 9	-8.97849	1.510774	-2.07012	-2.96852	1.51415	0.549172	-1.57002	1.424176	p11218	T-complex p	6	-1.32361			
CCT4 seq: 9	-8.92286	1.647693	-2.01449	-2.82677	1.651503	0.825656	-2.3386	1.521532	p09992	T-complex p	6	-1.30704			
CCT3 seq: 3	1.001956	1.634872	-9.70677	0.731927	3.968713	-5.1581	2.564483	4.259767	p09423	T-complex p	6	-0.08789			
CCT3 seq: 3	-8.24156	-8.09103	-2.97275	-3.12959	3.303186	0	0	0	p20937	T-complex p	6	-2.39147			
CCT2 seq: 1	-1.06463	-1.96634	-1.71491	-5.46741	1.263203	1.815824	-2.7611	3.491513	p12143	T-complex p	6	-0.80048			
CCDC86 pS	-0.28346	0.245306	-8.57034	0.716756	-0.19737	-0.12113	-0.05743	-0.14656	p10950	Coiled-coil	6	-1.05178			
CAPZA2 sec	-8.85293	-0.2934	-1.38432	-3.78132	1.678457	1.682963	0.218503	1.852246	p02067	F-actin-cap	6	-1.10998			
CAPZA1 sec	-1.93907	2.155701	-7.40158	-7.22599	0	0	0	0	p16420	F-actin-cap	6	-1.80137			
CAP1 seq: 3	-1.53039	0.813242	-3.63165	-7.85927	-0.98348	-1.395	-8.05735	-1.34696	p19004	Adenylyl cy	6	-2.99886		Arf6 signali	GO:0007165; P:signal transduction;
CAMSAP1 s	-0.42645	0.727913	-0.57232	-7.9639	-0.68843	0.039144	-0.3202	-0.83632	p13675	Calmodulin	6	-1.25507			
CALB2 seq:	0.32126	-4.88082	-4.89257	-4.71698	0.600441	0.697474	0.903463	1.064759	p17503	Calretinin	6	-1.36287			
C2CD2L sec	-1.00619	0.681825	-1.58835	-8.68889	0	0	0	0	p08473	C2 domain-	6	-1.3252			
C15orf52 s	0.089115	-3.7398	-0.69143	-11.3922	-0.43724	-0.46725	-0.49893	-0.43603	p00684	Uncharacte	6	-2.19672			
BUB3 seq: 1	-1.22389	-0.15385	-9.02461	-3.31098	1.030092	1.064713	0.71151	1.541986	p00518	Mitotic che	6	-1.17063		p73 transcription factor network;	
BRD4 seq: 4	-0.12957	3.516287	-7.62559	-1.45665	0	0	0	0	p12274	Bromodom	6	-0.71194			GO:0043123; P:positive regulation of I-kappaB kinase/NF-kappaB signaling;
BOLA1 pS8	-10.5884	-2.96714	-0.80363	-2.1367	0	0	0	0	p16796	BolA-like pr	6	-2.06199			
BLVRB pM1	0.329775	-0.00949	-0.61917	-9.63848	0	0	0	0	p00037	Flavin reduc	6	-1.24217			
BCLAF1 seq	-1.68637	-0.83597	-1.16269	-13.123	2.901763	3.963622	-0.39211	3.485358	p10248	Bcl-2-assoc	6	-0.85617			

[illegible]

ZNRF2 pS14	0.169534	0.32567	-0.50085	-1.26553	-1.12894	-0.62927	-0.99695	-0.63382	p10417	E3 ubiquitir	4	-0.58252		
ZNRF1 pS1	0.160066	-0.60084	0.058267	0.257274	-0.1817	-0.66866	0.222533	0.122039	p19593	E3 ubiquitir	4	-0.07888		PI3Kinh2 (PI-103);
ZNF787 pT	-0.18953	-0.02429	0.181868	0.175175	-0.21347	-0.33609	-0.38498	-0.62191	p14202	Zinc finger p	4	-0.17665		
ZNF768 sec	0.430693	0.729819	0.720272	0.631616	-2.13384	-1.09082	-0.19705	-1.04324	p02257	Zinc finger p	4	-0.24407		
ZNF768 pS	0.331793	0.087138	0.30057	0.351378	-0.13527	-0.45157	-0.39466	-0.69243	p00404	Zinc finger p	4	-0.07538		
ZNF687 sec	0.040792	-0.03782	0.095964	0.202196	-0.56022	-0.56536	-0.54517	-0.73902	p13362	Zinc finger p	4	-0.26358		
ZNF687 pS	-0.44355	0.74143	-2.8748	-0.29802	0.666386	1.385603	-3.97402	0.011302	p03621	Zinc finger p	4	-0.59821		Up with IGF - down with PI3Kinh2 (PI-103); Up with EGF - down with PI3Kinh2 (PI-103); Up with IGF - down with P
ZNF652 sec	0.040944	-0.35398	-0.07365	-0.02679	-1.25144	-0.97984	-0.85217	-1.65704	p07652	Zinc finger p	4	-0.64425		
ZNF646 sec	-1.30943	-1.29316	-1.48079	-1.59307	0.982291	0.24058	0.483566	0.003573	p04075	Zinc finger p	4	-0.4958		
ZNF638 sec	0.083542	-0.18324	0.336025	0.160616	-0.1917	-0.42886	-0.2165	-0.85867	p19236	Zinc finger p	4	-0.16235		
ZNF638 pS	-0.23941	-0.93577	0.389778	0.010579	-0.2305	-0.54432	-0.43162	-0.81748	p07831	Zinc finger p	4	-0.34984		
ZNF609 sec	-2.57092	-1.0444	-4.23966	-1.87052	-2.1817	-1.62638	-3.9767	-2.12202	p02464	Zinc finger p	4	-2.45404		
ZNF609 pS	-1.07545	-0.43686	-1.75654	-1.21794	-1.17075	-1.15643	-2.42473	-1.55104	p02461	Zinc finger p	4	-1.34872		
ZNF609 pS	-0.6728	-0.02951	-1.17314	-0.6204	-1.86465	-0.99615	-2.34467	-1.42345	p02463	Zinc finger p	4	-1.1406		
ZNF609 pS	-0.75322	-0.14709	-0.18581	-0.10068	-0.29447	-0.36561	-0.12309	0.000124	p19911	Zinc finger p	4	-0.24623		
ZNF592 pS	-0.17824	-0.1701	-0.45586	-0.6491	-0.47731	-0.2985	-0.23159	-0.67283	p01301	Zinc finger p	4	-0.39169		
ZNF516 sec	0.024837	-0.25842	0.138839	-0.06929	-1.06061	-1.74501	-0.59808	-0.33131	p06196	Zinc finger p	4	-0.48738		
ZNF428 pS	-0.40278	-0.36009	-0.28076	-0.39694	-0.58999	-0.48891	-0.59996	-0.67978	p04562	Zinc finger p	4	-0.4749		
ZNF326 sec	-0.64619	-0.93836	-2.71516	-0.99429	0.18211	-3.11043	-0.51731	-0.97124	p09150	DBIRD com	4	-1.21386		
ZNF318 sec	0.073318	-0.59329	0.2093	0.029679	-5.57723	0.281579	2.544682	0.305046	p06063	Zinc finger p	4	-0.34086		
ZNF318 sec	0.80211	-0.31667	0.765322	0.733528	-0.99188	0.767004	-2.26942	0.003411	p02650	Zinc finger p	4	-0.06332		
ZNF318 pS	0.389344	-0.14004	0.16302	0.433814	-0.18364	-0.28592	-0.11737	-0.4175	p13849	Zinc finger p	4	-0.01979		
ZNF318 pS	0.061829	-0.11906	0.350666	0.314469	-0.72837	-0.47793	-0.53477	-0.96485	p13759	Zinc finger p	4	-0.26225		ERKinh1 (ERK inhbitor Calbiochem); EGFRinh1 (PD-168393);
ZNF276 sec	0.085681	0.32578	0.09782	0.168025	-0.00862	-0.1848	-0.20453	-0.40847	p20207	Zinc finger p	4	-0.01614		AKTinh2 (MK-2206); AKTinh1 (Akt Inhibitor VIII);
ZNF217 sec	0.327646	0.060592	-0.11409	-0.00349	-0.97979	-0.90121	-1.02239	-1.219	p15803	Zinc finger p	4	-0.48147		p70S6Kinh2 (DG2); p70S6Kinh1 (PF-4708671); ERKinh1 (ERK inhbitor Calbiochem); EGFRinh1 (PD-168393); AKT
ZNF217 sec	0.262627	-0.04433	0.145937	0.038349	-3.76091	-0.33507	-1.50772	-2.46161	p02675	Zinc finger p	4	-0.95784		
ZHX2 pS71	-0.33014	-0.02974	-0.99572	-0.13363	-1.25041	-0.63666	-1.07446	-1.96521	p13372	Zinc fingers	4	-0.802		
ZFX seq: 35	-0.11831	-0.44524	-0.14969	-0.42916	-1.74315	-0.57085	-0.13401	-0.89159	p02861	Zinc finger X	4	-0.56025		
ZFPM2 pS1	0.591797	0.246394	0.431172	0.535757	-0.31213	-1.4199	0.249157	-2.59166	p18253	Zinc finger p	4	-0.28368		
ZFPM1 pS9	-0.01636	-0.28479	0.125253	0.215163	0.057321	0.025791	-0.18038	0.03612	p02434	Zinc finger p	4	-0.00273		Signaling events mediated by HDAC Class I; C-MYB transcription factor network;
ZFP91 seq:	0.16334	-0.08533	-0.08605	-0.10855	-0.36589	-0.56475	-0.37368	-0.62542	p06587	E3 ubiquitir	4	-0.25579		
ZFP36L1 pS	-0.34651	0.253048	-0.97614	-0.64441	-0.22189	-0.20177	-0.42611	-0.46983	p07288	Zinc finger p	4	-0.3792	MAPKAPK2;	Validated targets of C-MYC transcriptional repression;
ZFP36L1 pS	0.04728	0.050674	-0.42394	0.056311	-0.36834	-0.37729	-0.74091	-0.29128	p11118	Zinc finger p	4	-0.25594		Up with IGF Validated targets of C-MYC transcriptional repression;
ZFHX3 seq:	0.133271	-0.32996	-0.03118	0.08822	-0.58327	-0.02583	-0.43418	-0.56058	p20195	Zinc finger h	4	-0.21794		
ZFAND3 pS	-0.38708	-0.51742	-0.1642	-0.12398	-0.49501	-0.1469	-0.01347	-0.49352	p06152	AN1-type zi	4	-0.2927		
ZDHHC5 se	-0.52036	-12.9653	0.58534	0.1198	-0.18411	-0.66681	-2.4351	-0.09523	p02654	Palmitoyltra	4	-2.02023		
ZCCHC8 pT	-0.20778	-1.14639	0.322674	0.321088	0.169349	-0.2143	-0.57355	-0.57622	p15080	Zinc finger C	4	-0.23814		
ZCCHC8 pS	0.146198	0.390113	0.077919	0.258809	-0.38612	0.019371	-0.68912	-0.3492	p14203	Zinc finger C	4	-0.0665		
ZC3HC1 sec	-0.08164	-0.24103	0.45415	0.114359	-0.53378	-0.86898	0.390828	-0.43196	p03203	Nuclear-inte	4	-0.14976		
ZC3HC1 pS	-1.16443	-2.44994	1.306827	0.114969	-1.07642	-2.49036	0.620366	-0.86819	p03204	Nuclear-inte	4	-0.7509		
ZC3HAV1 se	0.042517	0.16793	-0.76394	-0.00164	0.114327	0.132862	-0.83594	-0.08032	p06127	Zinc finger C	4	-0.15303		GO:0043123; P:positive regulation of I-kappaB kinase/NF-kappaB signaling;
ZC3HAV1 p	0.149491	-0.22612	0.178881	0.208671	-0.27813	-0.76841	-0.24172	-0.34634	p18350	Zinc finger C	4	-0.16546		GO:0043123; P:positive regulation of I-kappaB kinase/NF-kappaB signaling;
ZC3H4 seq:	0.464733	0.15056	0.157069	0.215296	-0.52974	-0.238	-0.40202	-0.43059	p20202	Zinc finger C	4	-0.07659		
ZC3H4 seq:	0.01385	-0.18847	0.056427	0.128743	-0.06988	0.080429	-0.0475	0.026777	p16890	Zinc finger C	4	4.75E-05	Src;	
ZC3H4 pS1	-0.54853	-0.09103	-0.53241	-0.62198	-1.13022	-0.49727	-0.91	-0.96382	p02790	Zinc finger C	4	-0.66191		Up with IGF - down with mTORinh2 (Torin-1); mTORinh2 (Torin-1);
ZC3H4 pS1	-0.32201	0.262312	-0.38432	-0.36701	-0.91965	-0.27585	-0.71947	-0.76264	p02789	Zinc finger C	4	-0.43608		Up with IGF - down with mTORinh2 (Torin-1); mTORinh2 (Torin-1);
ZC3H4 pS1	-0.05166	0.316606	-0.13687	-0.25119	-0.68818	-0.17599	-0.52879	-0.64178	p02793	Zinc finger C	4	-0.26973		Up with IGF - down with mTORinh2 (Torin-1); mTORinh2 (Torin-1);
ZC3H18 sec	-0.03594	-0.30358	0.30449	0.204804	-0.01909	-0.08282	-0.08518	-0.18623	p03746	Zinc finger C	4	-0.02544	CDK2;	
ZC3H18 pS	-0.33137	-0.74515	-0.98097	-0.1534	-0.53552	-0.6869	-0.88502	-0.98176	p13043	Zinc finger C	4	-0.66251		
ZC3H18 pS	0.043535	0.412955	-0.53949	0.105123	-0.0961	-0.00602	-0.60838	-0.19031	p11662	Zinc finger C	4	-0.10984		
ZC3H14 pS	0.15348	0.499148	-1.66724	-0.08472	0.423942	1.063365	-1.05631	0.419401	p06858	Zinc finger C	4	-0.03112		
ZC3H13 sec	0.09477	-0.33148	-0.04819	-0.00101	-0.4368	-0.2059	-0.16044	-0.34815	p12473	Zinc finger C	4	-0.17965		
ZC3H13 sec	-0.02438	-0.57299	0.159803	-0.04573	0.063874	-0.05494	0.091908	-0.03615	p05403	Zinc finger C	4	-0.05232		
ZC3H13 pT	0.090685	-0.09547	0.148841	0.32743	-0.45423	-0.32264	-0.55354	-0.4879	p02516	Zinc finger C	4	-0.16835		
ZC3H13 pS	0.10044	-0.2537	-0.05951	-0.00902	-0.66564	-0.26338	-0.34782	-0.37952	p12474	Zinc finger C	4	-0.23477		
ZC3H13 pS	-0.54024	-0.15517	-0.26764	0.04182	-0.36316	-0.4028	-0.53095	-0.78242	p05401	Zinc finger C	4	-0.37507		
ZC3H11A p	0.155724	-0.19651	0.439458	0.274834	-0.19957	-0.39926	-0.37932	-0.62889	p10594	Zinc finger C	4	-0.11669		
ZC2HC1A se	-0.42593	-1.03338	-0.41002	-0.47642	-0.5752	-0.5356	-0.38108	-0.63897	p10927	Zinc finger C	4	-0.55957		
ZBTB7A seq	-0.13027	-0.23334	0.065075	-0.09382	-0.53496	-0.45675	-0.62563	-0.91423	p16076	Zinc finger a	4	-0.36549		
ZBTB7A seq	-0.13642	-0.05961	0.307813	0.193555	-0.08407	-0.22078	-0.09756	-0.60857	p16077	Zinc finger a	4	-0.0882		
ZBTB7A seq	-0.45949	-0.65698	-0.73003	-0.396	-0.92918	-0.75588	-0.84954	-1.1487	p00903	Zinc finger a	4	-0.74073		
ZBTB7A seq	-0.16624	-0.42538	-0.39351	-0.21827	-0.61735	-0.75315	-0.78014	-1.14851	p00902	Zinc finger a	4	-0.56282		
ZBTB7A seq	-0.03902	-0.15221	0.139171	-0.00201	-0.45695	-0.46231	-0.41142	-0.99998	p00904	Zinc finger a	4	-0.29809		
ZBTB21 seq	0.081277	0.508025	-0.0704	-0.01398	-0.84983	-0.31668	-0.40476	-0.68332	p14104	Zinc finger a	4	-0.21871		
ZBTB21 seq	-0.00624	0.151284	-0.00129	-0.00946	-0.3531	-0.25462	-0.50852	-0.7236	p14103	Zinc finger a	4	-0.21319		
ZAK pS637	-0.1722	-0.55171	-0.14768	-0.09614	0.043507	0.157109	-0.00635	0.0023	p03655	Mitogen-ac	4	-0.0964		Signaling m GO:0000186; P:activation of MAPKK activity; GO:0035556; P:intracellular signal transduc
ZAK pS633	-0.02729	-0.83053	0.144109	-0.03315	-0.0782	-0.58049	-0.15232	-0.01402	p00152	Mitogen-ac	4	-0.19649		Signaling m GO:0000186; P:activation of MAPKK activity; GO:0035556; P:intracellular signal transduc
YWHAZ seq	-0.52059	-0.40604	-0.17926	-0.10526	0.383057	0.220627	0.27618	0.137393	p03227	14-3-3 prot	4	-0.02424		p38 signalin GO:0007165; P:signal transduction;
YWHAQ sec	-0.51149	0.208801	-0.68091	-1.02516	-0.871	-0.0637	-0.51886	-0.52333	p07805	14-3-3 prot	4	-0.49821		p38 signalin GO:0007264; P:small GTPase mediated signal transduction;
YWHAG sec	-2.59585	-2.44181	-2.45237	-2.28475	-2.94417	0.398359	-2.22057	0.433938	p13101	14-3-3 prot	4	-1.7634		p38 signalin GO:0009966; P:regulation of signal transduction; GO:0006469; P:negative regulation of p
YWHAG sec	-0.41597	-0.5771	-0.32224	-0.65617	0.384424	0.250535	0.377726	0.143951	p08936	14-3-3 prot	4	-0.10185		p38 signalin GO:0009966; P:regulation of signal transduction; GO:0006469; P:negative regulation of p
YWHAE seq	-0.44752	0.399611	-0.86309	-1.04421	1.280964	0.91376	0.053584	0.120719	p18024	14-3-3 prot	4	0.051727		p38 signalin GO:0035329; P:hippo signaling; GO:0048011; P:neurotrophin TRK receptor signaling path
YTHDF1 sec	-0.64484	-0.45426	-0.30945	-0.30555	-0.31527	-0.13524	-0.36935	-0.44338	p09800	YTH domain	4	-0.37217		
YTHDF1 pS	-0.88886	-0.67526	-0.53377	-0.4276	-0.30725	-0.16341	-0.39423	-0.3054	p19851	YTH domain	4	-0.46197		AKTinh2 (MK-2206); AKTinh1 (Akt Inhibitor VIII);
YTHDC2 pS	-0.47879	-0.44067	-0.49478	-0.50073	-0.20748	0.145762	-0.27707	-0.24434	p15259	Probable AT	4	-0.31226		
YTHDC1 sec	-0.45687	-0.69128	-0.87327	-0.74717	-0.37056	-0.40522	-0.64821	-0.47967	p10640	YTH domain	4	-0.58403		
YEATS2 pS5	0.255911	-0.1019	0.311376	0.119542	-0.54792	-0.23043	-0.35207	-0.543	p00923	YEATS doma	4	-0.13606		
YBX1 pS20	-0.59395	0.086529	-0.84444	-0.76831	-0.51707	-0.65544	-0.78093	-0.70753	p06866	Nuclease-se	4	-0.59764		Up with IGF Signaling events mediated by PTP1B;
YAP1 seq: 1	-0.13722	-0.97438	-0.11962	-0.5447	-0.41278	-1.59453	-0.56695	-0.48317	p07847	Transcriptic	4	-0.60417	LATS1;	ErbB4 signa GO:0014065; P:phosphatidylinositol 3-kinase signaling; GO:0035329; P:hippo signaling;
YAP1 seq: 1	0.370239	-0.15595	0.092696	0.14442	-0.34898	-0.79039	-0.27939	-0.15878	p20503	Transcriptic	4	-0.14077		ErbB4 signa GO:0014065; P:phosphatidylinositol 3-kinase signaling; GO:0035329; P:hippo signaling;
YAP1 seq: 1	0	0	0	0	0.966802	-3.11985	0.764529	1.246295	p20506	Transcriptic	4	-0.01778		PKCinh1 (G ErbB4 signa GO:0014065; P:phosphatidylinositol 3-kinase signaling; GO:0035329; P:hippo signaling;
YAP1 seq: 1	-0.13057	-0.76889	-0.02398	-0.27598	-0.00383	-0.72629	-0.23761	-0.22484	p08807	Transcriptic	4	-0.299		ErbB4 signa GO:0014065; P:phosphatidylinositol 3-kinase signaling; GO:0035329; P:hippo signaling;
YAP1 seq: 1	-0.05495	-0.28096	-0.16529	-0.1276	-0.05305	-0.61943	-0.22703	-0.01625	p08810	Transcriptic	4	-0.19307		ErbB4 signa GO:0014065; P:phosphatidylinositol 3-kinase signaling; GO:0035329; P:hippo signaling;
YAP1 seq: 1	0.459279	0.048258	0.19647	0.341951	-0.28906	-1.10197	-0.5851	-0.18582	p08809	Transcriptic	4	-0.1395		ErbB4 signa GO:0014065; P:phosphatidylinositol 3-kinase signaling; GO:0035329; P:hippo signaling;
YAP1 seq: 1	-0.55468	-5.72925	-0.91443	-1.70589	-0.18426	-1.02024	-0.55125	-0.35173	p05561	Transcriptic	4	-1.37646		ErbB4 signa GO:0014065; P:phosphatidylinositol 3-kinase signaling; GO:0035329; P:hippo signaling;
YAP1 seq: 1	-0.18019	-1.15092	-0.50759	-0.87811	-0.38643	-1.30197	-0.71569	-0.1865	p05562	Transcriptic	4	-0.66343	LATS1;	ErbB4 signa GO:0014065; P:phosphatidylinositol 3-kinase signaling; GO:0035329; P:hippo signaling;
YAP1 pS13	0.550922	-1.70017	0.532278	0.368782	0.737796	-0.84289	0.365207	0.625052	p20505	Transcriptic	4	0.079622		PKCinh1 (G ErbB4 signa GO:0014065; P:phosphatidylinositol 3-kinase signaling; GO:0035329; P:hippo signaling;
YAP1 pS13	-0.17212	-0.09516	-0.0013	0.112823	0.125321	-0.73548	0.106652	0.23181	p20507	Transcriptic	4	-0.05343		PKCinh1 (G ErbB4 signa GO:0014065; P:phosphatidylinositol 3-kinase signaling; GO:0035329; P:hippo signaling;
XRN2 seq:	-0.55104	0.061649	-1.81313	-1.94558	0.776223	-0.78904	0.480565	0.705074	p09122	5'-3' exorib	4	-0.38441		
XRN1 pS16	-0.03702	-0.59353	-0.3639	-0.15481	-0.2079	-0.04884	-0.01082	-0.12197	p17923	5'-3' exorib	4	-0.19235		
WWTR1 sec	0	0	0	0	-0.96253	-0.56043	-0.80665	-0.95979	p05559	WW domain	4	-0.41118		Up with IGF - down with GO:0035329; P:hippo signaling; GO:0006469; P:negative regulation of protein kinase acti
WWC3 seq	-0.28814	-2.04785	-0.08834	-0.10552	0	0	0	0	p03834	Protein WW	4	-0.31623		GO:0007218; P:neuropeptide signaling pathway;
WWC3 seq	-0.21075	-1.81191	-0.07485	-0.12422	0	0	0	0	p03835	Protein WW	4	-0.27771		GO:0007218; P:neuropeptide signaling pathway;
WTAP seq:	-0.52589	-0.46425	-0.29018	-0.26973	-0.03424	-0.19122	-0.22689	-0.24252	p07933	Pre-mRNA-	4	-0.28061		
WRNIP1 se	-0.19249	-0.78844	-0.22342	-0.25355	-0.2278	-0.27167	-0.35236	-0.59208	p15280	ATPase WRI	4	-0.36272		
WRNIP1 pS	-0.04521	-0.19725	-0.63853	-0.36292	0.036142	0.36309	-2.2E-05	0.427671	p07139	ATPase WRI	4	-0.05213		
WNK2 pS1	-0.17919	-0.68057	-0.24034	-0.85099	-0.1804	-1.12713	-0.47836	-1.89701	p08821	Serine/threo	4	-0.70425		ERKinh2 (ERK inhibitor GO:0070373; P:negative regulation of ERK1 and ERK2 cascade; GO:0035556; P:intracellula
WNK1 seq:	0.848336	1.482449	0.734391	0.098943	-0.899	-1.497	-1.64714	-2.61328	p13290	Serine/threo	4	-0.43654		p70S6Kinh1 (PF-47086 GO:0035556; P:intracellular signal transduction;
WIZ pS113	0.246861	-0.12601	0.178755	0.021419	-0.55396	-0.52081	-0.21321	-0.75738	p04435	Protein Wiz	4	-0.21554		Up with EGF - down with PKCinh2 (Bisindolylmaleimide-1); Up with EGF - down with p70S6Kinh1 (PF-4708671); P
WIZ pS101	0.143647	0.044455	0.382005	0.112279	-0.26159	-0.43296	-0.41593	-0.69922	p09278	Protein Wiz	4	-0.14091		p70S6Kinh1 (PF-4708671);
WDR7 seq:	-0.37762	-0.64477	-0.28982	-0.41894	0.263124	-0.45008	-0.20631	-0.42947	p03644	WD repeat-	4	-0.31924		
WDR59 sec	-0.63357	-0.22271	-0.65852	-0.96849	0.839458	-0.71511	1.009879	1.413024	p17987	WD repeat-	4	0.007996		
WDR59 sec	-0.49604	-0.59166	-0.10779	-0.32433	-0.38812	-0.05645	-0.41357	-0.54197	p10292	WD repeat-	4	-0.36499		
WDR44 sec	-0.68792	-0.15388	-0.39955	-0.55778	-0.04955	0.297484	0.100515	0.056437	p15826	WD repeat-	4	-0.17428		
WDR44 pS	-0.81612	-0.30783	0.008998	-0.70768	-0.87428	0.518871	0.399472	-0.01634	p15827	WD repeat-	4	-0.22436		
WDR20 sec	-0.72932	-0.57432	-0.09843	-0.20553	-1.43462	-3.41028	-1.26584	-2.73263	p03935	WD repeat-	4	-1.30637		
WDR20 sec	0.228446	-0.03297	-0.2572	-0.16355	-0.67058	-1.19124	-0.60793	-0.59574	p04783	WD repeat-	4	-0.41135		
WDR20 sec	-0.03804	-0.27513	-0.30359	-0.17525	-0.38444	-1.04584	-0.50085	-0.39103	p04782	WD repeat-	4	-0.38927		
WDR13 sec	0.040869	0.155126	0.283832	-0.40787	-7.24398	0.245957	-1.11235	-0.94028	p17607	WD repeat-	4	-1.12234		
WDR1 seq:	-0.48565	-0.59345	-0.75249	-0.93568	-0.04015	0.549051	0.311139	-1.06373	p00446	WD repeat-	4	-0.37637		
WDR1 seq:	-0.58806	0.54466	-0.61139	-2.34966	-2.35851	-0.92317	-3.58201	-1.45763	p16833	WD repeat-	4	-1.41572		
WAPL pS77	-0.51757	-0.63631	-0.84343	-0.4726	-0.38025	-0.31665	-0.41276	-0.74392	p12779	Wings apar	4	-0.54044		Akt-CAMK2; CAMKinh2 (KN-62); CAMKinh1 (KN-93); AKTinh2 (MK-2206); AKTinh1 (Akt Inhibitor VIII);
VRK3 pS82	-1.02066	-0.54691	-3.54466	-1.34471	-0.11379	0.489272	0.345132	0.612829	p10760	Inactive seri	4	-0.64044		GO:0070373; P:negative regulation of ERK1 and ERK2 cascade;
VPS13C pS	-0.16666	-0.36693	-0.65525	-0.74407	-0.31833	0.159304	0.657643	-0.80004	p00329	Vacuolar pr	4	-0.27929		
VIM seq: 14	0	0	0	0	-0.01055	-0.10498	-0.21177	-0.14359	p10144	Vimentin	4	-0.05886		Aurora B signaling; Caspase cascade in apoptosis;
VIM pS73	0.314971	-0.01308	0.065083	0.380965	-0.05184	-0.48843	-0.6669	-0.45079	p10857	Vimentin	4	-0.11375	PKACA; AurB;	Aurora B signaling; Caspase cascade in apoptosis;
VIM pS55	-1.41903	-0.52646	-1.67418	-3.58578	0.084488	-0.09804	0.011933	-0.14624	p04925	Vimentin	4	-0.91916	CDK2; CDK1;	Aurora B signaling; Caspase cascade in apoptosis;
VGLL4 pS1	0.130548	0.381405	0.171411	0.091509	-0.53667	-0.31498	-0.40052	-0.57896	p09151	Transcriptic	4	-0.13203		
VDAC2 seq	-0.45178	-0.50353	-0.7621	-0.93435	0.472732	0.086341	0.834392	1.052814	p09344	Voltage-dep	4	-0.02569		
VDAC1 seq	-0.38687	-0.15138	-0.54677	-1.31774	-0.00758	0.607192	0.368073	-0.02808	p01059	Voltage-dep	4	-0.18289		
VCPIP1 seq	-0.41409	-0.53593	-0.25826	-0.36288	0.372967	0.808702	0.341998	0.562838	p17112	Deubiquitir	4	0.064418		
VCP seq: 71	-1.05556	0.856774	-0.59001	-2.30953	-8.49306	-0.17096	0.391437	-0.86348	p07791	Transitional	4	-1.5293		
VCP seq: 67	0.232297	0.189479	0.605952	0.465866	0.422898	-1.18332	-0.48272	-0.53576	p09853	Transitional	4	-0.03566		
VCP seq: 60	-1.58142	-0.69708	-1.69982	-1.74404	-6.37546	-0.32896	0.268058	2.047618	p01365	Transitional	4	-1.26389		
VCP seq: 48	-0.88577	0.060733	-0.4569	-0.67868	0.016396	0.350822	-2.55943	1.057619	p17418	Transitional	4	-0.3869		
VCP seq: 36	-0.99781	1.596917	-3.85876	-0.41176	0	0	0	0	p16809	Transitional	4	-0.45893		
VCP pM678	-3.14243	-0.56135	-1.08805	-0.7443	-0.41241	-0.20857	-0.18896	-0.45219	p09852	Transitional	4	-0.84978		
VCL seq: 28	0.078727	-0.17928	-0.75343	-0.53232	-0.27844	0.657949	-0.24542	-0.85355	p14954	Vinculin	4	-0.26322		Stabilization and expansion of the E-cadherin adherens junction; RhoA signaling pathway; Signaling eve
VCL pS290	-0.1268	-0.27253	-0.50673	-0.19054	-0.1384	-0.12651	-0.1253	-0.29534	p14953	Vinculin	4	-0.22277		Stabilization and expansion of the E-cadherin adherens junction; RhoA signaling pathway; Signaling eve
VASP seq: 3	-1.00975	-1.06157	-0.59911	-1.60272	0	0	0	0	p03619	Vasodilator	4	-0.53414		E-cadherin GO:0050852; P:T cell receptor signaling pathway;
VASP pS23	0.843889	-0.74287	-0.36989	0.230217	0.287054	-0.21294	-0.23242	0.139508	p12398	Vasodilator	4	-0.00718	PKG1; PKACA;	E-cadherin GO:0050852; P:T cell receptor signaling pathway;
USP9X pS1	0.009593	0.020323	-0.00939	0.170473	0.99758	-0.54451	-0.46286	-0.27842	p09609	Probable ub	4	-0.01215		GO:0030509; P:BMP signaling pathway; GO:0007179; P:transforming growth factor beta
USP6NL pS	-0.2985	-0.28665	-0.38226	-0.45116	-0.30176	-0.46328	-0.57159	-0.99999	p06928	USP6 N-terr	4	-0.4694		ErbB1 downstream signaling; PDGFR-beta signaling pathway;
USP6NL pS	-0.34781	-0.66179	-0.0827	-0.09265	0	0	0	0	p03339	USP6 N-terr	4	-0.14812		ErbB1 downstream signaling; PDGFR-beta signaling pathway;
USP47 pS1	-0.50981	-0.89823	-0.16501	-0.50527	0.520579	0.66775	0.750498	0.397261	p00660	Ubiquitin ca	4	0.032222		
USP43 seq:	0.108445	-2.75132	-0.27236	-0.15149	-1.17997	-1.39256	-1.69367	-1.54426	p03439	Ubiquitin ca	4	-1.10965		
USP43 seq:	-0.65063	-6.63714	0.06672	0.376696	0.411573	-0.39005	0.593295	0.647906	p18076	Ubiquitin ca	4	-0.6977		
USP39 pS8	-0.51955	-1.18556	-0.48081	-1.16843	0.183893	-0.02595	0.128086	-0.19544	p17016	U4/U6.U5 t	4	-0.40797		
USP36 seq:	-0.38946	-0.74791	-0.411	-0.37444	-0.4472	-0.50929	-0.16101	-0.69763	p15158	Ubiquitin ca	4	-0.46724		
USP24 pS2	0.150592	-1.69991	-0.72429	-0.65533	0.10558	0.087179	0.161679	0.207586	p00876	Ubiquitin ca	4	-0.29586		
USP18 pT8	0.17463	-1.98768	-0.99308	0.156121	-0.74976	-0.59598	-0.85519	-6.20268	p07237	Ubl carboxy	4	-1.3817		GO:0060338; P:regulation of type I interferon-mediated signaling pathway; GO:0060337;
USP15 seq:	-2.43983	0.197726	-0.277	-0.39333	-0.15958	-0.09163	-0.20664	-0.82817	p16268	Ubiquitin ca	4	-0.52481		GO:0030509; P:BMP signaling pathway; GO:0030512; P:negative regulation of transformi
USP15 seq:	-1.25358	0.168051	-0.29951	-1.20425	-0.67839	-0.75909	-0.38959	-0.3499	p13680	Ubiquitin ca	4	-0.59578		GO:0030509; P:BMP signaling pathway; GO:0030512; P:negative regulation of transformi
USP10 seq:	-1.30704	-0.57081	-1.23018	-1.06194	-0.77573	-0.4633	-0.62018	-0.64142	p02309	Ubiquitin ca	4	-0.83382		GO:0030330; P:DNA damage response, signal transduction by p53 class mediator;
USP1 seq: 3	0.193026	-0.12196	0.193659	-0.07099	-0.77026	-0.2668	-0.67508	-0.8405	p13370	Ubiquitin ca	4	-0.29486	CDK1;	Fanconi anemia pathway;
URI1 seq: 3	-0.55082	-0.95838	-1.57172	-0.84732	-0.92262	-0.7926	-2.71679	-0.55721	p09060	Unconventi	4	-1.11468		GO:0071363; P:cellular response to growth factor stimulus;
URI1 seq: 3	-0.79253	-0.86703	-1.87914	-0.60879	-0.56363	-0.53914	-1.34079	-0.60287	p12832	Unconventi	4	-0.89924		Up with IGF - down with GO:0071363; P:cellular response to growth factor stimulus;
UQCRC1 se	-0.20422	0.485549	-0.09063	-0.88738	-0.01224	-0.46947	-2.96411	-0.89205	p14516	Cytochrome	4	-0.62932		
UQCRC1 se	-1.07837	-3.2192	-2.19277	-2.18578	0	0	0	0	p05142	Cytochrome	4	-1.08451		
UPF1 seq: 1	-0.4151	-0.58693	-0.36923	-0.2267	-0.34493	-0.52625	-0.3639	-0.41333	p19116	Regulator o	4	-0.4058	SMG1;	
UNK seq: 1	0	0	0	0	0	0	0	0	p04295	RING finger	4	0		GO:0007165; P:signal transduction;
UNK pS411	-0.47194	0.273391	-0.79263	-0.62406	-2.24639	-1.73281	-1.62415	-0.5084	p12747	RING finger	4	-0.96587		GO:0007165; P:signal transduction;
UNG seq: 6	-0.82971	-1.27301	-0.2088	-0.76845	-0.63609	-0.61776	-0.65478	-0.54604	p02506	Uracil-DNA	4	-0.69183		
UNG seq: 5	-0.61861	-0.72564	-0.05314	-0.23952	-0.00073	-0.09507	-0.13269	-0.06308	p13418	Uracil-DNA	4	-0.24106	CDK1;	CAMKinh1 (KN-93); AKTinh2 (MK-2206); AKTinh1 (Akt Inhibitor VIII);
Uncharacte	-0.07461	-1.00513	-0.52701	-0.36246	-0.11755	-0.37929	-0.40343	-0.38888	p08859	Uncharacte	4	-0.40729		
Uncharacte	-0.7362	-0.16436	-0.89514	-0.45051	-0.11766	-0.2268	-0.38156	-0.51143	p13848	Uncharacte	4	-0.43546		
ULK1 seq: 4	-0.12233	-0.49736	-0.75183	-1.14296	0.505987	0.002668	0.861662	0.273338	p19529	Serine/threo	4	-0.10885		mTOR signa GO:0007265; P:Ras protein signal transduction;
ULK1 pS55	0.026233	-0.02724	-0.76516	-0.5136	-0.00153	-0.13468	0.498764	0.121825	p11635	Serine/threo	4	-0.09942		PI3Kinh1 (G mTOR signa GO:0007265; P:Ras protein signal transduction;
ULK1 pS47	-1.15895	-0.49335	-1.13347	-1.19158	-0.93941	-0.76974	-0.98116	-1.26052	p19530	Serine/threo	4	-0.99102		mTOR signa GO:0007265; P:Ras protein signal transduction;
ULK1 pS45	-0.67071	-0.36154	-0.3562	-0.3829	-0.21215	-0.30754	-0.34456	-0.27312	p09420	Serine/threo	4	-0.36359		mTOR signa GO:0007265; P:Ras protein signal transduction;
UIMC1 seq	-4.10411	0.354586	-0.45388	0.017951	-0.73764	-0.06395	-0.57497	-0.81721	p15270	BRCA1-A co	4	-0.7974		ATM pathway;
UFD1L pS2	-0.18247	-0.33442	-0.01981	-0.07965	-0.37738	-0.24906	-0.24639	-0.47379	p15044	Ubiquitin fu	4	-0.24537		
UBXN6 seq	-0.65558	-0.40688	-0.54669	-0.75517	-2.32506	-1.34551	-2.42161	-3.84165	p13286	UBX domai	4	-1.53727		
UBXN2B pS	-0.39779	1.539895	-0.07147	0.593063	-1.47947	-2.08835	-3.80288	-2.66731	p19295	UBX domai	4	-1.04679	CDK1;	
UBR5 pS15	-0.42747	-0.08667	-0.90838	-0.23239	0.11363	0.214112	-0.39197	0.013916	p07246	E3 ubiquitir	4	-0.21315		GO:0090263; P:positive regulation of canonical Wnt signaling pathway;
UBR5 pS15	-0.56797	-0.15941	-0.90339	-0.31403	0.174491	0.306247	-0.22157	0.191997	p07245	E3 ubiquitir	4	-0.1867		GO:0090263; P:positive regulation of canonical Wnt signaling pathway;
UBR3 seq:	-0.44412	-0.0597	-0.5081	-2.29661	0	0	0	0	p16520	E3 ubiquitir	4	-0.41357		
UBQLN4 se	-0.15471	0.073877	-0.27371	-0.24145	-0.82475	-0.34916	-0.73144	-0.65987	p19824	Ubiquilin-4	4	-0.39515		
UBL7 pM2	-0.49969	-0.69897	-0.26925	-0.04431	0.052661	0.034953	-0.03231	-0.02787	p18483	Ubiquitin-li	4	-0.1856		Up with IGF - down with PKCinh2 (Bisindolylmaleimide-1); Up with IGF - down with PKCinh1 (Go-6976); Up with I
UBL4A pS9	-0.22064	-0.90626	-0.02954	-0.11174	0.071059	0.225427	0.137665	-0.06216	p12286	Ubiquitin-li	4	-0.11202		
UBE4B seq:	-0.82679	-0.72311	-0.59208	-0.78103	-1.14627	-0.95119	-1.23768	-1.68838	p04166	Ubiquitin co	4	-0.99332		p73 transcription factor network;
UBE2O seq	-2.11766	-11.3394	-1.70605	-2.40128	0	0	0	0	p19471	(E3-indeper	4	-2.19555		
UBE2O pS4	-0.33771	-0.25447	-0.03571	0.115307	-0.24924	-0.21419	-0.52341	-0.38165	p13938	(E3-indeper	4	-0.23514		
UBE2O pM	-1.30151	-1.34218	-0.89537	-1.15417	-0.67755	-0.34345	-0.85973	-0.64441	p13939	(E3-indeper	4	-0.9023		
UBAP2L sec	-0.58995	-0.10623	-0.49603	-0.87663	-1.58557	-0.83168	-1.02877	-0.72593	p06402	Ubiquitin-a	4	-0.7801		
UBAP2L sec	-0.27264	-0.80863	0.096538	-0.04064	0.155241	-0.31506	0.067254	-0.03304	p06404	Ubiquitin-a	4	-0.14387		p70S6Kinh2 (DG2);
UBAP2L sec	-0.08794	-0.6132	0.216086	0.147735	0.242757	-0.22503	-0.00908	-0.03708	p06403	Ubiquitin-a	4	-0.04572		p70S6Kinh2 (DG2);
UBAP2L sec	-0.70454	-0.86135	-0.58013	-0.46507	0.100457	-0.14158	-0.30855	-0.20371	p03475	Ubiquitin-a	4	-0.39556		
UBAP2L pS	-0.14354	-0.073	-0.09478	-0.1967	-0.4699	-0.23233	-0.38642	-0.62089	p06401	Ubiquitin-a	4	-0.2772		
UBAP2 pS4	-0.23923	-0.58613	-0.1786	-0.24724	-0.06005	0.19576	0.15726	-0.07065	p10903	Ubiquitin-a	4	-0.12861		
UBAP2 pS2	-0.2882	-0.31985	-0.51193	-1.49489	-1.43789	-0.69978	-0.44994	-0.36979	p16248	Ubiquitin-a	4	-0.69653		
UBAP1 seq	-0.34259	-1.81559	-0.64118	-0.1369	0.034817	-1.14049	0.415186	0.291741	p12175	Ubiquitin-a	4	-0.41688		
UBA6 pS36	0.347871	0.299535	-0.27811	-0.57406	0.286245	0.812266	-7.6958	-1.71243	p09427	Ubiquitin-li	4	-1.06431		
UBA2 seq:	-0.98286	-1.36009	-0.05433	-1.10071	0.320983	-0.01836	-0.93137	-0.84943	p10820	SUMO-activ	4	-0.62202		
UBA1 seq:	0.50252	0.605276	0.53448	0.492275	1.869564	1.042324	2.409257	-7.77072	p19361	Ubiquitin-li	4	-0.03938		
UBA1 seq:	0.062622	0.708822	-0.77249	-2.71708	0	0	0	0	p20990	Ubiquitin-li	4	-0.33977		
UBA1 seq:	-0.93066	-0.58136	-1.15239	-1.14325	-2.51494	0.177299	-1.79205	-1.98309	p12776	Ubiquitin-li	4	-1.24006		
UBA1 seq:	0.130256	0.001598	-0.23377	-0.29846	-0.56958	0.918217	-0.24006	-1.74175	p09688	Ubiquitin-li	4	-0.25419		
UBA1 seq:	-0.89033	-0.17987	-1.24509	-1.1382	-0.2121	-0.74125	1.342113	-6.87248	p18430	Ubiquitin-li	4	-1.24215		
UBA1 seq:	-0.90382	-0.69817	-0.72111	-0.63586	0.22165	0.231046	-0.45876	0.298785	p20116	Ubiquitin-li	4	-0.33328		
UBA1 pS81	-1.15268	-0.57217	-2.10108	-1.36285	-0.08388	-0.1813	-0.14595	-0.25782	p14201	Ubiquitin-li	4	-0.73222		Akt-mTOR; Akt-mTOR-PI3K; PKCinh2 (Bisindolylmaleimide-1); PI3Kinh2 (PI-103); PI3Kinh1 (GDC-0941); p70S6Kin
U2AF2 pS7	0.2792	0.222803	0.253728	-0.08373	-0.6365	-0.69748	-0.60289	-1.14544	p16304	Splicing fact	4	-0.30129	CDK1;	
U2AF2 pS7	0.246594	0.316079	0.240663	-0.03566	-0.59745	-0.74208	-0.59401	-1.2254	p16303	Splicing fact	4	-0.29891	CDK1;	
TXNDC5 se	-0.55345	0.034207	-1.00341	-1.01334	0.045043	0.471713	0.411766	0.536145	p01702	Thioredoxir	4	-0.13392		
TXN seq: 8	-1.05189	-0.21245	-0.84158	-1.08398	0.417489	0.302189	-0.21486	-0.08683	p01497	Thioredoxir	4	-0.34649		p38 MAPK GO:0035872; P:nucleotide-binding domain, leucine rich repeat containing receptor signali
TXN seq: 8	-0.36748	-0.08631	-0.19228	-0.60102	0.507102	0.440818	0.264323	0.682682	p01498	Thioredoxir	4	0.08098		p38 MAPK GO:0035872; P:nucleotide-binding domain, leucine rich repeat containing receptor signali
TXLNA seq:	-0.45274	-0.20142	-0.23347	-0.13527	-0.09984	-0.3147	-0.34147	-0.23227	p03810	Alpha-taxilii	4	-0.2514		
TXLNA seq:	-0.26578	-0.69876	-0.48143	-0.49435	-0.52203	-0.48559	-0.5389	-0.6896	p07870	Alpha-taxilii	4	-0.52206		
TXLNA seq:	-1.81954	-1.93581	0.482717	-1.45859	-0.47328	-0.52277	-0.77386	-0.73647	p07871	Alpha-taxilii	4	-0.9047		
TWIST1 pS6	-0.50899	-0.71541	-0.35568	-0.44769	-0.05652	-2.2207	0.650171	-1.71425	p06338	Twist-relate	4	-0.67113	P38A; JNK1; ERK2; ERK1	HIF-2-alpha GO:0043518; P:negative regulation of DNA damage response, signal transduction by p53
TUBB8 seq:	-1.09014	-0.19749	-1.44651	-2.01473	0.251023	0.14582	-0.33216	-0.04684	p03000	Tubulin bet	4	-0.59138		
TUBB4B pM	-0.96135	-0.14114	-1.19113	-1.16397	0.969716	0.423702	0.208292	0.264686	p09368	Tubulin bet	4	-0.1989		
TUBB3 pM	-0.78868	-0.7917	-0.92258	-1.88844	0.6347	-1.69503	-2.0395	-0.98473	p09369	Tubulin bet	4	-1.05949		
TUBB pM3	-0.34033	-0.54992	-0.43055	-0.48889	0.189526	0.153252	-0.03785	-0.15169	p17013	Tubulin bet	4	-0.20706		
TUBA4A se	-0.55703	-0.56904	-1.04929	-1.09741	-0.12652	0.642731	0.78054	0.673917	p14215	Tubulin alpl	4	-0.16276		
TUBA3C se	-0.18494	0.048026	-0.82168	-1.19652	-0.90001	-0.10047	-0.08491	-0.52974	p07031	Tubulin alpl	4	-0.47128		
TUBA1C se	-0.16824	1.564405	-1.13961	-2.71391	-3.37248	-0.49241	-1.21498	-0.12684	p19124	Tubulin alpl	4	-0.78932		
TUBA1B se	-0.97823	-0.80752	-1.08285	-1.40476	0.304487	0.306883	0.209976	-0.14387	p10607	Tubulin alpl	4	-0.44949		Signaling events mediated by PRL; Signaling events mediated by HDAC Class III; Signaling events mediate
TUBA1B pS	-0.2477	-0.69608	0.075821	-0.03187	-0.10108	-0.10482	-0.21869	-0.15112	p05487	Tubulin alpl	4	-0.18444		Signaling events mediated by PRL; Signaling events mediated by HDAC Class III; Signaling events mediate
TTN seq: 20	-0.27566	0.635157	-1.1363	-1.80316	0.196864	-0.43367	-2.1645	1.413525	p14405	Titin	4	-0.44597		GO:0045859; P:regulation of protein kinase activity;
TTK pS436	-0.27685	-0.26241	-0.39284	-0.44606	-1.03784	-1.01712	-0.87407	-0.97055	p14578	Dual specifi	4	-0.65972	TTK; BRAF;	PI3Kinh2 (PI-103); mTORinh1 (KU-0063794);
TTC28 pS2	-0.38434	-0.26713	-0.15714	-0.70829	0	0	0	0	p04234	Tetratricope	4	-0.18961		
TSTD1 seq:	-1.56982	-3.24625	-3.7538	-1.40434	0	0	0	0	p15666	Thiosulfate	4	-1.24678		
TSC22D4 se	-0.32898	-0.02981	0.025144	-0.22266	-0.19551	-0.05824	-0.13625	-0.17606	p20829	TSC22 dom	4	-0.1403		
TSC22D4 p	-0.91115	-1.91733	-0.05454	-0.4078	-0.18561	-0.31394	-0.40711	-0.23648	p05806	TSC22 dom	4	-0.55425		
TSC22D1 se	0.313796	-1.02253	-0.44467	-0.20731	0.260412	-1.32479	-1.78245	-0.14486	p12898	TSC22 dom	4	-0.54405		
TSC2 seq: 9	-0.24986	-0.65519	-0.63089	-0.54187	-1.64229	-2.02027	-1.52965	-2.22804	p05449	Tuberin	4	-1.18726	Akt1;	AKTinh1 (A p38 signalin GO:0014067; P:negative regulation of phosphatidylinositol 3-kinase signaling; GO:00480
TSC2 seq: 1	-0.53933	-0.81766	-0.65594	-0.65767	-0.67863	-0.73416	-0.9623	-0.70822	p15385	Tuberin	4	-0.71924		p38 signalin GO:0014067; P:negative regulation of phosphatidylinositol 3-kinase signaling; GO:00480
TSC2 seq: 1	-0.12267	-1.27329	-0.18329	-0.27895	0.359775	0.478438	0.792108	1.16404	p03632	Tuberin	4	0.11702		AKTinh1 (A p38 signalin GO:0014067; P:negative regulation of phosphatidylinositol 3-kinase signaling; GO:00480
TSC2 seq: 1	-0.29112	-1.03166	-0.49638	-0.76137	0.161804	0.433907	-0.08067	0.517819	p04873	Tuberin	4	-0.19346		p38 signalin GO:0014067; P:negative regulation of phosphatidylinositol 3-kinase signaling; GO:00480
TSC2 pS93	0.263301	-0.28805	0.258412	0.197543	-0.47541	-0.96107	-0.48232	-0.76625	p03452	Tuberin	4	-0.28173	Akt1;	p38 signalin GO:0014067; P:negative regulation of phosphatidylinositol 3-kinase signaling; GO:00480
TSC2 pS142	-0.19124	-0.35645	-0.04609	0.228154	-0.6504	-3.31208	-0.28826	-0.30563	p04195	Tuberin	4	-0.61525		p38 signalin GO:0014067; P:negative regulation of phosphatidylinositol 3-kinase signaling; GO:00480
TRPS1 pS1	-0.10321	-1.2237	-0.3502	-0.39683	0.621879	0	0.966754	0	p14695	Zinc finger t	4	-0.06066		GO:0007178; P:transmembrane receptor protein serine/threonine kinase signaling pathwa
TRIP12 seq	-0.0926	-0.31489	-0.01998	0.003828	-0.21774	-0.35576	-0.18912	-0.82032	p06685	E3 ubiquitir	4	-0.25082		mTORinh2 (Torin-1); EGFRinh1 (PD-168393);
TRIP12 pS3	-0.12638	-0.34327	-0.05377	-0.05442	-0.19213	-0.32842	-0.16352	-0.79137	p05971	E3 ubiquitir	4	-0.25666		mTORinh2 (Torin-1); EGFRinh1 (PD-168393);
TRIP11 seq	0.275854	0.5423	0.122339	0.020234	-1.66826	-1.31526	-0.7903	-1.19571	p10660	Thyroid rec	4	-0.5011		
TRIP11 seq	0.258859	-0.10342	0.875118	1.045754	-0.79632	-1.08634	-0.42174	-0.71755	p10603	Thyroid rec	4	-0.11821		
TRIP10 pS4	-0.25464	-0.53501	-0.33236	-0.15816	-0.06366	-0.02753	-0.36086	-0.30077	p01173	Cdc42-inte	4	-0.25412		Insulin-mec GO:0051056; P:regulation of small GTPase mediated signal transduction; GO:0007264; P:
TRIP10 pS2	-0.03435	-0.16238	0.248436	0.193477	0.071716	-0.22723	-0.0248	-0.12248	p19924	Cdc42-inte	4	-0.0072		Insulin-mec GO:0051056; P:regulation of small GTPase mediated signal transduction; GO:0007264; P:
TRIOBP pS1	-1.09122	-1.61988	-0.64057	-0.72009	-0.26315	-0.32076	-0.50029	-0.37447	p08847	TRIO and F-	4	-0.6913		
TRIOBP pS1	-0.75051	-0.84861	-0.38158	-0.00447	0.052811	-0.77674	-1.05032	-0.31957	p08848	TRIO and F-	4	-0.50987		
TRIM36 sec	0.195099	0.436251	0.202031	0.071975	-0.81859	0.075752	-0.57233	-1.78961	p10886	E3 ubiquitir	4	-0.27493		
TRIM36 pS	-0.42168	-0.93533	0.057343	-0.0728	0	0	0	0	p04245	E3 ubiquitir	4	-0.17156		
TRIM3 pS4	-0.04428	-0.08523	-0.33505	0.062594	-2.40892	0.47856	-0.0345	-2.401	p01296	Tripartite m	4	-0.59598		
TRIM28 sec	-0.71915	-0.56357	-0.19722	-0.1717	-0.22153	-0.28165	-0.63193	-0.19125	p12183	Transcriptic	4	-0.37225		ATM pathway; p53 pathway; E2F transcription factor network; C-MYB transcription factor network;
TRIM28 sec	-0.2285	0.263861	-1.04397	-0.05118	0.535899	0.979718	-1.27985	0.183548	p04056	Transcriptic	4	-0.08006	Chk2;	ATM pathway; p53 pathway; E2F transcription factor network; C-MYB transcription factor network;
TRIM28 sec	-2.06492	-0.18998	-1.55964	-3.54252	0	0	0	0	p10059	Transcriptic	4	-0.91963		ATM pathway; p53 pathway; E2F transcription factor network; C-MYB transcription factor network;
TRIL seq: 2	3.009294	-5.66696	-5.6787	1.554786	-0.75072	0.082944	1.174543	-1.98057	p11742	TLR4 intera	4	-1.03192		GO:0034142; P:toll-like receptor 4 signaling pathway;
TRAFD1 pS	-0.83018	-0.86528	-0.76134	-0.70121	-0.0694	0.023965	-0.14989	-0.19011	p10721	TRAF-type z	4	-0.44304		
TRA2A pT2	0.779743	1.050662	-0.42589	-0.62303	-1.00894	-0.9305	-0.90811	-0.57564	p20496	Transforme	4	-0.33021		
TRA2A pT2	0.442956	0.487048	-0.20729	-0.2617	-0.47943	-0.38385	-0.41112	-0.37266	p20495	Transforme	4	-0.14826		
TPX2 pS48	-0.25677	-0.92515	0.237888	-0.00415	-0.48527	-0.34653	-0.04604	-0.47531	p01197	Targeting pr	4	-0.28767		Aurora A sig GO:0032147; P:activation of protein kinase activity;
TPX2 pS48	0.098016	-0.50062	0.452677	0.226387	0.141708	0.007852	0.109485	-0.15783	p04732	Targeting pr	4	0.04721		Aurora A sig GO:0032147; P:activation of protein kinase activity;
TPT1 pS46	1.2508	-0.68249	0.387248	0.486584	-2.52842	-1.11671	0.104794	0.248694	p02911	Translationa	4	-0.23119		Up with IGF PLK1 signaling events;
TPRN pS24	-0.10901	-0.16622	-0.01471	-0.00686	-1.44859	-1.26444	-1.07917	-1.10867	p12254	Taperin	4	-0.64971		GO:0019221; P:cytokine-mediated signaling pathway;
TPRN pS24	-0.25982	-0.27942	-0.18982	-0.08039	-0.82637	-1.12753	-0.75332	-0.87272	p12253	Taperin	4	-0.54867		GO:0019221; P:cytokine-mediated signaling pathway;
TPR seq: 64	-1.65731	-0.67982	-1.33109	-2.5683	-1.08489	-0.73781	-1.57287	-3.20188	p06838	Nucleoprot	4	-1.60424		GO:0019221; P:cytokine-mediated signaling pathway;
TPR seq: 61	-2.80707	-9.28107	-0.26441	0.52616	-0.36042	0.172641	0.436068	-0.02263	p14019	Nucleoprot	4	-1.45009		GO:0019221; P:cytokine-mediated signaling pathway;
TPM2 seq:	-0.47654	0.304905	-0.52186	-0.4034	-0.49744	-0.15601	0.102534	0.765662	p13806	Tropomyos	4	-0.11027		
TPI1 seq: 5	-0.59311	-0.34775	-1.86327	-0.69333	-0.04495	0.528477	0.546034	0.946184	p12648	Triosephos	4	-0.19022		
TPI1 seq: 2	-0.26806	0.717351	-0.58721	-0.37352	-0.5337	-0.38307	-1.31984	-0.7756	p04739	Triosephos	4	-0.44045		
TPI1 seq: 2	-0.44757	-0.32676	-0.69268	-0.77013	0.355107	0.542944	0.383189	0.789191	p04740	Triosephos	4	-0.02084		
TPI1 seq: 1	-0.89057	0.281642	-0.91178	-1.16701	0.882027	0.423005	-0.18431	0.729667	p01409	Triosephos	4	-0.10467		
TPI1 seq: 1	-0.30929	-0.06231	-0.47264	-0.96161	0.284574	0.664891	0.400708	1.067512	p14569	Triosephos	4	0.076479		
TPI1 pS58	0.613018	0.351337	0.135436	-0.42272	-1.26438	-0.88879	-0.77902	0.340323	p12644	Triosephos	4	-0.23935		
TPD52L2 se	-0.18396	-0.35782	-0.13653	-0.59589	-1.86185	-0.88342	-1.44618	-1.55301	p12478	Tumor prot	4	-0.87733		
TPD52L2 se	2.200911	1.07157	-6.9186	-0.3897	-0.36757	-0.14418	-0.29947	-0.32369	p10201	Tumor prot	4	-0.64634		AKTinh1 (Akt Inhibitor VIII);
TPD52L1 p	-0.9577	-0.86341	-1.96265	-3.25575	0	0	0	0	p01478	Tumor prot	4	-0.87994		
TPD52L1 p	-0.37369	-0.04214	-0.4167	-0.9642	-0.10666	-0.45556	-0.22688	-0.00019	p01479	Tumor prot	4	-0.32325		
TPD52L1 p	-0.04831	-0.26513	-0.36344	-1.37478	-3.49467	-1.35736	-2.12672	-1.28839	p09109	Tumor prot	4	-1.28985		p70S6Kinh2 (DG2);
TPD52L1 p	0.11511	-0.46359	-0.16028	-0.85871	-0.54136	-1.32119	-0.98809	-1.95724	p05920	Tumor prot	4	-0.77192		p70S6Kinh2 (DG2);
TPD52 pS1	0.526511	0.397491	-0.42609	-1.03161	-1.29527	-3.78736	-1.29228	-1.6064	p09113	Tumor prot	4	-1.06438	CAMK2D is	p70S6K-PKC; PAKinh (PF3758309); p70S6Kinh2 (DG2);
TP53BP2 se	-0.78114	-0.84759	-0.78196	-1.54931	-3.4947	-2.19195	-1.62964	-1.25195	p03868	Apoptosis-	4	-1.56603		Direct p53 GO:0009967; P:positive regulation of signal transduction; GO:0007165; P:signal transduc
TP53BP2 se	-0.27137	-0.32462	-0.17595	-0.29035	-0.5515	-2.38639	-0.32293	-0.49876	p06610	Apoptosis-	4	-0.60273		Direct p53 GO:0009967; P:positive regulation of signal transduction; GO:0007165; P:signal transduc
TP53BP1 se	-0.29414	-0.73192	-0.16599	-0.0614	0.176959	0.038348	-0.2732	-0.15946	p07854	Tumor supp	4	-0.18385		CAMKinh1 ATM pathway;
TP53BP1 p	-0.50322	-0.23918	-0.3405	-0.32604	-0.58194	-0.40577	-0.64448	-0.75342	p17077	Tumor supp	4	-0.47432		Akt-mTOR-F ATM pathway;
TP53BP1 p	-0.03425	-0.77133	0.200632	0.290113	0.207103	0.192879	0.224311	0.185292	p17078	Tumor supp	4	0.061844		Akt-mTOR-F ATM pathway;
TOR4A seq:	-0.41004	-1.31325	-0.59451	-0.39465	-0.19056	0.07364	0.010695	-0.04407	p11304	Torsin-4A	4	-0.35784		
TOPORS pS	-0.48718	-0.02622	0.055095	-0.12563	-0.57748	-1.23662	-1.56536	-1.27285	p09743	E3 ubiquitir	4	-0.65453		
TOP2B seq:	0.182972	0.064601	-0.30349	-0.13536	-0.80665	-1.08721	-0.50609	-1.30521	p14316	DNA topois	4	-0.48706		
TOP2B seq:	0.217701	0.449396	-0.16087	0.250773	0.711704	0.315561	1.881005	-8.83398	p01331	DNA topois	4	-0.64609		AKTinh1 (Akt Inhibitor VIII);
TOP2A pM	0.004205	-1.10405	0.013008	-0.35018	0.23266	-0.20059	-0.00182	-0.20426	p02274	DNA topois	4	-0.20138	ERK2; ERK1	CAMKinh1 Validated tr GO:0048015; P:phosphatidylinositol-mediated signaling;
TNS3 seq: 7	0.282779	-0.56385	0.034331	0.242303	-0.32645	-0.57056	-0.70569	-0.23443	p12921	Tensin-3	4	-0.2302		Up with IGF - down with PI3Kinh1 (GDC-0941); Up with EGF - down with PI3Kinh1 (GDC-0941); Up with IGF - dow
TNS3 pS77	-0.21171	-3.3729	0.218815	-0.14237	-0.36984	-0.6164	-0.77009	-0.33418	p12922	Tensin-3	4	-0.69983		Up with IGF - down with PI3Kinh1 (GDC-0941); Up with EGF - down with PI3Kinh1 (GDC-0941); Up with IGF - dow
TNS2 seq: 8	-0.22275	0.967289	-0.45597	0.43535	-1.23237	-0.65425	0.186604	-0.42121	p20577	Tensin-2	4	-0.17466		
TNS1 seq: 1	-0.1072	-0.12684	0.111434	0.130729	-4.31073	-0.2887	-2.69368	0.562035	p01742	Tensin-1	4	-0.84037		Integrin-linked kinase signaling;
TNRC6A pS	-0.24741	-0.16229	-3.28124	-1.40117	-0.5423	-0.89278	-1.97672	-1.85362	p11699	Trinucleotic	4	-1.29469		GO:0007219; P:Notch signaling pathway; GO:0048015; P:phosphatidylinositol-mediated
TNRC18 pS	0.063433	-0.09827	0.12811	0.057379	-0.4895	-1.32303	-0.18179	-1.22014	p04678	Trinucleotic	4	-0.38298		
TNRC18 pS	0.452936	-0.04469	0.127328	0.369934	-1.58072	-0.71551	0.002995	-0.70253	p12272	Trinucleotic	4	-0.26128		
TNRC18 pS	1.101218	1.793212	1.300056	1.794131	0.341495	-1.16953	-0.96317	-6.46578	p12231	Trinucleotic	4	-0.28355		
TNKS1BP1	-0.62028	-0.34345	-0.95762	-1.16749	-0.27429	0.239861	-0.43161	-0.2464	p00623	182 kDa tar	4	-0.47516		
TNKS1BP1	0.553921	-2.82355	0.432718	0.416619	-0.54543	-0.26271	-0.58731	-0.71085	p03884	182 kDa tar	4	-0.44082		
TNKS1BP1	-0.71377	-1.72876	0.020272	0.344399	-0.45052	-0.3702	-0.21953	0.005938	p17482	182 kDa tar	4	-0.38902		
TNKS1BP1	-0.31435	-0.84394	-0.19832	-0.39064	-0.48086	-1.08447	-0.58567	-0.11749	p02060	182 kDa tar	4	-0.50197		AKTinh1 (Akt Inhibitor VIII);
TNKS1BP1	-0.76469	-0.79025	-0.26149	-0.38332	-0.10044	-0.24081	-0.31303	-0.24607	p15037	182 kDa tar	4	-0.38751		
TNKS1BP1	1.893354	3.300304	1.100532	-7.98496	-0.54428	-0.66417	0.59824	0.641438	p05907	182 kDa tar	4	-0.20744		
TNKS1BP1	-0.22986	0.184352	-1.23552	-0.62951	-1.20495	-0.57323	-0.39049	1.401523	p18550	182 kDa tar	4	-0.33471		
TNKS1BP1	-0.83032	-2.23119	-0.82667	-0.64124	-0.91128	-1.12795	-0.81964	-0.16683	p12041	182 kDa tar	4	-0.94439		AKTinh1 (Akt Inhibitor VIII);
TNKS1BP1	0.24904	-1.12402	-0.35462	-0.08544	-0.24801	-0.44829	-0.25453	-0.37874	p07437	182 kDa tar	4	-0.33058		
TNKS1BP1	0.241333	-1.32659	-0.26741	-0.01518	0.260385	-0.52274	-0.08356	0.103062	p07436	182 kDa tar	4	-0.20134		
TNKS1BP1	-0.42302	-0.80896	-0.21202	-0.17278	-0.25023	-0.01453	-0.07097	-0.25151	p01065	182 kDa tar	4	-0.2755		
TNKS1BP1	-0.19518	-0.68367	-0.17457	-0.2244	-0.2481	-0.83138	-0.4745	-0.37012	p02061	182 kDa tar	4	-0.40024		AKTinh1 (Akt Inhibitor VIII);
TNK1 seq: 5	-0.33498	-0.67919	-0.04446	-0.22041	-0.01257	-0.07271	-0.08837	-0.0775	p05243	Non-recept	4	-0.19127		GO:0046580; P:negative regulation of Ras protein signal transduction; GO:0007165; P:sig
TNFRSF10A	-1.98184	-0.29983	-0.50025	-1.42291	-0.45351	0.007561	0.034613	0.15964	p16658	Tumor necr	4	-0.55707		mTORinh1 TRAIL signal GO:0007165; P:signal transduction;
TMX1 pS27	-0.00866	-0.46345	-0.8014	-0.72654	0.910849	1.208823	-5.41171	-5.37086	p07958	Thioredoxir	4	-1.33287		
TMSB4X se	-1.06845	0.087331	-1.22023	-1.18856	0.296791	-0.02455	-0.51988	0.221486	p12502	Thymosin b	4	-0.42701		
TMSB4X se	-0.4818	-0.3423	-0.4623	-0.8284	-0.07736	-0.45026	0.02379	-0.14054	p12500	Thymosin b	4	-0.3449		
TMSB10 se	-0.68065	-0.76033	-0.90274	-1.14375	-0.14563	-0.35791	-0.45833	-0.67772	p09020	Thymosin b	4	-0.64088		
TMSB10 se	-0.49703	-0.13169	-0.70927	-1.19121	-0.17381	-0.19487	-0.3437	-0.51385	p09021	Thymosin b	4	-0.46943		
TMPRSS13	0.008086	-0.10738	-0.72265	-0.17066	-1.35362	-0.76573	-1.97764	-0.39889	p19560	Transmemb	4	-0.68606		
TMPRSS13	-0.0803	-0.6626	-0.12442	-1.78424	0.373604	0.487262	0.090362	1.413515	p09505	Transmemb	4	-0.03585		
TMPO seq:	-0.44521	0.005633	-0.35509	-0.64652	0.23815	0.490589	0.570681	0.128458	p06013	Lamina-ass	4	-0.00166		
TMPO pS6	-0.06643	-0.52382	-1.00076	-0.81721	-0.10119	-1.03406	-1.37854	-0.15888	p09187	Lamina-ass	4	-0.63511		
TMPO pS42	-0.25258	-1.06297	0.189378	0.145295	-0.52575	-0.33188	0.237577	-0.15684	p16517	Lamina-ass	4	-0.21972	CDK1;	
TMPO pS3	0.005977	-0.60634	0.300933	0.086115	0.013905	-0.1386	0.048564	-0.15819	p05597	Lamina-ass	4	-0.05595		
TMF1 seq:	-0.55838	-1.30621	-0.66754	-0.64758	-0.90458	-0.63004	-0.24602	-0.17851	p03282	TATA eleme	4	-0.64236		Coregulation of Androgen receptor activity;
TMEM132A	-0.41512	-0.90463	-0.47165	-0.49173	-0.26985	-0.55663	-0.20371	-0.30854	p10910	Transmemb	4	-0.45273		
TLN1 seq: 4	-0.14286	-0.54716	-0.1602	-0.30441	0.124094	-0.3038	-0.03613	-0.39662	p05435	Talin-1	4	-0.22089	CDK5;	Nectin adhe GO:0006987; P:activation of signaling protein activity involved in unfolded protein respor
TLN1 seq: 1	-0.69364	-0.37611	-0.93547	-0.43003	0	0	0	0	p20312	Talin-1	4	-0.30441		Nectin adhe GO:0006987; P:activation of signaling protein activity involved in unfolded protein respor
TLE3 pT328	-0.01014	-0.38733	0.129276	0.072457	0.299068	-0.56881	0.066273	0.051079	p09718	Transducin	4	-0.04351		GO:0007219; P:Notch signaling pathway; GO:0016055; P:Wnt signaling pathway; GO:000
TLE3 pS217	-0.13011	-0.11975	-0.28169	-0.71822	-0.56608	-0.62208	-0.32147	-0.33956	p14664	Transducin	4	-0.38737		GO:0007219; P:Notch signaling pathway; GO:0016055; P:Wnt signaling pathway; GO:000
TKT seq: 31	-0.97823	-1.20949	-1.06019	-1.58503	0.873381	0.505725	-0.74261	0.122302	p13360	Transketola	4	-0.50927		
TJP3 seq: 3	-0.1263	-0.61109	-0.1314	-0.23389	-1.64806	-2.9792	-0.48746	-1.41658	p17105	Tight juncti	4	-0.95425		
TJP3 pS591	-0.31722	-0.50083	-0.09981	-0.2213	0.274095	-0.2473	-0.05703	0.025614	p04130	Tight juncti	4	-0.14297		
TJP2 seq: 9	-0.52068	-1.02032	-0.4237	-0.30491	-0.29005	-0.62337	-0.71229	-0.42993	p21015	Tight juncti	4	-0.54066		Validated ta GO:0035329; P:hippo signaling;
TJP2 seq: 9	-0.29384	-0.59448	-0.10261	-0.374	0.101153	-0.15079	-0.19123	-0.21098	p07654	Tight juncti	4	-0.2271		Validated ta GO:0035329; P:hippo signaling;
TJP2 seq: 9	-0.29008	-0.60418	-0.09206	-0.3923	0.12073	-0.13692	-0.18223	-0.2002	p07655	Tight juncti	4	-0.22215		Validated ta GO:0035329; P:hippo signaling;
TJP2 seq: 4	-0.15052	-0.67657	-0.18967	-0.1901	-0.29251	-0.71972	-0.08937	-0.37991	p10126	Tight juncti	4	-0.33605		Validated ta GO:0035329; P:hippo signaling;
TJP2 seq: 4	-0.15717	-0.52755	-0.14188	-0.12433	-0.33655	-0.69947	-0.02703	-0.32535	p10127	Tight juncti	4	-0.29242		Validated ta GO:0035329; P:hippo signaling;
TJP2 seq: 3	0.152309	-1.34033	-0.93009	0.195891	-0.84022	-0.32705	-1.62755	-0.56266	p20059	Tight juncti	4	-0.65996		Validated ta GO:0035329; P:hippo signaling;
TJP2 seq: 1	-0.98812	-0.21376	-0.22914	-0.99212	0.251005	-0.0889	-0.15063	0.038291	p05516	Tight juncti	4	-0.29667		Validated ta GO:0035329; P:hippo signaling;
TJP2 pT925	-0.07716	-0.65214	0.08968	0.169202	0.073866	0.194651	-0.15194	0.031903	p11556	Tight juncti	4	-0.04024		Validated ta GO:0035329; P:hippo signaling;
TJP2 pT102	-0.20325	-0.5402	0.032611	0.066732	-0.01705	-0.24795	0.204201	-0.06604	p04822	Tight juncti	4	-0.09637		Validated ta GO:0035329; P:hippo signaling;
TJP2 pS325	-0.54281	-1.40962	0.038522	0.077139	0.360656	-0.6524	0.335992	0.186763	p11686	Tight juncti	4	-0.20072		Validated ta GO:0035329; P:hippo signaling;
TJP2 pS130	-7.6992	1.256481	0.288196	0.103573	-0.05579	-0.07588	-0.05309	-0.03253	p00917	Tight juncti	4	-0.78353		Validated ta GO:0035329; P:hippo signaling;
TJP1 seq: 9	-7.90307	3.92696	2.141684	0.824704	-0.23571	-0.20999	-0.11866	-0.36296	p20866	Tight juncti	4	-0.24213		Nephrin/Ne GO:0035329; P:hippo signaling;
TJP1 seq: 1	0.181263	-0.12096	0.058791	-0.74007	-0.50395	-0.52679	-0.2049	-0.49004	p05575	Tight juncti	4	-0.29333		Nephrin/Ne GO:0035329; P:hippo signaling;
TJAP1 seq:	0.15697	-0.16054	0.122146	0.022195	-0.18364	-0.49617	-0.5362	-0.44374	p13321	Tight juncti	4	-0.18987		
TICRR seq:	0.148008	-0.26666	0.218003	0.140886	-0.83627	-0.73632	-0.3195	-0.98215	p09464	Treslin	4	-0.32925		
THYN1 pS1	0.381937	-0.6207	-0.39538	-0.11244	-0.21051	-0.62791	-0.66473	-0.5443	p12250	Thymocyte	4	-0.34926		
THRAP3 pS	-0.22075	-0.26715	-0.39149	-1.47752	-0.35932	0.108694	-1.08092	-0.04869	p10197	Thyroid hor	4	-0.46714		CAMKinh1 (KN-93); GO:0030518; P:intracellular steroid hormone receptor signaling pathway; GO:0030521; P
THRAP3 pS	-0.48344	-0.99205	-0.15212	-0.17866	-0.50721	-1.23955	-0.93743	-0.57496	p03906	Thyroid hor	4	-0.63318		GO:0030518; P:intracellular steroid hormone receptor signaling pathway; GO:0030521; P
THRAP3 pS	-0.27625	-0.33454	-0.15625	-0.2691	0.251641	-0.0589	0.380323	-0.00438	p04545	Thyroid hor	4	-0.05843		GO:0030518; P:intracellular steroid hormone receptor signaling pathway; GO:0030521; P
THRAP3 pM	0.000894	-0.21321	-0.55893	-0.96071	-0.14902	0.189205	-1.12729	-0.75648	p10198	Thyroid hor	4	-0.44694		CAMKinh1 (KN-93); GO:0030518; P:intracellular steroid hormone receptor signaling pathway; GO:0030521; P
TGS1 pS89	-0.36688	-0.05497	0.152643	-0.38433	-0.23656	-0.20601	-0.34409	-0.11183	p15896	Trimethylgu	4	-0.194		AKTinh1 (Akt Inhibitor VIII);
TGFBR2 sec	-0.13307	-1.16794	-1.0576	-0.40379	-0.3335	-0.62641	-0.89832	-0.56525	p11683	TGF-beta re	4	-0.64824		ALK1 signal GO:0032147; P:activation of protein kinase activity; GO:0007224; P:smoothened signaling
TGFB1I1 se	-1.81149	-0.69201	-2.99961	-0.33366	0.228939	0.085732	0.077052	0.174874	p12624	Transformir	4	-0.65877		Coregulatio GO:0030511; P:positive regulation of transforming growth factor beta receptor signaling
TFRC seq: 7	-1.08575	-1.08208	-1.60175	-1.01792	0.25423	0.628444	0.325777	0.06249	p06348	Transferrin	4	-0.43957		FOXA2 and FOXA3 transcription factor networks; HIF-1-alpha transcription factor network; Validated ta
TFRC seq: 7	-1.28256	-1.43842	-1.12992	-1.3793	0.272592	0.608057	0.289379	0.075129	p06349	Transferrin	4	-0.49813		FOXA2 and FOXA3 transcription factor networks; HIF-1-alpha transcription factor network; Validated ta
TFPT pS252	-0.38467	-0.56253	-0.19185	-0.10029	0.193046	-0.10114	-0.58111	0.034397	p11322	TCF3 fusion	4	-0.21177		
TFPT pS180	-0.65326	-0.74418	-0.69334	-0.6881	-0.67654	-0.72393	-0.67427	-1.3145	p06512	TCF3 fusion	4	-0.77101	CDK1;	
TFPT pS180	-0.44242	-0.51182	-0.35073	-0.18029	0.105123	-0.00901	-0.2036	-0.10506	p06513	TCF3 fusion	4	-0.21223	CDK1;	
TFF3 seq: 3	-0.19086	0.756884	-0.64931	-0.70305	0	0	0	0	p18314	Trefoil facto	4	-0.09829		IL4-mediated signaling events; HIF-1-alpha transcription factor network;
TFEB seq: 3	-1.12182	-0.3085	-0.8184	-0.84682	-3.63626	-1.82068	-2.54103	-1.63343	p01422	Transcriptic	4	-1.59087		
TFAP4 seq:	0.188014	-0.62306	0.18523	0.113604	-0.54779	-0.35398	-0.73332	-0.69917	p16676	Transcriptic	4	-0.30881		
TFAP4 seq:	-0.03288	-0.60346	0.060065	0.069077	-0.4284	-0.27472	-0.56645	-0.52737	p16675	Transcriptic	4	-0.28802		
TFAP4 seq:	-0.12649	-0.0646	-0.30064	-0.61402	-0.2821	-0.01781	-0.40362	-0.61	p07444	Transcriptic	4	-0.30241		
TEX2 seq: 2	-0.04207	-0.04053	0.152108	-0.17731	-0.41494	-0.69005	-0.69337	-1.53215	p04977	Testis-expre	4	-0.42979		GO:0007165; P:signal transduction;
TEX2 pS26	0.084851	0.212282	-0.03305	0.079546	-4.77203	0.658228	-2.23425	-0.59027	p03067	Testis-expre	4	-0.82434		GO:0007165; P:signal transduction;
TERF2 pS36	-0.82122	-1.35996	-0.34795	-0.31167	-0.28939	-0.2984	-0.34753	-1.01395	p18516	Telomeric re	4	-0.59876	CDK2;	ATM pathway; Regulation of Telomerase;
TDRD7 pS8	-1.3593	-3.2203	-2.19255	-2.647	-0.02309	-0.06925	-0.20247	0.580572	p18116	Tudor doma	4	-1.14167		CAMKinh1 Aurora A signaling;
TCP1 seq: 5	0.063257	-0.33776	-0.50314	-0.48639	-0.5216	-0.41262	-0.2833	-0.79291	p18912	T-complex p	4	-0.40931		
TCOF1 seq:	0.057432	-0.7823	0.170312	-0.02794	-0.23885	-0.28163	-0.03978	-0.44922	p21052	Treacle prot	4	-0.199		
TCOF1 seq:	-0.16866	-1.40742	0.557296	0.070534	0.043658	-0.28519	0.002498	-0.21157	p21053	Treacle prot	4	-0.17486		
TCOF1 pS9	-0.15294	-0.2417	0.067831	0.168361	-0.58003	-0.26829	-0.27774	-0.26645	p21108	Treacle prot	4	-0.19387		
TCN1 seq: 3	0.00503	0.09711	-0.2016	-0.29403	0	0	0	0	p03197	Transcobala	4	-0.04919		
TCF3 seq: 3	-0.17006	0.230705	-0.05625	-0.30663	-0.95571	-0.20847	-0.37943	-0.96788	p20717	Transcriptic	4	-0.35172	MLK2;	p38 signaling mediated by MAPKAP kinases; Notch-mediated HES/HEY network; Regulation of nuclear S
TCF3 pS37	-0.17229	0.071159	-0.14686	-0.15869	-0.50665	-0.20381	-0.27879	-0.46919	p20716	Transcriptic	4	-0.23314	MLK2;	p38 signaling mediated by MAPKAP kinases; Notch-mediated HES/HEY network; Regulation of nuclear S
TCF20 seq:	-0.23156	-1.16524	-0.54269	-1.48776	-0.23627	-0.20791	-0.038	-0.52959	p17855	Transcriptic	4	-0.55488		
TCF20 seq:	0.439215	0.336058	-2.37761	-0.5203	-0.05443	0.967761	0.007985	-0.04673	p06223	Transcriptic	4	-0.15601		
TCF20 seq:	-0.05781	-0.40057	-0.57822	-0.26476	-0.25296	-0.19427	-0.44405	-0.43716	p15321	Transcriptic	4	-0.32873		
TCF20 pS8	-0.42448	-0.92296	-0.40862	-0.32425	-0.58853	-0.21645	-0.43378	-0.63298	p03338	Transcriptic	4	-0.49401		
TCF20 pS1	-0.48442	0.674779	-1.61158	-1.52622	-1.19834	-0.43298	-0.7051	-0.70446	p13578	Transcriptic	4	-0.74854		
TCF20 pS1	-2.94216	-8.92576	-3.87357	-1.19683	-0.68801	-0.39765	-3.96145	-1.14366	p05898	Transcriptic	4	-2.89113		
TCF12 seq:	-0.07009	-0.70515	0.028132	-1.09173	-1.34791	-0.22059	-0.25513	-0.79223	p16088	Transcriptic	4	-0.55684		
TCEB1 seq:	1.885709	-0.75167	-0.07267	-1.69543	0.158851	-0.19318	-3.707	-0.65477	p01870	Transcriptic	4	-0.62877		Hypoxic and oxygen homeostasis regulation of HIF-1-alpha; HIF-2-alpha transcription factor network;
TCEA1 seq:	-0.79072	-0.82076	-1.53548	-0.96066	0.782895	0.333778	0.325086	0.218052	p09722	Transcriptic	4	-0.30598		
TCEA1 seq:	-0.99938	-0.54835	-2.13885	-1.64459	0.852033	0.687307	-0.28902	-0.17343	p09798	Transcriptic	4	-0.53179		
TBX3 seq: 4	-0.16548	-0.66171	-0.16652	-0.39307	-0.62334	-0.07188	1.846208	-7.82958	p19552	T-box trans	4	-1.00817		
TBX3 pS68	-0.10721	-1.11014	-0.3214	-0.3896	0	0	0	0	p01844	T-box trans	4	-0.24104		
TBX3 pS40	-0.31099	-1.01101	-0.31423	-0.44569	0	0	0	0	p13639	T-box trans	4	-0.26024		
TBCA seq: 5	-1.89724	-1.21206	-1.55172	-2.46081	-0.20541	0.436971	-0.33523	0.343831	p12698	Tubulin-spe	4	-0.86021		
TBC1D9B p	-0.30312	-0.0083	-0.1451	-0.23304	0.004244	-0.1519	-0.18597	-0.24348	p13374	TBC1 doma	4	-0.15833		
TBC1D9B p	-0.10765	-0.2607	-0.63251	-0.31471	-0.03531	0.190967	0.0154	-0.04806	p13375	TBC1 doma	4	-0.14907		
TBC1D5 se	-0.58784	-0.11007	-0.70335	-0.53514	-0.21222	-0.25571	-0.52532	-0.49811	p17780	TBC1 doma	4	-0.42847		
TBC1D5 pS	0.139771	-0.57337	-0.35407	-0.21061	-1.68754	-2.01653	-1.8007	-1.22769	p15290	TBC1 doma	4	-0.96634		
TBC1D5 pS	-0.54775	-0.09803	-0.63069	-0.52258	-0.21817	-0.21023	-0.48149	-0.42277	p17781	TBC1 doma	4	-0.39146		
TBC1D5 pS	-1.23111	-0.63391	-0.72058	-1.12158	-0.93959	-1.25978	-0.9455	-0.91194	p09532	TBC1 doma	4	-0.9705		
TBC1D4 sec	-0.65862	0.264459	-0.42006	-0.19718	0.108946	0.267047	-0.07804	0.140622	p19003	TBC1 doma	4	-0.0716		Insulin-mediated glucose transport; Class I PI3K signaling events mediated by Akt;
TBC1D4 sec	-0.26666	-0.70193	-1.15368	-1.28801	-0.84632	-0.84634	-1.19306	-1.61684	p14883	TBC1 doma	4	-0.9891		Insulin-mediated glucose transport; Class I PI3K signaling events mediated by Akt;
TBC1D4 pS	-1.38122	-1.5177	-1.44594	-1.48885	-0.55083	-1.08202	-1.14234	-1.02713	p19806	TBC1 doma	4	-1.2045		Insulin-mediated glucose transport; Class I PI3K signaling events mediated by Akt;
TBC1D4 pS	-1.08301	-1.61195	-1.16961	-1.54329	-0.19621	-0.67757	-0.76346	-0.4352	p19807	TBC1 doma	4	-0.93504		Insulin-mediated glucose transport; Class I PI3K signaling events mediated by Akt;
TBC1D4 pS	-0.25409	-0.4483	0.002329	-0.1631	-0.15512	-0.40102	-0.51376	-0.22119	p11677	TBC1 doma	4	-0.26928		PAKinh (PF Insulin-mediated glucose transport; Class I PI3K signaling events mediated by Akt;
TBC1D4 pS	-0.08178	-0.23734	-0.05166	0.132995	-0.31688	-0.53931	-0.51349	-0.28799	p15345	TBC1 doma	4	-0.23693		Insulin-mediated glucose transport; Class I PI3K signaling events mediated by Akt;
TBC1D4 pS	-1.13037	-1.53511	-1.15365	-1.38168	-1.39273	-1.43254	-1.12985	-1.06188	p04941	TBC1 doma	4	-1.27723		Insulin-mediated glucose transport; Class I PI3K signaling events mediated by Akt;
TBC1D4 pS	-0.40804	-0.9069	-0.85108	-0.92497	-0.38884	-0.45857	-0.43591	-0.1548	p14884	TBC1 doma	4	-0.56614		Insulin-mediated glucose transport; Class I PI3K signaling events mediated by Akt;
TBC1D22B	-0.19313	-0.59739	-0.24219	-1.07616	-0.43158	-0.73874	-0.53791	-0.96028	p08006	TBC1 doma	4	-0.59717		
TBC1D15 se	0.355801	-1.51408	-0.43146	-0.14282	0.153284	-0.07026	-0.12475	0.079194	p13317	TBC1 doma	4	-0.21189		
TBC1D15 p	0.318424	-1.50152	-0.45147	-0.15542	-0.15799	-1.47038	-1.66628	0.793572	p13318	TBC1 doma	4	-0.53638		
TBC1D14 p	-0.52591	-0.94396	-1.24214	-1.04734	-2.73898	-2.14644	-1.34818	-2.56075	p17361	TBC1 doma	4	-1.56921		
TBC1D10B	0.537028	-0.23272	0.230325	0.240532	-0.10963	-2.96952	-0.95924	-0.6961	p07597	TBC1 doma	4	-0.49492		
TBC1D10B	-0.90435	-2.16321	-1.02881	-1.1599	-0.45695	-0.4056	-0.36632	-1.43126	p14823	TBC1 doma	4	-0.98955		
TBC1D1 sec	-0.30204	-0.23951	-0.45203	-0.52576	-0.63518	-2.83294	-0.5961	-1.88619	p00320	TBC1 doma	4	-0.93372		
TBC1D1 sec	-0.28487	-0.67198	-0.62952	-0.93408	0	0	0	0	p20016	TBC1 doma	4	-0.31506	AMPKA1; Akt1;	
TBC1D1 sec	-0.44998	-0.56939	-0.60008	-0.75244	0	0	0	0	p15297	TBC1 doma	4	-0.29649		
TAT pS448	-0.83339	-0.79063	-0.67281	-1.34697	0	0	0	0	p13831	Tyrosine am	4	-0.45548		FOXA2 and FOXA3 transcription factor networks;
TAT pS448	-0.75715	-0.6355	-0.60661	-1.36238	0	0	0	0	p13832	Tyrosine am	4	-0.42021		FOXA2 and FOXA3 transcription factor networks;
TARDBP sec	-0.19793	-0.15788	-1.09365	-0.4476	-1.01999	-0.44612	-0.89831	-1.0076	p16757	TAR DNA-bi	4	-0.65864		
TANK seq: 1	-0.52297	-0.82498	-0.66051	-0.76031	-0.0335	0.324444	-0.41945	-0.04894	p05212	TRAF family	4	-0.36828		GO:0007249; P:I-kappaB kinase/NF-kappaB signaling; GO:0007165; P:signal transduction;
TANK pS12	-0.42564	-0.68065	-0.32077	-0.79511	0.069739	0.438045	0.300696	0.092167	p05213	TRAF family	4	-0.16519		GO:0007249; P:I-kappaB kinase/NF-kappaB signaling; GO:0007165; P:signal transduction;
TANC2 seq:	-1.1093	-1.10758	-1.01196	-2.06502	0	0	0	0	p03643	Protein TAN	4	-0.66173		ROCKinh1 (H-1152);
TANC2 seq:	-0.40097	-0.43737	0.099514	-0.66939	-0.41885	-0.1219	-0.92263	-0.41954	p04216	Protein TAN	4	-0.41139		
TANC2 seq:	0.33994	0.151492	0.01615	0.208814	-0.76592	-0.35208	-0.1078	-0.33737	p00153	Protein TAN	4	-0.10585		
TANC2 pS4	-0.11203	-0.31465	-0.05048	-0.12304	-0.49965	-0.07966	0.097021	-1.14962	p08551	Protein TAN	4	-0.27901		
TANC2 pS1	0.069502	-0.0349	-0.13998	-0.7247	-0.20972	-0.18401	-0.02759	-0.28747	p03641	Protein TAN	4	-0.19236		
TANC2 pS1	-0.43828	-0.42315	0.004663	-0.83031	0	0	0	0	p04217	Protein TAN	4	-0.21089		
TANC2 pS1	-1.02679	-0.69339	-0.20271	-2.48791	0	0	0	0	p00150	Protein TAN	4	-0.55135		
TANC2 pS1	-0.14224	0.151927	0.006022	-0.12724	-0.47755	-0.26673	-0.74464	-0.56022	p19541	Protein TAN	4	-0.27008		
TANC1 seq:	-0.38034	-0.67115	-0.19894	-0.64175	-0.15363	0.50795	0.222236	0.55005	p14700	Protein TAN	4	-0.0957		
TANC1 pS6	-0.34388	-0.95924	0.150364	0.179831	-0.29029	-0.48623	-0.35816	-0.17149	p14990	Protein TAN	4	-0.28489		
TAGLN3 sec	0	0	0	0	-0.71709	-0.55596	-0.87735	-0.50768	p16261	Transgelin-	4	-0.33226		
TAGLN2 sec	-0.29078	0.200957	-0.68511	-0.56656	-0.37641	0.112386	0.882369	0.100551	p08249	Transgelin-	4	-0.07782		
TAGLN2 sec	-1.25655	0.34412	-1.3761	-1.93327	0.994789	0.313107	0.307905	0.129165	p17344	Transgelin-	4	-0.3096		
TAF15 pM9	-0.10632	-0.52991	-0.09754	-0.0193	-0.1208	-0.93933	-0.5386	-0.58569	p07824	TATA-bindir	4	-0.36719		
TACC3 pS5	-0.18404	-1.65711	-0.64257	-0.73353	-0.30803	-0.82053	-0.40646	-0.49173	p12641	Transformir	4	-0.6555	AurA;	Aurora A signaling; Integrin-linked kinase signaling;
TAB2 seq: 5	0.991251	-0.32927	0.190922	0.273735	0.374248	-0.33848	-0.47011	-0.52895	p12916	TGF-beta-ac	4	0.020418		p38 MAPK GO:0070423; P:nucleotide-binding oligomerization domain containing signaling pathway;
SYTL4 pS21	0.229863	0.212947	-0.26653	-0.47557	-1.04014	-1.50152	-1.02621	-0.71255	p07512	Synaptotag	4	-0.57246		
SYTL1 seq:	0.995626	0.984016	0.720379	1.131841	0.490668	-0.40616	-0.59794	-6.34273	p09998	Synaptotag	4	-0.37804		
SYNRG seq:	-0.15386	-0.59794	-0.10061	-0.09336	0.140245	-0.41808	-0.16898	-0.52206	p17039	Synergin ga	4	-0.23933		
SYNRG seq:	-1.119	-0.799	-1.04298	-1.03696	-1.21611	-0.57141	-0.98135	-1.11954	p10711	Synergin ga	4	-0.98579		
SYNRG seq:	-1.75484	-9.86684	-0.22219	-1.60133	-0.21259	-0.17222	-0.14458	0.055565	p14561	Synergin ga	4	-1.73988		
SYNRG seq:	-0.15062	-0.15566	-0.40568	-0.39655	-0.08412	0.024826	0.022712	0.176676	p08304	Synergin ga	4	-0.12105		
SYNRG seq:	-0.46155	-0.20499	-0.86135	-0.45596	-0.37489	-0.62871	-0.66709	-0.47886	p05044	Synergin ga	4	-0.51668		Up with IGF - down with PI3Kinh2 (PI-103); Up with EGF - down with PI3Kinh2 (PI-103); PI3Kinh2 (PI-103);
SYNRG pS9	-0.618	-0.52541	-0.30764	0.024656	-0.58269	-0.57043	-0.45461	-0.30428	p13265	Synergin ga	4	-0.4173		
SYNRG pS9	-0.96627	-0.8374	-0.39574	-0.67364	-1.2346	-0.76942	-1.22891	-1.52881	p10710	Synergin ga	4	-0.95435		
SYNRG pS7	0.024491	0.187274	-0.33138	-0.19481	-1.32004	0.30711	-0.96054	-0.38693	p08305	Synergin ga	4	-0.33435		
SYNRG pS1	0.38996	-3.26409	-0.73237	-1.29473	-4.45309	-0.94212	0.499662	-0.32992	p03778	Synergin ga	4	-1.26584		
SYNRG pS1	-0.27925	-0.00532	-2.51558	-0.49825	-0.25871	-0.96248	-0.79219	-0.83922	p05045	Synergin ga	4	-0.76888		Up with IGF - down with PI3Kinh2 (PI-103); Up with EGF - down with PI3Kinh2 (PI-103); PI3Kinh2 (PI-103);
SYMPK seq:	0.20537	-0.17172	0.166672	-0.11696	-0.21634	-0.12189	-0.14822	-0.21187	p04450	Symplekin	4	-0.07687		
SYAP1 seq:	0.510228	0.403167	-0.72888	-1.30694	-2.08893	-0.09745	-0.23901	2.026145	p02288	Synapse-as	4	-0.19021		
SVIL seq: 96	-0.59114	-1.37654	-0.36097	-1.34237	-0.46946	-1.17526	-0.68903	-0.55209	p18285	Supervillin	4	-0.81961		Coregulation of Androgen receptor activity;
SVIL pS968	-0.11846	-0.37096	0.045889	-0.68128	-0.02131	-0.78175	-0.58923	-0.17169	p00343	Supervillin	4	-0.3361		Coregulation of Androgen receptor activity;
SVIL pS968	-0.16901	-0.36912	0.000708	-0.66483	0.12628	-0.45054	-0.46973	-0.08327	p00344	Supervillin	4	-0.25994		Coregulation of Androgen receptor activity;
SVIL pS914	-0.05551	-0.35991	-0.00667	-0.94841	-1.18157	-1.57605	-0.47907	-0.57946	p16442	Supervillin	4	-0.64833		Coregulation of Androgen receptor activity;
SUPT5H sec	0.019123	0.066147	-0.14293	0.084199	-0.67578	-0.46827	-0.54436	-0.59539	p13655	Transcriptic	4	-0.28216		
SUPT5H sec	0.183924	0.190931	0.142357	0.28377	-0.49251	-0.40998	-0.48747	-0.65816	p13654	Transcriptic	4	-0.15589		
SUPT5H sec	-0.55233	-0.73515	-0.20968	-0.42266	-0.34747	-0.43921	-0.95233	-0.53308	p13656	Transcriptic	4	-0.52399		
SUGP1 seq:	1.315612	1.100954	0.148225	-0.31494	-1.07357	-1.4385	-0.95046	-0.31658	p19198	SURP and G	4	-0.19116		
SUCO seq: 1	0.318193	-0.23109	0.23407	0.299954	-0.50628	-0.7216	-0.21706	-0.58766	p05682	SUN domai	4	-0.17643		
SUCO pS10	-0.08519	-0.14569	-0.4513	-0.32424	-0.24688	-0.24421	-0.21975	-0.56458	p05366	SUN domai	4	-0.28523		
STXBP5 seq	-0.31217	-0.53218	-0.63977	-0.58747	0	0	0	0	p06823	Syntaxin-bi	4	-0.25895		
STX17 seq:	-0.14605	-0.87122	-0.17192	-0.01341	-0.09699	-0.71635	-0.35374	0.064712	p10490	Syntaxin-17	4	-0.28812		
STX17 seq:	-0.23991	-0.74456	-0.08257	0.124664	-0.02859	-0.5321	-0.33522	-0.03405	p10489	Syntaxin-17	4	-0.23404		
STUB1 pS1	-1.08965	-1.92174	-0.99092	-0.31694	-0.42258	-0.47214	-0.82549	-0.34413	p11836	E3 ubiquitir	4	-0.79795		Alpha-synu GO:0030512; P:negative regulation of transforming growth factor beta receptor signaling
STUB1 pS1	-0.97455	-1.31615	-0.95445	-0.24177	-0.38273	-0.50761	-0.77725	-0.36881	p11835	E3 ubiquitir	4	-0.69042		Alpha-synu GO:0030512; P:negative regulation of transforming growth factor beta receptor signaling
STRN3 seq:	-2.76778	-11.3226	-2.00023	-0.86437	0	0	0	0	p03902	Striatin-3	4	-2.11937		GO:0033147; P:negative regulation of intracellular estrogen receptor signaling pathway;
STRN seq: 3	-0.07532	-0.89606	0.257266	0.104759	0.143461	-0.11613	0.359896	0.188856	p18121	Striatin	4	-0.00416		Plasma mer GO:0016055; P:Wnt signaling pathway;
STMN3 seq	-0.60261	-0.99187	-0.76321	-0.30385	0.104939	-0.14428	-0.07595	-0.21535	p20283	Stathmin-3	4	-0.37402		
STMN2 seq	0.074307	0.119706	0.073406	0.181629	-0.14163	-0.32958	-0.22268	-0.31871	p20284	Stathmin-2	4	-0.07044		GO:1990090; P:cellular response to nerve growth factor stimulus;
STMN1 pS3	0.193532	1.365993	0.063785	-0.17791	-1.12564	-0.79641	-0.55945	-1.0863	p17092	Stathmin	4	-0.2653	PKCB; PAK1	PAKinh (PF Signaling m GO:0035556; P:intracellular signal transduction; GO:0007165; P:signal transduction;
STMN1 pS3	0.144589	0.859219	-0.26275	-0.42698	-0.73409	-0.3332	-0.43043	-0.56702	p05422	Stathmin	4	-0.21883	PKCB; PAK1	PAKinh (PF Signaling m GO:0035556; P:intracellular signal transduction; GO:0007165; P:signal transduction;
STMN1 pS3	0.132474	0.558552	0.063703	-0.03991	-0.65142	-0.47721	-0.44	-0.78516	p17091	Stathmin	4	-0.20487	PKCB; PAK1	PAKinh (PF Signaling m GO:0035556; P:intracellular signal transduction; GO:0007165; P:signal transduction;
STK25 pT1	-0.44543	-0.38746	-0.73823	-0.73652	-1.71184	-0.25602	-0.0201	-0.83335	p06995	Serine/threo	4	-0.64112	MST3;	GO:0023014; P:signal transduction by phosphorylation; GO:0007165; P:signal transducti
STK25 pT1	0.055335	-0.44313	-0.05555	0.01603	-0.5533	-0.54856	-0.41072	-0.77099	p12291	Serine/threo	4	-0.33886		p70S6Kinh2 (DG2); CAI GO:0023014; P:signal transduction by phosphorylation; GO:0007165; P:signal transducti
STK10 pS4	-0.13834	-2.73862	-0.81348	-1.16922	-1.81934	-1.57161	-1.83019	-1.82269	p07753	Serine/threo	4	-1.48794		GO:0023014; P:signal transduction by phosphorylation;
STIP1 seq: 6	-0.4528	0.39301	-0.76144	-0.38115	0.717177	-0.44294	-0.39286	-0.23609	p01975	Stress-indu	4	-0.19464		
STIM2 seq:	-0.19881	0.50609	-1.76169	-0.63626	0.055163	-0.39311	-1.28048	-0.46064	p14579	Stromal inte	4	-0.52122		
STIM2 pS52	-0.06897	0.02977	0.148671	0.090556	-0.22395	-0.129	0.050277	-0.46667	p05443	Stromal inte	4	-0.07117		
STIM1 seq:	0.270377	0.031732	0.228755	0.134932	-0.53518	-0.04238	-0.07549	-0.34259	p18520	Stromal inte	4	-0.04123		TCR signaling in naïve CD8+ T cells; TCR signaling in naïve CD4+ T cells;
STIM1 pS5	-0.17649	-0.83648	-0.32219	-0.08274	0.399235	-2.44004	-0.67577	-2.23045	p10566	Stromal inte	4	-0.79562		TCR signaling in naïve CD8+ T cells; TCR signaling in naïve CD4+ T cells;
STAU1 seq:	-0.59986	-1.97451	-2.54958	-1.34576	-0.86765	-1.22159	-0.32083	-0.60369	p06042	Double-stra	4	-1.18544		
STARD10 se	-1.04609	-1.22379	-1.18914	-1.24415	0	0	0	0	p12299	PCTP-like pr	4	-0.5879		
STARD10 se	-0.30019	-0.34105	-0.43648	-0.61095	0	0	0	0	p17089	PCTP-like pr	4	-0.21108		
STARD10 se	-0.35987	-0.26374	-0.01775	-0.72812	-0.40818	-1.06889	-0.73563	-0.41216	p19192	PCTP-like pr	4	-0.49929		
ST13P4 seq	-0.20403	-0.26354	-0.38701	-0.51679	0.330735	-0.13304	0.272793	0.25449	p01846	Putative pro	4	-0.0808		
SSH3 seq: 6	0.079636	0.160367	0.135575	-0.0269	-1.06903	0.228082	-0.48725	-8.75371	p02291	Protein pho	4	-1.21665		
SSH3 seq: 5	-1.0084	-1.84754	-0.55718	-2.34024	0	0	0	0	p04077	Protein pho	4	-0.71917		
SSFA2 seq:	-0.22373	-0.37611	-0.32153	-0.7653	0.000239	0.030544	-0.18644	-0.36311	p12583	Sperm-spec	4	-0.27568		
SSFA2 pS33	-0.14142	-0.26717	0.051098	-0.57807	-0.24238	-0.46903	-0.00074	-0.52788	p14012	Sperm-spec	4	-0.27195		
SSFA2 pM1	0.134112	-1.97597	0.238612	-0.20167	-0.65138	-0.08805	-1.22183	-3.02077	p09945	Sperm-spec	4	-0.84837		
SSBP3 pT36	0.032618	-0.15225	0.291379	0.187684	-2.00235	-0.62556	-0.07172	-0.72593	p09118	Single-stran	4	-0.38327		
SRSF9 seq:	-0.07146	-0.51456	0.038622	-0.02284	0.142467	-0.1859	-0.10871	0.203002	p15224	Serine/argin	4	-0.06492		
SRSF4 seq:	-0.59302	-0.69359	-0.20237	-0.44639	-0.13741	-0.37986	-0.14145	-0.75809	p04786	Serine/argin	4	-0.41902		
SRSF11 seq	0.07241	-1.21851	-0.50263	-0.29769	0.175722	1.419751	0.532074	0.781176	p17835	Serine/argin	4	0.120288		
SRSF11 seq	-0.51934	-2.3203	-0.91194	0.160911	-0.9073	-0.78275	-1.05615	0.00843	p04072	Serine/argin	4	-0.79105		
SRSF11 pS4	-0.23173	-0.03961	-0.56933	0.011568	0.141097	-0.04538	-0.41598	-0.04639	p02670	Serine/argin	4	-0.14947		
SRSF1 seq:	-0.22136	-0.39977	0.088894	-0.12053	-0.31805	-0.38236	-0.38109	-0.44115	p01287	Serine/argin	4	-0.27193		
SRRT seq: 5	-0.77252	-0.1735	-0.318	-1.42349	-0.47697	-0.22997	0.012004	0.0361	p11593	Serrate RNA	4	-0.41829		AKTinh1 (Akt Inhibitor VIII);
SRRM2 seq	-0.35065	-0.6765	-0.54298	-0.42255	-0.98225	-0.61537	-0.78998	-1.89869	p14652	Serine/argin	4	-0.78487		
SRRM2 seq	-0.29687	-0.47379	-0.27193	-0.29314	-0.86045	-0.69128	-0.81931	-1.00075	p14651	Serine/argin	4	-0.58844		
SRRM2 seq	0.194986	-0.02491	-0.04005	-0.47073	-0.64251	-0.41716	-0.34151	-0.75061	p14876	Serine/argin	4	-0.31156		
SRRM2 seq	0.18729	0.247393	0.345667	0.074864	-0.66137	-0.08726	-0.06531	-0.55304	p06191	Serine/argin	4	-0.06397		
SRRM2 seq	-0.25175	-0.3614	0.009646	0.026213	-0.36362	-0.41271	-0.5602	-0.7646	p09843	Serine/argin	4	-0.3348		
SRRM2 seq	-0.17423	-0.32096	-0.01253	-7.8E-05	-0.41702	-0.43747	-0.49983	-0.69549	p09844	Serine/argin	4	-0.3197		
SRRM2 seq	-0.15332	-0.45879	-0.30328	-0.29803	-0.78727	-0.79858	-0.99975	-1.1149	p09846	Serine/argin	4	-0.61424		
SRRM2 seq	-0.24779	-0.92415	0.214041	-0.24715	-0.53631	-0.54972	-0.58457	-1.34851	p02438	Serine/argin	4	-0.52802		
SRRM2 seq	0.015801	-0.02325	0.041621	-0.30651	-0.51126	-0.36789	-0.2014	-0.87926	p09580	Serine/argin	4	-0.27902		
SRRM2 seq	0.112022	0.170303	0.387446	-0.35096	-0.676	-0.31837	-0.21595	-0.95616	p09579	Serine/argin	4	-0.23096		
SRRM2 seq	-0.23761	-0.32536	-0.33926	-0.14787	-1.10736	0.099877	-0.08793	0.172203	p03962	Serine/argin	4	-0.24666		
SRRM2 seq	-0.26554	-1.13845	1.325377	0.368268	0.022287	-1.76572	0.453302	-0.60674	p03734	Serine/argin	4	-0.2009		
SRRM2 seq	-0.46378	-0.17921	-1.09406	-0.569	-0.26703	0.152357	-0.7216	-0.4243	p05653	Serine/argin	4	-0.44583		
SRRM2 seq	-0.24457	0.136124	-0.68813	-0.3088	-0.18641	0.406289	-0.24434	-0.08712	p05654	Serine/argin	4	-0.15212		
SRRM2 seq	-0.85846	-0.11948	-1.04581	-0.84063	-0.69286	-0.12647	-0.70079	-0.79431	p04053	Serine/argin	4	-0.64735		
SRRM2 seq	-0.183	-0.50994	-0.05101	-0.03251	0.0235	0.065353	-0.12335	-0.13004	p01982	Serine/argin	4	-0.11763		
SRRM2 seq	-0.22716	-0.19011	-0.25487	-0.52481	-0.74946	-1.49723	-0.71534	-1.19286	p14636	Serine/argin	4	-0.66898		
SRRM2 seq	-1.09536	-1.44615	-1.0671	-1.12304	0.38843	-1.83557	0.313808	0.590166	p03583	Serine/argin	4	-0.65935		
SRRM2 pT2	0.27427	-0.07589	0.270988	0.183962	-0.64021	-0.4474	0.045911	-0.1536	p17691	Serine/argin	4	-0.06775		
SRRM2 pS7	0.258159	-0.32875	0.609185	0.24099	-0.20506	-0.66535	0.193084	-0.27796	p06663	Serine/argin	4	-0.02196		
SRRM2 pS2	0.02092	-0.33313	0.677994	0.000159	0.081241	-0.10369	-0.05682	-0.36897	p04961	Serine/argin	4	-0.01029		
SRRM2 pS2	0.143981	-0.33746	-0.33619	0.047264	-0.02714	-0.19591	-0.4161	-0.19812	p19675	Serine/argin	4	-0.16496		
SRRM2 pS1	-0.23864	-0.1934	-0.18975	-0.29249	-0.50262	-0.35222	-0.39658	-0.48504	p20613	Serine/argin	4	-0.33134	CDK1;	
SRRM2 pM	-0.28502	-0.95077	-1.44508	-0.72266	-0.47902	-1.04411	-0.70139	-0.71894	p09845	Serine/argin	4	-0.79337		
SRRM2 pM	-0.62528	-0.6476	-0.37772	-0.27203	-0.74957	-0.32736	-0.73858	-0.89908	p20612	Serine/argin	4	-0.57965	CDK1;	
SRRM2 pM	-0.34699	-0.6927	-0.3135	-0.11526	-0.0295	-0.24841	-0.41912	-0.39862	p10261	Serine/argin	4	-0.32051		
SRRM1 seq	0.055117	-0.77428	0.2212	0.175315	-0.26999	0.0674	0.091095	-0.07807	p19282	Serine/argin	4	-0.06403		
SRRM1 seq	-0.2298	-0.2784	0.163746	0.071853	0.172127	-0.0228	0.10188	-0.02767	p06388	Serine/argin	4	-0.00613		
SRRM1 seq	-0.80239	-1.14899	-1.45577	-0.9505	-1.22464	-1.08729	-1.08913	-2.37547	p15107	Serine/argin	4	-1.26677		
SRRM1 pT8	0.479232	-0.61691	-1.20486	-1.45109	-1.76781	-1.39994	-2.27645	-2.00437	p13273	Serine/argin	4	-1.28027		
SRRM1 pT8	0.005693	-1.02165	0.147296	-0.11753	-0.36707	0.201174	0.216177	-0.11687	p19283	Serine/argin	4	-0.1316		

[illegible]	[illegible]	-0.33036	0.059173	0.059932	0.073482	0.278195	0.076302	0.038279	p07861	Serine/argin	4	0.010148				
[illegible]	[illegible]	-0.31363	0.380235	0.236517	0.032331	-0.14999	-0.02199	-0.29076	p03017	Serine/argin	4	-0.02807				
[illegible]	[illegible]	-0.14163	-0.19043	-0.13453	-0.1154	-0.01958	-0.00816	-0.09826	p14658	Serine/argin	4	-0.10117				
[illegible]	[illegible]	-0.40161	-0.74874	-0.44185	-0.62595	-0.53075	-0.49282	-0.35991	p01918	SRSF protei	4	-0.50686				GO:0035556; P:intracellular signal transduction;
[illegible]	[illegible]	-0.71883	-0.70339	-0.72549	-0.02925	-0.02067	0.150697	0.039205	p01888	Signal recog	4	-0.35537				
[illegible]	[illegible]	-0.37826	-0.88861	-0.77629	0.677817	0.204951	0.223658	0.442995	p13142	Signal recog	4	-0.139				
[illegible]	[illegible]	-0.98682	-0.03058	-0.03862	0.2408	-0.41438	0.020084	0.257768	p09107	SLIT-ROBO	4	-0.13717			Regulation (	GO:0007266; P:Rho protein signal transduction; GO:0051056; P:regulation of small GTPase mediated signal transduction; GO:0007264; P:small GTPase mediated signal transduction;
[illegible]	[illegible]	-1.15196	-1.1523	-0.65272	-0.12204	-0.31632	-0.54271	-0.53028	p13112	SLIT-ROBO	4	-0.64268			Regulation (	GO:0007266; P:Rho protein signal transduction; GO:0051056; P:regulation of small GTPase mediated signal transduction; GO:0007264; P:small GTPase mediated signal transduction;
[illegible]	[illegible]	-0.74452	-0.08117	-0.14406	0.167786	-0.09527	-0.18957	-0.11064	p17006	Sequestoso	4	-0.21827		AKTinh1 (Al	IL1-mediate	GO:0043122; P:regulation of I-kappaB kinase/NF-kappaB signaling; GO:0048011; P:neurotrophin TRK receptor signaling pathway; GO:0035556; P:intracellular signal transduction;
[illegible]	[illegible]	-0.39772	-0.18638	-0.30087	-0.28718	-0.49562	-0.3591	-0.59383	p04095	Sequestoso	4	-0.36668	CDK1;		IL1-mediate	GO:0043122; P:regulation of I-kappaB kinase/NF-kappaB signaling; GO:0048011; P:neurotrophin TRK receptor signaling pathway; GO:0035556; P:intracellular signal transduction;
[illegible]	[illegible]	0.478409	0.718218	-4.06014	0.260914	0.121057	0.317372	-0.04311	p20690	Sequestoso	4	0.002272			IL1-mediate	GO:0043122; P:regulation of I-kappaB kinase/NF-kappaB signaling; GO:0048011; P:neurotrophin TRK receptor signaling pathway; GO:0035556; P:intracellular signal transduction;
[illegible]	[illegible]	-0.58844	-0.38446	-0.47757	-0.55845	-0.01451	0.097	-0.09353	p19740	Spectrin bet	4	-0.28962		ROCKinh2 (	TGF-beta receptor signaling;	
[illegible]	[illegible]	0.317844	-4.77632	-1.03007	-1.12077	-1.13012	0.663036	0.416694	p18643	Spectrin bet	4	-1.03399			TGF-beta receptor signaling;	
[illegible]	[illegible]	-2.85906	0.404304	0.508455	-2.63259	0.588346	0.624947	-8.61407	p12061	Spectrin alp	4	-1.50575		ROCKinh1 (H-1152);		
[illegible]	[illegible]	-1.03004	-0.44928	-0.81281	-0.09457	0.25855	0.103352	-0.153	p03016	Spectrin alp	4	-0.37563				
[illegible]	[illegible]	-0.08321	0.284784	0.05868	-1.17403	-2.82844	-0.81117	-2.46432	p05856	Protein spir	4	-0.83117				
[illegible]	[illegible]	-1.12743	-1.17025	-0.55876	-0.67382	-0.67516	-0.95133	-0.78806	p11267	Msx2-intera	4	-0.79386			Notch-med	GO:0007219; P:Notch signaling pathway;
[illegible]	[illegible]	0.159597	0.081478	-0.1456	-1.20991	-0.11345	-0.17165	-0.60462	p14798	Msx2-intera	4	-0.17698			Notch-med	GO:0007219; P:Notch signaling pathway;
[illegible]	[illegible]	1.023269	-1.04644	-0.24872	-0.83348	-0.32518	-0.81191	-0.79333	p18729	Cytospin-A	4	-0.43538				
[illegible]	[illegible]	-0.51497	-0.4008	0.095856	-0.17461	-0.27841	-0.27379	-0.33671	p06565	Cytospin-A	4	-0.2399				
[illegible]	[illegible]	-0.49863	-0.51577	0.131797	0.078962	-0.20334	-0.23171	-0.01061	p06566	Cytospin-A	4	-0.13677				
[illegible]	[illegible]	-0.87214	-0.3533	-0.64753	-0.99167	-1.15986	-1.18366	-1.1971	p02592	Cytospin-B	4	-0.8377				
[illegible]	[illegible]	-3.03849	-1.00538	-0.83619	-0.92306	-0.87639	-0.31438	-0.78458	p08579	SPATS2-like	4	-1.23355				
[illegible]	[illegible]	0.072882	-1.18181	-1.15305	0.042935	-0.17058	0.328521	0.154536	p16550	SPATS2-like	4	-0.28323				
[illegible]	[illegible]	-0.54152	-0.10914	-0.19276	-0.12234	-0.07017	0.165004	0.081919	p00210	C-Jun-amin	4	-0.10079	CDK2;		Arf6 trafficking events;	
[illegible]	[illegible]	-0.68306	-0.09615	0.097399	-0.06859	-0.77581	-0.26581	-0.3644	p07599	Sperm-asso	4	-0.2254				
[illegible]	[illegible]	-0.61959	-0.07726	0.045183	-0.09734	-0.62933	-0.25298	-0.29602	p07600	Sperm-asso	4	-0.19227				
[illegible]	[illegible]	-0.52567	-0.24439	-0.2973	-0.2456	-0.30944	-0.36308	-0.36977	p06421	Sp110 nucl	4	-0.32524				GO:0007165; P:signal transduction;
[illegible]	[illegible]	-0.48285	-0.21768	-1.71027	0.308618	0.755089	-0.0337	1.041047	p05723	Nuclear aut	4	-0.04237				GO:0048384; P:retinoic acid receptor signaling pathway; GO:0060337; P:type I interferon signaling pathway; GO:0060333; P:interferon-gamma-mediated signaling pathway; GO:0019221; P:cytokine-mediated signaling pathway;
[illegible]	[illegible]	0.277771	0.32819	0.260209	-0.36243	-0.44135	-0.90606	-0.59684	p06967	Transcriptic	4	-0.12229				GO:0071560; P:cellular response to transforming growth factor beta stimulus;
[illegible]	[illegible]	-1.71617	-0.10127	-1.49889	-0.05041	0.073259	-1.31404	-8.0798	p18546	Transcriptic	4	-1.72401				
[illegible]	[illegible]	-0.63152	-0.01669	-0.21211	-0.14578	-0.4176	-0.35487	-0.4027	p19995	Transcriptic	4	-0.29335				
[illegible]	[illegible]	-0.73151	-0.45901	-0.36863	-4.24221	-1.7979	-2.28348	-1.35579	p10209	Transcriptic	4	-1.45532				GO:0017015; P:regulation of transforming growth factor beta receptor signaling pathway; GO:0030513; P:positive regulation of BMP signaling pathway;
[illegible]	[illegible]	-0.84094	-0.50211	-0.41177	-0.26027	-0.31661	-0.54306	-0.48547	p18513	Ankyrin rep	4	-0.50197				
[illegible]	[illegible]	-0.16897	-0.50143	-0.30476	-3.97162	-2.01171	-0.58061	-2.33779	p18950	Sorbin and	4	-1.30438				
[illegible]	[illegible]	0.05309	-0.46473	-0.19383	0	0	0	0	p18951	Sorbin and	4	-0.1281				
[illegible]	[illegible]	-0.49752	-0.27181	-0.26089	-1.61154	-0.42315	-1.20268	-4.75973	p05804	Sorbin and	4	-1.1917				
[illegible]	[illegible]	-0.7046	-0.46534	-0.20903	0.227923	0.155958	0.13451	-0.067	p07213	Sorbin and	4	-0.11754				
[illegible]	[illegible]	-0.74852	-0.11042	0.056154	-0.46141	-0.46411	-0.65508	-0.57408	p01952	Protein SON	4	-0.37316				
[illegible]	[illegible]	0.139855	0.51012	0.427134	0.373054	-2.0256	0.025309	-0.81128	p00553	Superoxide	4	-0.12254			FOXA1 tran	GO:0045859; P:regulation of protein kinase activity; GO:0000187; P:activation of MAPK activity;
[illegible]	[illegible]	1.286612	0.49546	0.322625	-2.93955	-1.23073	-0.31237	-0.73316	p07441	Sorting nexi	4	-0.26105		ROCKinh1 (	PAR1-mediated thrombin signaling events;	
[illegible]	[illegible]	-0.48107	-0.30029	-0.27985	-0.20471	-0.03151	-0.24758	0.05322	p16327	Beta-1-synt	4	-0.25103				
[illegible]	[illegible]	-0.37457	0.384104	0.179559	-0.07657	-0.19244	-0.10332	-0.25084	p05339	U2 small nu	4	0.00655		ROCKinh1 (H-1152);		
[illegible]	[illegible]	-0.13047	-0.0716	-0.16339	-0.33524	-0.21317	-0.20328	-0.25589	p20084	SNARE-assc	4	-0.19447	CDK2;	AKTinh2 (MK-2206); AKTinh1 (Akt Inhibitor VIII);		
[illegible]	[illegible]	-1.15887	-0.54351	-0.35603	0.056573	-0.13305	-0.54922	-0.39925	p20085	SNARE-assc	4	-0.49502	CDK2;	AKTinh2 (MK-2206); AKTinh1 (Akt Inhibitor VIII);		
[illegible]	[illegible]	-0.31719	-0.46981	-0.55393	-0.23245	-0.01669	0.293757	0.390811	p04203	Smoothelin	4	-0.15604				
[illegible]	[illegible]	-3.29533	0.310851	0.328689	0	0	0	0	p11395	Smoothelin	4	-0.29648				
[illegible]	[illegible]	-0.90463	-0.47165	-0.49173	-0.26985	-0.55663	-0.20371	-0.30854	p13769	Smoothelin	4	-0.45273				
[illegible]	[illegible]	-0.44708	-0.26068	-0.32797	-0.0243	-0.24083	-1.06675	0.340734	p17312	Protein SM(	4	-0.34383				
[illegible]	[illegible]	-0.98703	-0.71915	-0.73632	-0.67158	-0.51311	-0.46362	-0.66917	p20603	Protein SM(	4	-0.72239		AKTinh1 (Akt Inhibitor VIII);		
[illegible]	[illegible]	-0.80113	-0.35348	-0.3874	-0.57289	-0.72149	-0.69563	-0.43804	p02237	Telomerase-	4	-0.57473		AKTinh1 (Al	Regulation of Telomerase;	
[illegible]	[illegible]	-0.6742	-0.34198	-0.80724	-0.35691	-0.40071	-1.14054	-0.66291	p09479	Serine/thre	4	-0.5816				GO:0046854; P:phosphatidylinositol phosphorylation;
[illegible]	[illegible]	-0.35999	-0.08055	-0.12482	0.090455	-0.19625	-0.42787	-0.18022	p06761	Structural n	4	-0.16593			Aurora B signaling;	
[illegible]	[illegible]	-0.23411	-0.97049	-0.60646	1.097668	0.269903	0.461368	0.828781	p07227	SWI/SNF co	4	0.066038			Regulation (	GO:0008286; P:insulin receptor signaling pathway;
[illegible]	[illegible]	-0.08083	0.523365	-0.5992	0.554166	-8.36698	2.964009	0.943623	p16000	SWI/SNF-rel	4	-0.65398				
[illegible]	[illegible]	-0.49672	0.373365	0.260605	-0.06509	-0.60296	-0.05448	0.143427	p12372	Stromal me	4	-0.04332				
[illegible]	[illegible]	-0.56903	-1.06852	0.019629	-0.18495	0.007838	0.006441	-0.11598	p13932	Small acidic	4	-0.36001				
[illegible]	[illegible]	-0.34001	-0.19335	-0.21752	-0.55289	-0.48455	-0.4362	-0.97282	p10616	Protein SLX	4	-0.40634				
[illegible]	[illegible]	-0.1787	-0.12807	0.136248	-0.68938	-0.52487	-0.69732	-0.82852	p13663	SAFB-like tra	4	-0.32644		PAKinh (PF3758309); p70S6Kinh2 (DG2);		
[illegible]	[illegible]	-0.03518	-0.62435	-0.00278	-0.07259	-0.05578	-0.70234	-0.01349	p13664	SAFB-like tra	4	-0.17582		PAKinh (PF3758309); ROCKinh2 (Y-27632); p70S6Kinh2 (DG2);		
[illegible]	[illegible]	0.347233	-1.13193	0.144918	0.065161	0.044642	-0.85934	0.159146	p13665	SAFB-like tra	4	-0.11579		PAKinh (PF3758309); p70S6Kinh2 (DG2);		
[illegible]	[illegible]	-0.78505	-0.59036	-0.17963	-0.01374	-0.29587	-0.13236	0.099781	p04640	SAFB-like tra	4	-0.29366		PAKinh (PF3758309); p70S6Kinh2 (DG2);		
[illegible]	[illegible]	-0.51383	-0.34478	-0.05667	0.124663	0.073496	-0.17316	-0.10135	p04641	SAFB-like tra	4	-0.14443		PAKinh (PF3758309); p70S6Kinh2 (DG2);		

[illegible]

PKP3 pS12	-0.53516	-1.68376	0.376437	0.313802	-0.27484	-0.74002	-0.27325	-0.70571	p06181	Plakophilin-	4	-0.44031		
PKP2 pS32	0.327997	-1.16905	0.64435	0.21936	-0.08021	-0.54073	0.030557	0.176404	p20528	Plakophilin-	4	-0.04892		
PKN2 seq:	-0.28083	-1.13948	-0.04016	-0.13459	0.259437	-0.20993	0.220004	-0.10912	p02074	Serine/thre	4	-0.17933		RhoA signal GO:0007165; P:signal transduction;
PKN2 pS36	-0.53352	-1.28924	-0.08257	-0.23869	0.032565	0.046884	0.168925	0.011616	p19300	Serine/thre	4	-0.2355		RhoA signal GO:0007165; P:signal transduction;
PKN1 seq:	-0.532	-1.07432	0.218015	-0.26406	-0.55258	-0.69827	0.282138	-0.16135	p02076	Serine/thre	4	-0.3478		Signaling m GO:0007165; P:signal transduction;
PKM seq: 4	-0.66752	-0.68879	-1.13123	-1.00339	1.134063	0.436056	0.778975	0.408416	p08852	Pyruvate kir	4	-0.09168		HIF-1-alpha transcription factor network;
PKM seq: 2	0.866163	1.347497	-1.34	-1.55893	-0.79387	0.028139	-0.31842	0.225759	p13134	Pyruvate kir	4	-0.19296		HIF-1-alpha transcription factor network;
PKM pM29	-0.19474	-0.08196	-0.67927	-0.95474	-1.25934	1.011371	0.662947	0.968789	p07448	Pyruvate kir	4	-0.06587		HIF-1-alpha transcription factor network;
PIR seq: 85	-0.55619	0.179831	-2.98802	-0.59481	-0.35513	-0.37383	-0.34368	-0.48008	p09979	Pirin	4	-0.68899		
PIKFYVE pS	-0.13024	-0.41332	-0.12308	-0.02695	-0.00658	-0.35724	-0.0746	-0.21964	p06263	1-phosphat	4	-0.16895	Akt1;	GO:0036092; P:phosphatidylinositol-3-phosphate biosynthetic process; GO:0046854; P:phosphatidylinositol phosphorylation; GO:0006661; P:phosphatidylinositol biosynthetic p
PIGK pS142	-0.06514	-0.13905	0.024581	0.302595	0.847117	-0.39158	-0.7105	-6.97586	p13867	GPI-anchor	4	-0.88848		
PI4K2A pS4	-0.91901	-0.90008	-2.12916	-0.98373	0.050217	-0.28731	-0.84658	-0.26325	p03718	Phosphatid	4	-0.78486		Canonical W GO:0046854; P:phosphatidylinositol phosphorylation; GO:0006661; P:phosphatidylinositol biosynthetic process;
PHLDB2 se	0	0	0	0	-0.00542	-0.85471	-0.69441	0.012952	p20530	Pleckstrin h	4	-0.1927		
PHLDB2 se	-1.10059	-6.87763	-0.24665	1.077217	0.186343	0.331505	-0.25414	0.493637	p09628	Pleckstrin h	4	-0.79879		
PHLDB2 pS	-0.90562	-0.19022	-0.57573	-0.70471	0.293842	0.048622	0.162045	0.401464	p06642	Pleckstrin h	4	-0.18379		
PHLDB2 pS	-0.97142	-8.90925	-0.24648	-1.18831	0.108185	0.171552	0.081876	0.049166	p02197	Pleckstrin h	4	-1.36308		
PHLDB2 pS	0.479915	-8.4705	-0.971	0.176197	0.23755	-0.14092	-0.38348	0.296878	p12855	Pleckstrin h	4	-1.09692		
PHLDB1 pS	-0.35647	-0.91709	-0.22411	0.038947	-0.08974	-0.39309	-0.187	-0.2049	p12906	Pleckstrin h	4	-0.29168		
PHLDB1 pS	0.627948	1.14026	-0.05886	-2.0968	-0.12114	-0.07689	0.191083	0.075412	p07628	Pleckstrin h	4	-0.03987		
PHLDB1 pS	-0.9487	-0.66982	-0.53166	-0.85854	-0.28607	-0.21559	-0.39082	-0.28959	p01130	Pleckstrin h	4	-0.52385		
PHLDA2 se	-9.36778	0.893617	-0.73691	-0.98455	-0.88448	-0.88115	-0.87153	-0.81841	p03070	Pleckstrin h	4	-1.7064		
PHLDA2 se	-0.20494	0.038653	-0.34525	-0.32031	-0.7852	-0.79446	-0.80436	-0.69679	p03069	Pleckstrin h	4	-0.48908		
PHLDA2 pS	-0.85139	-0.70943	-0.9789	-0.98781	-0.53085	-0.61321	-0.79295	-0.68574	p10761	Pleckstrin h	4	-0.76878		
PHKA2 seq:	-0.3183	0.635157	-1.1363	-1.80316	0.196864	-2.69484	-2.1645	1.413525	p18742	Phosphoryl	4	-0.73395		
PHIP seq: 1	-0.26583	-0.49165	-0.54567	-0.58117	-2.52208	-1.01393	-0.08669	-0.11281	p03088	PH-interact	4	-0.70248		GO:0043568; P:positive regulation of insulin-like growth factor receptor signaling pathway; GO:0008286; P:insulin receptor signaling pathway;
PHIP pS131	1.109806	2.156871	-3.65894	0.952093	-7.35793	1.485126	0.068209	0.477542	p19747	PH-interact	4	-0.5959		Up with IGF - down with GO:0043568; P:positive regulation of insulin-like growth factor receptor signaling pathway; GO:0008286; P:insulin receptor signaling pathway;
PHF8 seq: 1	-0.57193	-0.27556	-1.32124	-0.67823	-1.33459	0.22589	-2.57814	-0.03834	p06834	Histone lysi	4	-0.82152		
PHF8 pS88	0.242148	0.011144	0.53364	0.231223	-1.26314	-0.56952	0.275051	-5.8207	p09168	Histone lysi	4	-0.79502		
PHF8 pS12	-0.18122	-0.80335	0.190734	-0.11421	-0.60036	-1.21367	-0.73709	-1.42083	p02784	Histone lysi	4	-0.61	CDK1;	
PHF6 seq: 1	0.557885	1.117737	0.072153	-1.2929	-1.36314	-0.51069	-0.62027	-0.7455	p12513	PHD finger	4	-0.34809	CDK2;	
PHF2 pS88	-0.24662	-0.41675	0.035523	-0.23208	-0.08027	-0.26765	-0.55057	-0.81115	p18298	Lysine-spec	4	-0.3212		ERKinh1 (ERK inhbitor Calbiochem); EGFRinh1 (PD-168393); AKTinh1 (Akt Inhibitor VIII);
PHF2 pS70	-0.33757	-0.47279	-0.40448	-0.45521	-0.14513	-0.54128	-0.54007	-0.33789	p18540	Lysine-spec	4	-0.4043		
PHB seq: 1	0.095123	0.22041	-0.3204	0.0329	-0.70154	-0.31958	-0.278	-0.48012	p17782	Prohibitin	4	-0.2189		GO:0060766; P:negative regulation of androgen receptor signaling pathway; GO:0007165; P:signal transduction;
PHACTR2 s	0.795984	1.029112	0.542281	0.504164	-3.14931	-1.86995	-1.23941	-1.34158	p12917	Phosphatas	4	-0.59109		
PGRMC1 p	0.201189	-0.05072	0.204236	-0.02869	-0.09353	-0.26771	-0.18129	-0.52393	p13537	Membrane-	4	-0.09255		
PGM3 pM6	-0.18644	-0.22534	-0.03957	-0.02637	0.311015	-0.05267	0.106199	0.356182	p03513	Phosphoac	4	0.030375		
PGM2L1 pl	-0.17004	-0.64681	-0.04184	0.099513	0.060544	-0.34119	-0.15416	-0.6069	p19277	Glucose 1,6	4	-0.22511		
PGK1 seq:	0.23497	0.152212	0.249831	0.526455	-0.07066	-0.93086	-0.89285	-0.73243	p12357	Phosphogly	4	-0.18292		HIF-2-alpha transcription factor network; HIF-1-alpha transcription factor network;
PGK1 seq:	-1.0242	-0.66711	-1.14953	-1.13604	1.277431	-0.34008	0.123461	1.097203	p20510	Phosphogly	4	-0.22736		HIF-2-alpha transcription factor network; HIF-1-alpha transcription factor network;
PGK1 seq:	-1.29277	-0.70385	-1.487	-1.37248	-0.00611	-0.00247	-0.35411	-0.25249	p20509	Phosphogly	4	-0.68391		HIF-2-alpha transcription factor network; HIF-1-alpha transcription factor network;
PFN2 seq:	-0.70382	0.04268	-0.67033	-1.01197	0.087426	0.406294	-0.86952	-0.82699	p04251	Profilin-2	4	-0.44328		
PFKL pS775	-0.12419	-0.19598	-0.52365	-0.83225	-0.58435	-0.33011	-2.81183	-1.17766	p02550	ATP-depen	4	-0.8225		
PFKFB2 seq	-0.74524	-1.26528	-1.21284	-1.25685	-1.55197	-1.47372	-1.89549	-1.59458	p08908	6-phospho	4	-1.3745	Akt1;	Up with IGF - down with PKCinh2 (Bisindolylmaleimide-1); Up with EGF - down with PKCinh2 (Bisindolylmaleimide-1); Up with IGF - down with PI3Kinh2 (PI-103); Up with EGF - down with PI3Kinh2 (PI-103); Up with IGF - down with PI3Kinh1 (GDC-0941); Up with EGF - down with PI3Kinh1 (GDC-0941); Up with IGF - down with p70S6Kinh2 (DG2); Up with EGF - down with p70S6Kinh2 (DG2); Up with IGF - down with p70S6Kinh1 (PF-4708671); Up with EGF - down with p70S6Kinh1 (P
PFKFB2 seq	-0.39551	-1.03913	-0.67137	-0.64369	-1.96247	-2.17981	-3.08073	-2.36211	p07023	6-phospho	4	-1.54185		
PFKFB2 pS	-1.27643	-3.4811	-0.81589	-0.83748	-1.52845	-2.36329	-2.12816	-4.1244	p09159	6-phospho	4	-2.0694	PKACA; AMPKA1; Akt1;	
PFDN4 pS1	-0.19109	-0.85791	0.040854	-0.32144	-0.06674	-0.86723	-0.50613	-0.30194	p16733	Prefoldin su	4	-0.38395		ERKinh1 (ERK inhbitor Calbiochem); EGFRinh2 (PD-153035); EGFRinh1 (PD-168393); AKTinh2 (MK-2206); AKTinh1 (Akt Inhibitor VIII);
PEBP1 seq:	-0.40766	-0.35675	-0.53143	-0.59454	0.321666	0.284955	0.20565	0.511791	p15594	Phosphatid	4	-0.07079		Aurora B signaling; ErbB1 downstream signaling;
PEBP1 seq:	-0.5756	-0.19591	-0.76664	-0.52098	-0.84216	-0.91845	0.07011	0.268585	p09185	Phosphatid	4	-0.43513		Aurora B signaling; ErbB1 downstream signaling;
PEBP1 seq:	-0.63254	0.415726	-0.45317	-0.88667	-0.4525	-0.07614	0.334914	0.478539	p19864	Phosphatid	4	-0.15898		Aurora B signaling; ErbB1 downstream signaling;
PEAK1 seq:	-0.50872	-0.9134	-0.4338	-0.74487	0.52097	0.395554	-0.26896	0.74555	p05235	Pseudopod	4	-0.15096		
PEAK1 seq:	0.116026	-0.30692	0.242824	0.133834	-0.4966	-0.88564	-0.58065	-0.5598	p14565	Pseudopod	4	-0.29212		
PEA15 pS1	0.016587	0.080229	-0.199	-0.57498	-0.18955	-0.35452	-0.73825	-0.38009	p00294	Astrocytic p	4	-0.29245	Akt1;	PAKinh (PF: p73 transcription factor network;
PEA15 pS1	-0.06599	-0.14591	-0.13786	-0.39445	0.00933	-0.25001	-0.51426	-0.50306	p18750	Astrocytic p	4	-0.25028	Akt1;	PAKinh (PF: p73 transcription factor network;
PEA15 pS1	0.44354	-11.0419	-1.29441	-0.29179	-0.05086	-0.90315	-0.04482	-0.54067	p10498	Astrocytic p	4	-1.71551		p73 transcription factor network;
PDZD2 pS9	-0.22581	-0.22899	-0.56509	-0.31399	0.535731	0.111736	1.112072	-0.96455	p20030	PDZ domair	4	-0.06736		
PDZD2 pS5	-0.14018	-0.95677	-0.39436	-0.46305	0	0	0	0	p04969	PDZ domair	4	-0.24429		
PDXDC1 se	-0.1526	-0.17136	-0.07217	-0.23748	-1.00982	-0.55542	-0.08162	-0.36235	p15304	Pyridoxal-d	4	-0.33035		
PDXDC1 se	-0.02105	-0.44816	0.077924	-0.03513	-0.29686	-0.36909	-0.30859	-0.62944	p15306	Pyridoxal-d	4	-0.2538		
PDXDC1 pS	0.263998	-0.12265	0.222845	0.192749	-0.9103	-1.54703	-2.03764	-1.09128	p15307	Pyridoxal-d	4	-0.62866		
PDS5B seq:	0.056664	-0.24336	0.306282	-0.44212	-1.33546	-1.84997	0.316078	-0.75227	p15346	Sister chror	4	-0.49302		mTORinh2 (Torin-1); ERKinh1 (ERK inhbitor Calbiochem); EGFRinh1 (PD-168393); AKTinh1 (Akt Inhibitor VIII);
PDS5B seq:	-0.23057	-0.41228	-0.38589	-0.61618	-0.85541	-0.88795	-0.64007	-1.08092	p10152	Sister chror	4	-0.63866		
PDS5B seq:	-0.48374	-0.45832	-0.67584	-0.72483	-0.54989	-0.23082	-0.53217	-0.73187	p10155	Sister chror	4	-0.54844		
PDS5B pS1	-0.12039	-1.39104	-1.39253	-2.86627	-1.63981	-1.78609	-0.81911	-1.71589	p20802	Sister chror	4	-1.46639		
PDS5A seq:	0.246413	0.043191	0.300588	0.111859	-0.3732	-0.27034	-0.00642	-0.78007	p14130	Sister chror	4	-0.091		
PDLIM2 se	-0.22935	-0.65372	-0.17966	-0.04022	-0.2027	0.126032	-0.42357	-0.09343	p20569	PDZ and LIN	4	-0.21208		
PDLIM2 se	-0.47726	-0.54659	0.074604	0.162889	0.563022	0.148657	0.081545	0.190157	p03565	PDZ and LIN	4	0.024628		
PDLIM2 pS	-0.7196	0.160812	-0.35667	-0.05617	-0.16428	-0.18568	-0.54341	-0.31881	p15949	PDZ and LIN	4	-0.27298		
PDLIM2 pS	-0.66393	0.507767	-0.31321	-0.05727	-0.22407	-0.13183	-0.60244	-0.47335	p15950	PDZ and LIN	4	-0.24479		
PDLIM2 pS	-0.52561	-2.84653	0.422965	-1.0088	-0.19358	0.167092	-0.26989	-0.21377	p03564	PDZ and LIN	4	-0.55852		
PDIA3 seq:	-0.60974	-0.75618	-1.10182	-0.8606	0.823736	0.536344	0.394885	0.497442	p10774	Protein disu	4	-0.13449		GO:0007165; P:signal transduction;
PDIA3 seq:	-1.4424	-1.8924	-1.51887	-1.58221	1.005065	-1.54385	0.413901	1.557134	p11192	Protein disu	4	-0.62545		GO:0007165; P:signal transduction;
PDIA3 seq:	0.863412	1.15233	-0.40496	-1.54304	-0.80864	-0.32916	-0.12885	0.303914	p16673	Protein disu	4	-0.11187		GO:0007165; P:signal transduction;
PDIA3 pM4	-1.55917	-0.50537	-0.66741	-0.88667	0.036256	-0.01726	-0.27553	0.080395	p10222	Protein disu	4	-0.47434		GO:0007165; P:signal transduction;
PDCD6IP s	-0.43608	-1.10972	-0.12055	-0.35471	-0.54345	-0.19087	-0.18014	-0.65463	p18551	Programme	4	-0.44877		
PDCD4 seq	-0.0532	0.015316	-1.03336	0.145572	-0.04195	-0.6411	-4.38805	-0.12583	p18283	Programme	4	-0.76532		PAKinh (PF: mTOR signaling pathway;
PDCD4 pS4	-1.44224	-1.34505	-1.63749	-1.32775	-0.31419	-0.01426	-0.24178	-0.35147	p07410	Programme	4	-0.83428	Akt1;	mTOR signaling pathway;
PDAP1 seq	-0.24976	0.011801	-0.25478	-0.03563	-0.78201	-1.01071	-1.2197	-1.08207	p07057	28 kDa heat	4	-0.57786		GO:0007165; P:signal transduction;
PDAP1 seq	-1.39303	-0.07256	-0.05427	0.148664	0.233311	-0.10344	-0.06756	0.029084	p07660	28 kDa heat	4	-0.15997		GO:0007165; P:signal transduction;
PDAP1 pS1	-0.30559	-0.18627	-0.04046	-0.17482	-0.85996	-1.14083	-1.45277	-1.02407	p09923	28 kDa heat	4	-0.6481		GO:0007165; P:signal transduction;
PDAP1 pM	-0.22543	-0.30692	0.006201	-0.00956	-0.54022	-0.62718	-0.78179	-0.68799	p09924	28 kDa heat	4	-0.39661		GO:0007165; P:signal transduction;
PCYT1B pM	-0.1831	-0.20766	0.145572	0.124992	0.242522	0.059124	-0.0876	0.091502	p10006	Choline-ph	4	0.023169		
PCYT1A seq	-0.1831	-0.20766	0.145572	0.124992	0.242522	0.059124	-0.0876	0.091502	p10009	Choline-ph	4	0.023169		
PCNA seq:	-1.23936	0.619319	-0.93836	-1.10376	0.393437	0.458153	0.22331	0.481031	p18539	Proliferating	4	-0.13828		BARD1 sign GO:0048015; P:phosphatidylinositol-mediated signaling;
PCNA pM1	-0.42475	-3.15631	0.43054	0.470349	-0.48868	-0.09121	0.023982	-0.38587	p09950	Proliferating	4	-0.45274		BARD1 sign GO:0048015; P:phosphatidylinositol-mediated signaling;
PCM1 seq:	-0.28733	-0.10768	-0.0327	-0.32138	-0.26219	-0.22431	-0.12251	-0.18643	p01936	Pericentriol	4	-0.19307		
PCM1 seq:	0.947326	-0.26573	-1.80951	-0.1642	-0.05879	0.396078	-0.09051	0.368993	p12743	Pericentriol	4	-0.08454		
PCF11 pS7	0.027749	-0.08051	0.105642	-0.09349	-0.85375	-0.24583	-0.27006	-0.66018	p10074	Pre-mRNA c	4	-0.2588		
PCDH1 pS9	-0.83773	-0.67617	-0.39268	-0.54537	-0.40447	0.382693	1.221838	0.116378	p16556	Protocadhe	4	-0.14194		GO:0007267; P:cell-cell signaling;
PCBP2 seq:	0	0	0	0	0	0	0	0	p20783	Poly(rC)-bir	4	0		
PCBP2 seq:	-0.37262	0.070522	-1.07039	-1.58348	-1.66607	-1.00329	-1.37297	-1.4125	p11640	Poly(rC)-bir	4	-1.05135		Up with IGF - down with Akt-mTOR-p70S6K-PI3K; Up with EGF - down with Akt-mTOR-p70S6K-PI3K; Up with IGF - down with PKCinh2 (Bisindolylmaleimide-1); Up with EGF - down with PKCinh2 (Bisindolylmaleimide-1); Akt-mTOR-p70S6K-PI3K; PKCinh2 (Bisindolylmaleimide-1);
PCBP2 seq:	-0.91755	-0.02883	-1.23021	-1.62131	-2.3442	-1.31043	-1.66681	-2.15165	p11642	Poly(rC)-bir	4	-1.40887		Up with IGF - down with Akt-mTOR-p70S6K-PI3K; Up with EGF - down with Akt-mTOR-p70S6K-PI3K; Up with IGF - down with PKCinh2 (Bisindolylmaleimide-1); Up with EGF - down with PKCinh2 (Bisindolylmaleimide-1); Akt-mTOR-p70S6K-PI3K; PKCinh2 (Bisindolylmaleimide-1);
PCBP2 seq:	0.465557	0.594304	0.328803	-0.36672	-1.66426	-1.06549	-0.50764	-0.70357	p14979	Poly(rC)-bir	4	-0.36488		
PCBP1 seq:	-0.63653	-0.48396	-0.62179	-0.89405	-1.51566	-1.12999	-1.25896	-1.51841	p08012	Poly(rC)-bir	4	-1.00742		Up with IGF - down with mTOR-PI3K; Up with EGF - down with mTOR-PI3K; Up with IGF - down with PI3Kinh2 (PI-103); Up with EGF - down with PI3Kinh2 (PI-103); Up with IGF - down with PI3Kinh1 (GDC-0941); Up with EGF - down with PI3Kinh1 (GDC-0941); Up with IGF - down with mTORinh2 (Torin-1); Up with EGF - down with mTORinh2 (Torin-1); Up with IGF - down with mTORinh1 (KU-0063794); Up with EGF - down with mTORinh1 (KU-0063794); Up with IGF - down with ERKinh
PCBP1 seq:	-0.48884	-0.437	-0.5759	-0.80943	-1.56563	-1.1413	-1.22646	-1.50939	p08013	Poly(rC)-bir	4	-0.96924		Up with IGF - down with mTOR-PI3K; Up with EGF - down with mTOR-PI3K; Up with IGF - down with PI3Kinh2 (PI-103); Up with EGF - down with PI3Kinh2 (PI-103); Up with IGF - down with PI3Kinh1 (GDC-0941); Up with EGF - down with PI3Kinh1 (GDC-0941); Up with IGF - down with mTORinh2 (Torin-1); Up with EGF - down with mTORinh2 (Torin-1); Up with IGF - down with mTORinh1 (KU-0063794); Up with EGF - down with mTORinh1 (KU-0063794); Up with IGF - down with ERKinh
PCBP1 seq:	-0.82372	-1.0619	-0.879	-0.99568	-2.70852	-1.73861	-2.26173	-2.85493	p08014	Poly(rC)-bir	4	-1.66551		Up with IGF - down with mTOR-PI3K; Up with EGF - down with mTOR-PI3K; Up with IGF - down with PI3Kinh2 (PI-103); Up with EGF - down with PI3Kinh2 (PI-103); Up with IGF - down with PI3Kinh1 (GDC-0941); Up with EGF - down with PI3Kinh1 (GDC-0941); Up with IGF - down with mTORinh2 (Torin-1); Up with EGF - down with mTORinh2 (Torin-1); Up with IGF - down with mTORinh1 (KU-0063794); Up with EGF - down with mTORinh1 (KU-0063794); Up with IGF - down with ERKinh
PCBP1 seq:	-0.91951	-0.47352	-0.75003	-0.88004	-1.42113	-0.865	-0.98117	-1.12234	p08015	Poly(rC)-bir	4	-0.92659		Up with IGF - down with mTOR-PI3K; Up with EGF - down with mTOR-PI3K; Up with IGF - down with PI3Kinh2 (PI-103); Up with EGF - down with PI3Kinh2 (PI-103); Up with IGF - down with PI3Kinh1 (GDC-0941); Up with EGF - down with PI3Kinh1 (GDC-0941); Up with IGF - down with mTORinh2 (Torin-1); Up with EGF - down with mTORinh2 (Torin-1); Up with IGF - down with mTORinh1 (KU-0063794); Up with EGF - down with mTORinh1 (KU-0063794); Up with IGF - down with ERKinh
PCBP1 seq:	-1.19456	-0.93124	-1.03186	-1.20228	-1.53796	-1.19909	-1.80377	-1.86374	p08017	Poly(rC)-bir	4	-1.34556		Up with IGF - down with mTOR-PI3K; Up with EGF - down with mTOR-PI3K; Up with IGF - down with PI3Kinh2 (PI-103); Up with EGF - down with PI3Kinh2 (PI-103); Up with IGF - down with PI3Kinh1 (GDC-0941); Up with EGF - down with PI3Kinh1 (GDC-0941); Up with IGF - down with mTORinh2 (Torin-1); Up with EGF - down with mTORinh2 (Torin-1); Up with IGF - down with mTORinh1 (KU-0063794); Up with EGF - down with mTORinh1 (KU-0063794); Up with IGF - down with ERKinh
PCBP1 seq:	-0.744	-0.6409	-1.15007	-1.24886	-2.05192	-1.29069	-1.42653	-1.88727	p08018	Poly(rC)-bir	4	-1.30503		Up with IGF - down with mTOR-PI3K; Up with EGF - down with mTOR-PI3K; Up with IGF - down with PI3Kinh2 (PI-103); Up with EGF - down with PI3Kinh2 (PI-103); Up with IGF - down with PI3Kinh1 (GDC-0941); Up with EGF - down with PI3Kinh1 (GDC-0941); Up with IGF - down with mTORinh2 (Torin-1); Up with EGF - down with mTORinh2 (Torin-1); Up with IGF - down with mTORinh1 (KU-0063794); Up with EGF - down with mTORinh1 (KU-0063794); Up with IGF - down with ERKinh
PCBP1 seq:	-0.31942	-0.51498	-0.24642	-0.30938	0.62028	-1.09231	-0.19094	-0.94339	p01096	Poly(rC)-bir	4	-0.37457		
PBK seq: 5	-0.1489	-0.8794	-0.11134	-0.24314	-0.19872	-0.23584	-0.11472	-0.39162	p15646	Lymphokine	4	-0.29046		
PAWR seq:	-0.58562	-0.99246	-0.52537	-1.43325	-0.8118	-0.72714	-1.33467	-1.147	p00284	PRKC apopt	4	-0.94466		Ceramide si GO:0050860; P:negative regulation of T cell receptor signaling pathway;
PATL1 seq:	-0.72369	-0.40725	-0.4879	-0.58333	-0.94685	-1.02371	-1.155	-1.13692	p03444	Protein PAT	4	-0.80808	mTOR;	
PATL1 seq:	-0.68952	-0.69481	-0.50238	-0.80405	-0.92452	-0.64443	-1.15219	-0.99955	p06552	Protein PAT	4	-0.80143	mTOR;	
PATL1 seq:	-0.52343	-0.40707	-0.41732	-0.55811	-0.9502	-0.57801	-0.99896	-0.9601	p06553	Protein PAT	4	-0.67415	mTOR;	
PATL1 pT1	-0.74069	-0.49507	-0.86332	-1.04307	-1.1526	-0.60306	-1.03335	-1.09511	p19214	Protein PAT	4	-0.87828		
PARVB pT3	-0.64634	2.263582	-0.05712	-0.59929	-2.08293	-3.13029	-0.66986	-1.28321	p11764	Beta-parvin	4	-0.77568		Integrin-linked kinase signaling;
PARN pS55	0.000981	-0.18863	-0.66459	-0.46106	-1.74969	-0.79453	-0.55174	-3.69213	p09304	Poly(A)-spe	4	-1.01267	MAPKAPK2;	
PARN pS55	-0.39305	-0.79258	-0.54204	-0.37645	-0.59176	0.073609	-0.05785	-0.36431	p09303	Poly(A)-spe	4	-0.38055	MAPKAPK2;	
PARK7 seq:	-1.16682	-1.08239	-1.01756	-0.75813	0.933542	0.298316	-0.18825	-0.14472	p19963	Protein deg	4	-0.39075		Alpha-synuclein signaling;
PARD6A pS	-0.60309	-0.85458	-1.01692	-0.54664	0.084663	-0.08445	0.020675	-0.03503	p10553	Partitioning	4	-0.37942		Nephrin/Ne GO:0007179; P:transforming growth factor beta receptor signaling pathway;
PARD3 seq	-0.18512	-1.46491	-0.13668	0.203174	-1.05415	-2.01539	-2.55813	-0.62008	p07253	Partitioning	4	-0.97891		GO:0007205; P:protein kinase C-activating G-protein coupled receptor signaling pathway; GO:0007179; P:transforming growth factor beta receptor signaling pathway;
PARD3 seq	0	0	0	0	-0.74261	-1.29088	-0.11777	-0.25774	p18087	Partitioning	4	-0.30112		GO:0007205; P:protein kinase C-activating G-protein coupled receptor signaling pathway; GO:0007179; P:transforming growth factor beta receptor signaling pathway;
PARD3 pS8	-0.19968	-0.55433	-0.21689	-0.37693	-0.4271	-0.3736	-0.20795	-0.66326	p17753	Partitioning	4	-0.37747		GO:0007205; P:protein kinase C-activating G-protein coupled receptor signaling pathway; GO:0007179; P:transforming growth factor beta receptor signaling pathway;
PAPOLA se	-0.04311	-0.07956	-0.11888	-0.15659	-0.6911	0.246203	0.108236	-0.23302	p14543	Poly(A) poly	4	-0.12098		
PANK4 pS3	-0.49823	-0.41571	-0.35605	-0.6814	-0.95881	-2.8271	-2.81153	-0.77043	p16329	Pantothena	4	-1.16491		
PANK2 seq	0.10651	0.479062	-1.01887	0.435599	-0.10732	-0.07445	-0.37853	-0.09	p18347	Pantothena	4	-0.081		
PANK2 seq	-0.41783	-0.44359	-0.25806	-0.16226	-0.36744	-0.25082	-0.60653	-0.37093	p19510	Pantothena	4	-0.35968		
PANK2 seq	-0.24771	-0.61561	-0.54775	-0.49151	-0.15033	-0.15129	-0.29218	-0.13941	p07557	Pantothena	4	-0.32947		
PANK2 seq	-0.41783	-0.44359	-0.25806	-0.16226	-0.36744	-0.25082	-0.60653	-0.37093	p07558	Pantothena	4	-0.35968		
PANK2 seq	-0.24771	-0.62372	-0.54775	-0.49151	-0.15033	-0.15129	-0.29218	-0.13941	p06767	Pantothena	4	-0.33048		
PAN3 seq:	-1.43335	-2.04786	-1.80364	-4.32097	-1.0812	-1.84398	-1.04889	-1.24694	p13590	PAB-depen	4	-1.85335		
PAN3 seq:	-0.56876	0.05626	-0.44735	-0.28457	-0.62403	0.030708	-0.77878	-0.87307	p13589	PAB-depen	4	-0.4362		
PAN2 seq:	-0.45415	-0.29817	-0.72681	-1.10367	0.390348	0.030293	0.441439	0.577655	p11561	PAB-depen	4	-0.14288		
PALM pS11	-0.29224	-0.67946	-0.27283	-0.30629	0	0	0	0	p17350	Paralemmin	4	-0.19385		
PALM pS11	-0.30289	-0.88021	-0.67447	-0.69255	0	0	0	0	p17349	Paralemmin	4	-0.31876		
PALLD seq:	0.033918	0.812403	-0.03547	0.153118	-0.47662	-0.22179	-0.40571	-0.3942	p08691	Palladin	4	-0.06679	CDK1;	
PALLD seq:	-0.30612	0.66245	-0.02351	0.24603	-0.97103	0.346496	-0.96733	-0.62225	p08693	Palladin	4	-0.20441		
PALLD seq:	-0.73902	0.510629	-0.37636	-0.1271	-0.6066	-0.02698	-1.26961	-2.28566	p08694	Palladin	4	-0.61509		
PAK4 pS47	-0.68093	-1.8338	-0.03924	-0.87773	-1.23078	-1.31859	-2.73365	-2.70681	p04932	Serine/thre	4	-1.42769	PAK4;	EGFRinh2 (FGF signalir GO:0023014; P:signal transduction by phosphorylation; GO:0007165; P:signal transduction;
PAK2 pS13	0.0845	-0.64875	0.211017	0.247086	-0.30098	-0.51857	-0.04201	-0.27561	p16372	Serine/thre	4	-0.15541		C-MYC path GO:0023014; P:signal transduction by phosphorylation; GO:0006469; P:negative regulation of protein kinase activity; GO:0050852; P:T cell receptor signaling pathway; GO:0038095; P:Fc-epsilon receptor signaling pathway; GO:0007165; P:signal transduction;
PAK1 seq:	0.207438	-0.01462	-0.10231	-0.50059	-0.27343	-0.10805	-6.79254	-0.50662	p03497	Serine/thre	4	-1.01134	PDK1; PAK2; PAK1;	EPHA2 forv GO:0023014; P:signal transduction by phosphorylation; GO:0050852; P:T cell receptor signaling pathway; GO:0038096; P:Fc-gamma receptor signaling pathway involved in phagocytosis; GO:0038095; P:Fc-epsilon receptor signaling pathway;
PAK1 seq:	-0.25843	-0.28268	-0.12357	-0.21109	-0.06719	-0.08156	-0.11286	-0.04661	p00213	Serine/thre	4	-0.148		EPHA2 forv GO:0023014; P:signal transduction by phosphorylation; GO:0050852; P:T cell receptor signaling pathway; GO:0038096; P:Fc-gamma receptor signaling pathway involved in phagocytosis; GO:0038095; P:Fc-epsilon receptor signaling pathway;
PAGR1 pS2	-0.40427	-0.56641	-0.10447	0.114785	-1.07581	-0.61714	-0.82696	-1.3394	p18612	PAXIP1-ass	4	-0.60246		
PAGR1 pS2	-0.2054	-0.5295	-0.41254	-0.28325	-0.50485	-0.43109	-0.72892	-0.66488	p18613	PAXIP1-ass	4	-0.47005		
PADI2 seq:	-0.108	2.243423	-0.39681	-3.63832	-1.26833	-0.35694	-1.51704	-1.55869	p17461	Protein-argi	4	-0.82509		GO:0030520; P:intracellular estrogen receptor signaling pathway;
PACSIN2 se	0.880036	2.133476	0.09619	-0.63321	-3.71325	-1.91418	-1.27805	-0.87186	p20050	Protein kina	4	-0.66261		
PACS1 seq:	-1.69579	-0.75554	-0.9922	-1.25781	-0.50635	-0.79215	-1.13889	-0.97674	p01463	Phosphofu	4	-1.01443		
PACS1 pS5	-0.32735	-0.28206	-0.25068	-0.89033	0.169649	-1.12419	-0.2881	-0.14126	p08311	Phosphofu	4	-0.39179		
PACS1 pS5	-0.12365	-0.36261	-0.5387	-1.34107	0	0	0	0	p08309	Phosphofu	4	-0.29575		
PABPN1 se	0.231881	0.010558	0.488032	0.335904	-0.53162	-0.10023	-1.05422	-0.92067	p08243	Polyadenyla	4	-0.19255		
PABPN1 pS	-1.38162	0.323483	-0.36599	-1.21573	-2.49031	-2.86763	-2.73051	-0.31621	p08240	Polyadenyla	4	-1.38057		
PABPC1 se	-1.53893	-1.18707	0.731457	-1.06487	-0.05535	-2.00652	-1.3708	0.409651	p13251	Polyadenyla	4	-0.7603		
PA2G4 seq	-0.22709	-0.09949	-0.20191	-0.63749	0.388932	0.410203	-0.15381	-0.09728	p17759	Proliferatio	4	-0.07724		Coregulation of Androgen receptor activity;
P4HB seq:	-0.15178	-1.09911	0.105016	-0.03682	-0.39081	-1.26695	-0.66259	-1.15844	p09343	Protein disu	4	-0.58268		
OTUD5 seq	-0.39038	-0.18388	-0.18674	0.038333	-0.6168	-0.43139	-0.81829	-0.58152	p19318	OTU domai	4	-0.39633		
OTUD5 pS6	0.152994	-0.02515	-0.27478	-0.04385	-0.62708	-0.46917	-0.93816	-0.78029	p19538	OTU domai	4	-0.37568		
OSTF1 pS2	-0.16841	-0.63587	0.439438	0.846708	0.094571	-0.22104	-0.20199	-0.46374	p02545	Osteoclast-	4	-0.03879		GO:0007165; P:signal transduction;
OSBPL7 pS	-0.01586	-1.50129	-0.03935	-0.03317	-0.15679	-1.093	-0.11841	-0.20159	p11649	Oxysterol-b	4	-0.39493		
OSBPL11 p	-1.48422	-0.27723	-0.37825	-0.8785	0.190699	0.276001	-0.29132	0.583207	p05839	Oxysterol-b	4	-0.28245		
OSBPL11 p	-0.8466	-0.457	-0.2573	-0.30179	0.079574	0.221235	-0.08428	0.122428	p05840	Oxysterol-b	4	-0.19047		
OSBPL11 p	-1.15004	-0.38931	-0.15819	-0.2804	-0.0798	-1.37783	-0.09287	-0.73338	p05841	Oxysterol-b	4	-0.53273		
OSBPL10 p	0.48242	-0.38944	-0.25072	-0.52129	0.021554	0.582197	0.580367	-0.59676	p04951	Oxysterol-b	4	0.017119		
OSBP pS35	1.39824	1.961081	1.668311	-7.40364	-1.69494	-0.59851	-1.81583	-0.07651	p16211	Oxysterol-b	4	-0.82022		Up with IGF - down with AKTinh1 (Akt Inhibitor VIII); AKTinh1 (Akt Inhibitor VIII);
ORC6 pT19	0.181398	-0.84933	0.106484	-0.00176	-0.29921	-0.18561	-0.00867	-0.13631	p14242	Origin recog	4	-0.14913	CDK1;	
ORC2 seq:	-0.20252	-0.68449	-0.24641	-0.36688	-0.3582	-0.37151	-0.05811	-0.23035	p00578	Origin recog	4	-0.31481	CDK2;	GO:0007218; P:neuropeptide signaling pathway;
OGFR pS48	-0.5328	-0.66886	-0.22223	-0.23677	-0.03509	-0.00456	-0.02717	-0.15151	p01697	Opioid grov	4	-0.23488		ROCKinh1 (H-1152);
OCIAD1 se	0.750961	0.541833	0.638388	0.458509	-0.94508	-1.51145	-0.76896	-0.72237	p06593	OCIA domai	4	-0.19477		
OCIAD1 se	0.271458	-0.0249	0.173402	0.074194	-0.25484	-0.49842	-0.53808	-0.72039	p06592	OCIA domai	4	-0.1897		
NUPR1 pS5	-0.16539	-0.13059	0.070415	-0.00315	0.113477	0.024166	-1.57065	-2.41681	p18026	Nuclear pro	4	-0.50982		
NUP35 seq	-0.66445	-0.54686	0.958315	0.262887	-0.71963	0.165423	-0.07757	-0.25226	p05440	Nucleoporin	4	-0.10927		GO:0019221; P:cytokine-mediated signaling pathway;
NUP210 se	0.149545	-0.15833	-0.60234	0.230014	-0.59113	-0.62965	-1.28483	-0.82958	p13382	Nuclear por	4	-0.46454		GO:0019221; P:cytokine-mediated signaling pathway;
NUP153 se	-0.11036	0.333806	0.112283	-0.20989	-0.64787	0.318001	0.114519	-0.54671	p14391	Nuclear por	4	-0.07953		AKTinh1 (A Regulation GO:0019221; P:cytokine-mediated signaling pathway;
NUP153 se	-1.15677	-2.34316	-0.56645	-0.70511	-0.13455	-0.1813	-0.05815	-0.25041	p08977	Nuclear por	4	-0.67449		Regulation GO:0019221; P:cytokine-mediated signaling pathway;
NUP133 se	0.067453	-0.50256	0.406326	0.336369	-0.18949	-0.06338	-0.10842	-0.25963	p13184	Nuclear por	4	-0.03917	CDK2;	GO:0019221; P:cytokine-mediated signaling pathway;
NUP107 pS	-0.42256	0.067456	-0.13176	-1.37489	-0.2825	-0.48145	-0.17596	-0.1055	p01209	Nuclear por	4	-0.3634		GO:0019221; P:cytokine-mediated signaling pathway;
NUMA1 se	0.250148	-0.52364	0.245236	0.393845	0.11348	-0.16245	0.087945	-0.19619	p13013	Nuclear mit	4	0.026046		Caspase cascade in apoptosis;
NUMA1 pT	0.127389	-0.9554	0.662789	0.198359	-0.17449	-0.22773	0.216056	-0.28598	p14462	Nuclear mit	4	-0.05488		ROCKinh2 (Caspase cascade in apoptosis;
NUMA1 pT	0.180939	-0.68146	0.61657	0.155633	-0.15265	-0.23222	0.143218	-0.18278	p14461	Nuclear mit	4	-0.0191		ROCKinh2 (Caspase cascade in apoptosis;
NUMA1 pT	0.067666	-0.13079	-0.01035	-0.05873	-0.31862	-0.42715	-0.18187	-0.51624	p00829	Nuclear mit	4	-0.19701		Caspase cascade in apoptosis;
NUMA1 pS	0	0	0	0	1.585282	3.88671	-8.11492	0.687444	p07573	Nuclear mit	4	-0.24444	AurB;	ROCKinh1 (Caspase cascade in apoptosis;
[illegible]														

LSM14A se	-0.53476	-1.07213	-0.89382	-0.82603	-0.53012	-0.46325	-0.52389	-0.23588	p04330	Protein LSM	4	-0.63498				
LRWD1 pS2	0.085128	-0.37393	-0.1298	-0.80062	-0.37084	0.040371	-0.19076	-0.41366	p20028	Leucine-rich	4	-0.26926				
LRRN3 pT3	0.114515	-0.31332	0.401698	-0.11297	-0.01651	-1.12926	-0.22467	-0.29301	p13109	Leucine-rich	4	-0.19669				
LRRFIP1 se	0.072947	-0.75782	0.411596	0.42238	0.294123	-0.61512	0.076434	0.407572	p09367	Leucine-rich	4	0.039015		PKCinh2 (Bisindolylmaleimide-1);		
LRRC30 se	0.555839	0.277848	0.391317	-0.58766	-0.28927	-0.52673	-0.32513	-1.86364	p11486	Leucine-rich	4	-0.29593				
LRBA seq: 1	-2.25445	-1.3295	-1.31571	-1.17087	0.708278	-2.77791	-3.53721	-1.06415	p19340	Lipopolysac	4	-1.59269				
LRBA seq: 1	0.721049	1.736571	-0.94967	0.012883	-3.97569	-0.54823	0.048029	-0.79246	p12023	Lipopolysac	4	-0.46844				
LRBA pS17	-0.40796	-0.59942	-0.27814	-0.19767	0.12738	0.026091	0.41977	0.332006	p06167	Lipopolysac	4	-0.07224		AKTinh2 (MK-2206);		
LPIN2 seq:	0.137348	-0.52037	-0.41107	0.224289	-0.52694	-0.38569	-0.24967	-0.40322	p06089	Phosphatid	4	-0.26691				
LPIN1 pS44	1.353363	0.927636	-6.67899	1.242676	0.162842	0.399905	0.626801	0.601593	p06306	Phosphatid	4	-0.17052				
LMO7 seq:	-0.40953	-0.73001	-0.15697	0.099649	-0.59083	-1.22084	-0.65478	-0.63995	p03116	LIM domain	4	-0.53791				
LMO7 seq:	0.576824	-1.93984	-0.04766	-0.14446	0.468267	0.184216	0.504087	0.620946	p07202	LIM domain	4	0.027798	AurB;			
LMO7 seq:	-0.06715	-0.48716	-0.14446	-0.40777	-0.30338	-0.53028	-0.54248	-0.23644	p06213	LIM domain	4	-0.33989				
LMO7 seq:	0.064506	-0.31497	-0.10768	-0.35481	-0.29835	-0.60949	-0.60926	-0.32393	p06212	LIM domain	4	-0.31925				
LMO7 seq:	-0.30954	-0.89179	-0.6311	-0.81195	-0.27008	-0.74835	-0.8875	-0.55713	p06214	LIM domain	4	-0.63843				
LMO7 seq:	0	0	0	0	-0.62195	-0.77842	-0.75481	-1.05773	p19357	LIM domain	4	-0.40161				
LMO7 pT10	-2.3454	-1.01246	-1.5972	-3.17231	-0.14374	-0.40225	-0.36391	-0.31537	p13726	LIM domain	4	-1.16908				
LMO7 pS15	-0.6479	-0.35396	-0.84167	-0.6879	-0.27231	-0.30471	-0.51442	-0.35448	p02071	LIM domain	4	-0.49717				
LMO7 pS15	-1.56504	-3.69781	-0.27626	-0.57701	-0.05171	-0.50816	0.024399	0.079475	p08111	LIM domain	4	-0.82151				
LMNB2 pS4	-0.42664	-1.09529	-1.08457	-0.42502	-0.28894	-1.16458	-1.80432	-0.2107	p19303	Lamin-B2	4	-0.81251			Caspase cascade in apoptosis;	
LMNB2 pS3	0.064804	-0.42812	0.236768	0.294809	-0.29142	-0.50008	-0.25329	-0.68441	p20659	Lamin-B2	4	-0.19512	CDK2;		Caspase cascade in apoptosis;	
LMNB1 seq	-0.55684	-1.07644	-0.62754	-0.87413	0.671881	0.763532	0.937628	0.702484	p12137	Lamin-B1	4	-0.00743			Caspase cascade in apoptosis;	
LMNB1 seq	-0.02425	-0.57338	0.161918	-0.04846	0.063874	-0.05708	0.091908	-0.03615	p11503	Lamin-B1	4	-0.0527	CDK1;		Caspase cascade in apoptosis;	
LMNB1 seq	1.034413	1.321422	-1.58999	-2.2071	-7.18632	1.224126	-0.28614	-0.71218	p14334	Lamin-B1	4	-1.05022			Caspase cascade in apoptosis;	
LMNB1 pT2	0.079074	-0.41918	0.177958	0.335419	-0.29608	-0.46833	-0.34925	-0.69699	p20656	Lamin-B1	4	-0.20467			Caspase cascade in apoptosis;	
LMNB1 pS3	-0.10381	-0.15517	-0.12181	0.04182	-0.36316	-0.4028	-0.53095	-0.78242	p11501	Lamin-B1	4	-0.30229			Caspase cascade in apoptosis;	
LMNB1 pS3	0.246144	0.379702	-0.23465	0.040031	-0.51005	-0.34417	-0.23446	-0.54579	p12241	Lamin-B1	4	-0.15041			Caspase cascade in apoptosis;	
LMNA seq:	-0.46616	-0.12784	-0.29935	-1.31449	-1.11646	-1.77327	-0.16266	-1.52024	p08217	Prelamin-A/	4	-0.84756			Caspase cas	GO:0006987; P:activation of signaling protein activity involved in unfolded protein response;
LMNA seq:	-1.89488	-1.29217	-2.38314	-0.58252	-0.28648	-0.07293	0.024319	0.13401	p15354	Prelamin-A/	4	-0.79423			Caspase cas	GO:0006987; P:activation of signaling protein activity involved in unfolded protein response;
LMNA seq:	-0.13296	-0.6066	-0.15485	-0.17411	-0.2435	-0.07073	-0.43792	-0.2097	p10883	Prelamin-A/	4	-0.2538	CDK1;		Caspase cas	GO:0006987; P:activation of signaling protein activity involved in unfolded protein response;
LMNA seq:	-0.06335	-0.54335	-0.06411	-0.12197	-0.1655	-0.0351	-0.38134	-0.23314	p10884	Prelamin-A/	4	-0.20098	CDK1;		Caspase cas	GO:0006987; P:activation of signaling protein activity involved in unfolded protein response;
LMNA seq:	0.418559	-0.10197	-0.33099	-0.04577	-0.99929	-1.15372	-1.2041	-0.88259	p13771	Prelamin-A/	4	-0.53748			Caspase cas	GO:0006987; P:activation of signaling protein activity involved in unfolded protein response;
LMNA seq:	-0.86335	-0.68812	-1.21285	-1.11788	0.753963	0.603136	0.463588	0.594056	p18014	Prelamin-A/	4	-0.18343			Caspase cas	GO:0006987; P:activation of signaling protein activity involved in unfolded protein response;
LMNA seq:	-0.12876	0.6772	-0.60339	-0.79305	1.027127	0.360067	-0.10661	-0.27434	p17770	Prelamin-A/	4	0.019781			Caspase cas	GO:0006987; P:activation of signaling protein activity involved in unfolded protein response;
LMNA pS45	-0.41246	-0.16814	-0.55029	-0.61537	-0.27853	-0.30971	-0.51285	-0.00989	p09492	Prelamin-A/	4	-0.35715			Caspase cas	GO:0006987; P:activation of signaling protein activity involved in unfolded protein response;
LMNA pS30	-0.13836	-0.06843	-0.24972	-0.14717	-0.86937	-0.82309	-0.91282	-0.67719	p14396	Prelamin-A/	4	-0.48577			Caspase cas	GO:0006987; P:activation of signaling protein activity involved in unfolded protein response;
LMBRD2 se	-0.04162	-1.05325	-0.04818	-0.0653	-0.14697	-0.92523	-0.00274	-0.21347	p00235	LMBR1 dor	4	-0.3121				
LIMK1 seq:	-0.56513	-0.54167	-0.16721	-0.51123	0.07276	0.164104	-0.5903	0.217612	p06137	LIM domain	4	-0.24013	P38A;		RhoA signal	GO:0007266; P:Rho protein signal transduction; GO:0038096; P:Fc-gamma receptor signaling pathway involved in phagocytosis; GO:0007165; P:signal transduction;
LIMD2 pS2	-0.33731	-0.52523	-0.53307	-0.08196	0.021984	-0.28803	-0.06461	0.251641	p05369	LIM domain	4	-0.19457				
LIMA1 seq:	0.268878	-0.2373	-0.1349	-0.6355	0.127387	0.106514	-0.04898	-0.18281	p13129	LIM domain	4	-0.09209			Stabilization and expansion of the E-cadherin adherens junction;	
LIMA1 seq:	-1.00482	-0.03171	-2.50489	-0.98214	-0.93161	-0.84654	-1.802	-0.79196	p13072	LIM domain	4	-1.11196		Up with IGF	Stabilization and expansion of the E-cadherin adherens junction;	
LIMA1 seq:	-0.49479	0.073117	-1.92227	-0.85319	-0.85092	-0.67498	-1.40586	-0.66292	p13074	LIM domain	4	-0.84898			Stabilization and expansion of the E-cadherin adherens junction;	
LIMA1 pS7	0.270198	0.068174	0.145145	-0.5617	-0.69076	-0.56298	-3.20789	-0.60564	p04275	LIM domain	4	-0.64318			Stabilization and expansion of the E-cadherin adherens junction;	
LIMA1 pS6	0.280117	-0.19289	-0.13479	-0.70711	-0.00978	-0.64497	-0.45959	-0.17965	p13130	LIM domain	4	-0.25608			Stabilization and expansion of the E-cadherin adherens junction;	
LIMA1 pS2	-0.99987	-0.03037	-2.69848	-0.83595	-0.87425	-0.80978	-1.92765	-0.42984	p13073	LIM domain	4	-1.07577		Up with IGF	Stabilization and expansion of the E-cadherin adherens junction;	
LGALS3BP s	-0.57899	1.596167	-1.36023	-2.64064	0	0	0	0	p06100	Galectin-3-l	4	-0.37296				GO:0007165; P:signal transduction;
LGALS1 seq	-0.35386	-0.49603	-0.36698	-2.29945	0.611781	0.203601	0.200177	0.263734	p18829	Galectin-1	4	-0.27963			Regulation (	GO:0043123; P:positive regulation of I-kappaB kinase/NF-kappaB signaling; GO:0007165; P:signal transduction;
LEO1 seq: 5	-0.41512	-0.90463	-0.47165	-0.49173	-0.26985	-0.55663	-0.20371	-0.30854	p10911	RNA polym	4	-0.45273				GO:0016055; P:Wnt signaling pathway;
LEO1 pS65	-9.67834	-0.71583	-0.24792	-0.01312	0.292787	0.274229	0.661052	0.507416	p00024	RNA polym	4	-1.11496				GO:0016055; P:Wnt signaling pathway;
LDB1 seq: 3	0.401901	-0.14743	-0.25327	-0.41548	-2.36378	-1.30392	-1.87988	-1.65217	p12857	LIM domain	4	-0.95175				GO:0016055; P:Wnt signaling pathway;
LATS1 seq:	-0.37667	0.096136	-0.53394	-0.43344	-0.19705	-0.00441	0.002667	-0.11336	p04809	Serine/thre	4	-0.19501	NuaK2; NuaK1;			GO:0009755; P:hormone-mediated signaling pathway; GO:0035329; P:hippo signaling; GO:0090090; P:negative regulation of canonical Wnt signaling pathway;
LATS1 pS61	-0.35543	0.073053	-0.16199	-0.26046	0.05191	-0.93295	-0.15141	-0.21402	p08486	Serine/thre	4	-0.24391	CDK1;	MEKinh1 (GSK-112021		GO:0009755; P:hormone-mediated signaling pathway; GO:0035329; P:hippo signaling; GO:0090090; P:negative regulation of canonical Wnt signaling pathway;
LATS1 pS61	0.083579	-0.02534	0.204456	0.103144	-0.10212	-0.7919	-0.1389	-0.25136	p08485	Serine/thre	4	-0.1148	CDK1;	MEKinh1 (GSK-112021		GO:0009755; P:hormone-mediated signaling pathway; GO:0035329; P:hippo signaling; GO:0090090; P:negative regulation of canonical Wnt signaling pathway;
LASP1 seq:	-0.82875	-0.21744	-0.90564	-0.80757	0.486586	0.338882	0.227325	0.328062	p08022	LIM and SH	4	-0.17232				GO:0009967; P:positive regulation of signal transduction;
LASP1 seq:	-0.64044	-0.65021	-1.13564	-0.89611	0.670891	0.403097	-0.00709	0.155618	p11493	LIM and SH	4	-0.26248				GO:0009967; P:positive regulation of signal transduction;
LASP1 seq:	-0.83342	1.204584	-0.43409	-1.09392	1.664348	1.894707	-5.19878	2.211658	p07920	LIM and SH	4	-0.07311				GO:0009967; P:positive regulation of signal transduction;
LASP1 seq:	-0.78941	-0.93023	-0.58342	-1.11813	-0.35704	1.414894	0.993845	-0.76527	p10099	LIM and SH	4	-0.26684				GO:0009967; P:positive regulation of signal transduction;
LASP1 pT1	-0.66833	-0.82053	-0.51113	-0.74871	-0.5577	-0.36697	-0.3237	-0.5106	p15825	LIM and SH	4	-0.56346		ROCKinh1 (H-1152);		GO:0009967; P:positive regulation of signal transduction;
LASP1 pT1	-0.95618	-0.79826	-0.61032	-0.48452	-0.3994	-0.18639	-0.14928	-0.23853	p16089	LIM and SH	4	-0.47786		ROCKinh1 (H-1152);		GO:0009967; P:positive regulation of signal transduction;
LASP1 pT1	-0.69985	-0.73221	-0.54936	-0.55558	-0.22355	-0.18532	0.086489	-0.11709	p16090	LIM and SH	4	-0.37206		ROCKinh1 (H-1152);		GO:0009967; P:positive regulation of signal transduction;
LASP1 pS14	0.608214	-0.12934	-0.09339	0.265613	-0.01791	0.345088	-0.69314	-0.22202	p10093	LIM and SH	4	0.007891	PKG2; PKG1 iso2; PKG1; PKACA;			GO:0009967; P:positive regulation of signal transduction;
LASP1 pM6	-0.0975	-1.34824	0.455766	0.515663	0.249783	-0.79684	-0.96775	-0.78281	p07918	LIM and SH	4	-0.34649				GO:0009967; P:positive regulation of signal transduction;
LASP1 pM1	0.203062	-0.77872	-0.51148	0.036093	0.154013	0.278127	-0.71522	-0.28455	p10096	LIM and SH	4	-0.20234	PKG2; PKG1 iso2; PKG1; PKACA;			GO:0009967; P:positive regulation of signal transduction;
LARP7 seq:	-0.05096	0.13654	-0.92163	-0.18823	-0.20802	-0.04339	-0.2103	-0.24895	p12613	La-related p	4	-0.21687				
LARP7 seq:	-0.02471	-0.25208	0.265084	0.059858	-0.10449	0.073063	0.199581	-0.02378	p04085	La-related p	4	0.024067				
LARP4B se	-0.55533	-0.45938	-0.42674	-0.25331	-0.45456	-0.32432	-0.35645	-0.50064	p06977	La-related p	4	-0.41634				
LARP4B pS	0.351498	-1.40372	-1.03039	-0.14229	-1.12241	-1.42628	-1.50499	-1.39794	p12840	La-related p	4	-0.95957				
LARP1B pS	-0.24696	-0.73875	-0.00122	0.131709	-0.2107	-0.27497	-0.11669	0.004459	p15635	La-related p	4	-0.18164		AKTinh1 (Akt Inhibitor VIII);		
LARP1B pS	-0.23574	-0.83309	-0.19226	-0.07922	-0.82692	0.002743	-0.53296	0.170799	p14232	La-related p	4	-0.31583				
LARP1 seq:	-1.1641	-0.73254	-1.61446	-1.63497	-1.4999	-0.96286	-1.83565	-1.73967	p05094	La-related p	4	-1.39802	mTOR;			GO:0031929; P:TOR signaling;
LARP1 seq:	-0.23506	-1.56222	0.862048	-0.93146	-0.50971	-0.41647	-1.24364	-0.05678	p17671	La-related p	4	-0.51166		Up with IGF - down witl		GO:0031929; P:TOR signaling;
LARP1 seq:	-0.41026	-0.33236	-1.00967	-1.12296	-0.89016	-0.63526	-1.18992	-0.88246	p19028	La-related p	4	-0.80913		Up with IGF - down witl		GO:0031929; P:TOR signaling;
LARP1 pT5	-0.38256	-0.47349	-0.5212	-0.50259	-0.17403	-0.17336	-0.17726	-0.29048	p19159	La-related p	4	-0.33687				GO:0031929; P:TOR signaling;
LARP1 pS7	-1.1641	-0.73254	-1.60925	-1.61416	-1.49996	-0.96292	-1.8357	-1.73972	p05098	La-related p	4	-1.39479	mTOR; CDK	Akt-mTOR-PI3K; PI3Kinl		GO:0031929; P:TOR signaling;
LARP1 pS1	-0.68305	-0.1601	-0.87189	-0.54612	-0.00436	0.019816	-0.07968	-0.03056	p09766	La-related p	4	-0.29449				GO:0031929; P:TOR signaling;
LAMTOR2 s	-0.21019	0.471287	-1.03029	-0.35243	0.010389	0.042665	0.676764	0.462437	p09603	Ragulator c	4	0.008829				GO:0032008; P:positive regulation of TOR signaling; GO:0000186; P:activation of MAPKK activity;
LAMTOR1 s	-0.22888	-0.73476	0.270248	0.158325	-0.03174	-0.20969	-0.20092	-0.43945	p11364	Ragulator c	4	-0.17711				GO:0032008; P:positive regulation of TOR signaling; GO:0043410; P:positive regulation of MAPK cascade;
LAMTOR1 p	0.291566	-0.48295	0.504009	0.412815	0.037176	-0.18361	-0.08092	-0.41135	p12959	Ragulator c	4	0.010843				GO:0032008; P:positive regulation of TOR signaling; GO:0043410; P:positive regulation of MAPK cascade;
LAD1 seq: 3	-0.53591	-1.0373	-0.90285	-1.23326	0.339508	-0.27637	-0.36758	-0.11273	p06256	Ladinin-1	4	-0.51581				
LAD1 seq: 2	-0.37014	-1.00972	-0.08376	-0.15751	-0.11684	-0.22124	-0.22954	-0.37662	p07547	Ladinin-1	4	-0.32067				
LAD1 seq: 2	-0.49589	0.180104	-0.4341	-0.33264	-0.16813	-0.13787	-0.02511	-0.22469	p20317	Ladinin-1	4	-0.20479				
LAD1 seq: 1	-0.37038	0.515729	-0.61977	-0.0351	-0.53284	0.072869	-0.6018	-0.53555	p09142	Ladinin-1	4	-0.26336				
L1RE1 seq:	-0.19919	0.606735	0.12683	-1.07649	-0.67965	-0.21805	-0.37068	0.527256	p03672	LINE-1 retrc	4	-0.1604				
KTN1 pS75	-0.00743	0.291956	0.20695	0.155682	-0.23868	-0.35689	-0.20168	-0.52209	p17604	Kinectin	4	-0.08402				
KRT80 seq:	-10.2065	3.69582	-0.0336	0.816996	-0.21321	-0.32684	-0.25841	-0.38387	p04956	Keratin, typ	4	-0.8637				
KRT8 seq: 4	-0.56078	-0.07621	-0.78629	-0.67902	1.209227	0.218531	0.202394	0.103818	p11928	Keratin, typ	4	-0.04604			Signaling m	GO:0033209; P:tumor necrosis factor-mediated signaling pathway;
KRT8 seq: 3	-0.81329	0.505363	-1.08617	-0.39356	0.955892	-0.46282	-0.31256	0.785729	p10813	Keratin, typ	4	-0.10268			Signaling m	GO:0033209; P:tumor necrosis factor-mediated signaling pathway;
KRT8 seq: 3	-0.67492	-0.69899	-1.03309	-1.06398	0.326449	0.188233	-0.16191	0.121164	p16157	Keratin, typ	4	-0.37463			Signaling m	GO:0033209; P:tumor necrosis factor-mediated signaling pathway;
KRT8 seq: 3	-0.79559	1.070758	-1.57871	-3.17769	-2.43611	-1.6973	-1.00828	0.096033	p19588	Keratin, typ	4	-1.19086			Signaling m	GO:0033209; P:tumor necrosis factor-mediated signaling pathway;
KRT8 seq: 3	-0.50557	-0.01239	-0.95381	-0.97472	0.544497	0.405059	-0.16957	0.423664	p11049	Keratin, typ	4	-0.15535			Signaling m	GO:0033209; P:tumor necrosis factor-mediated signaling pathway;
KRT8 seq: 3	-0.90639	-0.27748	-0.58398	-0.44909	0.324763	0.084302	0.122208	0.036189	p02642	Keratin, typ	4	-0.20618			Signaling m	GO:0033209; P:tumor necrosis factor-mediated signaling pathway;
KRT8 seq: 2	-0.57347	0.558827	-0.55445	-1.01838	0.253771	0.361668	0.03445	0.113135	p08255	Keratin, typ	4	-0.10306			Signaling m	GO:0033209; P:tumor necrosis factor-mediated signaling pathway;
KRT8 seq: 1	0.094527	-0.39195	-0.06673	0.597675	0.738113	-1.00215	-0.41917	-0.38442	p20743	Keratin, typ	4	-0.10426			Signaling m	GO:0033209; P:tumor necrosis factor-mediated signaling pathway;
KRT8 pS74	-10.883	-0.16246	0.092317	-0.4709	-10.8503	0.388748	2.447803	0.673101	p16032	Keratin, typ	4	-2.34559	PKCD; P38A	Up with IGF	Signaling m	GO:0033209; P:tumor necrosis factor-mediated signaling pathway;
KRT8 pS25	0.789476	0.049638	0.410189	-0.77751	-2.2021	-0.25138	-0.18437	0.336816	p05289	Keratin, typ	4	-0.22866			Signaling m	GO:0033209; P:tumor necrosis factor-mediated signaling pathway;
KRT75 pS3	-0.14713	-1.06768	-0.15124	-0.22585	0.387796	-0.0398	0.376025	0.401722	p19724	Keratin, typ	4	-0.05827				
KRT7 seq: 3	-0.53882	0.087351	-0.98706	-0.63266	0.491673	0.03578	-0.24473	0.075603	p11051	Keratin, typ	4	-0.21411				
KRT7 seq: 2	-0.7549	0.130899	-0.73109	-0.82457	1.092505	0.506427	-0.05543	0.224619	p16789	Keratin, typ	4	-0.05144				
KRT7 seq: 2	-0.24153	-0.79436	0.480885	0.104373	0.039837	-0.36534	-0.2909	-0.33195	p05522	Keratin, typ	4	-0.17487				
KRT6C seq:	-0.45796	0.678532	-0.6731	-0.91659	1.397513	-0.02294	0.236653	0.099548	p16897	Keratin, typ	4	0.042708				
KRT19 seq:	-0.12765	-0.2072	-0.44385	-0.51806	0.702438	0.714121	0.266849	0.343761	p21102	Keratin, typ	4	0.091301			Signaling mediated by p38-alpha and p38-beta;	
KRT19 pS2	0.517991	0.657209	-0.18369	-0.56237	-0.04977	-0.01445	-0.56417	-0.34591	p15366	Keratin, typ	4	-0.06814			Signaling mediated by p38-alpha and p38-beta;	
KRT19 pM2	-0.20591	-0.36166	-0.56964	-0.58477	-0.27838	-0.5073	-0.66919	-0.98328	p04145	Keratin, typ	4	-0.52002			Signaling mediated by p38-alpha and p38-beta;	
KRT18 seq:	-0.22472	-0.56135	-0.03284	-0.01313	-0.20104	-0.54249	-0.1987	-0.49608	p03531	Keratin, typ	4	-0.2838			Caspase cas	GO:0033209; P:tumor necrosis factor-mediated signaling pathway;
KRT18 seq:	-0.06343	-0.72484	0.787401	0.059856	-0.61998	-0.62875	-2.85189	-1.30038	p16022	Keratin, typ	4	-0.66775			Caspase cas	GO:0033209; P:tumor necrosis factor-mediated signaling pathway;
KRT18 seq:	-0.31256	-1.00893	-0.63011	-0.81971	-0.65062	-0.75162	-1.01191	-1.06722	p11432	Keratin, typ	4	-0.78159		CAMKinh1	Caspase cas	GO:0033209; P:tumor necrosis factor-mediated signaling pathway;
KRT18 seq:	-0.08363	-0.8877	-0.30852	-0.41449	-0.25168	-0.54241	-0.88465	-0.58694	p11431	Keratin, typ	4	-0.495		CAMKinh1	Caspase cas	GO:0033209; P:tumor necrosis factor-mediated signaling pathway;
KRT18 seq:	-0.01127	-1.36773	-0.61953	-0.3242	-0.1029	-0.76574	-1.46023	-0.63974	p11433	Keratin, typ	4	-0.66142		CAMKinh1	Caspase cas	GO:0033209; P:tumor necrosis factor-mediated signaling pathway;
KRT18 pM7	0.148566	0.343548	-0.37176	-0.07604	-1.1358	-0.04337	-0.06607	0.241051	p16013	Keratin, typ	4	-0.11999			Caspase cas	GO:0033209; P:tumor necrosis factor-mediated signaling pathway;
KRT17 seq:	-0.6134	-0.44059	-1.10893	-0.49942	1.203547	0.651486	0.289455	0.102256	p02662	Keratin, typ	4	-0.05195			Glucocortic	GO:0007165; P:signal transduction;
KRI1 pS628	0.543417	-0.10018	-0.07644	0.549039	0.577489	-10.3415	1.47302	1.150766	p08341	Protein KRI	4	-0.77805				GO:0006469; P:negative regulation of protein kinase activity;
KNOP1 seq	-0.42978	-0.62238	-0.16714	-0.43875	-0.37275	-0.36454	-0.31202	-0.22375	p04570	Lysine-rich	4	-0.36639				
KNOP1 pS4	-0.36277	-0.67643	-0.15249	0.033192	-0.17948	-0.26561	-0.31856	-0.13039	p00058	Lysine-rich	4	-0.25657				
KMT2D pS3	0.209638	-0.0817	0.143089	0.150589	-0.5235	-0.34946	-0.63594	-0.58402	p01638	Histone-lys	4	-0.20891				
KMT2D pS2	-0.49979	-0.55897	-0.4066	-0.38931	-1.97426	-2.07863	-2.06751	-2.56543	p05739	Histone-lys	4	-1.31756				
KMT2B seq	-1.05664	-1.24222	-1.15834	-1.42335	0	0	0	0	p19939	Histone-lys	4	-0.61007				
KMT2B pS8	0.193253	0.321306	0.109003	-0.06016	-0.60124	-0.19342	-0.42574	-0.78138	p01836	Histone-lys	4	-0.1798				
KMT2B pS3	0.067661	-0.10168	0.261993	0.164662	-0.57421	-0.41997	-0.3799	-0.76858	p01450	Histone-lys	4	-0.21875				
KMT2A seq	0.352476	0.240499	0.081137	0.182331	-0.39273	-0.17477	-0.3145	-0.39224	p02734	Histone-lys	4	-0.05222				
KLRG2 seq:	-0.45662	-0.71464	-0.25222	0.019531	0	0	0	0	p02888	Killer cell lec	4	-0.17549				
KLHDC4 se	0.289499	-9.50023	-0.08296	-0.62193	-1.80103	0.131974	-0.38802	-0.83312	p07714	Kelch doma	4	-1.60073				
KLHDC4 se	-0.47943	-0.6537	-0.22117	-0.46991	0.035773	-0.08396	-0.17683	-0.14371	p07716	Kelch doma	4	-0.27412				
KLF16 seq:	-0.23415	-0.39628	-0.2397	-0.03269	-0.33679	-0.18773	-0.60958	-0.81162	p15998	Krueppel-lik	4	-0.35607				
KLC4 seq: 5	-0.2996	-0.55405	-0.25848	-0.343	-0.84266	-1.26547	-0.67776	-0.91159	p07657	Kinesin ligh	4	-0.64407				
KLC4 pS56	0.016836	-0.764	0.267694	0.113343	-0.16001	-4.49557	-0.05635	-0.42516	p03922	Kinesin ligh	4	-0.6879				
KLC2 seq: 5	-0.4777	-0.50715	-0.42096	-1.02185	0.204217	-0.54285	-0.70093	-0.38787	p19489	Kinesin ligh	4	-0.48189	AMPKA1;			
KLC2 seq: 5	-0.3035	-0.502	0.313827	0.069923	-1.63457	-0.81065	-2.75625	-1.433	p03743	Kinesin ligh	4	-0.88203				
KLC2 seq: 5	0.496113	-0.87539	-0.29272	0.031914	-0.17916	-0.88989	-0.76311	-0.61075	p06577	Kinesin ligh	4	-0.38537				
KLC2 pS61	-0.367	-0.99525	-0.19437	-0.10819	0.11126	-0.33736	-0.29124	-0.23347	p02535	Kinesin ligh	4	-0.30195				
KIF4A seq:	-0.84287	-0.43004	-2.42965	-1.10093	-0.60527	-0.06586	-2.12918	-0.95072	p02839	Chromosor	4	-1.06932		Up with IGF - down with PAKinh (PF3758309); Up with EGF - down with PAKinh (PF3758309); PAKinh (PF3758309);		
KIF4A seq:	0.031221	-0.42822	-0.30345	0.212465	0.229852	-0.02106	-0.36769	-0.321	p10809	Chromosor	4	-0.12099				
KIF23 pM8	-0.09421	-0.71341	0.25442	-0.02634	-0.01726	-0.27406	-0.26061	-0.45775	p00219	Kinesin-like	4	-0.19865			Aurora B signaling;	
KIF21A seq	-0.46319	-0.30004	0.011465	-0.21986	0	0	0	0	p14650	Kinesin-like	4	-0.12145				
KIF1A pS15	-0.34546	-0.12474	-0.21193	-0.26963	-0.42441	-0.56992	-0.5711	-0.30381	p12942	Kinesin-like	4	-0.35262				
KIF16B pS1	-0.21985	-0.98597	-0.19108	0.205182	0	0	0	0	p05234	Kinesin-like	4	-0.14896				GO:0008543; P:fibroblast growth factor receptor signaling pathway; GO:0007173; P:epidermal growth factor receptor signaling pathway;
KIF13B seq	0.022168	-0.09888	-0.44142	0.104881	-0.32571	-0.4788	-0.70078	-0.35883	p06698	Kinesin-like	4	-0.28467			Arf6 signali	GO:0007165; P:signal transduction;
KIF13B seq	-0.62751	-0.3586	-0.49988	-0.65091	-0.91462	-0.71183	-0.43046	-1.06016	p15912	Kinesin-like	4	-0.65675			Arf6 signali	GO:0007165; P:signal transduction;
KIF12 seq:	1.265275	-2.7503	0.14348	0.197625	0	0	0	0	p07030	Kinesin-like	4	-0.14299				
KIAA1671 s	0.120862	-0.13827	0.060897	-0.2866	-0.29705	-0.23138	-1.42716	-0.73498	p18331	Uncharacte	4	-0.36671				
KIAA1671 s	-0.55183	-0.80128	-0.37633	-0.44001	-0.31118	-0.25343	-0.32648	-0.32888	p20397	Uncharacte	4	-0.42368				
KIAA1671 p	0.098016	-0.50062	0.452677	0.226387	0.141708	0.007852	0.109485	-0.15783	p03186	Uncharacte	4	0.04721				
KIAA1671 p	-0.24142	-2.07071	-1.17901	-0.73536	-1.07526	-1.15706	-0.99351	-0.89589	p06941	Uncharacte	4	-1.04353				
KIAA1671 p	-0.90362	-1.65811	-2.77251	-0.85594	0.171471	-0.38541	-0.242	-0.36758	p05709	Uncharacte	4	-0.87671				
KIAA1671 p	-0.4976	-0.56345	-0.23135	-0.31524	-0.17458	-0.14264	-0.22021	-0.32384	p02096	Uncharacte	4	-0.30861				
KIAA1522 s	0.019077	0.45921	-0.18719	-0.26656	-0.6657	-0.22159	-0.70068	-0.43951	p12935	Uncharacte	4	-0.25037		p70S6Kinh2 (DG2);		
KIAA1522 s	-0.18951	-0.23317	-0.36072	-0.1203	-0.63325	-0.10332	-0.35346	-0.63673	p20180	Uncharacte	4	-0.32881				
KIAA1522 s	-0.25596	-11.0805	-2.69982	-0.61294	-0.05348	-1.47991	-1.20209	-1.17171	p00678	Uncharacte	4	-2.31955				
KIAA1522 s	-0.24088	-0.21601	-0.21621	-0.30945	-0.63894	-0.6182	-0.786	-0.57735	p15210	Uncharacte	4	-0.45038				
KIAA1522 s	-0.02171	-0.96556	-0.12383	0.017094	-1.30944	-2.68051	0.837475	-0.56965	p19954	Uncharacte	4	-0.60202				
KIAA1522 s	0.062765	-0.54269	-0.05136	0.196339	-0.32341	-0.83112	-0.19483	-0.23149	p03404	Uncharacte	4	-0.23947		PI3Kinh2 (PI-103);		
KIAA1522 s	-0.08086	-0.35638	-0.42219	0.021896	-0.37709	-0.97779	-0.00744	-0.0285	p06549	Uncharacte	4	-0.27854		PI3Kinh2 (PI-103);		
KIAA1522 p	0.460626	-0.76619	-0.45846	-0.38433	0.496359	-0.65213	-0.45608	0.493477	p12715	Uncharacte	4	-0.15834				
KIAA1522 p	-0.2183	-0.34832	-0.19737	-0.42739	-0.09106	-0.744	-0.45115	-0.33827	p20181	Uncharacte	4	-0.35198				
KIAA1522 p	-0.00602	-0.44954	-0.03325	0.044426	-0.41572	-0.79416	-0.40258	-0.7514	p15778	Uncharacte	4	-0.35103				
KIAA1522 p	0.178662	-0.51238	-0.97631	-0.3383	-0.57192	0.243524	0.374613	0.172401	p19016	Uncharacte	4	-0.17871				
KIAA1522 p	0.008811	-0.05079	-0.1144	0.132501	-0.30831	-0.81024	-0.14231	0.025725	p00822	Uncharacte	4	-0.15738		AKTinh1 (Akt Inhibitor VIII);		
KIAA1217 p	0.161433	-2.97078	0.015329	-0.12232	0.350279	-0.61095	0.187818	0.470013	p02234	Sickle tail pr	4	-0.3149				
KIAA1211L	0.335126	0.181731	0.188363	0.173679	-0.64964	-0.44469	-0.21024	-0.32514	p20177	Uncharacte	4	-0.09385				
KIAA1143 s	-0.1417	-0.60531	0.42959	-0.23536	-0.09409	-0.37533	-0.73538	-0.72584	p09079	Uncharacte	4	-0.31043				
KIAA1143 s	-0.37537	-0.30714	0.183848	0.040666	0.26543	-0.49563	0.519181	0.308341	p00111	Uncharacte	4	0.017415				
KIAA0930 p	-0.62303	-0.73503	0.399206	-0.53277	-0.46987	-0.23453	-0.08912	-0.44287	p09311	Uncharacte	4	-0.341		ROCKinh1 (H-1152); p70S6Kinh1 (PF-4708671);		
KIAA0226 s	-0.28352	-0.62251	-0.47422	-0.38243	0.246763	-0.93525	0.018525	-0.08718	p03465	Run domair	4	-0.31498				
KIAA0101 p	-0.35266	-0.19574	0.185912	-0.02892	-0.54331	-0.35242	-0.01003	-0.29256	p15899	PCNA-assoc	4	-0.19872				
KHSRP pS1	-0.04376	-0.08751	0.026882	-0.0148	0.107501	-0.08643	-0.04268	-0.06791	p00948	Far upstrea	4	-0.02609				
KHDRBS1 s	-0.26321	-0.74827	-0.3681	-0.00014	0.126783	-0.1662	-0.49287	-0.4843	p19556	KH domain-	4	-0.29954				GO:0009967; P:positive regulation of signal transduction; GO:0007166; P:cell surface receptor signaling pathway; GO:0007165; P:signal transduction;
KHDRBS1 s	-0.21224	-0.67726	-0.3992	-0.02522	0.152364	0.01723	-0.36772	-0.03731	p19557	KH domain-	4	-0.19367		AKTinh1 (Akt Inhibitor V		GO:0009967; P:positive regulation of signal transduction; GO:0007166; P:cell surface receptor signaling pathway; GO:0007165; P:signal transduction;
KHDRBS1 s	-0.32526	-1.00667	-0.21504	-0.24485	-0.13335	-0.58438	-0.47947	-0.56767	p07783	KH domain-	4	-0.44458				GO:0009967; P:positive regulation of signal transduction; GO:0007166; P:cell surface receptor signaling pathway; GO:0007165; P:signal transduction;
KHDRBS1 s	0.160152	-0.74999	0.217766	0.316849	-0.17707	-0.36722	-0.10176	-0.1809	p05675	KH domain-	4	-0.11027				GO:0009967; P:positive regulation of signal transduction; GO:0007166; P:cell surface receptor signaling pathway; GO:0007165; P:signal transduction;
KHDRBS1 s	0.042517	0.16793	-0.76394	-0.00164	0.114327	0.132862	-0.83594	-0.08032	p05676	KH domain-	4	-0.15303				GO:0009967; P:positive regulation of signal transduction; GO:0007166; P:cell surface receptor signaling pathway; GO:0007165; P:signal transduction;
KHDRBS1 s	0.057888	0.197954	-0.71921	-0.03136	0.080689	0.136301	-0.81742	-0.04396	p05677	KH domain-	4	-0.14239				GO:0009967; P:positive regulation of signal transduction; GO:0007166; P:cell surface receptor signaling pathway; GO:0007165; P:signal transduction;
KHDRBS1 s	-0.53403	-0.5322	-1.28102	-0.53998	-0.09706	-0.03904	-1.12718	-0.36441	p05679	KH domain-	4	-0.56436				GO:0009967; P:positive regulation of signal transduction; GO:0007166; P:cell surface receptor signaling pathway; GO:0007165; P:signal transduction;
KHDRBS1 s	-0.62963	-0.58085	-1.29083	-0.51861	-0.03095	0.018035	-1.08281	-0.29995	p05678	KH domain-	4	-0.55195				GO:0009967; P:positive regulation of signal transduction; GO:0007166; P:cell surface receptor signaling pathway; GO:0007165; P:signal transduction;
KDM3B sec	-0.23802	-2.01514	0.209168	-0.4261	0.011726	-0.67563	0.015021	0.286737	p03652	Lysine-spec	4	-0.35403				
KDM3B sec	-0.19056	-0.93642	0.138653	-0.13977	-0.00353	-0.39588	-0.03989	0.250182	p03651	Lysine-spec	4	-0.16465				
KDM3A sec	0.285116	0.460667	-0.14268	-0.36205	-0.31312	-0.05037	0.018515	-0.18172	p11462	Lysine-spec	4	-0.03571			Coregulatio	GO:0009755; P:hormone-mediated signaling pathway; GO:0030521; P:androgen receptor signaling pathway;
KDM1B pS	-0.19853	-0.33041	-0.15692	-0.02713	-0.62119	0.050246	-0.99822	-0.11198	p21196	Lysine-spec	4	-0.29926				
KDM1A pS	-0.24798	-0.03039	-0.10583	-0.02315	-0.84127	-0.2604	-0.39191	-0.42444	p11093	Lysine-spec	4	-0.29067		AKTinh2 (M	Notch-med	GO:0043518; P:negative regulation of DNA damage response, signal transduction by p53 class mediator;
KCTD15 sec	-0.30509	-0.56332	0.092977	0.065022	-0.41219	-0.35941	-0.6087	-1.21986	p04301	BTB/POZ dc	4	-0.41382				
KCNIP4 pS1	3.009294	-5.66696	-5.6787	1.554786	-0.75072	0.082944	1.174543	-1.98057	p15645	Kv channel-	4	-1.03192			Regulation of nuclear beta catenin signaling and target gene transcription;	
KAT7 seq: 4	-0.0127	-0.72375	0.229424	-0.1296	-0.48558	-1.52629	0.306267	-0.63719	p10690	Histone ace	4	-0.37243	PLK1;		Regulation of Androgen receptor activity;	
KAT6A pS1	0.394572	0.350131	-0.064	-0.60854	-0.42487	-0.14191	-1.06026	-0.41996	p12578	Histone ace	4	-0.24686				
JUP pT54	-0.03082	0.450162	0.461568	-0.56562	-0.4315	-0.98848	-0.27784	-0.60286	p07662	Junction pla	4	-0.24817			E-cadherin	GO:0090263; P:positive regulation of canonical Wnt signaling pathway;
JUN pS243	-0.35991	1.470504	0.180535	-0.08563	-1.07386	-0.42081	-0.69429	-0.81823	p11038	Transcriptic	4	-0.22521	GSK3B; CK2	Up with IGF	JNK signalir	GO:0060395; P:SMAD protein signal transduction; GO:0051403; P:stress-activated MAPK cascade; GO:0034166; P:toll-like receptor 10 signaling pathway; GO:0034146; P:toll-like receptor 5 signaling pathway; GO:0038124; P:toll-like receptor TLR6:TLR2 signaling pathway; GO:0038123; P:toll-like receptor TLR1:TLR2 signaling pathway; [illegible]
JPH1 pS16	0.181299	-7.5793	0.668836	-7.41546	-0.00956	-0.21883	-1.07151	-0.37739	p02171	Junctophilin	4	-1.97774				
JOSD1 seq:	-2.31424	-2.73054	-2.26226	-0.60886	-0.04862	-0.56891	-0.24481	-1.78767	p20375	Josephin-1	4	-1.32074				
JMJD1C sec	-0.01274	0.214674	-1.42879	-2.71152	0.187049	0.188986	0.076055	-0.00015	p03777	Probable Jn	4	-0.4358				
ITSN2 seq:	-0.42317	-0.51299	-0.32117	-0.26568	1.017834	0.162504	0.480568	0.206627	p14146	Intersectin-	4	0.043066			Regulation (	GO:0009967; P:positive regulation of signal transduction;
ITSN2 seq:	-0.85466	-0.35091	-0.82813	-0.79014	0.446002	0.149132	-0.64113	0.077919	p08353	Intersectin-	4	-0.34899			Regulation (	GO:0009967; P:positive regulation of signal transduction;
ITSN2 pS21	-0.01246	-0.4937	0.029432	0.026971	-0.00635	-0.25365	0.26225	0.218515	p19766	Intersectin-	4	-0.02862			Regulation (	GO:0009967; P:positive regulation of signal transduction;
ITSN1 seq:	-0.67227	-0.72269	-0.61312	-0.71705	-0.04368	0.136756	-0.01819	0.054736	p03450	Intersectin-	4	-0.32444			Regulation (	GO:0051897; P:positive regulation of protein kinase B signaling; GO:0051056; P:regulation of small GTPase mediated signal transduction; GO:0048011; P:neurotrophin TRK receptor signaling pathway; GO:0007264; P:small GTPase mediated signal transduction;
ITSN1 seq:	0.339659	0.137202	0.248739	0.270235	-1.4474	-1.54031	-1.537	-1.59717	p05693	Intersectin-	4	-0.64076			Regulation (	GO:0051897; P:positive regulation of protein kinase B signaling; GO:0051056; P:regulation of small GTPase mediated signal transduction; GO:0048011; P:neurotrophin TRK receptor signaling pathway; GO:0007264; P:small GTPase mediated signal transduction;
ITSN1 seq:	-0.40822	-0.72502	-0.339	-0.54835	0.21348	-1.18704	-1.27975	-1.35829	p19768	Intersectin-	4	-0.70402			Regulation (	GO:0051897; P:positive regulation of protein kinase B signaling; GO:0051056; P:regulation of small GTPase mediated signal transduction; GO:0048011; P:neurotrophin TRK receptor signaling pathway; GO:0007264; P:small GTPase mediated signal transduction;
ITGB4 seq:	-1.25265	-0.49974	-2.28417	-0.80157	-0.58714	-0.81355	-0.33468	-1.05354	p01146	Integrin bet	4	-0.95338			Alpha6 beta	GO:0007229; P:integrin-mediated signaling pathway;
ITGB4 seq:	-0.36036	-0.43916	0.239449	0.119344	-0.13535	-0.58588	0.106627	0.169249	p10218	Integrin bet	4	-0.11076		PKCinh1 (G	Alpha6 beta	GO:0007229; P:integrin-mediated signaling pathway;
ITGB4 pS10	0.223836	-0.36784	-0.19129	0.322026	-0.41068	-0.10882	0.010087	-0.03512	p11416	Integrin bet	4	-0.06972			Alpha6 beta	GO:0007229; P:integrin-mediated signaling pathway;
ITGB3BP se	-0.48488	0.360617	-0.18712	-0.0424	-0.29457	-0.09578	-0.52041	-0.37025	p03327	Centromere	4	-0.20435				GO:0048011; P:neurotrophin TRK receptor signaling pathway; GO:0007165; P:signal transduction;
IRS2 seq: 5	-0.09385	0.328025	0.121704	0.095713	-1.19143	-1.02191	-1.20952	-1.34053	p07970	Insulin rece	4	-0.53897			IGF1 pathw	GO:0060397; P:JAK-STAT cascade involved in growth hormone signaling pathway; GO:0048015; P:phosphatidylinositol-mediated signaling; GO:0008286; P:insulin receptor signaling pathway; GO:0008543; P:fibroblast growth factor receptor signaling pathway; GO:0007173; P:epidermal growth factor receptor signaling pathway; GO:0048011; [illegible]
IRS2 seq: 5	-0.24014	0.599193	0.082164	0.16096	-1.30489	-1.02258	-1.55116	-1.06562	p07972	Insulin rece	4	-0.54276			IGF1 pathw	GO:0060397; P:JAK-STAT cascade involved in growth hormone signaling pathway; GO:0048015; P:phosphatidylinositol-mediated signaling; GO:0008286; P:insulin receptor signaling pathway; GO:0008543; P:fibroblast growth factor receptor signaling pathway; GO:0007173; P:epidermal growth factor receptor signaling pathway; GO:0048011; [illegible]
IRS2 pS59	-0.04818	0.43763	-0.11532	0.002334	-1.26759	-1.15707	-1.46263	-1.29328	p07971	Insulin rece	4	-0.61301			IGF1 pathw	GO:0060397; P:JAK-STAT cascade involved in growth hormone signaling pathway; GO:0048015; P:phosphatidylinositol-mediated signaling; GO:0008286; P:insulin receptor signaling pathway; GO:0008543; P:fibroblast growth factor receptor signaling pathway; GO:0007173; P:epidermal growth factor receptor signaling pathway; GO:0048011; [illegible]
IRS2 pS57	0.164555	0.295881	-0.13415	-0.01003	-0.42591	-0.63259	-0.95347	-0.70632	p01864	Insulin rece	4	-0.30025			IGF1 pathw	GO:0060397; P:JAK-STAT cascade involved in growth hormone signaling pathway; GO:0048015; P:phosphatidylinositol-mediated signaling; GO:0008286; P:insulin receptor signaling pathway; GO:0008543; P:fibroblast growth factor receptor signaling pathway; GO:0007173; P:epidermal growth factor receptor signaling pathway; GO:0048011; [illegible]
IRS1 seq: 5	0.046308	0.126875	0.076619	-0.02572	-1.31731	-0.21497	-0.52745	-1.0827	p02751	Insulin rece	4	-0.36479			S1P2 pathw	GO:0048009; P:insulin-like growth factor receptor signaling pathway; GO:0014065; P:phosphatidylinositol 3-kinase signaling; GO:0046627; P:negative regulation of insulin receptor signaling pathway; GO:0060397; P:JAK-STAT cascade involved in growth hormone signaling pathway; GO:0043552; P:positive regulation of phosphatidylinositol 3-kina [illegible]
IRF2BPL se	-0.63578	-1.1982	-0.0514	-0.40659	-1.74867	-4.0215	0.209047	-1.22363	p07008	Interferon r	4	-1.13459				
IRF2BPL pS	-0.06258	-0.68811	0.379404	-0.00505	-0.28828	-1.36082	0.157508	-0.29006	p09068	Interferon r	4	-0.26975				
IRF2BP2 pS	0	0	0	0	-0.5956	-0.37959	-0.31346	-0.32763	p20534	Interferon r	4	-0.20203				
IRF2BP2 pS	0.139664	-0.32933	0.207172	0.021376	-0.73081	-1.01095	0.424898	-0.06604	p09133	Interferon r	4	-0.168				
IRF2BP1 se	-1.8341	-0.07006	-0.44846	-1.48844	0.266747	0.167539	0.360796	0.133622	p09612	Interferon r	4	-0.36404				
IRF2BP1 pS	-0.28908	-0.73993	-0.27093	-0.11691	-0.72526	-1.05801	-0.55085	-1.04836	p04625	Interferon r	4	-0.59992				
IRF2BP1 pS	-0.03492	-0.69448	0.294991	0.257439	-0.30635	-0.83529	0.393138	0.271772	p11111	Interferon r	4	-0.08171				
IQSEC1 seq	-0.06744	-0.51676	-0.44661	-0.41215	-0.11019	0.658647	-1.70577	2.598874	p10995	IQ motif an	4	-0.00018			Plexin-D1 S	GO:0032012; P:regulation of ARF protein signal transduction;
IQSEC1 pS1	-0.20924	-0.11136	-0.40101	-0.50482	-3.34069	-0.54646	0.152934	0.099639	p09935	IQ motif an	4	-0.60763		ROCKinh2 (	Plexin-D1 S	GO:0032012; P:regulation of ARF protein signal transduction;
IQGAP1 sec	-0.43116	3.005682	-0.0736	-0.30595	-2.08293	-3.14135	-0.67207	-1.27659	p13785	Ras GTPase	4	-0.62225			Nectin adhe	GO:0045860; P:positive regulation of protein kinase activity; GO:0007264; P:small GTPase mediated signal transduction; GO:0007165; P:signal transduction;
INTS5 seq:	-1.2813	-1.40651	-1.99703	-0.94036	0	0	0	0	p11751	Integrator c	4	-0.70315				
INTS12 seq	-0.06348	-0.63121	0.048127	0.014632	-0.45546	0.192792	-0.91559	-0.46188	p11970	Integrator c	4	-0.28401				
INPP5F seq	-0.00825	-0.03257	-0.19009	-0.85847	-0.13032	0.013244	-0.2463	-0.49812	p04183	Phosphatid	4	-0.24386				GO:0048015; P:phosphatidylinositol-mediated signaling;
INPP5F pS9	-0.16431	-0.12294	-0.27323	-0.83824	0.035741	0.009147	0.062759	0.000187	p04184	Phosphatid	4	-0.16136				GO:0048015; P:phosphatidylinositol-mediated signaling;
ING3 seq: 2	-0.5871	-0.62575	-0.27343	-0.15816	0.392507	-0.0117	-0.15128	-0.32179	p19432	Inhibitor of	4	-0.21709				
INCENP seq	-0.25544	-1.04688	0.596946	0.239741	0.146839	-0.17785	-0.01888	-0.6754	p01275	Inner centro	4	-0.14887		ERKinh1 (EI	Aurora C signaling; Aurora B signaling; PLK1 signaling events; Regulation of nuclear beta catenin signaling and target gene transcription;	
INCENP seq	-0.21221	-0.94221	0.447261	0.031521	0.146456	-0.08381	0.000553	-0.22771	p01276	Inner centro	4	-0.10502		ERKinh1 (EI	Aurora C signaling; Aurora B signaling; PLK1 signaling events; Regulation of nuclear beta catenin signaling and target gene transcription;	
INCENP pS	0.183533	-0.20407	0.36268	0.052308	-0.30577	-0.26638	-0.29348	-0.58505	p02946	Inner centro	4	-0.13203			Aurora C signaling; Aurora B signaling; PLK1 signaling events; Regulation of nuclear beta catenin signaling and target gene transcription;	
IMPDH2 se	-0.60625	0.308604	-0.7573	-0.98135	0.986665	0.439994	0.903401	0.254677	p18655	Inosine-5'-r	4	0.068556				
ILF3 seq: 6	-0.74252	-0.73505	-0.77041	-0.35893	-0.12799	-0.10452	0.004821	-0.04493	p14289	Interleukin	4	-0.35994				
ILF3 seq: 4	-1.03171	-1.21478	-1.57922	-1.67731	0.157502	-0.11676	0.054746	-0.15715	p11171	Interleukin	4	-0.69558				
IGF2BP2 se	-0.22501	-2.36248	1.030819	-0.25064	0.337584	0.229014	0.169124	0.173764	p13627	Insulin-like	4	-0.11223				
IFT81 seq: 2	-0.14722	-0.32138	-0.00874	0.006499	0.225588	-0.17758	-0.2199	-0.16199	p14476	Intraflagella	4	-0.10059				
IFI16 pS15	-1.27394	-7.36399	1.68388	0.541625	0.251561	0.242081	0.341303	0.100818	p00870	Gamma-inte	4	-0.68458				
IDH1 seq: 2	-0.28628	-0.48128	-0.66909	-0.50871	-0.29929	-0.26502	0.508607	-0.0727	p08926	Isocitrate d	4	-0.25922				
IDH1 seq: 2	-0.29281	-0.25691	0.128281	-0.20989	0.848494	-0.762	-1.49686	1.104159	p06028	Isocitrate d	4	-0.11719				
ID2B pS5	-0.19298	-1.15463	-0.47088	-0.59622	0	0	0	0	p20745	Putative DN	4	-0.30184				
ICE1 pS925	0.055636	0.076757	-0.15599	0.410505	-0.23031	-0.16793	-0.37032	-0.61328	p05465	Little elonga	4	-0.12437				
HUWE1 sec	-11.31	0.083353	0.149949	0.335288	0	0	0	0	p04844	E3 ubiquitir	4	-1.34267			p53 pathway; Validated targets of C-MYC transcriptional activation;	
HUWE1 sec	-0.22694	-0.73057	-0.41229	-0.26183	-0.32626	-0.61591	-0.44243	-0.28902	p20552	E3 ubiquitir	4	-0.41316			p53 pathway; Validated targets of C-MYC transcriptional activation;	
HUWE1 sec	0.09144	0.77078	-0.23539	0.041541	-0.81461	-0.52635	-2.12875	0.03354	p20551	E3 ubiquitir	4	-0.34598			p53 pathway; Validated targets of C-MYC transcriptional activation;	
HUWE1 pS	-0.42773	-0.93974	-0.24479	0.134208	-0.31126	-0.16209	-0.46264	-0.34401	p11684	E3 ubiquitir	4	-0.34501			p53 pathway; Validated targets of C-MYC transcriptional activation;	
HSPH1 seq	-1.17368	-0.08646	-0.99242	-1.37813	0.325251	0.431728	-1.38599	0.624389	p10146	Heat shock	4	-0.45441				
HSPD1 seq	-0.7116	-0.62902	-0.85146	-0.67826	-0.09248	0.113369	-0.08327	-0.46128	p01495	60 kDa heat	4	-0.42425			Endogenou	GO:0002755; P:MyD88-dependent toll-like receptor signaling pathway;
HSPD1 seq	-0.65585	-0.69543	-1.31729	-1.01293	1.055169	0.62619	0.338742	0.364246	p16285	60 kDa heat	4	-0.16214			Endogenou	GO:0002755; P:MyD88-dependent toll-like receptor signaling pathway;
HSPB8 pS2	-0.2159	-0.56169	-0.09789	-0.20466	-0.96488	-1.25896	-2.67164	-1.07168	p18407	Heat shock	4	-0.88091	ERK1;			
HSPB8 pS2	-0.41613	-0.13131	-0.56871	-0.28367	-0.52211	-1.1214	-2.25583	-1.24533	p07526	Heat shock	4	-0.81806	ERK1;			
HSPB1 seq:	-0.77656	0.238396	-0.47925	-0.83035	0.702966	0	0	0	p19791	Heat shock	4	-0.1431			p38 signali	GO:0043122; P:regulation of I-kappaB kinase/NF-kappaB signaling; GO:0035924; P:cellular response to vascular endothelial growth factor stimulus; GO:0006469; P:negative regulation of protein kinase activity; GO:0035556; P:intracellular signal transduction;
HSPB1 seq:	-0.98372	0.861383	-1.24116	-1.44826	0	0	0	0	p12328	Heat shock	4	-0.35147			p38 signali	GO:0043122; P:regulation of I-kappaB kinase/NF-kappaB signaling; GO:0035924; P:cellular response to vascular endothelial growth factor stimulus; GO:0006469; P:negative regulation of protein kinase activity; GO:0035556; P:intracellular signal transduction;
HSPB1 seq:	-0.96315	0.314692	-1.35828	-2.03749	0	0	0	0	p02674	Heat shock	4	-0.50553			p38 signali	GO:0043122; P:regulation of I-kappaB kinase/NF-kappaB signaling; GO:0035924; P:cellular response to vascular endothelial growth factor stimulus; GO:0006469; P:negative regulation of protein kinase activity; GO:0035556; P:intracellular signal transduction;
HSPB1 pS8	0.11367	0.979537	0.095566	0.417434	-0.53771	-1.29426	-1.7144	-1.8483	p08280	Heat shock	4	-0.47356	PKG1 iso2; PKD1; PKAC		p38 signali	GO:0043122; P:regulation of I-kappaB kinase/NF-kappaB signaling; GO:0035924; P:cellular response to vascular endothelial growth factor stimulus; GO:0006469; P:negative regulation of protein kinase activity; GO:0035556; P:intracellular signal transduction;
HSPA9 seq:	-0.53399	0.111038	-0.74636	-0.91131	-2.75713	0.150517	0.881616	0.828885	p17269	Stress-70 p	4	-0.37209				
HSPA8 seq:	-0.15941	-0.28305	-0.19955	-0.51119	0.347235	0.209468	0.093522	-0.09445	p09206	Heat shock	4	-0.07468			Regulation of nuclear SMAD2/3 signaling; C-MYB transcription factor network;	
HSPA8 seq:	-0.17032	1.243109	-0.39615	-0.4141	-1.31015	-0.35634	-0.45419	-0.15807	p08997	Heat shock	4	-0.25203			Regulation of nuclear SMAD2/3 signaling; C-MYB transcription factor network;	
HSPA8 seq:	0.136846	-0.00639	-0.97371	-1.24228	-1.54241	0.551184	-0.2171	1.262443	p15530	Heat shock	4	-0.25393			Regulation of nuclear SMAD2/3 signaling; C-MYB transcription factor network;	
HSPA8 seq:	-1.03702	-0.84653	-1.55484	-1.84057	0.611231	-0.15793	-0.25737	0.909141	p10055	Heat shock	4	-0.52174			Regulation of nuclear SMAD2/3 signaling; C-MYB transcription factor network;	
HSPA8 pM	-0.70636	-0.24641	-0.25621	-0.45348	0.680769	0.544039	0.233594	0.761887	p09205	Heat shock	4	0.069728			Regulation of nuclear SMAD2/3 signaling; C-MYB transcription factor network;	
HSPA5 seq:	-0.49844	0.438084	-0.69776	-0.73949	0.563937	-0.5189	-2.87242	-0.61509	p09222	78 kDa gluc	4	-0.61751				GO:0006987; P:activation of signaling protein activity involved in unfolded protein response; GO:0030512; P:negative regulation of transforming growth factor beta receptor signaling pathway;
HSPA5 seq:	-0.21563	0.11925	-0.68983	-0.819	0.24994	-0.23535	-0.43324	0.133999	p09223	78 kDa gluc	4	-0.23623				GO:0006987; P:activation of signaling protein activity involved in unfolded protein response; GO:0030512; P:negative regulation of transforming growth factor beta receptor signaling pathway;
HSPA5 seq:	-0.72551	0.92392	-0.90819	-1.48966	0.504616	-0.1063	-0.4151	0.375169	p09821	78 kDa gluc	4	-0.23013				GO:0006987; P:activation of signaling protein activity involved in unfolded protein response; GO:0030512; P:negative regulation of transforming growth factor beta receptor signaling pathway;
HSPA5 seq:	-0.59829	0.353689	-0.64959	-0.91322	0.2286	0.813468	0.316215	0.852457	p00541	78 kDa gluc	4	0.050417				GO:0006987; P:activation of signaling protein activity involved in unfolded protein response; GO:0030512; P:negative regulation of transforming growth factor beta receptor signaling pathway;
HSPA5 pM	-0.85751	0.044897	-0.2741	-0.67288	0.058562	0.83185	0.364926	0.852754	p18980	78 kDa gluc	4	0.043563				GO:0006987; P:activation of signaling protein activity involved in unfolded protein response; GO:0030512; P:negative regulation of transforming growth factor beta receptor signaling pathway;
HSPA4 seq:	-2.72301	1.700174	-1.76933	-2.68252	-0.65992	0.448632	-0.76573	1.361132	p09737	Heat shock	4	-0.63632			Validated targets of C-MYC transcriptional activation;	
HSPA4 seq:	0.080316	2.804239	0.789357	-7.52408	-1.17187	-0.11662	0.401795	0.336691	p16631	Heat shock	4	-0.55002			Validated targets of C-MYC transcriptional activation;	
HSPA1B se	-1.09733	0.537537	-0.77876	-1.20835	0.27452	0.379392	-0.62056	0.341547	p13247	Heat shock	4	-0.2715				
HSPA1A se	-1.23943	-0.96753	-1.27291	-1.00992	0.180479	0.538957	0.486669	0.884563	p09207	Heat shock	4	-0.29989				
HSPA1A se	-0.08195	-0.20051	-2.9676	-0.30909	-0.03437	-0.64672	-0.38693	-0.26428	p09818	Heat shock	4	-0.61143				
HSP90B1 s	-9.92472	0.714904	0.554684	-0.77211	0	0	0	0	p10753	Endoplasmi	4	-1.17841			IL6-mediate	GO:0006987; P:activation of signaling protein activity involved in unfolded protein response; GO:0002224; P:toll-like receptor signaling pathway;
HSP90B1 s	-0.92246	-1.2153	0.658033	-1.95559	-1.13924	-2.13218	-3.71257	-1.74305	p18712	Endoplasmi	4	-1.5203			IL6-mediate	GO:0006987; P:activation of signaling protein activity involved in unfolded protein response; GO:0002224; P:toll-like receptor signaling pathway;
HSP90B1 s	-1.41497	-0.98015	-2.79154	-1.76844	1.21914	0	0	0	p15754	Endoplasmi	4	-0.71699			IL6-mediate	GO:0006987; P:activation of signaling protein activity involved in unfolded protein response; GO:0002224; P:toll-like receptor signaling pathway;
HSP90B1 s	-0.69013	-1.5555	-0.8955	-0.98785	-0.16258	0.824295	1.249303	1.01296	p02870	Endoplasmi	4	-0.15063			IL6-mediate	GO:0006987; P:activation of signaling protein activity involved in unfolded protein response; GO:0002224; P:toll-like receptor signaling pathway;
HSP90AA1	-0.53453	-0.03755	-0.54385	-0.93673	0.390539	0.52687	-0.13515	0.308535	p18361	Heat shock	4	-0.12023			ErbB recept	GO:0038096; P:Fc-gamma receptor signaling pathway involved in phagocytosis; GO:0007165; P:signal transduction;
HSD17B10	0.592467	0.694487	0.518781	0.689014	-2.11896	-2.10191	-0.9768	-1.91384	p11728	3-hydroxya	4	-0.57709				
HSD17B10	-0.328	-0.05236	-0.20005	-0.02528	-0.21285	0.626984	-0.97827	-0.92862	p12975	3-hydroxya	4	-0.26231				
HSD17B10	-0.22706	0.114353	-0.29469	-1.28181	0	0	0	0	p15031	3-hydroxya	4	-0.21115				
HPS4 seq: 3	-1.24302	-0.44838	-1.30665	-0.92166	-0.82889	-1.39093	-1.0938	-1.74189	p16147	Hermansky	4	-1.1219				
HPGD seq:	1.844589	1.182415	-7.50132	0.245475	0	0	0	0	p12079	15-hydroxy	4	-0.52861				GO:0007179; P:transforming growth factor beta receptor signaling pathway;
HP1BP3 se	-0.35045	-0.76443	-0.48589	-0.52752	-0.50871	-0.94629	-0.67522	-5.88035	p07787	Heterochro	4	-1.26736				
HP1BP3 se	-0.426	-0.57533	-0.70945	-0.55365	-0.45089	-1.01134	-0.45999	-0.6877	p07789	Heterochro	4	-0.6093				
HOOK1 seq	0.1321	-0.13294	-1.21819	-0.54888	-0.25045	-0.34301	0.152998	-0.28934	p14118	Protein Hoc	4	-0.31221				
HOMEZ pT	-0.59048	-0.93272	-0.13669	-0.21816	-0.09175	0.061338	0.147086	-0.09666	p20793	Homeobox	4	-0.23225				
HNRNPUL1	0.548818	1.041362	0.075808	-1.86954	-1.81438	-0.4066	-4.44527	-0.82078	p09733	Heterogene	4	-0.96132				
HNRNPU se	-0.72677	-0.66943	-0.79655	-0.92724	0.415952	1.025398	-0.15809	1.179738	p18564	Heterogene	4	-0.08212				
HNRNPU se	-1.88285	-0.50839	-1.07252	-2.73957	-0.59647	0.486909	-4.07343	0.676417	p13361	Heterogene	4	-1.21374				
HNRNPU se	-0.35708	0.302512	-0.37	-1.53641	0.216595	0.051408	0.072364	0.230409	p09643	Heterogene	4	-0.17377				
HNRNPU p	-0.41741	-0.7533	-0.11549	-0.058	-0.15472	-0.28173	-0.53401	-0.24177	p03889	Heterogene	4	-0.31955		PI3Kinh2 (PI-103); ERKinh2 (ERK inhibitor II); AKTinh2 (MK-2206);		
HNRNPU pl	-0.09067	-3.43407	0.539795	0.485705	0	0	0	0	p11060	Heterogene	4	-0.31241	DNAPK;			
HNRNPM p	-0.04028	-0.23019	-0.53803	-2.3074	0	0	0	0	p10111	Heterogene	4	-0.38949				
HNRNPM p	-0.02649	-0.08509	-0.16967	-0.81097	-0.14008	0.453032	-0.64539	-0.51145	p10117	Heterogene	4	-0.24201				
HNRNPM p	-1.75105	0.786276	-3.08872	-1.43367	-2.25396	1.236646	-2.55191	-0.85111	p10103	Heterogene	4	-1.23844				
HNRNPL pS	-0.3647	-0.54299	-0.1617	-0.11826	-0.09319	0.035072	-0.2359	-0.40513	p00009	Heterogene	4	-0.23585				
HNRNPH3	-0.58806	-0.59238	-0.39065	-0.3198	0.04156	0.156385	-0.03066	-0.06728	p18808	Heterogene	4	-0.22386				
HNRNPF se	-1.06911	-1.26878	-2.49588	0.206296	1.765068	1.722525	2.429975	-11.4177	p07912	Heterogene	4	-1.26595				
HNRNPDL	-1.02439	0.479044	-0.30794	-3.42744	0.684687	0.473365	0.317812	0.170968	p18999	Heterogene	4	-0.32924				
HNRNPC se	-1.28269	-0.1168	0.209572	-0.48789	0.530698	-0.04962	0.824785	0.91515	p17988	Heterogene	4	0.0679	CK1A;		Regulation of Telomerase;	
HNRNPC se	-0.65874	-0.7577	-0.98053	-0.9746	0.194068	0.192482	-0.65967	-0.07085	p11509	Heterogene	4	-0.46444			Regulation of Telomerase;	
HNRNPC p	-0.1261	-0.32728	0.165845	-0.59261	0.190558	-0.1496	0.129905	-0.04697	p08795	Heterogene	4	-0.09453			Regulation of Telomerase;	
HNRNPA3	-0.56935	-0.5514	-0.6979	-1.04066	0.202973	-0.00059	0.254091	-0.28215	p17863	Heterogene	4	-0.33562				
HNRNPA2B	-1.24491	-0.66886	-1.34591	-1.30261	-1.01256	0.478513	-0.82131	-0.9747	p10576	Heterogene	4	-0.86154				
HNRNPA2B	-0.26719	0.77954	-0.94615	-1.88504	-0.54524	0.64864	-0.00719	1.456009	p16119	Heterogene	4	-0.09583				
HNRNPA1	0.019271	0.358218	-0.23473	-0.25518	-0.73491	-0.2798	-0.80057	-0.7175	p16059	Heterogene	4	-0.33065			Coregulation of Androgen receptor activity;	
HN1L seq:	-0.35709	0.750917	-0.2554	-1.41816	0.361002	0.44347	-0.41635	-0.35935	p18774	Hematologi	4	-0.15637				
HN1L pS97	-0.5414	-0.65578	-0.18968	-0.28108	-0.38902	-0.28263	-0.34507	-0.376	p02192	Hematologi	4	-0.38258	CDK2;			
HN1 pT54	-0.26354	-0.22162	0.096719	-0.02518	-1.09675	-0.97013	-0.9391	-1.55288	p10241	Hematologi	4	-0.62156		PI3Kinh2 (F	Regulation (	GO:0035584; P:calcium-mediated signaling using intracellular calcium source; GO:0007218; P:neuropeptide signaling pathway; GO:0051056; P:regulation of small GTPase mediated signal transduction; GO:0007186; P:G-protein coupled receptor signaling pathway; GO:0007264; P:small GTPase mediated signal transduction; GO:0007165; P:signal t [illegible]
HN1 pT54	-0.13442	-0.17525	0.155733	-0.13052	-0.58184	-0.50204	-0.46682	-0.58965	p10242	Hematologi	4	-0.3031		PI3Kinh2 (F	Regulation (	GO:0035584; P:calcium-mediated signaling using intracellular calcium source; GO:0007218; P:neuropeptide signaling pathway; GO:0051056; P:regulation of small GTPase mediated signal transduction; GO:0007186; P:G-protein coupled receptor signaling pathway; GO:0007264; P:small GTPase mediated signal transduction; GO:0007165; P:signal t [illegible]
HN1 pS31	-0.38464	-0.75894	-0.36154	-0.73219	-0.93478	-0.8505	-0.61786	-0.5302	p01183	Hematologi	4	-0.64633			Regulation (	GO:0035584; P:calcium-mediated signaling using intracellular calcium source; GO:0007218; P:neuropeptide signaling pathway; GO:0051056; P:regulation of small GTPase mediated signal transduction; GO:0007186; P:G-protein coupled receptor signaling pathway; GO:0007264; P:small GTPase mediated signal transduction; GO:0007165; P:signal t [illegible]
HN1 pS31	-0.29946	-0.7285	-0.3061	-0.67175	-0.43656	-0.996	-0.41711	-0.42531	p01182	Hematologi	4	-0.5351			Regulation (	GO:0035584; P:calcium-mediated signaling using intracellular calcium source; GO:0007218; P:neuropeptide signaling pathway; GO:0051056; P:regulation of small GTPase mediated signal transduction; GO:0007186; P:G-protein coupled receptor signaling pathway; GO:0007264; P:small GTPase mediated signal transduction; GO:0007165; P:signal t [illegible]
HN1 pM47	-0.76419	-1.18231	-0.27842	-0.50996	-0.09411	-0.66432	-0.84374	-1.11418	p10243	Hematologi	4	-0.6814		PI3Kinh2 (F	Regulation (	GO:0035584; P:calcium-mediated signaling using intracellular calcium source; GO:0007218; P:neuropeptide signaling pathway; GO:0051056; P:regulation of small GTPase mediated signal transduction; GO:0007186; P:G-protein coupled receptor signaling pathway; GO:0007264; P:small GTPase mediated signal transduction; GO:0007165; P:signal t [illegible]
HMGXB4 s	0.408109	-0.65584	0.456582	0.408767	-0.74652	-0.52882	-0.34486	-0.8577	p20418	HMG doma	4	-0.23254				GO:0030178; P:negative regulation of Wnt signaling pathway; GO:0016055; P:Wnt signaling pathway;
HMGN2 pS	-0.11688	-0.67201	0.056149	0.010217	0.035817	-0.07057	0.169721	-0.11557	p10842	Non-histon	4	-0.08789	AurB;			
HMGCS2 se	-1.05664	-1.24222	-1.47915	-1.42335	0	0	0	0	p19973	Hydroxyme	4	-0.65017			Validated targets of C-MYC transcriptional repression;	
HMGA1 se	0.050269	-0.55365	0.496761	0.150435	0.074921	-0.15329	0.105388	-0.1356	p12664	High mobili	4	0.004404	HIPK2; CDK	ROCKinh1 (	IL4-mediated signaling events; Validated targets of C-MYC transcriptional activation;	
HMGA1 pT	0	0	0	0	-0.3473	-0.19984	0.638946	-1.35848	p17300	High mobili	4	-0.15833	HIPK2; CDK1;		IL4-mediated signaling events; Validated targets of C-MYC transcriptional activation;	
HMGA1 pS	0.024545	-0.65396	0.447305	0.055734	0.112217	-0.19878	0.049584	-0.15443	p12663	High mobili	4	-0.03972	HIPK2; CDK	ROCKinh1 (	IL4-mediated signaling events; Validated targets of C-MYC transcriptional activation;	
HMG20A p	0.161008	0.182733	0.211794	0.364526	-0.24893	-0.33165	-0.32379	-0.52791	p18238	High mobili	4	-0.06403				
HJURP seq:	-0.83216	-1.69289	-0.54815	-0.31327	0.575437	0.502882	0.244504	0.017193	p05767	Holliday jur	4	-0.25581				
HJURP pS1	-1.28553	-0.08416	-0.25121	0.452994	-0.2694	-0.52251	-0.62539	-1.72333	p00693	Holliday jur	4	-0.53857				
HIVEP1 seq	0.490038	0.519229	0.417235	0.62812	-2.07268	-1.7784	-2.04294	-1.43757	p13635	Zinc finger p	4	-0.65962		p70S6Kinh2 (DG2); mTORinh2 (Torin-1);		
HIVEP1 pS1	0.232061	-0.01885	-0.09778	-0.32396	0.911727	-1.4132	-0.57362	-1.73201	p01394	Zinc finger p	4	-0.37695				
HIST3H3 se	-0.44179	-0.41598	-0.77152	-0.83425	1.286448	0.660116	0.624677	0.17215	p17634	Histone H3	4	0.034982				
HIST2H2AC	-0.5869	-0.37861	-0.68776	-1.04061	-0.01955	0.433324	0.311021	0.003095	p09716	Histone H2	4	-0.24575				
HIST1H4A s	-0.37491	-0.19756	-0.4594	-1.04384	0.445417	-0.04535	-0.19524	-0.11703	p18938	Histone H4	4	-0.24849			Signaling ev	GO:0043408; P:regulation of MAPK cascade; GO:0032012; P:regulation of ARF protein signal transduction; GO:0030522; P:intracellular receptor signaling pathway; GO:0007218; P:neuropeptide signaling pathway; GO:0048015; P:phosphatidylinositol-mediated signaling; GO:0035556; P:intracellular signal transduction; GO:0007165; P:signal trans [illegible]
HIST1H4A s	0.493437	-0.51174	-0.73838	-0.97827	0.702101	0.254344	0.182454	-0.2117	p18939	Histone H4	4	-0.10097			Signaling ev	GO:0043408; P:regulation of MAPK cascade; GO:0032012; P:regulation of ARF protein signal transduction; GO:0030522; P:intracellular receptor signaling pathway; GO:0007218; P:neuropeptide signaling pathway; GO:0048015; P:phosphatidylinositol-mediated signaling; GO:0035556; P:intracellular signal transduction; GO:0007165; P:signal trans [illegible]
HIST1H4A s	-0.72475	-0.37422	-0.78465	-1.15381	0.771045	0.345152	0.131072	-0.00546	p13734	Histone H4	4	-0.22445			Signaling ev	GO:0043408; P:regulation of MAPK cascade; GO:0032012; P:regulation of ARF protein signal transduction; GO:0030522; P:intracellular receptor signaling pathway; GO:0007218; P:neuropeptide signaling pathway; GO:0048015; P:phosphatidylinositol-mediated signaling; GO:0035556; P:intracellular signal transduction; GO:0007165; P:signal trans [illegible]
HIST1H2AJ	-0.64519	-0.1353	-0.7418	-1.21177	0.088754	0.340181	0.115405	0.305561	p09715	Histone H2	4	-0.23552				
HIST1H1D	-0.96116	-1.52058	-2.81068	-0.67816	-2.04391	-2.07147	-1.7485	-1.8851	p13434	Histone H1	4	-1.71494			Aurora B sig	GO:0043123; P:positive regulation of I-kappaB kinase/NF-kappaB signaling; GO:0007165; P:signal transduction;
HIST1H1C s	-1.30943	-1.29316	-1.48079	-1.59307	0.982291	0.24058	0.483566	0.003573	p05618	Histone H1	4	-0.4958			Canonical V	GO:0030509; P:BMP signaling pathway; GO:0016055; P:Wnt signaling pathway;
HIST1H1B s	-0.813	-1.17728	-1.5699	-0.58538	-0.64691	-0.743	0.120996	-0.50506	p20336	Histone H1	4	-0.73994				
HIST1H1B s	-0.50521	-1.02509	-1.18833	-0.44727	-0.48348	-0.67906	-0.18351	-0.56762	p20335	Histone H1	4	-0.63495				
HIST1H1B s	-1.34949	-1.25744	-1.66549	-1.5204	0.459804	-0.1915	-0.35484	-0.15136	p13373	Histone H1	4	-0.75384				
HIST1H1B s	0.01285	-0.44482	-0.2785	-0.07369	-0.32557	-0.61252	-0.28	-0.83697	p05963	Histone H1	4	-0.3549				
HIST1H1B p	0.053912	0.202444	0.070156	-0.57276	-0.71164	-0.51205	0.243599	-0.53115	p20398	Histone H1	4	-0.21969				
HIST1H1B p	-0.08291	-0.28699	-0.55893	-0.61858	-0.90796	-0.72181	-0.38773	-1.03359	p05965	Histone H1	4	-0.57481				
HIST1H1B p	-0.03748	-0.421	-0.14918	-0.26341	-0.64445	-0.64576	-0.37723	-0.89562	p05964	Histone H1	4	-0.42927				
HIST1H1B p	-0.08621	-0.57848	-0.55596	-0.36342	-0.36638	-0.37977	-0.11235	-0.69811	p05962	Histone H1	4	-0.39259				
HIRIP3 seq:	-0.00781	0.653228	-0.06191	-0.37145	-0.28583	-0.07481	-0.07346	-0.14978	p02623	HIRA-intera	4	-0.04648		PKCinh2 (Bisindolylmaleimide-1);		
HID1 seq: 5	-0.51018	-0.79181	-0.51532	-0.6026	1.527732	-3.49821	0.821105	1.404778	p02783	Protein HID	4	-0.27056				
HID1 pT58	-0.90422	-0.78336	-1.0413	-1.05953	0.393455	-1.7046	-1.11251	-2.2073	p06712	Protein HID	4	-1.05242				
HIBADH se	-0.24963	0.070308	-0.73367	-3.59518	-0.65058	-0.38814	-3.27751	-3.72563	p18607	3-hydroxyis	4	-1.56875				
HEY2 pS39	-1.21276	-1.43161	-0.64834	-0.78538	-0.68304	-0.62286	-0.95527	-0.79063	p11159	Hairy/enhar	4	-0.89124			Notch-med	GO:0045746; P:negative regulation of Notch signaling pathway; GO:0007219; P:Notch signaling pathway;
HEXB seq: 2	-0.72511	-0.56287	-1.09778	-0.91949	0.908103	0.064097	0.523103	0.637028	p15878	Beta-hexos	4	-0.14662				
HEXA seq: 1	-0.72511	-0.56287	-1.09778	-0.91949	0.908103	0.064097	0.523103	0.637028	p15709	Beta-hexos	4	-0.14662				
HES1 seq: 9	-0.1792	0.037124	-0.27145	0.177285	-0.95653	-1.24582	-1.18169	-1.13804	p09070	Transcriptic	4	-0.59479			Fanconi anc	GO:0046427; P:positive regulation of JAK-STAT cascade; GO:0045747; P:positive regulation of Notch signaling pathway; GO:0030513; P:positive regulation of BMP signaling pathway; GO:0007224; P:smoothened signaling pathway; GO:0007219; P:Notch signaling pathway;
HELZ pS16	-0.29052	-0.3788	-0.24813	-0.35842	0.621944	0.026858	-0.09062	-8.47663	p07700	Probable he	4	-1.14929				
HECTD1 pS	0.110289	-1.26729	0.183933	0.351272	-0.05223	-0.5667	0.367194	0.278377	p10593	E3 ubiquitir	4	-0.07439		ROCKinh1 (H-1152); ERKinh1 (ERK inhbitor Calbiochem); AKTinh1 (Akt Inhibitor VIII);		
HEBP2 seq:	-1.77221	0.128632	-0.70212	-1.08767	0	0	0	0	p05932	Heme-bindi	4	-0.42917				
HDGFRP2 p	-1.49168	-0.46368	-2.03729	-1.2304	-0.40587	-0.19317	-0.39151	-0.91709	p17252	Hepatoma-	4	-0.89134				
HDGFRP2 p	-0.56563	-0.00989	-0.67983	-0.15332	-0.01847	-0.18039	-0.17272	-0.22867	p19715	Hepatoma-	4	-0.25112				
HDGFRP2 p	-1.52594	-0.7383	-2.91334	-2.00351	-1.33506	-1.21096	-1.91133	-2.24049	p12620	Hepatoma-	4	-1.73486		Up with IGF - down with mTOR-PI3K; Up with EGF - down with mTOR-PI3K; Up with IGF - down with PAKinh (PF3758309); Up with EGF - down with PAKinh (PF3758309); Up with IGF - down with PI3Kinh2 (PI-103); Up with EGF - down with PI3Kinh2 (PI-103); Up with IGF - down with PI3Kinh1 (GDC-0941); Up with EGF - down with PI3Kinh1 (GDC-0941); Up with IGF - do [illegible]		
HDGFRP2 p	-0.94724	-0.03138	-2.12029	-1.28527	-0.67273	-0.60427	-0.88846	-0.66973	p12619	Hepatoma-	4	-0.90242		Up with IGF - down with mTOR-PI3K; Up with EGF - down with mTOR-PI3K; Up with IGF - down with PAKinh (PF3758309); Up with EGF - down with PAKinh (PF3758309); Up with IGF - down with PI3Kinh2 (PI-103); Up with EGF - down with PI3Kinh2 (PI-103); Up with IGF - down with PI3Kinh1 (GDC-0941); Up with EGF - down with PI3Kinh1 (GDC-0941); Up with IGF - do [illegible]		
HDGF pS16	-0.19782	-0.21246	-0.61318	-0.36456	-0.53398	-0.29499	-0.77113	-0.46671	p07634	Hepatoma-	4	-0.43185				GO:0006987; P:activation of signaling protein activity involved in unfolded protein response; GO:0007165; P:signal transduction;
HDGF pS16	-0.14462	-0.33223	-0.37329	-0.32878	-0.55693	-0.48112	-0.46066	-0.6514	p07635	Hepatoma-	4	-0.41613				GO:0006987; P:activation of signaling protein activity involved in unfolded protein response; GO:0007165; P:signal transduction;
HDAC6 pS2	-0.37092	-0.5846	-0.22931	-0.25893	-0.05042	-0.12449	-0.23514	-0.69897	p08197	Histone dea	4	-0.3191			Signaling ev	GO:0070848; P:response to growth factor; GO:0009967; P:positive regulation of signal transduction;
HDAC1 seq	-0.59614	-0.31727	-1.18631	-1.05813	-0.07242	0.238066	-1.32168	-0.48715	p13730	Histone dea	4	-0.60013			Sumoylatio	GO:0060766; P:negative regulation of androgen receptor signaling pathway; GO:0007219; P:Notch signaling pathway; GO:0007179; P:transforming growth factor beta receptor signaling pathway; GO:0048011; P:neurotrophin TRK receptor signaling pathway;
HCFC1 seq:	-0.41285	-0.69259	-0.00261	-0.07147	-0.37252	-0.22285	-0.22197	-0.59868	p01750	Host cell fa	4	-0.32444				
HCFC1 pS1	0.073298	-0.99801	-0.07646	-0.50517	0.039615	0.045107	0.209817	0.152503	p17313	Host cell fa	4	-0.13241		ERKinh1 (ERK inhbitor Calbiochem);		
HBS1L seq:	-0.35661	-0.66129	-0.15123	-0.42213	-0.18277	0.176336	0.182956	0.02511	p06308	HBS1-like p	4	-0.1737				GO:0007165; P:signal transduction;
HAPLN4 pS	0.076434	-0.53892	-0.07234	-0.08571	0.106994	-0.35857	-0.20166	0.037534	p20767	Hyaluronan	4	-0.12953				
HABP4 pS1	-0.23826	-0.81044	-0.73469	-0.27081	-0.70919	-0.42298	-0.78873	-0.87938	p05125	Intracellular	4	-0.60681				
H1FX seq: 2	-0.39565	-1.28812	-1.87113	-1.10957	-3.23642	-0.35912	-1.00305	-3.93677	p20652	Histone H1	4	-1.64998				
GYS1 seq: 7	0.140227	-1.70044	-0.67705	-0.56121	0	0	0	0	p06998	Glycogen [s	4	-0.34981			Insulin-mediated glucose transport;	
GTSE1 seq:	0.160855	-0.56263	0.490067	0.363281	0.031217	-0.32458	0.003259	-0.0418	p11466	G2 and S pl	4	0.014959				
GTF2I seq:	-0.08321	-0.61764	-0.78207	-0.22717	-1.00078	-0.98555	-0.71007	-2.02471	p12801	General trar	4	-0.8039				GO:0007165; P:signal transduction;
GTF2I seq:	-0.22993	-0.3415	-0.29401	-0.31229	-0.57226	-0.4838	-0.36882	-0.35034	p02444	General trar	4	-0.36912	CDK2;			GO:0007165; P:signal transduction;
GTF2I seq:	0.259329	-0.22856	0.219706	0.154228	-0.55853	-0.18118	-0.11998	-0.47622	p02443	General trar	4	-0.1164	CDK2;			GO:0007165; P:signal transduction;
GTF2I pS82	-0.56204	-0.48767	-0.9305	-1.17996	-0.34635	-0.13849	-0.21713	-0.77212	p12800	General trar	4	-0.57928				GO:0007165; P:signal transduction;
GTF2F1 seq	-0.45485	0.429591	-0.52918	-0.63083	-1.19938	-0.34617	-1.17407	-1.15099	p15564	General trar	4	-0.63199	GTF2F1;			
GTF2F1 pS	0.706723	-9.41367	0.871627	1.287189	0.831737	0.22512	0.445722	0.444735	p10890	General trar	4	-0.5751				
GSPT1 seq:	-0.18124	0.366519	-0.19486	-0.5445	0.275358	0.126347	0.198462	-1.24879	p15904	Eukaryotic p	4	-0.15034				
GSN seq: 3	-1.19069	-0.1947	-0.96214	-1.59347	0	0	0	0	p07770	Gelsolin	4	-0.49262			Osteoponti	GO:0048015; P:phosphatidylinositol-mediated signaling;
GSK3B seq:	0.049895	-0.46925	-0.02487	-0.15891	-1.51473	-2.03905	-2.0461	-1.60698	p02021	Glycogen sy	4	-0.97625	RSK2; PKCH	Up with IGF	Degradatior	GO:0060070; P:canonical Wnt signaling pathway; GO:0090090; P:negative regulation of canonical Wnt signaling pathway; GO:0048015; P:phosphatidylinositol-mediated signaling; GO:0008543; P:fibroblast growth factor receptor signaling pathway; GO:0007173; P:epidermal growth factor receptor signaling pathway; GO:0048011; P:neurotrophin [illegible]
GSK3A seq:	-0.21587	-0.73565	-0.17472	-0.19469	-1.30885	-2.15378	-2.13079	-1.79631	p02111	Glycogen sy	4	-1.08883	RSK2; PKCH; PKCG; PKC		Degradatior	GO:0032007; P:negative regulation of TOR signaling; GO:0046627; P:negative regulation of insulin receptor signaling pathway; GO:0006987; P:activation of signaling protein activity involved in unfolded protein response; GO:0090090; P:negative regulation of canonical Wnt signaling pathway; GO:0048015; P:phosphatidylinositol-mediated signaling; [illegible]
GSK3A pT1	-0.20535	-1.02923	-0.40923	-0.52859	-1.53201	-1.94325	-2.13098	-1.72932	p19669	Glycogen sy	4	-1.1885			Degradatior	GO:0032007; P:negative regulation of TOR signaling; GO:0046627; P:negative regulation of insulin receptor signaling pathway; GO:0006987; P:activation of signaling protein activity involved in unfolded protein response; GO:0090090; P:negative regulation of canonical Wnt signaling pathway; GO:0048015; P:phosphatidylinositol-mediated signalin [illegible]
GSG2 seq:	0	0	0	0	-8.38044	0.127322	-0.84165	1.478416	p16783	Serine/thre	4	-0.95204				GO:0035556; P:intracellular signal transduction;
GSE1 pT43	-0.90778	-3.38432	0.027746	-3.1593	-0.31772	-0.0503	-0.73433	-0.83678	p15805	Genetic sup	4	-1.17035				
GSDMD sec	0	0	0	0	0.20269	-1.24486	-1.10886	-1.22575	p03468	Gasdermin-	4	-0.4221				
GRWD1 sec	-0.49208	-0.55526	-0.22555	-0.31005	-0.17639	-0.13922	-0.20783	-0.33985	p05660	Glutamate-	4	-0.30578				
GRIPAP1 se	-0.17634	0.381312	-0.08931	-0.08337	-1.14673	-0.21568	-0.66364	-0.64906	p04980	GRIP1-asso	4	-0.33035		CAMKinh1 (KN-93); AKTinh2 (MK-2206); AKTinh1 (Akt Inhibitor VIII);		
GRIPAP1 se	0.275995	0.424693	0.083445	0.124554	-0.7033	-0.22176	-0.67542	-0.48752	p04981	GRIP1-asso	4	-0.14741		CAMKinh1 (KN-93); AKTinh2 (MK-2206); AKTinh1 (Akt Inhibitor VIII);		
GRIP1 pS84	-0.15921	-1.01339	0.359042	-0.24373	-0.54805	-0.2775	-0.52015	-0.51965	p07980	Glutamate r	4	-0.36533				GO:0030521; P:androgen receptor signaling pathway; GO:0035556; P:intracellular signal transduction;
GREB1L sec	-1.37317	-1.49838	-2.0889	-1.03223	0	0	0	0	p11737	GREB1-like	4	-0.74909				
GREB1 pS1	0.243427	0.133089	0.259371	-0.04311	1.034082	0.436659	0.594901	-8.33842	p14630	Protein GRE	4	-0.71			Validated nuclear estrogen receptor alpha network;	
GRB7 pS66	-0.63581	-0.30945	-1.05969	-0.97124	0.486203	-0.41706	-0.1684	0.080257	p07543	Growth fact	4	-0.3744			Signaling ev	GO:0009967; P:positive regulation of signal transduction; GO:0007173; P:epidermal growth factor receptor signaling pathway;
GRB10 seq	-0.99341	-1.06193	-0.73033	-0.77425	-2.87556	-3.0962	-1.25975	-2.51138	p03280	Growth fact	4	-1.66285			IGF1 pathw	GO:0048009; P:insulin-like growth factor receptor signaling pathway; GO:0030949; P:positive regulation of vascular endothelial growth factor receptor signaling pathway; GO:0046627; P:negative regulation of insulin receptor signaling pathway; GO:0030178; P:negative regulation of Wnt signaling pathway; GO:0008286; P:insulin receptor signaling [illegible]
GRB10 pS1	-0.88264	-0.89988	-0.67628	-1.08266	-1.08388	-1.18275	-1.1123	-0.82284	p05483	Growth fact	4	-0.9679			IGF1 pathw	GO:0048009; P:insulin-like growth factor receptor signaling pathway; GO:0030949; P:positive regulation of vascular endothelial growth factor receptor signaling pathway; GO:0046627; P:negative regulation of insulin receptor signaling pathway; GO:0030178; P:negative regulation of Wnt signaling pathway; GO:0008286; P:insulin receptor signaling [illegible]
GPS1 seq: 4	0.279697	0.122904	-0.54695	-0.56629	-1.01411	-0.69891	-0.62493	-0.81399	p09230	COP9 signa	4	-0.48282	ATR;			GO:0000188; P:inactivation of MAPK activity;
GPRIN1 pS	-0.34787	-0.58561	-0.08455	-0.05855	-1.06157	-0.74562	-0.42295	-0.27741	p01446	G protein-re	4	-0.44802				
GPRIN1 pS	-0.38168	-0.67657	-0.21237	-0.1302	-0.14302	-0.31482	-0.33373	-0.17551	p01445	G protein-re	4	-0.29599				
GPRASP2 s	0.218267	-0.04644	-0.07593	0.14851	-0.26142	-0.13679	-0.13864	-0.29165	p03445	G-protein c	4	-0.07301				
GPHN pS1	-3.46205	0.140129	-0.72057	-2.01775	0.246649	0.239033	0.232623	0.305563	p01320	Gephyrin	4	-0.62955				
GPBP1 seq	-0.91528	-0.66462	-0.7562	-0.78385	-0.87214	-0.7304	-0.80687	-0.89121	p03598	Vasculin	4	-0.80257				
GPATCH8 s	-0.19034	-0.39854	-0.07223	-0.02166	-0.28186	-0.42177	-0.45735	-0.65324	p15496	G patch dor	4	-0.31212				
GPATCH8 s	-1.30864	-1.36493	-2.43712	-0.86314	-0.38005	-0.17739	-0.83651	-0.4744	p12711	G patch dor	4	-0.98027				
GPATCH8 p	-0.40879	0.013216	-1.27767	-0.47763	0.162499	0.474647	-0.36862	0.208142	p12712	G patch dor	4	-0.20928				
GPATCH2 s	-0.07027	-1.45985	-0.7998	-0.10012	-0.12011	1.094514	-0.73136	0.866174	p06843	G patch dor	4	-0.1651				
GPALPP1 p	-0.21119	-0.67236	0.1258	0.150376	0.039606	-0.09771	-0.31347	-0.02596	p08776	GPALPP mc	4	-0.12561				
GON4L pS3	0.092112	-0.10256	0.339418	0.233653	-0.68419	-0.27438	-0.08605	-0.46247	p14969	GON-4-like	4	-0.11806				
GOLGA4 se	-0.59499	-0.77689	-0.20819	-0.1753	0.298134	-0.30974	0.046293	0.304406	p13746	Golgin subf	4	-0.17704				
GOLGA4 se	-0.177	-11.0669	-0.51201	-0.1253	0.028225	-0.5248	-0.16485	0.102377	p08456	Golgin subf	4	-1.55503				
GOLGA4 se	-0.16963	-0.24769	-0.27044	-0.26465	-0.43018	-0.31985	-0.56054	-0.43899	p02108	Golgin subf	4	-0.33774				
GOLGA4 pS	-0.32023	-0.64263	-0.1143	-0.27822	0.159158	-0.2829	-0.11688	-0.07081	p00982	Golgin subf	4	-0.20835		PAKinh (PF3758309);		
GOLGA2 pS	0.137653	0.111007	0.29837	0.203684	-0.5457	-0.55305	-0.28986	-0.10038	p17222	Golgin subf	4	-0.09228			PLK1 signaling events;	
GNS seq: 4	0.158856	-0.52591	-0.91115	-0.49155	-0.10751	-1.00078	-0.99753	-0.16019	p06065	N-acetylglu	4	-0.50447				
GNB4 seq:	-0.47055	-0.61645	-1.97185	-1.14835	0.217946	0.452561	0.501891	0.428412	p11585	Guanine nu	4	-0.3258				
GLCCI1 seq	-0.58701	-0.48859	-0.82243	-0.64308	0.374896	-0.23602	-0.07373	-0.20336	p06206	Glucocortic	4	-0.33491				
GLCCI1 seq	-0.14798	-0.34552	0.02183	-0.23843	-0.1653	-0.10806	-0.07813	0.048995	p06207	Glucocortic	4	-0.12657				
GLCCI1 seq	-0.5036	-0.25158	-0.52739	-0.45858	0.260583	0.113853	0.200831	0.149428	p06208	Glucocortic	4	-0.12706				
GIT2 seq: 4	-0.38927	-0.47996	-0.2237	-1.10547	-0.41528	0.757607	0.811008	0.494083	p08433	ARF GTPase	4	-0.06887			Integrin-linl	GO:0008277; P:regulation of G-protein coupled receptor protein signaling pathway;
GIT2 seq: 3	0.282571	0.28437	-0.07468	0.164475	-1.45237	-0.42678	-0.42713	-0.66799	p08058	ARF GTPase	4	-0.28969			Integrin-linl	GO:0008277; P:regulation of G-protein coupled receptor protein signaling pathway;
GIT1 seq: 5	-0.52374	-0.1697	-0.16729	-0.29311	0.074725	0.163927	0.046119	-0.0146	p14993	ARF GTPase	4	-0.11046			Regulation (	GO:0008277; P:regulation of G-protein coupled receptor protein signaling pathway;
GIGYF2 seq	0.232039	0.135092	-0.04613	-0.08341	-0.26598	-0.21307	-0.08132	-0.14201	p01430	PERQ amin	4	-0.0581				GO:0048009; P:insulin-like growth factor receptor signaling pathway;
GIGYF2 seq	-0.38104	-1.78754	-0.25841	-0.31442	0.100154	-0.16354	-0.02762	-0.4387	p18114	PERQ amin	4	-0.40889				GO:0048009; P:insulin-like growth factor receptor signaling pathway;
GIGYF2 pS1	-0.18621	0.109451	-0.26119	-0.84621	0.128564	-0.5315	-0.58484	-0.27443	p05933	PERQ amin	4	-0.3058				GO:0048009; P:insulin-like growth factor receptor signaling pathway;
GIGYF2 pS1	-0.39362	-0.72492	-0.45773	-0.28485	-0.32495	-0.51413	-0.37047	-0.264	p20902	PERQ amin	4	-0.41683				GO:0048009; P:insulin-like growth factor receptor signaling pathway;
GGCT seq:	-0.27091	-0.22453	-0.48802	-1.04191	-0.98859	-1.11217	-1.29864	-0.89328	p04842	Gamma-glu	4	-0.78976				
GFPT1 seq:	-0.43597	-1.00357	-0.54802	-0.59752	-1.22298	-3.22671	-0.72686	-1.27991	p15308	Glutamine-	4	-1.13019	CAMK2A; AMPKA1;			GO:0006987; P:activation of signaling protein activity involved in unfolded protein response;
GBF1 seq: 3	0.843678	0.20959	0.845895	1.337335	0.456713	-0.88424	0.059105	-4.32171	p04218	Golgi-speci	4	-0.18171			Arf1 pathw	GO:0032012; P:regulation of ARF protein signal transduction;
GAS2L1 pS	-0.0938	-1.003	-0.64754	-0.57611	-0.51478	-0.51653	-0.58245	-0.41865	p07318	GAS2-like p	4	-0.54411				
GAPVD1 se	-0.54809	1.233358	-0.41241	-2.08749	-0.70705	-0.47026	-0.3949	-0.02485	p16947	GTPase-acti	4	-0.42646				GO:0051056; P:regulation of small GTPase mediated signal transduction; GO:0007165; P:signal transduction;
GAPVD1 pS	0.024087	0.009667	0.055056	-0.41592	0.263393	8.76E-05	0.093786	0.087801	p03937	GTPase-acti	4	0.014745				GO:0051056; P:regulation of small GTPase mediated signal transduction; GO:0007165; P:signal transduction;
GAPDH seq	-0.53614	-0.275	-0.9154	-1.20283	-0.21589	-0.0346	-0.24353	0.374159	p00648	Glyceraldeh	4	-0.38115			Validated targets of C-MYC transcriptional activation;	
GAPDH seq	-0.36878	-0.42419	-0.44811	-0.01735	0.617829	0.414339	0.03973	0.606515	p00612	Glyceraldeh	4	0.052498			Validated targets of C-MYC transcriptional activation;	
GAPDH seq	-0.16779	-0.18237	-0.25982	-0.34897	0.806879	-0.05056	-0.01251	0.062949	p14135	Glyceraldeh	4	-0.01902			Validated targets of C-MYC transcriptional activation;	
GAPDH seq	-0.84287	0.280067	-1.36034	-1.15413	-0.22267	-0.37773	-0.03596	0.467586	p01378	Glyceraldeh	4	-0.40576			Validated targets of C-MYC transcriptional activation;	
GAPDH pM	-1.00368	-0.96091	-0.33513	-1.66916	-0.2229	0.279638	-0.11316	0.026611	p00499	Glyceraldeh	4	-0.49984			Validated targets of C-MYC transcriptional activation;	
GANAB seq	-1.11385	-1.21959	-1.43534	-2.12767	2.464669	2.023875	-5.68319	-0.18369	p08742	Neutral alph	4	-0.90935				
GAB2 seq: 4	-0.64245	-0.8658	-0.35501	-1.08833	0.839419	1.514813	0.004449	-0.15866	p06072	GRB2-asso	4	-0.09395			IL3-mediate	GO:0007169; P:transmembrane receptor protein tyrosine kinase signaling pathway; GO:0048015; P:phosphatidylinositol-mediated signaling; GO:0038095; P:Fc-epsilon receptor signaling pathway;
GAB2 seq: 4	-0.05075	-0.13391	0.046647	-0.24038	-0.11353	-0.42945	-0.39322	-0.37506	p19509	GRB2-asso	4	-0.21121			IL3-mediate	GO:0007169; P:transmembrane receptor protein tyrosine kinase signaling pathway; GO:0048015; P:phosphatidylinositol-mediated signaling; GO:0038095; P:Fc-epsilon receptor signaling pathway;
GAB2 seq:	-0.08393	0.247564	-0.06888	-0.47699	-0.79279	-0.92726	-0.47311	-0.76577	p03827	GRB2-asso	4	-0.41765			IL3-mediate	GO:0007169; P:transmembrane receptor protein tyrosine kinase signaling pathway; GO:0048015; P:phosphatidylinositol-mediated signaling; GO:0038095; P:Fc-epsilon receptor signaling pathway;
GAB2 seq:	-0.04781	0.067136	-0.07512	-0.38987	-0.67079	-0.58368	-0.45767	-0.36664	p03826	GRB2-asso	4	-0.31555			IL3-mediate	GO:0007169; P:transmembrane receptor protein tyrosine kinase signaling pathway; GO:0048015; P:phosphatidylinositol-mediated signaling; GO:0038095; P:Fc-epsilon receptor signaling pathway;
GAB2 pT39	0.342015	-0.34551	-0.52628	0.009743	-1.89304	-2.68346	-2.30791	-2.23381	p06991	GRB2-asso	4	-1.20478			IL3-mediate	GO:0007169; P:transmembrane receptor protein tyrosine kinase signaling pathway; GO:0048015; P:phosphatidylinositol-mediated signaling; GO:0038095; P:Fc-epsilon receptor signaling pathway;
GAB2 pT39	0.239104	-0.08106	-0.65441	-0.26755	-1.379	-0.33024	-1.15264	-0.73793	p06992	GRB2-asso	4	-0.54547			IL3-mediate	GO:0007169; P:transmembrane receptor protein tyrosine kinase signaling pathway; GO:0048015; P:phosphatidylinositol-mediated signaling; GO:0038095; P:Fc-epsilon receptor signaling pathway;
GAB1 seq: 3	0.49399	0.558912	-0.53195	0.149031	-0.92576	-1.39427	-2.624	-0.12982	p04746	GRB2-asso	4	-0.55048			EPO signali	GO:0048008; P:platelet-derived growth factor receptor signaling pathway; GO:0048015; P:phosphatidylinositol-mediated signaling; GO:0008286; P:insulin receptor signaling pathway; GO:0008543; P:fibroblast growth factor receptor signaling pathway; GO:0007173; P:epidermal growth factor receptor signaling pathway; GO:0048011; P:neurotrop [illegible]
GAB1 seq:	0.159665	-0.60303	0.172122	0.297815	-0.15333	-0.74177	-0.0257	-0.4526	p00792	GRB2-asso	4	-0.16835			EPO signali	GO:0048008; P:platelet-derived growth factor receptor signaling pathway; GO:0048015; P:phosphatidylinositol-mediated signaling; GO:0008286; P:insulin receptor signaling pathway; GO:0008543; P:fibroblast growth factor receptor signaling pathway; GO:0007173; P:epidermal growth factor receptor signaling pathway; GO:0048011; P:neurotrop [illegible]
GAB1 pS25	-0.45038	-0.42483	-1.21855	-1.28055	-1.52019	-4.43856	-0.72806	-0.18505	p19929	GRB2-asso	4	-1.28077			EPO signali	GO:0048008; P:platelet-derived growth factor receptor signaling pathway; GO:0048015; P:phosphatidylinositol-mediated signaling; GO:0008286; P:insulin receptor signaling pathway; GO:0008543; P:fibroblast growth factor receptor signaling pathway; GO:0007173; P:epidermal growth factor receptor signaling pathway; GO:0048011; P:neurotrop [illegible]
G6PD seq:	-0.26425	0.683036	-1.0557	-1.33586	-0.17714	0.537311	-0.22599	0.353061	p11003	Glucose-6-	4	-0.18569				
FZD6 pS62	-0.06575	-0.08648	0.100588	-0.0624	-0.0578	0.137048	0.340053	-0.24894	p10907	Frizzled-6	4	0.007039			Wnt signali	GO:0060071; P:Wnt signaling pathway, planar cell polarity pathway; GO:0090090; P:negative regulation of canonical Wnt signaling pathway;
FUBP3 pS5	-0.54104	-0.65364	-0.12405	-0.39846	-0.06609	0.145152	0.224336	0.256589	p07993	Far upstrea	4	-0.14465		ERKinh2 (ERK inhibitor II);		
FUBP1 seq:	-0.34663	1.494274	-2.46446	-1.71886	0.433913	0.387782	-7.16874	1.653449	p09822	Far upstrea	4	-0.96616				
FRS2 seq: 3	-1.7296	-1.06644	-1.94211	-1.6068	0	0	0	0	p10485	Fibroblast g	4	-0.79312			Signaling ev	GO:0000186; P:activation of MAPKK activity; GO:0000187; P:activation of MAPK activity; GO:0048015; P:phosphatidylinositol-mediated signaling; GO:0008286; P:insulin receptor signaling pathway; GO:0008543; P:fibroblast growth factor receptor signaling pathway; GO:0007173; P:epidermal growth factor receptor signaling pathway; GO:00480 [illegible]
FRMD8 pS4	-0.12791	0.090113	-0.12426	-0.52532	0.019258	-0.71201	-0.60578	-0.28762	p02020	FERM doma	4	-0.28419				
FRMD6 pS5	-0.06314	-0.09987	-0.32088	-0.22032	-0.54678	-0.17075	-0.42723	-0.62477	p02078	FERM doma	4	-0.30922				
FRMD5 seq	0.595982	-0.16491	0.588717	0.363596	-0.6243	-1.13062	-0.26999	-0.62892	p20611	FERM doma	4	-0.15881				
FRMD4B se	-0.43951	-1.12992	0.265953	-0.20624	-0.39589	-0.76474	-0.39466	-0.33315	p12667	FERM doma	4	-0.42477				
FOXQ1 pS2	-0.56034	-1.29925	-2.6038	-1.51589	-1.02001	-1.92184	-1.17124	-0.98478	p19860	Forkhead b	4	-1.38464				
FOXP1 pS8	0.370485	0.193144	0.451582	0.301871	-1.46285	-1.49302	-0.38031	-2.03012	p08356	Forkhead b	4	-0.50615				
FOXO3 seq	0.506668	-0.3245	0.577542	0.407152	-0.71532	-0.82702	-0.71334	-0.81507	p21090	Forkhead b	4	-0.23799			Signaling ev	GO:0030330; P:DNA damage response, signal transduction by p53 class mediator; GO:0048015; P:phosphatidylinositol-mediated signaling; GO:0008286; P:insulin receptor signaling pathway; GO:0008543; P:fibroblast growth factor receptor signaling pathway; GO:0007173; P:epidermal growth factor receptor signaling pathway; GO:0048011; [illegible]
FOXK2 seq:	-0.32462	0.464173	-0.66391	-0.61526	-1.60275	-0.64172	-1.27293	-1.70581	p06304	Forkhead b	4	-0.79535	CDK2; CDK1;			
FOXK2 pS3	-0.05472	0.193814	-0.12809	-0.0731	-0.45683	-0.31222	-0.52229	-0.41547	p17681	Forkhead b	4	-0.22111				
FOXK1 seq:	-0.08365	-0.13025	-0.04942	0.021189	-0.4048	-0.23195	-0.55764	-0.58747	p05748	Forkhead b	4	-0.253		mTORinh2 (Torin-1); mTORinh1 (KU-0063794);		
FOXK1 seq:	-0.83633	0.327784	-0.68725	-0.64196	-2.03746	-1.13281	-1.98672	-1.57746	p04859	Forkhead b	4	-1.07153		Akt-mTOR-PI3K; PI3Kinh2 (PI-103); PI3Kinh1 (GDC-0941); mTORinh2 (Torin-1); mTORinh1 (KU-0063794); AKTinh2 (MK-2206); AKTinh1 (Akt Inhibitor VIII);		
FOXK1 seq:	-0.87205	0.417438	-0.88275	-0.71766	-1.30283	-0.79046	-2.06813	-1.47261	p04861	Forkhead b	4	-0.96113				
FOXK1 pS4	-0.08694	0.011937	-0.23076	-0.01418	-0.35533	-0.18958	-0.55897	-0.52372	p17676	Forkhead b	4	-0.24344				
FOXK1 pS4	-0.73242	-0.43283	-0.94496	-0.5931	-0.21827	-0.20016	-0.54853	-0.35487	p06298	Forkhead b	4	-0.50314				
FOXK1 pS4	-0.63231	-0.29094	-0.68258	-0.42961	-0.33309	-0.11289	-0.79364	-0.58971	p06297	Forkhead b	4	-0.4831				
FOXK1 pS4	-0.07258	0.253802	-0.1575	-0.0074	-0.76939	-0.73535	-0.90073	-0.94862	p06295	Forkhead b	4	-0.41722				
FOXK1 pS4	-0.04233	0.342698	-0.20369	-0.015	-0.57528	-0.64893	-0.71015	-0.73281	p06296	Forkhead b	4	-0.32319				
FOXD2 pS9	-1.43798	-0.38086	-1.40057	-1.06121	-1.07609	-0.38218	-0.71546	-0.58202	p16360	Forkhead b	4	-0.87955				
FOXA1 seq:	-0.18416	-1.05774	0.304857	0.152769	0	0	0	0	p19129	Hepatocyte	4	-0.09803			FOXA trans	GO:0045880; P:positive regulation of smoothened signaling pathway;
FOXA1 pS3	-0.3122	-0.98572	-0.13853	-0.30969	-2.02746	-1.10346	-1.42819	-0.26628	p13328	Hepatocyte	4	-0.82144			FOXA trans	GO:0045880; P:positive regulation of smoothened signaling pathway;
FOXA1 pS3	-0.1218	-0.71955	-0.02272	-0.14542	-0.27365	-0.31644	-0.37245	-0.46713	p13329	Hepatocyte	4	-0.30489			FOXA trans	GO:0045880; P:positive regulation of smoothened signaling pathway;
FOXA1 pS3	-0.06082	-0.59066	0.010323	-0.18243	0	0	0	0	p13330	Hepatocyte	4	-0.10295			FOXA trans	GO:0045880; P:positive regulation of smoothened signaling pathway;
FOSL1 pS1	-0.67328	-1.36037	-0.38328	-1.13773	-0.2186	-0.24522	-0.41267	-0.42717	p06752	Fos-related	4	-0.60729			Calcium signaling in the CD4+ TCR pathway; Validated transcriptional targets of AP1 family members Fra1 and Fra2; Calcineurin-regulated NFAT-dependent transcription in lymphocytes; Downstream signaling in naïve CD8+ T cells; AP-1 transcription factor network; Validated targets of C-MYC transcriptional activation;	
FNIP1 seq:	0.273514	0.476674	0.134031	0.167498	0.458823	-0.13883	-3.71443	0.858549	p06266	Folliculin-in	4	-0.18552				GO:0031929; P:TOR signaling; GO:0032007; P:negative regulation of TOR signaling;
FNBP1L pS	0.044912	-0.37434	-0.09022	-0.02554	-0.11126	-0.57252	-0.20271	-0.38661	p02698	Formin-bin	4	-0.21479				
FNBP1 seq:	0.336652	-0.98404	-0.02929	0.189686	-0.7549	-0.73134	-0.46963	-0.42587	p06799	Formin-bin	4	-0.35859				
FLYWCH2 s	0.194368	-0.36967	0.000314	0.054462	-0.89785	-0.22408	-0.47834	-0.50001	p19522	FLYWCH far	4	-0.2776		mTORinh2 (Torin-1);		
FLYWCH2 s	0.023014	-0.52171	-0.05745	0.068855	-0.45763	-0.14736	-0.44262	-0.48548	p19521	FLYWCH far	4	-0.25255		mTORinh2 (Torin-1);		
FLYWCH1 p	0.133971	-0.01406	0.136838	0.024418	-1.61691	-0.8492	-1.51482	-3.27247	p20593	FLYWCH-ty	4	-0.87153				
FLNC pS22	0	0	0	0	-2.09794	-2.21632	-2.60384	-2.15894	p11700	Filamin-C	4	-1.13463	Akt2; Akt1;			
FLNB seq: 1	0.159412	0.056992	0.083886	0.093669	-1.14666	-0.88294	-0.02191	-0.66504	p13845	Filamin-B	4	-0.29032				GO:0019221; P:cytokine-mediated signaling pathway; GO:0007165; P:signal transduction;
FLNB seq: 1	-0.73693	-0.3087	-0.77171	-0.79096	1.010235	0.612548	-0.57319	0.859455	p01285	Filamin-B	4	-0.08741				GO:0019221; P:cytokine-mediated signaling pathway; GO:0007165; P:signal transduction;
FLNB pS24	-0.60999	-0.5191	-0.69464	0.046718	-0.55141	-0.77871	-0.12464	-0.30456	p00372	Filamin-B	4	-0.44204				GO:0019221; P:cytokine-mediated signaling pathway; GO:0007165; P:signal transduction;
FLNA seq: 1	-1.18447	-0.17927	-1.86863	-2.06281	0.409314	0.380266	-0.20334	0.479809	p01420	Filamin-A	4	-0.52864			Noncanoni	GO:0043123; P:positive regulation of I-kappaB kinase/NF-kappaB signaling;
FLNA seq: 1	0	0	0	0	0	0	0	0	p01580	Filamin-A	4	0			Noncanoni	GO:0043123; P:positive regulation of I-kappaB kinase/NF-kappaB signaling;
FLNA pS26	0.488008	0.221784	-2.82306	-1.76518	0.129769	0.025703	-0.02656	-0.17853	p10287	Filamin-A	4	-0.49101			Noncanoni	GO:0043123; P:positive regulation of I-kappaB kinase/NF-kappaB signaling;
FLII seq: 12	-0.90024	-0.78155	-0.67539	-0.53963	0.002389	-0.11593	-0.38588	0.678677	p15051	Protein fligh	4	-0.33969				
FKBP4 seq:	-0.60403	-0.97054	-0.21768	-0.36473	0.366324	0.074536	-0.05647	-0.36017	p16795	Peptidyl-pr	4	-0.2666			Coregulatio	GO:0030521; P:androgen receptor signaling pathway;
FKBP1A sec	-0.45633	0.377558	-0.61132	-1.2328	1.089188	0.990929	0.059464	0.211394	p14998	Peptidyl-pr	4	0.053511			ALK1 pathw	GO:0007179; P:transforming growth factor beta receptor signaling pathway; GO:0043123; P:positive regulation of I-kappaB kinase/NF-kappaB signaling;
FKBP15 sec	0.410783	0.254221	0.11287	-0.83918	-0.77984	-0.56787	-0.41367	-0.31228	p17223	FK506-binc	4	-0.26687				
FKBP15 sec	-0.3987	-0.29027	-0.44232	-0.18855	-1.23955	-0.93734	-0.55062	-0.84401	p04779	FK506-binc	4	-0.61142		Up with IGF - down with PI3Kinh2 (PI-103); Up with EGF - down with PI3Kinh2 (PI-103); Up with IGF - down with PI3Kinh1 (GDC-0941); Up with EGF - down with PI3Kinh1 (GDC-0941); Up with IGF - down with p70S6Kinh2 (DG2); Up with EGF - down with p70S6Kinh2 (DG2); Up with IGF - down with p70S6Kinh1 (PF-4708671); Up with EGF - down with p70S6Kinh1 (PF- [illegible]		
FKBP15 sec	0.075984	-0.15685	-0.16395	-0.01472	-1.16856	-1.187	-0.89196	-1.04525	p04780	FK506-binc	4	-0.56904		Up with IGF - down with PI3Kinh2 (PI-103); Up with EGF - down with PI3Kinh2 (PI-103); Up with IGF - down with PI3Kinh1 (GDC-0941); Up with EGF - down with PI3Kinh1 (GDC-0941); Up with IGF - down with p70S6Kinh2 (DG2); Up with EGF - down with p70S6Kinh2 (DG2); Up with IGF - down with p70S6Kinh1 (PF-4708671); Up with EGF - down with p70S6Kinh1 (PF- [illegible]		
FKBP15 sec	-0.42975	-0.0978	-0.2448	-0.28998	-2.82435	0.53642	-1.99701	-3.00655	p18311	FK506-binc	4	-1.04423		CAMKinh1 (KN-93); AKTinh2 (MK-2206); AKTinh1 (Akt Inhibitor VIII);		
FKBP15 sec	-0.65987	-0.38707	-0.61383	-0.5004	-0.60146	-0.10049	-0.75199	-0.61474	p18310	FK506-binc	4	-0.52873		CAMKinh1 (KN-93); AKTinh2 (MK-2206); AKTinh1 (Akt Inhibitor VIII);		
FKBP15 sec	-0.97157	-0.8099	-0.53604	-0.59147	-0.26408	-0.47369	-0.61118	-0.69998	p03838	FK506-binc	4	-0.61974				
FIP1L1 pS4	1.059473	1.127825	-0.04191	-0.06812	-1.30061	-0.77508	-0.86266	-0.965	p17247	Pre-mRNA 3	4	-0.22826				GO:0043123; P:positive regulation of I-kappaB kinase/NF-kappaB signaling; GO:0007165; P:signal transduction;
FIP1L1 pS2	-0.22518	-0.55471	0.24756	-0.00398	-0.00564	-0.29296	-0.18155	-0.33388	p20843	Pre-mRNA 3	4	-0.16879				GO:0043123; P:positive regulation of I-kappaB kinase/NF-kappaB signaling; GO:0007165; P:signal transduction;
FILIP1L pT9	0	0	0	0	-0.52878	-0.14086	-0.19605	-0.3199	p13610	Filamin A-in	4	-0.1482				
FILIP1L pS1	-0.29414	0.397965	1.007863	-4.03555	-0.28461	0.162694	-0.08717	-0.24532	p14900	Filamin A-in	4	-0.42228				
FGD1 pT71	-0.02209	-0.0457	-0.07603	-0.0126	-0.66979	-0.1949	-0.37917	-0.28478	p11342	FYVE, RhoG	4	-0.21063			Regulation (	GO:0051056; P:regulation of small GTPase mediated signal transduction; GO:0048011; P:neurotrophin TRK receptor signaling pathway; GO:0007264; P:small GTPase mediated signal transduction; GO:0007165; P:signal transduction;
FDXR seq: 8	-1.03885	-0.85666	-1.17888	-1.55018	0	0	0	0	p16727	NADPH:adr	4	-0.57807			Validated transcriptional targets of TAp63 isoforms; Direct p53 effectors;	
FBRSL1 seq	-0.05415	-0.39916	-0.02854	-0.00662	-0.37398	-0.31152	-0.54689	-0.48075	p18034	Fibrosin-1-l	4	-0.2752				
FBRSL1 pT1	-0.63092	-0.45733	-0.41225	-0.48618	-0.73169	-0.13738	-0.45431	-0.46953	p02362	Fibrosin-1-l	4	-0.47245		AKTinh1 (Akt Inhibitor VIII);		
FBP1 seq: 3	-0.27769	-0.77661	-1.33977	-0.92629	0	0	0	0	p19861	Fructose-1,	4	-0.41505				GO:0046580; P:negative regulation of Ras protein signal transduction;
FBP1 seq: 3	-0.63118	-0.40483	-1.0611	-1.1191	0	0	0	0	p17903	Fructose-1,	4	-0.40202				GO:0046580; P:negative regulation of Ras protein signal transduction;
FASN seq: 9	-0.69617	0.904042	-0.97059	-0.9245	0.405555	0.125917	-0.51632	-2.07828	p00523	Fatty acid s	4	-0.46879			Validated transcriptional targets of deltaNp63 isoforms; p73 transcription factor network;	
FASN seq: 6	-0.76239	-0.72129	-1.21039	-1.02911	-0.27941	0.269709	0.644704	1.447851	p16819	Fatty acid s	4	-0.20504			Validated transcriptional targets of deltaNp63 isoforms; p73 transcription factor network;	
FASN seq: 1	0.885978	2.530756	-3.01156	-3.84052	0	0	0	0	p08581	Fatty acid s	4	-0.42942			Validated transcriptional targets of deltaNp63 isoforms; p73 transcription factor network;	
FASN seq: 1	-0.648	0.797424	-1.11771	-1.40236	-7.91456	0.617331	-1.63084	0.646287	p06828	Fatty acid s	4	-1.33155			Validated transcriptional targets of deltaNp63 isoforms; p73 transcription factor network;	
FASN seq: 1	-0.51511	-0.64862	-0.91768	-0.88512	1.010296	0.564959	0.054711	-0.3405	p06807	Fatty acid s	4	-0.20963			Validated transcriptional targets of deltaNp63 isoforms; p73 transcription factor network;	
FASN pT22	-0.68281	-0.23088	-0.56418	-0.4002	-0.69623	-0.10534	1.334035	1.365351	p20926	Fatty acid s	4	0.002468		Up with IGF	Validated transcriptional targets of deltaNp63 isoforms; p73 transcription factor network;	
FAM83H se	0.072385	0.011072	0.104481	0.124017	-0.47888	-0.44699	-0.66778	-0.53812	p15242	Protein FAN	4	-0.22748				
FAM83H se	0.521191	0.308932	0.240708	0.204997	-0.44707	-0.4484	-0.51927	-0.28182	p07334	Protein FAN	4	-0.05259				
FAM83H se	0.004571	-0.24245	0.060867	-0.07746	-0.42657	-0.34639	-0.69753	-0.56968	p03759	Protein FAN	4	-0.28683				
FAM83H se	-0.38132	-0.1739	-0.27707	-0.34485	-0.22745	-0.02375	-0.55496	-0.55455	p07324	Protein FAN	4	-0.31723				
FAM83H se	-0.2693	-0.28829	-1.24318	-0.70612	-0.1542	-0.42501	-1.08298	-0.37131	p10675	Protein FAN	4	-0.56755				
FAM83H pS	-0.48252	-0.73172	-0.48971	-0.33224	-0.47203	-0.41675	-0.68226	-0.72043	p15187	Protein FAN	4	-0.54096				
FAM83H pS	-0.37809	-0.61133	-0.39891	-0.29137	-0.47984	-0.47605	-0.67201	-0.72502	p07322	Protein FAN	4	-0.50408				
FAM83H pS	-0.3343	-0.48726	-0.3864	-0.32385	-0.4304	-0.42112	-0.62973	-0.67243	p15188	Protein FAN	4	-0.46069				
FAM83H pS	-0.3114	-0.50822	-0.36527	-0.33993	-0.43409	-0.4105	-0.59965	-0.68335	p07323	Protein FAN	4	-0.45655				
FAM83H pS	-0.17675	-0.37109	-0.31487	-0.22285	-0.3973	-0.2707	-0.51183	-0.47938	p07321	Protein FAN	4	-0.3431				
FAM83H pS	-0.09665	0.839154	-0.27354	-0.33542	-0.94426	0.338898	-2.42376	-1.01783	p15191	Protein FAN	4	-0.48918				
FAM83H pS	0.065858	0.509712	0.192982	0.127249	-1.53178	-3.97889	-1.96949	-1.85749	p00972	Protein FAN	4	-1.05523				
FAM83H pS	-0.17643	-0.09639	-0.34357	-0.03489	0.188488	-0.12811	-0.61018	0.332674	p10677	Protein FAN	4	-0.10855				
FAM83H pS	0.073467	-0.81247	-0.33851	-0.21195	-0.1354	-0.47047	-0.5393	-0.40783	p07125	Protein FAN	4	-0.35531				
FAM83E pS	-0.44002	-0.78397	-0.06697	-0.14894	0	0	0	0	p00807	Protein FAN	4	-0.17999				
FAM83E pS	0.028653	-1.21839	-0.43946	-0.47532	0	0	0	0	p06715	Protein FAN	4	-0.26306				
FAM83B se	-0.01138	-0.68654	0.286422	0.010971	0.275282	-0.95	0.235703	0.106158	p12115	Protein FAN	4	-0.09167				

FAM83B pS	-0.501	-1.24881	-0.52251	-0.08748	-1.25888	-1.80835	-0.9718	-1.34097	p16977	Protein FAN	4	-0.96747		
FAM83B pS	-0.91437	-0.68102	-0.78662	-1.12717	-5.14413	-1.50777	0.600252	-0.04857	p06681	Protein FAN	4	-1.20117		
FAM65A pS	1.617487	-0.22168	2.034424	-6.24158	-0.17753	-0.34866	-0.61465	-0.19352	p05866	Protein FAN	4	-0.51822		
FAM53C pS	0.116295	-0.64014	-0.0363	0.288491	-0.04518	-0.39876	-0.0254	-0.15652	p16596	Protein FAN	4	-0.11219		
FAM53B pS	-0.59023	-0.75671	-0.50452	-0.66838	-0.2813	-0.36181	-0.54801	-0.38424	p03710	Protein FAN	4	-0.5119		
FAM53B pS	-0.58118	-1.01352	-1.14834	-1.10689	-1.28264	-0.26886	-0.52464	-1.26043	p05059	Protein FAN	4	-0.89831		
FAM49B se	-0.88148	-1.0681	-1.20346	-1.37772	-1.60586	-1.52901	-1.21046	-1.6092	p13904	Protein FAN	4	-1.31066		
FAM46C se	0.600425	-1.23553	-0.48156	0.081914	1.560975	1.787409	-2.62582	-0.69276	p13679	Protein FAN	4	-0.12562		
FAM21C pS	-0.21695	0.098607	-0.13773	-0.2306	-0.42488	-0.36018	-0.38084	-0.34347	p17030	WASH com	4	-0.2495		
FAM21A se	0.010364	-0.23053	0.01984	-0.55217	-0.27215	-2.43819	-0.39738	-0.33592	p02639	WASH com	4	-0.52452		PI3Kinh2 (PI-103);
FAM21A se	0.050958	-0.23226	0.063491	0.111998	0.004346	-0.08697	-0.02597	0.100323	p19654	WASH com	4	-0.00176		
FAM21A se	0.191853	-3.41164	0.337995	-0.50951	-0.32038	-0.06583	-0.79907	-0.69674	p16186	WASH com	4	-0.65916		
FAM21A pS	-0.92142	0.069821	-0.48533	-0.26977	-0.39809	-0.18683	-0.39304	-0.32439	p10575	WASH com	4	-0.36363		Up with IGF - down with PI3Kinh2 (PI-103); Up with EGF - down with PI3Kinh2 (PI-103); Up with IGF - down with AKTinh1 (Akt Inhibitor VIII); Up with EGF - [illegible]
FAM195A s	-0.11755	-0.20441	-0.35627	-0.56539	-0.27621	-0.01117	-0.03748	-0.04254	p07609	Protein FAN	4	-0.20138		
FAM177A1	-0.12353	-0.19758	0.050821	0.122593	-0.31133	-0.35959	-0.21564	-0.00169	p01382	Protein FAN	4	-0.12949		Akt-CAMK2-EGFR; EGFRinh2 (PD-153035); EGFRinh1 (PD-168393); CAMKinh2 (KN-62); CAMKinh1 (KN-93); AKTinh2 (MK-2206); AKTinh1 (Akt Inhibitor V[illegible]
FAM13B pS	-0.45211	-0.65913	-0.39223	-0.32029	-1.15524	-1.43513	-1.37452	-1.42256	p19594	Protein FAN	4	-0.9014		GO:0051056; P:regulation of small GTPase mediated signal transduction; GO:0007264; P:small GTPase mediated signal transductio[illegible]
FAM13A se	-2.82996	-0.0725	-2.47855	-1.05514	0.004197	-1.71178	-1.31552	-0.29663	p03691	Protein FAN	4	-1.21949		GO:0051056; P:regulation of small GTPase mediated signal transduction; GO:0007264; P:small GTPase mediated signal transductio[illegible]
FAM134A p	-2.43366	0.396812	-0.63243	-0.70959	-0.04055	-1.02585	-5.2937	-0.93191	p09753	Protein FAN	4	-1.33386		
FAM122B s	-0.48165	-0.41913	-0.84248	-0.4288	-0.05307	0.17757	-0.83889	-0.15635	p07001	Protein FAN	4	-0.38035		
FAM122B s	-0.02838	-0.17908	0.146798	0.046109	-0.23309	-0.03637	-0.15164	-0.1938	p15311	Protein FAN	4	-0.07868		
FAM122B s	0.058356	-3.36775	0.306652	0.410373	0.425748	-0.26409	-0.37193	-0.11785	p10138	Protein FAN	4	-0.36506		
FAM122B s	-0.57725	-1.53952	-0.66311	-0.6365	-0.07194	-0.3861	-0.572	-0.2467	p07275	Protein FAN	4	-0.58664		
FAM122B s	-0.25444	1.403358	0.102325	-0.03587	-1.91432	-0.45895	-1.53693	-1.71891	p07277	Protein FAN	4	-0.55172		
FAM122B p	-0.12777	-0.273	-0.16813	-0.24291	-0.29668	-0.08153	-0.55264	-0.09992	p07003	Protein FAN	4	-0.23032		
FAM122B p	-0.38294	-0.31762	-0.77732	-0.33231	-0.07567	0.166091	-0.77819	-0.1709	p07002	Protein FAN	4	-0.33361		
FAM122B p	-0.20474	0.136238	-0.28242	-0.41029	-1.0314	-0.29264	-1.07084	-1.15381	p07276	Protein FAN	4	-0.53874		
FAM122A s	-0.48165	-0.41913	-0.84248	-0.4288	-0.05307	0.17757	-0.83889	-0.15635	p07004	Protein FAN	4	-0.38035		
FAM122A s	-0.19032	0.493572	0.093464	-0.00858	-0.12297	-0.24938	-0.56616	-0.30063	p04817	Protein FAN	4	-0.10638		
FAM122A s	-0.47212	-0.57622	-0.15126	-0.00141	0.014713	-0.05798	-0.32996	-0.09419	p00761	Protein FAN	4	-0.20855		
FAM122A s	-0.19525	0.945443	-0.55862	-0.20744	-1.45158	-0.29884	-1.39116	-1.23499	p07281	Protein FAN	4	-0.54906		
FAM122A p	-0.24351	-0.23253	-0.68332	-0.29323	-0.09096	0.170827	-0.75613	-0.18253	p07005	Protein FAN	4	-0.28892		
FAM122A p	-0.14997	0.43113	-0.33125	-0.25347	-0.85583	-0.20291	-0.9717	-0.83103	p07280	Protein FAN	4	-0.39563	CDK2;	
FAM120A p	-0.18501	-0.91807	-0.10901	-0.26723	-0.4107	-0.62301	-0.3934	-0.32106	p09246	Constitutive	4	-0.40343		p70S6Kinh2 (DG2);
FAM117B s	0.131465	0.2029	0.420039	0.269414	-0.64389	-0.86144	-0.28388	-0.31814	p09593	Protein FAN	4	-0.13544		
FAM117B p	-0.40942	-0.78139	-0.13022	-0.13591	0.172124	-0.00351	-0.29802	0.08145	p15214	Protein FAN	4	-0.18811		
FAM117A p	-0.35754	-0.50569	-0.36469	-0.29562	-0.19042	-0.4039	-0.3235	-0.12932	p16236	Protein FAN	4	-0.32133		
FAM117A p	-0.71607	-0.781	-0.70073	-0.52242	0.118202	-0.00175	0.223849	0.074452	p17233	Protein FAN	4	-0.28818		
FAM117A p	-1.31904	-1.18431	-1.32321	-1.73645	-1.03432	-0.98654	-0.63819	-0.51075	p06205	Protein FAN	4	-1.0916		
FAM110C p	0.046982	0.317145	0.077185	0.060812	-1.40248	-6.83565	-1.12992	-0.06334	p17341	Protein FAN	4	-1.11616		GO:0051897; P:positive regulation of protein kinase B signaling;
FAM110A p	-1.12306	-2.989	-2.10164	-0.43486	0.362459	-0.60658	-0.36915	-0.55997	p08128	Protein FAN	4	-0.97773		
EXPH5 seq:	-0.7419	-0.85222	-0.51885	-0.34455	-0.92839	-0.59089	-0.8396	-0.59186	p00782	Exophilin-5	4	-0.67603		
EXPH5 seq:	-0.5288	-0.59204	-0.26369	-0.28684	-0.24373	-0.3173	-0.55601	-0.24854	p00783	Exophilin-5	4	-0.37962		
EXOSC1 pS	-0.61332	-1.09987	-0.24248	-1.05431	-0.30006	-0.35068	-0.5855	-0.50537	p12118	Exosome cc	4	-0.59395		
EVPL seq: 6	-0.6771	-0.26071	-1.4862	-0.96079	-0.04934	0.049505	0.211914	-0.07116	p11163	Envoplakin	4	-0.40549		Validated transcriptional targets of TAp63 isoforms;
EVL pS369	-0.17945	-0.25494	0.013636	0.000894	-0.34102	-2.87093	-0.50338	0.688481	p10049	Ena/VASP-li	4	-0.43084		PAKinh (PF3758309); GO:0007166; P:cell surface receptor signaling pathway;
ETFB pT194	1.152751	0.118122	0.109933	-0.12576	-0.54389	-0.83502	-0.60754	-0.74917	p00439	Electron tra	4	-0.18507		
ETFA seq: 7	-0.84684	-1.42232	-1.07914	-1.11826	1.497545	0.785856	0.299586	-0.27962	p01127	Electron tra	4	-0.2704		
ESYT2 seq:	-0.90645	0.04786	-0.44008	-0.19462	-0.46667	0.075644	-0.06813	0.047331	p05590	Extended sy	4	-0.23814		
ESYT1 seq:	2.591914	-4.92732	2.63624	1.758235	-1.10218	-0.22332	-0.29422	-0.91768	p14729	Extended sy	4	-0.05979		
ERRFI1 pS3	-2.7267	0.115209	-2.59629	-2.34807	-0.00249	0.087813	0.110225	0.19866	p06411	ERBB recept	4	-0.89521		
ERP29 seq:	-0.50071	0.342242	-0.54081	-0.92167	0.595645	0.431015	-0.08082	0.988836	p05137	Endoplasmi	4	0.039217		
ERF seq: 52	2.705844	2.63732	-5.30624	0.543468	-0.57496	-0.99083	-0.94924	-0.63742	p06429	ETS domain	4	-0.32151		Up with EGI IL2 signaling events mediated by STAT5; IL12 signaling mediated by STAT4; Cas[illegible]
ERCC6L pS	-0.02295	-0.50089	0.203519	0.243271	0.028896	-0.51487	-0.06907	0.124832	p16111	DNA excisic	4	-0.06341		PLK1 signaling events;
ERCC6 pS1	-0.05148	-0.26396	0.31276	0.029298	-0.32897	-0.32758	-0.32089	-0.67035	p14180	DNA excisic	4	-0.20265		
ERC1 seq: 7	-0.28818	-0.30767	-0.34366	-0.4104	-0.76605	-1.41244	-0.51506	-0.42337	p03343	ELKS/Rab6-	4	-0.55835		Canonical NF-kappaB pathway; IL1-mediated signaling events;
ERC1 seq: 7	-0.55205	-0.62397	-0.32215	-0.40914	-0.24683	-0.20522	-0.29553	-0.38901	p03344	ELKS/Rab6-	4	-0.38049		Canonical NF-kappaB pathway; IL1-mediated signaling events;
ERBB2IP se	-0.08699	-0.83392	-0.91539	-0.50142	-0.25895	0.189479	-0.21134	-0.10872	p06200	Protein LAP	4	-0.34091		TCR signalin GO:0030522; P:intracellular receptor signaling pathway; GO:000[illegible]
ERBB2IP se	0.197421	-1.01394	0.34575	0.465214	0.157941	-0.46356	0.060257	0.273932	p05385	Protein LAP	4	0.002877		TCR signalin GO:0030522; P:intracellular receptor signaling pathway; GO:000[illegible]
ERBB2IP se	-0.16937	-1.37112	0.481422	-0.44108	-1.19512	-1.82373	-1.17491	-1.09292	p02493	Protein LAP	4	-0.84835		PI3Kinh2 (F TCR signalin GO:0030522; P:intracellular receptor signaling pathway; GO:000[illegible]
ERBB2IP pS	-0.24553	-1.24278	-0.07702	-0.3464	-1.05254	-1.71392	-1.26601	-1.32181	p01453	Protein LAP	4	-0.90825		TCR signalin GO:0030522; P:intracellular receptor signaling pathway; GO:000[illegible]
ERBB2IP pS	-0.20727	-1.12963	-0.16668	-0.46806	-0.91176	-1.50984	-1.30041	-1.14952	p01452	Protein LAP	4	-0.8554		TCR signalin GO:0030522; P:intracellular receptor signaling pathway; GO:000[illegible]
ERBB2 seq:	-8.88799	-8.73745	-0.7516	-0.49783	-0.74715	-1.49938	-0.86133	-2.76753	p04568	Receptor ty	4	-3.09378		ErbB recept GO:0070372; P:regulation of ERK1 and ERK2 cascade; GO:0014[illegible]
ERBB2 seq:	-0.5873	-3.89135	-0.67836	-1.76664	0	0	0	0	p05757	Receptor ty	4	-0.86546		ERKinh1 (EI ErbB recept GO:0070372; P:regulation of ERK1 and ERK2 cascade; GO:0014[illegible]
ERBB2 seq:	-0.32321	-1.77769	-0.41676	-0.76765	0	0	0	0	p05758	Receptor ty	4	-0.41066		ERKinh1 (EI ErbB recept GO:0070372; P:regulation of ERK1 and ERK2 cascade; GO:0014[illegible]
ERBB2 seq:	-0.20418	-0.82011	-0.19813	-0.34695	0	0	0	0	p05760	Receptor ty	4	-0.19617		ERKinh1 (EI ErbB recept GO:0070372; P:regulation of ERK1 and ERK2 cascade; GO:0014[illegible]
ERBB2 seq:	-0.19219	-1.73117	-0.15768	-0.6303	0.308317	0.774737	0.308753	0.931746	p03614	Receptor ty	4	-0.04847		ERKinh1 (EI ErbB recept GO:0070372; P:regulation of ERK1 and ERK2 cascade; GO:0014[illegible]
ERBB2 pS1	-0.83054	-0.44853	-0.60692	-1.02358	-1.62501	-0.51713	-0.0952	-2.34541	p15660	Receptor ty	4	-0.93654		ErbB recept GO:0070372; P:regulation of ERK1 and ERK2 cascade; GO:0014[illegible]
ERBB2 pS1	0.871864	0.911459	0.805355	1.145105	-0.84992	0.066497	0.658641	-7.63411	p04569	Receptor ty	4	-0.50314		ErbB recept GO:0070372; P:regulation of ERK1 and ERK2 cascade; GO:0014[illegible]
EPS8L1 seq	-0.36946	-0.62025	-0.22054	-0.25764	-0.83486	-0.34179	-0.57641	-0.53407	p05133	Epidermal g	4	-0.46938		GO:0007266; P:Rho protein signal transduction;
EPS8 pS815	-0.32191	-0.21816	-0.04463	-0.99971	0.219483	0.405982	0.299149	0.417778	p13760	Epidermal g	4	-0.03025		Regulation GO:0016601; P:Rac protein signal transduction; GO:0009967; P:[illegible]
EPS15L1 se	0.866298	0.233789	0.082194	0.175778	-1.73632	-3.54167	-1.0379	-0.95065	p05251	Epidermal g	4	-0.73856		GO:0042059; P:negative regulation of epidermal growth factor re[illegible]
EPS15 pT77	-0.62457	0.02689	-0.52313	-0.37853	-0.41834	-0.10459	-0.39967	-0.53642	p14223	Epidermal g	4	-0.3698		Internalizati GO:0042059; P:negative regulation of epidermal growth factor re[illegible]
EPPK1 pM2	-0.33429	0.839681	-0.36115	0.090341	-0.5407	-0.10584	-0.76202	-0.35969	p20105	Epiplakin	4	-0.19171		
EPN3 seq: 5	0.555489	2.39567	1.40529	-7.72516	0	0	0	0	p09273	Epsin-3	4	-0.42109		
EPN3 seq: 4	-0.17369	0.383689	-0.07459	-0.08124	-1.18995	-0.09865	-0.63494	-0.40051	p17727	Epsin-3	4	-0.28374		
EPN3 seq: 4	0.275995	0.424693	0.083445	0.124554	-0.70323	-0.21546	-0.65826	-0.46754	p17728	Epsin-3	4	-0.14197		
EPN3 pS26	-0.54878	0.082717	-0.36345	-0.54128	-0.51058	-0.59979	-0.49123	-0.91257	p03182	Epsin-3	4	-0.48562		
EPN1 pT47	-0.32892	-0.7547	-0.21898	-0.0893	-0.26022	-0.10554	-0.1302	-0.07549	p12484	Epsin-1	4	-0.24542		Internalizati GO:0042059; P:negative regulation of epidermal growth factor re[illegible]
EPB41L2 se	-0.08243	-0.45019	0.015441	-0.65456	-0.10143	-0.1399	-0.30337	-0.25684	p03117	Band 4.1-lik	4	-0.24666		
EPB41 seq:	0.369932	-1.65999	0.147726	-0.15591	-0.63263	-1.66053	-0.20889	-0.29911	p11855	Protein 4.1	4	-0.51243		Syndecan-2 GO:0043408; P:regulation of MAPK cascade; GO:0060337; P:typ[illegible]
EP400 seq:	0.382055	0.179895	0.268664	0.321382	-0.52762	-0.4315	-0.11441	-0.60821	p02391	E1A-binding	4	-0.06622		
EP400 pS1	0.213547	-0.48262	0.312679	0.376814	-0.28817	-0.06661	0.109291	-1.09736	p14673	E1A-binding	4	-0.1153		
ENO1 seq:	-0.54013	0.266959	-0.99934	-0.85409	-0.40104	-0.22495	-0.48081	-0.60279	p12950	Alpha-enola	4	-0.47952		Notch signaling pathway; HIF-1-alpha transcription factor network; Validated [illegible]
ENO1 seq:	-0.32978	-1.13262	-0.45564	-0.46197	1.010045	0.338304	0.713834	0.757527	p06145	Alpha-enola	4	0.054963		Notch signaling pathway; HIF-1-alpha transcription factor network; Validated [illegible]
ENO1 seq:	-0.62852	-0.49159	-0.95683	-1.09083	0.67024	0.629697	0.414165	0.597857	p14282	Alpha-enola	4	-0.10698		Notch signaling pathway; HIF-1-alpha transcription factor network; Validated [illegible]
ENO1 seq:	-0.48485	-1.00987	-0.12156	0.112003	0.262289	0.149968	0.193628	0.7566	p12227	Alpha-enola	4	-0.01772		Notch signaling pathway; HIF-1-alpha transcription factor network; Validated [illegible]
ENO1 pS26	-0.27915	-1.38834	-0.13257	-0.16212	-0.03204	-0.34805	-0.14256	-0.01049	p05731	Alpha-enola	4	-0.31191		Notch signaling pathway; HIF-1-alpha transcription factor network; Validated [illegible]
ENAH seq:	-1.39088	-1.08261	-1.29802	-1.00554	-2.25641	-1.40841	-2.19233	-3.25083	p19469	Protein ena	4	-1.73563		E-cadherin GO:0050852; P:T cell receptor signaling pathway;
ENAH seq:	-0.36407	-0.46382	-0.32127	-1.60119	0	0	0	0	p15153	Protein ena	4	-0.34379		E-cadherin GO:0050852; P:T cell receptor signaling pathway;
ENAH pS53	-0.65016	-0.45218	-0.72181	-0.43676	-0.45885	-0.34926	-0.25547	-0.30618	p03172	Protein ena	4	-0.45383		E-cadherin GO:0050852; P:T cell receptor signaling pathway;
EMSY seq: 2	-0.17977	-0.16776	-0.37411	-0.17239	-0.38234	-0.38502	-0.50152	-0.37814	p02452	Protein EM	4	-0.31763		
EMSY seq: 2	-0.34181	-0.21273	-0.45643	-0.34526	-0.72002	-0.59459	-0.83273	-1.2666	p06516	Protein EM	4	-0.59627		
EMSY pS12	-0.21756	-0.28814	0.023672	-0.04008	-0.00473	-0.00855	-0.00414	-0.09057	p17181	Protein EM	4	-0.07876		
EMSY pS11	-0.27824	0.213655	-0.14699	0.084595	-0.33324	-0.17844	-0.23029	-0.52133	p10491	Protein EM	4	-0.17378		
EML4 seq:	-0.20183	-0.4799	-1.17671	-1.1526	0.238181	1.040808	0.734475	0.669338	p00503	Echinoderm	4	-0.04103		
EML4 pS14	-1.61869	-0.80461	-1.48757	-1.323	-0.11014	0.791394	0.841251	-1.59359	p19528	Echinoderm	4	-0.66312		
ELMSAN1 s	-0.3439	-0.01881	-0.03533	-0.57992	-0.37793	-1.43418	0.372028	0.427867	p02470	ELM2 and S	4	-0.24877		
ELK3 seq: 2	1.143858	0	0	0	-0.75704	-0.66744	-2.88447	-2.22515	p04413	ETS domain	4	-0.67378		GO:0007165; P:signal transduction;
EIF5B pS21	-0.03023	-0.29864	-0.0703	-0.17997	-0.25211	-0.4143	-0.53062	-0.52272	p09506	Eukaryotic t	4	-0.28736		PI3Kinh2 (PI-103); p70S6Kinh1 (PF-4708671);
EIF5B pS10	-0.30028	-0.39752	-0.36996	-1.06151	0.244404	0.014928	-0.08682	0.123145	p12650	Eukaryotic t	4	-0.2292		
EIF4H pS21	-0.02354	-0.7401	-0.04907	0.033351	0.106451	-0.33562	-0.15561	-0.21689	p19097	Eukaryotic t	4	-0.17263		
EIF4H pS14	-0.14032	-1.01336	-0.35069	-0.25405	0.464156	-1.87998	-1.32295	-0.47	p19099	Eukaryotic t	4	-0.6209		
EIF4G1 seq	-1.44755	-1.01848	-0.81117	-0.76166	0.365909	0.843677	0.558956	0.739425	p19945	Eukaryotic t	4	-0.19136		Validated ta GO:0008286; P:insulin receptor signaling pathway; GO:0019221[illegible]
EIF4G1 seq	0.027639	0.006887	-0.40671	-1.54183	-0.59037	-0.33584	-1.4736	-1.70261	p06678	Eukaryotic t	4	-0.75205		PAKinh (PF Validated ta GO:0008286; P:insulin receptor signaling pathway; GO:0019221[illegible]
EIF4G1 seq	-0.24012	-0.33623	-0.28701	-0.77226	0.512206	0.312921	0.511883	0.576097	p13602	Eukaryotic t	4	0.034686		Validated ta GO:0008286; P:insulin receptor signaling pathway; GO:0019221[illegible]
EIF4G1 pS1	0.083586	1.196279	-1.48939	-1.5315	-1.45016	0.021764	-0.56636	-0.63874	p04120	Eukaryotic t	4	-0.54682		Validated ta GO:0008286; P:insulin receptor signaling pathway; GO:0019221[illegible]
EIF4ENIF1 s	-0.37197	-0.22136	0.159606	-1.43544	-1.06849	-0.35851	-0.15597	-0.07334	p09056	Eukaryotic t	4	-0.44068		
EIF4ENIF1 p	-0.08912	0.075988	-0.0677	-0.00372	-0.06584	0.189183	-0.3269	-0.31902	p21056	Eukaryotic t	4	-0.07589		
EIF4EBP2 se	-0.51768	-0.13714	-0.33253	0.075202	-0.14375	-0.32078	-0.55777	-0.12803	p01987	Eukaryotic t	4	-0.25781		PI3Kinh2 (PI-103); mTO GO:0019933; P:cAMP-mediated signaling; GO:0008286; P:insuli[illegible]
EIF4EBP1 se	-1.70454	-1.00289	-1.37647	-1.42915	-1.14082	-0.95662	-1.73265	-1.22929	p09100	Eukaryotic t	4	-1.32155	mTOR; ERK2	mTORinh2 Signaling m GO:0031929; P:TOR signaling; GO:0008286; P:insulin receptor si[illegible]
EIF4EBP1 se	-0.75999	-0.40492	-0.75291	-0.89216	-0.89574	-0.5042	-0.9178	-0.837	p09101	Eukaryotic t	4	-0.74559	mTOR; ERK2	Up with IGF Signaling m GO:0031929; P:TOR signaling; GO:0008286; P:insulin receptor si[illegible]
EIF4EBP1 se	-1.16444	-0.31035	-0.44796	-0.66387	-0.77678	-0.73651	-0.37442	-0.82623	p09105	Eukaryotic t	4	-0.66257	mTOR; ERK2	Up with IGF Signaling m GO:0031929; P:TOR signaling; GO:0008286; P:insulin receptor si[illegible]
EIF4EBP1 se	-0.31771	-0.50925	-0.17624	-0.20811	-0.51161	-0.34303	-0.52001	-0.13469	p00607	Eukaryotic t	4	-0.34008	mTOR;	PI3Kinh2 (F Signaling m GO:0031929; P:TOR signaling; GO:0008286; P:insulin receptor si[illegible]
EIF4EBP1 se	-0.60297	-0.54115	-0.1434	-0.21637	-0.09681	-0.13886	-0.43486	-0.17584	p06419	Eukaryotic t	4	-0.29378	mTOR; ERK2	Akt-mTOR-F Signaling m GO:0031929; P:TOR signaling; GO:0008286; P:insulin receptor si[illegible]
EIF4E seq: 1	-0.12626	-0.0805	-0.39461	-0.30884	-0.49185	-0.35575	-0.49552	-0.75262	p14504	Eukaryotic t	4	-0.37574		Signaling m GO:0008286; P:insulin receptor signaling pathway; GO:0019221[illegible]
EIF4B seq: 4	-1.1063	-1.66438	-0.88389	-0.78018	0.87676	1.195503	-0.02373	0.27117	p03986	Eukaryotic t	4	-0.26438		mTOR signa GO:0008286; P:insulin receptor signaling pathway;
EIF4B seq: 2	0.076434	-0.53892	-0.07234	-0.08571	0.106994	-0.35857	-0.20166	0.037534	p20768	Eukaryotic t	4	-0.12953		mTOR signa GO:0008286; P:insulin receptor signaling pathway;
EIF4B pS35	0.023216	-0.658	-0.35635	0.254379	-0.22004	-0.67298	-0.3438	-0.23165	p21040	Eukaryotic t	4	-0.27565		mTOR signa GO:0008286; P:insulin receptor signaling pathway;
EIF4B pS20	-0.26219	-0.31778	-0.36934	-0.83698	0.125372	-0.13768	-0.4689	-0.32767	p19694	Eukaryotic t	4	-0.3244		mTOR signa GO:0008286; P:insulin receptor signaling pathway;
EIF4B pS20	-0.20916	-0.20547	-0.50422	-0.96466	-0.01261	0.106661	-0.17436	0.46288	p19695	Eukaryotic t	4	-0.18762		mTOR signa GO:0008286; P:insulin receptor signaling pathway;
EIF3J pS11	-1.0866	-0.73373	-0.32549	-0.47896	-0.25356	-0.2732	-0.52772	-0.25731	p21212	Eukaryotic t	4	-0.49207		AKTinh1 (Akt Inhibitor VIII);
EIF3I seq: 2	-0.48522	-0.4029	-0.6203	-0.73156	0.928853	1.191121	-0.09476	0.262989	p11905	Eukaryotic t	4	0.006028		
EIF3D seq:	-0.63089	-0.46724	-0.3681	-0.93387	-0.11289	-0.00579	-0.08687	0.089698	p18424	Eukaryotic t	4	-0.31449		
EIF3B seq: 1	-0.71945	-1.05791	-0.49473	-0.863	-1.12902	-0.54812	-1.38267	-1.75841	p06074	Eukaryotic t	4	-0.99416		
EIF3A seq: 8	-0.6648	-0.78367	-0.76749	-0.9168	-1.04829	-0.8068	-1.19467	-1.53595	p07178	Eukaryotic t	4	-0.96481		IL2 signaling events mediated by PI3K;
EIF3A seq: 8	-0.45906	-0.36514	-0.36272	-0.30071	-0.61165	-0.2847	-0.77033	-0.60314	p07179	Eukaryotic t	4	-0.46968		IL2 signaling events mediated by PI3K;
EIF3A pS58	-1.85817	-1.24058	-4.30784	-2.42709	-1.23372	-1.49263	-2.26708	-1.49342	p11925	Eukaryotic t	4	-2.04006		IL2 signaling events mediated by PI3K;
EIF3A pS13	-0.51399	-0.34824	-0.56119	-0.33561	-0.22751	0.087877	-0.46104	-0.30964	p06455	Eukaryotic t	4	-0.33367		IL2 signaling events mediated by PI3K;
EIF3A pS11	-0.43552	-0.31237	-0.5221	-0.46912	-0.47711	-0.05006	-0.60955	-0.58501	p19719	Eukaryotic t	4	-0.43261		IL2 signaling events mediated by PI3K;
EIF2A seq: 1	-0.44949	-0.15336	-0.604	-0.75507	-0.217	-0.14858	-0.01343	-0.07288	p08958	Eukaryotic t	4	-0.30173		Signaling ev GO:0009967; P:positive regulation of signal transduction;
EHMT1 seq	-0.52335	-0.30168	-0.53415	-0.35811	-0.30191	-0.1013	0.097295	-0.14936	p02948	Histone-lys	4	-0.27157		
EHD2 seq:	0	0	0	0	-0.15745	-0.51692	-2.19069	-1.32589	p00429	EH domain-	4	-0.52387		
EGLN1 pS1	-0.06959	-0.32923	-0.09612	-0.09034	-0.36163	0.112379	-0.50163	-0.26987	p20385	Egl nine hor	4	-0.20076		Hypoxic and oxygen homeostasis regulation of HIF-1-alpha; HIF-2-alpha trans[illegible]
EFS pS331	0.152902	-1.24243	-0.62776	-0.05078	0	0	0	0	p13161	Embryonal	4	-0.22101		GO:0035556; P:intracellular signal transduction;
EFS pS319	-0.45349	-0.54835	-0.54706	-0.36223	0	0	0	0	p06918	Embryonal	4	-0.23889		GO:0035556; P:intracellular signal transduction;
EFS pS298	-1.46651	-0.75866	-1.6508	-1.39868	-1.25952	-0.86532	-1.57409	-0.93358	p11081	Embryonal	4	-1.2384		GO:0035556; P:intracellular signal transduction;
EFHD2 seq:	-0.48238	-0.75537	-0.48395	-0.53368	-0.27716	-0.03102	-0.47862	-0.20092	p20906	EF-hand do	4	-0.40539	CDK2; CDK1;	
EFHD2 pS7	-0.5085	-0.81781	-0.57922	-0.64119	-0.36301	-0.10123	-0.34857	-0.42994	p07647	EF-hand do	4	-0.47368	CDK2; CDK1;	
EFHD2 pS7	-0.48238	-0.75537	-0.48395	-0.53368	-0.27716	-0.03102	-0.47862	-0.20092	p07649	EF-hand do	4	-0.40539	CDK2; CDK1;	
EFHD2 pS7	-0.49665	-0.77367	-0.46021	-0.454	-0.24757	-0.07498	-0.49349	-0.22473	p20905	EF-hand do	4	-0.40316	CDK2; CDK1;	
EFHD2 pS7	-0.49665	-0.77367	-0.46021	-0.454	-0.24757	-0.07498	-0.49349	-0.22473	p07648	EF-hand do	4	-0.40316	CDK2; CDK1;	
EFHD2 pS7	-0.65217	-0.8084	-0.06218	-0.3739	-0.19651	-0.26762	-0.33649	-0.23698	p20907	EF-hand do	4	-0.36678	CDK2; CDK1;	
EEPD1 pS2	-1.99604	-2.21045	-1.0927	-3.37577	0.060286	-1.47076	-0.16086	-0.47683	p05496	Endonuclea	4	-1.34039		
EEF2K seq:	-1.02844	-0.79361	-0.77052	-1.06843	-1.00696	-0.51079	-0.91795	-0.95882	p00066	Eukaryotic e	4	-0.88194	mTOR;	mTOR-PI3K Signaling m GO:0008286; P:insulin receptor signaling pathway;
EEF2K pS74	-0.26467	-0.41026	0.10226	-0.02567	-0.88126	-0.36165	-0.41078	-0.62335	p00068	Eukaryotic e	4	-0.35942	mTOR;	mTOR-PI3K Signaling m GO:0008286; P:insulin receptor signaling pathway;
EEF2K pS74	-0.09358	-0.33747	0.169004	0.018533	-0.30308	-0.11235	-0.16383	-0.33252	p00067	Eukaryotic e	4	-0.14441	mTOR;	mTOR-PI3K Signaling m GO:0008286; P:insulin receptor signaling pathway;
EEF2K pS72	-1.02844	-0.79361	-0.77052	-1.06843	-0.9861	-0.47501	-0.91795	-0.95882	p00069	Eukaryotic e	4	-0.87486	mTOR;	mTOR-PI3K Signaling m GO:0008286; P:insulin receptor signaling pathway;
EEF2K pS72	-0.93876	-0.78466	-0.82531	-0.87848	-0.82718	-0.40933	-0.63701	-0.918	p00070	Eukaryotic e	4	-0.77734	mTOR;	mTOR-PI3K Signaling m GO:0008286; P:insulin receptor signaling pathway;
EEF2 seq: 5	-1.36456	0.4837	-0.92142	-1.33475	0.901215	0.159954	0.013559	0.021266	p17029	Elongation f	4	-0.25513		mTOR signaling pathway;
EEF1G seq:	-0.76565	-0.07626	-1.2113	-0.94552	0.854461	0.122381	0.050716	0.179794	p02406	Elongation f	4	-0.22392		
EEF1D seq:	-0.98665	-0.83959	-1.11685	-1.60549	0.989816	0.613248	0.225081	0.258952	p05319	Elongation f	4	-0.30768		GO:0043123; P:positive regulation of I-kappaB kinase/NF-kappaB[illegible]
EEF1D seq:	-0.7326	0.065717	-0.23237	-0.37743	-1.33933	-1.94642	-2.02993	-1.3102	p16369	Elongation f	4	-0.98782		GO:0043123; P:positive regulation of I-kappaB kinase/NF-kappaB[illegible]
EEF1D seq:	-0.79482	0.160917	-1.05345	-0.86206	0.519999	0.304697	0.425305	0.5486	p19421	Elongation f	4	-0.09385		GO:0043123; P:positive regulation of I-kappaB kinase/NF-kappaB[illegible]
EEF1D seq:	-1.77254	-2.60737	-3.1372	-1.48377	-0.45873	-0.49029	-0.56093	-0.34231	p11147	Elongation f	4	-1.35664		GO:0043123; P:positive regulation of I-kappaB kinase/NF-kappaB[illegible]
EEF1D pS13	-0.47624	-0.70966	-0.46581	-0.44241	-0.11058	-0.14914	-0.28515	-0.35212	p19418	Elongation f	4	-0.37389	CDK1;	GO:0043123; P:positive regulation of I-kappaB kinase/NF-kappaB[illegible]
EEF1D pS13	-0.33391	-0.60934	-0.46253	-0.42529	-0.17548	-0.14351	-0.21512	-0.31161	p19419	Elongation f	4	-0.3346	CDK1;	GO:0043123; P:positive regulation of I-kappaB kinase/NF-kappaB[illegible]
EEA1 seq: 4	-0.59688	-0.20275	-0.59695	-0.5654	0.075728	-0.02359	0.102427	-0.52676	p08057	Early endos	4	-0.29177		
EDF1 seq: 9	-0.94221	0.225532	-0.05167	0.445935	-0.3902	0.268707	-2.99368	0.357811	p13927	Endothelial	4	-0.38497		
EDC3 pS13	-0.67279	-0.20945	-0.05834	-0.403	-0.39217	-0.27739	-0.46594	-0.58831	p04276	Enhancer o	4	-0.38342		
ECH1 seq: 1	-0.70225	0.354719	-3.12439	-1.10657	0.089778	0.587479	-1.65569	-1.85866	p00434	Delta(3,5)-I	4	-0.92695		
ECH1 seq: 1	-1.51357	-1.59151	-1.59173	-4.2507	0	0	0	0	p00163	Delta(3,5)-I	4	-1.11844		
EAF1 seq: 1	0.56515	-14.5524	0.405646	-0.31473	0.184635	-0.04892	-0.1271	0.079758	p02149	ELL-associa	4	-1.726		
DYNLRB2 se	-0.70095	0.544519	-0.55857	-0.7252	0.489469	0.345401	0.356921	0.57708	p18762	Dynein light	4	0.041083		
DYNC1LI2 s	-0.17016	-0.39301	-0.29089	-0.38699	-0.66201	-0.26621	-0.56408	-0.50392	p12492	Cytoplasmic	4	-0.40466		
DYNC1LI2 s	-0.68285	0.023441	-1.88852	-2.08259	0.018398	-0.17932	-0.10551	0.259548	p12495	Cytoplasmic	4	-0.57967		
DYNC1LI2 s	-0.54434	-0.74231	-0.18851	-0.2105	-0.59749	-0.32419	-0.63823	-0.70436	p12493	Cytoplasmic	4	-0.49374		
DYNC1LI2 s	-0.20488	-0.72983	-0.14875	-0.16152	0.113451	0.008595	0.002396	-0.03296	p16003	Cytoplasmic	4	-0.14419		
DYNC1LI2 p	-0.39375	-0.64722	-0.19783	-0.21237	-0.99725	-0.34364	-0.66467	-0.6964	p12494	Cytoplasmic	4	-0.51914		
DYNC1LI1 p	-0.25272	-1.07884	0.075313	-0.35836	-0.3618	-0.29236	-0.00132	-0.67014	p03249	Cytoplasmic	4	-0.36753		
DYNC1LI1 p	-0.19455	-0.67031	0.259715	-0.02022	0.000716	-0.0669	-0.09002	-0.34036	p03250	Cytoplasmic	4	-0.14024		
DYNC1LI1 p	-0.13837	-0.66107	0.019848	-0.15026	-0.0251	-0.13701	0.430452	0.036015	p08113	Cytoplasmic	4	-0.07819		
DYNC1H1 s	-0.63586	-1.42783	-0.74855	-0.74129	0.459429	0.38997	-0.20706	0.816101	p02941	Cytoplasmic	4	-0.26189		Lissencephaly gene (LIS1) in neuronal migration and development;
DVL3 seq: 1	0.431711	0.05174	0.15598	-0.05747	-0.36989	-0.45302	-0.24608	-0.31691	p02818	Segment po	4	-0.10049		Degradatior GO:0060071; P:Wnt signaling pathway, planar cell polarity pathw[illegible]
DVL2 seq: 4	-0.18404	-1.65711	-0.64257	-0.36069	-0.29612	-0.80005	-0.39455	-0.47982	p12356	Segment po	4	-0.60187		Degradatior GO:0060071; P:Wnt signaling pathway, planar cell polarity pathw[illegible]
DUT seq: 8	-0.02207	-0.22515	0.231501	0.282699	-0.59619	-0.20372	-0.46902	-0.63864	p20650	Deoxyuridir	4	-0.20507		
DUSP3 seq:	-0.61421	-0.83166	-0.17594	-0.37483	-0.34295	-0.27399	-0.21529	-0.1127	p17627	Dual specifi	4	-0.3677		GO:0050860; P:negative regulation of T cell receptor signaling pat[illegible]
DUSP16 pS	0.498186	1.150666	0.478564	0.274226	-0.42746	-1.027	-1.27462	-0.89983	p03192	Dual specifi	4	-0.15341		Regulation GO:0000188; P:inactivation of MAPK activity;
DTL seq: 67	-0.44868	-0.96272	-0.66918	-0.34283	-0.98911	-0.80047	-1.52724	-0.98631	p13447	Denticleless	4	-0.84082		
DTL seq: 48	0.046162	-0.7605	0.586365	0.531648	-0.63664	-0.34157	0.300371	-0.11269	p15140	Denticleless	4	-0.04836		
DTL seq: 48	0.025507	-1.02266	-0.7484	-0.35509	-2.67344	-1.49604	-3.0957	-1.06533	p07309	Denticleless	4	-1.30389		
DTL seq: 48	-0.00203	-0.10846	-0.51363	-0.23148	-0.36372	-0.11135	-0.77662	-0.28802	p07310	Denticleless	4	-0.29941		
DTL seq: 41	-0.54392	-0.42207	-0.0657	-0.11558	-0.58619	-0.38721	-0.3452	-0.4396	p17128	Denticleless	4	-0.36318	CDK2;	
DTL pT429	-0.60941	-0.29639	0.102197	-0.0418	-0.60829	-0.2964	-0.28773	-0.41158	p17129	Denticleless	4	-0.30618	CDK2;	
DTD1 pS19	-9.00917	1.514657	1.250539	2.084122	0.150221	-0.13932	0.002346	-0.11824	p06225	D-tyrosyl-tR	4	-0.53311		
DSP seq: 46	0	0	0	0	-2.35356	-0.3658	1.216205	-0.1812	p15028	Desmoplaki	4	-0.21054		Posttranslational regulation of adherens junction stability and dissassembly;
DSP pS22	-0.79336	-2.06152	-0.61956	-0.40933	-0.4249	-0.5565	-0.43914	-0.52645	p10066	Desmoplaki	4	-0.72884		Posttranslational regulation of adherens junction stability and dissassembly;
DSP pS176	-0.60448	-1.009	-0.12809	-0.31237	0.000378	-0.40581	0.276644	-2.21198	p16053	Desmoplaki	4	-0.54934		ROCKinh2 (Posttranslational regulation of adherens junction stability and dissassembly;
DSP pM28	-0.46385	-0.71994	-0.4012	-0.45682	-0.48504	-0.25251	-1.34648	-1.15688	p15671	Desmoplaki	4	-0.66034		Posttranslational regulation of adherens junction stability and dissassembly;
DSG2 seq:	-0.27866	-1.75703	-0.06182	-0.50224	-1.45515	-1.15922	-0.59163	-0.99022	p15159	Desmoglein	4	-0.8495		
DSG2 seq:	0.427096	0.083079	-2.69676	-0.87725	0.074468	0.038619	0.225632	-0.0513	p14073	Desmoglein	4	-0.34705		
DPYSL2 seq	-0.06979	-2.22419	-1.03413	-0.02598	-0.50167	-0.33595	-0.52002	-0.61244	p02161	Dihydropyr	4	-0.66552	DYRK2; CDK5;	GO:0007165; P:signal transduction;
DPYSL2 seq	-0.19714	-0.66794	-0.41665	-0.4251	-0.60581	-0.47282	-0.68351	-0.80337	p01878	Dihydropyr	4	-0.53404	DYRK2; CDK5;	GO:0007165; P:signal transduction;
DPYSL2 pS	-0.36825	-0.56747	-0.31712	-1.06784	-0.20989	-0.64132	-0.68613	-0.17657	p09455	Dihydropyr	4	-0.50432		GO:0007165; P:signal transduction;
DPF2 pT17	0.199745	-0.01976	0.161251	0.287923	-2.16163	-0.41387	-0.04403	-0.54179	p14055	Zinc finger	4	-0.31652	CDK2;	AKTinh2 (MK-2206);
DOCK7 seq	-0.52291	-1.41897	-0.11635	-0.78499	0.293075	-0.24463	-0.2454	-0.01987	p04044	Dedicator o	4	-0.38251		ErbB2/ErbB GO:0007264; P:small GTPase mediated signal transduction;
DOCK7 pS9	-0.39609	-0.18058	-0.27327	-1.55736	0.265573	0.071736	0.097026	0.138271	p04751	Dedicator o	4	-0.22934		ErbB2/ErbB GO:0007264; P:small GTPase mediated signal transduction;
DOCK6 seq	-0.98361	-0.40067	0.261681	-0.4672	0	0	0	0	p05368	Dedicator o	4	-0.19872		Regulation GO:0007264; P:small GTPase mediated signal transduction;
DOCK4 seq	-0.62883	-1.31173	-4.02759	-0.10393	0.24397	-0.0084	0.124052	0.688906	p14575	Dedicator o	4	-0.62794		PDGFR-bet GO:0007264; P:small GTPase mediated signal transduction;
DOCK3 pS8	-0.43116	3.005682	-0.0736	-0.30595	-2.08293	-3.14135	-0.67207	-1.27659	p10935	Dedicator o	4	-0.62225		GO:0007264; P:small GTPase mediated signal transduction;
DOCK1 seq	0.128593	-1.68417	-0.05647	0.04524	-0.3153	-1.18738	-0.34548	-2.26879	p07414	Dedicator o	4	-0.71047		Netrin-med GO:0007229; P:integrin-mediated signaling pathway; GO:003809[illegible]
DNTTIP2 se	1.303998	-0.32075	0.330552	-0.80019	-0.64482	-0.8491	-0.49438	-0.68549	p09416	Deoxynucle	4	-0.27002		
DNTTIP2 pS	-0.07904	1.169945	-0.29829	-0.52679	-0.2843	-0.22851	-0.46498	-0.70409	p04582	Deoxynucle	4	-0.17701		
DNMT1 pS	0.077672	-0.2418	-0.35335	-0.53187	-0.75773	-0.60504	-0.46253	-0.98538	p06466	DNA (cytos	4	-0.48251		Regulation of retinoblastoma protein;
DNAJC9 pS	0.118336	0.275131	-0.36171	-0.35863	-0.52495	-0.561	-0.50356	-0.2013	p13069	DnaJ homo	4	-0.26471		
DNAJC17 p	-0.05862	0.747516	-0.25292	0.357179	-0.16077	-0.14709	-0.15185	-0.97977	p17208	DnaJ homo	4	-0.08079		
DNAJB1 se	-0.45486	0.505923	-0.54038	-0.95032	1.146709	-0.1169	-1.94168	0.232673	p18798	DnaJ homo	4	-0.26485		
DNAJB1 se	0.130775	0.066614	-0.31547	-0.7089	0.450341	0.156882	0.512784	0.02137	p17766	DnaJ homo	4	0.0393		
DMXL2 seq	-0.68283	-0.86055	-0.43802	-0.37281	0.402934	-0.13346	-0.09167	-0.14544	p13911	DmX-like pr	4	-0.29023		
DMXL1 seq	0.348583	1.014497	0.816178	0.940002	-0.83625	-5.75917	-0.4585	-0.60883	p18927	DmX-like pr	4	-0.56794		
DMAP1 se	1.534137	-8.05018	0.413479	0.096558	-1.24289	-0.95146	-0.41968	-0.88137	p20544	DNA methy	4	-1.18768	CDK2;	
DLGAP4 se	0.947503	-0.01822	-0.29603	-0.12238	0.002352	-2.91467	-1.71081	-0.42852	p07225	Disks large-	4	-0.5676		GO:0007267; P:cell-cell signaling;
DLGAP4 se	0.295242	0.069201	0.211331	0.075157	-0.35892	-0.66568	-0.30014	-0.40658	p05943	Disks large-	4	-0.13505		GO:0007267; P:cell-cell signaling;
DLGAP4 pS	-0.30521	-0.54925	-0.05891	-0.24962	-0.08903	-0.73794	-0.85977	-0.39502	p08193	Disks large-	4	-0.40559		mTORinh2 (Torin-1); m[illegible] GO:0007267; P:cell-cell signaling;
DLGAP4 pS	-0.35154	-0.46387	-0.20272	-0.80705	-0.37601	-2.19411	-0.59271	-0.11031	p09396	Disks large-	4	-0.63729		GO:0007267; P:cell-cell signaling;
DLGAP4 pS	-0.12019	-0.38012	-0.38164	-0.63506	0.389392	0.565549	-3.05424	0.257074	p09398	Disks large-	4	-0.4199		GO:0007267; P:cell-cell signaling;
DLGAP4 pS	-0.2703	-0.69637	-0.3026	-0.56416	0.367852	0.147627	-0.5979	0.291007	p09397	Disks large-	4	-0.20311		GO:0007267; P:cell-cell signaling;
DLGAP4 pS	-0.21371	-0.36512	-0.31514	-1.0816	0	0	0	0	p05124	Disks large-	4	-0.24695		GO:0007267; P:cell-cell signaling;
DLG5 seq:	0.210251	-0.2037	-0.48901	-0.2845	-1.81379	-1.93888	-1.53926	-2.15812	p07109	Disks large	4	-1.02713		GO:0035556; P:intracellular signal transduction; GO:0007165; P[illegible]
DLG5 pT10	0.057741	0.432216	-0.26096	-0.10949	-0.44769	0.025852	-0.10162	-0.21053	p20601	Disks large	4	-0.07681		GO:0035556; P:intracellular signal transduction; GO:0007165; P[illegible]
DLG1 pM5	-0.58543	-0.97051	-0.29083	-0.36143	0.214289	-0.19989	0.239337	0.170072	p17274	Disks large	4	-0.22305		E-cadherin GO:0032147; P:activation of protein kinase activity;
DIS3 seq: 5	-0.6134	-0.44059	-1.10893	-0.49942	0.656026	0.135936	0.407443	-0.16106	p19572	Exosome cc	4	-0.203		
DIP2B seq:	0.022142	0.025377	0.952479	-0.54601	-1.2032	-0.9706	-1.14665	-1.49649	p05486	Disco-inter	4	-0.54537		
DIP2B pS2	-0.00459	-1.07772	0.272062	0.161012	-0.14413	-0.56806	-0.02272	0.122131	p15072	Disco-inter	4	-0.15775		
DIDO1 seq:	-0.2623	-0.55822	0.00564	-0.03219	0.04316	-0.20369	-0.13171	-0.45739	p05521	Death-indu	4	-0.19959		
DHX9 seq:	-0.24865	-0.37633	-0.15374	0.05004	-0.74706	0.242832	0.708351	0.298863	p06038	ATP-depen	4	-0.02821		
DHX29 pS2	-0.03798	0.131816	-0.99612	-0.04605	-0.21	-0.14807	-0.3021	-0.19025	p16510	ATP-depen	4	-0.22484		
DHX16 pS1	0.078698	0.178667	0.176905	-0.09386	-0.22198	-0.19829	0.097762	-0.31072	p07999	Putative pre	4	-0.0366		
DHRS2 seq	-1.55685	-0.5086	-3.38092	-1.85151	0	0	0	0	p13979	Dehydroger	4	-0.91224		
DGCR8 pS9	-0.19626	-0.50185	0.258059	0.168089	-0.35723	-0.44768	-0.41762	-0.57479	p11383	Microproce	4	-0.25866		Direct p53 effectors;
DGCR8 pS9	0.102051	-0.63672	0.351375	0.123545	-0.31369	-0.37166	-0.33071	-0.40304	p11384	Microproce	4	-0.18486		Direct p53 effectors;
DGCR14 se	-0.17184	-0.70265	-1.40011	-1.81907	0.257858	0.344718	0.000211	0.115455	p00733	Protein DG	4	-0.42193		
DFFA pS11	-0.34838	-0.71484	-0.21112	-1.11878	-0.21223	-0.8923	-0.18188	-0.49768	p00509	DNA fragme	4	-0.52215		HIV-1 Nef: Negative effector of Fas and TNF-alpha; Caspase cascade in apopto[illegible]
DENND4C s	0.359208	-0.20389	0.543423	0.776538	-0.88693	0.050824	-0.90501	-0.90861	p03831	DENN dom	4	-0.14681		
DENND4C s	-0.66921	-1.21583	-0.77495	-1.24938	0.067923	-0.78005	-3.02494	-3.38637	p12543	DENN dom	4	-1.3791		
DENND4C s	0.317859	-0.40803	-0.05689	0.298084	0.323801	-0.22291	-0.0006	0.155194	p03451	DENN dom	4	0.050813		PAKinh (PF3758309);
DENND4B s	0.368914	-1.54118	-0.96529	-0.58766	-1.06354	-1.00098	-1.09091	-1.60242	p12955	DENN dom	4	-0.93538		
DENND4A s	-0.08436	-0.24117	0.067783	-0.02695	-0.47055	-0.52586	-0.70274	-0.35464	p03774	C-myc pron	4	-0.29231		
DENND4A p	-0.77535	0.206384	0.228403	-0.13609	-0.56577	-0.57975	-1.63954	-1.94962	p03659	C-myc pron	4	-0.65142		
DENND1A s	-1.48591	1.435447	-1.55616	-2.52296	0	0	0	0	p10322	DENN dom	4	-0.5162		
DENND1A s	0.416053	-0.58904	-0.01618	0.267844	-0.65536	-0.0883	-0.50173	-0.53981	p06478	DENN dom	4	-0.21331		
DENND1A p	-0.2896	-0.78698	-0.12106	-0.15012	0.176458	-0.85006	-0.07968	-0.34334	p02064	DENN dom	4	-0.30555		
DDX6 seq:	-0.92932	-0.62188	-0.99666	-0.5208	-0.07899	-0.32174	0.060182	-0.50128	p15419	Probable AT	4	-0.48881		
DDX5 seq:	-0.45415	-0.29817	-0.72681	-1.10367	0.390348	0.030293	0.441439	0.577655	p11306	Probable AT	4	-0.14288		Direct p53 GO:0043517; P:positive regulation of DNA damage response, sig[illegible]
DDX46 pS8	0.046503	-0.5601	-0.23901	-0.01284	-0.75033	-0.47872	-0.28967	0.051578	p21100	Probable AT	4	-0.27908		
DDX3X seq	0.274128	-0.85515	0.345493	0.303215	0.36413	-0.49024	0.086962	0.174495	p03910	ATP-depen	4	0.025379		GO:0035556; P:intracellular signal transduction;
DDX3X seq	-0.93473	-1.80956	-1.6357	-1.43417	0.493575	-0.51248	-1.14401	-0.03321	p03911	ATP-depen	4	-0.87629		GO:0035556; P:intracellular signal transduction;
DDX3X seq	-0.27319	0.296908	-0.44491	-1.00462	0.507757	-0.26228	0.48441	-5.95279	p17530	ATP-depen	4	-0.83109		GO:0035556; P:intracellular signal transduction;
DDX39A se	-0.3027	-0.09954	-0.81357	-0.69924	-1.53716	1.287903	0.869482	0.540714	p18877	ATP-depen	4	-0.09426		
DDX20 seq	-0.74409	1.265502	-0.49942	-0.42627	-0.01176	0.196146	-0.1633	0.028354	p09046	Probable AT	4	-0.04435		
DDX10 seq	-0.43092	-0.85747	-0.39602	-0.50561	-0.64975	-0.47382	-0.3942	-0.78485	p15796	Probable AT	4	-0.56158		
DDI2 seq: 1	0.070472	0.703521	-0.11057	-0.08408	-0.40991	0.007793	-0.28762	-0.2667	p07962	Protein DD	4	-0.04714		
DDB2 seq:	0.001244	-0.86146	0.088627	0.015554	-0.15367	-0.05098	0.051118	-0.26303	p04031	DNA damag	4	-0.14657		Direct p53 effectors;
DDA1 pS33	0.105599	0.060919	-0.44594	0.003218	0.058734	0.208702	-1.1476	-0.28931	p06832	DET1- and	4	-0.18071	AurB;	
DCLK1 seq:	-0.76252	-0.60525	-0.19849	-1.18376	-6.62019	1.827335	1.995948	1.473161	p01712	Serine/thre	4	-0.50922		
DCLK1 seq:	-0.78973	-0.90078	-0.32751	-0.45942	-1.3537	-0.67734	-1.85968	-1.56394	p19174	Serine/thre	4	-0.99151		
DCLK1 pS1	-1.04589	-1.37283	-0.35644	-0.77858	0	0	0	0	p19175	Serine/thre	4	-0.44422		
DCAF7 seq:	0.04584	-0.28375	-0.19425	0.048515	-0.21858	-0.09732	-0.10179	-0.22243	p12007	DDB1- and	4	-0.12797		
DBP pS86	0.180597	-0.83191	0.199684	-0.00265	0	0	0	0	p13473	D site-bindi	4	-0.05678		
DBNL seq:	-2.44752	-2.3162	-1.64134	-1.62842	0	0	0	0	p20072	Drebrin-like	4	-1.00418	CDK2;	JNK signalir GO:0016601; P:Rac protein signal transduction;
DBNL seq:	-0.24377	-0.64638	0.006387	-0.22263	-0.61414	-0.76955	-0.18027	-0.27221	p17253	Drebrin-like	4	-0.36782		PAKinh (PF JNK signalir GO:0016601; P:Rac protein signal transduction;
DBNL seq:	-0.07198	0.098959	-0.19829	-0.38644	-0.00626	0.265721	0.363624	0.007943	p16522	Drebrin-like	4	0.009159		JNK signalir GO:0016601; P:Rac protein signal transduction;
DBNL pT29	-0.1892	-0.26578	-0.09912	-0.69781	-0.49335	-0.63022	-0.55941	-0.59631	p08265	Drebrin-like	4	-0.4414		PAKinh (PF JNK signalir GO:0016601; P:Rac protein signal transduction;
DBNL pS23	-0.14671	-0.55208	0.067111	-0.30576	-0.29394	-0.18377	-0.39669	-0.41334	p00164	Drebrin-like	4	-0.27815		JNK signalir GO:0016601; P:Rac protein signal transduction;
DBNL pM2	-0.50977	-0.84503	-0.45357	-0.71144	0.037968	-0.3427	-0.70594	-0.23576	p20071	Drebrin-like	4	-0.47078		PAKinh (PF JNK signalir GO:0016601; P:Rac protein signal transduction;
DAXX seq:	0.023788	-0.68692	0.59331	0.145796	0.016438	-0.03291	0.214806	0.14947	p01660	Death dom	4	0.052972		HIV-1 Nef: GO:0030521; P:androgen receptor signaling pathway;
DAP seq: 3	1.106611	1.651989	-0.09609	-1.06322	-1.32855	-1.09352	-0.31667	-0.64599	p17837	Death-asso	4	-0.22318	mTOR;	Up with IGF Arf6 signali GO:0007166; P:cell surface receptor signaling pathway;
DAB2 pS40	-0.53182	0.186824	-0.80153	-0.64121	0.016082	-0.1825	-0.29031	-0.21722	p03800	Disabled ho	4	-0.30771		TGF-beta re GO:0060766; P:negative regulation of androgen receptor signalin[illegible]
CUL4B seq:	-1.00538	-0.06616	0.089707	-0.10895	-0.50612	-0.56109	-0.48789	-0.64428	p10275	Cullin-4B	4	-0.41127		
CTU2 seq: 4	-1.72468	-0.7257	-0.43166	-0.61112	0.141963	0.262248	0.050176	0.187279	p02358	Cytoplasmic	4	-0.35644		
CTTNBP2NL	-0.31577	-0.52581	-0.53306	-0.4304	-0.37226	-0.26454	-0.2076	-0.06508	p00777	CTTNBP2 N	4	-0.33932		
CTTNBP2NL	-0.69389	0.876317	-0.76254	-0.17213	0.200533	0.113324	-0.07466	0.308059	p00779	CTTNBP2 N	4	-0.02562		
CTTNBP2NL	-1.73272	-1.4223	-0.75361	-1.82303	0.217395	0.120928	0.48763	-0.96067	p11390	CTTNBP2 N	4	-0.7333		
CTTNBP2NL	-1.77599	0.156446	-2.01156	-0.37114	0.306465	0.014298	0.31702	-1.24829	p11391	CTTNBP2 N	4	-0.57659		
CTTNBP2 se	-0.35552	-1.00432	-0.43098	-0.34675	0	0	0	0	p15299	Cortactin-b	4	-0.2672		
CTTN seq: 3	-0.12628	-0.60921	-0.23455	-0.22378	-0.71346	-0.63481	-0.66618	-0.81669	p20346	Src substrat	4	-0.50312		Syndecan-3-mediated signaling events; N-cadherin signaling events; E-cadheri[illegible]
CTTN seq: 3	0.022578	-0.03103	-0.25944	-0.21752	-0.90047	-0.79662	-0.79196	-0.66732	p20347	Src substrat	4	-0.45522		Syndecan-3-mediated signaling events; N-cadherin signaling events; E-cadheri[illegible]
CTTN seq: 3	-0.12069	-0.50177	-0.20198	-0.09873	-0.29409	-0.328	-0.41648	-0.25395	p20353	Src substrat	4	-0.27696		Syndecan-3-mediated signaling events; N-cadherin signaling events; E-cadheri[illegible]
CTTN pS26	-0.23281	-0.2395	-1.43561	-1.23774	-0.46161	-0.7023	-2.10052	-1.62238	p10979	Src substrat	4	-1.00406		Syndecan-3-mediated signaling events; N-cadherin signaling events; E-cadheri[illegible]
CTPS1 seq:	-0.38209	0.169497	-0.40034	-0.36599	-0.52628	-0.04017	-0.58966	-0.55328	p05649	CTP syntha	4	-0.33604		
CTPS1 seq:	-0.43105	0.623246	-0.45614	-0.38506	-0.72981	-0.04793	-0.68464	-0.52275	p05650	CTP syntha	4	-0.32927		
CTPS1 seq:	-0.66275	-0.78413	-0.7175	-0.38626	0.179938	0.073863	-0.11049	0.031524	p18137	CTP syntha	4	-0.29698		
CTPS1 seq:	-0.50215	-0.60087	-0.02408	0.136818	0.053098	-0.39285	-0.49578	-0.06894	p18138	CTP syntha	4	-0.23684		
CTPS1 seq:	-0.53916	-0.93377	-0.72315	-0.38626	0.184405	0.071146	-0.11833	0.031524	p18140	CTP syntha	4	-0.3017		
CTNND2 se	0.71325	-0.9462	0.127429	0.112435	-0.61653	-0.84785	-0.57539	-0.31124	p02779	Catenin del	4	-0.29301		GO:0007165; P:signal transduction;
CTNND2 se	0.427761	-0.74072	0.009309	-0.08629	0	0	0	0	p02780	Catenin del	4	-0.04874		GO:0007165; P:signal transduction;
CTNND2 se	-0.17471	-0.7374	-0.0459	-0.2456	0	0	0	0	p19428	Catenin del	4	-0.15045		GO:0007165; P:signal transduction;
CTNND1 se	0.179439	-1.16604	0.211623	0.09312	-0.13548	-0.11761	0.091612	0.269196	p02624	Catenin del	4	-0.07177		ROCKinh1 (E-cadherin GO:0090090; P:negative regulation of canonical Wnt signaling pa[illegible]
CTNND1 se	-0.18756	-0.44405	-0.22608	-0.1585	-0.24217	-0.5691	-0.06195	-0.335	p04164	Catenin del	4	-0.27805		E-cadherin GO:0090090; P:negative regulation of canonical Wnt signaling pa[illegible]
CTNND1 se	-0.29613	-0.61347	-1.07356	-2.05032	-0.08359	0.337139	0.339836	0.34959	p00168	Catenin del	4	-0.38631		E-cadherin GO:0090090; P:negative regulation of canonical Wnt signaling pa[illegible]
CTNND1 se	-0.35511	-0.90547	0.068046	0.025406	-0.05706	-0.26904	-0.04723	0.068723	p01484	Catenin del	4	-0.18397		E-cadherin GO:0090090; P:negative regulation of canonical Wnt signaling pa[illegible]
CTNND1 se	-1.01756	-10.1726	-0.13155	-0.67278	0.391023	0.149598	0.864179	0.556687	p01925	Catenin del	4	-1.25412		E-cadherin GO:0090090; P:negative regulation of canonical Wnt signaling pa[illegible]
CTNND1 pS	-0.34675	-0.61372	-0.1	0.048694	0.110774	-1.07094	-0.08928	-0.06886	p06109	Catenin del	4	-0.26626		E-cadherin GO:0090090; P:negative regulation of canonical Wnt signaling pa[illegible]
CTNND1 pS	-0.25338	-0.6937	0.032374	-0.04705	-0.34708	-0.41249	-0.27369	-0.29981	p19885	Catenin del	4	-0.28685		E-cadherin GO:0090090; P:negative regulation of canonical Wnt signaling pa[illegible]
CTNND1 pS	-0.15877	-0.8285	-0.17362	-0.36134	0.300027	-0.0333	0.151029	0.077349	p14549	Catenin del	4	-0.12839		E-cadherin GO:0090090; P:negative regulation of canonical Wnt signaling pa[illegible]
CTNND1 pS	-0.0612	-0.63063	-0.0055	-0.09797	0.297905	-0.04576	0.081884	0.064984	p14548	Catenin del	4	-0.04954		E-cadherin GO:0090090; P:negative regulation of canonical Wnt signaling pa[illegible]
CTNND1 pS	-0.39794	-1.4355	0.223396	0.161564	0.076649	-0.63378	0.026536	0.213624	p01927	Catenin del	4	-0.22068		E-cadherin GO:0090090; P:negative regulation of canonical Wnt signaling pa[illegible]
CTNND1 pM	-0.88411	-2.98713	-0.19063	-0.14637	0.204273	-0.49078	-0.0889	0.066484	p01928	Catenin del	4	-0.56465		E-cadherin GO:0090090; P:negative regulation of canonical Wnt signaling pa[illegible]
CTNNB1 se	0.545599	-0.21984	0.133267	0.171182	-0.12108	-0.37234	-0.23585	-0.24276	p06489	Catenin bet	4	-0.04273		E-cadherin GO:0030521; P:androgen receptor signaling pathway; GO:00713[illegible]
CTNNB1 se	0.536528	-0.34586	0.063778	0.057879	-0.49636	-0.53801	-0.503	-0.76028	p06491	Catenin bet	4	-0.24817		E-cadherin GO:0030521; P:androgen receptor signaling pathway; GO:00713[illegible]
CTNNB1 pS	0.487198	-0.0741	-0.03375	0.104776	-0.20181	-0.53092	-0.21351	-0.31373	p06490	Catenin bet	4	-0.09698	PKACA; Akt2; Akt1;	E-cadherin GO:0030521; P:androgen receptor signaling pathway; GO:00713[illegible]
CTDSPL2 se	-2.7476	-1.02981	-2.61784	-1.18039	-0.16303	-0.15982	0.308345	-0.19201	p18763	CTD small p	4	-0.97277		
CTDP1 seq:	-0.75475	-0.91305	-0.31278	-0.25143	0.135038	0.033014	0.327666	0.345617	p20292	RNA polym	4	-0.17383		
CSTF2T pS5	-0.19733	-0.66088	-0.02011	0.009359	0	0	0	0	p08646	Cleavage sti	4	-0.10862		
CSTF2 seq:	-0.27684	-0.82981	0.094062	0.157484	0.3786	0.103608	0.093455	0.133017	p07710	Cleavage sti	4	-0.0183		
CSPP1 pS9	0.219179	0.180242	-0.17942	-0.06335	-0.48468	-0.51791	-0.39838	-0.25251	p10938	Centrosom	4	-0.1871		
CSNK1E se	-0.3144	-0.32073	0.177388	0.04475	0.018183	0.02053	0.342606	0.214249	p13801	Casein kina	4	0.022823		Circadian rh GO:0030178; P:negative regulation of Wnt signaling pathway; GC[illegible]
CSNK1E pS	-0.32419	-0.49229	-0.1695	-0.10367	-0.58821	-0.13497	-0.3641	-0.2879	p16283	Casein kina	4	-0.3081	AMPKA1;	Circadian rh GO:0030178; P:negative regulation of Wnt signaling pathway; GC[illegible]
CSNK1D pS	-0.16844	-0.62693	-0.10899	-0.11728	-0.16255	-0.30356	-0.12041	0.038757	p16273	Casein kina	4	-0.19617		Degradatior GO:0090263; P:positive regulation of canonical Wnt signaling pa[illegible]
CSF3R seq:	-0.65585	-0.69543	-1.31729	-1.01406	1.052857	0.629132	0.341684	0.367188	p11947	Granulocyte	4	-0.16147		GO:0007165; P:signal transduction;
CSDE1 seq:	-0.21161	-0.12624	-0.57208	-0.48751	0.091116	0.267252	-0.26196	-0.31728	p09220	Cold shock	4	-0.20229		Validated targets of C-MYC transcriptional repression;
CSDE1 pS1	-3.01928	0.225725	-0.82638	-0.80545	-0.43278	-0.50876	-0.50172	-0.40969	p04735	Cold shock	4	-0.78479		Validated targets of C-MYC transcriptional repression;
CRYBG3 se	0	0	0	0	-1.25977	-1.19616	-1.4308	-0.85674	p09179	Very large A	4	-0.59293		
CRTC3 seq:	-0.24428	0.871198	0.224809	-0.13487	0.216494	-0.25031	-0.65406	-0.35315	p11861	CREB-regul	4	-0.04052		
CRTC3 pS3	-0.49565	-0.5436	0.086028	-0.01835	0.379258	-0.01361	0.006664	0.59765	p04821	CREB-regul	4	-0.0002		
CRTC2 seq:	0.120779	-0.10011	0.133809	0.099378	-0.14217	-0.00916	-0.29273	0.017571	p08670	CREB-regul	4	-0.02158		LKB1 signaling events;
CRIP2 seq:	-0.119	-0.25322	-0.01297	-0.72302	-1.57093	-1.97125	-1.54533	-0.8141	p19460	Cysteine-ric	4	-0.87623		
CRIP2 seq:	-0.1419	-0.18686	0.048436	-0.52792	-0.14105	-0.21329	-0.36284	-0.32281	p19461	Cysteine-ric	4	-0.23103		
CRIP2 seq:	-0.59474	-2.55311	-0.00594	-1.72158	0	0	0	0	p15432	Cysteine-ric	4	-0.60942		
CRIP2 seq:	-0.52575	-2.6557	-0.20333	-1.23603	0	0	0	0	p19613	Cysteine-ric	4	-0.5776		
CREM seq:	0.218444	0.202728	0.44029	0.436937	0.364446	-0.63843	-7.73092	1.021387	p19869	cAMP-resp	4	-0.71064		Calcium sig GO:0007165; P:signal transduction;
CRABP2 se	-0.96136	-0.67969	-1.18967	-1.40266	0.188473	1.041224	-1.12802	0.295807	p14460	Cellular reti	4	-0.47949		GO:0007165; P:signal transduction;
CPNE6 seq:	-0.6051	-0.43229	-1.10063	-0.49112	1.1907	0.685351	0.315269	0.13612	p08122	Copine-6	4	-0.03771		
CORO7 seq	-0.06997	-0.47436	0.112812	-1.37961	0	0	0	0	p11725	Coronin-7	4	-0.22639		
CORO7 pS4	-0.47245	-0.76067	-0.18438	-0.59272	0	0	0	0	p05078	Coronin-7	4	-0.25128		
CORO1C se	-0.61469	-1.74399	-1.34922	-0.65794	-0.32822	-0.27821	-0.76032	-0.28517	p14777	Coronin-1C	4	-0.75222		GO:0007165; P:signal transduction;
COL17A1 p	0	0	0	0	-0.25674	-0.14858	-0.0921	-2.45268	p07883	Collagen alp	4	-0.36876		Syndecan-1-mediated signaling events; a6b1 and a6b4 Integrin signaling; Inte[illegible]
COIL seq: 2	-0.00252	-0.20191	-0.04352	-0.1844	-0.22664	-0.11057	-0.20754	-0.12751	p09085	Coilin	4	-0.13807		
COG2 seq:	0.51455	-1.69614	-0.15979	0.571669	0.089477	-3.43044	-0.49902	0.763902	p12770	Conserved	4	-0.48072		
COBLL1 se	-0.53191	0.728028	-0.63508	-0.09371	-0.25595	-0.17186	-0.26899	-0.13172	p17079	Cordon-ble	4	-0.17015		
COBL seq:	0.338497	-1.55401	-0.93751	-0.68904	0.359709	0.264221	0.264108	-0.47425	p12899	Protein cor	4	-0.30353		
COBL seq: 8	0.056098	-2.8835	-0.82651	-0.20844	-0.32842	-4.95992	0.664996	0.645781	p06482	Protein cor	4	-0.97999		
COBL pS32	0.158248	-0.89744	0.02552	0.200235	-0.71019	-2.79893	-1.18913	-0.42932	p00826	Protein cor	4	-0.70513		
CNTNAP3 s	-0.78367	-0.31787	-1.29135	-0.84611	1.034531	0.193806	0.286706	0.377103	p10962	Contactin-a	4	-0.16836		
CNOT2 pM	-0.31075	-0.85472	-0.12864	-0.04763	-0.30453	-0.25335	-0.06707	-0.29144	p15603	CCR4-NOT	4	-0.28226		GO:0033147; P:negative regulation of intracellular estrogen recep[illegible]
CNN3 seq:	0.738009	-0.20577	-0.8178	-1.13177	-0.82341	-0.33435	0.264652	-0.3353	p18822	Calponin-3	4	-0.33072		
CNKSR1 pS	0.128395	-0.5493	-0.16282	-0.04537	-0.38632	-0.68608	-0.51126	-0.28222	p04414	Connector	4	-0.31187		IL3-mediate GO:0007266; P:Rho protein signal transduction; GO:0007169; P[illegible]
CNBP pS47	-1.04912	-1.00672	0.037621	-0.8067	-0.46957	-0.53843	-0.51075	-0.60262	p16102	Cellular nuc	4	-0.61829		Akt-mTOR-p70S6K-PI3K; PI3Kinh2 (PI-103); PI3Kinh1 (GDC-0941); p70S6Kinh2 (DG2); p[illegible]
CLTB seq: 1	0.164712	0.288775	-0.13164	-0.01526	-0.40354	-0.63259	-0.95347	-0.71109	p18630	Clathrin ligh	4	-0.29926		Arf1 pathway;
CLSPN seq:	0.694141	0.560014	-0.80927	0.174883	-0.43764	0.120229	-0.81967	-0.21623	p16413	Claspin	4	-0.09169		ATR signalir GO:0032147; P:activation of protein kinase activity;
CLSPN seq:	-1.11862	-0.88161	-1.2034	-0.18336	-0.56408	-0.56311	-0.9194	-0.49092	p04996	Claspin	4	-0.74056		ATR signalir GO:0032147; P:activation of protein kinase activity;
CLMN seq:	0.058135	-0.54437	-1.42698	-0.31383	-1.32445	-2.05263	-3.34603	-1.41598	p03914	Calmin	4	-1.29577		
CLMN pS41	0.403292	-0.36134	0.249103	0.2757	0.078521	-1.0197	-0.70319	-8.29561	p04781	Calmin	4	-1.17165		
CLMN pS40	-8.50824	0.010273	0.636456	-0.83061	-0.76709	-0.51175	-0.41183	-0.45046	p02191	Calmin	4	-1.35416		
CLIP1 seq:	0.105675	-0.1121	0.498245	-0.06249	-0.51807	-0.34282	-0.20381	-0.47376	p14271	CAP-Gly do	4	-0.13864		Lissencephaly gene (LIS1) in neuronal migration and development; mTOR sign[illegible]
CLIP1 seq:	0.109861	0.313198	-0.53654	0.014871	0.0389	0.083991	-0.05105	0.041459	p19320	CAP-Gly do	4	0.001836		Lissencephaly gene (LIS1) in neuronal migration and development; mTOR sign[illegible]
CLASRP pS	-0.01974	-0.2034	0.110623	0.095338	-0.10868	-0.31413	-0.27053	-0.21726	p13068	CLK4-assoc	4	-0.11597		
CLASP2 seq	-0.42987	-0.94927	-2.49012	-0.17637	0.11384	-0.4799	-0.39445	-0.26137	p19488	CLIP-associ	4	-0.63344		
CLASP2 pS	0.247312	0.478223	-0.73091	-1.28365	-0.00406	-0.46739	0.181797	0.18544	p01200	CLIP-associ	4	-0.17415		ERKinh2 (ERK inhibitor II);
CLASP1 seq	-0.10594	-1.47468	0.095424	0.344436	0.11798	-0.18693	-0.00759	0.271335	p01144	CLIP-associ	4	-0.11825		
CLASP1 seq	0	0	0	0	-0.33807	-0.98862	-0.56291	-0.35935	p03628	CLIP-associ	4	-0.28112		
CLASP1 seq	1.193891	0	0	0	-0.10164	-0.85342	-0.52951	-0.39606	p03627	CLIP-associ	4	-0.08584		
CLASP1 pS	-0.05243	-1.12424	-0.17019	0.151607	0.188324	-0.04794	0.091807	0.302551	p01145	CLIP-associ	4	-0.08256		p70S6Kinh2 (DG2);
CLASP1 pS	0.323549	-1.27464	-0.16442	-0.17488	0.215905	0.112448	0.227389	0.232799	p06782	CLIP-associ	4	-0.06273		
CIC pS496	-0.16692	-0.16061	-0.12863	-0.20919	-0.4062	0.077846	0.002591	-0.47451	p12431	Protein cap	4	-0.1832		
CHTOP pT3	-0.09499	-0.16536	-0.09684	0.29314	-0.3582	-0.12376	-0.31317	-0.57219	p09496	Chromatin	4	-0.17892		
CHTF8 seq:	-0.06787	-1.06936	0.264921	-0.03899	-0.22146	-0.25929	0.172392	0.290794	p09250	Chromosor	4	-0.11611		
CHTF18 se	-0.28035	-1.04562	-0.0291	-0.16602	-0.09056	-0.45115	-0.45216	-0.30994	p16233	Chromosor	4	-0.35311		
CHTF18 pS	0.190187	-0.51067	0.097625	0.145896	-0.00266	-0.0178	-0.06641	-0.01846	p01607	Chromosor	4	-0.02279		
CHMP3 se	-1.83467	-3.20693	-0.99807	-0.74972	-0.29859	-0.68571	-0.54527	-0.7738	p00744	Charged mu	4	-1.13659		Internalization of ErbB1;
CHMP3 pM	-0.54665	-0.87611	0.147805	0.080198	0.011485	-0.36607	-0.46955	-0.38431	p00745	Charged mu	4	-0.3004		Internalization of ErbB1;
CHM seq: 2	-0.32482	0.314061	-0.3766	-1.46207	0.164235	0.017313	0.111733	0.166495	p16559	Rab protein	4	-0.17371		
CHEK1 pS2	-0.59986	-0.33509	-0.41084	0.003846	0.136543	0.168839	-0.3198	0.173534	p00680	Serine/thre	4	-0.14785	Akt1;	Circadian rhythm pathway; ATR signaling pathway; Fanconi anemia pathway; p[illegible]
CHD3 pS32	-0.29848	-0.53135	-0.12566	-0.60738	0	0	0	0	p15951	Chromodor	4	-0.19536		Signaling events mediated by HDAC Class I;
CHAMP1 p	0.245811	0.130446	0.316862	0.210394	-0.65872	-0.53593	-0.35829	-0.5286	p03063	Chromosor	4	-0.14725		MEKinh2 (U0126);
CHAMP1 p	-0.34862	0.448382	-0.48541	-0.21789	-1.62561	-0.53744	-1.5326	-1.71711	p04515	Chromosor	4	-0.75204		
CHAF1B se	-0.04697	-0.50943	-0.33928	-0.19606	0.188511	0.179566	-0.05871	0.220399	p15170	Chromatin	4	-0.07025		
CGNL1 seq	-0.17944	-1.8084	0.055033	0.014932	0.296042	-0.37441	-0.65689	-0.22367	p03622	Cingulin-lik	4	-0.3596		
CGNL1 seq	-0.5108	-2.30126	-0.51983	-0.34856	-0.00807	-0.75178	-1.05498	-0.30306	p06569	Cingulin-lik	4	-0.72479		
CGN seq: 1	-0.58398	-0.92217	-0.2166	0.005409	-0.01035	-0.61075	-0.33088	-0.27461	p05565	Cingulin	4	-0.36799		GO:0007179; P:transforming growth factor beta receptor signalir[illegible]
CGN pS131	0.028609	-1.91074	-0.75883	0.963396	-0.02974	-0.86616	-0.09856	-0.15815	p05566	Cingulin	4	-0.35377		GO:0007179; P:transforming growth factor beta receptor signalir[illegible]
CFL2 seq: 1	-0.50194	0.06291	-0.63801	-0.50856	0.726466	0.322535	0.339561	0.546329	p01542	Cofilin-2	4	0.043661		Caspase cascade in apoptosis;
CFL1 seq: 8	-0.42247	0.019244	-0.66721	-0.80795	1.089958	0.705616	0.180698	0.212694	p00447	Cofilin-1	4	0.038822		RhoA signal GO:0007266; P:Rho protein signal transduction; GO:0038096; P[illegible]
CFL1 seq: 7	-0.83188	0.607135	-0.57147	-0.78087	0.726728	0.837883	0.050167	0.050522	p10022	Cofilin-1	4	0.011027		RhoA signal GO:0007266; P:Rho protein signal transduction; GO:0038096; P[illegible]
CEP170P1	-3.05766	-0.55567	-1.51955	-0.41699	-0.26851	-0.41898	-0.63181	-0.64015	p03762	Cep170-like	4	-0.93867		
CEP170B se	-0.51643	-1.05669	-0.86739	-0.63669	-1.73887	-1.96673	-2.35863	-1.92097	p04197	Centrosom	4	-1.3828		
CEP170B p	-0.24612	-0.09077	0.007826	-0.17339	-0.34416	-0.23452	-0.45676	-0.56556	p12778	Centrosom	4	-0.26293		
CEP170B p	0.094355	-0.64951	0.06626	0.133862	-0.11428	0.126761	-0.26872	0.043122	p02423	Centrosom	4	-0.07102		
CEP170B p	0.898906	-0.10799	0.460888	-0.17674	-0.71165	-1.03752	-0.89518	-1.05569	p03363	Centrosom	4	-0.32812		
CEP170B p	-0.34524	-1.84659	-1.16429	-0.83515	-1.50648	-1.6683	-1.90171	-1.64279	p20578	Centrosom	4	-1.36382		
CEP170 se	0.44354	-11.0419	-1.29441	-0.29179	-0.05086	-0.90315	-0.04482	-0.54067	p10852	Centrosom	4	-1.71551		
CEP170 se	-0.06761	-1.27876	-0.17068	0.185243	-0.48018	-0.85098	-1.47145	-0.96606	p11697	Centrosom	4	-0.63756		
CEP170 pS	0.029266	0.224273	-0.64946	0.343123	-0.0908	-0.74897	-0.17806	-0.71468	p13776	Centrosom	4	-0.22316		
CEP170 pS	-0.46996	-0.60021	0.017028	0.289071	0.067887	0.219074	0.197343	-0.38014	p10376	Centrosom	4	-0.08249		
CEP170 pS	-0.07372	-0.1117	-0.18383	-0.08908	-0.49957	-0.45348	-0.38472	-0.70658	p11695	Centrosom	4	-0.31284		
CEP131 se	0.072133	-0.3322	0.121963	-0.00852	-0.79985	0.284906	0.444627	-0.02186	p05667	Centrosom	4	-0.02985		
CENPV seq:	0	0	0	0	-0.95891	0.355407	0.356797	-0.9202	p19820	Centromere	4	-0.14586		
CENPT seq:	-0.30433	-0.57773	-0.0738	-0.02539	-0.44116	-0.12856	-0.25729	-0.85322	p07631	Centromere	4	-0.33269		
CENPC pT1	0.154927	-0.1921	0.080106	-0.03628	-0.37132	-0.20898	-0.23213	-0.62116	p14089	Centromere	4	-0.17837		
CDV3 seq:	-0.53425	-0.63768	-0.82901	-0.86721	0.018101	0.012068	0.488892	-0.80886	p12482	Protein CDV	4	-0.39474		
CDV3 pS8	0.207339	-0.90964	-0.20615	0.218613	-0.04213	0.103645	-0.04243	0.120276	p05287	Protein CDV	4	-0.06881		
CDT1 pS31	-1.02223	-1.08721	-1.34855	-1.4004	-0.33179	-0.32566	-0.26263	-0.41218	p02327	DNA replica	4	-0.77383	CDK2;	
CDR2 seq:	-0.53003	-0.27519	-0.34466	-0.35188	-0.06636	0.140499	-0.17802	-0.15328	p03717	Cerebellar d	4	-0.21986		
CDKN1A pS	-1.2306	-0.83008	-0.85774	-0.73659	-0.48523	-0.02633	-0.33851	-0.37308	p05769	Cyclin-depe	4	-0.60977	P38A; JNK1; ERK2; CDK[illegible]	Signaling ev GO:0045860; P:positive regulation of protein kinase activity; GO:[illegible]
CDKL5 seq:	-0.3302	-0.14612	0.058484	-0.6805	-5.02208	0.402736	1.766822	-0.38762	p04788	Cyclin-depe	4	-0.54231		
CDK18 pT1	0.085667	-0.47544	0.116269	0.065236	-0.27644	-0.55451	-0.01256	-0.49884	p11802	Cyclin-depe	4	-0.19383		
CDK17 seq	-0.48825	0.379527	-7.82429	0.196154	-0.91342	-2.33433	-0.07633	-1.20298	p15183	Cyclin-depe	4	-1.53299		
CDK16 pS1	0.515771	0.356816	0.56002	0.408586	1.098062	1.242024	2.276523	-6.50089	p08291	Cyclin-depe	4	-0.00539	PKACA;	
CDK13 pT8	-0.2167	-0.6275	-0.20022	-0.30247	-0.45949	-0.15804	-0.46264	-0.50206	p10288	Cyclin-depe	4	-0.36614		
CDK13 pT8	-0.24404	-0.72014	-0.06957	-0.05022	-0.1414	-0.1107	0.269838	-0.91423	p10289	Cyclin-depe	4	-0.24756		
CDK13 pS5	-0.41893	-0.55835	-0.32418	-0.33346	-0.11157	-0.20293	-0.00875	-0.27355	p14354	Cyclin-depe	4	-0.27896		
CDK12 pT8	0.16065	-0.16837	0.262643	-0.17607	-0.10365	-0.21665	0.29922	-0.14613	p10297	Cyclin-depe	4	-0.01105		
CDK1 pT14	0.085667	-0.47544	0.116269	0.065236	-0.27644	-0.55451	-0.01256	-0.49884	p14279	Cyclin-depe	4	-0.19383	Myt1;	Retinoic aci GO:0000186; P:activation of MAPKK activity; GO:0051403; P:str[illegible]
CDCA5 pS7	0.220485	-0.17275	0.431924	0.001385	-0.27971	-0.49716	-0.18837	-0.73099	p13569	Sororin	4	-0.1519	CDK1;	
CDCA5 pS7	0.089052	-0.13366	0.367638	-0.02736	-0.13015	-0.3331	-0.2172	-0.66628	p13568	Sororin	4	-0.13138	CDK1;	
CDCA3 seq	0.117599	0.019315	-0.11796	-0.13059	-0.15785	-0.03092	-0.23308	-0.22984	p14887	Cell divisior	4	-0.09542		
CDCA3 seq	0.109939	-0.25824	0.263662	0.176391	-1.01839	-0.53593	-0.5834	-1.14731	p04536	Cell divisior	4	-0.37416		
CDCA3 pS2	-0.02244	-0.52324	-0.0587	0.124695	-0.64349	-1.21566	-0.6051	-0.95115	p04535	Cell divisior	4	-0.48689		
CDC42EP4	0.329101	-0.40011	0.372565	0.298241	-0.84587	-1.57102	-0.71745	-0.77819	p04142	Cdc42 effe	4	-0.41409		GO:0007266; P:Rho protein signal transduction;
CDC42EP4	-0.88523	-1.27559	-0.26926	-0.24738	-0.27367	-1.68307	-0.06012	-0.90444	p09765	Cdc42 effe	4	-0.69984		GO:0007266; P:Rho protein signal transduction;
CDC42EP4	-0.68078	-1.39683	-0.80472	-0.63666	-0.19056	0.07364	0.010695	-0.04407	p09761	Cdc42 effe	4	-0.45866		GO:0007266; P:Rho protein signal transduction;
CDC42EP4	-0.12047	-0.69276	0.096526	-0.14454	-0.31897	-1.11942	-0.43818	-0.61315	p09762	Cdc42 effe	4	-0.41887		GO:0007266; P:Rho protein signal transduction;
CDC42EP4	0.13957	-0.29308	0.251308	0.200007	0.099206	-0.67755	0.06535	0.141646	p09764	Cdc42 effe	4	-0.00919		GO:0007266; P:Rho protein signal transduction;
CDC42EP2	1.348001	-5.13732	2.223865	1.083085	-1.13918	-1.56019	-0.63416	-0.62647	p09773	Cdc42 effe	4	-0.5553		Up with IGF - down with PI3Kinh2 (PI-103); Up with EGF - down with PI3Kinh2 (PI-103); [illegible]
CDC42EP2	0.357756	-0.30573	0.718677	0.529894	-1.05149	-1.19725	-0.6459	-0.5626	p09774	Cdc42 effe	4	-0.26958		Up with IGF - down with PI3Kinh2 (PI-103); Up with EGF - down with PI3Kinh2 (PI-103); [illegible]
CDC25B se	0.283283	-0.23807	0.339807	-0.06349	-0.34012	-0.68281	-0.24945	-0.42786	p04410	M-phase in	4	-0.17234	MELK; MAPKAPK2;	p38 signalir GO:0045860; P:positive regulation of protein kinase activity;
CD81 seq:	0.347835	0.359403	-0.3052	0.092207	0.167862	-1.93385	-0.23282	-3.01001	p08667	CD81 antig	4	-0.56432		Alpha4 beta GO:0046488; P:phosphatidylinositol metabolic process; GO:000[illegible]
CD81 seq:	0.257055	-0.06811	0.19977	0.110788	-0.7084	-1.39207	-1.28224	-2.86799	p08665	CD81 antig	4	-0.7189		Alpha4 beta GO:0046488; P:phosphatidylinositol metabolic process; GO:000[illegible]
CD3EAP se	0.05954	-0.81814	0.262032	0.757587	-0.66969	-0.81111	-0.58837	-0.89523	p01954	DNA-direct	4	-0.33792	CDK1;	GO:0007169; P:transmembrane receptor protein tyrosine kinase[illegible]
CD3EAP se	-0.31642	-1.04321	0.368041	0.165816	-0.26465	-0.32189	-0.1209	-0.53524	p06321	DNA-direct	4	-0.25856		GO:0007169; P:transmembrane receptor protein tyrosine kinase[illegible]
CD3EAP se	0.155896	-0.19345	0.767184	0.186467	-0.25926	-0.50773	-0.52709	-0.42011	p14063	DNA-direct	4	-0.09976		AKTinh1 (Akt Inhibitor V GO:0007169; P:transmembrane receptor protein tyrosine kinase[illegible]
CD2BP2 se	-0.16021	-0.50447	-0.35753	-0.14201	-0.0807	-0.1493	-0.13232	-0.234	p15510	CD2 antiger	4	-0.22007		
CD2BP2 se	-0.28553	-0.33822	-0.20146	-0.07184	-0.18506	-0.18671	-0.22647	-0.28687	p13176	CD2 antiger	4	-0.22277		
CD2AP pS2	0.828014	-0.94527	0.621115	0.488453	0.001451	-1.23981	-0.21281	0.306065	p15898	CD2-associ	4	-0.0191		VEGFR1 spe GO:0007165; P:signal transduction;
CD2AP pS2	0.918804	-0.49013	0.30226	0.267455	0.002912	-1.36962	-0.00766	0.285273	p15897	CD2-associ	4	-0.01134		VEGFR1 spe GO:0007165; P:signal transduction;
CCT8 pS31	-0.45845	-0.11907	0.340668	-0.10728	-1.24668	0.241562	0.273297	0.369897	p11160	T-complex	4	-0.08826		
CCT6A seq:	-0.03469	1.205418	-0.82238	-1.43955	1.094322	1.381846	-7.36731	1.420978	p20194	T-complex	4	-0.57017		
CCT3 seq: 7	0.314829	-0.81056	0.353598	0.897117	0.12783	-0.97885	-1.99286	-0.91562	p04892	T-complex	4	-0.37556		
CCT2 seq: 2	-1.01988	-0.3749	-1.16829	-0.96782	0.693786	-0.04636	0.126414	1.286601	p14037	T-complex	4	-0.18381		
CCNL1 pS3	0.164685	0.053278	-0.07496	-0.2583	-0.5959	0.092965	-0.35007	-0.52945	p16978	Cyclin-L1	4	-0.18722		
CCNK pS34	-0.29848	-0.17518	-0.2648	0.020229	0.167093	-0.03504	0.02313	0.001094	p07538	Cyclin-K	4	-0.07024		Direct p53 effectors;
CCNK pS34	-0.16209	-0.08761	0.081114	0.106655	0.049248	0.184425	0.03285	-0.01536	p19143	Cyclin-K	4	0.023655		Direct p53 effectors;
CCNE2 seq:	-0.27638	-0.66698	-0.07233	-0.29845	-0.16522	-0.03599	-0.46916	-0.28777	p08035	G1/S-specif	4	-0.28403		E2F transcription factor network; p73 transcription factor network;
CCDC92 se	-0.72424	-0.44457	-0.63511	-1.23143	-0.10027	-0.55954	-0.10507	-0.10234	p05072	Coiled-coil	4	-0.48782		
CCDC9 seq	-0.42149	-0.07552	-0.31731	0.035563	-0.45193	-0.31764	-0.27299	-0.70334	p17773	Coiled-coil	4	-0.31558		
CCDC9 pS8	-0.55233	-0.69899	-0.37006	-0.27329	-0.05592	-0.21307	-0.37232	-0.15886	p04602	Coiled-coil	4	-0.33686		
CCDC9 pS8	-0.44007	-0.59995	-0.3588	-0.22261	-0.08631	-0.20224	-0.33389	-0.14518	p04603	Coiled-coil	4	-0.29863		
CCDC88C s	0.004443	-0.1966	0.027398	-0.09859	-0.37739	-0.62845	-0.27881	-0.33155	p09000	Protein Dap	4	-0.23494		GO:0016055; P:Wnt signaling pathway;
CCDC88C s	-0.0301	-0.25382	0.132068	-0.07977	-0.2781	-0.52337	-0.33525	-0.26265	p08999	Protein Dap	4	-0.20387		GO:0016055; P:Wnt signaling pathway;
CCDC88C p	0.034685	-1.33518	-0.2354	-0.1533	-0.82299	-1.75071	-0.4534	-0.71607	p07427	Protein Dap	4	-0.67905		PKCinh2 (Bisindolylmal GO:0016055; P:Wnt signaling pathway;
CCDC88C p	-0.49328	-0.7935	-0.15712	-0.20619	-0.24776	-0.79467	-0.2877	-0.46706	p20746	Protein Dap	4	-0.43091		Up with EGF - down wit GO:0016055; P:Wnt signaling pathway;
CCDC88C p	-0.57794	-0.43109	-0.73144	-0.90794	-0.23051	-1.34136	-1.44642	-0.22257	p05990	Protein Dap	4	-0.73616		GO:0016055; P:Wnt signaling pathway;
CCDC86 se	-0.09631	-0.0344	0.025625	-0.17469	-0.24992	-0.58823	-0.55013	-0.29761	p20117	Coiled-coil	4	-0.24571		
CCDC86 se	-0.07072	-0.14907	-0.12222	-0.30533	-0.49995	-0.57252	-0.65807	-0.69348	p07622	Coiled-coil	4	-0.38392		
CCDC86 se	0.158633	0.021702	-0.03619	-0.22874	-0.57675	-0.57293	-0.65457	-0.72653	p07623	Coiled-coil	4	-0.32692		
CCDC86 pS	-0.28644	-0.17233	-0.10306	-0.08455	-0.53698	-0.52403	-0.82992	-0.78247	p20635	Coiled-coil	4	-0.41497		
CCDC86 pS	-0.12645	-0.02322	0.024746	0.000474	-0.58747	-0.48791	-0.79017	-0.82951	p20636	Coiled-coil	4	-0.35244		
CCDC85C s	-0.10546	-13.6077	-0.36411	-0.29149	0	0	0	0	p21113	Coiled-coil	4	-1.79609		
CCDC85C p	-0.10362	-0.51185	-0.11898	-0.03038	-0.27995	-0.46999	-0.40862	-0.27338	p05306	Coiled-coil	4	-0.2746		
CCDC81 se	-0.45853	-0.30629	-0.72349	-0.39588	-0.12001	-0.09363	-0.79849	-0.12145	p16599	Coiled-coil	4	-0.37722		
CCDC71L p	-4.46255	-1.03671	-1.90673	-1.66814	-0.55029	-0.25135	-0.25792	-0.18991	p20996	Coiled-coil	4	-1.29045		
CCDC64B p	-0.28194	-0.56215	-0.18589	-0.21282	-0.73799	-0.39216	-0.1873	-0.35294	p07514	Bicaudal D-	4	-0.36415		
CCDC6 seq	-0.20855	-0.83007	0.306247	-0.02798	0.172518	-0.54	-0.28194	-0.09883	p01892	Coiled-coil	4	-0.18857		
CCDC43 se	-1.814	-1.13155	-0.43436	-0.21399	-0.12714	-1.14745	-0.72121	-0.42483	p21097	Coiled-coil	4	-0.75181		AKTinh2 (MK-2206); AKTinh1 (Akt Inhibitor VIII);
CCDC43 pT	0.138028	0.262636	0.201302	0.281803	-0.321	-0.30821	-0.20978	-0.28506	p21098	Coiled-coil	4	-0.03003		AKTinh2 (MK-2206); AKTinh1 (Akt Inhibitor VIII);
CCDC39 pT	-0.17088	-0.72737	-0.14516	-0.43913	-0.63254	-1.02036	-0.443	-0.27612	p19224	Coiled-coil	4	-0.48182		
CCDC125 s	-0.56992	-0.7356	-0.25174	-0.27533	-0.82147	-1.06239	-0.70978	-0.47638	p05574	Coiled-coil	4	-0.61283		
CCDC120 s	-1.60447	-0.09933	-1.67478	-1.14918	-0.64837	-9.10963	0.591024	-0.44567	p04764	Coiled-coil	4	-1.76755		
CCDC120 s	-0.24651	-1.67783	-0.36091	-0.13212	-0.68553	-0.93651	-0.73191	-1.00895	p06999	Coiled-coil	4	-0.72253		
CCDC120 p	-0.04009	-0.7294	0.210942	-0.09321	-0.1804	-0.39653	-0.34411	-0.43615	p07674	Coiled-coil	4	-0.25101		
CCDC120 p	0.074825	-1.25681	0.179074	0.418718	0.483211	-0.18139	0.03115	0.221848	p03307	Coiled-coil	4	-0.00367		
CCDC120 p	-0.13914	-1.34477	-0.27263	-0.0856	-0.41889	-0.79837	-0.43221	-0.35729	p07000	Coiled-coil	4	-0.48111		
CCDC102A	-0.20534	-0.21049	-0.21442	-0.17233	-1.0901	-0.21342	-0.65671	-0.60052	p15247	Coiled-coil	4	-0.42042		
CBY1 seq: 1	-0.75855	-1.88183	-0.81951	-0.86445	-0.92051	-1.26864	-0.76518	-0.5321	p06258	Protein chib	4	-0.97635	Akt2; Akt1;	Regulation GO:0030178; P:negative regulation of Wnt signaling pathway; GC[illegible]
CBY1 seq: 1	-0.61769	-0.13154	-0.93856	-1.35293	-0.63568	-1.17143	-0.55845	-0.56667	p12607	Protein chib	4	-0.74662	Akt2; Akt1;	Regulation GO:0030178; P:negative regulation of Wnt signaling pathway; GC[illegible]
CBX8 seq: 1	0.26922	-0.04526	0.523013	0.255313	-0.13133	-0.49403	-0.24385	-0.57115	p13843	Chromobox	4	-0.05476		
CBX5 pS92	-0.11529	-0.60472	0.010764	-0.38614	-0.65213	-0.32581	-0.13151	-0.26135	p07209	Chromobox	4	-0.30827	AurB;	Aurora B signaling; E2F transcription factor network;
CBFA2T2 p	0.176703	0.278201	0.624992	0.416773	-2.64607	-1.33007	-1.8141	-1.75994	p08564	Protein CBF	4	-0.75669		
CAT seq: 43	-0.59672	-0.15206	-0.5536	-0.50521	-1.47809	-7.00199	-1.58473	0.677775	p16547	Catalase	4	-1.39933		FoxO family GO:0071363; P:cellular response to growth factor stimulus; GO:[illegible]
CAST seq: 2	-0.15185	0.538423	-0.06491	-0.01629	-0.24608	-0.09303	-0.18084	-0.17589	p17732	Calpastatin	4	-0.04881		AKTinh1 (Akt Inhibitor VIII);
CAST seq: 2	-0.66968	-0.28884	-0.37027	-0.3065	-0.27124	-0.29286	-0.46419	-0.30143	p17734	Calpastatin	4	-0.37063		AKTinh1 (Akt Inhibitor VIII);
CAST seq: 2	-0.38152	-0.112	-0.28015	-0.25142	-0.33604	-0.28105	-0.50237	-0.47255	p17735	Calpastatin	4	-0.32714		AKTinh1 (Akt Inhibitor VIII);
CAST seq: 2	-0.39802	0.486508	-0.69393	-0.39834	-0.28434	-0.36144	-0.29489	-0.15646	p13293	Calpastatin	4	-0.26261		AKTinh1 (Akt Inhibitor VIII);
CAST seq: 1	0.280625	-0.80786	-0.03302	0.248166	0.118489	-0.00601	-0.08412	-0.08521	p04944	Calpastatin	4	-0.04612		
CAST pS24	-0.40996	0.702207	-0.00926	-0.08382	-0.27995	-0.11742	-0.22792	-0.13279	p17733	Calpastatin	4	-0.06986		AKTinh1 (Akt Inhibitor VIII);
CAST pS24	-0.12921	0.540095	-0.06902	-0.01462	-0.24608	-0.09303	-0.18084	-0.17589	p13294	Calpastatin	4	-0.04607		AKTinh1 (Akt Inhibitor VIII);
CAST pS24	-0.08023	0.679442	-0.09922	-0.04178	-0.29016	-0.11863	-0.22708	-0.12824	p13295	Calpastatin	4	-0.03824		AKTinh1 (Akt Inhibitor VIII);
CASS4 seq:	0.172886	-0.06138	-0.04081	0.163513	0.121596	-0.50251	0.061391	0.051563	p02554	Cas scaffold	4	-0.00422		
CASKIN2 se	-0.72301	-0.03105	-0.68715	-0.32714	-0.00424	0.13345	-1.00663	0.119385	p15176	Caskin-2	4	-0.3158		
CASKIN2 se	0.092809	0.290856	-0.3964	-0.48204	-0.2386	-0.24701	-0.47791	-0.2228	p07076	Caskin-2	4	-0.21014		
CASKIN2 se	-0.69059	-0.94945	-0.68207	-0.34093	0.09222	-0.142	-0.08278	-0.20447	p02135	Caskin-2	4	-0.37501		
CASKIN2 se	-0.54658	-0.43124	-0.89692	-0.83712	-1.12948	-0.7746	-0.93315	-0.95784	p05571	Caskin-2	4	-0.81337		
CASKIN2 se	-0.36649	-0.38331	-0.78138	-0.70793	-0.11635	0.191617	-0.17979	-0.46818	p04110	Caskin-2	4	-0.35148		
CASKIN2 se	-0.56293	-0.76529	-0.57992	-0.73097	0.004672	0.032649	0.02675	0.348339	p08992	Caskin-2	4	-0.27834		
CASKIN2 se	-0.31943	-1.0372	-0.84421	-1.57367	0	0	0	0	p08994	Caskin-2	4	-0.47181		
CASKIN2 se	-0.70843	-0.80195	-0.59984	-0.74516	0.383555	0.565286	0.259016	-1.05062	p08993	Caskin-2	4	-0.33727		
CASKIN2 pS	-0.45998	-0.12775	-0.6796	-0.46701	-0.10712	-0.81636	-0.18457	-0.25641	p04205	Caskin-2	4	-0.38735		
CASKIN2 pS	-0.52339	-0.31833	-0.59158	-0.4926	0.064393	-0.17307	0.020013	0.077686	p01465	Caskin-2	4	-0.24211		
CASKIN1 pS	0.569926	-2.14831	-0.44739	0.226757	0	0	0	0	p07590	Caskin-1	4	-0.22488		GO:0007165; P:signal transduction;
CASC3 pS4	-1.42525	-0.45378	-0.4566	-0.61843	-2.00518	-1.94791	-2.63768	-1.0025	p16048	Protein CAS	4	-1.31842		
CARD11 pS	0	0	0	0	-1.48952	-1.35142	-1.10687	-1.06498	p14139	Caspase rec	4	-0.6266		TCR signalir GO:0050852; P:T cell receptor signaling pathway; GO:0043123; P[illegible]
CAPRIN1 se	-0.46245	-0.54643	-0.72819	-0.69814	0.143303	-0.12985	0.033751	-0.18489	p18783	Caprin-1	4	-0.32161		
CAP1 seq: 3	-1.0355	-0.04283	-1.28567	-1.7278	0.524318	0.232051	-0.02298	-0.23535	p13895	Adenylyl cy	4	-0.44922		Arf6 signali GO:0007165; P:signal transduction;
CAP1 seq: 1	-1.31855	-1.7176	-1.08887	-0.04259	1.122262	0.152322	-0.9072	0.458571	p16979	Adenylyl cy	4	-0.41771		Arf6 signali GO:0007165; P:signal transduction;
CAMSAP3 s	-0.31154	1.264826	-0.3864	-0.16136	-0.63496	-0.05894	-0.43158	-0.61819	p13225	Calmodulin	4	-0.16727		Stabilization and expansion of the E-cadherin adherens junction;
CAMSAP3 s	-0.69568	-0.45472	-0.00952	-0.19901	-0.89897	-0.74337	-0.90263	-0.63052	p06650	Calmodulin	4	-0.5668		Stabilization and expansion of the E-cadherin adherens junction;
CAMSAP3 s	-0.05134	-0.51057	-0.0445	-0.29238	-0.12808	-0.57457	-0.04819	-0.16609	p03254	Calmodulin	4	-0.22696		Stabilization and expansion of the E-cadherin adherens junction;
CAMSAP3 s	0.166616	-1.31326	0.121532	-0.09441	-0.64875	-2.3933	-1.09673	-0.03138	p14697	Calmodulin	4	-0.66121		Stabilization and expansion of the E-cadherin adherens junction;
CAMSAP3 p	0.105757	-0.05873	0.185883	0.07096	-0.33597	0.024264	-0.12892	-0.18657	p19897	Calmodulin	4	-0.04042		Stabilization and expansion of the E-cadherin adherens junction;
CAMSAP2 p	-0.01636	-0.23525	-0.18777	-0.36139	-0.50261	-0.6208	-0.51549	-0.78692	p05968	Calmodulin	4	-0.40332		
CAMSAP1 p	0.359516	-2.0018	-0.24766	0.188591	0.145056	-0.58033	-0.69985	0.181934	p06860	Calmodulin	4	-0.33182		
CAMSAP1 p	-0.17251	-0.20823	0.536403	0.395827	-0.86776	-0.54575	-0.47076	-0.68455	p15128	Calmodulin	4	-0.25217		
CAMK2G p	-0.37496	-0.76268	-0.1626	-1.34896	-0.96845	-0.36976	-2.89534	-0.92267	p16310	Calcium/cal	4	-0.97568		N-cadherin GO:0060333; P:interferon-gamma-mediated signaling pathway; G[illegible]
CAMK2D se	-0.16653	0.00309	-0.0649	-0.96192	-1.01666	-4.16181	-1.09876	-0.54597	p05154	Calcium/cal	4	-1.00168		IFN-gamma GO:0060333; P:interferon-gamma-mediated signaling pathway; G[illegible]
CAMK2D se	0.128034	-0.05285	0.081729	-0.69459	-1.33544	-2.2328	-0.36668	-2.45864	p05155	Calcium/cal	4	-0.8664		IFN-gamma GO:0060333; P:interferon-gamma-mediated signaling pathway; G[illegible]
CAMK2D se	-0.67357	-0.37707	-0.18181	-1.19449	-0.19615	-4.49787	0.308013	-1.45203	p09632	Calcium/cal	4	-1.03312		IFN-gamma GO:0060333; P:interferon-gamma-mediated signaling pathway; G[illegible]
CAMK2D p	-0.34061	-0.41947	-0.31732	-0.89564	-0.89817	-0.7581	-0.80538	-1.25649	p09633	Calcium/cal	4	-0.7114		IFN-gamma GO:0060333; P:interferon-gamma-mediated signaling pathway; G[illegible]
CALR seq: 9	-0.47381	0.56249	-0.74336	-0.85203	0.767619	-0.53103	0.106765	-0.46754	p14841	Calreticulin	4	-0.20386		GO:0048387; P:negative regulation of retinoic acid receptor signa[illegible]
CALR seq: 5	-0.57073	0.046089	-0.82892	-0.96839	0.113646	-0.16882	-0.62476	-0.39077	p16366	Calreticulin	4	-0.42408		GO:0048387; P:negative regulation of retinoic acid receptor signa[illegible]
CALR seq: 5	-0.47234	0.029168	-0.81982	-0.88457	0.377423	-0.00126	-0.07336	-0.05493	p16365	Calreticulin	4	-0.23746		GO:0048387; P:negative regulation of retinoic acid receptor signa[illegible]

CALML5 se	-0.55259	-0.68987	-0.74905	-0.86898	-0.01329	-4.01898	1.318693	0.667258	p09417	Calmodulin	4	-0.61335			GO:0007165; P:signal transduction;
CALM1 seq	-0.37345	-1.28334	-0.70956	-0.52482	-1.52763	-1.60788	-2.16927	-1.71234	p01570	Calmodulin	4	-1.23854		Regulation (	GO:0022400; P:regulation of rhodopsin mediated signaling pathway; GO:0016056; P:rhodopsin mediated signaling pathway; GO:00 [illegible]
CAD seq: 1	-0.02551	0.043185	-0.62084	-0.20654	-0.63183	-0.59879	-1.23439	-0.81262	p14206	CAD protei	4	-0.51092	PKACA; p70S6K;	Validated ta	GO:0071364; P:cellular response to epidermal growth factor stimulus;
CAD pS185	-0.54034	-0.5311	-1.089	-0.83202	-0.79466	-0.908	-1.37728	-1.42352	p14205	CAD protei	4	-0.93699	PKACA; p70S6K;	Validated ta	GO:0071364; P:cellular response to epidermal growth factor stimulus;
CACNG6 se	-1.11385	-1.21959	-1.43534	-2.12767	2.464669	2.023875	-5.68319	-0.18369	p16330	Voltage-dep	4	-0.90935			
CABLES1 se	-0.21543	-0.91621	-0.38558	-0.6555	-1.27435	-1.30529	-0.49937	-2.63926	p06993	CDK5 and A	4	-0.98637		Posttranslational regulation of adherens junction stability and dissassembly; Validated transcriptional targets of TAp63 isoforms;	
CABLES1 se	0.898269	0.185504	0.517427	0.35871	-1.72687	-1.20627	-1.23854	-0.73092	p03862	CDK5 and A	4	-0.36784		Posttranslational regulation of adherens junction stability and dissassembly; Validated transcriptional targets of TAp63 isoforms;	
CABLES1 pS	-0.33177	-0.35141	-0.13174	-0.30648	-2.25924	0.180673	-2.20658	-0.0256	p16138	CDK5 and A	4	-0.67902		Posttranslational regulation of adherens junction stability and dissassembly; Validated transcriptional targets of TAp63 isoforms;	
CA2 seq: 28	-0.40366	0.753425	0.100034	-0.62502	-0.82786	0.466049	-0.68836	-2.52223	p07859	Carbonic ar	4	-0.46845			
CA2 seq: 15	-0.50484	0.908178	-0.77415	-4.37277	0	0	0	0	p00641	Carbonic ar	4	-0.59295			
C9orf40 pS	-0.4866	0.262108	-1.33179	0.213127	-0.44052	-0.78975	-0.35639	-0.39115	p13115	Uncharacte	4	-0.41512	CDK2;		
C7orf43 se	-0.42301	-0.37186	-0.16533	-0.19085	-0.29338	-0.65931	-0.46859	-0.5277	p04198	Uncharacte	4	-0.3875			
C7orf43 pS	-0.33539	-0.29833	-0.16406	-0.18975	-0.38327	-0.70339	-0.43447	-0.49289	p04199	Uncharacte	4	-0.37519			
C6orf132 p	-0.15968	-0.20902	-0.00487	-0.08381	-0.84986	-0.5262	-1.08604	-1.05613	p16163	Uncharacte	4	-0.49695			
C6orf132 p	-0.89076	-0.86992	-0.44427	-0.29445	-0.30914	0.047784	-0.56621	-0.2838	p05223	Uncharacte	4	-0.45134			
C6orf106 p	0.679723	-3.09601	0.494665	0.464673	-1.26049	-0.95983	-1.01961	-1.39694	p12442	Uncharacte	4	-0.76173	CDK2;		
C2orf44 pS	-2.92926	0.446227	-0.6872	-0.29302	-0.14008	-0.04833	-0.02313	-0.17425	p11602	WD repeat	4	-0.48113			
C2orf44 pS	0	0	0	0	-1.10967	0.342501	0.989959	-0.55116	p11603	WD repeat	4	-0.04105			
C2CD5 seq:	-0.19424	-0.08293	-0.00627	-0.22932	0.09779	0.135479	-0.1028	-0.09033	p04209	C2 domain-	4	-0.05908			
C2CD5 pS8	-0.16088	0.532829	-0.35355	-0.0205	-1.06039	-1.21206	-0.20512	-0.97386	p20066	C2 domain-	4	-0.43169			
C2CD5 pS8	-0.05256	0.303144	-0.11792	0.017093	-0.65856	-0.50078	-0.52299	-0.62925	p20065	C2 domain-	4	-0.27023			
C2CD3 seq:	-2.22541	0.678657	0.226952	0.261719	0.053753	-0.02455	-0.29567	-0.03826	p03909	C2 domain-	4	-0.17035			GO:0008589; P:regulation of smoothened signaling pathway;
C2CD2L pS	0.353432	0.279647	0.32021	0.332909	-0.89998	-0.88913	-0.36931	-0.55077	p20257	C2 domain-	4	-0.17787			
C2CD2L pM	0.389081	0.008733	0.247216	0.346909	-0.79588	-1.82622	-0.39964	-0.877	p20258	C2 domain-	4	-0.36335			
C1orf226 s	-0.22356	-0.66123	0.114878	-0.04999	-0.40518	-0.62069	-0.92172	-0.62286	p02103	Uncharacte	4	-0.42379			
C1orf198 s	-0.29419	-0.81209	-0.27395	-0.54353	-0.02236	-0.36505	-0.1088	-0.19645	p03697	Uncharacte	4	-0.32705			
C1orf198 s	-0.26111	-0.56858	-0.21398	-0.35896	0.159158	-0.06681	0.129311	0.175359	p03699	Uncharacte	4	-0.1257			
C1orf106 s	-1.88994	-1.1851	-0.62228	0.166508	0.598545	0.619856	0.359129	0.574975	p02198	Uncharacte	4	-0.17229			
C17orf59 s	0.385151	-0.60387	-0.05917	0.071836	-0.36149	-0.90341	-0.38735	-0.57152	p07546	Uncharacte	4	-0.30373			
C15orf52 s	-0.03643	-0.27535	-0.15186	-0.07936	-0.36816	-0.47982	-0.44531	-0.54572	p00683	Uncharacte	4	-0.29775			
C12orf45 s	-0.10734	-0.29532	-0.28042	-0.34517	-0.59668	-0.26794	-0.72334	-0.83834	p19534	Uncharacte	4	-0.43182			
C12orf45 s	-0.15129	-0.34041	-0.10836	-0.02276	-0.40193	-0.22096	-0.68823	-0.75244	p19533	Uncharacte	4	-0.3358			
C12orf45 s	-0.88192	-0.71584	-1.204	-1.18995	-0.98099	-0.09786	-0.01452	-0.15406	p14321	Uncharacte	4	-0.65489			
C11orf86 s	-0.3476	-0.82335	-0.40336	-0.42421	-0.26914	-0.55663	-0.20371	-0.30854	p07629	Uncharacte	4	-0.41707			
C10orf12 s	0.081042	-0.11966	0.116933	-0.02825	-0.31794	-0.30833	-0.47494	-0.73747	p14107	Uncharacte	4	-0.22358			
C10orf12 s	0.393871	0.087549	0.294595	0.245197	-0.28634	-0.17521	-0.41111	-0.64955	p14108	Uncharacte	4	-0.06262			
BUD13 seq	-1.70049	-1.39881	-0.89199	-0.52222	0.125189	-0.01714	-0.92396	-0.01944	p00110	BUD13 hor	4	-0.66861			
BTF3 seq: 1	0.144463	-0.01315	0.129501	0.039372	-0.15176	-0.2891	-0.16881	-0.19284	p19972	Transcriptic	4	-0.06279			
BTF3 pS30	-0.40584	-0.82106	-0.22824	-0.36232	-0.30638	-0.29051	-0.36477	-0.56016	p19714	Transcriptic	4	-0.41741			
BTBD9 seq:	-0.60152	0.002715	-0.37827	-1.05594	0.145538	0.018361	-0.28698	-0.55016	p19920	BTB/POZ dc	4	-0.33828			
BRWD1 pS	-0.31007	-0.50597	-0.6613	-0.07154	-1.0955	-2.22744	0.239702	0.231041	p14151	Bromodom	4	-0.55014			
BRIP1 pS20	0.08166	-0.35235	0.14591	-0.18298	-0.67063	-1.51259	0.166499	-0.73907	p13896	Fanconi ane	4	-0.38294		Fanconi anemia pathway;	
BRF1 pS55	-0.15916	-0.22724	-0.36742	-0.51466	-0.28171	0.095221	0.410115	-0.24658	p15638	Transcriptic	4	-0.16143			
BRD2 pS29	-0.12729	-0.55682	-0.17847	-0.06905	-0.10722	0.040349	-0.06986	0.057984	p13459	Bromodom	4	-0.1263		Regulation of retinoblastoma protein;	
BRD1 pS48	-1.08301	-1.61195	-1.16961	-1.54329	-0.19621	-0.67757	-0.76346	-0.4352	p10687	Bromodom	4	-0.93504			
BRAF pT401	-0.19851	0.152024	-0.17288	-0.18741	-0.8322	-0.63323	-0.52323	-0.38093	p18382	Serine/thre	4	-0.34705	ERK2; ERK1;	Ras signalin	GO:0000186; P:activation of MAPKK activity; GO:0070374; P:pos [illegible]
BMP2K seq	-0.63757	-0.61084	-0.21158	-0.21775	-0.01808	0.199922	-0.12559	-0.13656	p06027	BMP-2-indu	4	-0.21976			
BMP2K pS1	0.249862	-0.21108	-0.10442	0.075962	-0.16166	-0.46587	-0.59938	-0.3249	p08180	BMP-2-indu	4	-0.19269	p70S6Kinh1 (PF-4708671);		
BLVRB seq:	-1.22895	-1.8677	-1.36295	-1.61016	0	0	0	0	p11030	Flavin reduc	4	-0.75872			
BIN1 seq: 2	0	0	0	0	-0.3495	-0.51605	-0.70217	-0.52681	p19776	Myc box-de	4	-0.26182		Arf6 trafficking events;	
BIN1 pS298	-0.17197	-0.27062	-0.48145	-0.79632	0.114755	0.066148	-0.00256	-0.06075	p15581	Myc box-de	4	-0.20034		Arf6 trafficking events;	
BICD2 seq:	0	0	0	0	-0.83597	-0.01655	-0.05711	-0.53445	p20918	Protein bica	4	-0.18051			
BICD2 pS58	-0.35512	-0.51352	-0.46528	-0.61078	-0.59222	-0.50001	-0.70028	-0.83706	p15327	Protein bica	4	-0.57178	p70S6Kinh1 (PF-4708671);		
BCLAF1 seq	-0.29113	-0.92654	-0.66927	0.166607	-2.42209	-0.03828	-1.86663	-1.57491	p13267	Bcl-2-assoc	4	-0.95278			
BCLAF1 pS4	0.214032	-1.18983	0.396055	-0.59695	-0.86393	-0.41483	-1.41239	-1.48956	p13266	Bcl-2-assoc	4	-0.66967			
BCLAF1 pS4	0.063652	-0.2718	-0.08826	-0.97482	0.484736	0.397509	0.188339	-0.21046	p03324	Bcl-2-assoc	4	-0.05139			
BCLAF1 pS2	0.346903	0.277191	-0.07386	0.230544	-0.28099	-0.14805	-1.19621	-0.20572	p00086	Bcl-2-assoc	4	-0.13127			
BCLAF1 pS2	0.610136	0.791937	-0.04356	-0.2665	-0.36487	-0.25098	-0.4359	-0.58544	p09008	Bcl-2-assoc	4	-0.06815			
BCL9L pS75	-0.44247	-0.43231	-0.10176	-0.14171	-0.08399	-1.10164	-0.39996	-0.63184	p15696	B-cell CLL/l	4	-0.41696			GO:0030512; P:negative regulation of transforming growth factor [illegible]
BCL7C seq:	-0.59996	0.255753	-0.94377	-1.00911	-2.16796	-1.16226	-2.46359	-1.7677	p15102	B-cell CLL/l	4	-1.23232	Akt-mTOR-PI3K; PI3Kinh2 (PI-103); PI3Kinh1 (GDC-0941); mTORinh2 (Torin-1); mTORinh [illegible]		
BCL7C pS1	-0.50884	0.296931	-0.66169	-0.7087	-1.66745	-0.71931	-1.55265	-1.81733	p15103	B-cell CLL/l	4	-0.91738	Akt-mTOR-PI3K; PI3Kinh2 (PI-103); PI3Kinh1 (GDC-0941); mTORinh2 (Torin-1); mTORinh [illegible]		
BCL7B seq:	-0.22921	0.347626	-0.14387	-0.27105	-0.3268	-0.60615	-0.52313	-0.43939	p01655	B-cell CLL/l	4	-0.274			
BCL3 seq: 1	-1.26858	0.747989	0.476458	-1.6117	2.053366	3.034841	3.025369	-8.86997	p17526	B-cell lymph	4	-0.30153		Atypical NF-	GO:0030330; P:DNA damage response, signal transduction by p5 [illegible]
BCL11B seq	-0.38587	-0.75726	-0.01819	-0.22545	0	0	0	0	p16440	B-cell lymph	4	-0.17335			
BCL11B pT	-0.19904	-0.6242	0.006204	-0.05663	-0.26833	1.161141	-1.03469	1.076128	p04673	B-cell lymph	4	0.007572			
BCKDHA se	0.940991	0.49059	0.874504	0.76054	-1.52652	-0.82255	-0.84438	-1.67474	p14262	2-oxoisoval	4	-0.22519			
BCAS3 seq:	-0.34588	-0.24351	-0.28253	-0.23764	-0.76039	-0.71391	-0.75094	-0.80219	p08650	Breast carci	4	-0.51712			
BCAS1 pS5	-0.247	-0.90262	-0.44266	-0.97229	0	0	0	0	p08008	Breast carci	4	-0.32057			
BCAR3 seq:	0.103631	-1.0996	0.111598	-0.04644	0.818561	-0.52747	0.284421	0.69645	p20524	Breast canc	4	0.042644		Regulation (	GO:0007264; P:small GTPase mediated signal transduction; GO:0 [illegible]
BCAR3 seq:	0.068343	-0.90185	0.181225	0.095545	0.489505	-0.42498	0.151052	0.588057	p05657	Breast canc	4	0.030862		Regulation (	GO:0007264; P:small GTPase mediated signal transduction; GO:0 [illegible]
BCAR3 pS3	0.21678	-0.2574	0.671411	0.518006	0.636961	-1.9007	-1.60891	0.647637	p02799	Breast canc	4	-0.13453		Regulation (	GO:0007264; P:small GTPase mediated signal transduction; GO:0 [illegible]
BCAR3 pS3	-8.62883	1.513145	-0.1382	-0.30208	0.221607	0.576991	0.41016	0.520086	p11092	Breast canc	4	-0.72839		Regulation (	GO:0007264; P:small GTPase mediated signal transduction; GO:0 [illegible]
BCAR3 pS3	-0.35567	-1.03241	-0.21939	-0.18738	0.760756	-0.68478	0.262535	0.546408	p20525	Breast canc	4	-0.11374		Regulation (	GO:0007264; P:small GTPase mediated signal transduction; GO:0 [illegible]
BCAR1 pS1	-0.90491	-0.68721	-0.61367	-0.76957	-0.95791	-0.50644	-0.65272	-0.69019	p19772	Breast canc	4	-0.72283		EPHA2 forw	GO:0048010; P:vascular endothelial growth factor receptor signa [illegible]
BAZ2A seq:	0.494071	-0.99776	0.552058	0.302444	-0.12628	-0.60291	0.189966	-0.16697	p10184	Bromodom	4	-0.04442	CDK2;		
BAZ1B pS3	0.204721	-0.39229	-0.20397	-0.45962	-0.24539	-0.25012	-0.19831	-0.70835	p05054	Tyrosine-pr	4	-0.28167			
BARD1 pS3	-0.15429	-0.42222	0.173831	-0.29329	-0.68934	-0.60055	-0.45007	-0.75601	p10648	BRCA1-asso	4	-0.39899		BARD1 signaling events;	
BAP18 seq:	2.465908	-9.36788	0.833938	0.692535	0.542458	0.51941	0.611256	-0.92897	p08477	Chromatin	4	-0.57892	AKTinh1 (Akt Inhibitor VIII);		
BAP18 seq:	0.453871	0.338196	0.526041	0.322691	-0.87593	-0.90851	-0.28636	-0.79717	p12456	Chromatin	4	-0.15339			
BAP18 seq:	0.243857	0.125296	0.353192	0.149682	-0.53145	-0.35647	-0.12451	-0.55925	p12455	Chromatin	4	-0.08746			
BAP18 pS9	0.106814	0.046937	0.167066	0.27889	-0.40288	-0.1425	-0.19416	-0.30167	p00543	Chromatin	4	-0.05519			
BAP18 pS1	0.408637	0.154719	0.453613	0.265564	-3.19661	-0.54942	-0.82866	-1.17042	p01760	Chromatin	4	-0.55782			
BAP1 seq: 5	-3.84961	-1.12096	-1.30033	-1.64619	1.184954	-0.20234	-0.36156	-0.2837	p06307	Ubiquitin ca	4	-0.94747			
BANF2 pM1	-0.252	-0.59837	-0.32351	-0.821	0.243046	-1.07509	-0.01618	-0.26056	p10212	Barrier-to-a	4	-0.38796			
BAIAP2L1 s	-6.97256	0.032835	-0.05463	0.23149	-0.70593	-0.64145	0.413372	0.426756	p05454	Brain-specil	4	-0.90877			GO:0007165; P:signal transduction;
BAIAP2L1 s	-1.49812	0.259235	-0.06033	-0.85871	-0.46158	-0.90069	-0.54277	-0.45901	p14958	Brain-specil	4	-0.56525			GO:0007165; P:signal transduction;
BAIAP2L1 s	-0.94134	-0.92865	-0.64809	-1.09068	-0.43878	-0.85702	-0.23166	-0.35547	p03232	Brain-specil	4	-0.68646			GO:0007165; P:signal transduction;
BAIAP2L1 s	-0.25085	-0.76072	-0.94354	-1.47652	-0.54502	-1.30651	-0.46773	-0.87533	p03234	Brain-specil	4	-0.82828			GO:0007165; P:signal transduction;
BAIAP2L1 s	-0.57688	-4.01408	-0.24826	0.090711	-1.1042	-0.82728	-0.79554	-0.42379	p19082	Brain-specil	4	-0.98741			GO:0007165; P:signal transduction;

[illegible]

ULK1 seq: 6	0.034337	0.512907	0.229869	0.464419	0.38525	0.142033	0.351426	0.310198	p09269	Serine/threo	3	0.303805		PI3Kinh2 (P	mTOR signa	GO:0007265; P:Ras protein signal transduction;
UCK1 seq: 2	0.261526	0.36152	0.049645	-0.04579	-0.30388	0.639594	-0.01139	-0.30509	p02845	Uridine-cyti	3	0.080766		Up with IGF - down with Akt-mTOR-p70S6K-PI3K; Up with EGF - down with Akt-mTOR-p70S6K-PI3K; Up with IGF - down with PI3Kinh2 (PI-103); Up with EG [illegible]		
UCK1 seq: 2	0.283614	0.584565	0.193484	0.213262	0.399865	1.062884	0.541011	0.446504	p02846	Uridine-cyti	3	0.465649		Up with IGF - down with Akt-mTOR-p70S6K-PI3K; Up with EGF - down with Akt-mTOR-p70S6K-PI3K; Up with IGF - down with PI3Kinh2 (PI-103); Up with EG [illegible]		
UBXN7 seq	0.483584	0.091592	0.653783	0.681684	0.347864	0.250947	-0.12367	0.101137	p05984	UBX domai	3	0.310865				
UBXN7 pS3	1.7163	2.023222	1.101319	1.132054	0.045714	1.029921	0.850631	0.648829	p04594	UBX domai	3	1.068499				
UBXN1 seq	-0.52013	1.111934	0.612918	0.540207	0.200928	0.356411	0.305442	0.496738	p00370	UBX domai	3	0.388056	CDK2;			
UBR5 seq: 6	0.46446	1.170507	-0.81381	0.354355	0.548924	1.123511	-0.49024	0.546406	p03491	E3 ubiquitir	3	0.363015				GO:0090263; P:positive regulation of canonical Wnt signaling pathway;
UBR5 pS24	-0.14666	-0.02719	0.005491	0.17073	0.794368	0.582192	0.351805	0.925532	p03219	E3 ubiquitir	3	0.332034				GO:0090263; P:positive regulation of canonical Wnt signaling pathway;
UBR4 seq: 2	-0.25284	-0.65564	1.371291	1.602266	0.737253	-0.04164	1.419741	0.043953	p02164	E3 ubiquitir	3	0.528049				
UBE2V2 sec	0.511421	1.428376	0.490302	0.430069	0.223866	0.154478	0.614416	0.509763	p13910	Ubiquitin-c	3	0.545336				
UBE2O seq	0.121676	1.791197	0.179531	0.651311	0.423257	1.126252	1.81318	1.155838	p09356	(E3-indeper	3	0.90778				
UBE2O seq	0.536015	0.803408	0.711503	-0.13618	0.705661	0.518582	0.785366	0.733252	p11594	(E3-indeper	3	0.582201				
UBE2B seq:	0.377039	0.286452	0.039261	0.107077	-0.29423	-0.1776	-0.10699	-0.2171	p06448	Ubiquitin-c	3	0.001739				GO:0060070; P:canonical Wnt signaling pathway;
UBAP1 seq	0.59916	-0.06547	0.708496	0.822669	0.64122	-0.90572	0.558819	0.706205	p05060	Ubiquitin-a	3	0.383172				
UBAP1 seq	0.834467	0.853101	-0.64687	0.69817	-0.07901	0.772056	2.413789	2.355375	p09545	Ubiquitin-a	3	0.900134				
UBAP1 pS1	-0.00873	1.552949	0.382675	0.257433	0.704651	0.712168	0.612804	0.480125	p01158	Ubiquitin-a	3	0.58676				
UBA1 seq: 6	1.908916	4.159175	-5.86689	1.061296	0	0	0	0	p08152	Ubiquitin-li	3	0.157812				
UBA1 seq: 4	2.810408	4.490209	-1.42414	-5.22944	0	0	0	0	p00384	Ubiquitin-li	3	0.08088				
UBA1 seq: 1	0.081329	0.689573	-0.33844	0.223703	1.032003	1.23464	1.250584	1.388689	p06442	Ubiquitin-li	3	0.69526				
UBA1 seq: 1	1.120247	2.753644	1.185081	1.80865	0	0	0	0	p06444	Ubiquitin-li	3	0.858453				
UBA1 pS81	0.199118	0.425561	1.078813	0.2248	-0.50308	0.237485	-0.3589	-0.09071	p14200	Ubiquitin-li	3	0.151636				
U2AF2 seq:	0.946464	-0.0628	0.397348	0.382378	1.340933	-0.16562	1.54421	0.977324	p14292	Splicing fact	3	0.67003				
U2AF2 seq:	0.168475	0.674326	0.250025	0.279738	0.052555	-1.57518	1.034463	0.438181	p11774	Splicing fact	3	0.165323				
TXNRD1 se	-0.0049	-0.16159	-0.07868	-1.06567	-0.00282	0.568027	1.033044	0.982793	p00655	Thioredoxin	3	0.158777				GO:0007165; P:signal transduction;
TXNRD1 se	-0.6901	1.52524	-0.04914	0.412411	0.450156	1.36932	1.361978	1.341799	p11230	Thioredoxin	3	0.715208				GO:0007165; P:signal transduction;
TXNL4A pS	-0.02298	0.1836	0.111814	0.009508	0.990703	1.220699	1.335735	1.218944	p15617	Thioredoxin	3	0.631003				
TXNL1 seq:	-0.32118	0.159884	-0.32684	-0.27865	0.576447	-0.01835	0.521014	0.450595	p16515	Thioredoxin	3	0.095364				
TXNIP seq:	1.39209	-0.39892	1.390556	0.653724	-0.19699	0.225849	0.404259	1.348858	p02113	Thioredoxin	3	0.602429				GO:0048008; P:platelet-derived growth factor receptor signaling pathway; GO:0035872; P:nucleotide-binding domain, leucine rich [illegible]
TXNDC17 s	1.105273	1.098653	1.244139	1.169401	0.227167	0.301393	0.231954	0.130694	p03206	Thioredoxin	3	0.688584				GO:0033209; P:tumor necrosis factor-mediated signaling pathway;
TXN seq: 9	-0.18092	0.144263	-0.32757	-0.70018	0.385952	0.582243	0.641226	0.691137	p03091	Thioredoxin	3	0.15452			p38 MAPK	GO:0035872; P:nucleotide-binding domain, leucine rich repeat containing receptor signaling pathway; GO:0007267; P:cell-cell signa [illegible]
TXLNG seq:	0.212165	1.379231	-0.04946	0.228375	-7.33888	3.465908	1.408649	0.703007	p09388	Gamma-tax	3	0.001124			Validated transcriptional targets of AP1 family members Fra1 and Fra2;	
TWF1 seq: 1	0.693948	0.82528	0.95115	1.265419	0.96976	1.655061	1.550169	1.428182	p00257	Twinfilin-1	3	1.167371		Up with EGF - down with AKTinh2 (MK-2206); Up with EGF - down with AKTinh1 (Akt Inhibitor VIII); AKTinh2 (MK-2206); AKTinh1 (Akt Inhibitor VIII);		
TUBB8 seq:	0.019427	1.285208	-0.23306	-0.2137	1.195262	0.882372	0.482933	0.147617	p09370	Tubulin bet	3	0.445757				
TUBB4B sec	-0.09694	1.270163	-0.0481	-0.35645	1.241503	1.315553	0.881057	0.584751	p19221	Tubulin bet	3	0.598942				
TUBB seq: 7	0.434627	1.49481	0.497418	-0.40333	-1.15702	-0.6719	-0.34331	0.452132	p05711	Tubulin bet	3	0.037928				
TUBB seq: 2	2.949328	4.331384	2.944772	-4.65091	-2.54229	1.033978	-5.68434	0.513115	p11652	Tubulin bet	3	-0.13812				
TUBB seq: 2	-0.34116	0.622048	-0.28759	-0.95293	1.299539	0.524487	0.390432	0.479457	p16536	Tubulin bet	3	0.216786				
TUBA1A sec	-0.48317	0.565295	-0.59606	-0.92413	1.113704	0.968666	0.26777	0.294121	p18104	Tubulin alpl	3	0.150775			p73 transcription factor network;	
TTLL12 seq	0.238933	0.652595	0.297589	-0.2652	-1.15391	-0.43589	0.212327	-0.18545	p06978	Tubulin--tyı	3	-0.07987		AKTinh1 (Akt Inhibitor VIII);		
TTK seq: 81	0.002984	-0.66888	0.298404	0.095192	0.957376	0.845175	1.228148	0.919829	p00019	Dual specifi	3	0.459779	TTK; ERK2; ERK1;			
TTC7A pS18	1.923116	1.518892	2.361334	3.51536	0.57722	-0.00649	0.606957	-0.00253	p11102	Tetratricope	3	1.311732				
TSC22D4 se	1.889784	3.629369	2.82937	2.195456	-0.97761	-0.81542	-0.80854	-0.10968	p16465	TSC22 dom	3	0.979091		AKTinh1 (Akt Inhibitor VIII);		
TSC22D4 p	0.093817	0.937652	0.81514	0.452461	-0.10446	0.237225	0.191725	0.15224	p20997	TSC22 dom	3	0.346974				
TSC2 pS113	0.196965	-0.00419	0.256792	0.174289	0.735635	0.248454	0.546768	0.617637	p04872	Tuberin	3	0.346544			p38 signalir	GO:0014067; P:negative regulation of phosphatidylinositol 3-kinase signaling; GO:0048009; P:insulin-like growth factor receptor si [illegible]
TSC1 seq: 3	0.759647	1.139633	-0.02093	0.420213	0.005885	1.234945	-0.10579	-2.1603	p15081	Hamartin	3	0.159163			LKB1 signal	GO:0032007; P:negative regulation of TOR signaling; GO:0045859; P:regulation of protein kinase activity; GO:0008286; P:insulin re [illegible]
TRUB1 seq:	1.314917	3.237478	0.916266	2.153437	0	0	6.366084	0	p02936	Probable tR	3	1.748523		ROCKinh1 (H-1152);		
TRPS1 pS10	0.309563	0.159436	0.562248	0.311302	0	0	0	0	p14696	Zinc finger t	3	0.167819				GO:0007178; P:transmembrane receptor protein serine/threonine kinase signaling pathway;
TRPM5 seq	-0.12197	0.251446	0.062912	0.134993	0.388577	-0.00671	0.348545	0.273313	p07521	Transient re	3	0.166388				
TRPM3 seq	0.229157	0.127659	0.654972	0.599132	0.658776	0.220898	0.077951	0.335079	p03189	Transient re	3	0.362953				
TRIP10 seq	-0.08834	-0.00479	0.556044	0.495269	0.121118	-0.05484	-0.11933	0.008061	p19926	Cdc42-inter	3	0.114148			Insulin-med	GO:0051056; P:regulation of small GTPase mediated signal transduction; GO:0007264; P:small GTPase mediated signal transductio [illegible]
TRIP10 pS2	0.007126	-0.27889	0.229589	0.751805	-0.01212	-0.30577	0.018926	-0.10094	p19925	Cdc42-inter	3	0.038716			Insulin-med	GO:0051056; P:regulation of small GTPase mediated signal transduction; GO:0007264; P:small GTPase mediated signal transductio [illegible]
TRIOBP seq	1.477967	2.382326	1.478855	1.616142	0.216283	0.811617	0.639238	0.614889	p14952	TRIO and F-	3	1.154665				
TRIO seq: 2	-0.33618	0.747755	-0.42605	-0.868	1.221134	0.582027	0.730726	0.622208	p11994	Triple funct	3	0.284203			Netrin-med	GO:0051056; P:regulation of small GTPase mediated signal transduction; GO:0048011; P:neurotrophin TRK receptor signaling pathw [illegible]
TRIM47 pS	0.516163	1.609506	0.625281	0.7919	0.423897	0.520498	0.425938	0.33054	p07385	Tripartite m	3	0.655465				
TRIM47 pS	0.614714	1.8502	0.737291	0.898526	0.762936	0.808964	0.848899	0.856541	p16039	Tripartite m	3	0.922259				
TRIM34 sec	-0.45126	-0.74534	0.056234	0.166261	0.597202	0.56918	0.274932	0.939389	p12002	Tripartite m	3	0.175825				
TRIM33 pS	0.147777	0.761008	-0.33067	-0.0463	0.065996	0.34524	0.318377	0.811704	p07910	E3 ubiquitir	3	0.259142				GO:0017015; P:regulation of transforming growth factor beta receptor signaling pathway; GO:0030514; P:negative regulation of BM [illegible]
TRIM3 pS7	0.536911	0.728483	0.705849	0.523776	0.503285	0.369796	0.49918	-1.56418	p07489	Tripartite m	3	0.287887				
TRIM3 pS4	0.026574	0.114274	-0.0408	-0.00175	0	0	4.463092	0	p20186	Tripartite m	3	0.570174				
TRIM28 sec	0.331878	0.967647	-0.30035	0.395343	0.547713	1.384191	-0.78115	0.530588	p04054	Transcriptic	3	0.384483	Chk2;		ATM pathway; p53 pathway; E2F transcription factor network; C-MYB transcription factor network;	
TRIM28 sec	0.324504	0.974822	-0.26306	0.415529	0.530101	1.333814	-0.75911	0.551445	p04055	Transcriptic	3	0.388506	Chk2;		ATM pathway; p53 pathway; E2F transcription factor network; C-MYB transcription factor network;	
TRIM28 sec	0.103946	0.632277	-0.7316	0.150202	0.389543	1.301281	-0.96108	0.382595	p04057	Transcriptic	3	0.158396	Chk2;		ATM pathway; p53 pathway; E2F transcription factor network; C-MYB transcription factor network;	
TRIM28 pS	0.596751	0.309565	0.28513	0.655552	0.171529	0.721346	-0.12068	0.177389	p12073	Transcriptic	3	0.349573			ATM pathway; p53 pathway; E2F transcription factor network; C-MYB transcription factor network;	
TRIM28 pS	0.09772	0.492565	0.09495	0.151448	-0.3056	0.347346	-1.04338	0.610328	p00828	Transcriptic	3	0.055672		ROCKinh1 (	ATM pathway; p53 pathway; E2F transcription factor network; C-MYB transcription factor network;	
TRIM28 pS	-0.04785	1.022456	-1.82665	0.25866	1.036097	1.836359	-2.50168	0.860466	p05773	Transcriptic	3	0.079732	Chk2;		ATM pathway; p53 pathway; E2F transcription factor network; C-MYB transcription factor network;	
TRIM28 pS	0.520939	1.144055	-0.09937	0.588461	0.706827	1.302372	-0.92701	0.503417	p05772	Transcriptic	3	0.467462	Chk2;		ATM pathway; p53 pathway; E2F transcription factor network; C-MYB transcription factor network;	
TRIM16 sec	0.284107	0.427905	0.548585	0.404721	0.371985	0.525743	0.304234	0.17266	p11707	Tripartite m	3	0.379993				
TRAPPC12	0.964634	1.146979	0.77631	0.79538	-0.0704	0.198002	-0.04756	0.045923	p04429	Trafficking p	3	0.476158				
TRAPPC10	0.908639	1.258541	0.58308	0.484062	0.744779	0.568699	-0.08084	-0.33587	p06816	Trafficking p	3	0.516386		EGFRinh1 (PD-168393);		
TRAF3IP3 s	1.031467	2.252637	1.001138	1.395326	1.473972	2.239293	2.339099	1.789168	p07241	TRAF3-inter	3	1.690263				
TRA2B seq:	2.706495	3.907438	1.727943	0.781335	-0.72316	0.206076	0.641998	0.272561	p14643	Transforme	3	1.190086				
TRA2B seq:	0.755056	0.619434	0.027384	-0.49013	0.027665	0.148456	-0.14187	-0.45595	p06927	Transforme	3	0.061255		ROCKinh1 (H-1152); PKCinh2 (Bisindolylmaleimide-1); PKCinh1 (Go-6976);		
TRA2A seq:	0.512843	-0.04051	0.152257	-0.14221	0.03581	-0.12413	-0.02269	0.162514	p20499	Transforme	3	0.066735				
TRA2A pS8	0.267968	0.286846	0.62936	0.754965	-0.06747	0.218784	0.32363	0.189031	p03115	Transforme	3	0.32539				
TPX2 pT72	0.606039	-0.11036	1.051642	0.955099	-0.06878	0.045466	0.578682	0.048884	p20047	Targeting pr	3	0.388334	CDK2;		Aurora A si	GO:0032147; P:activation of protein kinase activity;
TPT1 seq: 3	0.868659	2.222025	0.278048	-0.47188	-0.89978	-1.23813	-0.50421	-0.31823	p02912	Translation	3	-0.00794			PLK1 signaling events;	
TPRN seq: 3	-0.13495	0.923226	-0.13851	0.54706	0.245334	0.339595	0.11449	0.41776	p16215	Taperin	3	0.28925				GO:0019221; P:cytokine-mediated signaling pathway;
TPRN pS27	0.43203	1.193719	0.072807	0.42245	-0.90799	-0.45521	-0.90471	-0.61707	p01596	Taperin	3	-0.0955				GO:0019221; P:cytokine-mediated signaling pathway;
TPR seq: 16	0	0	8.977516	7.91726	-0.88911	-0.77296	-0.12663	-0.46533	p15908	Nucleoprot	3	1.830094	CDK2;			GO:0019221; P:cytokine-mediated signaling pathway;
TPR pT213	1.64811	2.19925	1.63125	1.931977	0.063401	0.247214	0.840927	0.581346	p01930	Nucleoprot	3	1.142934				GO:0019221; P:cytokine-mediated signaling pathway;
TPR pT211	0.740411	1.307193	-0.80887	-2.8685	-0.01129	0.553342	0.699176	0.572874	p15664	Nucleoprot	3	0.023042				GO:0019221; P:cytokine-mediated signaling pathway;
TPR pS215	0.935091	1.235436	1.036832	0.934603	0.334544	0.435676	0.58021	0.49952	p02966	Nucleoprot	3	0.748989		Up with IGF - down wit		GO:0019221; P:cytokine-mediated signaling pathway;
TPPP seq: 3	0.461679	1.908623	0.860707	0.728384	-1.08239	0.434375	2.787486	1.25795	p07128	Tubulin pol	3	0.919601	PKACA;			
TPP2 seq: 1	0.270175	1.086787	0.529181	0.526242	0.003601	0.1732	2.216561	2.824587	p12692	Tripeptidyl-	3	0.953792				
TPM3 seq:	1.162516	3.013457	1.149884	-0.61234	0.748387	0.788015	0.105632	1.104886	p10183	Tropomyos	3	0.932555				
TPM1 seq:	1.162516	3.013457	1.149884	-0.61234	0.748387	0.788015	0.105632	1.104886	p10186	Tropomyos	3	0.932555				
TPI1 seq: 2	-0.31081	0.139221	-0.56516	-0.64008	0.949461	0.597598	0.256489	0.624862	p03004	Triosephos	3	0.131446				
TPI1 pS58	0.789295	-0.0352	0.659271	0.625627	-0.64784	-0.00789	0.214965	0.221081	p07888	Triosephos	3	0.227415				
TPI1 pS117	-0.06872	1.315066	0.279595	-0.20338	1.198563	0.806573	0.768611	1.293234	p00697	Triosephos	3	0.673694				
TPD52L2 se	0.396884	-0.09301	0.669383	0.748837	1.305611	0.618757	1.082675	1.556417	p15700	Tumor prot	3	0.785694				
TPD52L2 p	0.160396	-0.28414	0.312055	0.472537	1.35222	0.631764	1.097171	1.280834	p15702	Tumor prot	3	0.627855		AKTinh1 (Akt Inhibitor VIII);		
TPD52L2 p	0.123764	-0.07358	0.415816	0.526519	1.040696	0.374903	0.852684	1.262103	p15701	Tumor prot	3	0.565363		AKTinh1 (Akt Inhibitor VIII);		
TPD52 pS1	0.232215	0.606263	0.797788	0.629871	0.562851	0.396763	0.692054	0.307852	p12004	Tumor prot	3	0.528207				
TP53BP1 se	-0.86891	-0.19569	0.667278	0.575452	0.255004	0.6902	0.154985	-0.50968	p09592	Tumor supp	3	0.09608			ATM pathway;	
TP53BP1 p	0.258169	-0.02327	0.321617	0.20939	0.184324	0.213336	0.225755	0.526253	p13315	Tumor supp	3	0.239447		AKTinh2 (M	ATM pathway;	
TP53BP1 p	0.176521	0.612556	0.365473	0.404052	0.294736	0.131467	0.245258	0.195342	p04926	Tumor supp	3	0.303176			ATM pathway;	
TP53BP1 p	0.571958	0.513184	0.359974	0.547434	0.128349	0.545442	0.64952	0.851746	p17278	Tumor supp	3	0.520951		Up with IGF	ATM pathway;	
TP53BP1 p	0.670421	0.577627	0.652928	0.421652	-0.20708	0.138171	0.585177	0.116176	p06045	Tumor supp	3	0.369383			ATM pathway;	
TP53BP1 p	2.723881	2.823748	0.945164	2.330502	-0.75808	-0.19339	0.002382	0.181096	p09829	Tumor supp	3	1.006912		AKTinh1 (A	ATM pathway;	
TOX4 pS17	0.477099	0.814556	0.670587	0.65025	-0.24944	0.049418	0.026237	-0.21776	p10610	TOX high m	3	0.277618				
TOR1AIP1 s	0.270799	0.124253	0.598335	0.18622	0.005988	-0.42017	0.147539	-0.47841	p18275	Torsin-1A-ir	3	0.05432				
TOM1L2 se	-0.21835	1.110721	0.333103	0.021407	-0.15373	-0.21561	-0.09611	-0.16071	p21170	TOM1-like p	3	0.077589				GO:0007165; P:signal transduction;
TOM1 seq:	-0.01429	1.406327	-0.09741	0.288202	0.106663	0.205642	0.108351	-0.1148	p21183	Target of M	3	0.236086			C-MYB transcription factor network;	
TOM1 pS35	0.920436	-0.44262	0.210412	0.587169	0.639574	-0.08223	0.806615	0.590275	p20626	Target of M	3	0.403704			C-MYB transcription factor network;	
TOM1 pS16	0.882927	0.78033	1.199818	0.75502	0.236787	0.58479	1.385334	1.141327	p13187	Target of M	3	0.870792			C-MYB transcription factor network;	
TOM1 pS16	0.542227	2.019668	0.671466	0.09468	-0.05936	-0.12263	0.210987	-0.02348	p13186	Target of M	3	0.416695			C-MYB transcription factor network;	
TOM1 pS16	1.373095	2.309617	1.011271	1.180041	0.425554	0.600792	1.038511	1.509874	p15762	Target of M	3	1.181094			C-MYB transcription factor network;	
TNRC6C sec	2.030454	1.439443	2.434055	3.131484	0	0	0	0	p08475	Trinucleotic	3	1.12943				GO:0007219; P:Notch signaling pathway; GO:0048015; P:phosphatidylinositol-mediated signaling; GO:0008543; P:fibroblast grow [illegible]
TNRC6C sec	0.366924	1.81965	0.253937	0.203165	-1.01852	-0.89511	-0.60039	-0.09136	p05745	Trinucleotic	3	0.004788				GO:0007219; P:Notch signaling pathway; GO:0048015; P:phosphatidylinositol-mediated signaling; GO:0008543; P:fibroblast grow [illegible]
TNRC6C sec	1.369077	0.965097	0.54371	0.424203	0	0	0	0	p17107	Trinucleotic	3	0.412761				GO:0007219; P:Notch signaling pathway; GO:0048015; P:phosphatidylinositol-mediated signaling; GO:0008543; P:fibroblast grow [illegible]
TNRC6B sec	0.445337	1.506561	0.378757	0.66223	-0.75649	0.190785	-0.01549	0.14661	p15861	Trinucleotic	3	0.319787				GO:0007219; P:Notch signaling pathway; GO:0048015; P:phosphatidylinositol-mediated signaling; GO:0008543; P:fibroblast grow [illegible]
TNRC18 sec	1.925989	1.833375	-0.14435	1.34389	-2.22284	-0.78621	-0.79681	-0.22977	p01661	Trinucleotic	3	0.115408				
TNRC18 pS	0.372625	0.471737	0.621617	0.418993	-0.12181	-0.02678	-0.01762	-0.19521	p02757	Trinucleotic	3	0.190444				
TNKS1BP1	0.457591	0.651189	0.422738	0.400789	-0.14314	0.070783	0.122048	0.244573	p05905	182 kDa tar	3	0.278322				
TNKS1BP1	0.348544	0.605036	0.373776	0.387761	0.055776	0.009256	0.271707	0.397388	p05904	182 kDa tar	3	0.306156				
TNKS1BP1	2.391632	3.352523	2.706001	2.42433	-2.29353	-2.89153	-1.19066	-1.19179	p18995	182 kDa tar	3	0.413372		Up with IGF - down with PI3Kinh1 (GDC-0941); PI3Kinh1 (GDC-0941);		
TNKS1BP1	-0.33771	1.278936	-0.20274	-0.09453	-0.69674	-0.04991	-0.22787	-0.22161	p07403	182 kDa tar	3	-0.06902				
TNKS1BP1	0.11069	0.721704	-0.13224	0.123534	-0.51651	0.126005	0.117522	0.131407	p16225	182 kDa tar	3	0.085264				
TNKS1BP1	-0.00395	1.097094	0.07951	-0.03362	-0.20638	0.148626	-0.09252	0.190916	p14716	182 kDa tar	3	0.147459				
TNKS1BP1	0.206248	1.983335	0.666712	0.698225	0.489783	0.571506	0.264364	0.146375	p09383	182 kDa tar	3	0.628319				
TNKS1BP1	0.40648	1.149946	-2.05466	0.65145	0.673761	0.587148	0.101672	0.560831	p15285	182 kDa tar	3	0.259578				
TNKS1BP1	0.276316	0.236823	0.775619	0.742387	0.22598	0.201879	0.089601	0.30196	p18263	182 kDa tar	3	0.356321		Up with IGF - down with PI3Kinh1 (GDC-0941); PI3Kinh1 (GDC-0941);		
TNKS1BP1	0.493014	1.369612	0.925104	0.922276	1.513289	1.121334	2.030848	2.206658	p12634	182 kDa tar	3	1.322767				
TNKS1BP1	-0.52496	0.411082	-0.2972	-0.27028	0.281234	0.679694	0.155136	0.401935	p04274	182 kDa tar	3	0.10458				
TNKS1BP1	-0.28579	1.348753	0.506876	0.429916	0.300921	0.238004	0.023776	0.122833	p18155	182 kDa tar	3	0.335661				
TNKS1BP1	0.133992	0.589846	0.179599	0.324513	0.359951	0.470188	0.300868	0.377601	p00397	182 kDa tar	3	0.34207				
TNKS1BP1	0.205093	0.66547	0.140643	0.296494	0.477962	0.542001	0.433567	0.570447	p00398	182 kDa tar	3	0.41646				
TNKS1BP1	0.066729	0.931435	0.515217	0.396337	-0.08961	0.022314	0.147871	-0.07306	p16484	182 kDa tar	3	0.239654	CDK2;			
TNKS1BP1	0.575407	1.292099	0.681207	0.527144	0.484671	0.644433	0.68851	0.56149	p14944	182 kDa tar	3	0.68187				
TNKS1BP1	0.160911	0.638427	-0.1286	-0.07515	-0.32847	-0.06502	-0.1452	-0.28615	p13263	182 kDa tar	3	-0.02866				
TNKS1BP1	0.207417	0.678981	0.279391	0.18593	-0.68376	-0.12296	-0.18544	-0.27355	p16975	182 kDa tar	3	0.010751				
TNKS1BP1	0.294377	0.818904	0.259999	0.19829	0.227704	0.245472	0.161051	0.275082	p09181	182 kDa tar	3	0.31011				
TNKS1BP1	0.256206	0.923679	1.023584	0.679482	0.251767	0.794686	0.68735	1.217706	p00987	182 kDa tar	3	0.729307				
TNKS1BP1	-0.47383	0.980723	-0.31026	-0.30249	0.592999	0.442962	0.421857	0.623413	p14718	182 kDa tar	3	0.246921				
TNKS1BP1	0.250569	0.500684	0.128216	0.236151	-0.00521	0.347434	0.444771	0.819553	p15284	182 kDa tar	3	0.340271				
TNK2 seq: 8	0.492437	0.764432	0.428682	0.367675	-0.32831	-0.36287	-0.19793	-0.60219	p00436	Activated Cl	3	0.07024			CDC42 sign	GO:0007166; P:cell surface receptor signaling pathway; GO:0007264; P:small GTPase mediated signal transduction;
TNK2 seq: 8	0.48267	0.737605	0.428682	0.339441	-0.33342	-0.35172	-0.18678	-0.59104	p00437	Activated Cl	3	0.06568			CDC42 sign	GO:0007166; P:cell surface receptor signaling pathway; GO:0007264; P:small GTPase mediated signal transduction;
TNFRSF10D	0	0	0	0	-0.97973	0.613438	1.027553	-0.615	p11252	Tumor necr	3	0.005783			TRAIL signal	GO:0007165; P:signal transduction;
TMX1 pS27	0.099308	0.011414	-0.18735	-0.63045	0.471502	0.06186	0.888171	0.676933	p07959	Thioredoxin	3	0.173924				
TMX1 pS24	0.460339	0.397405	0.659798	0.35414	0.050804	0.106002	0.218712	-0.60061	p12446	Thioredoxin	3	0.205823				
TMPO seq:	0.116534	0.786364	-0.25664	-0.55015	0.99714	0.637797	-0.10952	0.657012	p18701	Lamina-ass	3	0.284817				
TMPO seq:	0.42016	0.363688	0.208436	-0.20087	0.528262	-0.09945	0.919539	0.336939	p08213	Lamina-ass	3	0.309589				
TMPO seq:	0.821086	-0.42353	0.25974	0.577285	0.823158	0.001925	0.916854	0.630856	p17285	Lamina-ass	3	0.450921				
TMPO pT20	-0.37487	-0.12751	0.302663	-0.01643	0.286216	0.035817	0.316645	0.135842	p20357	Lamina-ass	3	0.069796				
TMPO pS66	0.391117	0.280633	-0.31711	0.531393	0.160863	-0.11006	-0.91509	0.235196	p15477	Lamina-ass	3	0.032118				
TMPO pS42	0.237791	-0.33686	0.790269	0.354825	-0.17278	-0.2306	0.034893	-0.09248	p16518	Lamina-ass	3	0.073132	CDK1;			
TMPO pS37	0.001565	-0.50025	0.698417	0.350938	0.441376	-0.01476	0.390039	0.077547	p20282	Lamina-ass	3	0.180609				
TMPO pS31	0.269453	0.218154	0.507422	0.355961	-0.15921	0.035188	0.331053	-0.14206	p03624	Lamina-ass	3	0.176996				
TMPO pS18	0.371147	0.604574	0.864039	0.516265	0.211872	-0.29994	-0.039	-0.15283	p08215	Lamina-ass	3	0.259516				
TMOD3 sec	0.6644	0.785685	0.895119	0.241682	0.138362	0.476677	0.57762	0.431385	p17347	Tropomodu	3	0.526366				
TMOD1 sec	0.290095	1.090495	-0.10515	-0.79028	-5.76532	3.859178	3.942643	0.31405	p09349	Tropomodu	3	0.354464				
TMF1 pS77	0.562896	1.666558	0.712278	0.598011	0.37947	0.352992	0.319635	0.099605	p04748	TATA eleme	3	0.586431			Coregulation of Androgen receptor activity;	
TMF1 pS34	-0.07517	0.444213	0.917373	0.600587	0.289854	-0.00593	0.270821	0.012004	p03283	TATA eleme	3	0.306719			Coregulation of Androgen receptor activity;	
TMEM57 se	0.288719	-0.05896	0.360961	0.213343	1.264428	0.217793	1.138826	0.894277	p16400	Macoilin	3	0.539923				
TMEM151A	0.009196	-0.04808	0.033247	0.022539	-0.03202	0.066131	0.167134	0.087028	p12147	Transmemb	3	0.038148				
TMCC2 pS4	0.586911	0.530074	0.676195	0.519581	0.124465	-0.08877	0.086039	-0.05754	p16736	Transmemb	3	0.297118				
TMC8 seq:	-0.06145	0.300359	0.624023	0.465042	0.794691	0.80783	0.84758	0.423955	p19477	Transmemb	3	0.525253				
TLK2 seq: 7	1.762137	1.869881	0.79973	2.223619	0	0	0	0	p12518	Serine/threo	3	0.831921				GO:0035556; P:intracellular signal transduction;
TKT seq: 47	0.241639	0.327479	0.167019	0.087815	0.058592	0.253252	0.235371	0.128765	p02123	Transketola	3	0.187492				
TKT seq: 17	1.596586	1.480744	1.397771	0.105987	-0.17109	-0.02063	0.227029	0.682193	p12065	Transketola	3	0.662325				
TKT pS295	-0.1227	-0.17964	0.160029	-0.04776	0.316374	0.24612	0.383964	0.592779	p14085	Transketola	3	0.168647				
TK1 pS33	1.253305	0.54695	2.361414	0.194987	-0.30033	-0.32215	0.294413	-0.31424	p03543	Thymidine k	3	0.464293			E2F transcription factor network; Validated targets of C-MYC transcriptional activation;	
TJP3 seq: 1	-0.22552	0.170304	-0.10801	0.001045	0.273745	0.268596	0.941695	0.659679	p06651	Tight juncti	3	0.24769				
TJP3 pS319	1.597944	0.642387	1.062196	0.756089	0	0	0	0	p14886	Tight juncti	3	0.507327				
TJP3 pS319	1.177609	0.811586	1.591479	1.722653	0	0	0	0	p04692	Tight juncti	3	0.662916				
TJP3 pS164	-0.57872	-0.08797	-0.13995	-0.23633	0.560851	0.299724	0.928572	0.633914	p04644	Tight juncti	3	0.172511				
TJP2 seq: 1	0.739585	0.856668	0.726412	0.82549	0.374435	0.035475	0.522558	0.201824	p03993	Tight juncti	3	0.535306			Validated ta	GO:0035329; P:hippo signaling;
TJP2 seq: 1	0.369166	0.158773	0.680096	0.422638	0.345542	0.240754	0.12897	0.101646	p03995	Tight juncti	3	0.305948			Validated ta	GO:0035329; P:hippo signaling;
TJP2 seq: 1	0.387659	0.345846	0.740828	0.63121	0.476342	0.233687	0.214398	0.166649	p03994	Tight juncti	3	0.399577			Validated ta	GO:0035329; P:hippo signaling;
TJP2 seq: 1	0.888116	0.354658	0.190051	0.274729	0.066093	0.255766	0.020347	-0.04152	p14353	Tight juncti	3	0.25103			Validated ta	GO:0035329; P:hippo signaling;
TJP2 pS986	0.376851	1.066806	0.27653	0.914872	0.144306	0.135208	-0.01391	-0.12231	p21017	Tight juncti	3	0.347295			Validated ta	GO:0035329; P:hippo signaling;
TJP2 pS978	1.343037	2.167769	2.075319	1.800557	2.256616	2.557785	2.964173	2.340682	p07656	Tight juncti	3	2.188242			Validated ta	GO:0035329; P:hippo signaling;
TJP2 pS115	0.269644	0.136203	0.646655	0.891975	0.378502	0.253023	0.436709	0.551596	p15134	Tight juncti	3	0.445538		PKCinh2 (B	Validated ta	GO:0035329; P:hippo signaling;
TJP1 seq: 1	0.116638	0.045504	0.906119	0.585098	0.221569	0.093339	0.164923	0.194459	p03392	Tight juncti	3	0.290956			Nephrin/Ne	GO:0035329; P:hippo signaling;
TJP1 seq: 1	0.375539	1.581944	0.213242	0.371151	0.005946	0.248065	0.167296	0.062397	p20440	Tight juncti	3	0.378197			Nephrin/Ne	GO:0035329; P:hippo signaling;
TJP1 pS968	0.617903	-0.02481	0.569092	0.360372	-0.16277	-0.09375	-0.26704	-0.239	p11989	Tight juncti	3	0.095001			Nephrin/Ne	GO:0035329; P:hippo signaling;
TJP1 pS968	0.176353	0.891547	0.662425	0.124488	-0.35454	0.035709	-0.17432	-0.25235	p11990	Tight juncti	3	0.138665			Nephrin/Ne	GO:0035329; P:hippo signaling;
TIGAR seq:	0.315024	2.118256	-0.09136	1.591063	0.716317	2.130331	2.720384	2.801133	p17993	Fructose-2,	3	1.475144			Direct p53 effectors;	
TICRR pS10	0.535508	0.180787	0.534769	0.634061	-0.32414	-0.14324	0.036222	-0.44361	p05164	Treslin	3	0.126294		AKTinh1 (Akt Inhibitor VIII);		
TIAM1 pS3	0.058648	-0.18564	0.142426	0.218981	0.171665	0.067499	0.126596	0.668175	p04856	T-lymphom	3	0.158544			Arf6 downs	GO:0016601; P:Rac protein signal transduction; GO:0048013; P:ephrin receptor signaling pathway; GO:0051056; P:regulation of sr [illegible]
THRAP3 pT	0.332986	-0.27043	0.522635	0.298399	-0.24838	-0.23809	0.071465	-0.20598	p09198	Thyroid hor	3	0.032827	CDK2;			GO:0030518; P:intracellular steroid hormone receptor signaling pathway; GO:0030521; P:androgen receptor signaling pathway;
THRAP3 pT	0.211163	0.036013	0.988509	0.62909	-0.42662	-0.24617	0.160505	-0.19525	p09199	Thyroid hor	3	0.144653	CDK2;			GO:0030518; P:intracellular steroid hormone receptor signaling pathway; GO:0030521; P:androgen receptor signaling pathway;
THRAP3 pS	2.425129	1.062283	1.655078	1.172884	-0.22387	0.129016	0.70777	1.02345	p14810	Thyroid hor	3	0.993968				GO:0030518; P:intracellular steroid hormone receptor signaling pathway; GO:0030521; P:androgen receptor signaling pathway;
THRAP3 pS	0.643542	0.768578	0.870918	-0.02031	-0.04834	0.477771	0.760842	0.352519	p09511	Thyroid hor	3	0.47569				GO:0030518; P:intracellular steroid hormone receptor signaling pathway; GO:0030521; P:androgen receptor signaling pathway;
THRAP3 pS	0.70861	0.556472	1.030711	0.41029	-0.21279	0.123886	0.348728	-0.23811	p04723	Thyroid hor	3	0.340975				GO:0030518; P:intracellular steroid hormone receptor signaling pathway; GO:0030521; P:androgen receptor signaling pathway;
THRAP3 pS	0.350035	0.359117	0.514412	0.177523	-0.28288	-0.02497	0.103	-0.20194	p17203	Thyroid hor	3	0.124286				GO:0030518; P:intracellular steroid hormone receptor signaling pathway; GO:0030521; P:androgen receptor signaling pathway;
THRAP3 pS	0.210023	0.178228	0.628331	0.29122	-0.14075	0.081308	0.240603	-0.17578	p04724	Thyroid hor	3	0.164148				GO:0030518; P:intracellular steroid hormone receptor signaling pathway; GO:0030521; P:androgen receptor signaling pathway;
THOC2 pS1	0.004895	0.189388	0.323396	0.233143	-0.04287	0.386848	0.407487	-0.52382	p02298	THO compl	3	0.122308				
TGM2 seq:	4.585471	-1.04816	4.564986	4.997113	-0.20658	1.109002	0.895354	0.690927	p00505	Protein-glut	3	1.948514			Alpha9 beta	GO:0051482; P:positive regulation of cytosolic calcium ion concentration involved in phospholipase C-activating G-protein couplec [illegible]
TGFB1I1 se	0	0	0	0	0.035836	0.07111	0.13369	0.156328	p00943	Transformir	3	0.04962			Coregulatio	GO:0030511; P:positive regulation of transforming growth factor beta receptor signaling pathway; GO:0030521; P:androgen recepto [illegible]
TGFB1I1 pS	0	0	0	0	-0.18882	0.036149	0.623103	0.406717	p12623	Transformir	3	0.109643			Coregulatio	GO:0030511; P:positive regulation of transforming growth factor beta receptor signaling pathway; GO:0030521; P:androgen recepto [illegible]
TFRC seq: 4	-0.06964	0.969745	-0.48908	-0.06393	-0.14454	0.126824	0.06693	-0.24073	p12145	Transferrin	3	0.019446			FOXA2 and FOXA3 transcription factor networks; HIF-1-alpha transcription factor network; Validated targets of C-MYC transcriptional activation;	
TFPI2 seq: 1	0.300369	0.534543	0.055632	0.222316	-0.18409	0.107791	-0.17124	-0.43408	p02991	Tissue facto	3	0.053905				
TFPI2 seq: 1	0.481914	0.818161	0.171453	0.407589	-0.2898	-0.0816	0.086649	-0.56981	p02992	Tissue facto	3	0.128069				
TFIP11 seq:	2.46899	2.29072	1.919884	0.909083	-2.53629	-3.29501	-0.45532	-2.08283	p18832	Tuftelin-int	3	-0.0976				
TFAP2C pS	1.06772	0.906429	1.253291	1.200121	0	0	0	0	p03156	Transcriptic	3	0.553445			Validated tr	GO:0032486; P:Rap protein signal transduction; GO:0007267; P:cell-cell signaling;
TEX2 seq: 7	0.416707	1.036227	-0.40545	0.12159	0.175428	-0.01123	0.179016	-0.3523	p03700	Testis-expre	3	0.144999				GO:0007165; P:signal transduction;
TESK2 pS45	0.465327	0.373689	0.573103	0.423476	0	0	0	0	p05118	Dual specifi	3	0.229449				
TES pS168	-0.34854	1.35819	0.115154	0.107902	0.096316	0.152933	0.055305	-0.05878	p08344	Testin	3	0.184811				
TEAD3 seq:	0.132672	0.204009	0.21534	0.197753	0.242385	0.591442	0.22228	0.017588	p16452	Transcriptic	3	0.227934				GO:0035329; P:hippo signaling;
TDRD7 seq	1.540952	3.287802	1.604461	3.103287	-2.34252	0.62714	1.343284	0.168753	p14555	Tudor doma	3	1.166645			Aurora A signaling;	
TCP11L1 se	1.229199	0.920342	1.039749	0.942722	0.8024	1.178916	2.234183	1.960108	p00932	T-complex p	3	1.288452				
TCOF1 seq:	0.360062	0.714418	0.613418	0.242876	-0.17805	0.119417	0.095442	-0.00542	p05220	Treacle prot	3	0.245271	CDK2; CDK1;			
TCOF1 seq:	0.345028	0.405266	1.20932	1.003333	-0.01192	-0.10806	0.178385	-0.24153	p21051	Treacle prot	3	0.347478				
TCOF1 seq:	0.563946	0.406838	0.4694	0.983532	0.336352	0.284981	0.284066	0.548184	p12031	Treacle prot	3	0.484663				
TCOF1 seq:	0.190642	0.824106	0.709341	0.520926	-0.33405	0.045037	0.108317	-0.27759	p02269	Treacle prot	3	0.223341				
TCOF1 pT9	0.448024	-0.0666	0.663481	0.424923	-0.06974	0.066486	0.295735	0.3618	p13220	Treacle prot	3	0.265513				
TCOF1 pS5	0.430562	0.177042	0.537241	0.384835	0.02859	0.190401	0.434397	0.219791	p04448	Treacle prot	3	0.300358				
TCOF1 pS1	0.505222	0.309139	0.600612	0.823844	-0.00414	0.208758	0.149634	0.327636	p11844	Treacle prot	3	0.365089				
TCOF1 pS1	0.743084	-0.04571	0.581622	0.815117	0.681518	0.676562	0.858002	0.945854	p12925	Treacle prot	3	0.657007				
TCOF1 pS1	0.703844	0.62223	0.667455	0.630395	-0.23767	0.029669	0.165376	0.000544	p08743	Treacle prot	3	0.32273		p70S6Kinh1 (PF-4708671);		
TCF20 seq:	0.348455	0.874708	0.659511	0.733037	0.153104	0.145459	0.327409	0.264667	p20555	Transcriptic	3	0.438294				
TCF20 pS57	0.02621	1.107753	1.312357	0.598154	1.395892	0.946603	1.819429	1.027775	p11214	Transcriptic	3	1.029272				
TCF20 pS53	0	0	0	0	0.780133	0.955329	0.797172	0.83089	p08314	Transcriptic	3	0.42044				
TCF20 pS1	0.744927	0.646475	1.400129	0.371012	0.250032	0.234473	0.237654	-0.143	p18557	Transcriptic	3	0.467713				
TCEAL3 pS6	0.852301	0.817407	0.984139	0.515263	0.765603	0.221709	0.569199	0.551935	p07492	Transcriptic	3	0.659694				
TCEAL3 pS3	0.949122	0.611982	0.516672	0.395329	-0.10426	-0.08321	0.306823	0.278042	p09683	Transcriptic	3	0.358812				
TBX3 pS37	0.850943	0.809866	1.05621	0.812996	0	0	0	0	p18645	T-box trans	3	0.441252				
TBR1 seq: 8	0	1.342157	0	0	1.545282	1.526455	0.345152	1.312723	p10696	T-box brain	3	0.758971				
TBC1D5 pS	0.717229	0.582724	0.871646	0.798837	1.546639	0.50912	1.475088	1.396117	p05979	TBC1 doma	3	0.987175				
TBC1D4 sec	1.003217	0.91074	0.684078	0.47203	0.65	0.659864	0.354617	0.44349	p12610	TBC1 doma	3	0.647254			Insulin-mediated glucose transport; Class I PI3K signaling events mediated by Akt;	
TBC1D4 pS	-0.51765	0.248234	-0.01837	0.002996	-0.14366	0.283555	0.640829	0.710109	p15528	TBC1 doma	3	0.150756			Insulin-mediated glucose transport; Class I PI3K signaling events mediated by Akt;	
TBC1D10B	0.523674	0.381235	0.248667	0.280531	-0.47385	-0.13068	0.057279	0.076908	p06805	TBC1 doma	3	0.12047				
TBC1D10B	-1.15608	2.697008	-0.87217	0.416562	-0.12807	0.119162	0.208665	0.062743	p19660	TBC1 doma	3	0.168478				
TBC1D10B	0.416571	0.682768	0.282737	0.297778	-0.05961	0.773762	0.188221	0.51055	p20340	TBC1 doma	3	0.386597				
TAS2R42 se	0.833069	0.889107	-1.26965	-0.67264	-0.03102	0.387546	0.154515	0.250932	p10661	Taste recep	3	0.067731				
TARDBP sec	0.930101	2.960248	0.822094	0.457206	0	0	0	0	p17306	TAR DNA-bi	3	0.646206				
TAOK3 seq:	-0.01184	-0.00422	0.589287	0.014484	0.589683	1.092949	0.990686	0.785644	p06257	Serine/threo	3	0.505835			p38 MAPK	GO:0032874; P:positive regulation of stress-activated MAPK cascade; GO:0000186; P:activation of MAPKK activity; GO:0006469; P [illegible]
TANC2 seq:	0.258485	0.964216	0.664586	0.787505	-0.13069	0.209686	0.27643	0.039924	p08619	Protein TAN	3	0.383768				
TANC2 pS1	0.313007	1.25528	-0.20453	0.410023	-1.18024	0.538268	-0.78181	-0.87054	p06160	Protein TAN	3	-0.06507				
TAGLN2 sec	0.173067	0.870875	-0.86132	-0.95072	0.496016	0.030495	0.746837	-0.12128	p08971	Transgelin-2	3	0.047997				
TAF3 pS755	0.481053	0.391481	0.369123	0.347403	-0.72865	-0.19205	-0.54207	-0.5613	p14102	Transcriptic	3	-0.05438				
TACC3 seq:	-0.1733	0.914482	0.5433	0.537579	-0.14136	0.091928	-0.11694	-0.20675	p20067	Transformir	3	0.181118			Aurora A signaling; Integrin-linked kinase signaling;	
TACC3 pS7	0.311409	0.607687	0.232646	0.189755	-0.10509	0.055323	-0.01599	-0.10926	p13982	Transformir	3	0.14581			Aurora A signaling; Integrin-linked kinase signaling;	
TACC3 pS3	0.135107	0.975234	0.524741	0.440184	-0.31342	-0.03622	-0.16972	-0.2339	p20068	Transformir	3	0.16525			Aurora A signaling; Integrin-linked kinase signaling;	
TACC2 pS2	0.544021	1.49028	0.882466	0.943315	0.415851	0.587157	0.259019	0.496669	p09938	Transformir	3	0.702347				
TACC1 seq:	0.62249	1.724306	0.594641	0.894047	-0.07136	0.119886	0.069486	0.135663	p19430	Transformir	3	0.511145			Aurora A signaling; Aurora B signaling;	
SZRD1 pS1	-0.01346	1.004141	-0.0317	0.017734	0.003016	0.131061	-0.24395	-0.06557	p14038	SUZ domair	3	0.100159				
SZRD1 pS1	0.112954	1.131039	-0.28102	0.144895	-0.01182	0.227704	-0.1378	-0.0822	p14039	SUZ domair	3	0.13797				
SYTL1 pS39	0.221431	0.583171	0.285793	0.354235	0.061429	0.049403	-0.08872	0.093641	p00995	Synaptotag	3	0.195048				
SYNJ2 pS11	0.69622	0.973099	0.607439	0.924029	1.055051	0.873863	0.265474	1.392305	p12608	Synaptojani	3	0.848435				GO:0046856; P:phosphatidylinositol dephosphorylation; GO:0006661; P:phosphatidylinositol biosynthetic process;
SYNJ2 pS11	0.822129	0.775323	0.665002	0.827201	1.375679	1.409995	1.232113	0.973454	p06275	Synaptojani	3	1.010112				GO:0046856; P:phosphatidylinositol dephosphorylation; GO:0006661; P:phosphatidylinositol biosynthetic process;
SYNJ1 seq:	2.077377	3.0659	2.159769	1.157829	-1.17492	-1.1227	-0.44961	-0.14701	p06887	Synaptojani	3	0.695829			EPHB forwa	GO:0046856; P:phosphatidylinositol dephosphorylation; GO:0043647; P:inositol phosphate metabolic process; GO:0006661; P:p [illegible]
SYMPK pS4	0.945174	1.276137	-0.2315	0.656366	0.640512	1.519033	-0.58777	0.930034	p07148	Symplekin	3	0.643498				
SYBU pS55	0.264648	1.064285	0.013665	0.193187	0	0	0	0	p09264	Syntabulin	3	0.191973				
SVIL seq: 1	0.133583	1.674172	0.150369	0.395421	0.058054	0.56767	0.318725	0.255161	p13189	Supervillin	3	0.444144			Coregulation of Androgen receptor activity;	
SVIL pS221	0.322832	0.328499	0.170888	0.236437	-0.27092	-0.20042	-0.09275	-0.28221	p08854	Supervillin	3	0.026544			Coregulation of Androgen receptor activity;	
SVIL pS221	0.084903	0.293932	0.29036	0.272719	0.031574	-0.02231	-0.04203	0.12439	p08856	Supervillin	3	0.129192			Coregulation of Androgen receptor activity;	
SVIL pS122	0.308388	1.679561	0.346667	0.147669	0.341687	0.715171	0.671139	0.625965	p15772	Supervillin	3	0.604531			Coregulation of Androgen receptor activity;	
SURF6 seq:	1.194406	3.639148	0.052489	-4.7018	0	0	0	0	p11314	Surfeit locu	3	0.023031				
SURF6 pT2	1.459742	-0.732	0.96503	0.843328	0.015625	-0.49354	0.005125	-0.17952	p15577	Surfeit locu	3	0.235474				
SURF6 pS7	0.663063	-0.2637	1.0826	0.982243	-0.33245	-0.41706	0.021773	-0.58431	p05229	Surfeit locu	3	0.14402				
SULT2B1 se	0.203891	-0.15031	0.236039	0.033803	0.584845	0.264115	0.249054	0.324107	p20775	Sulfotransfe	3	0.218193				
SUGT1 seq:	0.11201	-0.06505	0.185891	-0.00565	0.230781	0.289245	0.39962	0.312807	p05261	Protein SGT	3	0.182456				
SUB1 pS11	1.325115	1.40846	0.51146	-0.00053	-1.46642	-0.96759	-1.25439	0.548515	p15847	Activated R	3	0.013078				
STX7 seq: 1	0.936689	0.588792	0.681762	0.880974	-0.75507	-0.36698	0.676864	1.959088	p19476	Syntaxin-7	3	0.575265				
STUB1 seq:	0.958331	0.727299	0.37982	0.274859	0.581187	0.674389	1.272121	0.644107	p11190	E3 ubiquitir	3	0.689014			Alpha-synu	GO:0030512; P:negative regulation of transforming growth factor beta receptor signaling pathway; GO:0007179; P:transforming gro [illegible]
STUB1 pS2	0.127242	0.102822	0.65145	0.412062	-0.1103	0.090333	0.049907	0.041846	p04534	E3 ubiquitir	3	0.170671			Alpha-synu	GO:0030512; P:negative regulation of transforming growth factor beta receptor signaling pathway; GO:0007179; P:transforming gro [illegible]
STRN3 pS2	0.227118	0.443593	-0.17001	0.367901	0.273938	0.743749	1.659019	1.432282	p06615	Striatin-3	3	0.622199		Up with IGF - down with		GO:0033147; P:negative regulation of intracellular estrogen receptor signaling pathway;
STRN seq: 2	0.736224	1.070105	-0.00177	0.405349	-0.31267	-0.18606	-0.59974	-0.40716	p17942	Striatin	3	0.088035			Plasma mer	GO:0016055; P:Wnt signaling pathway;
STMN1 pS3	0.162286	1.106195	0.000782	0.13291	-0.684	-0.41434	-0.31835	-0.46148	p17093	Stathmin	3	-0.0595	PKCB; PAK1	PAKinh (PF	Signaling m	GO:0035556; P:intracellular signal transduction; GO:0007165; P:signal transduction;
STMN1 pS3	0.142928	0.57852	0.227579	0.207476	-0.46636	-0.26533	-0.25295	-0.38048	p05424	Stathmin	3	-0.02608	PKCB; PAK1	PAKinh (PF	Signaling m	GO:0035556; P:intracellular signal transduction; GO:0007165; P:signal transduction;
STMN1 pS3	0.132264	0.648279	0.311169	0.198473	-0.18614	-0.16234	-0.11548	-0.11384	p17094	Stathmin	3	0.089049	PKCB; PAK1	PAKinh (PF	Signaling m	GO:0035556; P:intracellular signal transduction; GO:0007165; P:signal transduction;
STMN1 pS2	0.391272	0.820375	0.583344	0.568294	0.346184	0.280285	0.319633	0.212089	p19606	Stathmin	3	0.440184	PKCB; P38D; ERK1; CDK		Signaling m	GO:0035556; P:intracellular signal transduction; GO:0007165; P:signal transduction;
STMN1 pS2	0.52005	1.092528	0.536657	0.468395	0.066285	0.35971	0.471161	0.187149	p07581	Stathmin	3	0.462742	PKCB; P38D; ERK1; CDK		Signaling m	GO:0035556; P:intracellular signal transduction; GO:0007165; P:signal transduction;
STK3 seq: 3	0.081926	0.970705	0.314358	0.380702	0.053954	0.202461	0.289726	0.378042	p09739	Serine/threo	3	0.333984				GO:0035329; P:hippo signaling; GO:0023014; P:signal transduction by phosphorylation; GO:0051897; P:positive regulation of pro [illegible]
STK3 seq: 3	0.145458	1.226677	0.533997	0.861143	0.490841	0.37866	0.350168	0.633222	p09741	Serine/threo	3	0.577521				GO:0035329; P:hippo signaling; GO:0023014; P:signal transduction by phosphorylation; GO:0051897; P:positive regulation of pro [illegible]
STK3 pS31	0.474	0.62389	0.56234	0.365553	0.193124	0.391931	0.354419	0.458949	p17512	Serine/threo	3	0.428026				GO:0035329; P:hippo signaling; GO:0023014; P:signal transduction by phosphorylation; GO:0051897; P:positive regulation of pro [illegible]
STK17A pS	-0.01198	0.622584	0.040998	0.117689	-0.13297	-0.16583	-0.28817	-0.17721	p15648	Serine/threo	3	0.000638				GO:0035556; P:intracellular signal transduction;
STK11IP se	-0.1149	0.07932	0.234135	0.154971	0.183861	0.003974	0.266202	-0.15137	p07579	Serine/threo	3	0.082024			LKB1 signaling events;	
STK10 seq:	0.377019	0.884503	0.34276	0.307411	0.196792	0.58895	-0.11783	0.010558	p19514	Serine/threo	3	0.32377				GO:0023014; P:signal transduction by phosphorylation;
STK10 seq:	0.829101	0.704585	0.644741	0.918754	0.723272	0.408157	0.643242	0.614453	p19516	Serine/threo	3	0.685788				GO:0023014; P:signal transduction by phosphorylation;
STIP1 seq: 3	-0.20485	0.830902	-0.41117	-0.63981	1.484819	0.476939	0.696662	0.443332	p12133	Stress-indu	3	0.334604				
STIP1 pS48	1.495658	1.764978	1.357218	1.746386	1.158418	1.181314	1.722288	1.381892	p14855	Stress-indu	3	1.476019				
STAU2 seq:	0.666531	1.474258	0.613337	0.620231	0.088364	0.519231	0.474876	0.293892	p15163	Double-stra	3	0.59384				
STAU2 pS4	-0.65622	0.725223	0.144253	0.212235	-0.14529	-0.11502	-0.23225	-0.12448	p18485	Double-stra	3	-0.02394				
STAU1 pS3	0.264922	0.581636	0.28734	0.252437	0.513164	0.271933	0.193514	0.148998	p00730	Double-stra	3	0.314243				
STAT3 seq:	-0.03903	0.78189	-0.03492	0.35994	0.347583	0.411052	0.835422	0.740305	p04254	Signal trans	3	0.42528			IL27-media	GO:0045747; P:positive regulation of Notch signaling pathway; GO:0060397; P:JAK-STAT cascade involved in growth hormone signa [illegible]
STAT3 seq:	1.994462	3.846561	2.915213	2.90406	0.611952	0.477472	0.771965	1.066002	p04253	Signal trans	3	1.823461			IL27-media	GO:0045747; P:positive regulation of Notch signaling pathway; GO:0060397; P:JAK-STAT cascade involved in growth hormone signa [illegible]
STAT3 pS72	0.065947	0.54645	0.256321	0.217269	0.157729	0.249274	0.22866	0.097048	p16695	Signal trans	3	0.227337	RSK2; PKCE	Up with IGF	IL27-media	GO:0045747; P:positive regulation of Notch signaling pathway; GO:0060397; P:JAK-STAT cascade involved in growth hormone signa [illegible]
STAT3 pS72	0.237322	0.742838	0.323113	0.249596	0.152922	0.254717	0.236586	0.10262	p16696	Signal trans	3	0.287464	RSK2; PKCE	Up with IGF	IL27-media	GO:0045747; P:positive regulation of Notch signaling pathway; GO:0060397; P:JAK-STAT cascade involved in growth hormone signa [illegible]
STARD6 pS	0.139282	0.33019	0.471727	0.914901	-0.28648	-0.05408	-0.5354	-0.28975	p01634	StAR-relatec	3	0.086298				
STARD13 se	-0.67784	0.02971	-0.31042	-0.24119	0.648436	0.919571	0.387606	0.571454	p20175	StAR-relatec	3	0.165916				GO:0051056; P:regulation of small GTPase mediated signal transduction; GO:0007264; P:small GTPase mediated signal transductio [illegible]
STARD10 se	0.395359	0.016485	0.446892	0.545962	0	0	0	0	p12298	PCTP-like pı	3	0.175587				
STARD10 p	0.443522	0.570293	0.812027	0.297924	-0.68635	0.039451	-0.09366	0.083899	p10123	PCTP-like pı	3	0.183388	CK2A1;			
STAG2 seq:	0.641207	0.468972	0.447321	0.116244	0.33603	1.23869	0.502404	0.218286	p07300	Cohesin sul	3	0.496144			PLK1 signaling events;	
SSRP1 pM4	0.274857	-0.06754	0.645363	0.522168	0.429282	0.224541	0.208487	0.265959	p17712	FACT compl	3	0.31289		CAMKinh1	Validated transcriptional targets of TAp63 isoforms;	
SSR4 seq: 1	1.19329	1.462648	0.143581	-0.08057	-0.76968	-0.12284	-0.20657	0.097883	p09348	Translocon-	3	0.214717				
SSH3 pS9	0.185521	0.245179	0.243175	0.249726	0.4655	0.428916	0.300626	0.60503	p04504	Protein pho	3	0.340459		AKTinh2 (MK-2206);		
SSH3 pS64	0.485815	0.841172	1.349075	1.44861	-0.84554	-0.66955	0.501905	-2.36705	p08803	Protein pho	3	0.093055				
SSFA2 seq:	0.297384	0.302275	0.412219	0.506186	-0.12256	-0.2982	-0.39323	-0.25463	p04187	Sperm-spec	3	0.056181				
SSB pS366	-0.14703	-0.04301	-0.21184	-0.4045	0.278208	0.263763	0.484572	0.388226	p16846	Lupus La pr	3	0.076047	CK2A1;		Regulation	GO:0007265; P:Ras protein signal transduction; GO:0007218; P:neuropeptide signaling pathway; GO:0048011; P:neurotrophin TRK [illegible]
SRSF8 seq:	0.156646	0.485676	1.287953	0.66873	-0.30051	-0.11161	0.002928	-0.32119	p00077	Serine/argin	3	0.233578				
SRSF7 seq:	0.012759	0.245395	-0.37533	-0.32425	0.785437	0.720046	0.630778	0.98948	p09262	Serine/argin	3	0.335539				
SRSF7 pS23	0.452958	0.846991	0.916285	0.866595	0.6361	0.007254	0.787472	0.643233	p06699	Serine/argin	3	0.644611				
SRSF7 pS16	0.443217	0.066253	0.715789	0.16193	0.821937	0.72034	1.002159	0.723483	p04049	Serine/argin	3	0.581889				
SRSF6 pS31	1.385581	1.180524	1.056247	1.309571	1.576077	1.712125	1.81216	1.579449	p19673	Serine/argin	3	1.451467				
SRSF4 seq:	1.468881	1.55623	1.263829	1.293478	1.447138	1.704565	1.766473	1.630633	p13803	Serine/argin	3	1.516403				
SRSF4 seq:	0.545155	0.150496	0.629641	0.781425	0.071749	0.11465	-0.0819	0.023558	p11538	Serine/argin	3	0.279347				
SRSF4 pS43	1.673297	2.139493	1.355888	2.125827	0.759909	0.895946	0.819773	0.941697	p17692	Serine/argin	3	1.338979				
SRSF11 pS4	1.048248	1.355534	0.820417	0.927532	0.673885	0.400386	0.74871	0.951306	p01080	Serine/argin	3	0.865752				
SRSF11 pS2	-0.04415	0.101384	0.188282	0.513812	0.010996	0.181422	0.257293	-0.0378	p11187	Serine/argin	3	0.146404	CDK2;			
SRSF11 pS2	-0.03615	0.557232	0.507374	0.585678	0.022335	0.025032	0.162557	-0.0289	p11186	Serine/argin	3	0.224395	CDK2;			
SRSF10 seq	0.004524	0.054617	0.592543	0.263483	0.27196	0.322713	0.552825	0.115051	p03177	Serine/argin	3	0.272214				
SRSF10 seq	0.240426	0.099279	1.121451	0.345255	0.307671	0.318219	0.91349	0.254788	p04061	Serine/argin	3	0.450072				
SRSF10 seq	0.216221	0.212494	1.197652	0.276283	0.182061	0.508975	0.861932	0.270251	p04062	Serine/argin	3	0.465734				
SRSF10 pS1	0.318713	0.336285	0.187806	-0.1084	1.199421	0.785771	0.328423	1.050715	p04063	Serine/argin	3	0.512342				
SRRM2 seq	0.256043	0.445085	0.846614	0.352634	0.092743	0.251131	0.353431	-0.01642	p03997	Serine/argin	3	0.322658				
SRRM2 seq	0.581792	0.642179	0.655606	0.11756	0.358326	0.412788	0.472489	-0.01952	p03998	Serine/argin	3	0.402653				
SRRM2 seq	0.000324	-0.0011	0.387742	0.511787	0.151269	-0.07412	0.109904	0.377138	p08599	Serine/argin	3	0.182868	CDK1;			
SRRM2 seq	0.71565	1.665203	1.218448	1.380489	0.851191	1.253154	0.483329	1.170759	p08601	Serine/argin	3	1.092278				
SRRM2 seq	-0.21142	0.082909	0.593653	0.515011	0.349504	0.056517	0.168296	0.154443	p06192	Serine/argin	3	0.213614				
SRRM2 seq	-0.03301	0.157568	0.598965	0.601929	0.235834	-0.0378	0.17532	0.058635	p06193	Serine/argin	3	0.21968				
SRRM2 seq	0.088107	0.161122	0.421511	0.429268	-0.10597	-0.02836	-0.08838	-0.02666	p07458	Serine/argin	3	0.106329				
SRRM2 seq	0.378555	-0.33219	0.147218	1.017093	0.290671	0.141675	0.338439	0.165117	p06327	Serine/argin	3	0.268323				
SRRM2 seq	0.205274	0.507957	0.331037	0.243024	-0.4872	-0.15365	-0.01327	-0.41343	p03001	Serine/argin	3	0.027469		ROCKinh1 (H-1152); PKCinh1 (Go-6976);		
SRRM2 seq	0.263951	0.350678	0.33328	0.413204	0.327788	0.409859	0.356621	0.366284	p04942	Serine/argin	3	0.352708				
SRRM2 seq	0.329399	0.411367	1.404707	0.808165	0.231698	-0.01862	0.34283	-0.13252	p04943	Serine/argin	3	0.422129				
SRRM2 seq	-0.03259	1.186316	0.774196	0.344949	0.23456	0.85723	1.870491	0.870141	p15967	Serine/argin	3	0.763161				
SRRM2 seq	0.438392	0.455539	0.504281	0.600872	0.331964	0.08526	0.057145	0.021575	p06576	Serine/argin	3	0.311878				
SRRM2 seq	1.065256	2.251342	0.999122	1.393311	1.475223	2.239293	2.339099	1.789168	p04099	Serine/argin	3	1.693977				
SRRM2 seq	0.284295	0.516526	0.368359	0.304683	-0.20554	-0.23375	0.032453	0.008102	p17396	Serine/argin	3	0.134391				
SRRM2 pS9	0.190906	0.490272	0.799873	0.270191	-0.25813	-0.063	-0.03787	-0.52162	p03269	Serine/argin	3	0.108829				
SRRM2 pS7	0.100227	0.321292	0.61336	0.365619	-0.15228	0.272175	-0.35682	-0.18389	p05052	Serine/argin	3	0.12246				
SRRM2 pS3	0.326156	0.152307	0.350989	0.239354	-0.53287	0.004625	0.017507	-0.44477	p06190	Serine/argin	3	0.014162				
SRRM2 pS2	0.172271	0.489257	0.416584	0.342826	0.178841	0.363373	0.435636	0.634588	p06856	Serine/argin	3	0.379172				
SRRM2 pS2	0.208501	0.320412	0.853878	0.475452	-0.38451	-0.08158	0.028335	-0.12698	p04963	Serine/argin	3	0.161688				
SRRM2 pS2	0.509988	0.439855	0.432403	0.43574	0.239571	0.218074	0.211801	0.276602	p04631	Serine/argin	3	0.345504				
SRRM2 pS1	0.641871	0.501075	1.236247	0.80487	0.260199	0.332428	0.912584	0.619556	p03960	Serine/argin	3	0.663604				
SRRM2 pS1	0.266297	0.192941	0.658395	0.638675	-0.18046	-0.04477	0.091666	0.009057	p14637	Serine/argin	3	0.203974				
SRRM2 pS1	-0.46022	0.640979	1.27418	-0.40788	1.078756	1.351225	1.407538	0.934434	p05716	Serine/argin	3	0.727376				
SRRM2 pS1	0.511623	0.891994	1.352643	0.696586	-0.63468	-0.40668	-0.05363	-0.14762	p08757	Serine/argin	3	0.276279				
SRRM2 pS1	0.458954	0.086262	0.705476	0.512511	-0.05944	-0.00969	0.189459	0.321415	p07899	Serine/argin	3	0.275618				
SRRM2 pS1	0.583781	0.174865	0.787227	0.586191	-0.1546	-0.04628	0.224026	0.315741	p07900	Serine/argin	3	0.30887				
SRRM1 seq	1.351486	1.275372	1.266933	0.882909	0.848727	1.172365	1.337175	1.15458	p06733	Serine/argin	3	1.161194				
SRRM1 seq	0.834621	1.08263	0.514504	0.42448	0.624208	0.967216	1.912345	0.804198	p06744	Serine/argin	3	0.895525				
SRRM1 pT8	0.326848	0.536366	0.199461	0.502855	0.187313	0.22482	0.441617	0.328961	p13277	Serine/argin	3	0.34353				
SRRM1 pT5	0.162077	0.089227	0.44104	0.317424	-0.04265	-0.11829	-0.01561	0.084275	p06709	Serine/argin	3	0.114687	mTOR;			
SRRM1 pS8	0.39096	0.427657	0.675896	0.493511	0.037262	-0.0061	0.055145	0.208944	p13275	Serine/argin	3	0.28541				
SRRM1 pS8	0.488977	0.448161	0.786202	0.503453	0.080726	0.006969	0.013419	0.272073	p13274	Serine/argin	3	0.324997				
SRRM1 pS7	0.246664	0.600456	0.1328	0.148661	0.025219	0.234449	0.080644	0.183671	p12757	Serine/argin	3	0.20657	CDK2;			
SRRM1 pS7	0.157029	0.358462	0.755938	0.756004	0.030929	-0.51131	-0.41605	-0.15266	p21025	Serine/argin	3	0.122293				
SRRM1 pS6	0.616545	0.687017	0.679544	0.982848	1.543098	1.925043	2.059453	0.880306	p07562	Serine/argin	3	1.171732				
SRRM1 pS5	0.33235	-0.16548	0.488612	0.3218	0.226008	0.306051	0.381183	0.617249	p06726	Serine/argin	3	0.313472				
SRRM1 pS5	1.630413	0.79585	1.410891	1.345289	0.175724	0.254505	0.443556	0.648184	p06727	Serine/argin	3	0.838051				
SRRM1 pS4	0.31948	0.189157	0.579198	0.959705	0.240206	0.398418	0.501792	0.562156	p07462	Serine/argin	3	0.468764				
SRRM1 pS4	0.573605	0.362657	0.545553	1.018568	0.33283	0.421366	0.396535	0.547975	p07463	Serine/argin	3	0.524886				
SRRM1 pS4	0.541784	0.617641	0.859409	0.868982	-0.28982	-0.07303	0.010522	-0.0524	p07460	Serine/argin	3	0.310385				
SRRM1 pS4	0.617373	0.71896	0.820718	0.843035	-0.33564	-0.09172	0.031593	-0.04253	p07461	Serine/argin	3	0.320224				
SRRM1 pS4	0.321117	0.366246	0.553601	0.440178	0.151712	0.269144	0.524377	0.556612	p17135	Serine/argin	3	0.397873				
SRRM1 pS4	-0.23902	0.044041	0.941053	0.366748	0.422831	0.298714	0.949782	0.570616	p00757	Serine/argin	3	0.419346				
SRRM1 pS4	0.276274	0.157226	0.483378	0.188352	0.388246	0.016864	0.032762	-0.09944	p12546	Serine/argin	3	0.180458				
SRRM1 pS3	0.308809	0.110761	0.530672	0.579301	0.043444	0.030867	-0.44436	0.043883	p07093	Serine/argin	3	0.150422				
SRRM1 pS3	0.494029	0.395774	0.949327	1.098045	-0.17369	0.006121	-0.05564	0.165321	p07092	Serine/argin	3	0.359911				
SRP9 seq: 4	-0.3344	0.394308	-0.69699	-0.96704	1.197065	0.356794	0.507678	0.418378	p00741	Signal recog	3	0.109474			IL3-mediated signaling events;	
SRP72 seq:	0.726704	0.612405	1.02733	0.779163	0.285741	0.226422	0.21252	0.410477	p01887	Signal recog	3	0.535095				
SRGAP1 pS	0.723173	0.38522	0.630946	0.658899	0.211264	0.395286	-0.10286	0.197175	p09106	SLIT-ROBO	3	0.387388			Regulation	GO:0007266; P:Rho protein signal transduction; GO:0051056; P:regulation of small GTPase mediated signal transduction; GO:0007 [illegible]
SREK1IP1 s	0.290498	0.306244	-0.08475	-0.61556	-0.19262	-0.15553	0.027788	0.508944	p14602	Protein SRE	3	0.010626				
SRCIN1 pS8	0.694936	0.322409	0.641779	0.658329	0.573825	-1.5729	0.098726	0.067632	p07362	SRC kinase	3	0.185592				
SRA1 seq: 6	-0.00548	1.135304	-0.00373	0.087479	0.027533	0.116261	-0.15519	-0.27262	p01029	Steroid rece	3	0.116194			Validated nuclear estrogen receptor alpha network;	
SQSTM1 se	0.286975	1.119828	-2.93886	0.892907	1.28688	0.672516	0.158452	0.388582	p10509	Sequestoso	3	0.23341			IL1-mediate	GO:0043122; P:regulation of I-kappaB kinase/NF-kappaB signaling; GO:0048011; P:neurotrophin TRK receptor signaling pathway; G [illegible]
SQSTM1 se	0.652662	-0.4531	0.360783	0.19654	0.071255	-0.21921	0.015041	-0.0787	p07435	Sequestoso	3	0.068159			IL1-mediate	GO:0043122; P:regulation of I-kappaB kinase/NF-kappaB signaling; GO:0048011; P:neurotrophin TRK receptor signaling pathway; G [illegible]
SQSTM1 se	2.189312	1.73862	0.720108	0.077524	-0.39255	-0.83966	0.336144	0.728796	p12267	Sequestoso	3	0.569786			IL1-mediate	GO:0043122; P:regulation of I-kappaB kinase/NF-kappaB signaling; GO:0048011; P:neurotrophin TRK receptor signaling pathway; G [illegible]
SPTBN1 pT	0.715998	-0.04338	0.838252	0.688195	-0.09015	-0.65334	0.012019	-0.85572	p03078	Spectrin be	3	0.076484		ERKinh2 (E	TGF-beta receptor signaling;	
SPTBN1 pS	0.599962	-0.29594	1.077894	0.791811	0.344425	-0.04659	0.657307	0.719295	p05425	Spectrin be	3	0.482008		ROCKinh2 (	TGF-beta receptor signaling;	
SPTBN1 pS	0.304524	0.35011	0.589182	0.411586	0.284999	0.478501	0.669541	0.656709	p06883	Spectrin be	3	0.468144			TGF-beta receptor signaling;	
SPTBN1 pS	0.403508	0.432771	0.638354	0.538748	0.225718	0.428899	0.635444	0.612411	p06884	Spectrin be	3	0.489577			TGF-beta receptor signaling;	
SPTAN1 pS	0.415587	-0.38176	0.641612	0.62628	-0.30466	-0.42304	-0.2471	-0.05624	p05080	Spectrin alp	3	0.033834				
SPRY4 pS12	0.564488	1.427619	0.762096	0.864804	0.319635	-0.3062	0.305144	-0.40289	p11452	Protein spr	3	0.441836				
SPEN seq: 2	-4.99377	4.526959	2.792597	2.884517	-0.69127	0.783018	0.239818	0.009265	p01417	Msx2-intera	3	0.693892			Notch-med	GO:0007219; P:Notch signaling pathway;
SPEN seq: 1	0.481321	0.363014	0.215483	0.103749	-0.62285	-0.07951	0.03596	-0.39084	p11151	Msx2-intera	3	0.013292			Notch-med	GO:0007219; P:Notch signaling pathway;
SPEN pS10	0	0	3.681559	2.820883	1.721524	3.049282	2.126744	1.759188	p12537	Msx2-intera	3	1.894898			Notch-med	GO:0007219; P:Notch signaling pathway;
SPECC1 pS	-0.14008	0.729051	-0.12021	0.22099	0.009497	0.023015	-0.10376	-0.11708	p04543	Cytospin-B	3	0.062677				
SPECC1 pS1	0.976044	1.404484	2.12784	2.068811	-0.29132	-0.42509	-0.45834	-0.54326	p03248	Cytospin-B	3	0.607396				
SPDL1 seq:	0.277975	0.757453	0.182235	0.214428	-0.26794	0.562903	1.293663	0.815283	p03275	Protein Spir	3	0.4795				
SPAST seq:	2.243242	2.79916	1.687141	0.962691	-0.12517	-0.40545	0.076007	-0.07704	p19870	Spastin	3	0.895072				
SPAST seq:	0.591009	0.073281	0.048616	-0.03369	0.704097	-0.49292	0.81016	0.636211	p07232	Spastin	3	0.292096				
SPAG9 seq:	0.107587	0.863739	0.384288	0.384308	0.217929	0.407279	0.114074	0.10222	p06235	C-Jun-amin	3	0.322678			Arf6 trafficking events;	
SPAG9 seq:	0.050006	0.45873	0.580337	0.341728	0.204806	0.258192	0.33118	0.405627	p06237	C-Jun-amin	3	0.328826		AKTinh1 (A	Arf6 trafficking events;	
SPAG9 seq:	0.184174	1.151332	-0.07403	0.007069	-0.82664	0.35246	-0.2091	0.083621	p16062	C-Jun-amin	3	0.083611			Arf6 trafficking events;	
SPAG9 pS2	0.658995	0.435907	0.577776	0.551852	0.459819	0.588446	0.560558	0.844779	p17220	C-Jun-amin	3	0.584767			Arf6 trafficking events;	
SP3 pS73	0.500209	0.314949	0.720079	0.287916	0.534731	0.474526	0.246581	0.786974	p14247	Transcriptic	3	0.483246	ERK2; ERK1;		Syndecan-2	GO:0035329; P:hippo signaling; GO:0007267; P:cell-cell signaling; GO:0048011; P:neurotrophin TRK receptor signaling pathway; G [illegible]
SP100 pS3	0	0	0	0	0.118296	0.062881	0.469408	0.312289	p06004	Nuclear aut	3	0.120359				GO:0048384; P:retinoic acid receptor signaling pathway; GO:0060337; P:type I interferon signaling pathway; GO:0060333; P:interfe [illegible]
SOX5 seq: 9	0.065604	0.500651	0.23287	0.418658	0	0	0	0	p01098	Transcriptic	3	0.152223				GO:0071560; P:cellular response to transforming growth factor beta stimulus;
SOX13 seq:	0.181222	-0.1922	-0.03098	-0.01669	0.298913	0.086249	0.523025	0.225704	p09877	Transcriptic	3	0.134405				
SOWAHC se	0.183765	0.391731	0.333386	0.142204	-0.25755	0.210029	-0.04848	0.056678	p18514	Ankyrin rep	3	0.126471				
SOS1 pS11	2.492834	2.54031	2.710707	2.065813	0.757523	0.415776	0.608625	0.882576	p06751	Son of seve	3	1.55927			EGFR-deper	GO:0007265; P:Ras protein signal transduction; GO:0051056; P:regulation of small GTPase mediated signal transduction; GO:0008 [illegible]
SOS1 pS11	0.524203	0.929371	0.118239	0.42564	0.478577	0.117392	0.039183	0.342452	p06752	Son of seve	3	0.378632			EGFR-deper	GO:0007265; P:Ras protein signal transduction; GO:0051056; P:regulation of small GTPase mediated signal transduction; GO:0008 [illegible]
SORBS3 se	-0.27088	0.883371	-0.11207	-0.46318	-0.06802	0.155587	0.194081	-0.01161	p15442	Vinexin	3	0.03841				GO:0043410; P:positive regulation of MAPK cascade;
SORBS1 se	5.526157	0	5.236331	0	-0.00495	-0.1706	0.077536	0.031936	p06199	Sorbin and	3	1.337051			Insulin Path	GO:0009967; P:positive regulation of signal transduction; GO:0008286; P:insulin receptor signaling pathway;
SON seq: 2	-0.57422	-0.32908	1.251456	0.047693	0.352429	-0.01804	0.928744	0.362142	p05853	Protein SON	3	0.252641				
SON seq: 1	0.535474	1.053483	0.671056	0.53961	0.227498	0.313182	0.536788	0.672478	p18753	Protein SON	3	0.568696				
SON pS169	0.338676	0.870841	0.244557	0.349543	0.373389	-0.13298	0.553781	0.261583	p18752	Protein SON	3	0.357424				
SON pS169	3.38215	1.975099	1.725172	1.643661	-0.88431	-0.84146	0.277016	0.992281	p18186	Protein SON	3	1.033702				
SOCS6 pS1	0	0	5.472887	5.480536	0.483645	-1.09335	0.361352	0.141158	p05878	Suppressor	3	1.355778				GO:0009968; P:negative regulation of signal transduction; GO:0007259; P:JAK-STAT cascade;
SNX6 seq: 3	-0.04144	0.131169	-0.09845	-0.29233	0.223615	0.471391	0.559465	0.241672	p04936	Sorting nexi	3	0.149386				GO:0030512; P:negative regulation of transforming growth factor beta receptor signaling pathway;
SNX3 pS72	0.667558	0.34692	0.493746	0.409721	-0.34727	-0.16778	-0.33592	0.25876	p06400	Sorting nexi	3	0.165717				
SNX2 pS27	0.348627	0.418123	-0.34772	0.334765	4.061858	3.238174	4.695898	-5.39312	p19219	Sorting nexi	3	0.919575			PAR1-mediated thrombin signaling events;	
SNX1 pS18	1.346916	0.65336	0.819715	0.781965	0.218796	0.37234	0.445947	0.927721	p07440	Sorting nexi	3	0.695845		PKCinh1 (G	PAR1-mediated thrombin signaling events; Posttranslational regulation of adherens junction stability and dissassembly;	
SNW1 pS2	-0.30294	-0.21265	-0.415	0.954078	0.475669	0.262091	-0.1212	0.111238	p15465	SNW domai	3	0.093911	CDK2;			GO:0048384; P:retinoic acid receptor signaling pathway; GO:0030511; P:positive regulation of transforming growth factor beta rece [illegible]
SNW1 pS2	0.248693	0.445921	0.342997	0.533973	-0.07467	0.179502	-0.01519	-0.13274	p15460	SNW domai	3	0.191061	CDK2;			GO:0048384; P:retinoic acid receptor signaling pathway; GO:0030511; P:positive regulation of transforming growth factor beta rece [illegible]
SNW1 pS2	0.516769	0.457487	0.547281	0.403135	0.042217	0.393053	0.213865	0.111412	p15464	SNW domai	3	0.335652	CDK2;			GO:0048384; P:retinoic acid receptor signaling pathway; GO:0030511; P:positive regulation of transforming growth factor beta rece [illegible]
SNTB2 seq:	0.68423	0.983229	0.072363	-0.00561	0.213726	0.585331	0.540169	0.654074	p04417	Beta-2-synt	3	0.465939				
SNTB2 seq:	0.252578	0.749918	0.197314	-0.07431	-0.26588	0.156723	0.135408	-0.04985	p10418	Beta-2-synt	3	0.137737				
SNRPA1 sec	0.243342	-0.52445	0.521258	0.331301	0.044543	-0.11726	-0.02029	-0.11962	p05341	U2 small nu	3	0.044853				
SNRPA1 sec	0.055989	-0.71612	2.271926	0.142422	0.059272	-0.00692	-0.17992	-0.0497	p05340	U2 small nu	3	0.197119		ROCKinh1 (H-1152);		
SNRNP200	0.243085	0.009444	0.393764	0.658022	-0.26689	-0.0851	0.096466	0.158094	p17858	U5 small nu	3	0.15086				
SND1 seq: 2	0.701183	0.50171	1.932933	2.064181	1.902015	0.93874	2.064127	2.385502	p16777	Staphyloco	3	1.561299			C-MYB transcription factor network;	
SNAP23 pS	-0.1041	0.218703	0.053827	0.099205	0.097391	0.291835	0.20395	0.124302	p01989	Synaptosor	3	0.123139				
SMG7 pS8	1.508944	-1.23107	1.322801	1.212541	0.129991	0.123281	-0.09734	0.192956	p04600	Protein SM	3	0.395263				
SMC4 seq:	0.399701	0.22395	0.57248	0.524513	0.067589	-0.0117	0.272946	0.308694	p07485	Structural n	3	0.294772			Aurora B signaling;	
SMC3 seq:	0.304024	0.688553	0.157557	-0.1711	0.346191	0.176677	0.00499	0.846841	p13907	Structural n	3	0.294217			ATM pathw	GO:0007165; P:signal transduction;
SMARCA5 s	0.316197	0.235269	0.444375	-0.00972	-0.54458	-0.17656	0.111288	-0.21514	p07809	SWI/SNF-rel	3	0.020141				
SLTM pS28	0.54374	0.579619	0.592759	0.482967	0.097981	0.242116	0.32088	0.403099	p18802	SAFB-like tra	3	0.407895				
SLTM pS28	1.649408	1.062844	1.29833	1.465039	0.631518	-1.3238	0.42058	0.240552	p18801	SAFB-like tra	3	0.680558				
SLK seq: 18	0.191056	-0.04363	0.081703	-0.06726	0.14907	0.144099	0.433762	0.334282	p07516	STE20-like s	3	0.152886			Caspase cas	GO:0023014; P:signal transduction by phosphorylation;
SLK pS779	7.798203	0	0	5.333538	-0.05135	-0.85918	-0.4619	-0.47956	p18277	STE20-like s	3	1.409967			Caspase cas	GO:0023014; P:signal transduction by phosphorylation;
SLITRK3 sec	0.229157	0.127659	0.654972	0.599132	0.658776	0.220898	0.077951	0.335079	p03187	SLIT and NT	3	0.362953				
SLC9A4 seq	-0.05458	0.774648	-0.08232	0.4237	0	0	0	0	p07101	Sodium/hyd	3	0.132681				
SLC9A1 pS	0.71564	1.809825	0.262289	0.967773	0.546517	0.56058	0.340049	0.357941	p14241	Sodium/hyd	3	0.695077	p90RSK; Ak	Up with IGF	Signaling mediated by p38-alpha and p38-beta; RhoA signaling pathway; Endothelins; ErbB1 downstream signaling;	
SLC9A1 pS	2.183447	3.685126	1.148169	1.078608	-0.3754	0.032288	0.142793	0.435664	p14240	Sodium/hyd	3	1.041337	p90RSK; Ak	Up with IGF	Signaling mediated by p38-alpha and p38-beta; RhoA signaling pathway; Endothelins; ErbB1 downstream signaling;	
SLC4A2 seq	0	5.999639	0	0	0.299381	0.798723	0.243635	-0.51813	p02137	Anion excha	3	0.852906				
SLC4A1AP	0.821128	1.045471	0.962577	0.687055	0.022235	0.123906	0.308716	0.219167	p12867	Kanadaptin	3	0.523782				
SLC39A3 se	0.287988	0.511621	0.678713	0.60332	0.673538	0.203928	0.055738	0.328454	p06309	Zinc transp	3	0.417912				
SLAIN2 pS3	0.866681	0.895954	0.652139	0.709932	0.01157	-0.21819	0.254977	-0.23878	p10749	SLAIN motif	3	0.366785				
SLAIN2 pS3	2.689128	3.147076	2.176001	1.563814	0	0	0	0	p07308	SLAIN motif	3	1.197002				
SKA3 pS15	0.125871	0.445102	0.091606	0.330309	-0.0101	-0.21451	-0.12212	0.064649	p04463	Spindle and	3	0.088851				
SIPA1L3 sec	-0.02023	0.320968	0.549856	0.420295	0.201024	0.016629	0.039112	-0.23128	p03720	Signal-indu	3	0.162047				GO:0051056; P:regulation of small GTPase mediated signal transduction;
SIPA1L3 sec	0.25553	0.200489	0.740641	0.533513	-0.03612	-0.27118	-0.04163	-0.00603	p02568	Signal-indu	3	0.171903				GO:0051056; P:regulation of small GTPase mediated signal transduction;
SIPA1L3 pS	-0.09786	0.443823	-0.24281	0.108897	0.502213	1.574289	-0.65702	1.050016	p02566	Signal-indu	3	0.335194				GO:0051056; P:regulation of small GTPase mediated signal transduction;
SIPA1L1 sec	0.551671	0.027299	0.281753	0.336569	-0.10229	0.154381	0.271504	0.125714	p16907	Signal-indu	3	0.205825				GO:0048013; P:ephrin receptor signaling pathway;
SIPA1L1 sec	0.504747	-0.02608	0.262327	0.329061	-0.08955	0.175686	0.275302	0.131171	p16908	Signal-indu	3	0.195333				GO:0048013; P:ephrin receptor signaling pathway;
SIPA1L1 sec	-0.30301	0.008453	-0.00652	0.771405	1.254314	1.134916	0.355728	0.656192	p05198	Signal-indu	3	0.483935				GO:0048013; P:ephrin receptor signaling pathway;
SIPA1L1 sec	0.288586	2.186695	0.311695	0.996322	0.603043	0.576875	0.370454	0.606217	p16712	Signal-indu	3	0.742486				GO:0048013; P:ephrin receptor signaling pathway;
SIPA1L1 sec	0.102017	0.620564	1.209313	1.114842	-2.12365	-0.50367	0.224592	-0.46393	p09116	Signal-indu	3	0.022508				GO:0048013; P:ephrin receptor signaling pathway;
SIPA1L1 sec	0	0	0	0	-0.27534	0.512776	0.952987	0.273564	p10708	Signal-indu	3	0.182998				GO:0048013; P:ephrin receptor signaling pathway;
SIPA1 pS55	0	0	5.287988	0	-0.20276	-0.41693	-0.81136	-0.5913	p05697	Signal-indu	3	0.408205			PDGFR-beta	GO:0035556; P:intracellular signal transduction; GO:0007165; P:signal transduction;
SIN3A pT26	0.519384	0.34867	0.515482	0.41907	-0.47967	0.122292	0.044738	-0.27138	p00835	Paired ampl	3	0.152323			Hedgehog signaling events mediated by Gli proteins; Signaling events mediated by HDAC Class I; Regulation of Telomerase; Regulation of nuclear [illegible]	
SHTN1 pS5	0.352725	-0.58165	0.405608	0.589706	0.90353	-0.09796	0.678338	0.565897	p04891	Shootin-1	3	0.352024				
SHTN1 pS5	0.667014	0.131477	0.961427	0.823785	0.359534	-0.15564	0.403004	0.524142	p04890	Shootin-1	3	0.464343				
SHTN1 pS2	1.987851	1.743982	1.926594	1.562107	0.698281	1.871695	2.675811	2.444282	p06798	Shootin-1	3	1.863825		Up with IGF - down with PKCinh2 (Bisindolylmaleimide-1); Up with EGF - down with PKCinh2 (Bisindolylmaleimide-1); Up with IGF - down with PKCinh1 (Go [illegible]		
SHROOM3	2.693591	2.588632	1.615182	2.762685	0.484803	-1.21966	0.236188	0.559382	p01232	Protein Shr	3	1.215101				
SHROOM3	1.64489	1.692718	1.191551	0.575296	0.274864	0.434935	0.605881	0.919668	p04676	Protein Shr	3	0.917475				
SHROOM3	0.195346	-0.55283	0.68632	0.671961	0.204562	-0.21382	-0.01015	0.340397	p19769	Protein Shr	3	0.165224				
SHROOM3	0.073621	-0.21351	0.464407	0.190902	0.106082	-0.32891	0.224171	0.376413	p20938	Protein Shr	3	0.111648				
SHROOM3	1.575307	1.884159	1.393818	1.728694	-0.60628	0.274174	-0.01798	0.600191	p04677	Protein Shr	3	0.85401				
SHROOM2	0.309146	1.109732	0.152439	0.121266	0	0	6.288894	0	p03775	Protein Shr	3	0.997684				
SHROOM2	0.558207	0.742626	0.636603	4.68E-05	0.11702	0	2.159757	0	p14914	Protein Shr	3	0.526783				
SHROOM2	0.564576	0.465858	0.55428	0.69086	0	0	0	0	p07522	Protein Shr	3	0.284447				
SHROOM2	0.57059	0.465858	0.55428	0.69086	0	0	0	0	p07523	Protein Shr	3	0.285198				
SHROOM2	-0.004	-0.49565	-0.06156	0.020977	0.794547	0.842063	0.915087	1.67585	p19761	Protein Shr	3	0.460914				
SHROOM1	0.28748	0.836491	-0.30293	0.270288	-0.07428	-0.20498	-0.23562	-0.15228	p06261	Protein Shr	3	0.053022				
SHROOM1	1.803849	1.663235	1.635544	1.466693	0.316251	-0.11361	-0.10995	0.074941	p09895	Protein Shr	3	0.84212				
SHOC2 pT8	1.134349	0.52193	0.750808	1.011274	1.533034	1.710526	1.616763	1.022014	p06896	Leucine-rich	3	1.162587				GO:0046579; P:positive regulation of Ras protein signal transduction; GO:0007265; P:Ras protein signal transduction; GO:000854 [illegible]
SHC3 pS79	0.766917	0.881418	-1.3358	-0.71566	-0.02934	0.387546	0.155835	0.252281	p10635	SHC-transfo	3	0.0454			Neurotroph	GO:0007169; P:transmembrane receptor protein tyrosine kinase signaling pathway; GO:0007265; P:Ras protein signal transduction [illegible]
SHARPIN p	0.641528	0.929042	0.53676	0.54349	-0.10667	0.047248	-0.09485	-0.0119	p04629	Sharpin	3	0.310581				GO:0043123; P:positive regulation of I-kappaB kinase/NF-kappaB signaling;
SHANK2 pS	0.143232	0.871962	0.040487	0.145667	-0.18997	0.026449	-0.0753	-0.11552	p09917	SH3 and mu	3	0.105876				
SHANK2 pS	0.311871	1.044554	0.1124	0.3312	-0.28035	0.080459	0.038788	-0.08325	p09918	SH3 and mu	3	0.19446				
SHANK2 pS	0.41056	0.231282	0.278847	0.428959	0.454092	0.734939	1.19402	1.011405	p07613	SH3 and mu	3	0.593013				
SHANK2 pS	-0.05792	0.163177	0.875679	0.568207	-0.27951	-0.27449	-0.04076	-0.344	p04691	SH3 and mu	3	0.076297				
SHANK2 pS	0.233163	0.982288	0.542475	0.580611	-0.29433	0.077345	0.126521	-0.07049	p10326	SH3 and mu	3	0.272198				
SH3KBP1 p	-1.7584	1.638397	0.053378	1.129836	-0.09651	-0.36532	-0.28045	-0.26644	p02465	SH3 domair	3	0.006811			Internalizati	GO:0042059; P:negative regulation of epidermal growth factor receptor signaling pathway; GO:0007173; P:epidermal growth factor [illegible]
SH3GL1 pS	0.155954	0.566903	-0.05686	0.300736	-0.03245	-0.36792	-0.332	-0.08765	p14538	Endophilin-	3	0.018338			Signaling ev	GO:0007165; P:signal transduction;
SH3BP5L p	0.662312	0.479274	0.287995	0.610935	-0.07424	0.028047	-0.41093	0.3048	p01981	SH3 domair	3	0.236025				
SH3BP2 pS	0.311051	0.970727	0.579842	0.769857	0.290321	0.462941	-0.31616	0.251326	p04510	SH3 domair	3	0.414988			TCR signalir	GO:0009967; P:positive regulation of signal transduction; GO:0007265; P:Ras protein signal transduction; GO:0007165; P:signal tr [illegible]
SH3BGRL s	0.118395	0.103802	-0.01081	0.058815	-0.42324	0.092285	0.271198	0.075412	p17333	SH3 domair	3	0.035732				GO:0009967; P:positive regulation of signal transduction;
SH3BGRL s	0.193408	-0.26752	0.121652	0.198455	0.326415	0.509121	0.537938	0.894702	p08073	SH3 domair	3	0.314272				GO:0009967; P:positive regulation of signal transduction;
SH3BGR se	0.193408	-0.26767	0.121652	0.198455	0.326415	0.509121	0.537938	0.894702	p08066	SH3 domair	3	0.314252				GO:0009967; P:positive regulation of signal transduction;
SH2D3A se	-0.34796	0.937212	0.148784	0.046594	-0.04495	-0.26683	-0.06828	-0.08655	p02117	SH2 domair	3	0.039751				GO:0009967; P:positive regulation of signal transduction; GO:0007264; P:small GTPase mediated signal transduction;
SH2D3A pS	0.01035	0.095623	0.326252	0.094289	-0.009	0.215834	0.277124	0.172247	p05858	SH2 domair	3	0.14784		AKTinh2 (MK-2206); AK		GO:0009967; P:positive regulation of signal transduction; GO:0007264; P:small GTPase mediated signal transduction;
SGTA seq: 3	2.932109	2.608383	3.58466	2.995041	-0.13951	1.110082	2.077319	0.942912	p03972	Small glutar	3	2.013875				
SGTA pS77	0.51236	0.33842	0.788013	0.471393	0.494542	0.138349	0.308906	0.386955	p17356	Small glutar	3	0.429867				
SGOL1 seq:	0.534964	0.173905	0.274397	0.323528	-0.10616	-0.32045	-0.44177	-0.45236	p07116	Shugoshin-	3	-0.00174			Aurora B signaling; PLK1 signaling events;	
SGOL1 pS5	-0.15316	0.338661	0.399383	-1.05396	-0.17076	0.396919	0.346948	1.108064	p07399	Shugoshin-	3	0.151512	NEK2;	AKTinh1 (A	Aurora B signaling; PLK1 signaling events;	
SGMS2 seq	0.766917	0.881418	-1.3358	-0.71566	-0.02934	0.387546	0.155835	0.252281	p04994	Phosphatid	3	0.0454				
SGK223 sec	0.408856	0.289055	0.70069	-0.02636	-0.29442	-0.26876	-0.17206	-0.28748	p06267	Tyrosine-pr	3	0.04369				
SGK223 pS	0.282618	1.16193	0.447743	0.407935	0.096524	0.0991	0.041828	-0.00296	p00789	Tyrosine-pr	3	0.31684				
SGK223 pS	0.958318	4.367609	1.80967	1.891037	-0.22252	-0.06523	-0.28715	-0.50798	p02676	Tyrosine-pr	3	0.992968				
SFXN1 seq:	-0.26248	0.894555	-0.08504	0.27197	-0.07153	-0.05885	0.007083	-0.07273	p01469	Sideroflexin	3	0.077871				
SFRP2 seq:	0.017133	-0.01135	-0.01738	0.466662	0.213191	0.106236	0.09719	0.084796	p13669	Secreted fri	3	0.119559				GO:0030514; P:negative regulation of BMP signaling pathway; GO:0030178; P:negative regulation of Wnt signaling pathway; GO:00 [illegible]
SFPQ seq: 2	0.058498	0.832572	-0.39551	-0.95511	0.015671	0.403766	1.071014	1.16759	p11851	Splicing fact	3	0.274811				
SFN seq: 61	0.607495	2.268161	0.302008	0.281014	1.928579	1.680886	0.785651	1.549859	p01149	14-3-3 prot	3	1.175457			p38 signalir	GO:0006469; P:negative regulation of protein kinase activity; GO:0007165; P:signal transduction;
SFN seq: 14	0.911543	1.365242	2.237766	2.274478	-0.4418	-0.50083	-0.04266	-0.47158	p06165	14-3-3 prot	3	0.66652			p38 signalir	GO:0006469; P:negative regulation of protein kinase activity; GO:0007165; P:signal transduction;
SF3B3 seq:	2.346798	3.016946	1.178391	1.326843	-0.94624	-1.15625	-2.14387	-0.97256	p20610	Splicing fact	3	0.331258				
SF3B2 pS3	1.882703	2.790038	2.14275	2.653134	-0.12015	0.372357	0.294148	0.163125	p03859	Splicing fact	3	1.272263				
SF3B1 seq:	0.726219	0.740901	0.282137	0.294698	0.154941	-0.01725	0.350227	0.622371	p10580	Splicing fact	3	0.39428	DYRK1B; DY	AKTinh1 (Akt Inhibitor VIII);		
SF3B1 seq:	0.216896	0.517068	0.465797	0.103437	0.585078	1.110421	0.985311	0.726229	p03953	Splicing fact	3	0.58878				
SF3A1 pS3	0.594578	0.403761	0.231291	0.336858	0.131047	0.117289	0.204083	0.029004	p00409	Splicing fact	3	0.255989		AKTinh1 (Akt Inhibitor VIII);		
SF3A1 pS3	0.518482	0.447679	0.267516	0.383127	-0.44777	0.387983	0.808393	0.200979	p00410	Splicing fact	3	0.320798		AKTinh1 (Akt Inhibitor VIII);		
SF3A1 pS3	0.528177	0.613407	0.619227	0.399457	-0.03791	0.211672	0.244958	0.188789	p16765	Splicing fact	3	0.345972		AKTinh1 (Akt Inhibitor VIII);		
SET pS7	0.307093	-0.05798	0.523378	0.28228	-0.07968	-0.04375	0.103625	-0.068	p06790	Protein SET	3	0.120871				
SERPINB5 s	0.365199	0.282814	0.315038	0.71219	-0.08483	-0.06409	0.085474	0.158553	p18100	Serpin B5	3	0.221294			Validated transcriptional targets of TAp63 isoforms; ATF-2 transcription factor network; Direct p53 effectors;	
SERPINB5 s	0.930933	0.610814	0.237211	0.516702	-0.0975	0.054533	0.249646	0.20395	p18099	Serpin B5	3	0.338287			Validated transcriptional targets of TAp63 isoforms; ATF-2 transcription factor network; Direct p53 effectors;	
SERPINB5 s	0	0	0	0	-0.17351	0.897356	0.767068	0.676934	p16197	Serpin B5	3	0.270981			Validated transcriptional targets of TAp63 isoforms; ATF-2 transcription factor network; Direct p53 effectors;	
SERPINB5 s	6.509992	0	0	0	1.478937	0.82591	1.055357	0.929758	p14183	Serpin B5	3	1.349994			Validated transcriptional targets of TAp63 isoforms; ATF-2 transcription factor network; Direct p53 effectors;	
SERBP1 seq	1.984905	1.540055	0.257984	0.907921	-0.05412	-0.10862	0.415142	-0.1743	p06374	Plasminoge	3	0.59612				
SERBP1 pS	0.151152	0.915509	0.226955	0.103721	-0.25477	0.028595	-0.13787	-0.44737	p15970	Plasminoge	3	0.073241		Up with IGF - down with PKCinh2 (Bisindolylmaleimide-1); Up with EGF - down with PKCinh2 (Bisindolylmaleimide-1); PKCinh2 (Bisindolylmaleimide-1);		
SEPT9 seq:	2.594309	2.309631	1.469848	-0.24045	0	0	0	0	p09679	Septin-9	3	0.766667				
SENP1 pS1	0.347186	0.927245	0.599054	0.449989	0.228875	0.361192	0.588973	0.405952	p05868	Sentrin-spe	3	0.488558			Regulation of Androgen receptor activity;	
SEC31A pS	0.646458	0.643407	0.693063	0.513567	0.880611	-0.32576	-0.30076	0.112794	p19812	Protein trar	3	0.353015				GO:0006987; P:activation of signaling protein activity involved in unfolded protein response;
SEC24D sec	0.161235	0.427269	0.340793	0.070586	1.045922	0.900186	1.300642	1.713251	p13015	Protein trar	3	0.744985				
SEC24C seq	1.253778	0.977237	-0.00056	-0.12613	-0.55424	-0.18358	-0.16133	-0.21029	p07191	Protein trar	3	0.12436				
SEC16A seq	-0.06012	1.14636	-0.33955	0.063299	-0.38451	-0.17182	-0.38971	0.871005	p13439	Protein trar	3	0.091869				
SEC16A seq	0.309428	0.588155	0.502251	0.257312	-0.35386	-0.364	-0.23945	-0.18213	p15141	Protein trar	3	0.064713				
SEC16A seq	-0.03072	0.417644	0.279342	0.289517	0.520834	0.502973	0.514578	0.686006	p15143	Protein trar	3	0.397521				
SEC16A seq	0.132302	0.318128	0.369071	0.251259	0.310012	0.213141	0.249835	0.507671	p08205	Protein trar	3	0.293927				
SEC16A pT	0.189517	0.575992	0.374538	0.210659	0.269213	0.382652	0.213839	0.198817	p16858	Protein trar	3	0.301903				
SEC16A pS	-0.26626	0.694649	0.108211	0.071628	-0.10927	0.214932	0.207745	0.09175	p08530	Protein trar	3	0.126674				
SEC16A pS1	0.269693	0.914074	0.494845	0.549278	0.457432	0.336437	0.420256	0.593618	p11327	Protein trar	3	0.504454				

SEC16A pS1	0.29222	0.901537	0.488307	0.554723	0.509131	0.396667	0.424312	0.652952	p11328	Protein trar	3	0.527481
SDCBP seq:	7.163649	0	0	0	0.835136	0.822796	1.040773	0.761315	p10296	Syntenin-1	3	1.327959
SCYL3 pS70	-0.07037	1.278051	-0.19306	-0.07476	-0.28659	0.03159	0.127153	-0.34491	p13344	Protein-ass	3	0.058388
SCRIB seq:	0.565361	0.935598	0.354368	0.548743	0.412508	0.196933	0.070876	0.248387	p12244	Protein scri	3	0.416597
SCRIB seq:	-0.21907	1.175584	0.558047	0.498522	0.106303	0.114495	0.29306	0.063937	p20786	Protein scri	3	0.32386
SCRIB seq:	2.391972	1.885188	1.553228	-1.07254	-0.68288	-0.56944	-0.33032	-1.0782	p15663	Protein scri	3	0.262127
SCRIB pT13	0.204968	1.061941	0.582346	0.551071	0.240815	0.15513	0.271212	0.226679	p20787	Protein scri	3	0.41177
SCRIB pS14	0.315841	0.661995	0.356487	0.352422	0.319211	0.432559	0.07281	0.374463	p20169	Protein scri	3	0.360724
SCRIB pS14	0.323122	0.588166	0.000532	0.243212	-0.0914	0.218606	-0.22085	-0.30658	p10850	Protein scri	3	0.094351
SCRIB pS14	0.431841	0.810611	0.472254	0.560729	0.149027	0.276692	-0.1587	-0.13488	p00924	Protein scri	3	0.300946
SCRIB pS14	-0.15457	0.548764	-0.33918	0.180451	-0.11204	-0.13249	-0.01176	0.005595	p07868	Protein scri	3	-0.0019
SCRIB pS13	0.907631	0.221893	-0.27247	-0.1526	0.377487	-0.10651	0.596841	0.357444	p06433	Protein scri	3	0.241214
SCRIB pS13	0.93511	0.179751	0.386227	0.451521	0.189948	-0.15375	0.098198	0.216971	p06434	Protein scri	3	0.287997
SCRIB pS11	0.531078	0.850414	0.593735	0.447984	0.141226	0.089797	0.291794	0.218433	p18392	Protein scri	3	0.395558
SCEL pS312	0	0	0	5.741612	-0.62298	-0.4527	-0.55471	-0.45782	p15858	Sciellin	3	0.456676
SCAMP3 pS	0.096764	0.909339	0.323571	0.503863	0.258849	0.004625	0.217792	0.049003	p12910	Secretory ca	3	0.295476
SCAF11 pS8	0.387484	0.595765	0.224803	0.525867	0.179673	1.170066	1.024455	0.684745	p12625	Protein SCA	3	0.599107
SCAF1 seq:	0	0	10.5784	0	-0.17582	-0.91534	-0.30038	-0.48209	p13739	Splicing fact	3	1.088096
SCAF1 pS73	0.249138	0.999849	1.164868	1.122083	0.344414	0.283658	0.515365	0.514375	p07456	Splicing fact	3	0.649219
SCAF1 pS73	1.421177	1.650364	1.755742	1.562583	0.372551	0.416743	0.621172	0.613686	p07457	Splicing fact	3	1.051752
SCAF1 pS72	0.940505	0.855548	1.235936	1.032917	0.17651	0.356681	0.79681	0.370879	p15439	Splicing fact	3	0.720723
SBDS seq: 1	0	6.002795	0	0	1.880922	-0.67528	1.84904	2.778106	p18770	Ribosome r	3	1.479448
SAV1 seq: 8	0.42818	0.207596	0.716342	0.452667	0.466182	0.771857	0.681644	0.708076	p17143	Protein salv	3	0.554068
SAV1 pS94	0.416279	0.138104	0.790499	0.564366	0.549332	0.700039	0.550967	0.688109	p17142	Protein salv	3	0.549712
SASH1 seq:	0	0	6.71667	0	0.48543	0.458026	0.267322	0.404549	p05093	SAM and SH	3	1.0415
SARG seq: 5	0.43258	-0.13515	0.771295	0.067003	0.820652	1.117049	0.794823	1.294994	p07069	Specifically	3	0.645406
SARG seq: 4	0	0	0	0	0.168259	0.427546	0.672293	0.280649	p20318	Specifically	3	0.193593
SARG pS56	0.819338	0.493753	1.126348	1.172175	0.608477	0.57603	0.463885	0.635873	p07070	Specifically	3	0.736985
SAR1B seq:	0.013306	0.99103	-0.62469	0.144008	0.536617	1.02042	0.326121	0.19232	p10432	GTP-bindin	3	0.324892
SAP30L seq	0.807347	-0.25362	0.907292	0.573248	-0.22284	0.261749	0.744788	0.416946	p02193	Histone dea	3	0.404364
SAP30BP se	-0.31136	0.189782	-0.405	-0.1111	0.562313	1.245534	0.611501	1.114336	p08963	SAP30-binc	3	0.362001
SAP130 pS	1.121395	1.010717	0.793621	0.471498	-0.4173	-1.28837	-0.57141	-0.3805	p12657	Histone dea	3	0.092458
SAP130 pS	0.250365	0.058396	0.631123	0.324361	-0.2015	0.145363	0.056092	-0.14204	p19543	Histone dea	3	0.140271
SAMHD1 p	0.461048	0.837427	1.084849	0.821462	0.169028	0.053067	0.254376	0.339139	p02328	Deoxynucle	3	0.50255
SAMD4B se	0.399595	0.28044	0.491107	0.803243	-0.10777	-0.01087	0.156802	0.237753	p14962	Protein Sma	3	0.281288
SAMD4B se	0.020174	0.736713	0.005156	0.108316	0.060654	0.110307	0.005658	-0.07208	p21159	Protein Sma	3	0.121862
SAG seq: 19	0.531051	0.977435	0.169615	0.047421	0.458787	-0.08264	0.112654	0.681917	p13067	S-arrestin	3	0.36203
S100A6 seq	-0.30642	0.596306	-0.52064	-0.5152	0.902167	0.667313	0.159631	0.510425	p11027	Protein S10	3	0.186697
S100A16 se	-0.13999	0.098272	-0.28855	-0.22685	0.387248	0.478769	0.679975	0.517068	p17441	Protein S10	3	0.188242
S100A10 se	-0.13216	0.397345	-0.14953	0.043616	0.356427	0.415129	0.68884	0.859371	p17792	Protein S10	3	0.309879
RUVBL1 seq	-0.00084	0.942772	-0.42822	-0.6257	0.771715	0.88437	0.521806	0.557252	p12056	RuvB-like 1	3	0.327894
RTKN pS22	0.766917	0.881418	-1.3358	-0.71566	-0.02934	0.387546	0.155835	0.252281	p10634	Rhotekin	3	0.0454
RSRC2 seq:	0.441056	0.559681	0.715746	0.249101	0.363195	0.351561	0.600764	0.399727	p04997	Arginine/ser	3	0.460104
RSRC2 pS3	0.338826	0.422347	0.787771	0.542327	0.107638	0.062397	0.12123	0.071826	p17268	Arginine/ser	3	0.306795
RSF1 pT130	0.438299	0.762112	0.59315	0.371128	-0.06717	-0.09993	0.053983	-0.07751	p14330	Remodeling	3	0.246758
RSF1 pS473	-0.70581	2.974392	1.482553	3.078287	1.993602	0.410004	3.023216	1.914685	p17816	Remodeling	3	1.771366
RRP1B seq:	1.129752	-0.60854	1.230403	0.991169	1.028778	0.280331	1.274028	1.153764	p01816	Ribosomal	3	0.809961
RRM2 seq:	0.997022	-0.01132	0.564242	0.58268	-0.50368	-0.01152	-0.01521	-0.30933	p01006	Ribonucleo	3	0.16161
RRAGC seq	0.742259	0.811547	0.862888	1.08078	1.068008	1.140381	1.335768	1.894485	p09884	Ras-related	3	1.117014
RPS8 seq: 9	0.510805	0.61356	0.542764	0.500559	4.206242	3.241446	4.670076	-5.43404	p09729	40S ribosor	3	1.106426
RPS7 seq: 1	-0.49576	0.335173	-0.50466	-0.62041	1.230175	0.829865	0.463042	0.382109	p01594	40S ribosor	3	0.202442
RPS6KB1 se	-0.19076	0.643363	-0.56595	-0.15494	0.452059	0.718568	-0.10455	0.450906	p02294	Ribosomal	3	0.156088
RPS6KB1 p	0.321092	0.667914	0.543061	0.458206	-1.68424	2.405428	2.194168	0.878961	p02296	Ribosomal	3	0.723074
RPS6KA4 se	0.4201	1.259195	0.757066	1.041195	0.393537	1.115627	0.896109	0.284484	p03796	Ribosomal	3	0.770914
RPS6KA4 se	1.156597	1.593486	0.902473	1.06305	-0.16846	0.377298	0.442827	0.176459	p11943	Ribosomal	3	0.692966
RPS6KA4 p	0.538917	1.235747	0.817358	1.180452	-0.15326	0.775219	0.664443	0.260813	p07328	Ribosomal	3	0.664962
RPS6KA1 se	0.519609	1.30079	0.685034	0.589838	0.34837	0.322853	0.265339	0.287619	p12941	Ribosomal	3	0.539932
RPS6KA1 se	0.75796	1.007919	0.550368	0.516872	-0.07144	0.057097	-0.1492	-0.09227	p02383	Ribosomal	3	0.322163
RPS6KA1 p	1.155036	2.17134	1.080108	1.049828	0.333431	0.287325	0.511981	0.417467	p02384	Ribosomal	3	0.875815
RPS6KA1 p	0.228288	0.055949	0.036728	-0.08444	0.391475	0.183791	0.975063	0.169604	p16096	Ribosomal	3	0.244558
RPS6KA1 p	0.447094	0.157935	0.286995	-0.1184	0.25926	0.281864	0.765122	0.186291	p16095	Ribosomal	3	0.28327
RPS27 pS27	0.197841	-0.039	0.443617	0.01172	0.11282	0.181822	-0.11638	0.157331	p10366	40S ribosor	3	0.118722
RPS24 seq:	0	5.479421	0	0	0.658141	-2.90612	-2.95531	0.902085	p13239	40S ribosor	3	0.147277
RPS2 seq: 2	0.490322	0.989619	-0.6075	0.732561	1.617442	0.732818	-1.1371	-0.55409	p16235	40S ribosor	3	0.283009
RPS19 seq:	0.2203	0.5311	0.373274	-0.37731	-0.0786	0.131336	-0.37014	-0.06564	p12888	40S ribosor	3	0.045541
RPRD2 pS1	0.268877	1.450187	-0.39554	0.344583	0.119926	1.03844	-0.6041	0.301505	p07047	Regulation	3	0.315485
RPRD2 pS1	0.668326	1.325492	-0.13242	0.445311	0.427278	1.144137	-0.62958	0.59858	p07048	Regulation	3	0.480891
RPRD1B se	0.678352	0.639018	0.73212	0.74584	-0.26112	0.008711	0.399596	0.110353	p10732	Regulation	3	0.381609
RPRD1B pS	0.998031	0.754712	0.739882	0.381336	0.049152	-0.27244	-0.92475	-0.74216	p10729	Regulation	3	0.122971
RPLP2 seq:	-0.26147	0.699401	-0.33607	-0.65205	1.031453	0.725789	0.159194	1.071885	p12169	60S acidic r	3	0.304766
RPLP0P6 p	0.155966	-0.22293	0.185828	0.167805	0.478772	0.591045	0.650427	0.090092	p01646	60S acidic r	3	0.262126
RPL5 seq: 2	0.451393	2.127735	-0.37376	0.134163	-1.52891	0.266537	0.174441	0.467943	p00253	60S ribosor	3	0.214943
RPL34 seq:	1.057303	-0.30612	0.515914	0.038398	-0.81664	-0.15459	0.874719	0.184264	p06369	60S ribosor	3	0.174156
RPL27A seq	-0.08356	0.528889	-0.09276	-0.01502	0.593822	0.09006	0.801005	0.617893	p09214	60S ribosor	3	0.305042
RPL23 seq:	0	2.138557	1.191649	1.358899	-1.48271	-2.76919	-1.1217	-1.79652	p18792	60S ribosor	3	-0.31013
RPL19 pS13	2.246128	0.421395	2.138166	0.42214	0.387583	-2.41172	0.169552	-0.04041	p12177	60S ribosor	3	0.416604
RPL18A pS	-0.02701	0.542475	-0.16487	0.062492	-0.01685	0.634591	0.190355	0.082117	p03901	60S ribosor	3	0.162912
RPL18 seq:	0.287765	0.53742	-0.76324	-0.95049	1.33789	0.560509	0.561411	0.581635	p02994	60S ribosor	3	0.269112
RPL17 seq:	0.471362	0.151875	-0.07776	-0.54603	1.326346	0.570128	0.410482	0.977777	p00104	60S ribosor	3	0.410524
RPL17 seq:	-0.1104	0.501578	-0.10291	-0.06603	-0.23801	-0.17379	0.094538	0.283611	p15766	60S ribosor	3	0.023573
RPIA pS106	0.465187	0.460868	0.264995	-0.0356	-0.02149	0.356916	0.302673	0.151607	p09312	Ribose-5-pl	3	0.243144
ROCK2 seq:	0.32922	0.83164	0.236592	0.599141	-0.0428	0.212609	0.34839	0.255066	p02514	Rho-associ	3	0.346233
ROCK2 seq:	0.827769	3.332332	0.824947	1.213452	-0.20409	0.09366	-0.59789	0.121927	p04240	Rho-associ	3	0.701513
ROCK1 pS1	-0.10192	0.515229	-0.26203	-0.02956	1.025866	0.823129	0.69265	0.958476	p11447	Rho-associ	3	0.452731
RNPS1 pS1	0.727256	0.964144	0.622908	-0.01968	0.432474	0.33889	0.537016	1.473493	p06728	RNA-bindin	3	0.634562
RNPC3 pS1	0.544663	0.507112	0.753426	0.341483	0.061209	0.177542	0.431088	0.267496	p17294	RNA-bindin	3	0.385502
RNF31 pS4	1.778146	-0.6458	-0.05516	0.867146	-0.70173	-0.26167	-0.39151	-0.6784	p07151	E3 ubiquitir	3	-0.01112
RNF31 pS4	0.500273	-0.34089	-0.1051	0.134527	0.722861	0.511246	0.714693	0.528593	p07150	E3 ubiquitir	3	0.333276
RNF214 seq	0.12303	0.814421	0.117636	0.32985	-0.35468	0.420233	0.004925	0.042464	p05061	RING finger	3	0.187234
RNF20 seq:	0.139474	0.310306	-0.04311	-0.04653	0.279932	0.72474	0.241242	0.166642	p05698	E3 ubiquitir	3	0.221587
RNF169 pS	0.042898	0.461836	-0.95688	-0.16795	0.706131	1.610914	0.515812	0.613178	p13148	E3 ubiquitir	3	0.353242
RNF113A se	1.271405	2.896887	3.117281	3.371645	0.248457	0.63946	0.358421	0.323909	p20965	RING finger	3	1.528433
RIPK1 pS32	0.228734	1.187608	-0.05233	0.018102	-0.65493	1.274012	2.119611	1.042438	p09925	Receptor-in	3	0.645406
RIN1 pS351	2.236735	1.532605	2.736358	2.473949	-0.21895	-0.05632	-0.16847	-0.24527	p04883	Ras and Rab	3	1.036329
RICTOR seq	0.341337	0.314437	0.18859	0.258168	0.234287	-0.0519	0.359746	0.344448	p04753	Rapamycin-	3	0.248639
RGS3 seq: 9	0.672696	0.477206	0.490734	0.44605	0.206716	0.182483	0.130198	0.218501	p06595	Regulator o	3	0.353073
RGS12 seq:	0.191056	0.062938	0.21191	0.171046	-0.7309	-0.13494	1.131294	0.573687	p20210	Regulator o	3	0.184511
RGPD8 seq	-0.31925	0.833296	-0.27186	0.127537	-0.19683	0.117955	-0.29947	-0.27055	p03059	RANBP2-lik	3	-0.0349
RGCC seq:	0	0	6.915425	6.465307	0.82606	0.746094	0.333776	0.833614	p09160	Regulator o	3	2.015035
RGCC seq:	0	0	0	0	1.337213	0.927379	0.686452	1.054331	p09161	Regulator o	3	0.500672
RERE pS656	0.513202	0.312084	0.518964	0.110175	0.332239	0.323128	0.655166	0.393435	p01767	Arginine-glu	3	0.394799
REPS1 seq:	0.76031	1.212831	0.187816	0.296282	-0.09019	0.139981	0.119246	0.030053	p02800	RalBP1-ass	3	0.332041
REPS1 seq:	0.738467	0.318695	0.357679	0.385207	0.247755	0.311315	0.244645	0.461743	p06779	RalBP1-ass	3	0.383189
REPS1 seq:	0.839187	0.239477	0.108381	0.463034	0.256345	0.533946	0.525265	0.407816	p13147	RalBP1-ass	3	0.421694
REPS1 pS56	2.72498	3.919066	0.879663	2.853762	0.294825	0.507533	0.542653	0.518247	p03695	RalBP1-ass	3	1.530091
RELT seq: 3	0	0	7.14722	0	-0.59386	-0.00062	-0.96434	-3.74408	p02687	Tumor necr	3	0.23054
RELA seq: 1	0.203024	0.220425	0.086255	0.086905	-0.02985	-0.03326	0.031508	-0.15976	p13782	Transcriptic	3	0.050656
RECQL4 pS	0.842642	0.680582	0.595798	0.776364	0.675662	1.373931	1.21625	0.745771	p21000	ATP-depenc	3	0.863375
RCHY1 pS2	0.800899	-0.02318	0.019185	0.800923	0.108362	0.310502	-0.75102	0.006013	p07250	RING finger	3	0.158961
RBMX2 seq	-0.14063	0.43385	2.009735	0.921265	-0.23744	-0.07832	0.350125	0.17765	p16959	RNA-bindin	3	0.42953
RBMX seq:	0.290364	1.184387	-0.30539	-1.82748	0.055213	0.111961	0.339707	1.012567	p06096	RNA-bindin	3	0.107665
RBMX seq:	0.032306	0.687163	-0.64964	-0.98829	1.197541	0.012839	0.378525	0.310746	p11893	RNA-bindin	3	0.122649
RBM8A pS5	1.165923	0.604685	0.805018	0.602414	0.051575	0.149822	0.405509	0.171596	p09922	RNA-bindin	3	0.494568
RBM7 pS20	0.266569	1.956735	-0.11419	1.097915	0.861914	1.945152	-0.4372	0.870139	p09978	RNA-bindin	3	0.80588
RBM7 pS13	0.609995	1.706497	-0.09569	0.840049	0.869418	1.743836	-0.06513	0.723169	p05817	RNA-bindin	3	0.791517
RBM4B pS8	1.486549	2.092072	-0.33875	-0.99665	-1.1549	-0.41719	-0.31499	-0.3957	p11624	RNA-bindin	3	-0.00495
RBM34 pS2	0.869423	-0.01255	1.074663	0.422772	-0.06682	-0.20407	0.42238	0.030838	p03294	RNA-bindin	3	0.317079
RBM33 pS2	0.036109	0.554787	0.541132	0.357266	0.025281	-0.27212	0.04488	-0.14761	p18746	RNA-bindin	3	0.142465
RBM17 seq	0.233976	0.367611	0.36112	0.352569	0.157853	0.249963	0.286348	0.331454	p04342	Splicing fact	3	0.291987
RBM17 seq	0.25189	0.280673	0.170235	0.145658	-0.0725	-0.00138	0.140835	0.059412	p21123	Splicing fact	3	0.121853
RBM17 pS2	0.339125	0.587783	0.316222	0.32952	-0.01918	0.220354	0.31157	0.337442	p04343	Splicing fact	3	0.302854
RBM17 pS1	0.556867	0.649575	0.838522	0.613314	0.007259	0.078379	0.200644	-0.02026	p06959	Splicing fact	3	0.365537
RBM15B pS	0.438233	0.415004	0.7621	0.797845	0.071872	0.251626	0.182023	0.115089	p19619	Putative RN	3	0.379224
RBM15 seq	0.564648	0.634154	0.288407	0.280579	0.452787	0.316052	0.013111	0.024939	p04033	Putative RN	3	0.321835
RBM14 seq	1.472588	2.068986	0.841836	0.809635	0.865061	0.827676	1.522751	1.496049	p21064	RNA-bindin	3	1.238073
RBM12B se	1.753715	1.62314	2.358733	2.300376	0.357639	0.252369	0.38995	0.96126	p04348	RNA-bindin	3	1.249648
RBM12B pS	0.407788	0.345321	0.279285	0.258008	0.364426	0.284476	0.359408	1.194039	p04470	RNA-bindin	3	0.436594
RBM12 pS4	0.185906	0.437663	0.394484	0.211775	0.033999	0.168339	0.290728	0.119939	p04597	RNA-bindin	3	0.230354
RBBP8NL p	0.001469	0.825004	0.072033	0.030624	-0.17038	-0.24956	-0.36909	-0.44384	p20406	RBBP8 N-te	3	-0.03797
RB1CC1 sec	-0.17747	0.05453	0.124034	0.119819	0.803302	0.573236	0.874699	0.988993	p02043	RB1-inducil	3	0.420143
RB1CC1 sec	0.310727	-0.04991	0.512541	0.404211	0.532972	0.46095	0.452722	0.642682	p03542	RB1-inducil	3	0.408362
RB1 seq: 35	0.96224	0.732203	-0.18525	0.093583	-0.50457	-0.06951	-0.27493	-0.511	p12568	Retinoblast	3	0.030344
RASEF pS37	0.23085	0.560345	0.302567	0.267538	0.4577	0.199775	0.310155	0.54754	p05189	Ras and EF-	3	0.359559
RASAL2 seq	1.066056	0.237334	1.024029	0.839944	-0.057	0.20063	0.07209	0.068605	p17042	Ras GTPase	3	0.431461
RAPH1 seq:	0.518932	0.542098	0.640387	0.41232	-0.18732	-0.1098	-0.16776	-0.37869	p04544	Ras-associa	3	0.158771
RAPH1 seq:	0.333738	1.226677	0.241503	0.547214	-0.19728	-0.03801	-0.1193	0.309923	p10505	Ras-associa	3	0.288058
RAPH1 pS6	0.248782	0.921166	0.234287	0.326277	0.009926	0.088806	0.19468	-0.00788	p10160	Ras-associa	3	0.252005
RANBP2 sec	2.199901	2.621071	0.817777	0.254447	-3.2743	-0.03365	0.007393	0.062489	p14587	E3 SUMO-p	3	0.33189
RANBP2 sec	0.416628	0.827525	0.346612	0.341223	-0.05466	0.076514	0.219179	-0.22579	p05459	E3 SUMO-p	3	0.243403
RANBP2 sec	0.201124	0.842292	0.479482	0.418916	0.154179	0.264584	0.325428	0.161963	p05460	E3 SUMO-p	3	0.355996
RANBP2 sec	2.130925	0.544236	1.215097	1.344348	0.367961	-0.23723	-0.74027	-0.4379	p12642	E3 SUMO-p	3	0.523396
RANBP2 pT	2.80544	2.122891	2.81615	2.586064	-0.25254	-0.13745	0.207588	-0.07515	p16860	E3 SUMO-p	3	1.259124
RANBP2 pS	0.173201	0.485765	0.230537	0.352142	-1.99386	-0.50359	0.513024	1.168465	p01505	E3 SUMO-p	3	0.05321
RAN seq: 14	-0.42142	0.348494	-0.4637	-0.79445	1.016287	0.746671	0.283562	0.357849	p09418	GTP-bindin	3	0.134162
RAN pS135	0.109866	-0.17131	0.004927	0.504458	0.080948	0.0507	-0.14565	-0.12005	p05444	GTP-bindin	3	0.039236
RALY seq: 2	0.351802	0.552694	0.472594	0.413491	0.101111	0.217289	0.208055	0.212579	p02231	RNA-bindin	3	0.316202
RALY seq: 2	0.717123	0.667166	0.456481	0.462681	0.1498	0.153039	0.214533	0.268292	p02232	RNA-bindin	3	0.386139
RALY pT286	0.771638	0.813471	0.85226	0.816828	-0.27308	0.064386	0.113119	0.417161	p02233	RNA-bindin	3	0.446972
RALY pT151	0	0	0	6.205765	0.705562	1.235862	-1.23921	0.68556	p00664	RNA-bindin	3	0.949193
RALGPS2 se	0.541858	0.563947	0.429481	0.381266	0.172114	0.159804	0.127853	0.227904	p12521	Ras-specific	3	0.325528
RALGPS2 se	0.353626	0.667901	0.467843	0.6198	-0.22224	-0.2311	-0.27346	-0.43102	p06370	Ras-specific	3	0.118919
RALGAPA2	0.33397	0.432411	0.142157	0.083516	-0.23058	0.623339	-0.11211	-0.36429	p07106	Ral GTPase-	3	0.113552
RALBP1 pS	0.236936	0.147566	0.41053	0.24302	0.763507	0.563923	0.640299	0.714394	p06532	RalA-bindin	3	0.465022
RALBP1 pS	0.083845	0.180951	0.407815	0.291015	0.288864	0.57018	0.928336	0.512887	p20646	RalA-bindin	3	0.407986
RAI14 seq:	0.817642	1.444975	0.963361	1.165131	-0.47716	-0.43689	-0.35726	-0.24112	p13413	Ankycorbin	3	0.359835
RAI14 pS37	0.827624	1.870647	0.630597	0.944124	0.047885	-0.03837	0.143251	-0.2672	p17420	Ankycorbin	3	0.51982
RAI14 pS28	0.764918	1.483163	0.930146	1.023965	-0.43063	-0.16044	-0.57997	-0.12047	p13412	Ankycorbin	3	0.363835

[illegible]

NOMO1 se	0.945198	1.720374	0.69292	0.191492	-0.81889	-0.01491	0.024111	0.083542	p01397	Nodal mod	3	0.352979		
NOLC1 pS6	0.29251	0.578024	0.104176	-0.82352	-0.06969	-0.02102	-0.08437	0.019098	p01827	Nucleolar a	3	-0.0006		
NOLC1 pS5	1.851783	1.957698	1.728693	1.714917	1.52724	1.893159	2.156321	1.521361	p15213	Nucleolar a	3	1.793896		
NOLC1 pS5	0.622546	0.512067	0.52568	1.387163	0.204053	0.789645	0.918039	0.57638	p01811	Nucleolar a	3	0.691946		
NOL3 pT11	0.404809	0.208417	0.418298	0.402703	0.251954	0.390841	0.568355	0.28442	p03173	Nucleolar p	3	0.366225		
NOC2L pS5	0.944507	0.820889	1.549567	1.692997	0.938447	1.399949	1.711994	1.018019	p04702	Nucleolar c	3	1.259546		Validated transcriptional targets of TAp63 isoforms;
NOC2L pS4	0.285141	0.190939	0.618249	0.236845	-0.12715	-0.1643	0.102001	0.096365	p18013	Nucleolar c	3	0.154761		Validated transcriptional targets of TAp63 isoforms;
NME2 pT7	0.593757	0.39473	0.384493	0.385101	0.239619	1.492149	0.54786	1.653342	p01107	Nucleoside	3	0.711381	CDK1;	Validated ta GO:0007229; P:integrin-mediated signaling pathway;
NMD3 pS4	0.991463	0.783498	1.191738	0.888521	0.699329	0.870609	1.197025	0.792988	p18309	60S riboso	3	0.926897		
NKTR pS46	0.117534	0.136301	0.1656	0.20759	0.502511	0.157222	0.420976	0.400057	p14034	NK-tumor r	3	0.263474		
NKTR pS37	0.162584	0.236759	0.614227	0.493459	0.099758	0.101777	0.03195	0.307543	p19113	NK-tumor r	3	0.256007		
NIPBL seq:	0.088949	0.592723	-0.69834	0.452323	0.135403	0.537404	-1.01243	0.431626	p00890	Nipped-B-li	3	0.065957		
NIPBL seq:	-0.1449	0.396215	-0.71787	0.175791	0.276262	0.263837	1.392642	1.797589	p00889	Nipped-B-li	3	0.429946		
NHSL1 pS1	0.413671	0.844021	0.086959	0.293973	0.092265	0.250519	0.287247	0.007786	p19206	NHS-like pr	3	0.284555		
NFKB1 pS9	0.065417	0.910533	0.096149	0.237685	-0.20288	-0.00835	0.121187	-0.17501	p02005	Nuclear fac	3	0.130591		Alternative I GO:0090263; P:positive regulation of canonical Wnt signaling pathway; GO:0034166; P:toll-like receptor 10 signaling pathway; GO:
NFIL3 pS30	0.472714	0.818659	0.279213	0.36671	-0.75739	-0.60812	-0.58307	-0.5915	p15499	Nuclear fac	3	-0.07535		
NFIC seq: 4	8.597661	0	0	0	-0.1162	0.092728	0.127605	0.104667	p12234	Nuclear fac	3	1.100807		FOXA1 transcription factor network;
NFIC pS339	0.343276	-0.03254	0.376891	0.248546	0.330823	0.119001	0.59246	0.360336	p12505	Nuclear fac	3	0.29235		FOXA1 transcription factor network;
NF1 seq: 24	-0.12497	-0.33455	0.001019	-0.06808	0.659448	0.52011	0.983852	0.919182	p15298	Neurofibro	3	0.319502		Regulation GO:0043409; P:negative regulation of MAPK cascade; GO:0046580; P:negative regulation of Ras protein signal transduction; GO:00
NEURL4 se	0.764811	0.359625	0.555082	0.368273	0.342832	-0.65312	0.167685	0.190348	p20168	Neuralized-	3	0.261943		
NENF seq: 1	2.24179	1.449412	1.361135	-1.17769	-1.06407	-0.78022	-0.09319	-0.64123	p09897	Neudesin	3	0.161992		GO:0043410; P:positive regulation of MAPK cascade;
NELFE seq:	0.717099	0.482007	-0.26917	0.240176	0.260009	1.383204	0.098062	0.2475	p05063	Negative el	3	0.394861		
NELFE seq:	0.220013	0.88104	0.187765	0.550561	0.467345	1.196726	0.258752	0.336266	p05064	Negative el	3	0.512308		
NELFE seq:	0.240392	0.538126	0.208836	0.000618	0.485593	0.986355	0.373388	0.430321	p05482	Negative el	3	0.407954		
NELFA seq:	1.942969	0.629348	0.770662	0.306597	-2.47264	0.059902	-0.19804	-0.44674	p08842	Negative el	3	0.074007		
NEK9 seq: 8	0.18093	0.51365	0.098847	0.109909	-0.17274	0.06983	-0.17775	-0.21852	p01766	Serine/thre	3	0.050519		
NEDD4L se	2.073231	0.834059	1.664941	1.730434	0.125045	-0.55538	-0.18487	-0.26941	p11400	E3 ubiquiti	3	0.677257		TGF-beta re GO:0030512; P:negative regulation of transforming growth factor beta receptor signaling pathway; GO:0007179; P:transforming gro
NEDD4L se	0.266109	0.136487	0.351034	0.339823	0.058285	0.710869	-0.03284	-0.0627	p18168	E3 ubiquiti	3	0.220885		TGF-beta re GO:0030512; P:negative regulation of transforming growth factor beta receptor signaling pathway; GO:0007179; P:transforming gro
NEDD4L se	0.586274	0.419659	0.503004	0.873042	-0.00238	0.758561	0.019003	-0.08518	p18167	E3 ubiquiti	3	0.383998		TGF-beta re GO:0030512; P:negative regulation of transforming growth factor beta receptor signaling pathway; GO:0007179; P:transforming gro
NEDD4L se	0.574138	0.145898	0.320975	0.209895	0.188391	0.20792	0.002152	-0.06419	p19233	E3 ubiquiti	3	0.198147		TGF-beta re GO:0030512; P:negative regulation of transforming growth factor beta receptor signaling pathway; GO:0007179; P:transforming gro
NEDD4 seq	0.591627	-0.59999	0.420208	0.450167	1.130396	-0.35305	0.661315	0.702411	p10546	E3 ubiquiti	3	0.375386		ErbB4 signa GO:0014068; P:positive regulation of phosphatidylinositol 3-kinase signaling; GO:0019221; P:cytokine-mediated signaling pathway
NECAP2 se	0.471271	0.404921	0.698248	0.684544	0.388481	0.291385	0.341013	0.477956	p00897	Adaptin ear	3	0.469727		
NECAP2 se	1.509077	1.931474	1.898624	1.909792	0.409996	0.333131	0.557421	0.694779	p00898	Adaptin ear	3	1.155537		
NEB seq: 48	-0.09917	1.292437	0.083324	-0.59375	1.524832	1.695825	-0.94377	1.224841	p14873	Nebulin	3	0.523072		
NDRG2 seq	1.09395	-4.786	3.841708	1.909437	0	0	0	0	p03027	Protein ND	3	0.257387	SGK1; p90RSK; p70S6K;	Validated ta GO:0070373; P:negative regulation of ERK1 and ERK2 cascade; GO:0016055; P:Wnt signaling pathway;
NDRG2 seq	0.306262	2.141202	-0.23012	0.467139	0.095617	0.427627	0.030349	0.002285	p09547	Protein ND	3	0.405044		Validated ta GO:0070373; P:negative regulation of ERK1 and ERK2 cascade; GO:0016055; P:Wnt signaling pathway;
NDE1 seq:	0.412067	0.251825	0.234472	0.349004	-0.08855	-0.25423	0.249018	0.033841	p18578	Nuclear dis	3	0.14843		
NDE1 seq:	0.867305	-1.03856	0.073563	0.675332	0.814756	0.264118	-0.15961	0.963724	p06840	Nuclear dis	3	0.307579	PKACA;	
NDE1 pS30	0.438473	-0.83833	-0.29872	0.126632	0.510752	0.521588	-0.10367	0.766211	p06841	Nuclear dis	3	0.140367	PKACA;	
NCOR2 seq	0.011489	0.131038	0.138106	0.145426	0	0	6.870172	0	p04230	Nuclear rec	3	0.912029		RXR and RA GO:0007219; P:Notch signaling pathway; GO:0007179; P:transforming growth factor beta receptor signaling pathway;
NCOR2 pS2	0.706429	0.374387	0.07461	0.49388	0.667338	0.388488	-0.00596	1.09582	p05204	Nuclear rec	3	0.474374		PI3Kinh2 (P RXR and RA GO:0007219; P:Notch signaling pathway; GO:0007179; P:transforming growth factor beta receptor signaling pathway;
NCOR1 seq	1.070074	1.176708	1.580903	1.798468	-0.18036	-1.24707	-0.93314	-1.63064	p13511	Nuclear rec	3	0.204367		ErbB4 signa GO:0014067; P:negative regulation of phosphatidylinositol 3-kinase signaling; GO:0007219; P:Notch signaling pathway; GO:00071
NCOA3 seq	0.586677	0.604754	0.490217	0.539173	0.128386	0.324645	0.233935	0.296964	p00812	Nuclear rec	3	0.400594		Validated n GO:0030521; P:androgen receptor signaling pathway;
NCOA3 seq	0.586677	0.604754	0.490217	0.539173	0.128386	0.324645	0.233935	0.296964	p00813	Nuclear rec	3	0.400594		Validated n GO:0030521; P:androgen receptor signaling pathway;
NCOA2 seq	0.147865	0.339439	0.022105	0.213247	0.49805	1.595577	0.872335	0.482084	p11937	Nuclear rec	3	0.521338		Validated nuclear estrogen receptor beta network; Retinoic acid receptors-mediated signaling; Regulation of Androgen receptor activity; Coregulat
NCOA2 pS5	0.275254	0.426174	0.470739	0.350735	0.243916	0.600912	0.344722	0.203916	p10106	Nuclear rec	3	0.364546		Validated nuclear estrogen receptor beta network; Retinoic acid receptors-mediated signaling; Regulation of Androgen receptor activity; Coregulat
NCOA2 pS5	0.240981	0.748206	0.629804	0.557274	-0.00137	0.718765	0.3637	0.296753	p10107	Nuclear rec	3	0.444264		Validated nuclear estrogen receptor beta network; Retinoic acid receptors-mediated signaling; Regulation of Androgen receptor activity; Coregulat
NCL seq: 52	0.257617	0.944013	0.305477	-0.15267	-0.96423	0.22773	0.049696	-0.00211	p14941	Nucleolin	3	0.083191		Aurora B signaling; Urokinase-type plasminogen activator (uPA) and uPAR-mediated signaling; Regulation of Telomerase; Validated targets of C-M
NCL seq: 33	-0.74593	0.347228	-0.47977	-0.40041	0.647433	0.491306	0.392278	0.531658	p09707	Nucleolin	3	0.097974		Aurora B signaling; Urokinase-type plasminogen activator (uPA) and uPAR-mediated signaling; Regulation of Telomerase; Validated targets of C-M
NCL pT76	1.378856	0.660326	1.832228	1.662597	1.243627	1.652122	1.994051	0.660485	p01736	Nucleolin	3	1.385537	CDK2;	Aurora B signaling; Urokinase-type plasminogen activator (uPA) and uPAR-mediated signaling; Regulation of Telomerase; Validated targets of C-M
NCL pT76	0	3.321047	4.421614	5.151289	-0.14064	0.349659	1.134055	0.937385	p12454	Nucleolin	3	1.896801	CDK2;	Aurora B signaling; Urokinase-type plasminogen activator (uPA) and uPAR-mediated signaling; Regulation of Telomerase; Validated targets of C-M
NCBP1 seq	1.921388	2.962648	1.327463	1.581233	0.329605	0.449069	0.373631	0.431955	p12472	Nuclear cap	3	1.172124	p70S6K;	Up with IGF - down with PKCinh2 (Bisindolylmaleimide-1); Up with EGF - down with PKCinh2 (Bisindolylmaleimide-1); Up with IGF - down with PKCinh1 (Go
NCAPH pT4	0.271236	0.203045	0.716037	0.389338	-0.26787	0.373309	0.163152	-0.49386	p13414	Condensin	3	0.169299	CDK2;	PKCinh1 (G IL2 signaling GO:0045737; P:positive regulation of cyclin-dependent protein kinase activity;
NCAPG pS6	0.401488	0.406954	0.631731	0.33728	0.271501	0.183134	0.252811	0.259393	p02605	Condensin	3	0.343037		IL2 signaling GO:0045737; P:positive regulation of cyclin-dependent protein kinase activity; GO:0007165; P:signal transduction;
NCAPG pS6	0.362043	0.344548	0.535341	0.309579	0.363443	0.240861	0.335948	0.336182	p02606	Condensin	3	0.353493		IL2 signaling GO:0045737; P:positive regulation of cyclin-dependent protein kinase activity; GO:0007165; P:signal transduction;
NBR1 seq:	0.7936	-0.3179	0.778207	0.356432	0.720503	0.599049	0.658649	0.508308	p04953	Next to BR	3	0.512106		
NAV3 seq:	1.256782	1.03282	1.959397	1.447476	-2.16442	-0.67463	-0.43051	-0.62495	p08134	Neuron nav	3	0.225246		
NAV2 pS13	0.228976	2.61745	0.100248	0.521118	-0.23924	-0.06277	-0.22109	-0.22212	p00190	Neuron nav	3	0.340321		
NAV2 pS13	0.201471	1.654883	0.241691	0.417439	-0.07958	0.223641	0.084098	0.185177	p00191	Neuron nav	3	0.366102		
NAPRT pS5	0.435493	0.979871	0.530055	0.256739	-0.00471	-0.30764	-0.11557	0.061773	p11036	Nicotinate	3	0.229501		
NAP1L4 pS	0.382416	0.311223	0.368922	0.287288	0.047693	0.246785	0.21689	0.144948	p17798	Nucleosom	3	0.250771		AKTinh1 (Akt Inhibitor VIII);
NACA seq:	0.118029	-0.10755	0.108006	0.518634	0.179522	0.091721	0.252488	0.263886	p00959	Nascent po	3	0.178092		AKTinh2 (M Integrin-linked kinase signaling;
NAB2 seq:	0.140169	0.935745	0.628011	0.587962	2.228169	0	1.78585	0	p05823	NGFI-A-bin	3	0.788238		
NAB2 pT43	1.281656	1.403619	0.994039	1.339291	5.241317	0	0	0	p10522	NGFI-A-bin	3	1.28249		
NAB2 pS6	0.092185	0.326515	0.565409	0.709082	-4.56862	0.756769	0.829059	-0.00607	p19895	NGFI-A-bin	3	-0.16196		
NAA30 seq	0.541809	1.602416	0.601097	0.619382	0.404324	0.268958	0.391812	0.182603	p11272	N-alpha-ac	3	0.57655		AKTinh1 (Akt Inhibitor VIII);
NAA25 seq	0.171301	-0.38779	0.408732	0.276659	0.29745	0.225566	0.7958	0.668357	p13766	N-alpha-ac	3	0.307009		
NAA10 seq	0.244142	0.153567	0.56156	0.116519	0.191336	0.144063	0.021706	-0.02393	p15787	N-alpha-ac	3	0.17612		Hypoxic and oxygen homeostasis regulation of HIF-1-alpha;
NAA10 pS1	0.581422	-0.46774	0.976715	0.69782	-0.02553	-0.1491	-0.13932	-0.25231	p15566	N-alpha-ac	3	0.152746		Hypoxic and oxygen homeo
N4BP1 pS3	1.159975	0.943157	-0.41028	-0.05318	-1.94841	0.888658	0.362481	0.972756	p08674	NEDD4-bin	3	0.239394		
MYRIP pS2	0.485296	-0.4307	0.874688	0.697127	0	0	0	0	p04302	Rab effecto	3	0.203302		
MYRIP pS2	-0.19689	0.045338	0.27042	0.207055	0	0	0	0	p01830	Rab effecto	3	0.04074		
MYOF pS19	1.441311	0.276513	1.130497	1.089156	0.298964	0.449562	0.282214	0.338303	p14583	Myoferlin	3	0.663315		Signaling events mediated b
MYO9B seq	0.593071	0.74221	0.560114	0.693487	-2.3399	0.057927	-0.13914	-0.11098	p21140	Unconventi	3	0.007099		Regulation GO:0007266;
MYO9B pS1	0.108033	1.021838	0.37687	0.189949	0.012692	0.232391	0.068024	-0.16015	p11790	Unconventi	3	0.231206		Regulation GO:0007266;
MYO9B pS1	-0.24805	1.414117	-0.65427	0.020965	-0.32418	0.254006	0.047532	-0.08183	p13039	Unconventi	3	0.053536		Up with EG Regulation GO:0007266;
MYO9B pS1	-0.35285	2.004795	-0.57763	0.298195	0	0	0	0	p12796	Unconventi	3	0.171563		Up with EG Regulation GO:0007266;
MYO1E seq	0.048114	0.148427	0.05195	-0.07693	0.372889	0.168375	-0.1871	0.272866	p08001	Unconventi	3	0.099823		GO:0048008;
MYO18A se	1.159672	0.77441	0.731485	0.595173	-6.78197	0.4656	0.96067	3.026885	p06428	Unconventi	3	0.116491		
MYL9 seq:	0.134548	0.533464	-0.00019	-0.30763	0.071063	0.664887	0.935004	0.794354	p19329	Myosin reg	3	0.353187		Integrin-linked kinase signal
MYL12B pT	1.240342	1.178544	-0.33786	-0.07171	-0.37479	-0.55829	-0.41811	-0.38886	p17449	Myosin reg	3	0.033658	CK2A1;	
MYH9 seq:	0.543086	2.592519	0.559755	1.279339	-0.45122	-0.21711	-0.19538	-1.00894	p20048	Myosin-9	3	0.387757		GO:0038032;
MYH9 seq:	0.52569	0.953629	0.356896	0.415509	-0.24461	-0.15654	0.057468	-0.0661	p14488	Myosin-9	3	0.230242		GO:0038032;
MYH9 seq:	1.271444	0.863775	0.230613	0.445308	-0.15732	-0.25822	-0.08711	-0.06919	p14489	Myosin-9	3	0.279913		GO:0038032;
MYH9 seq:	2.198326	2.584777	0.386004	0.354856	0.394691	1.010879	0.831971	2.027939	p04907	Myosin-9	3	1.22368		GO:0038032;
MYH9 seq:	0.421034	0.693014	0.231353	0.216909	-0.13078	0.314627	0.369791	0.516025	p18858	Myosin-9	3	0.328997		GO:0038032;
MYH9 seq:	0.620978	1.027093	0.792284	0.44068	-0.21389	0.093539	0.353835	-0.08708	p18857	Myosin-9	3	0.378429		GO:0038032;
MYH9 seq:	0.542144	0.726906	0.339818	0.212643	0.033103	0.415449	0.430166	0.52846	p18859	Myosin-9	3	0.403586		GO:0038032;
MYH14 seq	1.058284	0.723241	0.460773	0.508806	-0.70862	-0.3359	-0.17736	-0.2979	p11712	Myosin-14	3	0.153915		
MYH14 seq	2.191422	2.427997	0.703662	0.817297	6.674662	2.392372	-5.54711	1.336231	p11713	Myosin-14	3	1.374567		
MYH14 pS1	-0.00329	-0.04566	0.071654	0.602918	0.331483	0.270911	0.278509	0.201621	p11998	Myosin-14	3	0.213518		
MYH10 seq	0.56959	0.244905	0.395749	0.308608	0.0594	0.208329	0.423676	0.105667	p06099	Myosin-10	3	0.289491		
MYH10 seq	0.291729	0.563779	0.229594	0.363081	-0.2931	-0.25855	0.070701	-0.05733	p14486	Myosin-10	3	0.113738		
MYH10 pS1	0.526172	0.539799	0.295109	0.628011	0.09497	0.300385	0.622025	0.761714	p08376	Myosin-10	3	0.471023	CK2A1;	PKCinh2 (Bisindolylmaleimide-1);
MYH10 pS1	1.589036	0.833244	0.65412	1.091514	-0.12511	0.455759	0.421824	0.237528	p06813	Myosin-10	3	0.644739	CK2A1;	PKCinh2 (Bisindolylmaleimide-1);
MYC pT58	0.579521	1.292823	0.520716	0.36008	0.074012	1.378168	0.142465	0.067817	p13253	Myc proto-	3	0.55195	mTOR; JNK	Up with IGF C-MYC path GO:0070848;
MVD pS96	0.517934	-0.05789	0.171764	0.221219	0.090877	-0.02568	-0.11895	-0.02963	p07015	Diphospho	3	0.096205		
MVD pS96	0.882454	-0.03275	0.343465	0.420652	0.162923	0.187496	-0.40935	-0.01636	p09092	Diphospho	3	0.192315		
MVD pS96	0.554869	-0.25563	0.125528	0.233682	0.482285	0.302356	-0.11015	0.230726	p09091	Diphospho	3	0.195458		
MTSS1 seq:	0.220721	0.076649	0.162284	0.216646	0.215858	0.054448	-0.04894	0.010225	p02301	Metastasis	3	0.113486		Hedgehog s GO:0007169;
MTMR3 se	1.937246	1.527452	1.20802	1.900182	0	0	0	0	p02206	Myotubula	3	0.821613		AKTinh2 (MK-2206); AK GO:0046856;
MTMR3 pS	0.540316	0.852475	0.456838	0.549707	0.385908	0.589085	0.702878	1.004248	p05260	Myotubula	3	0.635182		GO:0046856;
MTMR3 pS	0.119164	0.223294	0.203378	0.207688	0.512024	1.599266	0.180402	1.954866	p03175	Myotubula	3	0.62501		AKTinh2 (MK-2206); AK GO:0046856;
MTM1 seq:	0.069387	0.676202	-0.23758	0.005914	0.086613	0.244146	0.046964	0.289203	p10828	Myotubula	3	0.147606		GO:0046856;
MTHFD1 se	-0.1295	1.315149	-0.80107	-1.20167	0.518549	0.884692	-0.34433	1.152891	p14304	C-1-tetrahy	3	0.174339		
MTDH pS8	0.834621	1.08263	0.514504	0.42448	0.606869	0.648249	1.185614	1.674823	p06735	Protein LYR	3	0.871474		Validated ta GO:0031663;
MTDH pS2	-0.11725	0.847477	-0.42104	-0.19165	0.056903	0.81682	0.227768	-0.22555	p10643	Protein LYR	3	0.124183		Validated ta GO:0031663;
MTDH pS2	0.64301	1.907649	0.591874	0.615831	0.159176	1.005601	0.445545	-0.04806	p10644	Protein LYR	3	0.665078		Validated ta GO:0031663;
MT2A seq:	0	0	0	0	0.436132	0.569891	0.401631	0.362263	p10210	Metallothio	3	0.22124		Regulation GO:0022400;
MSL3 seq:	-0.08494	-0.05216	0.467772	0.42299	0.150744	0.09628	-0.16736	0.038536	p03660	Male-specif	3	0.108982		
MSL3 pS36	2.038602	1.338037	2.010685	2.35678	0.19205	-0.31301	0.360361	0.256873	p10807	Male-specif	3	1.030047		
MSL1 seq:	2.328605	2.793175	1.68596	2.629259	0.646148	0.465666	0.884403	0.631126	p17119	Male-specif	3	1.508043		
MRPS36 se	0.416531	0.460401	0.496513	0.551567	-0.29481	-0.2273	-0.0512	-0.1459	p15260	28S ribosor	3	0.150726		
MRPS16 se	0.53439	0.392119	0.615318	0.030527	-0.36606	-0.20018	-0.00638	-0.59593	p16972	28S ribosor	3	0.050476		
MRE11A se	0.519576	0.405366	0.437211	0.745527	0.189167	0.374637	1.188514	0.721917	p04210	Double-stra	3	0.57274		BARD1 signaling events; AT
MPZL1 seq	2.093881	2.480673	2.260718	2.668415	0.036702	-0.17766	0.14141	-0.19049	p05967	Myelin prot	3	1.164206	Src;	GO:0007169;
MPST pS3	5.541253	0	8.1614	0	-0.19133	0.237551	-0.53155	-0.61721	p19535	3-mercapto	3	1.575014		
MPRIP seq:	0.275507	0.177714	-0.75529	-0.00843	0.561775	0.386975	0.276238	0.285983	p05364	Myosin pho	3	0.15006		
MPRIP seq:	1.00607	3.800441	-0.36588	1.757639	-0.43241	0.393149	0.323925	0.553214	p02595	Myosin pho	3	0.879519		

HSP90AA1	-0.43182	0.910276	-0.62759	-0.75442	0.731406	0.728343	0.52083	0.864796	p14972	Heat shock	3	0.242728			ErbB recept	GO:0038096; P:Fc-gamma receptor signaling pathway involved in phagocytosis; GO:0007165; P:signal transduction;
HSD17B10	1.28041	1.267742	-0.07099	-0.14613	0	0	4.552534	0	p12974	3-hydroxya	3	0.860446				
HPS4 pS27	-0.30976	1.991985	-1.0885	-0.05503	-0.48115	0.201467	0.20003	0.098082	p05314	Hermansky-	3	0.06964				
HOMER3 p	0.335709	-1.0743	0.170622	0.037481	0.002398	0.586439	0.855398	1.395422	p17725	Homer prot	3	0.288645				
HNRNPUL2	0.944719	0.613375	0.982651	1.183212	1.279783	0.872151	1.541345	1.199498	p09644	Heterogene	3	1.077092				
HNRNPUL2	1.075878	0.916835	1.16066	0.704014	-0.31191	0.157735	0.410083	0.093674	p21174	Heterogene	3	0.525872				
HNRNPUL2	0.584033	0.533493	0.889942	0.407355	0.308889	0.500772	0.413447	0.554214	p05775	Heterogene	3	0.524018				
HNRNPUL2	0.383562	0.246129	0.553917	0.216296	0.222345	0.310692	0.290879	0.183305	p17847	Heterogene	3	0.300891				
HNRNPUL2	0.591743	0.482415	0.743504	0.161253	0.28599	0.544404	0.432053	0.316134	p17848	Heterogene	3	0.444687				
HNRNPUL2	1.473977	1.17309	0.615893	0.803439	0.827857	0.819387	0.754802	-0.48829	p21175	Heterogene	3	0.747519				
HNRNPUL2	0.334625	0.82522	0.937548	0.518299	0.14406	0.189722	0.32317	0.731244	p17850	Heterogene	3	0.500486				
HNRNPUL1	0.478734	0.487579	-0.47745	-0.82902	1.14766	1.83204	-2.42536	1.305207	p09263	Heterogene	3	0.189924				
HNRNPUL1	0.10657	-0.07318	0.213795	0.136451	-0.07179	0.001103	0.032743	-0.06482	p19946	Heterogene	3	0.035109	CDK2;			
HNRNPU p	0.426066	0.429546	0.604441	0.653382	0.360898	0.285175	0.452282	0.288179	p11059	Heterogene	3	0.437496	DNAPK;			
HNRNPM s	-0.16015	2.166501	-0.83656	0.879687	-3.01071	-2.50741	1.768175	-0.56142	p10104	Heterogene	3	-0.28274				
HNRNPM s	-0.34043	0.251924	0.242735	0.364438	-0.03374	-0.01345	0.160251	0.009335	p10100	Heterogene	3	0.080133				
HNRNPM p	0.826714	0.782417	0.904073	1.084193	-0.40397	-0.13116	-0.11575	0.141229	p10133	Heterogene	3	0.385967				
HNRNPM p	0	0	6.650901	0	0	0	0	0	p10136	Heterogene	3	0.831363				
HNRNPK se	1.139828	2.40283	0.718552	0.836161	0.084453	0.492704	0.850445	0.914865	p14145	Heterogene	3	0.92998		Up with IGF - down with		GO:0007165; P:signal transduction;
HNRNPK pS	-0.12018	-0.55987	0.291707	0.307444	0.375047	0.166215	0.241859	0.338003	p15136	Heterogene	3	0.130028		ROCKinh1 (H-1152);		GO:0007165; P:signal transduction;
HNRNPK pS	0.377498	0.395154	0.732237	0.327337	0.099774	0.203395	0.350046	-0.02682	p07493	Heterogene	3	0.307329	ERK1;			GO:0007165; P:signal transduction;
HNRNPK pS	0.489956	0.614233	1.228778	0.934475	0.109544	0.135592	0.317142	0.145693	p18063	Heterogene	3	0.496927	ERK1;			GO:0007165; P:signal transduction;
HNRNPK pS	1.207487	1.559996	0.388497	0.530828	-0.64616	-0.44346	-0.0753	0.433607	p13097	Heterogene	3	0.369437		Up with IGF - down with		GO:0007165; P:signal transduction;
HNRNPK pS	0.998402	2.254241	0.703812	0.849272	0.139136	0.446946	0.843941	0.824388	p14144	Heterogene	3	0.882517		Up with IGF - down with		GO:0007165; P:signal transduction;
HNRNPH2 s	-0.62701	1.207891	-0.22921	-0.75509	0.558456	1.305774	1.089213	0.304362	p18567	Heterogene	3	0.356797				
HNRNPH2 s	0.148385	0.753091	-0.10322	-0.18931	1.214633	0.90704	0.902452	0.823301	p03525	Heterogene	3	0.557046				
HNRNPH2 p	0.067933	-0.02702	0.130189	-0.00776	0.225008	0.249522	0.076612	-0.1558	p14584	Heterogene	3	0.069836	CDK2;			
HNRNPH1 p	0.451201	0.296468	0.675322	0.533269	0.144214	0.161744	0.533109	0.29375	p17689	Heterogene	3	0.386135				
HNRNPDL s	-0.23978	0.609354	-0.45279	-0.55926	1.400129	0.8807	0.519334	0.357986	p18522	Heterogene	3	0.314459				
HNRNPD se	0.531506	0.739549	0.815787	0.76264	0.079176	0.059598	0.189299	0.331239	p14447	Heterogene	3	0.438599	GSK3B;			
HNRNPD se	0.997871	1.168696	1.090192	0.974012	0.009119	0.05047	0.407518	0.314546	p14448	Heterogene	3	0.626553	GSK3B;			
HNRNPD p	0.194286	0.507592	0.419534	0.518942	0.124346	0.218547	0.300293	0.285011	p14290	Heterogene	3	0.321069				
HNRNPCL1	1.224024	1.596316	0.042963	-0.18949	0.964786	1.520324	1.149802	2.11563	p01662	Heterogene	3	1.053044				
HNRNPC se	1.033637	0.78221	-0.79216	-0.68271	0.746611	1.046619	0.953896	1.396457	p10078	Heterogene	3	0.56057			Regulation of Telomerase;	
HNRNPC se	-0.21587	-0.16635	-0.15401	-0.73305	0.503554	0.949269	0.398759	0.933254	p16144	Heterogene	3	0.189445			Regulation of Telomerase;	
HNRNPC se	0.537218	0.916923	0.499934	0.616482	0.325851	0.401524	0.986562	0.889435	p18920	Heterogene	3	0.646741			Regulation of Telomerase;	
HNRNPC se	1.816646	2.290294	2.426952	2.08675	0.005724	0.272037	0.568815	0.78848	p10174	Heterogene	3	1.281962	CK2A1; CK1	MEKinh1 (C	Regulation of Telomerase;	
HNRNPC pS	0.394402	0.607025	0.569421	0.5233	0.266911	0.279539	0.446292	0.635643	p10164	Heterogene	3	0.465317	CK2A1; CK1	MEKinh1 (C	Regulation of Telomerase;	
HNRNPC pS	0.732736	0.265729	0.385735	0.650152	0.357383	0.316529	0.498732	0.653638	p10171	Heterogene	3	0.482579	CK2A1; CK1	MEKinh1 (C	Regulation of Telomerase;	
HNRNPC pS	0.795264	0.78446	0.585006	0.575951	0.244601	0.296078	0.481911	0.812384	p10165	Heterogene	3	0.571957	CK2A1; CK1	MEKinh1 (C	Regulation of Telomerase;	
HNRNPC pS	0.279901	0.627777	0.748498	0.794749	0.167438	0.226893	0.363357	0.552638	p10169	Heterogene	3	0.470156	CK2A1; CK1	MEKinh1 (C	Regulation of Telomerase;	
HNRNPC pS	0.426273	0.783032	0.753755	0.568907	0.125589	-0.11182	-0.03853	-0.05685	p08796	Heterogene	3	0.306295			Regulation of Telomerase;	
HNRNPC pS	1.137129	1.309397	1.399212	1.972684	2.196454	2.968311	2.815623	0.751306	p09710	Heterogene	3	1.818764			Regulation of Telomerase;	
HNRNPC pS	1.151972	1.103317	1.428549	2.086603	2.193252	3.221967	2.990997	0.710119	p09709	Heterogene	3	1.860847			Regulation of Telomerase;	
HNRNPC pN	0.447005	0.557155	0.610736	0.588775	0.254345	0.38187	0.636502	0.806786	p10166	Heterogene	3	0.535397	CK2A1; CK1	MEKinh1 (C	Regulation of Telomerase;	
HNRNPAB p	0.365264	-0.85844	0.641939	0.523764	0.309466	-0.21105	0.027692	-0.09577	p16929	Heterogene	3	0.087858				
HNRNPA3 s	0.933918	2.47913	0.646306	0.272461	0	5.642479	0	0	p11890	Heterogene	3	1.246787				
HNRNPA2B	0.49814	0.487928	0.612029	0.506289	0.33904	0.218963	0.211392	0.012213	p09380	Heterogene	3	0.360749				
HNRNPA2B	-0.28725	1.25722	0.26875	-0.26445	0.242071	0.531171	0.379349	0.935345	p11886	Heterogene	3	0.382776				
HNRNPA2B	-0.41017	0.778961	-0.52484	-0.70057	1.120301	0.859927	0.720188	0.5701	p16120	Heterogene	3	0.301737				
HNRNPA2B	0.163642	0.722132	-0.11557	-0.05541	0.889648	0.089549	0.508559	0.995659	p14390	Heterogene	3	0.399777				
HNRNPA2B	0.38929	2.040959	0.622455	-0.11673	-0.27076	0.218057	0.114193	0.318088	p18049	Heterogene	3	0.414444				
HNRNPA1 s	0.047625	-0.2816	0.367835	0.281072	0.193382	-0.0655	-0.04105	-0.09955	p03886	Heterogene	3	0.050277		ROCKinh1 (	Coregulation of Androgen receptor activity;	
HN1L seq: 6	0.318338	-0.31358	0.584993	0.315614	0.131875	-0.03661	0.190223	-0.00154	p15281	Hematologi	3	0.148663				
HN1 seq: 7	0.946978	0.384707	0.953637	0.853955	0.527559	-0.08425	0.305486	-0.04739	p03897	Hematologi	3	0.480085			Regulation (	GO:0035584; P:calcium-mediated signaling using intracellular calcium source; GO:0007218; P:neuropeptide signaling pathway; GO:0051056; [illegible]
HMMR pS2	0.292091	-0.02303	1.302604	0.945951	0.487749	0.359349	0.506768	0.262015	p16571	Hyaluronan	3	0.516687				
HMGXB4 s	0.685433	0.797024	0.619735	0.600835	0.163558	-0.33243	0.114944	-0.05031	p13031	HMG doma	3	0.324848				GO:0030178; P:negative regulation of Wnt signaling pathway; GO:0016055; P:Wnt signaling pathway;
HMGXB4 p	0.432864	0.591326	0.679618	0.548789	0.025955	0.018212	0.121454	-0.32985	p11575	HMG doma	3	0.261046				GO:0030178; P:negative regulation of Wnt signaling pathway; GO:0016055; P:Wnt signaling pathway;
HMGN1 se	0.807228	1.468337	-0.33733	0.909941	0.246214	0.90962	-0.83811	0.111456	p12394	Non-histon	3	0.409669	CK2A1;			
HMGCR pS	1.10595	0.394652	0.289327	0.840249	0.589391	0.318759	0.757101	0.922111	p05407	3-hydroxy-	3	0.652193		Akt-mTOR-p70S6K-PI3K		GO:0032874; P:positive regulation of stress-activated MAPK cascade; GO:0070374; P:positive regulation of ERK1 and ERK2 cascade;
HMGB3 se	-1.42241	2.206361	0.0535	2.539763	0.084295	0.41274	0.50379	0.35262	p11797	High mobili	3	0.591333				
HMGB2 se	0.182074	0.133136	0.260271	0.177763	0.321418	0.210521	-0.46946	0.229886	p11796	High mobili	3	0.130701				GO:0048015; P:phosphatidylinositol-mediated signaling;
HMGA1 se	0.770464	0.345299	0.733428	0.697298	0.428419	0.236803	0.502741	0.141529	p12998	High mobili	3	0.481998			IL4-mediated signaling events; Validated targets of C-MYC transcriptional activation;	
HMGA1 se	1.006294	0.614587	0.984865	0.778584	0.18182	0.584524	0.78844	0.235656	p12999	High mobili	3	0.646846			IL4-mediated signaling events; Validated targets of C-MYC transcriptional activation;	
HMGA1 pS	-0.03976	-0.00087	0.166343	-0.23129	-0.10335	0.220186	0.281963	0.069289	p13000	High mobili	3	0.045314			IL4-mediated signaling events; Validated targets of C-MYC transcriptional activation;	
HMG20A p	0.315716	0.430036	0.459318	0.310927	0.14498	0.254669	0.179799	-0.04772	p04540	High mobili	3	0.255965				
HLA-B seq:	5.284437	0	6.59949	4.998785	3.436324	2.896768	2.118413	0.410766	p16808	HLA class I	3	3.218123				GO:0060337; P:type I interferon signaling pathway; GO:0060333; P:interferon-gamma-mediated signaling pathway; GO:0019221; P:cytokine-mediat[illegible]
HLA-A seq:	0.40908	0.722895	0.69524	0.822649	-0.06131	-0.30272	-0.00312	-0.10898	p13192	HLA class I	3	0.271718				GO:0060337; P:type I interferon signaling pathway; GO:0060333; P:interferon-gamma-mediated signaling pathway; GO:0019221; P:cytokine-mediat[illegible]
HIVEP1 pS2	0.450544	0.499851	0.2552	0.400631	-0.31666	0.02675	-0.17244	-0.55155	p20520	Zinc finger p	3	0.074041				
HIST3H3 se	0.492703	-0.82125	0.630057	-0.03344	1.085463	0.797701	1.834284	1.14502	p01768	Histone H3	3	0.641317				
HIST1H2AB	0.97165	0.793157	-0.62815	-0.90569	0.17649	0.200803	0.483082	1.236692	p09714	Histone H2	3	0.291003				
HIRIP3 pS1	0.15944	0.455805	0.647248	0.811701	0.062043	0.128807	-0.26509	0.19213	p17818	HIRA-intera	3	0.274011				
HIRIP3 pS1	0.981886	1.338786	1.52554	1.247928	0.850812	0.57334	0.873208	0.127135	p19257	HIRA-intera	3	0.939829				
HIRIP3 pS1	-0.12048	0.801413	-0.85063	0.132881	-0.1621	-0.01825	0.527433	0.240685	p09589	HIRA-intera	3	0.06887				
HGS seq: 2	0.426773	0.616468	-0.03581	0.099458	0.514951	0.53923	0.36919	0.627457	p01719	Hepatocyte	3	0.394715			Internalizati	GO:0042059; P:negative regulation of epidermal growth factor receptor signaling pathway; GO:0007173; P:epidermal growth factor receptor signalin[illegible]
HELZ pS16	0.321922	0.10483	0.280521	0.28121	0.700733	0.000571	0.424709	0.52339	p04512	Probable he	3	0.329736				
HDLBP seq	2.301619	2.84505	-0.57933	0.271519	-0.1024	-0.32488	0.127408	-0.13383	p10190	Vigilin	3	0.550645				
HDGFRP2 p	0.622324	0.398916	0.749635	0.832489	-0.20836	-0.2784	-0.11708	0.015656	p17707	Hepatoma-	3	0.251898				
HDGF seq:	0.473548	0.386838	0.575827	0.444115	-0.19773	0.073034	0.22336	-0.08948	p09053	Hepatoma-	3	0.236188				GO:0006987; P:activation of signaling protein activity involved in unfolded protein response; GO:0007165; P:signal transduction;
HDGF pS16	0.192636	-0.05484	-0.05344	0.05585	0.135388	0.059392	0.091918	-0.01978	p20702	Hepatoma-	3	0.050891				GO:0006987; P:activation of signaling protein activity involved in unfolded protein response; GO:0007165; P:signal transduction;
HDGF pS13	0.439946	0.438374	0.564414	0.380719	0.451712	0.184922	0.377576	0.584621	p15599	Hepatoma-	3	0.427785				GO:0006987; P:activation of signaling protein activity involved in unfolded protein response; GO:0007165; P:signal transduction;
HDGF pS13	0.416512	0.661621	0.806039	0.860424	0.390578	0.28936	0.518532	1.000473	p15601	Hepatoma-	3	0.617943				GO:0006987; P:activation of signaling protein activity involved in unfolded protein response; GO:0007165; P:signal transduction;
HDGF pS13	0.846727	1.321255	1.501689	1.456168	0.234719	0.396937	1.048214	1.731987	p15602	Hepatoma-	3	1.067212				GO:0006987; P:activation of signaling protein activity involved in unfolded protein response; GO:0007165; P:signal transduction;
HDAC5 pS2	0.095143	0.043207	0.228122	0.157876	0.484679	0.208221	-0.02404	-0.69922	p03804	Histone dea	3	0.061749	AurB;		Signaling ev	GO:0007219; P:Notch signaling pathway;
HDAC2 pS4	0.543639	0.456291	0.749237	0.457468	-0.01218	0.07913	0.149102	0.118831	p14536	Histone dea	3	0.317691			Hedgehog s	GO:0090090; P:negative regulation of canonical Wnt signaling pathway; GO:0048011; P:neurotrophin TRK receptor signaling pathway;
HDAC1 pS4	0.487633	0.508996	0.491997	0.648519	0.02938	0.155953	0.221284	0.187135	p14533	Histone dea	3	0.341362	CK2A1;		Sumoylatio	GO:0060766; P:negative regulation of androgen receptor signaling pathway; GO:0007219; P:Notch signaling pathway; GO:0007179; P:transforming [illegible]
HCFC1 seq:	0.444553	0.278533	0.524901	0.358226	-0.17164	0.000998	-0.06438	-0.28462	p01749	Host cell fa	3	0.135821				
HCFC1 seq:	0.132588	0.371498	0.805994	0.572273	-0.23465	-0.13362	0.179238	-0.22631	p06899	Host cell fa	3	0.183377				
HCFC1 seq:	6.538367	0	0	0	-1.1787	0.267071	-0.6703	-1.20666	p01971	Host cell fa	3	0.468723				
HCFC1 pS6	-0.01617	1.025497	0.219176	0.059095	-0.44743	0.035741	-0.05051	-0.38844	p02685	Host cell fa	3	0.054619		ERKinh2 (ERK inhibitor II);		
HCFC1 pS6	0.202662	0.975892	0.560611	0.546445	-0.20905	0.085505	-0.14591	-0.30381	p04574	Host cell fa	3	0.214042		ERKinh2 (ERK inhibitor II);		
HCFC1 pS1	0.383684	-0.09308	1.021835	0.821418	0.703941	0.58794	0.676132	0.792578	p04731	Host cell fa	3	0.611806		ERKinh1 (ERK inhbitor Calbiochem);		
H1FX seq: 2	0.660081	0.702972	0.866023	0.764854	1.079218	1.171715	1.781824	0.894094	p20653	Histone H1	3	0.990098				
H1FX pS31	0.635833	-0.26075	0.585514	0.622904	0.863067	1.003614	1.471505	0.875358	p20654	Histone H1	3	0.72463				
GTF3C2 se	0.382255	0.550143	0.51124	0.2626	-0.10085	0.230263	0.065206	0.022148	p20535	General tra	3	0.240376				
GTF3C2 se	0.432979	0.632915	0.557876	0.361448	-0.16904	0.234158	0.036542	-0.01625	p20536	General tra	3	0.258827				
GTF2I seq:	0.557935	-0.15901	0.979683	0.807843	-2.14507	0.100161	-0.1038	0.020794	p13894	General tra	3	0.007318		ERKinh1 (ERK inhbitor C		GO:0007165; P:signal transduction;
GTF2F1 seq	0.325608	-0.17114	0.106396	0.112927	0.798531	0.335578	0.577333	0.822718	p03481	General tra	3	0.363494	CDK2;			
GTF2F1 seq	0.795623	-0.65372	1.133144	1.336789	0.887306	0.250807	0.719978	0.855982	p10888	General tra	3	0.665739				
GTF2F1 pS	0.629333	-0.67953	0.872381	0.698216	0.510454	-0.08569	0.003919	0.22203	p10889	General tra	3	0.27139				
GTF2F1 pS	0.872493	-0.56651	1.401002	1.479912	1.027804	0.247154	0.58753	0.668057	p12029	General tra	3	0.71468				
GSE1 seq: 1	1.040184	0.741675	0.901492	0.652592	1.324771	0.982334	1.574229	0.209378	p13175	Genetic sup	3	0.928332				
GSE1 pS82	-0.2724	-0.20591	-0.02316	0.006986	0.615403	0.308831	0.453844	0.464965	p17192	Genetic sup	3	0.16857				
GSDMD se	2.259504	3.338126	1.188556	1.647036	-0.04994	-0.67745	-0.54653	-0.27775	p03466	Gasdermin-	3	0.860194				
GRIPAP1 p	0.62718	0.542721	0.709284	0.839585	0.546113	1.08434	0.758673	0.671087	p05729	GRIP1-asso	3	0.722373		CAMKinh2 (KN-62);		
GRB7 seq:	1.242052	0.642586	0.307777	1.296466	0	0	6.640432	0	p07541	Growth fact	3	1.266164			Signaling ev	GO:0009967; P:positive regulation of signal transduction; GO:0007173; P:epidermal growth factor receptor signaling pathway;
GRB7 seq:	0.070368	-0.32685	0.144471	-0.01004	0.451792	-0.25505	0.590827	0.741003	p06272	Growth fact	3	0.175815			Signaling ev	GO:0009967; P:positive regulation of signal transduction; GO:0007173; P:epidermal growth factor receptor signaling pathway;
[illegible]																

CTNNB1 pS	-0.09742	0.580693	0.261863	0.041459	-0.59983	-0.19068	0.026751	0.402235	p07072	Catenin bet	3	0.053134	PKACA; PAK4;		E-cadherin : GO:0030521; P:androgen receptor signaling pathway; GO:0071363; P:cellular response to growth factor stimulus; GO:0043410; P:p
CTNNB1 pS	0.36645	0.862254	0.199645	0.349211	0.264014	0.407736	0.123722	0.271627	p04459	Catenin bet	3	0.355582	CDK5;		E-cadherin : GO:0030521; P:androgen receptor signaling pathway; GO:0071363; P:cellular response to growth factor stimulus; GO:0043410; P:p
CTNNA2 se	0.269729	0.515324	0.261146	0.226971	0.232633	0.059346	-0.24428	-0.09705	p03955	Catenin alp	3	0.152978			
CTNNA2 se	0.358917	0.626604	0.220872	0.22011	0.257795	0.203263	-0.11155	-0.04964	p03956	Catenin alp	3	0.215797			
CTNNA1 se	0.66865	0.608361	0.366462	0.53457	0.222956	0.34193	0.666108	0.552825	p03958	Catenin alp	3	0.495233			E-cadherin : GO:0045880; P:positive regulation of smoothened signaling pathway;
CTBP2 seq:	1.055597	0.718218	0.353528	0.466965	0.084065	0.225396	0.429582	1.015383	p00181	C-terminal-l	3	0.543592		AKTinh1 (Akt Inhibitor VIII);	
CTAGE5 seq	1.021135	1.014842	1.008882	1.402066	-0.03646	0.059167	0.314035	0.226218	p19923	cTAGE famil	3	0.626235			
CTAGE5 seq	0.388991	0.190208	1.154216	0.934072	-0.36705	-0.58362	0.13988	-0.42807	p17644	cTAGE famil	3	0.178578			
CTAGE5 seq	0.388627	0.219498	1.212522	0.951574	-0.43249	-0.63743	0.029844	-0.24737	p17646	cTAGE famil	3	0.185597			
CSTF3 pS69	0.454228	0.954974	0.370322	-0.14021	-0.09557	-0.08	0.295056	-0.01017	p06898	Cleavage sti	3	0.218579		Up with IGF - down with PKCinh2 (Bisindolylmaleimide-1); PKCinh2 (Bisindolylmaleimide-1);	
CSTF2T seq:	0.127462	-0.01319	0.346193	0.620911	0	0	6.23727	0	p08648	Cleavage sti	3	0.914831			
CSPP1 pS92	0.700166	0.707995	0.507259	1.14069	0.580025	0.673459	0.820233	0.76633	p19760	Centrosom	3	0.73702			
CSPP1 pS52	1.844442	2.677638	2.05645	2.789935	0.366174	0.621795	0.383662	0.500341	p05718	Centrosom	3	1.405055			
CSNK1E seq	0.361082	0.618887	0.587678	0.299035	0	0	5.379088	0	p13886	Casein kinas	3	0.905721	CK1E;		Circadian rh GO:0030178; P:negative regulation of Wnt signaling pathway; GO:0090263; P:positive regulation of canonical Wnt signaling pathwa
CRYBG3 pS	0.490723	0.877189	-0.31792	0.693672	-0.46464	-0.31752	-0.42807	-0.55593	p05596	Very large A	3	-0.00281			
CRTC3 seq:	0.858944	1.109908	0.589803	0.781855	-0.02437	-0.00501	0.09576	-0.15242	p04236	CREB-regula	3	0.40681			
CRTC3 seq:	0.853966	1.10493	0.584825	0.781101	-0.02437	-0.00681	0.09576	-0.14924	p04237	CREB-regula	3	0.405021			
CRTC3 seq:	0.914595	1.095827	0.576467	0.796661	0.403575	0.456964	0.527959	0.175067	p04238	CREB-regula	3	0.618389			
CRTC3 seq:	0.269476	0.854182	0.660939	0.627402	0.073936	-0.13255	0.13394	0.435832	p06809	CREB-regula	3	0.365395			
CRTC3 pS37	0.465057	0.800609	0.270135	0.122174	-0.20829	-0.17333	-0.33985	-0.44156	p11860	CREB-regula	3	0.061868			
CRTC2 pS43	1.601793	2.941459	1.220007	1.072059	-0.09063	0.10151	0.524593	0.123228	p06457	CREB-regula	3	0.936752	CDK2;		LKB1 signaling events;
CRTC1 seq:	0.390399	0.494827	0.590051	0.518719	-0.1009	-0.07867	-0.01029	0.125316	p02468	CREB-regula	3	0.241181		ROCKinh1 (	AP-1 transcription factor network;
CRKL seq: 1	0.007281	-0.00101	0.30249	0.141502	0.247875	0.243032	0.353978	0.651332	p09128	Crk-like pro	3	0.24331	Bcr/Abl; Abl;		JNK signalir GO:0000186; P:activation of MAPKK activity; GO:0009967; P:positive regulation of signal transduction; GO:0007265; P:Ras protein
CRK seq: 19	-0.24397	2.157626	1.195295	1.496764	0	0	0	0	p00141	Adapter mo	3	0.575715	Arg; Abl;		JNK signalir GO:0048013; P:ephrin receptor signaling pathway; GO:0000186; P:activation of MAPKK activity; GO:0009967; P:positive regulation
CREBBP seq	0.397586	0.225388	1.16923	0.56173	0.454922	2.80329	-6.37285	2.285797	p10119	CREB-bindi	3	0.190637			Signaling ev GO:0008589; P:regulation of smoothened signaling pathway; GO:0007219; P:Notch signaling pathway; GO:0008543; P:fibroblast g
CREB1 seq:	0.417401	0.535388	-0.07382	-0.10702	0.717587	0.935231	-0.14819	0.41709	p13088	Cyclic AMP-	3	0.336708			LPA4-media GO:0051403; P:stress-activated MAPK cascade; GO:0034166; P:toll-like receptor 10 signaling pathway; GO:0034146; P:toll-like rece
CPSF7 seq:	0.842158	0.461599	0.628834	1.229714	0.309046	0.543354	0.601015	0.68443	p19341	Cleavage an	3	0.662519			
CPSF6 seq:	0.843757	0.947502	0.442411	0.465172	-0.1468	-0.04379	0.716418	0.377234	p02375	Cleavage an	3	0.450238			
CPSF6 seq:	0.565243	0.584611	0.098841	0.169009	-0.9406	-0.27147	-0.14966	0.199665	p17367	Cleavage an	3	0.031955			
COTL1 seq:	5.897281	0	0	0	1.99657	1.689328	1.309954	1.936155	p17513	Coactosin-l	3	1.603661			
CORO7 pS2	0.678249	0.067646	0.322931	0.353501	-2.19257	0.995003	1.620872	0.392058	p07459	Coronin-7	3	0.279712			
COMT pM1	0.268009	0.794615	-0.76058	-1.18261	0.842566	0.454039	0.324561	-0.34722	p09834	Catechol O-	3	0.049173			
COL21A1 s	-0.01395	0.856765	0.073381	0.14832	0.14388	0.182469	0.046914	-0.01131	p13808	Collagen alp	3	0.178308			
COG1 seq:	1.883294	1.880204	1.645977	1.343738	2.135033	1.789917	2.245116	2.459847	p17448	Conserved	3	1.922891			
COBL seq: 5	0.583553	0.501629	0.169796	0.191818	0.192686	-0.00675	0.21793	0.059476	p09325	Protein cor	3	0.238767			
COBL pS34	0.12601	0.283371	0.116483	0.316857	-1.61421	0.600343	0.604532	1.245636	p07619	Protein cor	3	0.209878			
COBL pS28	0.61599	-1.00098	0.467255	0.549339	0.332956	-0.2581	0.807492	0.552096	p04952	Protein cor	3	0.258256			
CNOT4 pS4	0.333684	0.61231	0.405608	0.405094	-0.09168	0.113307	0.003382	0.025739	p17390	CCR4-NOT	3	0.225931			
CNOT2 seq:	0.283789	0.566767	0.541939	0.578504	1.373773	1.330948	1.429717	1.166163	p03783	CCR4-NOT	3	0.90895			GO:0033147; P:negative regulation of intracellular estrogen receptor signaling pathway;
CNOT2 pS1	-0.05994	0.060332	0.392381	0.407316	0.184396	0.07605	-0.16103	0.155557	p02457	CCR4-NOT	3	0.131883			GO:0033147; P:negative regulation of intracellular estrogen receptor signaling pathway;
CLTC pS149	0.619678	-3.17831	0.63882	-0.07275	0	0	4.882577	0	p02176	Clathrin hea	3	0.361252		CAMKinh1	FAS (CD95) signaling pathway; Arf6 trafficking events;
CLSPN pS1	0.363145	0.346804	0.107119	0.856082	-0.29149	-0.40013	-0.16763	-0.12482	p09620	Claspin	3	0.086135		Up with IGF	ATR signalir GO:0032147; P:activation of protein kinase activity;
CLMN seq:	0.513561	0.525408	0.315147	0.484975	-0.19835	-0.1127	0.107707	-0.02917	p17346	Calmin	3	0.200821			
CLK4 seq: 3	-0.18945	0.041161	0.001413	-0.10706	-0.3994	-0.45449	1.986132	-0.28833	p00652	Dual specifi	3	0.073749			
CLK1 seq: 1	1.770941	1.641411	2.61479	1.239701	1.75922	1.292435	4.094762	1.628401	p02208	Dual specifi	3	2.005208			
CLK1 pS14	0.382199	0.339494	1.063025	0.100256	0.511105	0.310834	1.599506	0.421183	p03372	Dual specifi	3	0.59095			
CLIP2 seq:	1.125266	0.571607	0.262829	0.519085	-0.01856	-0.05161	-0.24537	-0.48787	p13070	CAP-Gly do	3	0.209423			
CLIP2 pS34	1.423909	1.465818	1.200172	1.249251	0.27181	0.194145	0.062434	0.119524	p13071	CAP-Gly do	3	0.748383		p70S6Kinh1 (PF-4708671);	
CLIC1 seq:	-0.00903	0.873014	-0.10978	-0.96141	1.126373	0.788154	0.204375	1.062486	p14981	Chloride int	3	0.371773			GO:0007165; P:signal transduction;
CLDN12 se	0.403319	-0.22225	0.15039	0.401333	0.813592	0.024041	1.263741	1.052071	p04101	Claudin-12	3	0.48578			
CLCC1 pS43	-0.11272	0.35234	-0.166	0.284655	0.177376	0.147267	0.072369	-0.2838	p16508	Chloride ch	3	0.058936			
CLCC1 pS43	0.135857	0.502358	0.395685	0.237058	0.653133	0.770833	1.015467	1.307885	p16509	Chloride ch	3	0.627285			
CLASP2 pS5	0.09306	0.320039	0.130018	0.217526	0.839756	1.103239	0.582645	0.911136	p13861	CLIP-associ	3	0.524677			
CLASP1 seq	1.229647	-0.12733	-3.08407	2.516443	0.519051	-0.01533	-0.06032	0.35094	p06783	CLIP-associ	3	0.166129			
CIT seq: 363	2.918639	1.928061	2.287841	2.143952	-0.26349	0.187954	0.463723	0.253564	p14388	Citron Rho-	3	1.240031			GO:0035556; P:intracellular signal transduction;
CIRBP seq:	1.189317	2.960117	0.425002	-0.90382	-0.63935	1.013367	1.394843	1.607834	p11852	Cold-induci	3	0.880914			
CIC seq: 55	0.227298	-0.0759	0.519014	0.377628	-0.50214	-0.38173	0.158042	-0.27917	p19819	Protein cap	3	0.00538	CDK2;		
CIC seq: 13	0.70618	0.452198	0.37955	0.308564	-0.16667	-0.27341	-0.10277	-0.12431	p16872	Protein cap	3	0.147417			
CIC pS173	0.242066	0.745005	0.346808	0.504031	-0.10956	-0.00466	-0.20963	-0.08119	p06497	Protein cap	3	0.179108	RSK2; p90RSK;		
CIC pS173	0.460115	0.786538	0.400673	0.598725	0.278056	0.054691	0.035824	0.122183	p02258	Protein cap	3	0.342101	RSK2; p90RSK;		
CIAPIN1 se	-0.8148	0.464181	0.255933	-0.536	0.052353	0.133824	0.954249	0.31558	p13998	Anamorsin	3	0.103166			
CHRAC1 se	0.243228	1.006523	1.08839	1.401928	0.202432	0.103557	0.413976	0.469404	p07487	Chromatin	3	0.61618			
CHMP7 pS	0.669033	1.156403	0.916725	0.888089	0.369928	-0.11172	0.114257	0.379071	p13752	Charged mu	3	0.547723		Up with IGF - down with PI3Kinh2 (PI-103); Up with IGF - down with p70S6Kinh1 (PF-4708671); PI3Kinh2 (PI-103); p70S6Kinh1 (PF-4708671);	
CHMP5 seq	0.335186	0.254231	-0.0538	0.035679	0.443927	0.387171	0.91288	0.830052	p18458	Charged mu	3	0.393165			
CHGB pS14	0.652953	-0.11807	1.291236	0.324971	0	0	0	0	p20923	Secretogran	3	0.268886			
CHD8 seq:	0.159984	-0.75806	0.288332	-0.48665	0.48715	0.446403	1.38171	1.592047	p09975	Chromodor	3	0.388865			Regulation GO:0030178; P:negative regulation of Wnt signaling pathway; GO:0060070; P:canonical Wnt signaling pathway;
CHD5 seq:	1.425676	0.546228	1.420012	1.466721	1.550758	1.245729	1.728979	1.579696	p17846	Chromodor	3	1.370475			
CHD1 seq:	0.167571	-0.383	0.538123	0.218257	0.05592	-0.0183	0.022431	-0.09194	p03782	Chromodor	3	0.063633			
CHCHD3 se	0.265522	0.18847	0.597857	0.541445	0.334165	0.072541	0.215511	0.61952	p00114	MICOS com	3	0.354379			
CHCHD3 se	0.286778	0.252172	0.5981	0.537873	0.324962	0.126583	0.349628	0.617103	p00115	MICOS com	3	0.38665			
CHCHD3 p	0.645634	0.391355	0.360712	0.534368	0.438633	0.145954	0.296397	-0.26944	p04228	MICOS com	3	0.317951			
CHAMP1 se	0.132946	0.326509	0.567343	0.545566	-0.05986	-0.09865	0.010374	0.304681	p06278	Chromosor	3	0.216114			
CHAF1A pS	0.370739	1.900499	1.24849	1.284282	-0.08703	-0.56141	0.45231	0.416759	p04700	Chromatin	3	0.628079		AKTinh1 (Akt Inhibitor VIII);	
CGNL1 seq:	1.190469	1.739858	-0.3844	-1.01786	-0.75341	-0.21416	0.337134	-0.71845	p16608	Cingulin-lik	3	0.022397			
CGNL1 seq:	0.290913	1.075313	0.206569	0.462552	0.455384	0.72485	0.342561	0.50489	p12701	Cingulin-lik	3	0.507879			
CGN pS332	0.917152	0.27354	1.142869	0.584444	0.103288	0.077283	-0.24134	-0.42703	p12396	Cingulin	3	0.303776			GO:0007179; P:transforming growth factor beta receptor signaling pathway;

VDAC1 seq	-1.20944	-0.36857	-1.22446	-1.93712	4.722212	6.537448	4.82227	0	p14932	Voltage-dep	2	1.417792				
VCP seq: 50	-1.96575	-0.36146	-2.9314	-0.8708	1.56561	1.665942	2.158986	1.295796	p16749	Transitional	2	0.069615				
VCP seq: 46	0.919553	0.780631	-0.89161	-0.89483	0.738492	1.832154	2.246542	2.933094	p17028	Transitional	2	0.958003				
VCP seq: 45	-4.92273	1.304332	-3.11032	1.001998	8.456277	0	0	8.336849	p00510	Transitional	2	1.383302				
VCP seq: 32	-1.53462	2.164192	3.330836	-7.62636	4.64481	7.047076	0	0	p13529	Transitional	2	1.003242				
VCP seq: 29	-1.70522	-11.2633	-2.79062	-0.56582	0	7.31318	7.305763	0	p09758	Transitional	2	-0.21325				
VCP seq: 27	-0.53766	-0.29102	-0.99792	-1.20071	-0.04323	0.655994	0.331304	1.621434	p12263	Transitional	2	-0.05773				
VCP seq: 21	-0.74101	1.075208	-1.12604	-1.40505	2.449102	2.273987	0.628842	2.624492	p17353	Transitional	2	0.722441				
VCP pM21	0.107227	-0.16703	-0.05874	-0.56932	0.630022	1.278383	0.459545	1.946498	p17352	Transitional	2	0.453323				
VCL seq: 34	-0.10646	0.154871	0.373944	0.204813	4.67879	0	0	0	p19709	Vinculin	2	0.663245			Stabilization and expansion of the E-cadherin adherens junction; RhoA signaling pathway; Signaling events mediated by focal adhesion kinase; Signaling events mediated by VEGFR1 and VEGFR2; I	
VBP1 seq: 1	0.880536	2.135409	0.320124	-7.30812	1.668109	1.867419	0.910205	1.755821	p16609	Prefoldin su	2	0.278688				
USP5 seq: 4	-2.09088	0.729156	-0.98278	-2.16032	2.014204	0.60037	-0.66542	0.800893	p16958	Ubiquitin ca	2	-0.21935				
USP5 seq: 1	-1.21886	0.344759	-0.83688	-1.32759	1.370668	1.010369	0.414077	0.098946	p18816	Ubiquitin ca	2	-0.01806				
UQCRC1 se	-0.71545	0.623984	-1.2693	-1.4107	6.78273	0	0	0	p20904	Cytochrom	2	0.501408				
UPF2 seq: 6	-1.46884	-1.59344	-2.37729	-1.77907	2.357493	0.799849	1.047958	0.944849	p20263	Regulator o	2	-0.25856				
UNKL pS15	-0.05334	-0.1205	-0.27039	-0.22891	2.515625	2.673857	2.79755	2.831263	p21207	Putative E3	2	1.268145				
UNCX pS44	0.347048	0.433571	0.589141	0.529892	0	0	0	7.442386	p04704	Homeobox	2	1.167755				
ULK1 seq: 6	-0.12955	-0.41687	-0.19494	-0.75375	-0.4023	0.703409	1.791173	2.121477	p02311	Serine/thre	2	0.339832			mTOR signa	GO:0007265; P:Ras protein signal transduction;
ULK1 seq: 4	0.136561	-0.36835	0.30732	0.126726	5.472997	0	6.761772	5.549563	p02320	Serine/thre	2	2.248324			mTOR signa	GO:0007265; P:Ras protein signal transduction;
ULK1 pS75	-0.24971	-0.08711	-0.08764	-0.11099	3.271454	2.60562	3.234156	3.421501	p20649	Serine/thre	2	1.49966	mTOR;		mTOR signa	GO:0007265; P:Ras protein signal transduction;
UHRF1BP1	2.926597	1.138074	2.268309	2.924153	0	0	7.412608	7.0136	p05694	UHRF1-bin	2	2.960417				
UFL1 seq: 5	0.726909	1.135104	0.633222	0.300071	1.901288	1.956022	2.647802	3.163725	p18554	E3 UFM1-p	2	1.558018				
UBFD1 seq	-0.7217	0.012289	-0.68774	-0.52708	0	0	8.202233	7.582576	p15245	Ubiquitin d	2	1.732572				
UBE4B seq:	-3.29933	-10.48	-1.28126	-2.32468	7.497096	0	0	0	p04167	Ubiquitin co	2	-1.23602			p73 transcription factor network;	
UBE2NL sec	-0.22011	1.048998	-1.17636	-1.49938	0.520571	2.067588	1.253857	2.474366	p18656	Putative ub	2	0.558691				
UBE2N seq	-0.04721	0.576371	-1.09784	-1.90783	1.196307	2.506305	1.603327	2.7957	p14456	Ubiquitin-c	2	0.703142			IL1-mediate	GO:0070423; P:nucleotide-binding oligomerization domain containing signaling pathway; GO:0035872; P:nucleotide-binding domain, leucine rich repeat containing receptor signalin
UBE2N seq	-0.08319	1.772667	-0.21066	-0.47493	3.557402	3.004369	2.364202	3.424848	p02492	Ubiquitin-c	2	1.669338			IL1-mediate	GO:0070423; P:nucleotide-binding oligomerization domain containing signaling pathway; GO:0035872; P:nucleotide-binding domain, leucine rich repeat containing receptor signalin
UBE2L3 sec	-0.06426	0.441502	-0.59624	-0.26062	0.560218	0.124786	2.300417	3.051461	p09536	Ubiquitin-c	2	0.694658			BARD1 signaling events; Alpha-synuclein signaling;	
UBE2I seq:	-0.71708	1.429904	-2.98337	-1.26485	4.766352	4.861884	2.079687	5.166625	p15074	SUMO-conj	2	1.667393			Sumoylation by RanBP2 regulates transcriptional repression; Regulation of cytoplasmic and nuclear SMAD2/3 signaling; Signaling events mediated by HDAC Class II; Coregulation of Androgen rece	
UBA6 pS36	0.074185	-0.17811	-0.27395	-0.6201	1.396248	1.666875	1.041193	0.869405	p09426	Ubiquitin-li	2	0.496968				
UBA1 seq: 9	-1.23682	-2.60406	-2.4273	-1.35584	6.652318	0	0	0	p09549	Ubiquitin-li	2	-0.12146				
UBA1 seq: 8	-0.69362	-0.63007	-0.98263	-0.7586	3.305577	3.170844	2.293345	3.541453	p19362	Ubiquitin-li	2	1.155787				
UBA1 seq: 6	-0.26445	1.039397	-0.51575	-0.50149	6.363947	5.414829	4.324726	0	p18902	Ubiquitin-li	2	1.982651				
UBA1 seq: 6	-1.11049	-1.44163	-1.36282	-1.28146	2.093214	0.869761	1.089041	1.940396	p05504	Ubiquitin-li	2	0.099501				
UBA1 seq: 1	-0.84338	0.451514	-1.27246	-1.52586	2.575645	1.738893	1.516547	1.661763	p10335	Ubiquitin-li	2	0.537833				
UBA1 pS46	0.503939	1.164876	0.743089	0.695496	5.940552	0	6.798471	0	p09597	Ubiquitin-li	2	1.980803				
UACA seq: 1	0	0	0	0	2.308915	1.682828	-0.21736	1.771058	p16938	Uveal autoa	2	0.69318				
U2SURP se	-1.37941	0.600117	-1.97589	-0.947	3.155874	1.239125	1.678797	2.01247	p16744	U2 snRNP-a	2	0.54801				
U2AF2 seq:	-1.0998	0.016968	-1.40574	-1.84958	1.942629	1.68724	0.535103	1.291564	p00433	Splicing fact	2	0.139798				
U2AF2 seq:	-0.12354	0.744551	-0.77989	-1.47185	2.569087	2.179797	1.554717	2.426616	p17450	Splicing fact	2	0.887436				
U2AF2 seq:	0.473866	1.460338	-0.3543	-1.89527	0.733839	1.028048	1.192797	1.377143	p03385	Splicing fact	2	0.502058				
U2AF1 seq:	-0.63382	-0.3358	-0.60834	-0.78929	0.779344	0.616953	1.176555	0.562985	p09256	Splicing fact	2	0.096074				
TXNDC5 se	-0.74688	1.289034	-0.9545	-0.68166	3.564294	3.405346	2.674538	3.266217	p16888	Thioredoxin	2	1.477048				
TXNDC12 s	-0.5176	1.707859	-0.078	-7.41653	5.410076	4.85544	0	5.517846	p14047	Thioredoxin	2	1.184886				
TWF1 seq: 2	-1.48596	-0.93962	-1.80488	-1.09114	3.313445	2.214133	1.879959	3.233502	p11418	Twinfilin-1	2	0.66493				
TWF1 pS14	0.748339	0.747188	0.862202	1.084827	1.952988	2.152221	2.508206	1.966382	p00258	Twinfilin-1	2	1.502794		Up with EGF - down with AKTinh2 (MK-2206); Up with EGF - down with AKTinh1 (Akt Inhibitor VIII); AKTinh2 (MK-2206); AKTinh1 (Akt Inhibitor VIII);		
TUFM seq: 1	-0.33073	0.601568	-0.44359	-0.62309	4.354755	4.416514	3.74849	3.932702	p20872	Elongation f	2	1.957078				
TUFM seq: 1	-0.14685	0.790837	-0.83349	-1.00182	4.241031	3.652953	1.78708	3.82934	p11459	Elongation f	2	1.539884				
TUFM seq: 1	0.270801	0.158107	0.326301	-0.41964	1.281913	2.272813	2.579863	0.98854	p09372	Elongation f	2	0.932337				
TUBB8 seq:	0.565581	0.344544	-1.43178	-1.07902	0.204215	2.399679	2.826847	2.322999	p17983	Tubulin bet	2	0.769134				
TUBB4B sec	-0.70773	0.360088	-0.54847	-0.98216	1.786357	1.401988	0.45787	0.656663	p13891	Tubulin bet	2	0.303075				
TUBB4B sec	0.565581	0.344544	-1.43178	-1.07902	0.204215	2.399679	2.826847	2.322999	p17985	Tubulin bet	2	0.769134				
TUBB4B pM	-0.55221	0.570207	-2.68409	-3.96058	2.425916	1.361679	0.974502	1.991732	p19220	Tubulin bet	2	0.015895				
TUBB4A sec	-0.53266	0.261828	-0.42958	-0.75499	0.630461	0.870234	0.848973	1.146065	p20128	Tubulin bet	2	0.255042				
TUBB3 seq:	-0.5976	-0.12387	-0.71318	-0.81387	0.505808	0.865573	0.77212	1.173151	p20129	Tubulin bet	2	0.133516				
TUBB2A pM	-1.09541	-0.18999	-0.17849	-0.53104	0.367697	1.056541	0.556708	0.992707	p20454	Tubulin bet	2	0.12234			Signaling events mediated by HDAC Class III; Signaling events mediated by HDAC Class II;	
TUBB seq: 6	-0.43079	0.937427	-0.61979	-0.5289	0.919455	1.088877	0.908332	0.785887	p20456	Tubulin bet	2	0.382563				
TUBB seq: 6	-0.55278	1.220314	-1.359	-1.35625	2.585782	2.59962	2.00989	2.778556	p20455	Tubulin bet	2	0.990765				
TUBB seq: 4	-0.49582	0.198232	-0.48691	-0.56132	1.368145	0.941479	0.374975	0.451582	p13630	Tubulin bet	2	0.223796				
TUBB seq: 3	1.202088	1.294288	-0.16557	-2.28236	0	6.880301	7.312126	0	p10229	Tubulin bet	2	1.780109				
TUBB seq: 3	0.527899	2.074369	-0.92926	-1.76516	2.65803	1.546224	0.892438	2.595687	p00228	Tubulin bet	2	0.950029				
TUBB seq: 2	-0.48233	0.361643	-1.15348	-0.92803	0.243147	0.765933	0.761628	0.958097	p20124	Tubulin bet	2	0.065827				
TUBB seq: 1	-1.05401	0.023067	-1.26026	-2.28552	3.009875	2.986333	2.190237	2.929886	p13774	Tubulin bet	2	0.817452				
TUBA4B sec	-0.94771	-0.77294	-1.24588	-1.59788	-0.0349	1.161034	0.273742	1.293904	p08525	Putative tub	2	-0.23383				
TUBA4B sec	-0.17498	0.16837	-0.3002	-0.70671	1.799098	1.835619	1.471201	1.372401	p08524	Putative tub	2	0.6831				
TUBA4A sec	-0.53046	0.441387	-0.70793	-1.02006	1.851494	1.186793	1.006471	1.155766	p01471	Tubulin alp	2	0.422932				
TUBA4A sec	-0.69608	0.198867	-0.89226	-1.32496	1.162846	1.068366	0.394103	1.088042	p05488	Tubulin alp	2	0.124865				
TUBA3E sec	-1.80091	-0.0276	-1.15251	-2.73707	1.414386	2.602238	1.154846	1.77501	p02701	Tubulin alp	2	0.153549				
TUBA3C sec	-0.93438	0.180063	-1.03371	-1.80167	1.655652	1.574686	0.789338	1.816813	p01470	Tubulin alp	2	0.280848				
TUBA1C sec	0.011179	0.520135	-1.15982	-0.63003	0.394947	0.927532	1.098253	1.019979	p19245	Tubulin alp	2	0.272773				
TUBA1B sec	-0.94771	-0.77294	-1.24588	-1.59788	-0.03595	1.159984	0.272692	1.292854	p08387	Tubulin alp	2	-0.23435			Signaling events mediated by PRL; Signaling events mediated by HDAC Class III; Signaling events mediated by HDAC Class II;	
TUBA1B sec	-0.17498	0.16837	-0.3002	-0.70671	1.799098	1.835619	1.471201	1.372401	p08386	Tubulin alp	2	0.6831			Signaling events mediated by PRL; Signaling events mediated by HDAC Class III; Signaling events mediated by HDAC Class II;	
TUBA1A sec	-0.06889	0.387374	-1.13242	-0.55374	0.444436	0.910455	0.953421	0.994984	p19246	Tubulin alp	2	0.241953			p73 transcription factor network;	
TUBA1A sec	-0.49268	0.479899	-0.79166	-1.21059	1.301765	0.783169	0.303462	0.882706	p10608	Tubulin alp	2	0.157008			p73 transcription factor network;	
TTN seq: 19	-1.52614	-2.01293	-3.70226	-2.01246	3.560535	1.415791	1.766447	1.98319	p10778	Titin	2	-0.06598				GO:0045859; P:regulation of protein kinase activity;
TTN seq: 12	-1.44572	-1.99661	-1.32845	-1.17309	2.14897	0.756894	1.47333	1.086816	p07059	Titin	2	-0.05973				GO:0045859; P:regulation of protein kinase activity;
TTC26 seq:	-1.41973	0.060188	-3.73931	-1.61579	3.2226	3.109401	2.533873	3.491157	p11995	Intraflagella	2	0.705298				
TRIP4 seq: 2	-1.30943	-1.29316	-1.48079	-1.5135	1.499618	0.677848	0.412747	0.737943	p05727	Activating s	2	-0.28359				
TRIP12 seq	0.289821	0.500459	0.84256	0.889023	2.118748	3.520241	3.980389	2.76708	p19744	E3 ubiquitin	2	1.86354				
TRIM3 pS7	0.353785	0.449269	0.589231	0.454219	1.199164	1.569085	3.37379	3.020131	p07488	Tripartite m	2	1.376084				
TRIM28 sec	-0.66782	2.146319	-1.74583	-3.17075	0	0	0	6.062784	p01536	Transcriptio	2	0.328087			ATM pathway; p53 pathway; E2F transcription factor network; C-MYB transcription factor network;	
TRAPPC8 se	-1.76404	0.682205	-2.00975	-1.29468	-0.76411	1.967137	2.167697	1.741148	p06949	Trafficking p	2	0.090701				
TRAF4 pS42	-0.45237	0.256792	-0.23717	0.501386	1.292158	2.044212	1.076197	2.551438	p15249	TNF recepto	2	0.87908	IKKA;		Validated tr	GO:0045860; P:positive regulation of protein kinase activity; GO:0007165; P:signal transduction;
TRA2B seq:	-0.58396	0.3922	-0.50556	-1.21744	2.731221	2.552696	1.18829	2.081631	p01689	Transforme	2	0.829884				
TPT1 seq: 2	-1.06571	0.00536	-0.17126	-1.13983	1.167905	0.563648	0.639912	1.259555	p17638	Translation	2	0.157447			PLK1 signaling events;	
TPT1 seq: 1	-1.93546	-2.02573	-2.42229	-2.11023	1.148018	1.876557	0.367351	0.081332	p14764	Translation	2	-0.62756			PLK1 signaling events;	
TPT1 seq: 1	-0.76211	0.213999	-1.07951	-1.83405	1.479014	1.224442	0.843335	0.580488	p15811	Translation	2	0.0832			PLK1 signaling events;	
TPR seq: 77	-1.9338	0.501588	-0.73659	-1.43557	3.870907	0	0	4.354131	p12146	Nucleoprot	2	0.577582				GO:0019221; P:cytokine-mediated signaling pathway;
TPM4 seq: 1	1.282019	2.790694	-7.86947	1.002503	0.927528	1.861287	2.164642	2.120839	p08934	Tropomyos	2	0.535005				
TPM4 seq:	-1.52614	-2.01293	-3.70226	-2.01246	3.560535	1.410477	1.766447	1.98319	p11295	Tropomyos	2	-0.06664				
TPM4 seq:	-0.55341	2.030302	0.564508	-0.13318	1.384691	1.074567	1.30939	1.804422	p14774	Tropomyos	2	0.935161				
TPM2 seq:	0.225687	3.520196	1.05367	-6.0934	2.648104	2.653545	1.424795	3.152965	p10411	Tropomyos	2	1.073195				
TPI1 seq: 9	-0.42477	0.637893	-1.02497	-0.51212	1.762796	0.346968	0.597085	1.119942	p14459	Triosephos	2	0.312854				
TPI1 seq: 7	-0.32452	0.523756	-0.30648	-0.76672	2.092843	1.721138	1.615473	1.047	p01036	Triosephos	2	0.700312				
TPI1 seq: 7	0	0	0	0	3.594475	3.171688	4.5668	4.483449	p01035	Triosephos	2	1.977052				
TPI1 seq: 5	2.696349	2.999938	-0.85216	-2.43258	2.39245	2.645176	1.784139	2.804554	p07890	Triosephos	2	1.504733				
TPI1 seq: 5	0.287089	0.054107	-0.46408	-0.04438	1.205436	1.568737	1.354643	1.095151	p07889	Triosephos	2	0.632087				
TPI1 seq: 4	-0.67231	1.295582	-0.3863	-1.44777	2.410737	1.163658	0.076798	2.58324	p16771	Triosephos	2	0.627955				
TPI1 seq: 2	-0.95102	-1.14784	-1.56793	-0.63006	1.646037	0.532424	0.610411	0.849925	p14121	Triosephos	2	-0.08226				
TPI1 seq: 2	-0.432	0.363	-0.62424	-1.00917	1.252819	1.067834	0.585116	1.321183	p03005	Triosephos	2	0.315568				
TPI1 seq: 1	-1.39952	-1.50843	-0.97842	-1.5164	1.760803	0.64152	0.672417	2.189627	p00610	Triosephos	2	-0.0173				
TPI1 seq: 1	-0.4303	-0.01565	-0.6244	-1.81707	1.716296	1.648658	0.799794	2.366352	p00609	Triosephos	2	0.455459				
TPI1 seq: 1	-0.46156	-0.10313	-0.23211	-0.66368	1.25441	1.221725	1.235154	0.837766	p01410	Triosephos	2	0.386072				
TPI1 seq: 1	-0.64105	-0.64762	-0.93398	-1.00352	1.205078	0.659452	0.65626	0.748978	p00627	Triosephos	2	0.00545				
TPD52L2 se	1.691725	1.623344	-0.89352	-7.4402	2.254621	1.806324	1.444617	2.682277	p11733	Tumor prot	2	0.396149				
TPD52L2 se	-0.13111	3.133671	0.703539	-0.40565	2.724156	1.966746	1.280652	2.606088	p00687	Tumor prot	2	1.484762				
TPD52L1 se	0.780697	0.505761	0.208172	-0.03297	1.524072	2.093675	1.092293	1.020664	p14641	Tumor prot	2	0.899046				
TPD52L1 p	-1.01689	-0.62524	-0.90877	-1.2688	0	0	7.602688	0	p01477	Tumor prot	2	0.472874				
TPD52 seq:	0.503476	0.680894	-1.56896	-2.18185	8.774379	8.198234	0	0	p01482	Tumor prot	2	1.800772				
TP53BP1 se	0.661793	0.528577	0.837192	-0.17825	2.985981	3.213239	4.081223	3.421545	p01893	Tumor supp	2	1.943913			ATM pathway;	
TOPORS sec	0.180375	0.580667	-1.42531	-2.67989	-0.69422	1.616304	2.675168	0.192869	p10940	E3 ubiquitin	2	0.055745				
TNS1 seq: 1	-0.07627	-0.87797	-0.17839	0.101917	0	7.283519	0	0	p05861	Tensin-1	2	0.7816			Integrin-linked kinase signaling;	
TNRC6B se	-0.09997	2.446865	0.769437	0.897224	2.667121	2.521875	3.87363	4.457798	p15443	Trinucleotic	2	2.191748				GO:0007219; P:Notch signaling pathway; GO:0048015; P:phosphatidylinositol-mediated signaling; GO:0008543; P:fibroblast growth factor receptor signaling pathway; GO:0007173; P:epidermal growth factor receptor signaling pathway; GO:0048011; P:neurotrophin TRK receptor signaling pathwa
TMSB4X se	1.717025	2.889349	0.131961	-6.32067	0.439443	0.370916	0.387118	1.00352	p09268	Thymosin b	2	0.077332				
TMSB4X se	-0.77689	0.174486	-0.87503	-1.20791	1.985821	1.254378	0.509396	1.801274	p02880	Thymosin b	2	0.358192				
TMF1 seq: 9	-9.6245	1.729785	-0.48117	-0.49147	0	7.310846	0	7.579579	p03703	TATA eleme	2	0.752883			Coregulation of Androgen receptor activity;	
TMEM57 se	1.384464	-2.47259	-0.34135	-1.77478	7.100815	6.001472	0	0	p07163	Macoilin	2	1.237253				
TLR8 seq: 6	-0.66254	0.836939	-0.83573	-1.06185	2.667041	1.123106	0.788932	2.142475	p11962	Toll-like rece	2	0.624797				GO:0007249; P:I-kappaB kinase/NF-kappaB signaling; GO:0034162; P:toll-like receptor 9 signaling pathway; GO:0002224; P:toll-like receptor signaling pathway;
TLN1 seq: 8	-1.26703	1.010076	-1.23714	-1.13688	4.251225	4.364157	2.238366	5.082175	p14098	Talin-1	2	1.663118			Nectin adhe	GO:0006987; P:activation of signaling protein activity involved in unfolded protein response;
TLN1 seq: 2	-0.77136	0.058557	-1.34278	-3.3517	5.879422	0	4.391136	6.202711	p20261	Talin-1	2	1.383248			Nectin adhe	GO:0006987; P:activation of signaling protein activity involved in unfolded protein response;
TLN1 seq: 2	-0.7704	0.348796	-0.98796	-1.09819	3.810684	2.926065	3.207454	3.603352	p01120	Talin-1	2	1.379976			Nectin adhe	GO:0006987; P:activation of signaling protein activity involved in unfolded protein response;
TLN1 seq: 1	-0.65213	0.004247	-0.76955	-0.71863	-0.58298	3.810651	0.876881	1.540448	p19176	Talin-1	2	0.438616			Nectin adhe	GO:0006987; P:activation of signaling protein activity involved in unfolded protein response;
TKTL2 seq:	-1.33146	-1.20504	-1.59308	-1.61153	1.637525	0.668929	0.054209	0.539785	p20781	Transketola	2	-0.35508				
TKT seq: 61	-0.69259	0.264976	-0.9338	-1.09683	1.845513	1.481329	0.798174	1.528042	p18976	Transketola	2	0.399352				
TKT seq: 54	-0.81098	-1.09763	-1.26994	-0.9329	1.940685	2.076494	1.437279	2.427151	p11583	Transketola	2	0.471269				
TKT seq: 53	-0.66572	-0.42618	-0.89467	-1.15733	0.517231	1.350682	0.786903	0.235862	p01255	Transketola	2	-0.03165				
TKT seq: 43	-0.51394	0.503837	-0.63233	-1.1857	2.624823	1.664551	1.646792	1.779967	p03296	Transketola	2	0.736001				
TKT seq: 39	-1.33146	0.437655	-1.21939	-1.59208	2.356987	1.922452	0.494387	1.973299	p20782	Transketola	2	0.380232				
TKT seq: 34	-0.76907	-0.46057	-0.74665	-2.28539	0.469979	0.952382	0.498533	1.193145	p09061	Transketola	2	-0.14346				
TKT seq: 33	-0.36984	0.664704	-0.54799	-0.82043	1.470939	1.111616	0.313177	1.199315	p14198	Transketola	2	0.377686				
TKT seq: 30	-1.33048	-1.55252	-1.01558	-1.40606	3.096113	1.492898	0.688587	2.501643	p09947	Transketola	2	0.309326				
TKT seq: 28	-0.53465	0.426429	-0.71381	-0.76826	1.429614	0.538268	0.546994	1.427773	p14086	Transketola	2	0.294044				
TKT seq: 23	-1.22516	-1.25146	-1.15711	-1.2684	2.040553	1.602488	0.650415	1.798688	p14671	Transketola	2	0.148752				
TKT seq: 10	-2.50477	-0.96015	-1.4915	-1.72448	1.56463	0.893207	0.468441	1.233566	p13668	Transketola	2	-0.31513				
TKT pM604	-1.0557	-1.34287	-0.95411	-1.1639	0.925289	1.255943	0.731194	0.557153	p10143	Transketola	2	-0.13088				
TKT pM266	-0.26545	-0.23074	-0.80289	-0.55482	-0.30699	1.012942	1.503685	1.799503	p09384	Transketola	2	0.269406				
TJP3 pS346	0.230965	0.280026	0.748423	0.238486	3.015056	2.8263	2.294659	2.178357	p02695	Tight juncti	2	1.476534				
THRAP3 pS	1.519665	2.503321	0.822807	1.44416	1.733258	2.91689	3.242415	2.471321	p17204	Thyroid hor	2	2.08173				GO:0030518; P:intracellular steroid hormone receptor signaling pathway; GO:0030521; P:androgen receptor signaling pathway;
THOP1 seq	-0.40177	0.00345	-1.0384	-0.9296	3.086641	2.923427	1.489049	2.602294	p19239	Thimet olig	2	0.966886				
THOC3 seq	-0.80554	0.37571	-2.34117	-1.32872	0.812224	1.137856	0.528414	1.488007	p12187	THO comple	2	-0.01665				
TGM2 seq:	-0.03331	1.596602	-0.73657	-2.98096	0.886097	1.173613	1.002366	1.505053	p10334	Protein-glut	2	0.301611			Alpha9 beta	GO:0051482; P:positive regulation of cytosolic calcium ion concentration involved in phospholipase C-activating G-protein coupled signaling pathway; GO:0043123; P:positive regulation of I-kappaB kinase/NF-kappaB signaling;
TGM2 seq:	0	0	0	0	1.539244	1.182948	1.015692	1.532043	p18500	Protein-glut	2	0.658741			Alpha9 beta	GO:0051482; P:positive regulation of cytosolic calcium ion concentration involved in phospholipase C-activating G-protein coupled signaling pathway; GO:0043123; P:positive regulation of I-kappaB kinase/NF-kappaB signaling;
TFG pS197	-0.30209	-0.48751	-7.47622	0.505898	0.454003	1.38342	1.004374	1.612544	p08960	Protein TFG	2	-0.4132				GO:0043123; P:positive regulation of I-kappaB kinase/NF-kappaB signaling; GO:0007165; P:signal transduction;
TFAP2B pS2	0.020347	0.046287	-0.82085	0.231895	0	0	0	8.16821	p10756	Transcriptio	2	0.955737				GO:0032486; P:Rap protein signal transduction; GO:0007165; P:signal transduction;
TCP1 seq: 4	-0.69986	0.631305	-0.71026	-1.21245	2.688867	2.320364	1.57884	2.439362	p17283	T-complex p	2	0.879521				
TCOF1 pS2	-1.02076	-0.36149	-1.49277	-2.77818	1.98896	2.810813	2.862269	1.929252	p19465	Treacle prot	2	0.492263				
TCOF1 pS2	-0.52661	-2.17897	0.249595	-0.84075	2.350048	3.280086	3.347437	2.4035	p19466	Treacle prot	2	1.010543				
TCOF1 pS2	0	0	0	0	1.411413	2.167844	5.982795	2.114646	p19464	Treacle prot	2	1.459587				
TCEB2 seq:	-0.06069	1.445305	-0.89919	-2.91479	2.712466	2.538552	1.33831	3.308009	p17108	Transcriptio	2	0.933496			Hypoxic and oxygen homeostasis regulation of HIF-1-alpha; HIF-2-alpha transcription factor network;	
TCEAL3 pS6	0.859389	0.53007	1.499211	1.258284	0	0	8.048331	7.006807	p17892	Transcriptio	2	2.400262				
TCEA2 seq:	-1.42321	0.056845	-0.77889	-1.68127	4.237139	3.551034	2.820056	4.035574	p17844	Transcriptio	2	1.35216				
TCEA1 seq:	-1.31873	1.113738	-1.21414	-1.16305	1.487107	1.770144	0.415888	2.658933	p18136	Transcriptio	2	0.468737				
TC2N pS16	-0.21231	-0.19585	-0.08779	-0.11284	1.532545	3.916574	3.010689	4.835028	p02486	Tandem C2	2	1.585756				
TBL1X seq:	-0.02312	-0.11314	-0.14838	-0.08293	1.361336	2.996235	2.987998	3.451286	p17749	F-box-like/W	2	1.303661			Regulation	GO:0060070; P:canonical Wnt signaling pathway; GO:0007219; P:Notch signaling pathway;
TBCA seq: 9	0.758681	0.530801	-0.9496	-1.67244	0.678748	0.376065	0.440095	1.447701	p10396	Tubulin-spe	2	0.201256				
TBCA seq: 7	0.668207	0.912109	-0.4605	-0.3664	1.241811	1.479367	0.722629	1.454629	p12011	Tubulin-spe	2	0.706482				
TBC1D8B s	-0.84555	-1.34416	-1.87435	-1.16576	3.832679	0.858669	0.261337	3.342829	p03330	TBC1 doma	2	0.383212				
TBC1D4 pT	0.569157	0.887568	0.482991	0.495672	1.982876	1.988794	1.008884	1.950018	p02090	TBC1 doma	2	1.170745			Insulin-mediated glucose transport; Class I PI3K signaling events mediated by Akt;	
TBC1D1 sec	-0.34529	0.224064	0.205557	-1.39521	0	0	5.125582	0	p03900	TBC1 doma	2	0.476838				
TAPT1 seq:	0.207964	-0.09425	-0.52086	-1.34348	2.261193	1.943584	1.313115	1.961107	p18586	Transmemb	2	0.716047				GO:0007186; P:G-protein coupled receptor signaling pathway;
TANK pS35	0.141051	-0.66464	-0.41246	-0.38732	1.08723	2.015282	2.413477	1.37094	p21191	TRAF family	2	0.695445				GO:0007249; P:I-kappaB kinase/NF-kappaB signaling; GO:0007165; P:signal transduction;
TANK pS35	-0.32535	-0.94381	-0.55622	-0.41385	0	0	8.579735	5.493231	p21190	TRAF family	2	1.479216				GO:0007249; P:I-kappaB kinase/NF-kappaB signaling; GO:0007165; P:signal transduction;
TANC2 pS1	0.098357	0.070306	-0.01268	-0.81655	0	6.811268	0	5.748832	p03642	Protein TAN	2	1.487442		ROCKinh1 (H-1152);		
TAGLN2 se	-0.97128	-1.01607	-0.91162	-0.87653	2.522449	0.879317	1.601392	1.378552	p15529	Transgelin-2	2	0.325776				
TAF15 seq:	-1.35093	1.128254	-2.18973	-1.63414	3.07384	3.119416	1.572992	3.23744	p21128	TATA-bindi	2	0.869642				
TACC3 pS2	-0.63071	-1.55677	-0.05347	-0.2085	1.769312	2.000456	2.250621	1.681791	p09030	Transformi	2	0.656593			Aurora A signaling; Integrin-linked kinase signaling;	
SYNCRIP se	-1.86159	0.27817	-1.51221	-1.8455	5.055538	4.504221	3.202599	4.547023	p20602	Heterogene	2	1.546032				
SYAP1 seq:	0.563116	0.631443	-2.24582	-3.50411	0	0	6.445896	8.774765	p02287	Synapse-as	2	1.333161				
SUCLG2 sec	-0.15892	1.001547	-0.18423	-0.75534	0.888409	2.621511	1.763462	2.624819	p17921	Succinyl-Co	2	0.975157				
SUCLG1 sec	-0.8277	1.634238	-3.64846	-2.29742	6.693001	0	0	0	p14194	Succinyl-Co	2	0.194207				
STT3B pS18	0.246878	-1.06066	0.206529	-0.23704	0	0	6.897054	0	p03848	Dolichyl-dip	2	0.756595				
STRN seq: 2	0.081544	-1.49599	0.46719	0.390115	3.326537	0.800983	3.653028	4.029261	p06014	Striatin	2	1.406583			Plasma mer	GO:0016055; P:Wnt signaling pathway;
STRAP seq:	-0.61749	1.206372	-1.22881	-1.29007	0.088878	2.748959	3.025989	2.846678	p08655	Serine-three	2	0.847563			TGF-beta re	GO:0030512; P:negative regulation of transforming growth factor beta receptor signaling pathway; GO:0007179; P:transforming growth factor beta receptor signaling pathway;
STMN1 seq	-0.72699	0.623614	-0.66462	-1.32023	0.409127	0.940413	0.303907	1.265034	p05425	Stathmin	2	0.103781			Signaling m	GO:0035556; P:intracellular signal transduction; GO:0007165; P:signal transduction;
STMN1 seq	-0.81872	-0.98918	-1.52945	-1.42706	1.986774	2.07402	1.566295	2.447105	p19611	Stathmin	2	0.413723			Signaling m	GO:0035556; P:intracellular signal transduction; GO:0007165; P:signal transduction;
STMN1 seq	-0.07603	0.170723	-2.23611	-2.41403	2.954247	2.996863	0.490568	3.638501	p19610	Stathmin	2	0.690592			Signaling m	GO:0035556; P:intracellular signal transduction; GO:0007165; P:signal transduction;
STK11IP pS	0.108965	0.166079	0.481476	0.220123	7.180293	5.732973	5.911624	0	p07580	Serine/thre	2	2.475192			LKB1 signaling events;	
STIP1 seq: 5	-0.80139	0.418854	-1.76128	-1.83951	3.11876	3.647213	2.492245	4.020201	p11240	Stress-indu	2	1.161886				
STIP1 seq: 5	-1.23444	3.706751	-1.65235	0.442068	0.619381	2.555517	0.669802	3.467922	p11580	Stress-indu	2	1.071831				
STIP1 seq: 1	-0.26643	1.227443	-0.48946	-1.44815	2.547011	1.99086	1.054214	2.234137	p02597	Stress-indu	2	0.856204				
STIP1 seq: 1	0.26404	0.793286	-0.54881	-2.24022	0.239258	0.812308	1.897579	1.51609	p20143	Stress-indu	2	0.341691				
ST13 seq: 5	-1.29772	0.244323	-0.40277	-1.86558	2.129748	-1.2262	-2.84806	3.159528	p12420	Hsc70-inte	2	-0.26334				
ST13 seq: 1	-1.18527	-0.60545	-1.05089	-1.03219	1.454311	0.627122	-0.16974	1.391858	p08759	Hsc70-inte	2	-0.07128				
SSH1 seq: 9	1.478353	0.102804	0.590581	-8.57104	0.225786	0.671525	1.095965	1.082087	p11693	Protein pho	2	-0.41549			FGF signaling pathway; CXCR4-mediated signaling events;	
SSFA2 pT11	-1.35879	-1.39569	-1.70203	-1.16496	0.984532	0.79633	1.277842	1.337501	p05338	Sperm-spec	2	-0.15316				
SRSF9 seq:	-0.47707	0.772268	-0.35377	-1.25031	1.976701	1.799341	0.937573	2.102343	p13480	Serine/argin	2	0.68901				
SRSF9 pS20	1.078647	1.645787	1.053597	0.852625	2.187392	3.531533	3.614057	2.720415	p15353	Serine/argin	2	2.085507				
SRSF9 pS20	1.010648	1.812687	1.038869	1.033296	2.272797	3.658111	3.941876	2.527744	p15352	Serine/argin	2	2.162004				
SRSF7 seq:	-0.06582	0.687308	-0.68236	-0.51405	0.692422	1.040496	1.178165	1.393135	p09260	Serine/argin	2	0.466162				
SRSF6 seq:	-0.57629	-0.20887	-0.81252	-0.60134	1.468052	0.823088	0.143656	1.260261	p11404	Serine/argin	2	0.187005				
SRSF6 seq:	-1.35736	0.261624	-1.61205	-1.33588	1.494425	1.175572	0.305552	0.367091	p02490	Serine/argin	2	-0.08763				
SRSF4 seq:	-0.57629	-0.20887	-0.81252	-0.60134	1.468052	0.823088	0.143656	1.260261	p14050	Serine/argin	2	0.187005				
SRSF4 seq:	-0.86954	-0.83521	-1.07898	-1.0144	1.530654	1.123671	0.197318	0.518962	p11539	Serine/argin	2	-0.05344				
SRSF3 seq:	-0.10384	0.752645	-0.63208	-0.50751	2.331863	1.776347	1.288911	2.62093	p09261	Serine/argin	2	0.940908				
SRSF3 seq:	-1.63649	-2.22193	-1.99234	-1.70957	2.390594	1.403184	1.626969	1.27265	p20766	Serine/argin	2	-0.10837				
SRSF2 seq:	-0.57166	0.444357	-0.84779	-1.24126	2.125468	1.493744	0.759399	1.763749	p01506	Serine/argin	2	0.490751				
SRSF2 seq:	0.197188	1.091601	-0.52752	-0.3182	2.209233	1.604421	0.876285	0.953887	p01669	Serine/argin	2	0.760863				
SRSF2 pS26	0.609922	0.93972	0.77359	0.740713	2.216737	2.387868	2.529699	2.58861	p02157	Serine/argin	2	1.598357				
SRSF11 pS4	0.958185	1.665827	0.828362	1.283185	2.065721	2.409392	2.539856	2.012276	p12782	Serine/argin	2	1.72035				
SRSF1 seq:	-0.47808	1.004256	0.825938	-0.09867	2.191453	1.653977	1.713933	1.995984	p18761	Serine/argin	2	1.101099				
SRSF1 seq:	-0.87566	0.930593	-0.52735	-1.33216	-0.08766	1.710745	0.365446	2.155518	p05609	Serine/argin	2	0.292435				
SRSF1 seq:	-0.46428	0.527308	-0.52042	-0.81637	1.344395	0.837711	0.587095	1.208763	p18794	Serine/argin	2	0.338026				
SRSF1 seq:	-0.30554	0.90083	-0.59742	-0.78681	3.551853	2.723772	2.269201	3.088716	p17971	Serine/argin	2	1.355575				
SRRT seq: 8	-1.42863	-0.53096	-2.94485	-2.46313	5.492658	0	1.567458	4.178307	p20414	Serrate RNA	2	0.483857				
SRRM2 seq	0.346601	0.005883	0.158221	0.579928	1.433095	1.23443	1.933845	1.381281	p04100	Serine/argin	2	0.884161				
SRRM2 seq	-3.64924	-0.30695	-1.74238	-0.77812	2.045497	1.637386	1.933402	2.257924	p04018	Serine/argin	2	0.174691				
SRRM2 seq	-0.02384	-0.32648	0.050311	-0.00563	1.861334	-0.00972	1.61632	2.178029	p03584	Serine/argin	2	0.667541				
SRRM2 pS1	1.434668	0.895343	1.54608	1.701901	2.31193	3.829476	2.623534	1.762966	p03959	Serine/argin	2	2.013237				
SRRM1 seq	0.137534	0.456598	-0.31143	0.213596	0.906738	0.987877	1.918941	1.681519	p02229	Serine/argin	2	0.748922				
SRRM1 pS7	-2.17043	0.013217	1.480398	0.839012	3.18244	2.430581	2.728105	3.29313	p13469	Serine/argin	2	1.474557				
SRRM1 pS7	0.403915	0.527755	0.319127	1.048447	1.902529	2.038066	2.27997	1.037971	p06430	Serine/argin	2	1.194722				
SRRM1 pS6	-5.86825	-1.10171	0.272509	-1.9046	2.496705	3.924698	4.847181	4.152756	p06441	Serine/argin	2	0.852411				
SRRM1 pS6	0.472582	0.754661	-0.25201	0.830933	1.367638	1.700065	2.175495	1.674507	p06440	Serine/argin	2	1.090483				
SRRM1 pS6	0.388089	0.583095	-0.23995	0.419835	2.478027	3.189192	3.148108	1.75858	p07563	Serine/argin	2	1.465622				
SRPR seq: 4	-1.07314	2.376562	-1.36562	-0.09007	2.617387	1.606677	1.476996	2.424775	p06472	Signal recog	2	0.996696				GO:0006987; P:activation of signaling protein activity involved in unfolded protein response;
SRI seq: 77	0	7.287629	0	0	3.430929	4.232092	3.016408	4.667086	p11216	Sorcin	2	2.829268				GO:0007165; P:signal transduction;
SQSTM1 se	-0.84009	-0.19507	-0.37634	-0.81372	0.526123	2.091953	1.204233	1.032459	p08925	Sequestoso	2	0.328694			IL1-mediate	GO:0043122; P:regulation of I-kappaB kinase/NF-kappaB signaling; GO:0048011; P:neurotrophin TRK receptor signaling pathway; GO:0035556; P:intracellular signal transduction;
SPTBN1 sec	0.234735	1.213134	-0.74296	-0.694	3.248359	2.136524	2.089338	3.087955	p11402	Spectrin be	2	1.321636			TGF-beta receptor signaling;	
SP4 pS757	0.428462	0.055523	0.075636	0.176547	0	5.660708	4.316824	6.84986	p16046	Transcriptio	2	2.195445			Caspase cascade in apoptosis;	
SP100 seq:	-0.56443	-1.35136	-1.72338	-1.20943	1.565648	1.849705	2.021135	2.046929	p07416	Nuclear aut	2	0.329353				GO:0048384; P:retinoic acid receptor signaling pathway; GO:0060337; P:type I interferon signaling pathway; GO:0060333; P:interferon-gamma-mediated signaling pathway; GO:0019221; P:cytokine-mediated signaling pathway;
SOS2 pS13	0.520966	1.193741	0.02134	0.379505	0	0	7.544655	8.191423	p17403	Son of seve	2	2.231454			Regulation	GO:0051056; P:regulation of small GTPase mediated signal transduction; GO:0048011; P:neurotrophin TRK receptor signaling pathway; GO:0007264; P:small GTPase mediated signal transduction;
SORL1 seq:	-0.50808	0.199775	-0.27018	-0.89708	2.047235	1.290097	-0.14392	1.227314	p07058	Sortilin-rela	2	0.368146				GO:0007165; P:signal transduction;
SOGA1 seq	0.035112	0.103021	0.121962	0.063841	1.35056	2.621175	3.562617	2.368053	p19891	Protein SOG	2	1.278293				GO:0008286; P:insulin receptor signaling pathway;
SNU13 seq	-0.80424	-0.85274	-1.20498	-1.35626	1.320719	0.723214	0.720743	2.481329	p19118	NHP2-like p	2	0.128473				
SNRPN seq	-2.72347	-2.99687	-3.34356	-2.83045	4.920646	2.339279	1.817371	3.876164	p14303	Small nucle	2	0.13239				
SNRPD3 se	-1.1176	-1.23137	-1.02524	-1.20463	2.146363	0.489686	0.79019	2.070325	p01772	Small nucle	2	0.114716				
SNRPD2 se	-0.44692	-0.10405	-1.56031	-0.09133	1.380687	1.136846	1.21458	1.709855	p16198	Small nucle	2	0.29242				
SNRPA1 se	-0.93861	2.462033	-0.65593	-6.00501	5.889437	0	0	6.564654	p01262	U2 small nu	2	0.914571				
SNRNP70 s	-0.68809	-0.26432	-1.32151	-1.48859	1.32003	0.928656	0.994121	2.079419	p17804	U1 small nu	2	0.194965				
SMYD3 seq	-0.47784	2.745358	-1.66475	-3.62182	1.913178	1.044712	-0.23586	1.60166	p13102	Histone-lys	2	0.163079				
SMG9 seq:	-0.35483	1.259651	-0.64679	-0.17821	1.074303	2.515959	1.343083	-0.23637	p17841	Protein SM	2	0.597099		AKTinh2 (MK-2206);		
SMG8 seq:	-0.05458	0.734159	-0.16895	0.026898	3.261736	4.376033	3.065004	3.144765	p08806	Protein SM	2	1.798133				GO:0045859; P:regulation of protein kinase activity;
SMARCC2 s	1.06206	1.510101	1.098848	1.123936	1.531732	2.528525	2.961012	2.342077	p13172	SWI/SNF co	2	1.769786			Glucocorticoid receptor regulatory network;	
SLC9A3R1 s	-0.56564	1.015513	-0.26657	-0.67589	0	7.931161	0	0	p08568	Na(+)/H(+)	2	0.929822			Thromboxa	GO:0014067; P:negative regulation of phosphatidylinositol 3-kinase signaling; GO:0007191; P:adenylate cyclase-activating dopamine receptor signaling pathway; GO:0045859; P:regulation of protein kinase activity; GO:0051898; P:negative regulation of protein kinase B signaling; GO:0070373; P:negative regulation of ERK1 and ERK2 cascade; GO:0016055; P:Wnt signaling pathway
SLC9A3R1 s	-0.71315	-0.39221	-0.7879	-1.1297	2.088115	2.750649	1.490727	1.821634	p21128	Na(+)/H(+)	2	0.641021			Thromboxa	GO:0014067; P:negative regulation of phosphatidylinositol 3-kinase signaling; GO:0007191; P:adenylate cyclase-activating dopamine receptor signaling pathway; GO:0045859; P:regulation of protein kinase activity; GO:0051898; P:negative regulation of protein kinase B signaling; GO:0070373; P:negative regulation of ERK1 and ERK2 cascade; GO:0016055; P:Wnt signaling pathway
SLC2A13 p	0.821914	-4.15448	-0.08934	0.055346	1.680926	0.281194	1.400771	2.106912	p13401	Proton myo	2	0.262905				
SLC25A27	-1.23108	-1.40947	-1.38168	-1.13573	3.063185	2.968956	1.518939	2.886697	p14096	Mitochond	2	0.659977				
SLAIN2 seq	0.099145	-0.95212	0.181367	0.188572	2.279545	0.516779	0.834807	3.853201	p03738	SLAIN motif	2	0.875162				
SKP2 pS64	1.488703	0.334113	-1.81659	-0.96657	0.985591	1.432141	1.940844	3.183471	p02186	S-phase kin	2	0.822712	Pim1; CDK2;		C-MYC pathway; FOXM1 transcription factor network; FoxO family signaling; p53 pathway; Notch signaling pathway; Regulation of retinoblastoma protein;	
SKP1 seq: 1	-0.80151	-0.45029	-1.09114	-1.53626	1.463659	0.920157	0.334323	1.262616	p15711	S-phase kin	2	0.012692			Degradatio	GO:0007219; P:Notch signaling pathway;
SIPA1L3 se	-0.19426	0.164352	0.076099	0.186306	1.312127	0.964026	1.148575	1.506632	p02032	Signal-indu	2	0.645482				GO:0051056; P:regulation of small GTPase mediated signal transduction;
SIPA1L3 pS	1.837893	1.242312	-0.67848	-1.24418	1.554907	4.562265	4.883672	5.075579	p19190	Signal-indu	2	2.154246				GO:0051056; P:regulation of small GTPase mediated signal transduction;
SIPA1L1 pS	0.035134	0.28436	0.298546	-2.00379	0	0	7.184478	9.36995	p15286	Signal-indu	2	1.896085				GO:0048013; P:ephrin receptor signaling pathway;
SIK3 pS920	0.12505	-1.13122	-0.38847	-0.33475	6.582029	0	2.334353	0	p14634	Serine/thre	2	0.898375			LKB1 signaling events;	
SHROOM2	0.241463	0.051863	0.30714	0.067839	8.138284	0	9.35882	0	p03141	Protein Shr	2	2.270676				
SHROOM2	-0.26628	-0.62393	0.008074	-0.32662	0	0	0	5.719554	p04000	Protein Shr	2	0.56385				
SHROOM2	0.096315	-0.58815	0.132337	-0.03384	0	0	0	8.435692	p11034	Protein Shr	2	1.005295				
SHISA8 seq	-1.07474	-1.30572	-1.45548	-1.29747	5.440163	0	5.595943	5.813173	p20135	Putative pro	2	1.464483				
SHANK2 se	0.211503	0.619375	0.17121	0.370537	4.621221	4.114605	5.064082	4.397918	p04019	SH3 and mu	2	2.446306				
SHANK2 se	0.011881	-0.10648	0.047097	-0.36444	0	0	7.456356	0	p03750	SH3 and mu	2	0.880552				
SHANK2 se	-0.43781	-0.5302	-0.0454	0.004212	1.949822	1.415134	0.612071	2.386098	p08641	SH3 and mu	2	0.66924				
SH3D19 se	-0.23645	-1.23689	0.206426	-0.16512	2.388921	2.370266	3.340637	1.797051	p03727	SH3 domain	2	1.058105				
SH3BP1 se	-1.53107	0.017514	-4.03182	-1.60685	1.04765	1.082004	0.282685	0.911446	p07062	SH3 domain	2	-0.47856				GO:0007265; P:Ras protein signal transduction; GO:0090090; P:negative regulation of canonical Wnt signaling pathway; GO:0007165; P:signal transduction;
SH3BGRL3	-0.80827	-0.54528	-1.4658	-1.29665	1.77384	0.413217	0.642944	1.007155	p00521	SH3 domain	2	-0.03486				
SGTA seq: 1	-2.1239	0.442518	-0.8475	-2.96631	2.055377	1.602993	0.969335	1.961734	p11724	Small glutar	2	0.13678				
SFRP2 seq:	-1.53107	0.323136	-3.17742	-1.60685	1.05134	1.085693	0.286374	0.915135	p07242	Secreted fri	2	-0.33171				GO:0030514; P:negative regulation of BMP signaling pathway; GO:0030178; P:negative regulation of Wnt signaling pathway; GO:0090263; P:positive regulation of canonical Wnt signaling pathway; GO:0090090; P:negative regulation of canonical Wnt signaling pathway; GO:0007267; P:cell-cell signaling;
SFPQ seq: 3	-6.82981	3.864301	1.473767	1.083917	3.016748	2.017686	1.650495	2.26468	p20828	Splicing fact	2	1.067723				
SFPQ seq: 2	-1.66794	-1.26384	-3.60066	-1.4019	2.904842	-0.48515	1.983287	0.944137	p20046	Splicing fact	2	-0.3234				
SFPQ seq: 2	-0.39705	0.550789	-0.69129	-1.13075	1.780258	1.056115	-0.43263	1.314613	p13750	Splicing fact	2	0.256256				
SFN seq: 14	-2.27942	-0.73435	-1.50723	-1.42398	1.412633	1.17425	0.614563	1.046123	p06164	14-3-3 prot	2	-0.21218			p38 signali	GO:0006469; P:negative regulation of protein kinase activity; GO:0007165; P:signal transduction;
SFN pM22	0.349166	-0.14531	-0.15368	-0.57747	1.231625	1.765236	1.19361	1.842789	p18202	14-3-3 prot	2	0.688246			p38 signali	GO:0006469; P:negative regulation of protein kinase activity; GO:0007165; P:signal transduction;
SF3B2 seq:	-1.18789	-2.42041	-2.02834	-2.01275	1.235654	1.051987	0.203505	1.850001	p16406	Splicing fact	2	-0.41353				
SF3B2 seq:	-1.13794	-1.39215	-1.17463	-1.25976	2.052919	1.388137	0.788947	2.119221	p14314	Splicing fact	2	0.173092				
SF3B2 seq:	-1.79434	0.672499	-1.68885	-3.06438	2.71165	2.28073	0.744922	2.14934	p13864	Splicing fact	2	0.251446				
SETX seq: 2	-1.85405	-1.84905	-2.27071	-3.83308	1.950488	2.342996	0	3.462146	p14160	Probable he	2	-0.25641				
SETX seq: 1	0.174436	-0.09265	-1.19335	-0.5392	2.79293	2.309479	2.788528	2.040496	p11264	Probable he	2	1.035084				
SERPINH1 s	-0.28814	0.512184	-0.17128	-0.39157	1.774817	0.853674	1.306029	0.8887	p18156	Serpin H1	2	0.560553				
SERPINB8 s	0	0	0	0	0.265133	2.70988	3.078198	2.523909	p16100	Serpin B8	2	1.07214				
SEPT9 seq:	-1.36554	0.223914	-1.47801	-1.79975	3.365044	2.278923	1.127424	2.087351	p07738	Septin-9	2	0.554919				
SEPT6 seq:	-1.11988	0.70259	-1.20902	-1.6326	1.627939	1.289729	0.652429	1.3683	p01062	Septin-6	2	0.209936				
SEPT2 seq:	0	0	0	0	1.804371	1.531002	2.381921	1.641011	p08034	Septin-2	2	0.919788			CDC42 sign	GO:0007224; P:smoothened signaling pathway;
SEPT14 seq	-0.40684	1.151856	-4.06272	-2.59874	2.898772	0	0	1.834676	p01071	Septin-14	2	-0.14787				
SEPT11 seq	-0.40684	1.151856	-4.06272	-2.59874	2.898772	0	0	1.834676	p01072	Septin-11	2	-0.14787				
SEC16A seq	-0.41431	0.722464	-0.27207	-0.05903	0	0	0	8.75713	p20585	Protein tran	2	1.091772				
SEC16A seq	-1.02069	1.653528	-1.51861	-1.21735	0	9.346002	8.42527	0	p08201	Protein tran	2	1.95852				
SCP2 seq: 4	-0.77202	1.287777	-0.81932	-2.39866	4.0344	3.973603	3.291997	4.608781	p17907	Non-specifi	2	1.650819				
SCEL pS68	0	0	0	0	1.555729	1.231959	1.613001	1.895666	p14706	Sciellin	2	0.787044				
Scavenger r	-0.93501	-0.86646	-1.76467	-1.42992	2.540628	1.537847	1.877489	2.727606	p00901	Scavenger r	2	0.460939				
SCAF4 pS1	1.165837	1.540283	-0.40997	0.757763	1.898017	3.120945	3.316254	1.189899	p14350	Splicing fact	2	1.572378		AKTinh1 (Akt Inhibitor VIII);		
SBF1 seq: 1	-0.78052	-3.30912	1.02909	0.258216	0	0	7.60284	0	p03435	Myotubula	2	0.600063				
SARNP seq:	0	0	0	0	0	0	0	6.017603	p16750	SAP domain	2	0.7522				
SAP30L seq	0.272293	0.105276	0.1511	-0.1361	6.64697	8.596973	0	0	p12471	Histone dea	2	1.954564				
SAFB seq: 2	-1.66033	-1.5335	-2.66356	-3.42083	2.434086	2.008729	1.001421	1.092444	p20892	Scaffold att	2	-0.34269			Validated n	GO:0030520; P:intracellular estrogen receptor signaling pathway;
S100A4 sec	-0.55011	0.630215	-0.46006	-1.25033	5.888182	0	0	0	p17426	Protein S10	2	0.532237				GO:0043123; P:positive regulation of I-kappaB kinase/NF-kappaB signaling;
S100A14 se	-1.07111	-1.30209	-1.45185	-1.29384	5.440163	0	5.595943	5.813173	p20481	Protein S10	2	1.466299				GO:0034162; P:toll-like receptor 4 signaling pathway;
S100A11 se	-0.67211	0.200864	-0.53507	-0.97292	1.182362	0.527173	0.25009	0.773455	p13653	Protein S10	2	0.094232				GO:0007165; P:signal transduction;
RUVBL2 se	-1.88237	1.10552	-1.73319	-2.45928	2.970647	2.599266	1.440312	3.150574	p15739	RuvB-like 2	2	0.648935			C-MYC pathway; Integrin-linked kinase signaling; ATF-2 transcription factor network; Validated targets of C-MYC transcriptional activation; Regulation of nuclear beta catenin signaling and target gene transcription;	
RUVBL1 sec	0.582709	0.86413	-1.33881	-1.86415	1.762444	2.028112	1.486149	2.462563	p18000	RuvB-like 1	2	0.747893			C-MYC pathway; Integrin-linked kinase signaling; Validated targets of C-MYC transcriptional activation;	
RUVBL1 sec	2.402866	4.134027	0.160361	-6.0948	2.172291	2.925824	2.168769	3.011957	p17743	RuvB-like 1	2	1.360162			C-MYC pathway; Integrin-linked kinase signaling; Validated targets of C-MYC transcriptional activation;	
RUVBL1 sec	0.438725	1.411758	-0.89948	-0.88446	0.818147	1.35812	1.257777	1.393721	p15109	RuvB-like 1	2	0.611788			C-MYC pathway; Integrin-linked kinase signaling; Validated targets of C-MYC transcriptional activation;	
RUVBL1 sec	-0.19541	1.037554	-1.65366	-4.44009	1.120054	0.97122	0.127982	0.712293	p08875	RuvB-like 1	2	-0.29001			C-MYC pathway; Integrin-linked kinase signaling; Validated targets of C-MYC transcriptional activation;	
RUVBL1 sec	-1.22627	0.471763	-0.71827	-1.4668	1.527885	1.255607	0.55655	1.444456	p15738	RuvB-like 1	2	0.230616			C-MYC pathway; Integrin-linked kinase signaling; Validated targets of C-MYC transcriptional activation;	
RPSA seq: 9	-0.72974	0.829993	-1.74197	-0.7584	3.262296	0.921579	1.735325	1.618799	p16882	40S riboson	2	0.642236				
RPSA seq: 6	-0.33517	0.41406	-0.3317	-0.71619	1.119541	1.052793	0.562342	1.098478	p20417	40S riboson	2	0.358019				
RPSA seq: 1	-0.99506	-1.13791	-1.20305	-1.06329	1.256495	1.76879	0.738909	0.799346	p11249	40S riboson	2	0.020529				
RPS8 seq: 1	-0.58813	0.847233	-0.94795	-1.52129	2.184439	1.904427	1.475854	2.617626	p17158	40S riboson	2	0.746525				
RPS8 seq: 1	-0.90953	-0.1431	-0.88877	-1.14983	1.515601	1.037377	-0.10385	0.239029	p20917	40S riboson	2	-0.05038				
RPS7 seq: 4	-0.52239	0.23569	-1.0613	-1.14293	1.638886	1.083556	0.099669	1.175703	p17440	40S riboson	2	0.188361				
RPS6KL1 se	-1.57884	0.29019	-0.92368	-0.79103	1.387813	0.914717	0.325263	1.138601	p14500	Ribosomal p	2	0.220378				
RPS6KA3 se	0.108909	-0.52378	-0.08833	-0.31282	3.185518	0	0	2.293639	p02381	Ribosomal p	2	0.582892			Coregulatio	GO:0051403; P:stress-activated MAPK cascade; GO:0034166; P:toll-like receptor 10 signaling pathway; GO:0034146; P:toll-like receptor 5 signaling pathway; GO:0038124; P:toll-like receptor TLR6:TLR2 signaling pathway; GO:0038123; P:toll-like receptor TLR1:TLR2 signaling pathway; GO:0034162; P:toll-like receptor 9 signaling pathway; GO:0034134; P:toll-like receptor 2 signaling
RPS6 seq: 2	1.012889	-0.02134	0.911865	1.051533	2.515596	2.042976	2.977805	2.169192	p10653	40S riboson	2	1.582565			IL2 signalin	GO:0031929; P:TOR signaling; GO:0008286; P:insulin receptor signaling pathway;
RPS6 seq: 2	0.008241	-0.22195	-1.37906	0.020627	2.957967	2.680649	2.173152	2.823343	p10655	40S riboson	2	1.132871	RSK2; PKCD; p90RSK; p		IL2 signalin	GO:0031929; P:TOR signaling; GO:0008286; P:insulin receptor signaling pathway;
RPS6 seq: 2	-0.24713	-0.06531	-1.55891	-0.40702	3.144466	2.83482	2.279851	3.048714	p07084	40S riboson	2	1.128685	RSK2; PKCD; p90RSK; p		IL2 signalin	GO:0031929; P:TOR signaling; GO:0008286; P:insulin receptor signaling pathway;
RPS6 seq: 1	-0.96756	0.648317	-1.35657	-1.42694	1.373062	0.943263	0.398916	1.314699	p18734	40S riboson	2	0.115899			IL2 signalin	GO:0031929; P:TOR signaling; GO:0008286; P:insulin receptor signaling pathway;
RPS6 pS24	0.602629	0.200721	0.603048	1.090578	2.614937	2.379137	3.194382	2.675557	p10656	40S riboson	2	1.670124	p70S6K;		IL2 signalin	GO:0031929; P:TOR signaling; GO:0008286; P:insulin receptor signaling pathway;
RPS6 pS23	-0.12855	-0.39792	-1.32268	-0.03962	2.895486	2.526429	1.970419	2.645022	p10654	40S riboson	2	1.018573	RSK2; PKCD; p90RSK; p		IL2 signalin	GO:0031929; P:TOR signaling; GO:0008286; P:insulin receptor signaling pathway;
RPS6 pS23	0.173432	0.014982	-0.98953	0.322347	3.033095	2.726238	2.189105	3.11787	p10657	40S riboson	2	1.323443	RSK2; PKCD; p90RSK; p		IL2 signalin	GO:0031929; P:TOR signaling; GO:0008286; P:insulin receptor signaling pathway;
RPS6 pS23	-1.53459	0.322581	-4.03182	-1.60685	1.049437	1.085693	0.287757	0.915135	p06753	40S riboson	2	-0.43908	RSK2; PKCD; p90RSK; p		IL2 signalin	GO:0031929; P:TOR signaling; GO:0008286; P:insulin receptor signaling pathway;
RPS4Y2 seq	-0.6045	0.597115	-1.26991	-2.84964	1.565106	1.956012	1.348666	2.312187	p10547	40S riboson	2	0.38188				
RPS4Y2 seq	-0.81654	0.684443	-0.99735	-1.63636	2.853798	2.235049	0.266913	2.304829	p00192	40S riboson	2	0.611848				
RPS4X seq:	-1.63685	-2.16245	-2.24758	-1.52886	1.932351	0.987969	1.196184	1.799888	p14221	40S riboson	2	-0.20742				
RPS3A seq:	4.379317	4.927865	-4.85314	-4.67756	2.694069	2.437789	2.853552	2.665522	p01560	40S riboson	2	1.303427				
RPS3A seq:	-0.84886	0.1661	-0.73161	-1.70159	2.946857	2.380239	1.199053	2.599189	p11569	40S riboson	2	0.751172				
RPS3 seq: 2	0.150768	3.223248	-0.51157	-7.46936	1.788056	1.36243	0.533049	1.93048	p16035	40S riboson	2	0.125888			Validated tr	GO:0008543; P:fibroblast growth factor receptor signaling pathway; GO:0007165; P:signal transduction;
RPS3 seq: 1	-0.56246	0.897452	-0.63049	-1.12384	2.445333	2.031085	1.067101	2.147051	p20821	40S riboson	2	0.783904			Validated tr	GO:0008543; P:fibroblast growth factor receptor signaling pathway; GO:0007165; P:signal transduction;
RPS26 seq:	-1.31238	-0.17765	-1.22645	-1.38361	1.041827	1.004985	0.8394	0.537511	p18728	40S riboson	2	-0.08455				
RPS25 seq:	-0.66778	-0.68516	-0.9321	-1.34478	1.738482	0.783808	0.617728	0.695973	p11543	40S riboson	2	0.025771				
RPS20 seq:	-0.9442	-0.59241	-1.13926	-1.21983	2.162516	1.634268	1.139113	1.982421	p02302	40S riboson	2	0.377827				
RPS20 seq:	-0.46807	-0.21335	-1.03013	-0.86745	1.020291	1.240686	0.553615	1.345747	p11611	40S riboson	2	0.197668				
RPS2 seq: 2	-0.32761	1.092386	-0.67835	-1.61082	1.587717	1.224047	0.364837	1.280433	p19386	40S riboson	2	0.36658			IGF1 pathw	GO:0060397; P:JAK-STAT cascade involved in growth hormone signaling pathway; GO:0007249; P:I-kappaB kinase/NF-kappaB signaling; GO:0000186; P:activation of MAPKK activity; GO:0000187; P:activation of MAPK activity; GO:0048015; P:phosphatidylinositol-mediated signaling; GO:0008286; P:insulin receptor signaling pathway; GO:0008543; P:fibroblast growth factor recep
RPS19 seq:	-2.31577	0.05927	-2.54652	-2.58968	1.459392	0.000378	0.226715	1.424121	p11473	40S riboson	2	-0.53526				
RPS19 seq:	-0.98937	-0.09378	-2.42889	-1.12072	1.771816	0.484627	0.880957	1.198296	p14512	40S riboson	2	-0.03713				
RPS19 seq:	-1.95371	-0.83457	-1.38077	-3.27641	1.205482	1.020864	1.454273	1.22795	p01192	40S riboson	2	-0.31711				
RPS13 seq:	-0.91257	0.498768	-3.2005	-2.90993	4.719265	3.086429	2.784729	3.759983	p15639	40S riboson	2	0.978273				
RPS12 seq:	-2.86938	1.479205	-2.35373	-2.27435	2.23286	1.986915	0.422836	2.041973	p11794	40S riboson	2	0.083291				
RPS12 seq:	-0.46899	0.480915	-1.0729	-0.24539	1.642856	1.907703	0.701977	2.225435	p10443	40S riboson	2	0.646451				
RPS11 seq:	-1.52614	-2.01293	-3.70226	-3.02504	3.560535	1.412385	1.766447	1.98319	p13988	40S riboson	2	-0.19298				
RPS11 seq:	-1.36707	-0.1283	-1.43263	-1.40402	2.17256	1.633176	0.909457	1.105748	p17957	40S riboson	2	0.186115				
RPS11 seq:	-1.52145	-1.24249	-1.50178	-1.66782	1.230084	0.648521	0.19119	0.561009	p08094	40S riboson	2	-0.41284				
RPS11 seq:	-0.72381	-0.44413	-3.05878	-2.99336	0.380529	0.628953	0.90205	0.948195	p18093	40S riboson	2	-0.54504				
RPN1 seq: 5	-1.44605	-1.1042	-0.25732	-1.00177	1.797116	1.367743	0.701355	1.70731	p20131	Dolichyl-dip	2	0.220523				
RPLP2 pS1	-11.7198	0.125992	0.248055	0.34744	1.041264	0.557673	0.772316	1.132337	p18897	60S acidic r	2	-0.93684		Up with IGF - down with MEKinh2 (U0126); Up with IGF - down with MEKinh1 (GSK-1120212); Up with IGF - down with ERKinh2 (ERK inhibitor II); Up with IGF - down with ERKinh1 (ERK inhibitor Calbiochem); Up with IGF - down with EGFRinh2 (PD-153035); Up with IGF - down with EGFRinh1 (PD-168393); Up with IGF - down with CAMKinh2 (KN-62); Up with IGF - down with CAMKinh1 (KN-93); Up wi		
RPLP2 pS1	-1.0381	-1.46856	-1.51149	-1.11476	0.893205	0.791505	0.866496	0.780496	p13299	60S acidic r	2	-0.22515		Up with IGF - down with MEKinh2 (U0126); Up with IGF - down with MEKinh1 (GSK-1120212); Up with IGF - down with ERKinh2 (ERK inhibitor II); Up with IGF - down with ERKinh1 (ERK inhibitor Calbiochem); Up with IGF - down with EGFRinh2 (PD-153035); Up with IGF - down with EGFRinh1 (PD-168393); Up with IGF - down with CAMKinh2 (KN-62); Up with IGF - down with CAMKinh1 (KN-93); Up wi		
RPLP1 seq:	-0.59745	-0.19814	-1.07596	-1.29319	1.091925	1.853531	1.931807	2.210139	p21134	60S acidic r	2	0.490335				
RPLP0P6 se	-0.50508	0.612501	-0.47947	-1.0172	1.39127	1.24264	1.018423	1.464028	p14142	60S acidic r	2	0.465888				
RPL8 seq: 3	-4.14691	-1.01931	-1.37033	-3.71739	3.257061	2.430384	2.157909	1.957118	p19264	60S riboson	2	-0.05643				
RPL8 seq: 1	-1.52159	0.244652	-2.6581	-0.95959	2.900889	1.050778	1.301294	1.512776	p19153	60S riboson	2	0.233889				
RPL7A seq:	-1.70075	-2.35064	-2.57905	-2.2586	2.565205	1.595964	1.544983	0.944776	p07815	60S riboson	2	-0.27976				
RPL7A seq:	-0.8227	0.384813	-1.39986	-1.9925	0.689163	0.787015	0.12856	1.028627	p20543	60S riboson	2	-0.14961				
RPL7 seq: 1	-0.91813	-0.92227	-1.96526	-1.46097	1.591094	1.364817	0.486649	0.650628	p16962	60S riboson	2	-0.14668				
RPL7 seq: 1	-0.36993	0.756984	-0.85342	-1.35187	1.417195	1.046397	-0.59631	1.175658	p16963	60S riboson	2	0.153088				
RPL6 seq: 8	-1.37461	-0.10493	-3.22092	-2.27651	4.027214	3.735781	2.326586	4.120698	p01270	60S riboson	2	0.904164				GO:0016055; P:Wnt signaling pathway; GO:0007264; P:small GTPase mediated signal transduction;
RPL6 seq: 2	-0.79919	0.662314	-2.71497	-2.0486	3.89396	1.935285	2.206672	2.279226	p20443	60S riboson	2	0.676837				GO:0016055; P:Wnt signaling pathway; GO:0007264; P:small GTPase mediated signal transduction;
RPL6 seq: 2	-1.04814	-1.0956	-1.58714	-1.21897	2.07692	1.832572	0.740449	1.814792	p19261	60S riboson	2	0.189362				GO:0016055; P:Wnt signaling pathway; GO:0007264; P:small GTPase mediated signal transduction;
RPL4 seq: 3	-1.4666	-1.67368	-2.70909	-1.49836	1.337401	0.651811	0.880246	1.426686	p21210	60S riboson	2	-0.38145				
RPL4 seq: 2	-0.95877	-1.1503	-1.32083	-1.07859	1.907516	1.194879	1.246462	1.384575	p08935	60S riboson	2	0.153118				
RPL4 seq: 1	-0.88523	-0.7401	-1.37142	-1.57587	3.03445	2.685392	1.635852	2.992991	p17902	60S riboson	2	0.722009				
RPL38 seq:	-2.05818	-1.17604	-1.89897	-3.22151	2.033883	2.940014	1.894398	3.468954	p18874	60S riboson	2	0.24782				
RPL36A sec	-1.22091	0.036041	-1.59983	-1.80099	1.443644	0.68747	0.56603	0.466492	p12006	60S riboson	2	-0.17776				
RPL35 seq:	-1.65924	-2.15962	-4.07133	-1.92871	2.378107	1.707035	1.938542	1.669149	p01128	60S riboson	2	-0.26576				
RPL30 seq:	-2.28815	-0.49401	-3.34842	-2.71732	2.36509	1.578992	1.26536	1.789135	p05248	60S riboson	2	-0.23117				
RPL29 seq:	-0.76465	0.501347	-2.20628	-0.74426	2.508215	0.589718	0.985572	1.303093	p19840	60S riboson	2	0.271595				
RPL28 seq:	-0.49059	1.02014	-0.76464	0.851217	2.736365	2.742056	1.002102	3.828171	p00206	60S riboson	2	1.365603				
RPL27A sec	-0.67558	-1.35679	-0.87538	-1.11304	1.569389	0.689628	0.679416	0.713887	p02828	60S riboson	2	-0.04606				
RPL27 seq:	-0.04717	0.467906	-4.8084	-1.32233	2.617158	1.386774	0.868848	2.038884	p12391	60S riboson	2	0.150209				
RPL26L1 se	-1.86468	0.726398	-1.26843	-2.36242	1.604188	1.624766	-0.25388	1.508077	p00023	60S riboson	2	-0.03575				
RPL24 seq:	-1.94562	0.531758	-1.26262	-2.07333	2.378598	2.0314	1.23477	2.301356	p20426	60S riboson	2	0.399538				
RPL23A sec	-0.5984	0.801594	-0.45916	-1.25988	1.688523	1.227571	0.419912	1.513489	p10309	60S riboson	2	0.416707				
RPL23 seq:	-1.39658	-1.62752	-2.14022	-1.56899	2.764448	1.328003	1.153953	1.722	p15314	60S riboson	2	0.029387			p53 pathway;	
RPL22 seq:	-0.43222	0.604725	-1.24896	-1.53085	2.780085	2.505654	0.73943	2.750838	p13573	60S riboson	2	0.771088				
RPL22 seq:	-0.80541	0.28365	-0.60701	-1.3196	1.440976	0.901433	-0.0661	1.371038	p20609	60S riboson	2	0.149872				
RPL21 seq:	-0.8352	-1.29499	-1.69159	-1.32347	1.014459	0.566749	0.035705	1.263945	p00524	60S riboson	2	-0.28305				
RPL18 seq:	-0.60412	0.236943	-0.78582	-1.11504	1.21781	1.439348	0.952763	0.922933	p13975	60S riboson	2	0.283102				
RPL17 seq:	-1.15716	0.835652	-1.26351	-1.56985	2.594619	1.10957	0.887744	1.220426	p08785	60S riboson	2	0.332186				GO:0007264; P:small GTPase mediated signal transduction;
RPL13 seq:	-0.86822	0.375222	-0.96683	-1.18717	1.517596	1.004605	0.269442	0.642267	p02711	60S riboson	2	0.098364				
RPL13 seq:	-0.82769	-0.73899	-1.75802	-1.26302	1.261267	1.060332	0.655282	0.555752	p16093	60S riboson	2	-0.13189				
RPL12 seq:	-1.16524	-1.02752	-1.22334	-1.98172	1.317129	1.165124	1.087786	1.037586	p20240	60S riboson	2	-0.09877				
RPL12 seq:	-0.94504	-0.44749	-1.35686	-2.68602	2.28873	2.895127	1.772486	3.117449	p08833	60S riboson	2	0.579797				
RPL12 seq:	-1.82704	-1.72766	-2.70143	-1.90599	1.81638	0.738934	1.041549	0.630832	p14249	60S riboson	2	-0.4918				
RPL12 seq:	-1.89668	-0.08267	-1.90141	-1.67862	2.22388	1.313027	0.499339	2.252533	p19011	60S riboson	2	0.091176				
RPL11 seq:	-0.27054	0.37464	-0.92532	-1.39253	2.160694	0.572569	0.893303	2.109935	p01238	60S riboson	2	0.440343			p53 pathw	GO:0007264; P:small GTPase mediated signal transduction;
RPL11 seq:	-0.61363	0.658858	-0.70045	-1.08657	1.711309	0.643407	0.490462	0.502851	p00427	60S riboson	2	0.200781			p53 pathw	GO:0007264; P:small GTPase mediated signal transduction;
RPL10 seq:	-0.44081	0.597152	-0.95328	-1.23259	2.823195	2.299654	0.96238	2.0591	p11570	60S riboson	2	0.76435				GO:0007264; P:small GTPase mediated signal transduction;
RNH1 seq:	-0.40032	0.561688	-1.08804	-1.12749	1.654679	1.013414	0.55892	0.56664	p18646	Ribonucleas	2	0.214936				
RHOC seq:	-0.95085	0.269291	-1.26338	-1.21135	1.943363	1.683895	-0.69312	2.277101	p16992	Rho-related	2	0.256869			Signaling ev	GO:0051056; P:regulation of small GTPase mediated signal transduction; GO:0043123; P:positive regulation of I-kappaB kinase/NF-kappaB signaling; GO:0007264; P:small GTPase mediated signal transduction;
RGPD3 pT2	1.367617	1.64292	0.957872	2.928164	3.740791	3.909049	3.660013	5.003284	p18261	RanBP2-like	2	2.897053				
RGCC seq:	0	0	0	0	1.485365	1.740423	0.675862	1.530883	p06626	Regulator o	2	0.679067			Direct p53 effectors;	
RERE seq: 1	-0.27319	0.584394	0.615393	0.640202	0.279824	2.002872	2.145915	2.656931	p04329	Arginine-glu	2	1.081542				
REPS2 pS2	0.356632	0.328565	0.625365	0.501185	1.975681	1.871981	1.798274	1.442985	p05808	RalBP1-ass	2	1.112583				GO:0007173; P:epidermal growth factor receptor signaling pathway;
RCN1 seq: 1	-1.01425	0.179665	-1.6203	-1.61397	2.081308	1.485788	0.825924	2.175549	p00303	Reticulocal	2	0.312464				
RBMX seq:	-0.76127	0.447701	-0.63632	-0.8576	1.478469	1.072483	0.149981	1.249806	p01604	RNA-bindin	2	0.267906				
RBMX seq:	-0.95706	0.651008	-0.99685	-2.12906	3.252932	2.682541	2.088489	3.058416	p20289	RNA-bindin	2	0.956303				
RBM43 seq	-0.68266	-1.02936	-0.89666	-1.34239	1.995751	1.002996	2.083473	1.752745	p01818	RNA-bindin	2	0.360487				
RBM39 seq	0.591129	0.772227	-0.80923	-1.0339	0.756149	1.901421	1.966601	1.68716	p18631	RNA-bindin	2	0.728945				
RBM34 pS1	-2.19044	0.022063	-3.44866	-0.98092	0.504429	0.832037	-0.00354	0.503689	p03295	RNA-bindin	2	-0.59517				
RBM33 pS	-0.24095	-0.72203	-0.79666	-0.44012	1.677299	1.480413	0.810832	0.879955	p01307	RNA-bindin	2	0.331093				
RBM27 pS1	-0.33094	0.523064	2.37482	0.649521	2.092194	2.151481	3.043629	2.429797	p12339	RNA-bindin	2	1.616696				
RBM15B p	0.079067	0.13913	-1.02325	-0.8483	1.885174	1.701254	2.586395	1.89498	p02050	Putative RN	2	0.801806				
RBBP7 seq:	-3.0998	0.823323	-0.93126	-1.66226	2.965911	2.546593	1.670142	2.778854	p01986	Histone-bin	2	0.636438			Hedgehog signaling events mediated by Gli proteins; Signaling events mediated by HDAC Class I; Regulation of Telomerase; Regulation of nuclear SMAD2/3 signaling;	
RBBP4 seq:	-0.65094	0.762149	-2.41344	-1.40946	1.482159	1.023311	-0.5669	1.502796	p18856	Histone-bin	2	0.003709			Hedgehog signaling events mediated by Gli proteins; Regulation of retinoblastoma protein; Signaling events mediated by HDAC Class I; Regulation of Telomerase; E2F transcription factor network; Regulation of nuclear SMAD2/3 signaling;	
RBBP4 seq:	-1.10479	1.271894	-2.14965	-1.63568	2.228153	1.628183	1.150294	2.065511	p11143	Histone-bin	2	0.431739			Hedgehog signaling events mediated by Gli proteins; Regulation of retinoblastoma protein; Signaling events mediated by HDAC Class I; Regulation of Telomerase; E2F transcription factor network; Regulation of nuclear SMAD2/3 signaling;	
RASGRF2 se	0.356632	0.328565	0.625365	0.501185	1.975681	1.871981	1.798274	1.442985	p00440	Ras-specific	2	1.112583			Regulation	GO:0051056; P:regulation of small GTPase mediated signal transduction; GO:0048011; P:neurotrophin TRK receptor signaling pathway; GO:0007264; P:small GTPase mediated signal transduction;
RASGRF2 se	0.356632	0.328565	0.625365	0.501185	1.975681	1.871981	1.802544	1.442985	p00441	Ras-specific	2	1.113117			Regulation	GO:0051056; P:regulation of small GTPase mediated signal transduction; GO:0048011; P:neurotrophin TRK receptor signaling pathway; GO:0007264; P:small GTPase mediated signal transduction;
RASA4 seq:	-0.43594	-0.44469	-0.65936	-0.58993	2.451467	1.931595	0.934066	0.932941	p01564	Ras GTPase	2	0.515019			Regulation	GO:0046580; P:negative regulation of Ras protein signal transduction; GO:0035556; P:intracellular signal transduction;
RAP1GAP s	1.190924	0.415577	0.554761	0.635939	0	5.281222	0	5.777429	p07515	Rap1 GTPas	2	1.731982				GO:0007165; P:signal transduction;
RANBP1 se	-1.69131	0.395871	-0.96559	-1.34946	1.95455	1.754766	-0.26213	1.800244	p16845	Ran-specific	2	0.204617			E2F transcri	GO:0007165; P:signal transduction;
RAN seq: 1	-0.79212	-0.96454	-0.66857	-1.16345	2.614072	2.020068	1.090259	2.135606	p10390	GTP-bindin	2	0.533915			Sumoylatio	GO:0030521; P:androgen receptor signaling pathway; GO:0007264; P:small GTPase mediated signal transduction; GO:0007165; P:signal transduction;
RALY seq: 5	-0.00547	1.044915	-8.71655	-0.89937	4.74489	3.399414	1.96164	3.857986	p11032	RNA-bindin	2	0.673433				
RALY seq: 1	-0.58901	-0.30042	-0.84367	-0.90255	1.819004	0.709284	0.856297	1.803156	p04843	RNA-bindin	2	0.319012				
RALGAPA1	-0.02088	-0.957	-0.04661	-0.62833	1.935635	2.313355	3.257272	1.936382	p08420	Ral GTPase-	2	0.973727		AKTinh1 (Akt Inhibitor VIII);		
RAD23B se	-2.65342	0.068384	-0.4801	-2.51075	1.142837	0.095034	0.539098	1.451304	p19622	UV excision	2	-0.29345				
RAD23B se	-0.18854	-0.79562	-0.36343	-0.33156	1.539461	1.154502	0.875324	1.683467	p17259	UV excision	2	0.446701				
RABGEF1 p	-0.89467	-0.96425	-0.35737	-0.63865	0.491712	1.25733	1.929697	2.597254	p19896	Rab5 GDP/	2	0.427631			Regulation of Ras family activation;	
RAB3IP seq	-0.30924	-0.43806	0.155211	-0.0405	1.268843	0.263074	0.037164	2.788474	p03438	Rab-3A-inte	2	0.465621				
RAB28 seq:	-0.79212	-0.96454	-0.66857	-0.87603	2.62545	2.031445	1.101636	2.146983	p13518	Ras-related	2	0.575531				GO:0007264; P:small GTPase mediated signal transduction;
RAB11FIP1	1.710275	1.474319	-0.42488	-10.0996	0	0	7.113605	8.9644	p04579	Rab11 fami	2	1.09227				
QPRT seq: 1	-1.14835	-0.97402	-1.58184	-1.13787	0	0	5.946063	5.580963	p00362	Nicotinate-	2	0.835618				
PURA seq:	-0.21354	2.101306	-0.42536	-4.43187	1.785612	2.769508	-1.58022	2.902239	p06033	Transcriptio	2	0.363459				
PUF60 seq:	-2.7156	0.864601	-0.7559	-4.49997	1.903347	1.500148	0.061953	2.186441	p03352	Poly(U)-bin	2	-0.18187				
PTMS seq: 5	-0.32812	0.556598	-0.60486	-0.88253	1.727993	0.960212	0.205147	1.258199	p03374	Parathymos	2	0.361581				
PTCD3 seq:	2.688025	1.673441	0.184917	0.170098	2.00911	2.915153	2.245097	3.073155	p19130	Pentatricop	2	1.869875				
PTBP1 seq:	0.976713	1.912726	-7.81468	0.589647	0	0	4.913219	0	p09634	Polypyrimic	2	0.072203				
PSPC1 seq:	-1.20472	1.017361	-2.58265	-4.21154	3.593222	2.023438	-1.0952	2.623892	p20827	Paraspeckle	2	0.020476				
PSME1 seq	-0.80749	0.384855	-0.99601	-1.41886	2.364709	0	0.651605	3.294691	p08129	Proteasome	2	0.434187				
PSMD7 seq	3.280326	4.722357	-0.29573	-6.90471	1.700797	1.469326	0.255992	0.309376	p03221	26S proteas	2	0.567217				
PSMD7 seq	-0.99502	0.564579	-1.05157	-3.23531	3.929163	3.249811	0	3.43489	p18147	26S proteas	2	0.737068				
PSMD2 seq	-1.09676	-0.10082	-1.82715	-1.92031	1.418805	1.088707	0.647287	1.230485	p16756	26S proteas	2	-0.06997				
PSMD11 se	-0.7355	0.330384	-0.76119	-0.79231	1.622425	1.050879	0.301787	0.547829	p00643	26S proteas	2	0.195538				
PSMD1 seq	-1.29372	-0.7279	-2.11408	-3.09135	2.214148	1.624131	0.392004	0.611418	p11985	26S proteas	2	-0.29817				
PSMC5 pM	-2.10054	0.853966	-3.7374	-2.71386	4.73067	1.906192	1.76684	1.87801	p01370	26S proteas	2	0.322985				
PSMC4 seq	-1.59141	0.540479	-0.62182	-1.26615	0.655839	2.954825	3.832459	2.912185	p07453	26S proteas	2	0.92705				
PSMC4 seq	-1.03667	-0.85855	-1.90037	-1.77846	3.336843	2.79082	0.612635	2.856889	p00621	26S proteas	2	0.502892				
PSMC3 seq	-0.92061	0.547573	-2.31125	-3.84597	3.28186	2.416998	1.092143	3.101251	p11442	26S proteas	2	0.420249				
PSMC3 seq	-0.12025	1.795484	-0.94919	-1.44129	1.915627	2.622189	2.289557	3.228515	p01681	26S proteas	2	1.167581				
PSMC3 seq	0	3.526344	0	0	0	5.432124	0	7.385475	p12256	26S proteas	2	2.042993				
PSMC2 seq	1.869406	1.544402	-2.00654	0.004058	0.968025	1.455988	1.888897	1.739585	p07744	26S proteas	2	0.932978				
PSMC2 seq	-0.48086	-0.71074	-0.96574	-1.56204	2.865463	2.418403	1.366604	2.463925	p01393	26S proteas	2	0.674377				
PSMC2 seq	-1.27496	0.236642	-1.56837	-1.91004	1.909779	2.174216	0.649365	2.159259	p20311	26S proteas	2	0.296986				
PSMB6 seq	-0.50664	0.441004	-0.85765	-1.32321	2.661833	1.753152	0.922584	1.745306	p07722	Proteasome	2	0.604548				
PSMB6 seq	-0.44761	-0.45536	-1.01496	-1.4624	7.042314	0	0	6.809127	p07721	Proteasome	2	1.308889				
PSMB1 seq	-8.97449	1.68645	0.064248	-0.66918	2.591298	3.534252	3.408179	2.956651	p18117	Proteasome	2	0.574675				GO:0035556; P:intracellular signal transduction;
PSMA7 seq	-1.16841	0.687264	-0.4734	-1.08669	4.062881	3.92769	2.23879	3.922608	p14013	Proteasome	2	1.513843				
PSMA7 seq	-1.61658	0.508182	-0.86846	-2.05916	0.707525	2.393671	-0.63818	2.221537	p08906	Proteasome	2	0.081068				
PSMA6 seq	-1.37465	0.937515	-1.36485	-0.97792	2.357872	1.715093	0	2.137696	p00419	Proteasome	2	0.428845				
PSMA6 seq	-2.39288	-1.85898	-1.14137	-1.36211	2.282866	3.360121	2.397624	3.792926	p11441	Proteasome	2	0.634774				
PSMA6 seq	-1.12518	-1.80628	-2.20759	-0.92449	3.765474	2.854012	2.696088	2.933461	p20451	Proteasome	2	0.773186				
PSMA5 seq	-1.2583	0.859471	-0.72621	-1.05005	1.602098	1.298697	0.538206	1.69418	p13581	Proteasome	2	0.369761				
PSMA5 seq	-1.44572	-1.99661	-1.32845	-1.17309	2.14897	0.756894	1.47333	1.086816	p03852	Proteasome	2	-0.05973				
PSMA4 seq	-0.58699	-0.58078	-0.81126	-0.959	1.159022	0.807526	0.740608	0.907845	p02048	Proteasome	2	0.084621				
PSMA4 seq	-0.34331	0.672006	-0.59173	-1.28787	1.342033	2.231872	0.483739	2.130562	p11458	Proteasome	2	0.579663				
PSMA3 seq	-0.55399	0.307578	-0.79277	-1.40059	2.345481	1.866323	0.64703	1.903378	p19252	Proteasome	2	0.540306			Aurora B signaling;	
PSMA1 seq	-1.62406	-0.52907	-2.03796	-2.20827	2.146838	1.221778	0.404855	1.576206	p17057	Proteasome	2	-0.13121				
PSAP seq: 4	-0.59835	0.781099	-0.96783	-1.37376	4.467527	4.106111	0.479477	3.447826	p16237	Prosaposin	2	1.292762				GO:0043408; P:regulation of MAPK cascade;
PSAP seq: 4	-0.6035	-0.00766	-0.65804	-1.03504	1.704367	2.696451	1.636871	3.074911	p08740	Prosaposin	2	0.851045				GO:0043408; P:regulation of MAPK cascade;
PSAP seq: 2	-0.36937	1.808062	-1.79399	-1.57477	2.136374	0.791668	0.347853	2.50305	p11719	Prosaposin	2	0.481109				GO:0043408; P:regulation of MAPK cascade;
PRRC2B pS	0.366147	0.355396	0.029223	0.073678	1.047927	2.273034	1.662654	0.635657	p08011	Protein PRR	2	0.805465				
PROSC seq:	-1.31754	-0.07539	-0.57253	-2.95446	1.603173	1.964004	1.858747	1.3193	p19970	Proline syn	2	0.228161				
PRKRA pS1	-0.09145	0.548878	0.070951	0.33133	1.183191	2.700511	1.765251	1.999532	p17867	Interferon-i	2	1.063525			Ceramide signaling pathway;	
PRKCSH se	-0.89253	-0.62727	-0.97897	-1.32669	0.708452	0.652288	0.753111	1.763145	p05180	Glucosidase	2	0.006442				GO:0035556; P:intracellular signal transduction;
PRKCSH se	-0.42737	1.10382	-0.36736	-1.05514	1.680741	0.37272	0.705039	1.58039	p10327	Glucosidase	2	0.449106				GO:0035556; P:intracellular signal transduction;
PRKCSH se	0.310555	1.427331	-0.96234	-6.79964	1.601324	1.667901	0.794181	1.703686	p05267	Glucosidase	2	-0.03212				GO:0035556; P:intracellular signal transduction;
PRKCSH se	-1.48256	-0.87192	-1.80149	-1.08775	3.361007	2.261694	1.927521	3.281064	p11604	Glucosidase	2	0.698447				GO:0035556; P:intracellular signal transduction;
PRKCSH se	-2.46617	-1.04357	-2.68488	-3.65923	2.740791	2.386171	1.794927	2.745646	p14036	Glucosidase	2	-0.02329				GO:0035556; P:intracellular signal transduction;
PRKCD seq	0.46554	0.656345	-0.09057	0.509577	4.769041	0	0	6.582069	p10588	Protein kin	2	1.6115			IGF1 pathw	GO:0046627; P:negative regulation of insulin receptor signaling pathway; GO:0060333; P:interferon-gamma-mediated signaling pathway; GO:0008543; P:fibroblast growth factor receptor signaling pathway; GO:0007173; P:epidermal growth factor receptor signaling pathway; GO:0038096; P:Fc-gamma receptor signaling pathway involved in phagocytosis; GO:0048011; P:neurotr
PRKAR2A p	1.250821	-0.5583	1.206683	0.49718	0	0	8.33085	7.328967	p06425	cAMP-depe	2	2.257026				GO:0034199; P:activation of protein kinase A activity; GO:0009967; P:positive regulation of signal transduction; GO:0008543; P:fibroblast growth factor receptor signaling pathway; GO:0007173; P:epidermal growth factor receptor signaling pathway; GO:0048011; P:neurotrophin TRK receptor signaling pathway; GO:0035556; P:intracellular signal transduction; GO:0007165; P:sig
PRDX6 seq	-0.94008	-1.07246	-1.7692	-1.30358	2.195031	1.338984	0.659303	2.91458	p12218	Peroxiredox	2	0.252822				
PRDX5 seq	1.634727	1.693969	-0.65071	-1.21679	0.703931	2.415646	1.696335	2.319274	p01517	Peroxiredox	2	1.074548				
PRDX5 seq	-0.37941	0.503874	-0.67022	-1.02242	3.480202	3.221382	2.420971	3.594281	p11470	Peroxiredox	2	1.393582				
PRDX5 seq	-0.94279	-0.09219	-1.049	-1.38401	1.273982	1.383666	0.882583	2.016504	p02753	Peroxiredox	2	0.261094				
PRDX4 seq	-0.51718	0.55497	-0.36964	-0.71231	1.726155	1.395035	0.927	1.93188	p03386	Peroxiredox	2	0.616988				
PRDX3 seq	-1.06894	-0.09908	-1.05988	-1.32399	1.338304	2.673531	0	1.837869	p14725	Thioredoxin	2	0.287226			Validated targets of C-MYC transcriptional activation;	
PRDX3 seq	-0.67209	0.023535	-0.90921	-1.00643	3.634985	3.673048	3.638242	4.001014	p18042	Thioredoxin	2	1.547888			Validated targets of C-MYC transcriptional activation;	
PRDX2 seq	-0.87159	-1.0515	-1.1566	-0.93371	2.29618	1.817576	1.263897	2.21017	p19410	Peroxiredox	2	0.446801				
PRDX2 seq	-1.10458	-0.95701	-1.47481	-1.32855	1.33159	1.272651	0.512966	0.475545	p15752	Peroxiredox	2	-0.15902				
PRDX1 seq	-0.5513	0.790633	-0.62893	-0.85132	2.323429	1.434009	0.765414	1.861002	p19412	Peroxiredox	2	0.642868			Coregulation of Androgen receptor activity;	
PRDX1 seq	-0.58987	0.640571	-0.22715	-0.77222	2.14086	0.855442	0.909767	2.048723	p08482	Peroxiredox	2	0.625764			Coregulation of Androgen receptor activity;	
PRDX1 seq	-0.5819	-0.49047	-0.86316	-0.8489	1.669908	0.455135	0.430466	0.500537	p02729	Peroxiredox	2	0.033952			Coregulation of Androgen receptor activity;	
PPRC1 seq:	0	0	0	0	2.308915	1.682828	-0.21736	1.771058	p16937	Peroxisome	2	0.69318				
PPP6R3 sec	0.488247	2.034234	0.27438	-0.27703	2.410442	2.356495	0.100387	2.818189	p03322	Serine/thre	2	1.275668				
PPP4R3A s	0.658889	-0.08206	0.515207	0.337802	1.985357	2.769898	2.525115	2.195943	p18394	Serine/thre	2	1.363268				
PPP4R3A s	0.471564	0.264311	0.194149	0.683234	6.113276	0	0	7.603574	p18393	Serine/thre	2	1.916263				
PPP2R1A s	1.416591	2.273045	-2.57953	-6.80649	2.721616	3.287153	3.57422	3.278622	p00211	Serine/thre	2	0.895654			IL8- and CX	GO:0030111; P:regulation of Wnt signaling pathway; GO:0000188; P:inactivation of MAPK activity; GO:0008543; P:fibroblast growth factor receptor signaling pathway;
PPP1R13L	0.24653	-1.09857	-0.08996	0.130208	3.119468	1.977914	2.164988	0	p07333	RelA-associ	2	0.806321		p70S6Kinh	p53 pathway;	
PPP1R12C	0.008173	-0.32367	-0.1223	-0.1416	0.868644	2.379278	0.183702	2.379453	p02777	Protein pho	2	0.65396				
PPM1G sec	-0.64784	0.323095	-0.94029	-0.88955	1.234319	0.530142	0.337031	0.902219	p19095	Protein pho	2	0.10614				
PPIG seq: 7	0.301095	0.290645	0.05159	0.068763	2.008018	1.719895	2.19574	1.990017	p13259	Peptidyl-pro	2	1.07822				
PPIG seq: 3	-1.83281	-0.62713	-1.48102	-0.79009	2.069451	3.049583	3.863144	2.883865	p09916	Peptidyl-pro	2	0.891874				
PPIB seq: 7	-0.27111	0.476119	-0.42865	-0.71342	1.273927	0.731128	0.970094	0.792622	p01974	Peptidyl-pro	2	0.353839			Syndecan-1-mediated signaling events;	
PPIB seq: 4	-0.7371	-0.92462	-1.05058	-0.94901	1.763475	0.711305	0.777751	0.792103	p00535	Peptidyl-pro	2	0.047915			Syndecan-1-mediated signaling events;	
PPIB seq: 1	-1.46319	0.772565	-0.70731	-0.7638	2.682118	3.615453	1.43783	3.902602	p18162	Peptidyl-pro	2	1.184533			Syndecan-1-mediated signaling events;	
PPIB pM17	0.073319	-0.52732	-0.54249	-0.45339	0.929041	1.514137	0.327657	1.454239	p01241	Peptidyl-pro	2	0.346899			Syndecan-1-mediated signaling events;	
PPIAL4G se	0.169158	-0.38554	-0.1051	-1.93392	3.485019	3.730673	4.259649	5.063592	p15309	Peptidyl-pro	2	1.785441				
PPIA seq: 9	-8.64586	2.559559	0.172089	-1.16347	0.05103	2.663236	2.881274	3.954233	p14588	Peptidyl-pro	2	0.309012				
PPIA seq: 8	-0.53413	0.934062	-0.41306	-0.56384	1.553277	0.432573	0.517	1.394403	p16803	Peptidyl-pro	2	0.415036				
PPIA seq: 7	-0.42247	0.323782	-0.72651	-0.79855	0.709339	0.791906	0.681521	1.197306	p05438	Peptidyl-pro	2	0.219542				
PPIA seq: 7	-0.61077	-0.34987	-1.18876	-0.63008	1.719206	2.247837	2.210816	2.935502	p05439	Peptidyl-pro	2	0.791736				
PPIA seq: 2	-0.81329	-0.44948	-1.25671	-1.60136	0.536079	1.091118	0.987057	1.565428	p00866	Peptidyl-pro	2	0.007356				
PPIA seq: 2	-0.42432	-0.32955	-0.88175	-0.96229	1.252948	1.463595	0.558914	0.982797	p00865	Peptidyl-pro	2	0.207541				
PPIA seq: 1	-0.45983	-0.73655	-0.63266	-1.16537	0.93028	1.245992	1.509945	1.057283	p01323	Peptidyl-pro	2	0.218637				
PPIA pM61	0.320798	-1.18688	-0.03119	-1.29339	1.224762	2.007106	1.162301	2.219682	p14148	Peptidyl-pro	2	0.552899				
PPIA pM13	-1.65268	0.643426	-1.17718	-1.36172	2.241676	1.646636	0.903447	1.806918	p17713	Peptidyl-pro	2	0.381316				
PPFIBP2 se	0.230502	-0.03755	0.06281	-0.062	4.081723	2.536628	-0.51733	1.75212	p06498	Liprin-beta-	2	1.005862				
POTEE seq:	-0.60508	0.212468	-0.73301	-0.9203	1.047707	0.724004	0.194366	0.850549	p20676	POTE ankyri	2	0.096339				
POLR3E pS	0.588064	-0.07177	-0.89809	-1.00358	1.348341	1.566538	1.657719	1.008265	p14117	DNA-direct	2	0.524436			RAC1 signal	GO:0038096; P:Fc-gamma receptor signaling pathway involved in phagocytosis;
POLR2I seq	1.251486	2.880995	-0.25013	-2.37524	1.447944	2.387651	2.796928	2.077206	p13612	DNA-direct	2	1.277106				
POLH pS38	0.691788	-0.14691	0.304343	0.758841	2.646167	2.507968	3.297477	2.788373	p07085	DNA polym	2	1.606007	RSK2; PKCD; p90RSK; p70S6K;			
POLH pS37	-0.72167	0.357514	-2.34983	-0.68897	0.880956	0.730839	0.151905	0.746683	p07086	DNA polym	2	-0.11157	RSK2; PKCD; p90RSK; p70S6K;			
POLD1 seq	-1.23377	-1.40947	-1.38168	-1.13573	2.316506	2.203697	0.75368	2.121438	p11411	DNA polym	2	0.279334				
PNPLA2 se	-0.25016	-0.29098	-0.45	-1.05753	4.681665	0	4.399593	0	p06450	Patatin-like	2	0.879073				
PMM2 seq	-0.98417	0.728788	-1.90231	-4.13556	6.410987	5.361774	0	0	p14218	Phosphoma	2	0.684938				
PML pS530	0	0	0	0	1.172238	0.748213	0.490091	1.886788	p04313	Protein PM	2	0.537166	ERK2; ERK1;		TGF-beta re	GO:0048384; P:retinoic acid receptor signaling pathway; GO:0007179; P:transforming growth factor beta receptor signaling pathway; GO:0060333; P:interferon-gamma-mediated signaling pathway; GO:0019221; P:cytokine-mediated signaling pathway;
PLIN3 seq:	-0.59458	0.893576	-0.54398	-1.96228	4.063835	4.023874	1.56471	5.124334	p11694	Perilipin-3	2	1.571186				
PLEKHJ1 se	-0.53404	0.138455	-0.22886	-0.92611	0.722323	0.790352	0.237815	1.056393	p17421	Pleckstrin h	2	0.157041				
PLEKHA6 p	0.056012	0.949764	1.119091	0.689803	6.823468	0	0	0	p04351	Pleckstrin h	2	1.204767				
PLEKHA5 p	-0.55092	-0.09193	-0.52476	-0.09123	1.410975	1.335494	1.089603	1.30292	p15682	Pleckstrin h	2	0.485019				
PLEKHA5 p	0.052623	-2.59976	0.528154	0.090522	3.7598	-2.18434	-0.13976	4.417213	p13542	Pleckstrin h	2	0.490556		ROCKinh1 (H-1152); PKCinh2 (Bisindolylmaleimide-1); PI3Kinh2 (PI-103); p70S6Kinh2 (DG2); EGFRinh2 (PD-153035); CAMKinh2 (KN-62); AKTinh2 (MK-2206);		
PLEC seq: 7	-2.49111	2.35145	-1.56549	-3.06675	3.502942	2.508909	0	1.923097	p06113	Plectin	2	0.395381				
PLEC seq: 4	-0.99262	-0.22897	-1.51571	-3.78512	1.807849	1.21071	0.603722	1.604994	p11439	Plectin	2	-0.16189				
PLEC seq: 4	-1.24734	1.254048	-1.02625	-2.3964	1.348411	0.60007	0.139907	1.188719	p04862	Plectin	2	-0.01735				

PLEC seq: 4	-0.83587	0.051057	-1.44131	-1.63739	1.814326	1.440915	-0.66117	1.175692	p19166	Plectin	2	-0.01172				
PLEC seq: 4	-1.90829	-0.64615	-0.74732	-2.02412	1.520405	0.832021	0.501551	0.985111	p18917	Plectin	2	-0.18585				
PLEC seq: 3	-1.77806	1.193187	-1.97849	-2.34455	2.807954	2.649878	1.237399	2.951277	p11461	Plectin	2	0.592324				
PLEC seq: 2	-0.56109	1.495342	-0.63944	-1.7081	1.455374	0.878691	-0.61966	0.870372	p17656	Plectin	2	0.146436				
PLEC seq: 2	-0.68152	-0.61387	-1.34609	-1.43348	2.246807	2.004396	0.240931	2.160084	p12200	Plectin	2	0.322157				
PLEC seq: 1	-0.47824	2.048137	-1.77887	-3.9363	0.950272	0.853854	-0.15051	1.745555	p16506	Plectin	2	-0.09326				
PLEC seq: 1	-1.06839	1.210092	-1.31229	-2.58621	2.048995	2.001794	0.734535	2.135697	p12269	Plectin	2	0.395529				
PLEC seq: 1	-0.90365	-0.84778	-2.05597	-1.22649	2.012208	1.579665	0.621912	1.943033	p19795	Plectin	2	0.140364				
PLEC seq: 1	-0.24645	1.252825	-1.38348	-3.36191	0.056904	0.173218	0.486418	0.948199	p17185	Plectin	2	-0.25928				
PLAA seq: 4	-2.63018	2.383164	0.401629	-0.9831	1.471489	1.24239	0.938981	1.428808	p16684	Phospholip	2	0.531647				GO:0007165; P:signal transduction;
PKP4 seq: 1	0.164856	-0.67151	1.595978	-0.29557	3.444868	2.834338	2.570075	5.083267	p03414	Plakophilin-	2	1.840788				GO:0007267; P:cell-cell signaling;
PKN1 seq: 7	-1.00829	-1.00467	0.294435	-0.50693	0	0	5.571417	6.751668	p02077	Serine/threo	2	1.262204	PKN1; PDK1	AKTinh2 (M	Signaling m	GO:0007165; P:signal transduction;
PKMYT1 pS	1.227109	0.995769	0.545867	0.630447	6.09449	5.367265	6.145852	0	p04868	Membrane-	2	2.62585				
PKM seq: 9	-0.40028	0.68593	-1.58882	-0.67566	0.508496	1.134599	1.154659	1.267196	p03008	Pyruvate kin	2	0.260765			HIF-1-alpha transcription factor network;	
PKM seq: 4	-1.20892	-1.08317	-3.57868	-2.38741	1.109886	0.987738	1.223027	1.533181	p01086	Pyruvate kin	2	-0.42554			HIF-1-alpha transcription factor network;	
PKM seq: 4	-2.89374	0.104988	-0.71566	-3.4111	1.27693	1.480966	-0.55687	1.568326	p08853	Pyruvate kin	2	-0.39327			HIF-1-alpha transcription factor network;	
PKM seq: 4	-1.05224	-1.65727	-1.90695	-0.94652	1.439586	0.798564	0.886539	0.847401	p19979	Pyruvate kin	2	-0.19886			HIF-1-alpha transcription factor network;	
PKM seq: 4	-0.54149	0.380677	-0.6834	-0.84421	0.924693	0.847974	0.660108	1.165822	p12215	Pyruvate kin	2	0.238772			HIF-1-alpha transcription factor network;	
PKM seq: 4	-0.45768	0.319587	-0.50834	-0.72634	0.969375	0.901348	0.743401	1.244614	p12216	Pyruvate kin	2	0.310746			HIF-1-alpha transcription factor network;	
PKM seq: 3	-0.45264	-0.35457	-0.7503	-0.59971	1.10386	1.415937	0.846813	1.732053	p16188	Pyruvate kin	2	0.36768			HIF-1-alpha transcription factor network;	
PKM seq: 3	1.664911	1.43034	-1.24416	-3.62311	1.669866	2.922686	4.063115	2.986867	p20830	Pyruvate kin	2	1.233814			HIF-1-alpha transcription factor network;	
PKM seq: 3	-0.31797	0.776884	-0.26423	-0.66102	1.212328	0.912102	0.41315	1.25831	p12081	Pyruvate kin	2	0.416195			HIF-1-alpha transcription factor network;	
PKM seq: 2	0	4.671674	2.025925	0	2.67412	2.930724	1.836819	4.109883	p14342	Pyruvate kin	2	2.281143			HIF-1-alpha transcription factor network;	
PKM seq: 2	-0.55659	-0.12807	-0.44779	-1.31006	0.506301	0.761849	0.92487	1.174371	p15013	Pyruvate kin	2	0.115609			HIF-1-alpha transcription factor network;	
PKM seq: 2	-0.84291	-0.56552	-0.87226	-0.79449	1.20507	1.009034	0.388696	1.354629	p13136	Pyruvate kin	2	0.110281			HIF-1-alpha transcription factor network;	
PKM seq: 1	-0.84172	1.034216	-1.1475	-0.98506	1.847607	1.802277	1.810538	2.487215	p16345	Pyruvate kin	2	0.750948			HIF-1-alpha transcription factor network;	
PKM seq: 1	-0.21243	0.873686	-0.38489	-0.59401	1.331548	1.109049	1.384209	1.432376	p16346	Pyruvate kin	2	0.617443			HIF-1-alpha transcription factor network;	
PKM seq: 1	1.531912	2.633963	-0.51538	-0.82383	1.462018	1.534985	1.549382	2.170162	p13482	Pyruvate kin	2	1.192902			HIF-1-alpha transcription factor network;	
PKM seq: 1	-1.11091	-0.88446	-1.22433	-1.69295	1.255884	1.116757	-0.36365	1.497064	p15272	Pyruvate kin	2	-0.17582			HIF-1-alpha transcription factor network;	
PIWIL2 pMS	2.041903	1.378868	2.482144	-1.5269	0	2.526558	0	4.595928	p17358	Piwi-like pro	2	1.437312				
PITPNC1 se	-0.43189	-0.83398	-0.20198	-0.19626	5.370421	0	0	0	p06286	Cytoplasmic	2	0.46329				GO:0007165; P:signal transduction;
PICK1 seq:	-1.82704	-1.72766	-2.70143	-1.90599	2.364495	0.796137	0.927024	1.219703	p11756	PRKCA-bind	2	-0.35685				GO:0007205; P:protein kinase C-activating G-protein coupled receptor signaling pathway;
PHPT1 seq:	-0.56736	-0.41688	-0.48992	-0.81918	1.019359	0.817659	1.311636	0.95975	p08658	14 kDa pho	2	0.226884				GO:0050860; P:negative regulation of T cell receptor signaling pathway;
PHGDH sec	0.012544	0.179304	-0.63012	-1.52932	-0.18611	3.983622	3.083907	3.923581	p18560	D-3-phospl	2	1.104676				
PHGDH sec	-2.131	0.329649	-0.96389	-1.39679	3.527085	2.635855	-2.57307	2.885057	p08871	D-3-phospl	2	0.289111				
PHGDH sec	-0.22126	0.883977	-0.43532	-1.13755	1.993072	1.648596	-0.43266	1.805635	p16038	D-3-phospl	2	0.513061				
PHB seq: 5	-0.43594	-0.44469	-0.65936	-0.58993	2.451467	1.931595	0.934066	0.932941	p01562	Prohibitin	2	0.515019				GO:0060766; P:negative regulation of androgen receptor signaling pathway; GO:0007165; P:signal transduction;
PHACTR2 se	-0.79349	-1.91597	-0.77905	-0.69964	0.635945	1.533549	1.973999	1.979553	p16899	Phosphatas	2	0.241862				
PHACTR2 se	0.396975	-0.55622	-0.92968	-0.45418	1.682579	1.819279	1.404499	1.119712	p19646	Phosphatas	2	0.560371				
PGRMC1 se	-1.37262	-0.96195	-2.72644	-1.29616	4.66791	-2.22221	3.730439	4.306734	p18849	Membrane-	2	0.515713				
PGM3 seq:	-1.0459	0.005202	-1.56302	-1.1286	5.812585	0	0	6.49785	p20734	Phosphoac	2	1.072263				
PGK2 seq:	-0.5182	-0.4617	-0.65157	-1.08288	1.105507	1.029414	0.576277	0.677396	p11806	Phosphogly	2	0.08428				
PGK1 seq:	-0.93594	-0.88406	-1.46065	-2.41087	1.684244	1.202437	-0.01759	1.606649	p16242	Phosphogly	2	-0.15197			HIF-2-alpha transcription factor network; HIF-1-alpha transcription factor network;	
PGK1 seq:	-0.9114	-0.29203	-0.95389	-0.89865	1.679438	1.170893	0.480866	1.339349	p20222	Phosphogly	2	0.201822			HIF-2-alpha transcription factor network; HIF-1-alpha transcription factor network;	
PGK1 seq:	-0.79728	-0.19537	-1.22153	-0.6851	0.426712	1.69216	1.937224	0.828064	p09315	Phosphogly	2	0.248108			HIF-2-alpha transcription factor network; HIF-1-alpha transcription factor network;	
PGK1 seq:	-0.34743	-0.07075	-0.90153	-0.84617	0.270527	1.317531	1.469851	1.573334	p20270	Phosphogly	2	0.308169			HIF-2-alpha transcription factor network; HIF-1-alpha transcription factor network;	
PGK1 seq:	-0.77409	-0.97533	-1.18595	-1.42089	1.728977	0.633687	0.503155	1.498292	p17438	Phosphogly	2	0.000982			HIF-2-alpha transcription factor network; HIF-1-alpha transcription factor network;	
PGK1 seq:	-0.8257	-0.68576	-1.32241	-1.34513	1.043781	0.790469	0.401616	0.617094	p20818	Phosphogly	2	-0.16575			HIF-2-alpha transcription factor network; HIF-1-alpha transcription factor network;	
PGK1 seq:	-0.63667	-0.69715	-0.99972	-0.84674	1.496472	1.0178	0.499122	1.390899	p20960	Phosphogly	2	0.153002			HIF-2-alpha transcription factor network; HIF-1-alpha transcription factor network;	
PGK1 pM2	-0.37648	-0.4424	-0.25851	-1.04382	1.426594	1.519222	0.290624	1.447681	p01692	Phosphogly	2	0.320365			HIF-2-alpha transcription factor network; HIF-1-alpha transcription factor network;	
PGK1 pM2	-1.7912	-0.7966	-1.05395	-1.56074	2.682223	3.230167	0.789936	3.630023	p01203	Phosphogly	2	0.641232			HIF-2-alpha transcription factor network; HIF-1-alpha transcription factor network;	
PGD seq: 1	-0.88866	-0.6015	-0.79487	-1.22831	2.470236	2.040588	0.36767	1.844342	p15882	6-phospho	2	0.401187				
PGAM4 sec	-0.16827	0.996827	-0.52977	-1.76404	0.688387	1.1809	1.002045	1.303117	p03171	Probable pl	2	0.33865				
PGAM2 sec	-0.41257	0.560869	-0.95769	-0.94653	0.776117	1.513731	1.510959	1.617345	p20208	Phosphogly	2	0.457779				
PGAM2 pM	-0.46165	0.341208	-0.60028	-1.91537	0.591297	0.708076	0.346947	0.892444	p20095	Phosphogly	2	-0.01216				
PGAM1 sec	0.011903	0.29533	-1.27032	-1.09446	1.230092	0.778494	0.7381	0.25167	p00460	Phosphogly	2	0.117601				
PFN1 seq: 9	-0.32219	-0.15716	-0.50534	-0.74099	1.474921	0.916951	0.61342	1.342679	p03524	Profilin-1	2	0.327786				GO:0071363; P:cellular response to growth factor stimulus;
PFN1 seq: 3	-0.54881	-0.70531	-0.73174	-1.05484	0.552537	1.213384	0.898124	1.033981	p02835	Profilin-1	2	0.082166				GO:0071363; P:cellular response to growth factor stimulus;
PFN1 seq: 3	0.26358	0.532499	-0.98397	-1.50107	1.098471	1.889257	1.444626	2.576302	p02834	Profilin-1	2	0.664963				GO:0071363; P:cellular response to growth factor stimulus;
PFN1 seq: 1	-1.77355	-0.94807	-1.88462	-2.08461	2.420472	1.629315	1.054107	1.845023	p19002	Profilin-1	2	0.032258				GO:0071363; P:cellular response to growth factor stimulus;
PEX1 seq: 1	-0.77113	2.273018	2.152417	1.301922	0	0	0	7.778753	p03015	Peroxisome	2	1.591873			HIF-2-alpha transcription factor network;	
PELO seq: 2	-1.9338	0.501588	-0.73659	-1.43557	3.870907	0	0	4.354131	p11358	Protein pelo	2	0.577582				
PEBP1 seq:	-0.58983	0.813183	-1.40772	-3.28306	2.735808	2.521941	0.944056	2.680139	p10284	Phosphatid	2	0.551814			Aurora B signaling; ErbB1 downstream signaling;	
PEBP1 seq:	-0.33365	0.677474	-0.54362	-0.46619	1.353926	0.941987	0.61405	0.799136	p10285	Phosphatid	2	0.380388			Aurora B signaling; ErbB1 downstream signaling;	
PEBP1 seq:	-0.7355	0.330384	-0.76119	-0.79231	1.622081	1.050536	0.301444	0.547485	p10306	Phosphatid	2	0.195366			Aurora B signaling; ErbB1 downstream signaling;	
PDS5B seq:	0.299969	-0.11536	0.689597	-0.3405	0	0	0	5.474493	p17345	Sister chror	2	0.751026				
PDLIM5 sec	-0.82991	-0.35562	0.04002	-0.32523	2.555478	2.314168	2.494449	1.696226	p13423	PDZ and LIM	2	0.948698				
PDIA6 seq:	-1.17272	0.779043	-0.73845	-1.35414	3.110692	2.325294	1.044253	2.036377	p10524	Protein disu	2	0.753794				GO:0006987; P:activation of signaling protein activity involved in unfolded protein response;
PDIA6 seq:	0.050239	0.061321	-1.46615	-0.90363	2.910316	3.147222	2.513158	2.357965	p15291	Protein disu	2	1.083806				GO:0006987; P:activation of signaling protein activity involved in unfolded protein response;
PDIA6 seq:	-0.42	-0.32424	-0.87351	-1.02583	0.791296	1.800871	1.380913	2.106479	p09042	Protein disu	2	0.429498				GO:0006987; P:activation of signaling protein activity involved in unfolded protein response;
PDIA6 seq:	-0.40563	0.127411	-0.66096	-0.78094	1.379405	0.972009	1.273175	0.812158	p16149	Protein disu	2	0.339579				GO:0006987; P:activation of signaling protein activity involved in unfolded protein response;
PDIA4 seq:	-0.47374	0.786645	-0.63934	-1.24374	4.635964	3.870589	1.954943	4.178281	p08394	Protein disu	2	1.6337				
PDIA4 seq:	-0.00057	0.511758	-0.25119	-0.38099	1.068687	2.223517	1.764484	2.640785	p00365	Protein disu	2	0.94706				
PDIA4 seq:	-0.36537	0.834108	-0.7016	-1.18805	2.188373	2.265508	0.47936	2.576604	p16804	Protein disu	2	0.761116				
PDIA4 seq:	-0.52836	0.386736	-0.49483	-0.72476	1.864616	1.450141	0.878256	1.062857	p14446	Protein disu	2	0.486833				
PDIA4 seq:	-1.34595	0.29777	-3.02656	-2.49862	6.298105	0	0	0	p18691	Protein disu	2	-0.03441				
PDIA3 seq:	-0.71358	-0.88636	-1.12717	-0.90235	2.086935	0.920854	0.872207	0.750498	p00334	Protein disu	2	0.12513				GO:0007165; P:signal transduction;
PDIA3 seq:	0.380332	0.95249	0.375381	-0.21918	1.447121	2.012083	2.298184	1.397013	p01701	Protein disu	2	1.080428				GO:0007165; P:signal transduction;
PDIA3 seq:	-0.65177	0.200721	-0.70626	-0.97019	1.599453	1.113514	0.665285	1.569263	p17912	Protein disu	2	0.352502				GO:0007165; P:signal transduction;
PDIA3 seq:	-0.174	0.11167	-0.65511	-0.9994	1.66506	1.272999	0.608311	0.709627	p16105	Protein disu	2	0.317394				GO:0007165; P:signal transduction;
PDIA3 seq:	-0.68085	0.354216	-0.83717	-1.30161	0.703989	0.897892	0.699055	1.213828	p16593	Protein disu	2	0.131168				GO:0007165; P:signal transduction;
PDIA3 seq:	-0.30148	-0.40837	-0.91546	-2.01856	2.523033	2.143856	1.930625	2.980376	p16634	Protein disu	2	0.741753				GO:0007165; P:signal transduction;
PDIA3 seq:	-2.06335	-1.48288	-1.91859	-1.86803	1.866594	0.05481	0.17416	1.19953	p15236	Protein disu	2	-0.50472				GO:0007165; P:signal transduction;
PDIA3 seq:	0.03309	1.505079	1.052245	-1.35453	2.368184	2.560953	2.277027	2.671852	p01978	Protein disu	2	1.389237				GO:0007165; P:signal transduction;
PDIA3 seq:	-0.5539	0.983827	-3.38966	-2.44048	3.737273	1.462388	1.720921	1.998854	p08858	Protein disu	2	0.439904				GO:0007165; P:signal transduction;
PDIA3 seq:	-0.28688	0.68506	-0.98943	-0.28069	1.06831	0.627775	0.68756	1.013879	p18833	Protein disu	2	0.315698				GO:0007165; P:signal transduction;
PDHA2 pS2	0.890048	0.501081	0.454901	0.935683	2.441404	2.129595	1.889944	2.530334	p00327	Pyruvate de	2	1.471624	PDK1; PDHK4; PDHK3; PDHK2; PDHK1;			
PDCD6IP se	-1.76664	-1.15026	-1.39413	-2.70103	1.436772	2.095939	0.045804	1.841402	p16429	Programme	2	-0.19902				
PDCD6IP se	-0.34386	0.097599	-1.10613	-1.40851	1.338007	0.600554	0.808659	0.494191	p16767	Programme	2	0.060064				
PDCD5 seq	-1.43259	-0.18705	-0.84083	-0.73064	1.215728	1.895559	2.317619	2.519892	p09155	Programme	2	0.594712				
PDAP1 seq	-0.46871	1.215895	-2.19224	-3.26399	1.781699	1.142217	0.069552	0.430335	p00688	28 kDa heat	2	-0.16065				GO:0007165; P:signal transduction;
PCNA seq:	-1.53709	-2.19787	-2.13477	-1.5527	2.236375	0.868708	0.953271	1.054121	p00008	Proliferatin	2	-0.28874			BARD1 sign	GO:0048015; P:phosphatidylinositol-mediated signaling;
PCNA seq:	-0.9907	0.428264	-3.1825	-1.77535	2.63006	1.565184	0.707764	1.903631	p00251	Proliferatin	2	0.160795			BARD1 sign	GO:0048015; P:phosphatidylinositol-mediated signaling;
PCMT1 seq	-0.12772	0.457019	-0.6895	-1.05849	1.633653	1.09283	-0.04075	1.393245	p00945	Protein-L-is	2	0.332536				
PCMT1 seq	-0.93809	0.538508	-1.21183	-0.96582	2.26303	1.511186	1.205303	1.043181	p13351	Protein-L-is	2	0.430684				
PCLO seq: 3	-0.82919	2.180037	-1.48825	-4.29196	3.07356	2.389701	0.548119	1.856867	p01708	Protein picc	2	0.429862				GO:0019933; P:cAMP-mediated signaling;
PCBP1 seq:	-1.16136	0.398119	-0.61091	-1.26806	1.5287	1.270281	0.54046	1.245987	p14492	Poly(rC)-bir	2	0.242902				
PARK7 seq:	-0.52196	0.757073	-0.74175	-0.69164	0.937125	1.071411	0.83986	0.752124	p18080	Protein deg	2	0.300281			Alpha-synuclein signaling;	
PARK7 seq:	-2.0135	1.17503	-2.13937	-3.5437	3.694701	3.479434	0	3.94056	p16333	Protein deg	2	0.574145			Alpha-synuclein signaling;	
PARD3B se	-0.19217	-1.66224	0.260703	-0.28007	7.576787	0	4.427731	6.102118	p03693	Partitioning	2	2.029107				
PAK4 pS47	0.461268	-0.57295	0.155063	-0.86574	0	0	7.730515	9.250708	p04931	Serine/threo	2	2.019858	PAK4;	EGFRinh2 (I	FGF signalir	GO:0023014; P:signal transduction by phosphorylation; GO:0007165; P:signal transduction;
PADI2 seq:	0.192337	3.656079	0.01669	-1.02505	0.990671	2.108756	1.824317	2.062016	p16616	Protein-argi	2	1.228227				GO:0030520; P:intracellular estrogen receptor signaling pathway;
PACSIN3 pS	-0.0092	0.668439	-0.14882	0.034249	4.616979	0	0	5.575435	p15976	Protein kina	2	1.342135		AKTinh1 (Akt Inhibitor VIII);		
PABPN1 pS	-0.21471	-0.68594	0.096815	-0.62505	0.443036	2.115604	2.839204	2.399926	p08241	Polyadenyla	2	0.796111				
PABPC4 sec	-1.23008	1.67189	-1.18349	-7.16264	4.143519	2.925692	2.547291	3.318585	p17784	Polyadenyla	2	0.628846				
PABPC3 sec	-0.64584	-0.55734	-1.68863	-0.7787	1.428237	1.287044	0.516235	0.473902	p16123	Polyadenyla	2	0.004363				
PABPC1L sc	-1.42235	-1.20481	-1.32917	-1.19544	2.260933	1.308678	1.11731	2.724174	p05620	Polyadenyla	2	0.282416				
PABPC1 sec	-0.68921	0.723448	-0.47924	-1.31106	1.580216	1.30935	-0.20443	1.323671	p17783	Polyadenyla	2	0.281594				
PA2G4 seq	-1.21001	-2.32238	-2.62782	-1.99948	5.293263	4.131635	3.663216	4.036119	p20231	Proliferatio	2	1.120567			Coregulation of Androgen receptor activity;	
P4HB seq: 8	-0.37045	-0.37489	-0.48053	-0.62805	1.14445	0.812714	0.828817	1.076764	p01706	Protein disu	2	0.251102				
P4HB seq: 5	-0.41368	-0.58138	-0.83469	-0.70507	1.873813	1.194311	0.666904	1.431361	p20322	Protein disu	2	0.328947				
P4HB seq: 4	-0.94419	0.563937	-0.83779	-0.90778	0.880143	0.108901	0.392364	1.148403	p01962	Protein disu	2	0.050499				
P4HB seq: 4	-1.90048	-1.08201	-1.61225	-2.06424	1.441636	1.899295	-0.4465	1.43224	p16782	Protein disu	2	-0.29154				
P4HB seq: 4	-1.65676	-0.62609	-1.71061	-1.08671	2.054309	3.157718	2.395394	3.501513	p08404	Protein disu	2	0.753595				

GOT1 seq: (	0.754468	-1.14508	-0.18323	-6.12593	0.336386	0.39281	0.431207	0.674441	p14495	Aspartate ar	2	-0.60812		
GOLGB1 se	-1.1652	-0.58201	-1.30903	-2.20471	2.490458	2.855383	1.94022	1.77293	p08817	Golgin subf	2	0.474757	AKTinh1 (Akt Inhibitor VIII);	
GOLGB1 se	0.604571	0.187201	-0.90418	-0.35361	6.968732	5.523047	0	0	p05322	Golgin subf	2	1.50322		
GNL1 seq: 4	2.158838	1.808248	1.49886	0.94909	3.204036	3.012689	2.999399	2.413946	p07484	Guanine nu	2	2.255638		GO:0007165; P:signal transduction;
GNB2L1 se	-1.19377	1.752289	-1.15825	-3.24125	1.93264	1.403099	0.622395	1.804086	p10328	Guanine nu	2	0.240154	Hypoxic an	GO:0051898; P:negative regulation of protein kinase B signaling; GO:0030178; P:negative [illegible]
GNB2L1 se	-0.5746	-0.20718	-0.80878	-0.59772	1.418137	0.821376	0.13117	1.237953	p14125	Guanine nu	2	0.177544	Hypoxic an	GO:0051898; P:negative regulation of protein kinase B signaling; GO:0030178; P:negative [illegible]
GNB2L1 se	-0.63927	0.973543	-1.33042	-1.2244	1.53568	1.101287	0.411164	0.390619	p13512	Guanine nu	2	0.152276	Hypoxic an	GO:0051898; P:negative regulation of protein kinase B signaling; GO:0030178; P:negative [illegible]
GNB2 seq: (	-0.80529	0.042151	-0.96369	-1.16067	1.935168	1.559199	0.52542	1.487553	p11251	Guanine nu	2	0.327481		GO:0007186; P:G-protein coupled receptor signaling pathway;
GMPS seq:	-1.93187	1.073961	-1.33399	-3.52265	2.096433	1.866632	0.9741	1.790467	p17486	GMP synth	2	0.126636		
GLUD1 seq	-3.44323	1.41716	-2.46465	0.817244	3.598677	3.676259	0	2.318821	p00202	Glutamate (	2	0.740035		
GLOD4 seq	-0.1083	0.83539	-1.20095	-3.21839	3.314684	2.686775	2.113122	3.265106	p00711	Glyoxalase (	2	0.960929		
GLO1 seq: 5	-1.85267	-1.92492	-3.78926	-1.95684	5.768772	0	0	6.265474	p01230	Lactoylgluta	2	0.31382		
GLO1 seq: 4	-0.70851	-0.49854	-0.74269	-1.15467	2.304651	1.261446	1.207982	1.968304	p05304	Lactoylgluta	2	0.454746		
GIT2 pS614	-0.05064	0.061315	-0.24704	-0.14693	1.396251	0.869384	1.238374	1.988947	p07955	ARF GTPase	2	0.638707	Integrin-link	GO:0008277; P:regulation of G-protein coupled receptor protein signaling pathway;
GIT1 seq: 5	-0.35015	0.206947	-0.45894	-0.76299	0	0	0	7.269327	p10964	ARF GTPase	2	0.738024	Regulation (	GO:0008277; P:regulation of G-protein coupled receptor protein signaling pathway;
GIGYF2 seq	-0.0433	-0.24796	-1.90458	-1.74258	1.027997	4.007372	3.922499	1.6338	p08028	PERQ aminc	2	0.831655		GO:0048009; P:insulin-like growth factor receptor signaling pathway;
GDI2 seq: 7	-0.95675	0.834474	-0.81134	-1.13537	1.098852	0	0	1.905107	p18069	Rab GDP di	2	0.116871		GO:0051056; P:regulation of small GTPase mediated signal transduction; GO:0007264; P: [illegible]
GDI2 seq: 4	-0.77578	1.131087	-0.95791	-1.50383	0.575092	1.767193	0.991149	1.758635	p09857	Rab GDP di	2	0.373205		GO:0051056; P:regulation of small GTPase mediated signal transduction; GO:0007264; P: [illegible]
GDI2 seq: 3	-0.6099	0.47453	-0.55408	-0.92994	1.21953	1.32442	0.721177	1.142458	p18604	Rab GDP di	2	0.348524		GO:0051056; P:regulation of small GTPase mediated signal transduction; GO:0007264; P: [illegible]
GDI2 seq: 2	-0.37975	0.654105	-0.77169	-0.97021	1.854178	0.629404	-0.19643	1.430818	p10293	Rab GDP di	2	0.281302		GO:0051056; P:regulation of small GTPase mediated signal transduction; GO:0007264; P: [illegible]
GDI2 seq: 1	0.510747	1.17713	0.162976	0.152292	1.964818	1.469938	0.442346	1.658234	p02871	Rab GDP di	2	0.94231		GO:0051056; P:regulation of small GTPase mediated signal transduction; GO:0007264; P: [illegible]
GCSAM seq	-1.85405	-1.84905	-2.27071	-3.83308	1.950488	2.342996	0	3.462146	p14035	Germinal ce	2	-0.25641		
GBA3 seq: 1	-0.5746	-0.20718	-0.80878	-0.59772	1.430402	0.821376	0.138555	1.247422	p14192	Cytosolic b	2	0.181184		
GAPVD1 se	0	0	0	0	2.708289	2.192069	1.788481	2.229281	p11009	GTPase-acti	2	1.114765		GO:0051056; P:regulation of small GTPase mediated signal transduction; GO:0007165; P: [illegible]
GAPDH seq	-0.3137	0.575989	-0.55133	-0.55619	1.131192	1.250381	1.490956	1.805931	p10406	Glyceraldeh	2	0.604154	Validated targets of C-MYC transcriptional activation;	

[illegible]

ABI1 seq: 3	0.261879	1.674017	1.445025	0.78947	4.399985	3.306478	5.025424	4.609862	p14702	Abl interact	2	2.689018			Alpha4 beta	GO:0007169; P:transmembrane receptor protein tyrosine kinase signaling pathway; GO:0038096; P:Fc-gamma receptor signaling pa [illegible]
ABCB6 seq:	-1.13266	-1.35638	-2.06718	-1.34134	1.919238	0.766172	1.195773	1.052671	p20438	ATP-binding	2	-0.12046				
ABCA2 seq:	-0.49636	1.509205	-1.43411	-1.92852	3.434492	3.36402	1.833381	3.792626	p02904	ATP-binding	2	1.259341				
ABCA13 sec	-1.14824	0.512887	-2.01053	-4.15519	1.178167	1.117871	-0.14861	1.18562	p10569	ATP-binding	2	-0.4335				
ABCA13 sec	-0.89869	-0.42485	-0.82333	-1.11731	0.998191	0.789371	0.249673	0.968858	p00911	ATP-binding	2	-0.03226				
AARS seq: 7	-1.78585	0.190603	-2.4646	-2.52295	8.020607	0	0	7.177622	p13564	Alanine--tRI	2	1.076929				
AARS seq: 1	-0.20666	-0.24864	-0.75964	-1.29405	0	6.551077	5.290354	0	p08480	Alanine--tRI	2	1.166555				
ZYX pS267	4.821022	9.025586	5.532637	6.089509	-0.87549	-0.20279	-0.34858	-0.6851	p15482	Zyxin	1	2.919598			E-cadherin s	GO:0007267; P:cell-cell signaling; GO:0007165; P:signal transduction;
ZFP36L2 se	10.88143	8.144679	10.47878	10.8211	0.444876	0.533516	-0.28312	0.547115	p07145	Zinc finger p	1	5.196048				
ZC3HAV1 s	0	0	8.589593	9.909181	0.685688	0.846799	0.794645	0.340061	p06126	Zinc finger C	1	2.645746		Up with IGF - down with		GO:0043123; P:positive regulation of I-kappaB kinase/NF-kappaB signaling;
YARS seq: 4	8.780221	8.742319	10.04963	8.037018	-4.21198	-4.1273	-0.74445	-2.75258	p15043	Tyrosine--tR	1	2.97161				GO:0007165; P:signal transduction;
WASF1 seq	12.47369	11.132	11.14348	10.61066	0	0	0	0	p01118	Wiskott-Ald	1	5.669978			RAC1 signal	GO:0016601; P:Rac protein signal transduction;
USP36 pS8	5.91459	5.510586	7.713898	6.466225	-0.07813	0.214973	-0.37232	-0.25139	p08133	Ubiquitin ca	1	3.139803				
UGGT1 seq	9.795645	0	9.354253	7.187691	0	0	0	0	p15362	UDP-glucos	1	3.292199				
U2SURP pS	7.647999	8.602599	7.242042	6.475081	-0.11152	0.204082	-0.18714	-0.32531	p03927	U2 snRNP-a	1	3.693478				
TTLL12 seq	10.93461	11.29391	10.2507	10.55707	0	0	0	0	p03809	Tubulin--tyr	1	5.379536		AKTinh1 (Akt Inhibitor VIII);		
TRIM47 pS	7.956985	11.42613	7.453566	8.860905	2.091791	2.34982	2.376308	2.350339	p07386	Tripartite m	1	5.60823				
TPR seq: 18	10.11203	7.534297	10.39574	9.501665	0.107967	-0.03626	0.180448	0.102895	p02230	Nucleoprot	1	4.737349				GO:0019221; P:cytokine-mediated signaling pathway;
TPR pS215	10.21455	10.1893	10.45418	8.486556	-0.15406	0.112131	0.371275	0.090559	p02967	Nucleoprot	1	4.970563		Up with IGF - down with		GO:0019221; P:cytokine-mediated signaling pathway;
TPM3 seq:	8.062345	7.10771	6.360309	5.700319	-0.55725	-1.5134	0.263467	0.879929	p19019	Tropomyos	1	3.287928				
TPD52L2 p	7.66484	5.385909	6.777768	6.179188	-0.29054	-1.11657	-0.87233	-10.9227	p03194	Tumor prot	1	1.600699				
TP53BP2 p	6.862612	9.668869	9.317133	8.765552	-0.1829	0.437682	1.387318	1.194096	p00896	Apoptosis-s	1	4.681295			Direct p53 (	GO:0009967; P:positive regulation of signal transduction; GO:0007165; P:signal transduction;
TP53BP1 se	9.084187	5.984637	6.117644	7.526306	-9.64494	0.105722	-2.71088	-0.69842	p09591	Tumor supp	1	1.970532			ATM pathway;	
TP53BP1 p	10.40789	9.742721	10.34916	10.39191	0	6.743255	8.182782	0	p10350	Tumor supp	1	6.977214			ATM pathway;	
TNRC6B sec	8.432915	10.26875	7.416976	7.548885	-1.05633	0.060834	1.17832	-1.46551	p15860	Trinucleotic	1	4.048104				GO:0007219; P:Notch signaling pathway; GO:0048015; P:phosphatidylinositol-mediated signaling; GO:0008543; P:fibroblast growt [illegible]
TJP2 pS413	7.868769	6.953628	8.641733	8.395199	-0.94531	0.463773	-0.01126	0.771303	p05826	Tight juncti	1	4.01723			Validated ta	GO:0035329; P:hippo signaling;
TJP2 pS115	8.337751	9.120836	7.066311	7.250687	-1.25846	-1.31157	-0.7327	0.230671	p15131	Tight juncti	1	3.587941		PKCinh2 (B	Validated ta	GO:0035329; P:hippo signaling;
TBC1D4 sec	9.3054	9.942092	9.513075	9.697821	2.828473	2.600303	-5.36237	-5.32152	p02089	TBC1 doma	1	4.150409			Insulin-mediated glucose transport; Class I PI3K signaling events mediated by Akt;	
TAB3 pM32	7.885152	9.605659	10.52019	10.39468	0.22746	-1.87358	-0.67002	-9.41592	p07983	TGF-beta-ac	1	3.334203				GO:0070423; P:nucleotide-binding oligomerization domain containing signaling pathway; GO:0035872; P:nucleotide-binding doma [illegible]
SVIL pS221	9.582563	9.033701	8.479373	7.271443	-1.50115	-0.82833	-0.92951	-0.79112	p08855	Supervillin	1	3.789621			Coregulation of Androgen receptor activity;	
STK4 pS320	7.061244	7.357808	8.03682	7.880844	0	7.477539	6.561613	0	p17004	Serine/threo	1	5.546984				GO:0035329; P:hippo signaling; GO:0023014; P:signal transduction by phosphorylation; GO:0090090; P:negative regulation of can [illegible]
STAU2 pS4	7.880518	10.38628	5.463563	8.01083	-0.60339	0.022612	0.015962	-0.43097	p18486	Double-stra	1	3.843176				
STAM seq:	9.121515	9.636676	8.010979	10.06557	-0.33581	-0.84746	0.359716	-0.27022	p08000	Signal trans	1	4.467622			IL2-mediate	GO:0042059; P:negative regulation of epidermal growth factor receptor signaling pathway; GO:0009967; P:positive regulation of sig [illegible]
SRRM2 pS2	0	0	11.16856	10.55273	0.222296	0.383018	2.978629	0.113062	p03981	Serine/argin	1	3.177288				
SPAG9 seq:	9.451315	7.559841	7.973664	8.31096	0	0	0	0	p06234	C-Jun-amin	1	4.161972		AKTinh1 (Al	Arf6 trafficking events;	
SPAG1 pS3	6.933229	5.839638	7.717582	8.620521	0.440977	0.12392	0.314067	0.317157	p09830	Sperm-asso	1	3.788386				
SP1 seq: 20	0	9.67694	7.561781	6.155837	0	0	0	0	p15038	Transcriptio	1	2.92432			p38 signalin	GO:0040037; P:negative regulation of fibroblast growth factor receptor signaling pathway; GO:0045747; P:positive regulation of No [illegible]
SON seq: 1	11.48667	9.805954	7.313831	6.311899	0	5.341822	0	7.48754	p18754	Protein SON	1	5.968464				
SNX3 seq: 1	7.27592	7.737538	8.097517	9.482973	2.708532	0.421502	3.608052	4.141702	p11545	Sorting nexi	1	5.434217				
SNX3 seq: 1	7.199852	7.737538	8.120733	9.36307	2.790274	0.27874	3.616925	4.140835	p11546	Sorting nexi	1	5.405996				
SNX1 pS32	9.589822	9.890747	10.26571	10.51023	1.266806	2.14072	1.894379	1.373687	p11098	Sorting nexi	1	5.866512			PAR1-mediated thrombin signaling events; Posttranslational regulation of adherens junction stability and dissassembly;	
SNX1 pS32	10.29562	10.55172	10.86132	11.3112	1.133367	1.954663	1.71239	1.53488	p11097	Sorting nexi	1	6.169395			PAR1-mediated thrombin signaling events; Posttranslational regulation of adherens junction stability and dissassembly;	
SNRPGP15	0	11.39177	7.824837	6.788993	2.356962	1.925398	0.251475	3.057707	p14559	Putative sm	1	4.199642				
SMARCC1 s	10.30328	11.54659	0	8.547776	2.406437	2.52488	1.882253	3.284671	p20209	SWI/SNF co	1	5.061986			Regulation (	GO:0008286; P:insulin receptor signaling pathway;
SLIT1 seq: 3	7.131963	9.350055	4.913622	7.173592	-1.31229	2.832294	-5.26701	3.219005	p11961	Slit homolo	1	3.505154			Posttranslational regulation of adherens junction stability and dissassembly;	
SLBP seq: 1	7.810471	0	6.795866	9.166193	0	0	0	10.06149	p01797	Histone RN	1	4.229253				
SHARPIN se	0	9.030441	11.07893	9.874999	-1.24174	-0.47467	-0.62569	-0.26175	p04767	Sharpin	1	3.422566				GO:0043123; P:positive regulation of I-kappaB kinase/NF-kappaB signaling;
SHARPIN p	9.808609	11.03275	9.858965	8.710567	0.14239	0.1745	0.187527	0.38909	p04630	Sharpin	1	5.038049				GO:0043123; P:positive regulation of I-kappaB kinase/NF-kappaB signaling;
SH3KBP1 s	6.992753	9.146638	9.210732	7.312224	0.047154	-0.34671	-0.12776	-0.08248	p17035	SH3 domain	1	4.019069			Internalizati	GO:0042059; P:negative regulation of epidermal growth factor receptor signaling pathway; GO:0007173; P:epidermal growth factor [illegible]
SEPT9 seq:	5.62958	6.433253	6.109117	5.744665	0.039392	-0.09729	0.443682	0.738971	p09678	Septin-9	1	3.130171				
SCRIB pS15	6.06691	9.943367	7.27978	6.704025	0	0	0	0	p12281	Protein scri	1	3.74926				
SCEL seq: 8	6.483569	8.016164	5.214238	7.378899	-0.41931	-0.44123	-0.63224	-0.76886	p00075	Sciellin	1	3.103902				
SAMD4B se	0	7.975367	8.679908	8.938001	0.758643	0.515036	0.723123	0.783348	p14895	Protein Sma	1	3.546678				
RRP36 pS7	9.21945	10.50641	8.660777	8.796014	-0.40324	-0.6141	-0.36169	-0.43417	p08263	Ribosomal I	1	4.421181				
RRM2 seq:	9.936914	12.57622	11.15911	9.632215	-1.20303	-0.85241	-1.05117	-0.67696	p01005	Ribonucleo	1	4.940111	CDK2; CDK	Akt-CAMK2	E2F transcription factor network;	
RPRD2 pS3	8.625096	8.541417	7.989347	6.488857	0	0	0	0	p18118	Regulation (	1	3.95559				
RPLP2 seq:	8.561945	10.45388	8.995084	9.191876	0.380289	-0.37233	-0.00505	0.659196	p12166	60S acidic r	1	4.733112				
RPL32 pM9	8.760397	7.329381	8.788098	7.795566	-7.02788	2.670143	1.634562	2.572582	p17496	60S ribosor	1	4.065357				
RPL27A sec	9.098824	10.72208	8.726224	0	0	0	0	0	p09213	60S ribosor	1	3.568391				
RNF19A sec	8.80445	9.363785	7.995865	9.077626	0.514752	-0.85773	-0.61779	-1.24297	p16505	E3 ubiquitin	1	4.129747				
RNF113A p	8.663399	8.765169	8.978812	9.04976	-0.18349	-0.10586	-0.04283	-0.17001	p20967	RING finger	1	4.369368				
RICTOR seq	8.78465	6.72427	8.646983	7.084434	0	0	0	0	p04200	Rapamycin-	1	3.905042			Class I PI3K	GO:0031929; P:TOR signaling; GO:0032008; P:positive regulation of TOR signaling; GO:0051897; P:positive regulation of protein ki [illegible]
RFC2 seq: 9	7.209474	7.500415	6.415534	6.747686	-0.01922	-0.72303	-1.241	-6.88374	p20236	Replication	1	2.375765			ATR signaling pathway; Fanconi anemia pathway;	
RBM27 seq	9.651087	10.03733	11.86269	12.68929	-0.95971	0.512592	-0.61698	0.254859	p08633	RNA-bindin	1	5.428896				
RASAL2 pS	0	8.858336	8.183694	7.846247	-0.27702	0.418403	-0.08209	-0.00981	p07997	Ras GTPase	1	3.117221				GO:0046580; P:negative regulation of Ras protein signal transduction; GO:0007165; P:signal transduction;
RANBP2 pS	11.87286	9.792576	11.29244	11.10068	0	0	0	0	p12643	E3 SUMO-p	1	5.50732			Sumoylation	GO:0006469; P:negative regulation of protein kinase activity; GO:0019221; P:cytokine-mediated signaling pathway;
RAD9A seq	9.408543	8.61931	6.795275	6.645113	-2.18747	-1.15552	-5.67625	-5.6354	p18259	Cell cycle ch	1	2.101701	CDK1; ATM	Up with IGF	ATM pathway; ATR signaling pathway; Fanconi anemia pathway; Regulation of Telomerase;	
RABGAP1L	10.53208	7.757309	10.64174	10.22181	-7.81238	-7.75803	-7.26597	-0.42085	p17180	Rab GTPase	1	1.986963				
PXN pS126	5.719791	8.443831	7.261085	7.52728	0.524559	0.36849	0.445258	0.560123	p06354	Paxillin	1	3.856302	ERK2; ERK1;		a4b7 Integr	GO:0007229; P:integrin-mediated signaling pathway; GO:0000187; P:activation of MAPK activity; GO:0007173; P:epidermal growth [illegible]
PTPN23 sec	13.299	14.14043	12.87967	13.34603	0.28278	0.459421	0.736979	0.739843	p10776	Tyrosine-pr	1	6.985518				
PTPN11 sec	5.716064	7.343453	6.693533	7.85011	0.153444	0.395482	0.232117	0.936005	p00537	Tyrosine-pr	1	3.665026	ALK;		IL5-mediate	GO:0060334; P:regulation of interferon-gamma-mediated signaling pathway; GO:0009755; P:hormone-mediated signaling pathway; [illegible]
PTGFRN pS	7.879237	8.418117	7.314081	8.834175	0	0	0	0	p07166	Prostagland	1	4.055701		mTORinh2 (Torin-1); EGFRinh1 (PD-168393); AKTinh1 (Akt Inhibitor VIII);		
PSMD2 seq	9.148109	8.915565	5.338457	5.517469	0	0	4.22598	0	p09691	26S proteas	1	4.143198				
PSAP seq: 4	7.51774	9.298701	7.402842	7.087274	1.489073	1.169028	-0.03938	0.930702	p18828	Prosaposin	1	4.356998				GO:0043408; P:regulation of MAPK cascade;
PRPF4B pS	8.564589	9.337527	9.195541	6.406036	0	0	0	0	p17270	Serine/threo	1	4.187962				
PPP1R18 p	8.447429	10.53619	9.19855	8.218426	0.437277	0.569129	0.440514	0.321648	p10709	Phostensin	1	4.771146				
PPP1R16A	10.5604	10.45502	9.32634	9.977264	0	0	0	0	p06436	Protein pho	1	5.039878				
PPL seq: 18	7.147791	8.593179	7.215939	5.333866	0	7.229979	6.188417	7.446456	p17271	Periplakin	1	6.144453				
POLA2 pS1	0	8.190242	6.814697	7.50743	-0.41936	0.006379	-0.23169	-0.11194	p04592	DNA polym	1	2.71947				
PNN seq: 3	6.716589	6.236217	6.078361	5.216755	-0.26069	0.215477	0.4901	-0.14925	p17167	Pinin	1	3.067945		AKTinh1 (Akt Inhibitor VIII);		
PLS3 seq: 2	7.647035	9.973531	8.155561	7.996796	0.387084	0.241469	0.284484	0.49523	p13906	Plastin-3	1	4.397649				
PLIN3 pS13	0	9.415867	8.387076	8.333111	0.68782	-0.00946	0.511391	0.596511	p17374	Perilipin-3	1	3.490289				
PLEKHA5 p	7.669099	0	10.19132	8.527272	0.375222	0.106359	0.202321	0.356071	p03309	Pleckstrin h	1	3.428458				
PLEC seq: 2	11.13564	9.951898	6.470761	5.159637	-9.17458	-0.02144	-1.8725	-0.08439	p06040	Plectin	1	2.695626				
PITHD1 seq	9.533865	9.170121	8.469044	0	-0.96057	-0.46359	0.353447	-1.02197	p09538	PITH domai	1	3.135043				
PHLDB1 se	7.642801	7.732827	8.834434	6.892615	-0.3308	-2.73662	0.042686	-0.05524	p15289	Pleckstrin h	1	3.502837				
PHLDB1 se	0	6.546509	7.236593	7.897307	-1.32969	-0.39054	-0.34439	-1.00042	p15694	Pleckstrin h	1	2.32692				
PFN1 seq: 9	8.95727	9.401207	9.173336	9.571682	-0.12179	0.077425	-0.0546	0.072863	p03523	Profilin-1	1	4.634673				GO:0071363; P:cellular response to growth factor stimulus;
PDIA3 seq:	12.21079	13.6665	11.10423	0	-9.32554	-1.3662	-3.00464	-2.08	p16670	Protein disu	1	2.650642				GO:0007165; P:signal transduction;
PCM1 pS93	9.446822	11.56233	9.185222	9.5923	0.494152	0.707961	0.430302	0.349311	p00212	Pericentriol	1	5.22105				
PALLD pS8	11.07356	13.0946	10.47048	11.307	0.434585	0.186333	-0.18523	0.122375	p14475	Palladin	1	5.812963				
PAK2 seq: 5	6.76015	8.988415	6.006136	5.12116	2.681563	2.895893	0.430657	3.5592	p14138	Serine/threo	1	4.555397			C-MYC path	GO:0023014; P:signal transduction by phosphorylation; GO:0006469; P:negative regulation of protein kinase activity; GO:0050852 [illegible]
PAIP2 seq:	5.837589	8.485996	6.116528	6.158575	1.073114	0.830858	-6.64397	1.627142	p17776	Polyadenyla	1	2.935729				
PA2G4 seq	11.74404	10.76689	10.06108	10.65617	-0.10821	0.027498	0.391909	-0.08634	p03098	Proliferation	1	5.431629			Coregulation of Androgen receptor activity;	
NQO1 seq:	8.948441	10.41469	8.174762	6.929081	10.21841	9.785203	7.898964	0	p17744	NAD(P)H de	1	7.796194			Validated transcriptional targets of TAp63 isoforms;	
NPM1 pS70	11.87786	12.94828	9.024488	0	-11.0708	-3.7108	-10.5244	-10.4835	p01906	Nucleophos	1	-0.24236	CDK2; CDK	Up with IGF	BARD1 sign	GO:0007165; P:signal transduction;
NFIX seq: 2	9.811193	8.188939	6.028634	7.245163	0.411534	1.280552	-0.17587	0.342572	p05537	Nuclear fact	1	4.141589				
NDRG3 seq	7.932845	8.062208	7.784409	7.687681	-5.17222	0.663325	3.085414	1.686442	p03222	Protein NDI	1	3.966263				
NCAPH seq	6.869961	9.198518	7.154722	6.227762	0.904739	1.383001	1.806901	1.671132	p15928	Condensin	1	4.402092			IL2 signaling	GO:0045737; P:positive regulation of cyclin-dependent protein kinase activity;
NCAPG pS9	8.897446	8.26309	9.240622	0	0.26808	0.060168	0.355671	0.337213	p07537	Condensin	1	3.427786			IL2 signaling	GO:0045737; P:positive regulation of cyclin-dependent protein kinase activity; GO:0007165; P:signal transduction;
NAP1L4 sec	10.81675	10.04627	0	8.669025	-0.1002	-0.08185	0.088146	0.083918	p17797	Nucleosom	1	3.690258		AKTinh1 (Akt Inhibitor VIII);		
NAA10 seq	0	8.762935	8.534707	10.37583	0.517463	0.621897	0.216526	0.587022	p15788	N-alpha-ace	1	3.702047			Hypoxic and oxygen homeostasis regulation of HIF-1-alpha;	
MYH9 seq:	9.646561	11.26351	0	9.129375	-0.10061	-0.08216	-0.17825	-0.1532	p04908	Myosin-9	1	3.690654				GO:0038032; P:termination of G-protein coupled receptor signaling pathway; GO:0007229; P:integrin-mediated signaling pathway; [illegible]
MSL1 seq:	9.87775	10.24746	8.398532	8.621095	-8.11014	2.853122	-7.56374	-7.52288	p17299	Male-specif	1	2.10015				
MPHOSPH8	8.48895	9.439646	9.340379	8.740662	-0.53211	-1.99652	1.037805	-7.82405	p15616	M-phase ph	1	3.336845				
MLLT6 seq:	11.37671	12.32786	11.52378	11.31127	-1.81225	-0.6216	-9.3565	0.864569	p11938	Protein AF-	1	4.451731				
MKL2 seq:	11.56912	13.57067	9.949072	11.35048	0.065804	0.273162	0.32495	0.105281	p17277	MKL/myoca	1	5.901068				
MINK1 pS6	0	0	9.535992	9.591983	-0.45789	-0.70389	-0.04286	-0.33226	p04185	Misshapen-	1	2.198883				GO:0035556; P:intracellular signal transduction;
MEAF6 seq	10.68532	10.28781	0	8.476931	0	0	7.403714	0	p17309	Chromatin	1	4.606722				
MDH2 seq:	7.920714	9.737681	7.156947	8.831351	0	0	0	0	p16397	Malate deh	1	4.205837				
MDC1 seq:	9.365485	0	7.591056	10.27107	-0.26937	-0.15727	-0.23389	-0.28023	p15412	Mediator of	1	3.285858			ATM pathway;	
MCL1 seq:	7.548807	11.13508	6.649202	6.945054	1.787531	0.61768	2.464225	2.662503	p06964	Induced my	1	4.97626			IL6-mediated signaling events; HIF-1-alpha transcription factor network; E2F transcription factor network; Direct p53 effectors;	
MARCKSL1	9.851797	7.2921	7.478316	7.580381	7.700214	8.886434	8.832787	8.697984	p17752	MARCKS-re	1	8.290002			Signaling events mediated by the Hedgehog family; Reelin signaling pathway; Lissencephaly gene (LIS1) in neuronal migration and development;	
MAPK3 pT2	10.23657	12.30449	10.16271	10.19354	0.683382	0.511971	0.698623	0.44343	p14524	Mitogen-ac	1	5.654339	Ret; MEK2;	Up with IGF	S1P4 pathw	GO:0070498; P:interleukin-1-mediated signaling pathway; GO:0060397; P:JAK-STAT cascade involved in growth hormone signaling p [illegible]
MAP7 seq:	6.651229	0	8.808079	9.496381	0	0	0	0	p05347	Ensconsin	1	3.119461				
MAP4K4 se	6.116864	0	6.612804	6.232027	0.296139	0.034417	0.37386	0.52496	p18231	Mitogen-ac	1	2.523884			EPHB forwa	GO:0023014; P:signal transduction by phosphorylation; GO:0035556; P:intracellular signal transduction;
MAP1B seq	6.196365	4.858434	4.914019	6.181796	-0.03542	-0.26271	-0.13718	-0.47866	p02044	Microtubul	1	2.65458			Reelin signaling pathway; Lissencephaly gene (LIS1) in neuronal migration and development; Netrin-mediated signaling events;	
MAP1B seq	7.958836	8.660774	7.196744	8.098845	-0.88511	-0.38511	-0.25776	-0.02187	p01161	Microtubul	1	3.795669			Reelin signaling pathway; Lissencephaly gene (LIS1) in neuronal migration and development; Netrin-mediated signaling events;	
MAFK pS25	0	8.565052	8.694163	7.754275	0.052098	0.268606	0.064788	0.710963	p17968	Transcriptio	1	3.263743				
LMO7 seq:	5.176016	5.039281	6.519961	6.258449	0.459544	-0.07544	0.135904	0.036692	p10484	LIM domain	1	2.9438				
LMO7 seq:	8.3672	0	6.48343	5.330257	-0.45332	-0.81555	-0.04036	0.010642	p08108	LIM domain	1	2.360288				
LMO7 seq:	10.16248	8.791697	8.322329	6.3673	0.283438	0.178312	0.38076	0.748706	p06719	LIM domain	1	4.404378				
LMAN1 seq	12.34191	13.09168	11.76184	11.40879	-10.5073	-10.453	-9.96092	-9.92007	p09320	Protein ERG	1	0.970366			Signaling events mediated by TCPTP;	
LASP1 seq:	10.23733	11.84572	10.72817	0	0	0	0	0	p19142	LIM and SH	1	4.101401				GO:0009967; P:positive regulation of signal transduction;
LAD1 pS17	9.624929	10.6341	9.068778	9.198029	-0.15378	0.028949	0.023156	0.003157	p15005	Ladinin-1	1	4.803416				
KRT18 seq:	10.93443	12.06811	8.01172	0	0	0	0	0	p17832	Keratin, typ	1	3.876782			Caspase cas	GO:0033209; P:tumor necrosis factor-mediated signaling pathway;
KMT2D seq	9.980844	11.00181	0	9.111921	0	0	0	0	p13305	Histone-lys	1	3.761822				
KLHDC8A s	7.244797	0	7.069467	5.503291	-0.31584	-0.15498	-0.49032	-0.64529	p12211	Kelch doma	1	2.276389				
KLF16 seq:	8.93173	10.14256	5.395936	9.210276	-0.2524	0.244779	0.224975	0.135637	p03442	Krueppel-lik	1	4.254187				
KIF15 pS11	7.582687	0	7.110302	7.903547	0.538328	0.46893	0.769262	0.356588	p08395	Kinesin-like	1	3.091206				
KHSRP seq:	8.964	9.044343	7.851753	6.36363	8.567385	9.180368	7.139004	9.699536	p14269	Far upstrea	1	8.351252				
KDM5A pS	10.61981	9.747598	7.921424	8.072118	0.355466	-0.79244	0.27753	0.647115	p12561	Lysine-spec	1	4.606078				
KDM2A sec	7.627346	7.56241	8.883127	7.240468	1.867057	3.298019	4.904824	3.806931	p03817	Lysine-spec	1	5.648773				
IWS1 seq: 4	7.374343	6.237233	8.46408	8.64089	0.997119	0.855525	1.130347	1.359503	p02727	Protein IWS	1	4.38238				
IRS2 seq: 7	10.30453	11.41095	10.34474	9.828752	10.24688	9.886796	10.19244	10.53911	p11331	Insulin rece	1	10.34427			IGF1 pathw	GO:0060397; P:JAK-STAT cascade involved in growth hormone signaling pathway; GO:0048015; P:phosphatidylinositol-mediated signaling; GO:0008286; P:insulin receptor signaling pathway; GO:0008543; P:fibroblast growth factor receptor signaling pathway; GO:0007173; P:epidermal growth fact [illegible]
IRS2 seq: 7	10.53749	11.73184	10.48771	10.69961	10.27728	9.989909	10.25319	10.60612	p11332	Insulin rece	1	10.57289			IGF1 pathw	GO:0060397; P:JAK-STAT cascade involved in growth hormone signaling pathway; GO:0048015; P:phosphatidylinositol-mediated signaling; GO:0008286; P:insulin receptor signaling pathway; GO:0008543; P:fibroblast growth factor receptor signaling pathway; GO:0007173; P:epidermal growth fact [illegible]
IRF2BPL se	6.756399	8.359229	8.562607	10.28469	0.484869	0.495046	0.239244	0.576073	p07009	Interferon r	1	4.469769				
IRF2BP2 se	0	10.31259	5.82647	8.222613	0	0	0	0	p13875	Interferon r	1	3.04521				
IQCE seq: 1	7.559248	9.043925	6.121011	8.151338	8.027463	9.365517	8.079257	8.147275	p06253	IQ domain-c	1	8.061879				
INCENP seq	7.944644	7.620865	6.284182	8.020449	-0.35192	0.279221	0.710872	-2.2765	p11238	Inner centro	1	3.528977			Aurora C signaling; Aurora B signaling; PLK1 signaling events; Regulation of nuclear beta catenin signaling and target gene transcription;	
INADL seq:	0	6.823926	8.698909	9.955005	0.147111	0.182862	0.16148	0.427209	p20137	InaD-like pr	1	3.299563				GO:0035556; P:intracellular signal transduction;
HTATSF1 pS	8.143226	8.712596	8.217696	8.117469	8.533842	8.274757	8.171655	9.037264	p01575	HIV Tat-spe	1	8.401063				
HSPB1 pS6	8.272798	12.11765	8.149992	7.580064	0	0	0	0	p11127	Heat shock	1	4.515064			p38 signalin	GO:0043122; P:regulation of I-kappaB kinase/NF-kappaB signaling; GO:0035924; P:cellular response to vascular endothelial growth factor stimulus; GO:0006469; P:negative regulation of protein kinase activity; GO:0035556; P:intracellular signal transduction;
HNRNPM s	6.446851	9.674515	4.912855	6.917154	1.092253	1.344827	-3.53488	2.09985	p10092	Heterogene	1	3.619178				
HNRNPK se	6.775288	6.731028	6.772708	6.141899	0.804204	0.486985	1.08752	0.815939	p14439	Heterogene	1	3.701946				GO:0007165; P:signal transduction;
HNRNPK se	10.72416	10.77288	10.69504	11.94763	-0.21119	-0.03336	-0.69611	-0.37248	p09036	Heterogene	1	5.353321				GO:0007165; P:signal transduction;
HNRNPC p	7.653456	5.86	7.863154	8.057319	1.838082	2.422648	4.525979	2.986769	p09708	Heterogene	1	5.150926			Regulation of Telomerase;	
HNRNPA2B	8.733093	12.00584	9.873297	0	7.986572	6.35852	0	7.27191	p18050	Heterogene	1	6.528654				
HN1 seq: 8	12.01685	12.19191	10.01931	9.507791	0	0	0	0	p09086	Hematologi	1	5.466983			Regulation (	GO:0035584; P:calcium-mediated signaling using intracellular calcium source; GO:0007218; P:neuropeptide signaling pathway; GO:0051056; P:regulation of small GTPase mediated signal transduction; GO:0007186; P:G-protein coupled receptor signaling pathway; GO:0007264; P:small GTPase me [illegible]
HN1 pS131	0	10.16468	9.747615	8.575617	0	0	0	0	p16170	Hematologi	1	3.560989		Up with IGF	Regulation (	GO:0035584; P:calcium-mediated signaling using intracellular calcium source; GO:0007218; P:neuropeptide signaling pathway; GO:0051056; P:regulation of small GTPase mediated signal transduction; GO:0007186; P:G-protein coupled receptor signaling pathway; GO:0007264; P:small GTPase me [illegible]
HMGCS1 se	11.58587	11.32282	11.15248	7.333396	-2.04226	-1.07038	-1.28224	-1.53443	p06830	Hydroxyme	1	4.433156	CDK1;	AKTinh2 (M	FOXA2 and FOXA3 transcription factor networks;	
HIRIP3 seq:	10.99742	8.384278	10.10364	8.209359	-0.96248	0.638616	-0.08965	0.805808	p15336	HIRA-intera	1	4.760874				
HDGF seq:	5.815272	9.302892	6.515899	7.543522	2.283367	1.357646	-5.2226	-0.1128	p19057	Hepatoma-d	1	3.435399				GO:0006987; P:activation of signaling protein activity involved in unfolded protein response; GO:0007165; P:signal transduction;
HDAC1 pS4	7.029108	8.037865	7.737538	7.858547	0.571749	0.304982	0.89784	0.4795	p14534	Histone dea	1	4.114641	CK2A1;		Sumoylation	GO:0060766; P:negative regulation of androgen receptor signaling pathway; GO:0007219; P:Notch signaling pathway; GO:0007179; P:transforming growth factor beta receptor signaling pathway; GO:0048011; P:neurotrophin TRK receptor signaling pathway;
GTSE1 seq:	10.61829	8.189085	11.12359	10.9232	5.723553	6.854421	9.021965	0	p13291	G2 and S ph	1	7.806763				
GPS1 pS47	8.043253	7.972783	6.529232	8.055275	-0.10966	0.017599	-0.0293	0.733289	p17675	COP9 signa	1	3.901559	ATR;			GO:0000188; P:inactivation of MAPK activity;
GPHN seq:	10.74224	11.87411	10.80855	10.688	0.368139	0.598207	0.579405	0.44223	p16984	Gephyrin	1	5.762611				
GORASP2 p	7.214791	11.80853	5.654522	9.280214	0	0	0	0	p13714	Golgi reasse	1	4.244758	ERK2; ERK1	PKCinh1 (Go-6976); EGFRinh1 (PD-168393);		
GOLGB1 pS	11.19174	10.79133	9.830718	10.62775	0.174029	0.41153	-0.54628	0.344153	p19971	Golgin subf	1	5.35312				
GOLGA5 pS	9.392013	10.51105	8.470658	6.32765	-0.34017	0.152497	0.439802	1.119329	p13038	Golgin subf	1	4.509104		PKCinh2 (Bisindolylmaleimide-1); PKCinh1 (Go-6976); p70S6Kinh1 (PF-4708671);		
GOLGA4 se	7.160677	8.618149	9.197033	8.588469	0.370804	0.482131	-0.05174	0.269239	p08933	Golgin subf	1	4.329345				
GNG7 seq:	8.835454	10.42986	9.542488	9.050915	1.125846	0.920112	-4.38785	1.291619	p10437	Guanine nu	1	4.601056				GO:0007168; P:receptor guanylyl cyclase signaling pathway; GO:0008277; P:regulation of G-protein coupled receptor protein signaling pathway; GO:0007186; P:G-protein coupled receptor signaling pathway;
GLOD4 seq	7.131963	9.350055	4.913622	7.173592	-1.31229	2.832294	-5.26701	3.225928	p11956	Glyoxalase	1	3.506019				
GIT1 seq: 3	7.546679	6.783229	9.733343	7.41883	0	0	0	0	p04213	ARF GTPase	1	3.93526			Regulation (	GO:0008277; P:regulation of G-protein coupled receptor protein signaling pathway;
GIT1 seq: 3	8.85231	8.220148	10.40165	10.00825	0	0	0	0	p04214	ARF GTPase	1	4.685295			Regulation (	GO:0008277; P:regulation of G-protein coupled receptor protein signaling pathway;
GDI2 seq: 3	9.234965	9.606263	8.456226	5.780496	2.126338	3.325245	0	3.122307	p16378	Rab GDP di	1	5.20648				GO:0051056; P:regulation of small GTPase mediated signal transduction; GO:0007264; P:small GTPase mediated signal transduction; GO:0007165; P:signal transduction;
GAPDH seq	11.87912	13.64463	10.13112	0	0.39559	2.407257	-4.73706	-4.69621	p01391	Glyceraldeh	1	3.628055			Validated targets of C-MYC transcriptional activation;	
GAPDH pM	10.50197	8.616587	8.664375	0	-7.24993	-1.16754	-3.39068	3.161008	p01389	Glyceraldeh	1	2.391974			Validated targets of C-MYC transcriptional activation;	
GAB2 pS54	0	9.007108	7.721037	7.369761	0.116998	-0.43293	0.099502	-0.02326	p09287	GRB2-assoc	1	2.982277			IL3-mediate	GO:0007169; P:transmembrane receptor protein tyrosine kinase signaling pathway; GO:0048015; P:phosphatidylinositol-mediated signaling; GO:0038095; P:Fc-epsilon receptor signaling pathway;
G6PD seq:	11.50325	12.08454	11.88897	11.64073	0.787887	1.977279	0.673055	1.734754	p12651	Glucose-6-p	1	6.536307				
G6PD seq:	9.71597	12.18194	9.549456	8.829735	2.870039	2.772704	-5.9545	3.218253	p07035	Glucose-6-p	1	5.39795				
G3BP1 seq	10.32349	11.42917	8.721133	0	-4.96455	-2.82603	-10.0503	-10.0095	p14574	Ras GTPase	1	0.327925				GO:0007265; P:Ras protein signal transduction; GO:0090090; P:negative regulation of canonical Wnt signaling pathway;
FUBP1 pT1	10.19271	9.851859	9.256124	7.87277	0	0	0	0	p06146	Far upstrea	1	4.646682				
FRYL pT195	9.386061	8.790138	11.84426	6.968494	2.607621	-0.76742	0.694011	-1.50553	p04745	Protein furr	1	4.752205				
FRMD8 pS	12.67424	12.09258	12.40671	12.32977	-1.25218	-0.87682	-1.02542	-0.81752	p08303	FERM doma	1	5.691418				
FLNC pS22	7.549501	12.47589	12.27943	10.70305	-0.05207	-1.4657	-0.18719	-0.28853	p17229	Filamin-C	1	5.126798	Akt2; Akt1;			
FLNB seq: 2	12.36383	13.35921	12.00127	12.27573	9.608161	9.048398	6.050289	7.858894	p19873	Filamin-B	1	10.32072				GO:0019221; P:cytokine-mediated signaling pathway; GO:0007165; P:signal transduction;
FLNA pS17	8.367184	12.47582	8.486827	9.522535	-0.59497	0.120314	-0.74328	0.376949	p16758	Filamin-A	1	4.751421			Noncanoni	GO:0043123; P:positive regulation of I-kappaB kinase/NF-kappaB signaling;
FKBP3 seq:	10.10687	9.640631	10.14343	10.3737	-8.13971	-8.08536	-7.5933	-7.55245	p13204	Peptidyl-pro	1	1.111728		Up with IGF	Signaling events mediated by HDAC Class I;	
FKBP15 sec	12.23688	12.86604	12.10474	11.94094	-13.0953	-0.59891	-0.64999	-0.60088	p17224	FK506-bind	1	4.275435				
FH pM151	10.09093	10.10116	8.745052	6.877406	0	0	0	0	p11203	Fumarate hy	1	4.476818				
FASN seq: 9	8.00971	10.70618	5.084827	5.858147	-0.17027	-0.56317	-1.79579	0.632174	p11909	Fatty acid sy	1	3.470226			Validated transcriptional targets of deltaNp63 isoforms; p73 transcription factor network;	
FAM129B p	9.32729	9.838649	12.29843	10.07344	3.295611	3.102992	3.522873	4.34348	p07681	Niban-like p	1	6.975345		EGFRinh1 (PD-168393); CAMKinh2 (KN-62);		
FAM129A s	11.19055	10.98834	9.37015	10.8395	0	0	0	0	p14712	Protein Nib	1	5.298569				
EWSR1 seq	6.446851	9.674515	4.912855	6.917154	1.092253	1.344827	-3.53488	2.09985	p10121	RNA-bindin	1	3.619178			BARD1 signaling events;	
ETV6 pS213	4.757565	6.966822	4.893574	6.663835	0.555011	0.784516	1.393519	0.646903	p07102	Transcriptio	1	3.332718	ERK1;			
ERF seq: 51	8.460054	9.860695	8.031435	7.789904	0.11269	0.250687	0.352539	0.106476	p16164	ETS domain	1	4.37056	ERK2;		IL2 signaling events mediated by STAT5; IL12 signaling mediated by STAT4; Caspase cascade in apoptosis; TCR signaling in naïve CD8+ T cells; Downstream signaling in naïve CD8+ T cells;	
EPPK1 pT1	0	9.221774	7.69327	9.45213	0	0	0	0	p12488	Epiplakin	1	3.295897				
ENTHD2 pS	6.520358	6.514602	5.337239	7.060438	0.035975	0.228538	-0.1983	-0.00194	p14837	AP-4 compl	1	3.187114				
EIF4G1 pS1	9.66132	9.22001	9.207776	9.118032	3.04123	3.423755	3.876271	3.615094	p05844	Eukaryotic t	1	6.395436		PAKinh (PF	Validated ta	GO:0008286; P:insulin receptor signaling pathway; GO:0019221; P:cytokine-mediated signaling pathway;
EEPD1 pS1	6.302253	7.283717	7.236647	7.819832	0.053457	-0.41126	0.300173	-0.22489	p03370	Endonuclea	1	3.544991				
DVL3 pS19	9.910472	10.17751	8.796957	8.781222	0	0	7.311479	0	p06760	Segment po	1	5.622205			Degradation	GO:0060071; P:Wnt signaling pathway, planar cell polarity pathway; GO:0060070; P:canonical Wnt signaling pathway; GO:0016055; P:Wnt signaling pathway; GO:0035556; P:intracellular signal transduction;
DOCK7 seq	6.913065	5.556276	9.417499	9.485017	0.480299	-0.09525	0.243123	0.237366	p05036	Dedicator o	1	4.029674			ErbB2/ErbB	GO:0007264; P:small GTPase mediated signal transduction;
DOCK7 seq	9.535002	9.887267	7.972192	8.823282	0.198543	0.334573	0.245846	0.15806	p11101	Dedicator o	1	4.644345			ErbB2/ErbB	GO:0007264; P:small GTPase mediated signal transduction;
DNTTIP2 se	8.506084	7.883272	8.219197	9.294201	-0.15173	0.116842	0.033287	-0.04993	p15101	Deoxynucle	1	4.231403		EGFRinh2 (PD-153035);		
DHX9 seq:	8.431978	7.139588	6.401792	5.843244	0.56321	0.995683	-6.05007	-0.78294	p09675	ATP-depend	1	2.817811				
DGCR14 pS	8.214353	8.682029	8.888375	8.96516	0.435563	0.111926	-0.17813	-0.5846	p15641	Protein DGC	1	4.316834				
DCAF6 pS3	10.00356	10.24273	8.318932	8.496196	0	0	0	0	p18831	DDB1- and	1	4.632678				
CYFIP2 seq:	0	11.39177	7.824837	6.788993	2.356962	1.925398	0.251475	3.057707	p14558	Cytoplasmic	1	4.199642			E-cadherin s	GO:0038096; P:Fc-gamma receptor signaling pathway involved in phagocytosis;
CUX1 seq:	10.80558	9.346359	10.67651	10.39788	-0.11347	-0.36258	0.249122	0.093969	p14601	Homeobox	1	5.136671	PKACA;			
CTNND1 se	8.004526	5.794871	8.294917	7.955196	0.752275	0.446979	0.583836	1.144797	p03166	Catenin delt	1	4.122175			E-cadherin s	GO:0090090; P:negative regulation of canonical Wnt signaling pathway; GO:0016055; P:Wnt signaling pathway;
CRKL seq: 1	9.073113	12.00579	10.07881	0	0	0	0	0	p14801	Crk-like pro	1	3.894714			JNK signalin	GO:0000186; P:activation of MAPKK activity; GO:0009967; P:positive regulation of signal transduction; GO:0007265; P:Ras protein signal transduction; GO:0048011; P:neurotrophin TRK receptor signaling pathway; GO:0035556; P:intracellular signal transduction;
CREBBP sec	9.123448	8.670308	10.58833	10.84531	-7.76526	1.203992	-7.21885	-7.178	p08952	CREB-bindin	1	2.28366			Signaling ev	GO:0008589; P:regulation of smoothened signaling pathway; GO:0007219; P:Notch signaling pathway; GO:0008543; P:fibroblast growth factor receptor signaling pathway; GO:0007165; P:signal transduction;
CNKSR1 sec	8.208038	5.636803	5.1872	7.50691	-0.26331	-0.04859	0.455819	0.659644	p02184	Connector o	1	3.417814			IL3-mediate	GO:0007266; P:Rho protein signal transduction; GO:0007169; P:transmembrane receptor protein tyrosine kinase signaling pathway; GO:0007265; P:Ras protein signal transduction;
CLIC6 seq:	9.203495	10.08189	7.699632	5.903713	-1.8456	1.757	2.185252	2.274205	p13244	Chloride int	1	4.657448				
CHORDC1 s	7.770226	10.42716	6.882628	6.995768	0.018357	1.94617	1.236097	-4.77102	p06708	Cysteine an	1	3.813173				
CHD4 pS42	7.798044	9.699072	7.687071	6.497467	-1.97652	-2.33202	-2.67844	-1.43697	p17737	Chromodor	1	2.907213			Signaling events mediated by HDAC Class I;	
CHAMP1 p	11.81182	12.15759	11.54721	10.98399	0	0	0	0	p11675	Chromosor	1	5.812575				
CEP170 sec	10.10767	10.83671	8.221807	9.867509	0.38501	0.522806	0.160861	0.252024	p09245	Centrosom	1	5.044299				
CDC6 pS54	7.522142	0	5.787021	5.602428	-0.32447	-0.1636	-0.49895	-0.65585	p20206	Cell divisior	1	2.15859	CDK2;		ATR signaling pathway; E2F transcription factor network;	
CDC26 pS4	0	7.792625	9.987286	8.715038	0.018812	0.238432	0.032829	0.034409	p08470	Anaphase-p	1	3.352429				
CCNT2 seq:	0	7.601668	8.60563	5.875408	-6.48923	-6.43489	0.54547	0.307099	p08621	Cyclin-T2	1	1.251395				GO:0007179; P:transforming growth factor beta receptor signaling pathway;
CCDC97 pS	9.75461	9.917597	10.01197	10.95203	1.067187	0.52432	1.430321	1.539448	p11303	Coiled-coil	1	5.649685				
CASKIN2 se	7.898653	9.409171	11.36493	9.784125	0	0	0	0	p02558	Caskin-2	1	4.80711				
C6orf132 s	10.93207	12.01402	7.154181	9.355044	-8.48414	-8.4298	-7.93773	-7.89688	p05816	Uncharacte	1	0.838345				
C2orf44 se	9.889308	9.132632	10.48001	8.57133	0.177978	-0.16199	-0.53098	0.018891	p17603	WD repeat	1	4.697147				
BRWD1 pS	7.885765	7.768844	7.943268	8.073654	-1.40741	0.690666	-5.93547	-0.39685	p07131	Bromodom	1	3.077808				
BIN1 seq: 2	4.921077	5.485516	8.979768	6.901759	0.483686	0.118687	0.140117	0.538739	p04390	Myc box-de	1	3.446169			Arf6 trafficking events;	
ATXN1L sec	7.71139	10.08086	7.715193	10.91063	0.830714	0.229721	-0.14264	0.093661	p00827	Ataxin-1-lik	1	4.678691				
ATRX seq: 5	10.97618	0	0	9.791676	1.710468	1.552792	1.715598	2.343685	p10512	Transcriptio	1	3.5113		mTOR-PI3K; PI3Kinh2 (PI-103); PI3Kinh1 (GDC-0941); mTORinh2 (Torin-1); mTORinh1 (KU-0063794);		
ATN1 seq: 7	0	6.333983	7.248609	8.557417	-0.1017	0.022103	-0.76019	0.339855	p06044	Atrophin-1	1	2.70501				
ARL6IP4 se	9.325218	10.49843	8.349964	11.38542	0.227698	0.300829	-0.24254	0.20722	p19459	ADP-ribosy	1	5.006531				
ARHGEF5 s	9.016448	10.07703	10.72428	10.96368	0	0	0	0	p08753	Rho guanin	1	5.09768			Regulation (	GO:0035556; P:intracellular signal transduction;
ARHGEF5 s	8.959977	10.07703	10.65841	10.94524	0	0	0	0	p08754	Rho guanin	1	5.080083			Regulation (	GO:0035556; P:intracellular signal transduction;
ARHGEF12	11.06486	11.49385	9.916779	9.652324	0	8.508478	5.992143	0	p13257	Rho guanin	1	7.078554			Regulation (	GO:0038032; P:termination of G-protein coupled receptor signaling pathway; GO:0051056; P:regulation of small GTPase mediated signal transduction; GO:0048011; P:neurotrophin TRK receptor signaling pathway; GO:0007186; P:G-protein coupled receptor signaling pathway; GO:0007264; P:small GTPase mediated signal transduction;
ARFGAP1 p	9.923882	10.16946	9.082412	9.764731	-0.10286	-0.18711	0.081789	-0.50928	p00027	ADP-ribosy	1	4.777879			Arf1 pathwa	GO:0006987; P:activation of signaling protein activity involved in unfolded protein response;
ANXA3 seq	0	0	10.31778	10.14212	1.259047	1.064245	0.925479	1.205314	p06068	Annexin A3	1	3.114248				
ANXA1 seq	7.581328	13.44451	13.53168	14.35742	0.365955	-0.02144	-0.2495	-0.19813	p15993	Annexin A1	1	6.101477				GO:0007166; P:cell surface receptor signaling pathway; GO:0007165; P:signal transduction;
ALDOA pS3	10.61733	7.160735	10.11654	10.215	3.452272	2.567715	1.954872	4.471794	p00047	Fructose-bi	1	6.319532			HIF-1-alpha transcription factor network;	
AKT1 seq: 1	11.08961	8.679852	10.4635	11.52383	-6.51646	-6.46211	-5.97005	-5.9292	p05664	RAC-alpha s	1	2.109872	CK2A1;		E-cadherin s	GO:0048009; P:insulin-like growth factor receptor signaling pathway; GO:0071364; P:cellular response to epidermal growth factor stimulus; GO:0043491; P:protein kinase B signaling; GO:0006469; P:negative regulation of protein kinase activity; GO:0048015; P:phosphatidylinositol-mediated signaling; GO:0008286; P:insulin receptor signaling pathway; GO:0008543; P:fibroblast growth factor receptor signaling pathway; GO:0007173; P:epidermal growth factor receptor signaling pathway; GO:0048011; P:neurotrophin TRK receptor signaling path [illegible]
AKAP12 sec	8.742052	6.358047	6.432164	5.347819	0.021185	-0.12725	-0.75075	0.362308	p01175	A-kinase an	1	3.298197				GO:0007186; P:G-protein coupled receptor signaling pathway;
AHNAK2 pS	11.68888	10.05855	8.708414	0	-0.56722	-0.0798	-0.30035	0.058187	p13853	Protein AHI	1	3.695833				
AHNAK seq	8.125871	6.300361	5.623373	7.058141	-0.3721	-0.2822	-0.26302	-0.23909	p01316	Neuroblast	1	3.243917				
AHNAK seq	9.263557	10.38845	11.76411	10.12828	-0.2622	-0.22426	-0.24324	-0.22683	p16988	Neuroblast	1	5.073484				
ACSS2 pS2	0	7.095416	9.410351	7.638239	0.344493	-0.10257	-0.09995	0.292953	p19671	Acetyl-coen	1	3.072367			Signaling events mediated by HDAC Class III;	
ACBD3 seq	9.990984	9.622271	9.857955	8.635844	5.433993	0	5.986242	0	p08332	Golgi reside	1	6.190911				
ABLIM3 pS	11.0652	0	0	9.575495	0.077132	-0.0699	0.10877	0.052426	p06262	Actin-bindi	1	2.601139				
ABCC2 seq:	10.89762	9.94958	10.3781	8.826679	0	0	0	0	p14590	Canalicular	1	5.006497				

HSPB1 pM	Heat shock protein beta-1 OS=Homo sapiens GN=HSPB1 PE=1 SV=2
HSPB1 pT	Heat shock protein beta-1 OS=Homo sapiens GN=HSPB1 PE=1 SV=2
LEO1 pS65	RNA polymerase-associated protein LEO1 OS=Homo sapiens GN=LEO1 PE=1 SV=1
C9orf142 p	Protein PAXX OS=Homo sapiens GN=C9orf142 PE=1 SV=2
C9orf142 p	Protein PAXX OS=Homo sapiens GN=C9orf142 PE=1 SV=2
HADHB seq	Trifunctional enzyme subunit beta, mitochondrial OS=Homo sapiens GN=HADHB PE=1 SV=3
KRT14 seq:	Keratin, type I cytoskeletal 14 OS=Homo sapiens GN=KRT14 PE=1 SV=4
NPM1 seq:	Nucleophosmin OS=Homo sapiens GN=NPM1 PE=1 SV=2
NPM1 seq:	Nucleophosmin OS=Homo sapiens GN=NPM1 PE=1 SV=2
FAM98C se	Protein FAM98C OS=Homo sapiens GN=FAM98C PE=2 SV=1
PLEC seq: 1	Plectin OS=Homo sapiens GN=PLEC PE=1 SV=3
PAK4 pT20	Serine/threonine-protein kinase PAK 4 OS=Homo sapiens GN=PAK4 PE=1 SV=1
PAK4 pT20	Serine/threonine-protein kinase PAK 4 OS=Homo sapiens GN=PAK4 PE=1 SV=1
PSMB6 seq	Proteasome subunit beta type-6 OS=Homo sapiens GN=PSMB6 PE=1 SV=4
PLEC seq: 1	Plectin OS=Homo sapiens GN=PLEC PE=1 SV=3
JUND pS25	Transcription factor jun-D OS=Homo sapiens GN=JUND PE=1 SV=3
JUND pS25	Transcription factor jun-D OS=Homo sapiens GN=JUND PE=1 SV=3
JUND pS25	Transcription factor jun-D OS=Homo sapiens GN=JUND PE=1 SV=3
JUND pS25	Transcription factor jun-D OS=Homo sapiens GN=JUND PE=1 SV=3
JUND pS25	Transcription factor jun-D OS=Homo sapiens GN=JUND PE=1 SV=3
JUND pS25	Transcription factor jun-D OS=Homo sapiens GN=JUND PE=1 SV=3
CLIC1 seq:	Chloride intracellular channel protein 1 OS=Homo sapiens GN=CLIC1 PE=1 SV=4
PPFIA1 pT5	Liprin-alpha-1 OS=Homo sapiens GN=PPFIA1 PE=1 SV=1
PPFIA1 pT5	Liprin-alpha-1 OS=Homo sapiens GN=PPFIA1 PE=1 SV=1
ATRIP pS22	ATR-interacting protein OS=Homo sapiens GN=ATRIP PE=1 SV=1
ATRIP pS22	ATR-interacting protein OS=Homo sapiens GN=ATRIP PE=1 SV=1
ANKZF1 pS	Ankyrin repeat and zinc finger domain-containing protein 1 OS=Homo sapiens GN=ANKZF1 PE=1 SV=1
DBN1 seq:	Drebrin OS=Homo sapiens GN=DBN1 PE=1 SV=4
STARD10 s	PCTP-like protein OS=Homo sapiens GN=STARD10 PE=1 SV=2
STARD10 p	PCTP-like protein OS=Homo sapiens GN=STARD10 PE=1 SV=2
EPPK1 seq:	Epiplakin OS=Homo sapiens GN=EPPK1 PE=1 SV=2
SEPT7 seq:	Septin-7 OS=Homo sapiens GN=SEPT7 PE=1 SV=2
LMNA seq:	Prelamin-A/C OS=Homo sapiens GN=LMNA PE=1 SV=1
BCAS3 pS8	Breast carcinoma-amplified sequence 3 OS=Homo sapiens GN=BCAS3 PE=1 SV=3
CELSR1 pS2	Cadherin EGF LAG seven-pass G-type receptor 1 OS=Homo sapiens GN=CELSR1 PE=1 SV=1
VCP seq: 39	Transitional endoplasmic reticulum ATPase OS=Homo sapiens GN=VCP PE=1 SV=4
STK25 pT1	Serine/threonine-protein kinase 25 OS=Homo sapiens GN=STK25 PE=1 SV=1
ARHGEF17	Rho guanine nucleotide exchange factor 17 OS=Homo sapiens GN=ARHGEF17 PE=1 SV=1
PFDN1 seq	Prefoldin subunit 1 OS=Homo sapiens GN=PFDN1 PE=1 SV=2
ALDH2 seq	Aldehyde dehydrogenase, mitochondrial OS=Homo sapiens GN=ALDH2 PE=1 SV=2
GPKOW pS	G patch domain and KOW motifs-containing protein OS=Homo sapiens GN=GPKOW PE=1 SV=2
UBL4A pS9	Ubiquitin-like protein 4A OS=Homo sapiens GN=UBL4A PE=1 SV=1
HECTD4 pS	Probable E3 ubiquitin-protein ligase HECTD4 OS=Homo sapiens GN=HECTD4 PE=1 SV=5
SCRIB pS15	Protein scribble homolog OS=Homo sapiens GN=SCRIB PE=1 SV=4
SCRIB pS15	Protein scribble homolog OS=Homo sapiens GN=SCRIB PE=1 SV=4
SCRIB pT15	Protein scribble homolog OS=Homo sapiens GN=SCRIB PE=1 SV=4
SCRIB pS15	Protein scribble homolog OS=Homo sapiens GN=SCRIB PE=1 SV=4
SCRIB pT15	Protein scribble homolog OS=Homo sapiens GN=SCRIB PE=1 SV=4
SCRIB pS15	Protein scribble homolog OS=Homo sapiens GN=SCRIB PE=1 SV=4
DIABLO seq	Diablo homolog, mitochondrial OS=Homo sapiens GN=DIABLO PE=1 SV=1
SF3B2 seq:	Splicing factor 3B subunit 2 OS=Homo sapiens GN=SF3B2 PE=1 SV=2
CLTC seq: 1	Clathrin heavy chain 1 OS=Homo sapiens GN=CLTC PE=1 SV=5
KRT83 seq:	Keratin, type II cuticular Hb3 OS=Homo sapiens GN=KRT83 PE=1 SV=2
BRD4 seq:	Bromodomain-containing protein 4 OS=Homo sapiens GN=BRD4 PE=1 SV=2
RBM14 seq	RNA-binding protein 14 OS=Homo sapiens GN=RBM14 PE=1 SV=2
TNRC18 pS	Trinucleotide repeat-containing gene 18 protein OS=Homo sapiens GN=TNRC18 PE=1 SV=3
ZC3H4 pS8	Zinc finger CCCH domain-containing protein 4 OS=Homo sapiens GN=ZC3H4 PE=1 SV=3
RFC4 seq: 3	Replication factor C subunit 4 OS=Homo sapiens GN=RFC4 PE=1 SV=2
PLEC seq: 1	Plectin OS=Homo sapiens GN=PLEC PE=1 SV=3
HDAC7 seq	Histone deacetylase 7 OS=Homo sapiens GN=HDAC7 PE=1 SV=2
SQSTM1 pS	Sequestosome-1 OS=Homo sapiens GN=SQSTM1 PE=1 SV=1
CXorf67 pS	Uncharacterized protein CXorf67 OS=Homo sapiens GN=CXorf67 PE=2 SV=1
KMT2D pS	Histone-lysine N-methyltransferase 2D OS=Homo sapiens GN=KMT2D PE=1 SV=2
VCP seq: 27	Transitional endoplasmic reticulum ATPase OS=Homo sapiens GN=VCP PE=1 SV=4
VCP seq: 27	Transitional endoplasmic reticulum ATPase OS=Homo sapiens GN=VCP PE=1 SV=4
PPP2R1A s	Serine/threonine-protein phosphatase 2A 65 kDa regulatory subunit A alpha isoform OS=Homo sapiens GN=PPP2R1A PE=1 SV=4
TJP2 seq: 5	Tight junction protein ZO-2 OS=Homo sapiens GN=TJP2 PE=1 SV=2
EIF4ENIF1 p	Eukaryotic translation initiation factor 4E transporter OS=Homo sapiens GN=EIF4ENIF1 PE=1 SV=2
HYOU1 seq	Hypoxia up-regulated protein 1 OS=Homo sapiens GN=HYOU1 PE=1 SV=1
DHRS13 pS	Dehydrogenase/reductase SDR family member 13 OS=Homo sapiens GN=DHRS13 PE=2 SV=1
DHRS13 pS	Dehydrogenase/reductase SDR family member 13 OS=Homo sapiens GN=DHRS13 PE=2 SV=1
PSMC3 seq	26S protease regulatory subunit 6A OS=Homo sapiens GN=PSMC3 PE=1 SV=3
MADD pS1	MAP kinase-activating death domain protein OS=Homo sapiens GN=MADD PE=1 SV=2
TPRN pS24	Taperin OS=Homo sapiens GN=TPRN PE=1 SV=2
TPRN pS24	Taperin OS=Homo sapiens GN=TPRN PE=1 SV=2
UBA1 seq:	Ubiquitin-like modifier-activating enzyme 1 OS=Homo sapiens GN=UBA1 PE=1 SV=3
UBA1 seq:	Ubiquitin-like modifier-activating enzyme 1 OS=Homo sapiens GN=UBA1 PE=1 SV=3
THYN1 pS1	Thymocyte nuclear protein 1 OS=Homo sapiens GN=THYN1 PE=1 SV=1
LINC01006	Putative uncharacterized protein encoded by LINC01006 OS=Homo sapiens GN=LINC01006 PE=5 SV=1
HIST3H2BB	Histone H2B type 3-B OS=Homo sapiens GN=HIST3H2BB PE=1 SV=3
MTHFD1 se	C-1-tetrahydrofolate synthase, cytoplasmic OS=Homo sapiens GN=MTHFD1 PE=1 SV=3
MLF2 pS23	Myeloid leukemia factor 2 OS=Homo sapiens GN=MLF2 PE=1 SV=1
VCP pS702	Transitional endoplasmic reticulum ATPase OS=Homo sapiens GN=VCP PE=1 SV=4
SCRIB pS14	Protein scribble homolog OS=Homo sapiens GN=SCRIB PE=1 SV=4
LMNB1 pS	Lamin-B1 OS=Homo sapiens GN=LMNB1 PE=1 SV=2
LMNB1 pS	Lamin-B1 OS=Homo sapiens GN=LMNB1 PE=1 SV=2
LMNB1 pS	Lamin-B1 OS=Homo sapiens GN=LMNB1 PE=1 SV=2
GOT1 seq:	Aspartate aminotransferase, cytoplasmic OS=Homo sapiens GN=GOT1 PE=1 SV=3
DCAF7 seq:	DDB1- and CUL4-associated factor 7 OS=Homo sapiens GN=DCAF7 PE=1 SV=1
DCAF7 seq:	DDB1- and CUL4-associated factor 7 OS=Homo sapiens GN=DCAF7 PE=1 SV=1
CROCC seq	Rootletin OS=Homo sapiens GN=CROCC PE=1 SV=1
MMACHC p	Methylmalonic aciduria and homocystinuria type C protein OS=Homo sapiens GN=MMACHC PE=1 SV=3
NUMA1 sec	Nuclear mitotic apparatus protein 1 OS=Homo sapiens GN=NUMA1 PE=1 SV=2
NFIC pS473	Nuclear factor 1 C-type OS=Homo sapiens GN=NFIC PE=1 SV=2
NFIC pS473	Nuclear factor 1 C-type OS=Homo sapiens GN=NFIC PE=1 SV=2
CKAP4 seq:	Cytoskeleton-associated protein 4 OS=Homo sapiens GN=CKAP4 PE=1 SV=2
TNRC18 pS	Trinucleotide repeat-containing gene 18 protein OS=Homo sapiens GN=TNRC18 PE=1 SV=3
CENPF pS3	Centromere protein F OS=Homo sapiens GN=CENPF PE=1 SV=2
CENPF pS3	Centromere protein F OS=Homo sapiens GN=CENPF PE=1 SV=2
CCT2 seq: 3	T-complex protein 1 subunit beta OS=Homo sapiens GN=CCT2 PE=1 SV=4
ENO1 seq:	Alpha-enolase OS=Homo sapiens GN=ENO1 PE=1 SV=2
ENO1 seq:	Alpha-enolase OS=Homo sapiens GN=ENO1 PE=1 SV=2
ENO1 pM1	Alpha-enolase OS=Homo sapiens GN=ENO1 PE=1 SV=2
ENO1 pM1	Alpha-enolase OS=Homo sapiens GN=ENO1 PE=1 SV=2
ENO1 pM1	Alpha-enolase OS=Homo sapiens GN=ENO1 PE=1 SV=2
ZNF462 pS	Zinc finger protein 462 OS=Homo sapiens GN=ZNF462 PE=1 SV=3
CHD4 seq:	Chromodomain-helicase-DNA-binding protein 4 OS=Homo sapiens GN=CHD4 PE=1 SV=2
FSIP2 seq: 5	Fibrous sheath-interacting protein 2 OS=Homo sapiens GN=FSIP2 PE=2 SV=4
RPL23A sec	60S ribosomal protein L23a OS=Homo sapiens GN=RPL23A PE=1 SV=1
PRDX6 seq	Peroxiredoxin-6 OS=Homo sapiens GN=PRDX6 PE=1 SV=3
PDIA3 seq:	Protein disulfide-isomerase A3 OS=Homo sapiens GN=PDIA3 PE=1 SV=4
PKM seq: 4	Pyruvate kinase PKM OS=Homo sapiens GN=PKM PE=1 SV=4
PKM seq: 4	Pyruvate kinase PKM OS=Homo sapiens GN=PKM PE=1 SV=4
PKM pS40	Pyruvate kinase PKM OS=Homo sapiens GN=PKM PE=1 SV=4
PKM pT40	Pyruvate kinase PKM OS=Homo sapiens GN=PKM PE=1 SV=4
NCR3 seq:	Natural cytotoxicity triggering receptor 3 OS=Homo sapiens GN=NCR3 PE=1 SV=1
KLHDC8A p	Kelch domain-containing protein 8A OS=Homo sapiens GN=KLHDC8A PE=2 SV=2
ATF7IP pS	Activating transcription factor 7-interacting protein 1 OS=Homo sapiens GN=ATF7IP PE=1 SV=3
ERP44 seq:	Endoplasmic reticulum resident protein 44 OS=Homo sapiens GN=ERP44 PE=1 SV=1
CHAMP1 p	Chromosome alignment-maintaining phosphoprotein 1 OS=Homo sapiens GN=CHAMP1 PE=1 SV=2
CHAMP1 p	Chromosome alignment-maintaining phosphoprotein 1 OS=Homo sapiens GN=CHAMP1 PE=1 SV=2
CHAMP1 p	Chromosome alignment-maintaining phosphoprotein 1 OS=Homo sapiens GN=CHAMP1 PE=1 SV=2
CHAMP1 p	Chromosome alignment-maintaining phosphoprotein 1 OS=Homo sapiens GN=CHAMP1 PE=1 SV=2
SLX4 pS102	Structure-specific endonuclease subunit SLX4 OS=Homo sapiens GN=SLX4 PE=1 SV=3
ENO1 seq:	Alpha-enolase OS=Homo sapiens GN=ENO1 PE=1 SV=2
DPP3 seq:	Dipeptidyl peptidase 3 OS=Homo sapiens GN=DPP3 PE=1 SV=2
DPP3 seq:	Dipeptidyl peptidase 3 OS=Homo sapiens GN=DPP3 PE=1 SV=2
PLEC seq: 2	Plectin OS=Homo sapiens GN=PLEC PE=1 SV=3
SH3PXD2A	SH3 and PX domain-containing protein 2A OS=Homo sapiens GN=SH3PXD2A PE=1 SV=1
FAM114A1	Protein NOXP20 OS=Homo sapiens GN=FAM114A1 PE=1 SV=2
SF3B2 seq:	Splicing factor 3B subunit 2 OS=Homo sapiens GN=SF3B2 PE=1 SV=2
RBM33 pS	RNA-binding protein 33 OS=Homo sapiens GN=RBM33 PE=1 SV=3
CELSR3 seq	Cadherin EGF LAG seven-pass G-type receptor 3 OS=Homo sapiens GN=CELSR3 PE=1 SV=2
PML pS504	Protein PML OS=Homo sapiens GN=PML PE=1 SV=3
AKAP8 pS3	A-kinase anchor protein 8 OS=Homo sapiens GN=AKAP8 PE=1 SV=1
RBM10 pS	RNA-binding protein 10 OS=Homo sapiens GN=RBM10 PE=1 SV=3
RBM10 pS	RNA-binding protein 10 OS=Homo sapiens GN=RBM10 PE=1 SV=3
ACTN4 seq:	Alpha-actinin-4 OS=Homo sapiens GN=ACTN4 PE=1 SV=2
ORC2 pT11	Origin recognition complex subunit 2 OS=Homo sapiens GN=ORC2 PE=1 SV=2
GGA3 seq:	ADP-ribosylation factor-binding protein GGA3 OS=Homo sapiens GN=GGA3 PE=1 SV=1
THOC3 seq	THO complex subunit 3 OS=Homo sapiens GN=THOC3 PE=1 SV=1
ARHGAP11	Rho GTPase-activating protein 11A OS=Homo sapiens GN=ARHGAP11A PE=1 SV=2
CCDC6 seq	Coiled-coil domain-containing protein 6 OS=Homo sapiens GN=CCDC6 PE=1 SV=2
JUN pS100	Transcription factor AP-1 OS=Homo sapiens GN=JUN PE=1 SV=2
TRIM28 pS	Transcription intermediary factor 1-beta OS=Homo sapiens GN=TRIM28 PE=1 SV=5
TRIM28 pS	Transcription intermediary factor 1-beta OS=Homo sapiens GN=TRIM28 PE=1 SV=5
TRIM28 pT	Transcription intermediary factor 1-beta OS=Homo sapiens GN=TRIM28 PE=1 SV=5
TRIM28 pS	Transcription intermediary factor 1-beta OS=Homo sapiens GN=TRIM28 PE=1 SV=5
TRIM28 pS	Transcription intermediary factor 1-beta OS=Homo sapiens GN=TRIM28 PE=1 SV=5
AP2B1 seq:	AP-2 complex subunit beta OS=Homo sapiens GN=AP2B1 PE=1 SV=1
RPL19 pS1	60S ribosomal protein L19 OS=Homo sapiens GN=RPL19 PE=1 SV=1
UBAP1 pS2	Ubiquitin-associated protein 1 OS=Homo sapiens GN=UBAP1 PE=1 SV=1
UBAP1 pS2	Ubiquitin-associated protein 1 OS=Homo sapiens GN=UBAP1 PE=1 SV=1
CLTC seq: 1	Clathrin heavy chain 1 OS=Homo sapiens GN=CLTC PE=1 SV=5
PPP1R12A	Protein phosphatase 1 regulatory subunit 12A OS=Homo sapiens GN=PPP1R12A PE=1 SV=1
PPP1R12A	Protein phosphatase 1 regulatory subunit 12A OS=Homo sapiens GN=PPP1R12A PE=1 SV=1
COASY pS1	Bifunctional coenzyme A synthase OS=Homo sapiens GN=COASY PE=1 SV=4
COASY pS1	Bifunctional coenzyme A synthase OS=Homo sapiens GN=COASY PE=1 SV=4
RPLP2 seq:	60S acidic ribosomal protein P2 OS=Homo sapiens GN=RPLP2 PE=1 SV=1
RPLP2 seq:	60S acidic ribosomal protein P2 OS=Homo sapiens GN=RPLP2 PE=1 SV=1
RPLP2 pS7	60S acidic ribosomal protein P2 OS=Homo sapiens GN=RPLP2 PE=1 SV=1
RPLP2 pS6	60S acidic ribosomal protein P2 OS=Homo sapiens GN=RPLP2 PE=1 SV=1
RPLP2 pS7	60S acidic ribosomal protein P2 OS=Homo sapiens GN=RPLP2 PE=1 SV=1
KRT16 seq:	Keratin, type I cytoskeletal 16 OS=Homo sapiens GN=KRT16 PE=1 SV=4
KRT18 seq:	Keratin, type I cytoskeletal 18 OS=Homo sapiens GN=KRT18 PE=1 SV=2
KRT18 pS9	Keratin, type I cytoskeletal 18 OS=Homo sapiens GN=KRT18 PE=1 SV=2
FAM83H p	Protein FAM83H OS=Homo sapiens GN=FAM83H PE=1 SV=3
CDCP1 pS8	CUB domain-containing protein 1 OS=Homo sapiens GN=CDCP1 PE=1 SV=3
WDR1 seq:	WD repeat-containing protein 1 OS=Homo sapiens GN=WDR1 PE=1 SV=4
WDR1 seq:	WD repeat-containing protein 1 OS=Homo sapiens GN=WDR1 PE=1 SV=4
LIG3 pS913	DNA ligase 3 OS=Homo sapiens GN=LIG3 PE=1 SV=2
MYO18A p	Unconventional myosin-XVIIIa OS=Homo sapiens GN=MYO18A PE=1 SV=3
CCT8 seq: 5	T-complex protein 1 subunit theta OS=Homo sapiens GN=CCT8 PE=1 SV=4
MAP4 pS92	Microtubule-associated protein 4 OS=Homo sapiens GN=MAP4 PE=1 SV=3
RPL13 seq:	60S ribosomal protein L13 OS=Homo sapiens GN=RPL13 PE=1 SV=4
HSPB1 seq:	Heat shock protein beta-1 OS=Homo sapiens GN=HSPB1 PE=1 SV=2
HSPB1 seq:	Heat shock protein beta-1 OS=Homo sapiens GN=HSPB1 PE=1 SV=2
HSPB1 pS1	Heat shock protein beta-1 OS=Homo sapiens GN=HSPB1 PE=1 SV=2
HSPB1 pS1	Heat shock protein beta-1 OS=Homo sapiens GN=HSPB1 PE=1 SV=2
DMGDH se	Dimethylglycine dehydrogenase, mitochondrial OS=Homo sapiens GN=DMGDH PE=1 SV=2
TMEM151A	Transmembrane protein 151A OS=Homo sapiens GN=TMEM151A PE=2 SV=1
TPR seq: 77	Nucleoprotein TPR OS=Homo sapiens GN=TPR PE=1 SV=3
TFRC seq: 4	Transferrin receptor protein 1 OS=Homo sapiens GN=TFRC PE=1 SV=2
HAS1 seq: 1	Hyaluronan synthase 1 OS=Homo sapiens GN=HAS1 PE=1 SV=2
CCT2 seq: 1	T-complex protein 1 subunit beta OS=Homo sapiens GN=CCT2 PE=1 SV=4
LAMTOR1 p	Ragulator complex protein LAMTOR1 OS=Homo sapiens GN=LAMTOR1 PE=1 SV=2
LAMTOR1 p	Ragulator complex protein LAMTOR1 OS=Homo sapiens GN=LAMTOR1 PE=1 SV=2
EPCAM seq	Epithelial cell adhesion molecule OS=Homo sapiens GN=EPCAM PE=1 SV=2
TUBB2A seq	Tubulin beta-2A chain OS=Homo sapiens GN=TUBB2A PE=1 SV=1
C4orf50 pT	Uncharacterized protein C4orf50 OS=Homo sapiens GN=C4orf50 PE=2 SV=1
LMNB1 seq	Lamin-B1 OS=Homo sapiens GN=LMNB1 PE=1 SV=2
LMNA seq:	Prelamin-A/C OS=Homo sapiens GN=LMNA PE=1 SV=1
PRRC2A pS	Protein PRRC2A OS=Homo sapiens GN=PRRC2A PE=1 SV=3
DNAJC7 sec	DnaJ homolog subfamily C member 7 OS=Homo sapiens GN=DNAJC7 PE=1 SV=2
STIP1 seq: 3	Stress-induced-phosphoprotein 1 OS=Homo sapiens GN=STIP1 PE=1 SV=1
DDTL seq: 2	D-dopachrome decarboxylase-like protein OS=Homo sapiens GN=DDTL PE=2 SV=1
PGM2 pM1	Phosphoglucomutase-2 OS=Homo sapiens GN=PGM2 PE=1 SV=4
PGM2 pS16	Phosphoglucomutase-2 OS=Homo sapiens GN=PGM2 PE=1 SV=4
PGM2 pS16	Phosphoglucomutase-2 OS=Homo sapiens GN=PGM2 PE=1 SV=4
SLC9A3R1 s	Na(+)/H(+) exchange regulatory cofactor NHE-RF1 OS=Homo sapiens GN=SLC9A3R1 PE=1 SV=4
SORBS3 pS	Vinexin OS=Homo sapiens GN=SORBS3 PE=1 SV=2
PRPF4B pS	Serine/threonine-protein kinase PRP4 homolog OS=Homo sapiens GN=PRPF4B PE=1 SV=3
PRPF4B sec	Serine/threonine-protein kinase PRP4 homolog OS=Homo sapiens GN=PRPF4B PE=1 SV=3
PRPF4B sec	Serine/threonine-protein kinase PRP4 homolog OS=Homo sapiens GN=PRPF4B PE=1 SV=3
MAP2K2 pS	Dual specificity mitogen-activated protein kinase kinase 2 OS=Homo sapiens GN=MAP2K2 PE=1 SV=1
MAP2K1 pS	Dual specificity mitogen-activated protein kinase kinase 1 OS=Homo sapiens GN=MAP2K1 PE=1 SV=2
CLTA seq: 2	Clathrin light chain A OS=Homo sapiens GN=CLTA PE=1 SV=1
CHCHD2 se	Coiled-coil-helix-coiled-coil-helix domain-containing protein 2 OS=Homo sapiens GN=CHCHD2 PE=1 SV=1
DNAH9 seq	Dynein heavy chain 9, axonemal OS=Homo sapiens GN=DNAH9 PE=1 SV=3
EXOSC1 pS	Exosome complex component CSL4 OS=Homo sapiens GN=EXOSC1 PE=1 SV=1
LAMC1 seq	Laminin subunit gamma-1 OS=Homo sapiens GN=LAMC1 PE=1 SV=3
LAMC1 seq	Laminin subunit gamma-1 OS=Homo sapiens GN=LAMC1 PE=1 SV=3
FAM83B pT	Protein FAM83B OS=Homo sapiens GN=FAM83B PE=1 SV=1
UQCRC1 se	Cytochrome b-c1 complex subunit 1, mitochondrial OS=Homo sapiens GN=UQCRC1 PE=1 SV=3
AKAP3 seq:	A-kinase anchor protein 3 OS=Homo sapiens GN=AKAP3 PE=1 SV=2
AHCYL1 sec	Adenosylhomocysteinase 2 OS=Homo sapiens GN=AHCYL1 PE=1 SV=2
PGLS seq: 2	6-phosphogluconolactonase OS=Homo sapiens GN=PGLS PE=1 SV=2
ACTA1 pM2	Actin, alpha skeletal muscle OS=Homo sapiens GN=ACTA1 PE=1 SV=1
ACTC1 pM2	Actin, alpha cardiac muscle 1 OS=Homo sapiens GN=ACTC1 PE=1 SV=1
ACTA1 pM2	Actin, alpha skeletal muscle OS=Homo sapiens GN=ACTA1 PE=1 SV=1
ACTG1 seq:	Actin, cytoplasmic 2 OS=Homo sapiens GN=ACTG1 PE=1 SV=1
ACTG1 seq:	Actin, cytoplasmic 2 OS=Homo sapiens GN=ACTG1 PE=1 SV=1
ACTB pM22	Actin, cytoplasmic 1 OS=Homo sapiens GN=ACTB PE=1 SV=1
ACTG1 pM	Actin, cytoplasmic 2 OS=Homo sapiens GN=ACTG1 PE=1 SV=1
ACTG1 pS2	Actin, cytoplasmic 2 OS=Homo sapiens GN=ACTG1 PE=1 SV=1
ACTG1 pS2	Actin, cytoplasmic 2 OS=Homo sapiens GN=ACTG1 PE=1 SV=1
POTEE seq:	POTE ankyrin domain family member E OS=Homo sapiens GN=POTEE PE=1 SV=3
KRT7 seq: 7	Keratin, type II cytoskeletal 7 OS=Homo sapiens GN=KRT7 PE=1 SV=5
DENR seq:	Density-regulated protein OS=Homo sapiens GN=DENR PE=1 SV=2
CHMP5 sec	Charged multivesicular body protein 5 OS=Homo sapiens GN=CHMP5 PE=1 SV=1
ALDH1A3 p	Aldehyde dehydrogenase family 1 member A3 OS=Homo sapiens GN=ALDH1A3 PE=1 SV=2
CAPNS1 sec	Calpain small subunit 1 OS=Homo sapiens GN=CAPNS1 PE=1 SV=1
CAPNS1 pM	Calpain small subunit 1 OS=Homo sapiens GN=CAPNS1 PE=1 SV=1
RABGEF1 p	Rab5 GDP/GTP exchange factor OS=Homo sapiens GN=RABGEF1 PE=1 SV=2
LGALSL pS2	Galectin-related protein OS=Homo sapiens GN=LGALSL PE=1 SV=2
EIF2S3 seq:	Eukaryotic translation initiation factor 2 subunit 3 OS=Homo sapiens GN=EIF2S3 PE=1 SV=3
NDUFS2 se	NADH dehydrogenase [ubiquinone] iron-sulfur protein 2, mitochondrial OS=Homo sapiens GN=NDUFS2 PE=1 SV=2
NDUFS2 se	NADH dehydrogenase [ubiquinone] iron-sulfur protein 2, mitochondrial OS=Homo sapiens GN=NDUFS2 PE=1 SV=2
GGCT seq:	Gamma-glutamylcyclotransferase OS=Homo sapiens GN=GGCT PE=1 SV=1
EPB41 seq:	Protein 4.1 OS=Homo sapiens GN=EPB41 PE=1 SV=4
NAP1L1 sec	Nucleosome assembly protein 1-like 1 OS=Homo sapiens GN=NAP1L1 PE=1 SV=1
NAP1L1 sec	Nucleosome assembly protein 1-like 1 OS=Homo sapiens GN=NAP1L1 PE=1 SV=1
NAP1L1 pT	Nucleosome assembly protein 1-like 1 OS=Homo sapiens GN=NAP1L1 PE=1 SV=1
NAP1L1 pT	Nucleosome assembly protein 1-like 1 OS=Homo sapiens GN=NAP1L1 PE=1 SV=1
WIPI2 pS39	WD repeat domain phosphoinositide-interacting protein 2 OS=Homo sapiens GN=WIPI2 PE=1 SV=1
MSH2 seq:	DNA mismatch repair protein Msh2 OS=Homo sapiens GN=MSH2 PE=1 SV=1
PKM seq: 3	Pyruvate kinase PKM OS=Homo sapiens GN=PKM PE=1 SV=4
PKM pS37	Pyruvate kinase PKM OS=Homo sapiens GN=PKM PE=1 SV=4
HPGD seq:	15-hydroxyprostaglandin dehydrogenase [NAD(+)] OS=Homo sapiens GN=HPGD PE=1 SV=1
PRIM1 seq:	DNA primase small subunit OS=Homo sapiens GN=PRIM1 PE=1 SV=1
HSPA8 seq:	Heat shock cognate 71 kDa protein OS=Homo sapiens GN=HSPA8 PE=1 SV=1
MKI67 pS1	Antigen KI-67 OS=Homo sapiens GN=MKI67 PE=1 SV=2
IRGQ seq: 2	Immunity-related GTPase family Q protein OS=Homo sapiens GN=IRGQ PE=1 SV=1
TRIM28 sec	Transcription intermediary factor 1-beta OS=Homo sapiens GN=TRIM28 PE=1 SV=5
TRIM28 pS	Transcription intermediary factor 1-beta OS=Homo sapiens GN=TRIM28 PE=1 SV=5
AHNAK2 pS	Protein AHNAK2 OS=Homo sapiens GN=AHNAK2 PE=1 SV=2
GANAB seq	Neutral alpha-glucosidase AB OS=Homo sapiens GN=GANAB PE=1 SV=3
MKI67 pS1	Antigen KI-67 OS=Homo sapiens GN=MKI67 PE=1 SV=2
MKI67 pS2	Antigen KI-67 OS=Homo sapiens GN=MKI67 PE=1 SV=2
MKI67 pS1	Antigen KI-67 OS=Homo sapiens GN=MKI67 PE=1 SV=2
DYNC1I2 se	Cytoplasmic dynein 1 intermediate chain 2 OS=Homo sapiens GN=DYNC1I2 PE=1 SV=3
DYNC1I2 se	Cytoplasmic dynein 1 intermediate chain 2 OS=Homo sapiens GN=DYNC1I2 PE=1 SV=3
TKT seq: 17	Transketolase OS=Homo sapiens GN=TKT PE=1 SV=3
WDR26 sec	WD repeat-containing protein 26 OS=Homo sapiens GN=WDR26 PE=1 SV=3
LGALS3 seq	Galectin-3 OS=Homo sapiens GN=LGALS3 PE=1 SV=5
NAP1L4 pS	Nucleosome assembly protein 1-like 4 OS=Homo sapiens GN=NAP1L4 PE=1 SV=1
SPTAN1 pS	Spectrin alpha chain, non-erythrocytic 1 OS=Homo sapiens GN=SPTAN1 PE=1 SV=3
MYH9 seq:	Myosin-9 OS=Homo sapiens GN=MYH9 PE=1 SV=4
QSOX2 seq:	Sulfhydryl oxidase 2 OS=Homo sapiens GN=QSOX2 PE=1 SV=3
HNRNPD se	Heterogeneous nuclear ribonucleoprotein D0 OS=Homo sapiens GN=HNRNPD PE=1 SV=1
AGAP1 pS5	Arf-GAP with GTPase, ANK repeat and PH domain-containing protein 1 OS=Homo sapiens GN=AGAP1 PE=1 SV=4

RUVBL1 se	RuvB-like 1 OS=Homo sapiens GN=RUVBL1 PE=1 SV=1
CWC15 pS1	Spliceosome-associated protein CWC15 homolog OS=Homo sapiens GN=CWC15 PE=1 SV=2
VCP seq: 63	Transitional endoplasmic reticulum ATPase OS=Homo sapiens GN=VCP PE=1 SV=4
PRAME seq	Melanoma antigen preferentially expressed in tumors OS=Homo sapiens GN=PRAME PE=1 SV=1
MKI67 pS1	Antigen KI-67 OS=Homo sapiens GN=MKI67 PE=1 SV=2
UBA1 seq:	Ubiquitin-like modifier-activating enzyme 1 OS=Homo sapiens GN=UBA1 PE=1 SV=3
CCDC6 pS2	Coiled-coil domain-containing protein 6 OS=Homo sapiens GN=CCDC6 PE=1 SV=2
CCDC6 pS2	Coiled-coil domain-containing protein 6 OS=Homo sapiens GN=CCDC6 PE=1 SV=2
CCDC6 pS2	Coiled-coil domain-containing protein 6 OS=Homo sapiens GN=CCDC6 PE=1 SV=2
SLAIN2 pS1	SLAIN motif-containing protein 2 OS=Homo sapiens GN=SLAIN2 PE=1 SV=2
EDC4 pS33	Enhancer of mRNA-decapping protein 4 OS=Homo sapiens GN=EDC4 PE=1 SV=1
NHSL1 pS3	NHS-like protein 1 OS=Homo sapiens GN=NHSL1 PE=1 SV=2
RB1CC1 se	RB1-inducible coiled-coil protein 1 OS=Homo sapiens GN=RB1CC1 PE=1 SV=3
EPS15 pS7	Epidermal growth factor receptor substrate 15 OS=Homo sapiens GN=EPS15 PE=1 SV=2
TNKS1BP1	182 kDa tankyrase-1-binding protein OS=Homo sapiens GN=TNKS1BP1 PE=1 SV=4
TNKS1BP1	182 kDa tankyrase-1-binding protein OS=Homo sapiens GN=TNKS1BP1 PE=1 SV=4
TNKS1BP1	182 kDa tankyrase-1-binding protein OS=Homo sapiens GN=TNKS1BP1 PE=1 SV=4
C2CD2L pS	C2 domain-containing protein 2-like OS=Homo sapiens GN=C2CD2L PE=1 SV=3
CXorf38 pT	Uncharacterized protein CXorf38 OS=Homo sapiens GN=CXorf38 PE=1 SV=1
TRAFD1 pS	TRAF-type zinc finger domain-containing protein 1 OS=Homo sapiens GN=TRAFD1 PE=1 SV=1
TRAFD1 pS	TRAF-type zinc finger domain-containing protein 1 OS=Homo sapiens GN=TRAFD1 PE=1 SV=1
TRAFD1 pS	TRAF-type zinc finger domain-containing protein 1 OS=Homo sapiens GN=TRAFD1 PE=1 SV=1
ESCO1 pS4	N-acetyltransferase ESCO1 OS=Homo sapiens GN=ESCO1 PE=1 SV=3
PDS5B pS1	Sister chromatid cohesion protein PDS5 homolog B OS=Homo sapiens GN=PDS5B PE=1 SV=1
TCOF1 pS1	Treacle protein OS=Homo sapiens GN=TCOF1 PE=1 SV=3
TCOF1 pS1	Treacle protein OS=Homo sapiens GN=TCOF1 PE=1 SV=3
TCOF1 pS1	Treacle protein OS=Homo sapiens GN=TCOF1 PE=1 SV=3
GTF2F1 pS4	General transcription factor IIF subunit 1 OS=Homo sapiens GN=GTF2F1 PE=1 SV=2
UCHL5 seq	Ubiquitin carboxyl-terminal hydrolase isozyme L5 OS=Homo sapiens GN=UCHL5 PE=1 SV=3
NKTR pT11	NK-tumor recognition protein OS=Homo sapiens GN=NKTR PE=1 SV=2
DNAH2 seq	Dynein heavy chain 2, axonemal OS=Homo sapiens GN=DNAH2 PE=2 SV=3
PPP1R13L	RelA-associated inhibitor OS=Homo sapiens GN=PPP1R13L PE=1 SV=4
FLNA seq: 9	Filamin-A OS=Homo sapiens GN=FLNA PE=1 SV=4
LRBA pS12	Lipopolysaccharide-responsive and beige-like anchor protein OS=Homo sapiens GN=LRBA PE=1 SV=4
FLNB seq: 9	Filamin-B OS=Homo sapiens GN=FLNB PE=1 SV=2
FLNB pS98	Filamin-B OS=Homo sapiens GN=FLNB PE=1 SV=2
FLNB pS98	Filamin-B OS=Homo sapiens GN=FLNB PE=1 SV=2
FLNB pS98	Filamin-B OS=Homo sapiens GN=FLNB PE=1 SV=2
CLIP1 seq:	CAP-Gly domain-containing linker protein 1 OS=Homo sapiens GN=CLIP1 PE=1 SV=2
FAM83H p	Protein FAM83H OS=Homo sapiens GN=FAM83H PE=1 SV=3
FAM83H p	Protein FAM83H OS=Homo sapiens GN=FAM83H PE=1 SV=3
PHRF1 pS1	PHD and RING finger domain-containing protein 1 OS=Homo sapiens GN=PHRF1 PE=1 SV=3
PHRF1 pS1	PHD and RING finger domain-containing protein 1 OS=Homo sapiens GN=PHRF1 PE=1 SV=3
LMNA seq:	Prelamin-A/C OS=Homo sapiens GN=LMNA PE=1 SV=1
GOLGA2 se	Golgin subfamily A member 2 OS=Homo sapiens GN=GOLGA2 PE=1 SV=3
TBCA seq: 7	Tubulin-specific chaperone A OS=Homo sapiens GN=TBCA PE=1 SV=3
KRT18 seq:	Keratin, type I cytoskeletal 18 OS=Homo sapiens GN=KRT18 PE=1 SV=2
CLTA seq: 1	Clathrin light chain A OS=Homo sapiens GN=CLTA PE=1 SV=1
TXN seq: 97	Thioredoxin OS=Homo sapiens GN=TXN PE=1 SV=3
DCAF7 seq:	DDB1- and CUL4-associated factor 7 OS=Homo sapiens GN=DCAF7 PE=1 SV=1
RPL36A se	60S ribosomal protein L36a OS=Homo sapiens GN=RPL36A PE=1 SV=2
G6PD seq:	Glucose-6-phosphate 1-dehydrogenase OS=Homo sapiens GN=G6PD PE=1 SV=4
TPD52 pS1	Tumor protein D52 OS=Homo sapiens GN=TPD52 PE=1 SV=2
CAPN2 seq:	Calpain-2 catalytic subunit OS=Homo sapiens GN=CAPN2 PE=1 SV=6
TRIM34 se	Tripartite motif-containing protein 34 OS=Homo sapiens GN=TRIM34 PE=1 SV=2
ERCC6L pS	DNA excision repair protein ERCC-6-like OS=Homo sapiens GN=ERCC6L PE=1 SV=1
ERCC6L seq	DNA excision repair protein ERCC-6-like OS=Homo sapiens GN=ERCC6L PE=1 SV=1
MYH14 pS1	Myosin-14 OS=Homo sapiens GN=MYH14 PE=1 SV=2
MYH14 pS1	Myosin-14 OS=Homo sapiens GN=MYH14 PE=1 SV=2
ABCF3 seq:	ATP-binding cassette sub-family F member 3 OS=Homo sapiens GN=ABCF3 PE=1 SV=2
ABCF2 seq:	ATP-binding cassette sub-family F member 2 OS=Homo sapiens GN=ABCF2 PE=1 SV=2
TTC26 seq:	Intraflagellar transport protein 56 OS=Homo sapiens GN=TTC26 PE=2 SV=1
TRIO seq: 2	Triple functional domain protein OS=Homo sapiens GN=TRIO PE=1 SV=2
CEP95 seq:	Centrosomal protein of 95 kDa OS=Homo sapiens GN=CEP95 PE=1 SV=1
IRF2BP2 pS	Interferon regulatory factor 2-binding protein 2 OS=Homo sapiens GN=IRF2BP2 PE=1 SV=2
IRF2BP2 pS	Interferon regulatory factor 2-binding protein 2 OS=Homo sapiens GN=IRF2BP2 PE=1 SV=2
TJP1 pS968	Tight junction protein ZO-1 OS=Homo sapiens GN=TJP1 PE=1 SV=3
TJP1 pS968	Tight junction protein ZO-1 OS=Homo sapiens GN=TJP1 PE=1 SV=3
FER1L4 seq	Fer-1-like protein 4 OS=Homo sapiens GN=FER1L4 PE=2 SV=1
C9orf16 pS	UPF0184 protein C9orf16 OS=Homo sapiens GN=C9orf16 PE=1 SV=1
PSME1 seq:	Proteasome activator complex subunit 1 OS=Homo sapiens GN=PSME1 PE=1 SV=1
PSMD1 seq	26S proteasome non-ATPase regulatory subunit 1 OS=Homo sapiens GN=PSMD1 PE=1 SV=2
CKAP4 seq:	Cytoskeleton-associated protein 4 OS=Homo sapiens GN=CKAP4 PE=1 SV=2
KRT8 seq: 2	Keratin, type II cytoskeletal 8 OS=Homo sapiens GN=KRT8 PE=1 SV=7
CUL3 seq: 7	Cullin-3 OS=Homo sapiens GN=CUL3 PE=1 SV=2
CLNS1A pS	Methylosome subunit pICln OS=Homo sapiens GN=CLNS1A PE=1 SV=1
DPP3 seq:	Dipeptidyl peptidase 3 OS=Homo sapiens GN=DPP3 PE=1 SV=2
SUCLG2 se	Succinyl-CoA ligase [GDP-forming] subunit beta, mitochondrial OS=Homo sapiens GN=SUCLG2 PE=1 SV=2
EEF2K pS18	Eukaryotic elongation factor 2 kinase OS=Homo sapiens GN=EEF2K PE=1 SV=2
SPTBN2 pS	Spectrin beta chain, non-erythrocytic 2 OS=Homo sapiens GN=SPTBN2 PE=1 SV=3
SPTBN2 pS	Spectrin beta chain, non-erythrocytic 2 OS=Homo sapiens GN=SPTBN2 PE=1 SV=3
SPTBN2 pS	Spectrin beta chain, non-erythrocytic 2 OS=Homo sapiens GN=SPTBN2 PE=1 SV=3
ANKRD26 s	Ankyrin repeat domain-containing protein 26 OS=Homo sapiens GN=ANKRD26 PE=1 SV=3
ZNF335 pS	Zinc finger protein 335 OS=Homo sapiens GN=ZNF335 PE=1 SV=1
CEP83 seq:	Centrosomal protein of 83 kDa OS=Homo sapiens GN=CEP83 PE=1 SV=2
GAPDH seq	Glyceraldehyde-3-phosphate dehydrogenase OS=Homo sapiens GN=GAPDH PE=1 SV=3
INTS12 pS1	Integrator complex subunit 12 OS=Homo sapiens GN=INTS12 PE=1 SV=1
INTS12 pS1	Integrator complex subunit 12 OS=Homo sapiens GN=INTS12 PE=1 SV=1
GIT2 pT595	ARF GTPase-activating protein GIT2 OS=Homo sapiens GN=GIT2 PE=1 SV=2
ATP5A1 se	ATP synthase subunit alpha, mitochondrial OS=Homo sapiens GN=ATP5A1 PE=1 SV=1
TSC22D4 p	TSC22 domain family protein 4 OS=Homo sapiens GN=TSC22D4 PE=1 SV=2
TSC22D4 p	TSC22 domain family protein 4 OS=Homo sapiens GN=TSC22D4 PE=1 SV=2
EPB41L4B s	Band 4.1-like protein 4B OS=Homo sapiens GN=EPB41L4B PE=2 SV=2
UBE2N seq:	Ubiquitin-conjugating enzyme E2 N OS=Homo sapiens GN=UBE2N PE=1 SV=1
TLR8 seq: 6	Toll-like receptor 8 OS=Homo sapiens GN=TLR8 PE=1 SV=1
SLIT1 seq: 3	Slit homolog 1 protein OS=Homo sapiens GN=SLIT1 PE=2 SV=4
MEX3D seq	RNA-binding protein MEX3D OS=Homo sapiens GN=MEX3D PE=1 SV=3
NCL seq: 55	Nucleolin OS=Homo sapiens GN=NCL PE=1 SV=3
NCL pS563	Nucleolin OS=Homo sapiens GN=NCL PE=1 SV=3
NCL pS563	Nucleolin OS=Homo sapiens GN=NCL PE=1 SV=3
GLOD4 seq	Glyoxalase domain-containing protein 4 OS=Homo sapiens GN=GLOD4 PE=1 SV=1
NAV1 pS37	Neuron navigator 1 OS=Homo sapiens GN=NAV1 PE=1 SV=2
LMNB1 pS2	Lamin-B1 OS=Homo sapiens GN=LMNB1 PE=1 SV=2
MGEA5 pS3	Protein O-GlcNAcase OS=Homo sapiens GN=MGEA5 PE=1 SV=2
MGEA5 pS3	Protein O-GlcNAcase OS=Homo sapiens GN=MGEA5 PE=1 SV=2
MTIF2 seq:	Translation initiation factor IF-2, mitochondrial OS=Homo sapiens GN=MTIF2 PE=1 SV=2
YTHDF3 se	YTH domain-containing family protein 3 OS=Homo sapiens GN=YTHDF3 PE=1 SV=1
OCIAD1 pS	OCIA domain-containing protein 1 OS=Homo sapiens GN=OCIAD1 PE=1 SV=1
PARG pS13	Poly(ADP-ribose) glycohydrolase OS=Homo sapiens GN=PARG PE=1 SV=1
CSF3R seq:	Granulocyte colony-stimulating factor receptor OS=Homo sapiens GN=CSF3R PE=1 SV=1
YY1AP1 pS1	YY1-associated protein 1 OS=Homo sapiens GN=YY1AP1 PE=1 SV=2
PLIN3 seq:	Perilipin-3 OS=Homo sapiens GN=PLIN3 PE=1 SV=3
NCOR2 pS1	Nuclear receptor corepressor 2 OS=Homo sapiens GN=NCOR2 PE=1 SV=2
RPS6KA4 p	Ribosomal protein S6 kinase alpha-4 OS=Homo sapiens GN=RPS6KA4 PE=1 SV=1
KRT19 seq:	Keratin, type I cytoskeletal 19 OS=Homo sapiens GN=KRT19 PE=1 SV=4
R3HDM1 s	R3H domain-containing protein 1 OS=Homo sapiens GN=R3HDM1 PE=1 SV=3
R3HDM2 s	R3H domain-containing protein 2 OS=Homo sapiens GN=R3HDM2 PE=1 SV=3
RBL2 seq: 4	Retinoblastoma-like protein 2 OS=Homo sapiens GN=RBL2 PE=1 SV=3
MLLT6 pS3	Protein AF-17 OS=Homo sapiens GN=MLLT6 PE=1 SV=2
NCOA2 pS7	Nuclear receptor coactivator 2 OS=Homo sapiens GN=NCOA2 PE=1 SV=2
PALLD pS9	Palladin OS=Homo sapiens GN=PALLD PE=1 SV=3
PALLD seq:	Palladin OS=Homo sapiens GN=PALLD PE=1 SV=3
RFC1 pT50	Replication factor C subunit 1 OS=Homo sapiens GN=RFC1 PE=1 SV=4
RNH1 seq:	Ribonuclease inhibitor OS=Homo sapiens GN=RNH1 PE=1 SV=2
MAP3K4 p	Mitogen-activated protein kinase kinase kinase 4 OS=Homo sapiens GN=MAP3K4 PE=1 SV=2
MAP3K4 p	Mitogen-activated protein kinase kinase kinase 4 OS=Homo sapiens GN=MAP3K4 PE=1 SV=2
P3H2 seq:	Prolyl 3-hydroxylase 2 OS=Homo sapiens GN=P3H2 PE=1 SV=1
KRT8 seq: 4	Keratin, type II cytoskeletal 8 OS=Homo sapiens GN=KRT8 PE=1 SV=7
KRT8 seq: 4	Keratin, type II cytoskeletal 8 OS=Homo sapiens GN=KRT8 PE=1 SV=7
KRT8 pM40	Keratin, type II cytoskeletal 8 OS=Homo sapiens GN=KRT8 PE=1 SV=7
KRT8 pM40	Keratin, type II cytoskeletal 8 OS=Homo sapiens GN=KRT8 PE=1 SV=7
EIF3A pS58	Eukaryotic translation initiation factor 3 subunit A OS=Homo sapiens GN=EIF3A PE=1 SV=1
ARHGEF5 p	Rho guanine nucleotide exchange factor 5 OS=Homo sapiens GN=ARHGEF5 PE=1 SV=3
ETFA seq: 1	Electron transfer flavoprotein subunit alpha, mitochondrial OS=Homo sapiens GN=ETFA PE=1 SV=1
ETFA pS140	Electron transfer flavoprotein subunit alpha, mitochondrial OS=Homo sapiens GN=ETFA PE=1 SV=1
CAPZB seq:	F-actin-capping protein subunit beta OS=Homo sapiens GN=CAPZB PE=1 SV=4
MYH9 seq:	Myosin-9 OS=Homo sapiens GN=MYH9 PE=1 SV=4
MYH11 seq	Myosin-11 OS=Homo sapiens GN=MYH11 PE=1 SV=3
KANK1 seq:	KN motif and ankyrin repeat domain-containing protein 1 OS=Homo sapiens GN=KANK1 PE=1 SV=3
ACBD3 pS4	Golgi resident protein GCP60 OS=Homo sapiens GN=ACBD3 PE=1 SV=4
RBBP6 pS1	E3 ubiquitin-protein ligase RBBP6 OS=Homo sapiens GN=RBBP6 PE=1 SV=1
RBBP6 pS1	E3 ubiquitin-protein ligase RBBP6 OS=Homo sapiens GN=RBBP6 PE=1 SV=1
CMBL seq:	Carboxymethylenebutenolidase homolog OS=Homo sapiens GN=CMBL PE=1 SV=1
SGK223 pT	Tyrosine-protein kinase SgK223 OS=Homo sapiens GN=SGK223 PE=1 SV=4
SGK223 pT	Tyrosine-protein kinase SgK223 OS=Homo sapiens GN=SGK223 PE=1 SV=4
SGK223 pT	Tyrosine-protein kinase SgK223 OS=Homo sapiens GN=SGK223 PE=1 SV=4
INADL pS64	InaD-like protein OS=Homo sapiens GN=INADL PE=1 SV=3
FASN seq: 9	Fatty acid synthase OS=Homo sapiens GN=FASN PE=1 SV=3
FASN pT97	Fatty acid synthase OS=Homo sapiens GN=FASN PE=1 SV=3
HSPB1 seq:	Heat shock protein beta-1 OS=Homo sapiens GN=HSPB1 PE=1 SV=2
GNAI3 seq:	Guanine nucleotide-binding protein G(k) subunit alpha OS=Homo sapiens GN=GNAI3 PE=1 SV=3
EIF3I seq: 2	Eukaryotic translation initiation factor 3 subunit I OS=Homo sapiens GN=EIF3I PE=1 SV=1
WEE1 pT19	Wee1-like protein kinase OS=Homo sapiens GN=WEE1 PE=1 SV=2
WEE1 pT19	Wee1-like protein kinase OS=Homo sapiens GN=WEE1 PE=1 SV=2
LARP1B pS	La-related protein 1B OS=Homo sapiens GN=LARP1B PE=1 SV=2
TCP11L2 se	T-complex protein 11-like protein 2 OS=Homo sapiens GN=TCP11L2 PE=2 SV=1
HSPA4 seq:	Heat shock 70 kDa protein 4 OS=Homo sapiens GN=HSPA4 PE=1 SV=4
HTATSF1 pS	HIV Tat-specific factor 1 OS=Homo sapiens GN=HTATSF1 PE=1 SV=1
FARSB seq:	Phenylalanine--tRNA ligase beta subunit OS=Homo sapiens GN=FARSB PE=1 SV=3
HSPA9 seq:	Stress-70 protein, mitochondrial OS=Homo sapiens GN=HSPA9 PE=1 SV=2
MPEG1 seq	Macrophage-expressed gene 1 protein OS=Homo sapiens GN=MPEG1 PE=2 SV=1
HDAC2 seq	Histone deacetylase 2 OS=Homo sapiens GN=HDAC2 PE=1 SV=2
THOC3 seq:	THO complex subunit 3 OS=Homo sapiens GN=THOC3 PE=1 SV=1
RBMX seq:	RNA-binding motif protein, X chromosome OS=Homo sapiens GN=RBMX PE=1 SV=3
HNRNPA0 s	Heterogeneous nuclear ribonucleoprotein A0 OS=Homo sapiens GN=HNRNPA0 PE=1 SV=1
U2AF2 seq:	Splicing factor U2AF 65 kDa subunit OS=Homo sapiens GN=U2AF2 PE=1 SV=4
HNRNPA3 s	Heterogeneous nuclear ribonucleoprotein A3 OS=Homo sapiens GN=HNRNPA3 PE=1 SV=2
HNRNPA3 p	Heterogeneous nuclear ribonucleoprotein A3 OS=Homo sapiens GN=HNRNPA3 PE=1 SV=2
HNRNPA1L	Heterogeneous nuclear ribonucleoprotein A1-like 2 OS=Homo sapiens GN=HNRNPA1L2 PE=2 SV=2
HNRNPA1L	Heterogeneous nuclear ribonucleoprotein A1-like 2 OS=Homo sapiens GN=HNRNPA1L2 PE=2 SV=2
HNRNPA2B	Heterogeneous nuclear ribonucleoproteins A2/B1 OS=Homo sapiens GN=HNRNPA2B1 PE=1 SV=2
HNRNPA2B	Heterogeneous nuclear ribonucleoproteins A2/B1 OS=Homo sapiens GN=HNRNPA2B1 PE=1 SV=2
RB1 pT356	Retinoblastoma-associated protein OS=Homo sapiens GN=RB1 PE=1 SV=2
EIF3H seq:	Eukaryotic translation initiation factor 3 subunit H OS=Homo sapiens GN=EIF3H PE=1 SV=1
EIF3H pM3	Eukaryotic translation initiation factor 3 subunit H OS=Homo sapiens GN=EIF3H PE=1 SV=1
PSMA3 seq	Proteasome subunit alpha type-3 OS=Homo sapiens GN=PSMA3 PE=1 SV=2
ZCCHC8 pS	Zinc finger CCHC domain-containing protein 8 OS=Homo sapiens GN=ZCCHC8 PE=1 SV=2
ILF3 seq: 58	Interleukin enhancer-binding factor 3 OS=Homo sapiens GN=ILF3 PE=1 SV=3
ILF3 pT592	Interleukin enhancer-binding factor 3 OS=Homo sapiens GN=ILF3 PE=1 SV=3
ARAP1 pS2	Arf-GAP with Rho-GAP domain, ANK repeat and PH domain-containing protein 1 OS=Homo sapiens GN=ARAP1 PE=1 SV=3
ARAP1 pS2	Arf-GAP with Rho-GAP domain, ANK repeat and PH domain-containing protein 1 OS=Homo sapiens GN=ARAP1 PE=1 SV=3
ARAP1 seq:	Arf-GAP with Rho-GAP domain, ANK repeat and PH domain-containing protein 1 OS=Homo sapiens GN=ARAP1 PE=1 SV=3
ARAP1 seq:	Arf-GAP with Rho-GAP domain, ANK repeat and PH domain-containing protein 1 OS=Homo sapiens GN=ARAP1 PE=1 SV=3
DNMBP se	Dynamin-binding protein OS=Homo sapiens GN=DNMBP PE=1 SV=1
HNRNPK se	Heterogeneous nuclear ribonucleoprotein K OS=Homo sapiens GN=HNRNPK PE=1 SV=1
HNRNPK se	Heterogeneous nuclear ribonucleoprotein K OS=Homo sapiens GN=HNRNPK PE=1 SV=1
COX7A2 se	Cytochrome c oxidase subunit 7A2, mitochondrial OS=Homo sapiens GN=COX7A2 PE=1 SV=1
PTTG2 pS1	Securin-2 OS=Homo sapiens GN=PTTG2 PE=2 SV=2
PSMA5 seq	Proteasome subunit alpha type-5 OS=Homo sapiens GN=PSMA5 PE=1 SV=3
YRDC pS37	YrdC domain-containing protein, mitochondrial OS=Homo sapiens GN=YRDC PE=1 SV=1
YRDC pS37	YrdC domain-containing protein, mitochondrial OS=Homo sapiens GN=YRDC PE=1 SV=1
DDI2 pS19	Protein DDI1 homolog 2 OS=Homo sapiens GN=DDI2 PE=1 SV=1
CREB1 pS1	Cyclic AMP-responsive element-binding protein 1 OS=Homo sapiens GN=CREB1 PE=1 SV=2
RAD21 seq:	Double-strand-break repair protein rad21 homolog OS=Homo sapiens GN=RAD21 PE=1 SV=2
CRTC3 pS3	CREB-regulated transcription coactivator 3 OS=Homo sapiens GN=CRTC3 PE=1 SV=2
CRTC3 pS3	CREB-regulated transcription coactivator 3 OS=Homo sapiens GN=CRTC3 PE=1 SV=2
CRTC3 pS3	CREB-regulated transcription coactivator 3 OS=Homo sapiens GN=CRTC3 PE=1 SV=2
MLLT4 pS1	Afadin OS=Homo sapiens GN=MLLT4 PE=1 SV=3
MLLT4 pS1	Afadin OS=Homo sapiens GN=MLLT4 PE=1 SV=3
MLLT4 pS1	Afadin OS=Homo sapiens GN=MLLT4 PE=1 SV=3
MBP pS96	Myelin basic protein OS=Homo sapiens GN=MBP PE=1 SV=3
EPB41 pS8	Protein 4.1 OS=Homo sapiens GN=EPB41 PE=1 SV=4
MAPK8IP3	C-Jun-amino-terminal kinase-interacting protein 3 OS=Homo sapiens GN=MAPK8IP3 PE=1 SV=3
CIRBP seq:	Cold-inducible RNA-binding protein OS=Homo sapiens GN=CIRBP PE=1 SV=1
CIRBP seq:	Cold-inducible RNA-binding protein OS=Homo sapiens GN=CIRBP PE=1 SV=1
SFPQ seq: 2	Splicing factor, proline- and glutamine-rich OS=Homo sapiens GN=SFPQ PE=1 SV=2
HNRNPR se	Heterogeneous nuclear ribonucleoprotein R OS=Homo sapiens GN=HNRNPR PE=1 SV=1
HNRNPR p	Heterogeneous nuclear ribonucleoprotein R OS=Homo sapiens GN=HNRNPR PE=1 SV=1
USP47 pS8	Ubiquitin carboxyl-terminal hydrolase 47 OS=Homo sapiens GN=USP47 PE=1 SV=3
DPP3 seq:	Dipeptidyl peptidase 3 OS=Homo sapiens GN=DPP3 PE=1 SV=2
MAP7 pT27	Ensconsin OS=Homo sapiens GN=MAP7 PE=1 SV=1
EPS8L1 seq	Epidermal growth factor receptor kinase substrate 8-like protein 1 OS=Homo sapiens GN=EPS8L1 PE=1 SV=3
TCOF1 pS1	Treacle protein OS=Homo sapiens GN=TCOF1 PE=1 SV=3
TCOF1 pS1	Treacle protein OS=Homo sapiens GN=TCOF1 PE=1 SV=3
TCOF1 pS1	Treacle protein OS=Homo sapiens GN=TCOF1 PE=1 SV=3
STUB1 pS1	E3 ubiquitin-protein ligase CHIP OS=Homo sapiens GN=STUB1 PE=1 SV=2
STUB1 pS1	E3 ubiquitin-protein ligase CHIP OS=Homo sapiens GN=STUB1 PE=1 SV=2
STUB1 pS1	E3 ubiquitin-protein ligase CHIP OS=Homo sapiens GN=STUB1 PE=1 SV=2
STUB1 pS1	E3 ubiquitin-protein ligase CHIP OS=Homo sapiens GN=STUB1 PE=1 SV=2
STUB1 pS1	E3 ubiquitin-protein ligase CHIP OS=Homo sapiens GN=STUB1 PE=1 SV=2
STUB1 pS1	E3 ubiquitin-protein ligase CHIP OS=Homo sapiens GN=STUB1 PE=1 SV=2
STUB1 pS1	E3 ubiquitin-protein ligase CHIP OS=Homo sapiens GN=STUB1 PE=1 SV=2
KIAA1468 p	LisH domain and HEAT repeat-containing protein KIAA1468 OS=Homo sapiens GN=KIAA1468 PE=1 SV=2
KIAA1468 p	LisH domain and HEAT repeat-containing protein KIAA1468 OS=Homo sapiens GN=KIAA1468 PE=1 SV=2
KIAA1468 p	LisH domain and HEAT repeat-containing protein KIAA1468 OS=Homo sapiens GN=KIAA1468 PE=1 SV=2
TCF12 pS98	Transcription factor 12 OS=Homo sapiens GN=TCF12 PE=1 SV=1
SIK3 pM34	Serine/threonine-protein kinase SIK3 OS=Homo sapiens GN=SIK3 PE=1 SV=3
FARP1 pT2	FERM, RhoGEF and pleckstrin domain-containing protein 1 OS=Homo sapiens GN=FARP1 PE=1 SV=1
FARP2 pT2	FERM, RhoGEF and pleckstrin domain-containing protein 2 OS=Homo sapiens GN=FARP2 PE=1 SV=3
RBM25 pS	RNA-binding protein 25 OS=Homo sapiens GN=RBM25 PE=1 SV=3
RBM25 pS	RNA-binding protein 25 OS=Homo sapiens GN=RBM25 PE=1 SV=3
RBM25 pS	RNA-binding protein 25 OS=Homo sapiens GN=RBM25 PE=1 SV=3
C19orf47 p	Uncharacterized protein C19orf47 OS=Homo sapiens GN=C19orf47 PE=1 SV=1
C19orf47 p	Uncharacterized protein C19orf47 OS=Homo sapiens GN=C19orf47 PE=1 SV=1
ITPK1 pS39	Inositol-tetrakisphosphate 1-kinase OS=Homo sapiens GN=ITPK1 PE=1 SV=2
NUDT4 pS1	Diphosphoinositol polyphosphate phosphohydrolase 2 OS=Homo sapiens GN=NUDT4 PE=1 SV=2
CEP170B p	Centrosomal protein of 170 kDa protein B OS=Homo sapiens GN=CEP170B PE=1 SV=4
FGD3 seq:	FYVE, RhoGEF and PH domain-containing protein 3 OS=Homo sapiens GN=FGD3 PE=1 SV=1
VIM seq: 14	Vimentin OS=Homo sapiens GN=VIM PE=1 SV=4
LPIN1 pS10	Phosphatidate phosphatase LPIN1 OS=Homo sapiens GN=LPIN1 PE=1 SV=2
LPIN1 pS10	Phosphatidate phosphatase LPIN1 OS=Homo sapiens GN=LPIN1 PE=1 SV=2
CNKSR2 pS	Connector enhancer of kinase suppressor of ras 2 OS=Homo sapiens GN=CNKSR2 PE=1 SV=1
SEPT9 pS22	Septin-9 OS=Homo sapiens GN=SEPT9 PE=1 SV=2
SEPT9 pS22	Septin-9 OS=Homo sapiens GN=SEPT9 PE=1 SV=2
SEPT9 pS22	Septin-9 OS=Homo sapiens GN=SEPT9 PE=1 SV=2
EIF3A pS88	Eukaryotic translation initiation factor 3 subunit A OS=Homo sapiens GN=EIF3A PE=1 SV=1
MRPL17 se	39S ribosomal protein L17, mitochondrial OS=Homo sapiens GN=MRPL17 PE=1 SV=1
VCP seq: 96	Transitional endoplasmic reticulum ATPase OS=Homo sapiens GN=VCP PE=1 SV=4
MAP1B pS1	Microtubule-associated protein 1B OS=Homo sapiens GN=MAP1B PE=1 SV=2
MAP1B pS1	Microtubule-associated protein 1B OS=Homo sapiens GN=MAP1B PE=1 SV=2
PGK2 seq:	Phosphoglycerate kinase 2 OS=Homo sapiens GN=PGK2 PE=1 SV=3
CEP170 pS	Centrosomal protein of 170 kDa OS=Homo sapiens GN=CEP170 PE=1 SV=1
CEP170 pS	Centrosomal protein of 170 kDa OS=Homo sapiens GN=CEP170 PE=1 SV=1
EFTUD2 se	116 kDa U5 small nuclear ribonucleoprotein component OS=Homo sapiens GN=EFTUD2 PE=1 SV=1
CDK18 pT1	Cyclin-dependent kinase 18 OS=Homo sapiens GN=CDK18 PE=1 SV=3
CDK18 pT1	Cyclin-dependent kinase 18 OS=Homo sapiens GN=CDK18 PE=1 SV=3
CDK18 seq:	Cyclin-dependent kinase 18 OS=Homo sapiens GN=CDK18 PE=1 SV=3
KIF12 seq:	Kinesin-like protein KIF12 OS=Homo sapiens GN=KIF12 PE=1 SV=3
GPATCH2L	G patch domain-containing protein 2-like OS=Homo sapiens GN=GPATCH2L PE=1 SV=3
HMGB3 se	High mobility group protein B3 OS=Homo sapiens GN=HMGB3 PE=1 SV=4
HMGB2 se	High mobility group protein B2 OS=Homo sapiens GN=HMGB2 PE=1 SV=2
EAF2 pS22	ELL-associated factor 2 OS=Homo sapiens GN=EAF2 PE=1 SV=1
RPS12 seq:	40S ribosomal protein S12 OS=Homo sapiens GN=RPS12 PE=1 SV=3
CCT5 seq: 3	T-complex protein 1 subunit epsilon OS=Homo sapiens GN=CCT5 PE=1 SV=1
CAPNS2 se	Calpain small subunit 2 OS=Homo sapiens GN=CAPNS2 PE=2 SV=2
COIL pT303	Coilin OS=Homo sapiens GN=COIL PE=1 SV=1
MYO9B pS1	Unconventional myosin-IXb OS=Homo sapiens GN=MYO9B PE=1 SV=3
AHNAK2 pS	Protein AHNAK2 OS=Homo sapiens GN=AHNAK2 PE=1 SV=2
ITPR3 pS26	Inositol 1,4,5-trisphosphate receptor type 3 OS=Homo sapiens GN=ITPR3 PE=1 SV=2
Uncharacte	Uncharacterized protein FLJ45252 OS=Homo sapiens PE=2 SV=2
Uncharacte	Uncharacterized protein FLJ45252 OS=Homo sapiens PE=2 SV=2
NCOR2 pS2	Nuclear receptor corepressor 2 OS=Homo sapiens GN=NCOR2 PE=1 SV=2
ETFA seq: 4	Electron transfer flavoprotein subunit alpha, mitochondrial OS=Homo sapiens GN=ETFA PE=1 SV=1
MCCC2 seq	Methylcrotonoyl-CoA carboxylase beta chain, mitochondrial OS=Homo sapiens GN=MCCC2 PE=1 SV=1
SNRNP70 s	U1 small nuclear ribonucleoprotein 70 kDa OS=Homo sapiens GN=SNRNP70 PE=1 SV=2
DHX9 seq:	ATP-dependent RNA helicase A OS=Homo sapiens GN=DHX9 PE=1 SV=4
PARD3B pS	Partitioning defective 3 homolog B OS=Homo sapiens GN=PARD3B PE=1 SV=2
KIAA1217 p	Sickle tail protein homolog OS=Homo sapiens GN=KIAA1217 PE=1 SV=2
CCDC86 pS	Coiled-coil domain-containing protein 86 OS=Homo sapiens GN=CCDC86 PE=1 SV=1
CCDC86 pS	Coiled-coil domain-containing protein 86 OS=Homo sapiens GN=CCDC86 PE=1 SV=1
CCDC86 pS	Coiled-coil domain-containing protein 86 OS=Homo sapiens GN=CCDC86 PE=1 SV=1
U2AF2 seq:	Splicing factor U2AF 65 kDa subunit OS=Homo sapiens GN=U2AF2 PE=1 SV=4
U2AF2 seq:	Splicing factor U2AF 65 kDa subunit OS=Homo sapiens GN=U2AF2 PE=1 SV=4
INO80B pS	INO80 complex subunit B OS=Homo sapiens GN=INO80B PE=1 SV=2
AAK1 pS73	AP2-associated protein kinase 1 OS=Homo sapiens GN=AAK1 PE=1 SV=3
CFL1 seq: 1	Cofilin-1 OS=Homo sapiens GN=CFL1 PE=1 SV=3
CFL1 pS156	Cofilin-1 OS=Homo sapiens GN=CFL1 PE=1 SV=3
CCT2 seq: 2	T-complex protein 1 subunit beta OS=Homo sapiens GN=CCT2 PE=1 SV=4
STRN4 pS2	Striatin-4 OS=Homo sapiens GN=STRN4 PE=1 SV=2
STRN4 pS2	Striatin-4 OS=Homo sapiens GN=STRN4 PE=1 SV=2
STRN4 pS2	Striatin-4 OS=Homo sapiens GN=STRN4 PE=1 SV=2
STRN4 pS2	Striatin-4 OS=Homo sapiens GN=STRN4 PE=1 SV=2
PARVB pT3	Beta-parvin OS=Homo sapiens GN=PARVB PE=1 SV=1
ZYX pS308	Zyxin OS=Homo sapiens GN=ZYX PE=1 SV=1
EIF3E pS39	Eukaryotic translation initiation factor 3 subunit E OS=Homo sapiens GN=EIF3E PE=1 SV=1
VRK2 seq: 1	Serine/threonine-protein kinase VRK2 OS=Homo sapiens GN=VRK2 PE=1 SV=3
HSP90AA1	Heat shock protein HSP 90-alpha OS=Homo sapiens GN=HSP90AA1 PE=1 SV=5
HSP90AA1	Heat shock protein HSP 90-alpha OS=Homo sapiens GN=HSP90AA1 PE=1 SV=5
HSP90AB1	Heat shock protein HSP 90-beta OS=Homo sapiens GN=HSP90AB1 PE=1 SV=4
TPD52 seq:	Tumor protein D52 OS=Homo sapiens GN=TPD52 PE=1 SV=2
PICK1 seq:	PRKCA-binding protein OS=Homo sapiens GN=PICK1 PE=1 SV=2
PNPLA2 se	Patatin-like phospholipase domain-containing protein 2 OS=Homo sapiens GN=PNPLA2 PE=1 SV=1
DOK5 seq:	Docking protein 5 OS=Homo sapiens GN=DOK5 PE=1 SV=2
PRPSAP1 p	Phosphoribosyl pyrophosphate synthase-associated protein 1 OS=Homo sapiens GN=PRPSAP1 PE=1 SV=2
PRPSAP1 p	Phosphoribosyl pyrophosphate synthase-associated protein 1 OS=Homo sapiens GN=PRPSAP1 PE=1 SV=2
INTS5 seq:	Integrator complex subunit 5 OS=Homo sapiens GN=INTS5 PE=1 SV=1
HSP90AA1	Heat shock protein HSP 90-alpha OS=Homo sapiens GN=HSP90AA1 PE=1 SV=5
HSP90AA1	Heat shock protein HSP 90-alpha OS=Homo sapiens GN=HSP90AA1 PE=1 SV=5
HSP90AA1	Heat shock protein HSP 90-alpha OS=Homo sapiens GN=HSP90AA1 PE=1 SV=5
HSP90AB1	Heat shock protein HSP 90-beta OS=Homo sapiens GN=HSP90AB1 PE=1 SV=4
HSP90AB1	Heat shock protein HSP 90-beta OS=Homo sapiens GN=HSP90AB1 PE=1 SV=4
SBF1 pS114	Myotubularin-related protein 5 OS=Homo sapiens GN=SBF1 PE=1 SV=3
BAZ1A pT1	Bromodomain adjacent to zinc finger domain protein 1A OS=Homo sapiens GN=BAZ1A PE=1 SV=2
EPPK1 seq:	Epiplakin OS=Homo sapiens GN=EPPK1 PE=1 SV=2
TRIL pS259	TLR4 interactor with leucine rich repeats OS=Homo sapiens GN=TRIL PE=1 SV=2
ELL pS444	RNA polymerase II elongation factor ELL OS=Homo sapiens GN=ELL PE=1 SV=1
ELL pS444	RNA polymerase II elongation factor ELL OS=Homo sapiens GN=ELL PE=1 SV=1
PUF60 seq:	Poly(U)-binding-splicing factor PUF60 OS=Homo sapiens GN=PUF60 PE=1 SV=1
SLC19A1 se	Folate transporter 1 OS=Homo sapiens GN=SLC19A1 PE=1 SV=3
GREB1L seq	GREB1-like protein OS=Homo sapiens GN=GREB1L PE=2 SV=2
MROH6 se	Maestro heat-like repeat-containing protein family member 6 OS=Homo sapiens GN=MROH6 PE=4 SV=2
CALB2 pM1	Calretinin OS=Homo sapiens GN=CALB2 PE=2 SV=2
LAMTOR3 s	Ragulator complex protein LAMTOR3 OS=Homo sapiens GN=LAMTOR3 PE=1 SV=1
TPD52L2 se	Tumor protein D54 OS=Homo sapiens GN=TPD52L2 PE=1 SV=2
WDR73 seq	WD repeat-containing protein 73 OS=Homo sapiens GN=WDR73 PE=1 SV=1
FASN pM20	Fatty acid synthase OS=Homo sapiens GN=FASN PE=1 SV=3
TPD52L1 se	Tumor protein D53 OS=Homo sapiens GN=TPD52L1 PE=1 SV=1
RPS17 seq:	40S ribosomal protein S17 OS=Homo sapiens GN=RPS17 PE=1 SV=2
HSD17B10	3-hydroxyacyl-CoA dehydrogenase type-2 OS=Homo sapiens GN=HSD17B10 PE=1 SV=3
HSD17B10	3-hydroxyacyl-CoA dehydrogenase type-2 OS=Homo sapiens GN=HSD17B10 PE=1 SV=3
SH2D5 pS2	SH2 domain-containing protein 5 OS=Homo sapiens GN=SH2D5 PE=1 SV=2
CORO7 pS9	Coronin-7 OS=Homo sapiens GN=CORO7 PE=1 SV=2
SGTA seq: 1	Small glutamine-rich tetratricopeptide repeat-containing protein alpha OS=Homo sapiens GN=SGTA PE=1 SV=1
ZDHHC8 pS	Probable palmitoyltransferase ZDHHC8 OS=Homo sapiens GN=ZDHHC8 PE=1 SV=3
ZFP36 pS93	Tristetraprolin OS=Homo sapiens GN=ZFP36 PE=1 SV=1
PKP3 pS19	Plakophilin-3 OS=Homo sapiens GN=PKP3 PE=1 SV=1
PKP3 seq: 1	Plakophilin-3 OS=Homo sapiens GN=PKP3 PE=1 SV=1
PSAP seq: 2	Prosaposin OS=Homo sapiens GN=PSAP PE=1 SV=2
DDX54 pS7	ATP-dependent RNA helicase DDX54 OS=Homo sapiens GN=DDX54 PE=1 SV=2
PRRC2C pS	Protein PRRC2C OS=Homo sapiens GN=PRRC2C PE=1 SV=4
ETFB seq: 9	Electron transfer flavoprotein subunit beta OS=Homo sapiens GN=ETFB PE=1 SV=3
CASKIN2 pS	Caskin-2 OS=Homo sapiens GN=CASKIN2 PE=1 SV=2
HDLBP seq:	Vigilin OS=Homo sapiens GN=HDLBP PE=1 SV=2
MYH14 seq	Myosin-14 OS=Homo sapiens GN=MYH14 PE=1 SV=2
MYH14 seq	Myosin-14 OS=Homo sapiens GN=MYH14 PE=1 SV=2
AGFG2 pS4	Arf-GAP domain and FG repeat-containing protein 2 OS=Homo sapiens GN=AGFG2 PE=1 SV=2
TKT seq: 18	Transketolase OS=Homo sapiens GN=TKT PE=1 SV=3
KMT2B seq	Histone-lysine N-methyltransferase 2B OS=Homo sapiens GN=KMT2B PE=1 SV=1
SLC9A3R1 s	Na(+)/H(+) exchange regulatory cofactor NHE-RF1 OS=Homo sapiens GN=SLC9A3R1 PE=1 SV=4
TRIM16 pS	Tripartite motif-containing protein 16 OS=Homo sapiens GN=TRIM16 PE=1 SV=3
PRIMPOL p	DNA-directed primase/polymerase protein OS=Homo sapiens GN=PRIMPOL PE=1 SV=2
COL19A1 p	Collagen alpha-1(XIX) chain OS=Homo sapiens GN=COL19A1 PE=1 SV=3
C15orf52 p	Uncharacterized protein C15orf52 OS=Homo sapiens GN=C15orf52 PE=1 SV=1
C15orf52 p	Uncharacterized protein C15orf52 OS=Homo sapiens GN=C15orf52 PE=1 SV=1
MYRIP pS5	Rab effector MyRIP OS=Homo sapiens GN=MYRIP PE=1 SV=2
MYRIP pS5	Rab effector MyRIP OS=Homo sapiens GN=MYRIP PE=1 SV=2
FLNC pS22	Filamin-C OS=Homo sapiens GN=FLNC PE=1 SV=3
TNRC6A pS	Trinucleotide repeat-containing gene 6A protein OS=Homo sapiens GN=TNRC6A PE=1 SV=2
PLIN5 pS11	Perilipin-5 OS=Homo sapiens GN=PLIN5 PE=1 SV=2
CEP170 pS	Centrosomal protein of 170 kDa OS=Homo sapiens GN=CEP170 PE=1 SV=1
CEP170 pS	Centrosomal protein of 170 kDa OS=Homo sapiens GN=CEP170 PE=1 SV=1
CEP170 pS	Centrosomal protein of 170 kDa OS=Homo sapiens GN=CEP170 PE=1 SV=1
PLIN3 seq:	Perilipin-3 OS=Homo sapiens GN=PLIN3 PE=1 SV=3
SSH1 pS98	Protein phosphatase Slingshot homolog 1 OS=Homo sapiens GN=SSH1 PE=1 SV=2
EIF3C seq: 8	Eukaryotic translation initiation factor 3 subunit C OS=Homo sapiens GN=EIF3C PE=1 SV=1
NUMA1 pS	Nuclear mitotic apparatus protein 1 OS=Homo sapiens GN=NUMA1 PE=1 SV=2
ZC3H18 pS	Zinc finger CCCH domain-containing protein 18 OS=Homo sapiens GN=ZC3H18 PE=1 SV=2
MICAL3 pS	Protein-methionine sulfoxide oxidase MICAL3 OS=Homo sapiens GN=MICAL3 PE=1 SV=2
MICAL3 pS	Protein-methionine sulfoxide oxidase MICAL3 OS=Homo sapiens GN=MICAL3 PE=1 SV=2
ZNF318 pS	Zinc finger protein 318 OS=Homo sapiens GN=ZNF318 PE=1 SV=2
TJP2 pS325	Tight junction protein ZO-2 OS=Homo sapiens GN=TJP2 PE=1 SV=2
TXNDC9 pS	Thioredoxin domain-containing protein 9 OS=Homo sapiens GN=TXNDC9 PE=1 SV=2
HUWE1 pS	E3 ubiquitin-protein ligase HUWE1 OS=Homo sapiens GN=HUWE1 PE=1 SV=3
TGFBR2 pS	TGF-beta receptor type-2 OS=Homo sapiens GN=TGFBR2 PE=1 SV=2
DLG5 pS12	Disks large homolog 5 OS=Homo sapiens GN=DLG5 PE=1 SV=4
NSFL1C seq	NSFL1 cofactor p47 OS=Homo sapiens GN=NSFL1C PE=1 SV=2
NSFL1C pS2	NSFL1 cofactor p47 OS=Homo sapiens GN=NSFL1C PE=1 SV=2
NSFL1C pS2	NSFL1 cofactor p47 OS=Homo sapiens GN=NSFL1C PE=1 SV=2
HNRNPM s	Heterogeneous nuclear ribonucleoprotein M OS=Homo sapiens GN=HNRNPM PE=1 SV=3
TBC1D4 pS	TBC1 domain family member 4 OS=Homo sapiens GN=TBC1D4 PE=1 SV=2
NOP2 pS58	Probable 28S rRNA (cytosine(4447)-C(5))-methyltransferase OS=Homo sapiens GN=NOP2 PE=1 SV=2
CHAMP1 p	Chromosome alignment-maintaining phosphoprotein 1 OS=Homo sapiens GN=CHAMP1 PE=1 SV=2
CHAMP1 p	Chromosome alignment-maintaining phosphoprotein 1 OS=Homo sapiens GN=CHAMP1 PE=1 SV=2
PANK2 pS1	Pantothenate kinase 2, mitochondrial OS=Homo sapiens GN=PANK2 PE=1 SV=3
ROBO4 pS1	Roundabout homolog 4 OS=Homo sapiens GN=ROBO4 PE=1 SV=1
P2RY12 pT	P2Y purinoceptor 12 OS=Homo sapiens GN=P2RY12 PE=1 SV=1
GRB14 pS4	Growth factor receptor-bound protein 14 OS=Homo sapiens GN=GRB14 PE=1 SV=2
SND1 seq:	Staphylococcal nuclease domain-containing protein 1 OS=Homo sapiens GN=SND1 PE=1 SV=1
SND1 pS78	Staphylococcal nuclease domain-containing protein 1 OS=Homo sapiens GN=SND1 PE=1 SV=1
NFATC2IP p	NFATC2-interacting protein OS=Homo sapiens GN=NFATC2IP PE=1 SV=1
SEMA3G se	Semaphorin-3G OS=Homo sapiens GN=SEMA3G PE=2 SV=1
HSP90B2P	Putative endoplasmin-like protein OS=Homo sapiens GN=HSP90B2P PE=5 SV=1
HSP90B2P	Putative endoplasmin-like protein OS=Homo sapiens GN=HSP90B2P PE=5 SV=1
FDXR seq: 3	NADPH:adrenodoxin oxidoreductase, mitochondrial OS=Homo sapiens GN=FDXR PE=1 SV=3
ZC3H18 pS	Zinc finger CCCH domain-containing protein 18 OS=Homo sapiens GN=ZC3H18 PE=1 SV=2
ZC3H18 pS	Zinc finger CCCH domain-containing protein 18 OS=Homo sapiens GN=ZC3H18 PE=1 SV=2
MDH1 seq:	Malate dehydrogenase, cytoplasmic OS=Homo sapiens GN=MDH1 PE=1 SV=4
ITIH4 seq: 1	Inter-alpha-trypsin inhibitor heavy chain H4 OS=Homo sapiens GN=ITIH4 PE=1 SV=4
RBBP6 pS5	E3 ubiquitin-protein ligase RBBP6 OS=Homo sapiens GN=RBBP6 PE=1 SV=1
PAFAH1B3	Platelet-activating factor acetylhydrolase IB subunit gamma OS=Homo sapiens GN=PAFAH1B3 PE=1 SV=1
ATRX pS34	Transcriptional regulator ATRX OS=Homo sapiens GN=ATRX PE=1 SV=5
ATRX pS34	Transcriptional regulator ATRX OS=Homo sapiens GN=ATRX PE=1 SV=5
RANBP2 pS	E3 SUMO-protein ligase RanBP2 OS=Homo sapiens GN=RANBP2 PE=1 SV=2
NUCB2 seq	Nucleobindin-2 OS=Homo sapiens GN=NUCB2 PE=1 SV=2
TUBB seq: 2	Tubulin beta chain OS=Homo sapiens GN=TUBB PE=1 SV=2
GTPBP1 pS	GTP-binding protein 1 OS=Homo sapiens GN=GTPBP1 PE=1 SV=3
OSBPL7 pS	Oxysterol-binding protein-related protein 7 OS=Homo sapiens GN=OSBPL7 PE=2 SV=1
OSBPL7 pS	Oxysterol-binding protein-related protein 7 OS=Homo sapiens GN=OSBPL7 PE=2 SV=1
OXSR1 pS3	Serine/threonine-protein kinase OSR1 OS=Homo sapiens GN=OXSR1 PE=1 SV=1
OXSR1 pS3	Serine/threonine-protein kinase OSR1 OS=Homo sapiens GN=OXSR1 PE=1 SV=1
OXSR1 pS3	Serine/threonine-protein kinase OSR1 OS=Homo sapiens GN=OXSR1 PE=1 SV=1
CDC42EP2	Cdc42 effector protein 2 OS=Homo sapiens GN=CDC42EP2 PE=1 SV=1
FASN seq: 8	Fatty acid synthase OS=Homo sapiens GN=FASN PE=1 SV=3
MAST2 pS1	Microtubule-associated serine/threonine-protein kinase 2 OS=Homo sapiens GN=MAST2 PE=1 SV=2
PCBP2 pM	Poly(rC)-binding protein 2 OS=Homo sapiens GN=PCBP2 PE=1 SV=1
PCBP2 pS2	Poly(rC)-binding protein 2 OS=Homo sapiens GN=PCBP2 PE=1 SV=1
PCBP2 pS2	Poly(rC)-binding protein 2 OS=Homo sapiens GN=PCBP2 PE=1 SV=1
SPAG9 pS1	C-Jun-amino-terminal kinase-interacting protein 4 OS=Homo sapiens GN=SPAG9 PE=1 SV=4
ARHGEF11	Rho guanine nucleotide exchange factor 11 OS=Homo sapiens GN=ARHGEF11 PE=1 SV=1
RHBDD1 p	Rhomboid-related protein 4 OS=Homo sapiens GN=RHBDD1 PE=1 SV=1
UBE2O pS4	(E3-independent) E2 ubiquitin-conjugating enzyme OS=Homo sapiens GN=UBE2O PE=1 SV=3
ULK1 pS55	Serine/threonine-protein kinase ULK1 OS=Homo sapiens GN=ULK1 PE=1 SV=2
CEP250 pS	Centrosome-associated protein CEP250 OS=Homo sapiens GN=CEP250 PE=1 SV=2
OSBPL3 pS	Oxysterol-binding protein-related protein 3 OS=Homo sapiens GN=OSBPL3 PE=1 SV=1
OSBPL3 pS	Oxysterol-binding protein-related protein 3 OS=Homo sapiens GN=OSBPL3 PE=1 SV=1
CDR2L pS1	Cerebellar degeneration-related protein 2-like OS=Homo sapiens GN=CDR2L PE=1 SV=2
CDR2L pS1	Cerebellar degeneration-related protein 2-like OS=Homo sapiens GN=CDR2L PE=1 SV=2
RBM15 pS1	Putative RNA-binding protein 15 OS=Homo sapiens GN=RBM15 PE=1 SV=2
GGA3 seq:	ADP-ribosylation factor-binding protein GGA3 OS=Homo sapiens GN=GGA3 PE=1 SV=1
GGA3 seq:	ADP-ribosylation factor-binding protein GGA3 OS=Homo sapiens GN=GGA3 PE=1 SV=1
RBM4 pS86	RNA-binding protein 4 OS=Homo sapiens GN=RBM4 PE=1 SV=1
RBM4 pS86	RNA-binding protein 4 OS=Homo sapiens GN=RBM4 PE=1 SV=1
RBM4B pS8	RNA-binding protein 4B OS=Homo sapiens GN=RBM4B PE=1 SV=1
RBM4B pS8	RNA-binding protein 4B OS=Homo sapiens GN=RBM4B PE=1 SV=1
LIN9 pS309	Protein lin-9 homolog OS=Homo sapiens GN=LIN9 PE=1 SV=1
FLNB seq: 7	Filamin-B OS=Homo sapiens GN=FLNB PE=1 SV=2
LDHB seq:	L-lactate dehydrogenase B chain OS=Homo sapiens GN=LDHB PE=1 SV=2
LDHB seq:	L-lactate dehydrogenase B chain OS=Homo sapiens GN=LDHB PE=1 SV=2
YWHAE seq	14-3-3 protein epsilon OS=Homo sapiens GN=YWHAE PE=1 SV=1
IDH1 seq: 2	Isocitrate dehydrogenase [NADP] cytoplasmic OS=Homo sapiens GN=IDH1 PE=1 SV=2
IDH2 seq: 2	Isocitrate dehydrogenase [NADP], mitochondrial OS=Homo sapiens GN=IDH2 PE=1 SV=2
DDX5 seq:	Probable ATP-dependent RNA helicase DDX5 OS=Homo sapiens GN=DDX5 PE=1 SV=1
DDX17 seq	Probable ATP-dependent RNA helicase DDX17 OS=Homo sapiens GN=DDX17 PE=1 SV=2
REPS1 pS39	RalBP1-associated Eps domain-containing protein 1 OS=Homo sapiens GN=REPS1 PE=1 SV=3
SIPA1L1 pS	Signal-induced proliferation-associated 1-like protein 1 OS=Homo sapiens GN=SIPA1L1 PE=1 SV=4
RPS20 seq:	40S ribosomal protein S20 OS=Homo sapiens GN=RPS20 PE=1 SV=1
VWA9 seq:	von Willebrand factor A domain-containing protein 9 OS=Homo sapiens GN=VWA9 PE=1 SV=2
SNRPD3 se	Small nuclear ribonucleoprotein Sm D3 OS=Homo sapiens GN=SNRPD3 PE=1 SV=1
HERC1 seq:	Probable E3 ubiquitin-protein ligase HERC1 OS=Homo sapiens GN=HERC1 PE=1 SV=2
STIM1 pS57	Stromal interaction molecule 1 OS=Homo sapiens GN=STIM1 PE=1 SV=3
STIM1 pS57	Stromal interaction molecule 1 OS=Homo sapiens GN=STIM1 PE=1 SV=3
SMC1A seq	Structural maintenance of chromosomes protein 1A OS=Homo sapiens GN=SMC1A PE=1 SV=2
PRKCSH se	Glucosidase 2 subunit beta OS=Homo sapiens GN=PRKCSH PE=1 SV=2
C2orf44 pS	WD repeat and coiled-coil-containing protein C2orf44 OS=Homo sapiens GN=C2orf44 PE=1 SV=1
C2orf44 pS	WD repeat and coiled-coil-containing protein C2orf44 OS=Homo sapiens GN=C2orf44 PE=1 SV=1
RPS6 seq: 1	40S ribosomal protein S6 OS=Homo sapiens GN=RPS6 PE=1 SV=1
CCDC88A s	Girdin OS=Homo sapiens GN=CCDC88A PE=1 SV=2
RPS9 seq: 3	40S ribosomal protein S9 OS=Homo sapiens GN=RPS9 PE=1 SV=3
MAPRE3 pS	Microtubule-associated protein RP/EB family member 3 OS=Homo sapiens GN=MAPRE3 PE=1 SV=1
MAPRE3 pS	Microtubule-associated protein RP/EB family member 3 OS=Homo sapiens GN=MAPRE3 PE=1 SV=1
S100A16 se	Protein S100-A16 OS=Homo sapiens GN=S100A16 PE=1 SV=1
S100A16 p	Protein S100-A16 OS=Homo sapiens GN=S100A16 PE=1 SV=1
UBE2O pS8	(E3-independent) E2 ubiquitin-conjugating enzyme OS=Homo sapiens GN=UBE2O PE=1 SV=3
SRRT pT544	Serrate RNA effector molecule homolog OS=Homo sapiens GN=SRRT PE=1 SV=1
DNAJA1 se	DnaJ homolog subfamily A member 1 OS=Homo sapiens GN=DNAJA1 PE=1 SV=2
LYRM4 seq:	LYR motif-containing protein 4 OS=Homo sapiens GN=LYRM4 PE=1 SV=1
COIL pS566	Coilin OS=Homo sapiens GN=COIL PE=1 SV=1
SIPA1L3 pS	Signal-induced proliferation-associated 1-like protein 3 OS=Homo sapiens GN=SIPA1L3 PE=1 SV=3
HSP90B1 s	Endoplasmin OS=Homo sapiens GN=HSP90B1 PE=1 SV=1
PSME3 seq:	Proteasome activator complex subunit 3 OS=Homo sapiens GN=PSME3 PE=1 SV=1
GTF3C1 seq	General transcription factor 3C polypeptide 1 OS=Homo sapiens GN=GTF3C1 PE=1 SV=4
GNB4 seq:	Guanine nucleotide-binding protein subunit beta-4 OS=Homo sapiens GN=GNB4 PE=1 SV=3
SMS seq: 2	Spermine synthase OS=Homo sapiens GN=SMS PE=1 SV=2
TKT seq: 54	Transketolase OS=Homo sapiens GN=TKT PE=1 SV=3
TNS3 pS73	Tensin-3 OS=Homo sapiens GN=TNS3 PE=1 SV=2
TNS3 pS73	Tensin-3 OS=Homo sapiens GN=TNS3 PE=1 SV=2
STIP1 seq: 5	Stress-induced-phosphoprotein 1 OS=Homo sapiens GN=STIP1 PE=1 SV=1
RPL13 seq:	60S ribosomal protein L13 OS=Homo sapiens GN=RPL13 PE=1 SV=4
RPS13 seq:	40S ribosomal protein S13 OS=Homo sapiens GN=RPS13 PE=1 SV=2
IDH2 seq: 7	Isocitrate dehydrogenase [NADP], mitochondrial OS=Homo sapiens GN=IDH2 PE=1 SV=2
[truncated: 979,159 more chars]
